# Dimeric and trimeric catenation of giant chiral [8 + 12] imine cubes driven by weak supramolecular interactions

**DOI:** 10.1038/s41557-022-01094-w

**Published:** 2022-12-01

**Authors:** Bahiru Punja Benke, Tobias Kirschbaum, Jürgen Graf, Jürgen H. Gross, Michael Mastalerz

**Affiliations:** Organisch-Chemisches Institut, Ruprecht-Karls-Universität Heidelberg, Heidelberg, Germany

**Keywords:** Interlocked molecules, Molecular capsules

## Abstract

Mechanically interlocked structures, such as catenanes and rotaxanes, are fascinating synthetic targets and some are used for molecular switches and machines. Today, the vast majority of catenated structures are built upon macrocycles and only a very few examples of three-dimensional shape-persistent organic cages forming such structures have been reported. However, the catenation in all these cases was based on a thermodynamically favoured *π*–*π-*stacking under certain reaction conditions. Here, we show that catenane formation can be induced by adding methoxy or thiomethyl groups to one of the precursors during the synthesis of chiral [8 + 12] imine cubes, giving dimeric and trimeric catenated organic cages. To elucidate the underlying driving forces, we reacted 11 differently 1,4-disubstituted terephthaldehydes with a chiral triamino tribenzotriquinacene under various conditions to study whether monomeric cages or catenated cage dimers are the preferred products. We find that catenation is mainly directed by weak interactions derived from the substituents rather than by *π*-stacking.

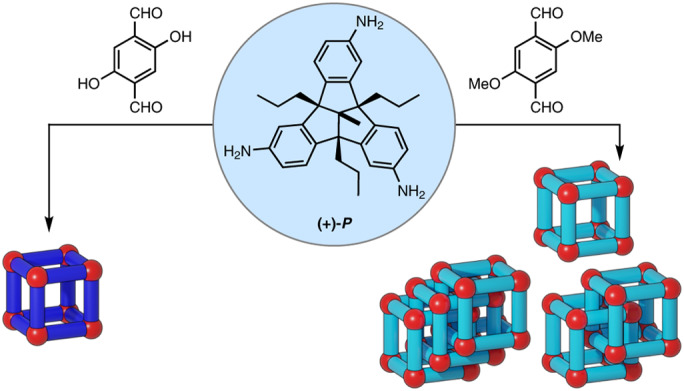

## Abstract (web summary)

Interlocked shape-persistent organic cages are rare structures and the majority are formed using *π*-stacking as the driving force. Now it is shown that weak dispersion interactions—which are modulated by changing the 1,4-substituents of the constituent dialdehyde linkers—can be used to form interlocked dimeric and trimeric catenated cages.

## Main

Since the first report by Wasserman in the early 1960s of a catenane as a statistically occurring by-product during a macrocyclization via acyloin condensation^1^, interest in interlocked molecular structures has developed rapidly in recent decades^2,3^, especially because such compounds provide fundamental knowledge for supramolecular switches and machines^4–6^. Although Schill and Lüttringhaus introduced rational synthetic approaches towards a number of interlocked structures as early as the 1960s^7^, the real spark for this research field was the work by Sauvage and co-workers to create a high-yielding catenane synthesis by exploiting the templated coordination of two molecular strands by a metal ion before closing these to two interlocked macrocycles via Williamson ether synthesis^8,9^. This concept of using a template was and still is the most frequently applied strategy for the synthesis of more complex interlocked structures such as Borromean rings^10^, various knots^11–14^, a Star of David catenane^15^, poly[*n*]catenanes^16^ or interlocked coordination cages^17–21^. In addition to ligand-to-metal ion coordination, weaker and less-directing supramolecular interactions—such as hydrogen-bonding or *π*–*π-*stacking—have been used to arrange molecular precursors in the right fashion to synthesize interlocked structures^22^.

In contrast to the relatively large number and diversity of interlocked coordination cages^18^, only a few examples of purely organic cage catenanes have been reported to date. The first example was reported by Beer et al.^23^ They exploited a template effect of sulfate anions interacting with carbamate units to prearrange two tripodal precursor molecules in such a way that the end-capping of these via a copper-mediated 1,3-dipolar cycloaddition resulted in the formation of a triply interlocked cage dimer in 21% yield. One year later, in 2010, Cooper and co-workers described that by changing conditions for the synthesis of a [4 + 6] imine cage by adding catalytic amounts of trifluoroacetic acid to the reaction solution in acetonitrile or dichloromethane (DCM), these [4 + 6] imine cages form triply interlocked dimers^24^, which was proven by single-crystal X-ray diffraction. It was suggested that *π*–*π-*stacking is most probably the driving force for the catenane formation, and if a competing aromatic solvent was present in certain amounts, this indeed suppressed the catenane formation. In 2014, the formation of a quadruply interlocked dimer of a giant [12 + 8] boronic ester cage was described^25^, which was clearly characterized by single-crystal X-ray diffraction. The only difference between the interlocked cage dimer and a corresponding monomeric [12 + 8] boronic ester cage^26^ published before is the position and length of solubilizing alkyl chains in the molecular precursors, which led to the hypothesis that weak dispersion interactions may additionally be responsible for the catenane formation by overcoming any entropic penalty. In similar fashion, albeit more distinct, this entropic penalty was balanced by dispersion interactions in the formation of a hydrocarbon cage and its catenated dimer made by alkyne metathesis^27^. Depending on the concentration of reacting monomers, the equilibrium between monomeric and interlocked cages can be shifted towards one or the other metathesis product. The authors assumed that a triply interlocked structure is energetically more favoured than a singly interlocked one due to a maximization of filled space. In 2015, Li et al. exploited the hydrophobic effect to achieve an interlocked cage dimer via a hydrazone bond formation in water^28^. Very recently, the group of Shaodong Zhang presented the formation of a triply interlocked catenane of a [2 + 3] imine cage^29,30^. Again, it was concluded that the energetic benefits of additional *π*–*π-*stacking provide the driving force. In contrast to the aforementioned examples, in the present work dimer formation has been studied in more detail by kinetic NMR measurements and time-dependent mass spectrometry; however, no thermodynamic assumptions were corroborated experimentally. It is worth mentioning that Greenaway et al. described the unexpected formation of a bridged cage catenane during large high-throughput screening^31^.

During our ongoing work on condensing chiral triamino-tribenzotriquinacenes (TBTQs) with aromatic aldehydes to study self-sorting of cages^32,33^, we serendipitously found a substituent-driven formation of dimeric and trimeric cage catenanes, which is described herein.

## Results and discussion

### Synthesis and characterization of cage and catenanes

**Fig. 1:**
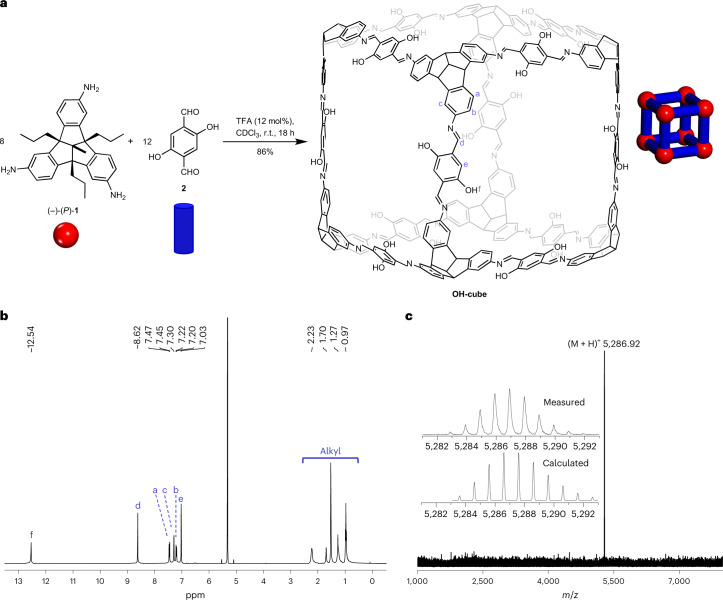
Synthesis and characterization of chiral OH-cube. **a**, Schematic representation of the acid-catalysed 24-fold imine condensation of chiral triamino-TBTQ **1** and 2,5-dihydroxy-terephthalaldehyde **2** to **OH-cube**. Note that the alkyl substituents of TBTQ are omitted from the cubic structure of **OH-cube** for clarity. Reactants and cube are also drawn as cartoons. Red balls represent the TBTQ units and blue struts the aldehyde or imine linker units. **b**, ^1^H NMR spectrum (500 MHz, CD_2_Cl_2_, r.t.) of pure **OH-cube**. For assignment, see atom labels in the molecular structure of **OH-cube** in **a** and Supplementary Information, section 2. **c**, MALDI–TOF mass spectrum (DCTB) of pure **OH-cube**. Inset: comparison of measured and calculated isotopic patterns for **OH-cube**.

Inspired by Xu and Warmuth’s chiral cube^34^, based on the condensation of eight molecules of cyclotriveratrylene trisaldehyde and *para*-phenylene diamine, we intended to use a chiral TBTQ precursor instead, which, in contrast to the cyclotriveratrylene, is structurally fixed and cannot racemize during cage formation. Indeed, the condensation of enantiopure triamino-TBTQ (*P*)*-***1** (ref. ^35^) with 2,4-dihydroxy-terephthalaldehyde **2** under typical conditions we have used before for similar systems (TFA catalyst, CDCl_3_, r.t)^34,36^ gave the clean chiral [8 + 12] cage **OH-cube** in 86% isolated yield (Fig. 1) which was identified by NMR and mass spectrometry.

**Fig. 2:**
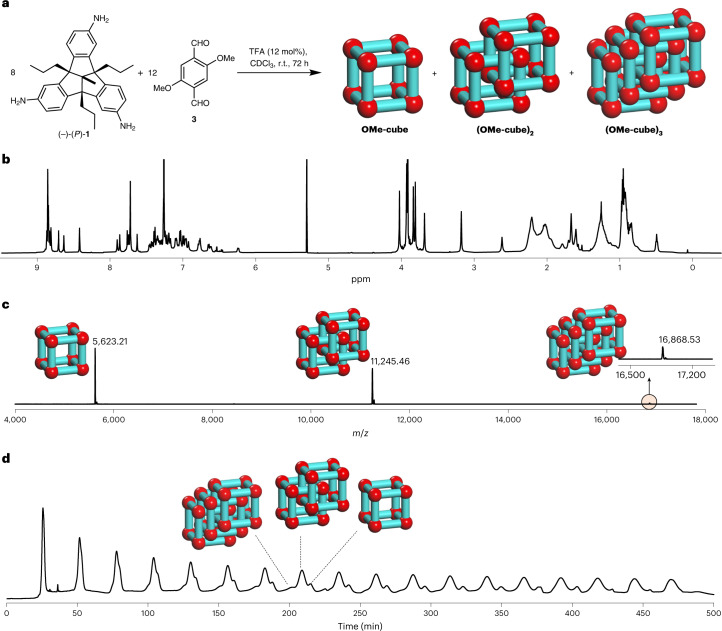
Synthesis and analysis of monomeric cage and dimeric and trimeric catenanes. **a**, Schematic representation of the acid-catalysed 24-fold imine condensation of **1** and **3** to **OMe-cubes**. **b**,**c**, ^1^H NMR (600 MHz, CDCl_3_) (**b**) and MALDI–TOF mass spectrum (DCTB) (**c**) of the crude reaction mixture of **OMe-cubes**. The ^1^H NMR spectrum of the crude product was very complex but that the MALDI–TOF mass spectrum was relatively clear with three distinct peaks. Peaks in the MALDI–TOF mass spectra are labelled with the structures of the products; the small peak for the trimeric catenane is highlighted and shown in the inset. **d**, r-GPC traces (solvent, DCM) of the crude reaction mixture of **OMe-cube**, (**OMe-cube)**_**2**_ and **(OMe-cube)**_**3**_ clearly show three peaks.

Originally we were interested in post-stabilizing the **OH-cube** by Pinnick oxidation to turn imine bonds into amide bonds^37^. As reported before, this does not work with the phenolic hydroxy groups present. To avoid a 24-fold post-synthetic Williamson etherification on **OH-cube** (ref. ^38^), we instead condensed TBTQ (*P*)-**1** with dimethoxy-terephthalaldehyde **3** under the same conditions (Fig. 2a). In contrast to the reaction with aldehyde **2**, here the ^1^H NMR spectrum of the crude product was very complex with a large number of peaks in the aromatic as well as in the aliphatic region (Fig. 2b). The corresponding matrix-assisted laser desorption/ionization–time of flight mass spectrometry (MALDI–TOF-MS) revealed that in addition to the [8 + 12] **OMe-cube** (*m/z* 5,623.21), a [16 + 24] condensation product (*m/z* 11,245.46) was generated and even a small peak with *m/z* 16,868.53 was detected (Fig. 2c), suggesting that a larger [24 + 36] species may have formed. Taking into consideration the complex ^1^H NMR spectra reported previously for triply interlocked cages^24^, it was assumed that these species are most probably catenated dimer **(OMe-cube)**_**2**_ and trimer **(OMe-cube)**_**3**_ rather than larger more symmetric and non-interlocked species. By applying recycling gel-permeation chromatography (r-GPC) with DCM as solvent, it was possible to separate the three compounds after multiple cycles (Fig. 2d and Supplementary Information, section 8).

As described previously in the literature, the equilibrium between monomeric and catenated cages shifts towards the latter by increasing the concentration of reactants and conversely to the monomeric cage by decreasing it. Therefore, the reaction was performed at different concentrations (between 0.42 and 42.8 mM) and analysed mainly by MALDI–TOF-MS (Supplementary Table 1). As expected, with higher concentrations more catenated compounds **(OMe-cube)**_**2**_ and **(OMe-cube)**_**3**_ were found and the concentration needs to be 0.42 mM or below to avoid the formation of those and to form monomeric cage **OMe-cube** exclusively. For comparison, reactions with dihydroxy terephthaldehyde **2** at various concentrations (up to 42.8 mM) did not give any catenated species and in each experiment only monomeric cage **OH-cube** was detected by ^1^H NMR spectroscopy (Supplementary Fig. 398). It is worth mentioning that as soon as a CHCl_3_ or DCM solution of monomeric cage **OMe-cube** was concentrated by rotary evaporation (50 °C, reduced pressure), the equilibrium immediately shifted towards the catenated products **(OMe-cube)**_**2**_ and **(OMe-cube)**_**3**_ as found by NMR and r-GPC analysis. On one hand, this clearly demonstrated the dynamic covalent chemistry character and thus thermodynamically driven formation of the catenane^39^. On the other hand, it made the separation and characterization of monomeric cage **OMe-cube** more challenging.

**Fig. 3:**
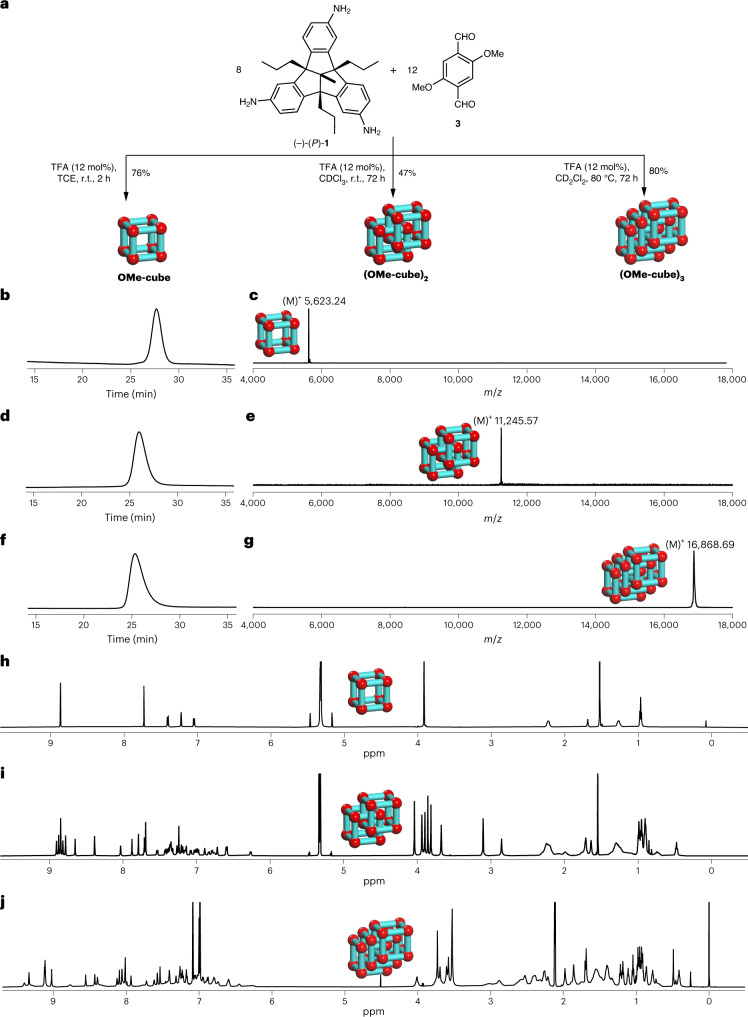
Selective synthesis and characterization of OMe-cube, (OMe-cube)_2_ and (OMe-cube)_3_. **a**, Schematic representation of the acid-catalysed 24-fold imine condensation of **1** and **3** in different solvents for the selective formation of **OMe-cube**, (**OMe-cube)**_**2**_ and **(OMe-cube)**_**3**_. For reaction details, see Supplementary Information, section 2. **b**–**g**, r-GPC traces and MALDI–TOF mass spectra of **OMe-cube**, (**OMe-cube)**_**2**_ and **(OMe-cube)**_**3**_. r-GPC traces (solvent, DCM) of pure **OMe-cube** (**b**), (**OMe-cube)**_**2**_ (**d**) and **(OMe-cube)**_**3**_ (**f**) show retention times of 27.7, 25.9 and 25.4 min, respectively, which are consistent with their sizes_._ Depicted is the first cycle for each. The corresponding MALDI–TOF mass spectra of pure **OMe-cube** (**c**), (**OMe-cube)**_**2**_ (**e**) and **(OMe-cube)**_**3**_ (**g**) show exclusively a single peak for each species_._ **h**,**i**, ^1^H NMR spectra (600 MHz, 295 K, CD_2_Cl_2_) of pure **OMe-cube** (**h**) and (**OMe-cube)**_**2**_ (**i**). **j**, ^1^H NMR spectra (700 MHz, 375 K, toluene-d_8_) of pure **(OMe-cube)**_**3**_.

Despite these findings, we were able to develop a synthetic protocol to isolate **OMe-cube** in 85% yield by using shorter reaction times, certain concentration and temperature thresholds, and exploiting the low solubility of the cage in acetonitrile (Supplementary Information, section 2). To our delight, by changing the solvent to 1,1,2,2-tetrachloroethane (TCE), monomeric cage **OMe-cube** could be synthesized even at higher concentration (5.4 mM), without the necessity of GPC separation, in 76% yield (Fig. 3a). On the other hand, running the reaction of **1** and **3** in CD_2_Cl_2_ instead of CHCl_3_ at 10.7 mM concentration (w.r.t. **1**) and 80 °C for 3 days allowed us to push the equilibrium towards the tricatenane **(OMe-cube)**_**3**_, which was isolated in 80% yield (Fig. 3a). The best results for the dimeric cage **(OMe-cube)**_**2**_ were achieved when **1** and **3** were reacted at 10.7 mM scale. However, **(OMe-cube)**_**2**_ still needed to be separated by r-GPC from **OMe-cube** and **(OMe-cube)**_**3**_ at 30°C to be obtained in 46% isolated yield (Fig. 3a).

### Mechanistic investigation of the monomeric cage to dimeric catenane reaction

To obtain some more mechanistic information on catenane formation, the transformation of **OMe-cube** to **(OMe-cube)**_**2**_ was studied in more detail. Kinetic NMR experiments indicated that full equilibrium between **OMe-cube** (*c*_0_ = 1.35 mM, CDCl_3_) and **(OMe-cube)**_**2**_ was achieved after ∼800 min (*k*_1_ = 10.5 ± 0.4 M^−1^ s^−1^) if catalytic amounts of TFA are present (Supplementary Fig. 506). In the absence of acid, no conversion at all was detected even after 24 h (Supplementary Fig. 507). Mixing a ^15^N-labelled cage *OMe-cube in a 1:1 stoichiometry with non-labelled **OMe-cube** under reaction conditions (TFA catalyst, CDCl_3_) and analysing the mixture after 3 days by MALDI–TOF-MS revealed that all units (TBTQ and linkers) are fully scrambling to a statistical mixture, suggesting that during catenane formation all TBTQ units must be disconnected from the cage scaffolds at a certain stage, opening the cages for catenation (Supplementary Figs. 508 and 509).

### Structural analysis of cage and dimeric and trimeric catenanes

**Fig. 4:**
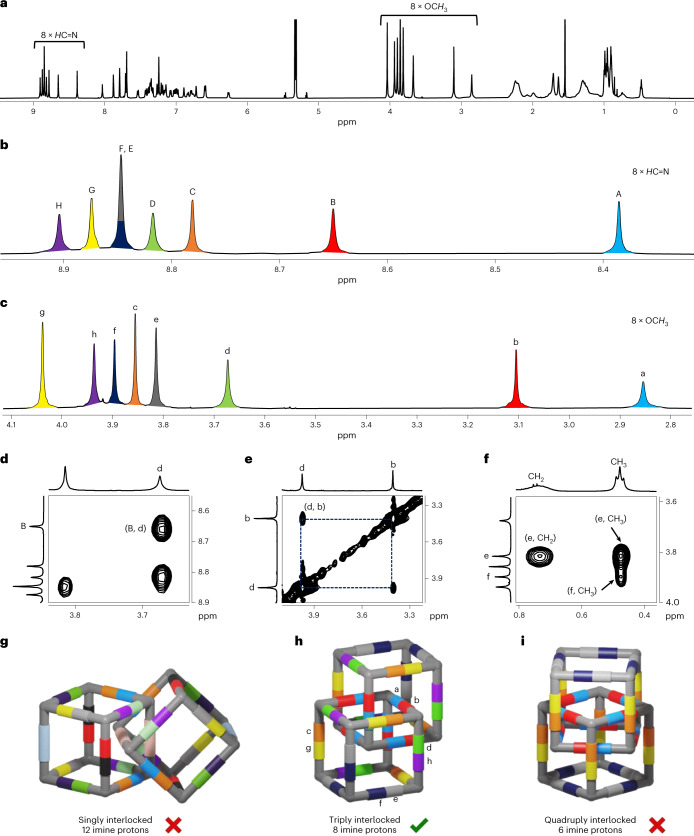
NMR spectroscopic analysis and assignment of (OMe-cube)_2_. **a**, Full ^1^H NMR (600 MHz, CD_2_Cl_2_) spectrum of (**OMe-cube)**_**2**_. **b**,**c**, Partial ^1^H NMR (600 MHz, CD_2_Cl_2_) showing eight different types of imine peaks (**b**) and methoxy peaks (**c**) of (**OMe-cube)**_**2**_. **d**, NOESY spectrum showing cross-peaks between imine proton B and methoxy proton d. **e**, NOESY spectrum showing cross-peaks between methoxy protons b and d. **f**, NOESY spectrum showing cross-peaks between highly shielded aliphatic protons of propyl chains and methoxy protons e and f. **g**, Cartoon of the singly catenated cubes highlighting the 12 different magnetically equivalent imine protons. **h**, Cartoon of the triply interlocked catenated cube, highlighting the eight different magnetically equivalent imine protons. The colour code and assignment are the same as in **b** and **c**. **i**, Cartoons of the quadruply interlocked catenated cubes, highlighting the six different magnetically equivalent imine protons.

The GPC-purified fractions were again separately injected into r-GPC, resulting in the detection of three single distinct peaks, each of nearly Gaussian shape with retention times of 25.4 min (first fraction), 25.9 min (second fraction) and 27.7 min (third fraction) (Fig. 3b,d,f). MALDI–TOF-MS analysis of each fraction (Fig. 3c,e,g) now show single peaks exclusively at *m/z* 16,868.69 (first fraction), *m/z* 11,245.57 (second fraction) and *m/z* 5,623.24 (third fraction), which exactly fit to a [24 + 36], a [16 + 24] and a [8 + 12] species, respectively. The ^1^H NMR spectrum (Fig. 3h) of the third fraction was very simple, showing signals comparable to **OH-cube**, and in combination with the mass spectrum (Fig. 3c) this compound was clearly identified as the monomeric chiral [8 + 12] **OMe-cube**. Diffusion-ordered NMR spectroscopy (DOSY) in deuterated DCM at 295 K showed only one trace with a diffusion coefficient of *D* = 3.09 × 10^−10^ m^2^ s^−1^, which according to the uncorrected Stokes–Einstein equation corresponds to a solvodynamic radius of *r*_S_ = 16.9 Å (Supplementary Fig. 308). In contrast to the relatively simple ^1^H NMR spectrum of monomeric **OMe-cube** (Fig. 3h), that of the [16 + 24] species was much more complex (Fig. 3i). Nevertheless, despite the large number of signals, most of them were sharp and did not superimpose, allowing a more detailed analysis of the structure (Fig. 4a; for detailed structural analysis, see Supplementary Information, section 13). Two-dimensional NMR experiments identified eight different types of imine protons and eight different methoxy protons (Fig. 4b,c). This is exactly the number expected for a triply interlocked cage dimer (see cartoon in Fig. 4h) Other possible catenanes, such as a singly interlocked dimer (Fig. 4g, 12 imine peaks) or quadruply interlocked dimer (Fig. 4i, six imine peaks), can clearly be ruled out. DOSY NMR in CD_2_Cl_2_ at 295 K showed only one trace of signals for (**OMe-cube)**_**2**_, confirming that this is a single species. The diffusion coefficient *D* = 2.67 × 10^−10^ m^2^ s^−1^ corresponds to a solvodynamic radius of 19.6 Å (Supplementary Fig. 309). This is slightly larger than for **OMe-cube** (16.9 Å) which is consistent with its slightly larger size.

**Fig. 5:**
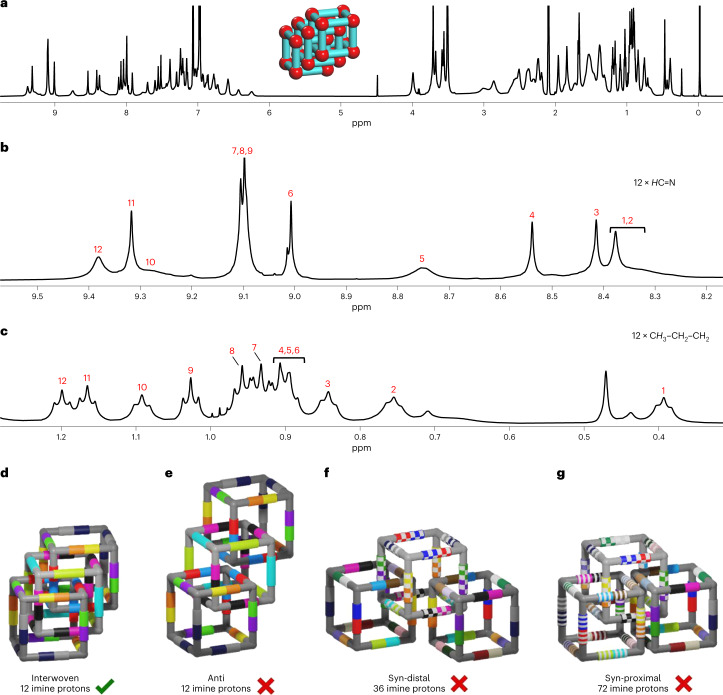
NMR spectroscopic analysis and assignment of (OMe-cube)_3_. **a**, Full ^1^H NMR (700 MHz, 375 K, toluene-d_8_) spectrum of (**OMe-cube)**_**3**_. **b**,**c**, Partial ^1^H NMR (700 MHz, 375 K, toluene-d_8_) showing 12 different types of imine peaks (**b**) and methyl peaks (**c**) of (**OMe-cube)**_**3**_. **d**–**g**, Cartoons of the interwoven **[(OMe-cube)@(OMe-cube)]@(OMe-cube)** (**d**), ***anti*** **(OMe-cube)@(OMe-cube)@(OMe-cube)** (**e**), *syn*-distal (**f**) and *syn*-proximal (**g**) trimeric catenanes, highlighting the 12, 12, 36 and 72 different magnetically equivalent imine protons in different colours, respectively.

The trimeric interlocked cage **(OMe-cube)**_**3**_ shows much less resolved multiple broad peaks at room temperature in the ^1^H NMR spectrum (Supplementary Fig. 72) in contrast to dimer **(OMe-cube)**_**2**_. However, in toluene-d_8_ at 375 K a much better resolved spectrum was obtained, showing sets of 12 magnetically different peaks, such as 12 imine protons and 12 signals of the terminal CH_3_ group of the propyl chains (Fig. 5a–c and Supplementary Figs. 88 and 89). This excludes a *syn*-distal connectivity (36 imine peaks; Fig. 5f, for models, see Supplementary Fig. 522) and a *syn*-proximal connectivity at two adjacent corners at the central cube (here 72 imine peaks are expected; Fig. 5d, for models, see Supplementary Fig. 523). For both a chain-like *anti*-connected catenane **(OMe-cube)@(OMe-cube)@(OMe-cube)** (Fig. 5e) and for an interwoven catenane [**(OMe-cube)@(OMe-cube)]@(OMe-cube**) (Fig. 5d) the same number—12—of magnetically different peaks are expected, as has been found (for models, see Supplementary Figs. 520 and 521). However, since the trimeric catenane **(OMe-cube)**_**3**_ is still very soluble under reaction conditions and no traces of larger oligomers such as tetrameric and pentameric cages **(OMe-cube)**_**4**_ or **(OMe-cube)**_**5**_ are found by mass spectrometry, it seems to be more likely that the interwoven catenane [**(OMe-cube)@(OMe-cube)]@(OMe-cube**) and not the chain-like *anti*-conformed catenane **(OMe-cube)@(OMe-cube)@(OMe-cube)** has formed. If it were the latter motif, we would expect at least some formation of longer oligomers, which is not the case. On the other hand, an interwoven tetrameric catenane is simply not possible for steric reasons, which once more would explain the absence of larger species and thus favours this motif for the trimeric catenane [**(OMe-cube)@(OMe-cube)]@(OMe-cube**). DOSY NMR of **(OMe-cube)**_**3**_ again shows a single trace with a diffusion coefficient *D* = 2.64 × 10^−10^ m^2^ s^−1^. The calculated solvodynamic radius of 19.8 Å (Supplementary Fig. 310) was found to be almost similar to that of the dicatenane **(OMe-cube)**_**2**_ (19.6 Å), once more suggesting a tightly packed, interlocked structure. In the DOSY NMR spectra of a 1:1 stoichiometric mixture of pure **(OMe-cube)**_**2**_ and **(OMe-cube)**_**3**_ (Supplementary Fig. 512) the difference between the diffusion coefficient values is very small, further supporting the more dense interwoven catenane [**(OMe-cube)@(OMe-cube)]@(OMe-cube**) model.

It is worth mentioning that for **OH-cube**, **OMe-cube**, **(OMe-cube)**_**2**_, and for **(OMe-cube)**_**3**_ and the other cage catenanes described below, innumerable large single crystals have been obtained from various solvents. Unfortunately, even synchrotron radiation did not provide sufficient resolution to elucidate the solid-state structures.

### Investigation of the driving force for catenation

We were interested in obtaining further insight into the driving force of the unique catenation of methoxy cage **OMe-cube** to dimer **(OMe-cube)**_**2**_ and even to trimer **(OMe-cube)**_**3**_ and why we do not see any such catenation for the hydroxyl-substituted **OH-cube** at any concentration. Due to the triply interlocked catenation of dimer **(OMe-cube)≡(OMe-cube)** in favour of a possible singly interlocked dimer (**OMe-cube)–(OMe-cube)**, the aforementioned *π*–*π-*stacking as driving force—found for almost all the interlocked organic cages previously described in the literature—was excluded (see above), otherwise singly interlocked catenation should have been formed preferably. In addition for **OH-cube** a higher tendency of dimerization would have been expected than for **OMe-cube**, because intramolecular hydrogen-bonding of the hydroxyl imine stiffens the *π*-backbone and strongly enhances intermolecular *π*–*π*-stacking^40^. This assumption is strengthened by the fact that a wide range of reaction conditions (different acid concentrations, different concentrations of reactants, different solvents, different and elevated temperatures, different reaction times (up to several months)) yielded no substantial catenane formation for **OH-cube** (see Supplementary Figs. 398, 400, 401 and 510). A kinetic formation driven by precipitation was also ruled out, because the reaction mixtures of **1**, **2** and **OH-cube** were at all times clear solutions^39^. Furthermore, mixing **OH-cube** and ^15^N-labelled *OH-cube in a 1:1 ratio and treating this mixture under reaction conditions gave the two highest MALDI–TOF mass peaks at *m/z* 5,297.56 (corresponding to **OH-cube-**^**15**^**N**_**12**_**)** and *m/z* 5,320.54 (corresponding to **(OH-cube-**^**15**^**N**_**12**_ + **Na)**, suggesting a complete scrambling in solution, supporting the thermodynamic formation of **OH-cube** and confirming that it is not a kinetic trap (Supplementary Figs. 510 and 511)^41^.

**Fig. 6:**
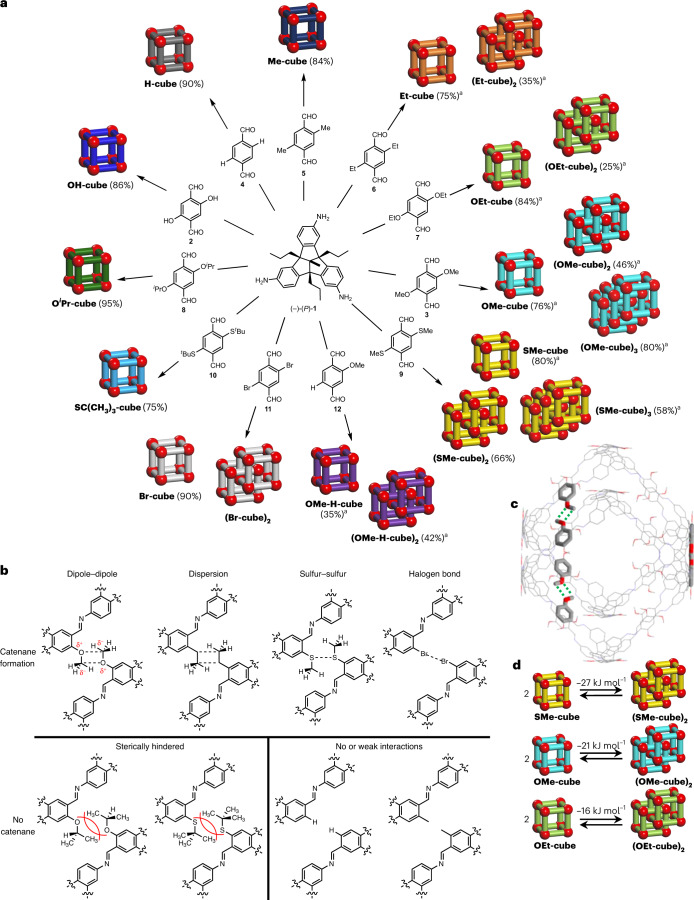
Summary of cage and catenanes with 12 different dialdehyde linkers. **a**, Reactions of various dialdehydes with TBTQ triamine **1**, giving cages (left side) or catenated cages (right side). ^a^Yields are given for optimized reactions. **b**, Summary of interactions resulting in catenane formation or their exclusion. **c**, Possible conformational arrangement of the two interlocked cages with spatial arrangement of methoxy groups interacting via weak dipole–dipole forces (green dotted lines). **d**, Gibbs enthalpy for catenation events determined by NMR spectroscopy.

Since *π*-stacking was ruled out as a driving force, we first hypothesized that dipole–dipole interactions (so-called Keesom interactions)^42^ of the methoxy groups may be responsible for the catenation, as found, for example, in single crystals of methoxy-substituted *π*-systems (*d*(Me*O*⋯*C*H_3_O) = 3.1 Å)^43^. In this respect, it is worth mentioning that the bridged cage catenane reported by Greenaway et al., when originally achieving cages based on dimethoxy terephthaldehyde **3** (ref. ^31^). could rely on such weak interactions, although a closer look at the X-ray structure shows the same methoxy–methoxy interaction motif, albeit with a larger distance between the functional groups of *d*(Me*O*⋯*C*H_3_O) = 3.5 Å (Supplementary Fig. 513). Conformational analysis by semi-empirical calculations (Supplementary Section 19) of **OMe-cube** as well as nuclear Overhauser enhancement spectroscopy (NOESY) cross-peaks between imine CH and the aromatic TBTQ protons revealed a low barrier of rotation of the linker units at room temperature, which is also present in the triply interlocked dimer (**OMe-cube)**_**2**_, allowing the mechanically interlocked molecule to adopt conformations that have three such methoxy–methoxy interactions (Fig. 6c). According to this assumption (Supplementary Fig. 513d,e), one methoxy group per dialdehyde unit should be enough to foster catenation, and indeed reacting dialdehyde **12** (with only one methoxy group present) with triamine **1** in CD_2_Cl_2_ clearly gave catenated **(H/OMe-cube)**_**2**_, as determined by mass spectrometry (Supplementary Fig. 345).

If methoxy groups are absent, no catenane formation should occur. Thus triamine **1** was reacted with non-substituted terephthalaldehyde **4** (Fig. 6a) under different conditions (various solvents, Supplementary Fig. 405) and no catenane formation was observed. Pure **H-cube** was isolated in 90% from THF. By adding two methyl substituents instead of two methoxy groups to the aldehyde (**5**) still almost no catenane formation is observed by ^1^H NMR (Supplementary Fig. 406) and monomeric **Me-cube** is formed in 84% yield. As soon as the alkyl substituents at the dialdehyde precursor (**6**) get longer (here ethyl), the possibility of intermolecular dispersion interactions^44^ (Fig. 6b) is slightly increased and now some catenane (**Et-cube)**_**2**_ was found by ^1^H NMR spectroscopy as well as mass spectrometry (Supplementary Fig. 407) besides monomeric **Et-cube** (which still is the main product). Comparing the different results of **Me-cube** versus **Et-cube**, based on the simple elongation of the alkyl chains by one methylene unit each, electronic effects to foster *π*–*π*-stacking can again be ruled out, because the methyl as well as the ethyl substituents have almost the same Hammett parameters (*σ*_m_(Me) = −0.07; *σ*_m_(Et) = −0.07; *σ*_p_(Me) = −0.17; *σ*_p_(Et) = −0.15)^45^. As for **OMe-cube** and **(OMe-cube)**_**2**_, the ratio of catenane (**Et-cube)**_**2**_ versus monomeric cage **Et-cube** was also strongly solvent dependent for the reaction of triamine **1** and aldehyde **6** and in THF the amount of catenane was higher than, for example, in CHCl_3_ and both compounds (monomer and catenane) were selectivity achieved by adjusting the conditions. The reaction in CHCl_3_ at room temperature gave monomeric cage **Et-cube** in 75% isolated yield, whereas running the reaction in THF gave after separation 35% of the catenated dimer (**Et-cube)**_**2**_ in pure form.

To further exclude pure electronic effects for a probable *π*-stacking, we reacted triamine **1** with diethoxy- and diisopropoxy dialdehydes **7** and **8** (Fig. 6a), where the substituents have comparable Hammett parameters to those in dimethoxy dialdehyde **3** (*σ*_m_(OMe) = 0.12; *σ*_m_(OEt) = 0.10; *σ*_m_(O^*i*^Pr) = 0.10), but are of different steric demand. Whereas for the diethoxy dialdehyde **7** some catenane formation of (**OEt-cube)**_**2**_ was observed, for diisopropoxy dialdehyde **8** no catenane (**OiPr-cube)**_**2**_ was observed by ^1^H NMR spectroscopy (Supplementary Figs. 408 and 409), supporting once more the hypothesis that the catenane formation is mainly driven by weak interactions derived from the substituents rather than by *π*-stacking, and in case of the latter steric repulsion is stronger than the weak attraction (Fig. 6b; Charton steric parameters for Me, Et and ^*i*^Pr are *ν*_Me_ = 0.52, *ν*_Et_ = 0.56, *ν*_iPr_ = 0.76)^46^.

By increasing these weak interactions, the equilibrium may be shifted towards the interlocked structures. By using dimethylthioether **9** in the condensation with triamine **1** in CDCl_3_, the triply interlocked catenated dimer **(SMe-cube)**_**2**_ was formed almost exclusively (Fig. 6a and Supplementary Fig. 410). Harsher conditions were needed to push the system to the trimeric cage **(SMe-cube)**_**3**_, which was isolated in 58% yield by using CD_2_Cl_2_ as a solvent in combination with elevated temperature (80 °C, screw-capped vessel, 4 days). By two-dimensional NMR spectroscopy, the same linear and interwoven catenation motif was found as for **(OMe-cube)**_**3**_ (Supplementary Fig. 495). Switching the solvent system to TCE, monomeric **SMe-cube** was isolated in 80% yield. Again, to rule out electronic effects based on the thioalkyl substituent donating to the *π*-system of the aromatic dialdehyde, di-*tert*-butylthioether-substituted dialdehyde **10** with two sterically demanding *tert*-butyl groups was investigated in the reaction (*ν*_Me_ = 0.52 versus *ν*_tBu_ = 1.24)^46^. As expected, only clean monomeric **SC(CH**_**3**_**)**_**3**_**-cube** was formed and isolated in 75% yield (Supplementary Fig. 411). Finally, we investigated the reaction of dibromo dialdehyde **11** with triamine **1**, to see whether halogen bond formation^47^ can also induce catenation. Although the mass spectrum of the reaction mixture in CD_2_Cl_2_ showed a pronounced peak at *m/z* 13,591.6, which is the double that of the monomeric **Br-cube** (*m/z* 6,796.4), the correlated ^1^H NMR spectrum showed only small detectable peaks of interlocked species and mainly contained signals of pure monomeric **Br-cube** (Supplementary Fig. 412). However, in contrast to all other reactions, here a precipitate of very low solubility was formed, which may contain insoluble (**Br-cube)**_**2**_.

### Thermodynamic studies

To correlate the weak interactions responsible for catenation, the systems where catenation occurred have been studied by concentration-dependent NMR spectroscopy (Supplementary Information, section 12), to estimate the Gibbs enthalpy of cage to catenane transformation. With Δ*G*_298_ = −26.7 kJ mol^−1^ the reaction of **2 SMe-cube** → **(SMe-cube)**_**2**_ is about 6 kJ mol^−1^ higher than for the methoxy cages **2 OMe-cube** → **(OMe-cube)**_**2**_ (Δ*G*_298_ = −20.8 kJ mol^−1^) and almost 10 kJ mol^−1^ higher than found for the ethoxy cages **2 OEt-cube** → **(OEt-cube)**_**2**_ (Δ*G*_298_ = −15.7 kJ mol^−1^) (Fig. 6d), Unfortunately, in CDCl_3_ the amount of **(Et-cube)**_**2**_ in relation to **Et-cube** was too small to determine reliable numbers by this method. As mentioned repeatedly above, the chosen solvents had clear impacts on whether catenation occurred or not. Therefore, we looked at the van’t Hoff plots of temperature-dependent ^1^H NMR measurements of the equilibria **2 OMe-cube** ↔ **(OMe-cube)**_**2**_ and **2 SMe-cube** ↔ **(SMe-cube)**_**2**_ to obtain further insights into whether the processes of catenation are enthalpy- or entropy-driven reactions. In both investigated cases the catenation is an entropy-driven reaction (Δ*S*_OMe_ = +114.3 J K^−1^ mol^−1^ and Δ*S*_SMe_ = +185.3 J K^−1^ mol^−1^), rather than an enthalpy-driven reaction (Δ*H*_OMe_ = +13.1 kJ mol^−1^ and Δ*H*_SMe_ = +29.4 kJ mol^−1^), which also explains the temperature dependency of the reaction. This further suggests that solvophobic effects^48^ are dominating the catenation process at least in the investigated solvent systems. These solvophobic effects are dependent on the polarity of side chains^49,50^, as observed here, and need to be investigated further for such systems.

## Conclusions

We observed the formation of dimeric and trimeric cage catenanes based on the weak interactions of the substituents of the constituent 1,4-disubstituted terephthaldehydes. Whereas *π*-stacking was ruled out as a driving force, Keesom and London dispersion interactions between the substituents and with the solvent were considered. Changing the methoxy groups to less polar ethyl groups decreased catenane formation substantially. In cases where there is only a methyl substituent or no substituent at the dialdehyde, the intermolecular forces are too weak to foster catenane formation. Finally, dialdehydes with thiomethyl substituents were beneficial for catenane formation and indeed a clear reaction to **(SMe-cube)**_**2**_ was observed, having a difference of |Δ*G*°| of ∼6 kJ mol^−1^ for catenane formation compared with **(OMe-cube)**_**2**_. Solvent effects play a crucial role in the cases where dimeric and trimeric catenation was observed. Both systems (with OMe and SMe substituents) showed the same trends. In TCE, monomeric cages **OMe-cube** and **SMe-cube** were formed selectively, whereas in DCM at elevated temperatures the clean formation of trimeric catenanes **(OMe-cube)**_**3**_ and **(SMe-cube)**_**3**_ was observed. Comparing the coherence energy densities of the solvents (TCE = 98.0 cal cm^−^^3^ versus DCM = 93.7 cal cm^−^^3^) in combination with the data from van’t Hoff plot analysis revealed that solvophobic effects may play a major role because reactions toward catenated cages are entropically favoured.

This motif of weak dispersion interactions in combination with solvophobic effects as a driving force for catenation of shape-persistent organic cages offers an approach for further study of the influence of subtle structural changes in combination with chosen solvents as reaction media to understand events of dynamic covalent chemistry of larger and more complex structures and to construct, for example, poly[*n*]catenated cages with *n* > 3, to create cages of higher molecular volumes.

## Methods

### Synthesis of (OMe-cube)_2_

To a solution of TBTQ **1** (20 mg, 0.043 mmol) and 2,5-dimethoxy-terephthaldehyde **3** (12.6 mg, 0.0649 mmol) in deuterated chloroform (4 ml) in a screw-capped 8 ml glass vial, a catalytic amount of TFA (0.4 µl, 0.0052 mmol) was added and the reaction mixture was stirred at r.t. for 3 days. Afterwards, the crude reaction mixture was washed with aqueous K_2_CO_3_ solution (0.25 M, 3 × 2 ml), dried over Na_2_SO_4_ and concentrated under reduced pressure. The resulting red solid was immediately dissolved in DCM and purified by r-GPC (DCM, 30 °C, 5 ml min^−1^) to give 14 mg (46%) of **(OMe-cube)**_**2**_ as a yellow solid. Melting point, 315 °C (decomposed). ^1^H NMR (600 MHz, CD_2_Cl_2_): *δ* (ppm) = 8.90 (s, 6H, *H*C=N), 8.87 (s, 6H, *H*C=N), 8.85 (s, 12H, *H*C=N), 8.82 (s, 6H, *H*C=N), 8.78 (s, 6H, *H*C=N), 8.65 (s, 6H, *H*C=N), 8.38 (s, 6H, *H*C=N), 8.03 (s, 6H, Ar–*H*), 7.88 (s, 6H, Ar–*H*), 7.79 (s, 6H, Ar–*H*), 7.71 (s, 6H, Ar–*H*), 7.69 (12H, Ar–*H*), 7.53 (d, ^3^*J* = 8.4 Hz, 6H, TBTQ Ar–*H*), 7.41 (d, ^3^*J* = 8.4 Hz, 6H, TBTQ Ar–*H*), 7.38 (d, ^3^*J* = 8.4 Hz, 6H, TBTQ Ar–*H*), 7.36–7.32 (m, 6H, TBTQ–Ar–*H* and Ar–*H*), 7.27 (s, 6H, TBTQ–Ar–*H*), 7.24 (s, 12H, Ar–*H*, TBTQ–Ar–*H*), 7.21 (s, 6H, TBTQ–Ar–*H*), 7.19 (s, 6H, TBTQ Ar–*H*), 7.17–7.14 (m, 6H, TBTQ–Ar–*H*), 7.08 (d, ^3^*J* = 8.4 Hz, 6H, TBTQ–Ar–*H*), 7.03–6.97 (m, 18H, TBTQ–Ar–*H*), 6.89 (s, 6H, TBTQ–Ar–*H*), 6.83 (d, ^3^*J* = 8.4 Hz, 6H, TBTQ–Ar–*H*), 6.78 (d, ^3^*J* = 8.4 Hz, 6H, TBTQ–Ar–*H*), 6.77 (d, ^3^*J* = 8.4 Hz, 6H, TBTQ–Ar–*H*), 6.72 (s, 6H, TBTQ–Ar–*H*), 6.59 (d, ^3^*J* = 8.4 Hz, 12H, TBTQ–Ar–*H*), 6.26 (d, ^3^*J* = 7.8 Hz, 6H, TBTQ–Ar–*H*), 4.04 (s, 18H, OC*H*_*3*_), 3.94 (s, 18H, OC*H*_*3*_), 3.90 (s, 18H, OC*H*_*3*_), 3.86 (s, 18H, OC*H*_*3*_), 3.82 (s, 18H, OC*H*_*3*_), 3.67 (s, 18H, OC*H*_*3*_), 3.11 (s, 18H, OC*H*_*3*_), 2.85 (s, 18H, OC*H*_*3*_), 2.30–1.74 (m, 96H, –C*H*_*2*_CH_2_CH_3_) 1.71 (s, 30H), 1.64 (s, 18H), 1.35–1.09 (m, 84H, –CH_2_C*H*_*2*_CH_3_), 1.0–0.90 (m, 126H, –CH_2_CH_2_C*H*_*3*_), 0.77–0.72 (m, 12H, –CH_2_C*H*_*2*_CH_3_), 0.48 (t, ^3^*J* = 7.2 Hz, 18H, –C*H*_*2*_CH_2_*CH*_*3*_). ^13^C NMR (151 MHz, CD_2_Cl_2_): *δ* (ppm) = 156.5, 155.7, 155.3, 155.2, 155.0, 154.6, 154.31, 154.26, 154.2, 153.9, 153.8, 153.1, 152.7, 152.5, 152.6, 152.3, 152.2, 150.1, 149.8, 149.64, 149.6, 149.5, 149.4, 149.3, 149.1, 146.7, 146.6, 146.4, 146.1, 146.0, 145.5, 145.4, 129.0, 128.9, 128.6, 128.55, 128.49, 127.8, 125.0, 124.6, 124.42, 124.36, 124.2, 124.0, 119.8, 119.7, 119.4, 119.04, 118.96, 118.8, 118.5, 118.4, 118.1, 117.5, 117.3, 116.1, 116.0, 111.2, 110.2, 109.83, 109.77, 109.63, 109.58, 73.5, 73.2, 73.0, 67.39, 67.35, 67.33, 67.23, 67.18, 66.9, 56.71, 56.68, 56.65, 56.61, 56.6, 56.4, 56.1, 55.4, 41.8, 41.2, 41.1, 41.0, 21.3, 21.1, 21.0, 20.9, 20.8, 20.3, 15.5, 15.4, 15.32, 15.29, 15.0, 14.9. Fourier transform infrared spectroscopy (neat, attenuated total reflectance): 𝜈̃ (cm^−1^) = 2,999 (w), 2,957 (m), 2,925 (m), 2,870 (m), 2,853 (m), 1,734 (w), 1,616 (m), 1,593 (m), 1,492 (s), 1,482 (s), 1,465 (s), 1,410 (s), 1,373 (m), 1,211 (s), 1,140 (m), 1,043 (s), 974 (w), 882 (m), 821 (m), 701 (w). Ultraviolet–visible spectroscopy (CH_2_Cl_2_): *λ*_max_ (nm) = 296, 406. MALDI–TOF (*trans*-2-[3-(4-*tert*-butylphenyl)-2-methyl-2-propenylidene]malononitrile (DCTB)): *m/z* [M]^+^ calculated for C_752_H_768_N_48_O_48_, 11,245.94; found, 11,245.57. Elemental analysis: calculated for C_752_H_768_N_48_O_48_·33CH_2_Cl_2_, C 67.11, H 5.98, N 4.79; found C 66.94, H 5.91, N 4.86.

## Online content

Any methods, additional references, Nature Research reporting summaries, source data, extended data, supplementary information, acknowledgements, peer review information; details of author contributions and competing interests; and statements of data and code availability are available at 10.1038/s41557-022-01094-w.

## Supplementary information

Supplementary Information Supplementary Information

Supplementary Data Xyz files of computational data.

## Data Availability

All data supporting the findings of this study are available within the paper and its Supplementary Information. The original experimental data to this paper has been deposited in the repository heiDATA and can be downloaded via https://heidata.uni-heidelberg.de/privateurl.xhtml?token=96ab88cd-5fc2-4b2c-b836-522dab565ba2
