# Publisher Correction: Dimeric and trimeric catenation of giant chiral [8 + 12] imine cubes driven by weak supramolecular interactions

**DOI:** 10.1038/s41557-022-01133-6

**Published:** 2023-01-04

**Authors:** Bahiru Punja Benke, Tobias Kirschbaum, Jürgen Graf, Jürgen H. Gross, Michael Mastalerz

**Affiliations:** Organisch-Chemisches Institut, Ruprecht-Karls-Universität Heidelberg, Heidelberg, Germany

**Keywords:** Interlocked molecules, Molecular capsules

Correction to: *Nature Chemistry* 10.1038/s41557-022-01094-w, published online 1 December 2022.

In the version of this article initially published, there were errors in Fig. 1a, where the stereochemistry display was incorrect; in Fig. 4c, where NMR assignment labels were incorrect; and in Fig. 6, where cube colour coding and reported yields (OH-cube, 86%, not 88%; OMe-cube, 76%, not 85%) were incorrect. The figures have been updated in the HTML and PDF versions of the article.

