# Supplementary material for: Dimeric and trimeric catenation of giant chiral [8 + 12] imine cubes driven by weak supramolecular interactions
Source: Nat Chem. 2022 Dec 1;15(3):413–23. doi: 10.1038/s41557-022-01094-w (PMC9986109; doi:10.1038/s41557-022-01094-w)
Supplement: Supplementary file 1 — Supplementary Information [file 41557_2022_1094_MOESM1_ESM.pdf]

nature chemistry

Article https://doi.org/10.1038/s41557-022-01094-w

Dimeric and trimeric catenation of giant chiral [8 + 12] imine cubes driven by weak supramolecular interactions

In the format provided by the authors and unedited

Dimeric and Trimeric Catenation of Giant Chiral [8+12] Imine Cubes Driven by Weak Supramolecular Interactions

Bahiru Punja Benke, [a] Tobias Kirschbaum,[a] Jürgen Graf, [a] Jürgen H. Gross,[a] and Michael Mastalerz*[a]

Table of Contents

1. Materials and methods 2
2. Synthesis and characterization 3
3. 1D and 2D NMR spectra 31
4. DOSY spectra 195
5. Mass spectra 206
6. IR spectra 217
7. UV spectra 228
8. GPC data 239
9. Variable concentration NMR studies 248
10. Variable catalyst concentrations 252
11. Different solvent NMR studies 253
12. Thermodynamic data 267
13. ^{1}H NMR assignment for dimeric catenane 287
14. Variable temperature NMR studies with trimeric catenane 333
15. ^{1}H NMR kinetic studies 338
16. Catenation studies without acid catalyst 341
17. Mechanistic studies of catenanes by mass spectrometry 342
18. Models of Weak interactions 347
19. Computational details 348
20. References 361

1. Materials and methods

All reagents for synthesis were purchased from commercial suppliers and used without further purification. Chloroform was passed through the basic alumina column (Type IV) before it was used it for all the cage and catenane reactions. All air-sensitive reactions were performed using oven-dried glassware under an inert atmosphere of argon. Analytical thin layer chromatography (TLC) was performed on MERCK precoated silica gel 60 F_{254} TLC plates. All the compounds were visualized under UV light. For flash column chromatography silica gel 60 (40–63 μm/230–400 mesh ASTM) was purchased from Macherey-Nagel. The NMR data were acquired on a Bruker Avance III 300 MHz spectrometer equipped with a 5-mm BB{1H} z-axis gradient probe (301 MHz) , a Bruker Avance DRX 300 MHz spectrometer with a 5-mm 13C/31P{1H} z-axis gradient probe (300 MHz), an Bruker Avance III 400 MHz instrument with a 5-mm BB{1H} z-axis gradient N2-cryogenic probe (400 MHz), a Bruker Avance III 500 MHz spectrometer with a 5-mm BB{1H/19F} z-axis gradient probe (500 MHz), an Bruker Avance III 600 MHz instrument with a 5-mm 1H{13C/15N/31P} z-axis gradient He-cryogenic probe (600 MHz) and a Bruker Avance Neo 700 MHz spectrometer with a 5-mm 1H{13C/15N} z-axis gradient He-cryogenic probe. The NMR spectra were referenced using residual solvent peak as the standard. Chemical shift values are denoted in parts per million (δ), coupling constants (J) are reported in Hertz (Hz), and spin multiplicities are reported as singlet (s), broad singlet (bs), doublet (d), triplet (t), quartet (q), and multiplet (m). Recycling gel permeation chromatography (rGPC) was carried out using Shimadzu DGU-20A3R degassing unit, LC-20AD pump unit, CTO-20AC column oven, CBM-20A communication bus module, SPD-M20A diode array detector, FRC-10A fraction collector, FCV-20AH2 valve unit, a PSS SDV (20 x 50 mm) pre-column and SDV 100 Å (20 x 300 mm) and 500 Å (20 x 300 mm) columns. For HRMS measurement, Fourier Transform Ion Cyclotron Resonance (FTICR) mass spectrometer solariX (Bruker Daltonik GmbH, Bremen, Germany) equipped with a 7.0 T superconducting magnet and interfaced to an Apollo II Dual ESI/MALDI source was used. A tims-TOF MALDI spectra were collected on Bruker timsTOF fleX instrument from Bruker Daltonics with DCTB (trans-2-[3-(4-tert-butylphenyl)-2-methyl-2-propenylidene]malononitrile) as a matrix. MALDI-TOF MS spectra were collected from a Bruker Daltonik Reflex III, on a Bruker ApexQe or on a Bruker AutoFlex Speed TOF with DCTB as a matrix. Elemental analysis was performed in Microanalytical Laboratory of the University of Heidelberg using an Elementar Vario EL machine. Melting points were measured using a Büchi Melting Point B-545 instrument. CompactStar CS4 centrifuge machine from VWR was used to collect the precipitate of the cages.

2. Synthesis and characterisation

a. Synthesis of TBTQ and dialdehyde linkers

Compound 1

TBTQ **1** was synthesized according to literature known procedures. [1a]

Compounds 2 and 3

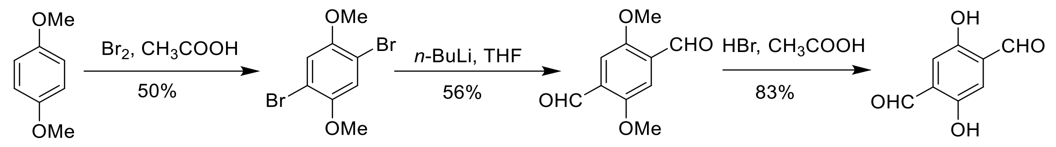

1,4-dibromo-2,5-dimethoxybenzene,[1b] 2,5-dimethoxyterephthalaldehyde,[1c] 2,5-dihydroxyterephthalaldehyde[1d] were synthesized according to literature known procedures. All obtained analytical data were consistent with those in literature.

Compound 5

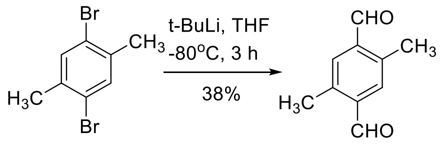

2,5-dimethylterephthalaldehyde [2,3] was obtained according to a literature known procedure. All obtained analytical data were consistent with those in literature.

Compound 6

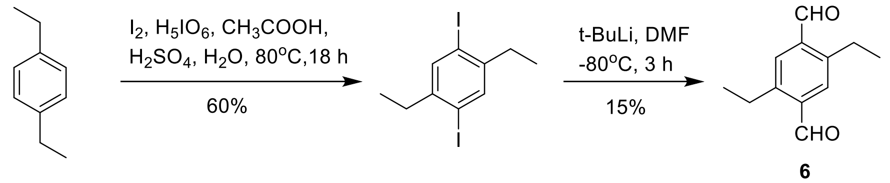

1,4-diethyl-2,5-diiodobenzene[2] 2,5-diethylterephthalaldehyde[2] were synthesized according to literature known procedures. All obtained analytical data were consistent with those in literature.

Compounds 7 and 8

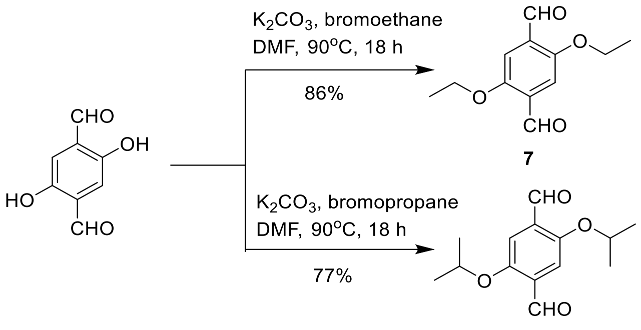

7

8

2,5-diethoxyterephthalaldehyde[4] and 2,5-isopropoxyterephthalaldehyde[4] were obtained according to literature known procedures. All obtained analytical data were consistent with the literature.

Compounds 9 and 10

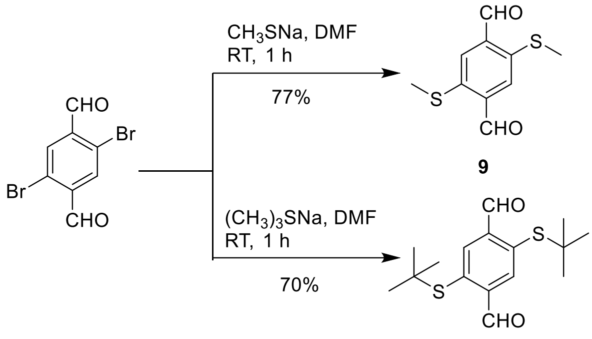

9

10

2,5-bis(methylthio)terephthalaldehyde[5] and 2,5-bis(tert-butylthio)terephthalaldehyde[5] were synthesized according to literature known procedure. All obtained analytical data were consistent with those in literature.

Compound 11

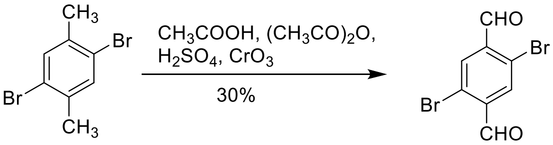

11

2.5-Dibromo-terephthaldehyde[6] was obtained according to literature known procedure. All obtained analytical data were consistent with those in literature.

Compound 12

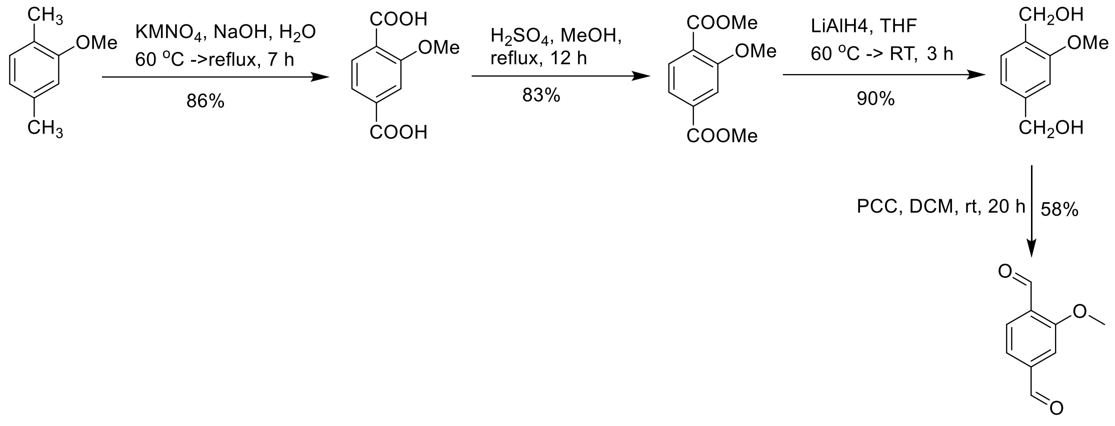

12

2-methoxy terephthaldehyde **12** was synthesized according to a literature procedure. [7] All obtained analytical data were consistent with those in literature.

Synthesis of ^{15}N labelled TBTQ-TA 14

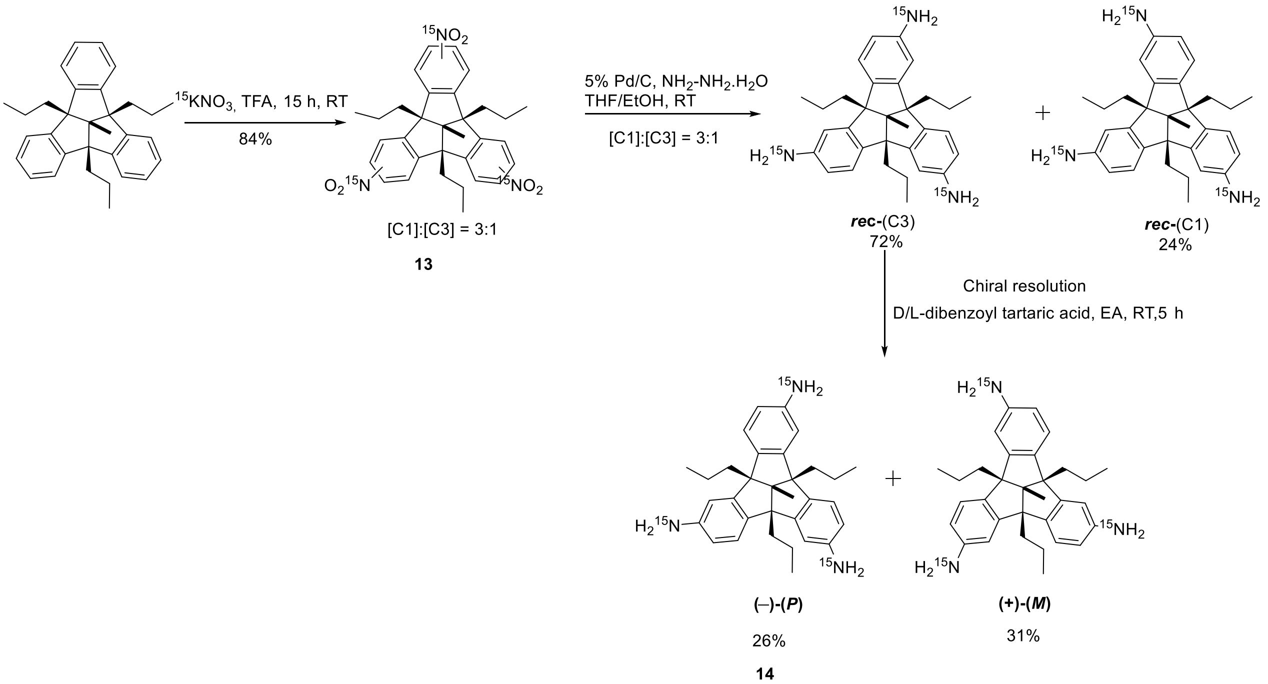

Enantiopure **14** was synthesized according to literature procedure[1a].

^{15}N labelled Trinitro TBTQ 13

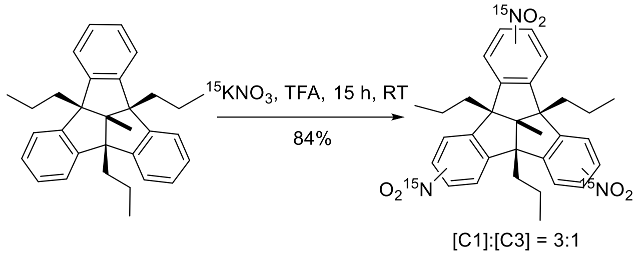

13

Mp: 256-258°C; **^{1}H NMR** (600 MHz, CD_2Cl_2): δ = 8.21-8.18 (m, 3H), 8.18-8.17 (m, 3H), 8.10-8.07 (m, 6H), 7.56 (d, *J* = 9 Hz, 3H), 7.49 (d, *J* = 8.4 Hz, 3H), 2.29-2.22 (m, 12H), 1.71 (s, 6H), 1.22-1.15 (m, 12H), 0.99-0.96 (m, 18H); **^{13}C NMR** (151 MHz, CD_2Cl_2): δ = 154.4, 154.19, 154.15, 154.0, 149.2, 149.02, 149.0, 148.97, 148.96, 148.91, 148.89, 148.83, 148.81, 148.8, 148.73, 148.7, 124.93, 124.91, 124.90, 124.88, 124.86, 124.85, 124.10, 124.09, 124.06, 124.05, 123.98, 123.97, 123.94, 123.93, 119.45, 119.44, 119.39, 119.39, 119.36, 119.35, 119.31, 119.30, 73.9, 68.1, 67.72, 67.70, 67.4, 40.7, 40.59, 40.57, 40.45, 20.8, 20.72, 20.68, 15.3, 15.0; **FT-IR** (neat, ATR): $\tilde{\nu}$ (cm^{-1}) 3070 (w), 2964 (w), 2933 (w), 2873 (w), 1591 (w), 1488 (s), 1315 (s), 1149 (w), 1109 (m), 1072 (w), 1056 (w), 906 (w), 896 (w), 839 (m), 825 (m), 759 (w), 723 (s), 686 (w), 665 (w), 622 (w); **HR-MS (ESI$^+$)**: *m/z* [M+Na]$^+$ calcd. for $C_{32}H_{33}{}^{15}N_3O_6Na$: 581.2178, found 581.2173. **Elemental analysis**: calcd. for $C_{32}H_{33}{}^{15}N_3O_6\cdot{}^1/_8\cdot CH_2Cl_2$: C 67.79, H 5.89, N 7.91, found C 67.79, H 5.30, N 7.80.

^{15}N labelled enantiopure (-)-(*P*) Triamino TBTQ 14

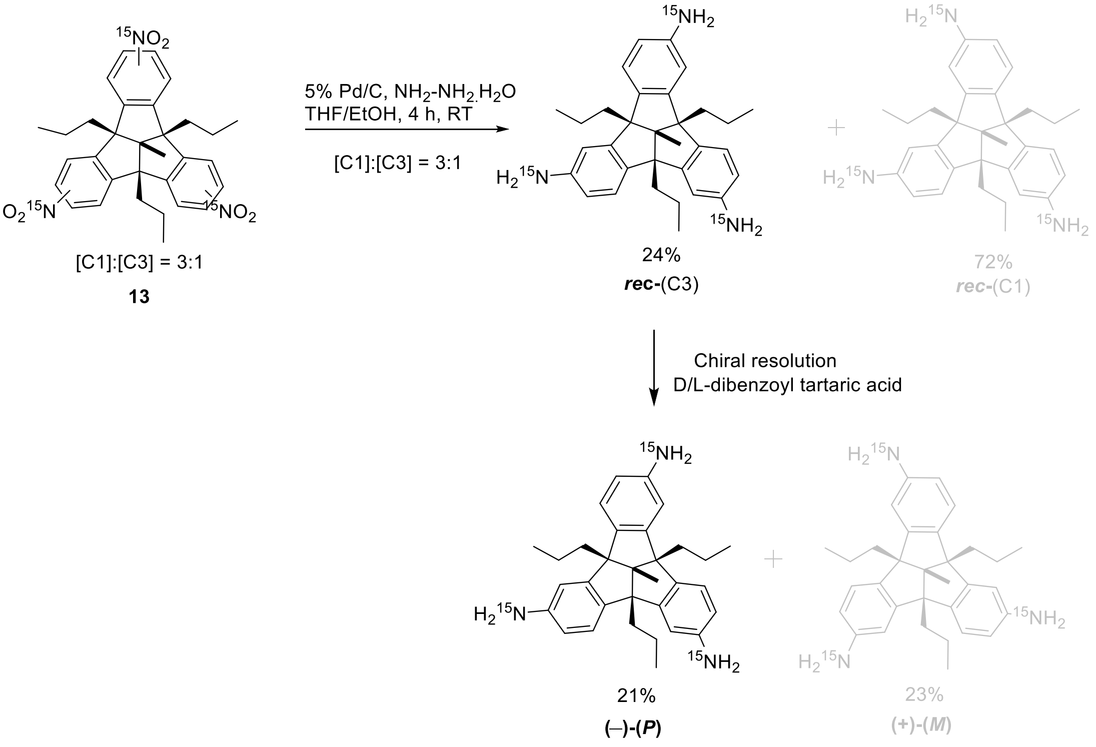

14

Mp: 289°C (decomposed); **^{1}H NMR** (600 MHz, CD_2Cl_2) δ = 6.99 (d, ^3J = 8.4 Hz, 3H), 6.52 (t, d, J = 1.8 Hz, 3H), 6.46 (dt, J = 1.8 Hz, 3H), 3.55 (s, 5H), 3.55 (s, 6H), 2.05-1.96 (m, 6H), 1.52 (s, 3H), 1.20-1.14 (m, 6H), 0.90 (t, J = 7.8 Hz, 9H); **^{13}C NMR** (151 MHz, CD_2Cl_2): δ = 150.2, 146.44, 146.37, 138.6, 124.14, 124.13, 114.98, 114.96, 109.47, 109.46, 73.0, 66.6, 41.1, 20.8, 15.4, 15.3; **FT-IR** (neat, ATR): $\tilde{\nu}$ (cm^{-1}) ; = 3433 (w), 3379 (w), 3330 (m), 3321 (m), 3190 (w), 3006 (w), 2958 (w), 2929 (w), 2910 (w), 2869 (w), 2852 (w), 1608 (s), 1583 (m), 1492 (s), 1456 (m), 1307 (s), 1263 (s), 1245 (w), 1149 (w), 1132 (w), 1101 (w), 1078 (w), 1055 (w), 929 (w), 850 (m), 802 (s), 763 (s), 750 (s), 686 (s), 628 (m); **HR-MS (ESI$^+$)**: *m/z* $[M+Na]^+$ calcd. for $C_{32}H_{39}{}^{15}N_3Na$: 491.2953, found 491.2948 **Elemental analysis:** calcd. for $C_{32}H_{39}{}^{15}N_3{\cdot}H_2O$: C 79.30, H 8.53, N 8.87, found C 79.03, H 8.05, N 8.60.

a. Synthesis of cages and catenanes

Synthesis of OH-cube

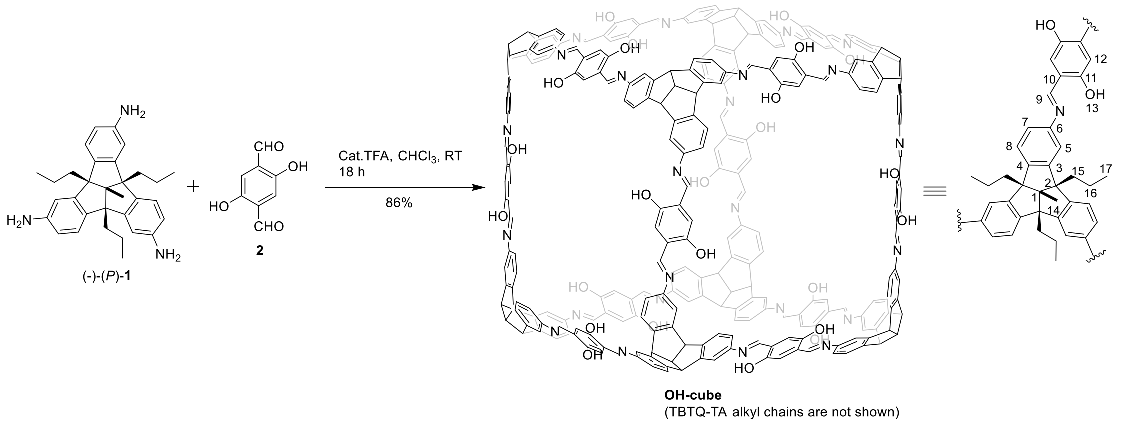

To a solution of TBTQ **1** (10 mg, 0.022 mmol) and 2,5-dihydroxy-terephthaldehyde **2** (5.4 mg, 0.033 mmol) in deuterated chloroform (2 mL) in a screw-cap 8 mL glass vial, a catalytic amount of TFA (0.2 µL, 0.0026 mmol) was added and the reaction mixture was stirred at RT for 18 hours. Afterwards, the reaction mixture was diluted with dichloromethane to a total volume of 30 mL and the solution was stirred with saturated aq. solution of Na_2SO_4 (4 mL) at RT for ten minutes. The organic layer was separated, dried over Na_2SO_4 and concentrated to ~ 0.5 mL by rotary evaporation. Acetonitrile (4 mL) was added and the formed precipitate was separated by centrifugation technique. The precipitate was sonicated in acetonitrile and separated by centrifugation technique to give 12.2 mg (86%) of **OH-cube** as a brown solid.

Mp: 314°C (decomposed); **^{1}H NMR** (400 MHz, CD_2Cl_2): δ = 12.54 (s, 24H, *H*-13), 8.62 (s, 24H, *H*-9), 7.46 (d, ^3J = 8.4 Hz, 48H, *H*-8), 7.29 (d, ^3J = 1.2 Hz, 24H, *H*-5), 7.20 (dd, ^3J = 8.4 Hz, ^4J = 1.2 Hz, 24 H, *H*-7), 7.03 (s, 24H, *H*-12), 2.22 (br (t), 48H, *H*-15) 1.69 (s, 24H, *H*-14), 1.28-1.15 (m, 48H, *H*-16), 0.97 (t, ^3J = 7.2 Hz, 72H, *H*-17); **^{13}C NMR** (151 MHz, CD_2Cl_2): δ = 161.3 (*C*-9), 153.3 (*C*-11), 149.7 (*C*-4), 148.0 (*C*-6), 147.7 (*C*-3), 124.9 (*C*-8), 122.8 (*C*-10), 119.4 (*C*-12), 119.2 (*C*-7), 118.8 (*C*-5), 73.5 (*C*-1), 67.4 (*C*-2), 41.0 (*C*-15), 20.9 (*C*-16), 15.4

(*C*-14), 15.2 (*C*-17); **FT-IR** (neat, ATR): $\tilde{\nu}$ (cm^{-1}) = 2957 (m), 2927 (m), 2869 (w), 1664 (w), 1612 (s), 1587 (s), 1481 (s), 1457 (w), 1334 (m), 1313 (m), 1257 (m), 1150 (s), 1099 (w), 937 (w), 873 (w), 765 (m), 695 (w), 668 (m); **UV/Vis** (CH_2Cl_2): λ_{max} (nm) = 294, 356, 433; **MALDI-TOF** (DCTB): *m/z* $[M+H]^+$ calcd. for $C_{352}H_{337}N_{24}O_{24}$: 5286.60, found 5286.92. **Elemental analysis** calcd. for $C_{352}H_{336}N_{24}O_{24}\cdot 7CH_2Cl_2$: C 73.60, H 6.15, N 5.79, found C 73.55, H 6.04, N 5.90.

Synthesis of ^{15}N labelled *OH-cube

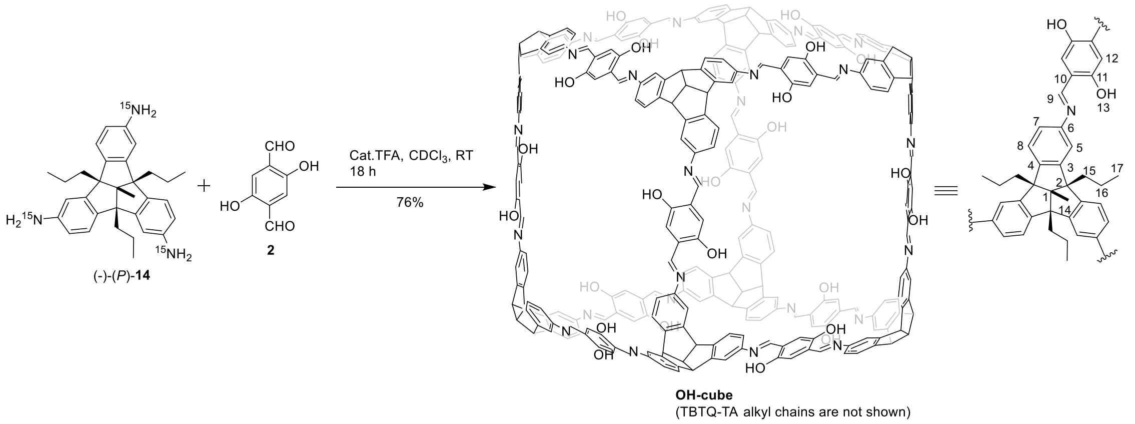

^{15}N-labelled ***OH-cube** was prepared from TBTQ **14** (5 mg, 0.022 mmol) and 2,5-dihydroxy-terephthaldehyde **2** (2.7 mg, 0.033 mmol) in deuterated chloroform (1 mL) as described above for **OH-cube**. Yield: 5.4 mg (76%) of ^{15}N-labelled ***OH-cube** as a brown solid.

Mp: >290°C; **^{1}H NMR** (700 MHz, CD_2Cl_2): δ = 12.56 (s, 24H, H-13), 8.62 (d, ^2J = 2.1 Hz, 24H, H-9), 7.46 (d, ^3J = 8.4 Hz, 24H, H-8), 7.29 (s, 24H, H-5), 7.20 (d, ^3J = 7.7 Hz, 24 H, H-7), 7.03 (s, 24H, H-12), 2.23 (br (t), 48H, H-15) 1.69 (s, 24H, H-14), 1.26-1.25 (m, 48H, H-16), 0.97 (t, ^3J = 7.0 Hz, 72H, H-17); **^{13}C NMR** (176 MHz, CD_2Cl_2): δ = 161.3, 161.2, 153.4, 149.7, 148.0, 147.7, 124.9, 122.8, 119.4, 119.2, 118.8, 73.5, 67.4, 41.0, 20.9, 15.5, 15.2; **FT-IR** (neat, ATR): $\tilde{\nu}$ (cm^{-1}) = 3386 (w), 2960 (m), 2931(w), 2873 (w), 1606 (w), 1579 (s), 1481(s), 1380 (w), 1332 (m), 1315 (m), 1217(m), 1151 (w), 972 (w), 871 (m), 856 (w), 806 (w), 792 (w), 734 (w), 646 (w); **UV/Vis** (CH_2Cl_2): λ_{max} (nm) = 296, 356, 433; **TIMS-TOF Flex** (DCTB): *m/z* M^+ calcd. for $C_{352}H_{336}{}^{15}N_{24}O_{24}$: 5309.5198, found 5309.5188.

Original protocol to get the mixture of mix of OMe-cube, (OMe-cube)$_2$ and (OMe-cube)$_3$

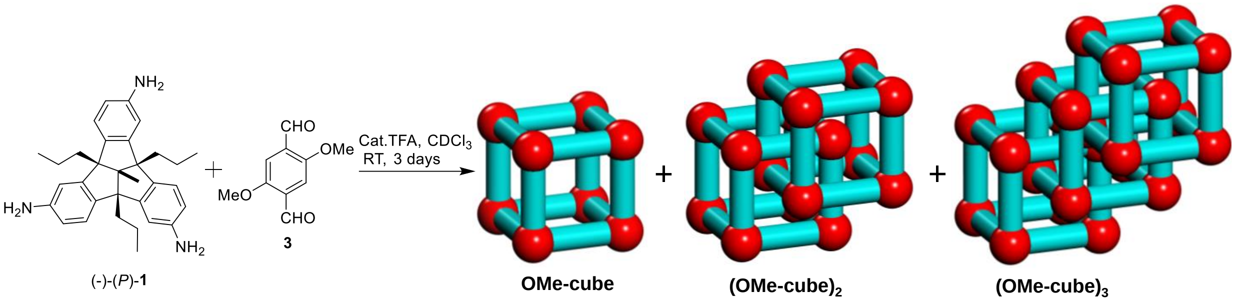

To a solution of TBTQ **1** (20 mg, 0.043 mmol) and 2,5-dimethoxy-terephthaldehyde **3** (12.6 mg, 0.0649 mmol) in deuterated chloroform (4 mL) in a screw-capped 8 mL glass vial, a catalytic amount of TFA (0.4 µL, 0.0052 mmol) was added and the reaction mixture was stirred at RT for 3 days. Afterward, the crude reaction mixture was washed with aq. K_2CO_3 (0.25 M, 3 × 2 mL) and dried over Na_2SO_4. After removal of the solvent by rotary evaporation, the resulting red color solid was immediately dissolved in dichloromethane and injected into a r-GPC which clearly shows three peaks for **OMe-cube, (OMe-cube)$_2$** and **(OMe-cube)$_3$**. (See Figure 390 for the GPC trace).

Synthesis of OMe-cube (improved protocol)

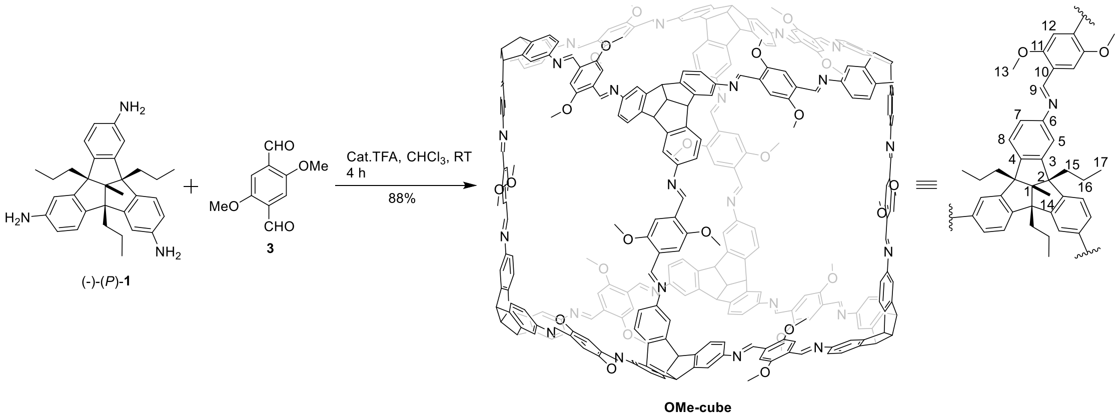

To a solution of TBTQ 1 (5 mg, 0.01 mmol) and 2,5-dimethoxy-terephthaldehyde **3** (3.1 mg, 0.016 mmol) in $CHCl_3$ (25 mL), a catalytic amount of TFA (0.01 µL 0.13 µmol) was added and the reaction mixture was stirred at RT for 4 hours. Afterward, the reaction mixture was diluted with dichloromethane to a total volume of 52 mL and the solution was stirred with aq. solution of K_2CO_3 (0.25 M, 3×18 mL) at RT for five minutes each. The organic layer was dried over Na_2SO_4 and diluted to a total volume of 150 mL with acetonitrile before the resulting solution was concentrated to 100 mL by rotary evaporation. The solution was again diluted to a total volume of 150 mL with the addition of acetonitrile and solvents were completely evaporated under reduced pressure. The obtained yellow solid was suspended in

acetonitrile (5 mL) and ultra-sonicated for 30 seconds. The resulting suspension was transferred into a centrifuge tube and the solid was separated by centrifugation technique. The solid was repeatedly washed with acetonitrile (3 × 8 mL) to give 6.6 mg (88%) of **OMe-cube** as a yellow solid.

Mp: 335ºC (decomposed); **^{1}H NMR** (400 MHz, CD_2Cl_2): δ = 8.87 (s, 24H, *H*-9), 7.73 (s, 24H, *H*-12), 7.41 (d, ^3J = 8 Hz, 24H, *H*-8), 7.22 (s, 24 H, *H*-5), 7.04 (dd, ^3J = 8 Hz, ^4J = 1.6 Hz, 24 H, *H*-7), 3.92 (s, 72H, *H*-13), 2.23 (br (t), 48H, *H*-15) 1.69 (s, 24H, *H*-14), 1.32-1.23 (m, 48H, *H*-16), 0.97 (t, ^3J = 7.2 Hz, 72H, *H*-17); **FT-IR** (ATR): $\tilde{\nu}$ (cm^{-1}) = 2959 (m), 2871 (w), 1615 (m), 1592 (m), 1482 (m), 1466 (m), 1410 (s), 1373 (w), 1286 (w), 1212 (s), 1043 (m), 881 (w), 817 (w), 717 (w), 679 (w), 649 (w), 617 (w), 608 (w); **UV/Vis** (CH_2Cl_2): λ_{max} (nm) = 296, 406; **MALDI-TOF** (DCTB): *m/z* $[M+H]^+$ calcd. for $C_{376}H_{385}N_{24}O_{24}$: 5623.98, found 5623.24; **Elemental analysis** calcd. for $C_{376}H_{384}N_{24}O_{24}\cdot 4CH_2Cl_2$: C 76.54, H 6.63, N 5.64, found C 76.10, H 6.96, N 5.56.

Please note: *A decent ^{13}C NMR spectrum was not available, even by recording 20000 scans on a 600 MHz NMR instrument (see Figure 28). Unfortunately, no concentrated sample could be used, because the compound forms a catenane immediately.*

Synthesis of OMe-cube in 1,1,2,2 tetrachloroethane (TCE)

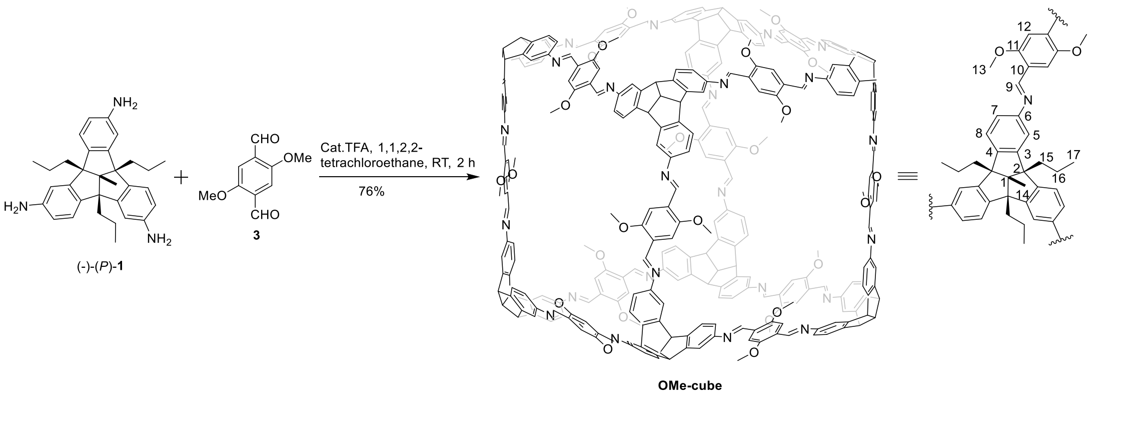

To a solution of TBTQ **1** (5.4 mg, 0.012 mmol) and 2,5-dimethoxy-terephthaldehyde **9** (3.1 mg, 0.016 mmol) in 1,1,2,2 tetrachloroethane (anhydrous, 2 mL), a catalytic amount of TFA (0.1 µL, 0.0013 mmol) was added and the reaction mixture was stirred at RT for 2 hours. A saturated solution of aq. $NaHCO_3$ (2 mL) was added to the reaction mixture and it was further stirred at RT for 10 minutes. The organic layer was separated from the aqueous phase, washed with water (4 x 3 mL) and precipitated by adding methanol into the organic layer (6 mL) and left at RT for 4 hours. The yellow precipitate was separated by centrifugation technique and

repeatedly washed with methanol (4 x 4 mL) to give 5.7 mg (76%) of **OMe-cube** as a yellow solid.

Analytical data is in accordance to those described above.

Synthesis of ^{15}N labelled *OMe-cube in 1,1,2,2 tetrachloroethane (TCE)

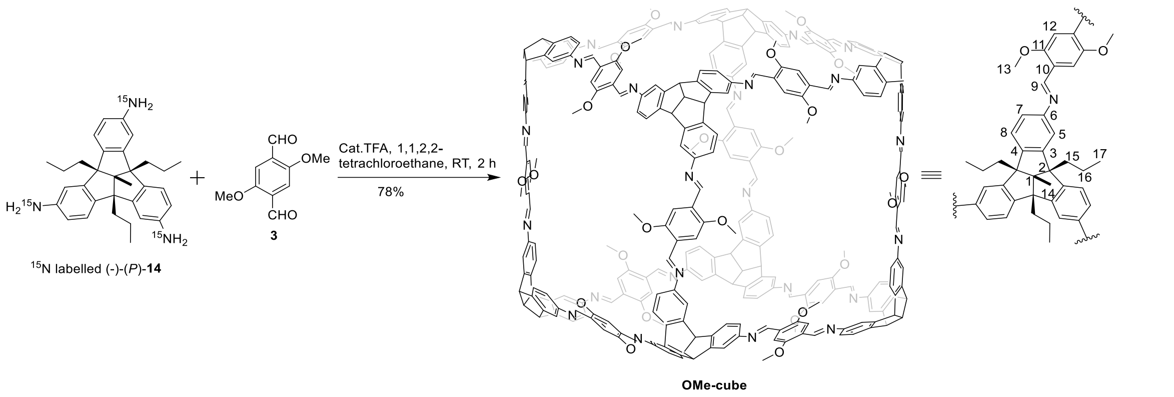

^{15}N-labeled ***OMe-cube** was prepared from TBTQ **14** (5.3 mg, 0.0114 mmol) and 2,5-dimethoxy-terephthaldehyde **9** (3.1 mg, 0.016 mmol) in 1,1,2,2 tetrachloroethane (anhydrous, 2 mL) as described above. Yield: 5.9 mg (78%) of ^{15}N labelled ***OMe-cube** as a yellow solid. **Mp**: 330°C (decomposed); **^{1}H NMR** (700 MHz, $C_2D_2Cl_4$): δ = 8.84 (d, *J* = 3.5 Hz 24H, *H*-9), 7.70 (s, 24H, *H*-12), 7.37 (d, ^3J = 7.7 Hz, 24H, *H*-8), 7.20 (s, 24 H, *H*-5), 7.03 (d, ^3J = 7.7 Hz, 24 H, *H*-7), 3.91 (s, 72H, *H*-13), 2.22 (br (t), 48H, *H*-15) 1.67 (s, 24H, *H*-14), 1.26 (br (m), 48H, *H*-16), 0.98 (t, ^3J = 6.3 Hz, 72H, *H*-17); **FT-IR** (neat, ATR): $\tilde{\nu}$ (cm^{-1}) = 2995 (w), 2956 (w), 2931 (w), 2869 (w), 1579 (m), 1481 (s), 1463 (s), 1406 (s), 1367 (m), 1284 (m), 1247 (w), 1209 (s), 1180 (w), 1166 (w), 1137 (m), 1037 (s), 970 (w), 875 (m), 819 (m), 798 (m), 734 (m), 698 (m), 644 (w); **UV/Vis** (CH_2Cl_2): λ_{max} (nm) = 297, 407; **TIMS-TOF Flex** (DCTB): m/z $[M+H]^+$ calcd. for $C_{376}H_{385}{}^{15}N_{24}O_{24}$: 5647.91, found 5647.91.

Note: A decent ^{13}C NMR spectrum was not available, even by recording 10240 scans on a 700 MHz NMR instrument (see Figure 41). It was also observed that the cage shows decomposition over time in deuterated 1,1,2,2 tetrachloroethane due to acidic nature of solvent.

Synthesis of (OMe-cube)$_2$

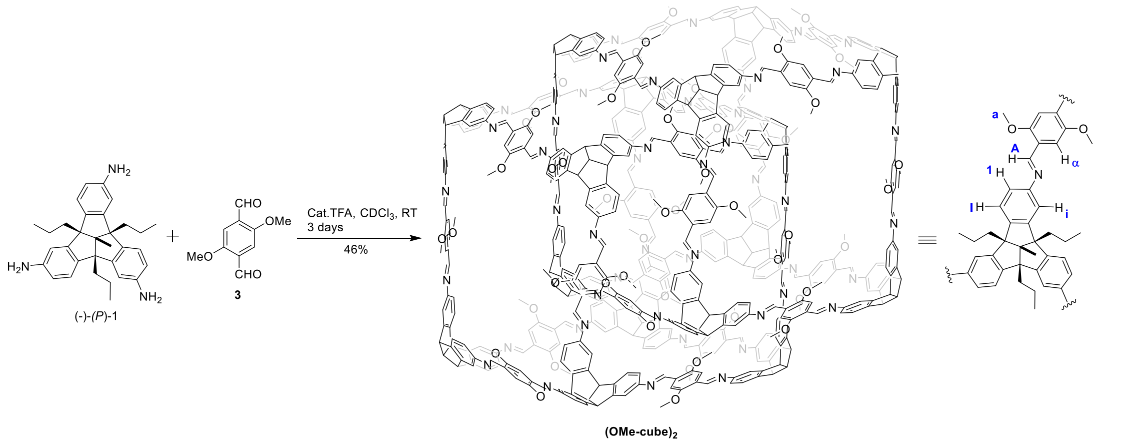

To a solution of TBTQ **1** (20 mg, 0.043 mmol) and 2,5-dimethoxy-terephthaldehyde **3** (12.6 mg, 0.0649 mmol) in deuterated chloroform (4 mL) in a screw-capped 8 mL glass vial, a catalytic amount of TFA (0.4 µL, 0.0052 mmol) was added and the reaction mixture was stirred at RT for 3 days. Afterward, the crude reaction mixture was washed with aq. K_2CO_3 solution (0.25 M, 3 × 2 mL) was dried over Na_2SO_4 and concentrated under reduced pressure. The resulting red-colored solid was immediately dissolved in dichloromethane and purified by recycling gel permeation chromatography (rGPC) (DCM, 30°C, 5 mL/min) to give 14 mg (46%) of **(OMe-cube)$_2$** as a yellow solid.

Mp: 315°C (decomposed); **^{1}H NMR** (600 MHz, CD_2Cl_2): δ = 8.90 (s, 6H, *H*C=N), 8.87 (s, 6H, *H*C=N), 8.85 (s, 12H, *H*C=N), 8.82 (s, 6H, *H*C=N), 8.78 (s, 6H, *H*C=N), 8.65 (s, 6H, *H*C=N), 8.38 (s, 6H, *H*C=N), 8.03 (s, 6H, Ar-*H*), 7.88 (s, 6H, Ar-*H*), 7.79 (s, 6H, Ar-*H*), 7.71 (s, 6H, Ar-*H*), 7.69 (12H, Ar-*H*), 7.53 (d, ^3J = 8.4 Hz, 6H, TBTQ Ar-*H*), 7.41 (d, ^3J = 8.4 Hz, 6H, TBTQ Ar-*H*), 7.38 (d, ^3J = 8.4 Hz, 6H, TBTQ Ar-*H*), 7.36-7.32 (m, 6H, TBTQ-Ar-*H* & Ar-*H*), 7.27 (s, 6H, TBTQ-Ar-*H*), 7.24 (s, 12H, Ar-*H*, TBTQ-Ar-*H*), 7.21 (s, 6H, TBTQ-Ar-*H*), 7.19 (s, 6H, TBTQ Ar-*H*), 7.17-7.14 (m, 6H, TBTQ-Ar-*H*), 7.08 (d, ^3J = 8.4 Hz, 6H, TBTQ-Ar-*H*), 7.03-6.97 (m, 18H, TBTQ-Ar-*H*), 6.89 (s, 6H, TBTQ-Ar-*H*), 6.83 (d, ^3J = 8.4 Hz, 6H, TBTQ-Ar-*H*), 6.78 (d, ^3J = 8.4 Hz, 6H, TBTQ-Ar-*H*), 6.77 (d, ^3J = 8.4 Hz, 6H, TBTQ-Ar-*H*), 6.72 (s, 6H, TBTQ-Ar-*H*), 6.59 (d, ^3J = 8.4 Hz, 12H, TBTQ-Ar-*H*), 6.26 (d, ^3J = 7.8 Hz, 6H, TBTQ-Ar-*H*), 4.04 (s, 18H, OCH_3), 3.94 (s, 18H, OCH_3), 3.90 (s, 18H, OCH_3), 3.86 (s, 18H, OCH_3), 3.82 (s, 18H, OCH_3), 3.67 (s, 18H, OCH_3), 3.11 (s, 18H, OCH_3), 2.85 (s, 18H, OCH_3), 2.30-1.74 (m, 96H, -CH_2CH$_2$CH$_3$) 1.71 (s, 30H), 1.64 (s, 18H), 1.35-1.09 (m, 84H, -CH$_2$CH_2CH$_3$), 1.0-0.90 (m, 126H, -CH$_2$CH$_2$CH_3), 0.77-0.72 (m, 12H, -CH$_2$CH_2CH$_3$), 0.48 (t, ^3J = 7.2 Hz, 18H, -CH_2CH$_2$CH_3); **^{13}C NMR** (151 MHz, CD_2Cl_2): δ = 156.5, 155.7, 155.3, 155.2, 155.0, 154.6, 154.31, 154.26, 154.2, 153.9, 153.8, 153.1, 152.7, 152.5, 152.6,

152.3, 152.2, 150.1, 149.8, 149.64, 149.6, 149.5, 149.4, 149.3, 149.1, 146.7, 146.6, 146.4, 146.1, 146.0, 145.5, 145.4, 129.0, 128.9, 128.6, 128.55, 128.49, 127.8, 125.0, 124.6, 124.42, 124.36, 124.2, 124.0, 119.8, 119.7, 119.4, 119.04, 118.96, 118.8, 118.5, 118.4, 118.1, 117.5, 117.3, 116.1, 116.0, 111.2, 110.2, 109.83, 109.77, 109.63, 109.58, 73.5, 73.2, 73.0, 67.39, 67.35, 67.33, 67.23, 67.18, 66.9, 56.71, 56.68, 56.65, 56.61, 56.6, 56.4, 56.1, 55.4, 41.8, 41.2, 41.1, 41.0, 21.3, 21.1, 21.0, 20.9, 20.8, 20.3, 15.5, 15.4, 15.32, 15.29, 15.0, 14.9; **FT-IR** (neat, ATR): $\tilde{\nu}$ (cm^{-1}) = 2999 (w), 2957 (m), 2925 (m), 2870 (m), 2853 (m), 1734 (w), 1616 (m), 1593 (m), 1492 (s), 1482 (s), 1465 (s), 1410 (s), 1373 (m), 1211 (s), 1140 (m), 1043 (s), 974 (w), 882 (m), 821 (m), 701 (w); **UV/Vis** (CH_2Cl_2): λ_{max} (nm) = 296, 406; **MALDI-TOF** (DCTB): *m/z* $[M]^+$ calcd. for $C_{752}H_{768}N_{48}O_{48}$: 11245.94, found 11245.57. **Elemental analysis** calcd. for $C_{752}H_{768}N_{48}O_{48}\cdot 33CH_2Cl_2$: C 67.11, H 5.98, N 4.79, found C 66.94, H 5.91, N 4.86.

Synthesis of (OMe-cube)$_3$ (improved protocol)

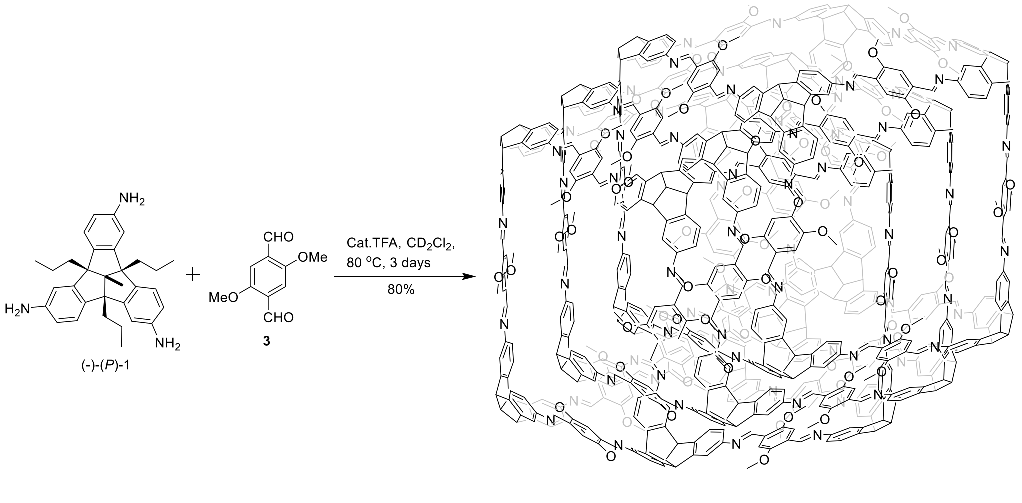

(OMe-cube)$_3$

To a solution of TBTQ **1** (20 mg, 0.043 mmol) and 2,5-dimethoxy-terephthaldehyde **3** (12.6 mg, 0.0649 mmol) in deuterated dichloromethane (4 mL) in a screw-capped 8 mL glass vial a catalytic TFA (0.4 µL, 0.005 mmol) was added and the reaction mixture was stirred at 80°C for 3 days. After cooling to room temperature, the crude reaction mixture was washed with aq. K_2CO_3 solution (0.25 M, 3 × 2 mL) and dried over Na_2SO_4. The solution was concentrated under reduced pressure and the resulting red-colored solid was immediately dissolved in dichloromethane and purified by rGPC (DCM, 30°C, 5 mL/min) to give 24 mg 80% of **(OMe-cube)$_3$** as a yellow solid.

Mp: 310°C (decomposed); **^{1}H NMR** (600 MHz, CD_2Cl_2): δ = 9.31-9.27 (m, 3H, *H*C=N), 9.06-8.98 (m, 12H, *H*C=N), 8.91-8.72 (m, 66H, *H*C=N), 8.61-8.60 (m, 12H, *H*C=N), 8.51 (s, 6H, *H*C=N), 8.41-8.30 (m, 26H, *H*C=N), 8.09-8.06 (m, 6H, Ar-*H*), 7.92-7.86 (m, 18H, Ar-*H*),

7.81-7.76 (m, 30H, Ar-*H*), 7.72-7.63 (m, 26H, Ar-*H*), 7.56-7.52 (m, 12H), 7.46-7.36 (m, 48H), 7.32-7.27 (m, 26H), 7.23-7.13 (m, 93H), 7.06-6.94 (m, 90H), 6.87 (m, 18H), 7.79-6.72 (m, 36H), 6.66-6.54 (m, 72H), 6.39-6.37 (m, 18H), 6.23-6.15 (m, 12H), 6.02-5.90 (m, 16H), 4.08-4.0 (m, 72H, OCH_3), 3.93-3.63 (m, 192H, OCH_3), 3.38-3.15 (m, 96H, OCH_3), 2.55-2.01 (m, 246H), 1.75-1.55 (m, 144H), 1.35-1.27 (m, 266H), 1.03-0.84 (m, 320H), 0.71-0.38 (m, 84H), 0.11(m, 3H);

^{1}H NMR (700 MHz, 375 K, Toluene-d$_8$): δ = 9.36 (s, 6H), 9.30 (s, 6H), 9.26 (s, 6H), 9.09-9.08 (m, 18H), 8.99 (s, 6H), 8.73 (s, 6H), 8.52 (s, 6H), 8.39 (s, 6H), 8.36 (s, 6H), 8.33-8.25 (m, 12H), 8.09 (s, 6H), 8.06 (s, 6H), 8.02-7.98 (m, 24H), 7.90 (s, 6H), 7.75 (br (s), 6H), 7.69 (s, 6H), 7.58-7.42 (m, 54H), 7.28-7.13 (66H), 7.03-7.01 (m, 12H), 6.99-6.98 (m, 12H), 6.92-9.91 (m, 30H), 6.78-6.70 (m, 30H), 6.58-6.55 (m, 12H), 6.41 (br (s) 6H), 6.23 (br (s), 6H), 3.98 (s, 18H), 3.69-3.48 (m, 144H), 3.04-2.84 (m, 54H), 2.63-2.16 (m, 128H), 1.94 (br (s), 18H), 1.86-1.82 (m, 30H), 1.72-1.30 (m, 154H), 1.18 (t, J = 7 Hz, 18H), 1.15 (t, J = 7 Hz, 18H), 1.08 (t, J = 7 Hz, 18H), 1.01 (t, J = 7 Hz, 18H), 0.94 (t, J = 7 Hz, 18H), 0.93-0.87 (m, 102H), 0.83 (t, J = 7 Hz, 18H), 0.74 (t, J = 7 Hz, 18H), 0.69 (s, 6H), 0.66 (br (s), 12H), 0.42 (s, 6H), 0.38 (t, J = 7 Hz, 18H); **FT-IR** (neat, ATR): $\tilde{\nu}$ (cm^{-1}) = 3002 (m), 2959 (m), 2936 (m), 2872 (m), 1614 (s), 1591 (s), 1491 (s), 1464 (s), 1372 (s), 1285 (m), 1140 (m), 1043 (s), 975 (w), 880 (m), 803 (m), 710 (m); **UV/Vis** (CH_2Cl_2): λ_{max} (nm) = 294, 405; **MALDI-TOF** (DCTB): m/z [M]$^+$ calcd. for $C_{1128}H_{1152}N_{72}O_{72}$: 16868.91, found 16868.69. **Elemental analysis** calcd. for $C_{1128}H_{1152}N_{72}O_{72}$ 19CH_2Cl_2: C 74.53, H 6.49, N 5.46, found C 74.15, H 6.64, N 5.42.

Please note: *No decent ^{13}C NMR spectrum despite of recording 12000 scans on a 600 MHz NMR instrument was achieved (see Figure 73).*

Synthesis of H-cube

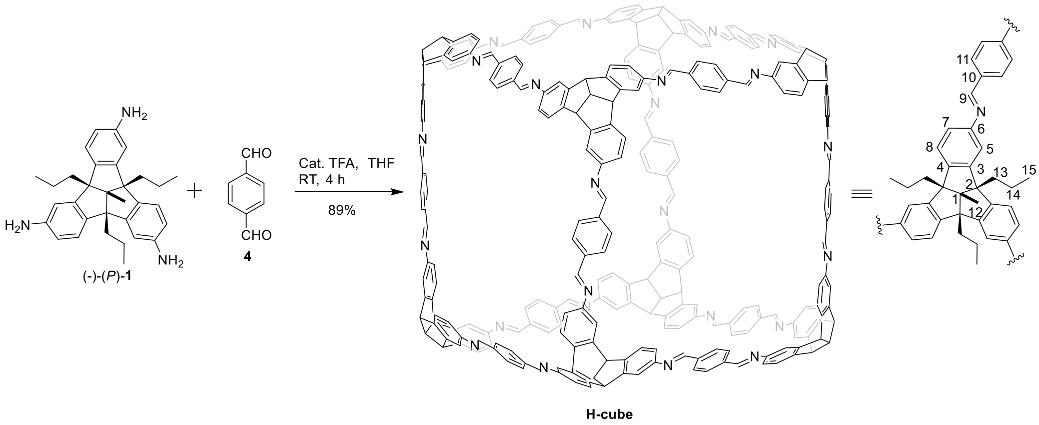

To a solution of TBTQ **1** (10 mg, 0.022 mmol) and terephthaldehyde **4** (4.4 mg, 0.033 mmol) in THF (6 mL) a catalytic amount of TFA (0.2 µL, 0.003 mmol) was added and the reaction

mixture was stirred at RT for 4 hrs. The reaction mixture was diluted to 50 mL with dichloromethane. An aqueous solution of K_2CO_3 (0.25 M, 6 mL) was added to the reaction mixture and stirred for five minutes at RT. The aqueous layer was separated and the organic layer was further washed with water (4 × 20 mL). The organic layer was collected, dried over Na_2SO_4 and concentrated at 5 mL on a rotary evaporator. The resulting solution was again diluted to total volume of 50 mL with methanol and then concentrated on a rotary evaporator. The resulting yellow solid was dissolved in a small amount of dichloromethane and precipitated by the addition of methanol (3 mL). The resulting mixture was ultrasonicated and then the precipitate was separated by centrifugation technique. The yellow solid was dried and further dissolved in a small amount of dichloromethane and reprecipitated by the addition of methanol to give 12 mg (89%) of **H-cube** as a yellow solid.

Mp: 335ºC (decomposed); **^{1}H NMR** (400 MHz, CD_2Cl_2): δ = 8.46 (s, 24H, *H*-9), 7.96 (s, 48H, *H* -11), 7.41 (d, ^{3}J = 8.4 Hz, 24H, *H* -8), 7.20 (d, ^{4}J = 1.6 Hz, 24H, *H* -5), 7.07 (dd, ^{3}J = 8.4 Hz, ^{4}J = 1.6 Hz, 24 H, *H* -7), 2.25 (br (t), 48H, *H* -13) 1.82 (s, 24H, *H* -12), 1.30-1.28 (m, 48H, *H* -14), 0.98 (t, ^{3}J = 6.8 Hz, 72H, *H* -15); **^{13}C NMR** (151 MHz, CD_2Cl_2): δ = 159.0 (*C*-9), 151.7 (*C*-6), 149.6 (*C*-4), 146.7 (*C*-3), 139.1 (*C*-10), 129.3 (*C*-11), 124.5 (*C*-8), 120.3 (*C*-7), 116.7 (*C*-5), 73.4 (*C*-1), 67.3 (*C*-2), 41.0 (*C*-13), 20.9 (*C*-14), 15.5 (*C*-12), 15.3 (*C*-15); **FT-IR** (neat, ATR): $\tilde{\nu}$ (cm^{-1}) = 3023 (w), 2956 (m), 2929 (w), 2869 (w), 1653 (w), 1622 (m), 1577 (w), 1480 (m), 1414 (w), 1295 (w), 1256 (m), 1204 (w), 1155 (w), 955 (s), 889 (w), 829 (m), 757 (m), 715 (s), 678 (w); **UV/Vis** (CH_2Cl_2): λ_{max} (nm) = 293, 363; **MALDI-TOF** (DCTB): *m/z* $[M+H]^+$ calcd. for $C_{352}H_{337}N_{24}$: 4902.72, found 4902.19.

Synthesis of Me-cube

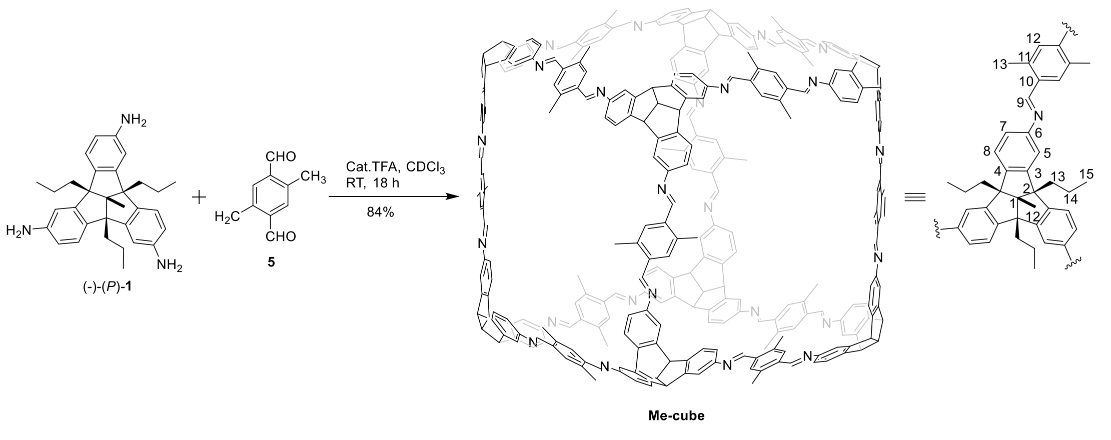

To a solution of TBTQ **1** (10 mg, 0.022 mmol) and 2,5-dimethyl-terephthaldehyde **5** (5.2 mg, 0.032 mmol) in deuterated chloroform (4 mL) in a screw-cap 8 mL glass vial, a catalytic

amount of TFA (0.2 µL, 0.003 mmol) was added and the reaction mixture was stirred at RT for 18 hours. The reaction mixture was concentrated to 1 mL by rotary evaporation and methanol (16 mL) was added. The precipitate was separated by the centrifugation technique. The precipitate was repeatedly dried and dissolved in dichloromethane (3 x 1 mL) and reprecipitated by the addition of methanol to give 11.8 mg (84%) of **Me-cube** as a yellow solid.

Mp: 355°C (decomposed); **^{1}H NMR** (600 MHz, CD_2Cl_2): δ = 8.66 (s, 24H, H-9), 7.89 (s, 24H, H-12), 7.39 (d, ^3J = 7.8 Hz, 24H, H-8), 7.17 (d, ^3J = 1.2 Hz, 24H, H-5), 7.00 (dd, ^3J = 8.4 Hz, ^4J = 1.8 Hz, 24 H, H-7), 2.54 (s, 24H, H-13), 2.23 (br (t), 48H, H-15) 1.70 (s, 24H, H-14), 1.29-1.27 (m, 48H, H-16), 0.97 (t, ^3J = 7.2 Hz, 72H, H-17); **^{13}C NMR** (151 MHz, CD_2Cl_2): δ = 158.1, 152.5, 149.6, 146.3, 136.7, 136.5, 130.1, 124.5, 119.5, 117.2, 73.3, 67.3, 41.1, 20.9, 18.9, 15.5, 15.3; **FT-IR** (neat, ATR): $\tilde{\nu}$ (cm^{-1}) = 2956 (s), 2925 (m), 2869 (m), 1728 (w), 1697 (m), 1618 (s), 1593 (s), 1481(s), 1456 (w), 1402 (w), 1375 (w), 1272 (m), 1186 (m), 1149 (w), 970 (w), 883 (m), 802 (m), 734 (m), 702 (w), 648 (w); **UV/Vis** (CH_2Cl_2): λ_{max} (nm) = 297, 366; **MALDI-TOF** (DCTB): m/z $[M]^+$ calcd. for $C_{376}H_{384}N_{24}$: 5239.09, found 5239.96.

Synthesis of Et-cube

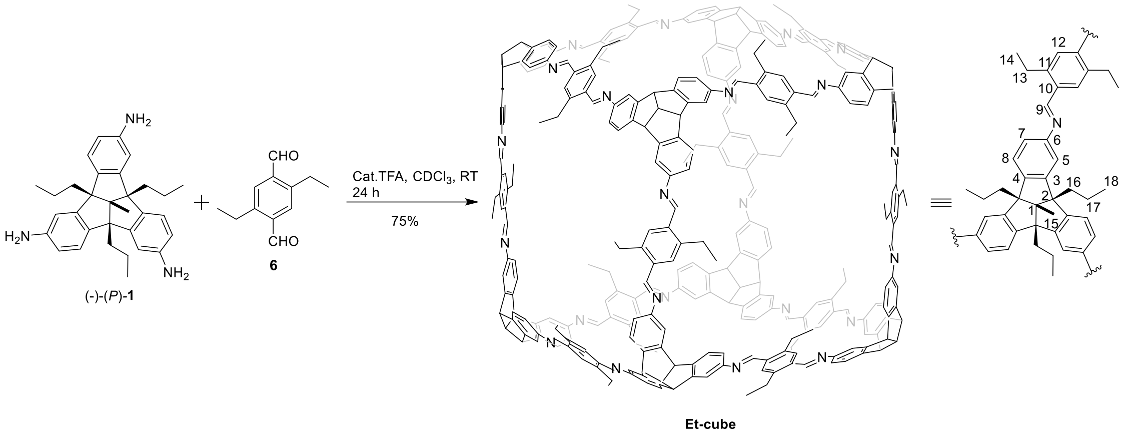

To a solution of TBTQ **1** (10 mg, 0.022 mmol) and 2,5-diethyl-terephthaldehyde **6** (6.1 mg, 0.032 mmol) in deuterated chloroform (4 mL) a catalytic amount of TFA (0.2 µL, 0.003 mmol) was added and the reaction mixture was stirred at RT for 24 hours. The reaction mixture was diluted to a total volume of 20 mL with acetonitrile and the resulting solution was concentrated to 5 mL under reduced pressure. The yellow precipitate was separated by centrifugation technique. The collected yellow precipitate was repeatedly suspended in acetonitrile (3 x 5 mL) sonicated (20 seconds) and separated by the centrifuge technique afforded 11.2 mg (75%) of **Et-cube** as a yellow solid.

Mp: >350°C; **^{1}H NMR** (600 MHz, CD_2Cl_2): δ = 8.68 (s, 24H, *H*-9), 7.96 (s, 24H, *H*-12), 7.40 (d, ^{3}J = 7.8 Hz, 48H, *H*-8), 7.18 (d, ^{3}J = 1.2 Hz, 24H, *H*-5), 6.99 (dd, ^{3}J = 7.2 Hz, ^{4}J = 1.2 Hz, 24 H, *H*-7), 2.92 (q, ^{3}J = 7.2 Hz, 48H, *H*-13), 2.23 (br (t), 48H, *H*-16), 1.70 (s, 24H, *H*-15), 1.29-1.27 (m, 48H, *H*-17), 1.23 (t, ^{3}J = 7.2 Hz, 72H, *H*-14);), 0.98 (t, ^{3}J = 7.2 Hz, 72H, *H*-18); **^{13}C NMR** (151 MHz, CD_2Cl_2): 157.9, 152.7, 149.9, 146.3, 143.2, 136.0, 128.9, 124.5, 119.3, 117.4, 73.4, 67.4, 41.1, 25.8, 20.9, 16.8, 15.5, 15.3; **FT-IR** (neat, ATR): $\tilde{\nu}$ (cm^{-1}) = 2960 (s), 2931 (m), 2871 (m), 1699 (w), 1618 (s), 1593 (s), 1548 (w), 1481(s), 1454 (m), 1411 (w) , 1375 (m), 1299 (w), 1271 (w), 1184 (m), 1151 (m), 1060 (w), 970 (m), 906 (s), 898 (s), 817 (s), 802 (s), 734 (w), 638 (w); **UV/Vis** (CH_2Cl_2): λ_{max} (nm) = 298, 362; **MALDI-TOF** (DCTB): *m/z* $[M]^+$ calcd. for $C_{400}H_{432}N_{24}$: 5576.48, found 5576.76.

Synthesis of (Et-cube)$_2$

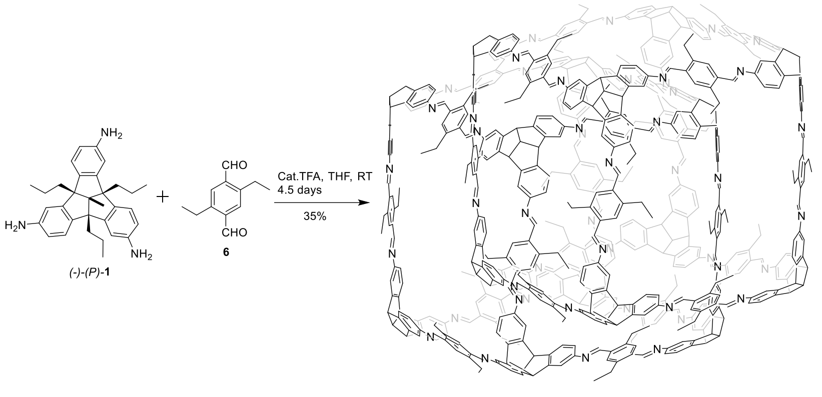

To a solution of TBTQ **1** (10 mg, 0.022 mmol) and 2,5-diethyl-terephthaldehyde **6** (6.1 mg, 0.032 mmol) in THF (2 mL), a catalytic amount of TFA (0.2 µL, 0.0026 mmol) was added and the reaction mixture was stirred at RT for 4½ days. Afterward, the crude reaction mixture was concentrated under reduced pressure and the resulting solid was immediately dissolved in chloroform and purified by rGPC ($CHCl_3$, 40°C, 3.5 mL/min) to give 5.2 mg (35%) of **(Et-cube)$_2$** as a yellow solid.

Mp: 240°C (decomposed); **^{1}H NMR** (600 MHz, CD_2Cl_2): δ = 8.77 (s, 6H, *H*C=N), 8.69 (s, 6H, *H*C=N), 8.67 (s, 12H, *H*C=N), 8.62 (s, 6H, *H*C=N), 8.59 (s, 6H, *H*C=N), 8.33 (s, 6H, *H*C=N), 8.25 (s, 6H, *H*C=N), 8.18-8.17 (12H, Ar-*H*), 7.97 (s, 6H, Ar-*H*), 7.96 (s, 6H, Ar-*H*), 7.94 (s, 6H, Ar-*H*), 7.93 (s, 6H, Ar-*H*), 7.72 (s, 6H, Ar-*H*), 7.48 (s, 6H, Ar-*H*), 7.44-7.41 (12H, TBTQ-Ar-*H*), 7.39-7.32 (18H, TBTQ-Ar-*H*), 7.23 (s, 6H, TBTQ-Ar-*H*), 7.22 (s, 6H, TBTQ-Ar-*H*), 7.20 (s, 6H, TBTQ-Ar-*H*), 7.18 (s, 6H, TBTQ-Ar-*H*), 7.15 (s, 6H, TBTQ-Ar-*H*), 7.10 (s, 6H, TBTQ-Ar-*H*), 7.02-7.00 (18H, TBTQ-Ar-*H*), 6.96 (d, ^3J = 7.8 Hz, 6H, TBTQ Ar-*H*), 6.92 (s, 6H, TBTQ-Ar-*H*), 6.89 (d, ^3J = 7.8 Hz, 6H, TBTQ Ar-*H*), 6.88 (s, 6H, TBTQ-Ar-*H*), 6.79 (d, ^3J = 8.4 Hz, 6H, TBTQ Ar-*H*), 6.67 (d, ^3J = 7.8 Hz, 6H, TBTQ Ar-*H*), 6.62 (d, ^3J = 8.4 Hz, 6H, TBTQ Ar-*H*), 6.49 (d, ^3J = 8.4 Hz, 6H, TBTQ Ar-*H*), 6.33 (d, ^3J = 7.8 Hz, 6H, TBTQ Ar-*H*), 5.98 (d, ^3J = 7.8 Hz, 6H, TBTQ Ar-*H*), 3.10-2.81 (m, 82H, -CH_2CH$_3$), 2.54-242 (m, 14H, -CH_2CH$_3$), 2.36-2.04 (m, 84H, -CH_2CH$_2$CH$_3$), 1.84-1.66 (m, 48H), 1.60 (q, ^3J = 7.2 Hz, 12H, -CH$_2$CH_2CH$_3$), 1.32 (t, ^3J = 7.8 Hz, 18H, -CH$_2$CH$_2$CH_3), 1.29-1.24 (m, 114H), 1.18 (t, ^3J = 7.2 Hz, 18H, -CH$_2$CH_3), 1.14 (t, ^3J = 7.2 Hz, 18H, -CH$_2$CH_3), 1.05 (t, ^3J = 7.2 Hz, 18H, -CH$_2$CH_3), 1.02-0.94 (m, 108H), 0.89-0.81 (m, 36H), 0.74 (t, ^3J = 7.8 Hz, 36H, -CH$_2$CH_3), 0.49 (t, ^3J = 7.2 Hz, 18H), -0.27 (t, ^3J = 7.8 Hz, 18H, -CH$_2$CH$_2$CH_3); **^{13}C NMR** (151 MHz, $CDCl_3$): 158.6, 158.3, 157.9, 157.8, 157.7, 157.6, 157.3, 154.1, 153.6, 153.0, 152.6, 152.6, 152.6, 152.5, 152.3, 152.3, 150.5, 149.8, 149.7, 149.7, 149.5, 149.4, 146.5, 146.4, 146.0, 146.0, 145.8, 145.6, 144.7, 143.3, 143.2, 143.0, 142.9, 142.7, 136.9, 136.1,

136.0, 135.9, 135.7, 135.6, 129.0, 128.8, 128.5, 127.9, 124.4, 124.2, 124.2, 119.2, 119.0, 118.1, 117.8, 117.5, 117.3, 116.7, 115.8, 73.5, 73.2, 73.1, 67.5, 67.4, 67.3, 67.3, 67.2, 67.0, 66.9, 41.7, 41.5, 41.5, 41.5, 41.1, 41.0, 40.9, 26.0, 26.0, 25.8, 25.7, 25.6, 24.8, 21.4, 21.1, 21.0, 20.9, 20.4, 16.9, 16.8, 16.7, 16.7, 16.4, 16.3, 16.3, 15.5, 15.4, 15.3, 15.3, 15.2, 15.1, 15.0; **FT-IR** (neat, ATR): $\tilde{\nu}$ (cm^{-1}) = 2961 (s), 2930 (m), 2872 (m), 1618 (s), 1593 (s), 1549 (w), 1481 (s), 1456 (m), 1414 (m), 1375 (m), 1296 (w), 1271 (w), 1240 (w), 1184 (m), 1150 (w), 1111 (w), 1101(w), 1061 (w), 970 (m), 953 (w), 910 (m), 878 (m), 820 (m), 802 (m), 781 (w), 712 (w), 677 (w), 642 (w); **UV/Vis** (CH_2Cl_2): λ_{max} (nm) = 297, 361; **MALDI-TOF** (DCTB): *m/z* $[M+H]^+$ calcd. for $C_{800}H_{865}N_{48}$: 11151.94, found 11152.45.

Synthesis of OEt-cube

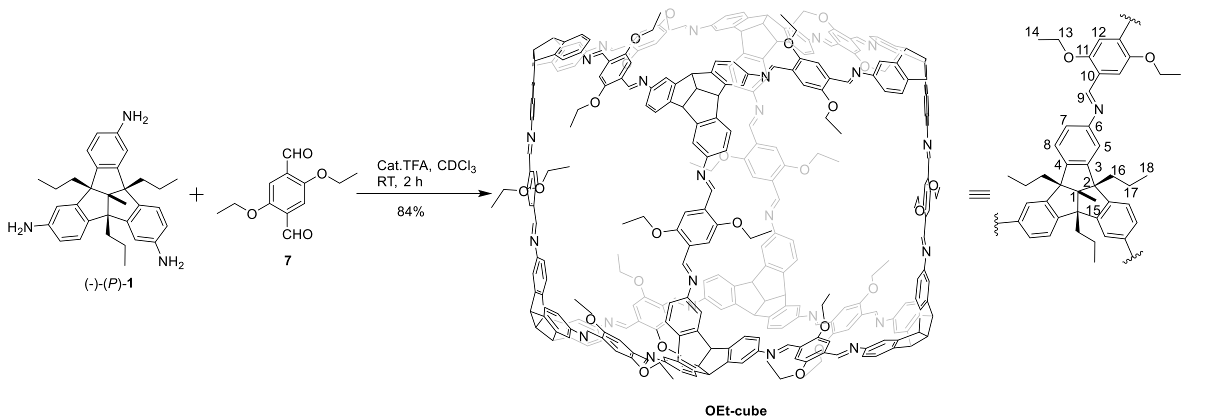

To a solution of TBTQ **1** (5 mg, 0.01 mmol) and 2,5-diethoxy-terephthaldehyde **7** (3.6 mg, 0.016 mmol) in deuterated chloroform (5 mL), a catalytic amount of TFA (0.1 µL, 0.001 mmol) was added and the reaction mixture was stirred for 4 hours at RT. Afterward, the crude reaction mixture was purified by r-GPC ($CHCl_3$) and then the collected fractions (25 mL) were diluted to a total volume of 50 mL with acetonitrile. The solvents were removed under reduced pressure to a volume of 10 mL and diluted again to 50 mL with acetonitrile before solvents were completely removed under vacuum to give 6.7 mg (84%) of **OEt-cube** as a yellow solid.

Mp: 345°C (decomposed); **^{1}H NMR** (600 MHz, $CDCl_3$): δ = 8.83 (s, 24H), 7.66 (s, 24H), 7.37 (d, ^3J = 7.8 Hz, 24H), 7.19 (s, 24H), 6.98 (d, ^3J = 7.8 Hz, 24 H), 4.14 (m, 48H), 2.21 (br (t), 48H), 1.67 (s, 24H), 1.38 (t, ^3J = 7.2 Hz, 72H), 1.25 (br (m), 48H), 0.95 (t, ^3J = 7.2 Hz, 72H) ; **FT-IR** (neat, ATR): $\tilde{\nu}$ (cm^{-1}) = 2956 (m), 2923 m), 2869 (w), 1647 (w), 1616 (w), 1558 (w), 1541 (m), 1529 (m), 1508 (m), 1488 (s), 1473 (s), 1456 (m), 1427 (s), 1396 (w), 1361 (w), 1286 (w), 1203 (s), 1110 (w), 1045 (s), 974 (w), 881 (w); **UV/Vis** ($CHCl_3$): λ_{max}

(nm) = 296, 407; **MALDI-TOF** (DCTB): *m/z* $[M+H]^+$ calcd. for $C_{400}H_{432}N_{24}O_{24}$: 5959.35, found 5959.77.1

***Please note: OEt-cube** is also forming catenane at higher concentration like **OMe-cube**. Therefore, no ^{13}C NMR spectrum was recorded.*

Synthesis of (OEt-cube)$_2$

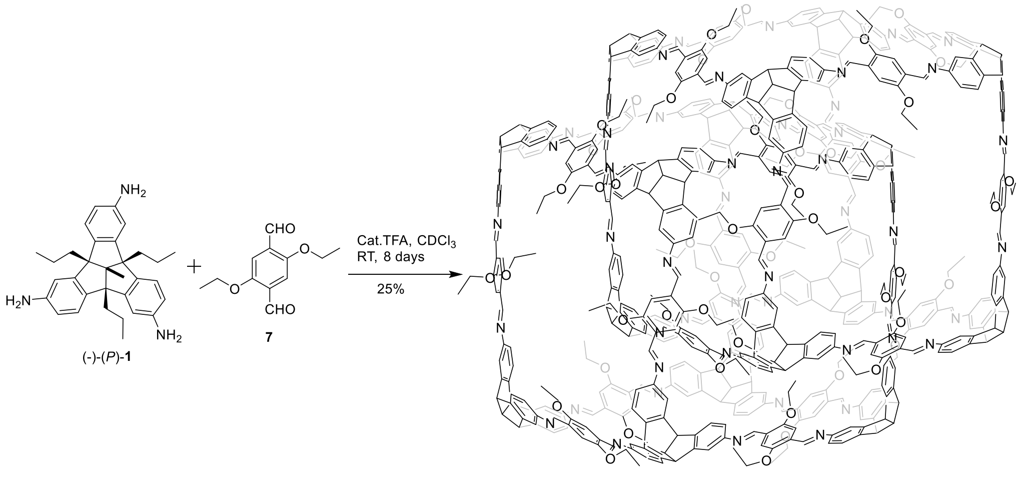

To a solution of TBTQ **1** (10 mg, 0.022 mmol) and 2,5-diethoxy-terephthaldehyde **7** (7.2 mg, 0.032 mmol) in deuterated chloroform (2 mL), a catalytic amount of TFA (0.2 µL, 0.003 mmol) was added and the reaction mixture was stirred at RT for 7 days. Afterward, the crude reaction mixture was concentrated to 1 mL and purified by r-GPC ($CHCl_3$, 40°C, 5 mL/min) to give 4 mg (25%) of **(OEt-cube)$_2$** as yellow solid.

Mp: 320°C (decomposed); **^{1}H NMR** (600 MHz, CD_2Cl_2): δ = 8.95 (s, 6H, *H*C=N), 8.92 (s, 6H, *H*C=N), 8.85 (s, 6H, *H*C=N), 8.81 (12H, *H*C=N), 8.78 (s, 6H, *H*C=N), 8.68 (s, 6H, *H*C=N), 8.41 (s, 6H, *H*C=N), 8.17 (s, 6H, Ar-*H*), 7.84 (s, 6H, Ar-*H*), 7.72 (s, 6H, Ar-*H*), 7.70 (s, 6H, Ar-*H*), 7.65 (s, 6H, Ar-*H*), 7.64 (s, 6H, Ar-*H*), 7.62 (s, 6H, Ar-*H*), 7.52 (d, ^3J = 7.8 Hz, 6H, TBTQ Ar-*H*), 7.46 (s, 6H, TBTQ-Ar-*H*), 7.39-7.37 (12H, Ar-*H*), 7.33 (d, ^3J = 8.4 Hz, 6H, TBTQ Ar-*H*), 7.30 (d, ^3J = 7.8 Hz, 6H, TBTQ Ar-*H*), 7.25 (s, 6H, Ar-*H*), 7.19-7.14 (36H, TBTQ-Ar-*H*), 7.02-6.93 (30H, TBTQ-Ar-*H*), 6.87 (s, 6H, TBTQ-Ar-*H*), 6.80 (d, ^3J = 8.4 Hz, 6H, TBTQ Ar-*H*), 6.71 (s, 6H, TBTQ-Ar-*H*), 6.57-6.55 (m, 12H, TBTQ-Ar-*H*), 6.47 (d, ^3J = 7.8 Hz, 6H, TBTQ Ar-*H*), 6.23 (d, ^3J = 8.4 Hz, 6H, TBTQ Ar-*H*), 4.29-4.03 (m, 60H, OCH_2CH_3), 3.75-3.51 (m, 36H, OCH_2CH_3), 2.23-2.12 (m, 72H, $-CH_2CH_2CH_3$), 1.95 (br (t), 12H, $-CH_2CH_2CH_3$), 1.77-1.64 (m, 60H, $-CH_2CH_2CH_3$ & TBTQ-CH_3), 1.51 (t, ^3J = 7.2 Hz, 18H, -OCH2CH_3), 1.47 (t, ^3J = 7.2 Hz, 18H, -OCH2CH_3), 1.39-1.26 (m, 138H, $-OCH_2CH_3$ & $-CH_2CH_2CH_3$), 0.98-0.83 (m, 162H, $-OCH_2CH_3$ & $-CH_2CH_2CH_3$), 0.76-0.74 (m, 30H, -

OCH_2CH_3 & -$CH_2CH_2CH_3$), 0.49 (t, ^3J = 7.2 Hz,18H, -$CH_2CH_2CH_3$); **FT-IR** (neat, ATR): $\tilde{\nu}$ (cm^{-1}) = 2956 (s), 2923 (s), 2854 (m), 1728 (w), 1616 (m), 1593 (m), 1483 (s), 1427 (s), 1393 (m), 1373 (m), 1280 (m), 1203 (s), 1141 (w), 1110 (m), 1047 (s), 972 (w), 883 (w), 804 (w), 707 (w); **UV/Vis** (CH_2Cl_2): λ_{max} (nm) = 297, 400; **MALDI-TOF** (DCTB): m/z $[M+H]^+$ calcd. for $C_{800}H_{865}N_{48}O_{48}$: 11920.70, found 11920.88.

Please note: *No decent ^{13}C NMR spectrum despite of recording 4096 scans on a 600 MHz NMR instrument was achieved.*

Synthesis of O^{i}Pr-cube

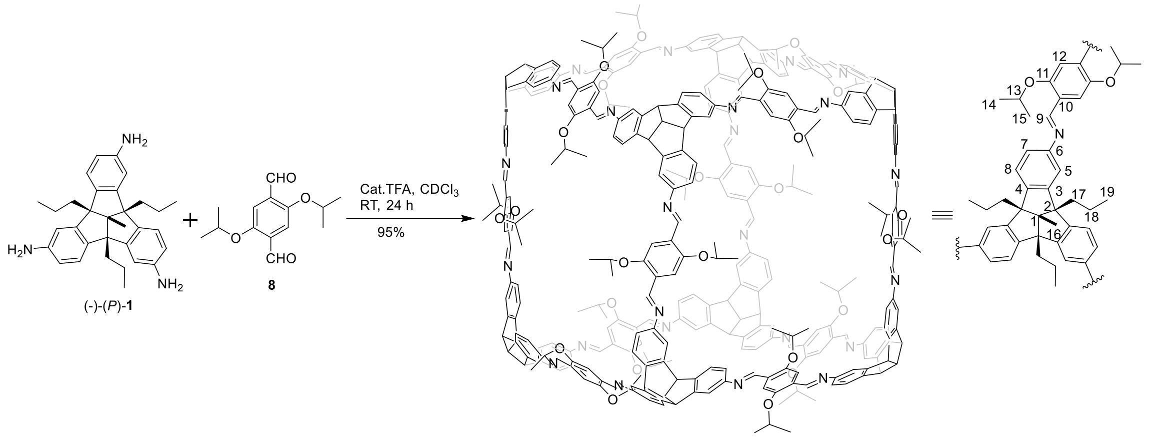

O^{i}Pr-cube

To a solution of TBTQ **1** (10 mg, 0.0215 mmol) and 2,5-isopropoxy-terephthaldehyde **8** (8.1 mg, 0.032 mmol) in chloroform (deuterated, 2 mL), a catalytic amount of TFA (0.2 µL, 0.003 mmol) was added and the reaction mixture was stirred at RT for 24 hours. The reaction mixture was concentrated to 1 mL under reduced pressure and then purified by r-GPC (chloroform, 40°C, 3.5 mL/min) to give 16.1 mg (95%) of **O^{i}Pr-cube** as yellow solid.

Mp: 328°C (decomposed); **^{1}H NMR** (600 MHz, $CDCl_3$): δ = 8.80 (s, 24H, *H*-9), 7.69 (s, 24H, *H*-12), 7.37 (d, ^3J = 8.4 Hz, 24H, *H*-8), 7.18 (s, 24H, *H*-5), 6.97 (dd, ^3J = 7.8 Hz, ^4J = 1.2 Hz, 24 H, *H*-7), 4.67 (m, 24, *H*-13), 2.21 (br (t), 48H, *H*-17), 1.67 (s, 24H, *H*-16), 1.31 (d, ^3J = 6 Hz, 72H, *H*-14), 1.29 (d, ^3J = 6 Hz, 72H, *H*-15), 1.26 (br (m), 48H, *H*-18), 0.96 (t, ^3J = 7.2 Hz, 72H, *H*-19); **^{13}C NMR** (151 MHz, $CDCl_3$): 155.8, 152.4, 152.1, 149.2, 146.1, 129.8, 124.2, 119.2, 117.5, 113.0, 73.1, 72.0, 67.0, 41.1, 22.33, 22.26, 20.7, 15.4, 15.4; **FT-IR** (neat, ATR): $\tilde{\nu}$ (cm^{-1}) = 2972 (s), 2961 (s), 2924 (s), 2872 (m), 2818 (w), 2359 (w), 2341(w), 2330 (w), 1616 (m), 1591 (m), 1489 (s), 1479 (s), 1423 (s), 1373 (m), 1335(w), 1277 (m), 1196 (m) ,1134 (m), 1109 (s), 1074 (m), 1067 (m), 1040 (w), 972 (m), 935 (m), 910 (m), 891 (w), 872 (w), 843 (w), 824 (w), 802(w),793 (w), 735 (m), 650 (w), 608 (m), 607.55 (m); **UV/Vis** ($CHCl_3$): λ_{max} (nm) = 297, 351, 405; **MALDI-TOF** (DCTB): *m/z* $[M+H]^+$ calcd. for $C_{424}H_{481}N_{24}O_{24}$: 6296.73, found 6297.58.

Synthesis of SMe-cube

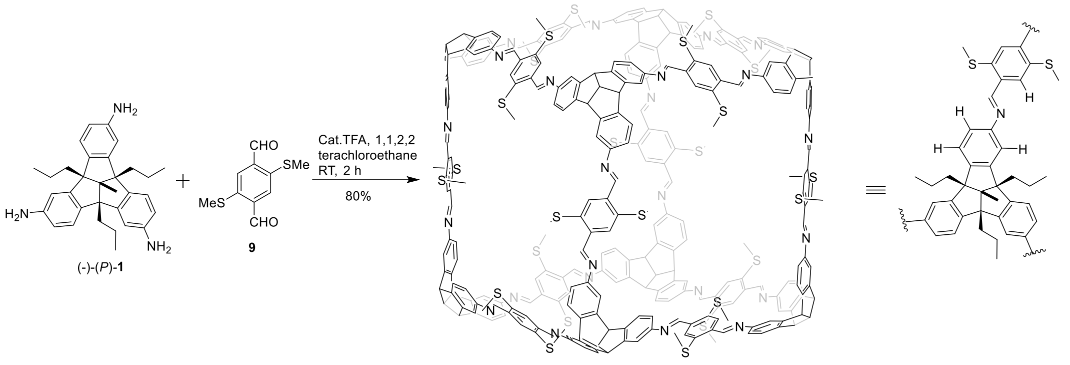

To a solution of TBTQ **1** (5.3 mg, 0.011 mmol) and 2,5-bis(methylthio)terephthalaldehyde) **9** (3.6 mg, 0.016 mmol) in 1,1,2,2 tetrachloroethane (anhydrous, 2 mL), a catalytic amount of TFA (0.1 µL, 0.001 mmol) was added and the reaction mixture was stirred at RT for 2 hours. The saturated solution of aq. $NaHCO_3$ (2 mL) was added to the reaction mixture and it was further stirred at RT for 10 minutes. The organic layer was separated, washed with water (4 x 3 mL) and precipitated by adding methanol into the organic layer (6 mL), and left at RT for 4 hours. The yellow precipitate was separated by centrifugation technique, repeatedly washed with methanol (4 x 4 mL) and dried to give 6.4 mg (80%) of **SMe-cube** as a yellow solid.

Mp: 347°C (decomposed); **^{1}H NMR** (700 MHz, $Cl_2CDCDCl_2$): δ = 8.87 (s, 24H), 8.01 (s, 24H), 7.40 (d, J = 7.0 Hz, 24H), 7.23 (s, 24 H), 7.08 (d, J = 7.7 Hz, 24 H), 2.53 (s, 72H), 2.24 (br (t), 48H) 1.69 (s, 24H), 1.31 (br (m), 48H), 0.99 (t, J = 6.3 Hz, 72H; **FT-IR** (neat, ATR): $\tilde{\nu}$ (cm^{-1}) = 2959 (m), 2922 (w), 2869 (w), 1616 (m), 1591 (m), 1481 (s), 1433 (m), 1359 (m), 1315 (w), 1267 (w), 1201 (w), 1143 (w), 1099 (m), 1016 (w), 968 (m), 879 (m), 817 (w), 790 (s), 752 (w), 736 (s), 713 (w), 644 (m); **UV/Vis** (CH_2Cl_2): λ_{max} (nm) = 274, 369; **TIMS-TOF Flex** (DCTB): m/z [M+Na+4(O)]$^+$ calcd. for $C_{376}H_{384}N_{24}S_{24}O_4Na$: 6095.39, found 6095.41.

Note:

1. A decent ^{13}C NMR spectrum was not available, even by recording 8192 scans on a 700 MHz NMR instrument (see Figure 185). It was also observed that the cage shows decomposition over time in deuterated 1,1,2,2 tetrachloroethane due to the acidic nature of solvent.

2. The mass spectrum shows several additional peaks due to oxidation of sulphur from the cage (See Figure 338).

Synthesis of (SMe-cube)$_2$

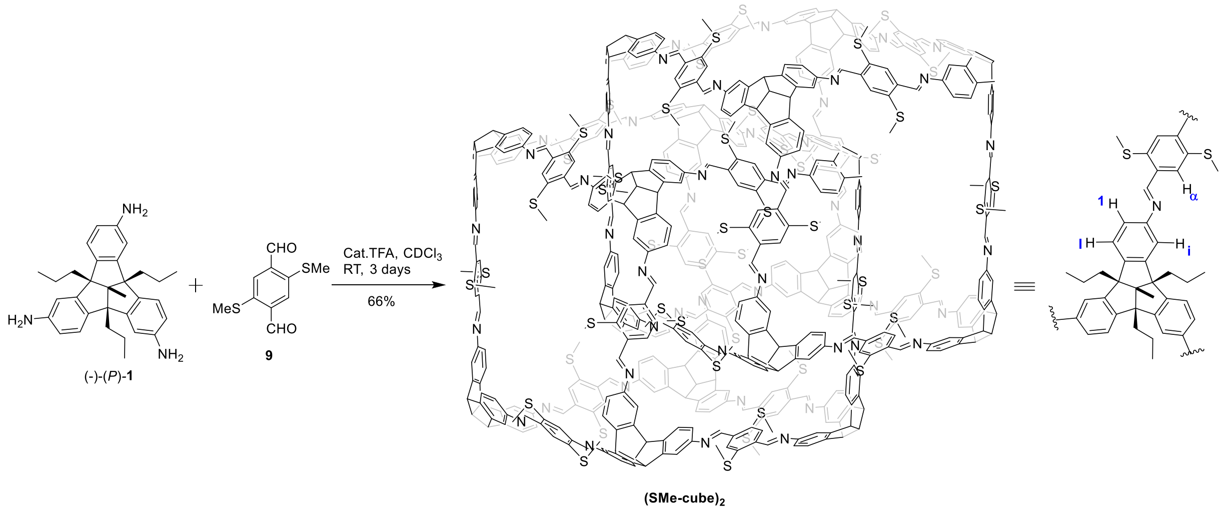

To a solution of TBTQ **1** (10 mg, 0.022 mmol) and 2,5-bis(methylthio)terephthalaldehyde **9** (7.3 mg, 0.032 mmol) in deuterated chloroform (2 mL), a catalytic amount of TFA (0.2 µL, 0.003 mmol) was added and the reaction mixture was stirred at RT for 3 days. Afterward, the crude reaction mixture was concentrated under reduced pressure and the resulting solid was immediately dissolved in chloroform and purified by r-GPC ($CHCl_3$, 40°C, 3.5 mL/min) to give 10.7 mg (66%) of **(SMe-cube)$_2$** as a yellow solid.

Mp: 332°C (decomposed); **^{1}H NMR** (600 MHz, CD_2Cl_2): δ = 9.02 (s, 6H, *H*C=N), 8.85-8.84 (24H, *H*C=N), 8.80 (s, 6H, *H*C=N), 8.59 (s, 6H, *H*C=N), 8.46 (s, 6H, *H*C=N), 8.25 (s, 6H, Ar-*H*), 8.20 (s, 6H, Ar-*H*), 8.10 (s, 6H, Ar-*H*), 8.07 (s, 6H, Ar-*H*), 8.02 (s, 6H, Ar-*H*), 7.98 (s, 6H, Ar-*H*), 7.80 (s, 6H, Ar-*H*), 7.62 (s, 6H, Ar-*H*), 7.56 (d, ^3J = 8.4 Hz, 6H, TBTQ Ar-*H*), 7.54 (s, 6H, TBTQ Ar-*H*), 7.41-7.36 (24H, TBTQ Ar-*H*), 7.27 (s, 6H, TBTQ-Ar-*H*), 7.23 (s, 6H, TBTQ-Ar-*H*), 7.20 (s, 6H, TBTQ-Ar-*H*), 7.12 (s, 12H, TBTQ-Ar-*H*), 7.05-7.00 (m, 36H, TBTQ-Ar-*H*), 6.93 (d, ^3J = 8.4 Hz, 6H, TBTQ-Ar-*H*), 6.81 (s, 6H, TBTQ-Ar-*H*), 6.66 (d, ^3J = 7.2 Hz, 6H, TBTQ-Ar-*H*), 6.61 (d, ^3J = 7.8 Hz, 6H, TBTQ-Ar-*H*), 6.55(d, ^3J = 7.8 Hz, 6H, TBTQ-Ar-*H*), 6.33 (brs, 6H, TBTQ-Ar-*H*), 6.28 (d, ^3J = 7.8 Hz, 6H, TBTQ-Ar-*H*), 2.62 (s, 18H, SCH_3), 2.51 (s, 18H, SCH_3), 2.46 (s, 18H, SCH_3), 2.42 (s, 18H, SCH_3), 2.38 (s, 18H, SCH_3), 2.27-2.10 (m, 102H, SCH_3 & -CH_2CH$_2$CH$_3$), 1.96 (s, 18H, SCH_3), 1.76-1.67 (m, 60H, -CH_2CH$_2$CH$_3$ & -CH_3), 1.30-1.26 (m, 72H, -CH$_2$CH_2CH$_3$), 1.12-1.10 (m, 30H, SCH_3 & -CH_2CH$_2$CH$_3$), 0.99-0.95 (m, 108H, -CH$_2$CH$_2$CH_3), 0.84 (t, ^3J = 7.2 Hz, 9H, -CH$_2$CH$_2$CH_3), 0.72-0.69 (m, 12H, -CH$_2$CH_2CH$_3$), 0.58 (t, ^3J = 7.2 Hz, 9H, -CH$_2$CH$_2$CH_3); **^{13}C NMR** (151 MHz, $CDCl_3$): 158.0, 157.5, 157.3, 156.8, 156.6, 156.5, 156.2, 153.3, 152.8, 152.1, 151.7, 151.6, 150.8, 149.8, 149.7, 149.6, 149.6, 149.4, 146.9, 146.8, 146.5, 146.1, 146.0, 145.8, 145.7, 138.9, 138.8, 138.3, 138.1, 138.0, 137.9, 137.8, 137.6137.5, 136.9, 136.8, 136.7, 136.5,

136.2, 136.1, 129.5, 128.0, 127.9, 127.8, 127.4, 127.3, 126.4, 126.3, 125.0, 124.9, 124.5, 124.4, 124.3, 124.2, 124.1, 120.4, 120.3, 119.7, 118.8, 188.8, 118.6, 118.5, 118.4, 117.5, 117.5, 117.2, 73.4, 73.2, 67.6, 67.5, 67.4, 67.4, 67.3, 67.1, 67.1, 67.0, 41.9, 41.8, 41.6, 41.4, 41.4, 41.3, 41.2, 41.2, 41.2, 41.1, 41.1, 41.0, 40.9, 40.6, 40.5, 21.5, 21.2, 21.0, 20.9, 20.8, 20.3, 17.8, 17.5, 17.4, 17.1, 17.0, 16.7, 16.5, 16.5, 15.5, 15.4, 15.3, 15.3, 15.3, 15.2, 15.1; **FT-IR** (neat, ATR): $\tilde{\nu}$ (cm^{-1}) = 3684 (w), 3674 (w), 2988 (m), 2972 (m), 2901 (m), 1452 (w), 1406 (w), 1394 (w), 1383 (w), 1259 (w), 1242 (w), 1215 (m), 1074 (s), 1067 (s), 1055 (s), 1028 (m), 928 (w), 893 (w), 879 (w), 870 (w), 748 (s), 669 (m), 629 (w), 611 (w); **UV/Vis** (CH_2Cl_2): λ_{max} (nm) = 277, 369; **MALDI-TOF** (DCTB): *m/z* $[M+H]^+$ calcd. for $C_{752}H_{769}N_{48}S_{48}$: 12017.84, found 12017.64. **Elemental analysis** calcd. for $C_{781}H_{826}N_{48}S48\cdot29CH_2Cl_2$: C 64.78, H 5.75, N 4.64, found C 64.49, H 5.64, N 4.40.

Synthesis of (SMe-cube)$_3$

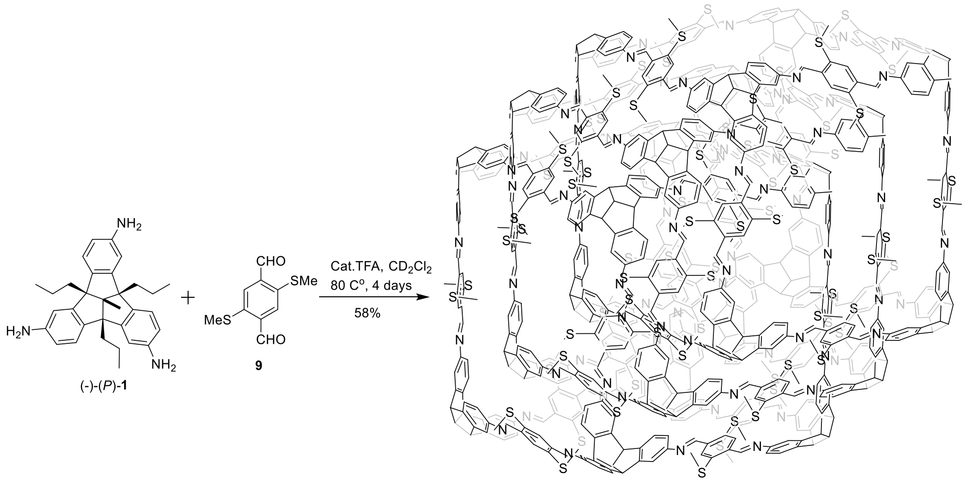

To a solution of TBTQ **1** (15.4 mg, 0.0331 mmol) and 2,5-bis(methylthio)terephthalaldehyde) (10.9 mg, 0.0482 mmol) in deuterated dichloromethane (3 mL) in a screw-capped 8 mL glass vial a catalytic TFA (0.3 µL, 0.0038 mmol) was added and the reaction mixture was stirred at 80°C for 4 days. After cooling down to room temperature, the crude reaction mixture was stirred with aq. K_2CO_3 solution (0.25 M, 3 × 2 mL) for 10 minutes, the organic layer was separated and dried over Na_2SO_4. The solution was concentrated under reduced pressure and the resulting yellow solid was immediately dissolved in dichloromethane and purified by rGPC (THF, 40°C, 3 mL/min) to give 14 mg (58%) of **(SMe-cube)$_3$** as a yellow solid. (Note: BHT from the cage was removed by pentane wash).

Mp: 299°C (decomposed); **^{1}H NMR** (700 MHz, 295 K, CD_2Cl_2): δ = 9.91-8.76 (m, 48H), 8.46-7.99 (m, 72H), 7.71-7.21 (m, 90H), 7.11-6.26 (m, 150H), 2.69-2.11 (m, 264H), 1.76-

0.28 (m, 456H); **^{13}C NMR** (176 MHz, 295 K, CD_2Cl_2): δ = 158.2, 157.9, 157.5, 157.0, 156.6, 156.3, 156.0, 153.4, 152.6, 152.0, 151.5, 150.1, 149.9, 149.8, 149.5, 149.1, 147.2, 146.8, 145.9, 145.6, 145.3, 144.8, 139.8, 138.2, 138.1, 138.0, 137.4, 137.0, 136.8, 136.6, 136.5, 135.8, 130.5, 128.0, 127.5, 127.3, 126.4, 125.6, 125.0, 124.8, 124.6, 124.4, 124.2, 123.9, 120.5, 120.0, 119.6, 118.7, 118.1, 117.0, 115.9, 115.0, 73.31, 73.26, 73.2, 72.83, 72.79, 68.1, 67.6, 67.5, 67.4, 67.3, 67.2, 67.1, 66.8, 42.1, 41.6, 41.4, 41.1, 40.9, 40.1, 21.5, 21.3, 21.0, 20.95, 20.90, 20.8, 20.7, 20.2, 17,9, 17.5, 17.4, 17.2, 17.1, 16.4, 15.54, 15.52, 15.39, 15.36, 15.33, 15.30, 15.27, 15.2, 15.03, 15.01*;*

^{1}H NMR (700 MHz, 410 K, $C_2D_2Cl_4$): δ = 9.11-9.09 (m, 18H), 9.03-9.01 (m, 12H), 8.96 (s, 6H), 8.90 (s, 6H), 8.88 (s, 6H), 8.53 (s, 6H), 8.50 (s, 6H), 8.40 (s, 6H), 8.37-8.36 (m, 12H), 8.31 (s, 6H), 8.29 (s, 6H), 8.282 (s, 6H), 8.275 (s, 6H), 8.20 (s, 6H), 8.17 (s, 6H), 8.16 (s, 6H), 7.79 (s, 6H), 7.74 (br (s), 6H), 7.71 (s, 6H), 7.61 (d, *J* = 8.4 Hz, 6 H), 7.59-7.57 (m, 12H), 7.47 (d, *J* = 8.4 Hz, 6 H), 7.42 (d, *J* = 7.7 Hz, 6 H), 7.40-7.38 (m, 18H), 7.34 (s, 6H), 7.31-7.30 (m, 12H), 7.26 (s, 6H), 7.18 (s, 6H), 7.15-7.13 (m, 12H), 7.10 (d, *J* = 8.4 Hz, 6 H), 7.04 (br (s), 18H), 7.00 (d, *J* = 7.7 Hz, 6 H), 6.96-6.89 (m, 30H), 6.82-6.78 (m, 36H), 6.64 (d, *J* = 7.7 Hz, 6 H), 6.52-6.31 (m, 24H), 6.22 (d, *J* = 8.4 Hz, 6 H), 2.79 (s, 18H), 2.73 (s, 18H), 2.64 (br (s) 12H), 2.60 (s, 18H), 2.55 (s, 18H), 2.49 (s, 18H), 2.48 (s, 18H), 2.39-2.19 (m, 132H), 1.99-1.96 (m, 24H), 1.81-1.74 (m, 86H), 1.52-1.25 (m, 160H), 1.15 (t, *J* = 7.7 Hz, 18H), 1.11-1.06 (m, 108H), 0.96 (t, *J* = 7.7 Hz, 18H), 0.92 (t, *J* = 7.0 Hz, 18H), 0.89-0.87 (m, 30H), 0.72 (t, *J* = 7.7 Hz, 18H), 0.69-0.59 (m, 36H), 0.42 (s, 6H), 0.34 (t, *J* = 7.0 Hz, 18H); **FT-IR** (neat, ATR): $\tilde{\nu}$ (cm^{-1}) = 2956 (m), 2922 (w), 2869 (w), 1616 (m), 1591 (m), 1483 (m), 1433 (m), 1363 (w), 1263 (m), 1143 (w), 1099 (m), 968 (m), 893 (m), 802 (m), 734 (s), 698 (m); **UV/Vis** (CH_2Cl_2): λ_{max} (nm) = 276, 366; **MALDI-TOF** (DCTB): m/z $[M+Na+O]^+$ calcd. for $C_{1128}H_{1152}N_{72}S_{72}ONa$: 18064.24, found 18063.63.

Synthesis of ^{15}N labelled *(SMe-cube)$_3$

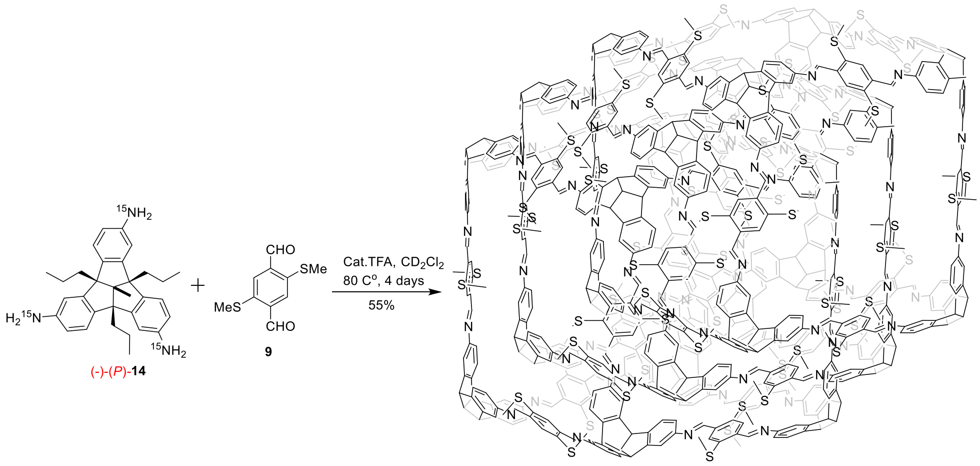

To a solution of TBTQ **14** (10.3 mg, 0.022 mmol) and 2,5-bis(methylthio)terephthalaldehyde) (7.2 mg, 0.032 mmol) in dichloromethane (deuterated, 2 mL) in a screw-capped 8 mL glass vial a catalytic TFA (0.2 µL, 0.003 mmol,) was added and the reaction mixture was stirred at 80°C for 4 days. After cooling down to room temperature, the crude reaction mixture was stirred with aq. K_2CO_3 solution (0.25 M, 3 × 2 mL) for 10 minutes. The organic layer was separated, dried over Na_2SO_4 and concentrated under reduced pressure. The resulting yellow solid was immediately dissolved in dichloromethane and purified by rGPC (THF, 40°C, 3 mL/min) to give 8.8 mg (55%) of ***(SMe-cube)$_3$** as a yellow solid. (Note: BHT from the cage was removed by pentane wash).

Mp: 332 ^{o}C (decomposed); **^{1}H NMR** (700 MHz, 380K Toluene-d_8): 9.50 (br (d), 12H), 9.37 (br (d), 6H), 9.23 (br (d), 6H), 9.18 (d, *J* = 2.8 Hz, 6H), 9.15 (d, *J* = 2.8 Hz, 6H), 8.99 (br (d), 6H), 8.95 (br (d), 6H), 8.75-8.73 (m, 12H), 8.54-8.48 (m, 42H), 8.40 (s, 6H), 8.37 (s, 6H), 8.12 (s, 6H), 8.06 (d, *J* = 7.7 Hz, 6H), 8.02 (s, 6H), 7.92-7.88 (m, 18H), 7.77 (s, 6H), 7.69 (s, 6H), 7.57 (s, 6H), 7.55 (s, 6H), 7.42 (s, 6H), 7.40-7.20 (m, 54H), 7.15-7.09 (m, 24H), 7.06-6.99 (m, 30H), 6.93-6.85 (m, 24H), 6.76-6.69 (m, 24H), 6.53-6.44 (m, 18H), 2.96-2.72 (m, 262H), 2.00-1.30 (m, 270H), 1.23 (t, *J* = 6.3 Hz, 18H), 1.18 (t, *J* = 7 Hz, 18H), 1.15 (t, *J* = 7 Hz, 18H), 1.04-0.92 (m, 140H), 0.82-0.58 (m, 48H), 0.43 (t, *J* = 5.6 Hz, 18H); **FT-IR** (neat, ATR): $\tilde{\nu}$ (cm^{-1}) = 2956 (m), 2920 (m), 2869 (w), 1579 (m), 1481 (s), 1433 (m), 1359 (m), 1315 (w), 1143 (w), 1099 (m), 968 (m), 879 (m), 802 (s), 733 (m), 653 (w); **UV/Vis** (CH_2Cl_2): λ_{max} (nm) = 273, 368; **MALDI-TOF** (DCTB): [M+H+8(O)]$^+$ calcd. for $C_{1128}H_{1153}{}^{15}N_{72}S_{72}O_8$: 18226.01 found 18226.83.

Synthesis of S^{t}Bu-cube

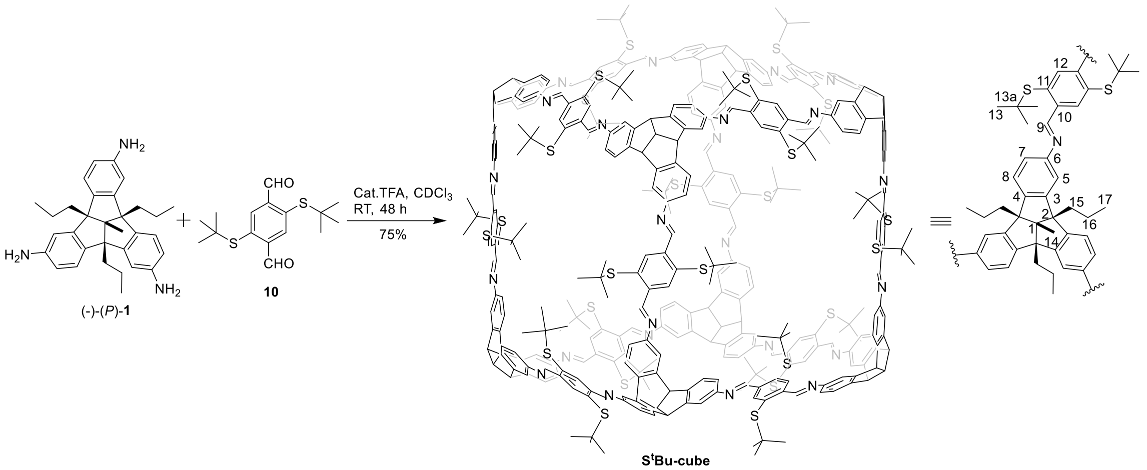

To a solution of TBTQ **1** (10 mg, 0.022 mmol) and 2,5-bis(tert-butylthio)terephthalaldehyde **10** (10 mg, 0.032 mmol) in chloroform (2 mL), a catalytic amount of TFA (0.2 µL, 0.003 mmol) was added and the reaction mixture was stirred for 18 hours at RT. The solvent was evaporated and the resulting solid was dissolved in DCM (0.3 mL). Methanol (4 mL) was added and the resulting yellow precipitate was separated by centrifugation technique. The precipitate was repeatedly dried and dissolved in a small amount of dichloromethane (3 x 0.3 mL) and reprecipitated by the addition of methanol to give 14 mg (75%) of **S^{t}Bu-cube** as a yellow solid.

Mp: 322°C (decomposed); **^{1}H NMR** (600 MHz, CD_2Cl_2): δ = 9.27 (s, 24H, *H*-9), 8.47 (s, 24H, *H*-12), 7.44 (d, ^3J = 8.4 Hz, 24H, *H*-8), 7.25 (d, ^3J = 1.2 Hz, 24H, *H*-5), 7.09 (dd, ^3J = 7.8 Hz, ^4J = 1.2 Hz, 24 H, *H*-7), 2.25-2.24 (m, 48H, *H*-15), 1.71 (s, 24H, *H*-14), 1.31-1.24 (m, 264H, *H*-13 & *H*-16), 0.98 (t, ^3J = 7.2 Hz, 72H, *H*-17); **^{13}C NMR** (151 MHz, CD_2Cl_2): δ =158.9 (*C*-9), 151.8 (*C*-11), 149.6 (*C*-4), 146.8 (*C*-6), 142.2 (*C*-3), 138.5 (*C*-8), 136.2 (*C*-10), 124.5 (*C*-12), 119.8 (*C*-7), 117.9 (*C*-5), 73.4 (*C*-1), 67.3 (*C*-2), 48.3 (*C*-13a) , 41.1 (*C*-15), 31.3 (*C*-13), 20.9 (*C*-18), 15.5 (*C*-14), 15.3 (*C*-17); **FT-IR** (neat, ATR): $\tilde{\nu}$ (cm^{-1}) = 3057 (w), 3024 (w), 2959 (m), 2926 (m), 2870 (w), 1628 (w) 1591 (s), 1578 (m), 1553 (m), 1526 (w), 1485 (m), 1462 (m), 1441 (m), 1391 (m), 1364 (m), 1348 (w), 1308 (s), 1261 (m), 1238 (m), 1217 (m), 1163 (s), 1150 (s), 1132 (s), 1101 (s),1063 (s), 1030 (m), 1001 (m), 986 (m), 978 (m), 922 (m), 903 (s), 876 (s), 833 (s), 825 (s), 798 (s), 787 (m), 760 (s), 735 (s), 698 (s), 687 (s), 665 (s), 631 (s), 608 (m); **UV/Vis** (CH_2Cl_2): λ_{max} (nm) = 265, 295, 363; **MALDI-TOF** (DCTB): *m/z* $[M-C_4H_9]^+$ calcd. for $C_{444}H_{519}N_{24}S_{24}$: 6960.48, found 6960.22.

Synthesis of Br-cube

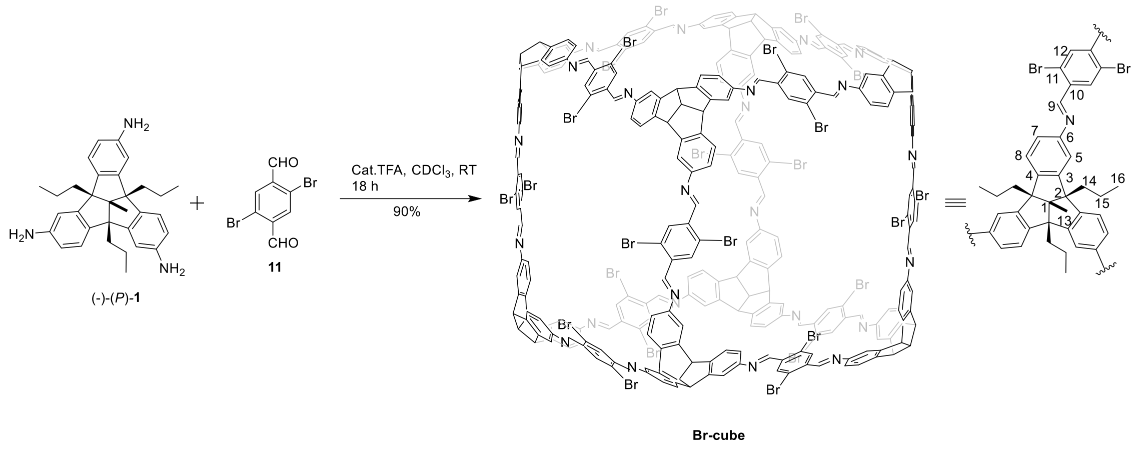

To a solution of TBTQ **1** (5 mg, 0.01 mmol) and 2,5-bromo-terephthaldehyde **11** (4.7 mg, 0.016 mmol) in chloroform (1 mL), a catalytic amount of TFA (0.1 µL, 0.001 mmol) was added and the reaction mixture was stirred at RT for 18 hours. The reaction mixture was concentrated to ~0.2 mL and acetonitrile (4 mL) was added. The resulting precipitate was separated by the centrifugation technique. The precipitate was repeatedly dried and dissolved in a small amount of dichloromethane (3 x 0.2 mL) and reprecipitated by the addition of acetonitrile afforded 8.4 mg (92%) of **Br-cube** as a yellow solid.

Mp: 332°C (decomposed); **^{1}H NMR** (600 MHz, CD_2Cl_2): δ = 8.76 (s, 24H, *H*-9), 8.45 (s, 24H, *H*-12), 7.45 (d, ^{3}J = 8.4 Hz, 48H, *H*-8), 7.28 (d, ^{3}J = 0.6 Hz, 24H, *H*-5), 7.11 (dd, ^{3}J = 8.4 Hz, ^{4}J = 1.2 Hz, 24 H, *H*-7), 2.24 (br (t), 48H, *H*-14) 1.70 (s, 24H, *H*-13), 1.31-1.25 (m, 48H, *H*-15), 0.98 (t, ^{3}J = 7.2 Hz, 72H, *H*-16); **^{13}C NMR** (151 MHz, CD_2Cl_2): δ = 156.6 (*C*-9), 150.8 (*C*-6), 149.6 (*C*-4), 147.5 (*C*-3), 138.0 (*C*-11), 133.3 (*C*-12), 125.0 (*C*-10), 124.7 (*C*-8), 119.1 (*C*-7), 118.9 (*C*-5), 73.4 (*C*-1), 67.4 (*C*-2), 41.1 (*C*-14), 20.9 (*C*-15), 15.5 (*C*-13), 15.2 (*C*-16); **FT-IR** (neat, ATR): $\tilde{\nu}$ (cm^{-1}) = 3077 (w), 3020 (w), 2956 (s), 2928 (m), 2869 (m), 1734 (w), 1696 (w), 1616 (m), 1593 (m), 1482 (s), 1456 (m), 1359 (s), 1260 (m), 1145 (w), 903 (w), 806 (w), 764 (s), 750 (s), 699 (w); **UV/Vis** (CH_2Cl_2): λ_{max} (nm) = 269, 300, 378; **MALDI-TOF** (DCTB): *m/z* [M]$^{+}$ calcd. for $C_{352}H_{312}N_{24}Br_{24}$: 6795.54, found 6795.56.

Synthesis H/OMe-cube

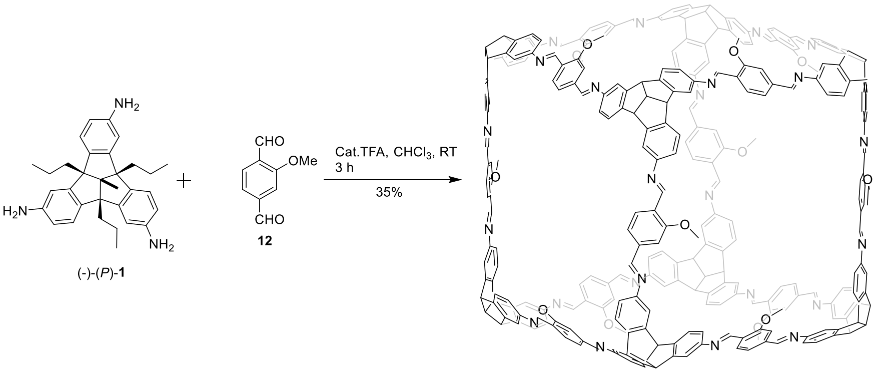

Mono OMe-cube

To a solution of TBTQ **1** (5.4 mg, 0.012 mmol) and 2-methoxy-terephthaldehyde **12** (2.6 mg, 0.016 mmol) in deuterated chloroform (1 mL) in a screw-capped 8 mL glass vial, a catalytic amount of TFA (0.1 µL, 0.001 mmol) was added and the reaction mixture was stirred at RT for 3 hours. Solid $NaHCO_3$ (200 mg) was added and the reaction mixture was further stirred at RT for 10 minutes. The organic layer was separated by decantation and concentrated to a total volume of 1 mL and then purified by recycling gel permeation chromatography (rGPC) (THF, 40°C, 5 mL/min) to give 2.4 mg (35%) of **Mono-OMe-cube** as a yellow solid.

Mp: >350°C; **^{1}H NMR** (600 MHz, $C_2D_2Cl_4$): δ = 8.85-8.84 (m, 12H), 8.40-8.39 (m, 12H), 8.16-8.15 (m, 12H), 7.52 (br (s), 12H), 7.46-7.44 (m, 12H), 7.40-7.37 (m, 24H), 7.20-7.18 (m, 24H), 7.05-7.04 (m, 24H), 3.94-3.92 (m, 36H), 2.22 (br (s), 4H), 1.67 (br (s). 24H), 1.30-1.26 (m, 48H), 0.99-0.96 (m, 72H); **^{13}C NMR** (151 MHz, $C_2D_2Cl_4$): δ =159.4, 158.71, 158.69, 155.12, 155.11, 155.08, 151.60, 151.59, 150.81, 150.78, 149.32, 149.30, 149.27, 149.23, 149.20, 149.16, 149.14, 146.04, 146.02, 145.95, 145.81, 145.78, 139.87, 139.84, 139.81, 139.78, 127.5, 127.2, 124.1, 124.0, 121.96, 121.92, 119.4, 119.33, 119.28, 116.9, 116.4, 109.86, 109.83, 72.8, 66.72, 66.69, 66.67, 55.8, 40.6, 40.5, 20.38, 15.17; **FT-IR** (neat, ATR): $\tilde{\nu}$ (cm^{-1}) = 2858 (m), 2931 (w), 2914 (w), 2871 (w), 1618 (m), 1595 (m), 1564 (w), 1481 (s), 1461 (m), 1415 (w), 1375 (w), 1303 (w), 1286 (w), 1259 (m), 1193 (w), 1153 (w), 1112 (w), 1076 (w), 1035 (m), 970 (w), 941 (w), 875 (m), 823 (m), 775 (w), 767 (w), 732 (w), 698 (w), 673 (w), 648 (w), 617 (w); **UV/Vis** (CH_2Cl_2): λ_{max} (nm) = 295, 375; **MALDI-TOF** (DCTB): m/z [M]$^+$ calcd. for $C_{364}H_{360}N_{24}O_{12}$: 5262.84, found 5262.84.

Synthesis (H/OMe-cube)₂

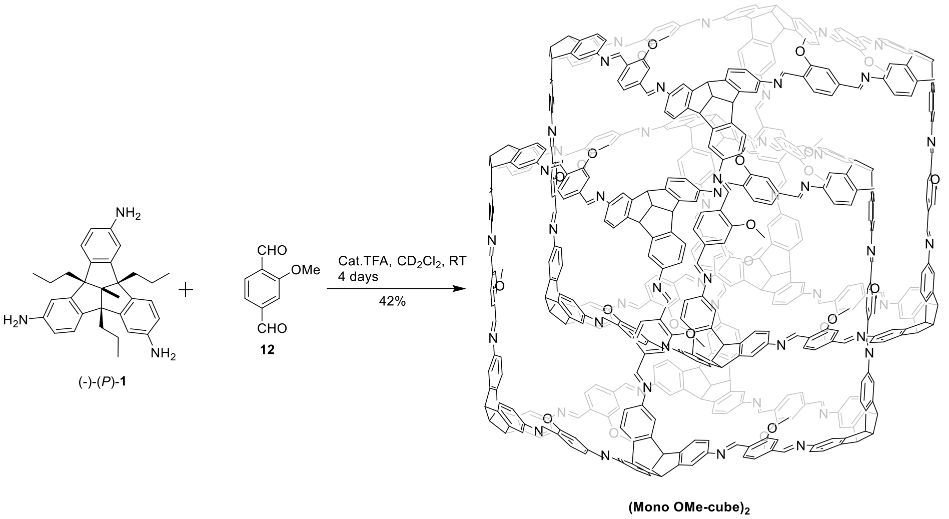

To a solution of TBTQ **1** (5.3 mg, 0.011 mmol) and 2-methoxy-terephthaldehyde **12** (2.6 mg, 0.016 mmol) in deuterated dichloromethane (1 mL) in a screw-capped 8 mL glass vial, a catalytic amount of TFA (0.1 µL, 0.001 mmol) was added and the reaction mixture was stirred at RT for 4 days. Afterward, the crude reaction mixture was stirred with aq. K_2CO_3 solution (0.25 M, 3 × 2 mL) for 10 minutes, the organic layer was separated, dried over Na_2SO_4 and concentrated under reduced pressure. The resulting yellow solid was dissolved immediately in dichloromethane and purified by recycling gel permeation chromatography (rGPC) (THF, 40°C, 3 mL/min) to give 2.9 mg (42%) of **(Mono-OMe-cube)₂** as a yellow solid.

Mp: decomposed above 290°C; **^{1}H NMR** (600 MHz, CD_2Cl_2): δ = 8.96-8.69 (m, 18H), 8.53-8.15 (m, 44H), 8.01-6.17 (m, 202H), 4.03-3.92 (m, 46H), 3.69-3.42 (m, 12 H), 2.94-2.48 (m, 14H), 2.24-2.00 (m, 88H), 1.72-1.65 (m, 52H), 1.30-0.38 (m, 244H); **FT-IR** (neat, ATR): $\tilde{\nu}$ (cm^{-1}) = 2960 (m), 2932 (w), 2913 (w), 2873 (w), 1618 (m), 1595 (m), 1564 (w), 1481 (m), 1461 (w), 1415 (m), 1371 (w), 1307 (w), 1263 (w), 1193 (w), 1151 (w), 1112 (w), 1035 (w), 952 (s), 865 (w), 825 (w), 711 (s), 676 (m); **UV/Vis** (CH_2Cl_2): λ_{max} (nm) = 294, 370; **TIMS-TOF Flex** (DCTB): m/z $[M+H]^+$ calcd. for $C_{352}H_{337}N_{24}O_{24}$: 10526.69, found 10526.70.

Note: A decent ^{13}C NMR spectrum was not available, even by recording 6144 scans on a 600 MHz NMR instrument (see Figure 296).

2. 1D and 2D NMR spectra

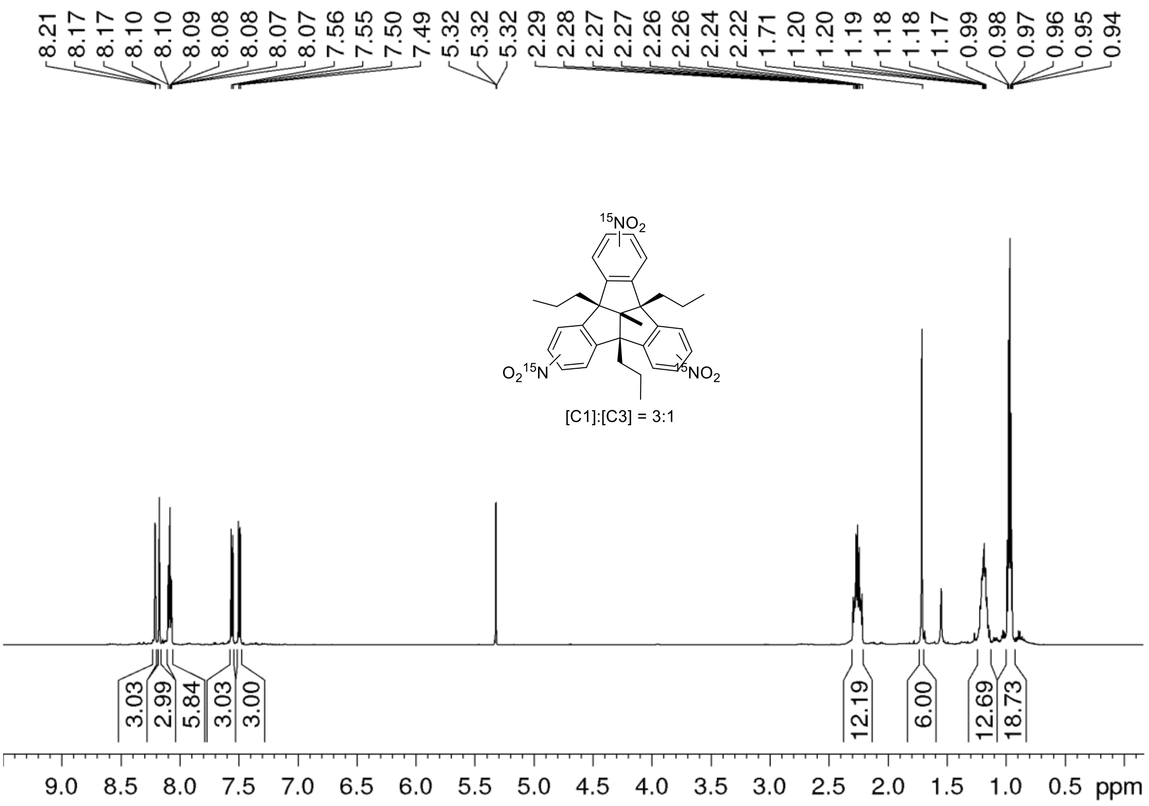

Figure 1. ^{1}H NMR (600 MHz, CD_2Cl_2) spectrum of **^{15}N labelled Trinitro-TBTQ (C1:C3, 3:1) 13**.

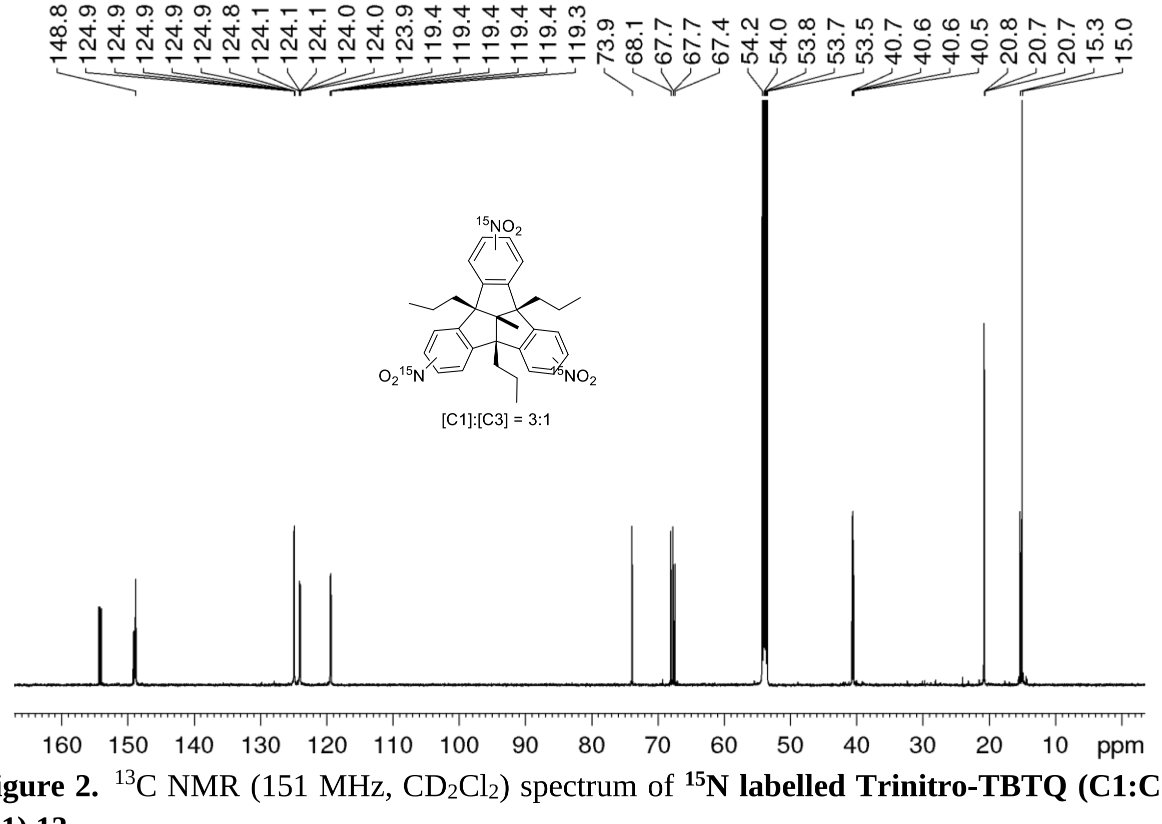

Figure 2. ^{13}C NMR (151 MHz, CD_2Cl_2) spectrum of **^{15}N labelled Trinitro-TBTQ (C1:C3, 3:1) 13**.

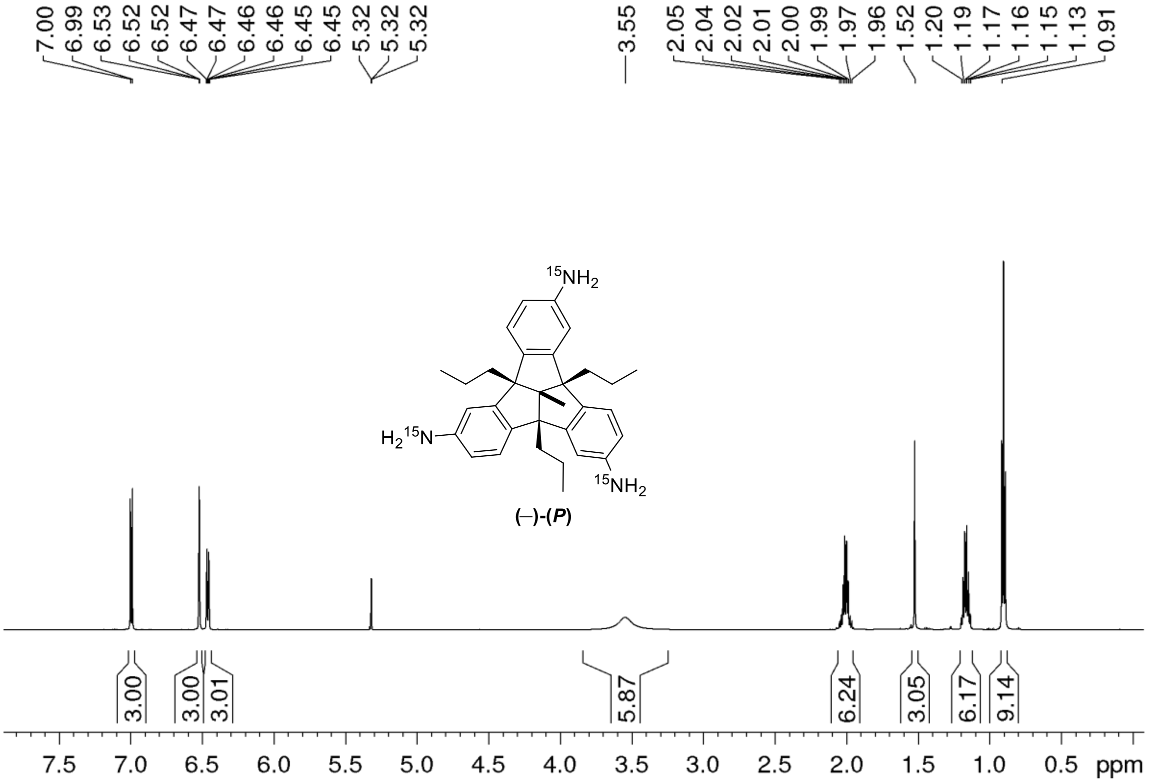

Figure 3. ^{1}H NMR (600 MHz, CD_2Cl_2) spectrum of enantiopure ^{15}**N labelled (-)-(*P)*-TA-TBTQ 14**.

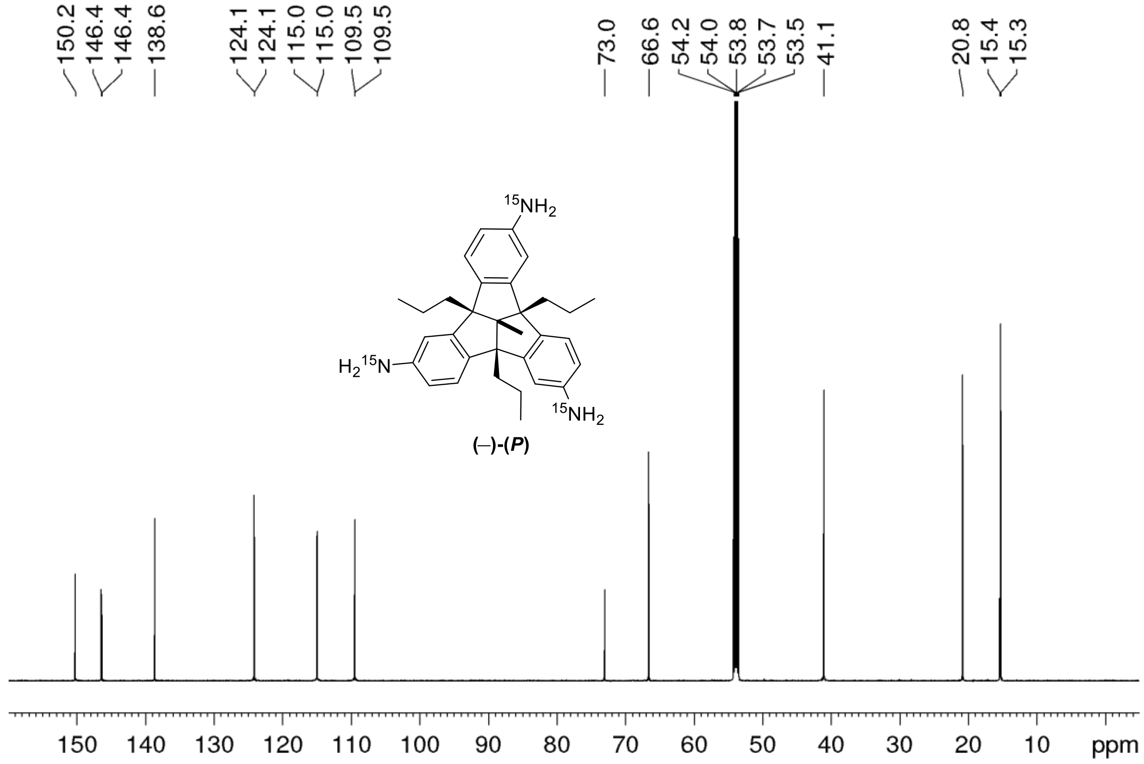

Figure 4. ^{13}C NMR (151 MHz, CD_2Cl_2) spectrum of enantiopure ^{15}**N labelled (-)-(*P)*-TA-TBTQ 14**.

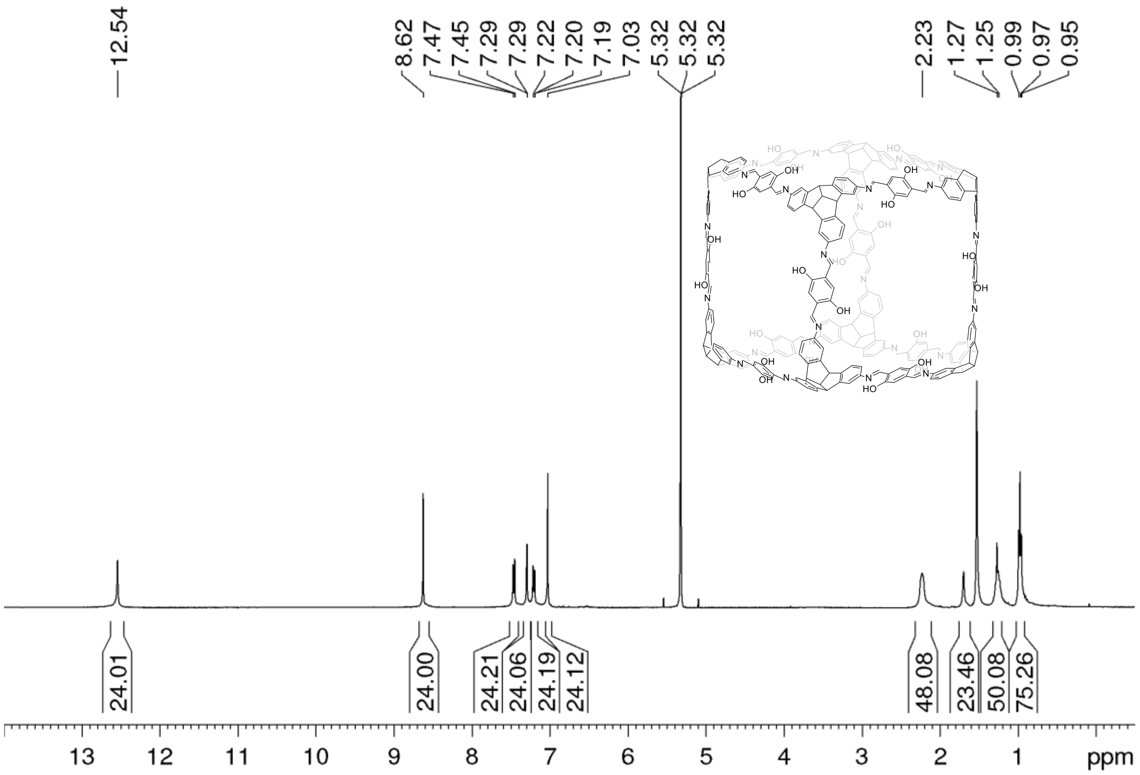

Figure 5. ^{1}H NMR (400 MHz, CD_2Cl_2) spectrum of **OH-cube**.

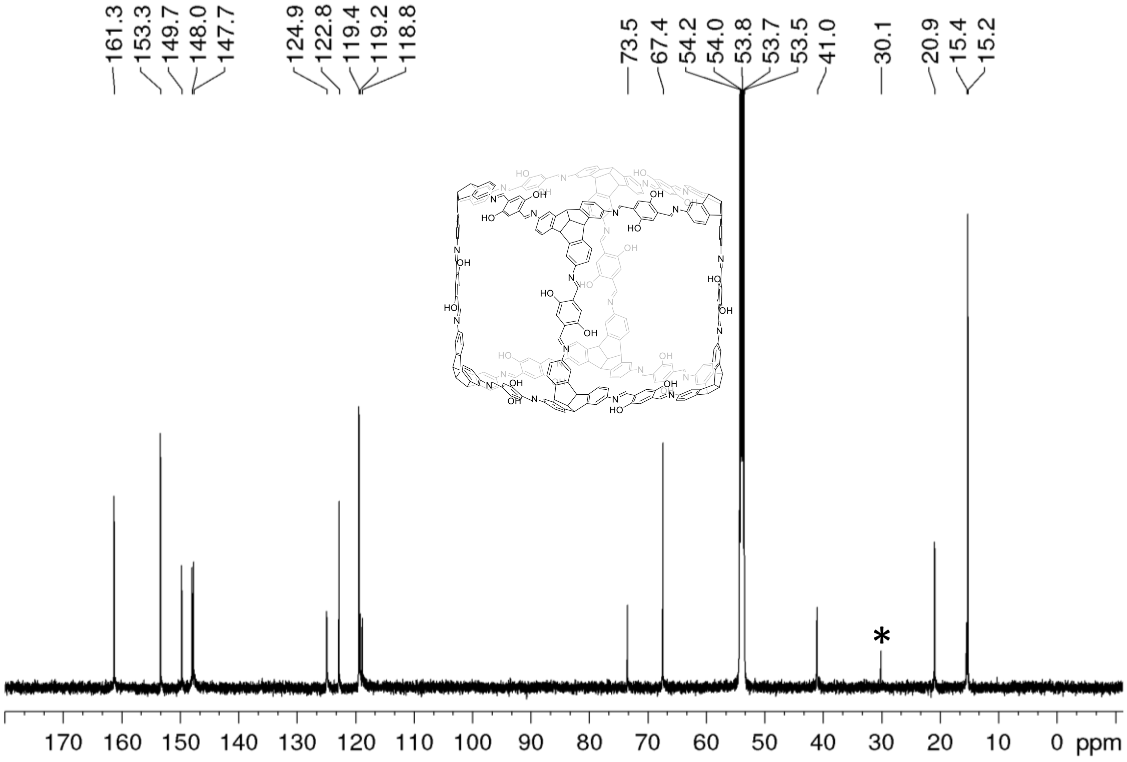

Figure 6. ^{13}C NMR (151 MHz, CD_2Cl_2) spectrum of **OH-cube**. * H-grease

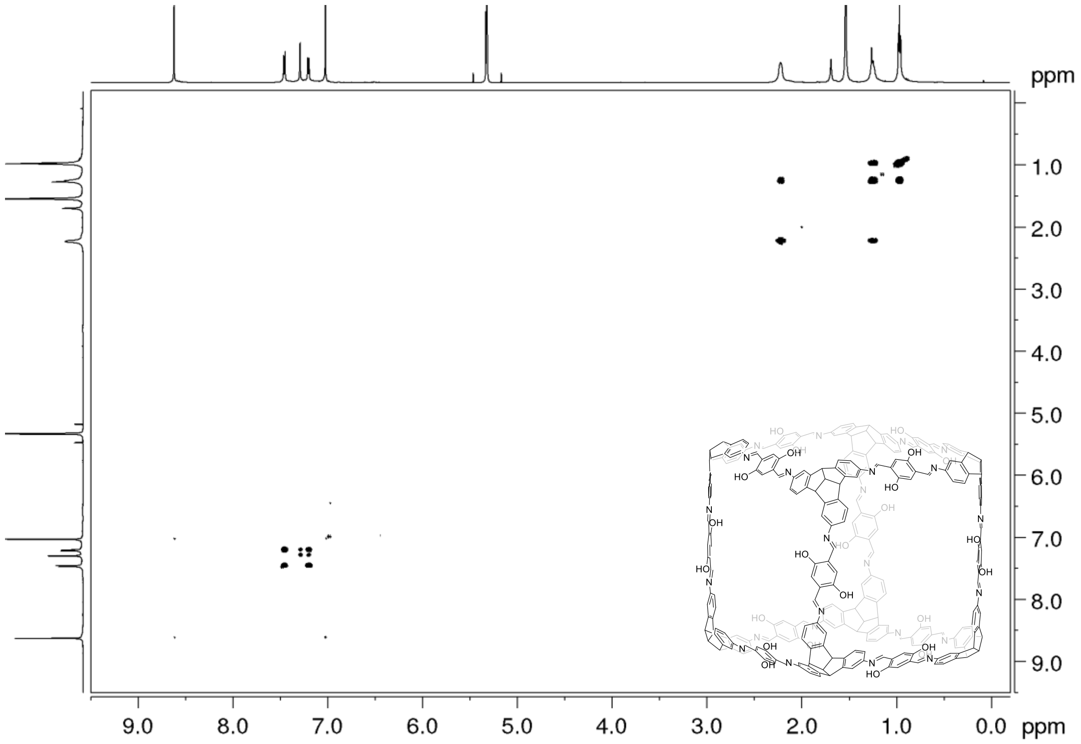

Figure 7. ^{1}H-^{1}H COSY NMR spectrum (600 MHz, CD_2Cl_2) of **OH-cube**.

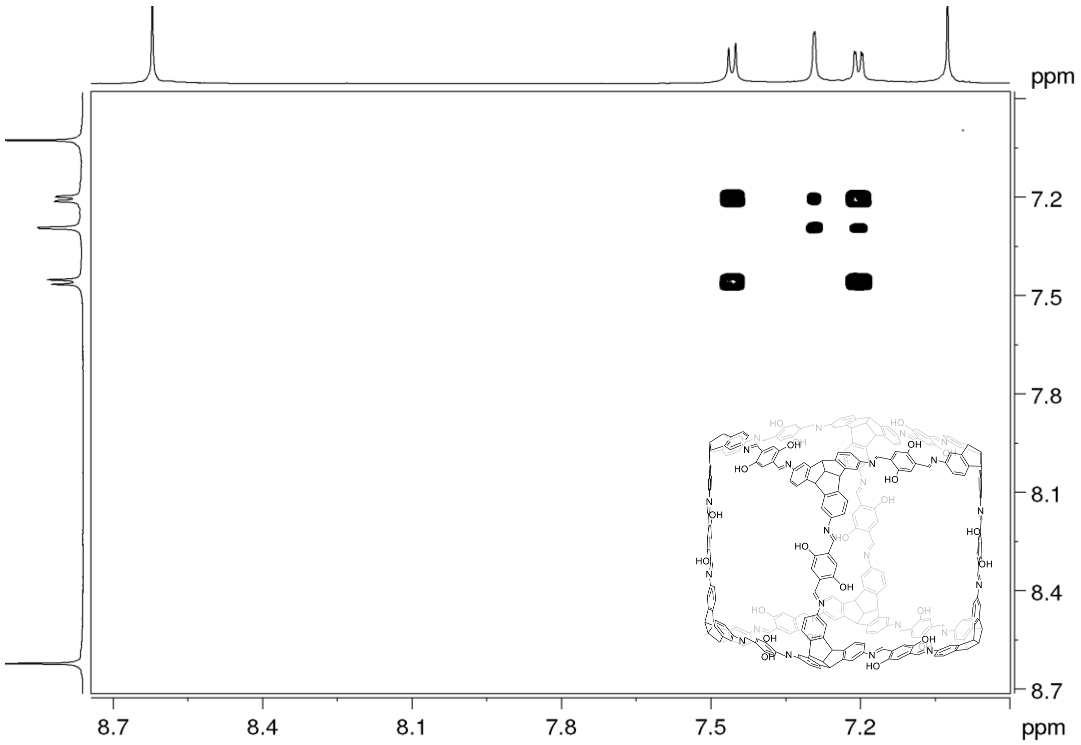

Figure 8. Partial ^{1}H-^{1}H COSY NMR spectrum (600 MHz, CD_2Cl_2) of **OH-cube**.

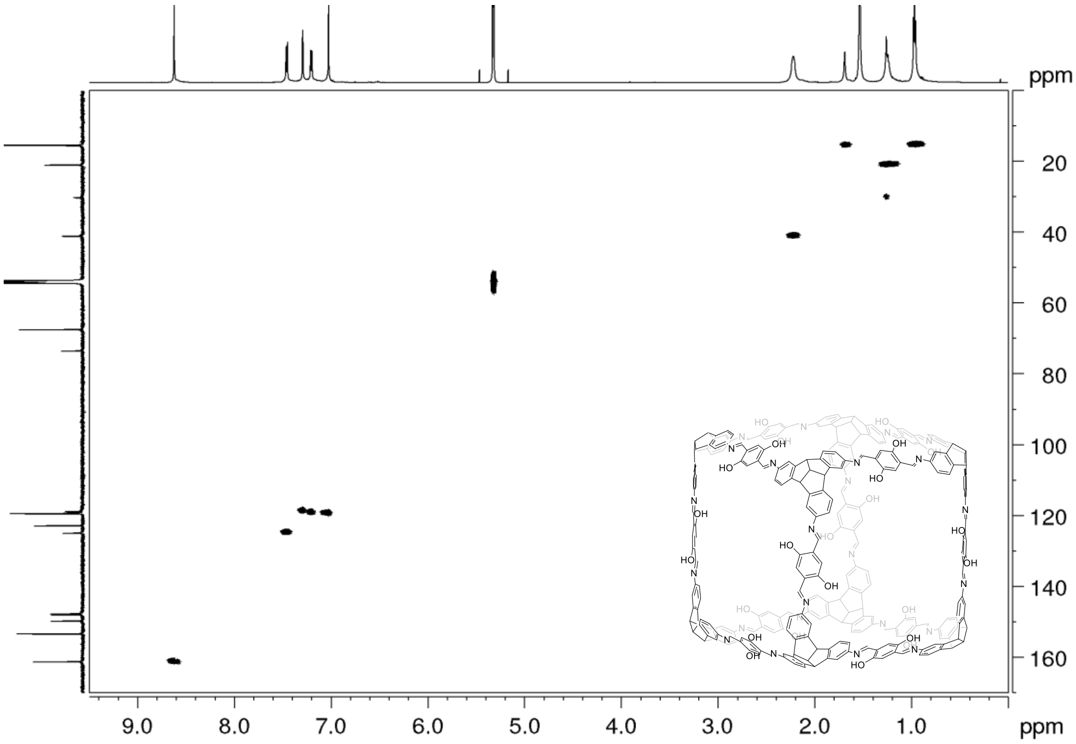

Figure 9. ^{1}H-^{13}C HSQC NMR (600 MHz and 151 MHz, CD_2Cl_2) spectrum of **OH-cube**.

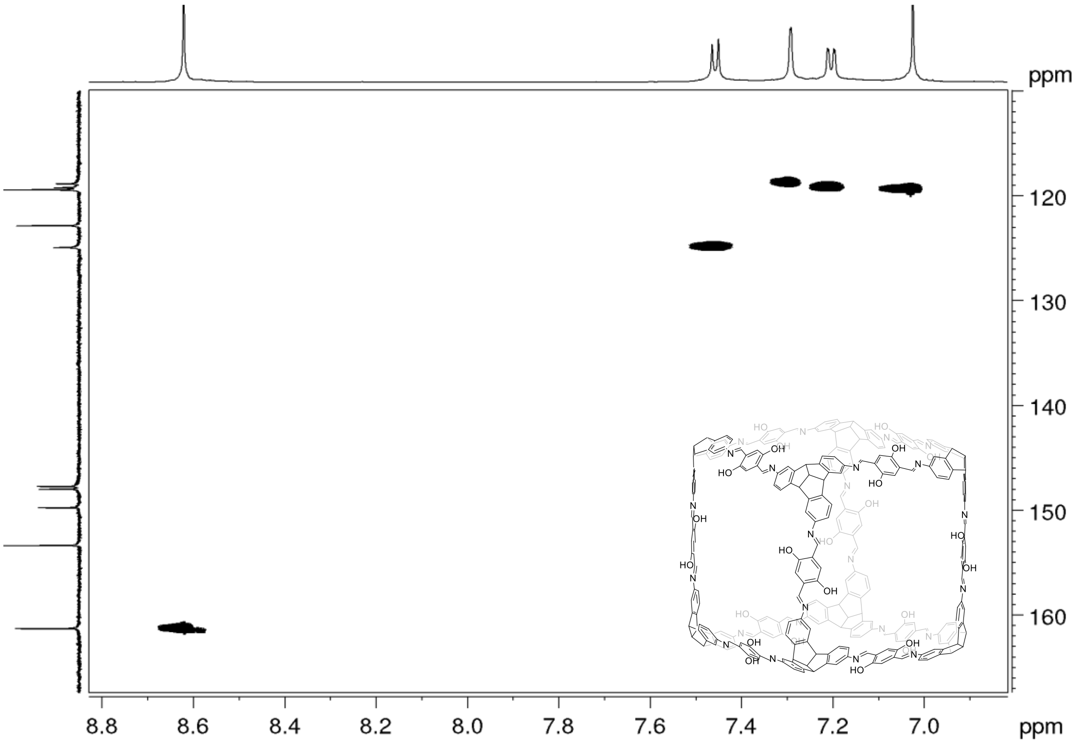

Figure 10. Partial ^{1}H-^{13}C HSQC NMR (600 MHz and 151 MHz, CD_2Cl_2) spectrum of **OH-cube**.

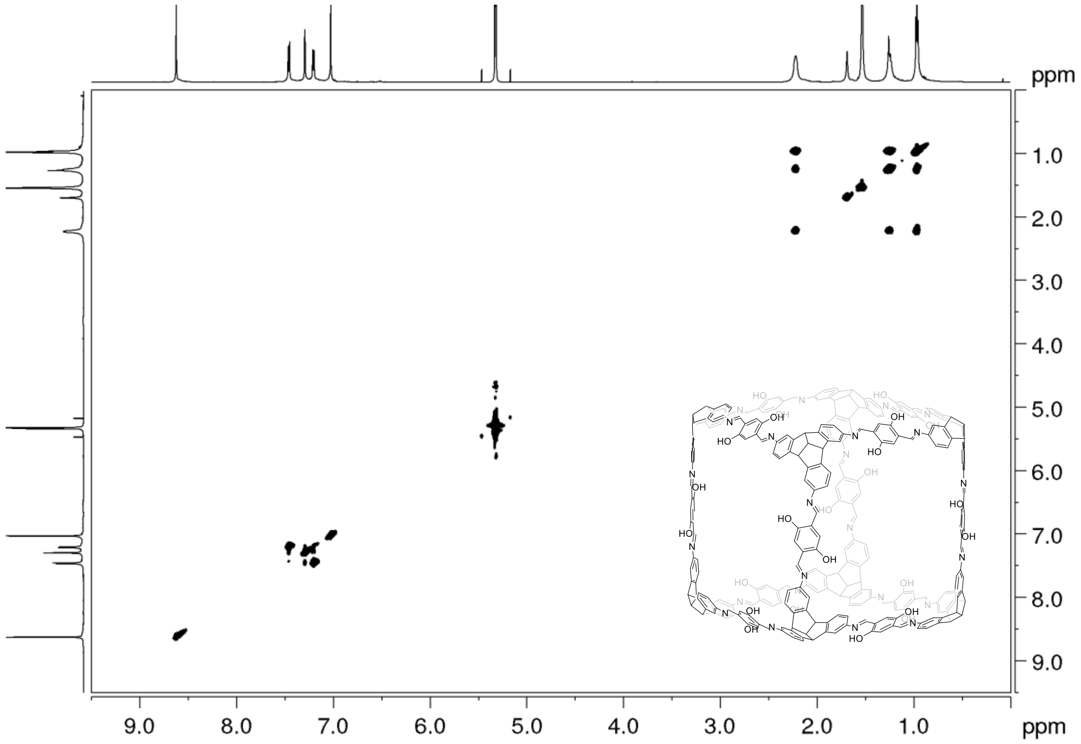

Figure 11. ^1H-^1H TOCSY NMR (600 MHz, CD_2Cl_2) spectrum of **OH-cube**.

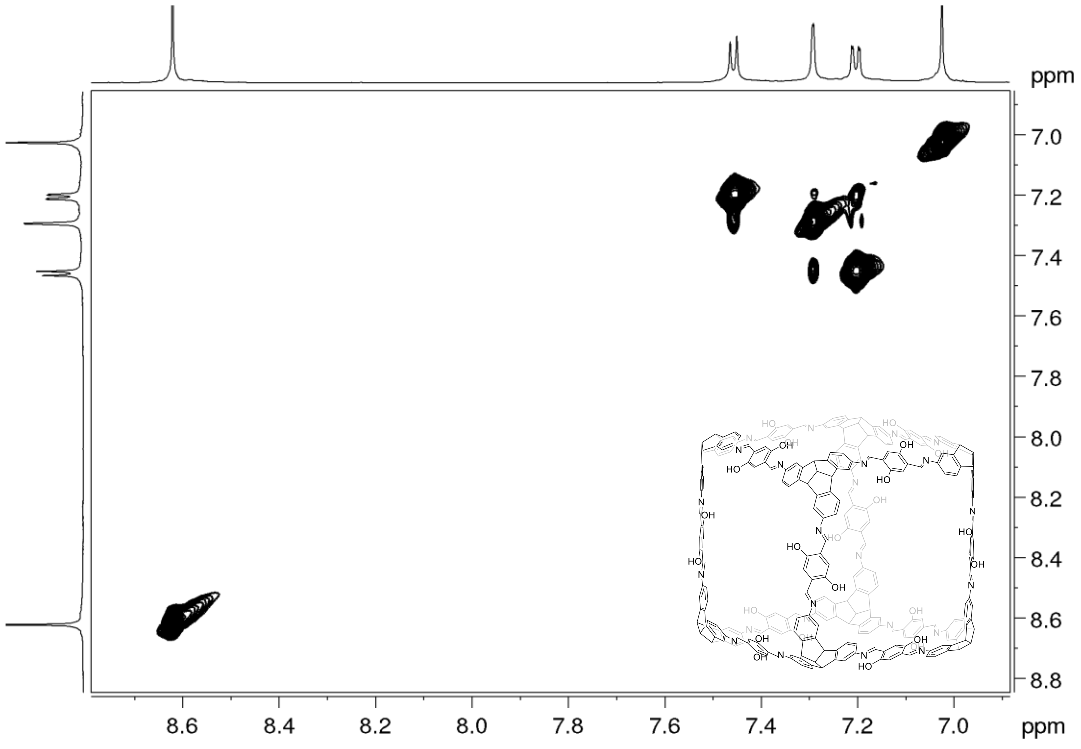

Figure 12. Partial ^1H-^1H TOCSY NMR (600 MHz, CD_2Cl_2) spectrum of **OH-cube**.

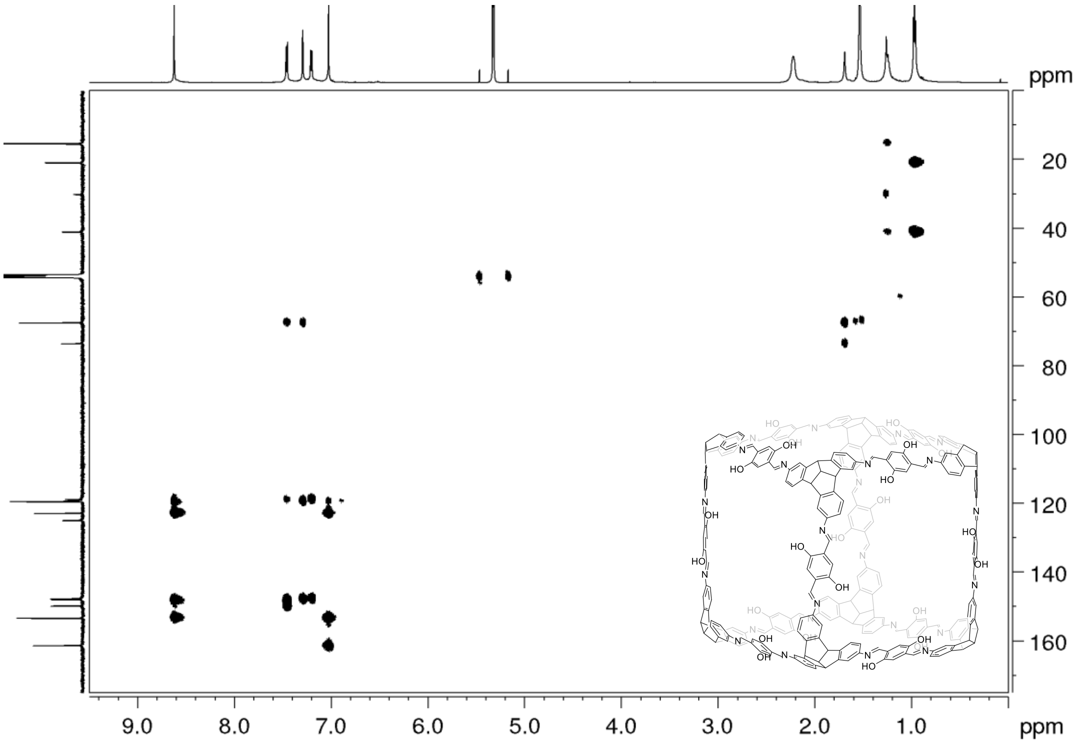

Figure 13. ^{1}H-^{13}C HMBC NMR (600 MHz and 151 MHz, CD_2Cl_2) spectrum of **OH-cube**.

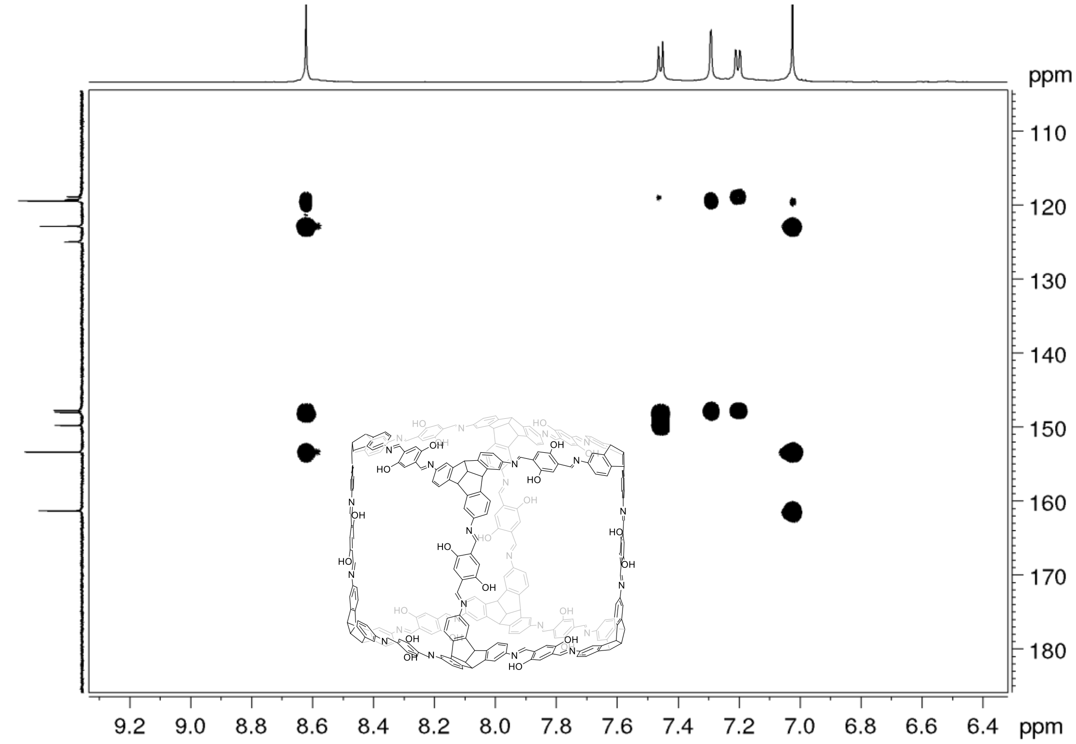

Figure 14. Partial ^{1}H-^{13}C HMBC NMR (600 MHz and 151 MHz, CD_2Cl_2) spectrum of **OH-cube**.

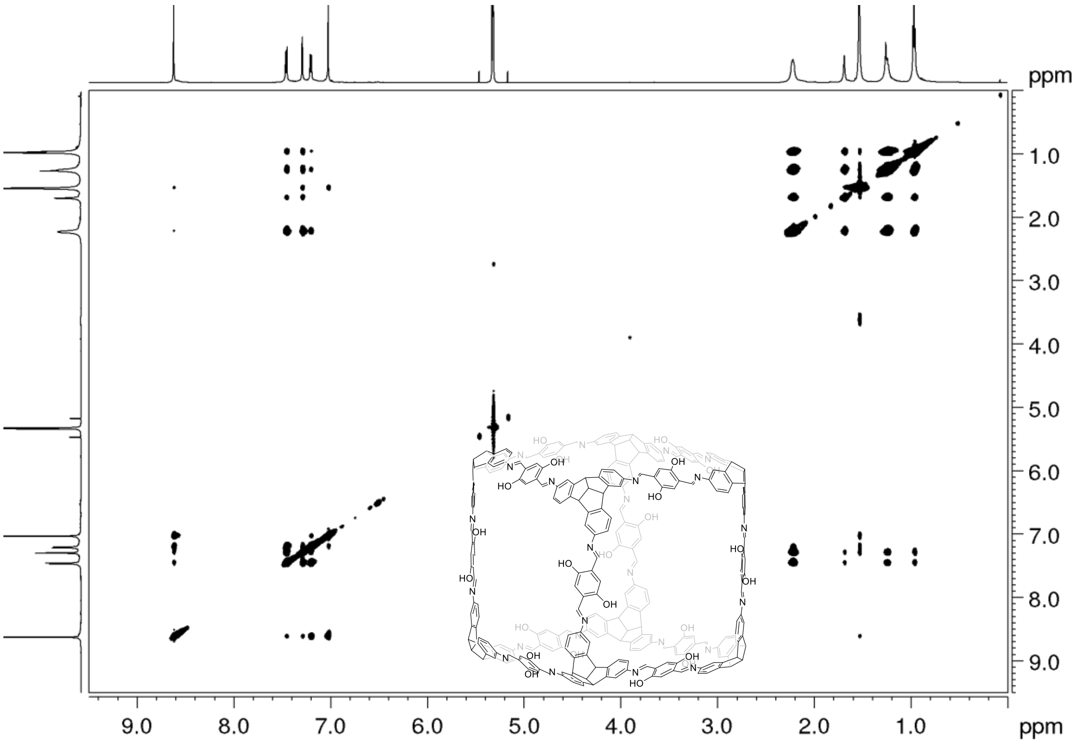

Figure 15. ^{1}H-^{1}H NOESY NMR (600 MHz, CD_2Cl_2) spectrum of **OH-cube**.

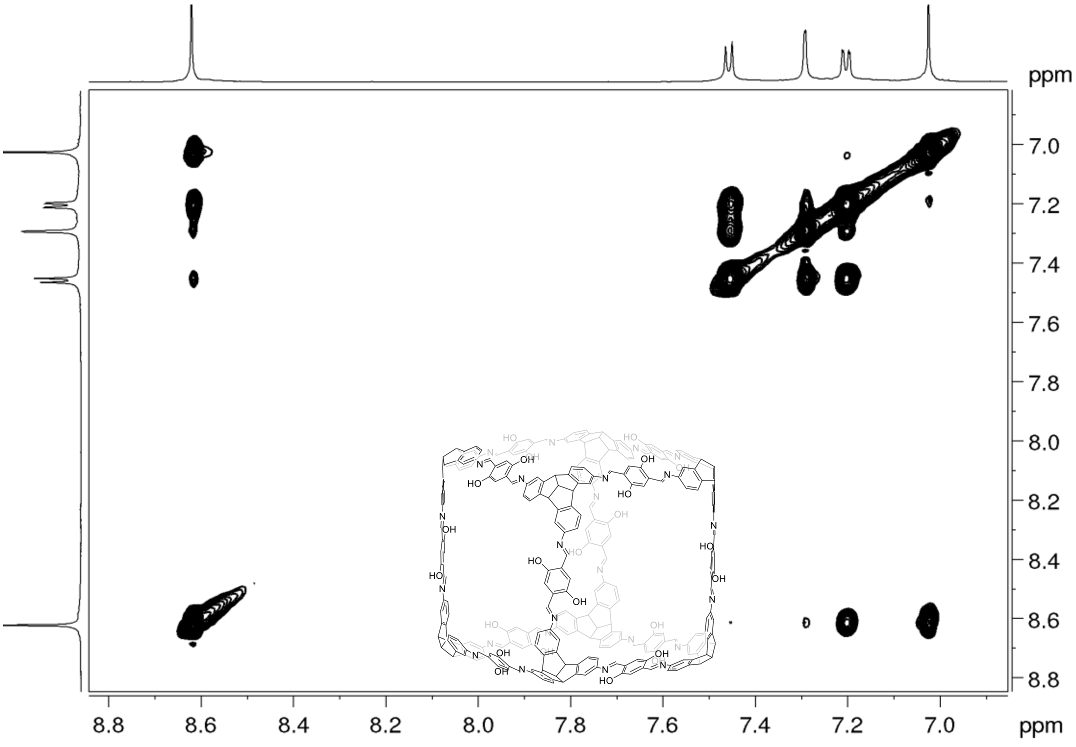

Figure 16. Partial ^{1}H-^{1}H NOESY NMR (600 MHz, CD_2Cl_2) spectrum of **OH-cube**.

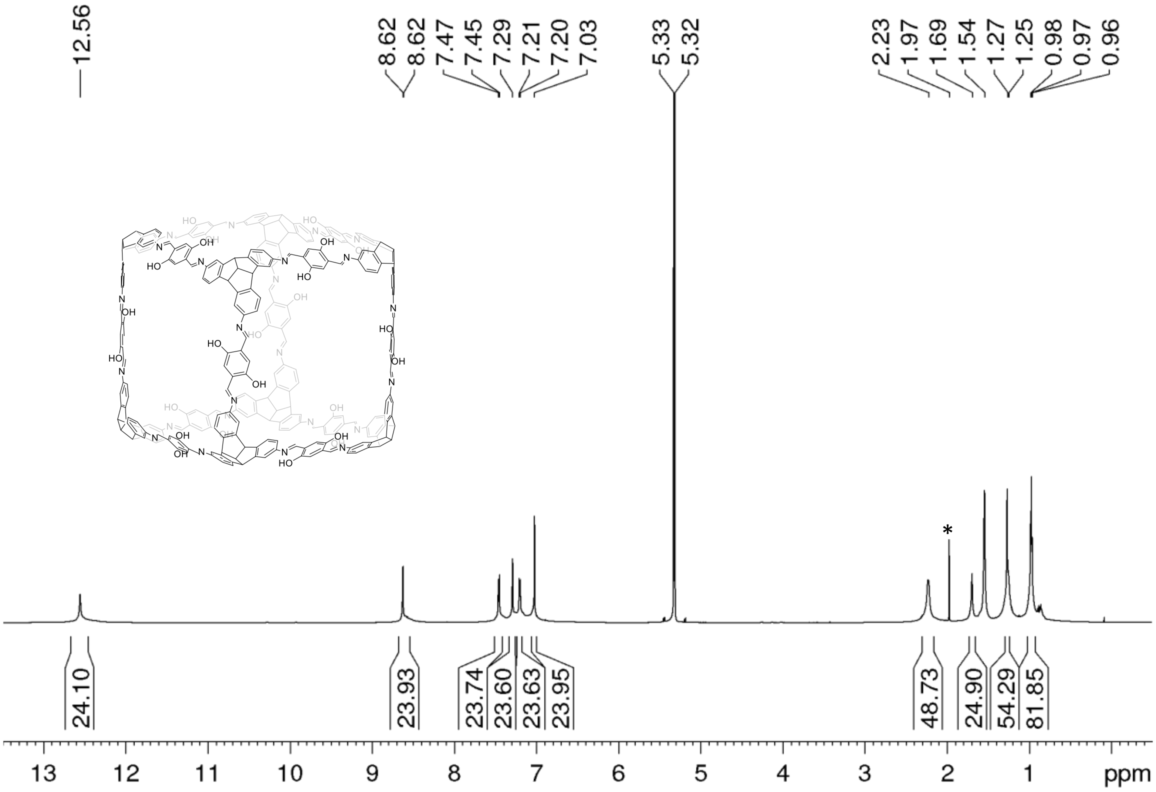

Figure 17. ^{1}H NMR (700 MHz, CD_2Cl_2) spectrum of ^{15}N labelled ***OH-cube**. *ACN.

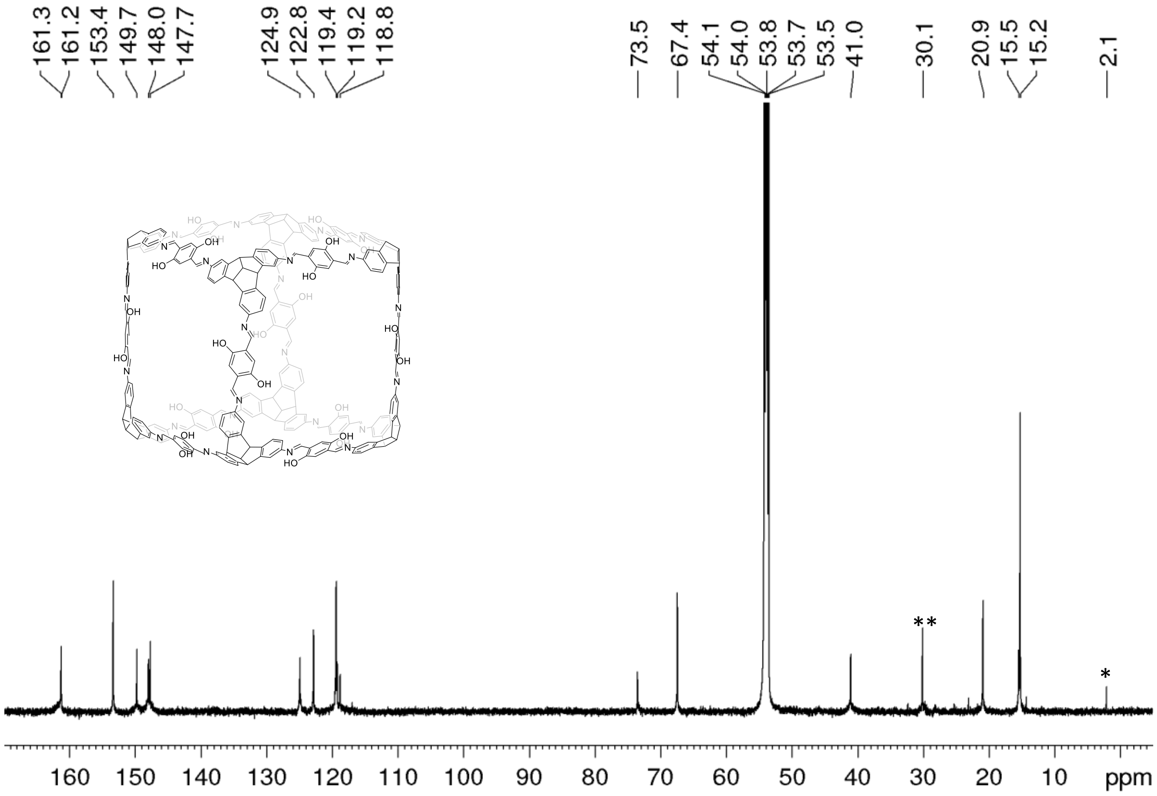

Figure 18. ^{13}C NMR (176 MHz, CD_2Cl_2) spectrum of ^{15}N labelled ***OH-cube**. *ACN, **H-grease.

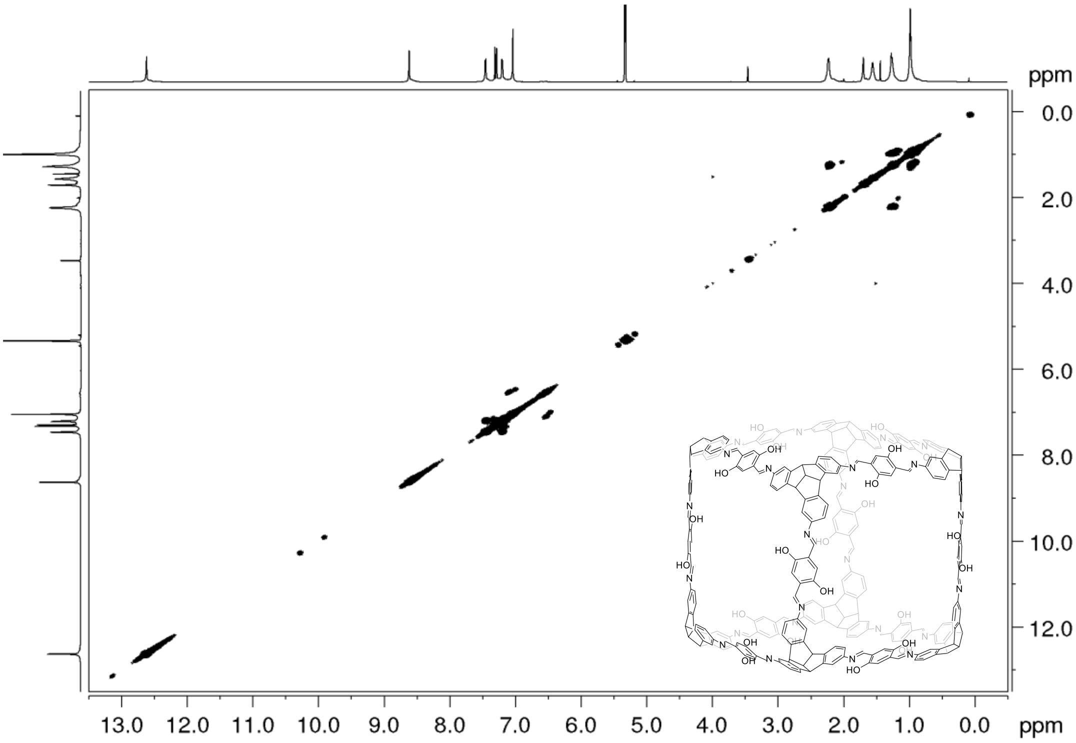

Figure 19. ^{1}H-^{1}H COSY NMR spectrum (700 MHz, CD_2Cl_2) of ^{15}N labelled ***OH-cube**.

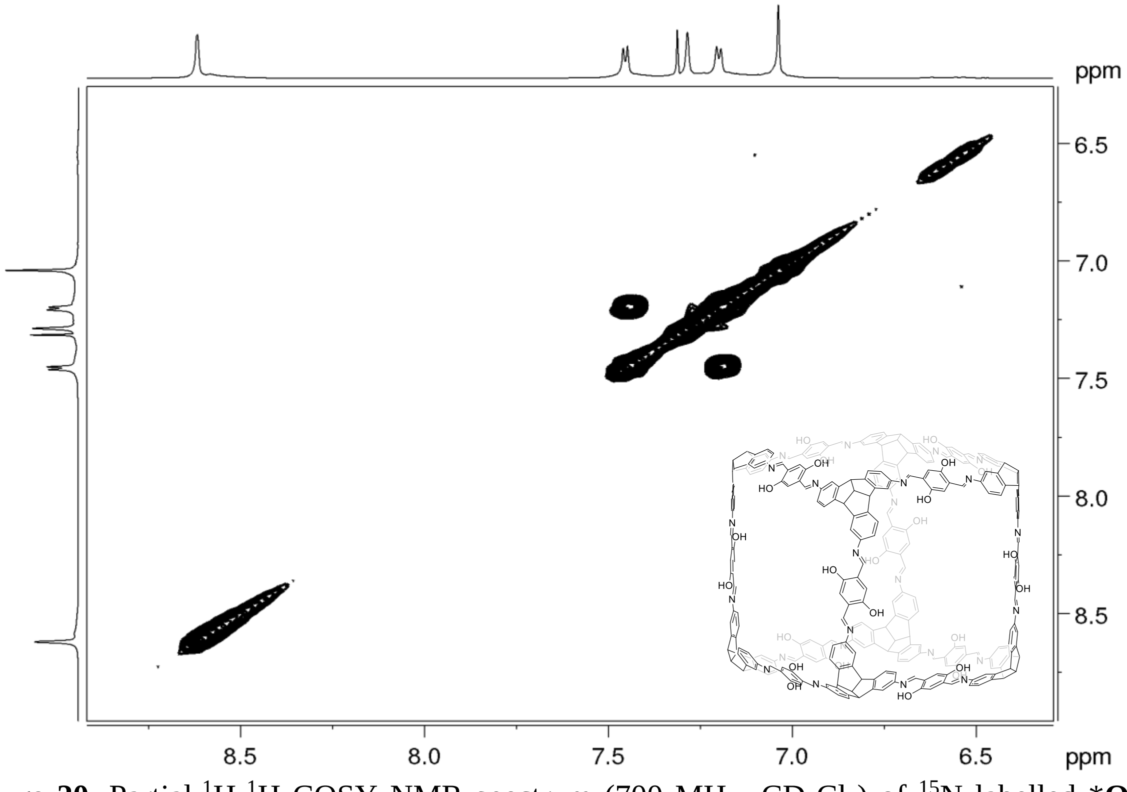

Figure 20. Partial ^{1}H-^{1}H COSY NMR spectrum (700 MHz, CD_2Cl_2) of ^{15}N labelled ***OH-cube**.

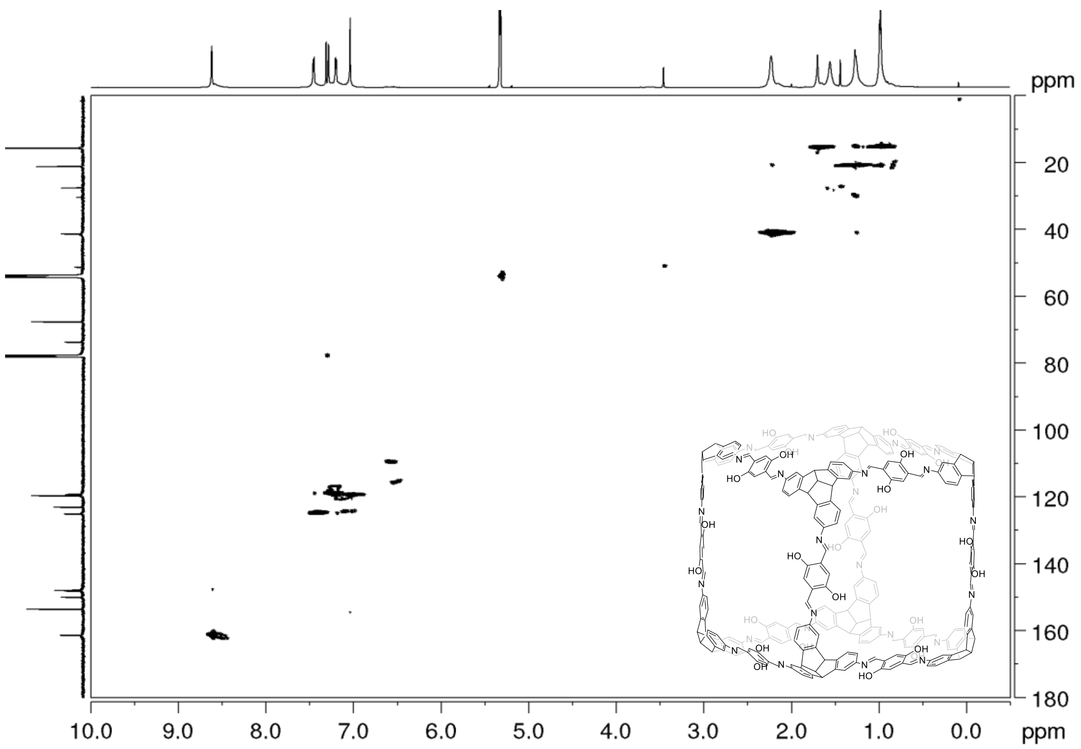

Figure 21. ^{1}H-^{13}C HSQC NMR (700 MHz and 151 MHz, CD_2Cl_2) spectrum of ^{15}N labelled ***OH-cube**.

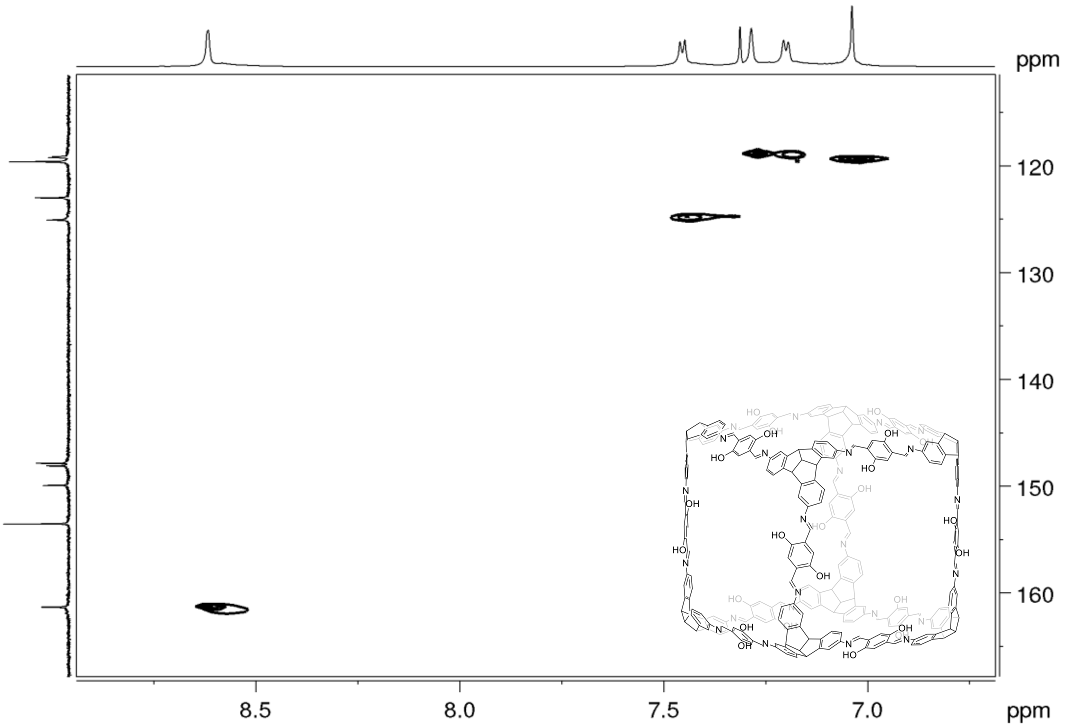

Figure 22. Partial ^{1}H-^{13}C HSQC NMR (700 MHz and 151 MHz, CD_2Cl_2) spectrum of ^{15}N labelled ***OH-cube**.

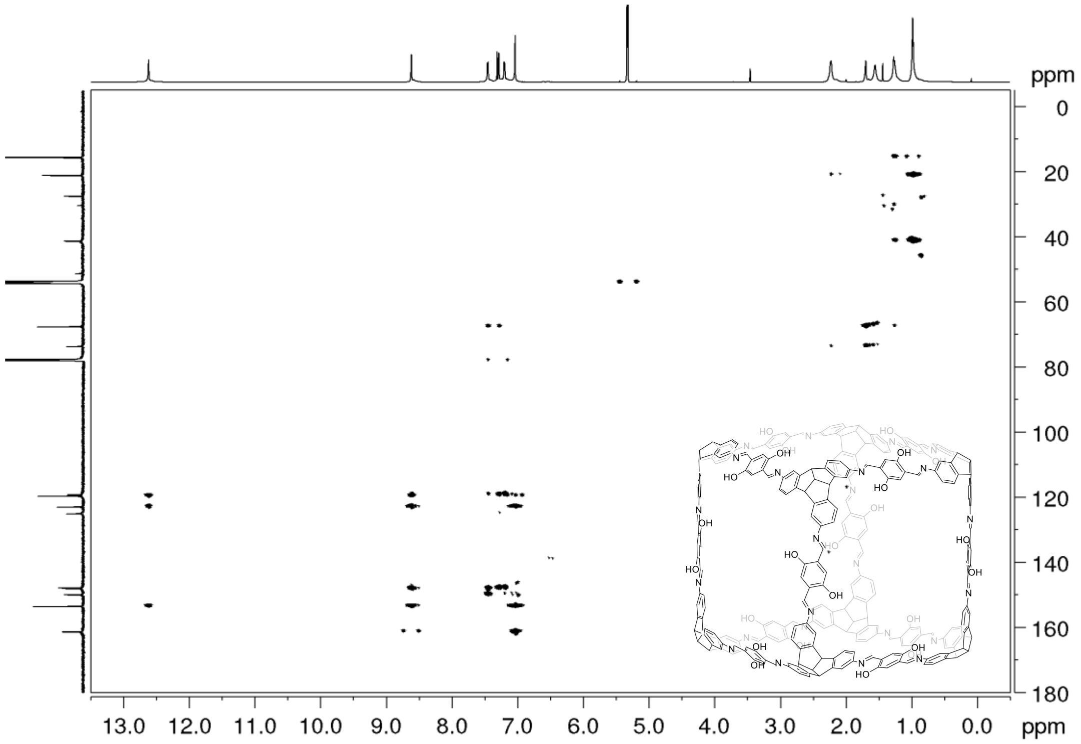

Figure 23. ^1H-^{13}C HMBC NMR (700 MHz and 176 MHz, CD_2Cl_2) spectrum of ^{15}N labelled ***OH-cube**.

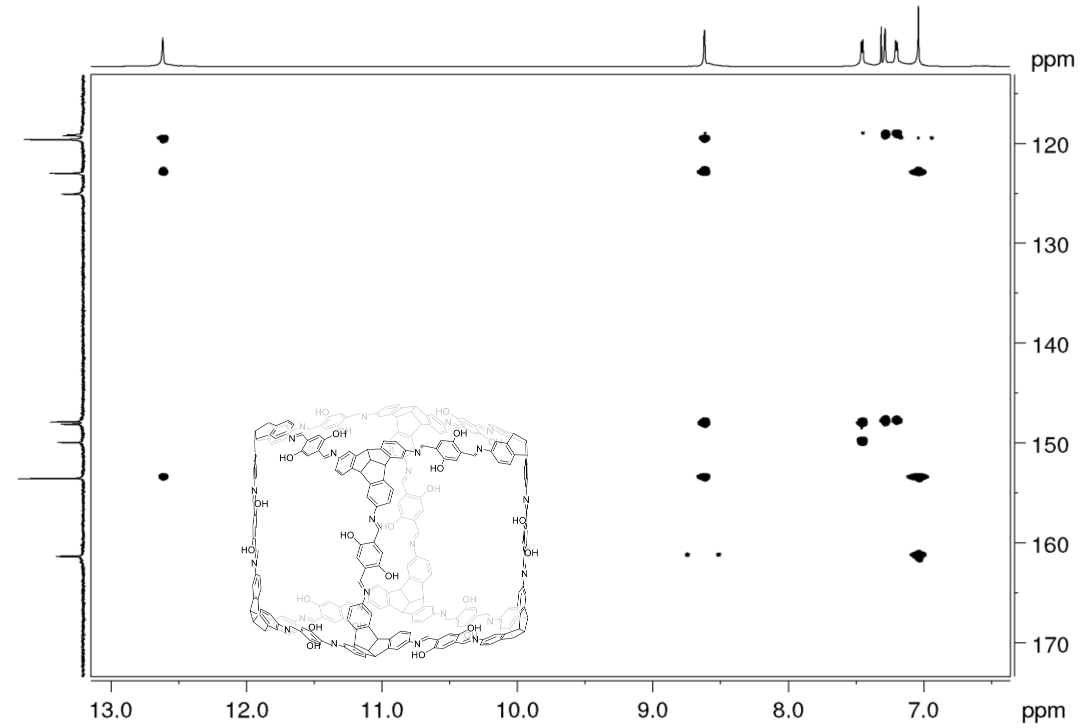

Figure 24. Partial ^1H-^{13}C HMBC NMR (700 MHz and 176 MHz, CD_2Cl_2) spectrum of ^{15}N labelled ***OH-cube**.

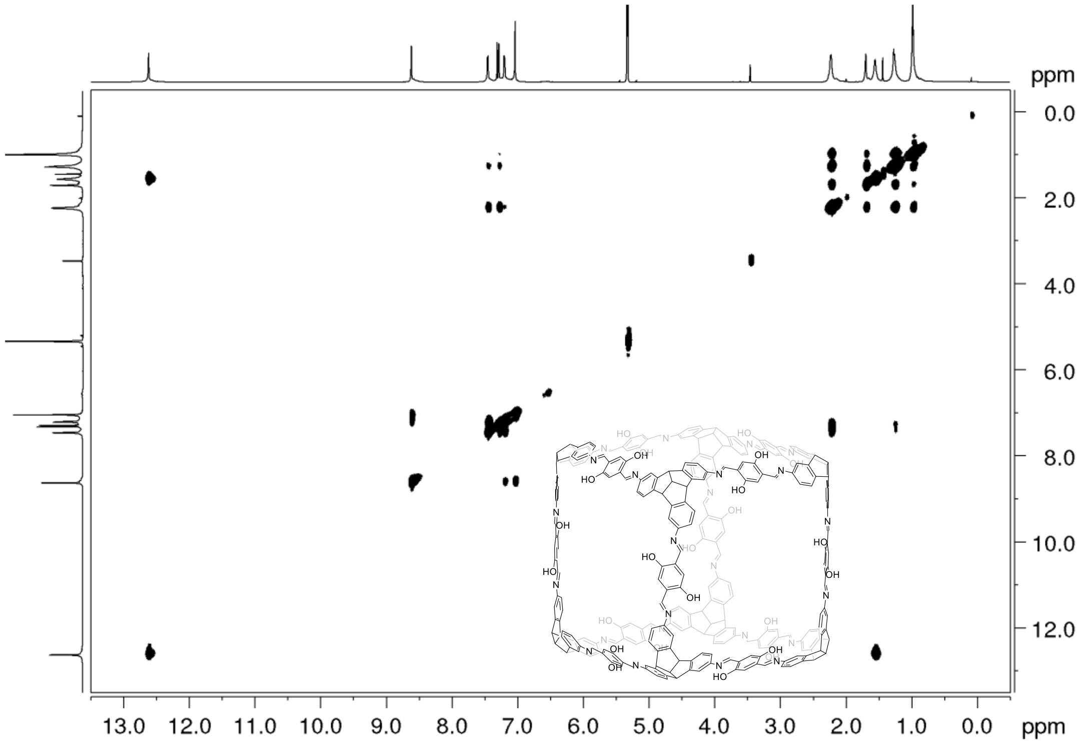

Figure 25. ^{1}H-^{1}H NOESY NMR (700 MHz, CD_2Cl_2) spectrum of ^{15}N labelled ***OH-cube**.

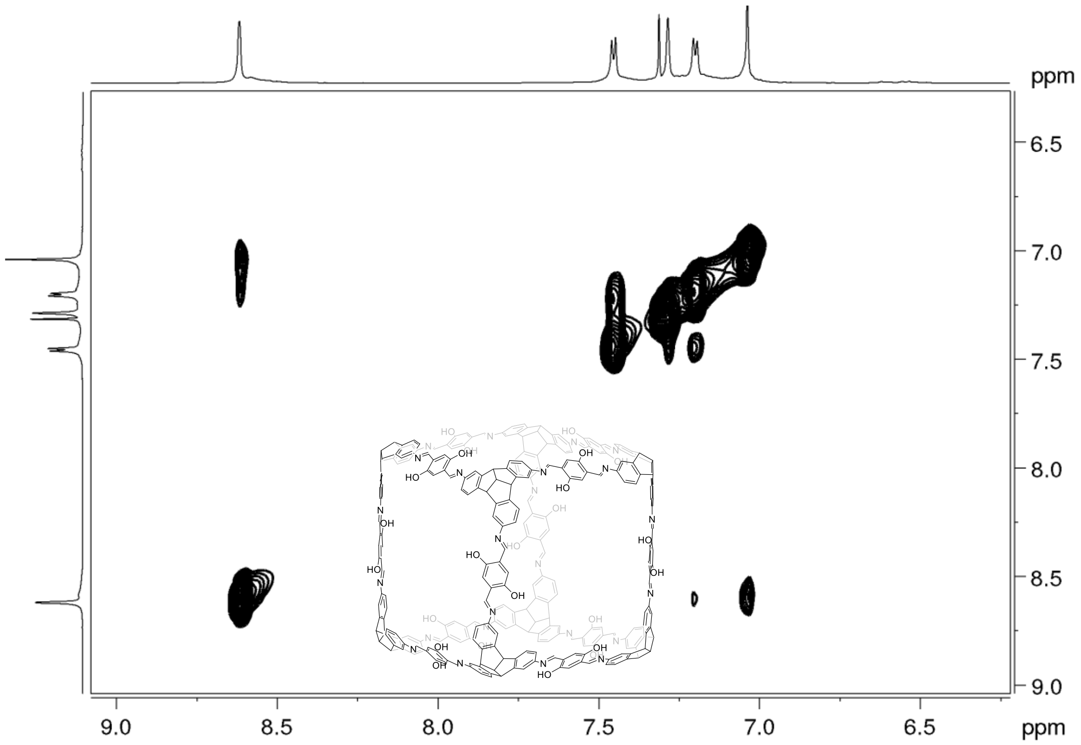

Figure 26. Partial ^{1}H-^{1}H NOESY NMR (700 MHz, CD_2Cl_2) spectrum of ^{15}N labelled ***OH-cube**.

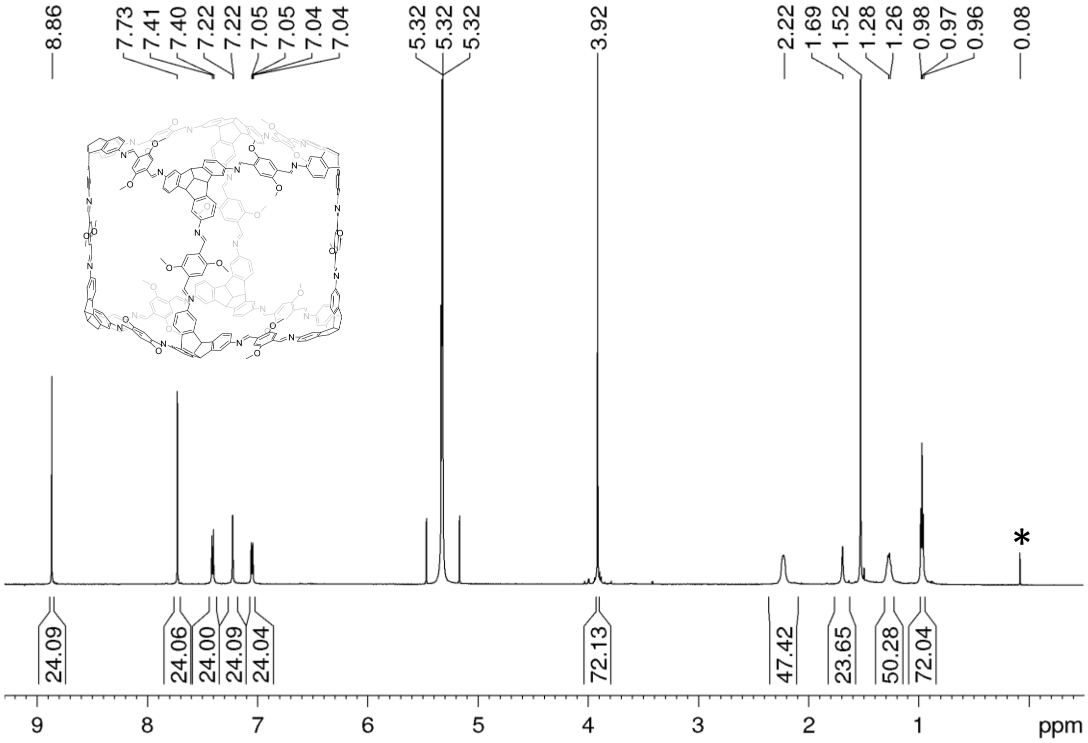

Figure 27. ^{1}H NMR (400 MHz, CD_2Cl_2) spectrum of **OMe-cube**. (*silicone grease).

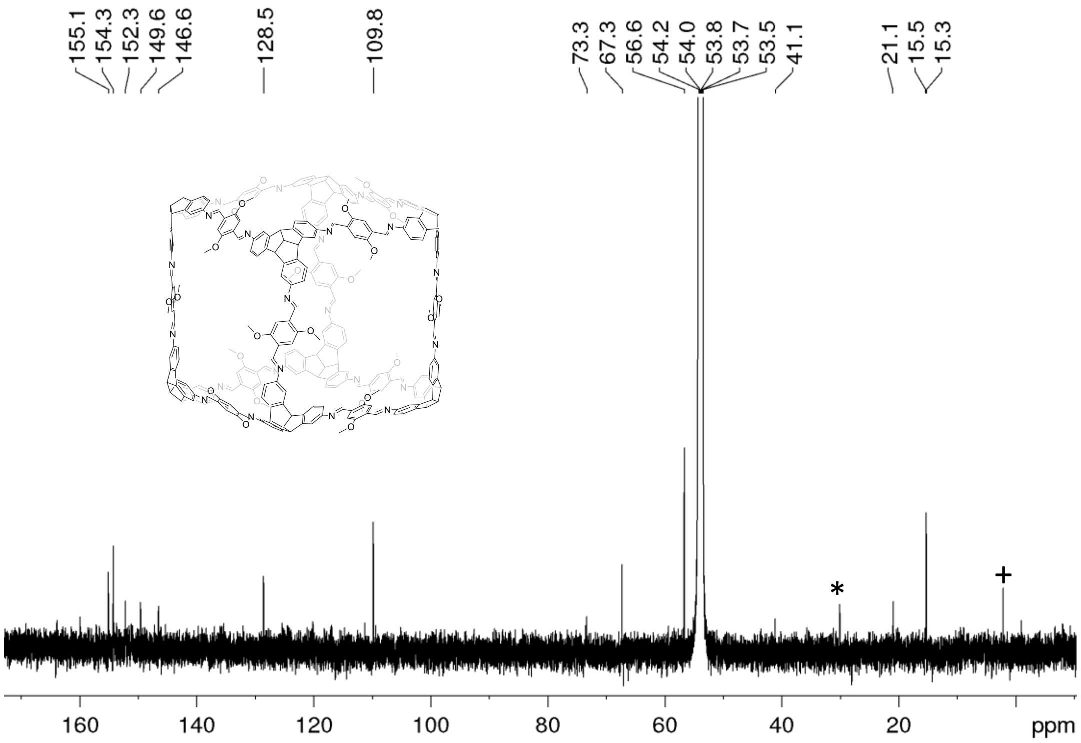

Figure 28. ^{13}C NMR (151 MHz, CD_2Cl_2) spectrum of **OMe-cube**. (*H-grease. $^{+}$silicon grease).

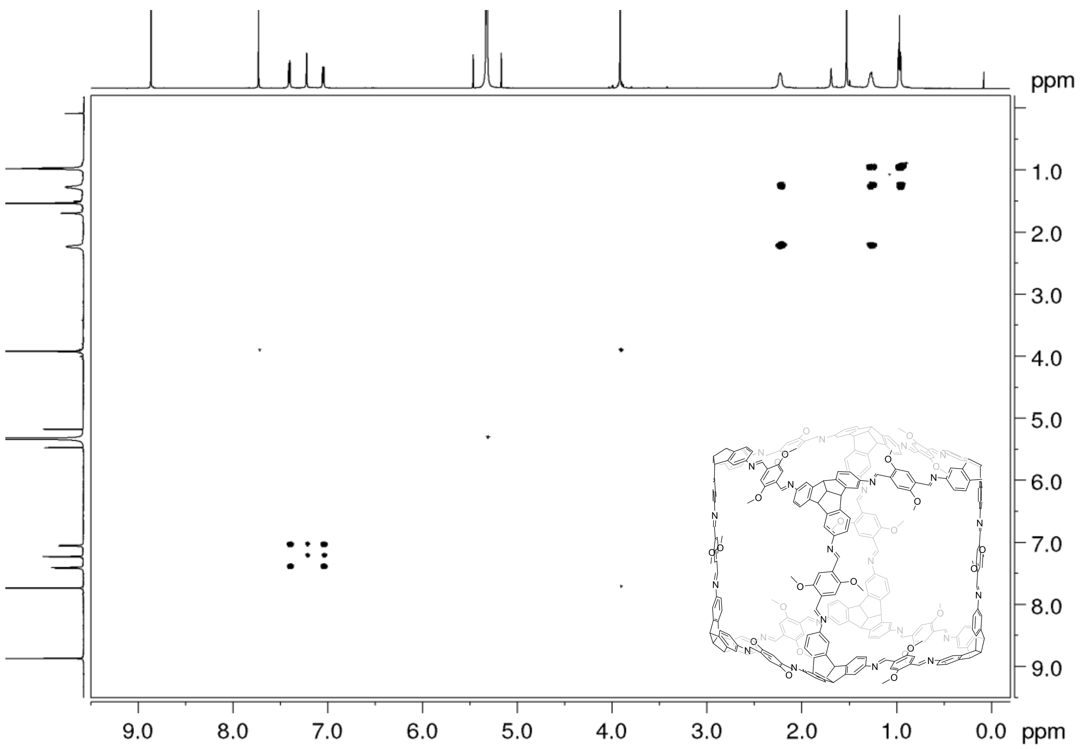

Figure 29. ^1H-^1H COSY NMR spectrum (600 MHz, CD_2Cl_2) of **OMe-cube**.

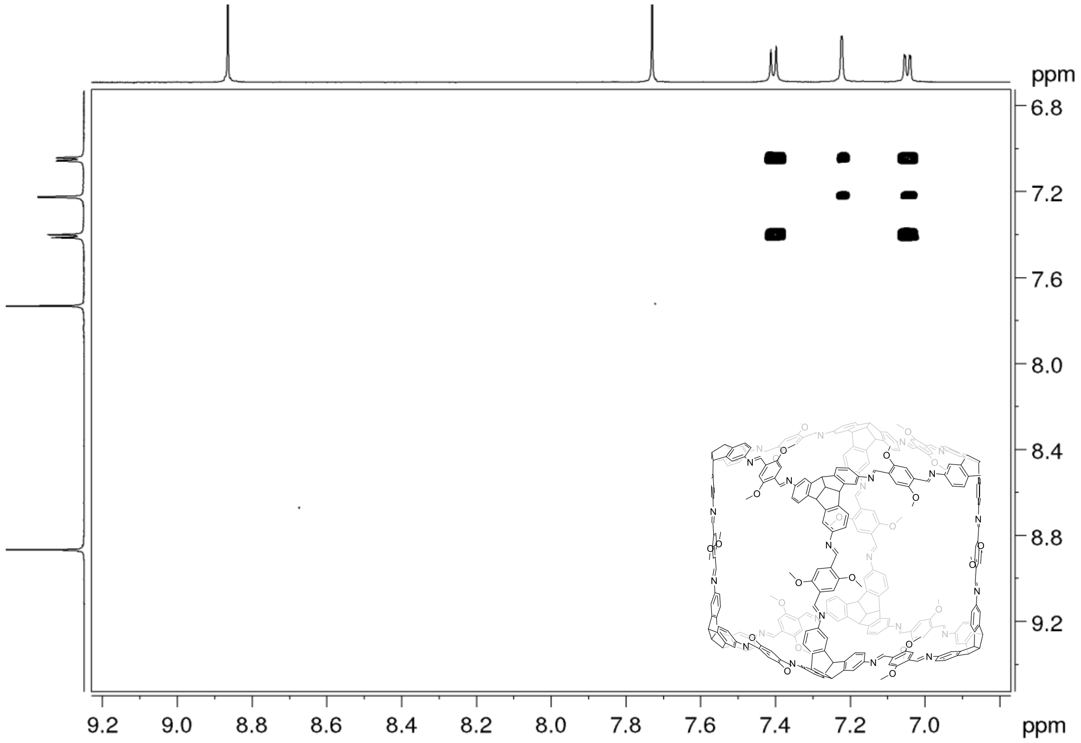

Figure 30. Partial ^1H-^1H COSY NMR spectrum (600 MHz, CD_2Cl_2) of **OMe-cube**.

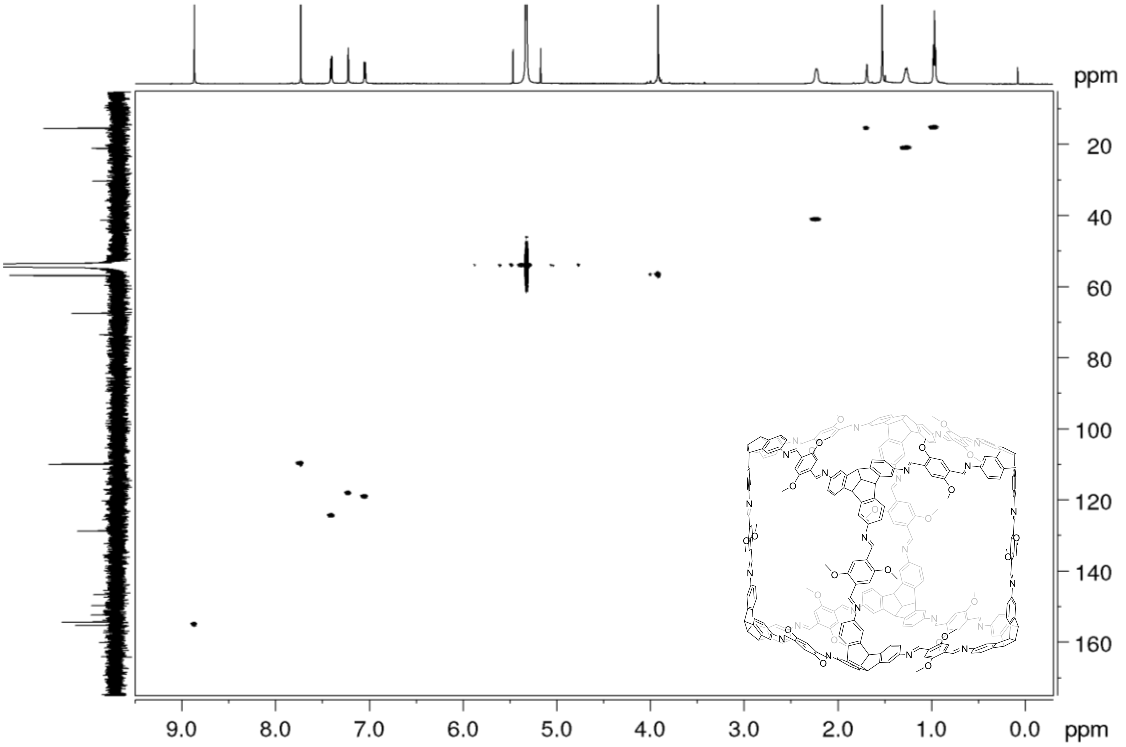

Figure 31. ^{1}H-^{13}C HSQC NMR (600 MHz and 151 MHz, CD_2Cl_2) spectrum of **OMe-cube**.

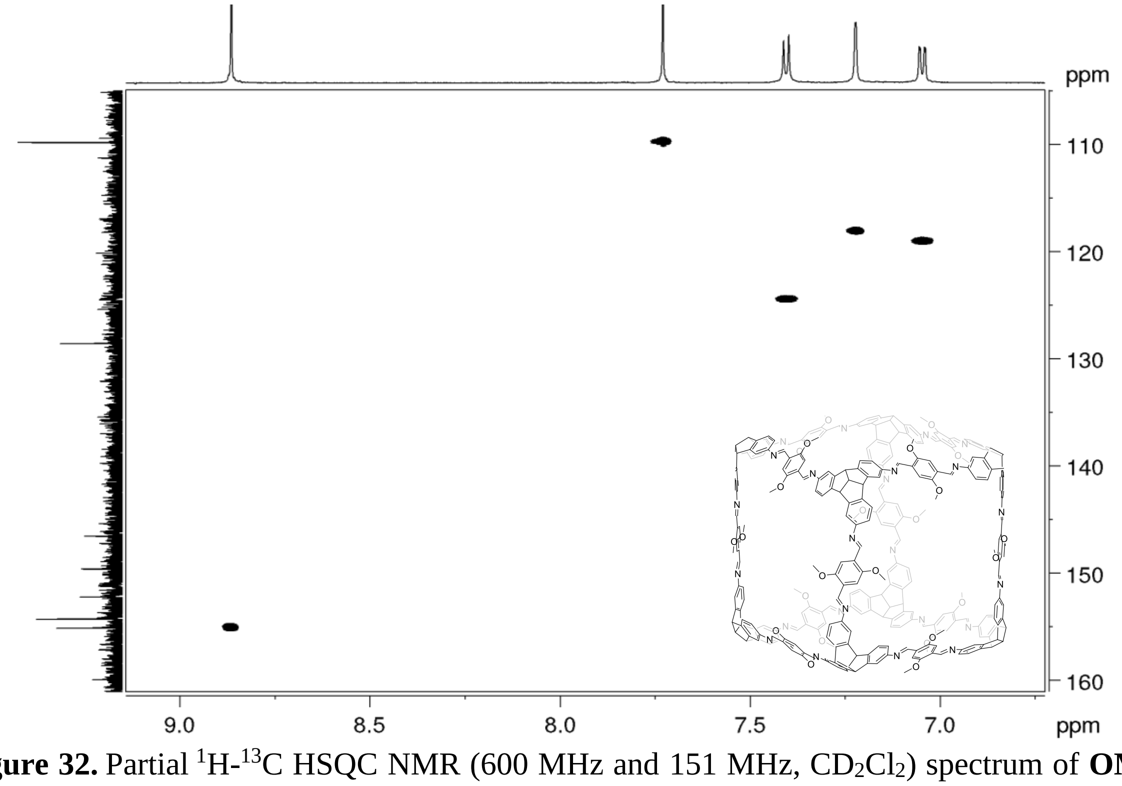

Figure 32. Partial ^{1}H-^{13}C HSQC NMR (600 MHz and 151 MHz, CD_2Cl_2) spectrum of **OMe-cube**.

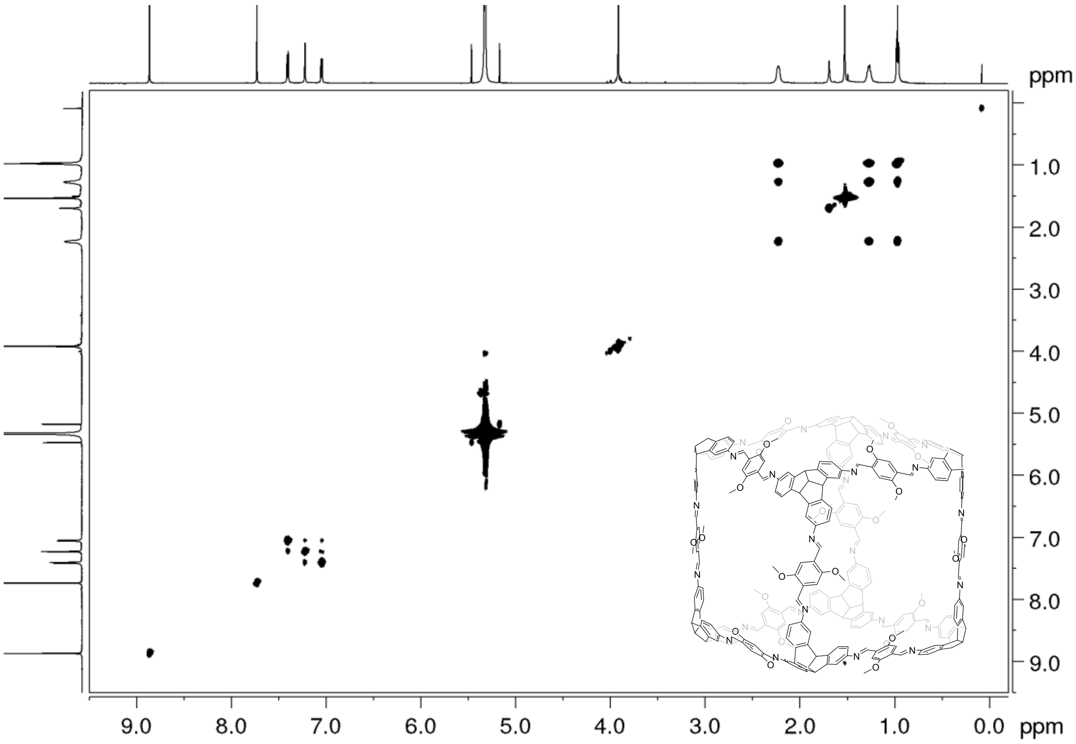

Figure 33. ^{1}H-^{1}H TOCSY NMR (600 MHz, CD_2Cl_2) spectrum of **OMe-cube**.

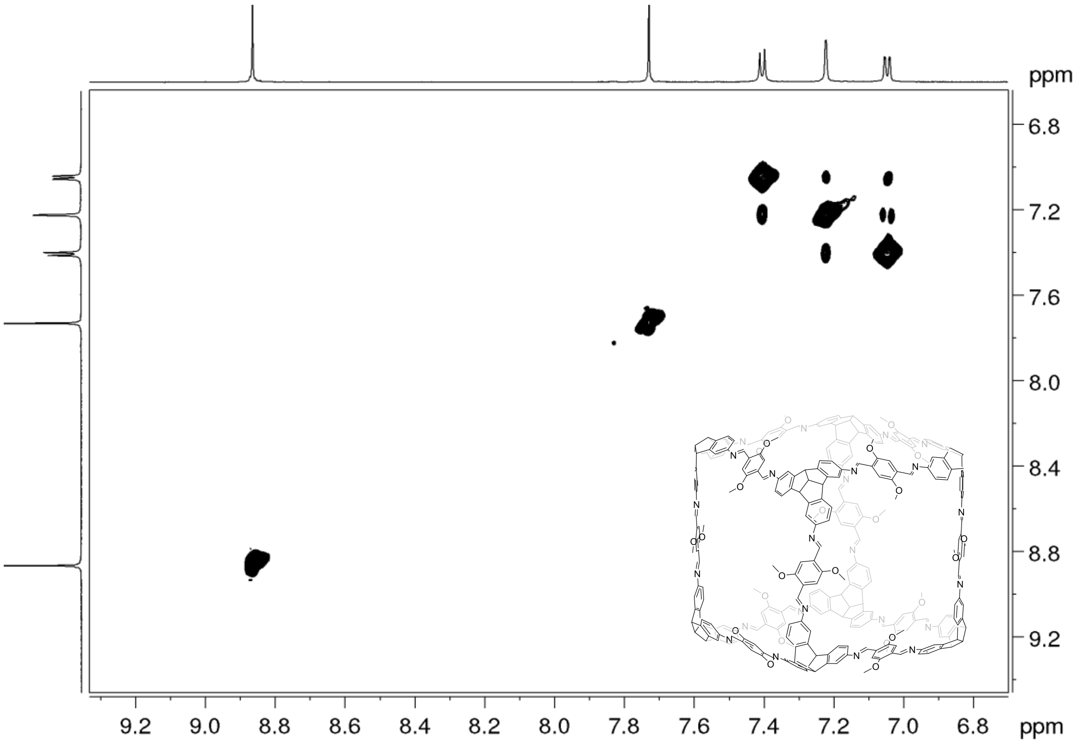

Figure 34. Partial ^{1}H-^{1}H TOCSY NMR (600 MHz, CD_2Cl_2) spectrum of **OMe-cube**.

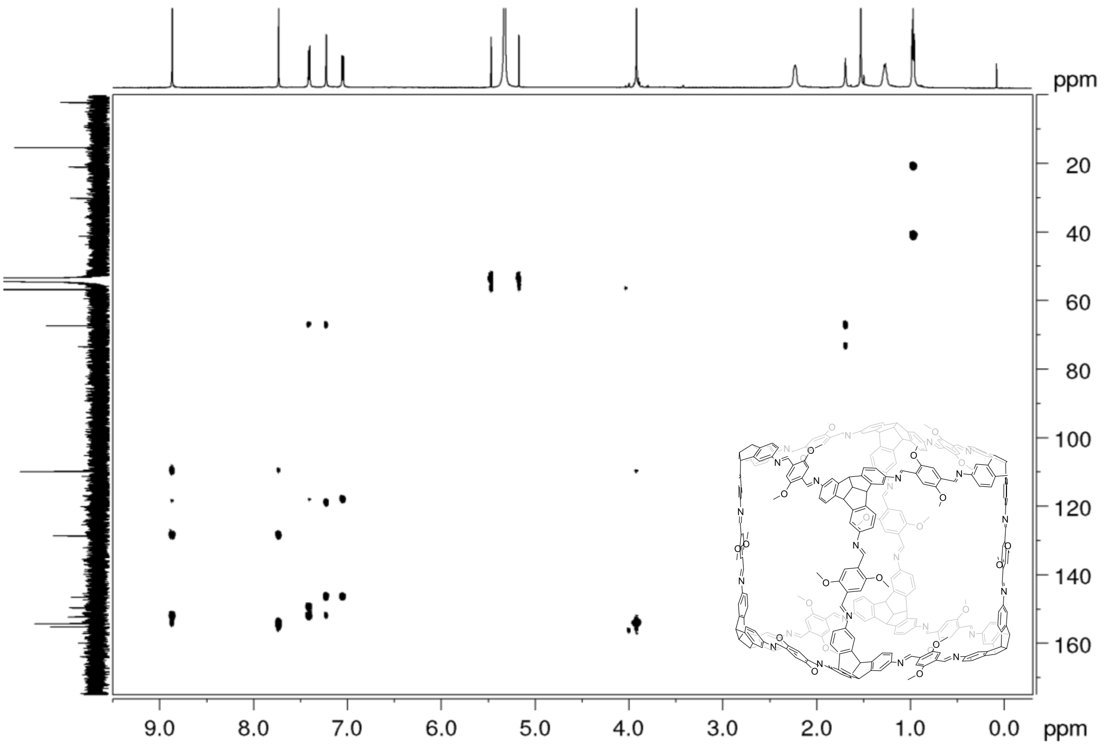

Figure 35. ^{1}H-^{13}C HMBC NMR (600 MHz and 151 MHz, CD_2Cl_2) spectrum of **OMe-cube**.

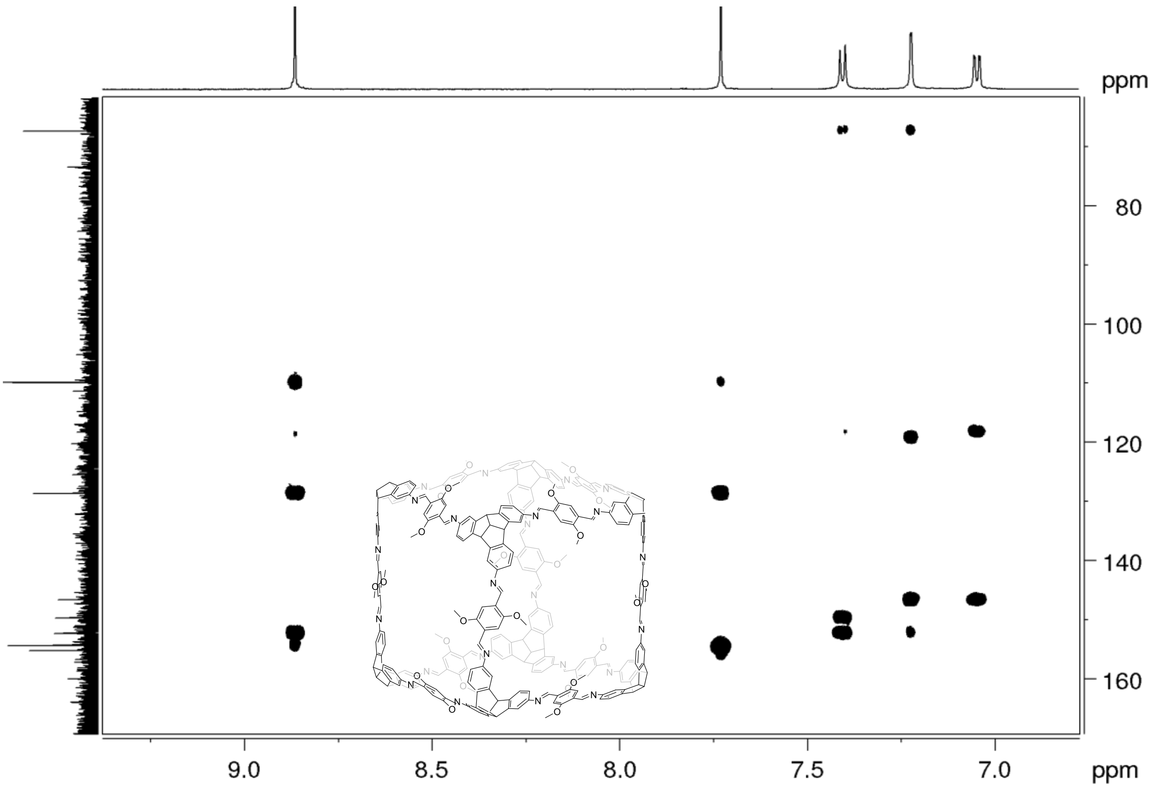

Figure 36. Partial ^{1}H-^{13}C HMBC NMR (600 MHz and 151 MHz, CD_2Cl_2) spectrum of **OMe-cube**.

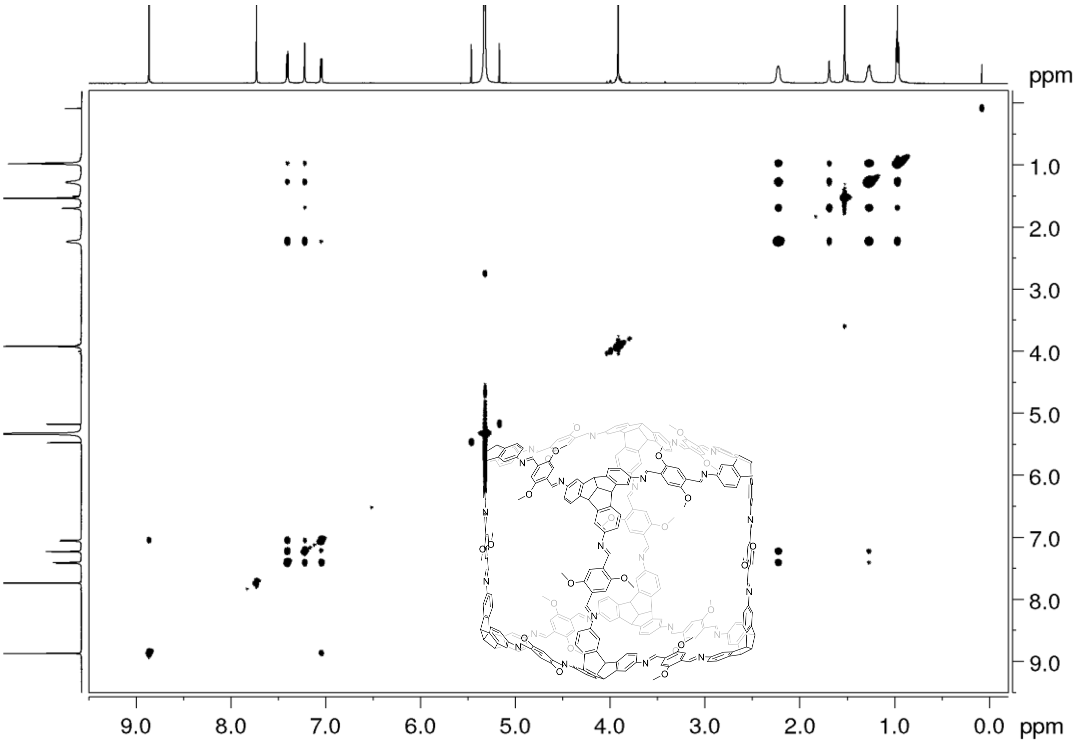

Figure 37. ^{1}H-^{1}H NOESY NMR (600 MHz, CD_2Cl_2) spectrum of **OMe-cube**.

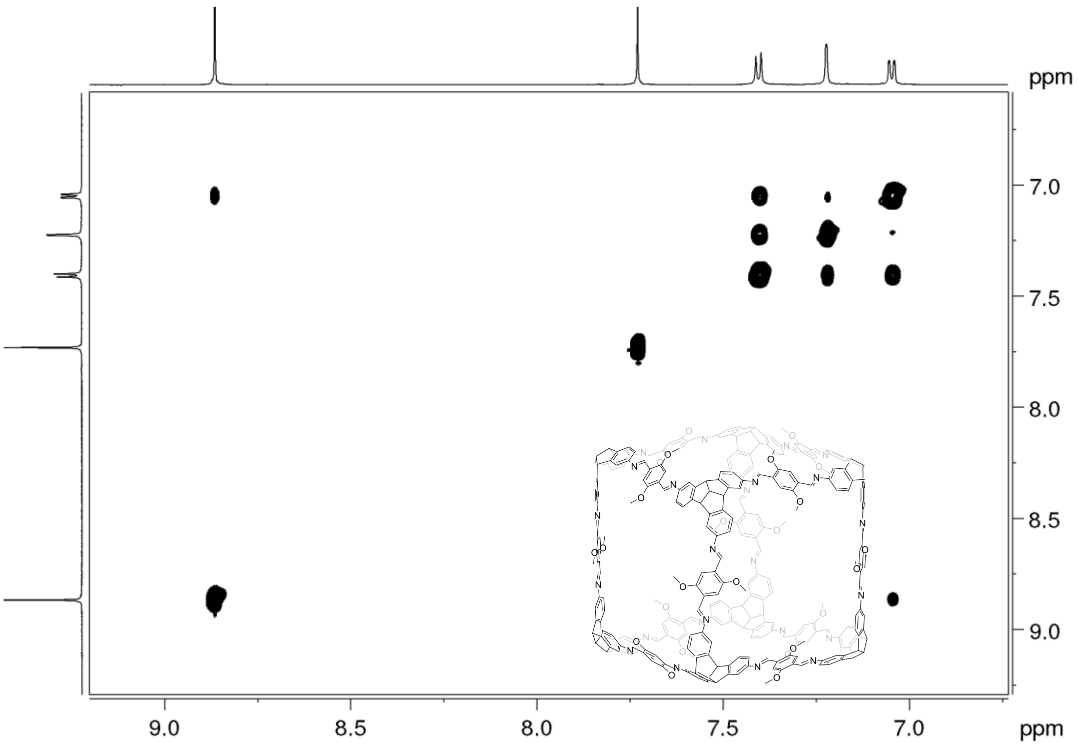

Figure 38. Partial ^{1}H-^{1}H NOESY NMR (600 MHz, CD_2Cl_2) spectrum of **OMe-cube**.

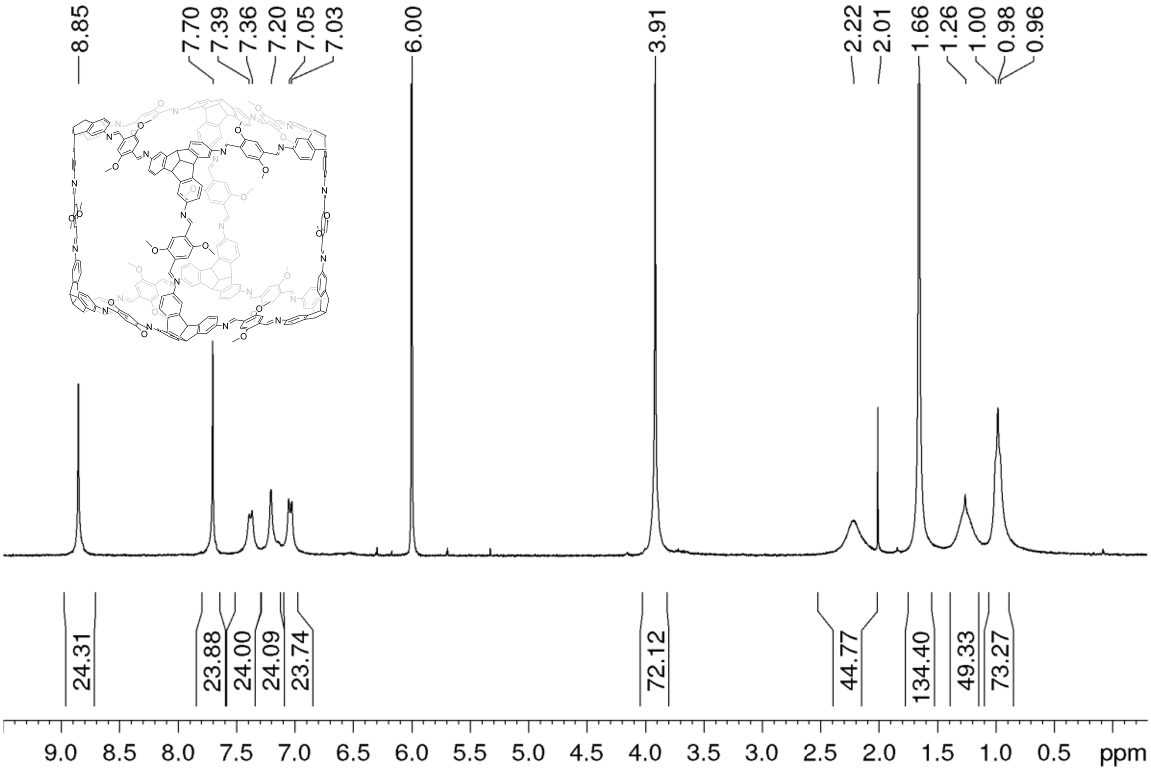

Figure 39. ^{1}H NMR (300 MHz, $C_2D_2Cl_4$) spectrum of **OMe-cube** synthesized in TCE.

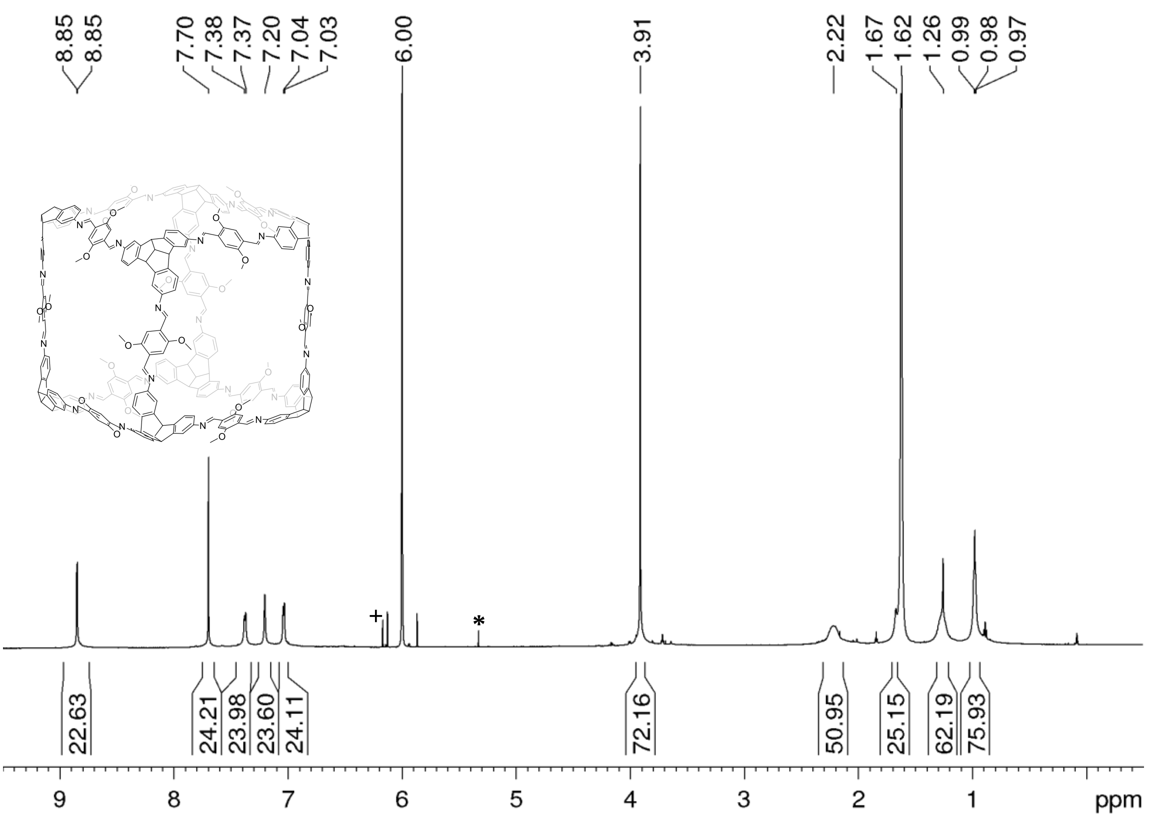

Figure 40. ^{1}H NMR (700 MHz, $C_2D_2Cl_4$) spectrum of **^{15}N labelled *OMe-cube**. ($^{+}CHCl_3$, *DCM).

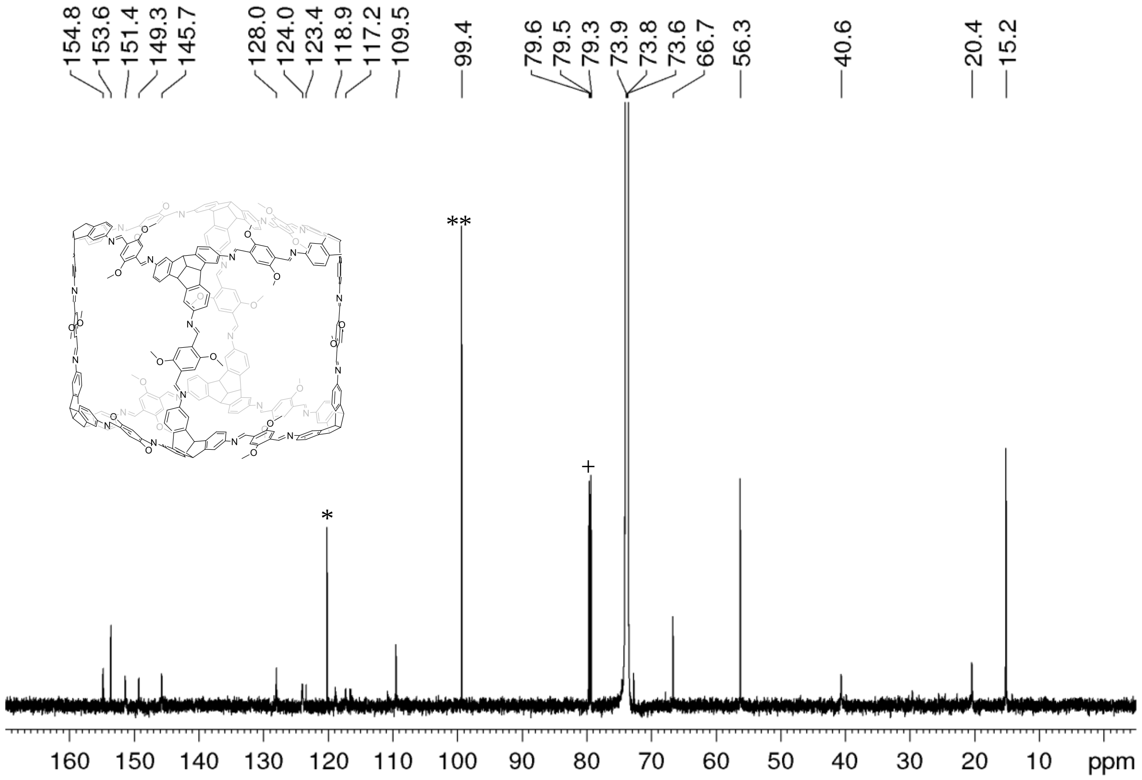

Figure 41. ^{13}C NMR (176 MHz, $C_2D_2Cl_4$) spectrum of **^{15}N labelled** *__OMe-cube__. *C_2Cl_4, **CCl_4, $^+CDCl_3$)

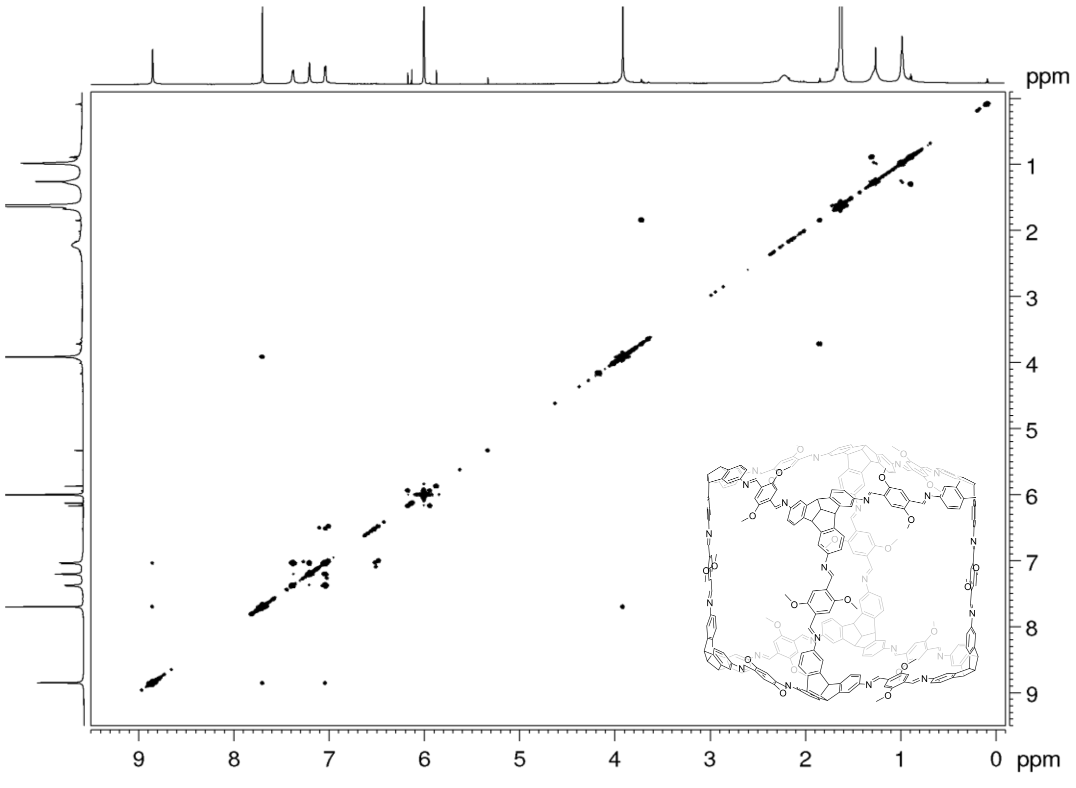

Figure 42. ^{1}H-^{1}H COSY NMR spectrum (700 MHz, $C_2D_2Cl_4$) of **^{15}N labelled** *__OMe-cube__.

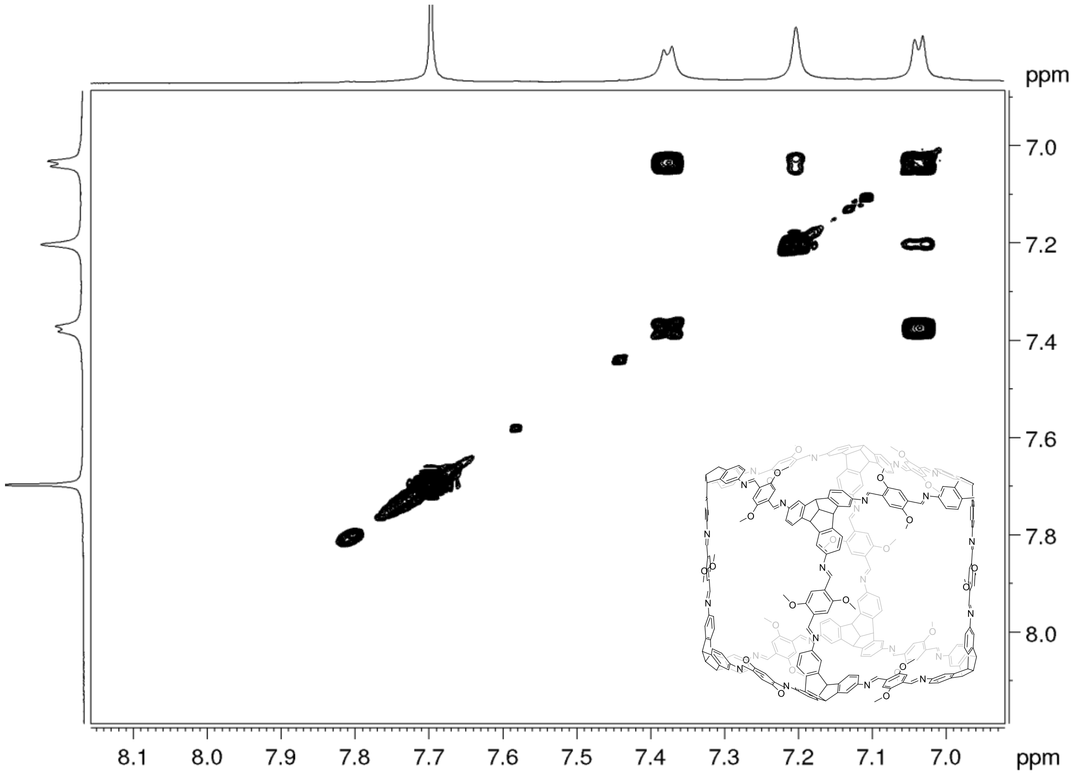

Figure 43. Partial ^{1}H-^{1}H COSY NMR spectrum (700 MHz, $C_2D_2Cl_4$) of **^{15}N labelled** *OMe-**cube**.

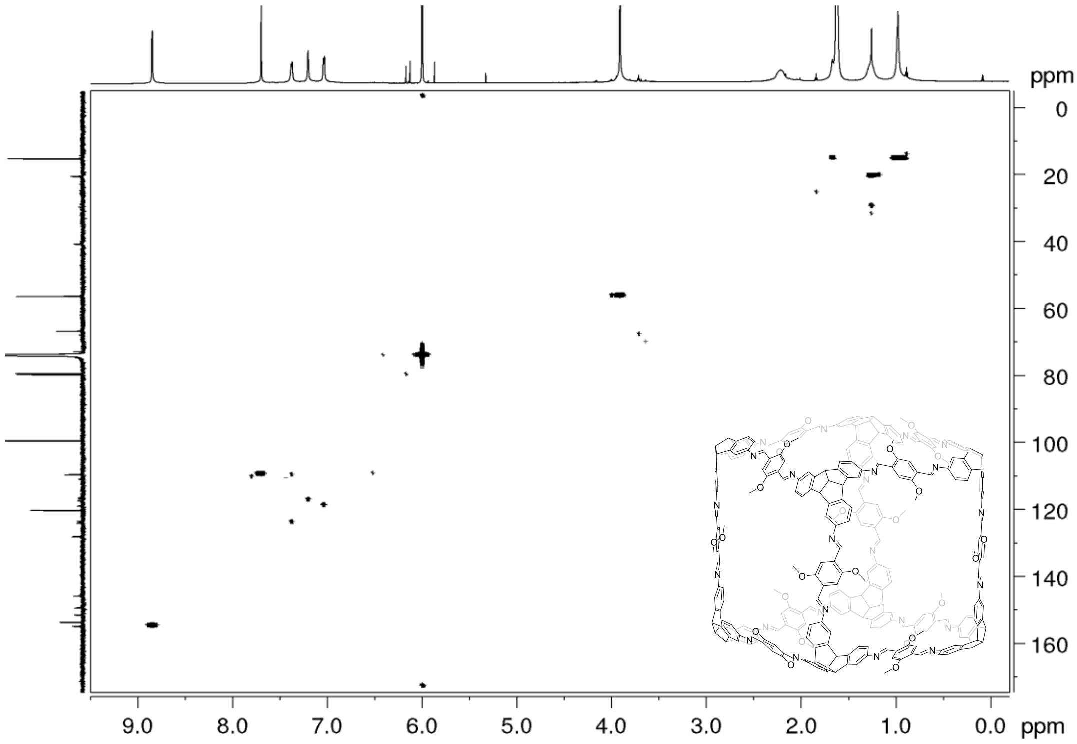

Figure 44. ^{1}H-^{13}C HSQC NMR (700 MHz and 176 MHz, $C_2D_2Cl_4$) spectrum of **^{15}N labelled** ***OMe-cube**.

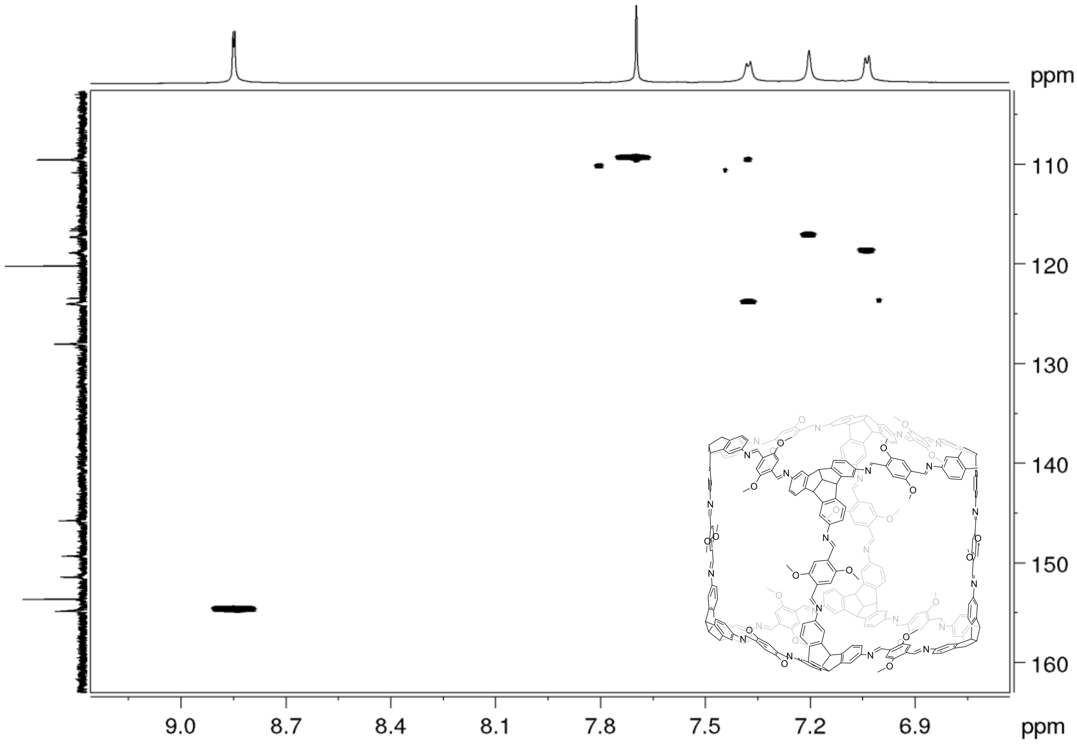

Figure 45. Partial ^{1}H-^{13}C HSQC NMR (700 MHz and 176 MHz, $C_2D_2Cl_4$) spectrum of **^{15}N labelled** *__OMe-cube__.

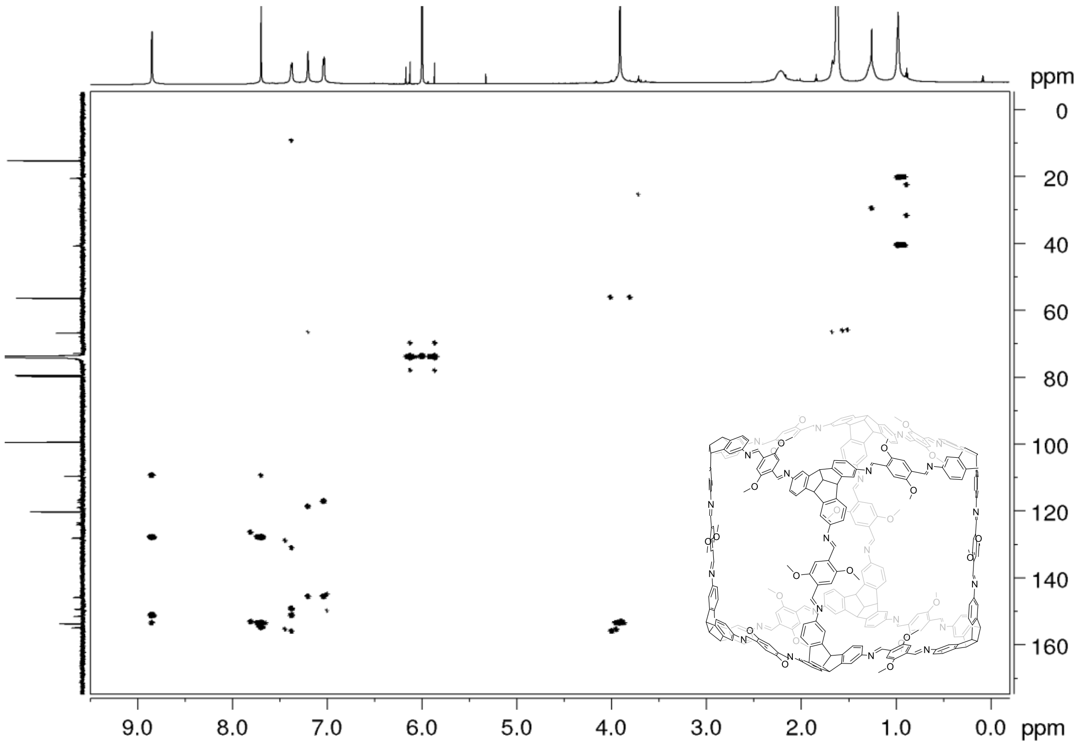

Figure 46. ^{1}H-^{13}C HMBC NMR (700 MHz and 176 MHz, $C_2D_2Cl_4$) spectrum of **^{15}N labelled** *__OMe-cube__.

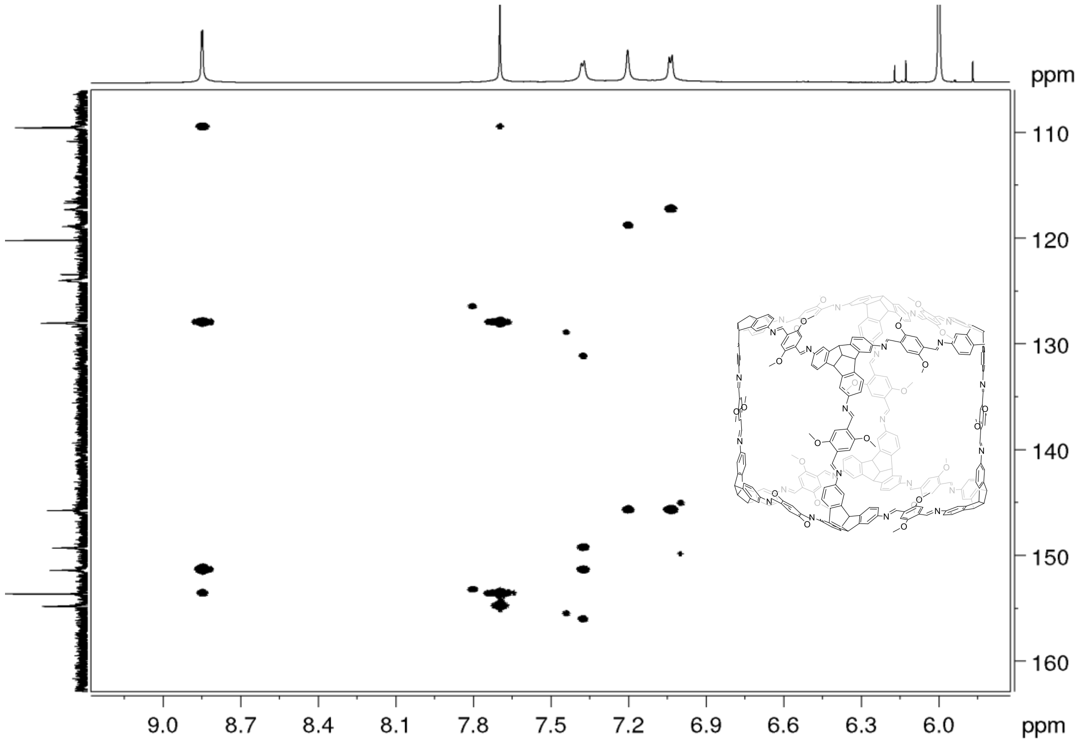

Figure 47. Partial ^{1}H-^{13}C HMBC NMR (700 MHz and 176 MHz, $C_2D_2Cl_4$) spectrum of **^{15}N labelled *OMe-cube**.

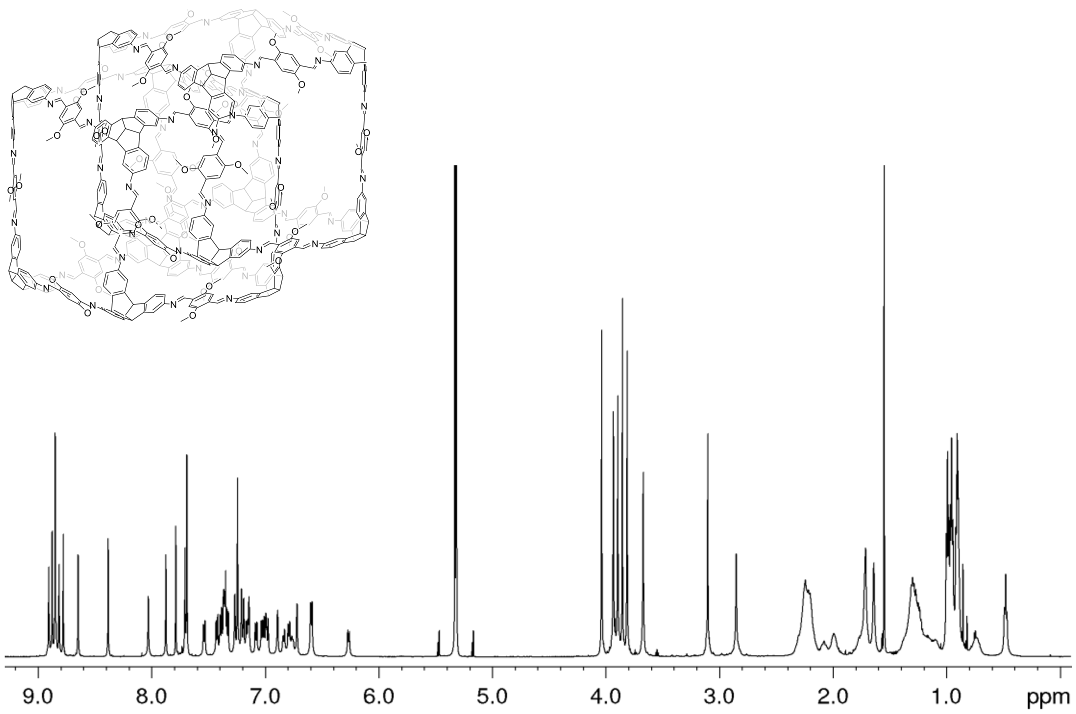

Figure 48. ^{1}H NMR (600 MHz, CD_2Cl_2) spectrum of **(OMe-cube)$_2$**.

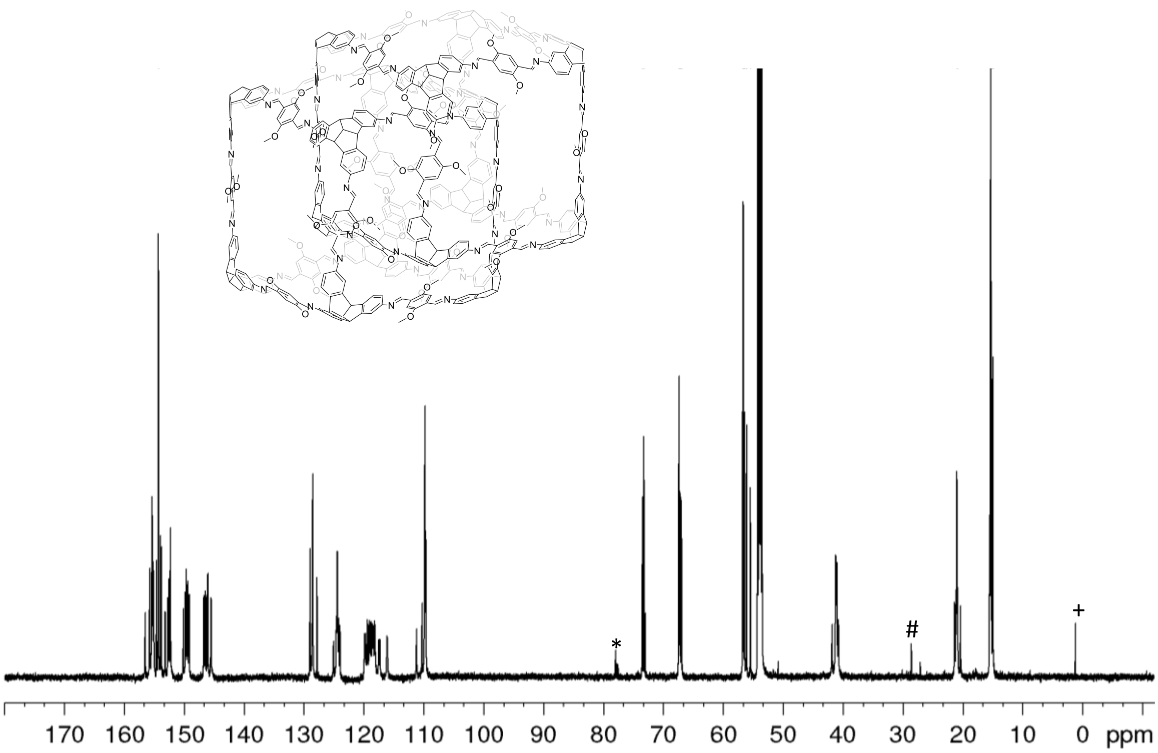

Figure 49. ^{13}C NMR (151 MHz, CD_2Cl_2) spectrum of **(OMe-cube)$_2$**. ($^{*}CHCl_3$ & $CDCl_3$, $^{\#}$H-grease, $^{+}$silicone grease).

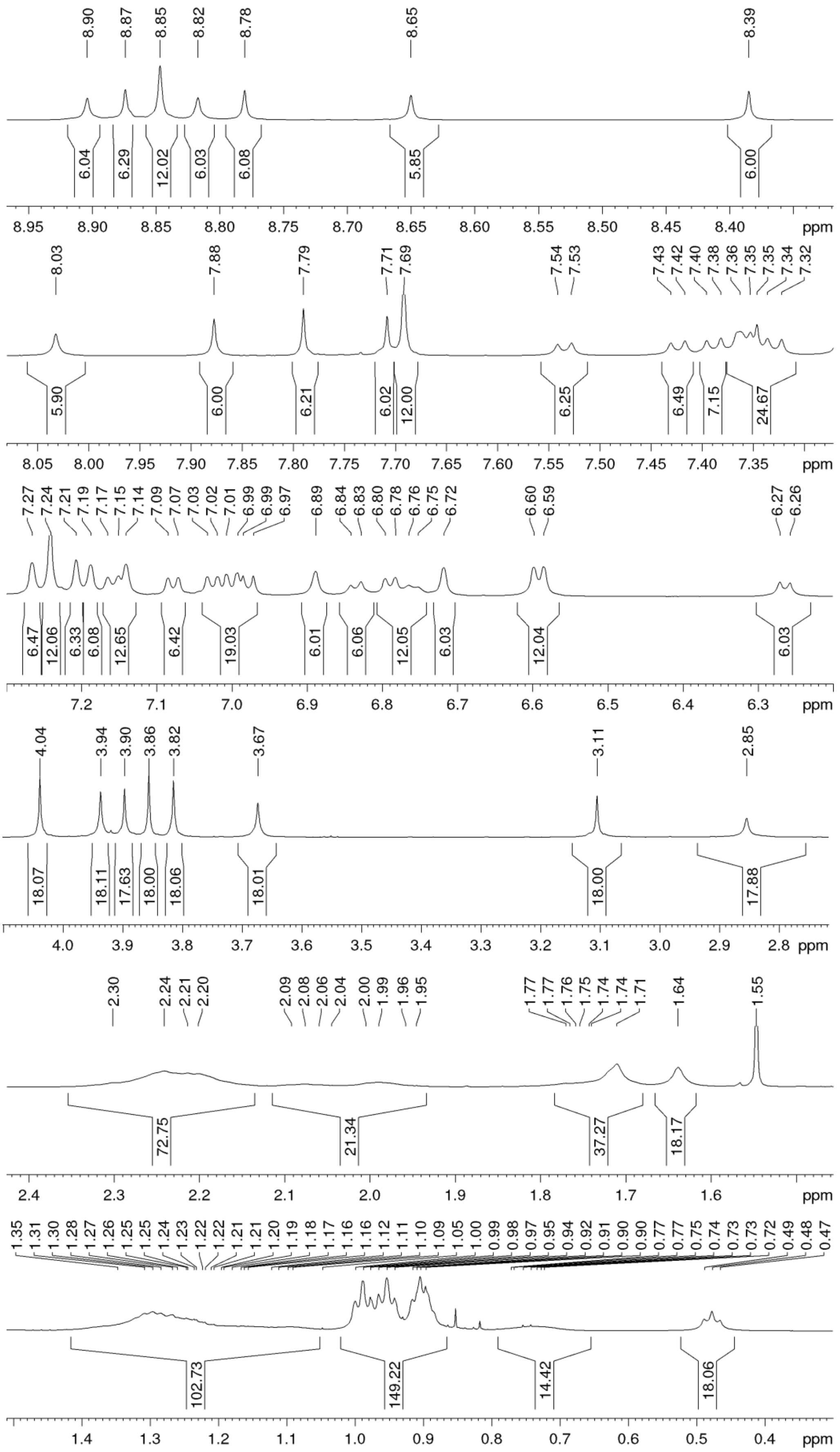

Figure 50. Expanded ^{1}H NMR (600 MHz, CD_2Cl_2) spectrum of **(OMe-cube)$_2$**.

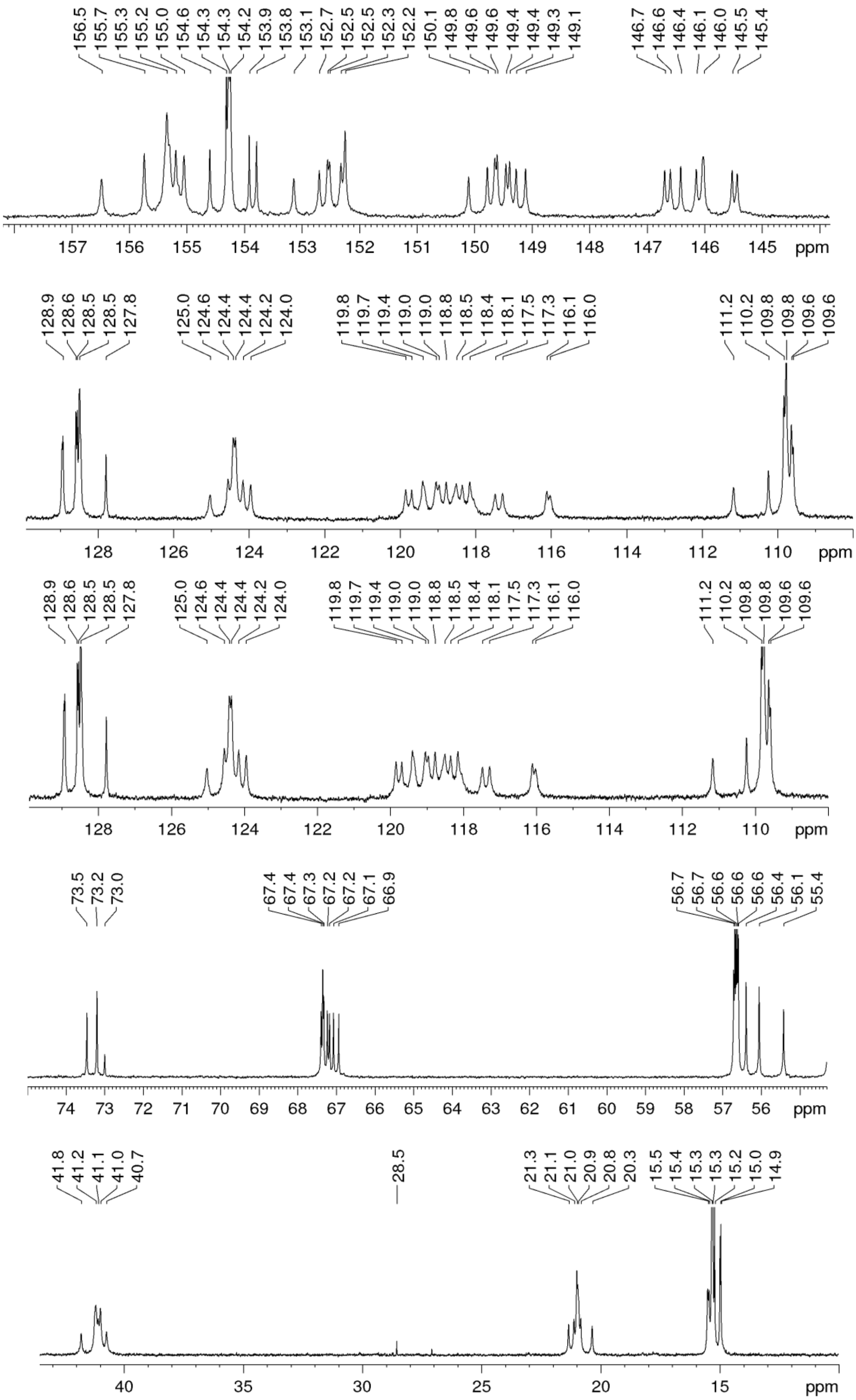

Figure 51. Expanded ^{13}C NMR (151 MHz, CD_2Cl_2) spectrum of **(OMe-cube)$_2$**.

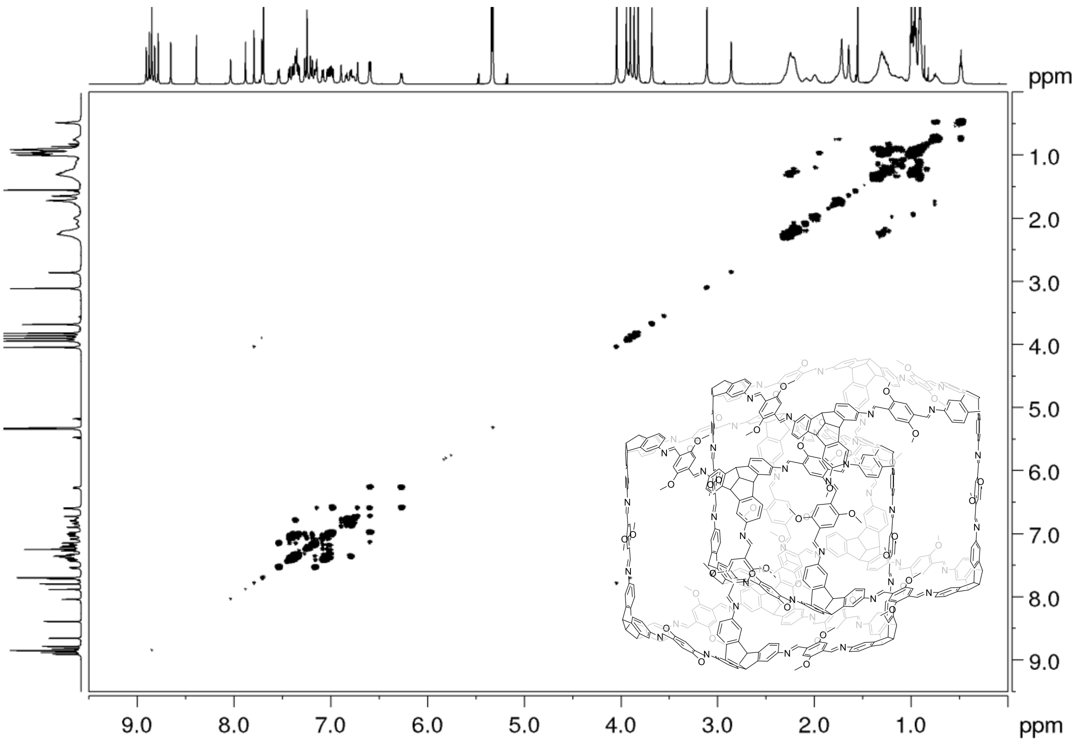

Figure 52. ^{1}H-^{1}H COSY NMR spectrum (600 MHz, CD_2Cl_2) of **(OMe-cube)₂**.

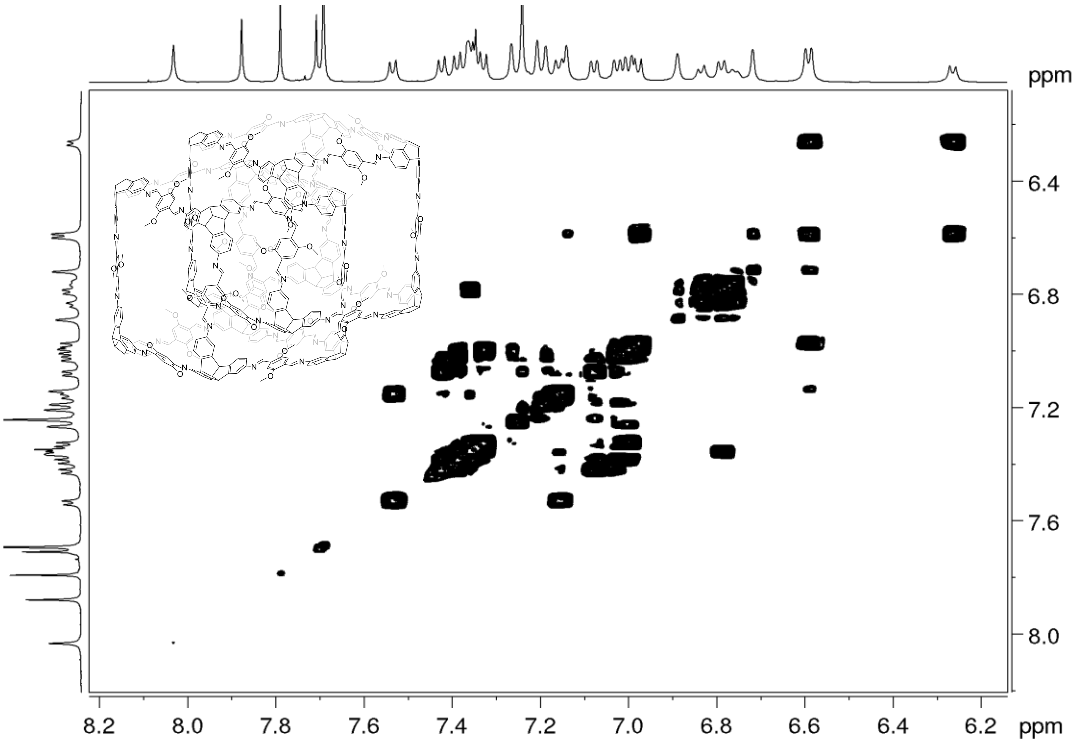

Figure 53. Partial ^{1}H-^{1}H COSY NMR spectrum (600 MHz, CD_2Cl_2) of **(OMe-cube)₂**.

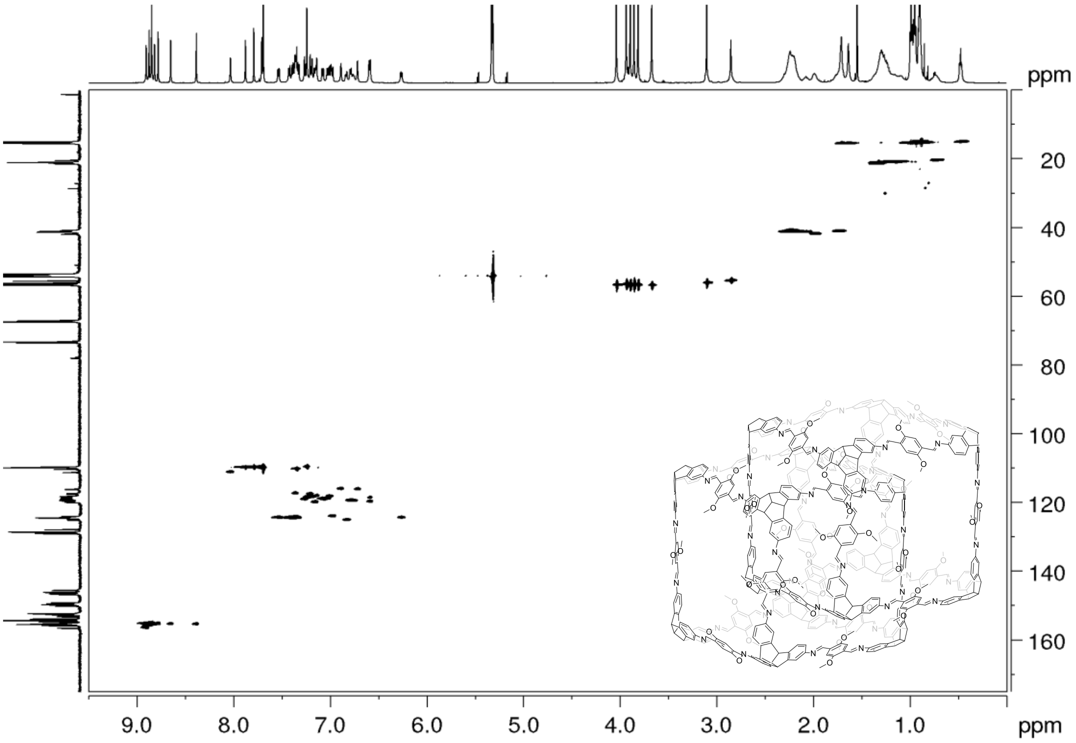

Figure 54. ^{1}H-^{13}C HSQC NMR (600 MHz and 151 MHz, CD_2Cl_2) spectrum of **(OMe-cube)$_2$**.

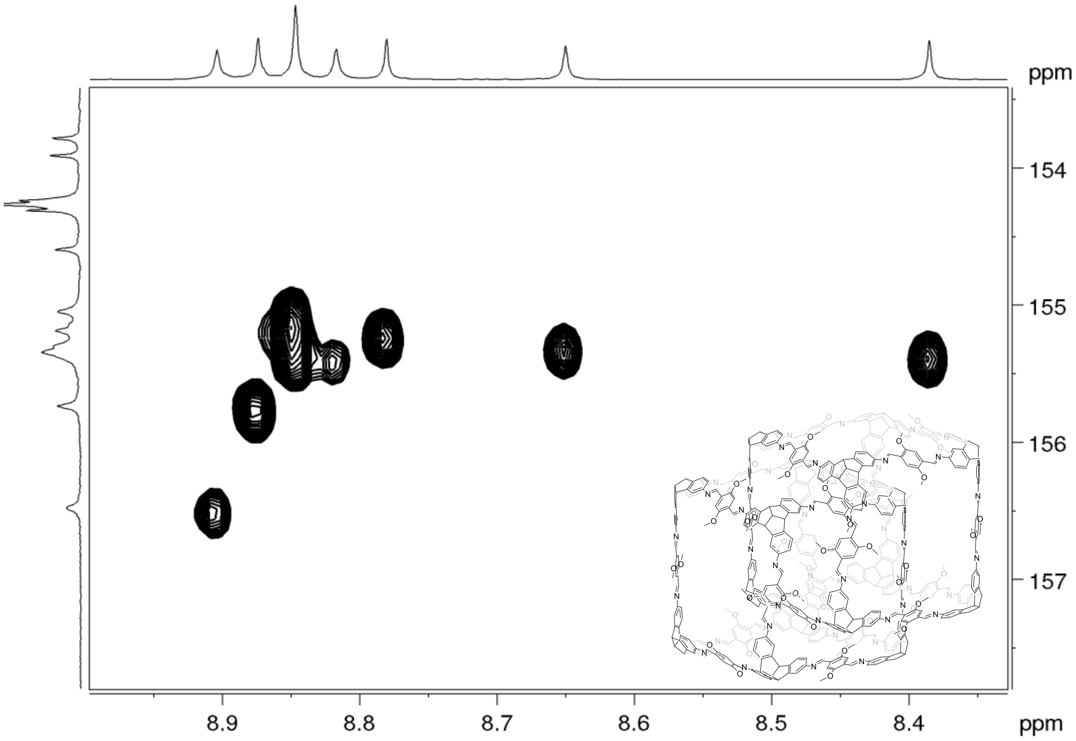

Figure 55. Partial ^{1}H-^{13}C HSQC NMR (600 MHz and 151 MHz, CD_2Cl_2) spectrum of **(OMe-cube)$_2$**.

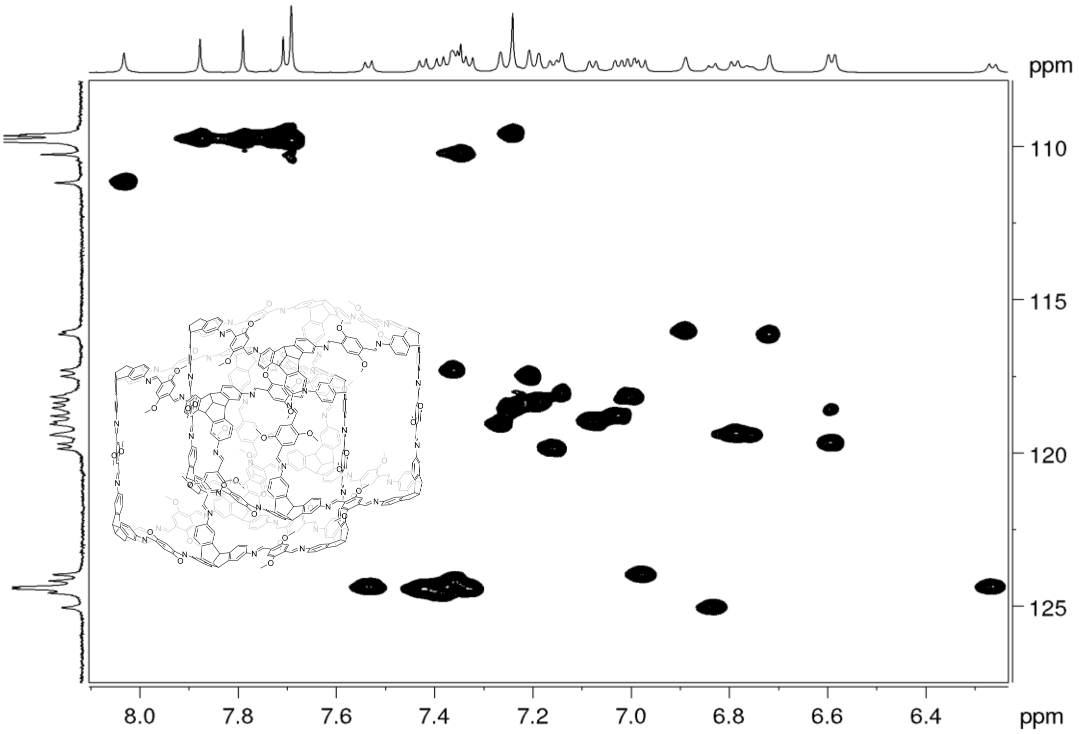

Figure 56. Partial ^{1}H-^{13}C HSQC NMR (600 MHz and 151 MHz, CD_2Cl_2) spectrum of **(OMe-cube)$_2$**.

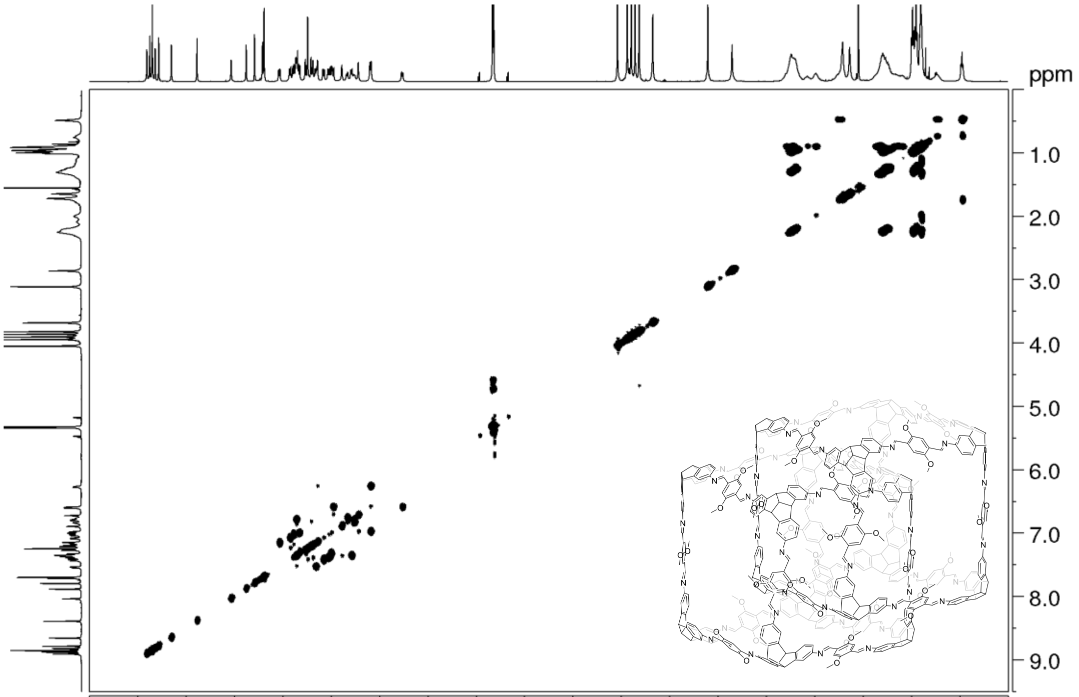

Figure 57. ^{1}H-^{1}H TOCSY NMR (600 MHz, CD_2Cl_2) spectrum of **(OMe-cube)$_2$**.

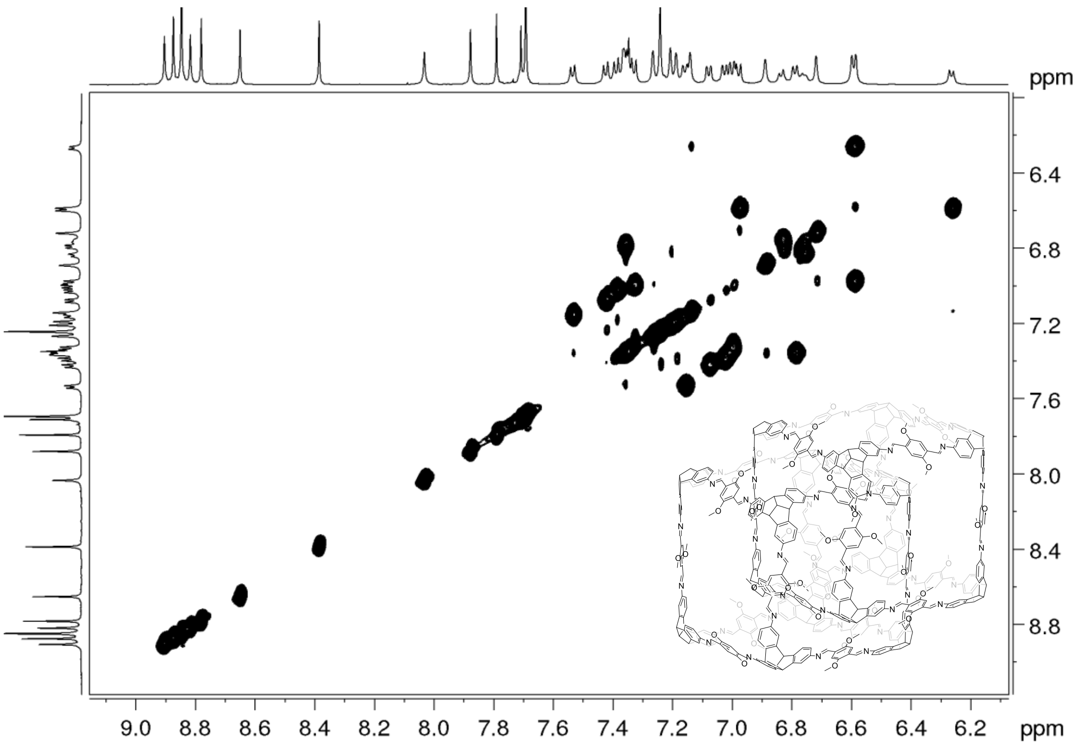

Figure 58. Partial ^{1}H-^{1}H TOCSY NMR (600 MHz, CD_2Cl_2) spectrum of **(OMe-cube)$_2$**.

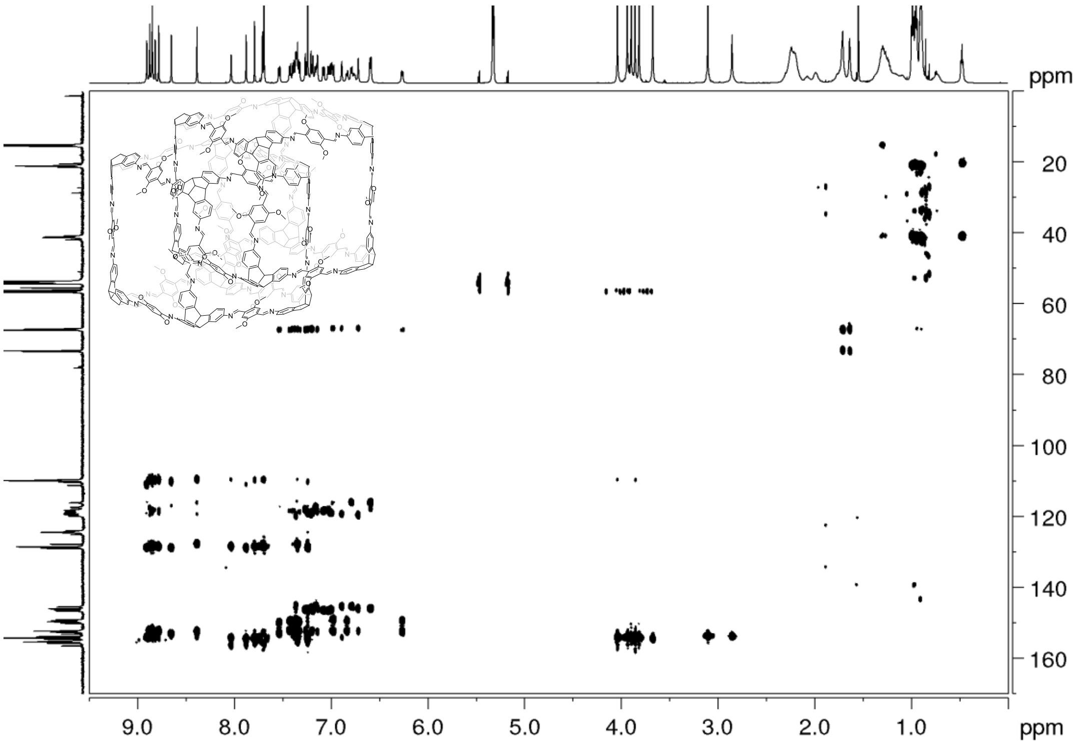

Figure 59. ^{1}H-^{13}C HMBC NMR (600 MHz and 151 MHz, CD_2Cl_2) spectrum of **(OMe-cube)$_2$**.

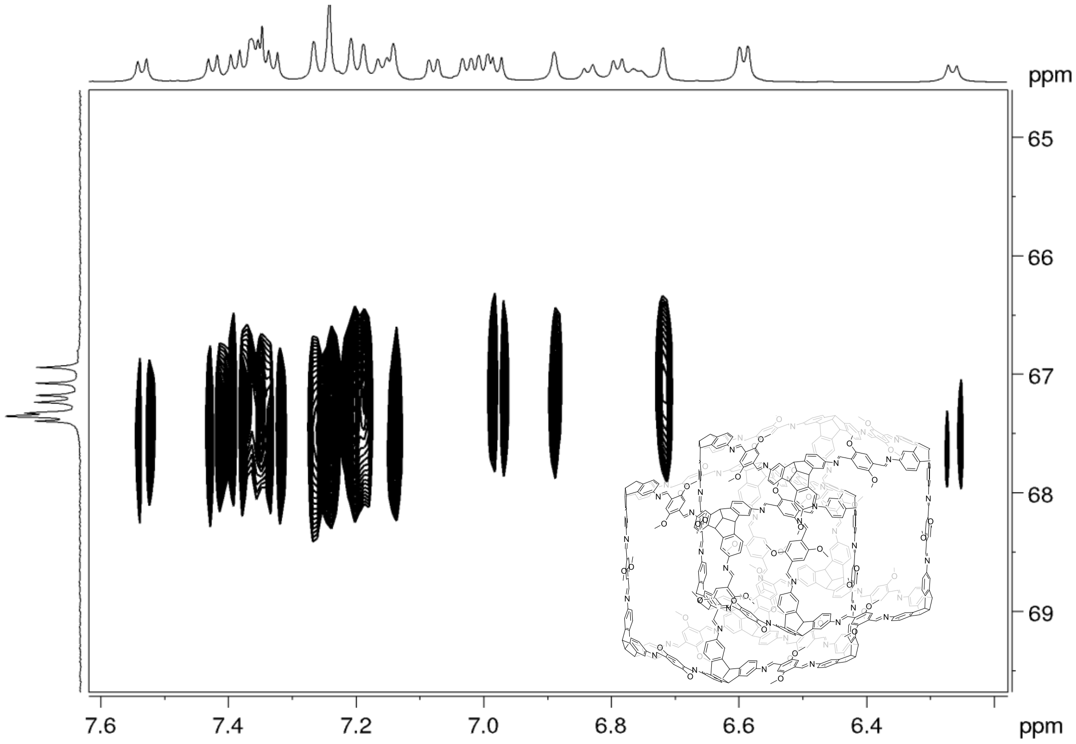

Figure 60. Partial ^{1}H-^{13}C HMBC NMR (600 MHz and 151 MHz, CD_2Cl_2) spectrum of **(OMe-cube)$_2$**.

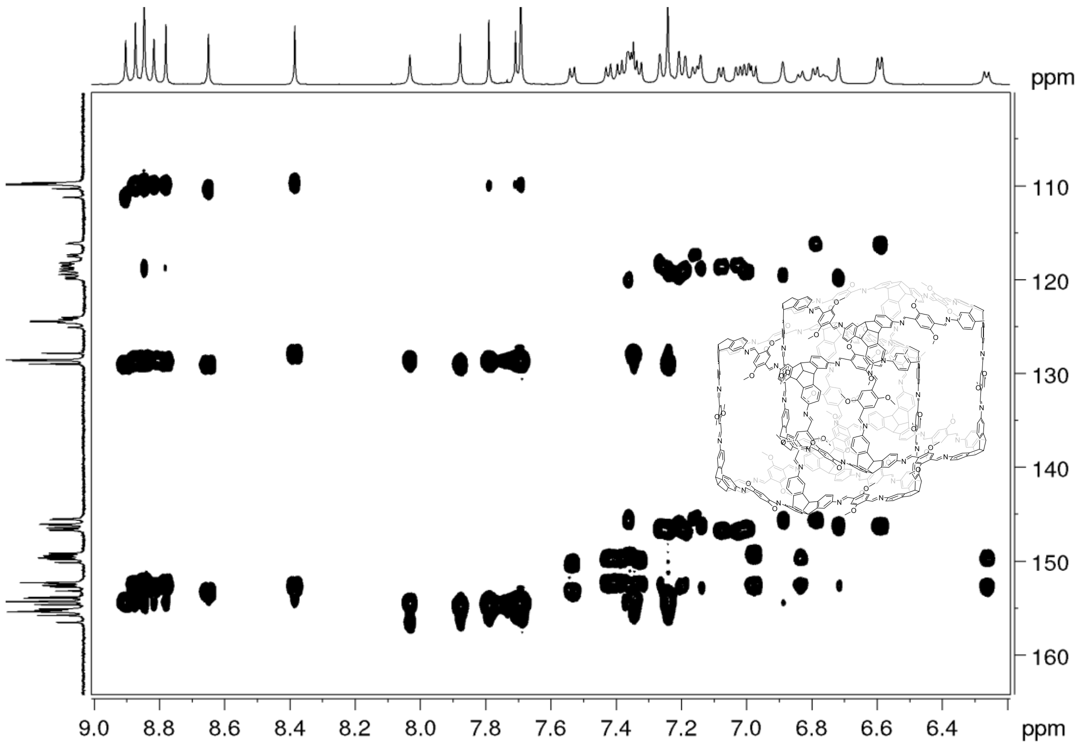

Figure 61. Partial ^{1}H-^{13}C HMBC NMR (600 MHz and 151 MHz, CD_2Cl_2) spectrum of **(OMe-cube)$_2$**.

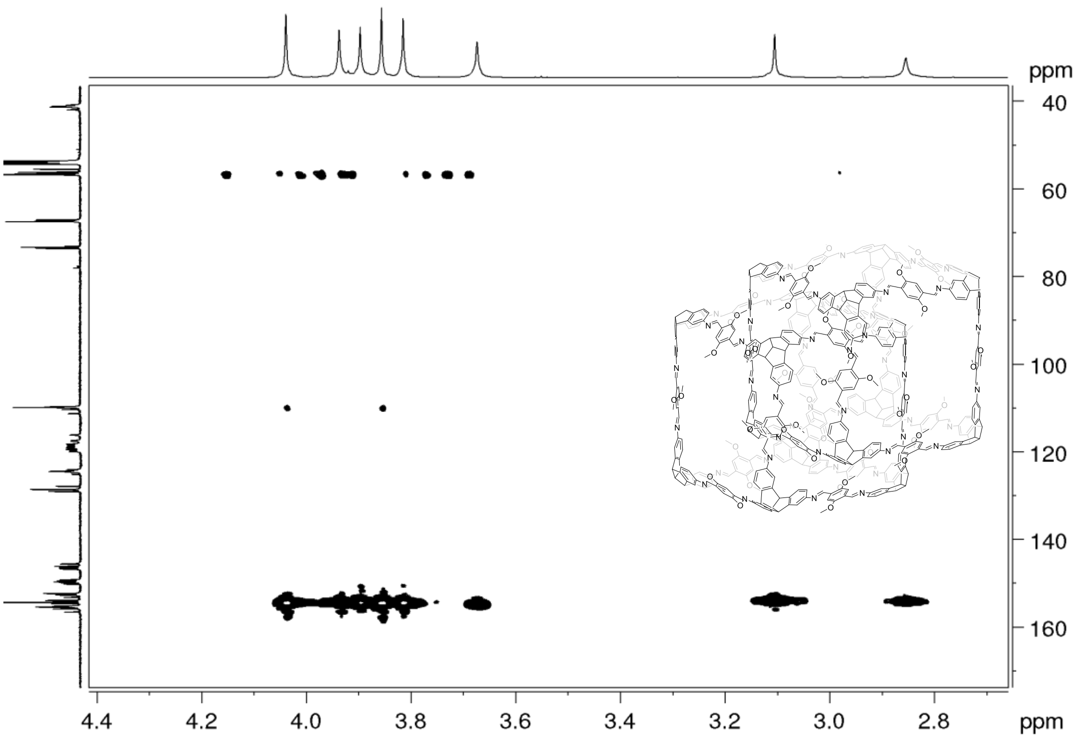

Figure 62. Partial ^{1}H-^{13}C HMBC NMR (600 MHz and 151 MHz, CD_2Cl_2) spectrum of **(OMe-cube)$_2$**.

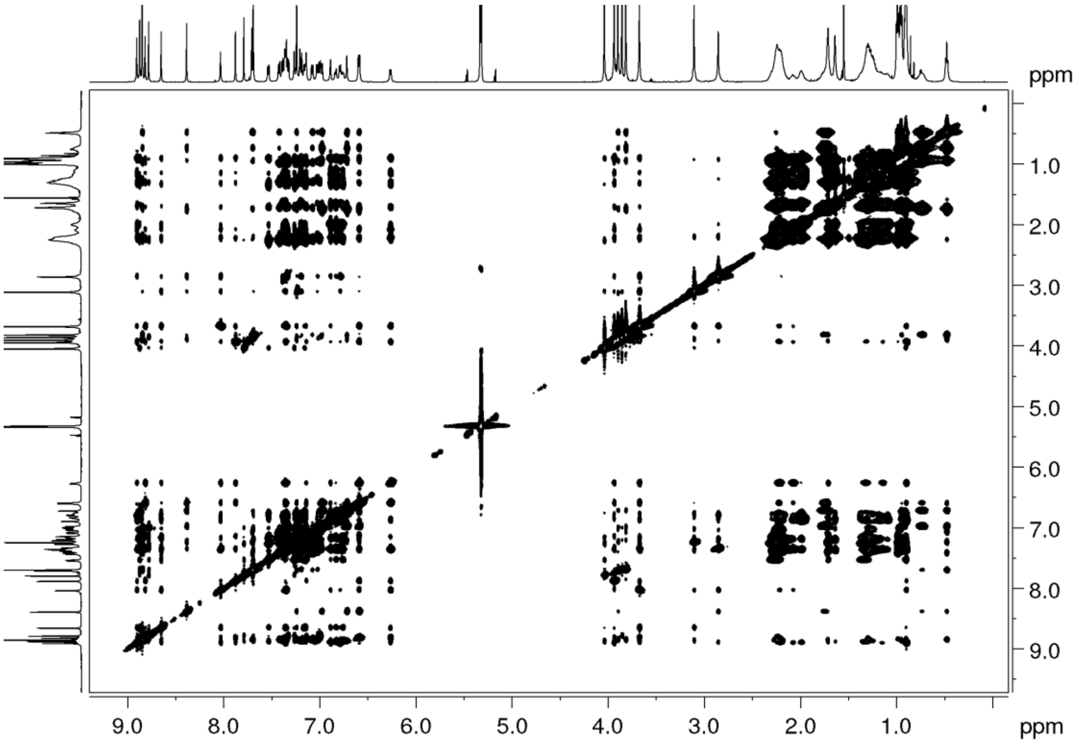

Figure 63. ^{1}H-^{1}H NOESY NMR (600 MHz, CD_2Cl_2) spectrum of **(OMe-cube)$_2$**.

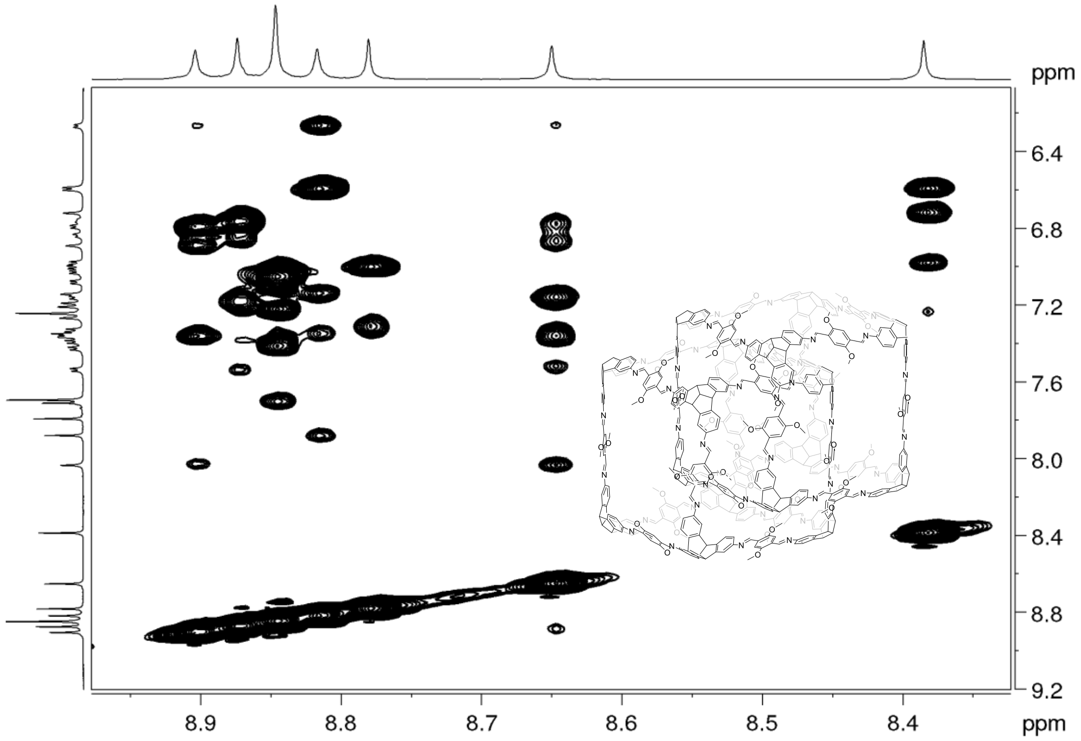

Figure 64. Partial ^{1}H-^{1}H NOESY NMR (600 MHz, CD_2Cl_2) spectrum of **(OMe-cube)$_2$**.

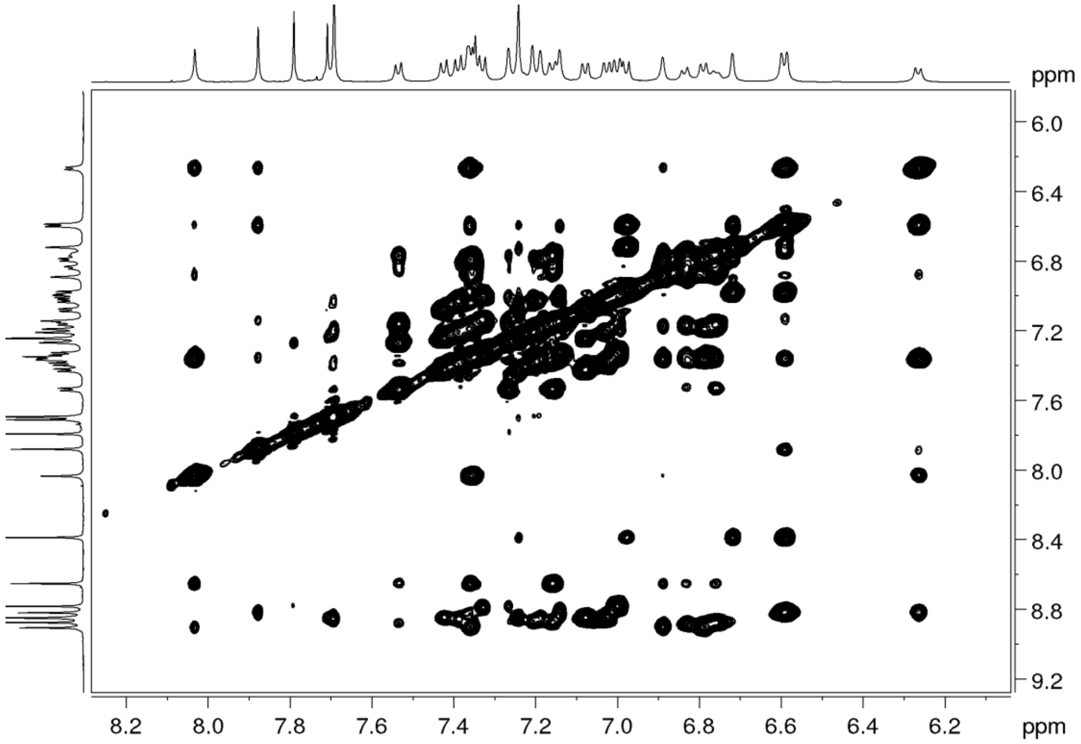

Figure 65. Partial ^{1}H-^{1}H NOESY NMR (600 MHz, CD_2Cl_2) spectrum of **(OMe-cube)$_2$**.

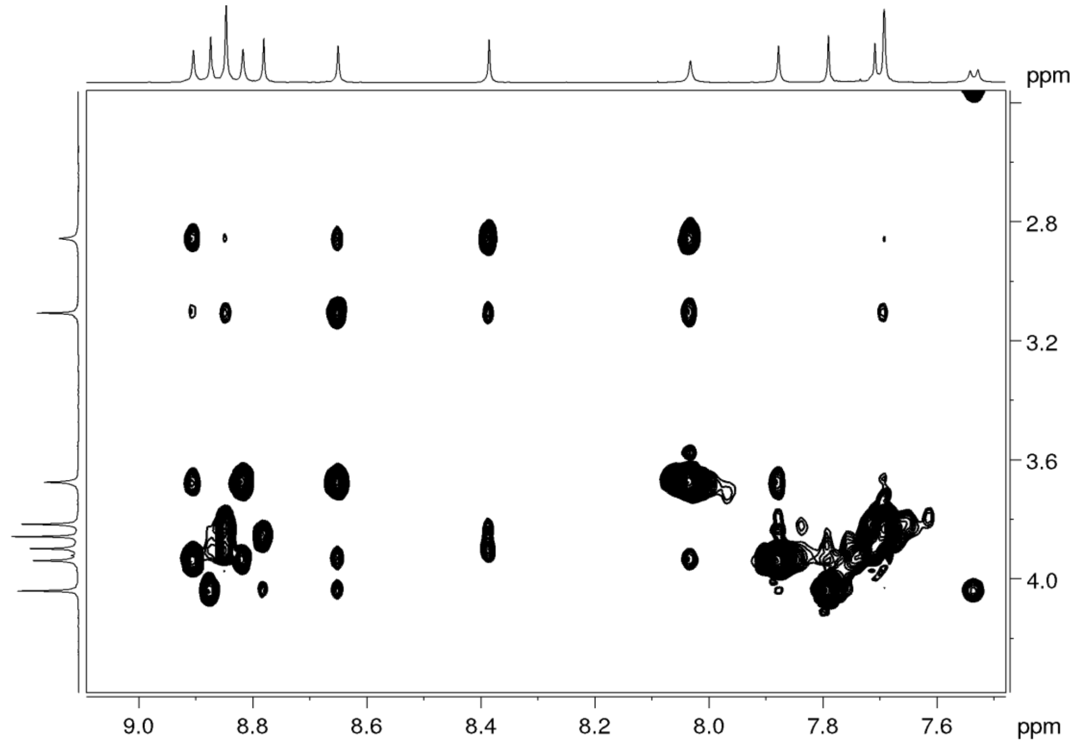

Figure 66. Partial ^{1}H-^{1}H NOESY NMR (600 MHz, CD_2Cl_2) spectrum of **(OMe-cube)$_2$**.

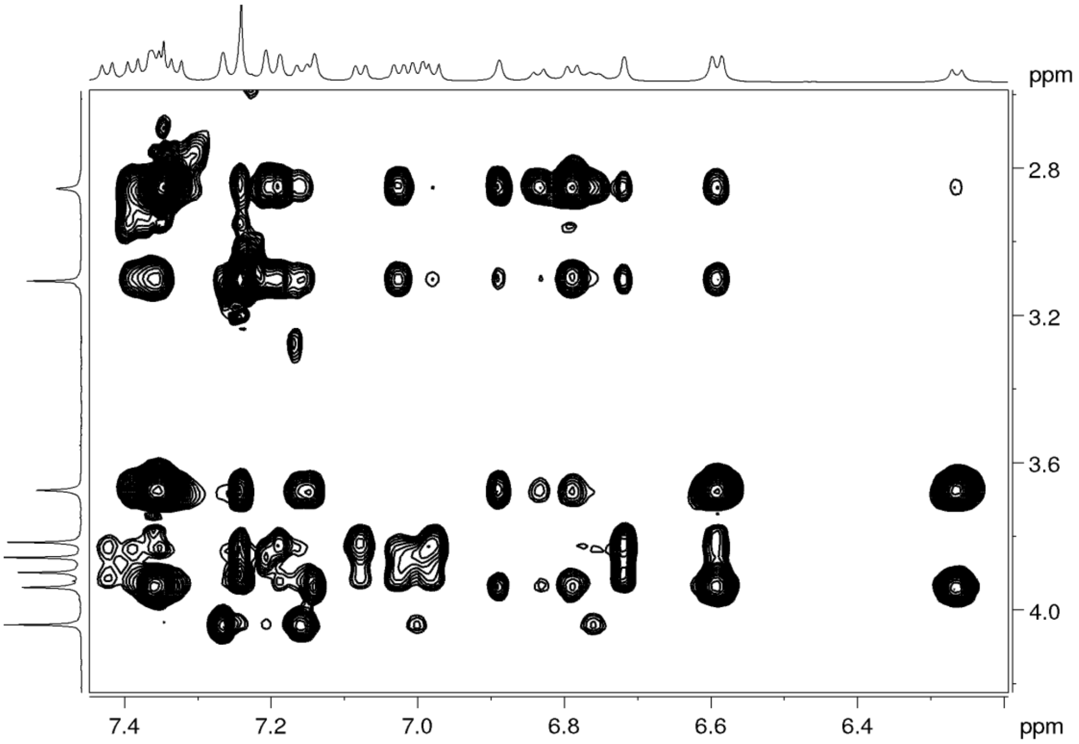

Figure 67. Partial ^{1}H-^{1}H NOESY NMR (600 MHz, CD_2Cl_2) spectrum of **(OMe-cube)$_2$**.

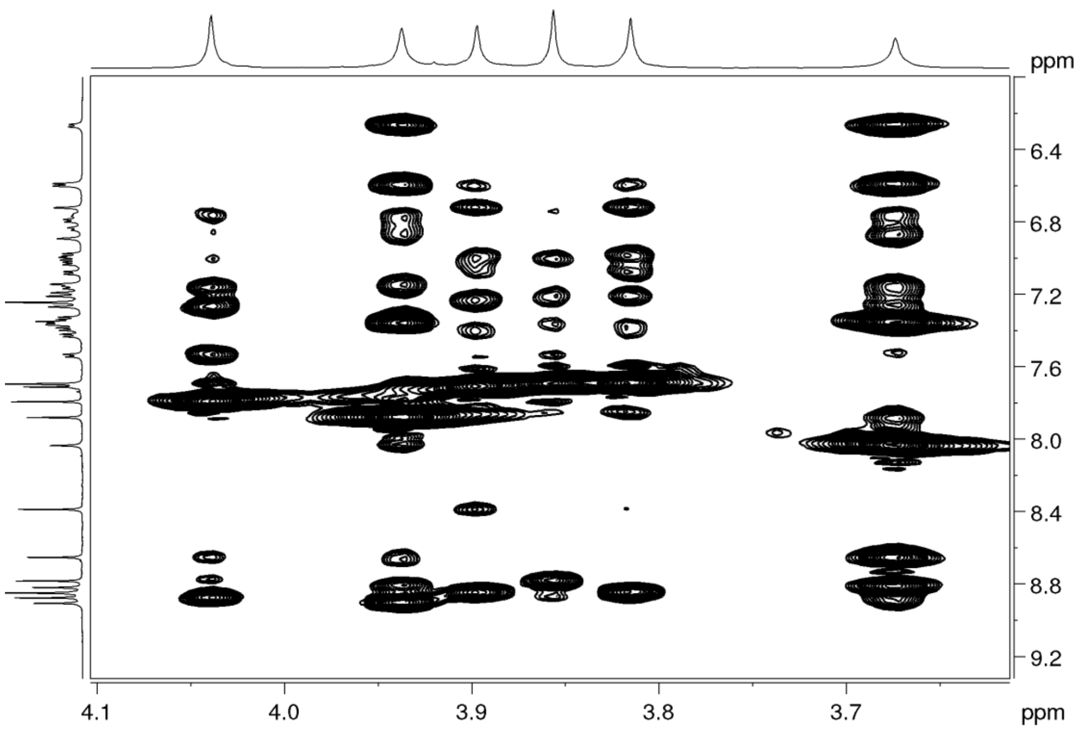

Figure 68. Partial ^{1}H-^{1}H NOESY NMR (600 MHz, CD_2Cl_2) spectrum of **(OMe-cube)$_2$**.

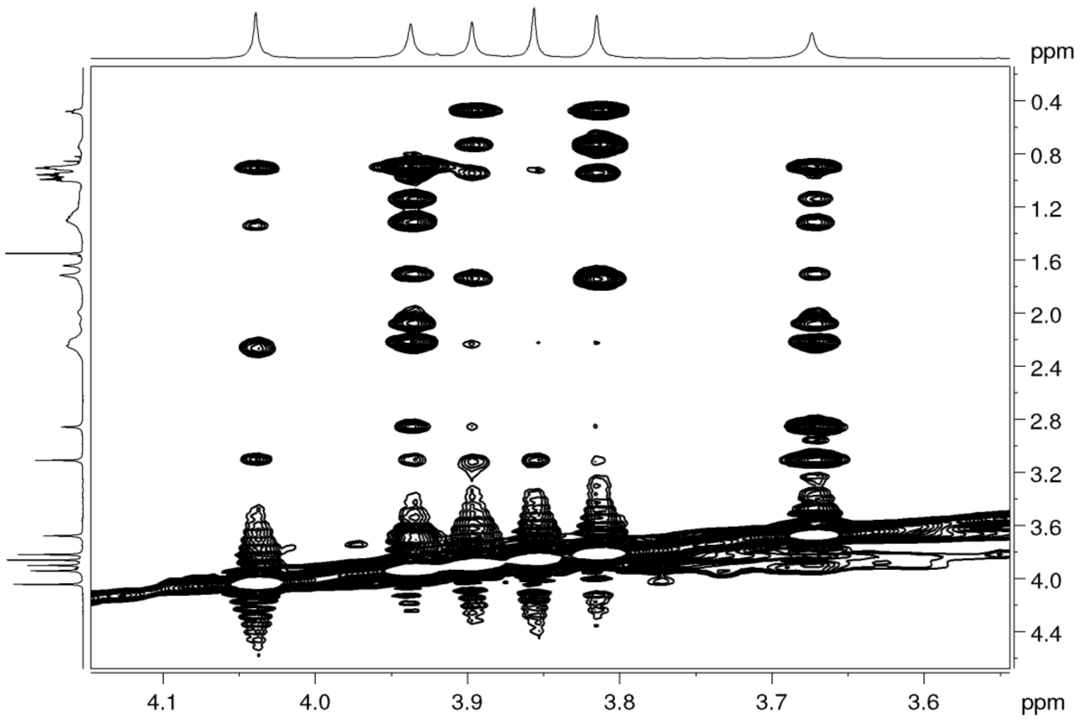

Figure 69. Partial ^{1}H-^{1}H NOESY NMR (600 MHz, CD_2Cl_2) spectrum of **(OMe-cube)$_2$**.

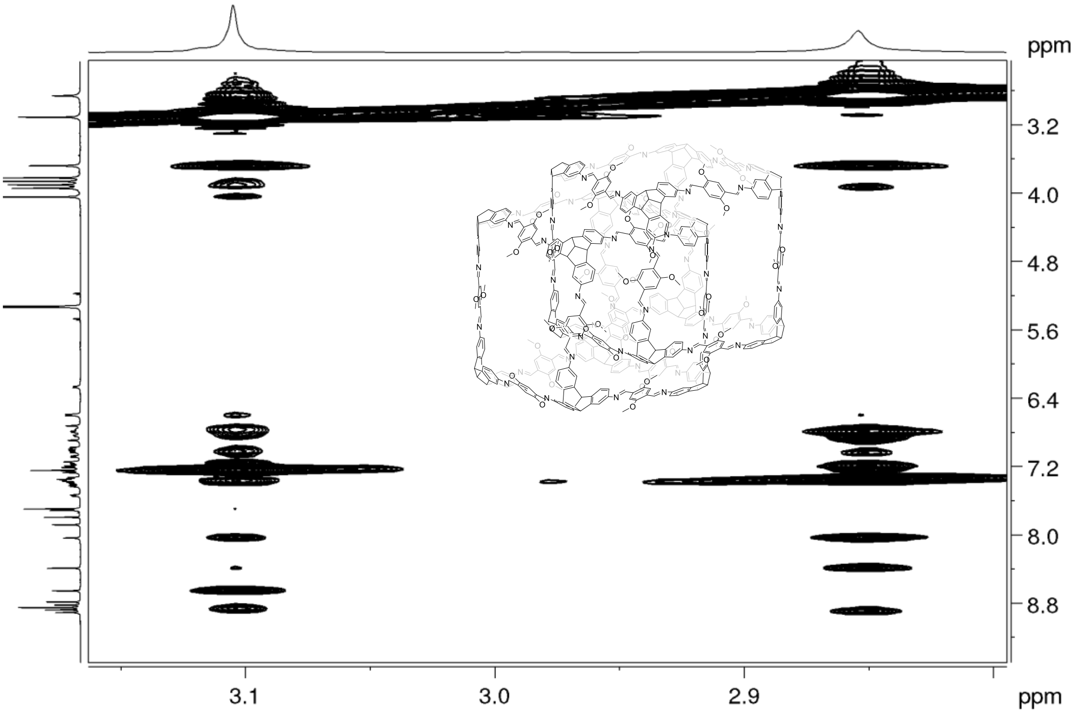

Figure 70. Partial ^1H-^1H NOESY NMR (600 MHz, CD_2Cl_2) spectrum of **(OMe-cube)$_2$**.

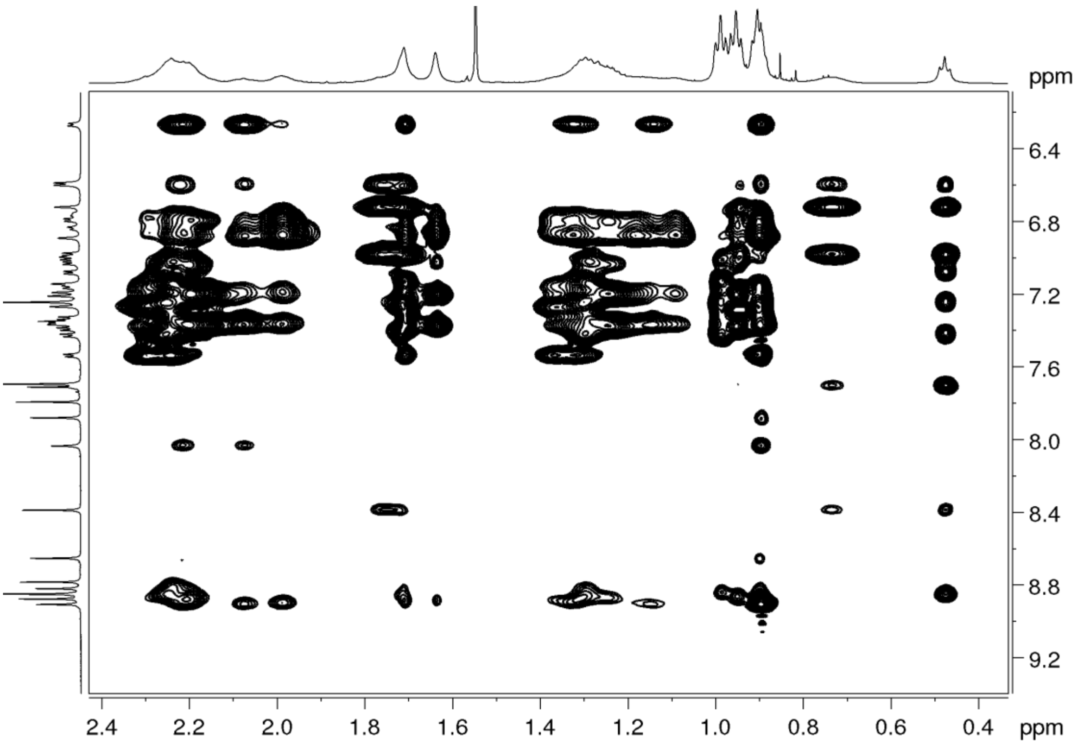

Figure 71. Partial ^1H-^1H NOESY NMR (600 MHz, CD_2Cl_2) spectrum of **(OMe-cube)$_2$**.

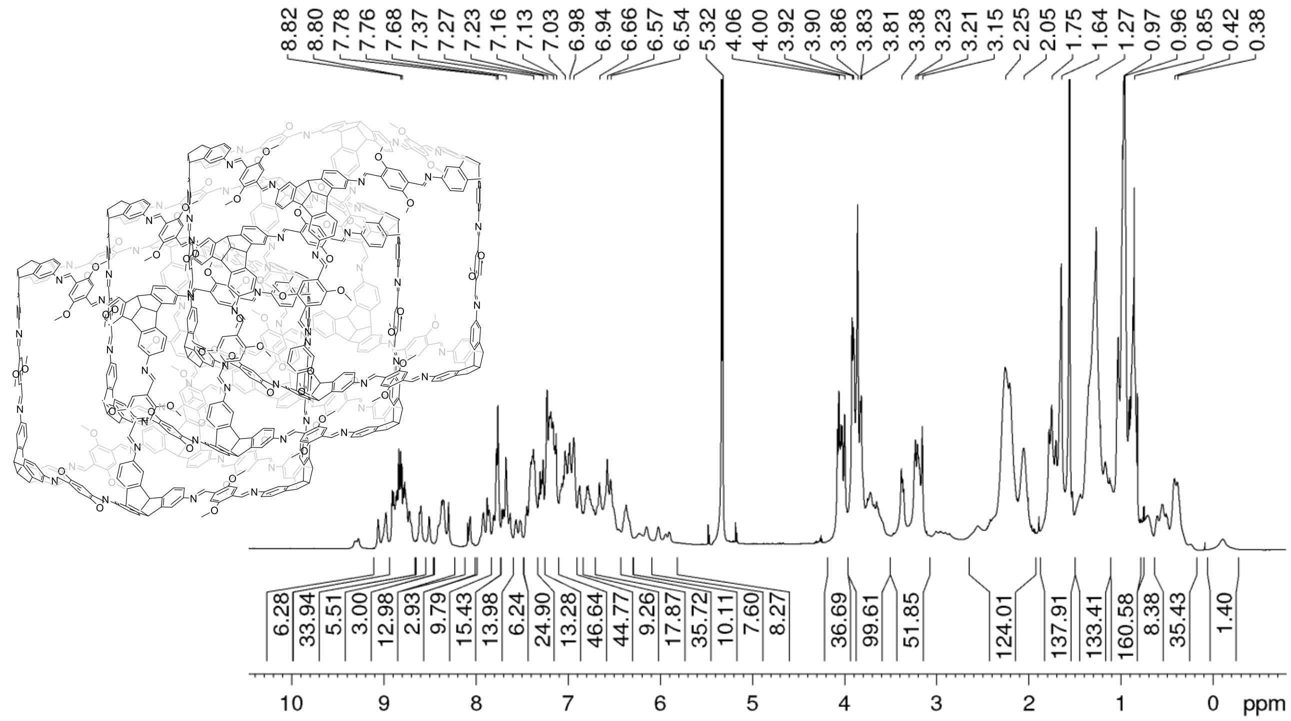

Figure 72. ^{1}H NMR (600 MHz, CD_2Cl_2) spectrum of **(OMe-cube)$_3$**.

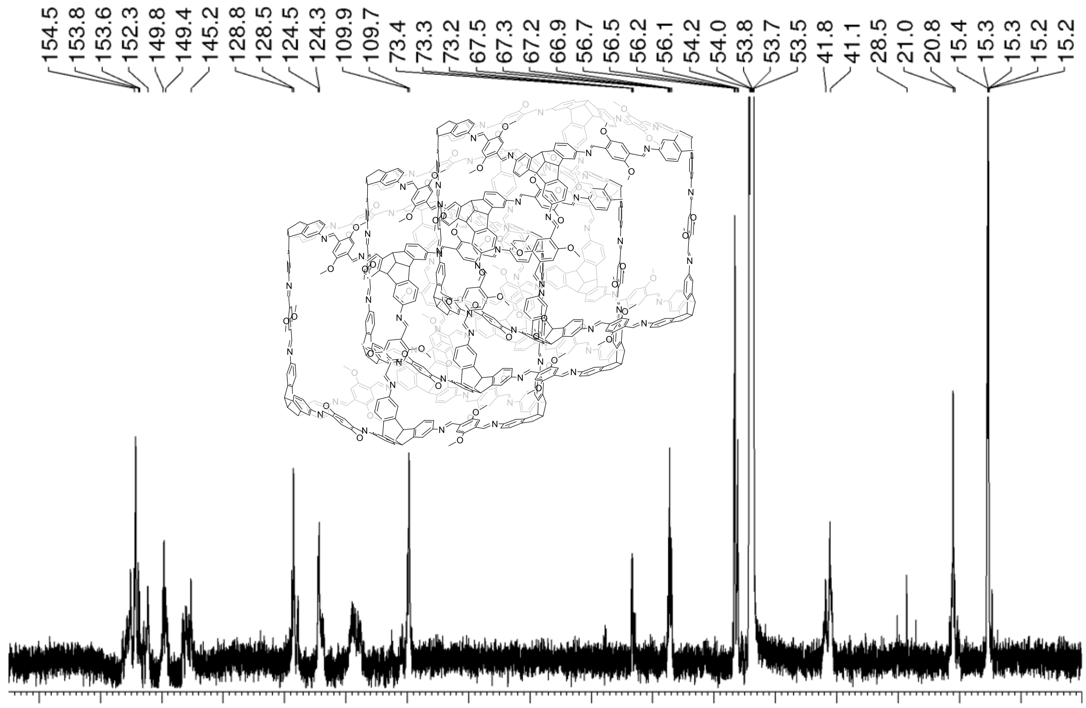

Figure 73. ^{13}C NMR (151 MHz, CD_2Cl_2) spectrum of **(OMe-cube)$_3$**.

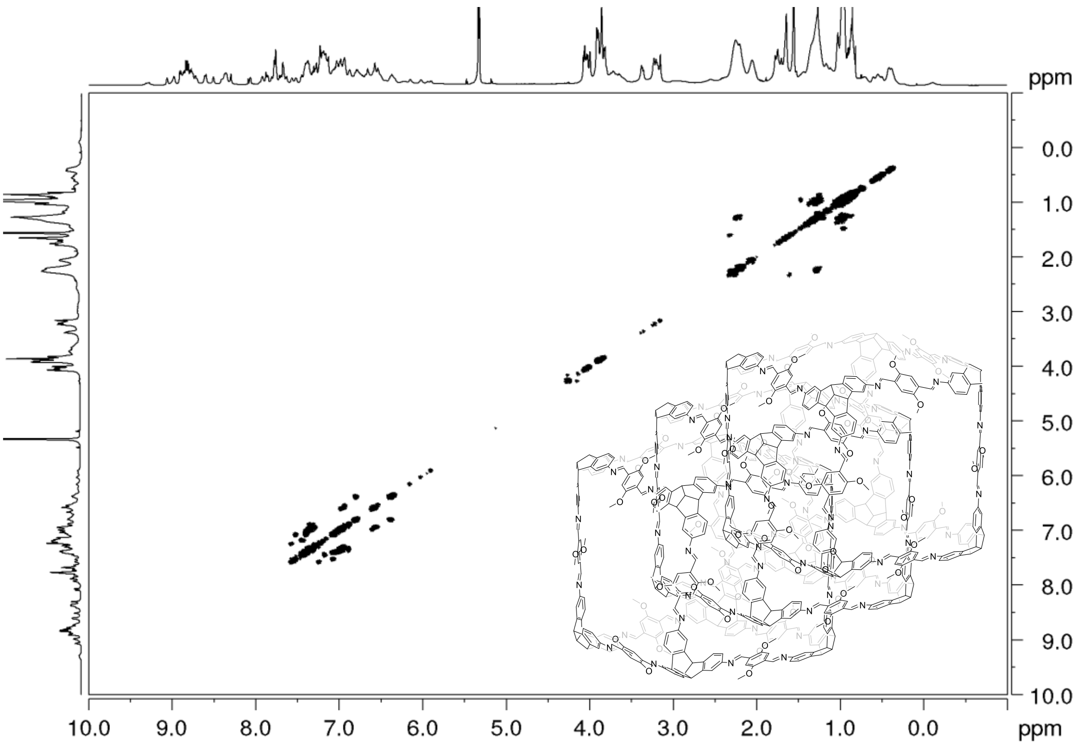

Figure 74. ^1H-^1H COSY NMR spectrum (600 MHz, CD_2Cl_2) of **(OMe-cube)$_3$**.

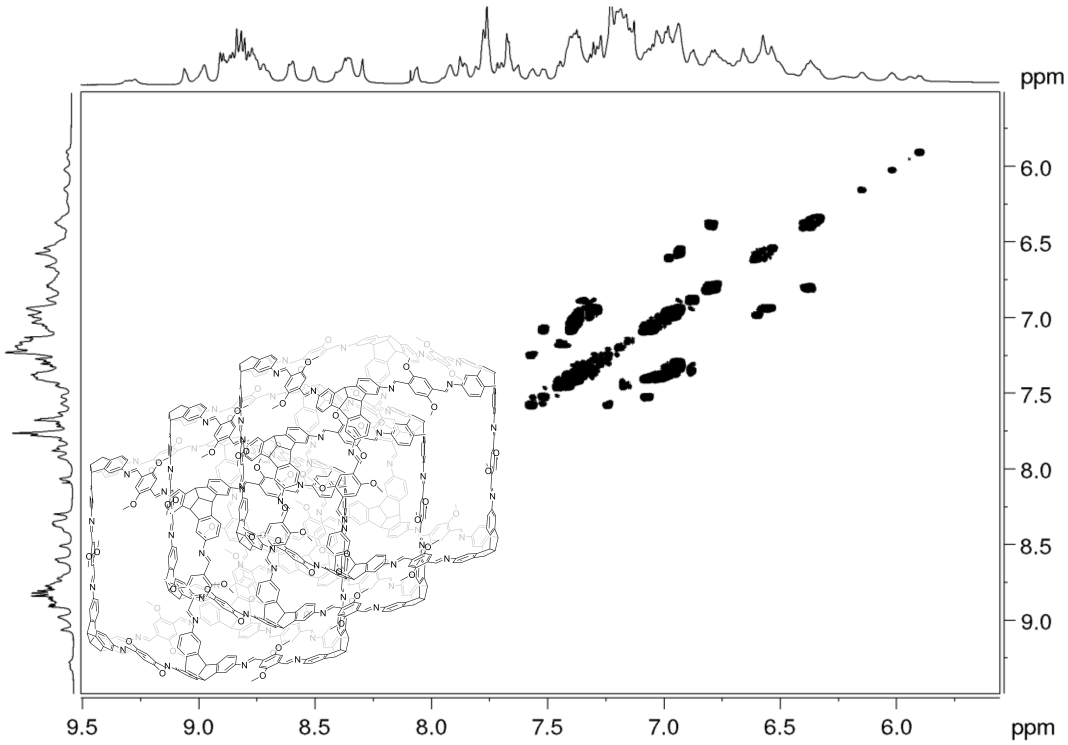

Figure 75. Partial ^1H-^1H COSY NMR spectrum (600 MHz, CD_2Cl_2) of **(OMe-cube)$_3$**.

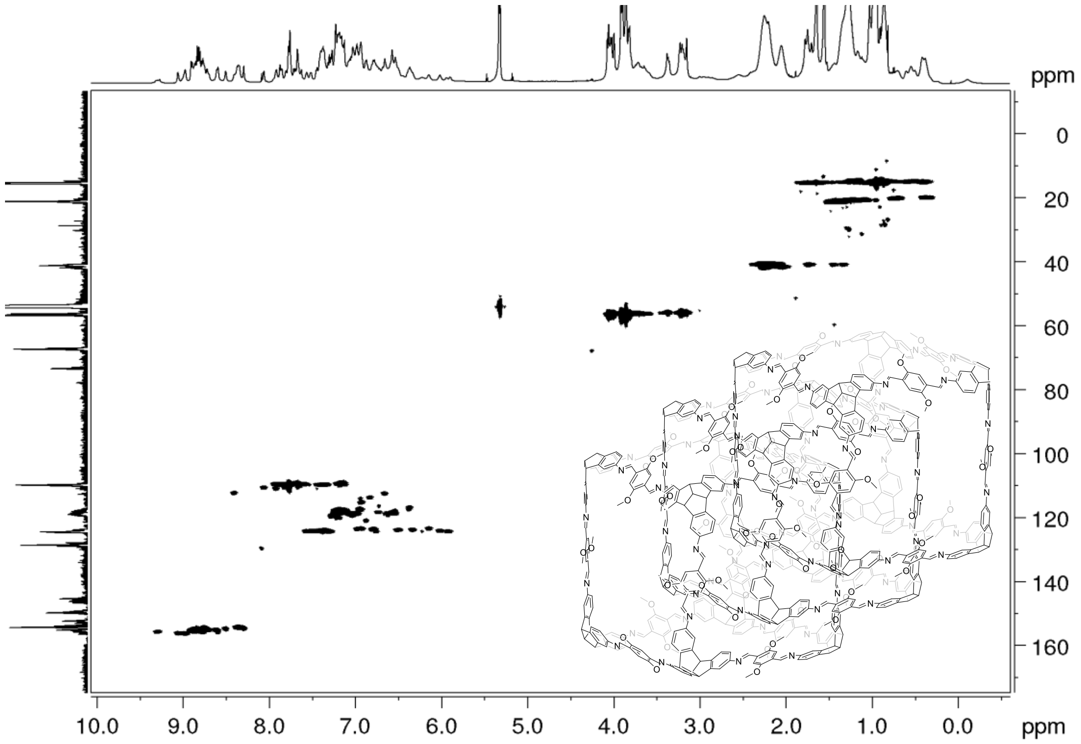

Figure 76. ^1H-^{13}C HSQC NMR (600 MHz and 151 MHz, CD_2Cl_2) spectrum of **(OMe-cube)₃**.

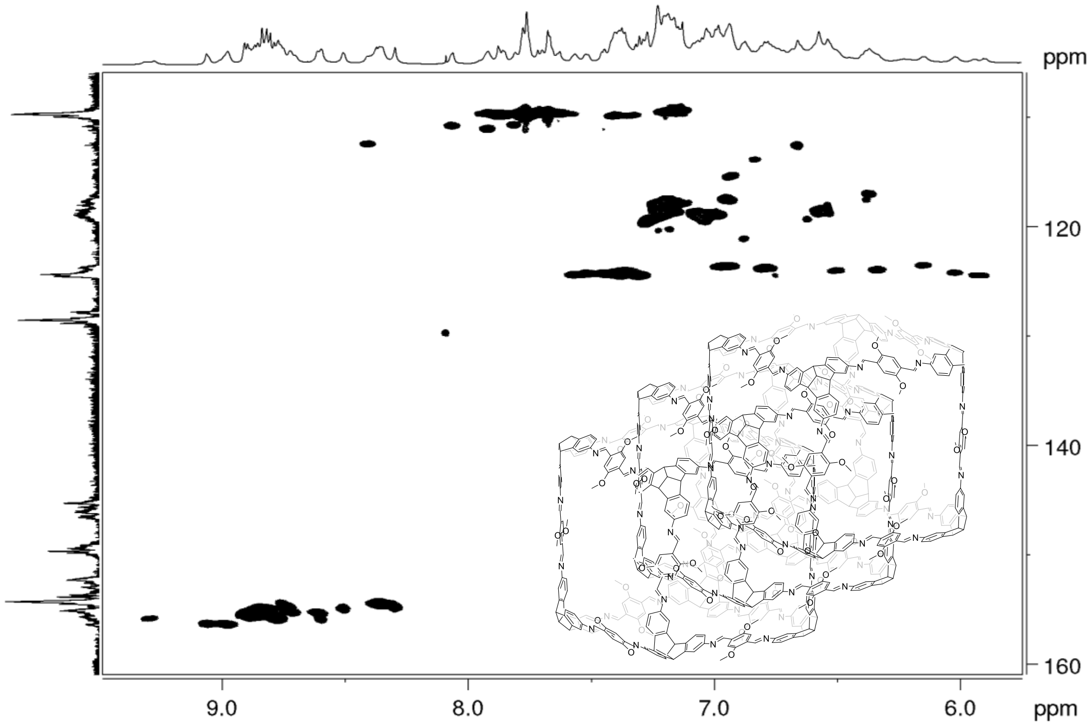

Figure 77. Partial ^1H-^{13}C HSQC NMR (600 MHz and 151 MHz, CD_2Cl_2) spectrum of **(OMe-cube)$_3$**.

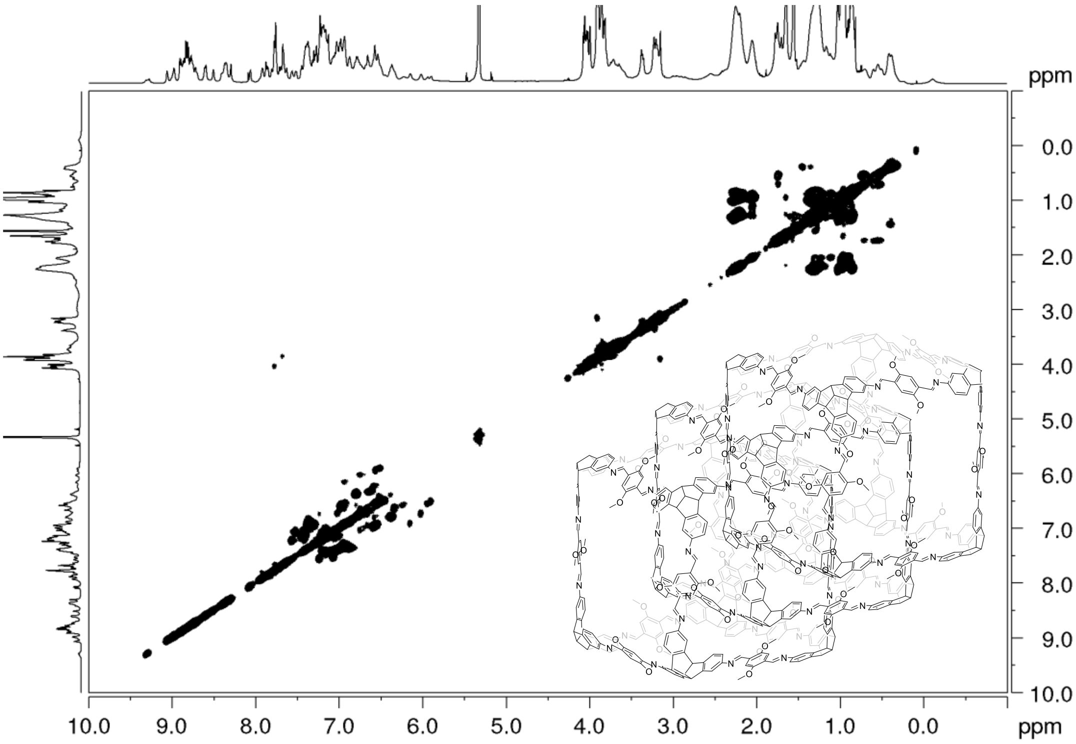

Figure 78. ^{1}H-^{1}H TOCSY NMR (600 MHz, CD_2Cl_2) spectrum of **(OMe-cube)$_3$**.

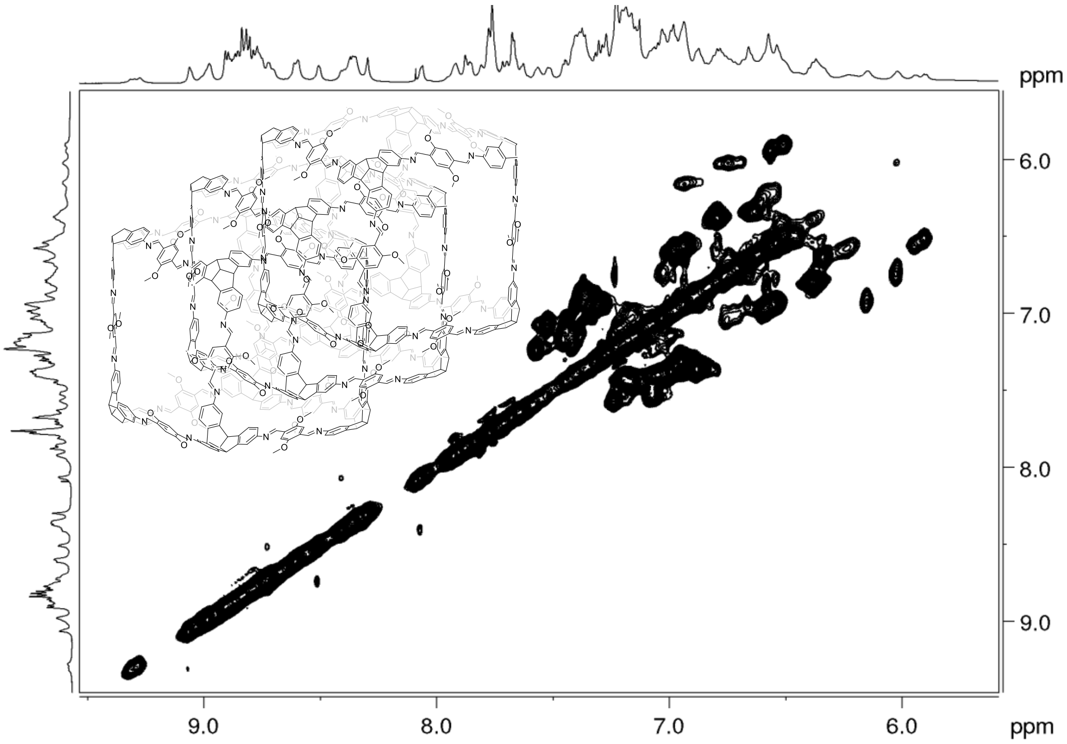

Figure 79. Partial ^{1}H-^{1}H TOCSY NMR (600 MHz, CD_2Cl_2) spectrum of **(OMe-cube)$_3$**.

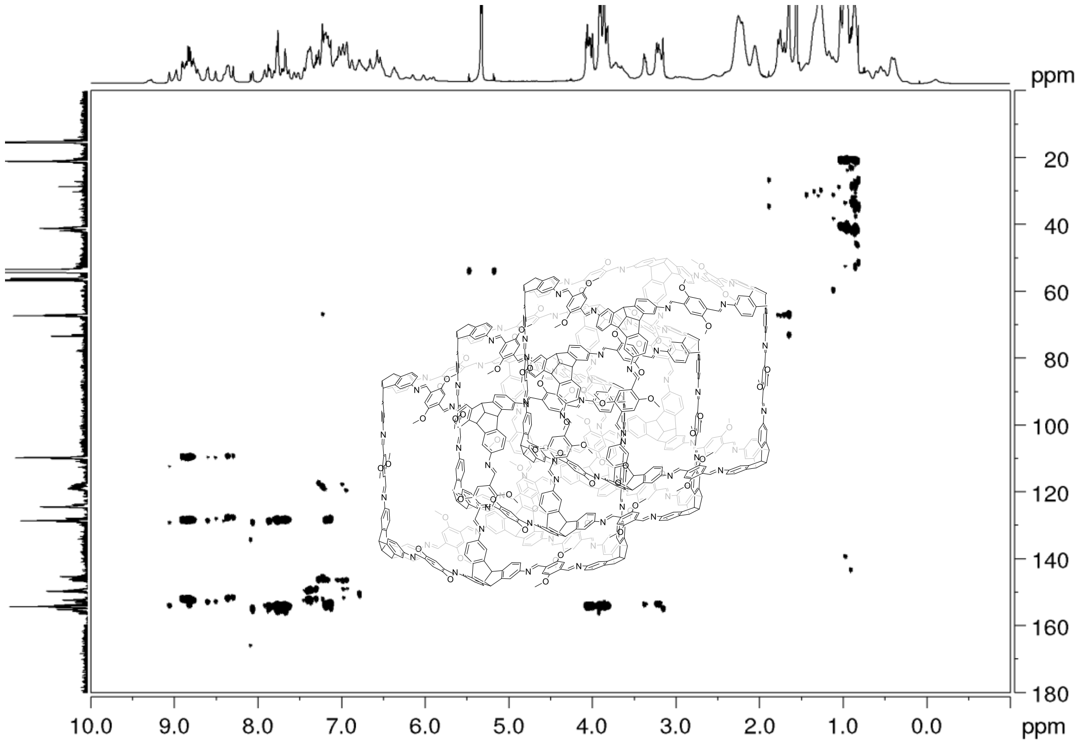

Figure 80. ^{1}H-^{13}C HMBC NMR (600 MHz and 151 MHz, CD_2Cl_2) spectrum of **(OMe-cube)$_3$**.

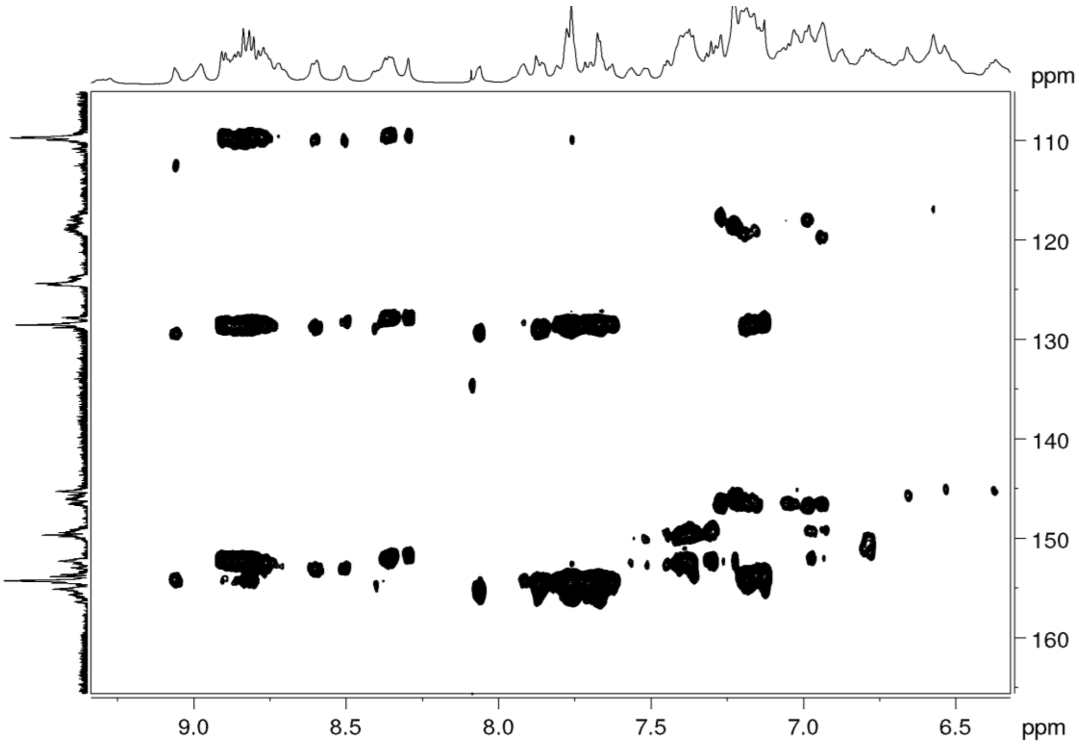

Figure 81. Partial ^{1}H-^{13}C HMBC NMR (600 MHz and 151 MHz, CD_2Cl_2) spectrum of **(OMe-cube)$_3$**.

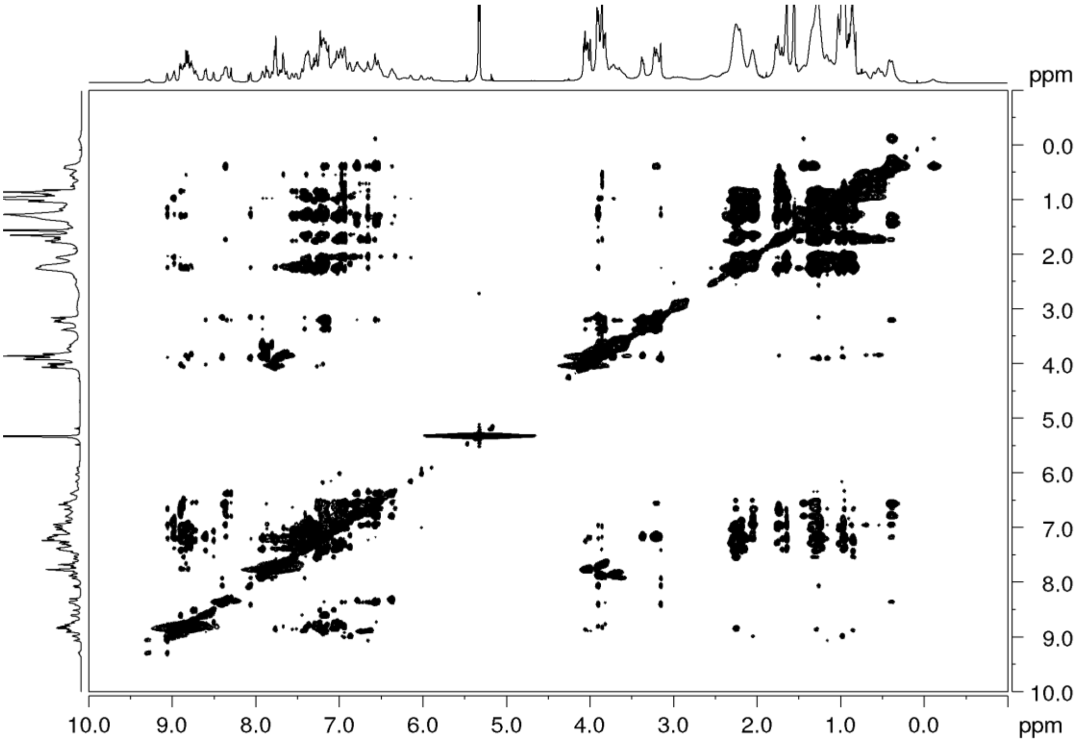

Figure 82. ^{1}H-^{1}H NOESY NMR (600 MHz, CD_2Cl_2) spectrum of **(OMe-cube)$_3$**.

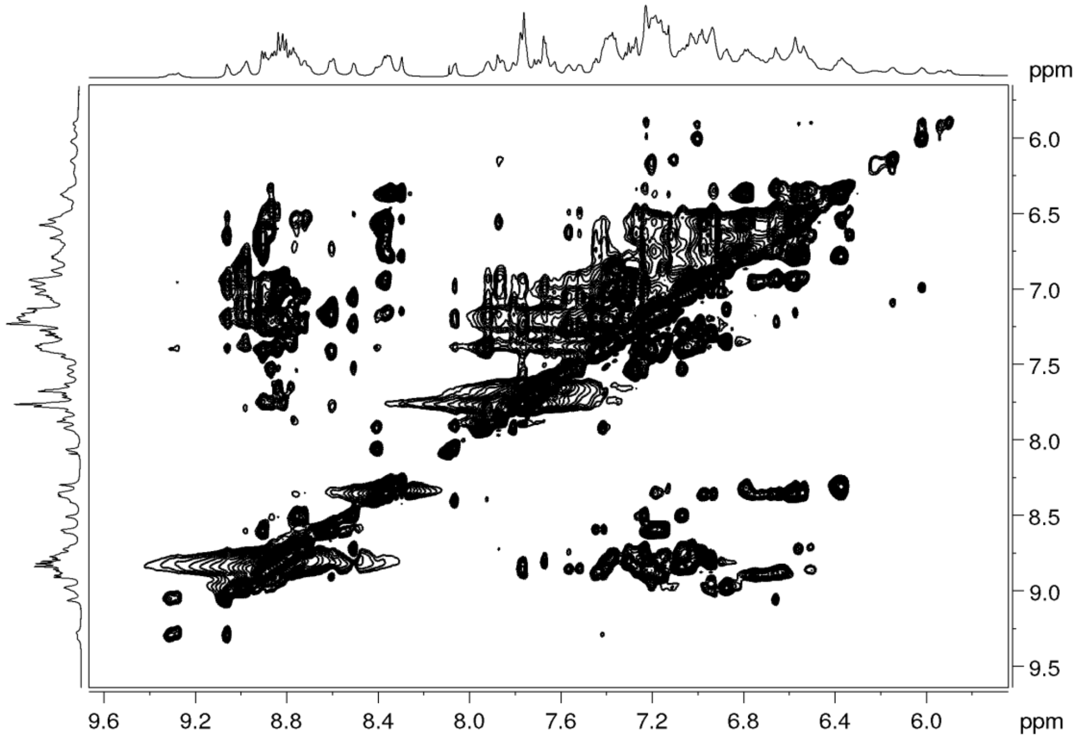

Figure 83. Partial ^{1}H-^{1}H NOESY NMR (600 MHz, CD_2Cl_2) spectrum of **(OMe-cube)$_3$**.

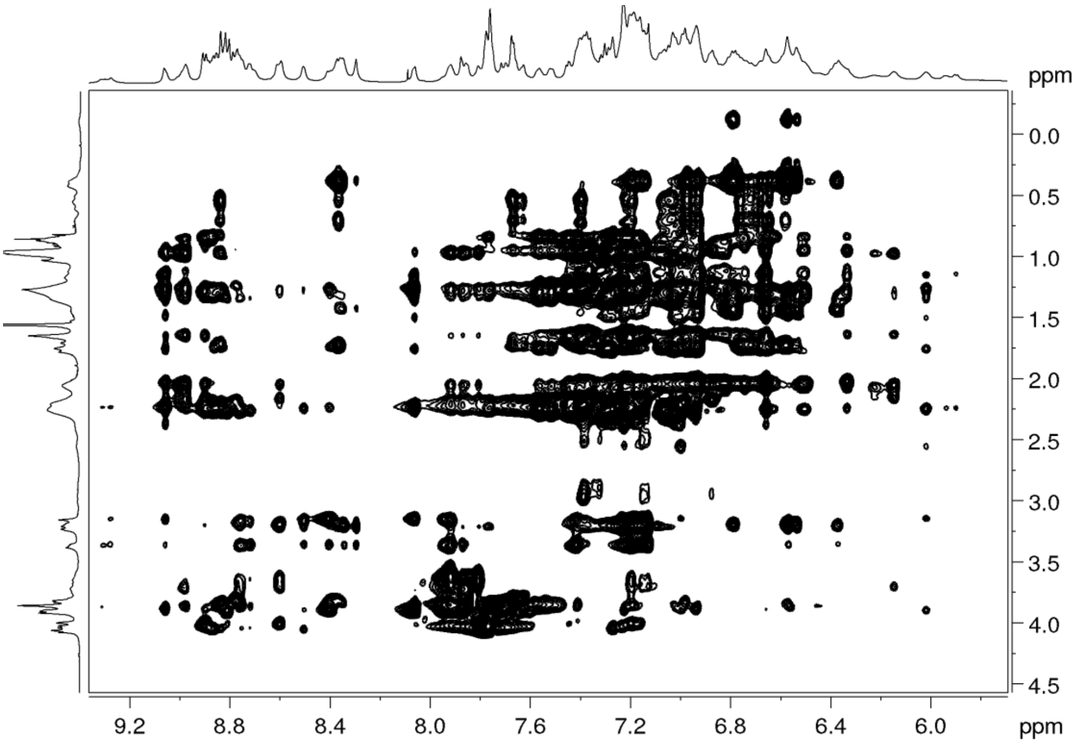

Figure 84. Partial ^{1}H-^{1}H NOESY NMR (600 MHz, CD_2Cl_2) spectrum of **(OMe-cube)$_3$**.

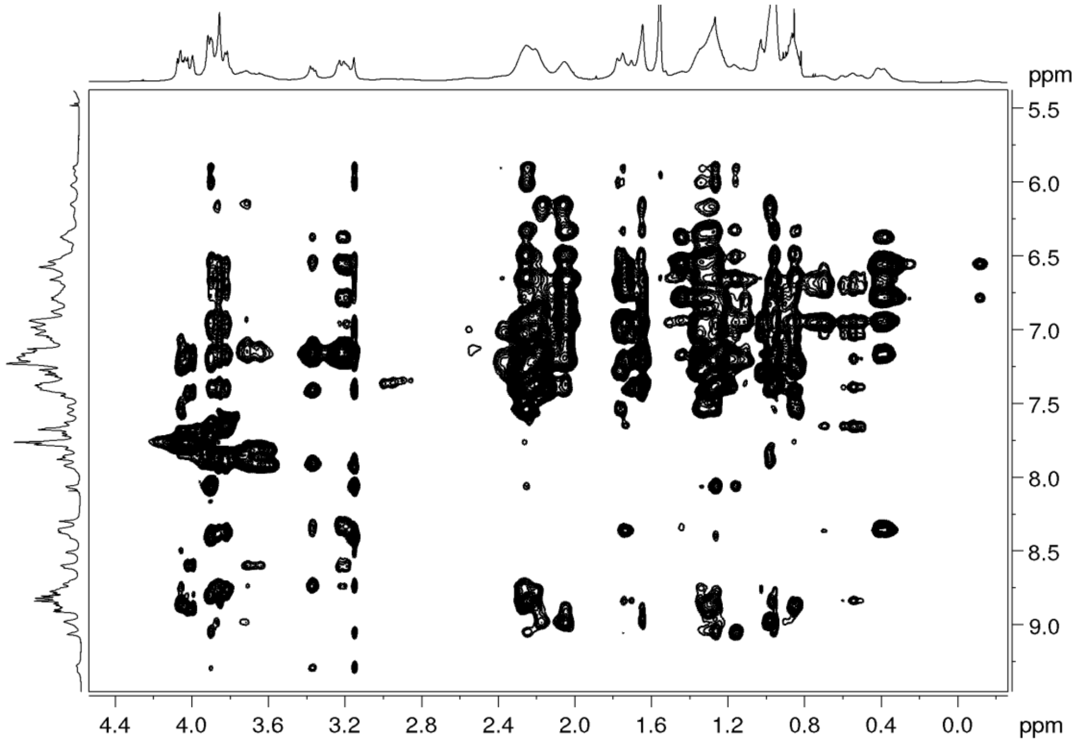

Figure 85. Partial ^{1}H-^{1}H NOESY NMR (600 MHz, CD_2Cl_2) spectrum of **(OMe-cube)$_3$**.

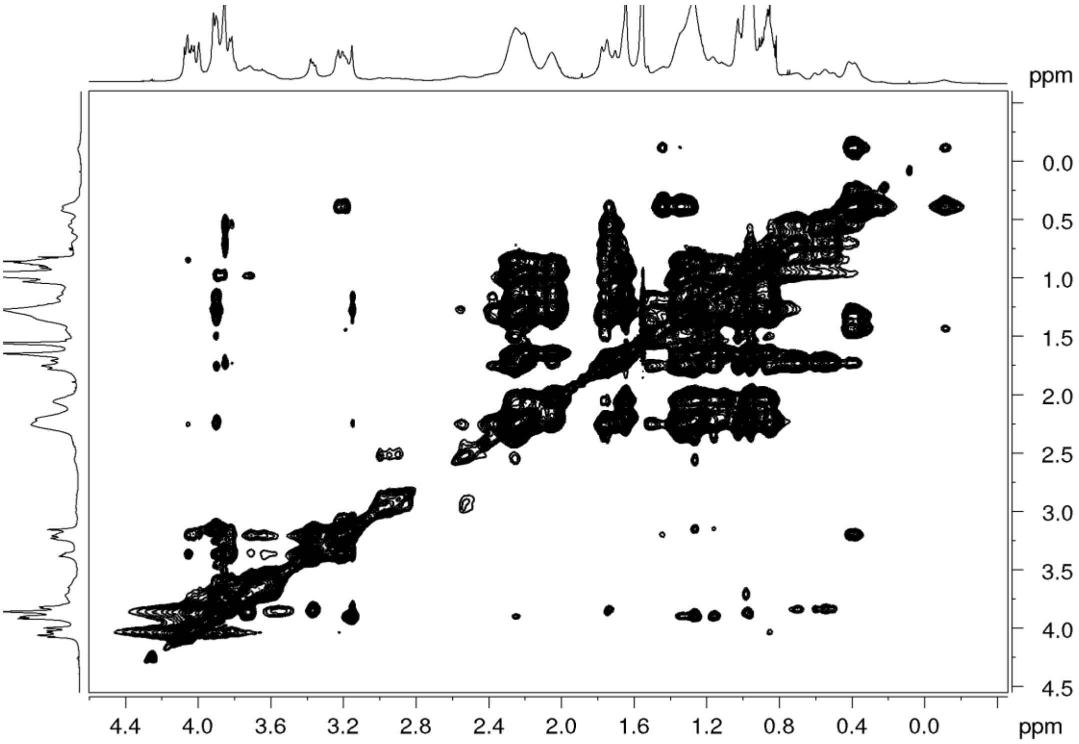

Figure 86. Partial ^{1}H-^{1}H NOESY NMR (600 MHz, CD_2Cl_2) spectrum of **(OMe-cube)$_3$**.

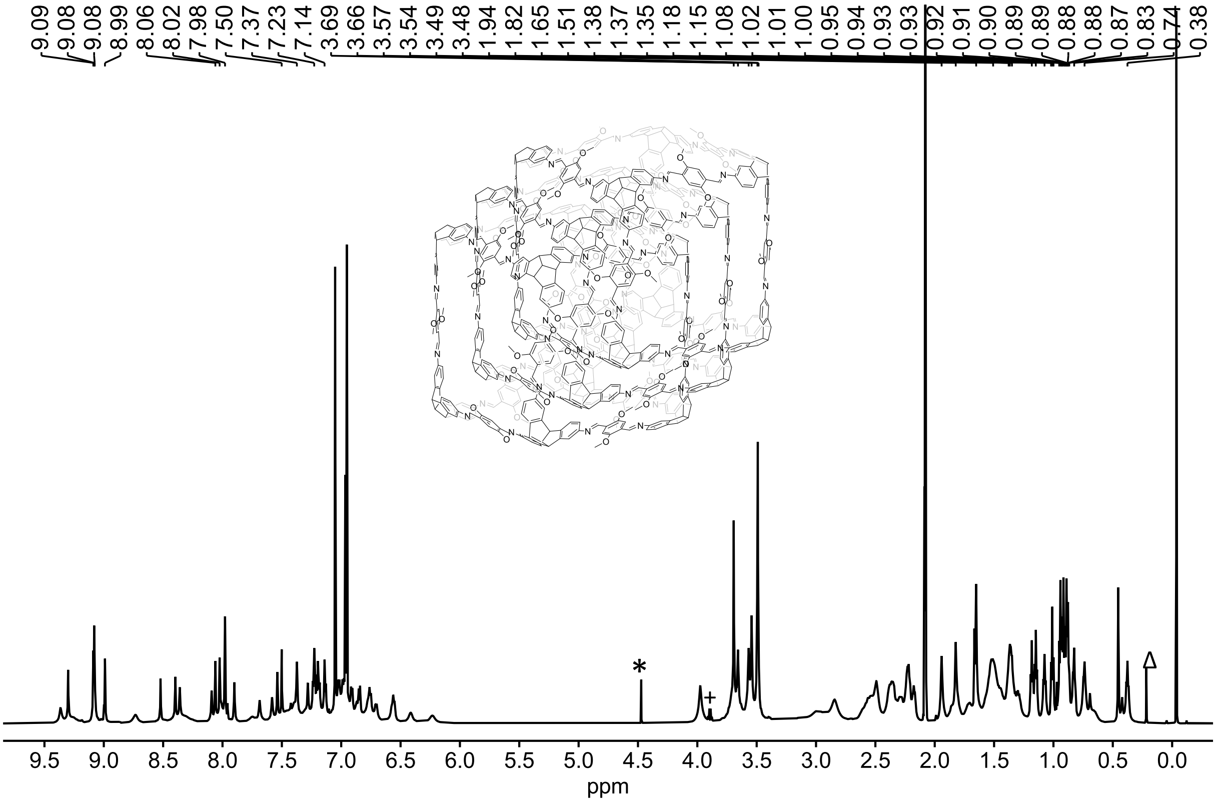

Figure 87. ^{1}H NMR (700 MHz, 375 K, Toluene-d_8) spectrum of **(OMe-cube)$_3$**.*unknown impurity, $^{+}$EA, $^{\Delta}$silicone grease

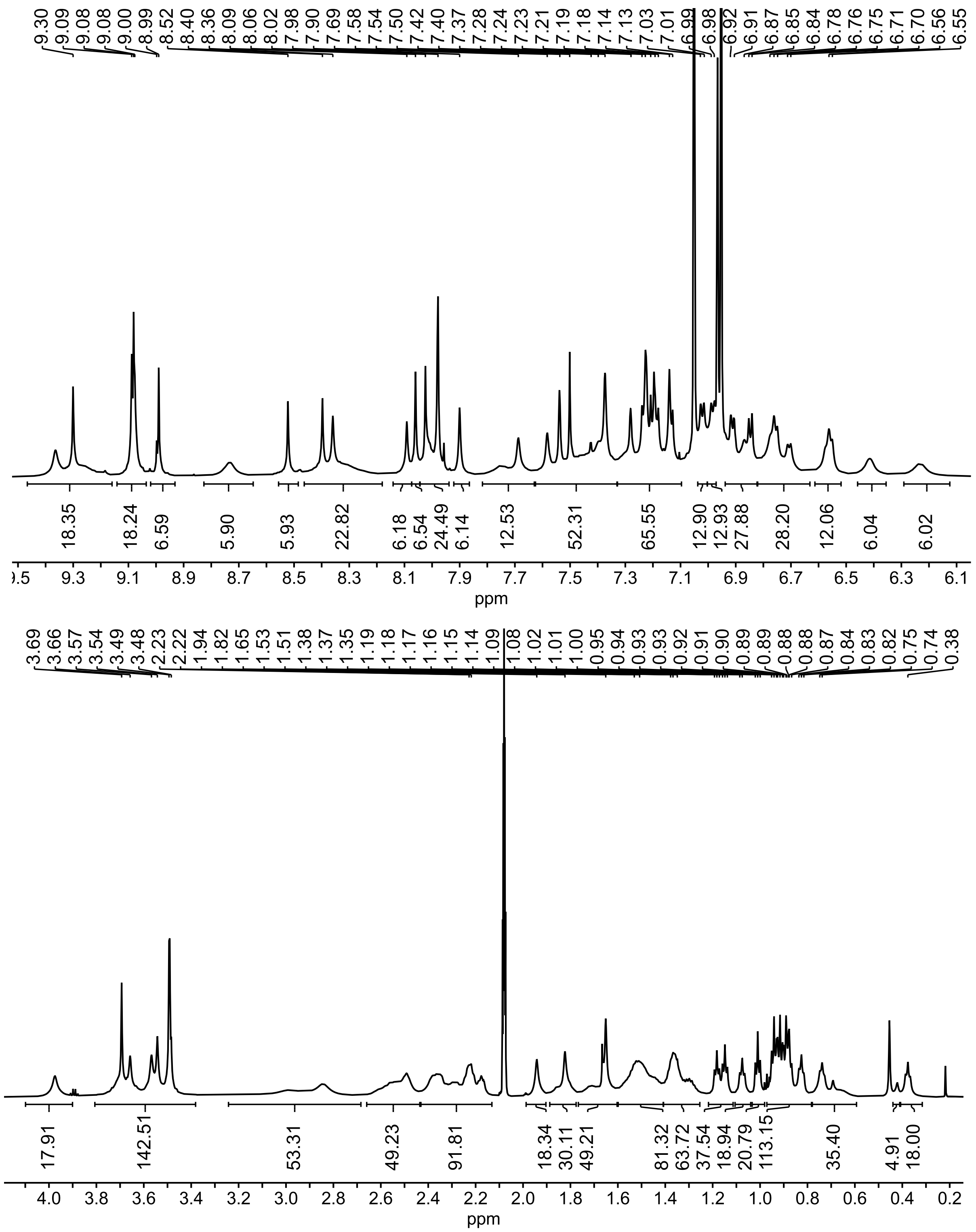

Figure 88. ^{1}H NMR (700 MHz, 375 K, Toluene-d_8) spectrum of **(OMe-cube)$_3$**.

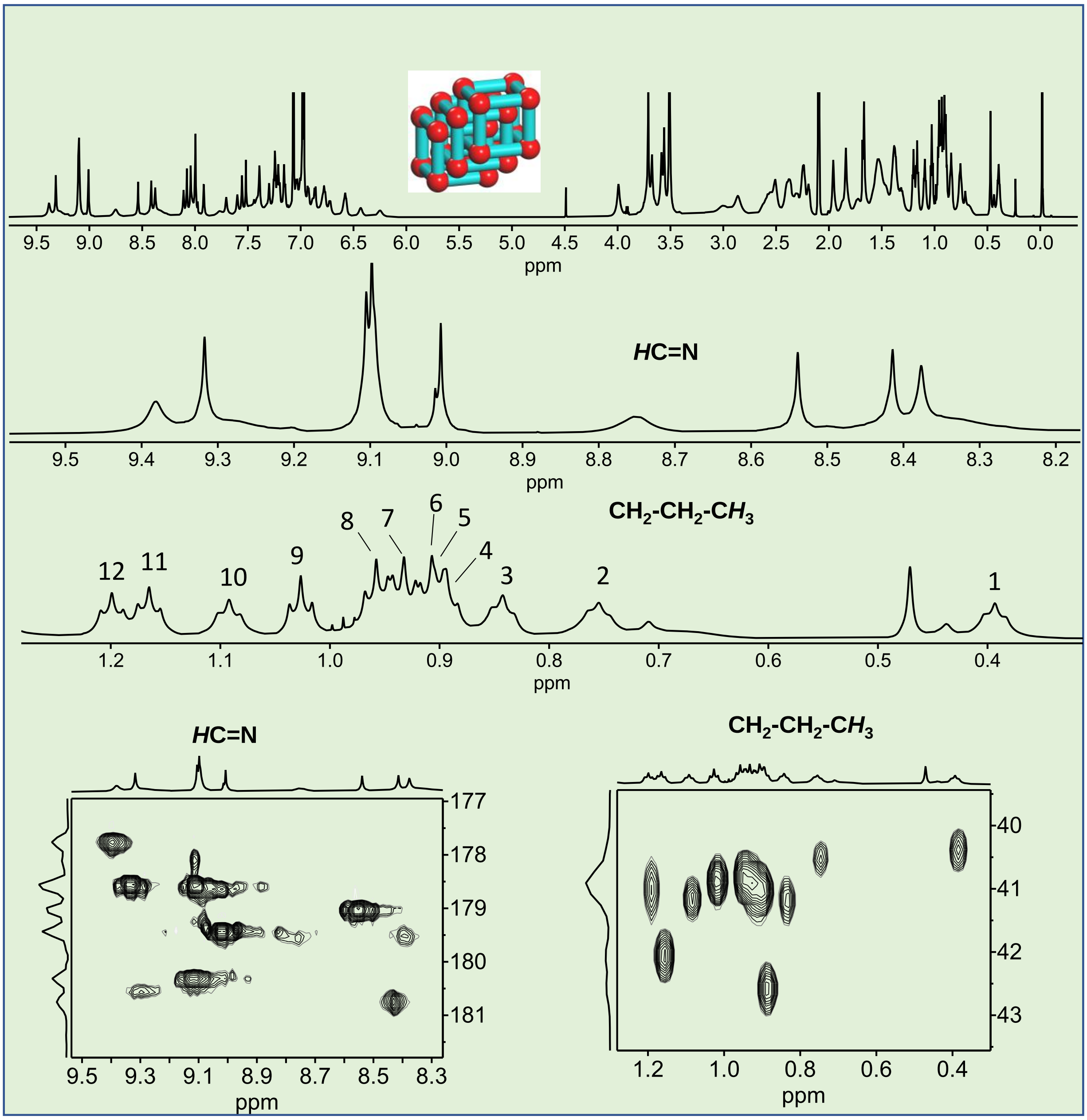

Figure 89. ^{1}H NMR (700 MHz, 375 K, Toluene-d$_8$) spectrum of **(OMe-cube)$_3$**.

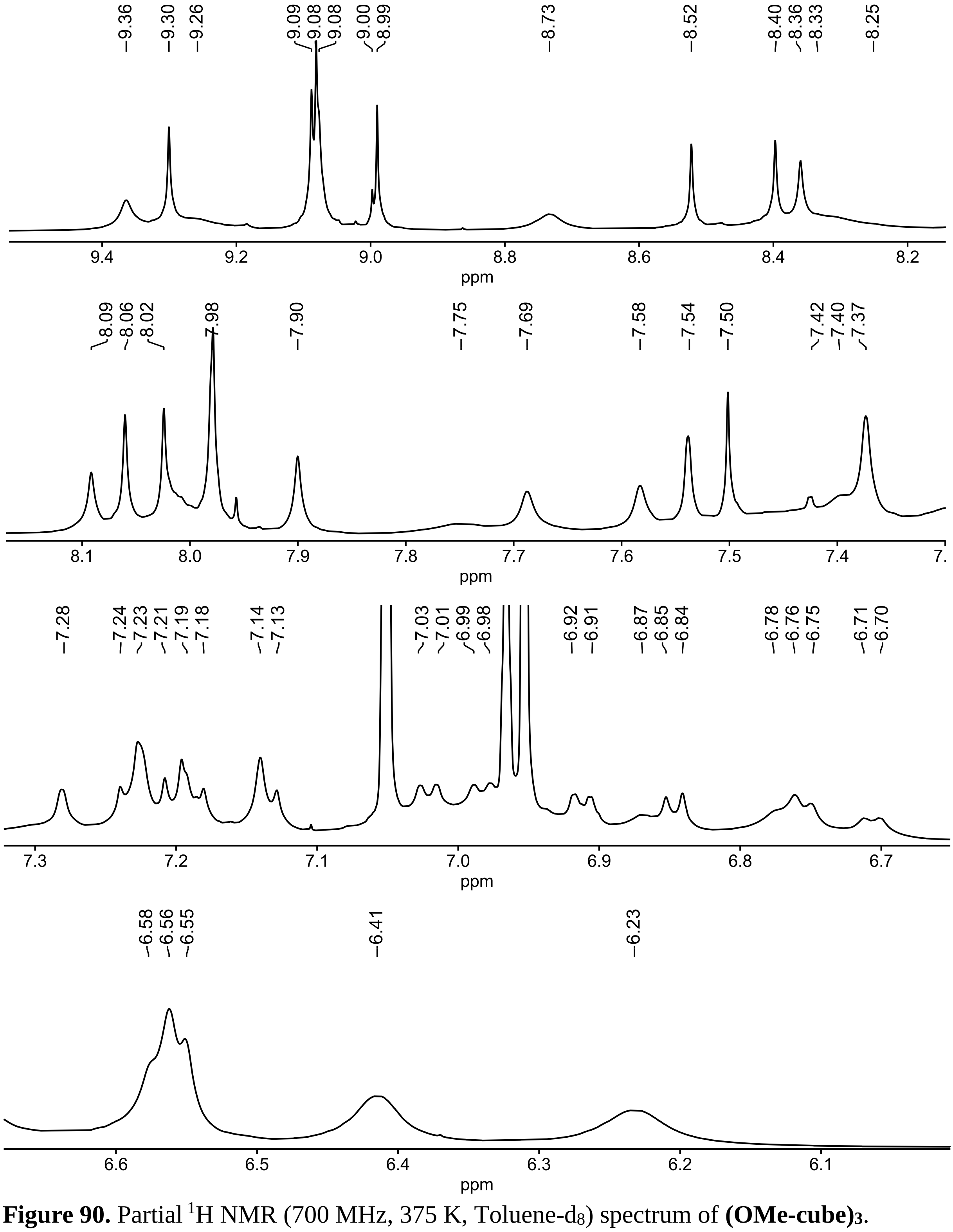

Figure 90. Partial ^{1}H NMR (700 MHz, 375 K, Toluene-d$_8$) spectrum of **(OMe-cube)$_3$**.

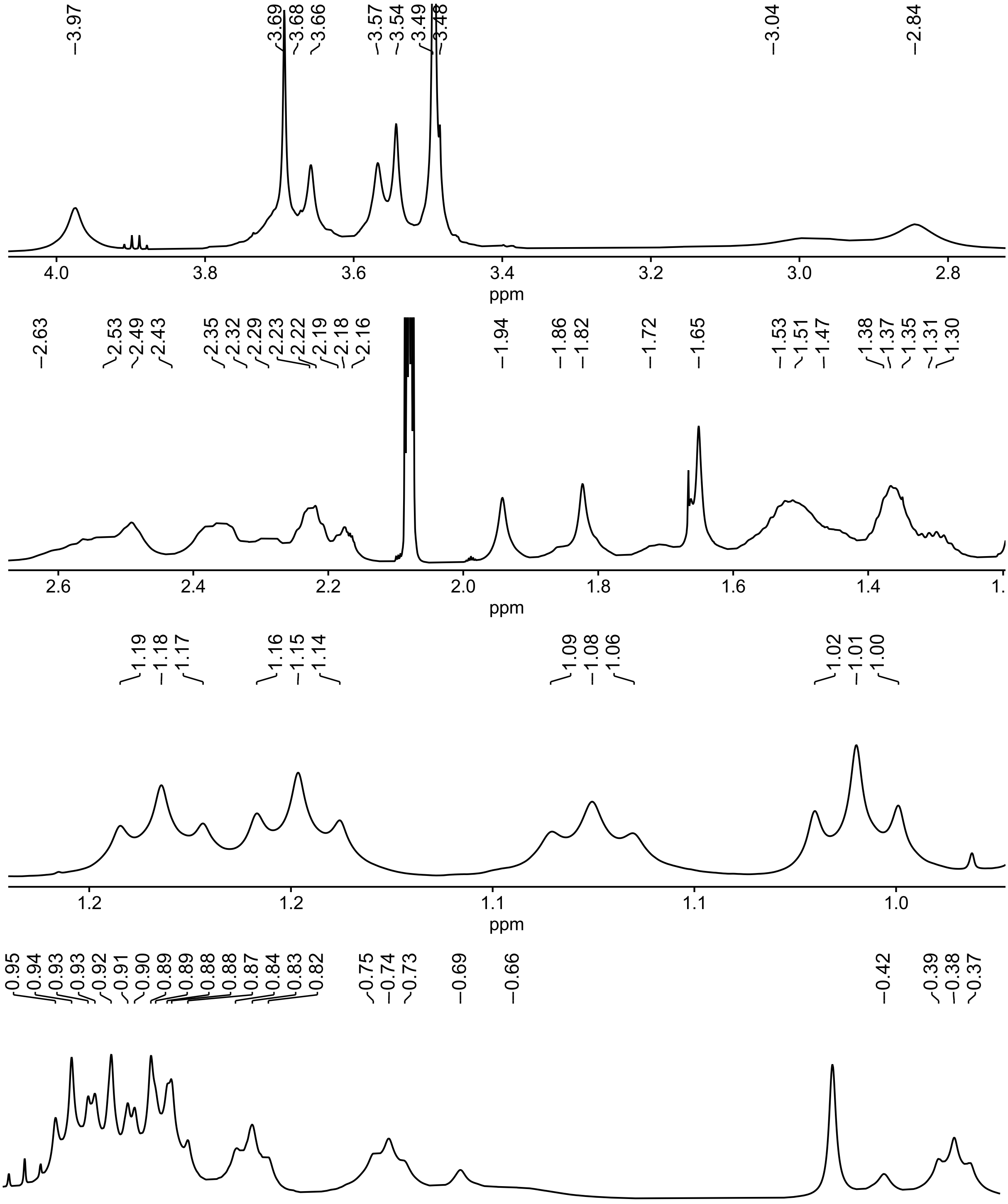

Figure 91. Partial ^{1}H NMR (700 MHz, 375 K, Toluene-d_8) spectrum of **(OMe-cube)$_3$**.

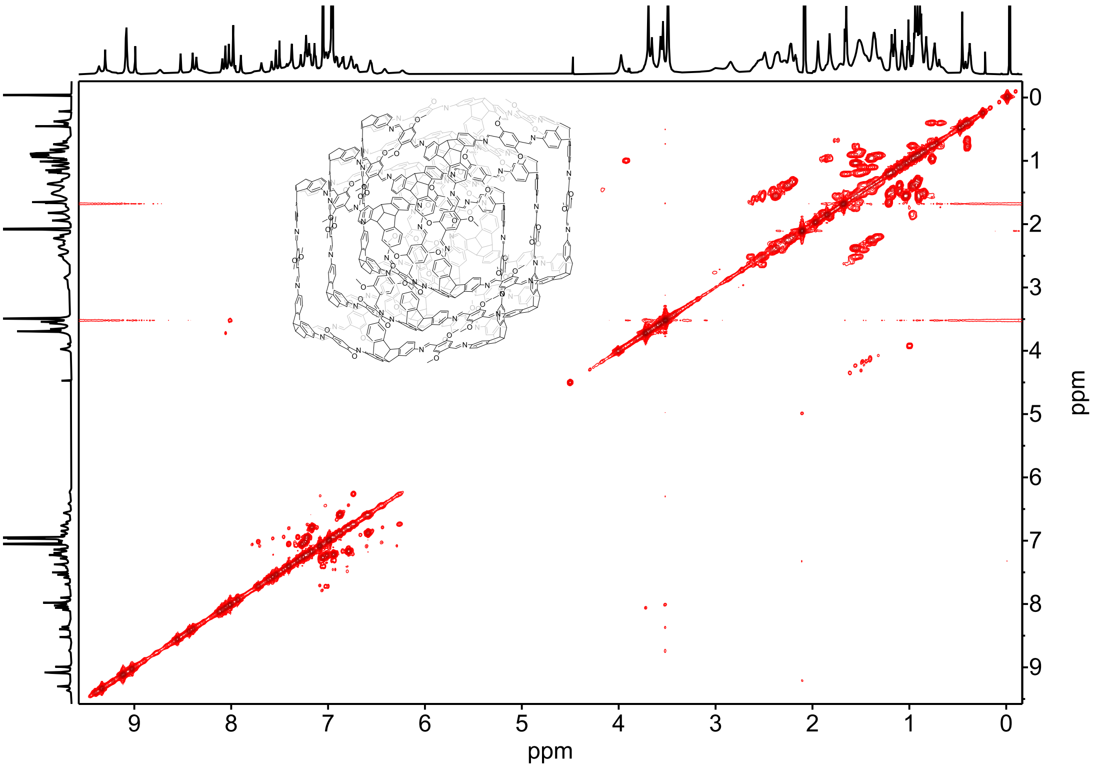

Figure 92. ^{1}H-^{1}H COSY NMR spectrum (700 MHz, 375 K, Toluene-d_8) of **(OMe-cube)$_3$**.

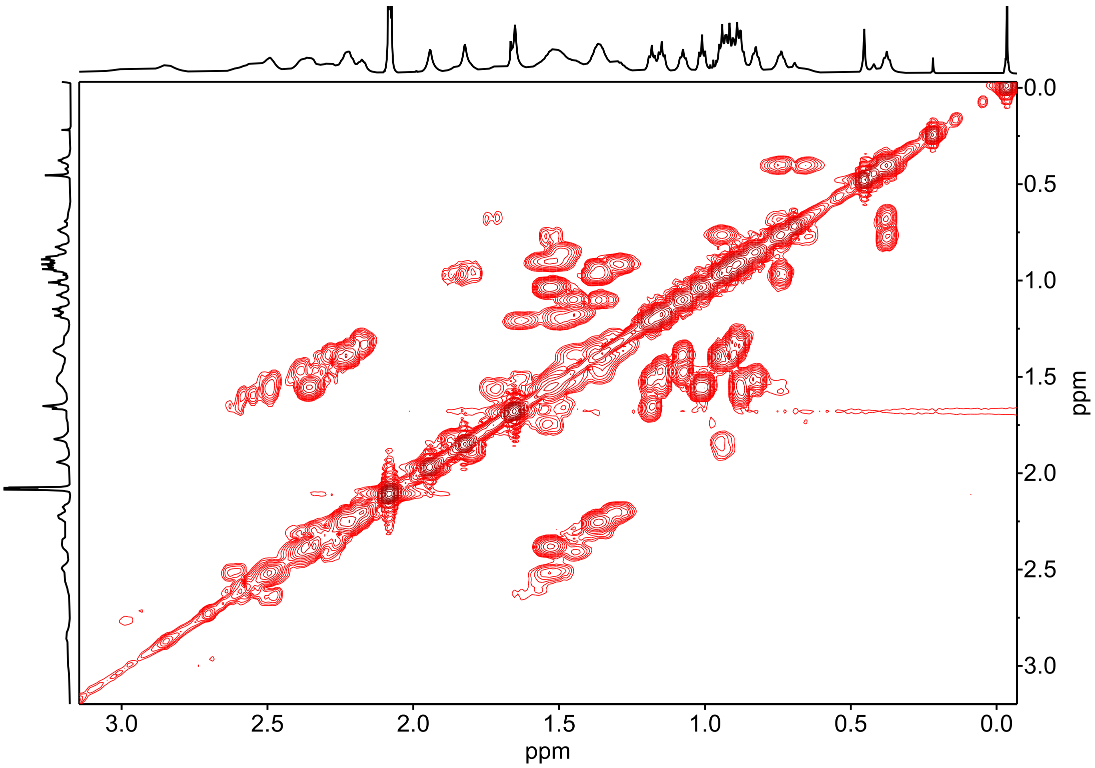

Figure 93. Partial ^{1}H-^{1}H COSY NMR spectrum (700 MHz, 375 K, Toluene-d_8) of **(OMe-cube)$_3$**.

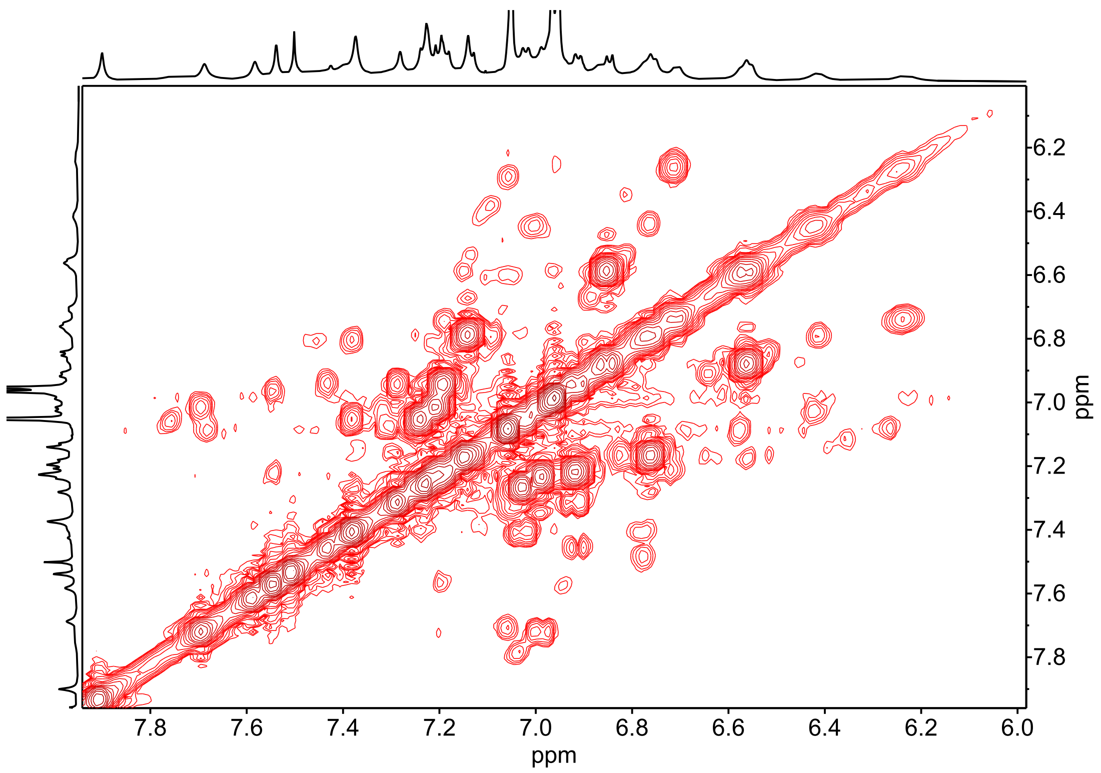

Figure 94. Partial ¹H-¹H COSY NMR spectrum (700 MHz, 375 K, Toluene-d_8) of **(OMe-cube)$_3$**.

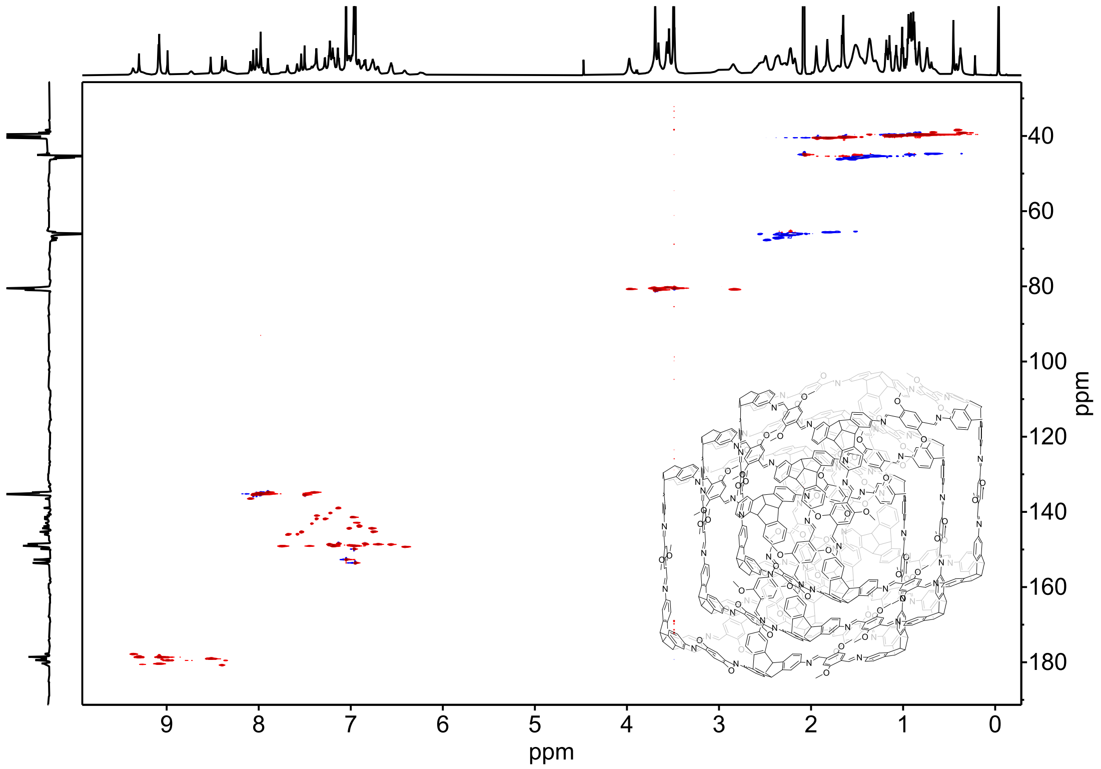

Figure 95. ¹H-¹³C HSQC NMR (700 MHz and 176 MHz, 375 K, Toluene-d_8) CD_2Cl_2) spectrum of **(OMe-cube)$_3$**.

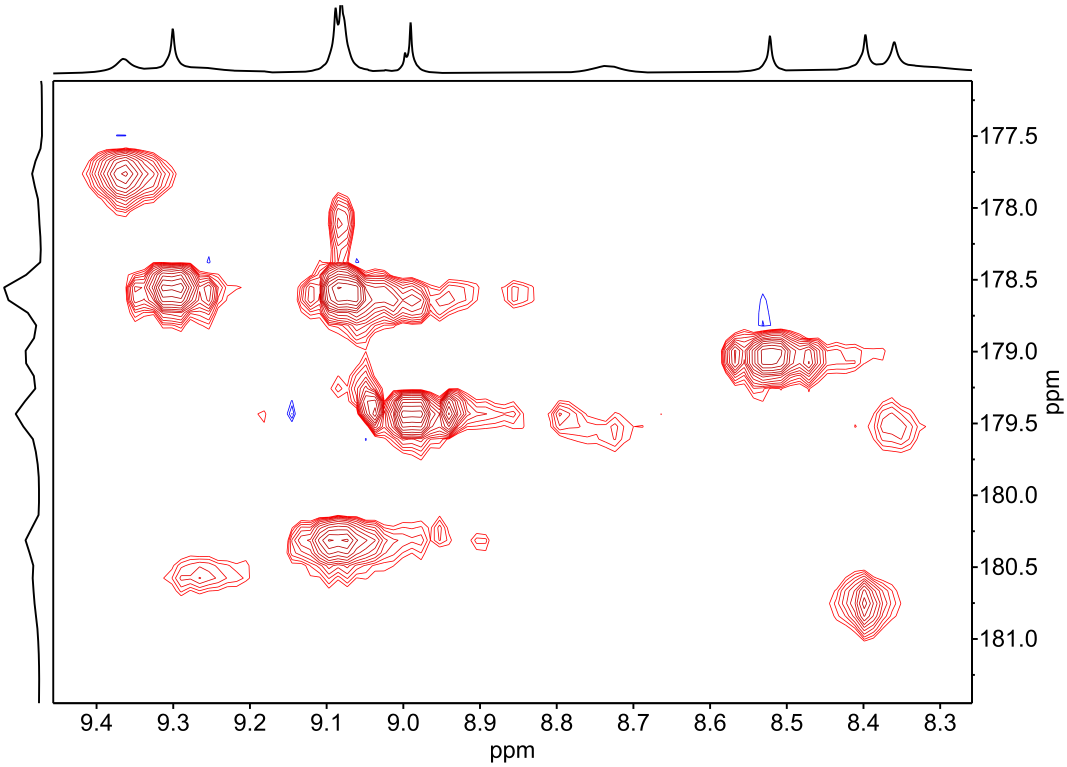

Figure 96. Partial ^{1}H-^{13}C HSQC NMR (700 MHz and 176 MHz, 375 K, Toluene-d_8) spectrum of **(OMe-cube)$_3$**.

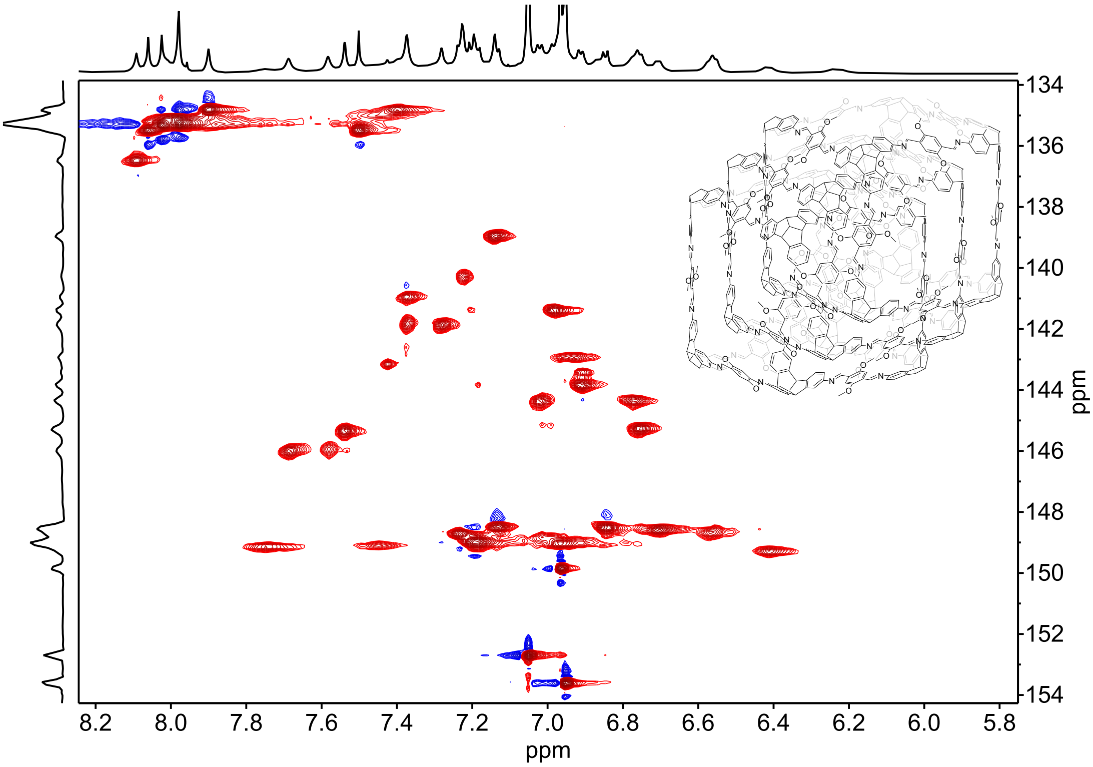

Figure 97. Partial ^{1}H-^{13}C HSQC NMR (700 MHz and 176 MHz, 375 K, Toluene-d_8) spectrum of **(OMe-cube)$_3$**.

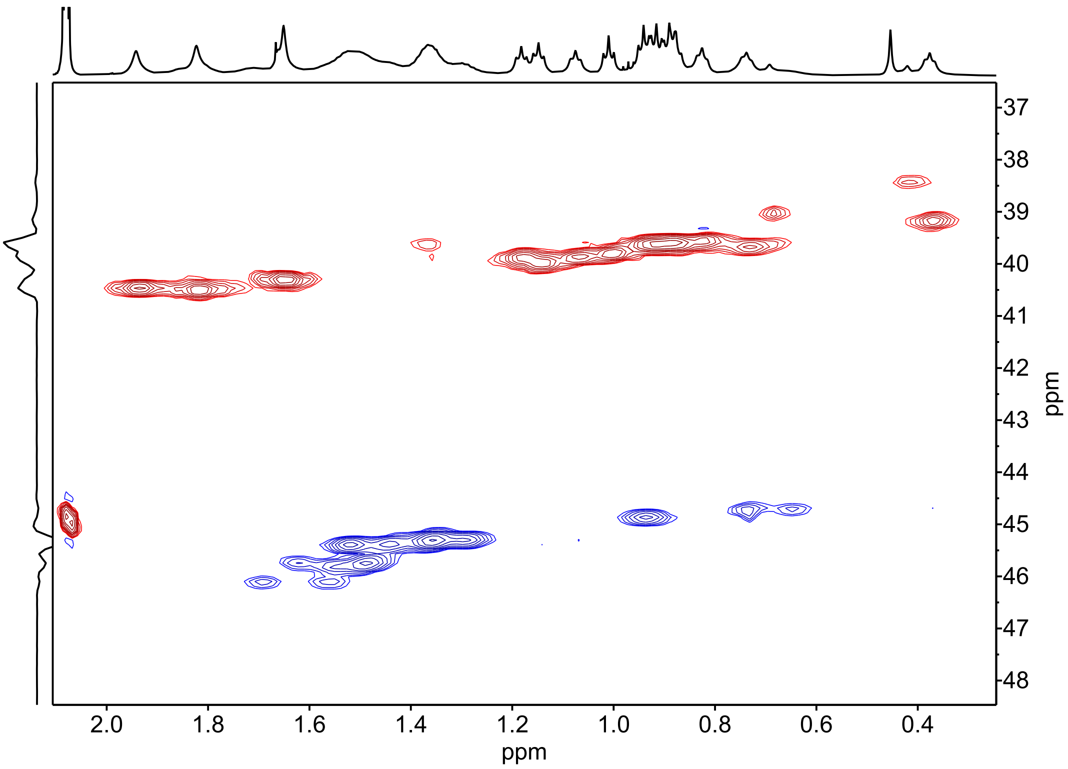

Figure 98. Partial ^{1}H-^{13}C HSQC NMR (700 MHz and 176 MHz, 375 K, Toluene-d_8) spectrum of **(OMe-cube)$_3$**.

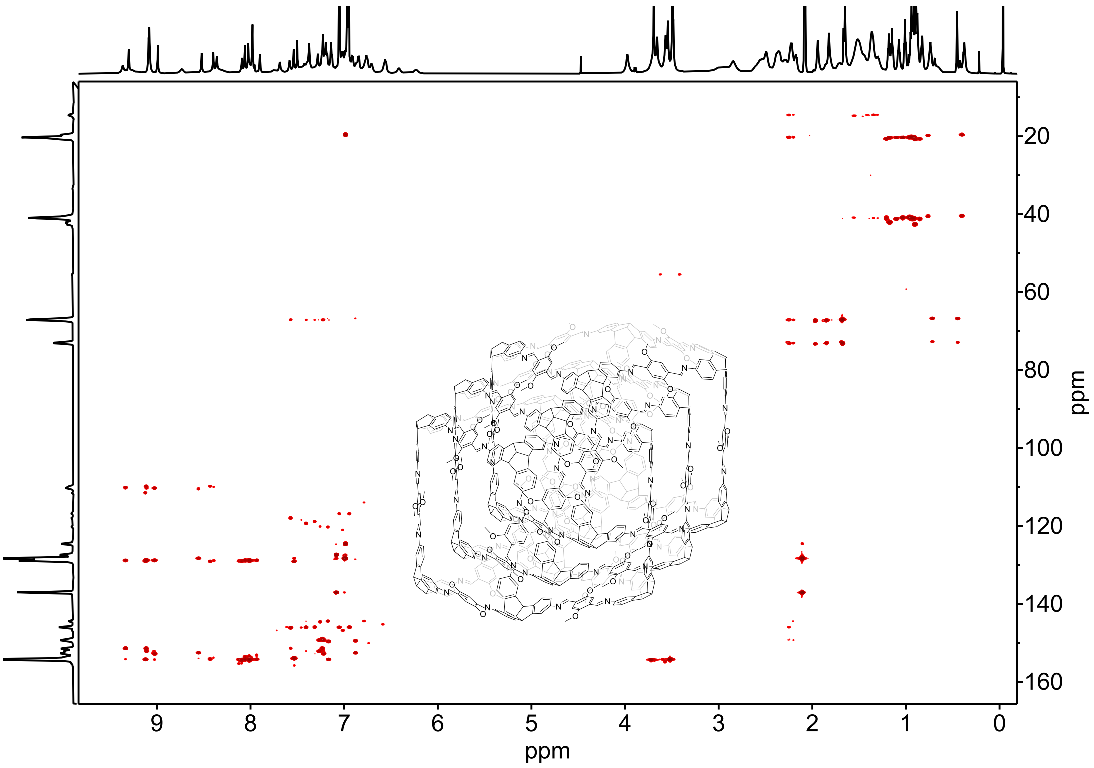

Figure 99. ^{1}H-^{13}C HMBC NMR (700 MHz and 176 MHz, 375 K, Toluene-d_8) spectrum of **(OMe-cube)$_3$**.

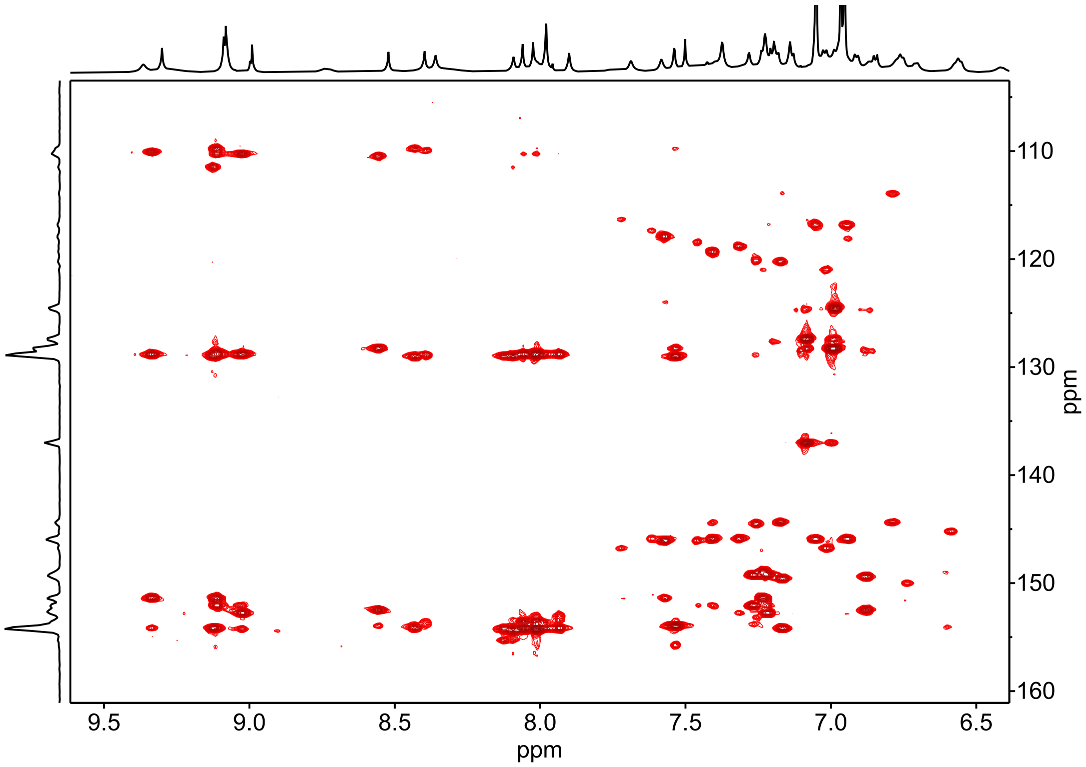

Figure 100. Partial ^{1}H-^{13}C HMBC NMR (700 MHz and 176 MHz, 375 K, Toluene-d$_8$) spectrum of **(OMe-cube)$_3$**.

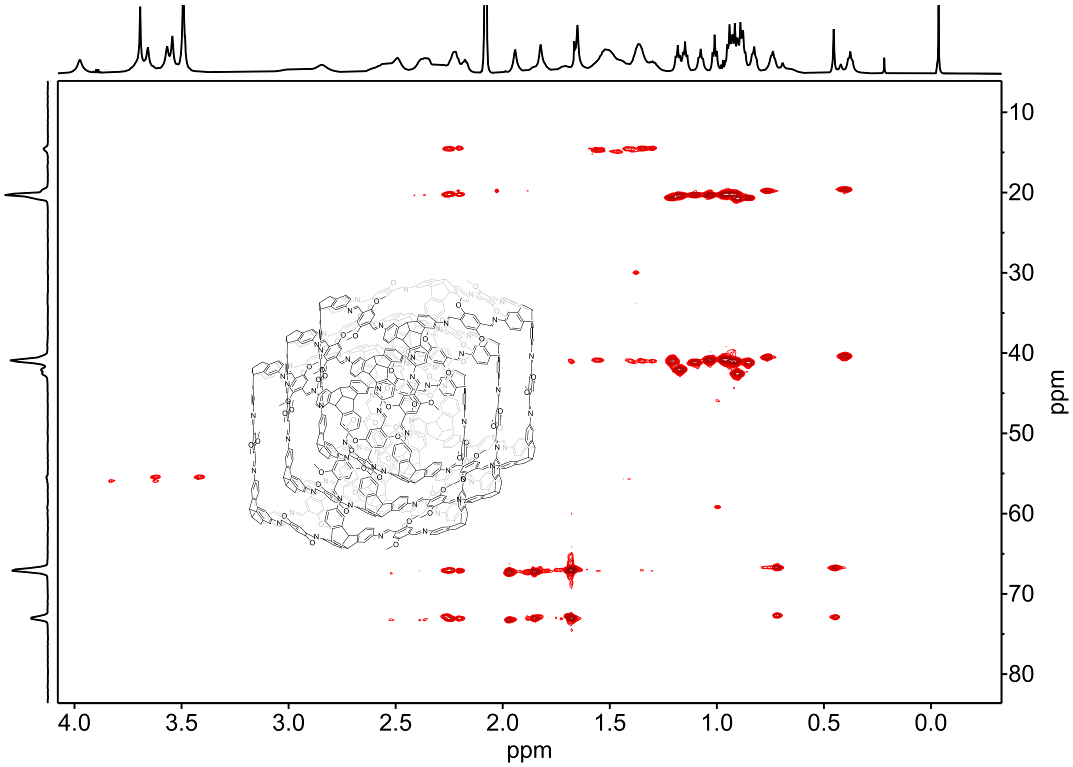

Figure 101. Partial ^{1}H-^{13}C HMBC NMR (700 MHz and 176 MHz, 375 K, Toluene-d$_8$) spectrum of **(OMe-cube)$_3$**.

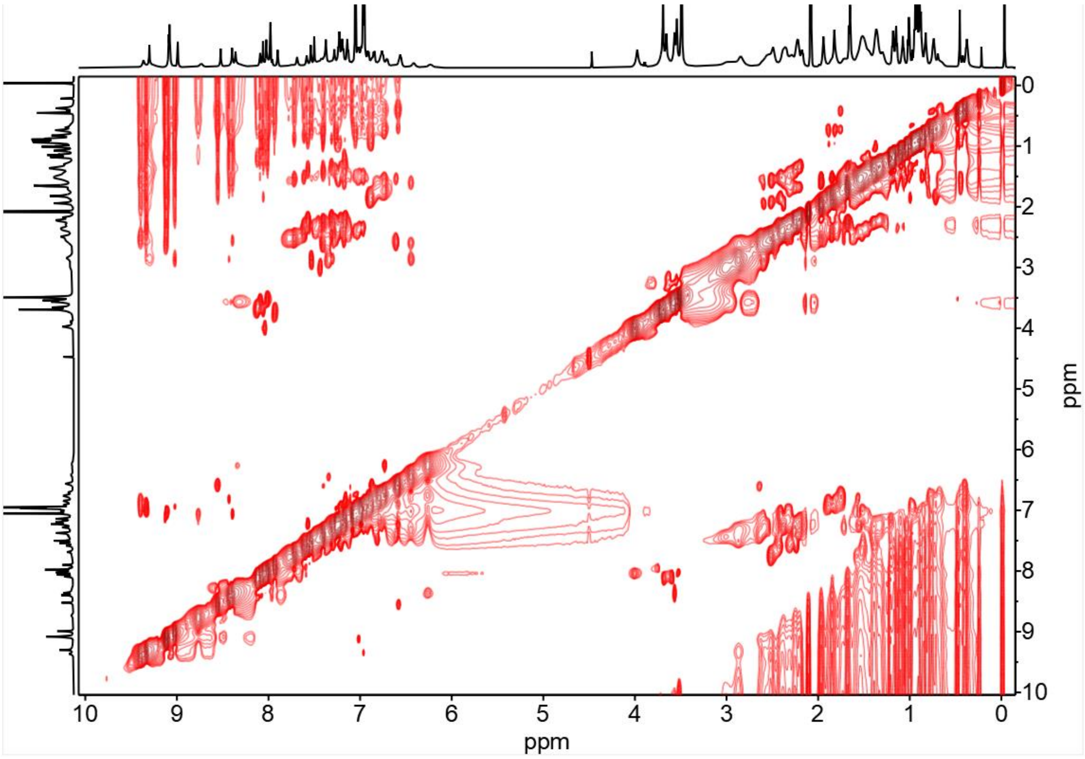

Figure 102. ^{1}H-^{1}H NOESY NMR (700 MHz, 375 K, Toluene-d$_8$) spectrum of **(OMe-cube)$_3$**.

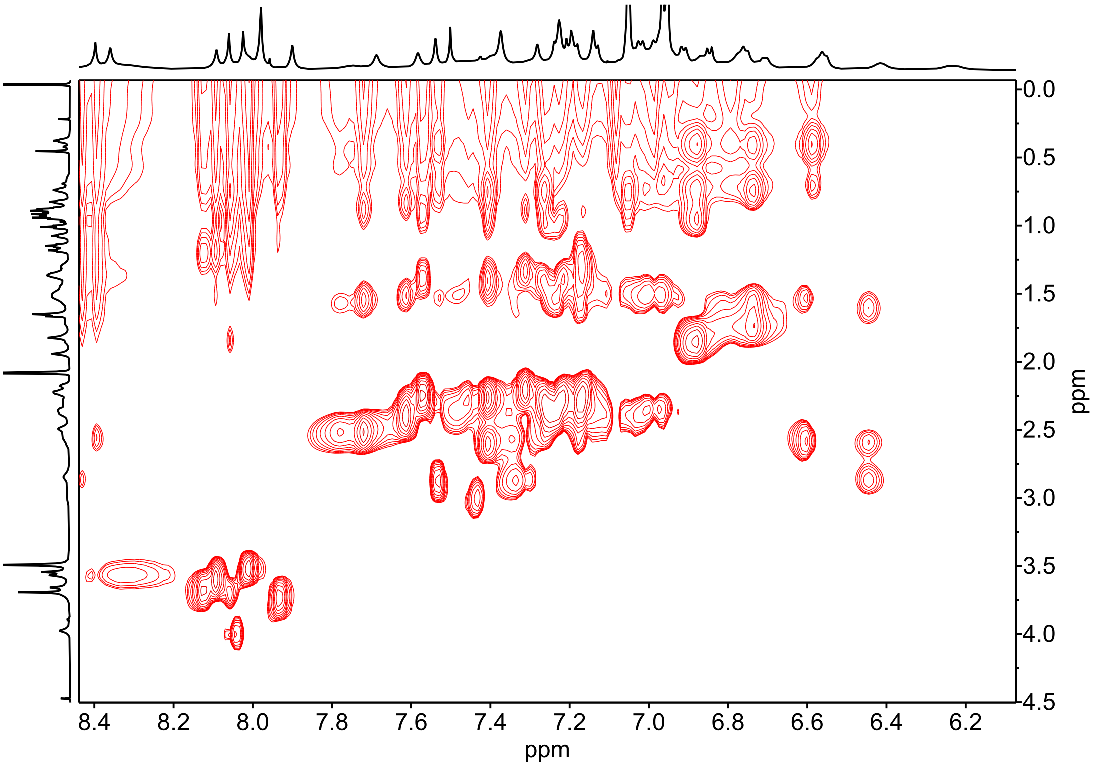

Figure 103. Partial ^{1}H-^{1}H NOESY NMR (700 MHz, 375 K, Toluene-d$_8$) spectrum of **(OMe-cube)$_3$**.

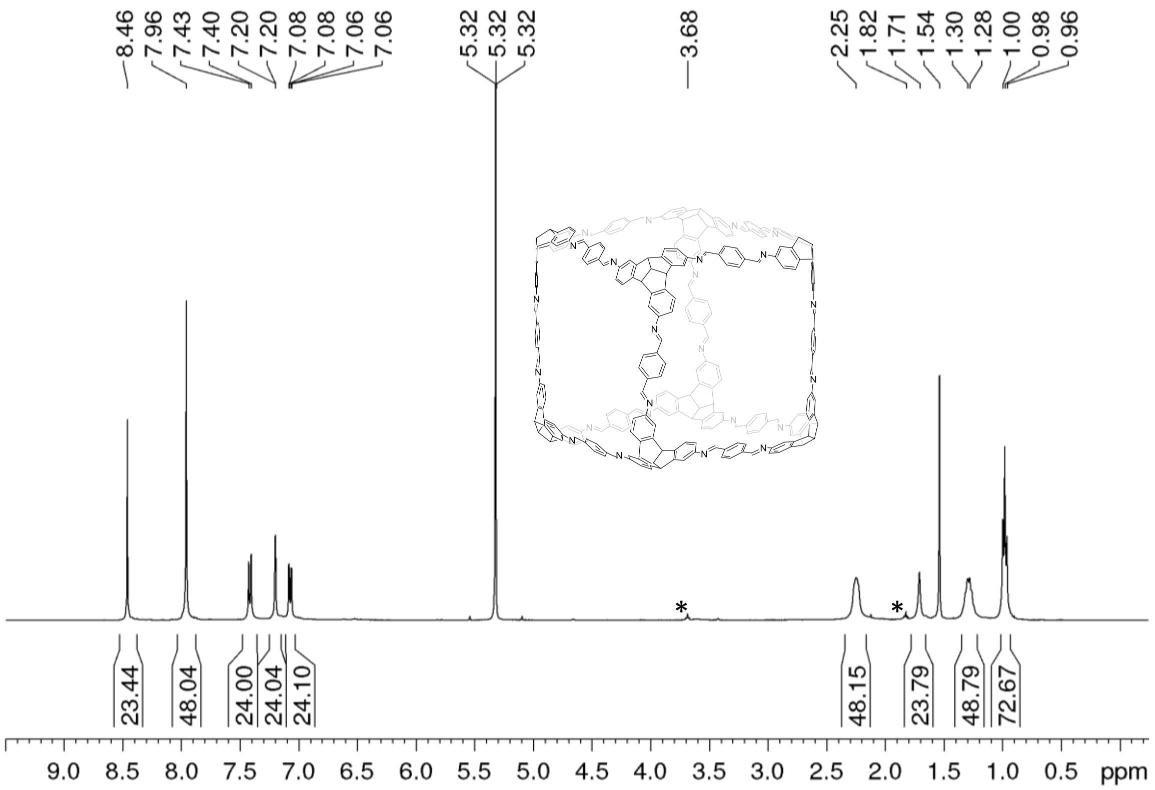

Figure 104. ^{1}H NMR (400 MHz, CD_2Cl_2) spectrum of **H-cube**. (*THF).

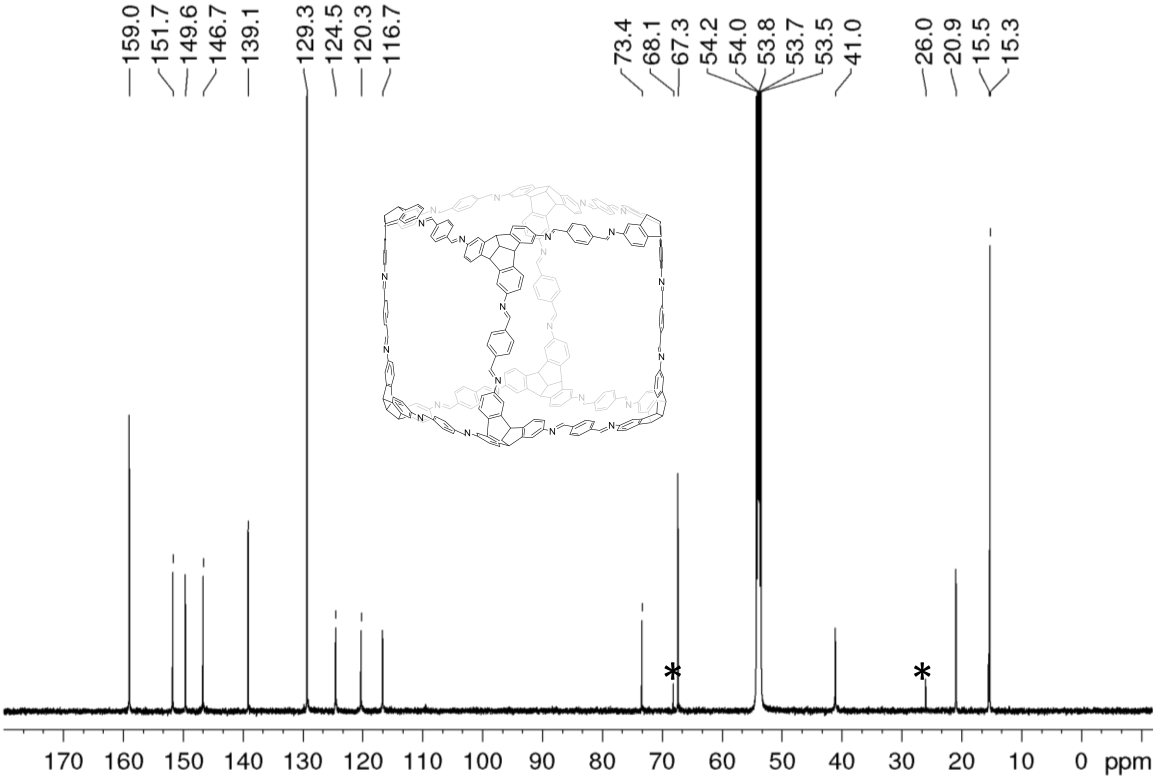

Figure 105. ^{13}C NMR (151 MHz, CD_2Cl_2) spectrum of **H-cube**. (*THF).

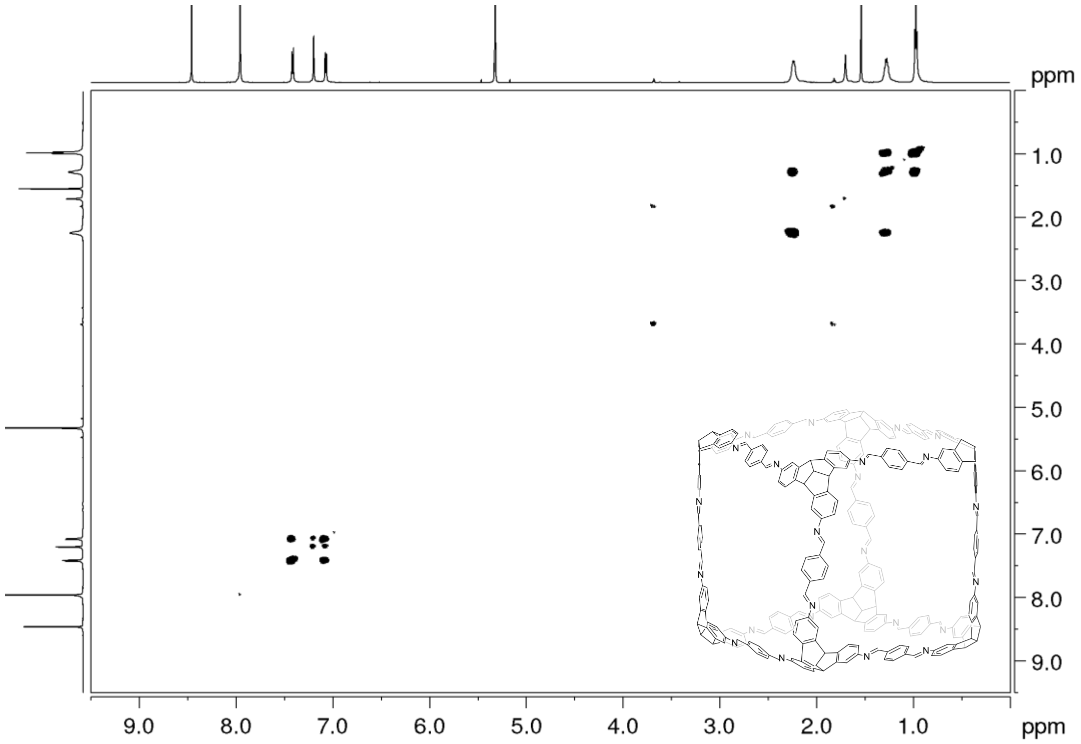

Figure 106. ^{1}H-^{1}H COSY NMR (600 MHz, CD_2Cl_2) spectrum of **H-cube**.

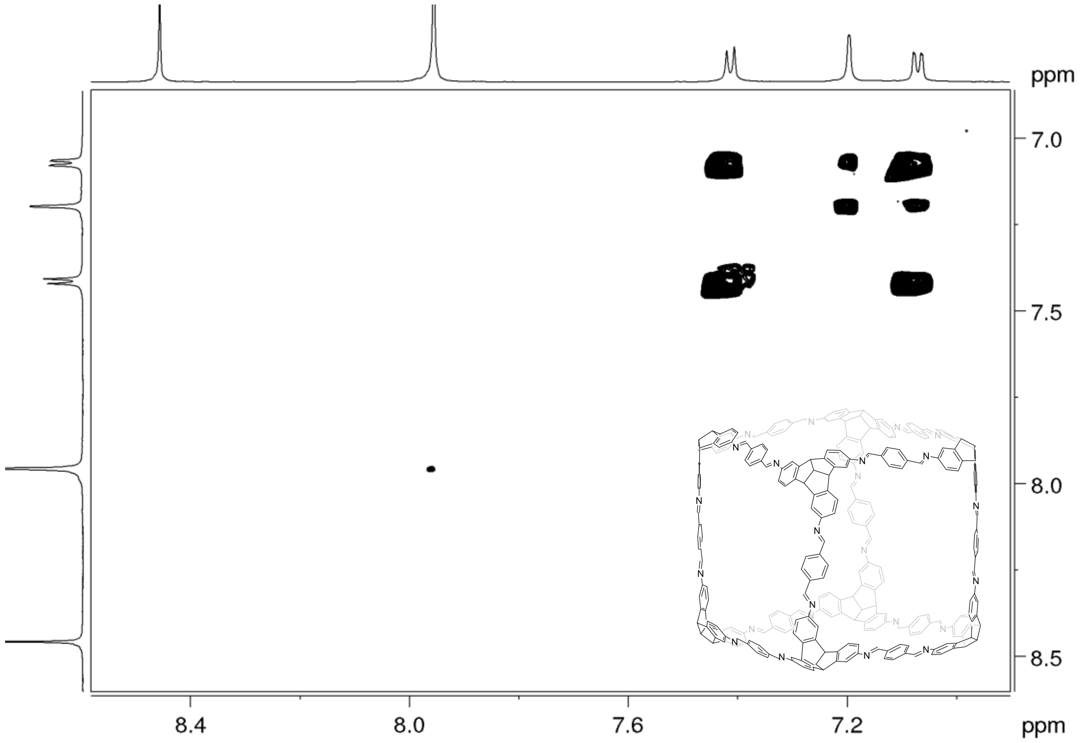

Figure 107. Partial ^{1}H-^{1}H COSY NMR (600 MHz, CD_2Cl_2) spectrum of **H-cube**.

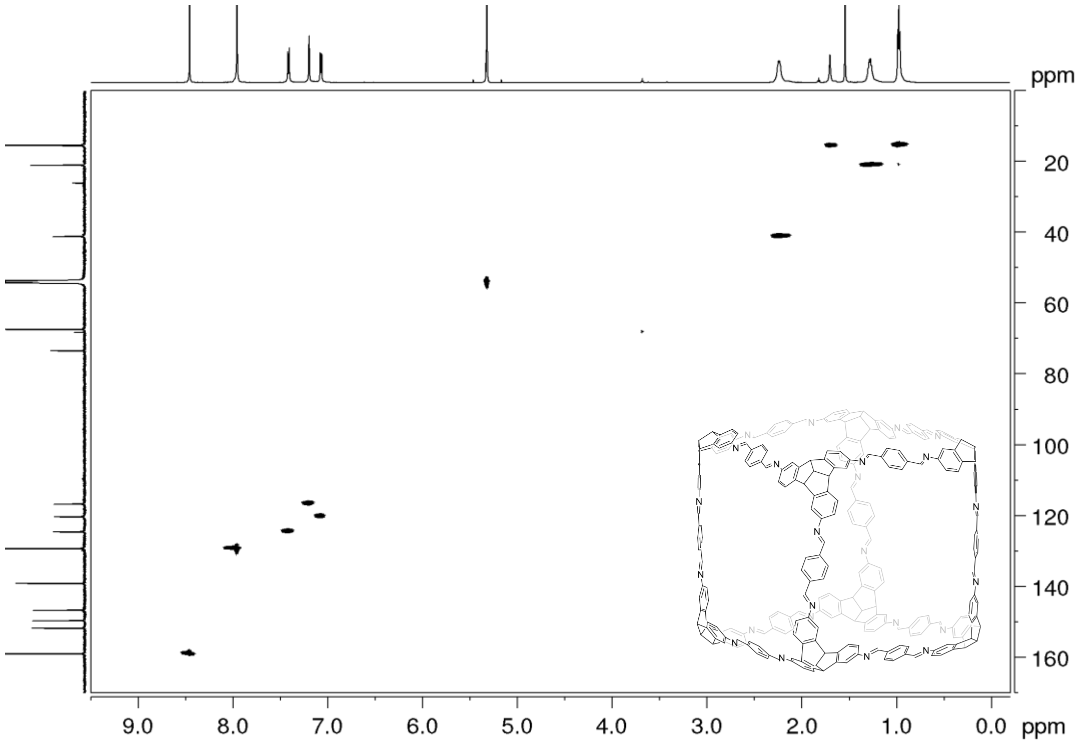

Figure 108. ^{1}H-^{13}C HSQC NMR (600 MHz and 151 MHz, CD_2Cl_2) spectrum of **H-cube**.

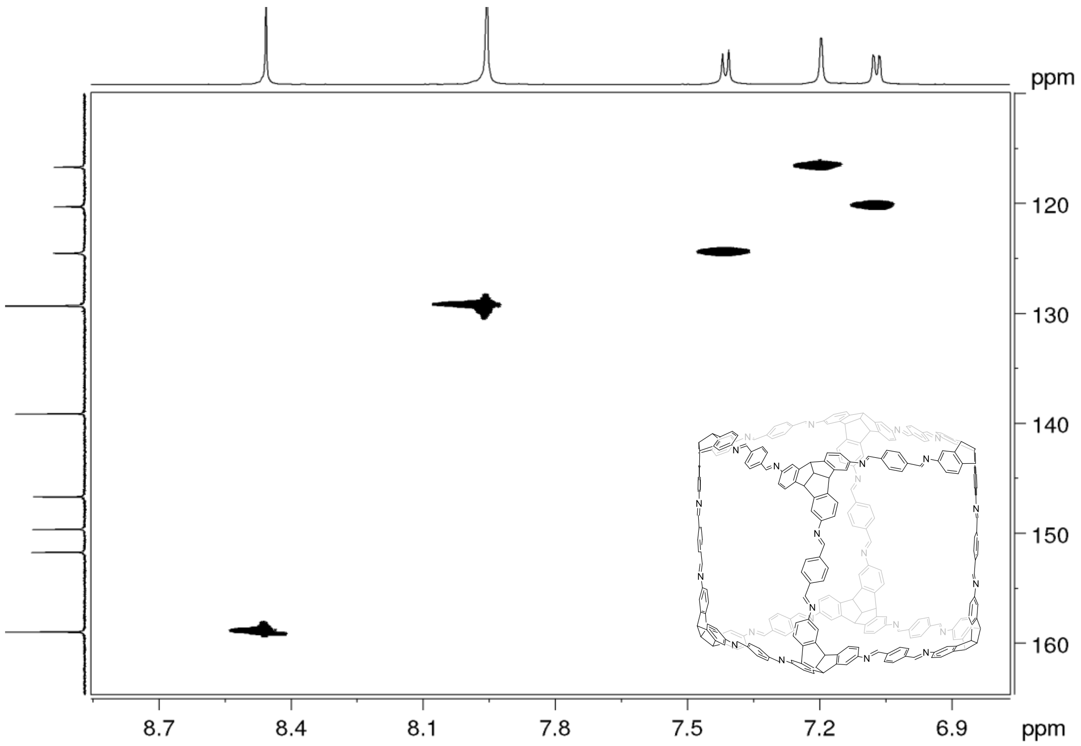

Figure 109. Partial ^{1}H-^{13}C HSQC NMR (600 MHz and 151 MHz, CD_2Cl_2) spectrum of **H-cube**.

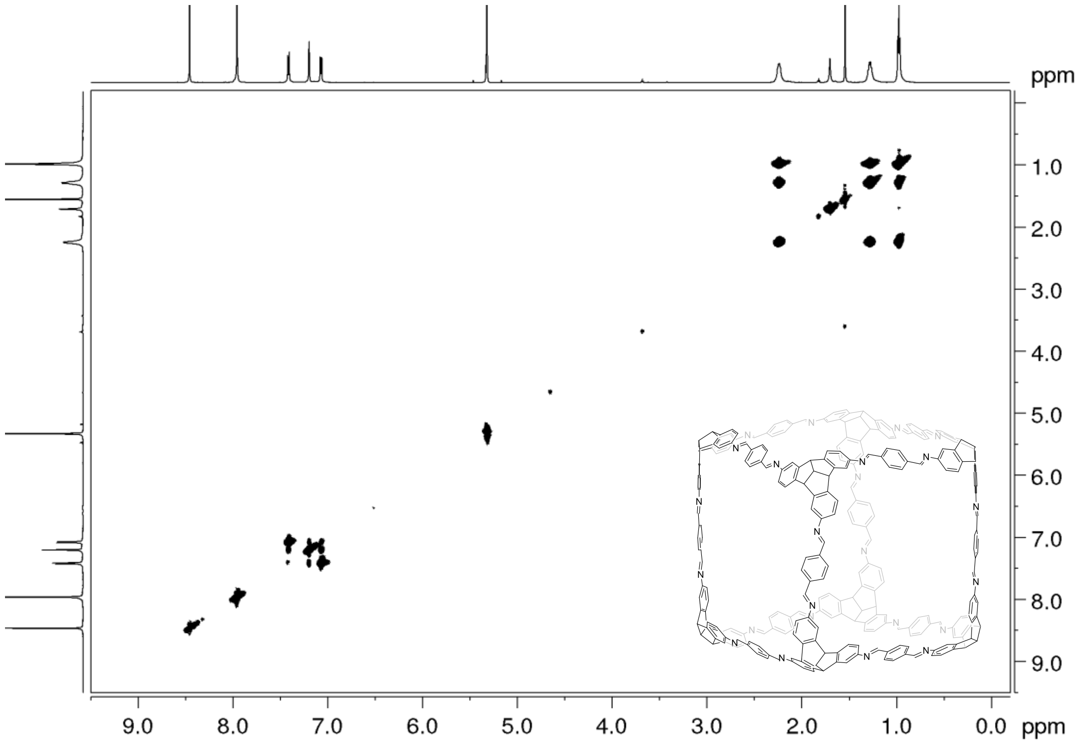

Figure 110. ^1H-^1H TOCSY NMR (600 MHz, CD_2Cl_2) spectrum of **H-cube**.

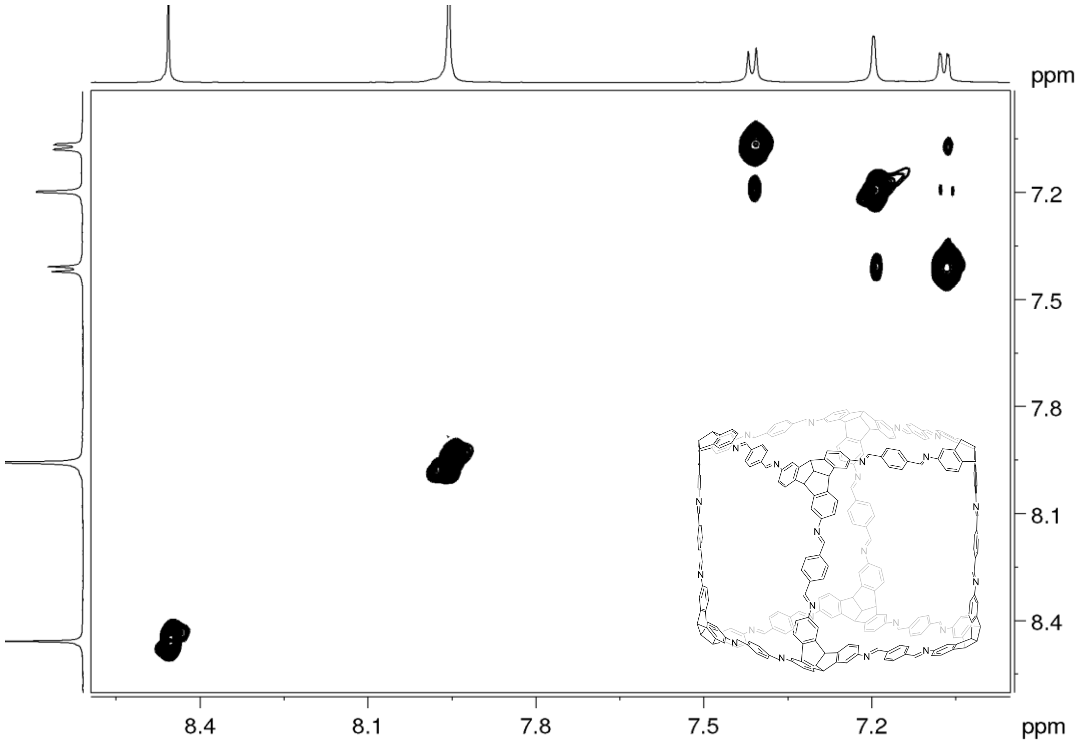

Figure 111. Partial ^1H-^1H TOCSY NMR (600 MHz, CD_2Cl_2) spectrum of **H-cube**.

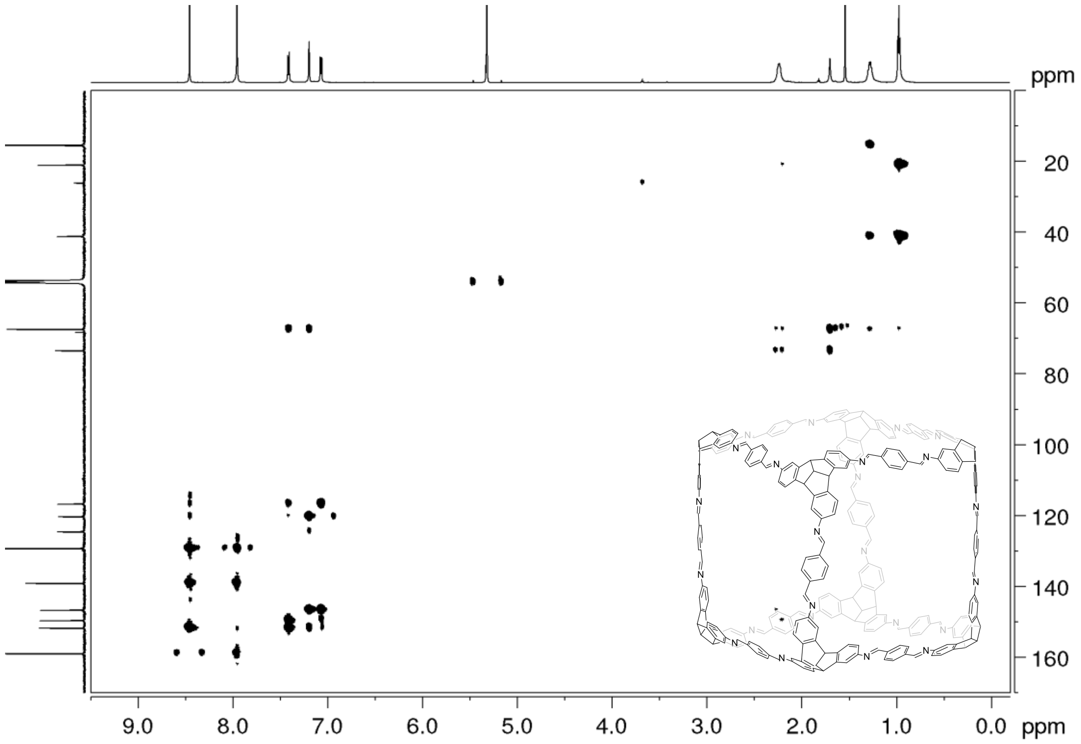

Figure 112. ^{1}H-^{13}C HMBC NMR (600 MHz and 151 MHz, CD_2Cl_2) spectrum of **H-cube**.

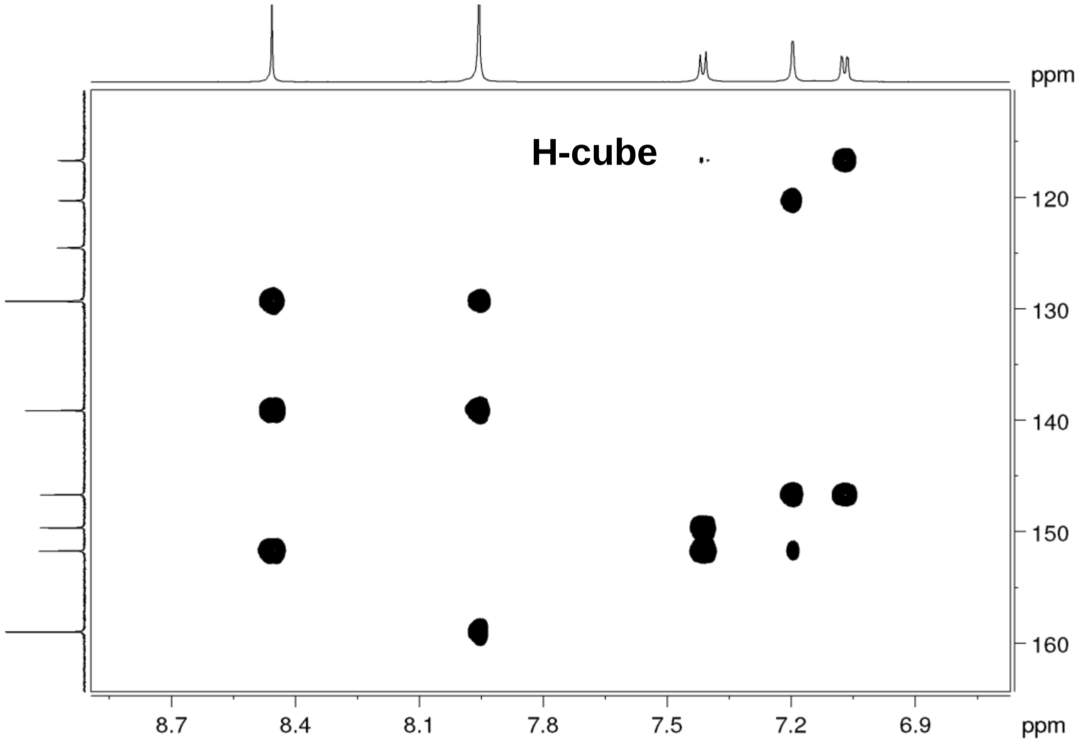

Figure 113. Partial ^{1}H-^{13}C HMBC NMR (600 MHz and 151 MHz, CD_2Cl_2) spectrum of **H-cube**.

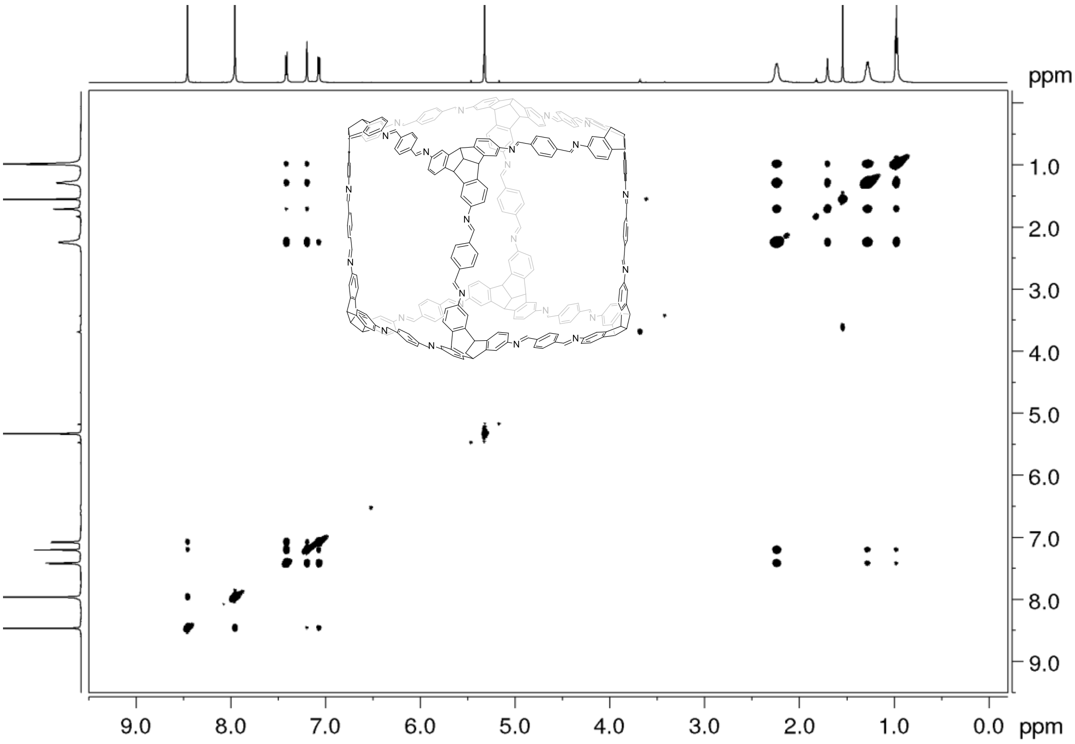

Figure 114. ^{1}H-^{1}H NOESY NMR (600 MHz, CD_2Cl_2) spectrum of **H-cube**.

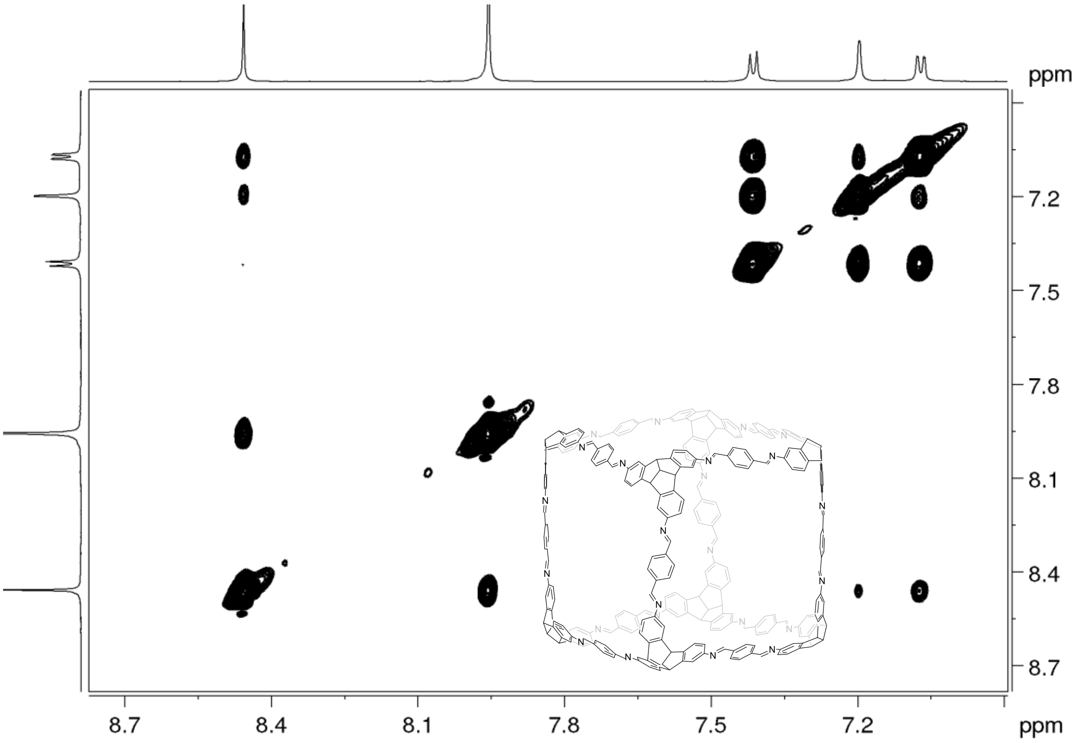

Figure 115. Partial ^{1}H-^{1}H NOESY NMR (600 MHz, CD_2Cl_2) spectrum of **H-cube**.

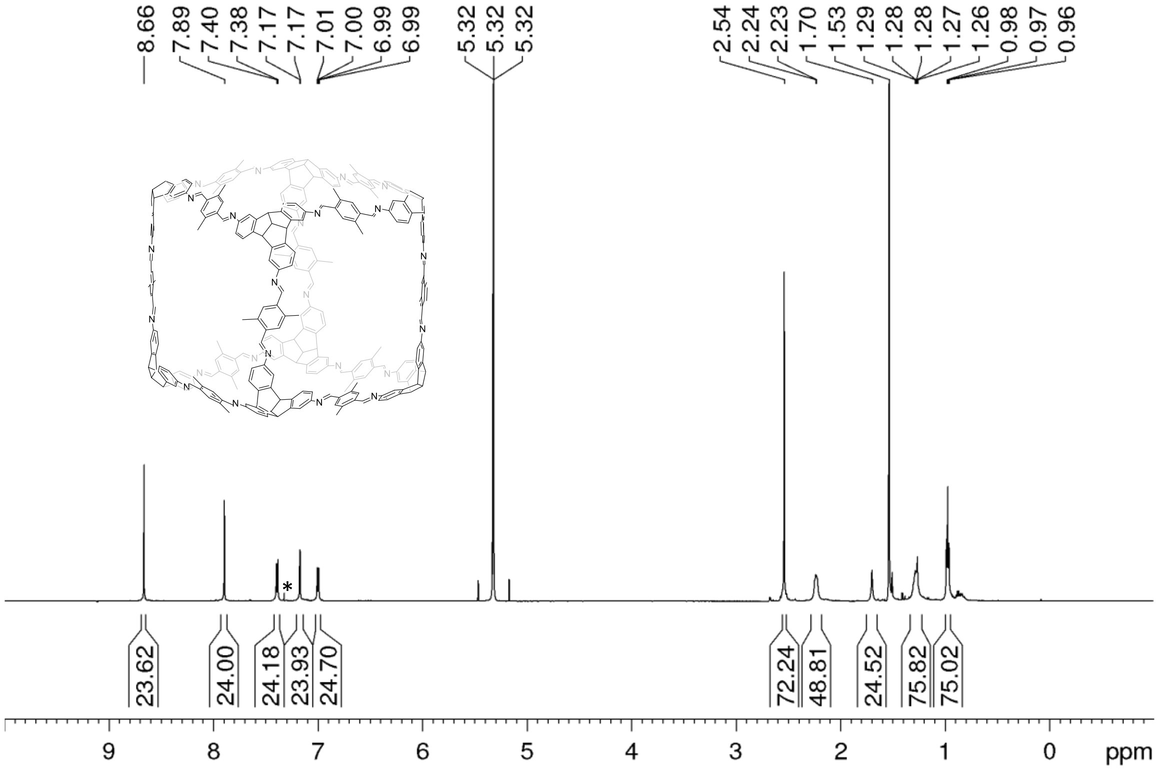

Figure 116. ^{1}H NMR (600 MHz, CD_2Cl_2) spectrum of **Me-cube**. (*$CHCl_3$).

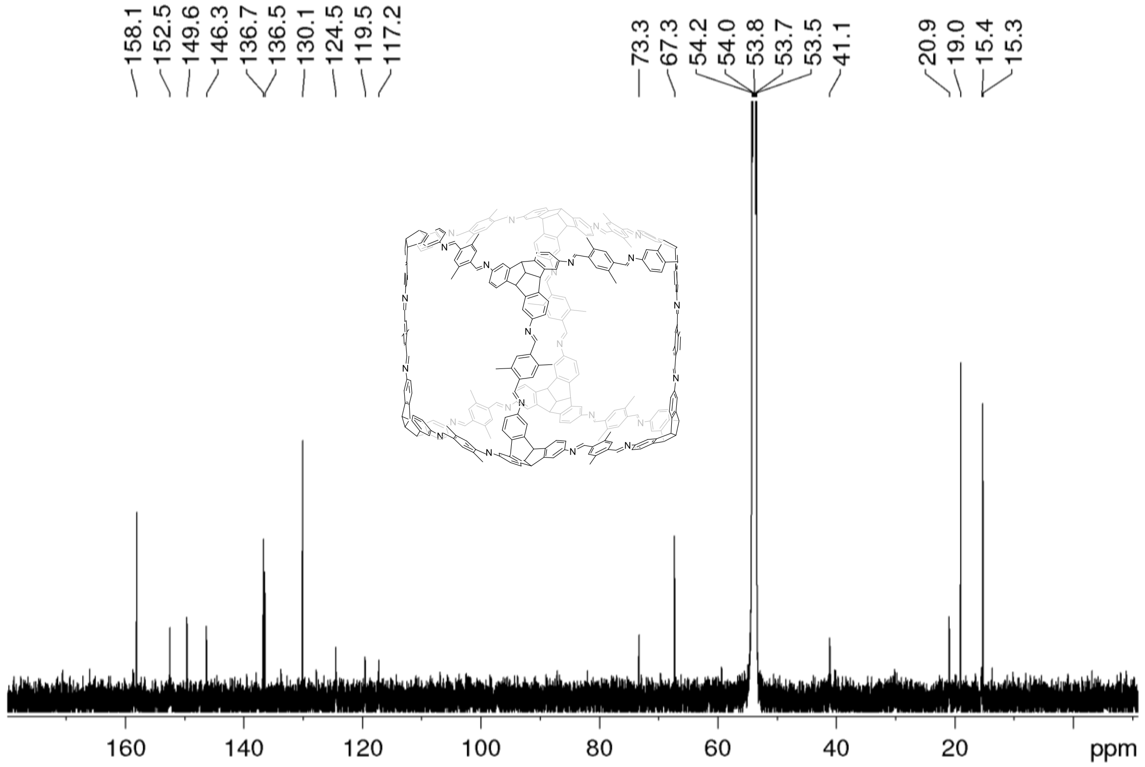

Figure 117. ^{13}C NMR spectrum (151 MHz, CD_2Cl_2) of **Me-cube**.

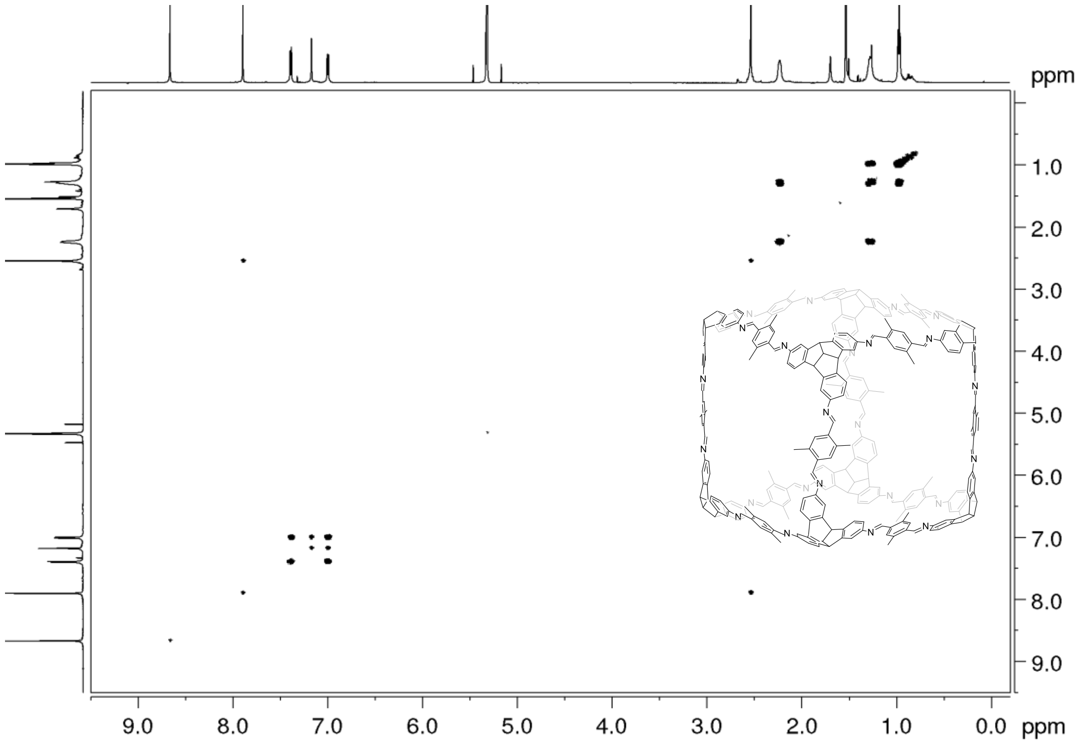

Figure 118. ^{1}H-^{1}H COSY NMR spectrum (600 MHz, CD_2Cl_2) of **Me-cube**.

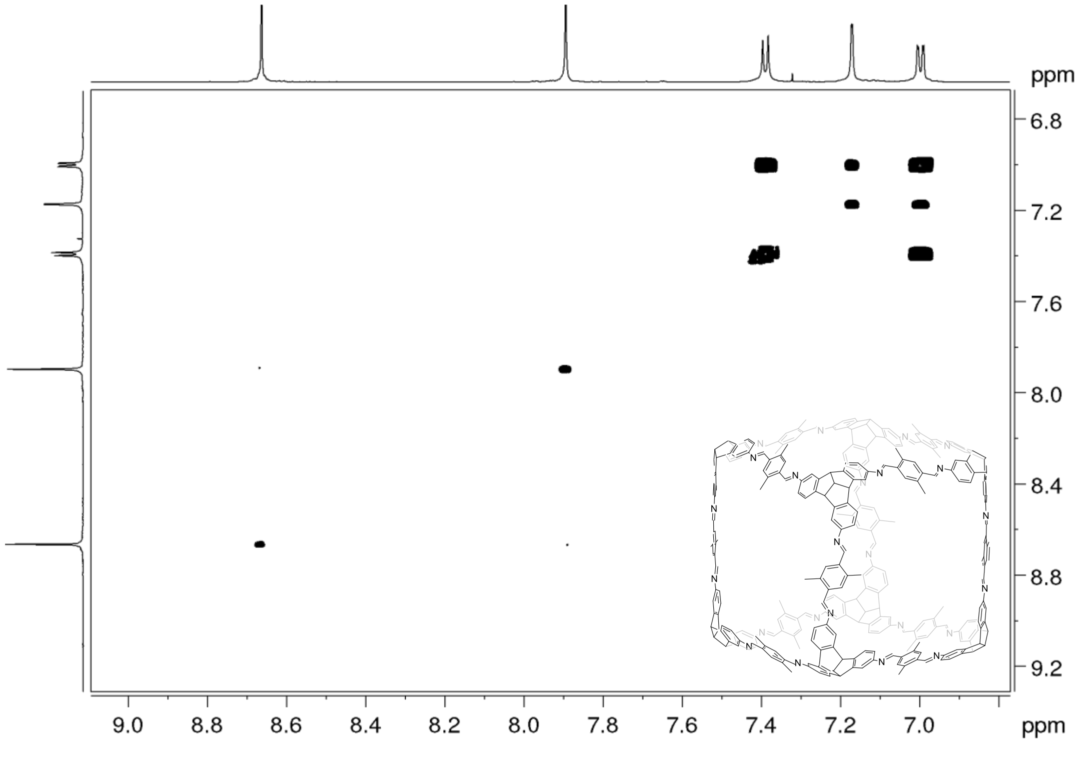

Figure 119. Partial ^{1}H-^{1}H COSY NMR spectrum (600 MHz, CD_2Cl_2) of **Me-cube**.

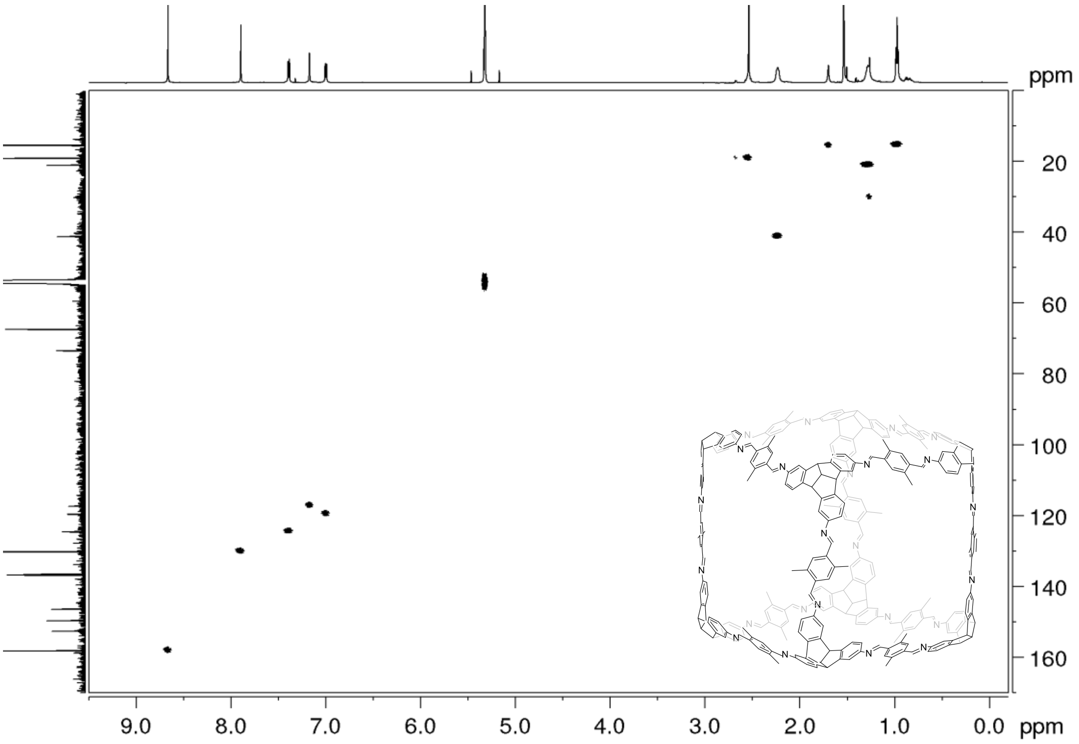

Figure 120. ^{1}H-^{13}C HSQC NMR (600 MHz and 151 MHz, CD_2Cl_2) spectrum of **Me-cube**.

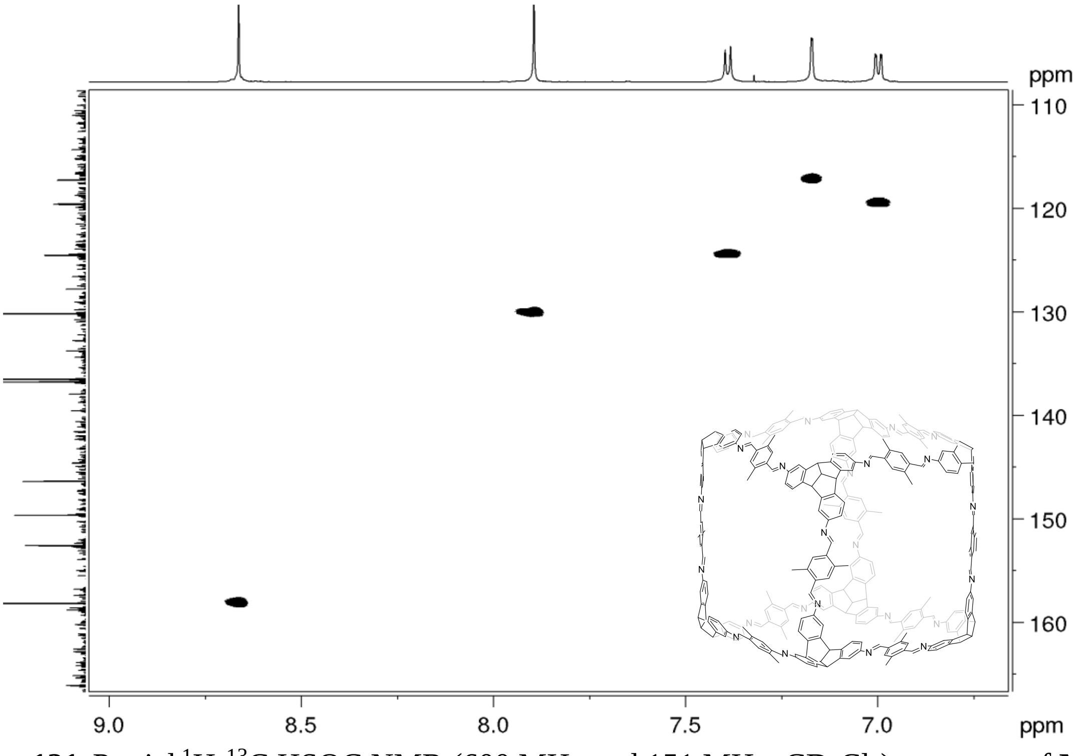

Figure 121. Partial ^{1}H-^{13}C HSQC NMR (600 MHz and 151 MHz, CD_2Cl_2) spectrum of **Me-cube**.

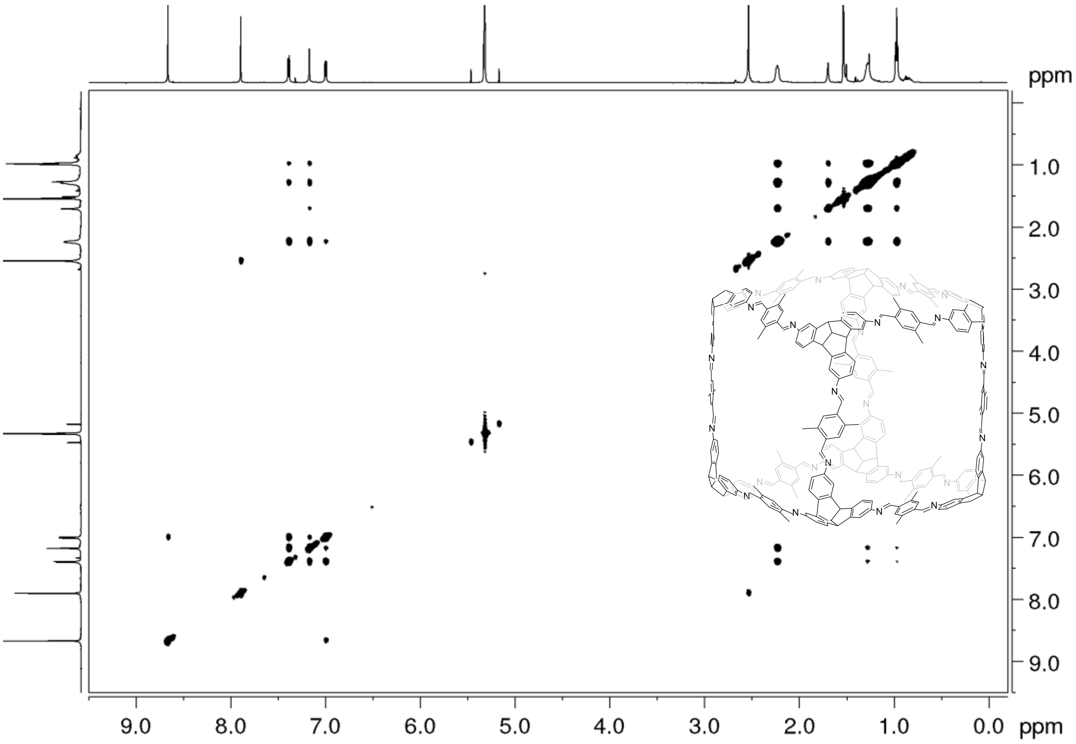

Figure 122. ^{1}H-^{1}H NOESY NMR (600 MHz, CD_2Cl_2) spectrum of **Me-cube**.

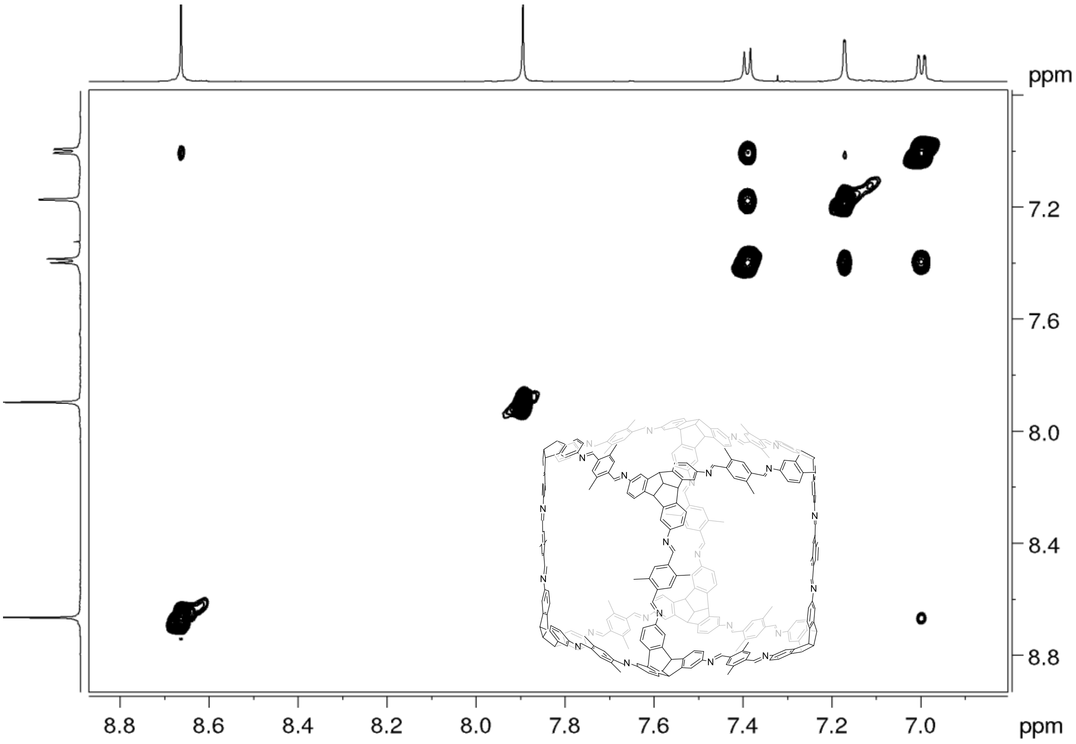

Figure 123. Partial ^{1}H-^{1}H NOESY NMR (600 MHz, CD_2Cl_2) spectrum of **Me-cube**.

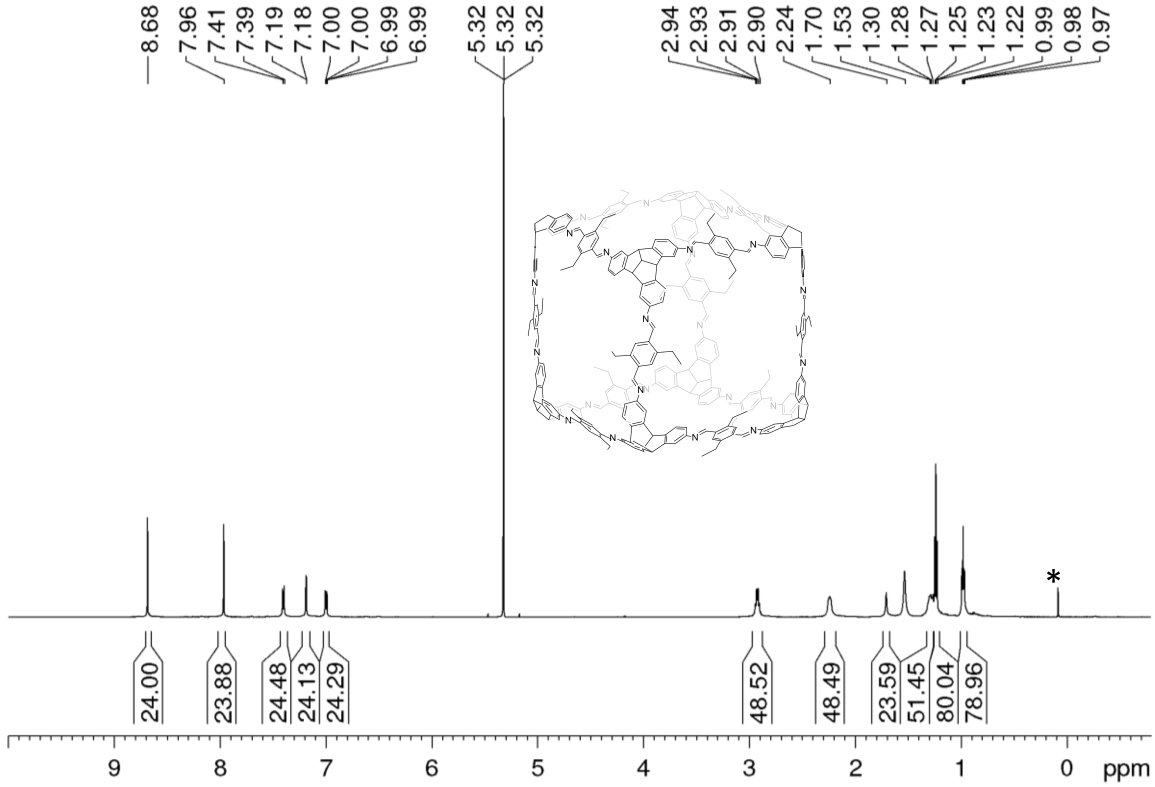

Figure 124. ^{1}H NMR (600 MHz, CD_2Cl_2) spectrum of **Et-cube**. (*silicon grease)

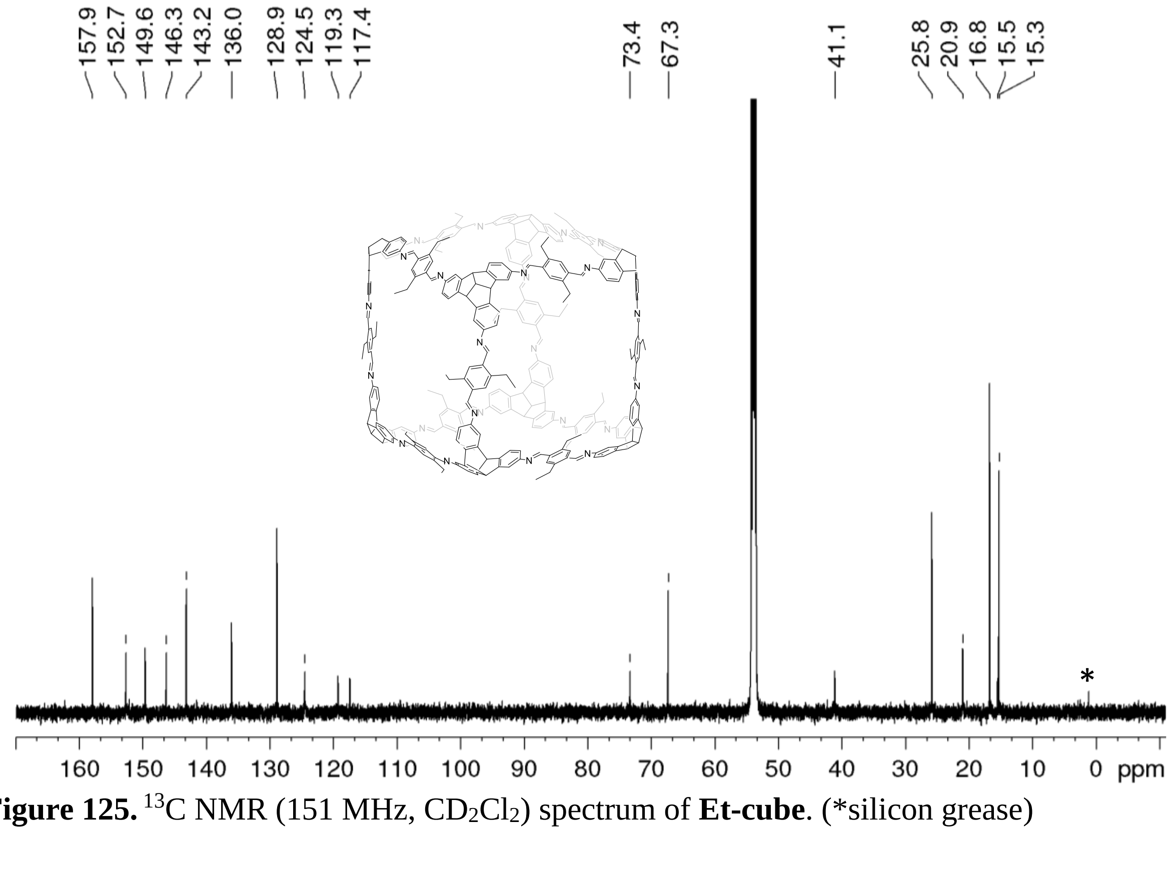

Figure 125. ^{13}C NMR (151 MHz, CD_2Cl_2) spectrum of **Et-cube**. (*silicon grease)

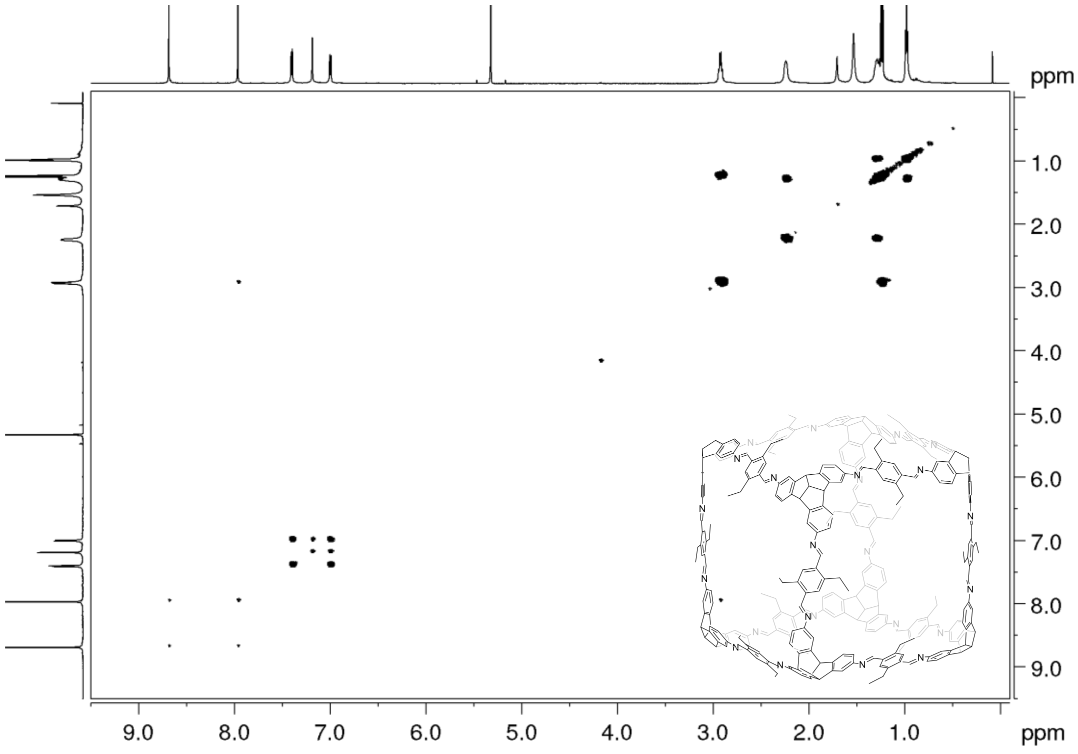

Figure 126. ^{1}H-^{1}H COSY NMR spectrum (600 MHz, CD_2Cl_2) of **Et-cube**.

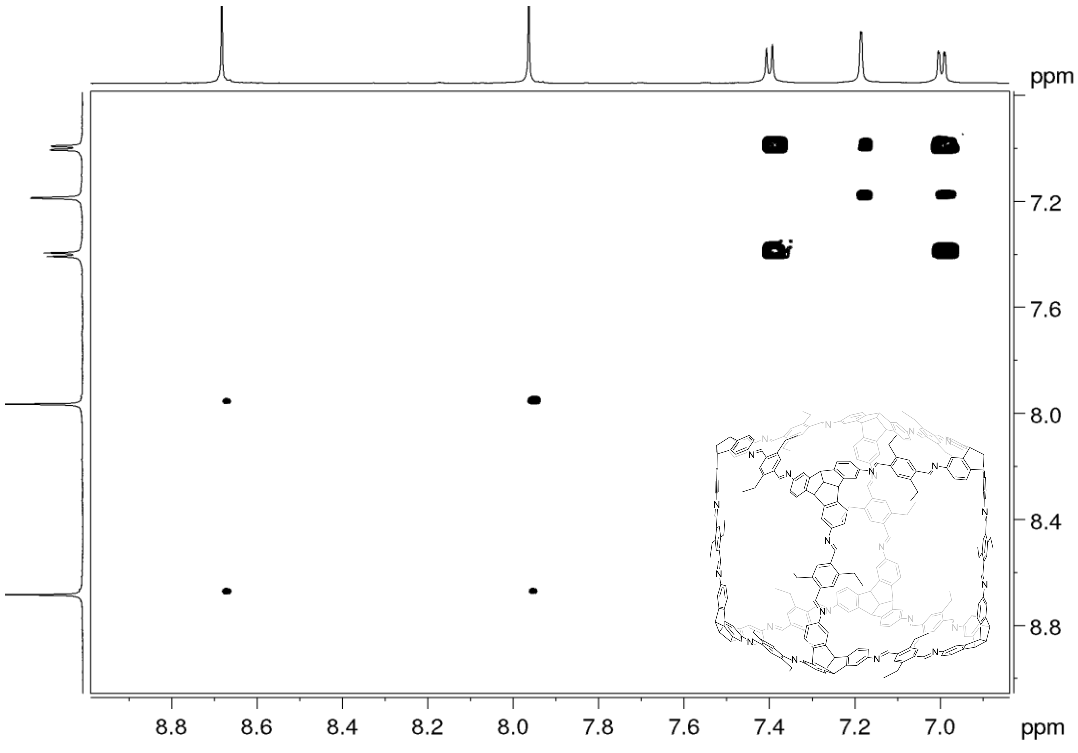

Figure 127. Partial ^{1}H-^{1}H COSY NMR spectrum (600 MHz, CD_2Cl_2) of **Et-cube**.

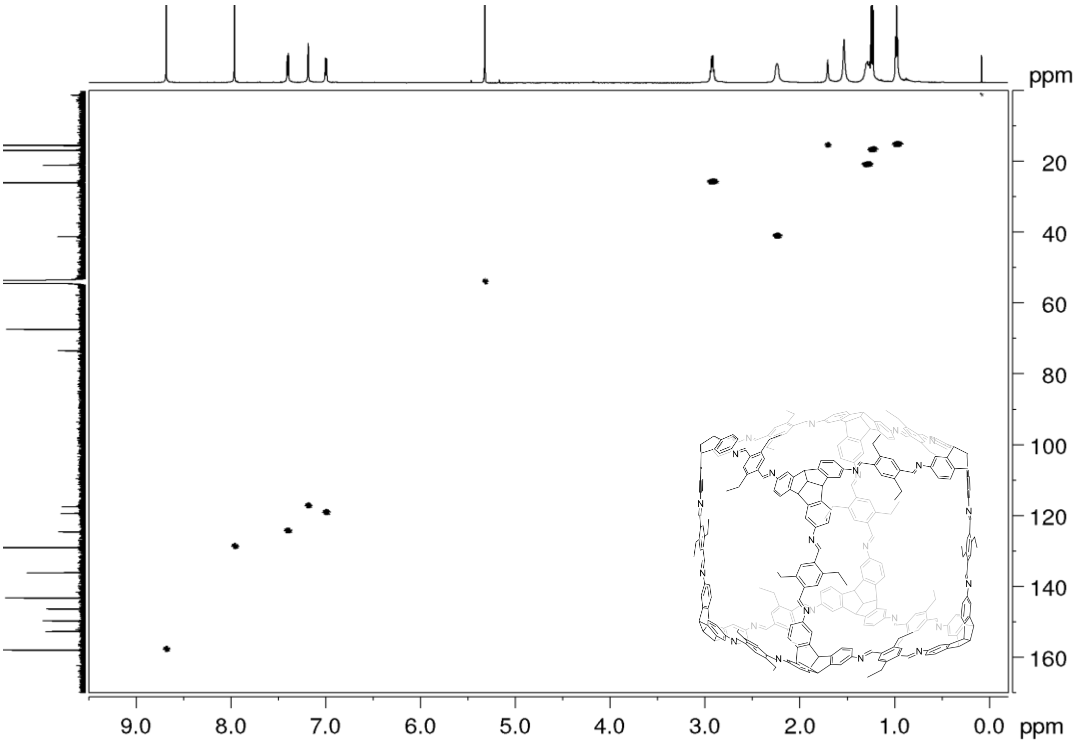

Figure 128. ^{1}H-^{13}C HSQC NMR (600 MHz and 151 MHz, CD_2Cl_2) spectrum of **Et-cube**.

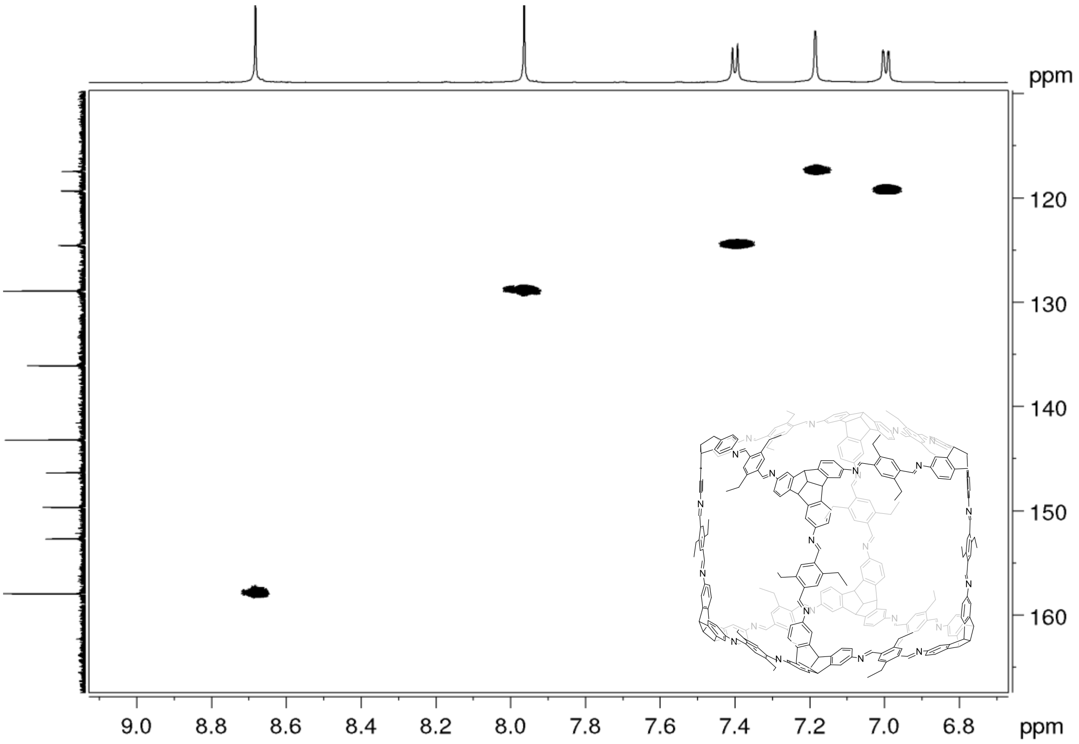

Figure 129. Partial ^{1}H-^{13}C HSQC NMR (600 MHz and 151 MHz, CD_2Cl_2) spectrum of **Et-cube**.

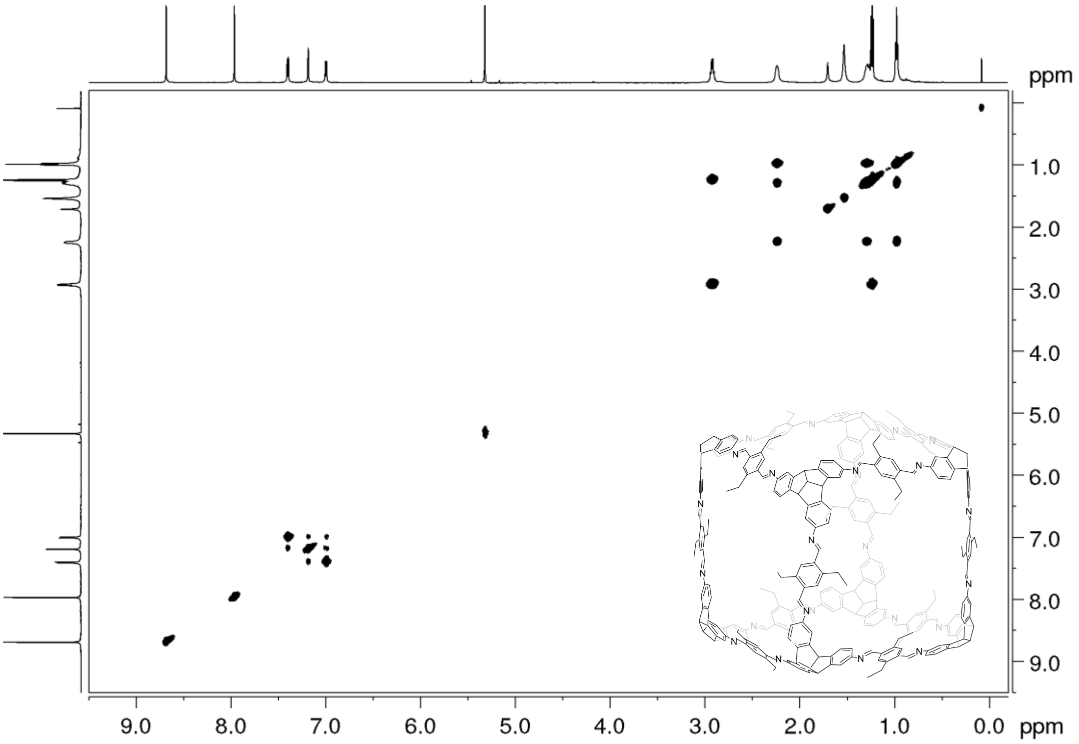

Figure 130. ^{1}H-^{1}H TOCSY NMR (600 MHz, CD_2Cl_2) spectrum of **Et-cube**.

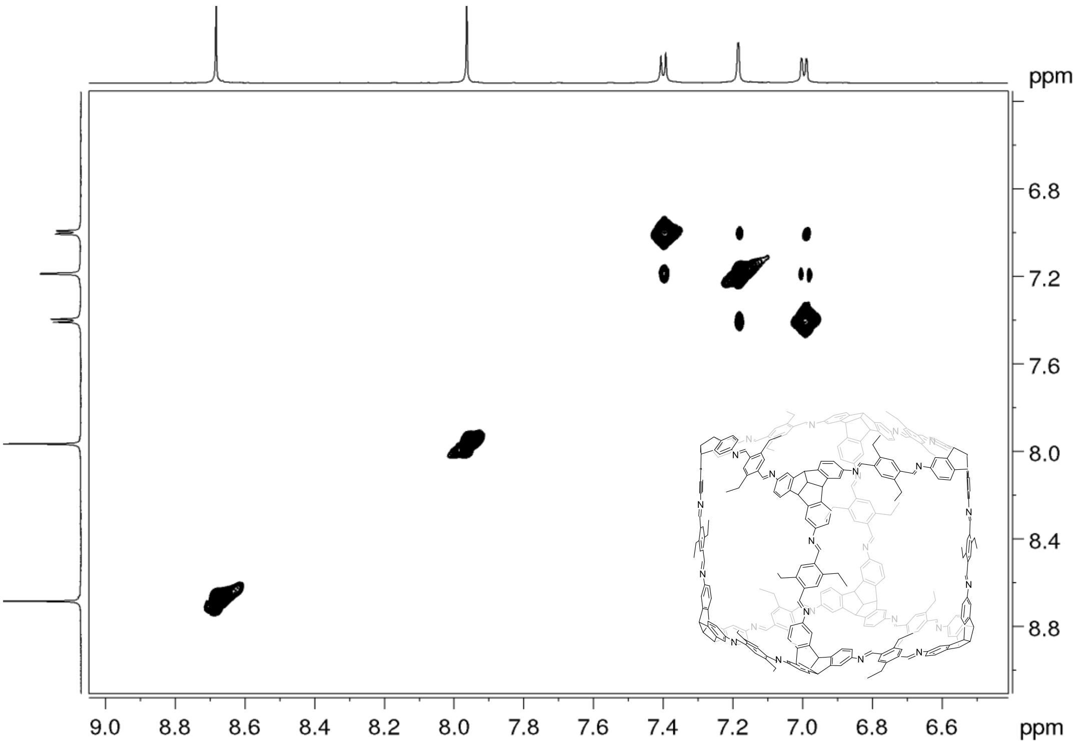

Figure 131. Partial ^{1}H-^{1}H TOCSY NMR (600 MHz, CD_2Cl_2) spectrum of **Et-cube**.

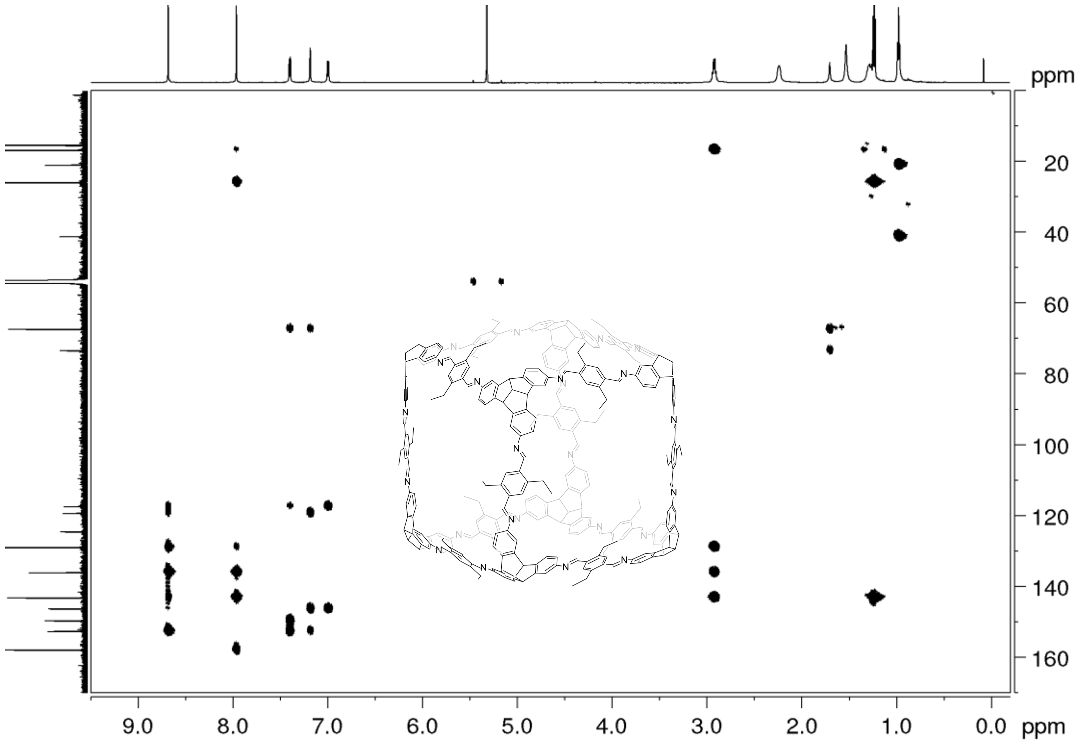

Figure 132. ^{1}H-^{13}C HMBC NMR (600 MHz and 151 MHz, CD_2Cl_2) spectrum of **Et-cube**.

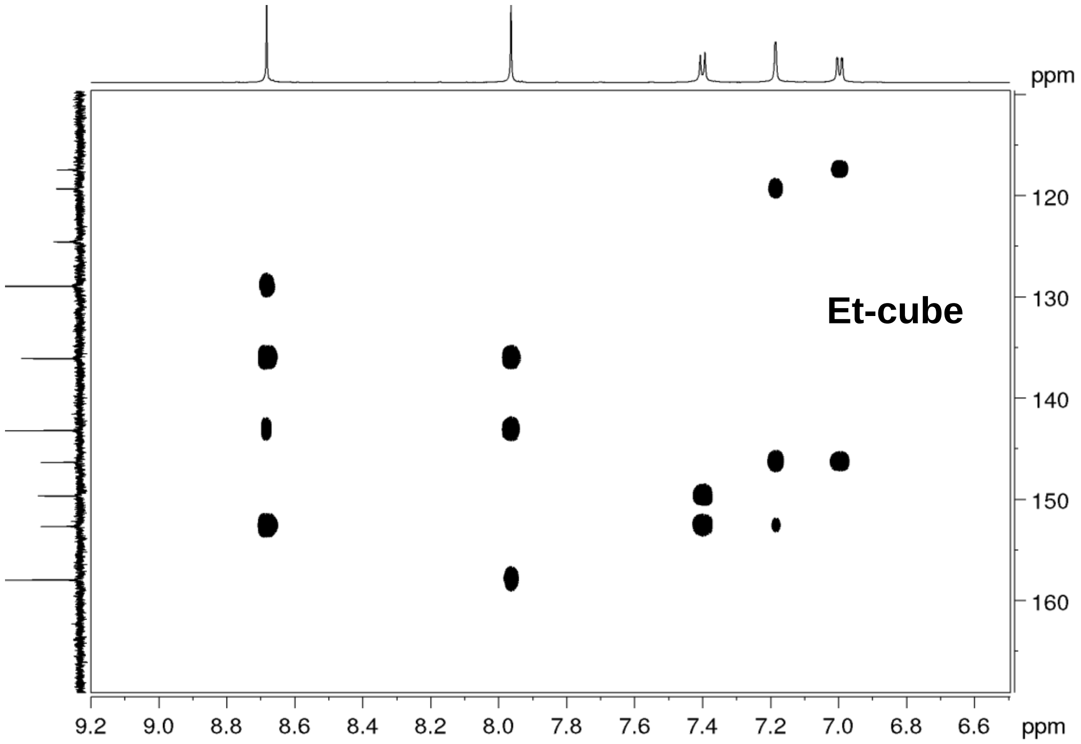

Figure 133. Partial ^{1}H-^{13}C HMBC NMR (600 MHz and 151 MHz, CD_2Cl_2) spectrum of **Et-cube**.

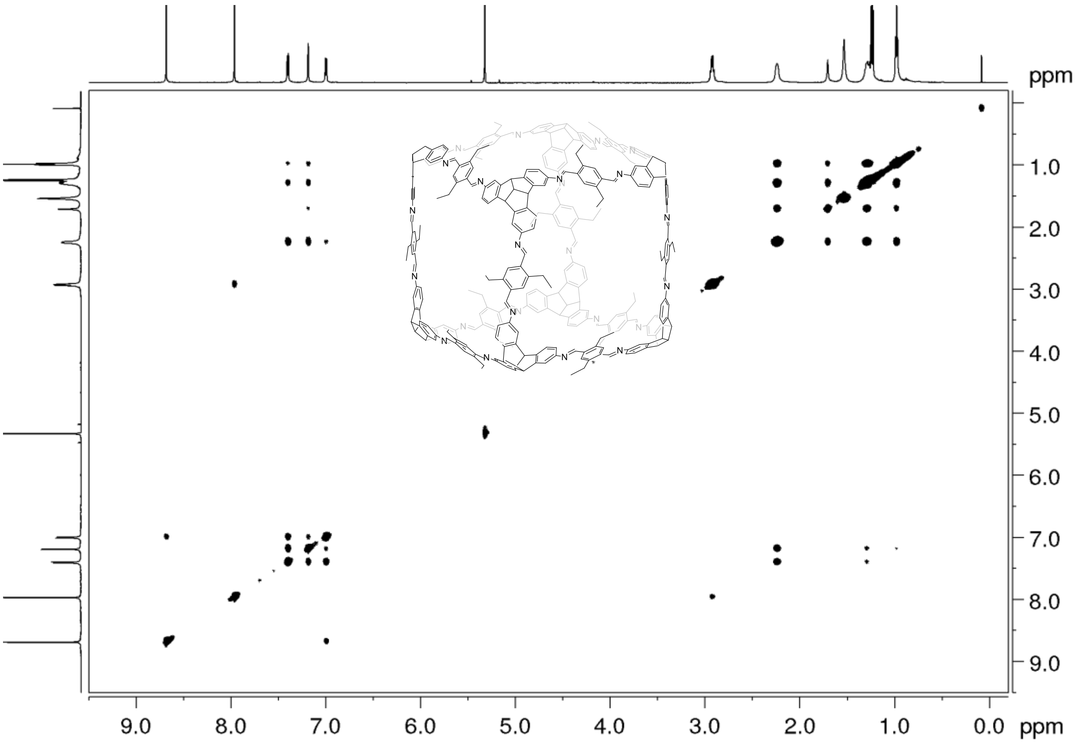

Figure 134. ^{1}H-^{1}H NOESY NMR (600 MHz, CD_2Cl_2) spectrum of **Et-cube**.

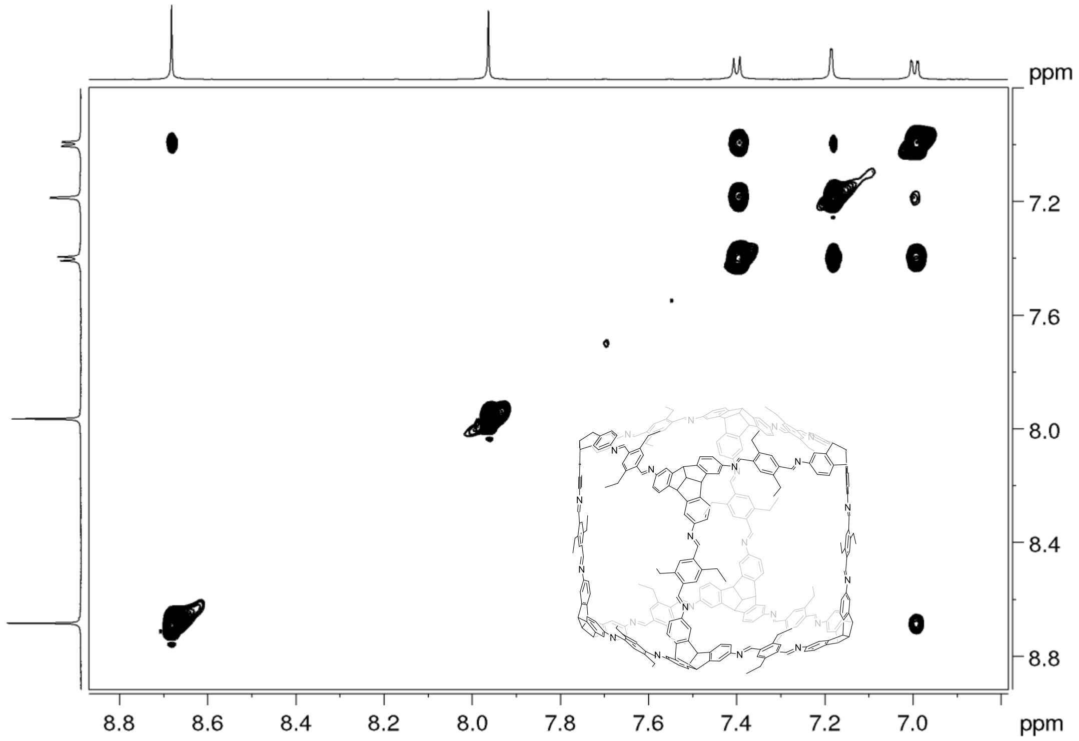

Figure 135. Partial ^{1}H-^{1}H NOESY NMR (600 MHz, CD_2Cl_2) spectrum of **Et-cube**.

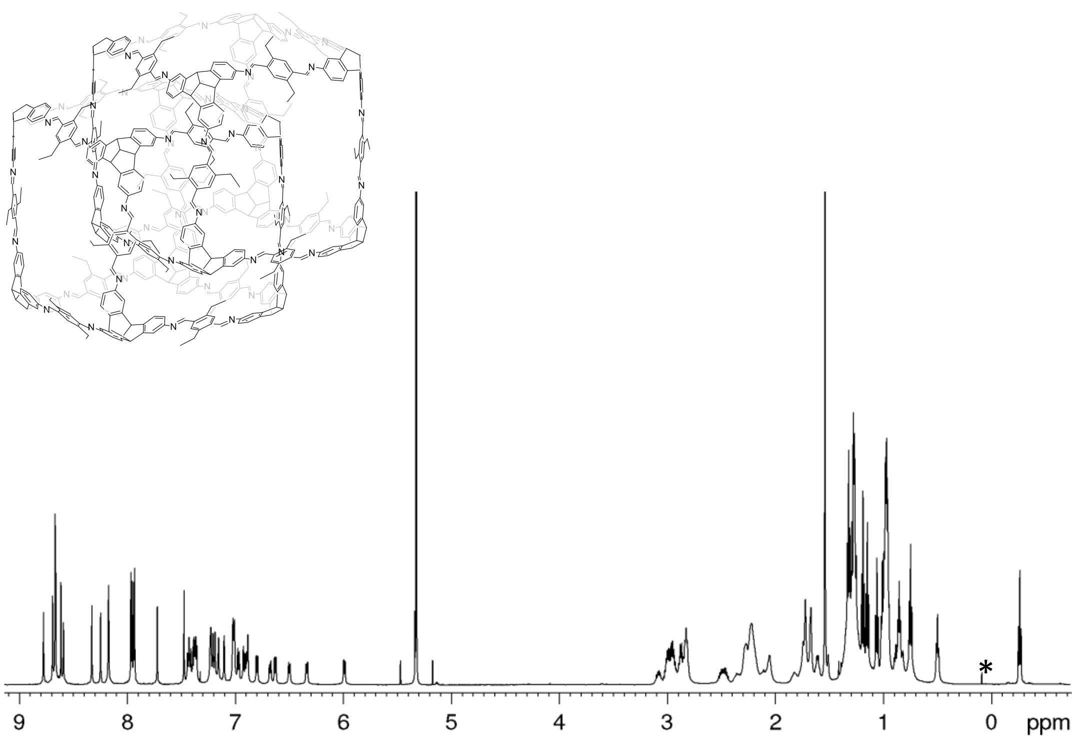

Figure 136. ^{1}H NMR (600 MHz, CD_2Cl_2) spectrum of **(Et-cube)$_2$**. (* silicon grease)

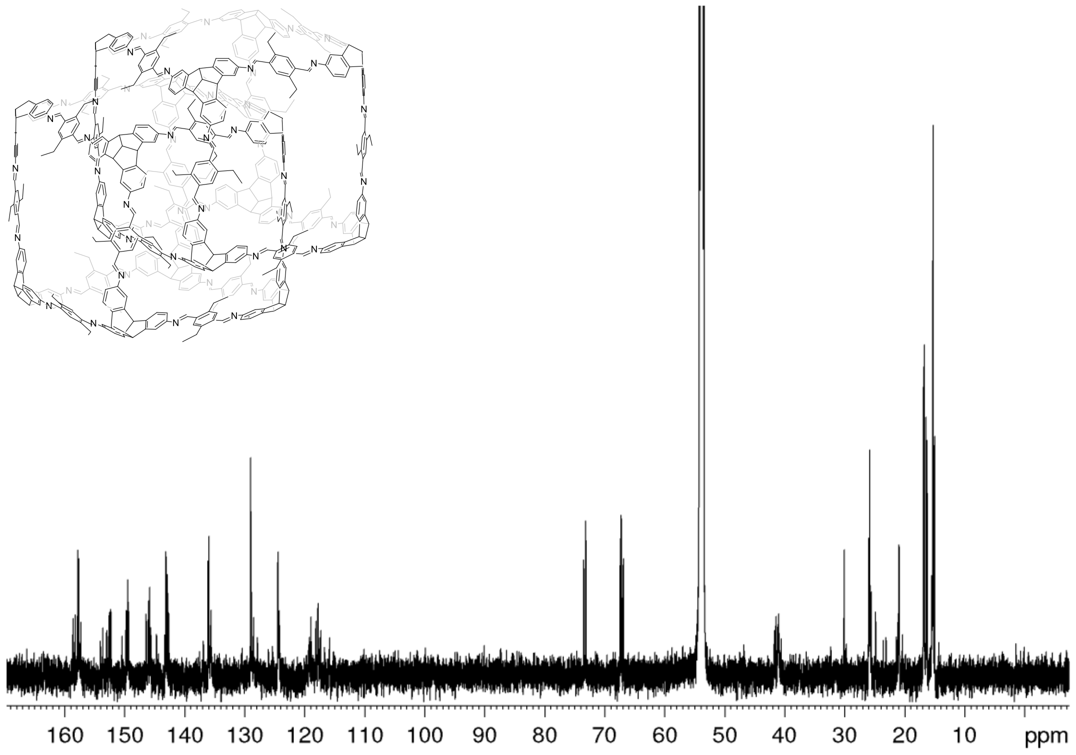

Figure 137. ^{13}C NMR (151 MHz, CD_2Cl_2) spectrum of **(Et-cube)$_2$**.

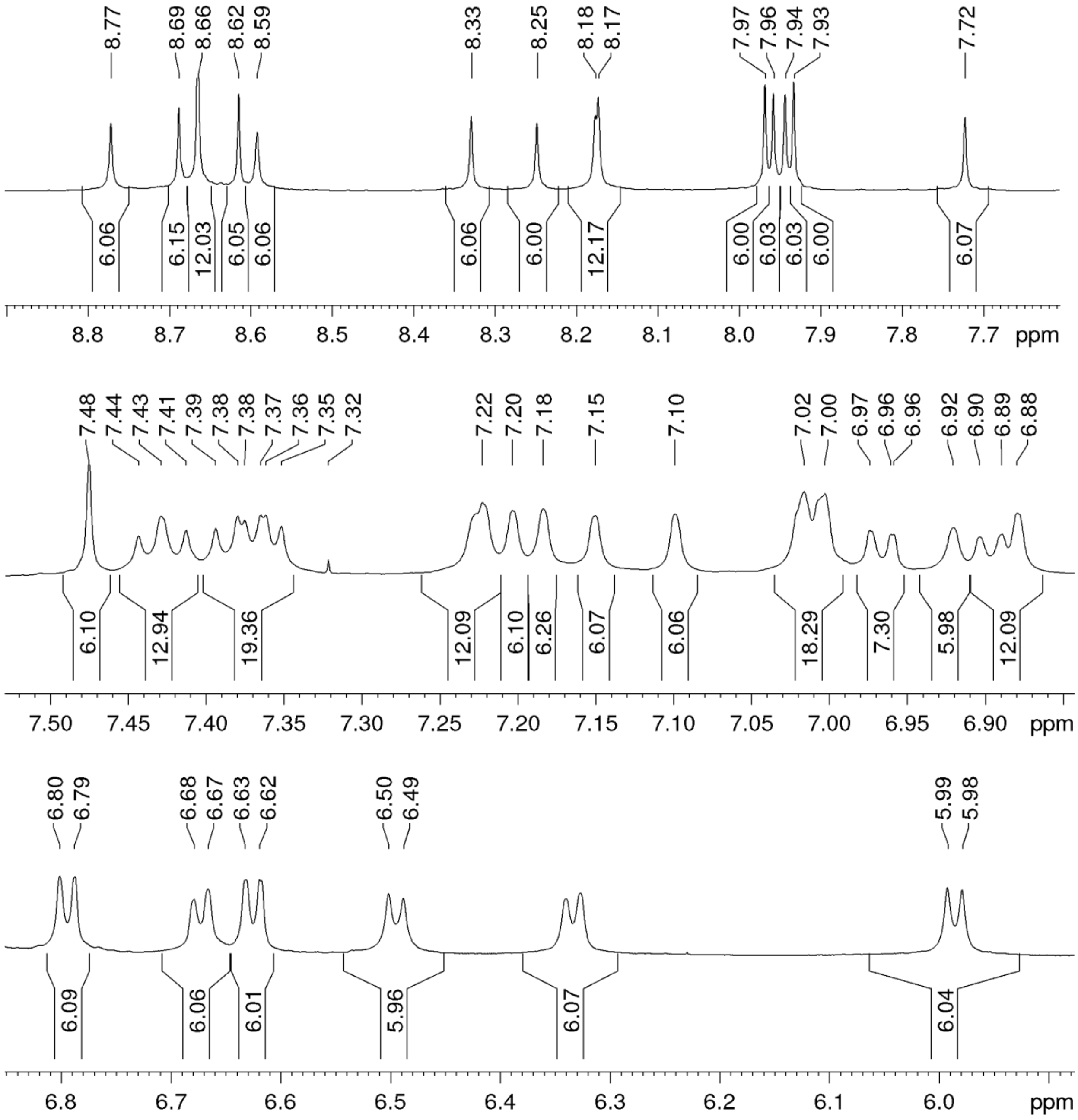

Figure 138. Expanded ^{1}H NMR (600 MHz, CD_2Cl_2) spectrum of **(Et-cube)$_2$**.

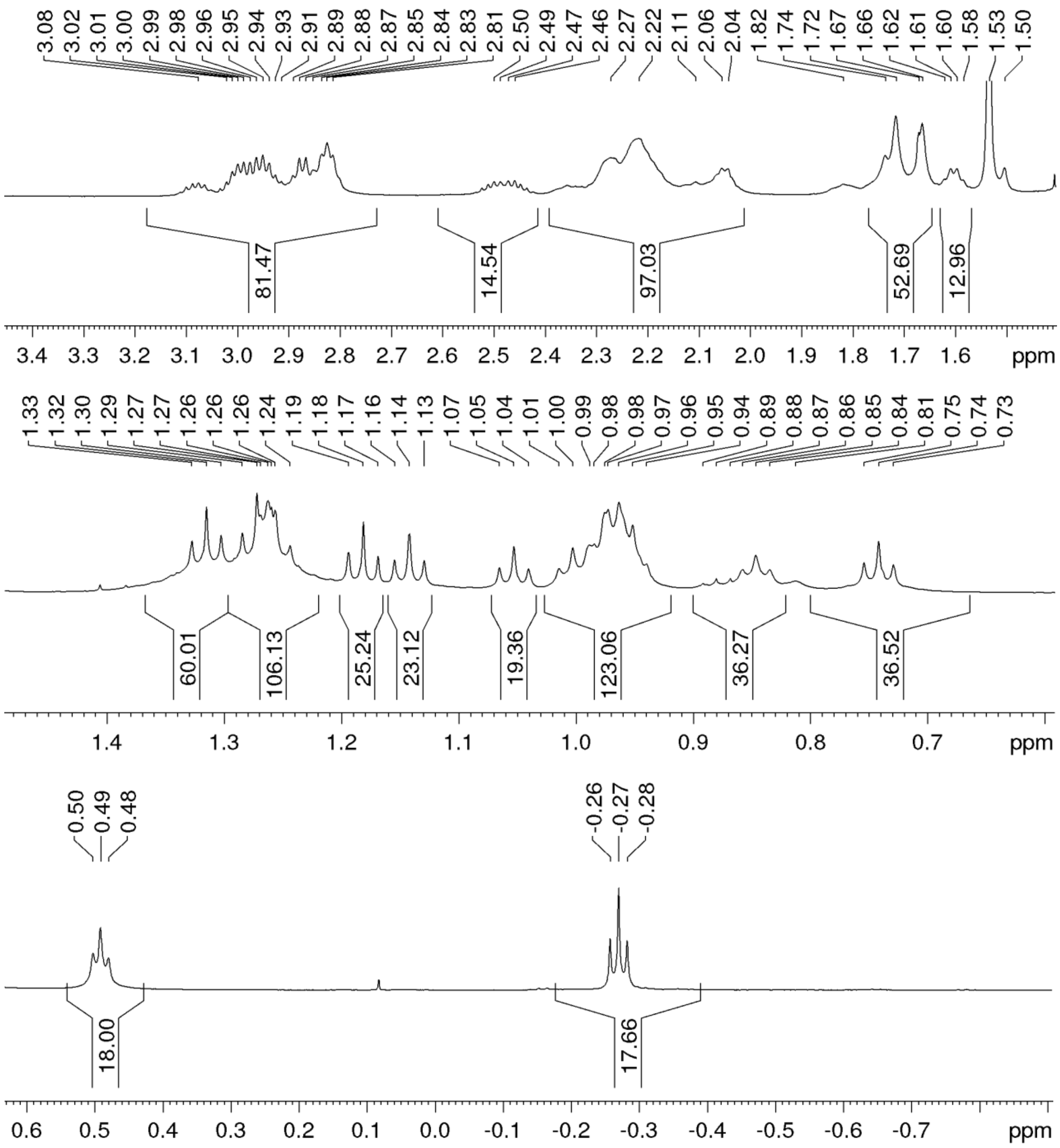

Figure 139. Expanded ^{1}H NMR (600 MHz, CD_2Cl_2) spectrum of **(Et-cube)$_2$**.

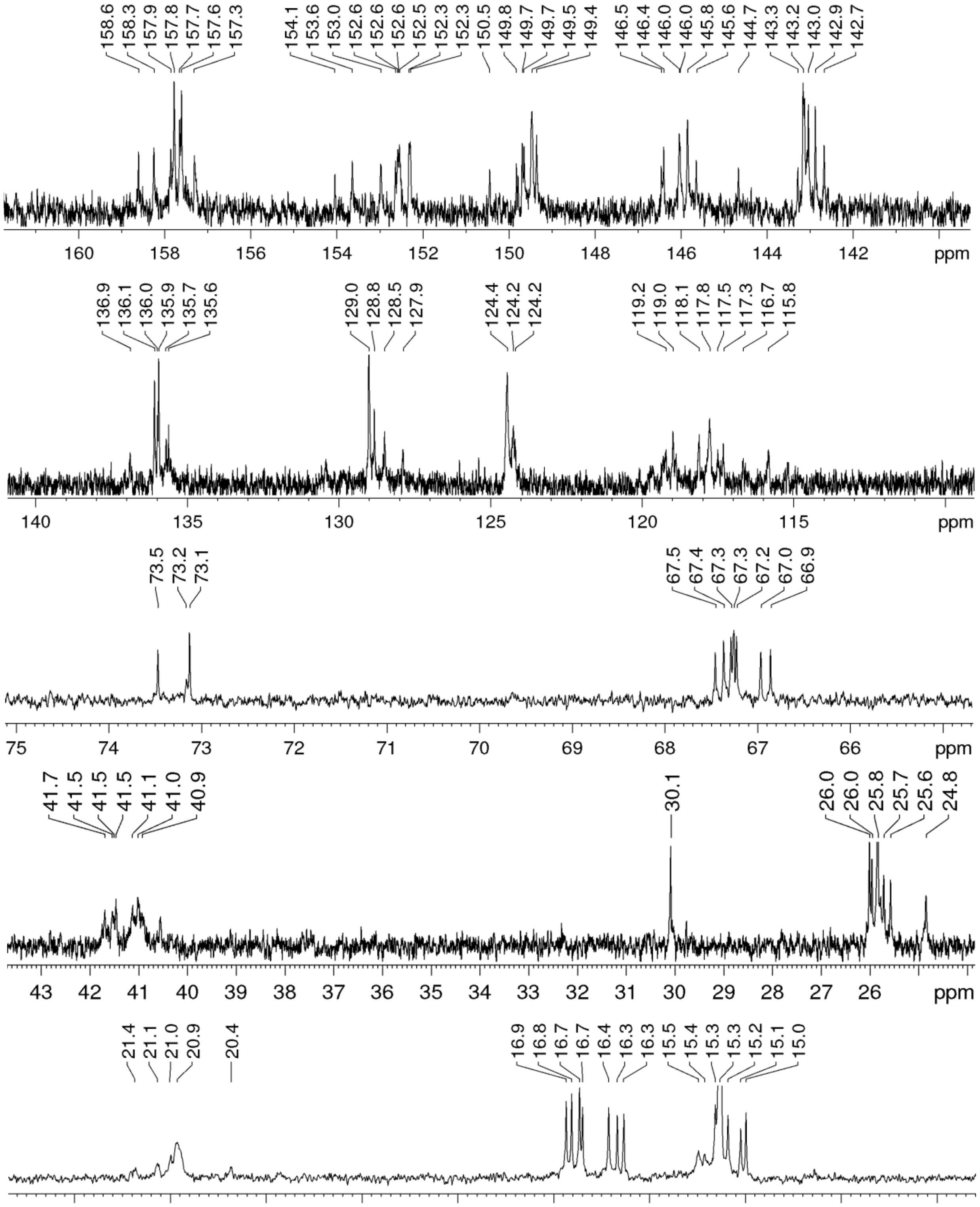

Figure 140. Expanded ^{13}C NMR (151 MHz, CD_2Cl_2) spectrum of **(Et-cube)$_2$**.

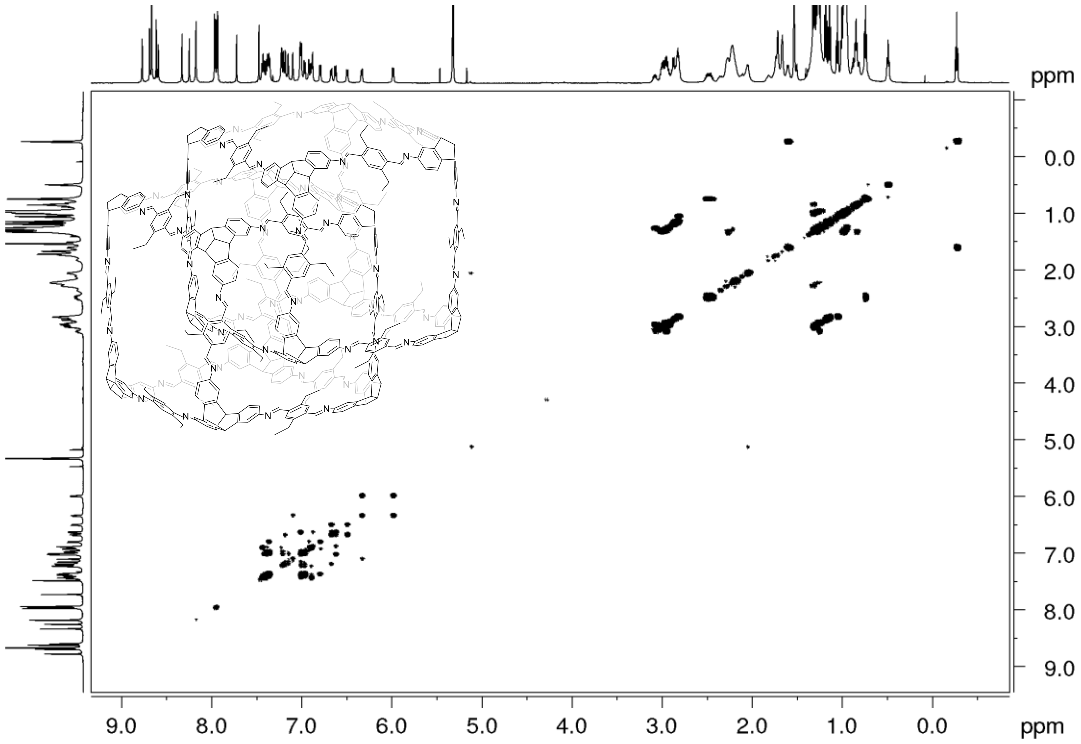

Figure 141. ^{1}H-^{1}H COSY NMR spectrum (600 MHz, CD_2Cl_2) of **(Et-cube)$_2$**.

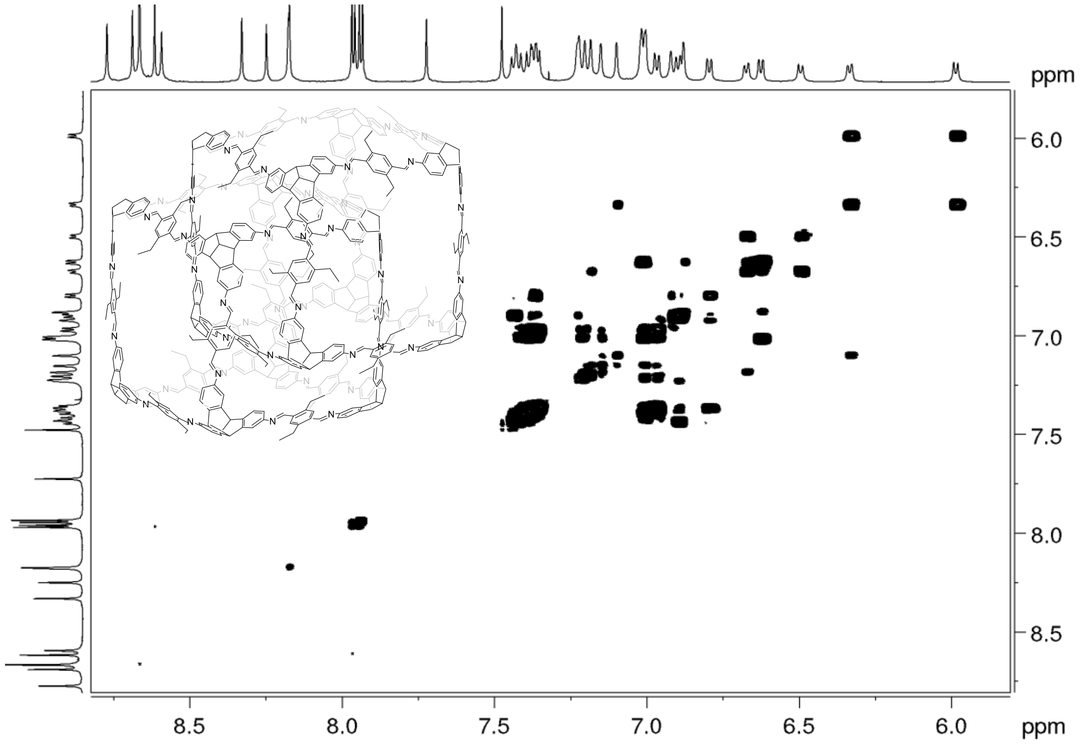

Figure 142. Partial ^{1}H-^{1}H COSY NMR spectrum (600 MHz, CD_2Cl_2) of **(Et-cube)$_2$**.

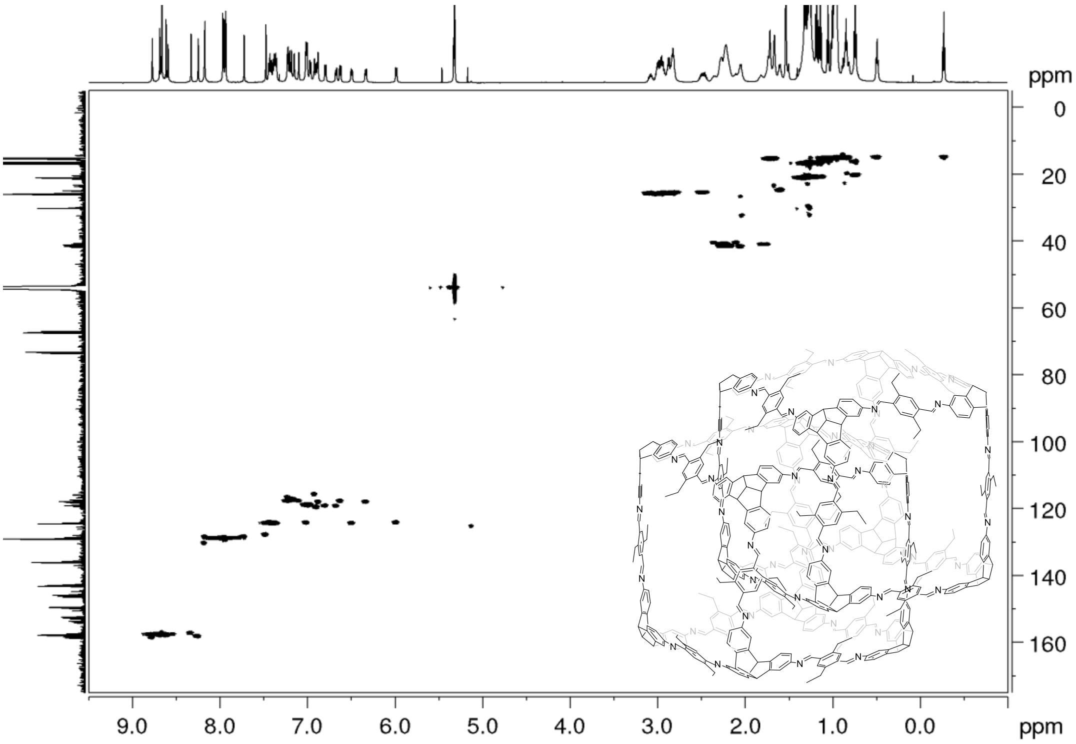

Figure 143. ^{1}H-^{13}C HSQC NMR (600 MHz and 151 MHz, CD_2Cl_2) spectrum of **(Et-cube)$_2$**.

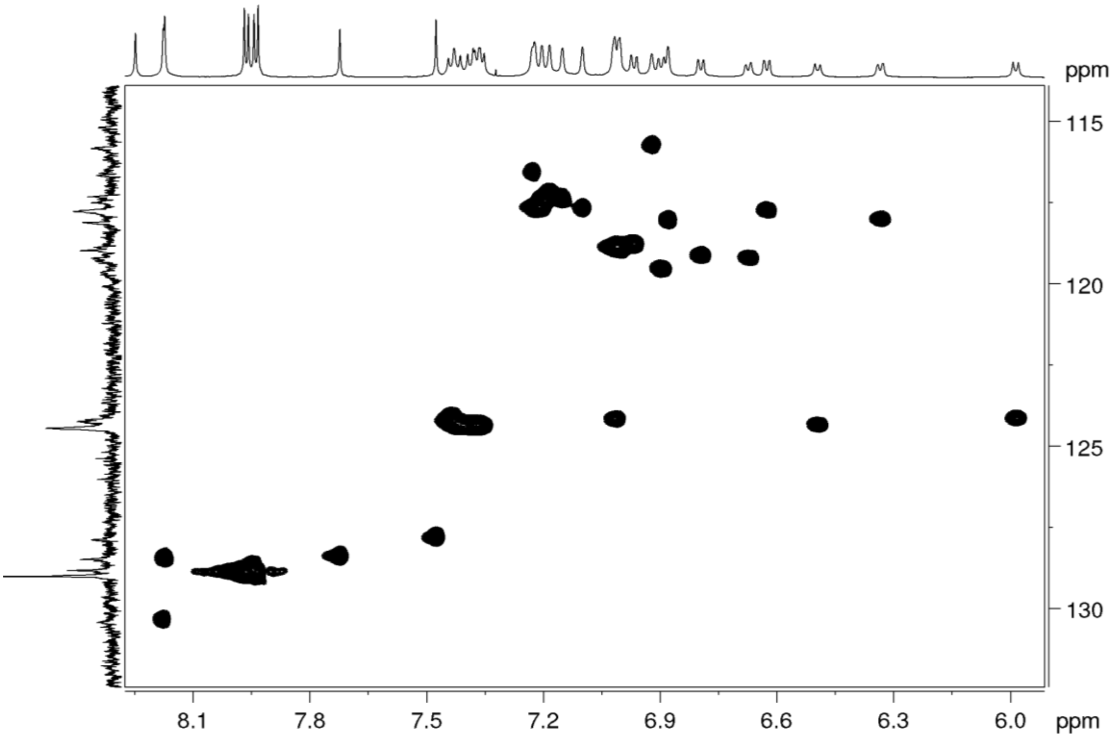

Figure 144. Partial ^{1}H-^{13}C HSQC NMR (600 MHz and 151 MHz, CD_2Cl_2) spectrum of **(Et-cube)$_2$**.

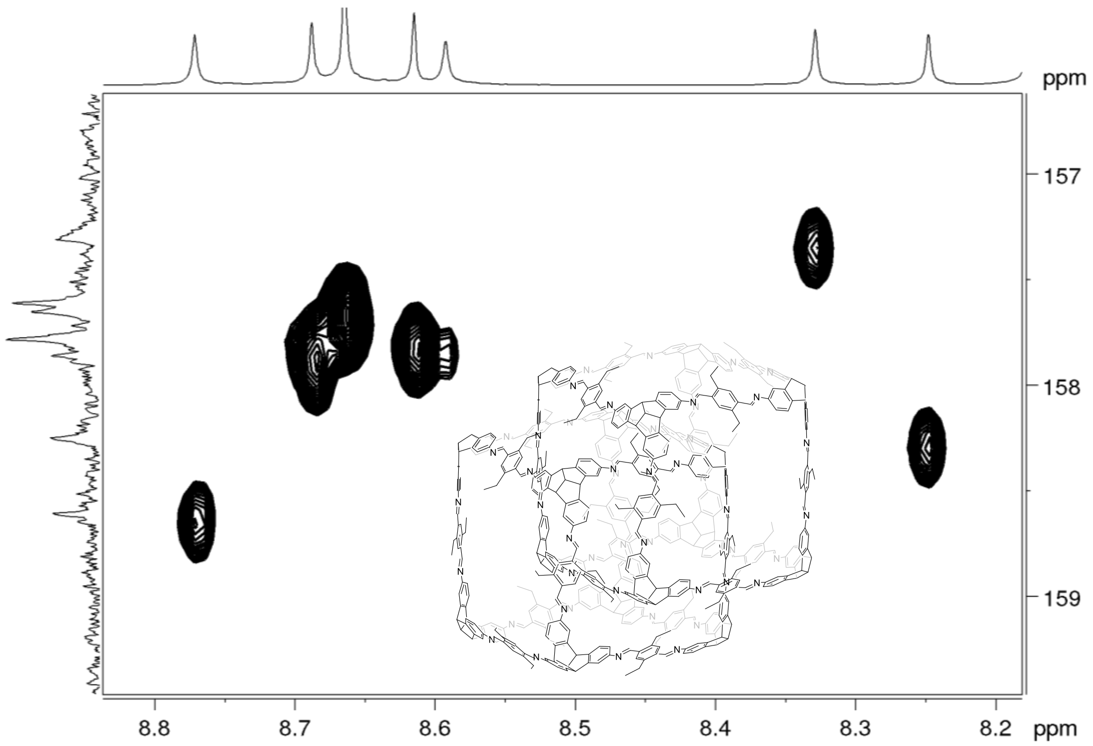

Figure 145. Partial ^{1}H-^{13}C HSQC NMR (600 MHz and 151 MHz, CD_2Cl_2) spectrum of **(Et-cube)$_2$**.

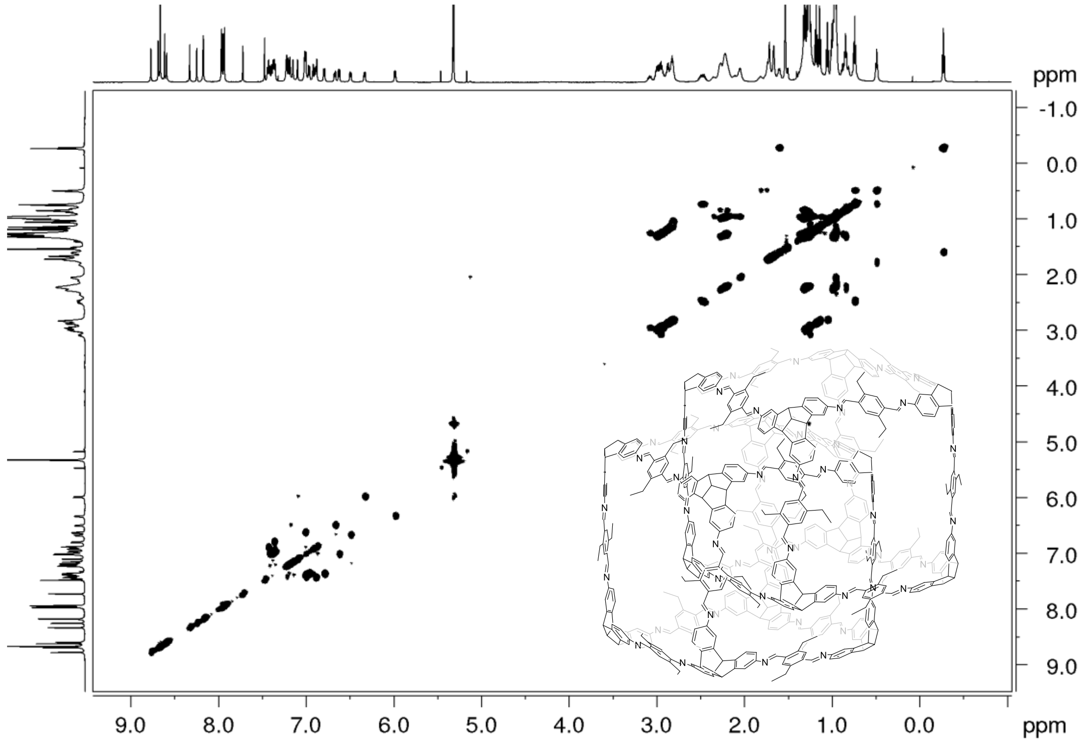

Figure 146. ^{1}H-^{1}H TOCSY NMR (600 MHz, CD_2Cl_2) spectrum of **(Et-cube)$_2$**.

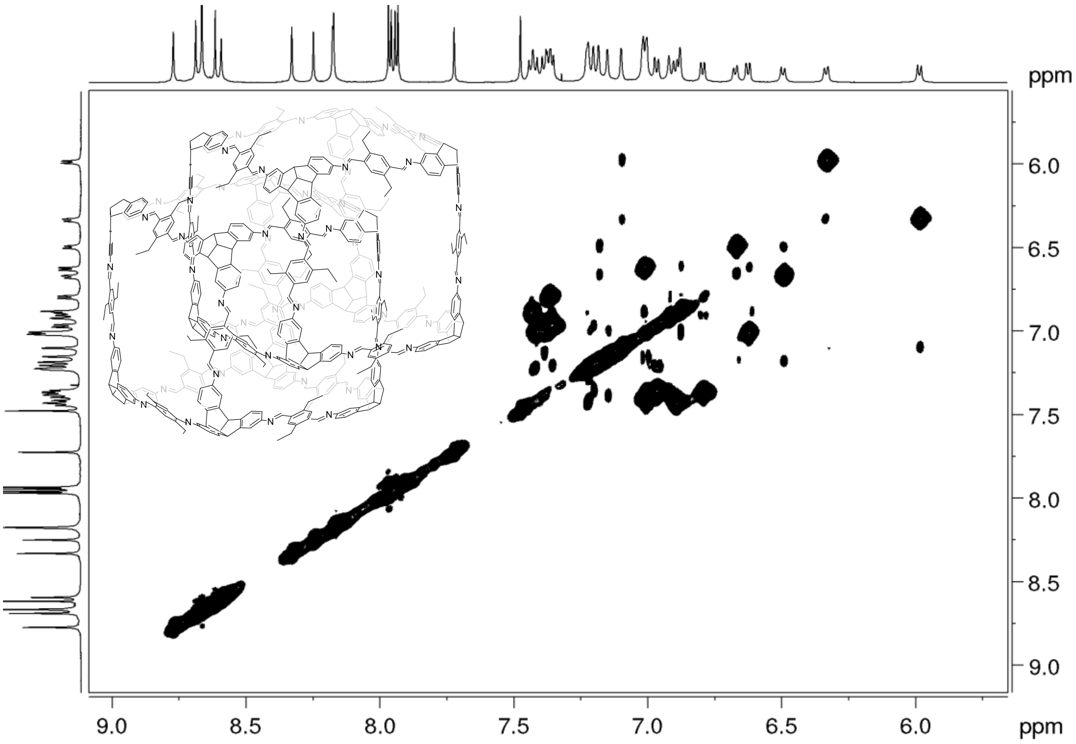

Figure 147. Partial ^{1}H-^{1}H TOCSY NMR (600 MHz, CD_2Cl_2) spectrum of **(Et-cube)$_2$**.

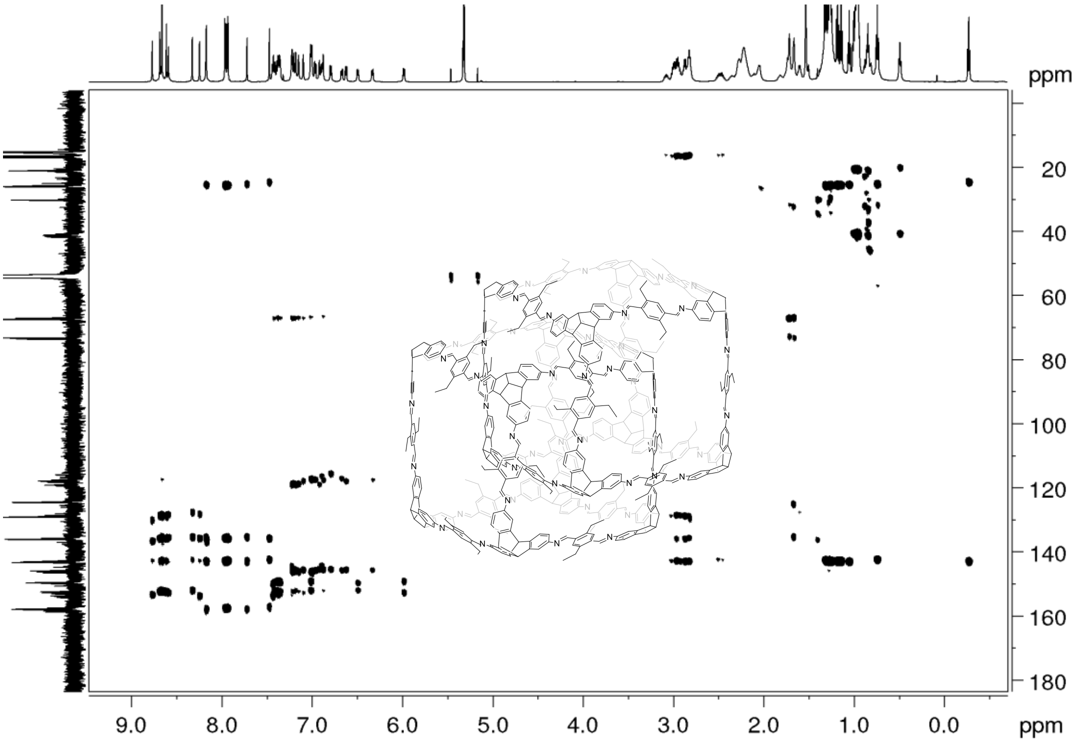

Figure 148. ^{1}H-^{13}C HMBC NMR (600 MHz and 151 MHz, CD_2Cl_2) spectrum of **(Et-cube)$_2$**.

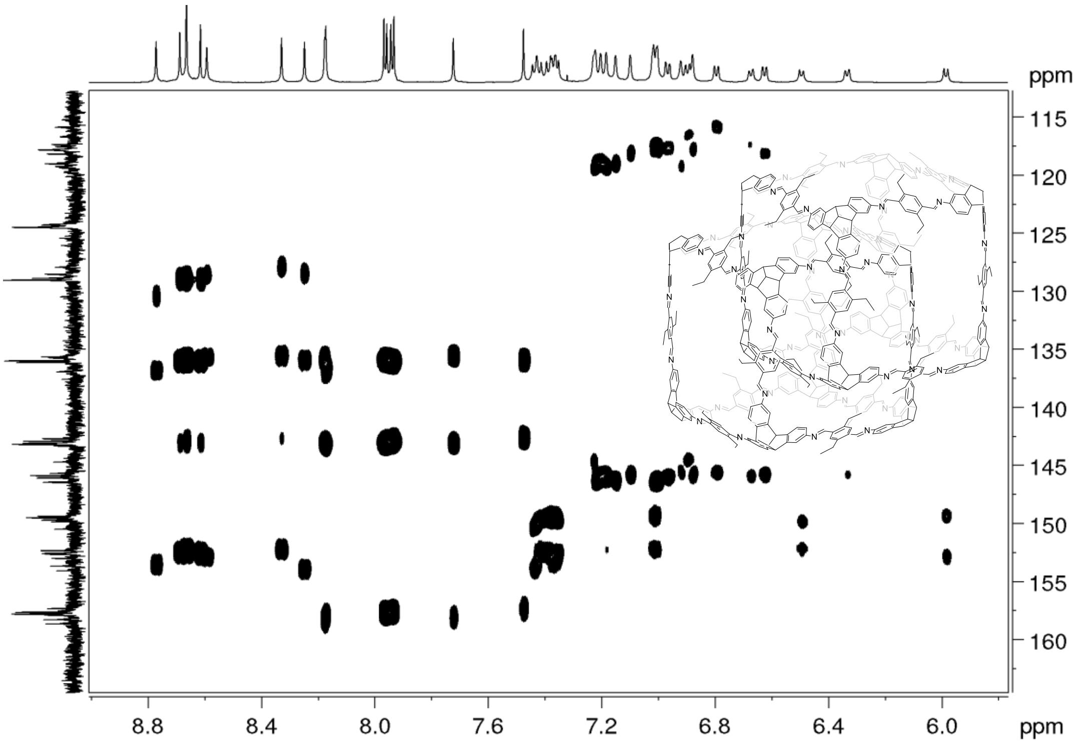

Figure 149. Partial ^{1}H-^{13}C HMBC NMR (600 MHz and 151 MHz, CD_2Cl_2) spectrum of **(Et-cube)$_2$**.

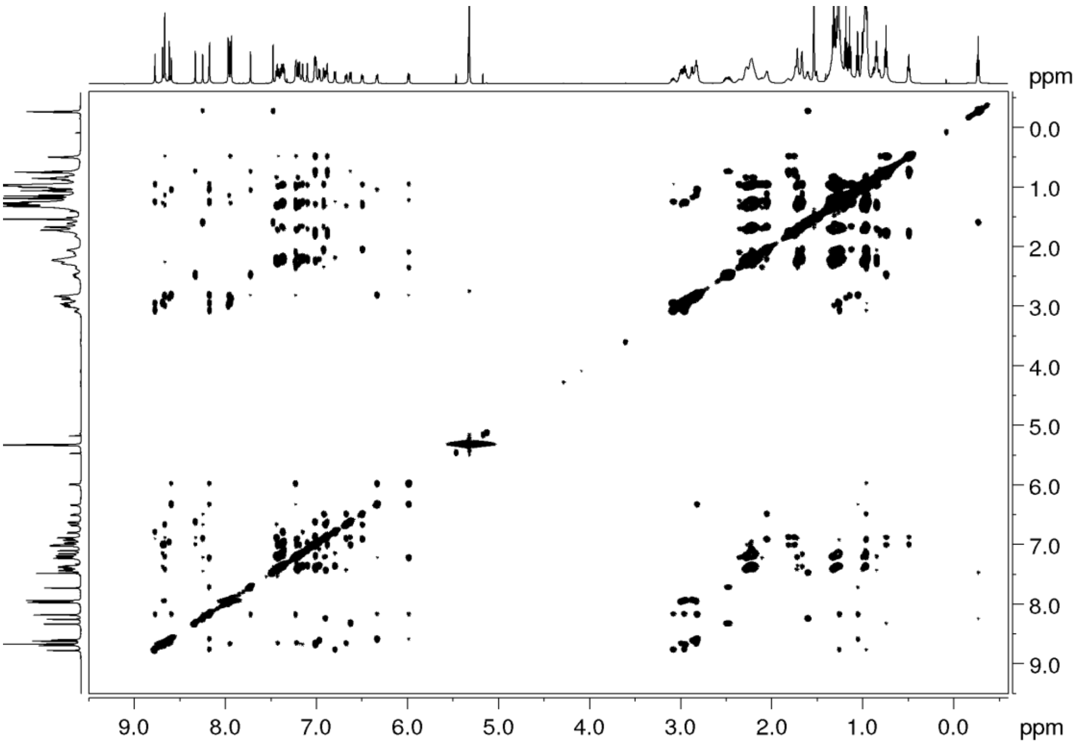

Figure 150. ^{1}H-^{1}H NOESY NMR (600 MHz, CD_2Cl_2) spectrum of **(Et-cube)$_2$**.

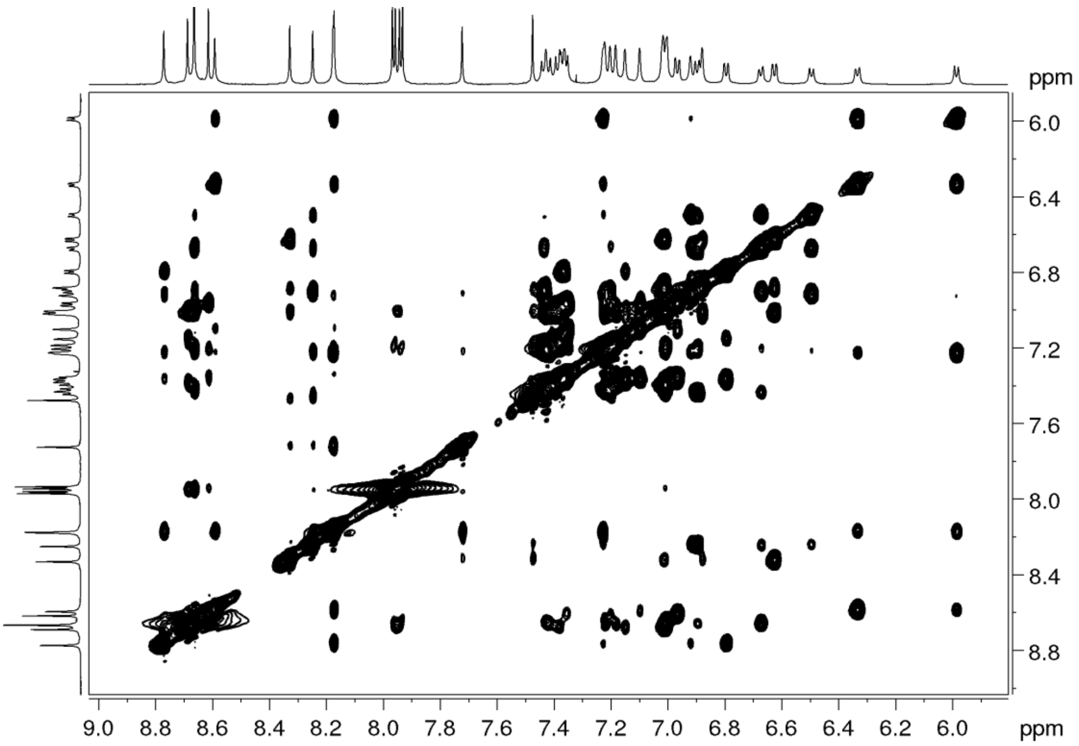

Figure 151. Partial ^{1}H-^{1}H NOESY NMR (600 MHz, CD_2Cl_2) spectrum of **$(Et\text{-}cube)_2$**.

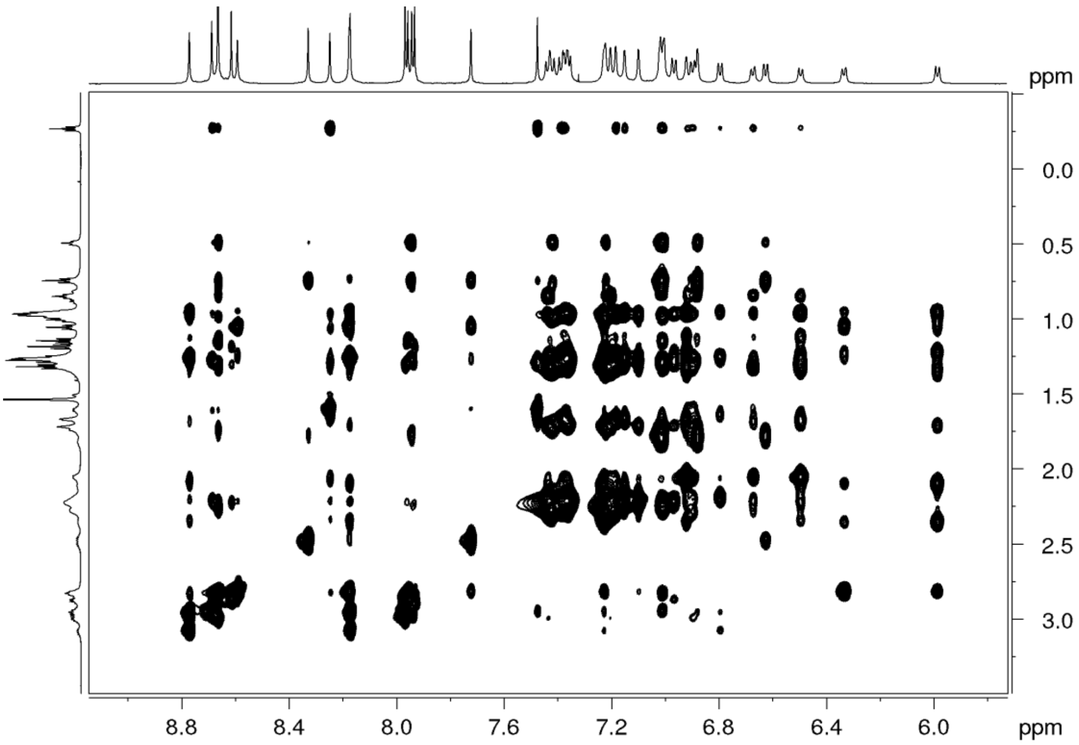

Figure 152. Partial ^{1}H-^{1}H NOESY NMR (600 MHz, CD_2Cl_2) spectrum of **$(Et\text{-}cube)_2$**.

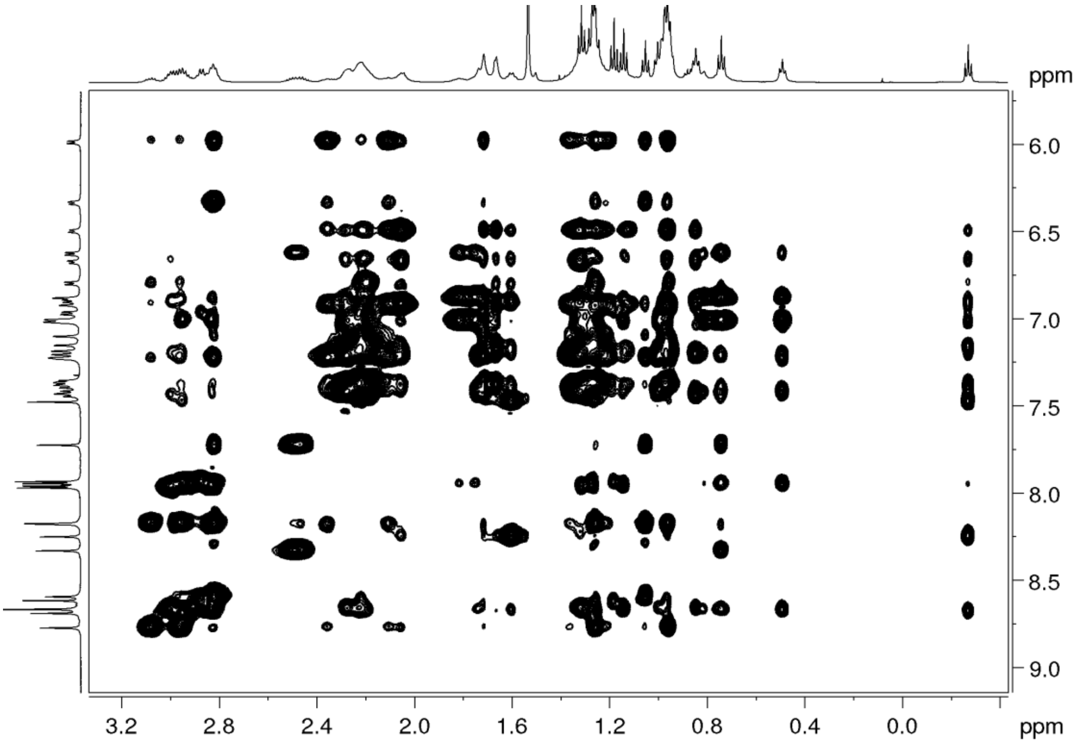

Figure 153. Partial ^{1}H-^{1}H NOESY NMR (600 MHz, CD_2Cl_2) spectrum of **(Et-cube)$_2$**.

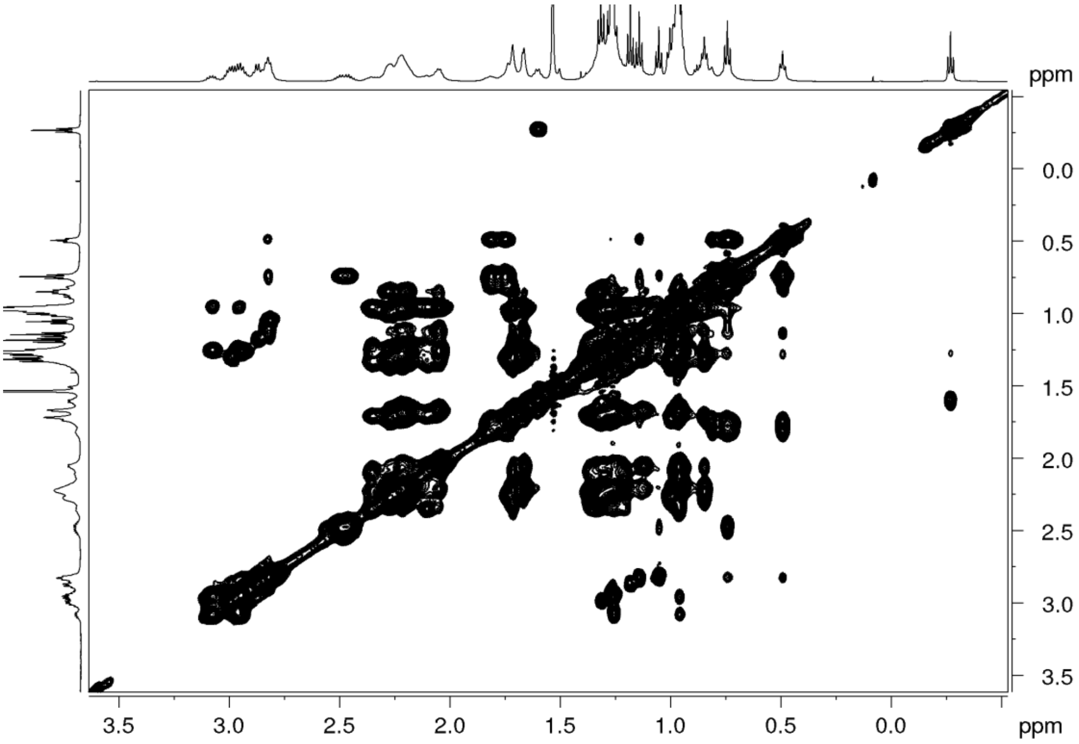

Figure 154. Partial ^{1}H-^{1}H NOESY NMR (600 MHz, CD_2Cl_2) spectrum of **(Et-cube)$_2$**.

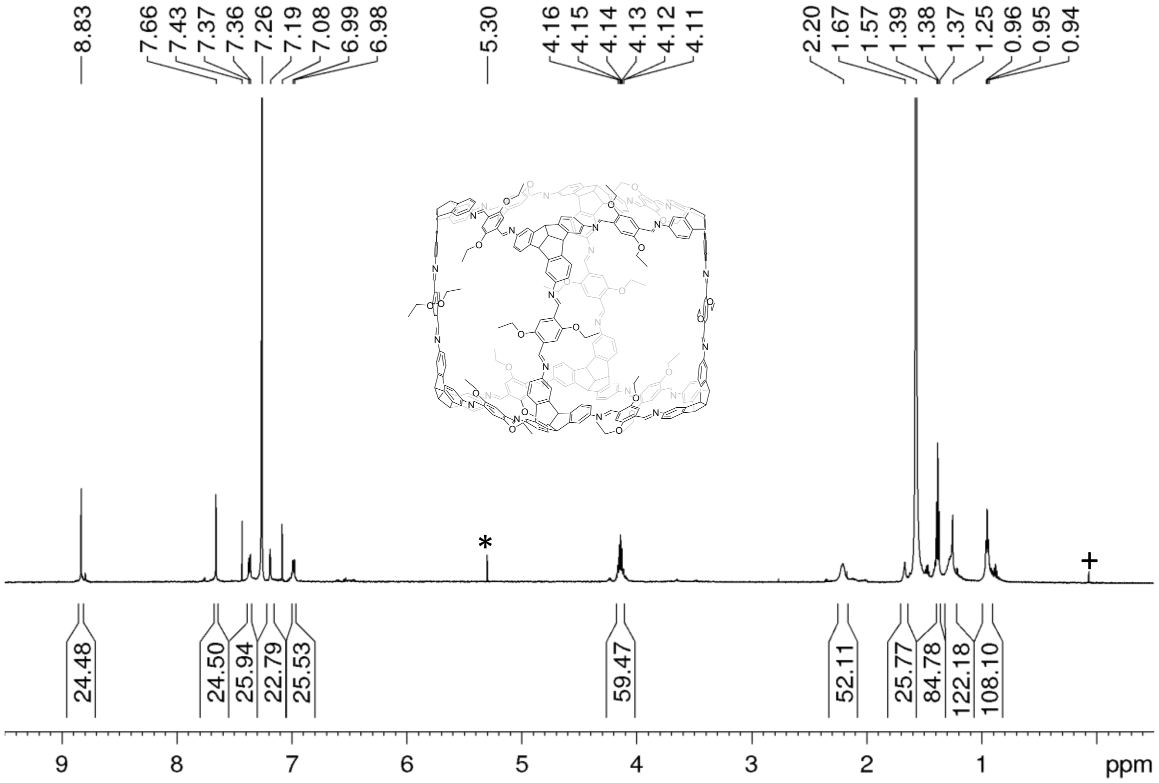

Figure 155. ^{1}H NMR (600 MHz, $CDCl_3$) spectrum of **OEt-cube**. (*DCM, + silicone grease)

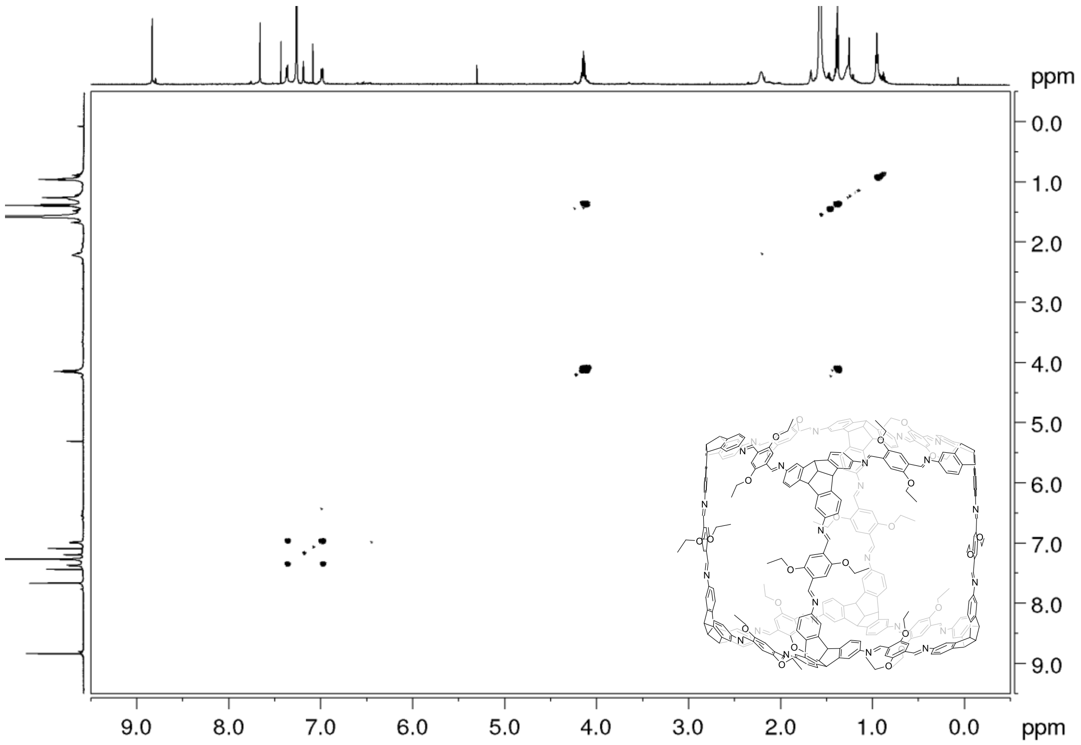

Figure 156. ^{1}H-^{1}H COSY NMR spectrum (600 MHz, $CDCl_3$) of **OEt-cube**.

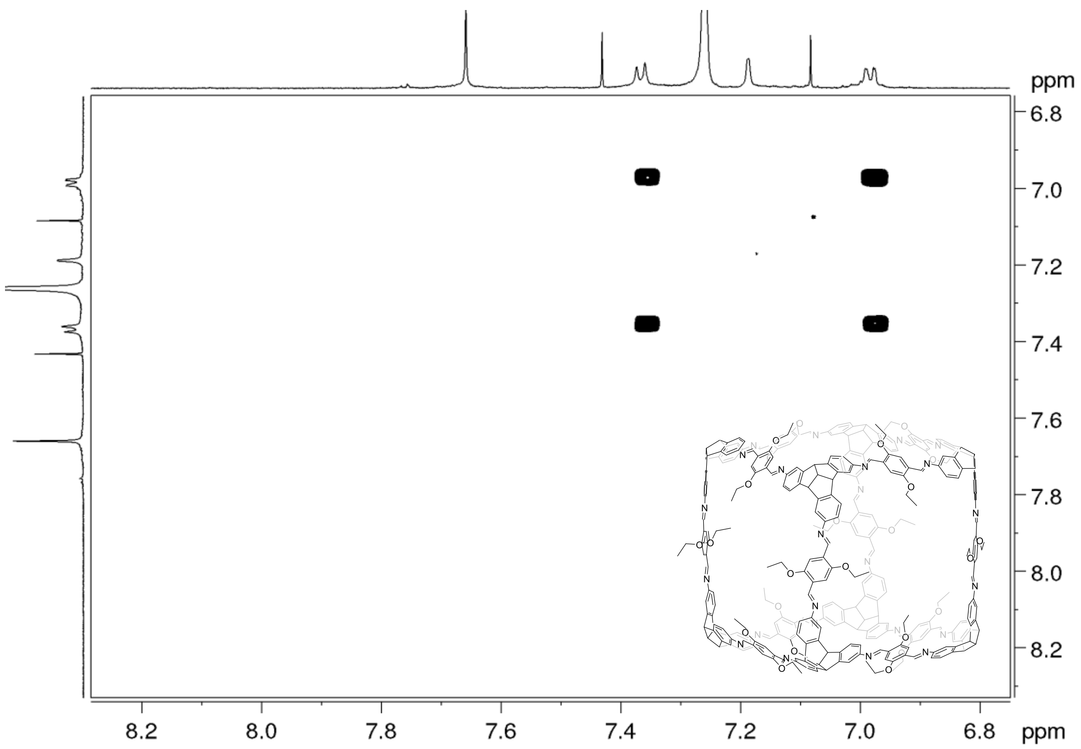

Figure 157. Partial ^{1}H-^{1}H COSY NMR spectrum (600 MHz, $CDCl_3$) of **OEt-cube**.

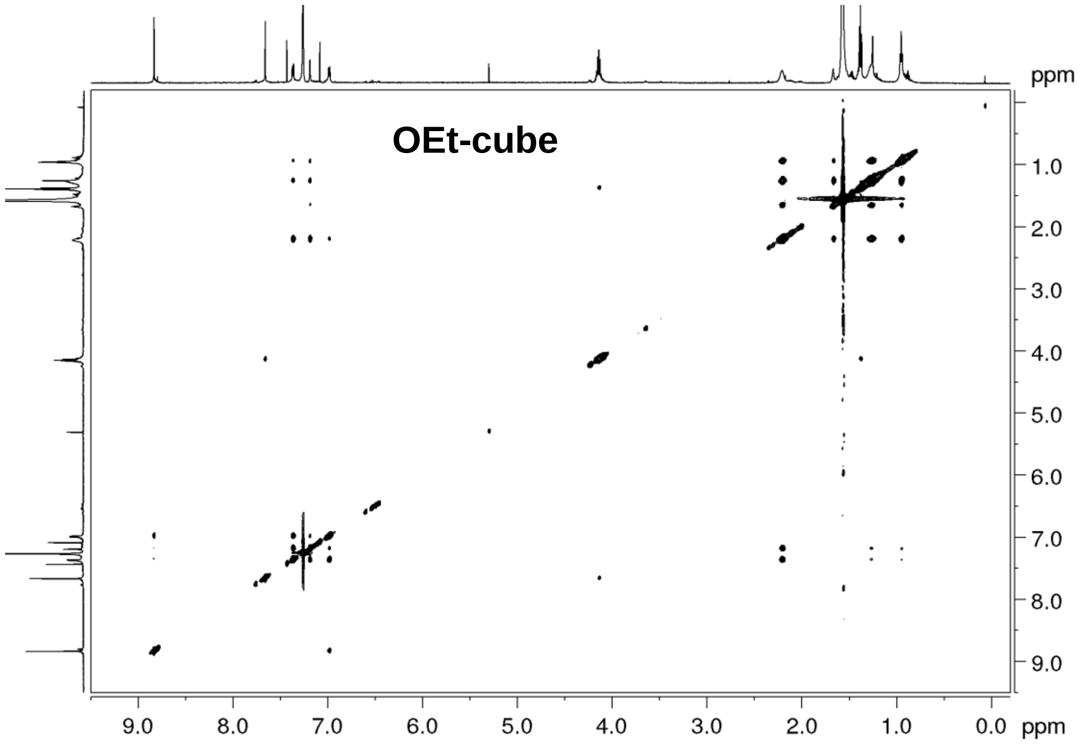

Figure 158. ^{1}H-^{1}H NOESY NMR (600 MHz, $CDCl_3$) spectrum of **OEt-cube**

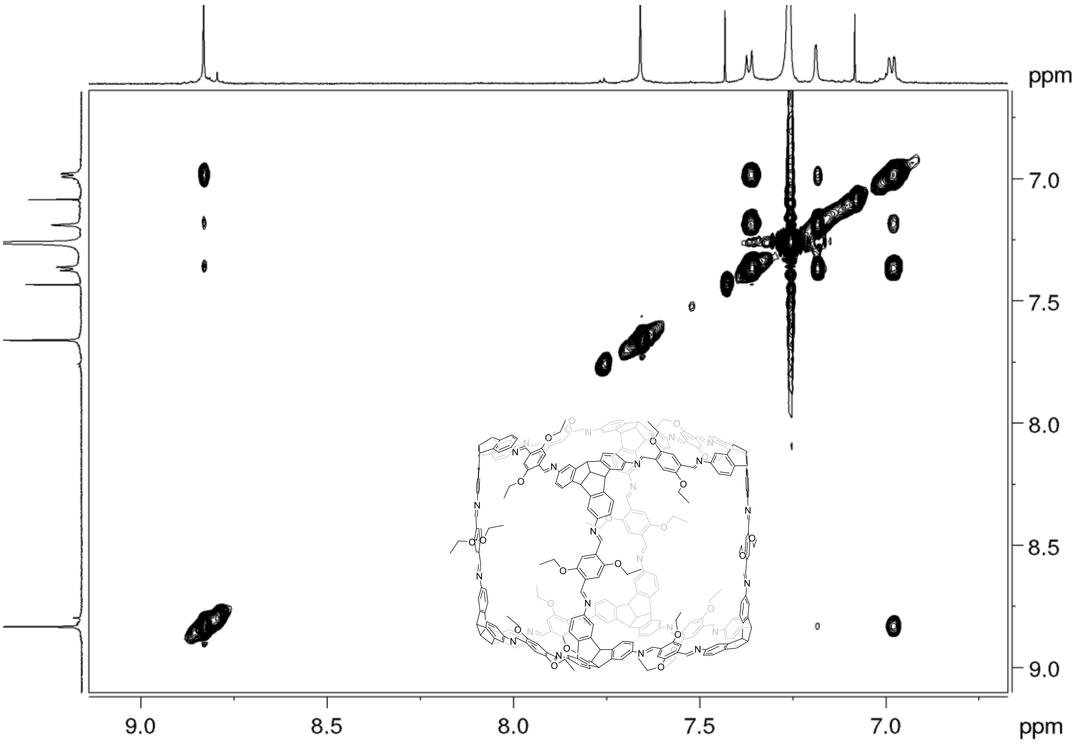

Figure 159. Partial ^1H-^1H NOESY NMR (600 MHz, $CDCl_3$) spectrum of **OEt-cube**.

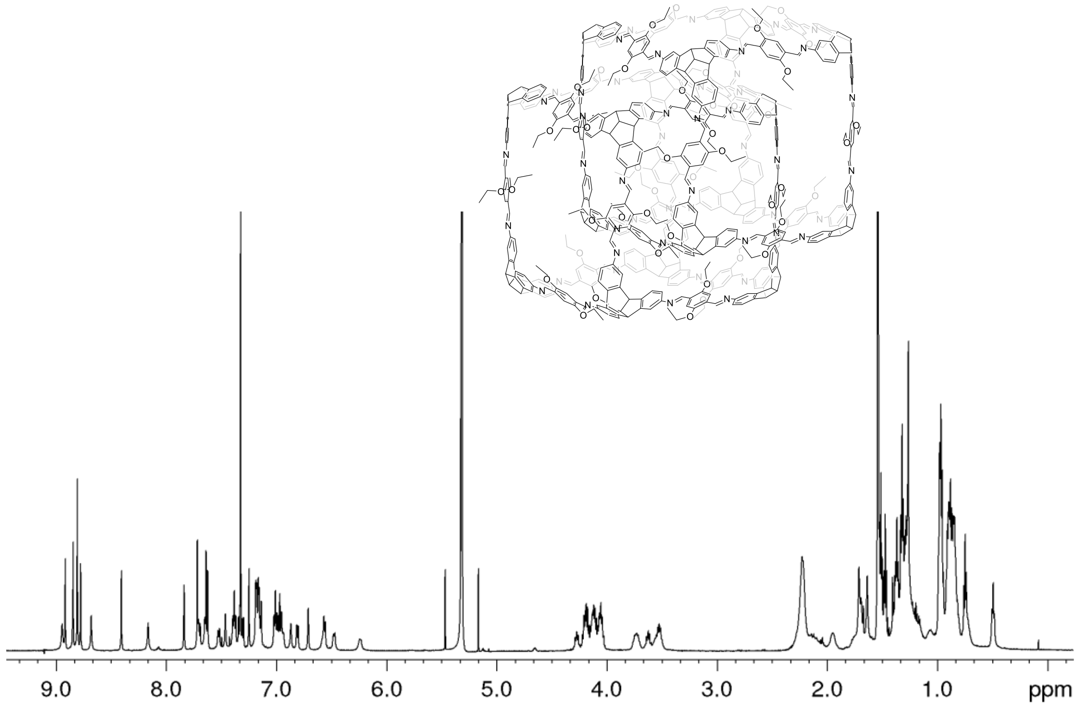

Figure 160. ^{1}H NMR (600 MHz, CD_2Cl_2) spectrum of **(OEt-cube)$_2$**.

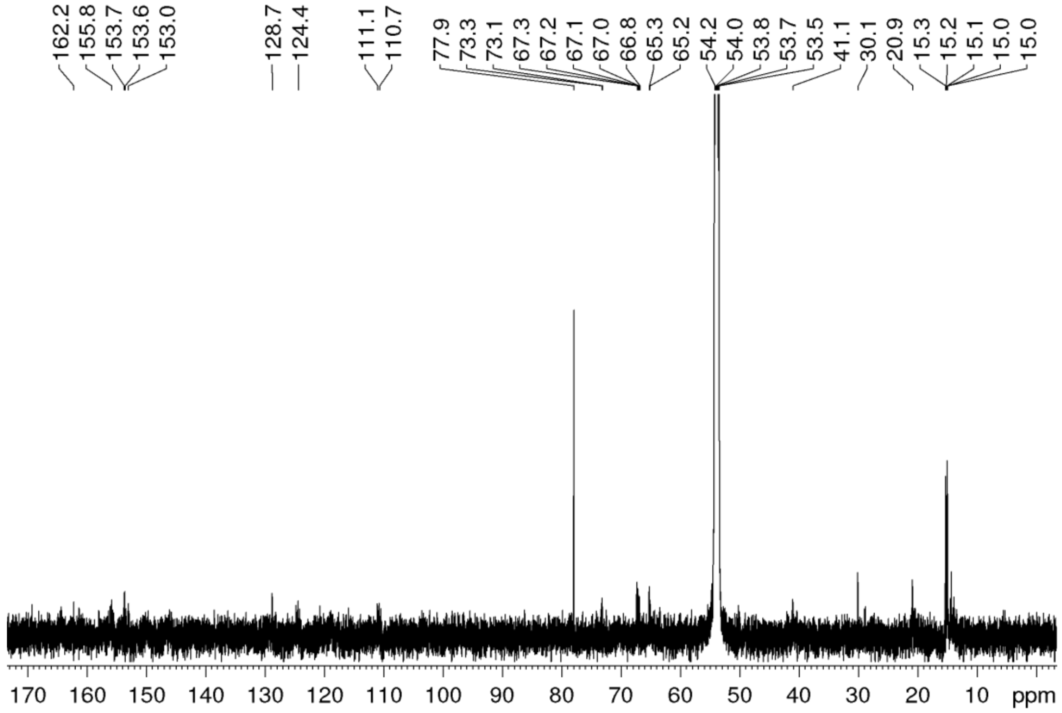

Figure 161. ^{13}C NMR (151 MHz, CD_2Cl_2) spectrum of compound **(OEt-cube)$_2$**. (* H-grease).

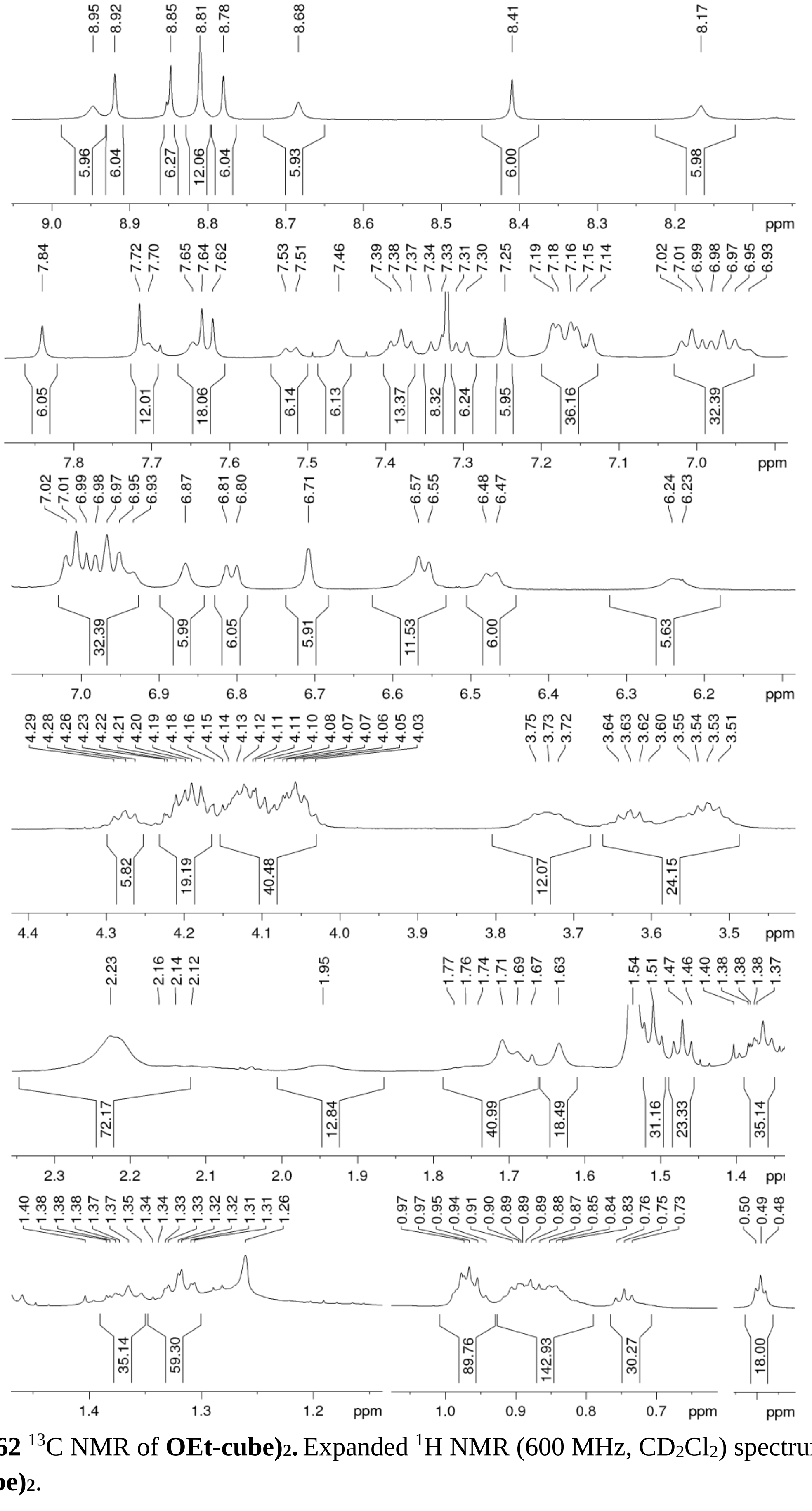

Figure 162 ^{13}C NMR of **OEt-cube)$_2$.** Expanded ^{1}H NMR (600 MHz, CD_2Cl_2) spectrum of **(OEt-cube)$_2$**.

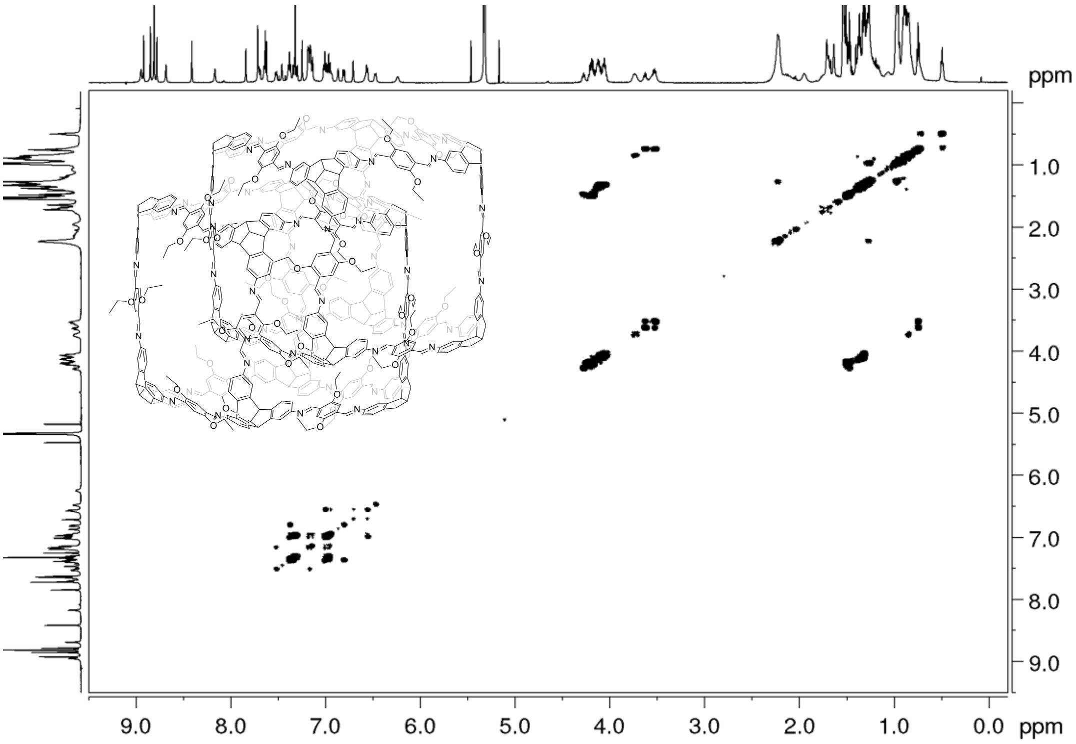

Figure 163. ^{1}H-^{1}H COSY NMR spectrum (600 MHz, CD_2Cl_2) of **(OEt-cube)$_2$**.

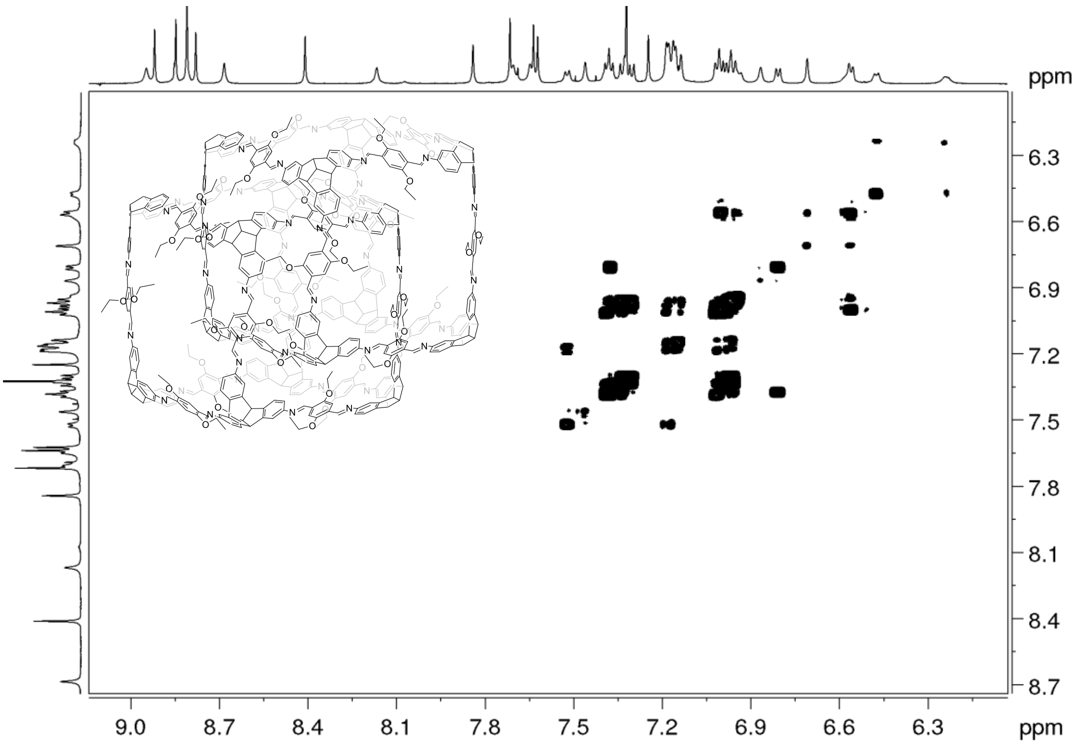

Figure 164. Partial ^{1}H-^{1}H COSY NMR spectrum (600 MHz, CD_2Cl_2) of **(OEt-cube)$_2$**.

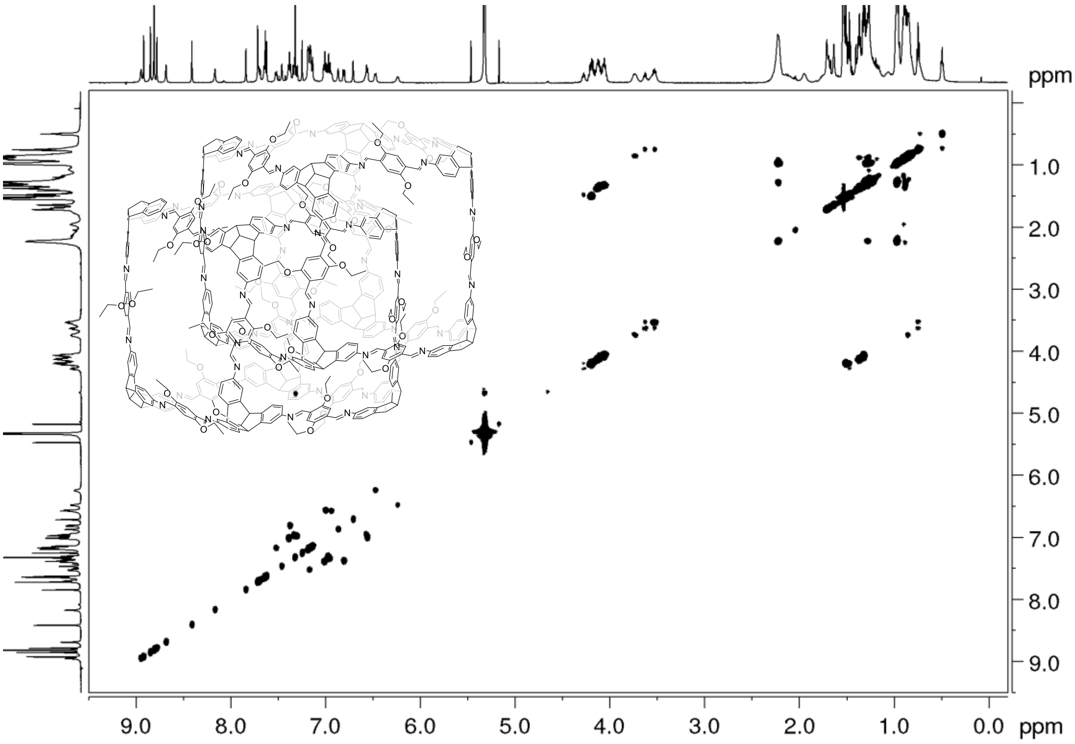

Figure 165. ^{1}H-^{1}H TOCSY NMR (600 MHz, CD_2Cl_2) spectrum of **(OEt-cube)$_2$**.

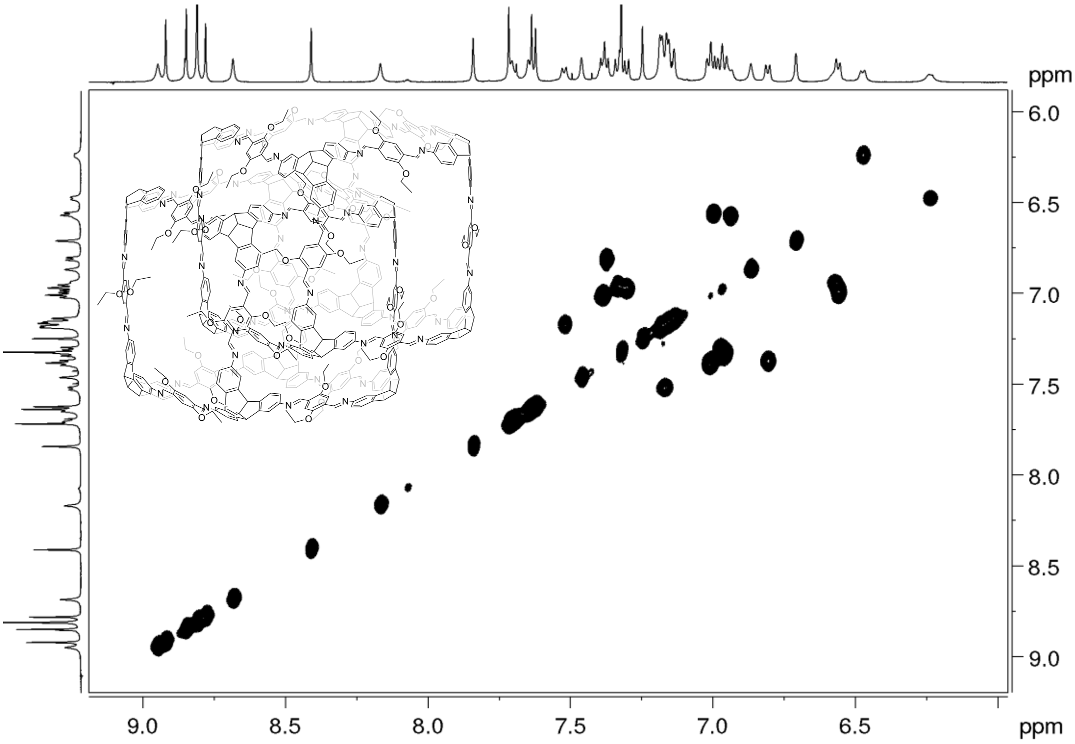

Figure 166. Partial ^{1}H-^{1}H TOCSY NMR (600 MHz, CD_2Cl_2) spectrum of **(OEt-cube)$_2$**.

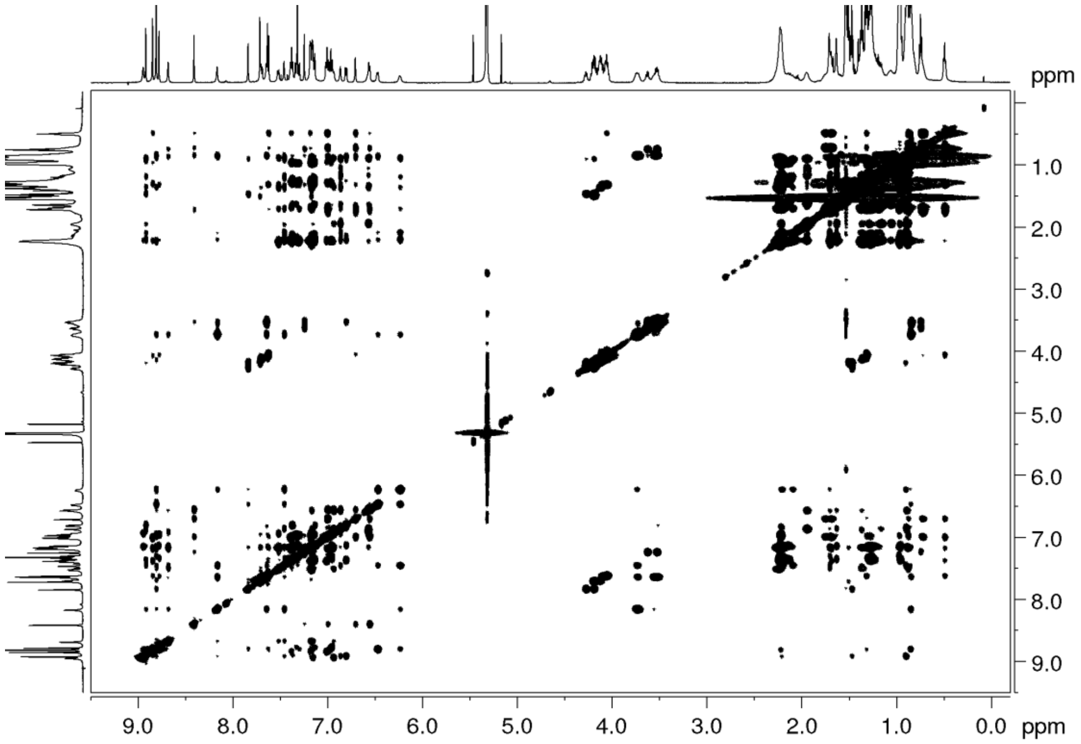

Figure 167. ^{1}H-^{1}H NOESY NMR (600 MHz, CD_2Cl_2) spectrum of **(OEt-cube)$_2$**.

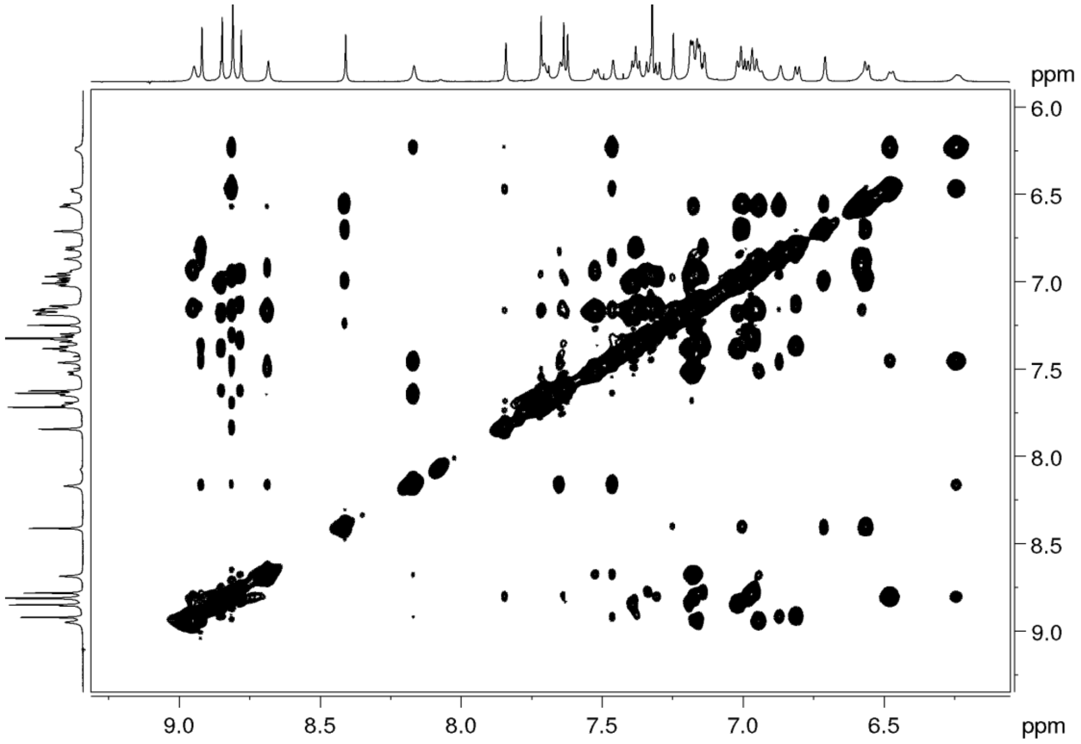

Figure 168. Partial ^{1}H-^{1}H NOESY NMR (600 MHz, CD_2Cl_2) spectrum of **(OEt-cube)$_2$**.

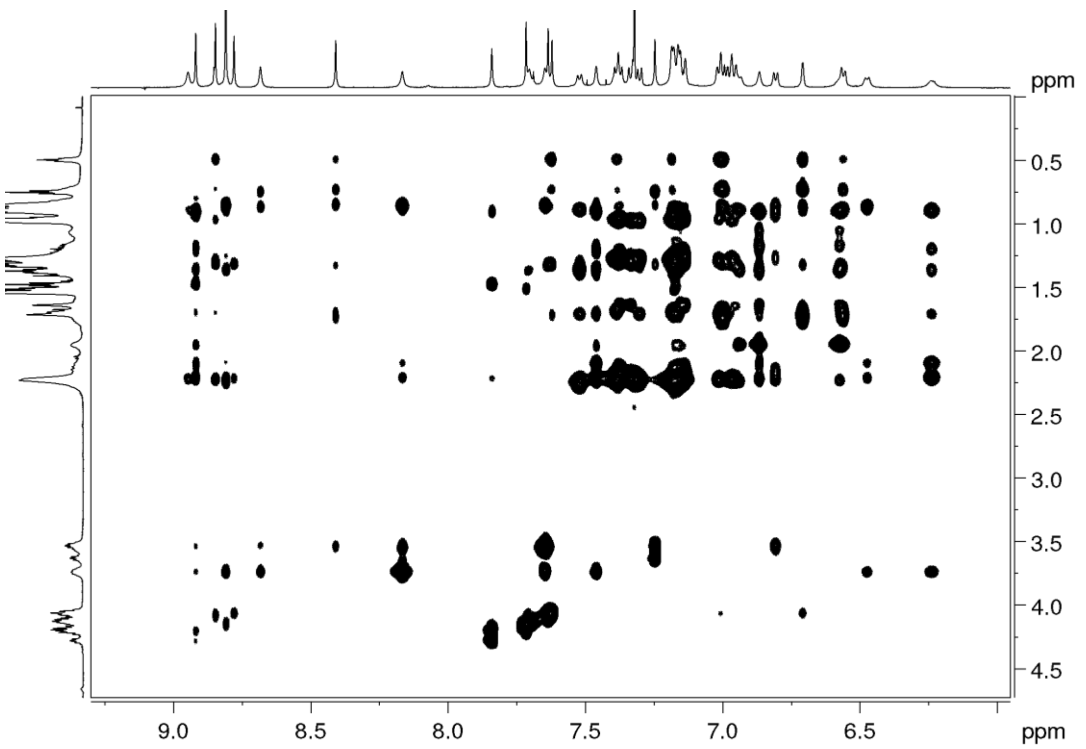

Figure 169. Partial ^1H-^1H NOESY NMR (600 MHz, CD_2Cl_2) spectrum of **(OEt-cube)$_2$**.

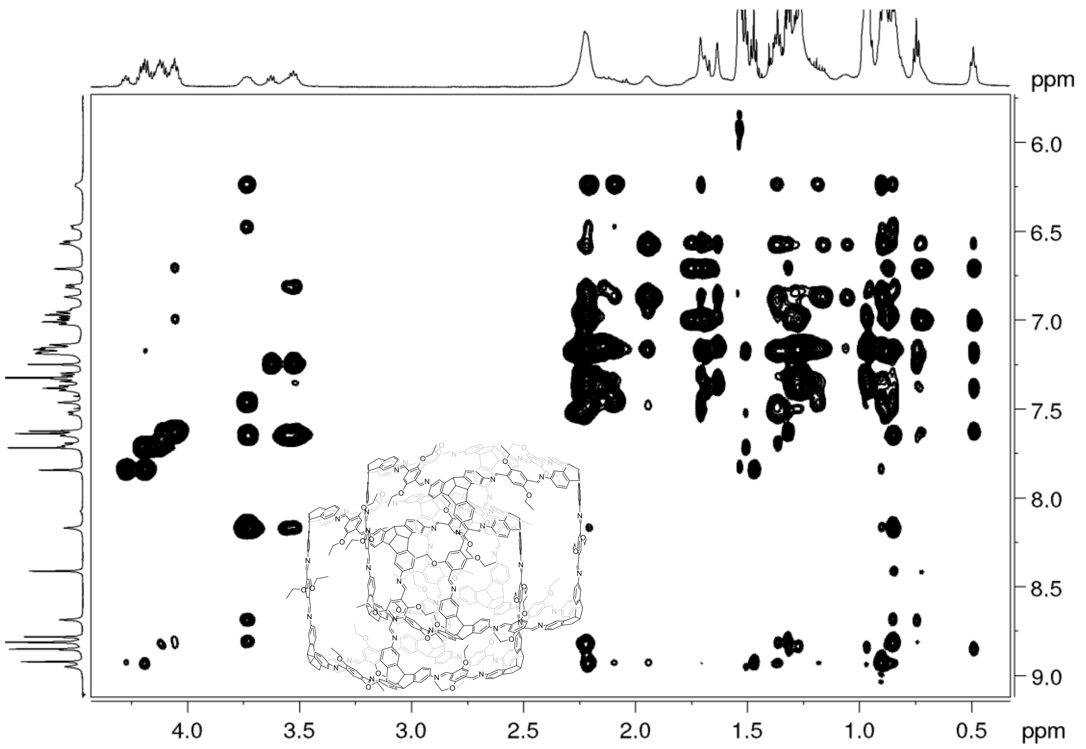

Figure 170. Partial ^1H-^1H NOESY NMR (600 MHz, CD_2Cl_2) spectrum of **(OEt-cube)$_2$**.

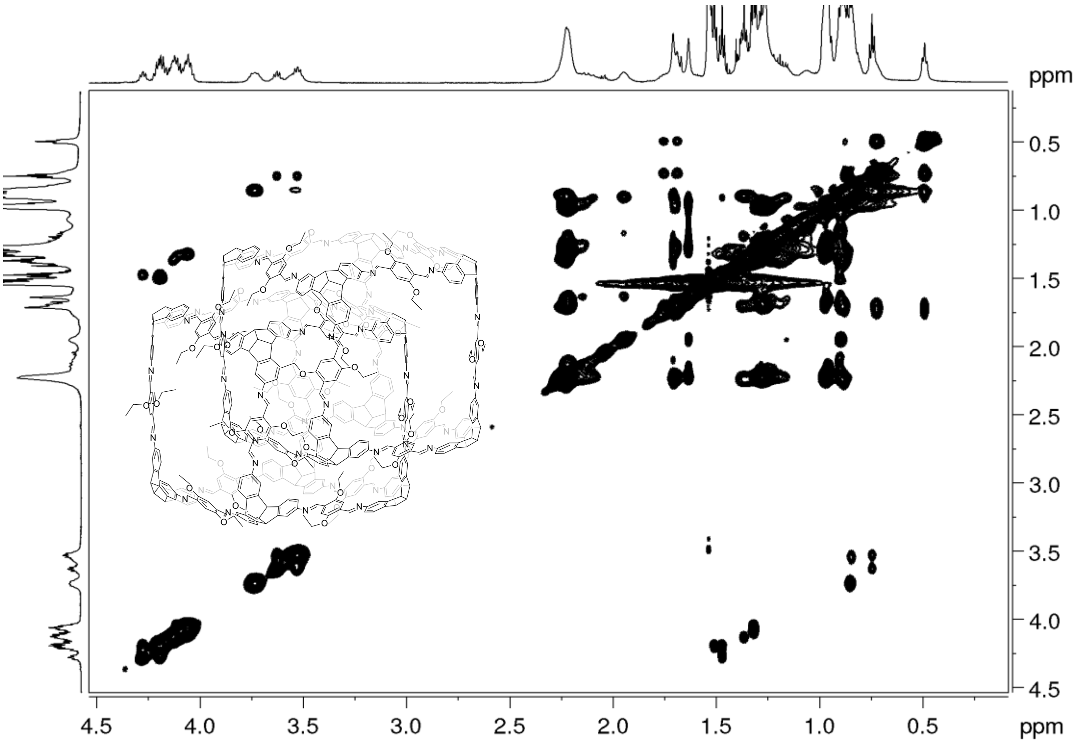

Figure 171. Partial ^1H-^1H NOESY NMR (600 MHz, CD_2Cl_2) spectrum of **(OEt-cube)$_2$**.

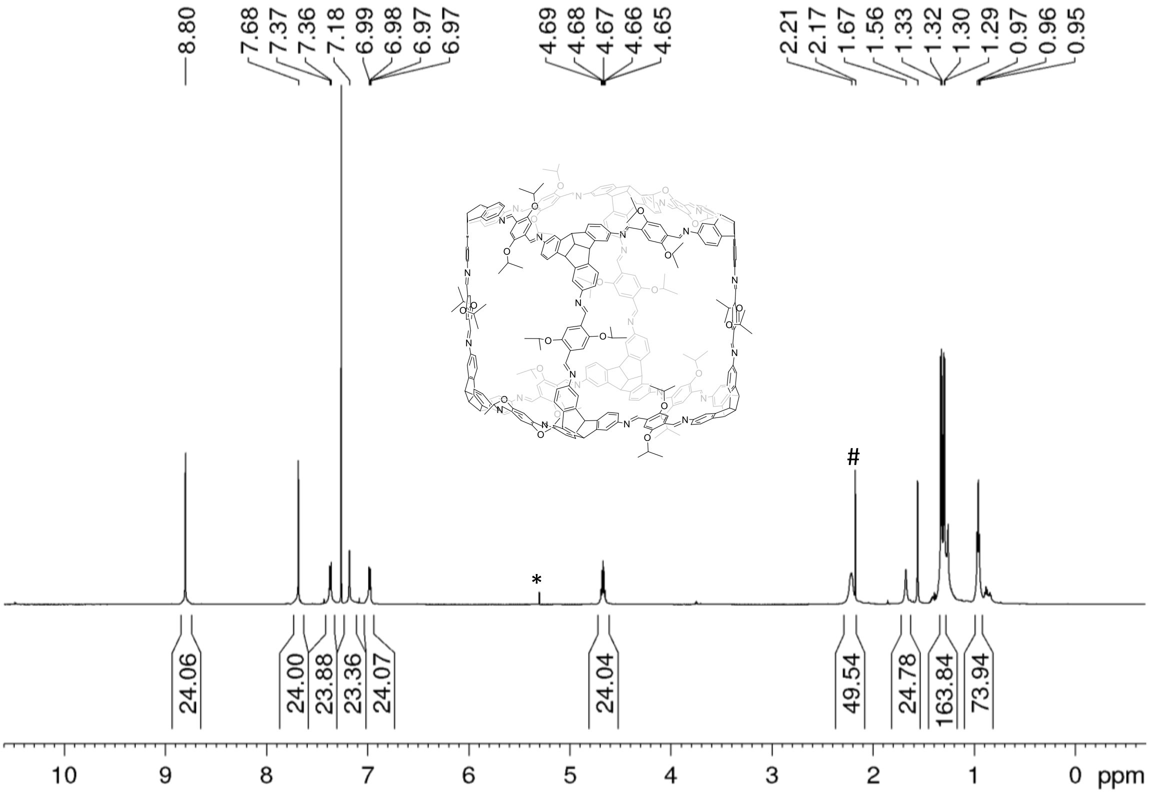

Figure 172. ^{1}H NMR (600 MHz, $CDCl_3$) spectrum of **O^{i}Pr-cube**. (*DCM, #acetone)

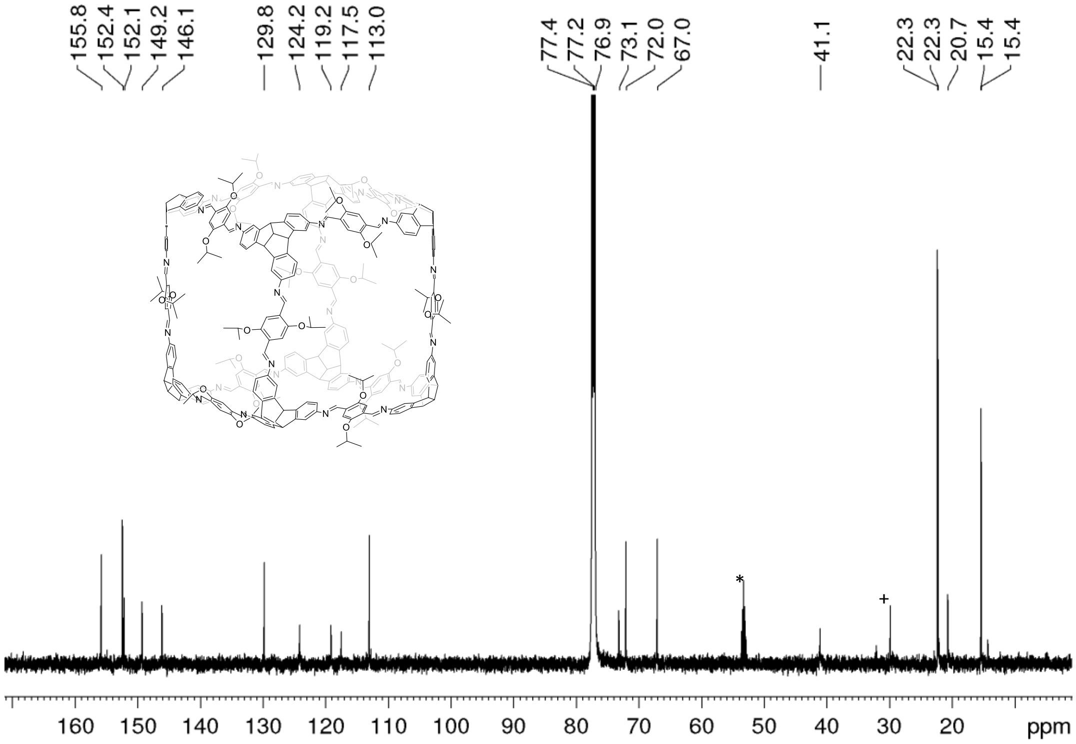

Figure 173. ^{13}C NMR (151 MHz, CD_2Cl_2) spectrum of **O^{i}Pr-cube**. (*CD_2Cl_2, ^{+}H grease)

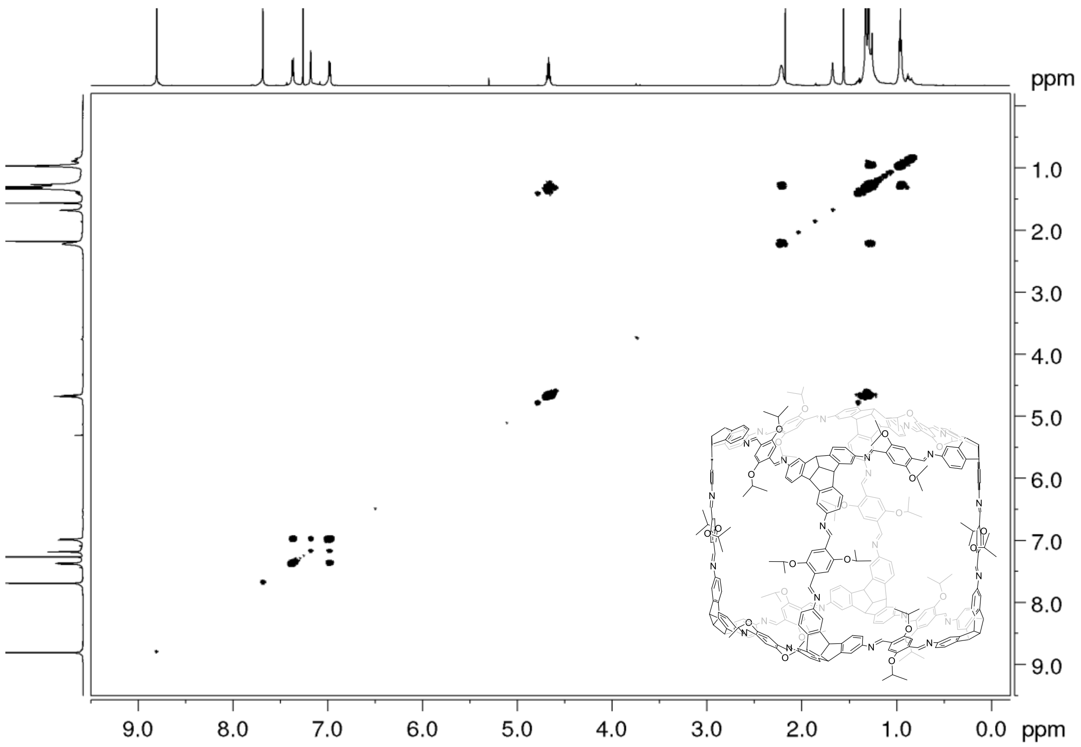

Figure 174. ^{1}H-^{1}H COSY NMR (600 MHz, $CDCl_3$) spectrum **O^{i}Pr-cube**.

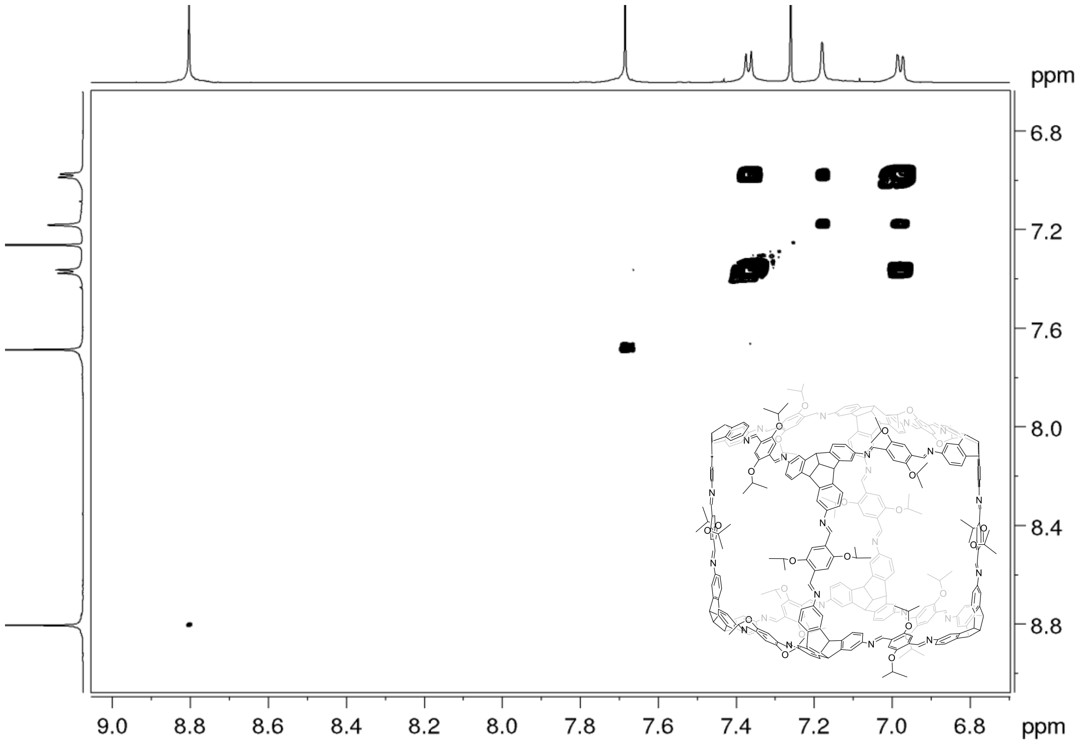

Figure 175. Partial ^{1}H-^{1}H COSY NMR spectrum (600 MHz, $CDCl_3$) of **O^{i}Pr-cube**.

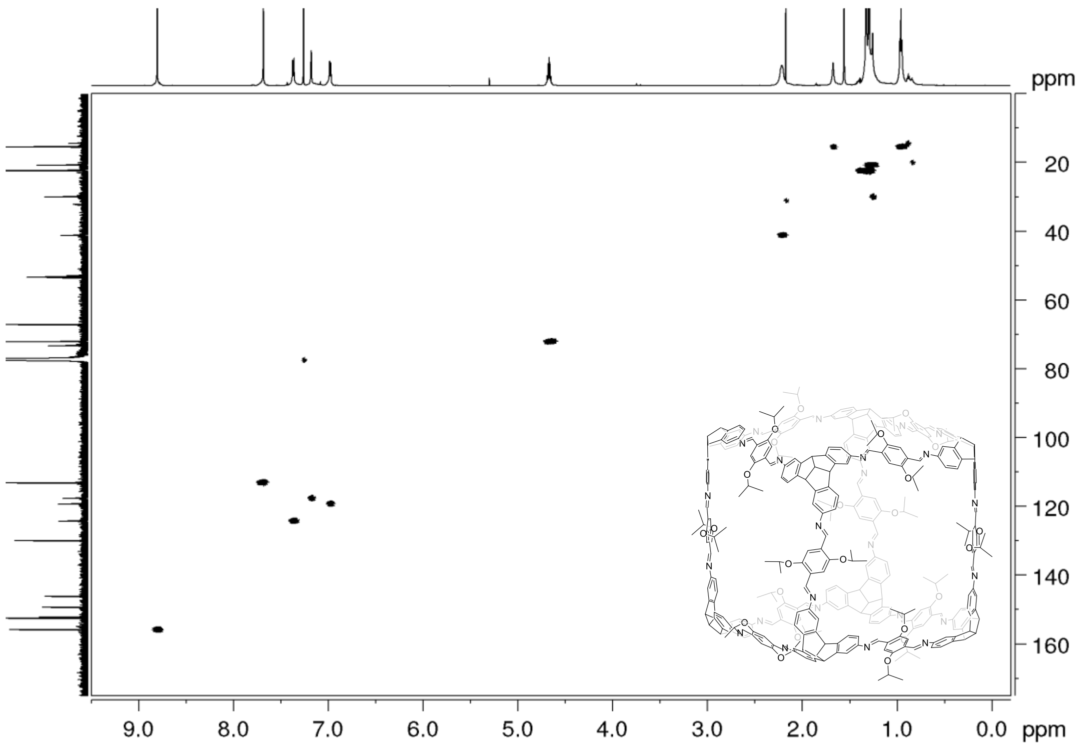

Figure 176. ^{1}H-^{13}C HSQC NMR (600 MHz and 151 MHz, $CDCl_3$) spectrum of **O^{i}Pr-cube**.

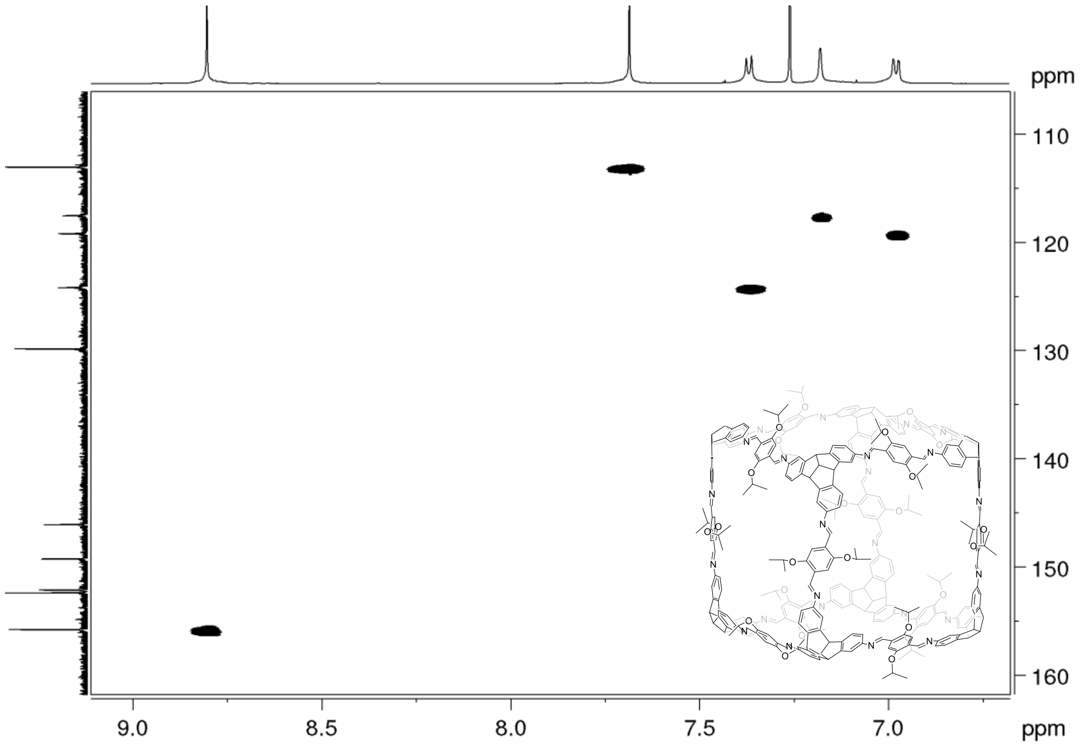

Figure 177. Partial ^{1}H-^{13}C HSQC NMR (600 MHz and 151 MHz, $CDCl_3$) spectrum of **O^{i}Pr-cube**.

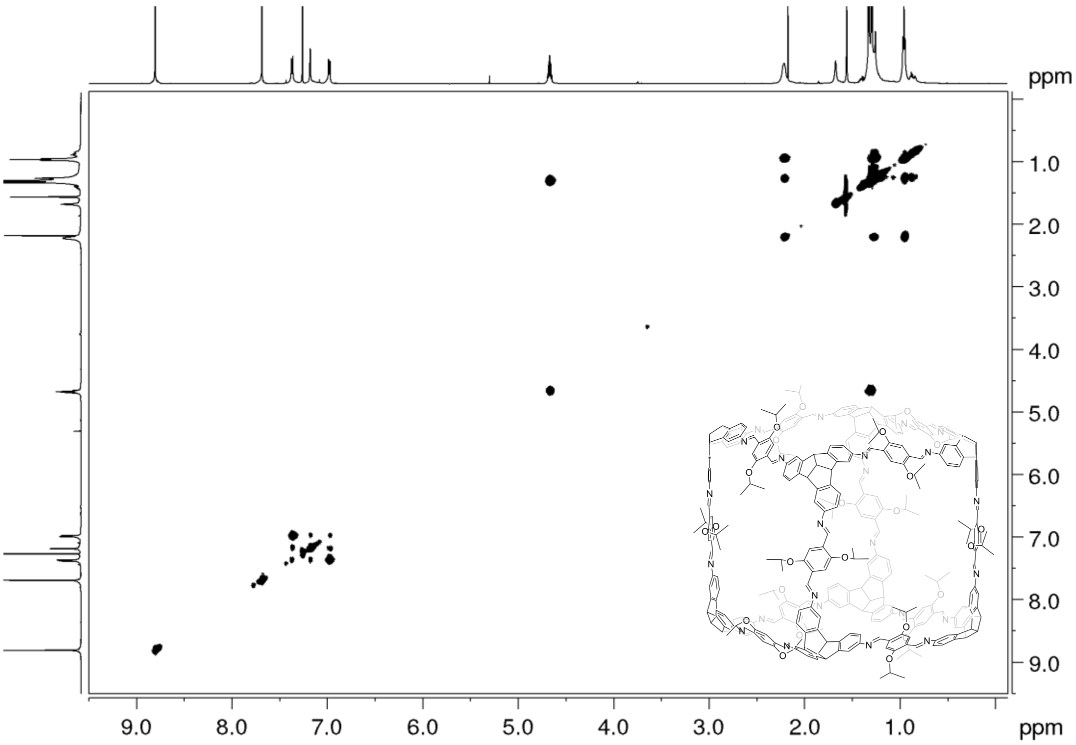

Figure 178. ^{1}H-^{1}H TOCSY NMR spectrum (600 MHz, $CDCl_3$) of **O^{i}Pr-cube**.

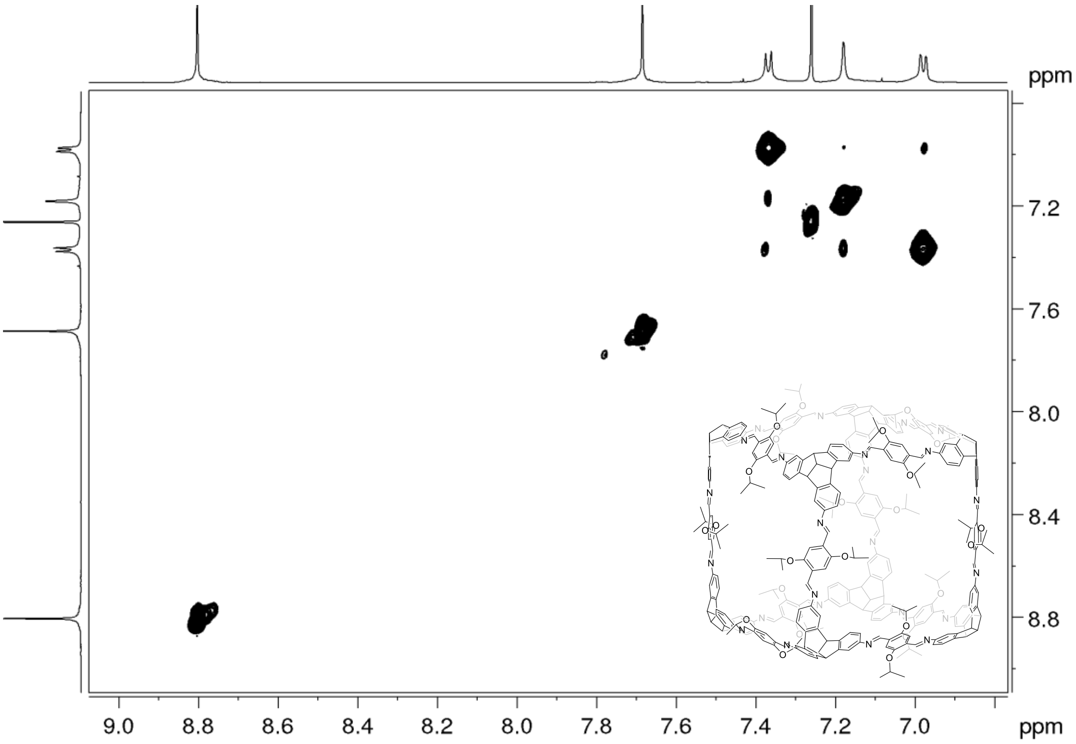

Figure 179. Partial ^{1}H-^{1}H TOCSY NMR (600 MHz, $CDCl_3$) spectrum of **O^{i}Pr-cube**.

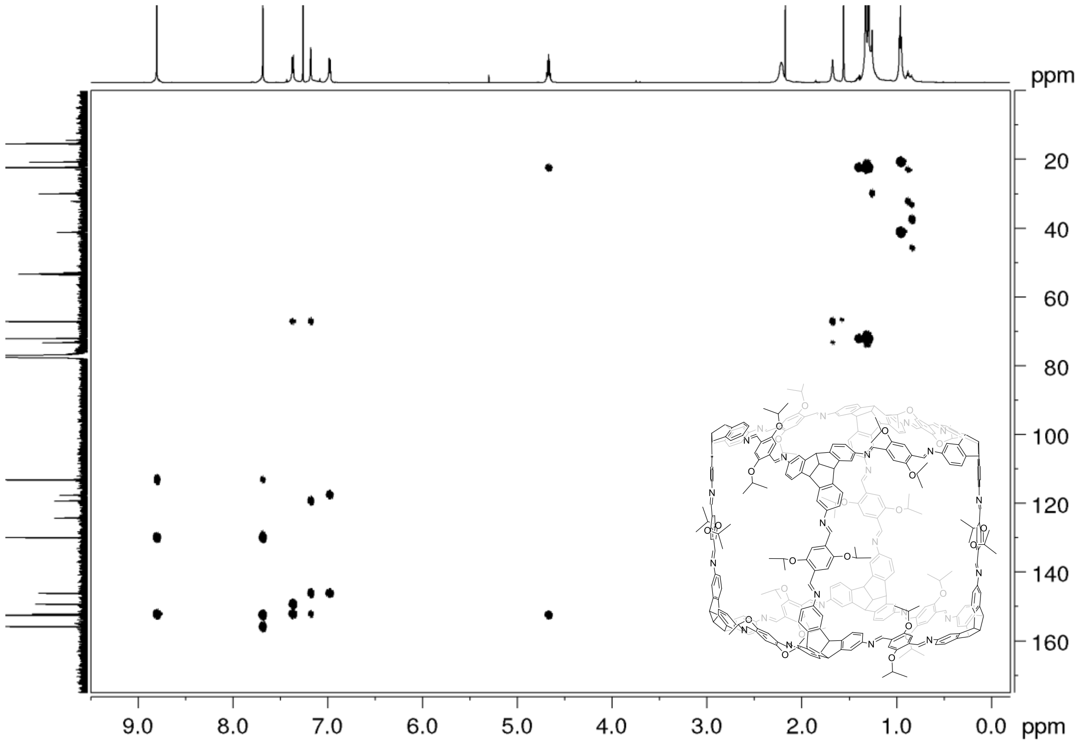

Figure 180. ^{1}H-^{13}C HMBC NMR (600 MHz and 151 MHz, $CDCl_3$) spectrum of **O^{i}Pr-cube**.

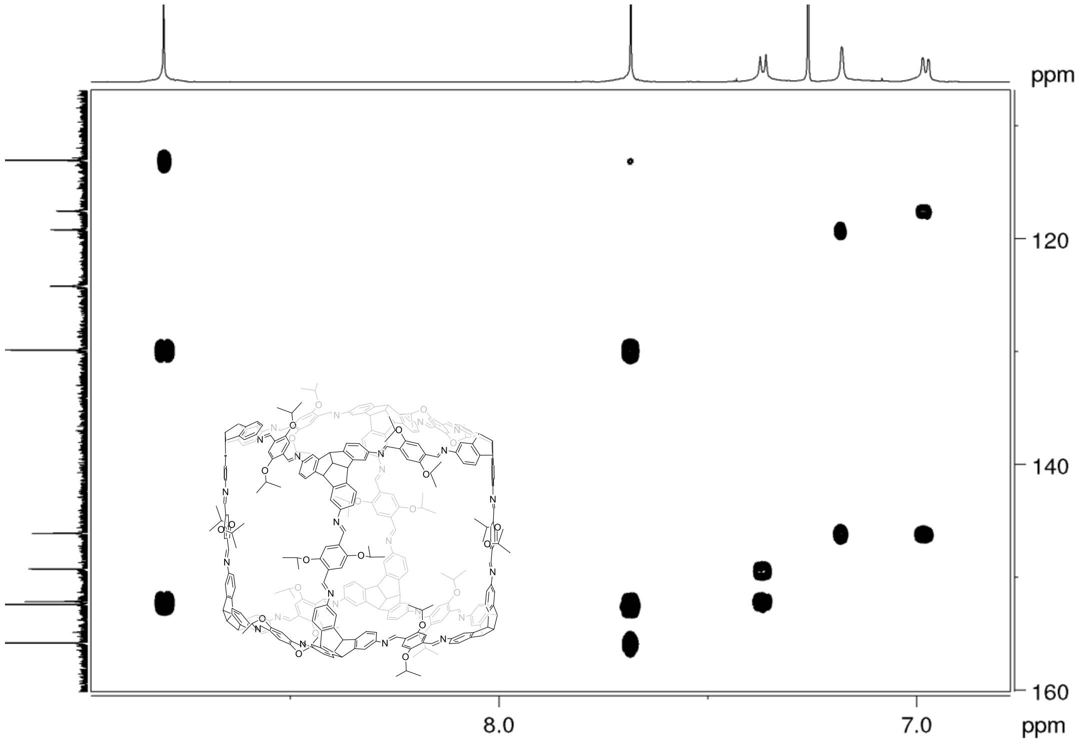

Figure 181. Partial ^{1}H-^{13}C HMBC NMR (600 MHz and 151 MHz, $CDCl_3$) spectrum of **O^{i}Pr-cube**.

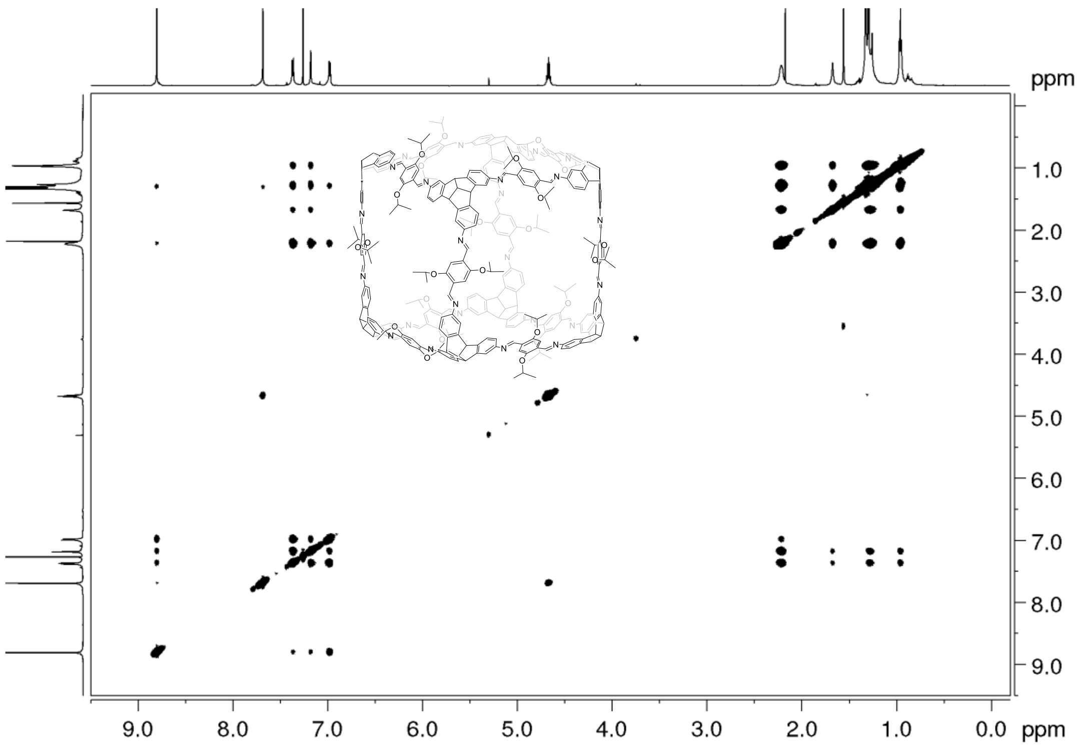

Figure 182. ${}^{1}H$-${}^{1}H$ NOESY NMR (600 MHz, $CDCl_3$) spectrum of **O^{i}Pr-cube**.

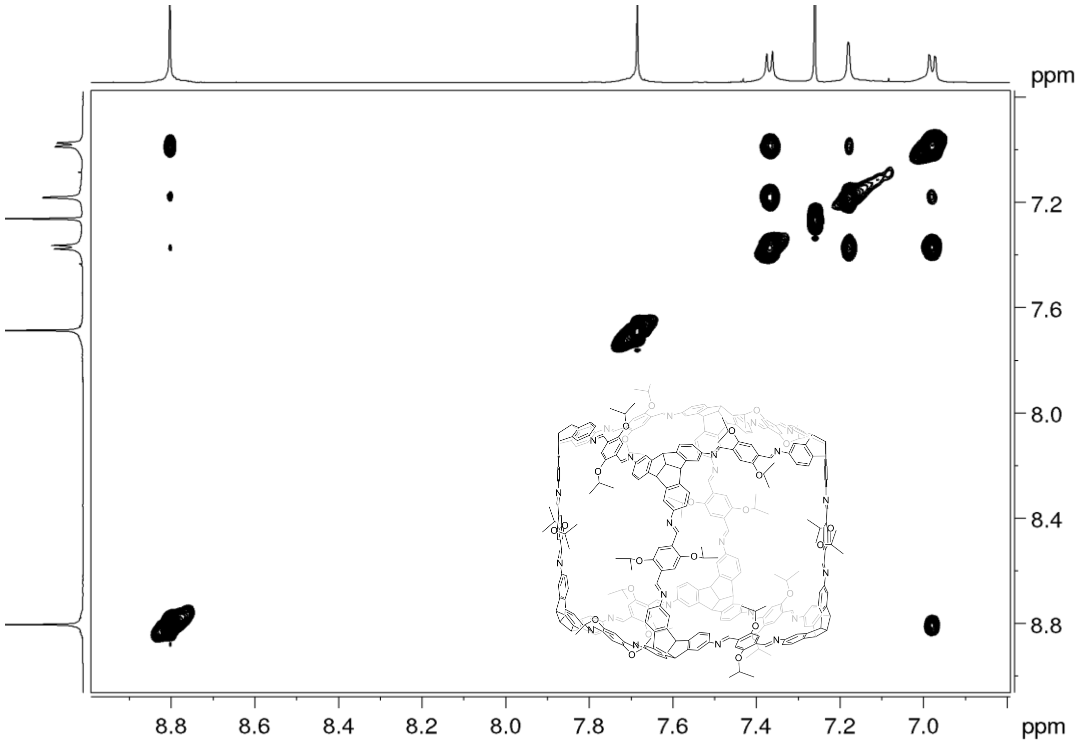

Figure 183. Partial ${}^{1}H$-${}^{1}H$ NOESY NMR (600 MHz, $CDCl_3$) spectrum of **O^{i}Pr-cube**.

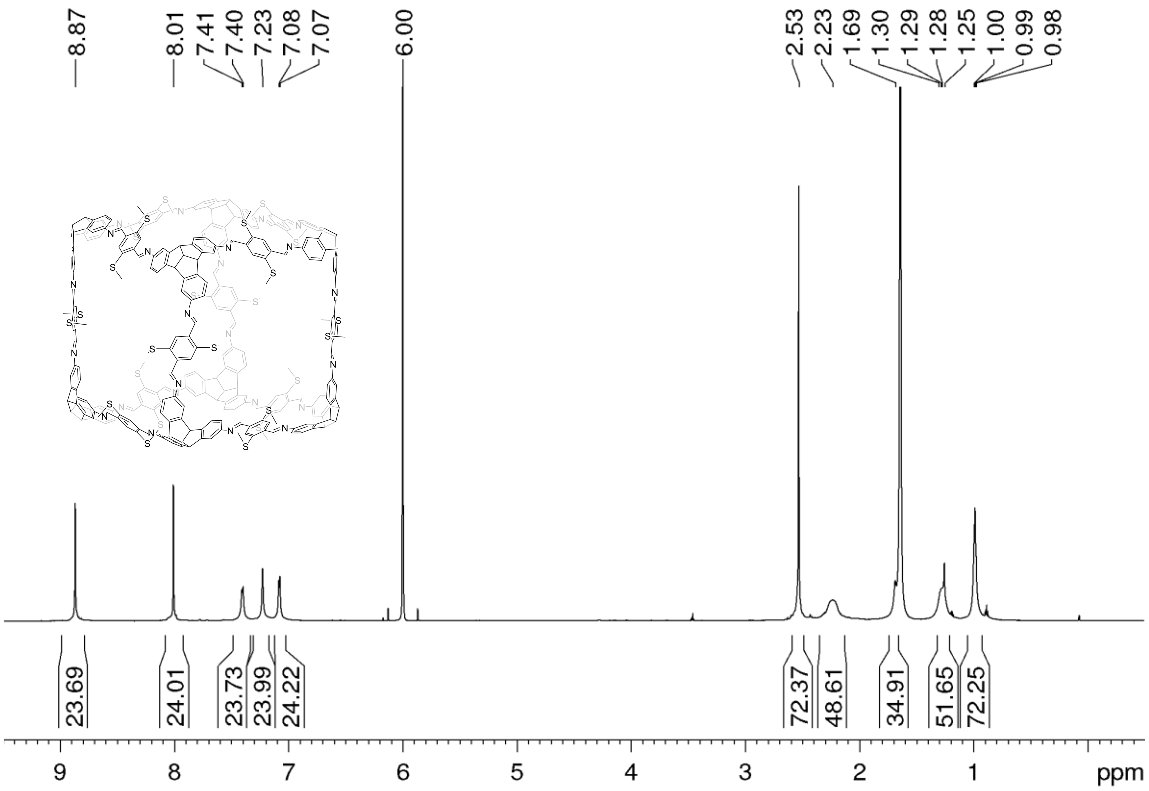

Figure 184. ^{1}H NMR (700 MHz, $C_2D_2Cl_4$) spectrum of **SMe-cube**.

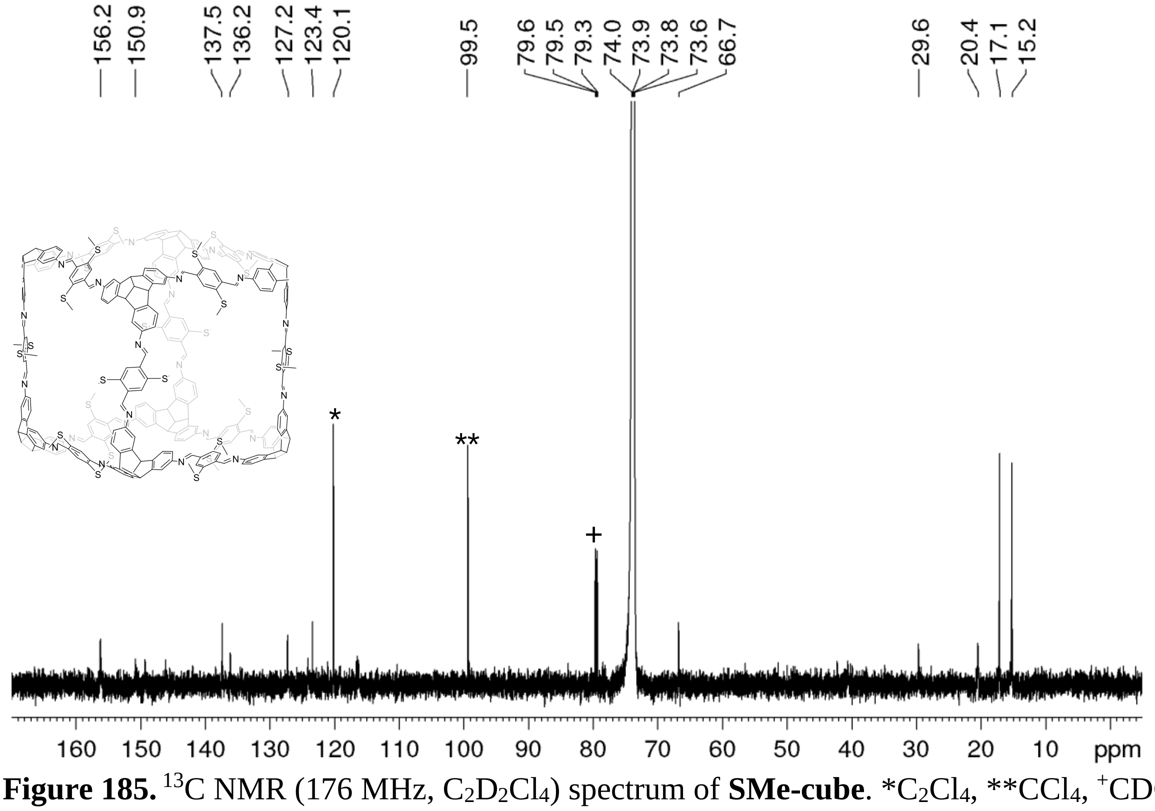

Figure 185. ^{13}C NMR (176 MHz, $C_2D_2Cl_4$) spectrum of **SMe-cube**. *C_2Cl_4, **CCl_4, $^{+}CDCl_3$)

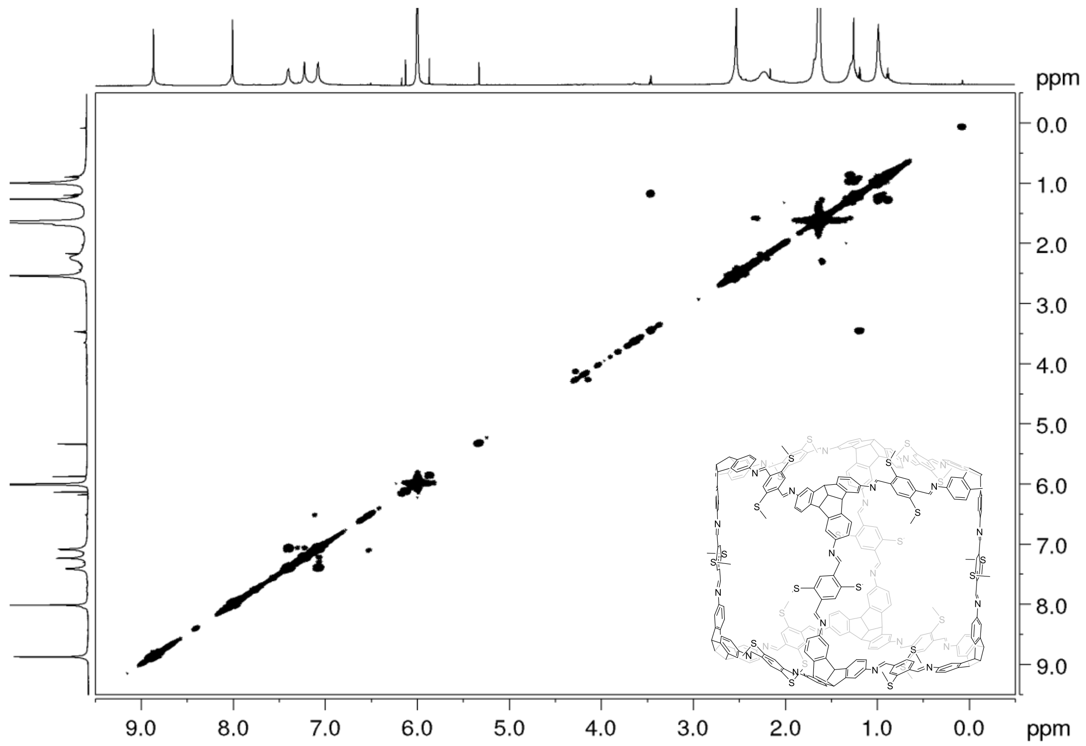

Figure 186. ^1H-^1H COSY NMR spectrum (700 MHz, $C_2D_2Cl_4$) of **SMe-cube**.

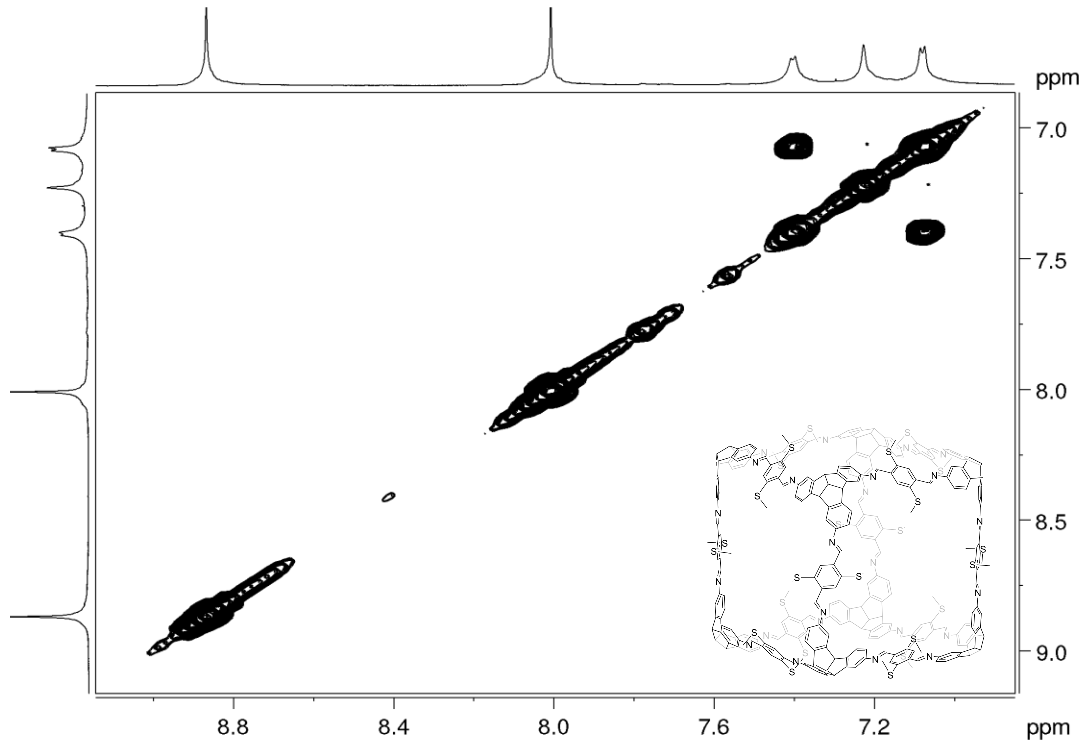

Figure 187. Partial ^1H-^1H COSY NMR spectrum (700 MHz, $C_2D_2Cl_4$) of **SMe-cube**.

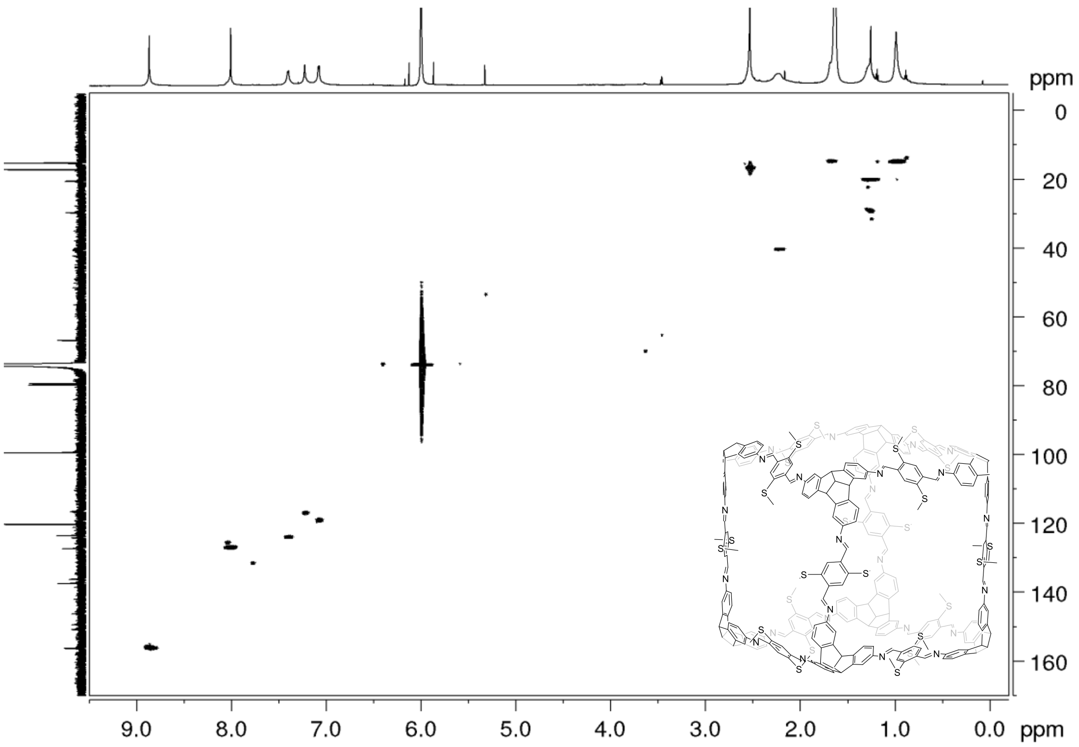

Figure 188. ^{1}H-^{13}C HSQC NMR (700 MHz and 176 MHz, $C_2D_2Cl_4$) spectrum of **SMe-cube**.

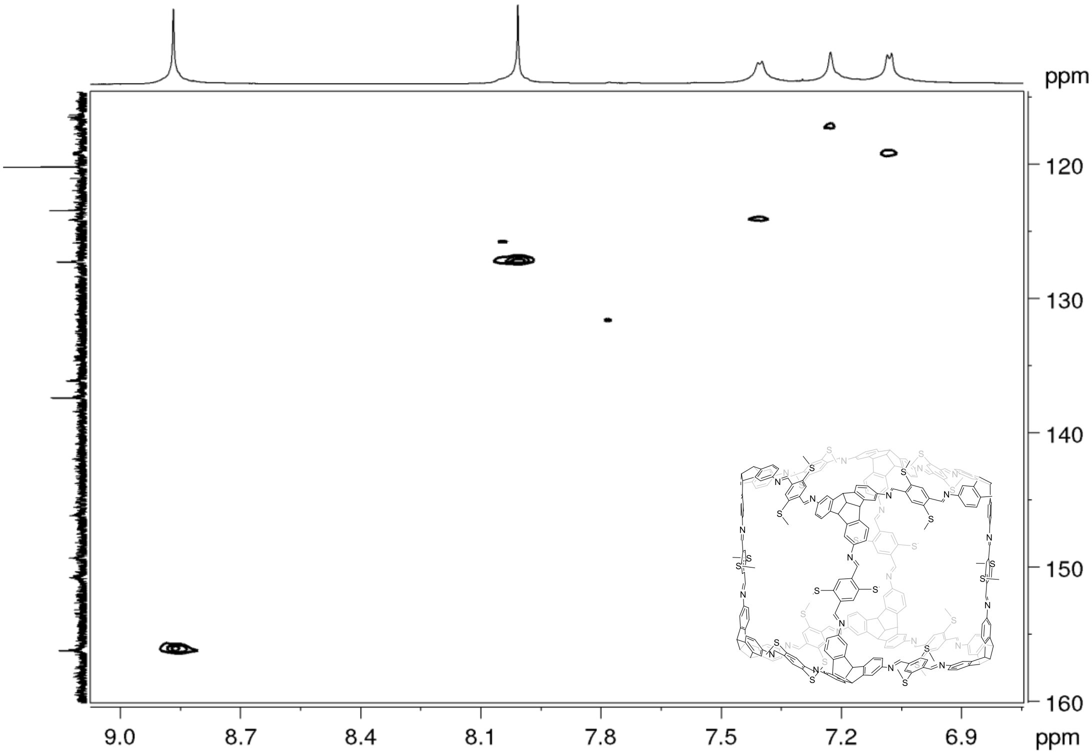

Figure 189. Partial ^{1}H-^{13}C HSQC NMR (700 MHz and 176 MHz, $C_2D_2Cl_4$) spectrum of **SMe-cube**.

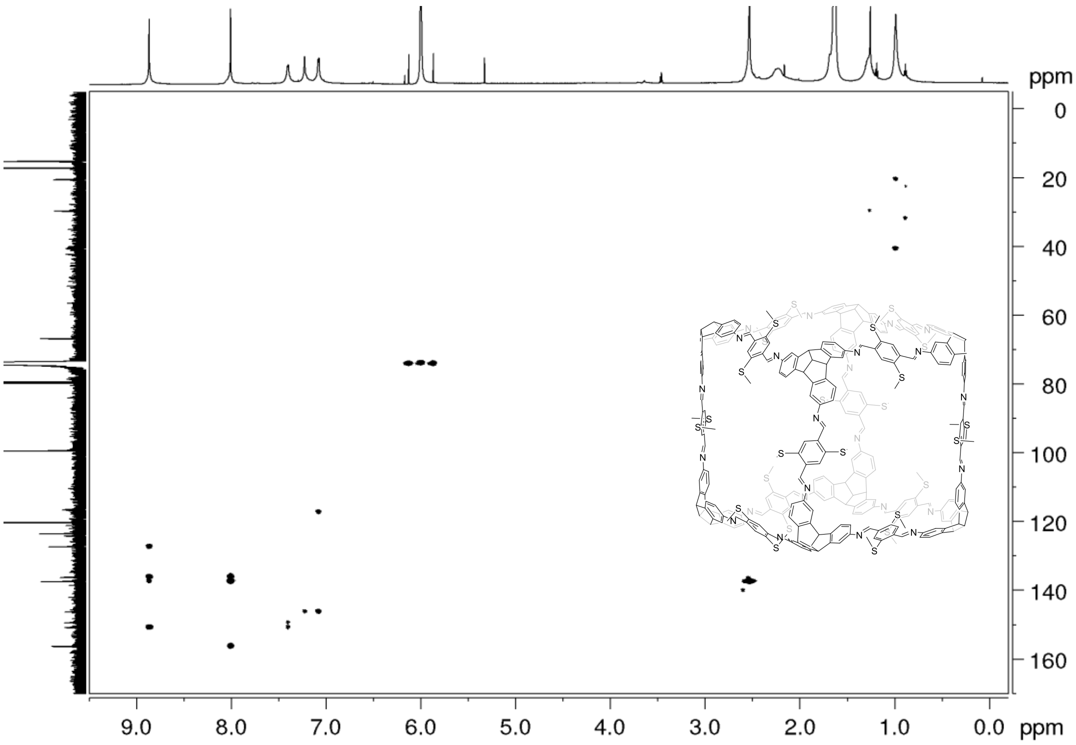

Figure 190. ^{1}H-^{13}C HMBC NMR (700 MHz and 176 MHz, $C_2D_2Cl_4$) spectrum of **SMe-cube**.

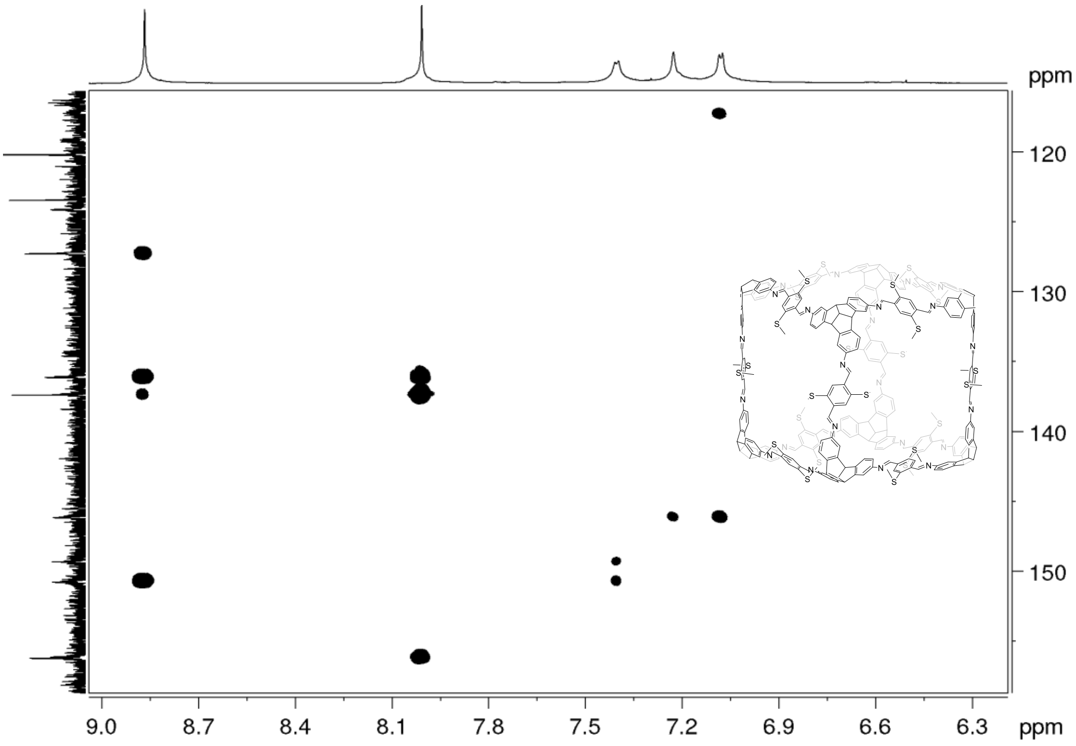

Figure 191. Partial ^{1}H-^{13}C HMBC NMR (700 MHz and 176 MHz, $C_2D_2Cl_4$) spectrum of **SMe-cube**.

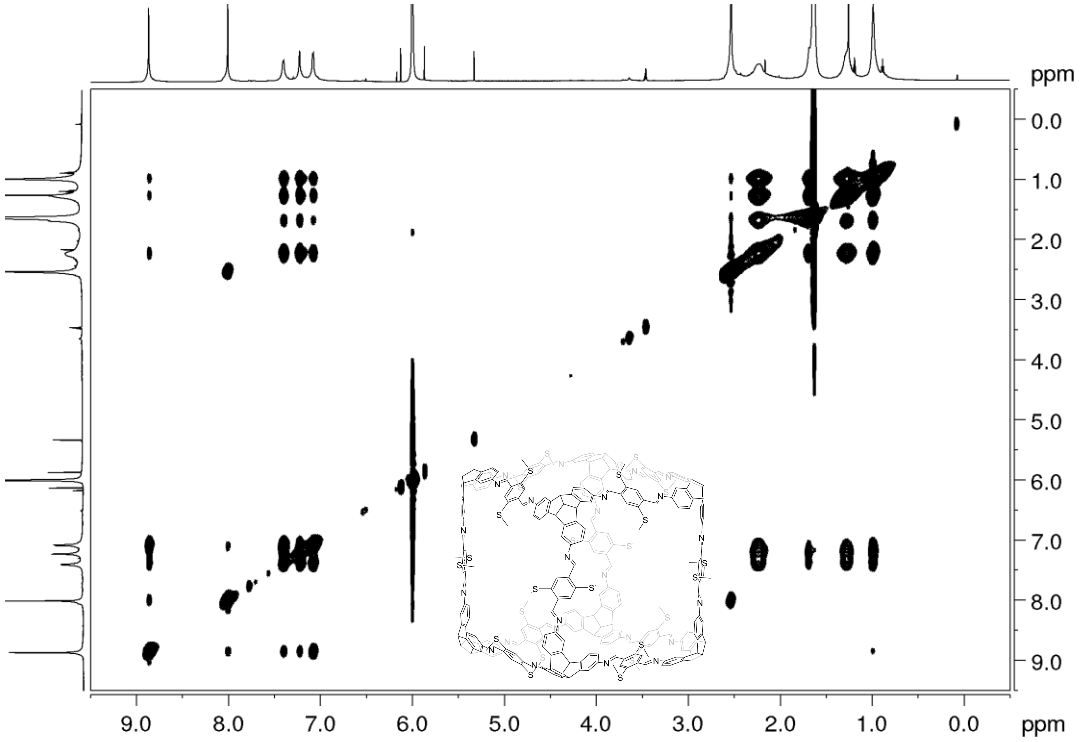

Figure 192. ^{1}H-^{1}H NOESY NMR (700 MHz, CD_2Cl_2) spectrum of **SMe-cube**.

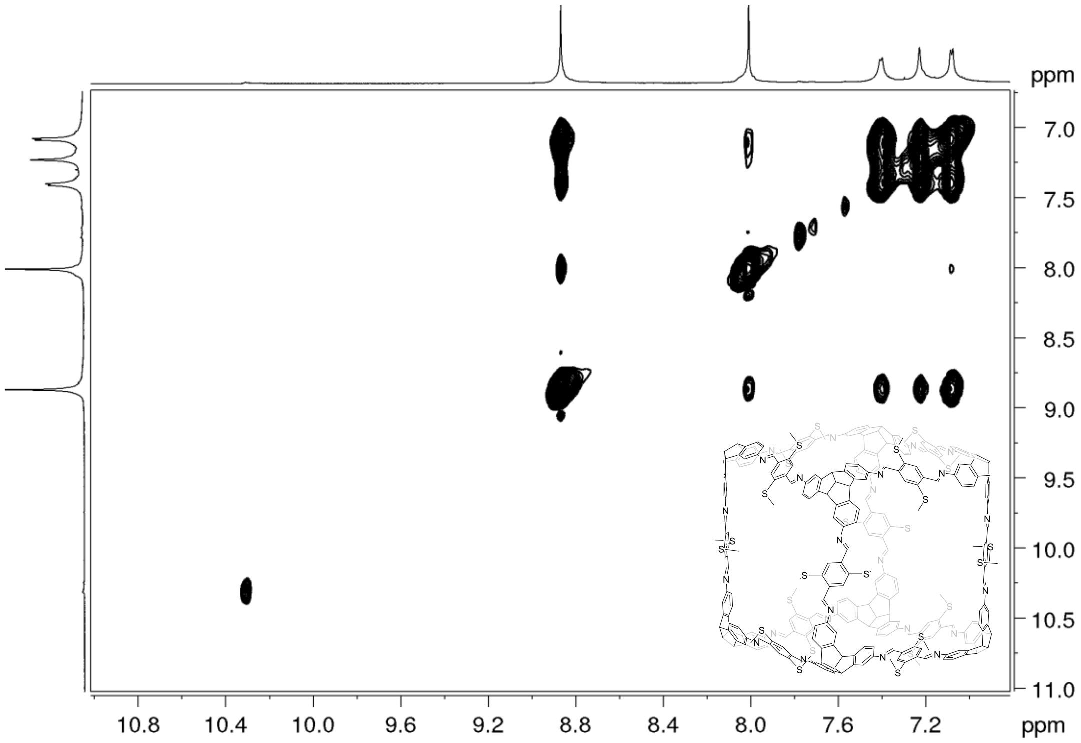

Figure 193. Partial ^{1}H-^{1}H NOESY NMR (700 MHz, CD_2Cl_2) spectrum of **SMe-cube**.

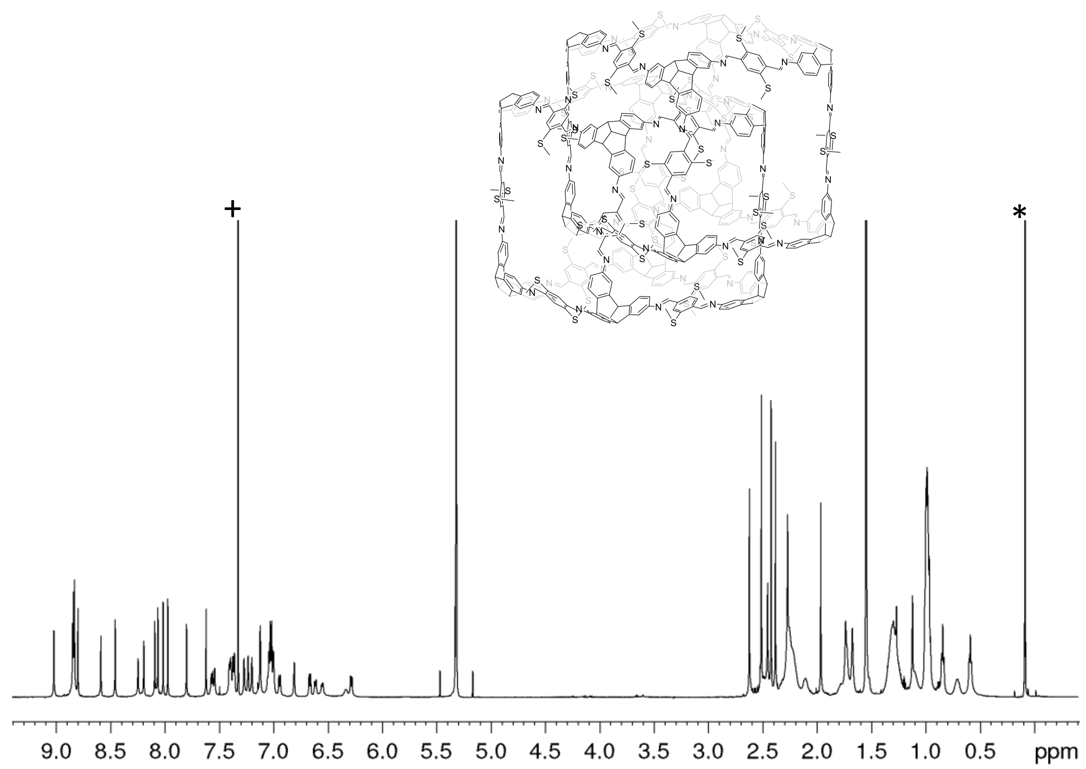

Figure 194. ^{1}H NMR (600 MHz, CD_2Cl_2) spectrum of **(SMe-cube)₂**. (+$CHCl_3$, *silicone grease).

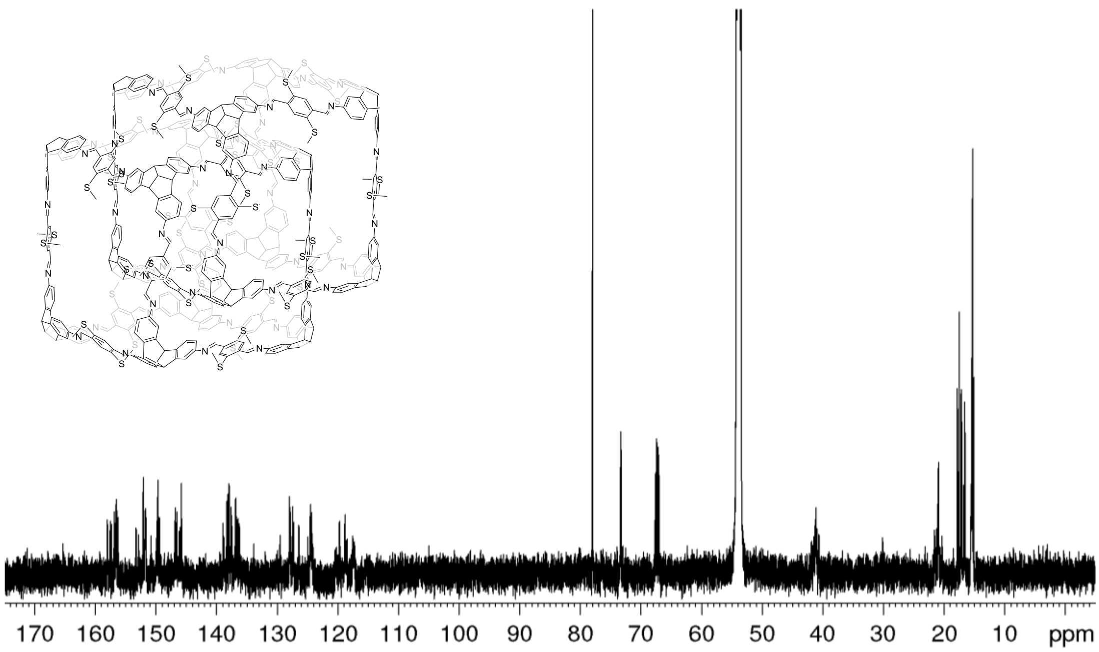

Figure 195. ^{13}C NMR (151 MHz, CD_2Cl_2) spectrum of **(SMe-cube)$_2$**.

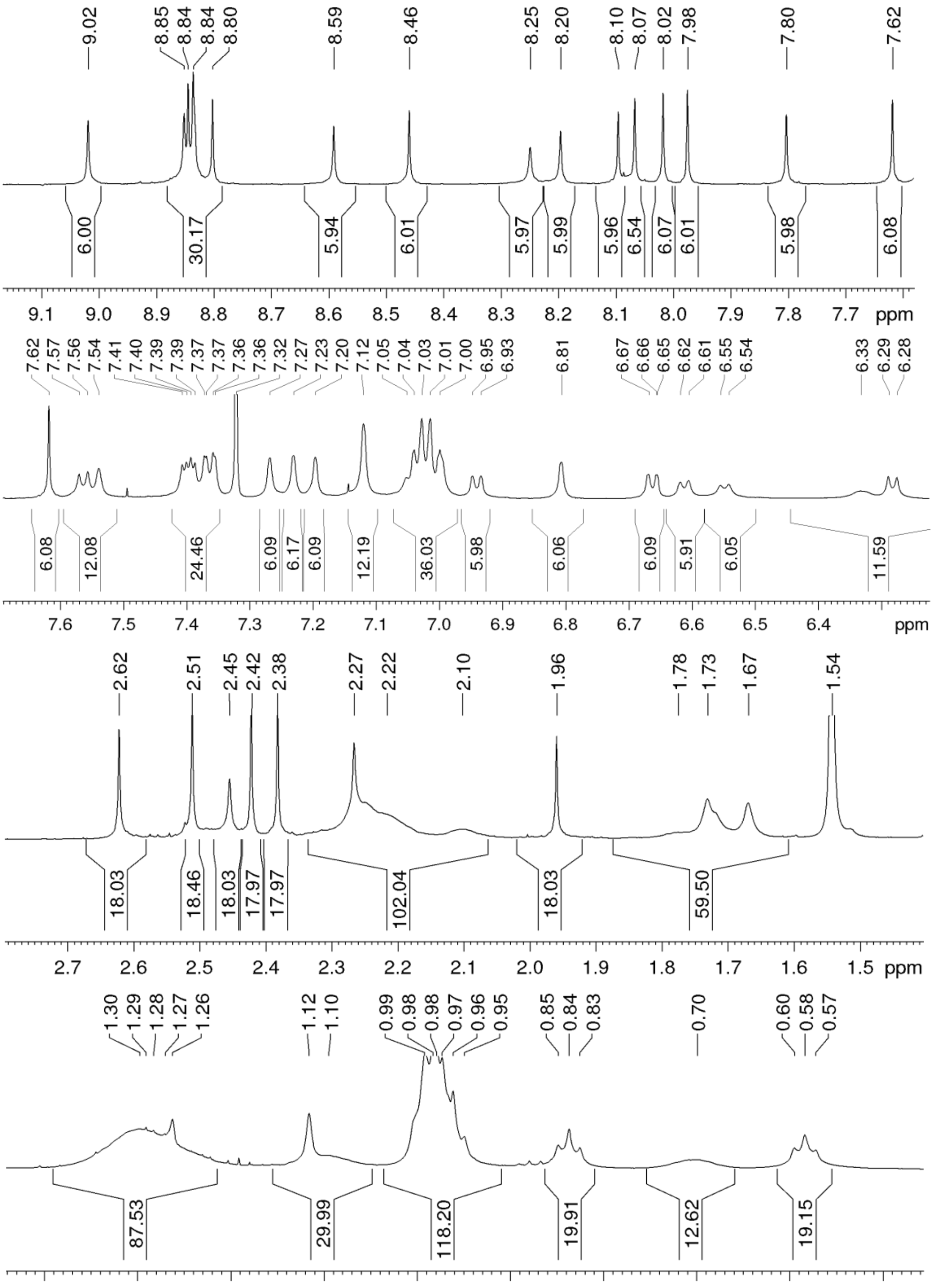

Figure 196. Expanded ^{1}H NMR (600 MHz, CD_2Cl_2) spectrum of **(SMe-cube)$_2$**.

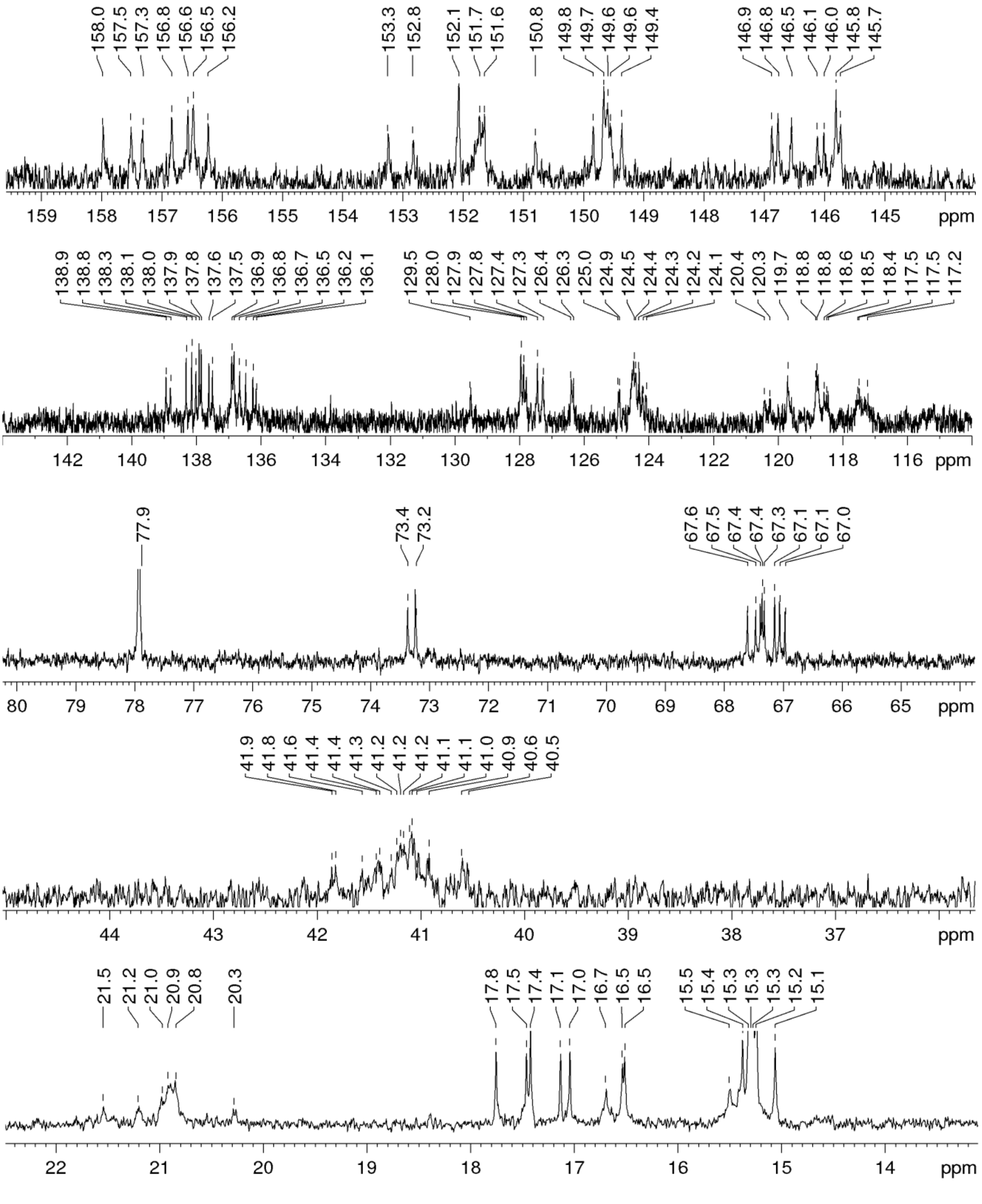

Figure 197. Expanded ^{13}C NMR (151 MHz, CD_2Cl_2) spectrum of **(SMe-cube)$_2$**.

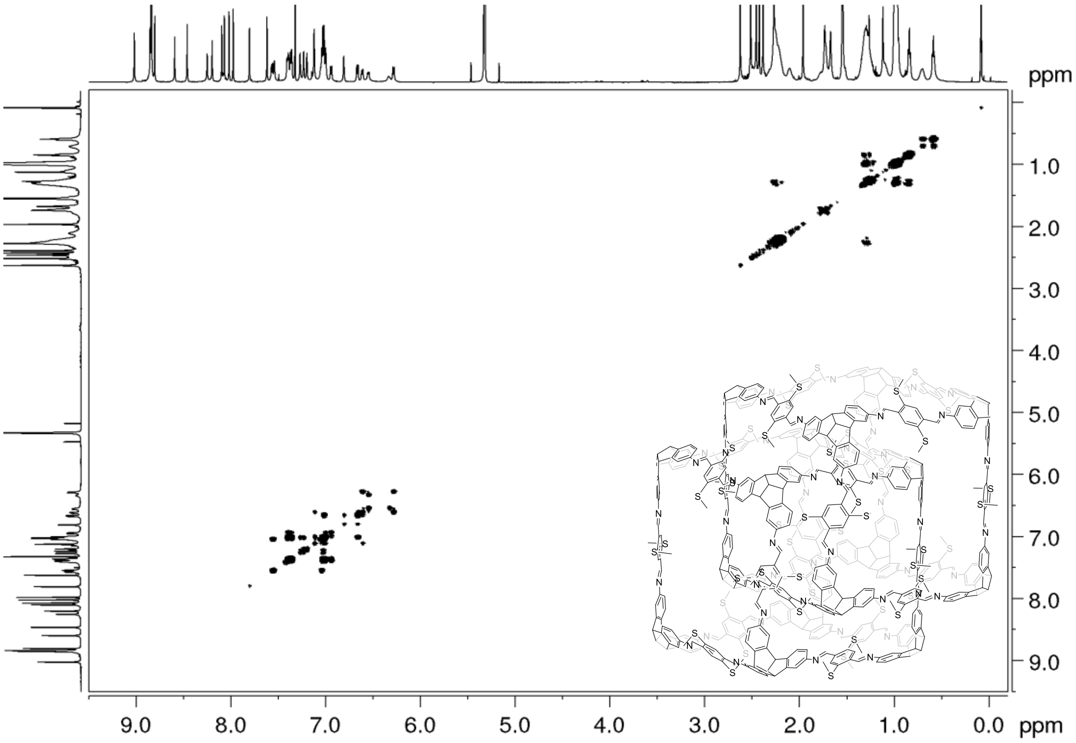

Figure 198. ^{1}H-^{1}H COSY NMR spectrum (600 MHz, CD_2Cl_2) of **(SMe-cube)$_2$**.

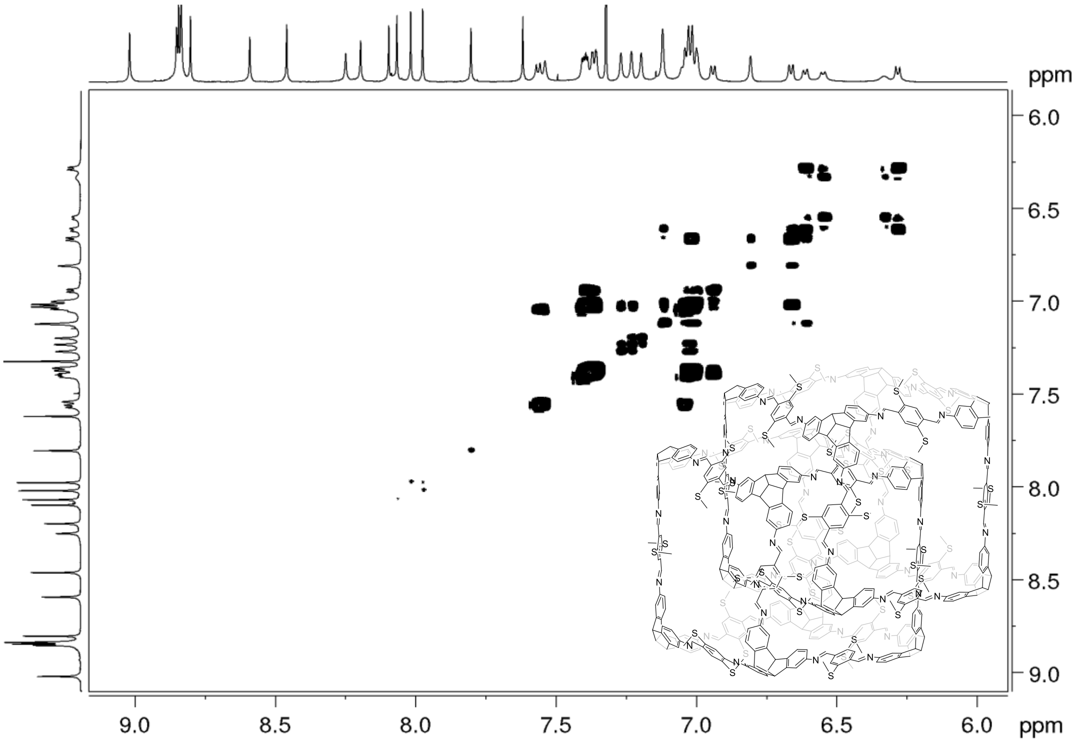

Figure 199. Partial ^{1}H-^{1}H COSY NMR spectrum (600 MHz, CD_2Cl_2) of **(SMe-cube)$_2$**.

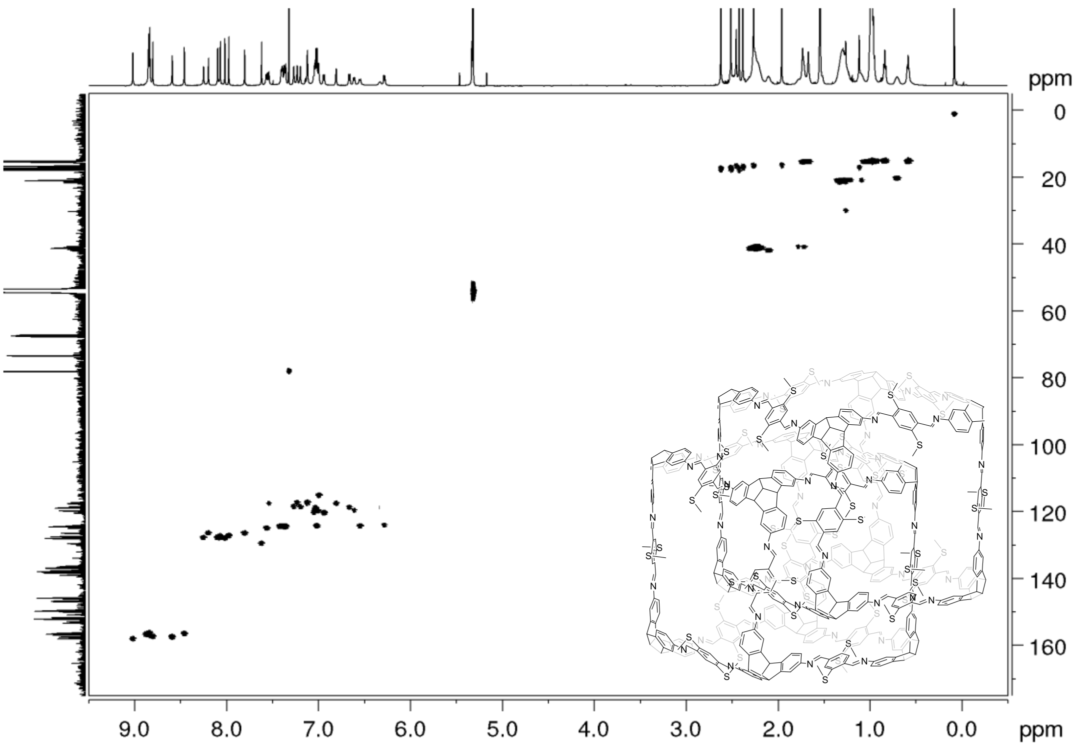

Figure 200. ^{1}H-^{13}C HSQC NMR (600 MHz and 151 MHz, CD_2Cl_2) spectrum of **(SMe-cube)$_2$**.

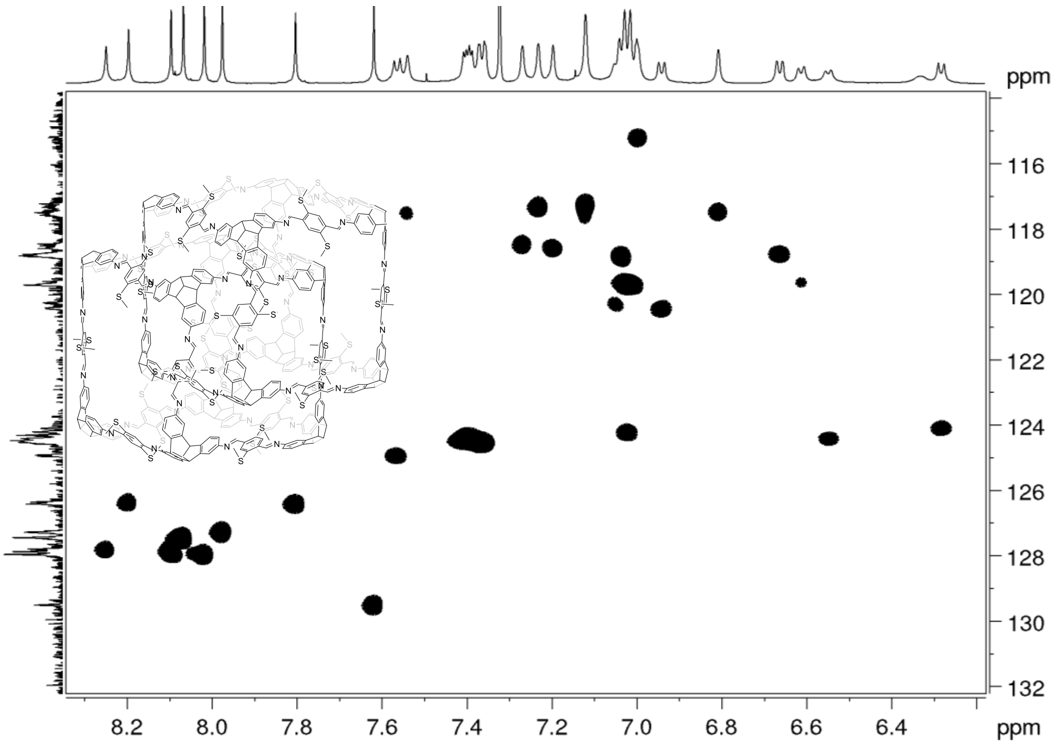

Figure 201. Partial ^{1}H-^{13}C HSQC NMR (600 MHz and 151 MHz, CD_2Cl_2) spectrum of **(SMe-cube)$_2$**.

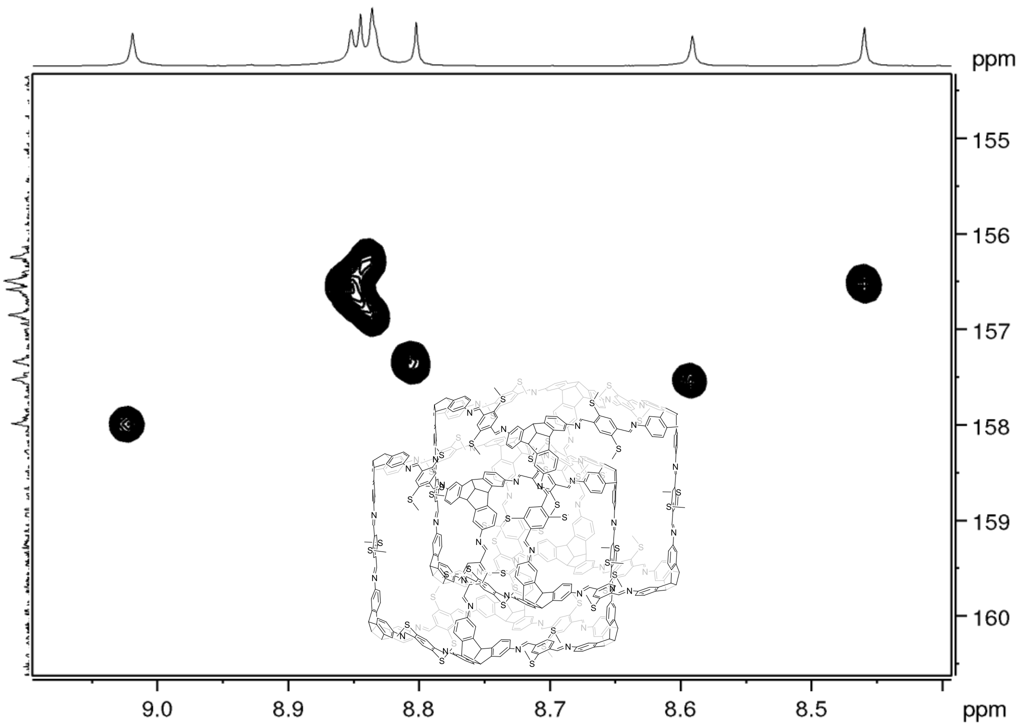

Figure 202. Partial ^{1}H-^{13}C HSQC NMR (600 MHz and 151 MHz, CD_2Cl_2) spectrum of **(SMe-cube)$_2$**.

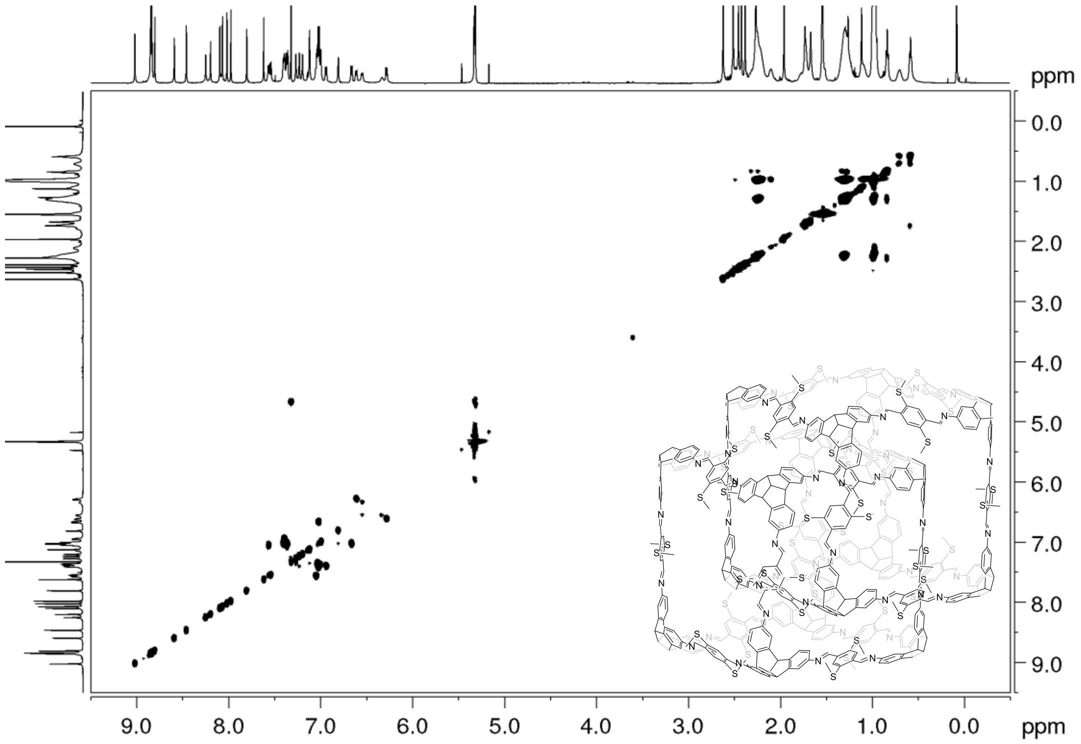

Figure 203. ^{1}H-^{1}H TOCSY NMR (600 MHz, CD_2Cl_2) spectrum of **(SMe-cube)$_2$**.

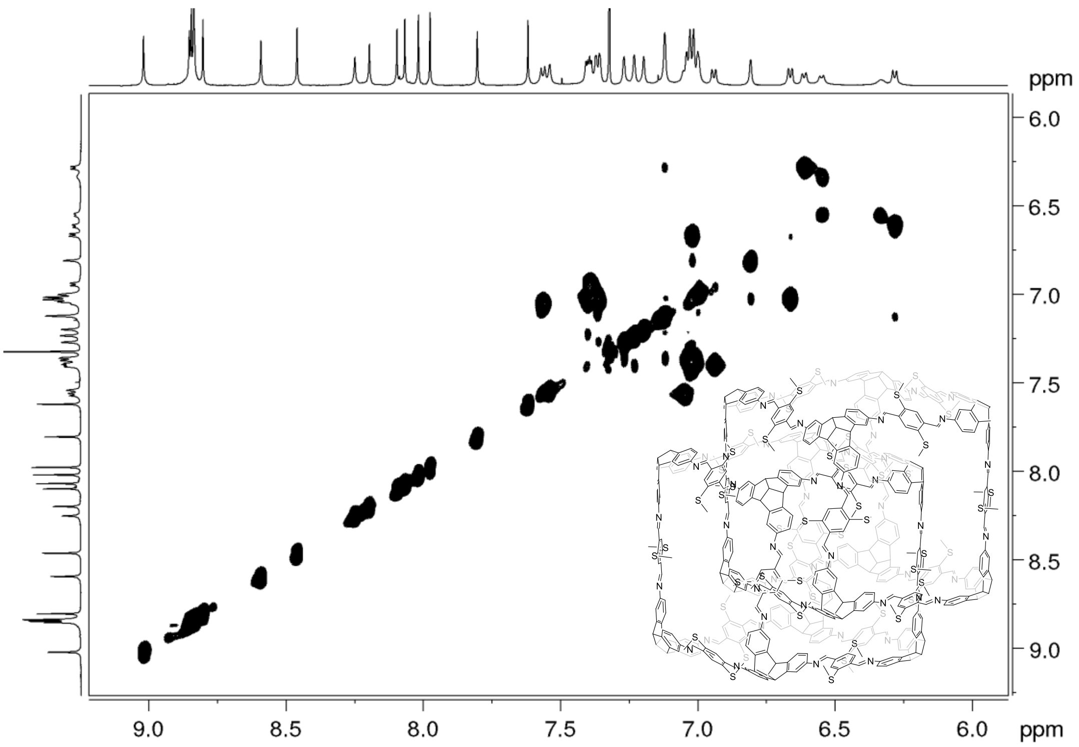

Figure 204. Partial ^{1}H-^{1}H TOCSY NMR (600 MHz, CD_2Cl_2) spectrum of **(SMe-cube)$_2$**.

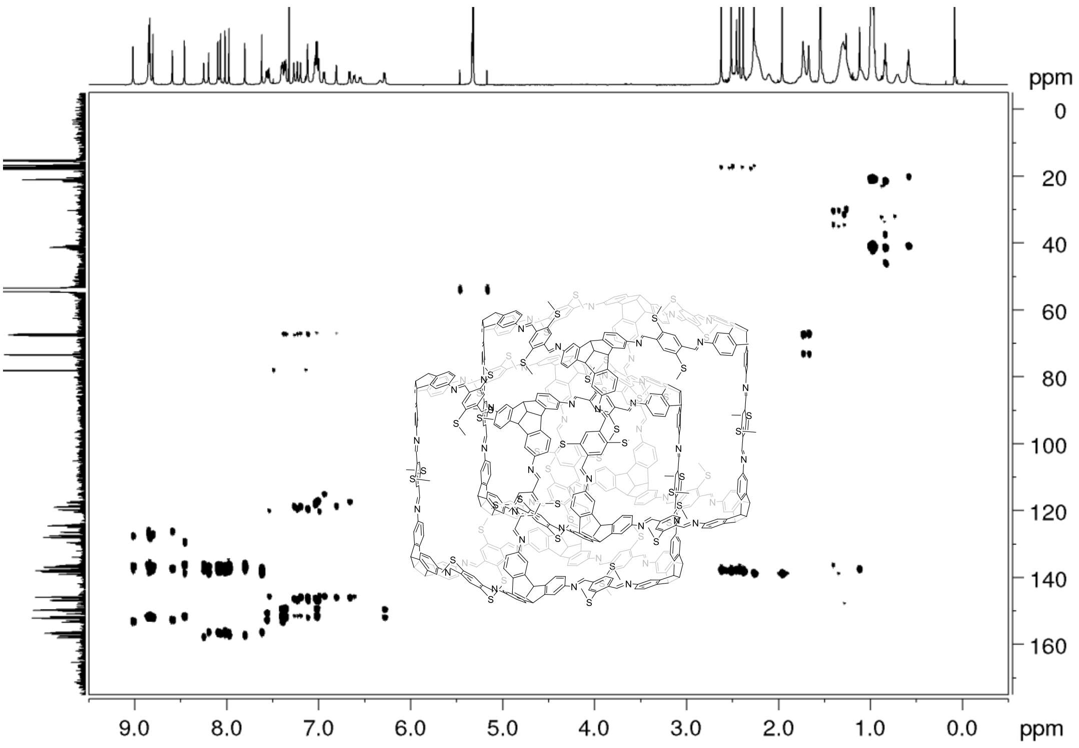

Figure 205. ^{1}H-^{13}C HMBC NMR (600 MHz and 151 MHz, CD_2Cl_2) spectrum of **(SMe-cube)$_2$**.

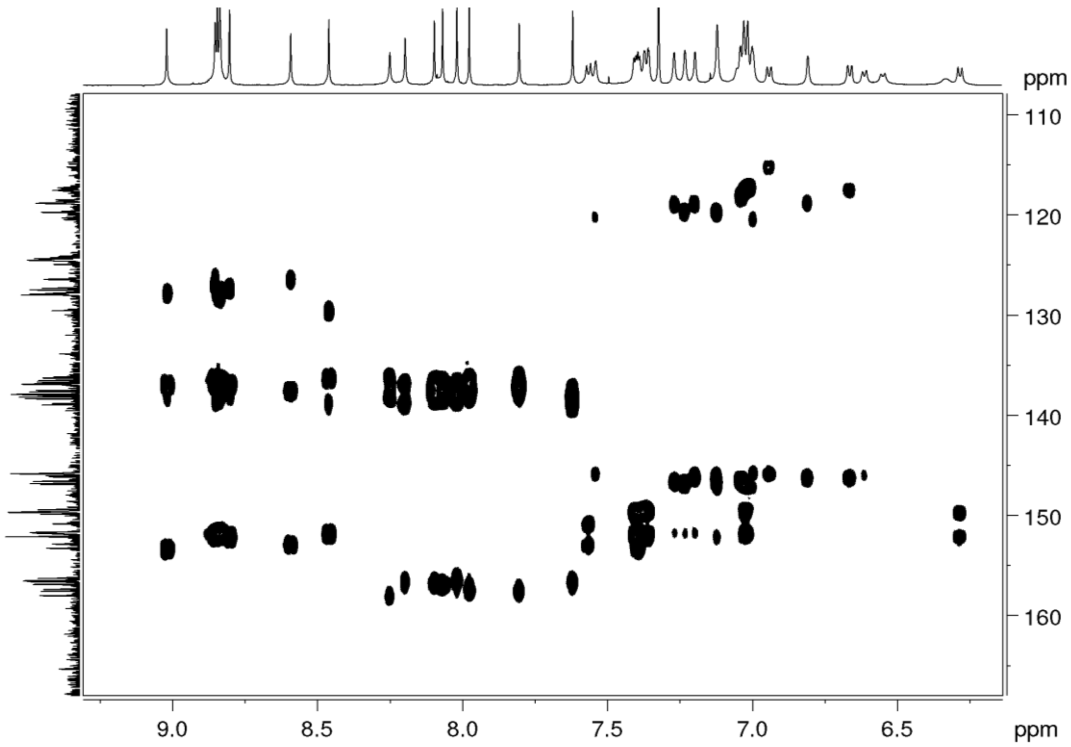

Figure 206. Partial ^{1}H-^{13}C HMBC NMR (600 MHz and 151 MHz, CD_2Cl_2) spectrum of **(SMe-cube)$_2$**.

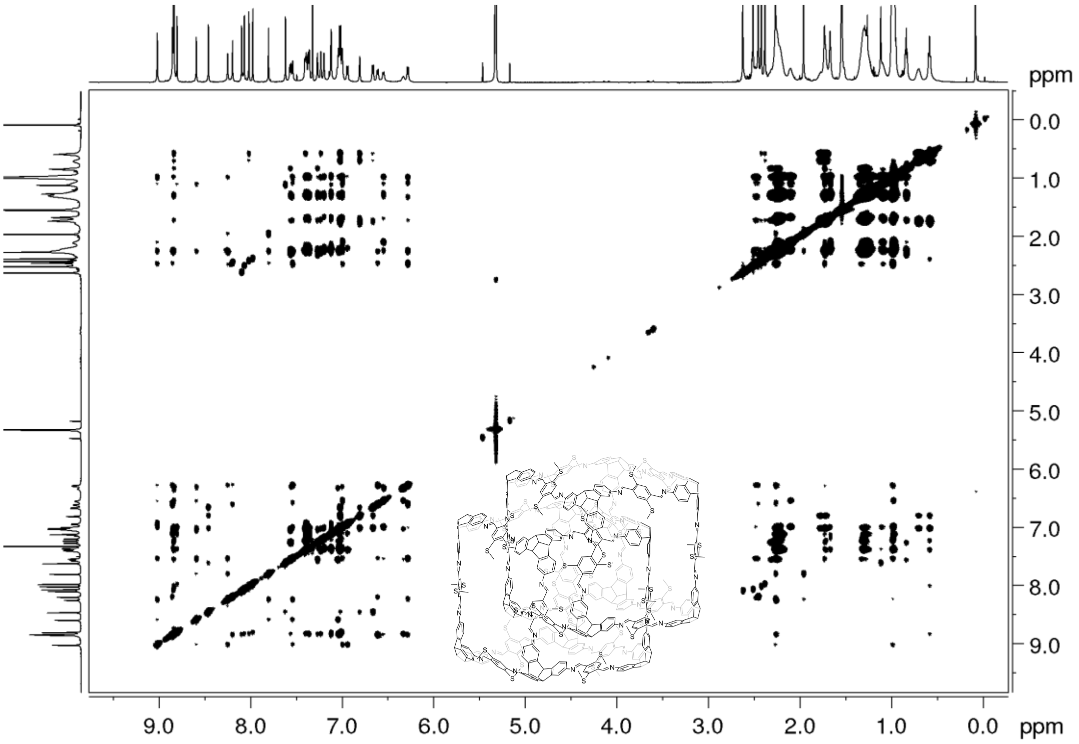

Figure 207. ^{1}H-^{1}H NOESY NMR (600 MHz, CD_2Cl_2) spectrum of **(SMe-cube)$_2$**.

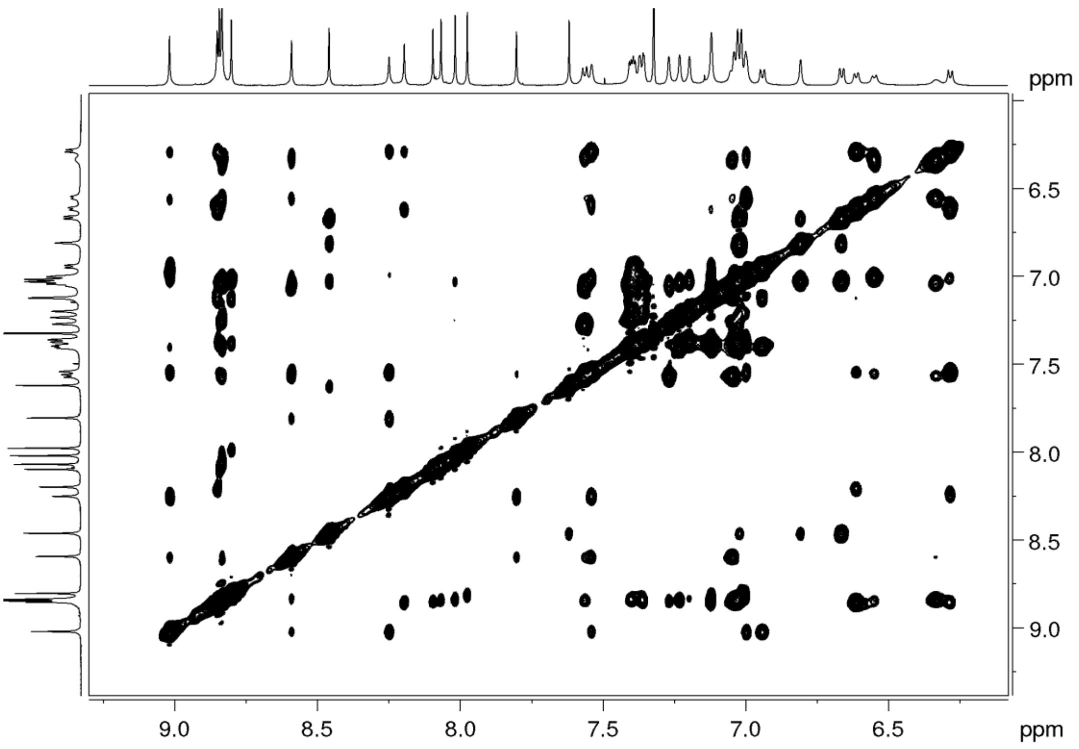

Figure 208. Partial ^{1}H-^{1}H NOESY NMR (600 MHz, CD_2Cl_2) spectrum of **(SMe-cube)$_2$**.

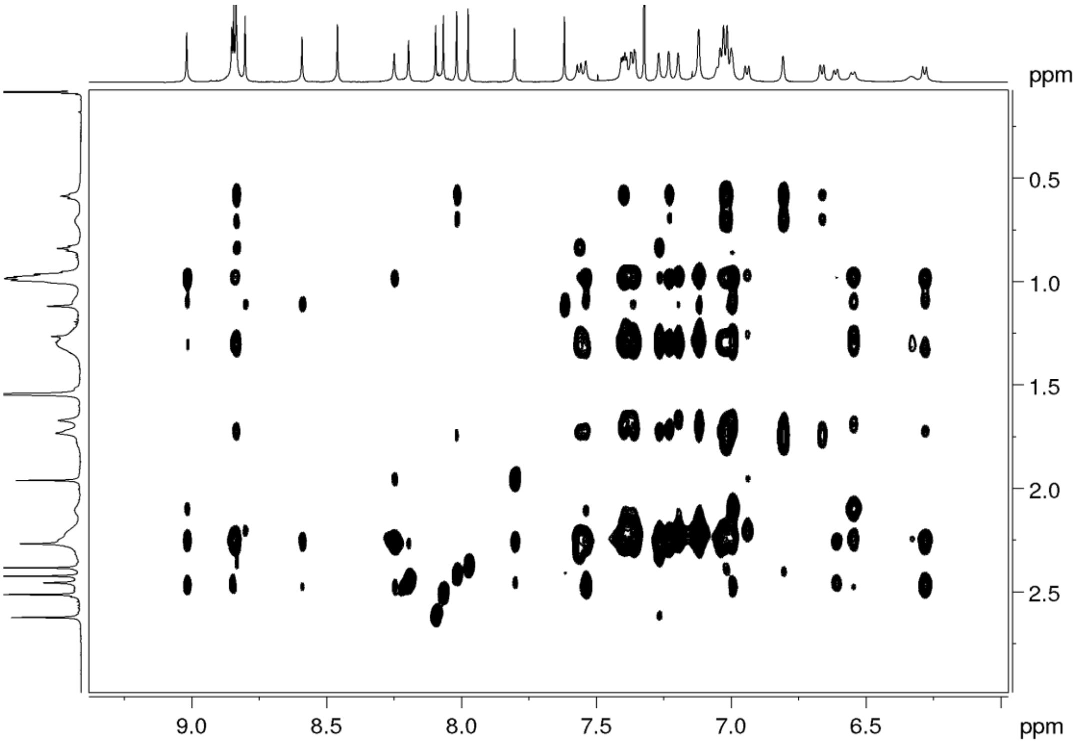

Figure 209. Partial ^{1}H-^{1}H NOESY NMR (600 MHz, CD_2Cl_2) spectrum of **(SMe-cube)$_2$**.

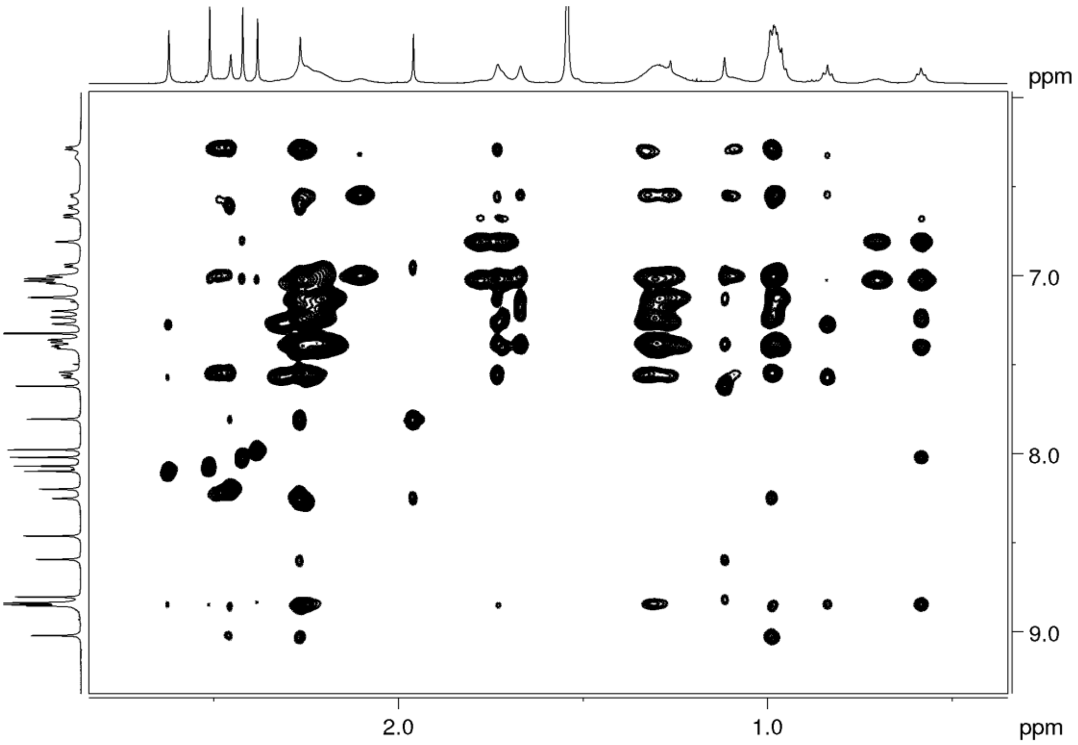

Figure 210. Partial ^{1}H-^{1}H NOESY NMR (600 MHz, CD_2Cl_2) spectrum of **(SMe-cube)$_2$**.

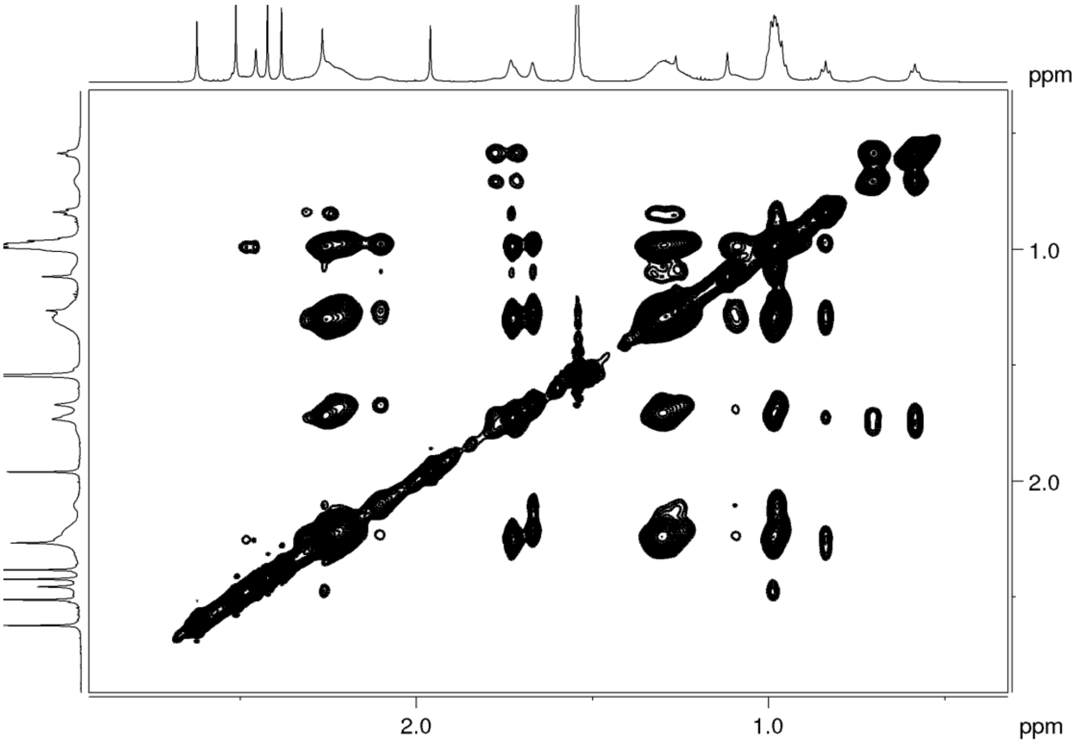

Figure 211. Partial ^{1}H-^{1}H NOESY NMR (600 MHz, CD_2Cl_2) spectrum of **(SMe-cube)$_2$**.

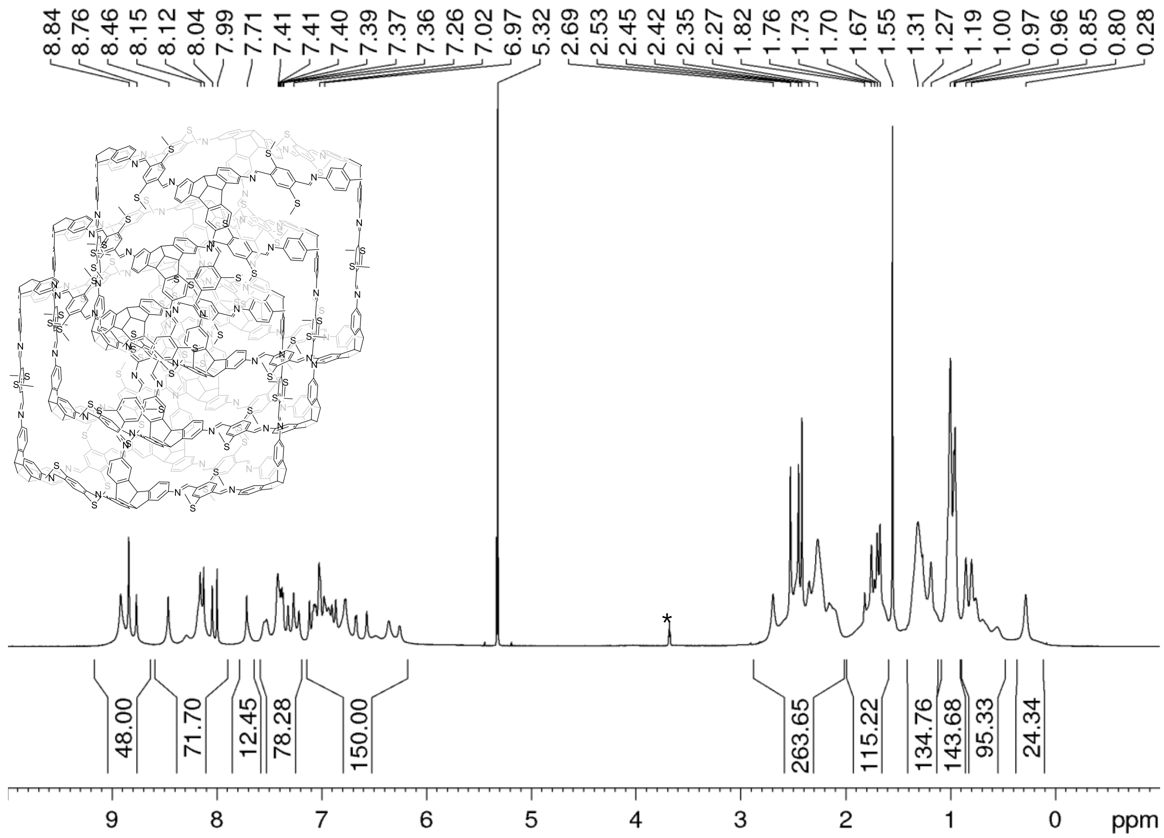

Figure 212. ^{1}H NMR (700 MHz, CD_2Cl_2) spectrum of **(SMe-cube)$_3$**. (*THF)

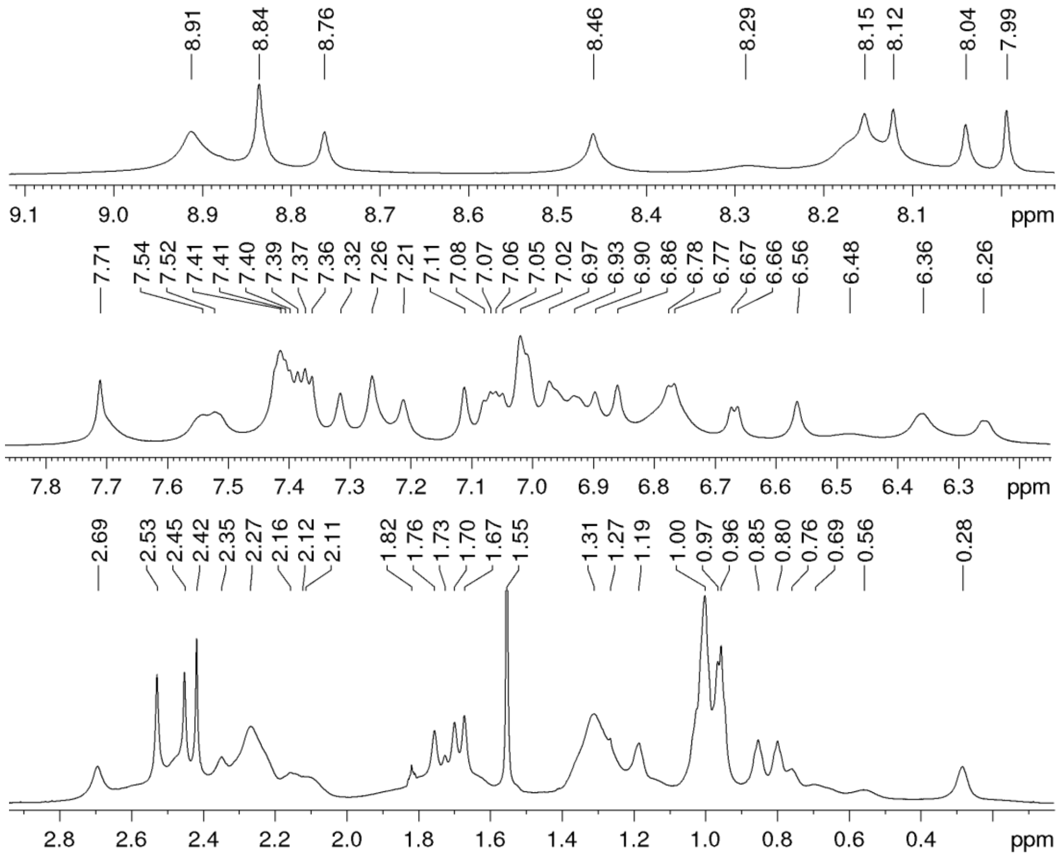

Figure 213. Expanded ^{1}H NMR (700 MHz, CD_2Cl_2) spectrum of **(SMe-cube)$_3$**.

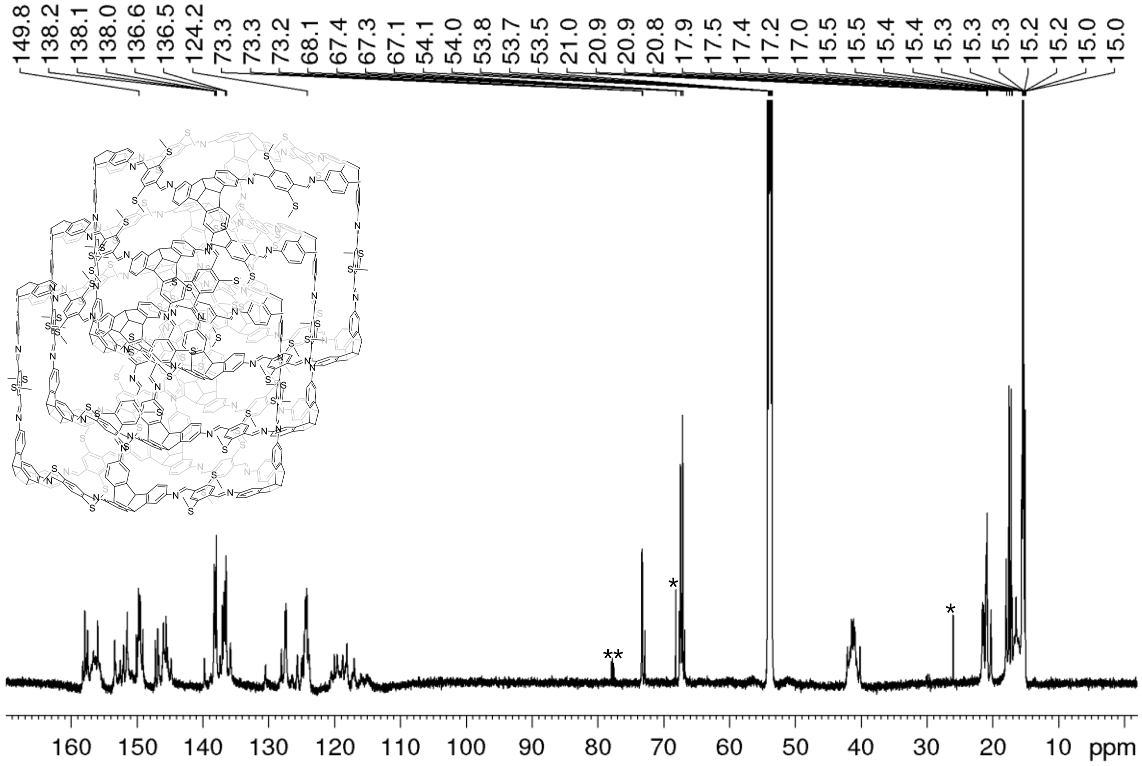

Figure 214. ^{13}C NMR (176 MHz, CD_2Cl_2) spectrum of **(SMe-cube)$_3$**. **$CDCl_3$, *THF.

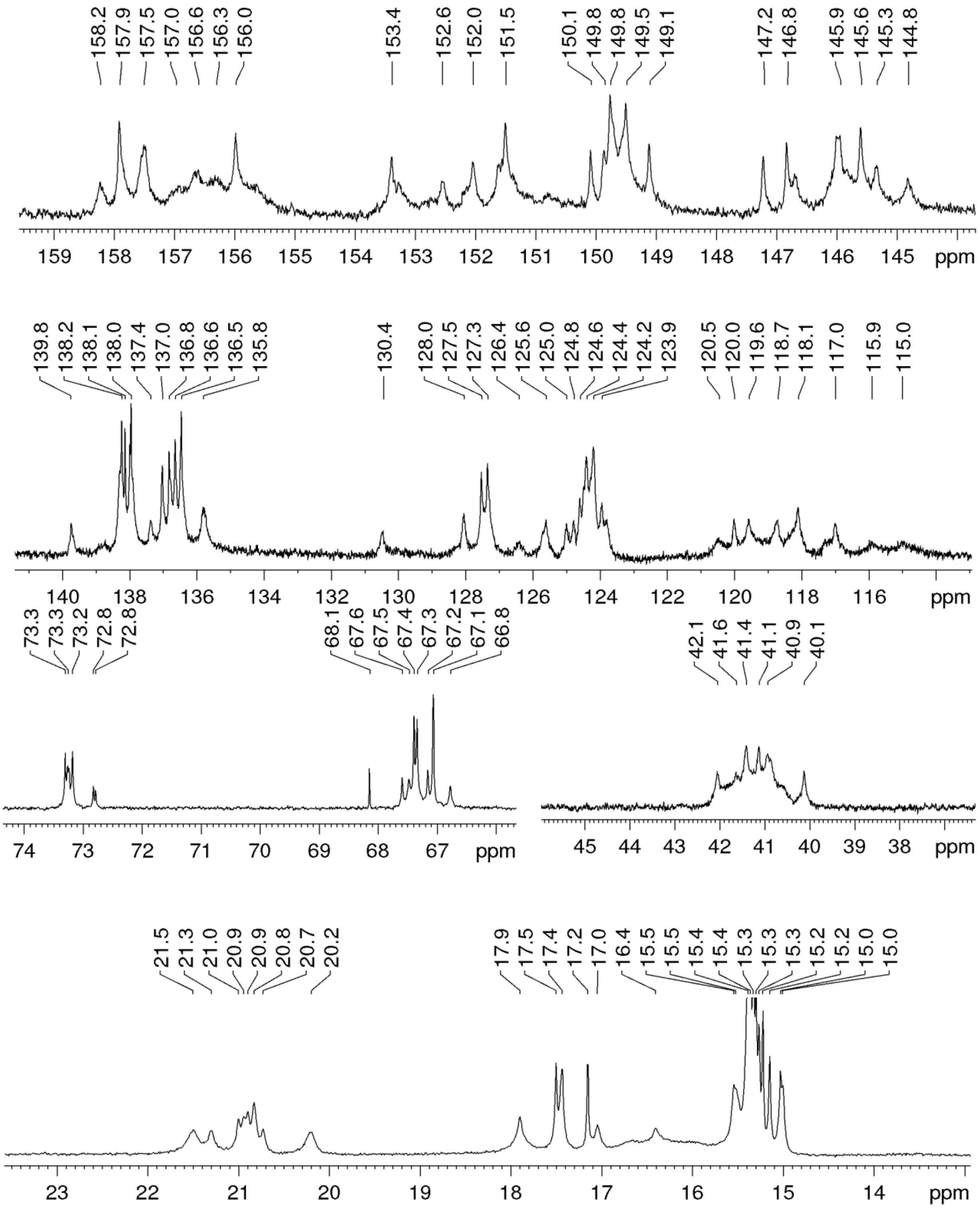

Figure 215. ^{13}C NMR (176 MHz, CD_2Cl_2) spectrum of **(SMe-cube)3**.

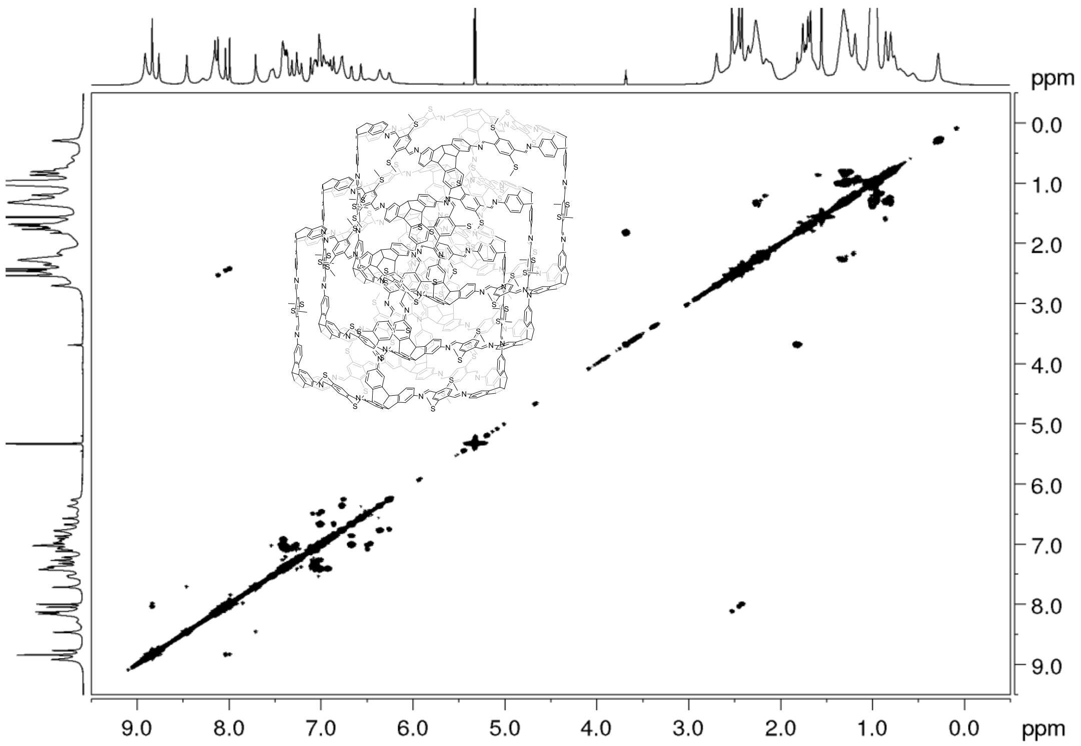

Figure 216. ^{1}H-^{1}H COSY NMR spectrum (700 MHz, CD_2Cl_2) of **(SMe-cube)$_3$**.

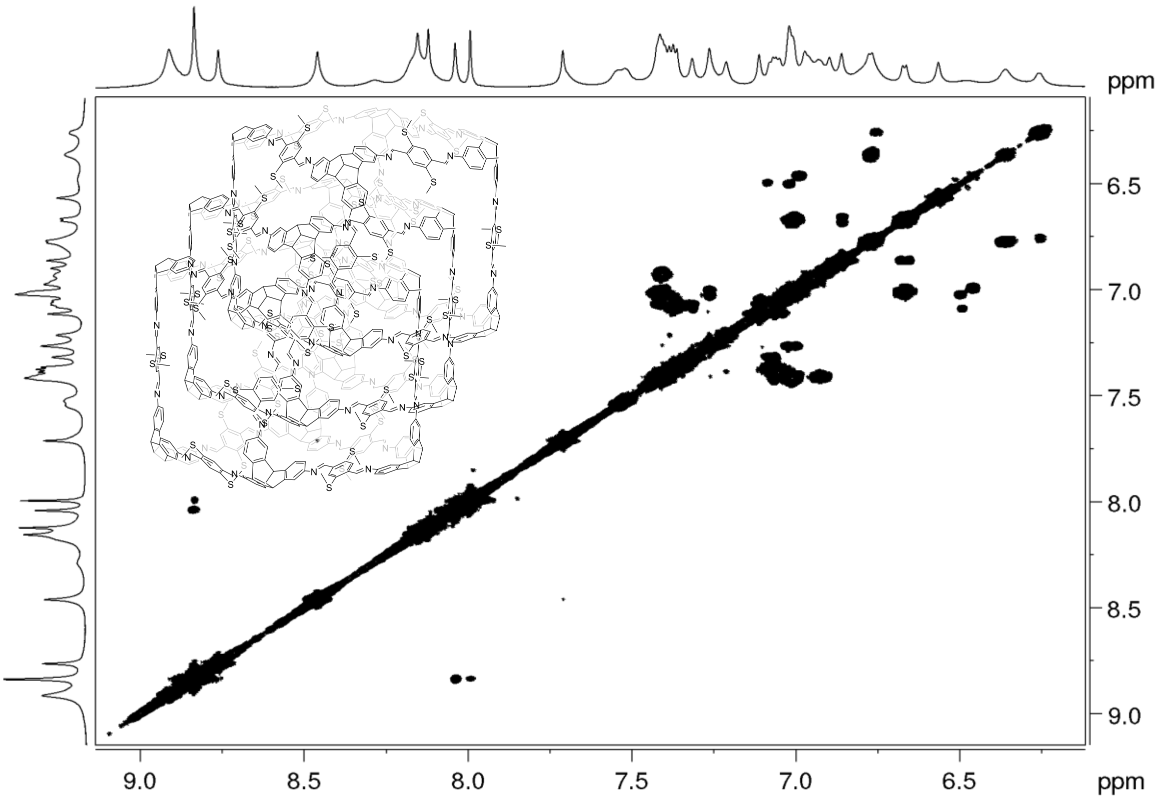

Figure 217. Partial ^{1}H-^{1}H COSY NMR spectrum (700 MHz, CD_2Cl_2) of **(SMe-cube)$_3$**.

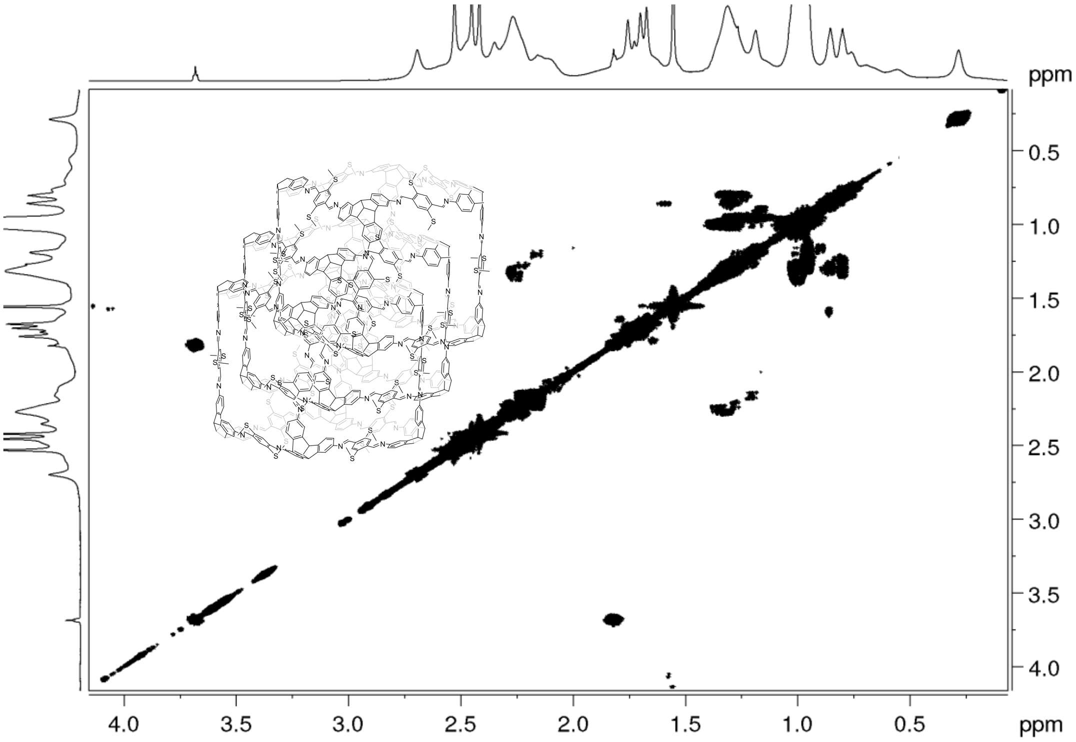

Figure 218. Partial ^{1}H-^{1}H COSY NMR spectrum (700 MHz, CD_2Cl_2) of **(SMe-cube)$_3$**.

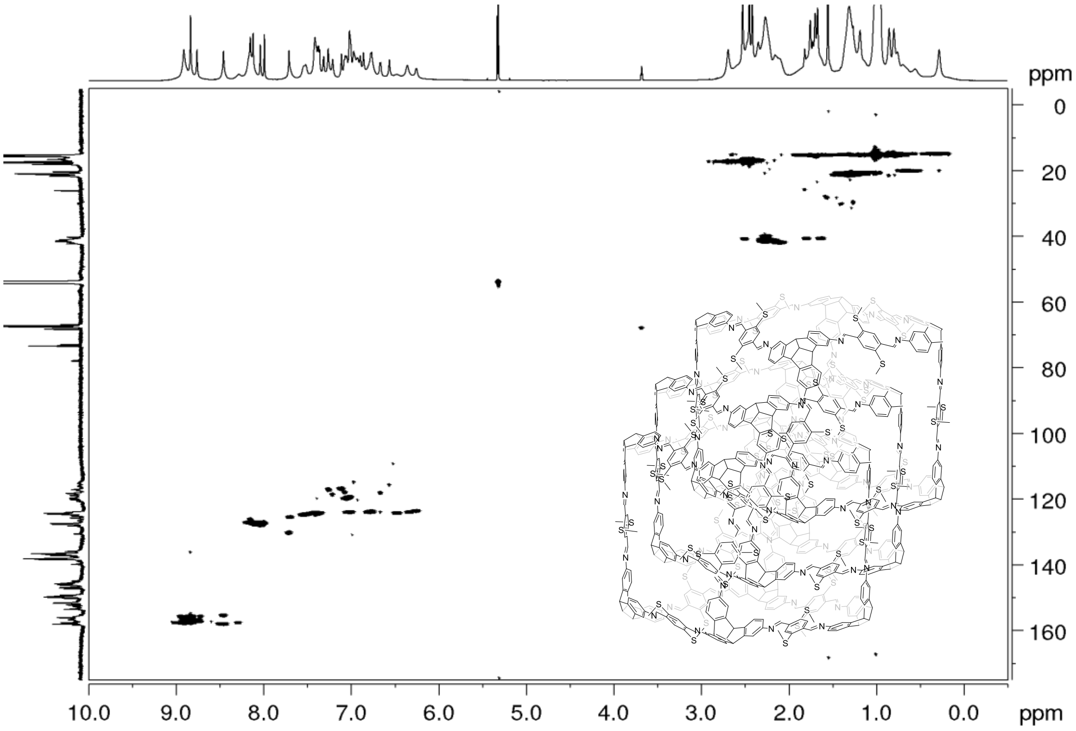

Figure 219. ^{1}H-^{13}C HSQC NMR (700 MHz and 176 MHz, CD_2Cl_2) spectrum of **(SMe-cube)$_3$**.

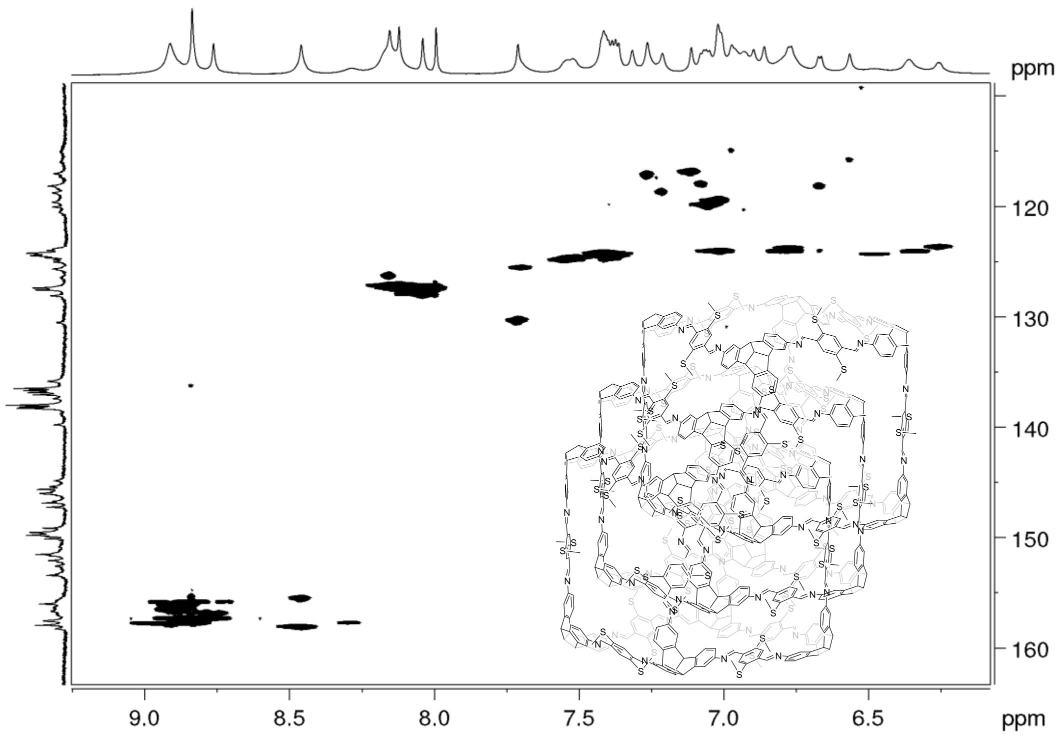

Figure 220. Partial ^{1}H-^{13}C HSQC NMR (700 MHz and 176 MHz, CD_2Cl_2) spectrum of **(SMe-cube)$_3$**.

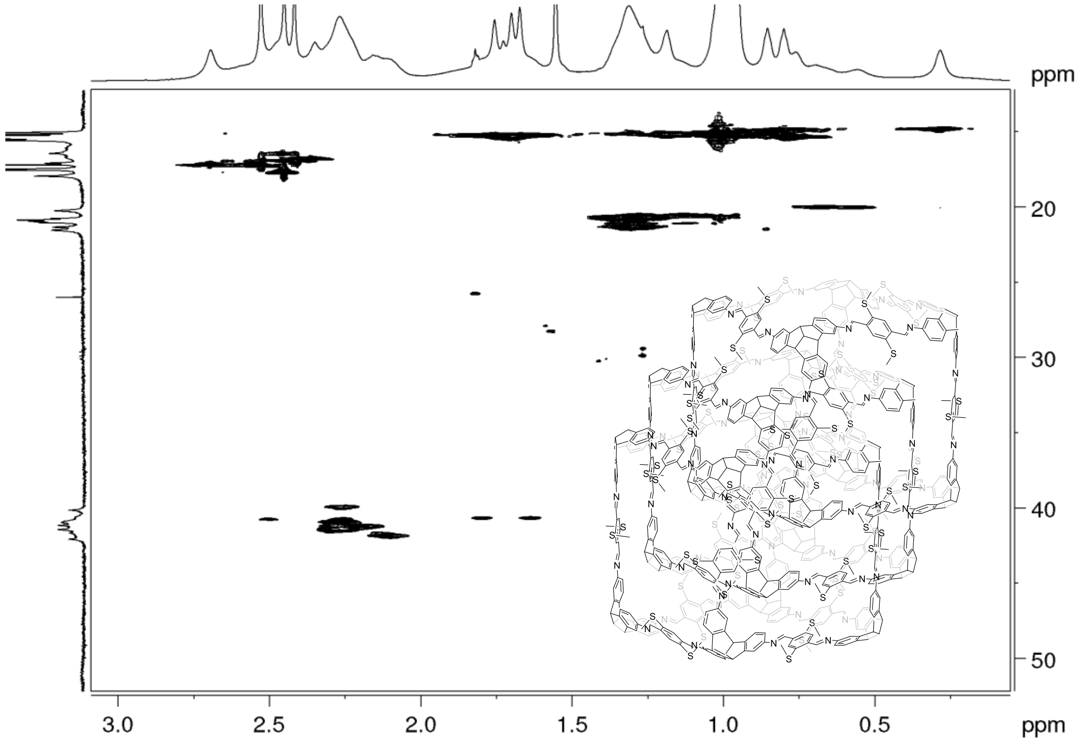

Figure 221. Partial ^{1}H-^{13}C HSQC NMR (700 MHz and 176 MHz, CD_2Cl_2) spectrum of **(SMe-cube)$_3$**.

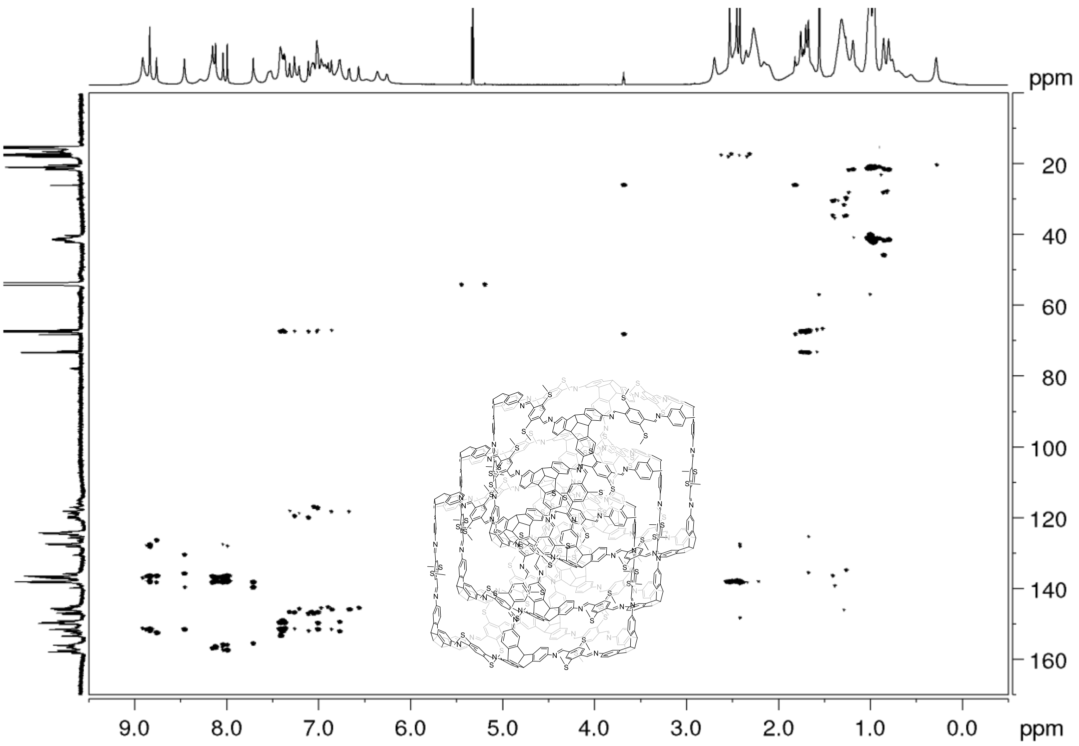

Figure 222. ^{1}H-^{13}C HMBC NMR (700 MHz and 176 MHz, CD_2Cl_2) spectrum of **(SMe-cube)$_3$**.

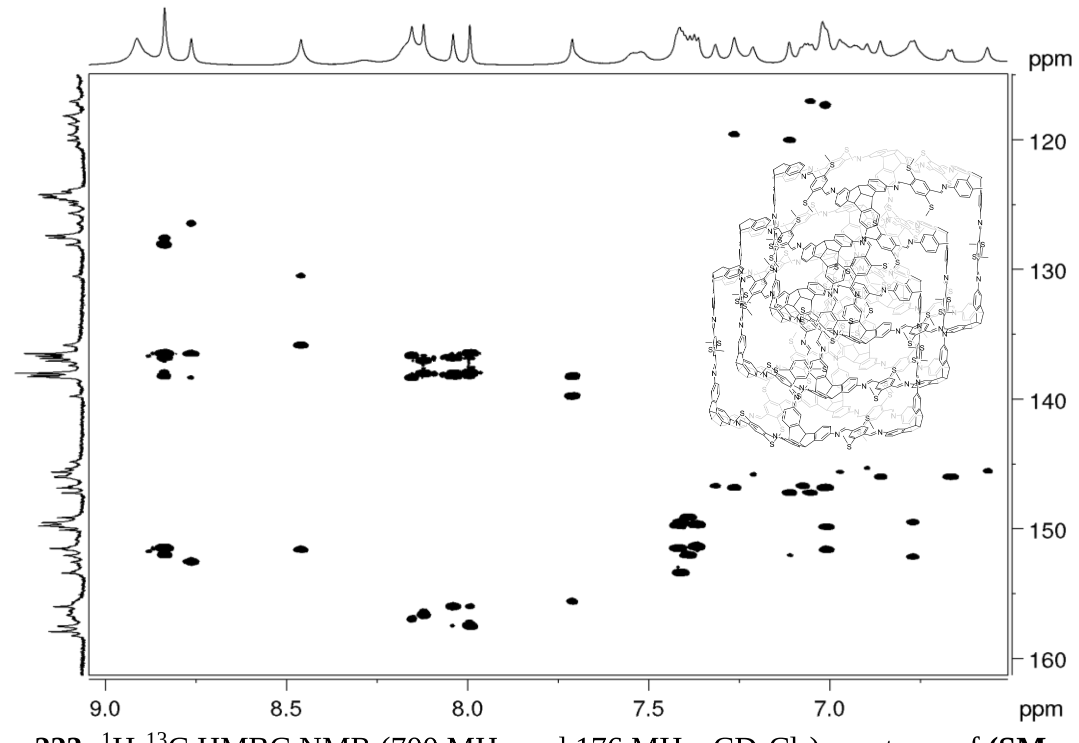

Figure 223. ^{1}H-^{13}C HMBC NMR (700 MHz and 176 MHz, CD_2Cl_2) spectrum of **(SMe-cube)$_3$**.

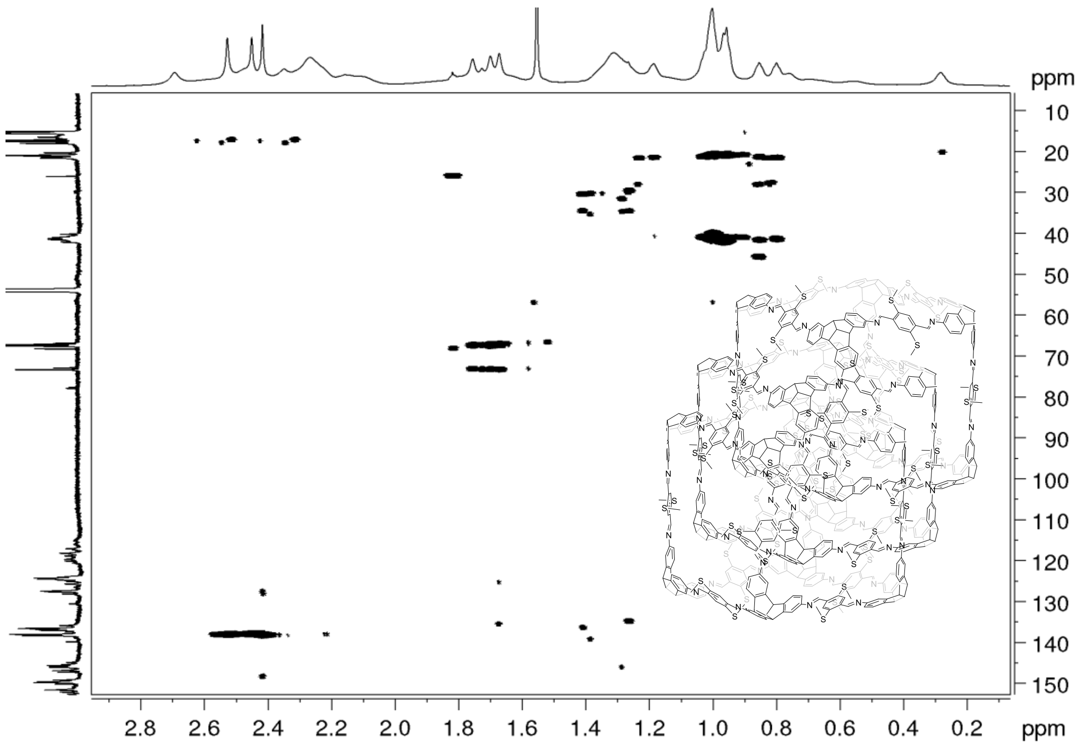

Figure 224. Partial ^{1}H-^{13}C HMBC NMR (700 MHz and 176 MHz, CD_2Cl_2) spectrum of **(SMe-cube)$_3$**.

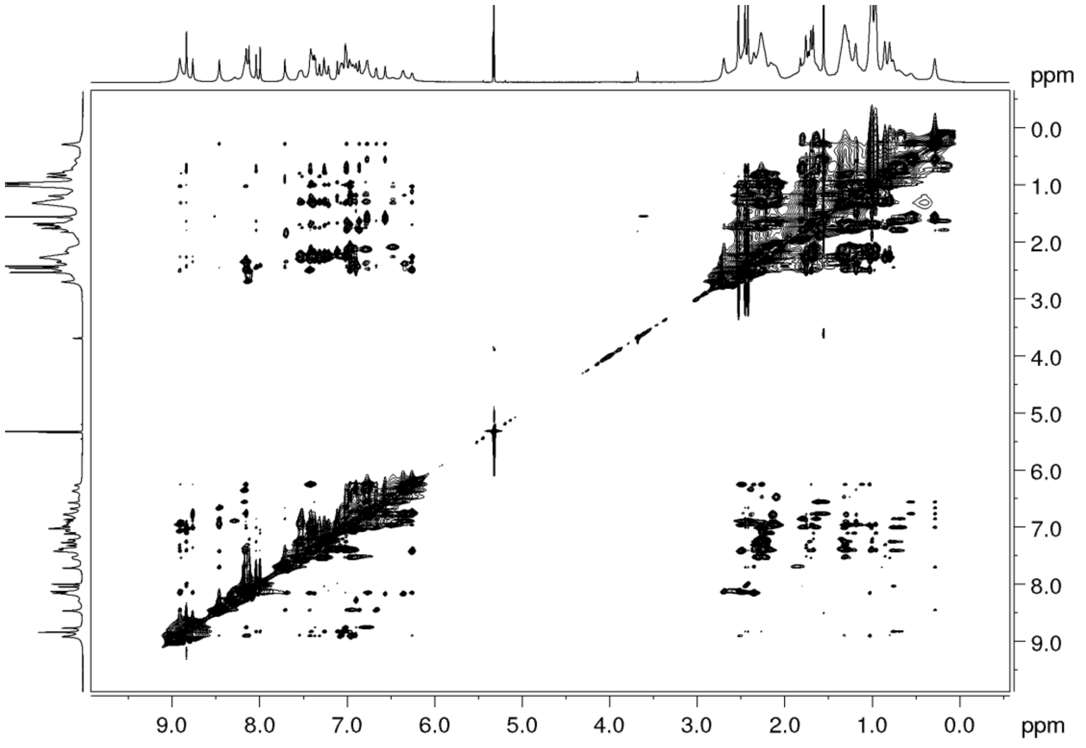

Figure 225. ^{1}H-^{1}H NOESY NMR (700 MHz, CD_2Cl_2) spectrum of **(SMe-cube)$_3$**.

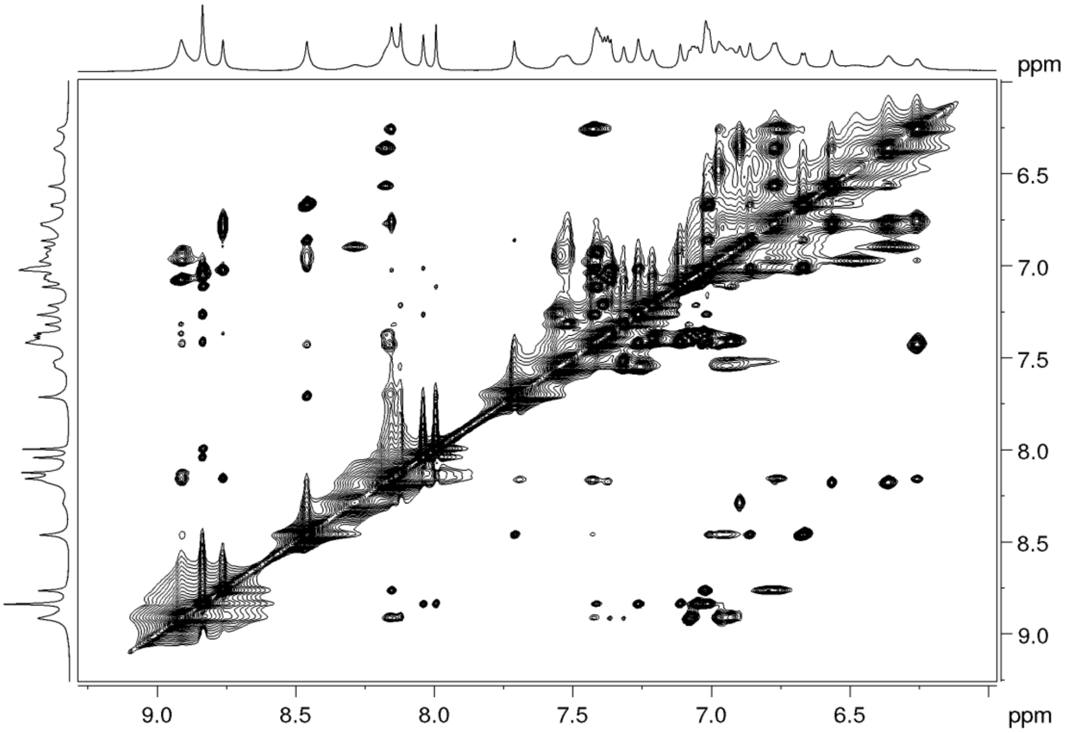

Figure 226. Partial ^{1}H-^{1}H NOESY NMR (700 MHz, CD_2Cl_2) spectrum of **(SMe-cube)$_3$**.

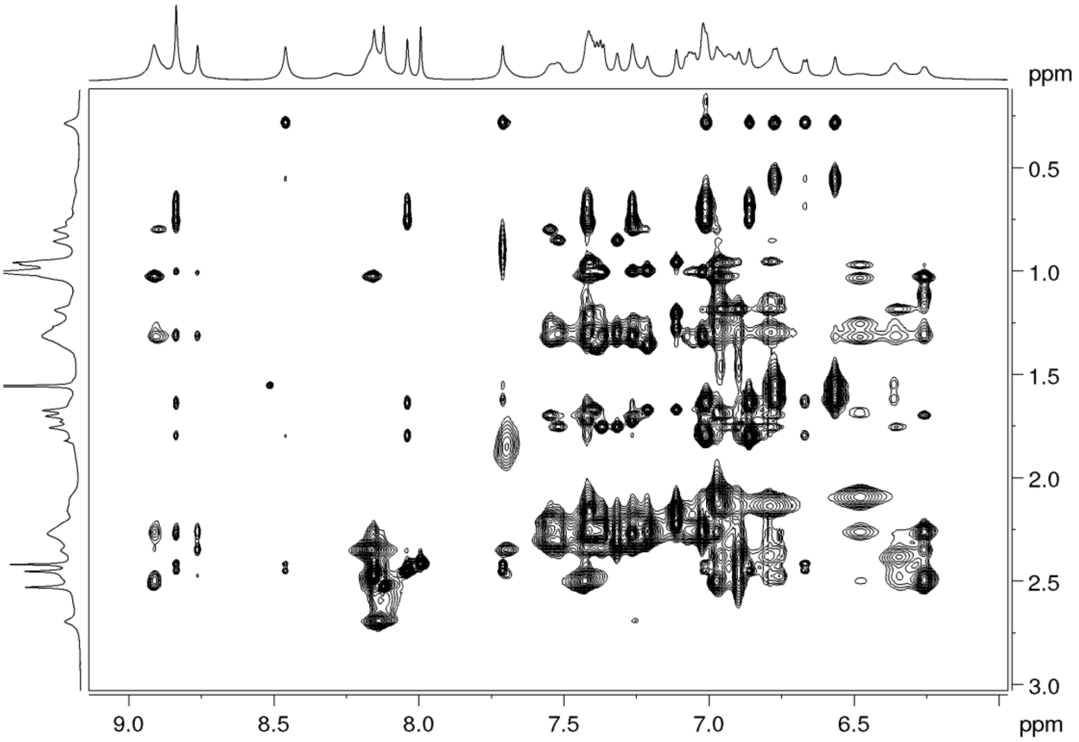

Figure 227. Partial ^{1}H-^{1}H NOESY NMR (700 MHz, CD_2Cl_2) spectrum of **(SMe-cube)$_3$**

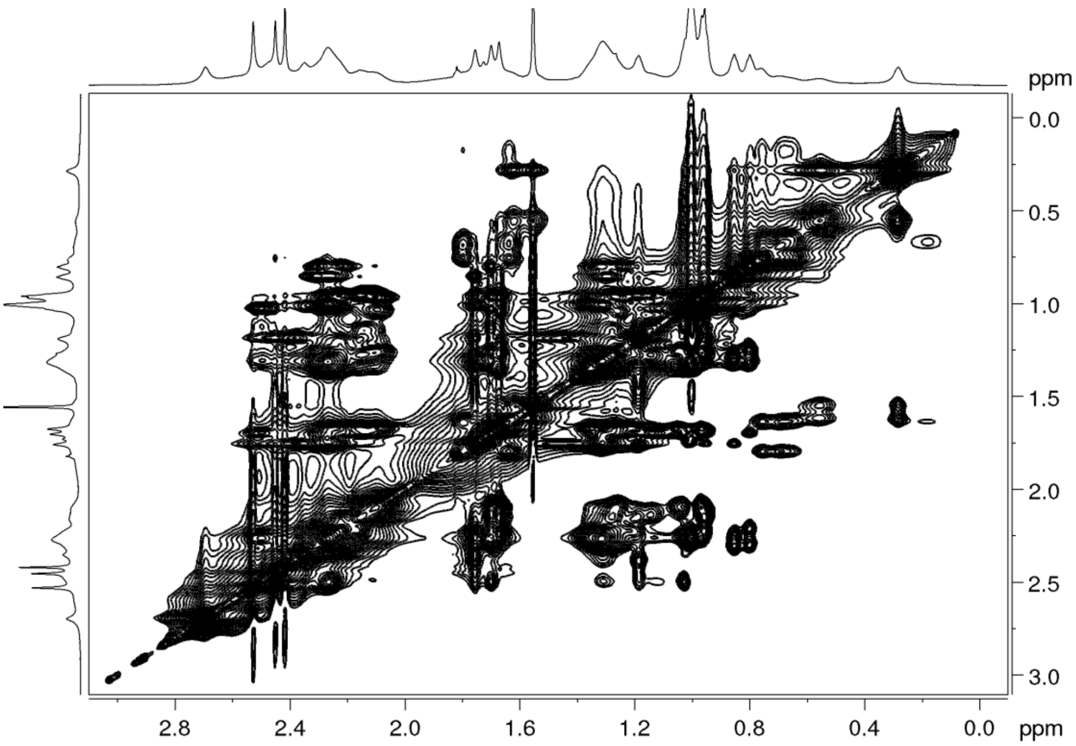

Figure 228. Partial ^1H-^1H NOESY NMR (700 MHz, CD_2Cl_2) spectrum of **(SMe-cube)$_3$**

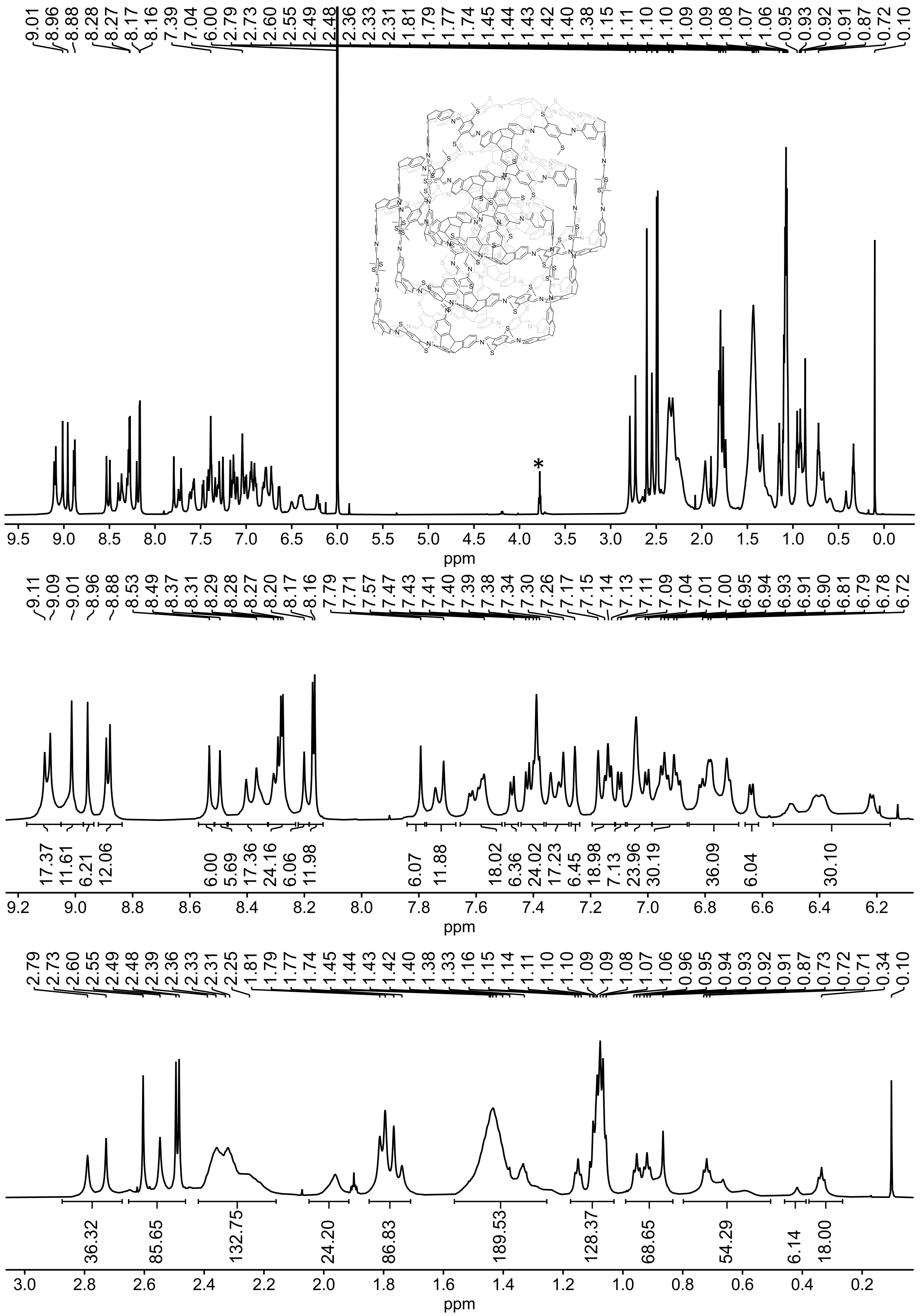

Figure 229. ^{1}H NMR (700 MHz, 410 K, $C_2D_2Cl_4$) spectrum of **(SMe-cube)$_3$**, .*THF

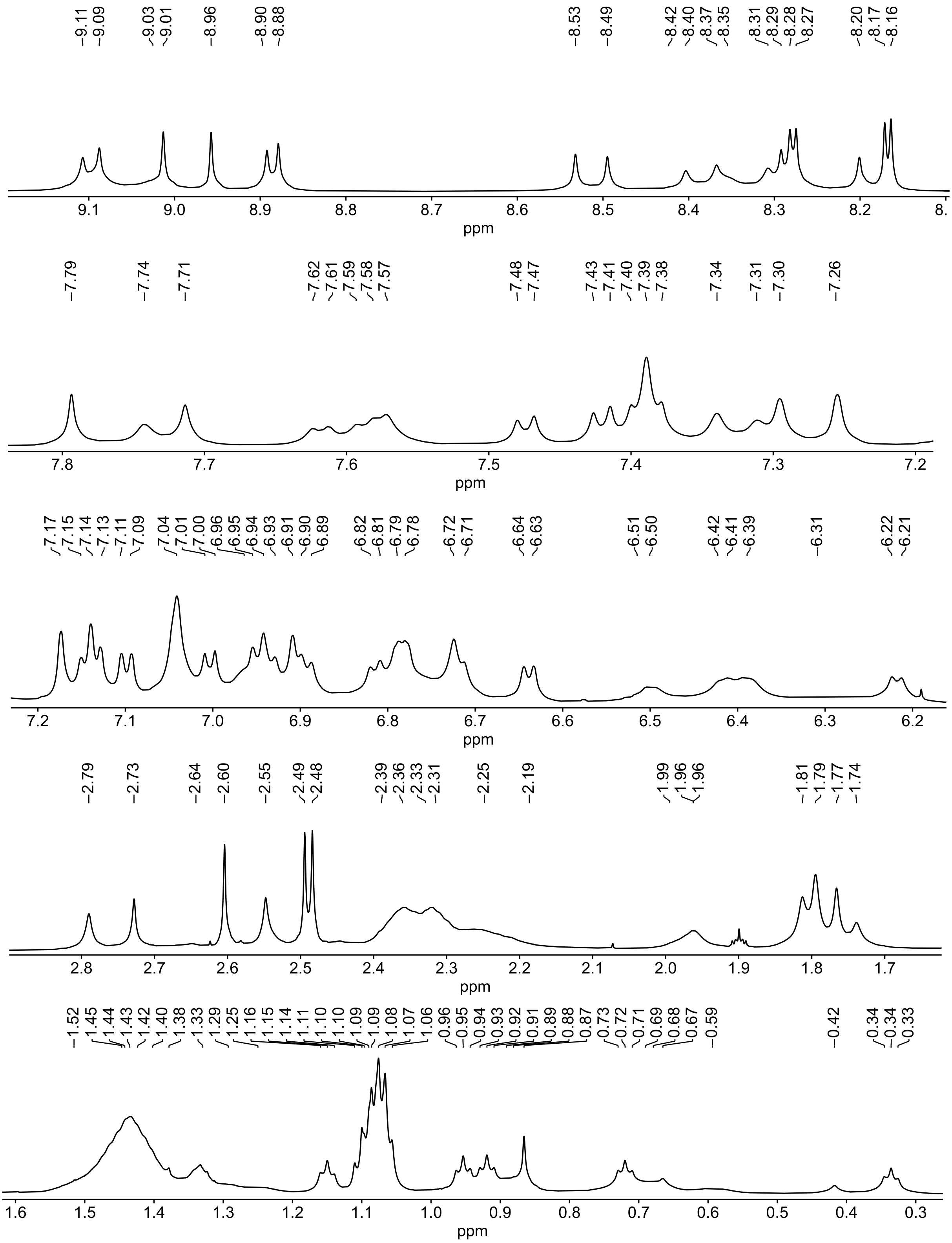

Figure 230. Expanded ^{1}H NMR (700 MHz, 410 K, $C_2D_2Cl_4$) spectrum of **(SMe-cube)$_3$**.

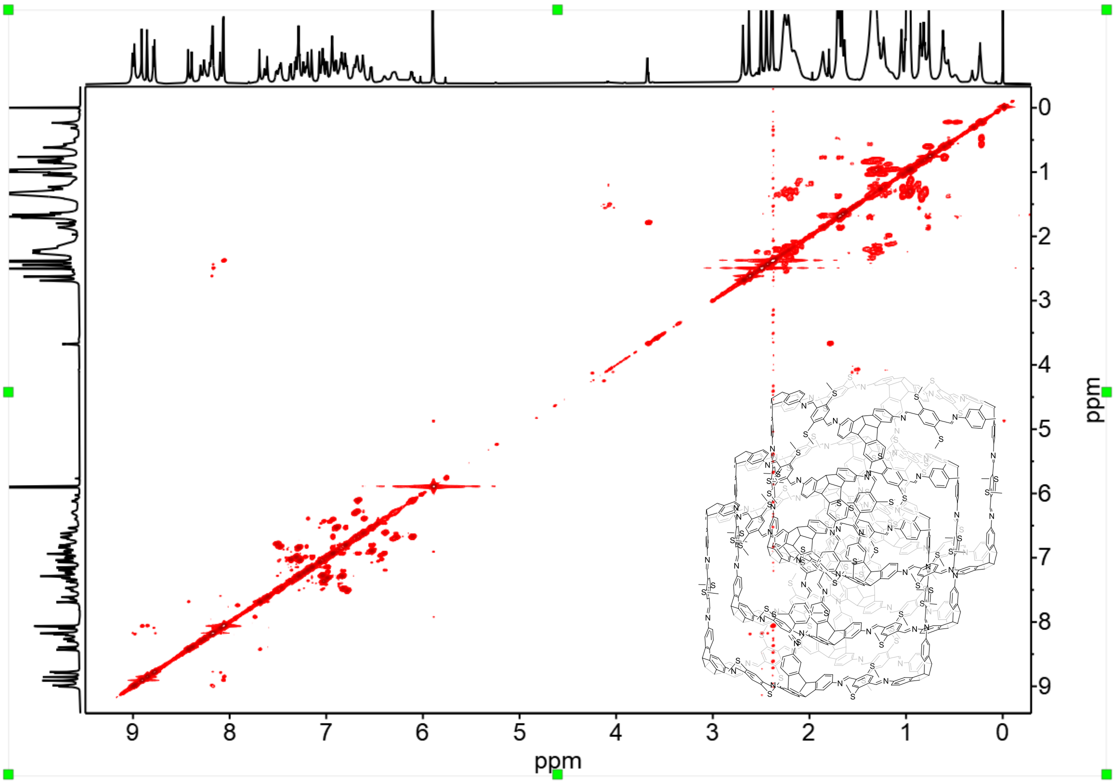

Figure 231. ^{1}H-^{1}H COSY NMR spectrum (700 MHz, $C_2D_2Cl_4$, **410 K**) of **(SMe-cube)$_3$**.

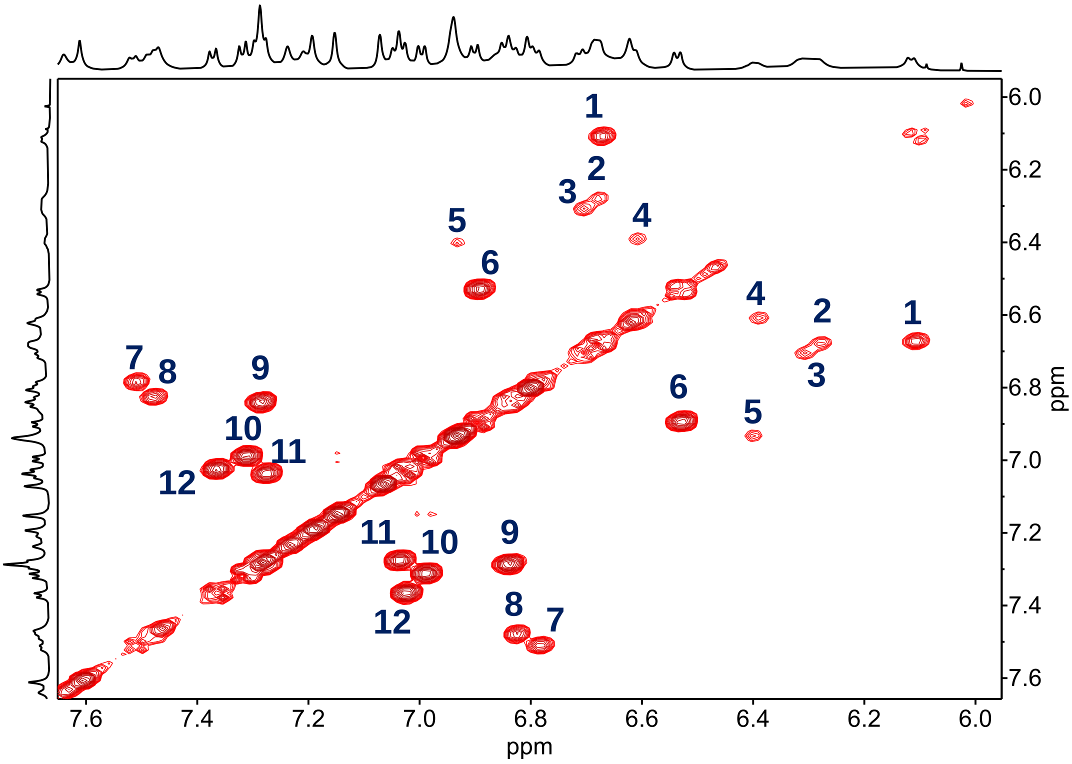

Figure 232. Partial ^{1}H-^{1}H COSY NMR spectrum (700 MHz, $C_2D_2Cl_4$, 410 K) of **(SMe-cube)$_3$**.

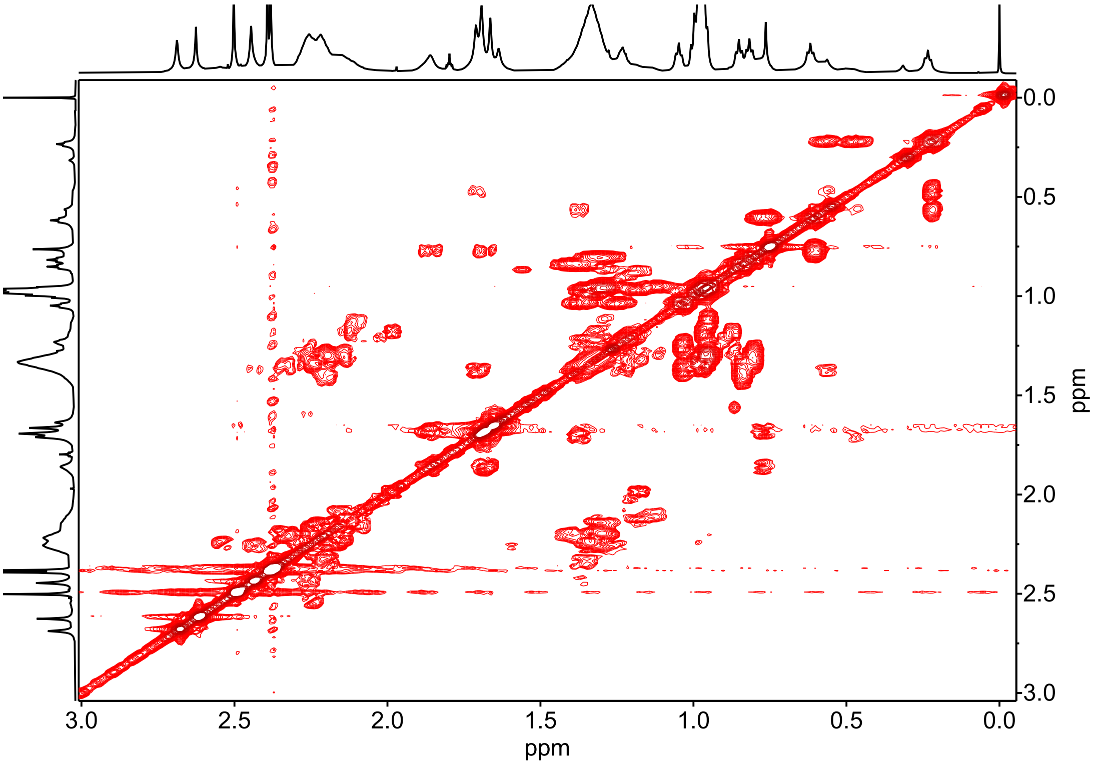

Figure 233. Partial ^{1}H-^{1}H COSY NMR spectrum (700 MHz, $C_2D_2Cl_4$, 410 K) of **(SMe-cube)$_3$**.

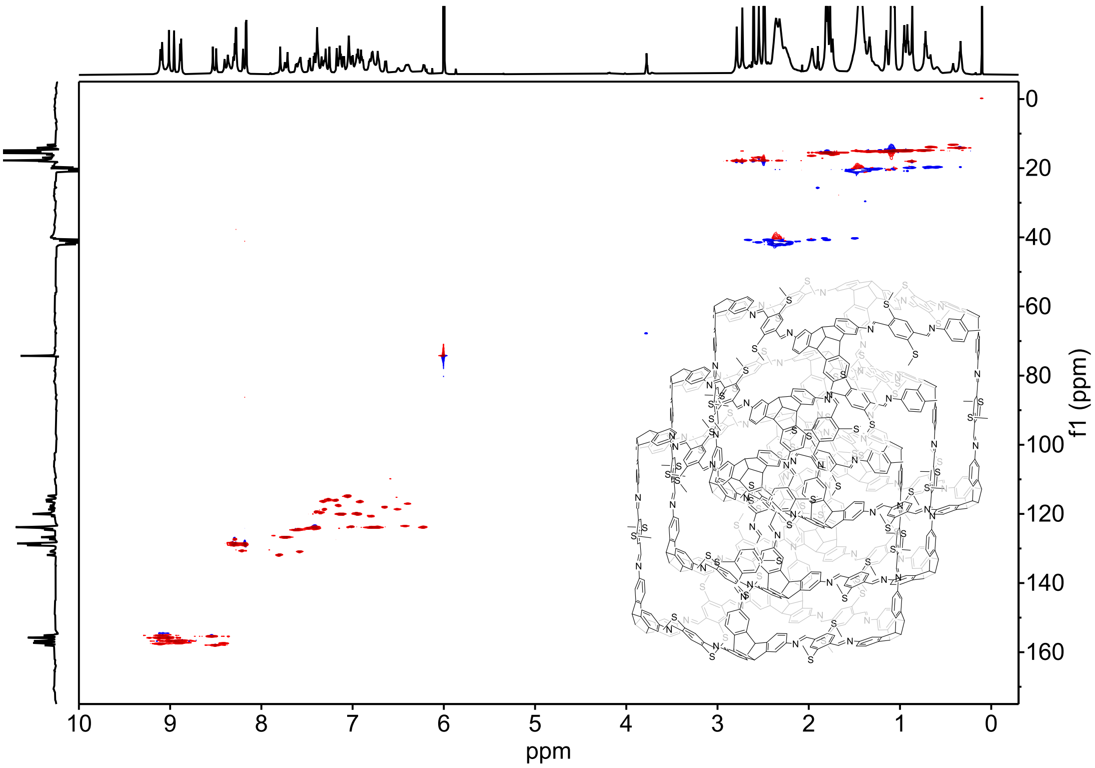

Figure 234. ^{1}H-^{13}C HSQC NMR (700 MHz and 176 MHz, $C_2D_2Cl_4$, 410 K) spectrum of **(SMe-cube)$_3$**.

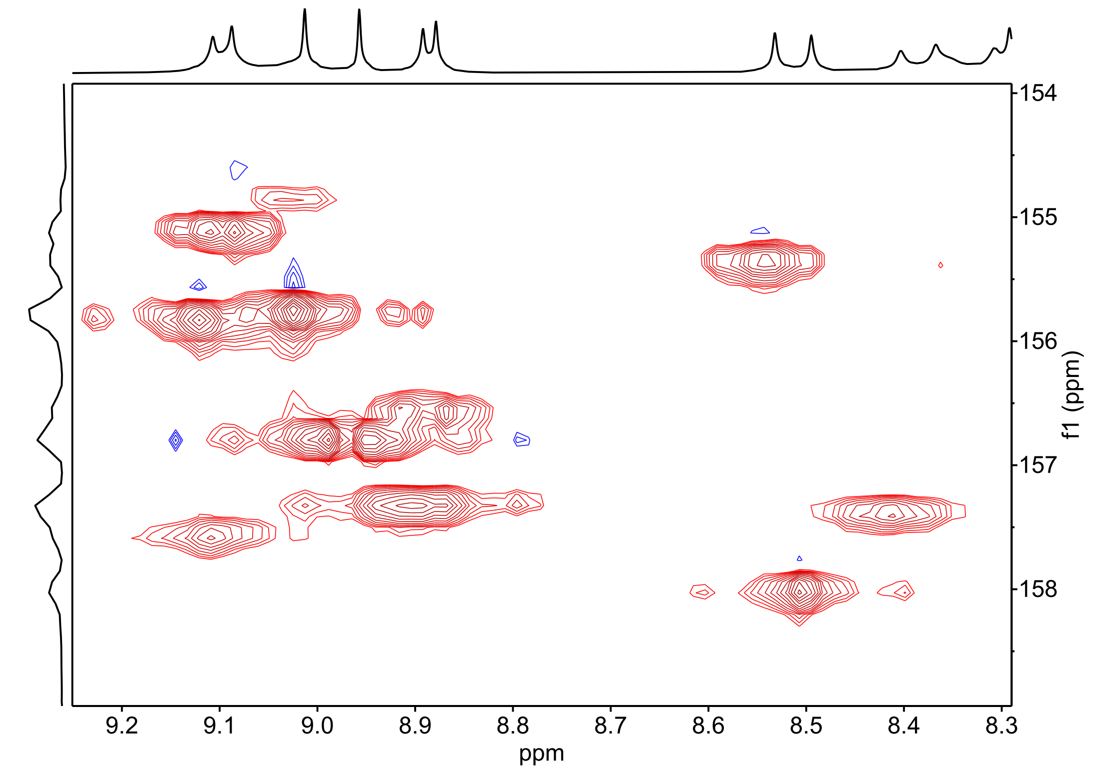

Figure 235. Partial ^{1}H-^{13}C HSQC NMR (700 MHz and 176 MHz, $C_2D_2Cl_4$, 410 K) spectrum of **(SMe-cube)$_3$**.

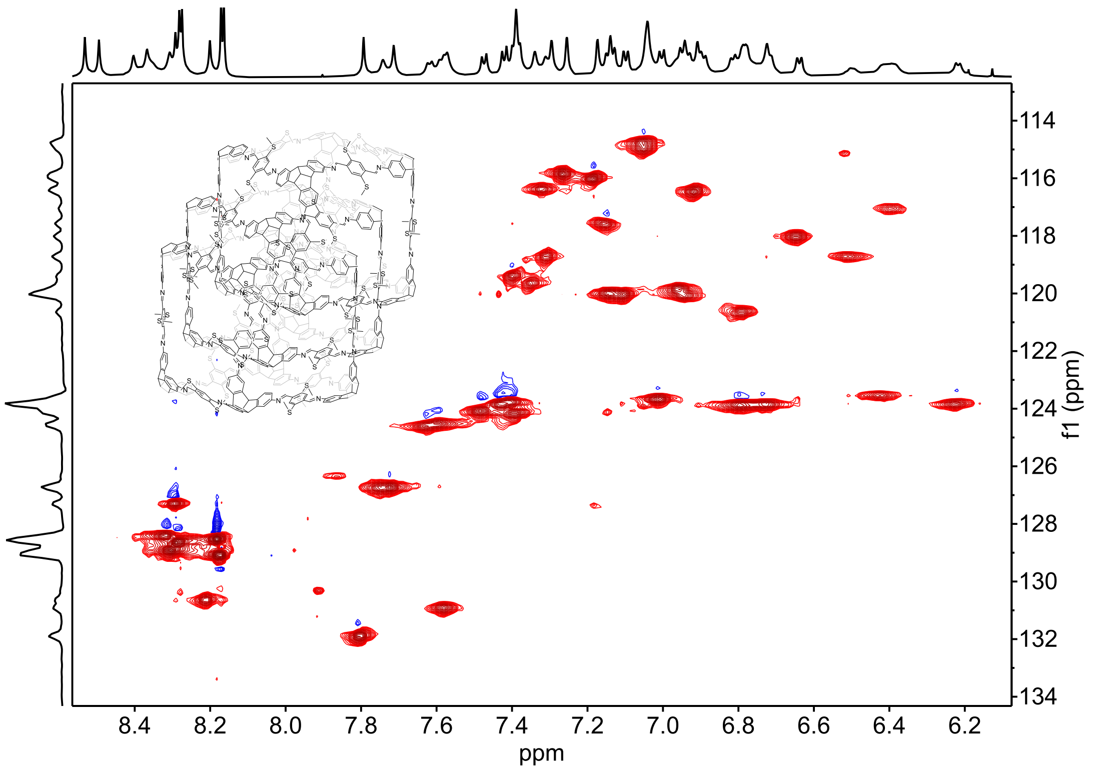

Figure 236. Partial ^{1}H-^{13}C HSQC NMR (700 MHz and 176 MHz, $C_2D_2Cl_4$, 410 K) spectrum of **(SMe-cube)$_3$**.

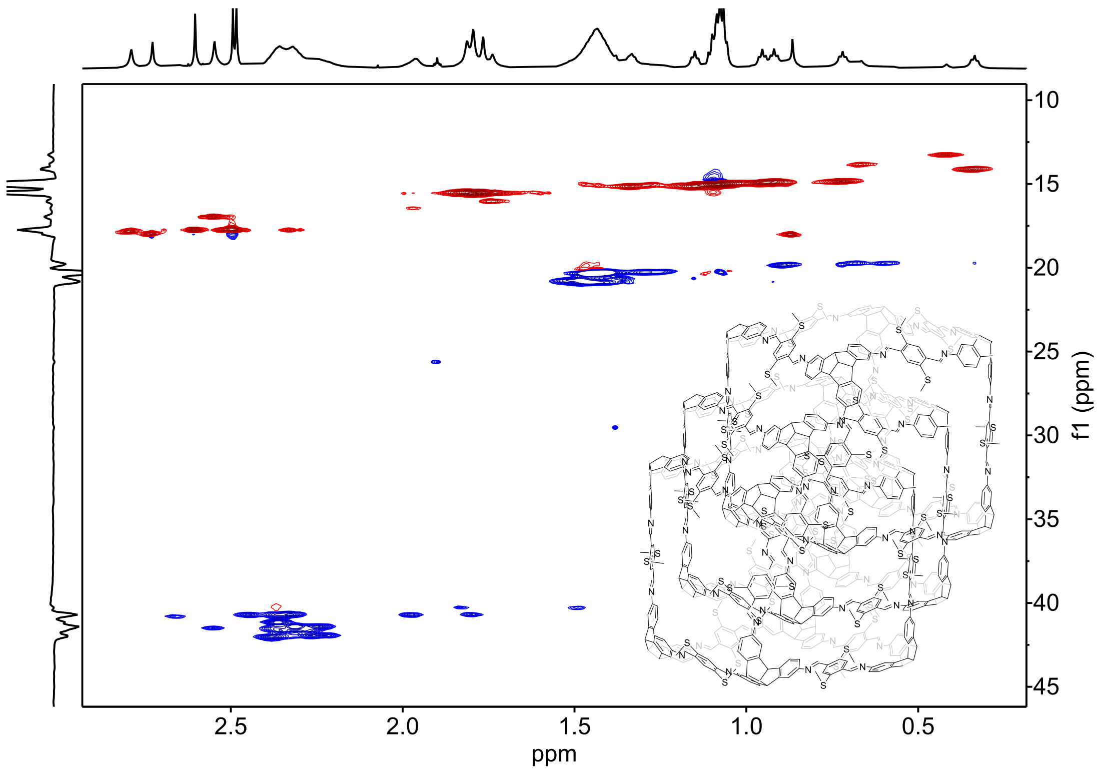

Figure 237. Partial ^{1}H-^{13}C HSQC NMR (700 MHz and 176 MHz, $C_2D_2Cl_4$, 410 K) spectrum of **(SMe-cube)$_3$**.

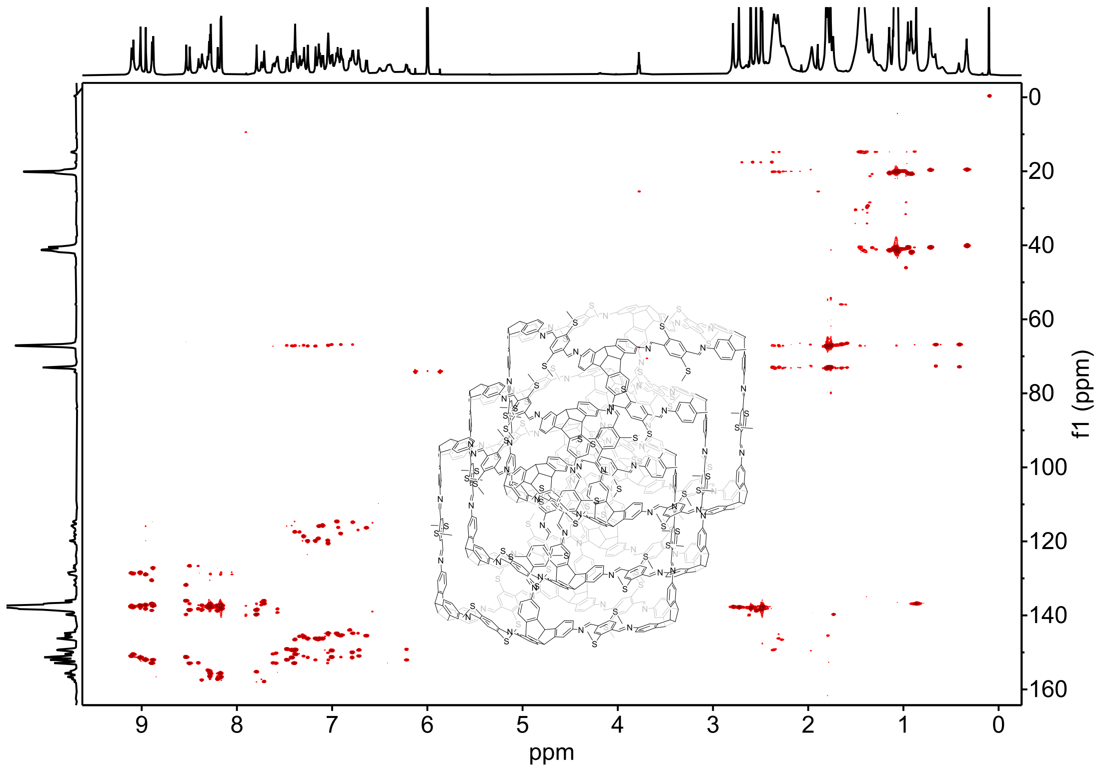

Figure 238. ^{1}H-^{13}C HMBC NMR (700 MHz and 176 MHz, $C_2D_2Cl_4$, 410 K) spectrum of **(SMe-cube)$_3$**.

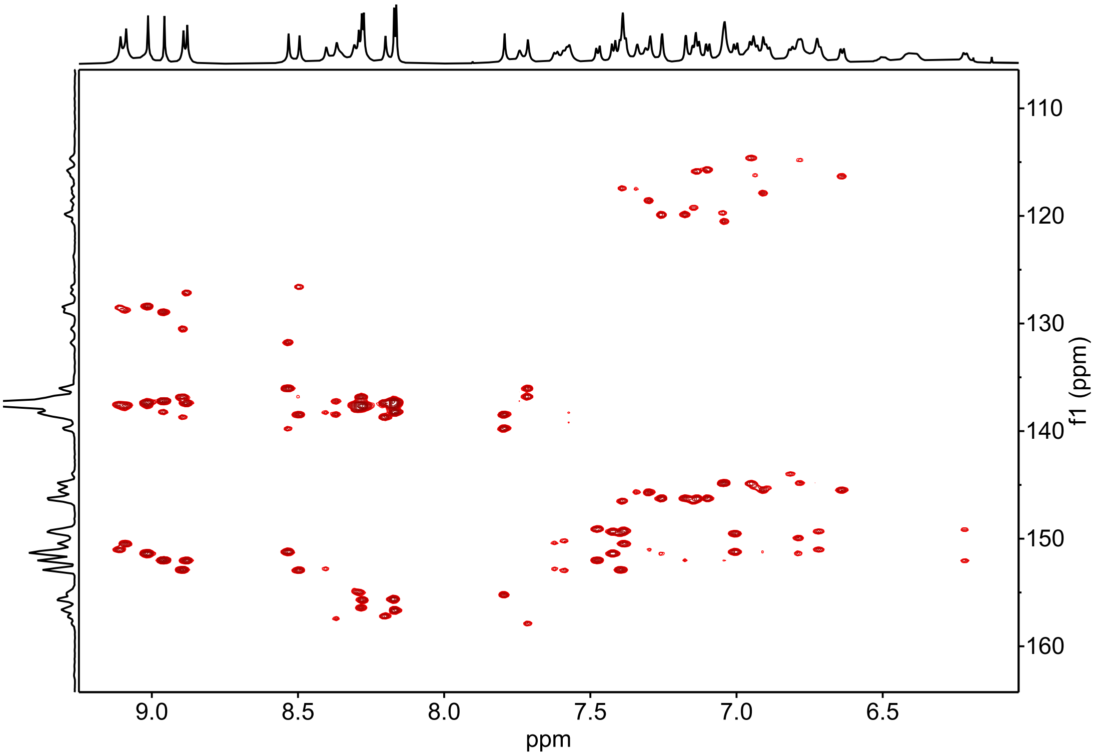

Figure 239. Partial ^{1}H-^{13}C HMBC NMR (700 MHz and 176 MHz, $C_2D_2Cl_4$, 410 K) spectrum of **(SMe-cube)$_3$**.

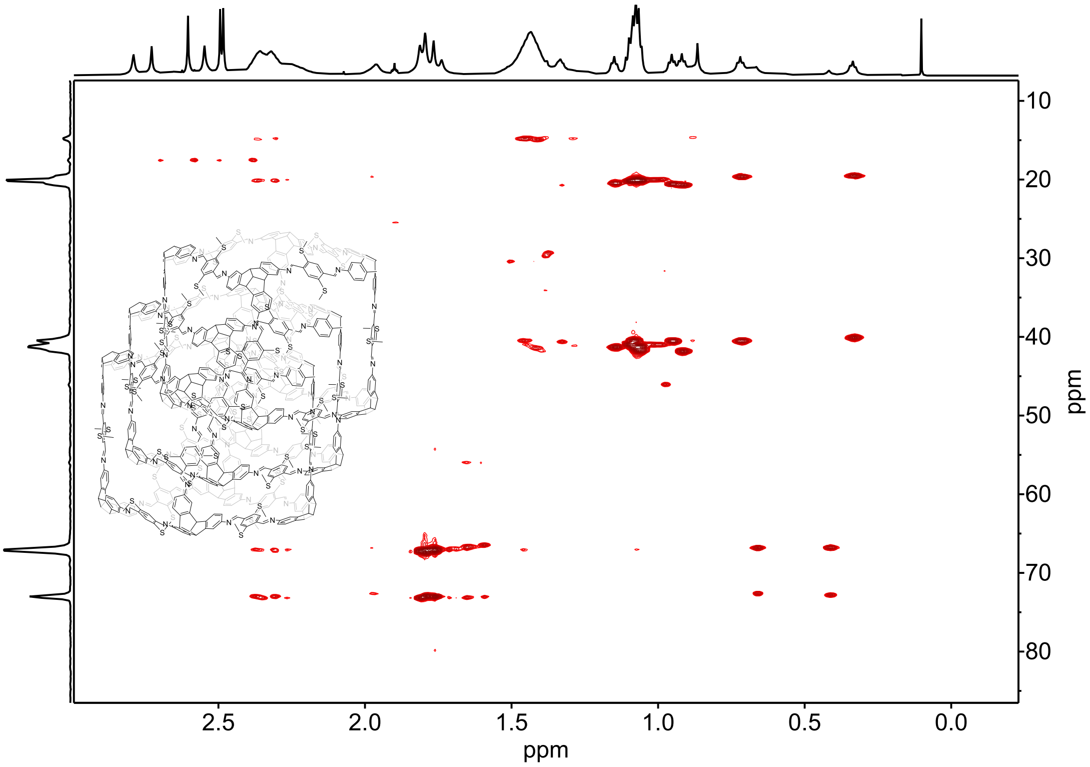

Figure 240. Partial ^{1}H-^{13}C HMBC NMR (700 MHz and 176 MHz, $C_2D_2Cl_4$, 410 K) spectrum of **(SMe-cube)$_3$**.

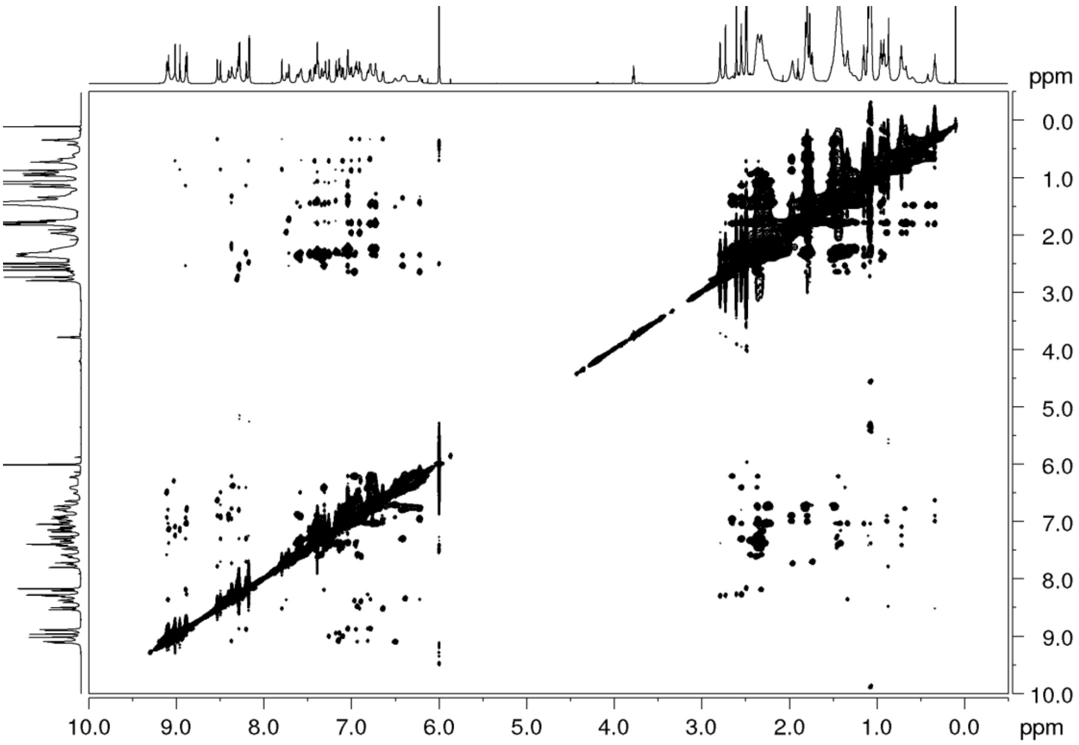

Figure 241. ¹H-¹H NOESY NMR (700 MHz, $C_2D_2Cl_4$, 410 K) spectrum of **(SMe-cube)$_3$**.

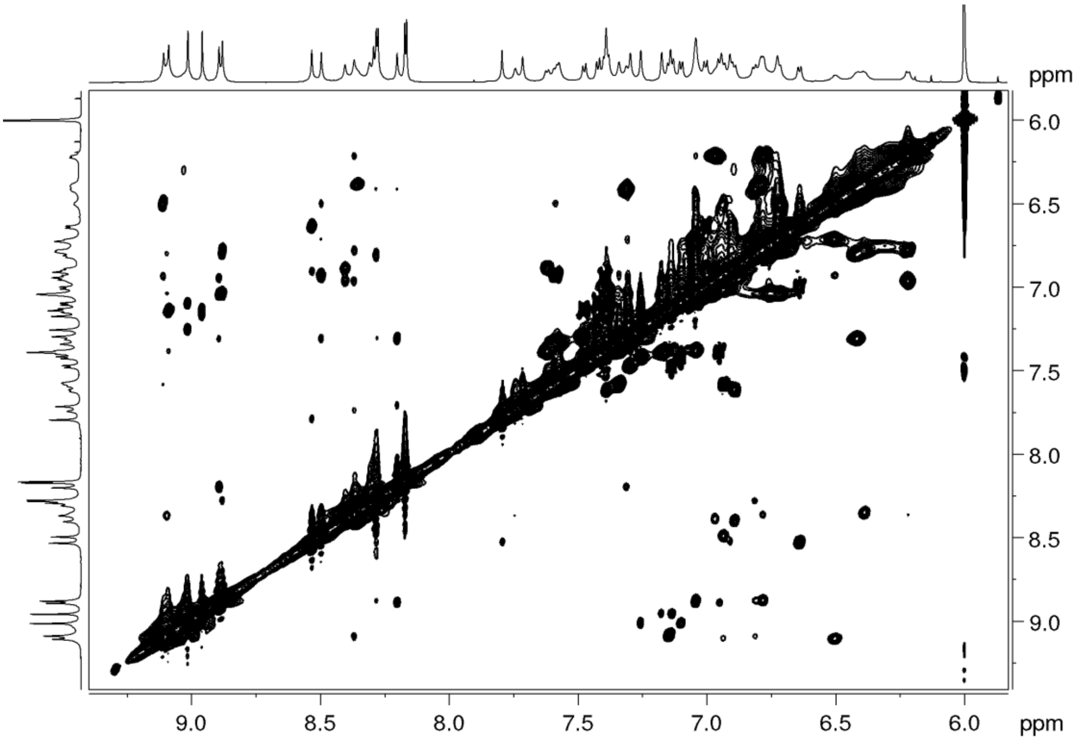

Figure 242. Partial ¹H-¹H NOESY NMR (700 MHz, $C_2D_2Cl_4$, 410 K) spectrum of **(SMe-cube)$_3$**.

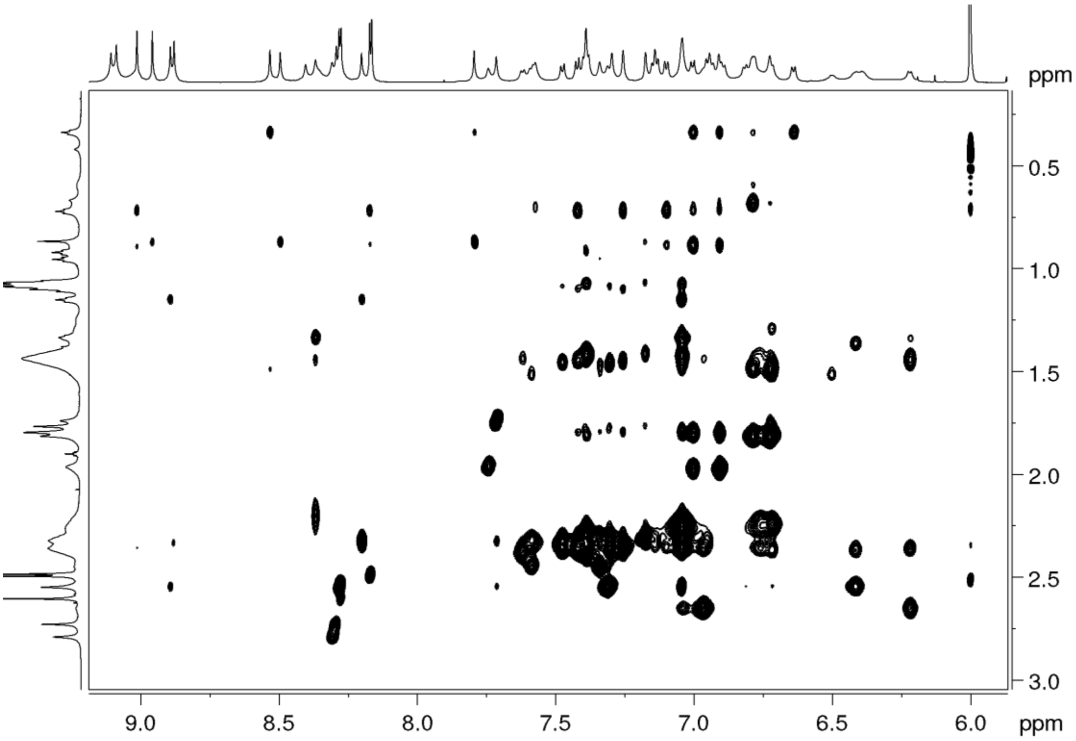

Figure 243. Partial ^{1}H-^{1}H NOESY NMR (700 MHz, $C_2D_2Cl_4$, 410 K) spectrum of **(SMe-cube)$_3$**.

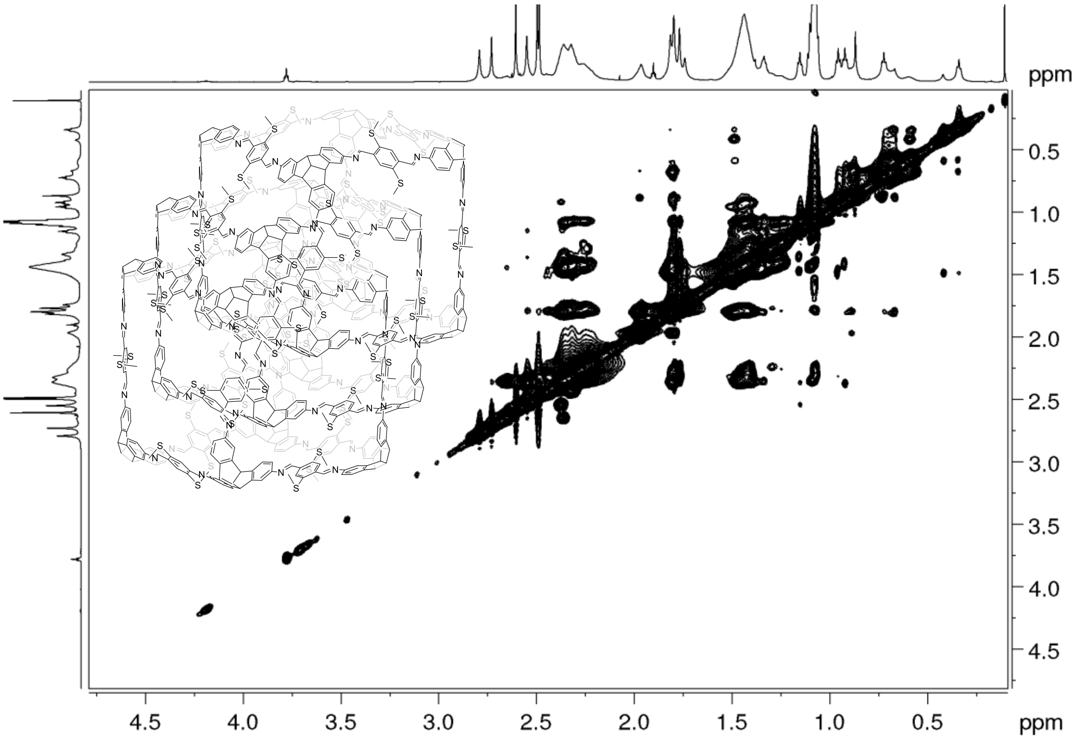

Figure 244. Partial ^{1}H-^{1}H NOESY NMR (700 MHz, $C_2D_2Cl_4$, 410 K) spectrum of **(SMe-cube)$_3$**

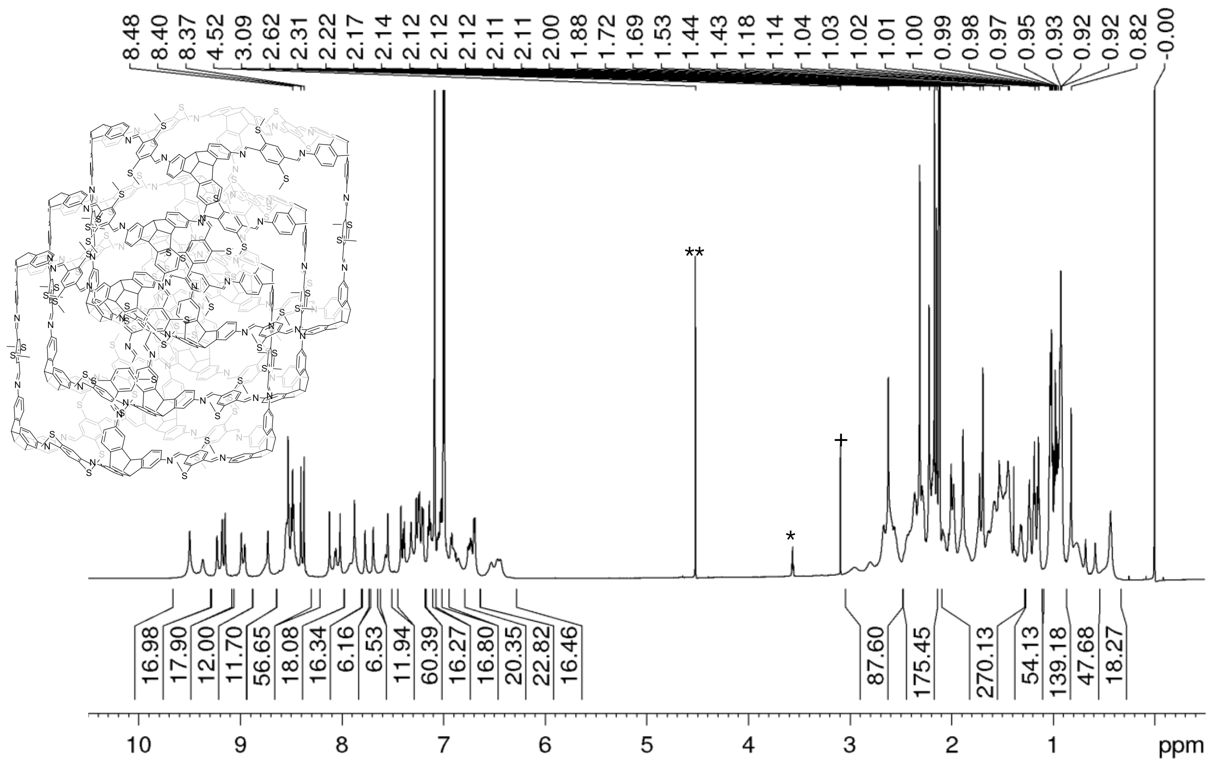

Figure 245. ^{1}H NMR (700 MHz, Toluene-d$_8$, 380 K) spectrum of **^{15}N labelled** *(**SMe-cube)$_3$**. **DCM, *THF. $^{+}$MeOH.

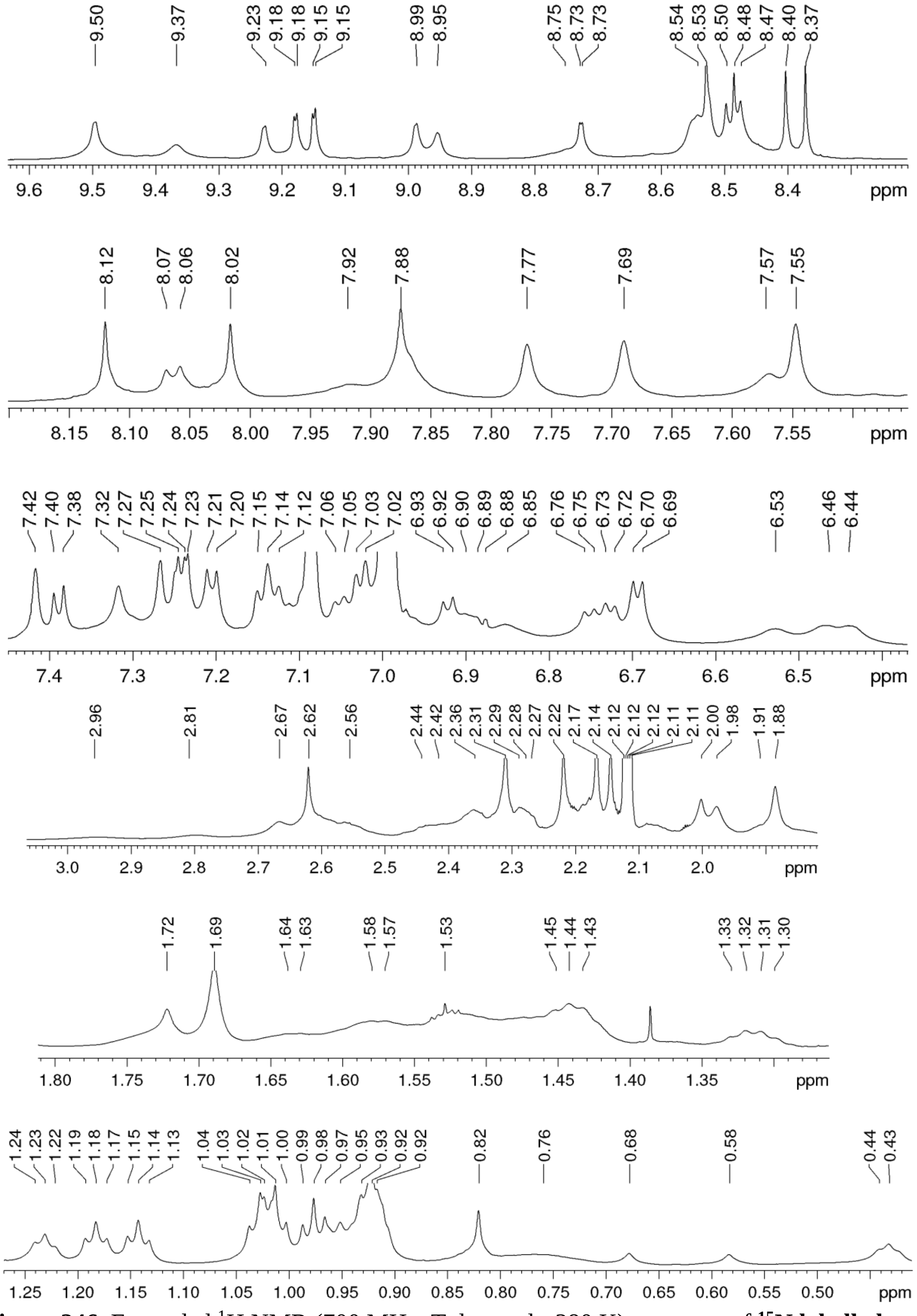

Figure 246. Expanded ^{1}H NMR (700 MHz, Toluene-d$_8$, 380 K) spectrum of **^{15}N labelled** *(**SMe-cube)$_3$**.

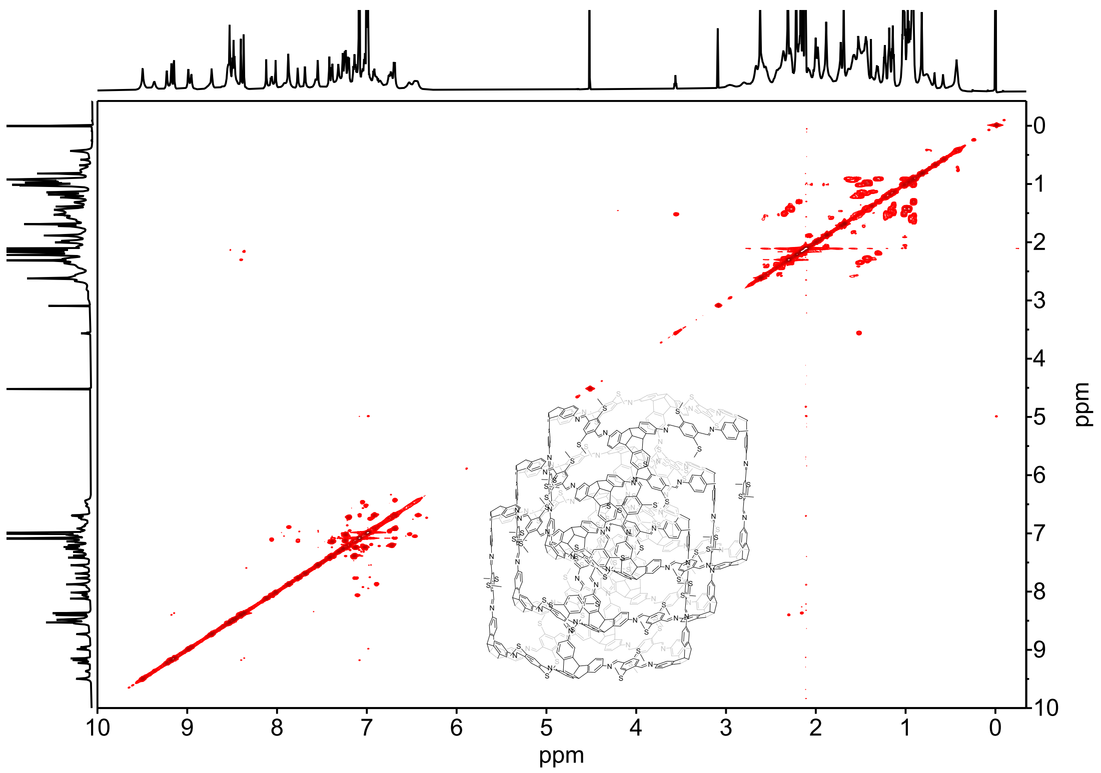

Figure 247. ^{1}H-^{1}H COSY NMR spectrum (700 MHz, Toluene-d$_8$, 380 K) of **^{15}N labelled *(SMe-cube)$_3$**..

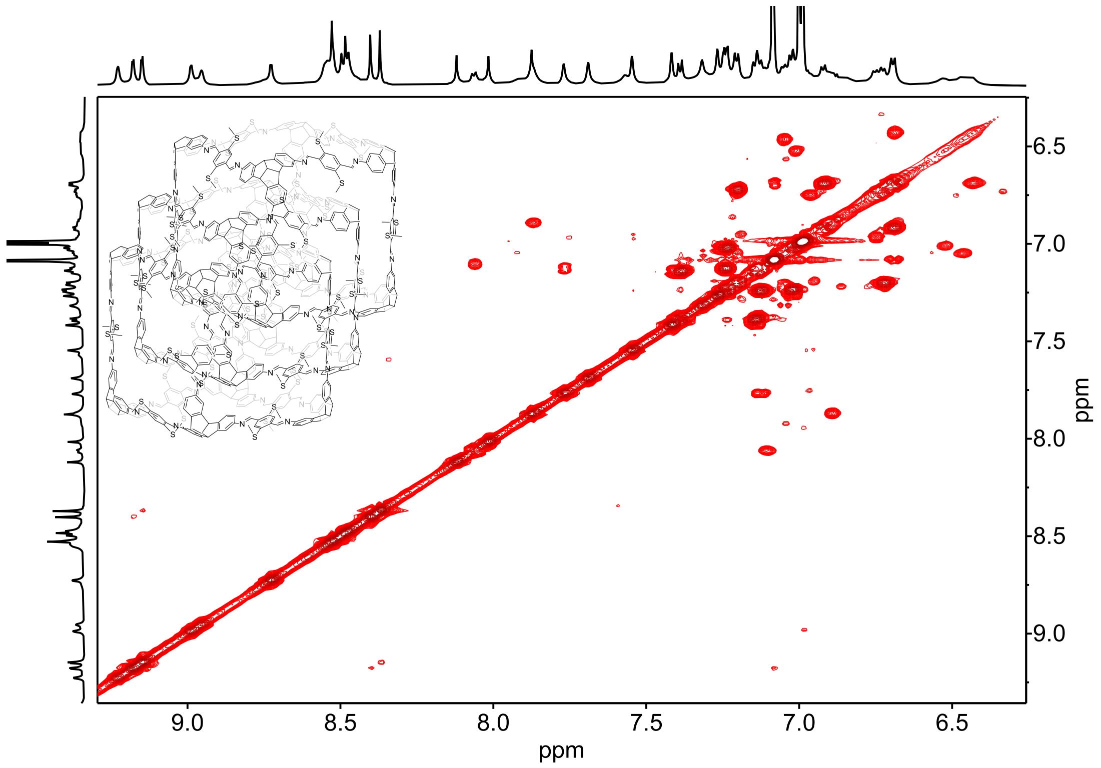

Figure 248. Partial ^{1}H-^{1}H COSY NMR spectrum (700 MHz, Toluene-d$_8$, 380 K) of **^{15}N labelled *(SMe-cube)$_3$**.

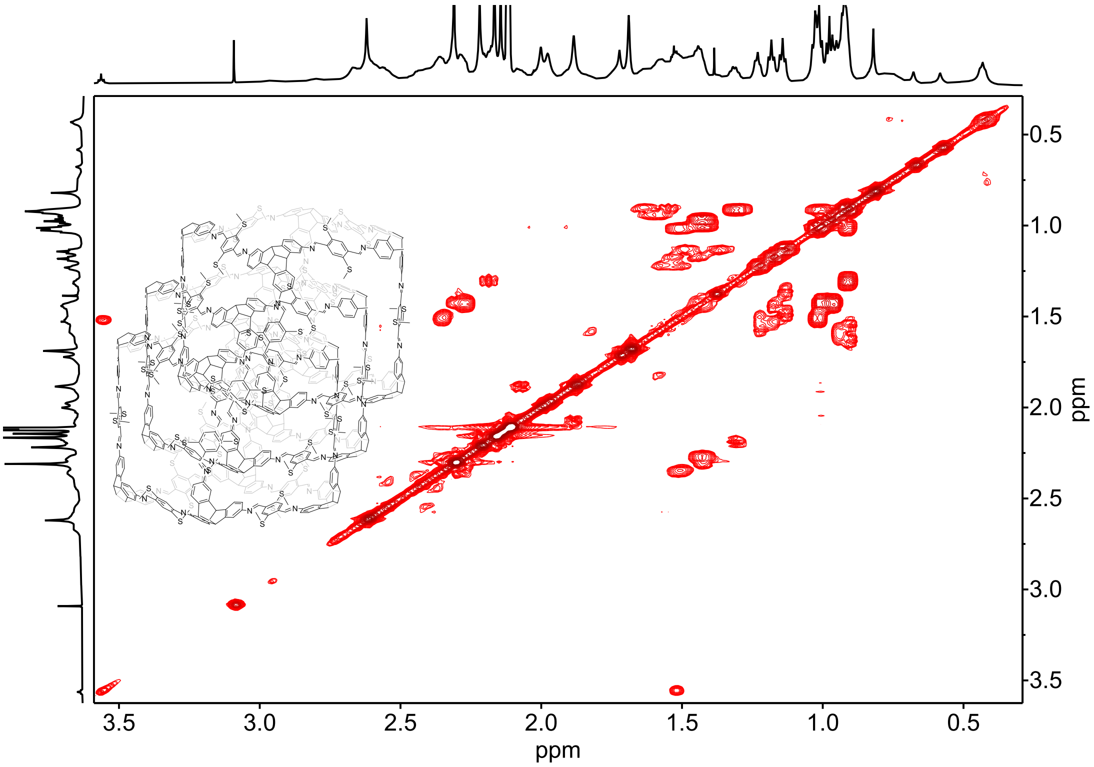

Figure 249. Partial ^{1}H-^{1}H COSY NMR spectrum (700 MHz, Toluene-d$_8$, 380 K) of **^{15}N labelled *(SMe-cube)$_3$.**

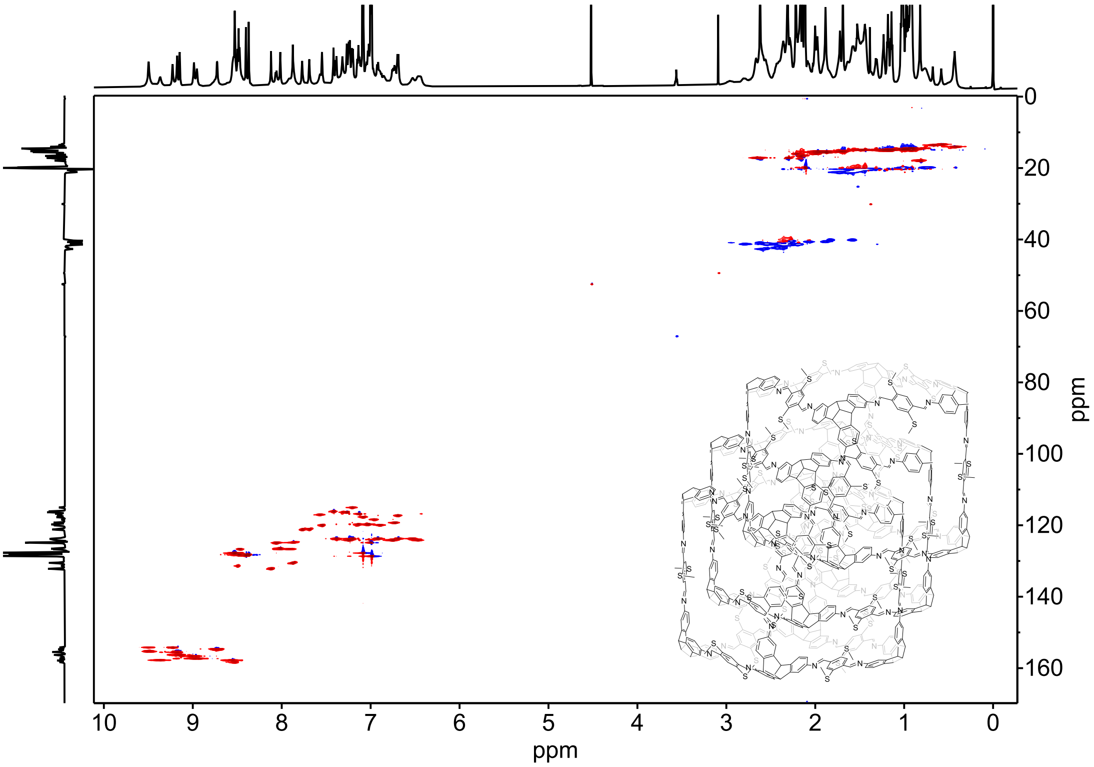

Figure 250. ^{1}H-^{13}C HSQC NMR (700 MHz and 176 MHz, Toluene-d$_8$, 380 K) spectrum of **^{15}N labelled *(SMe-cube)$_3$.**

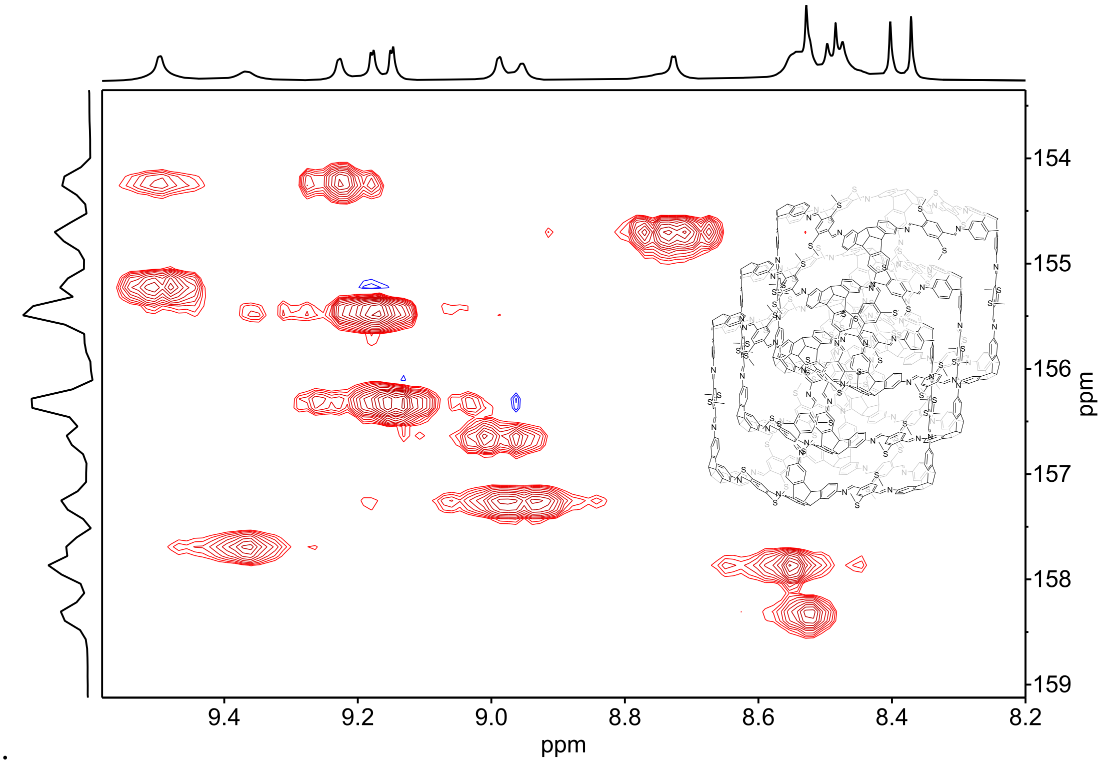

Figure 251. Partial ^{1}H-^{13}C HSQC NMR (700 MHz and 176 MHz, Toluene-d_8, 380 K) spectrum of **^{15}N labelled *(SMe-cube)$_3$**.

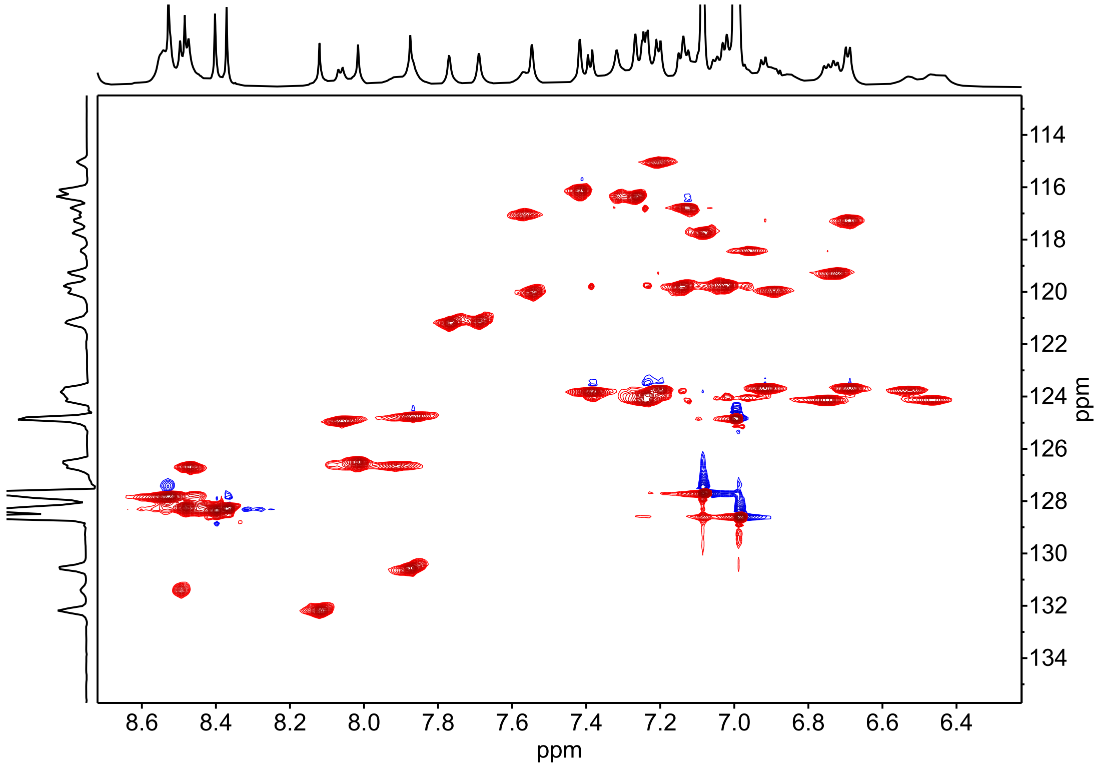

Figure 252. Partial ^{1}H-^{13}C HSQC NMR (700 MHz and 176 MHz, Toluene-d_8, 380 K) spectrum of **^{15}N labelled *(SMe-cube)$_3$**.

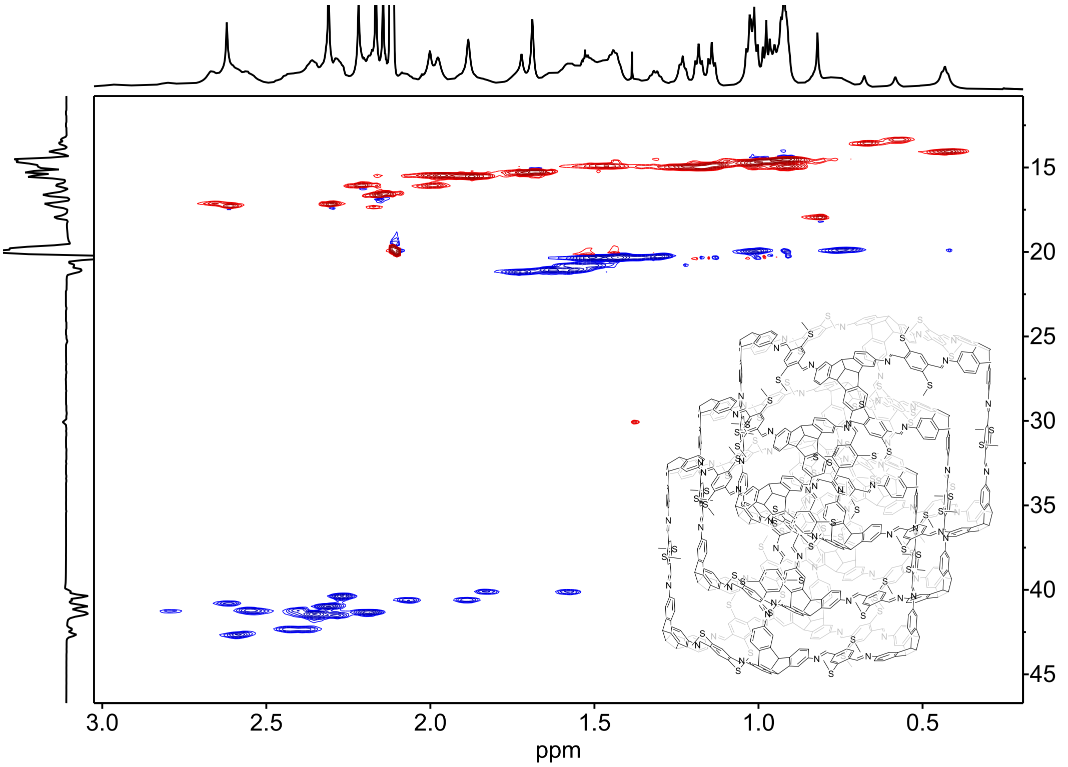

Figure 253. Partial ^{1}H-^{13}C HSQC NMR (700 MHz and 176 MHz, Toluene-d_8, 380 K) spectrum of **^{15}N labelled *(SMe-cube)$_3$**.

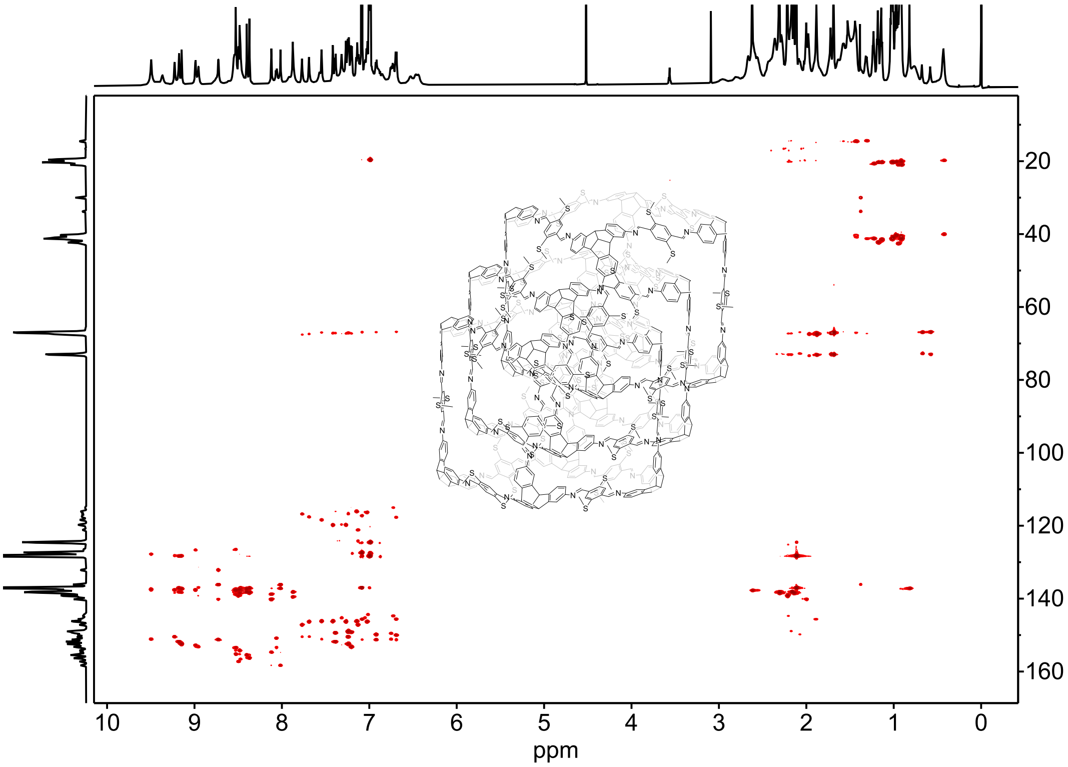

Figure 254. ^{1}H-^{13}C HMBC NMR (700 MHz and 176 MHz, Toluene-d_8, 380 K) spectrum of **^{15}N labelled *(SMe-cube)$_3$**.

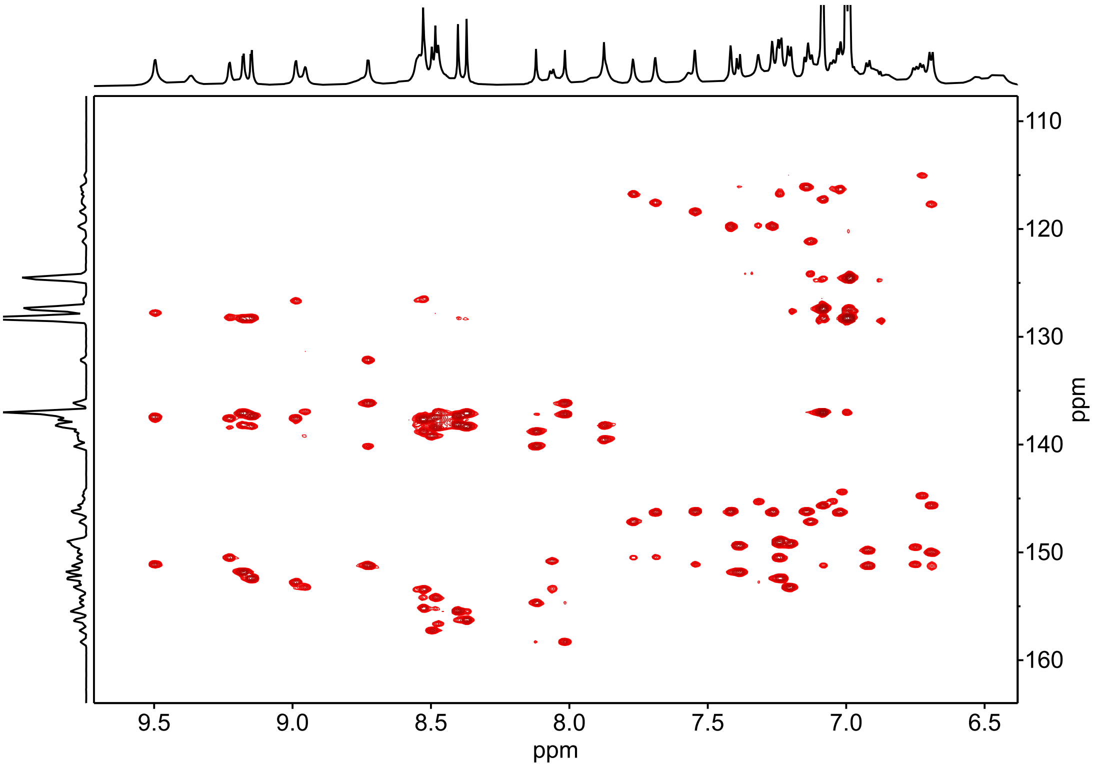

Figure 255. Partial ^{1}H-^{13}C HMBC NMR (700 MHz and 176 MHz, Toluene-d_8, 380 K) spectrum of **^{15}N labelled *(SMe-cube)$_3$**.

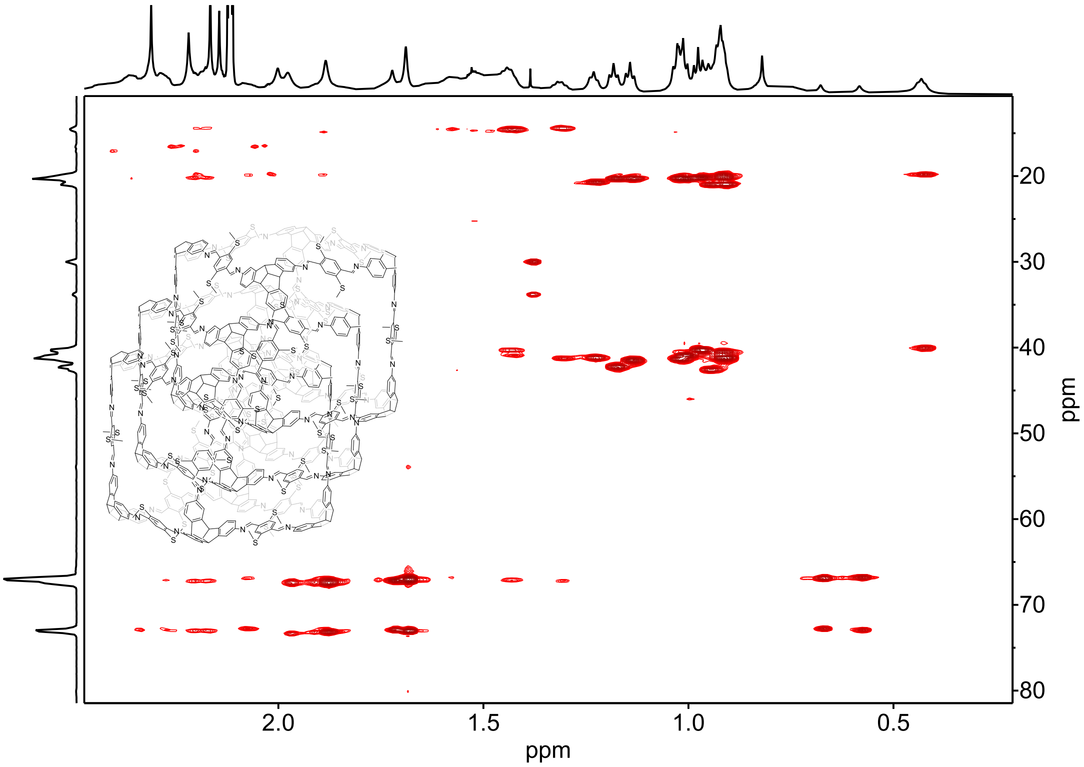

Figure 256. Partial ^{1}H-^{13}C HMBC NMR (700 MHz and 176 MHz, Toluene-d_8, 380 K) spectrum of **^{15}N labelled *(SMe-cube)$_3$**.

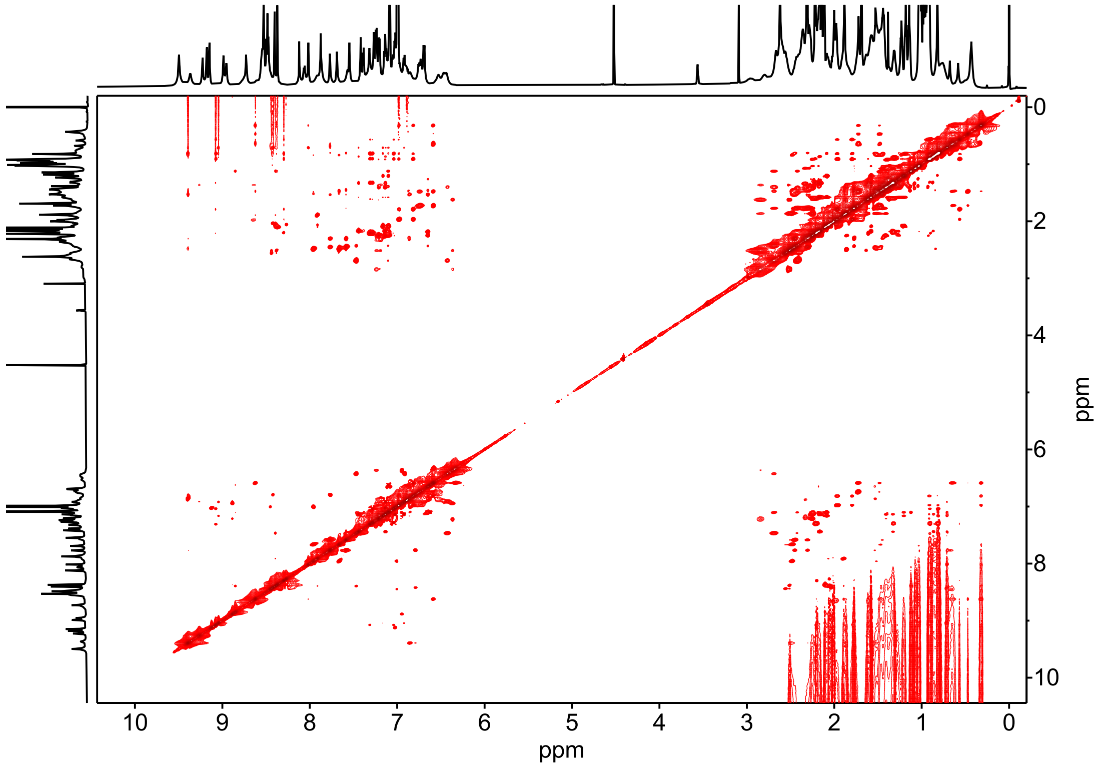

Figure 257. ^{1}H-^{1}H NOESY NMR (700 MHz, Toluene-d_8, 380 K) spectrum of **^{15}N labelled *(SMe-cube)$_3$**.

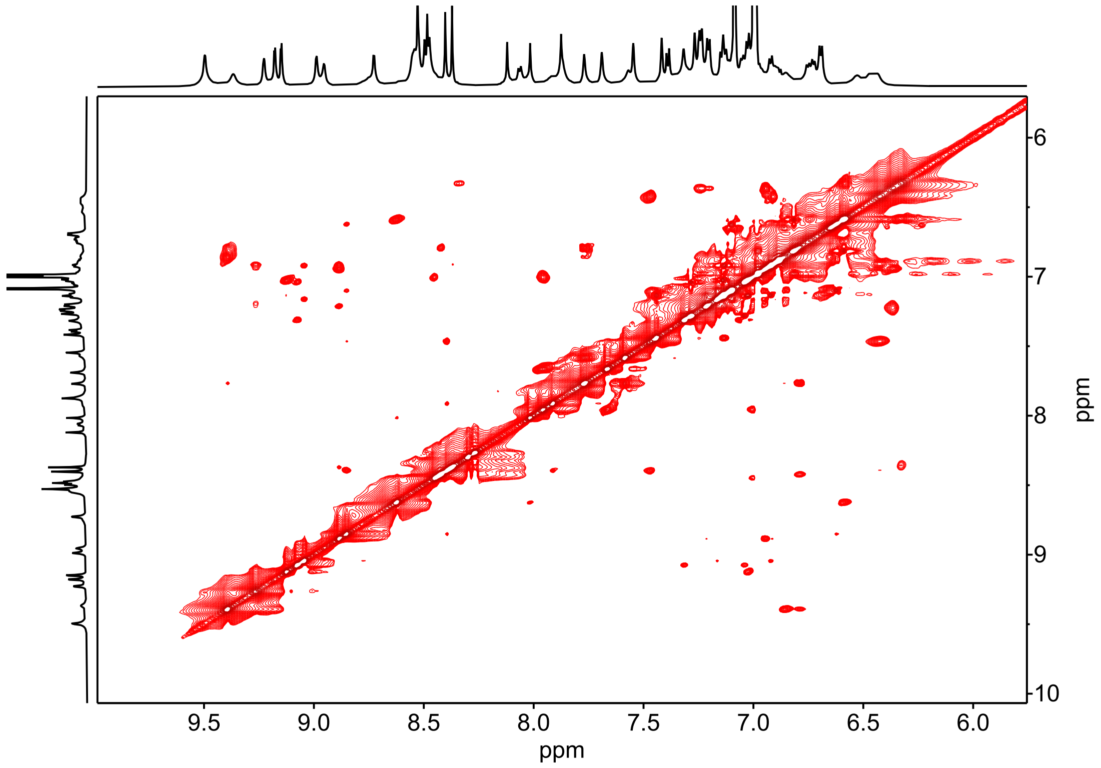

Figure 258. Partial ^{1}H-^{1}H NOESY NMR (700 MHz, Toluene-d_8, 380 K) spectrum of **^{15}N labelled *(SMe-cube)$_3$**.

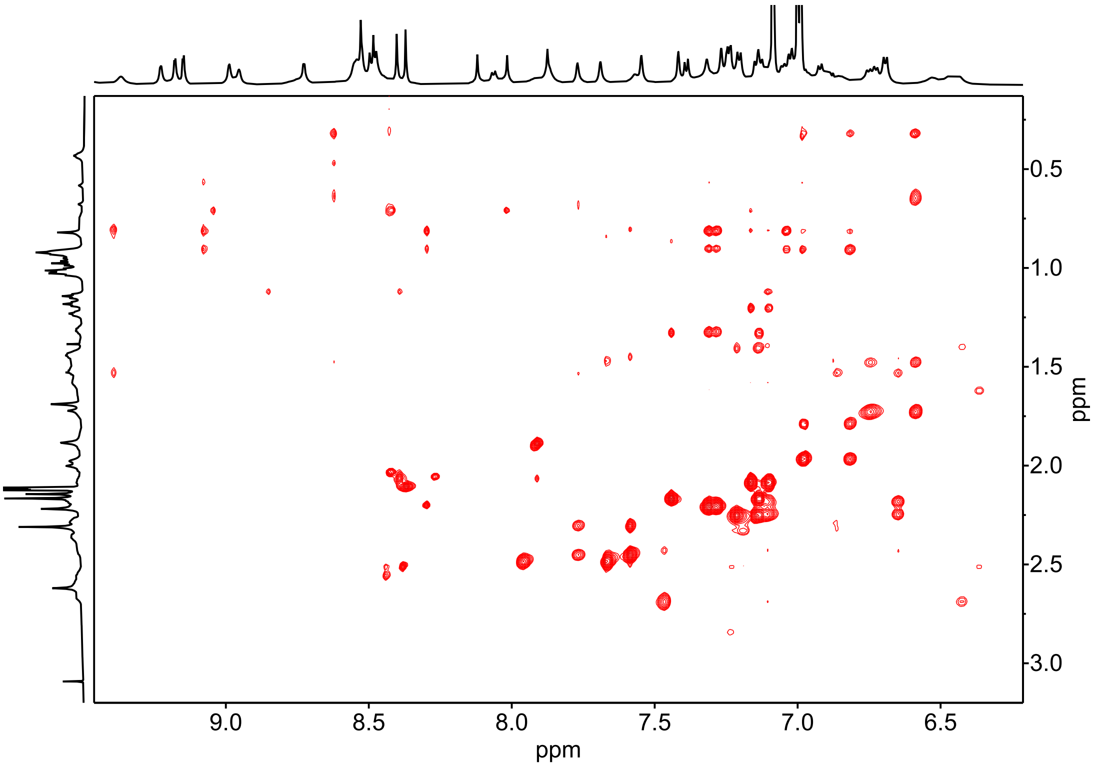

Figure 259. Partial ^{1}H-^{1}H NOESY NMR (700 MHz, Toluene-d$_8$, 380 K) spectrum of **^{15}N labelled *(SMe-cube)$_3$**.

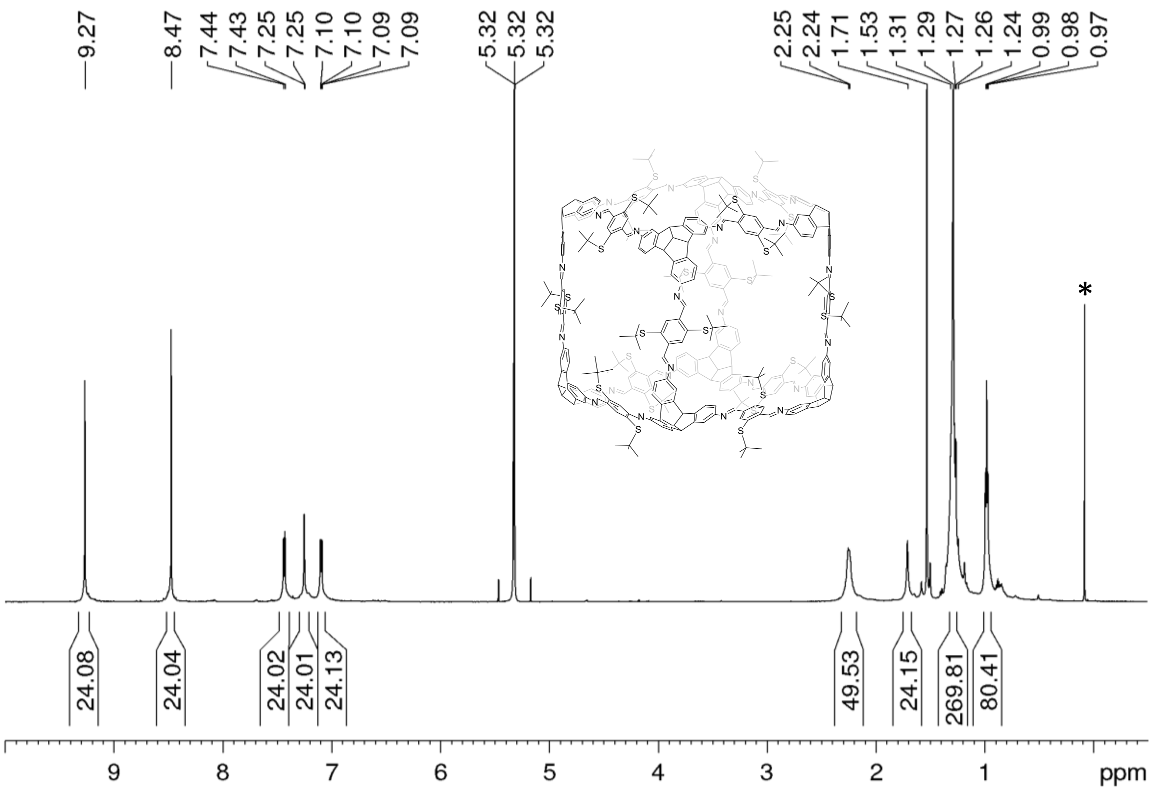

Figure 260. ^{1}H NMR (600 MHz, CD_2Cl_2) spectrum of **SC(CH$_3$)$_3$-cube**. (*silicone grease).

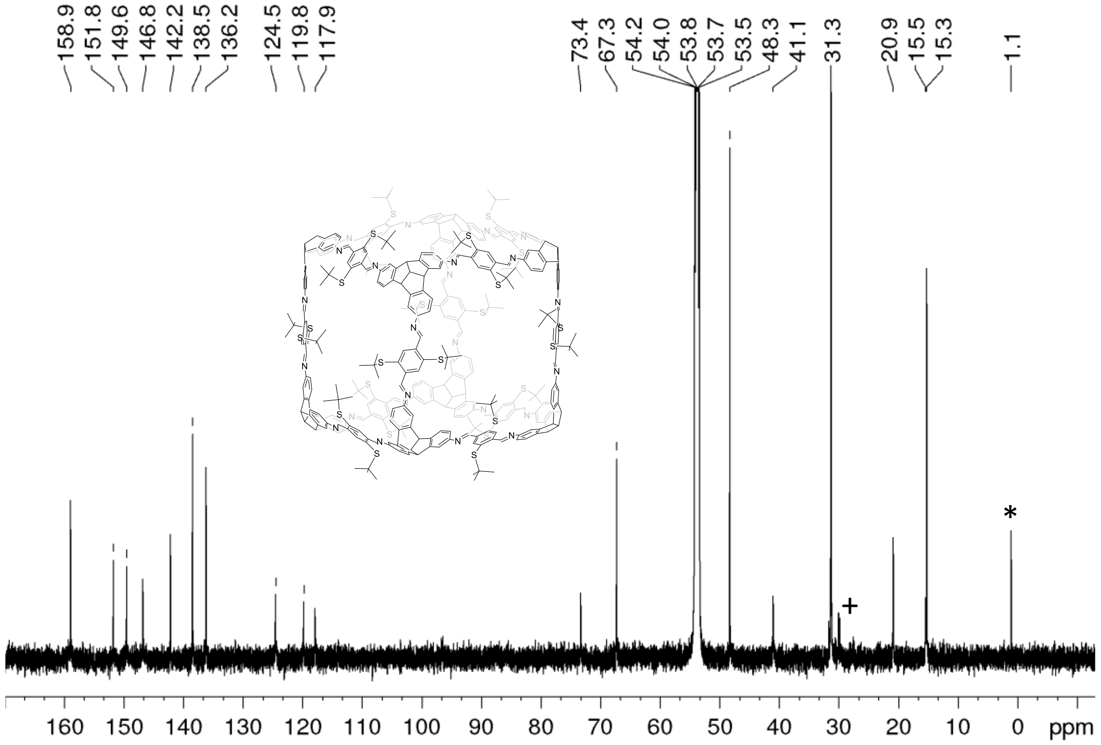

Figure 261. ^{13}C NMR spectrum (151 MHz, CD_2Cl_2) of **SC(CH$_3$)$_3$-cube**. ($^{+}$H grease, *silicone grease).

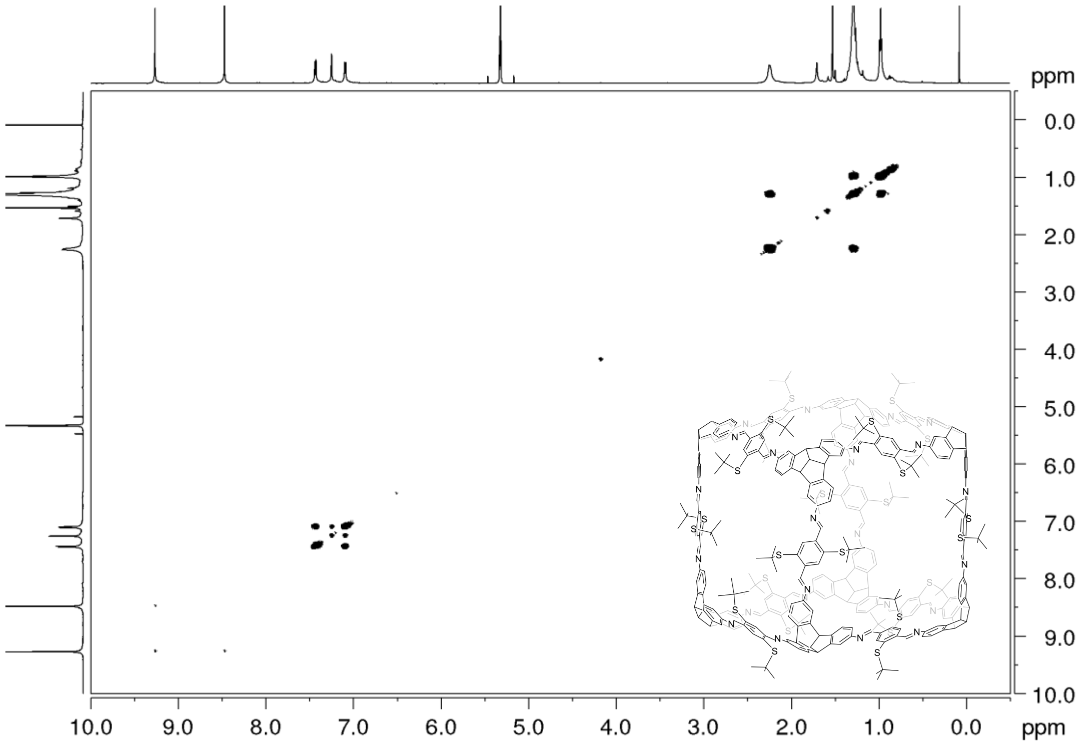

Figure 262. ^{1}H-^{1}H COSY NMR spectrum (600 MHz, CD_2Cl_2) of **$SC(CH_3)_3$-cube**.

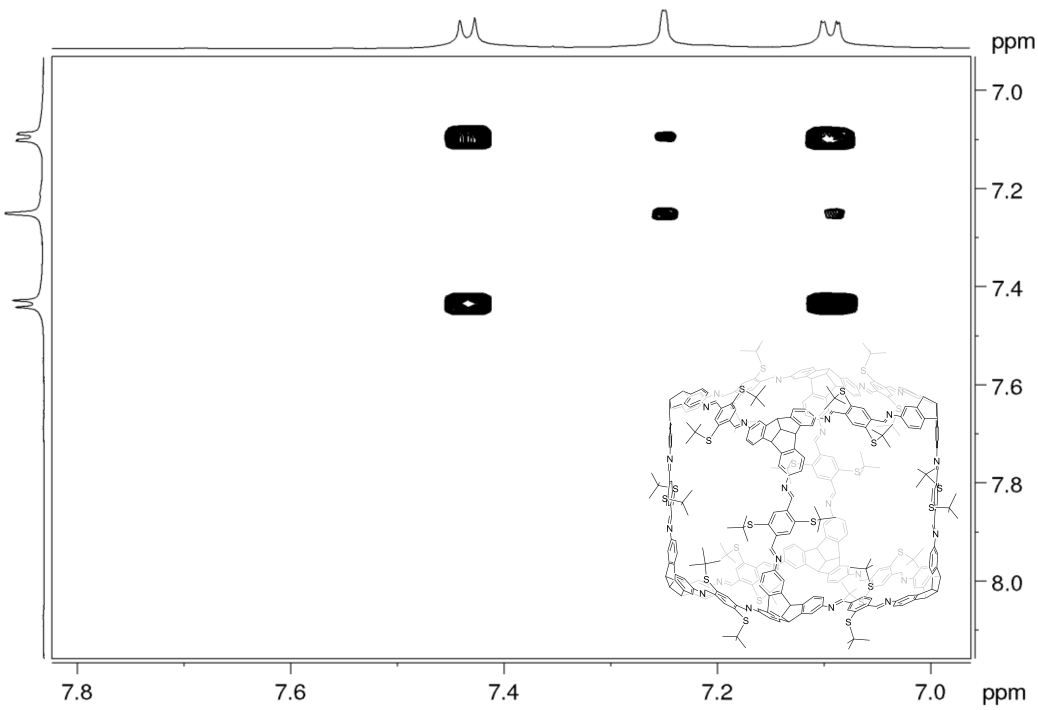

Figure 263. Partial ^{1}H-^{1}H COSY NMR spectrum (600 MHz, CD_2Cl_2) of **$SC(CH_3)_3$-cube**.

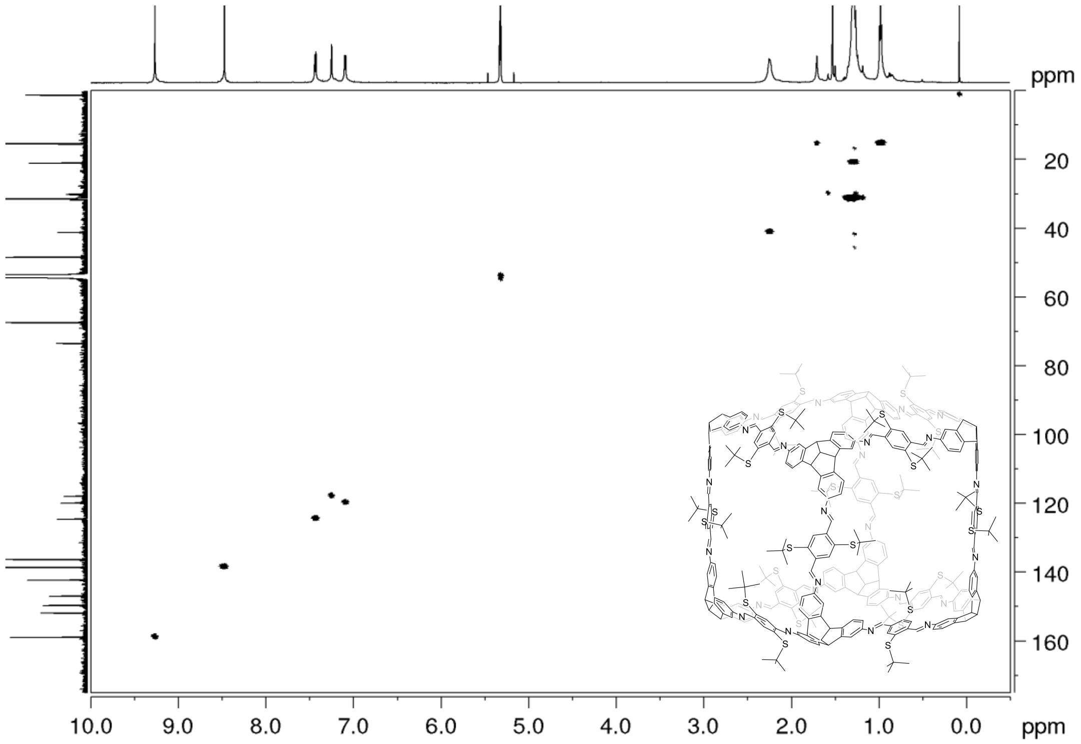

Figure 264. ^{1}H-^{13}C HSQC NMR (600 MHz and 151 MHz, CD_2Cl_2) spectrum of **$SC(CH_3)_3$-cube**.

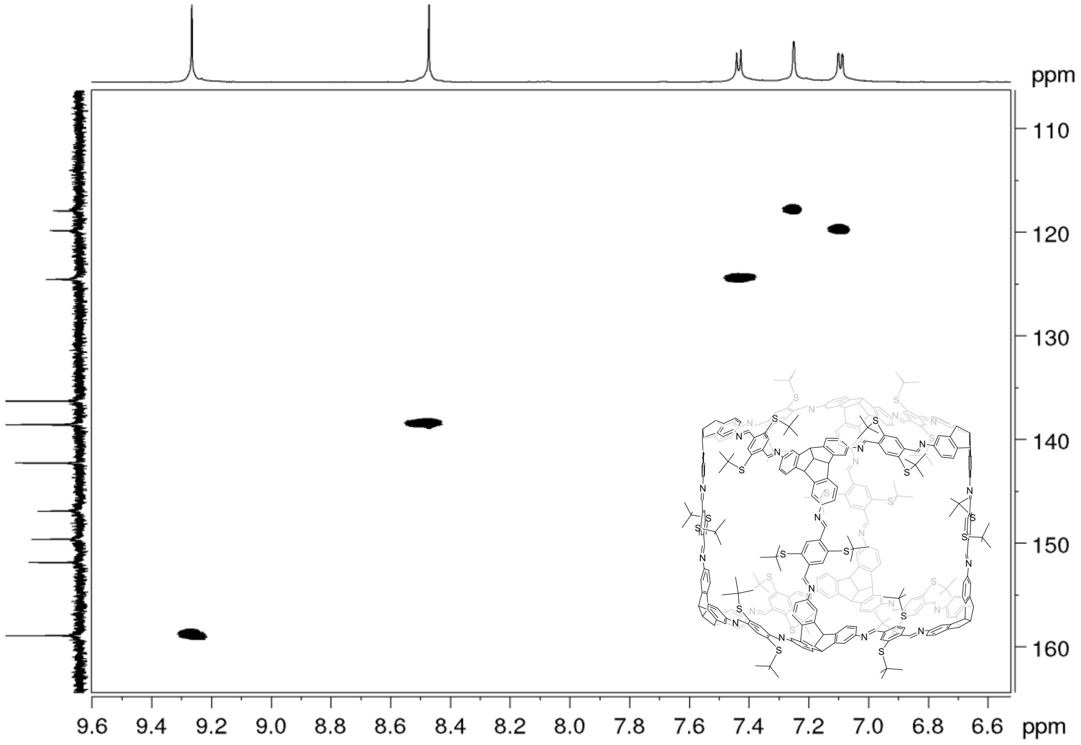

Figure 265. Partial ^{1}H-^{13}C HSQC NMR (600 MHz and 151 MHz, CD_2Cl_2) spectrum of **$SC(CH_3)_3$-cube**.

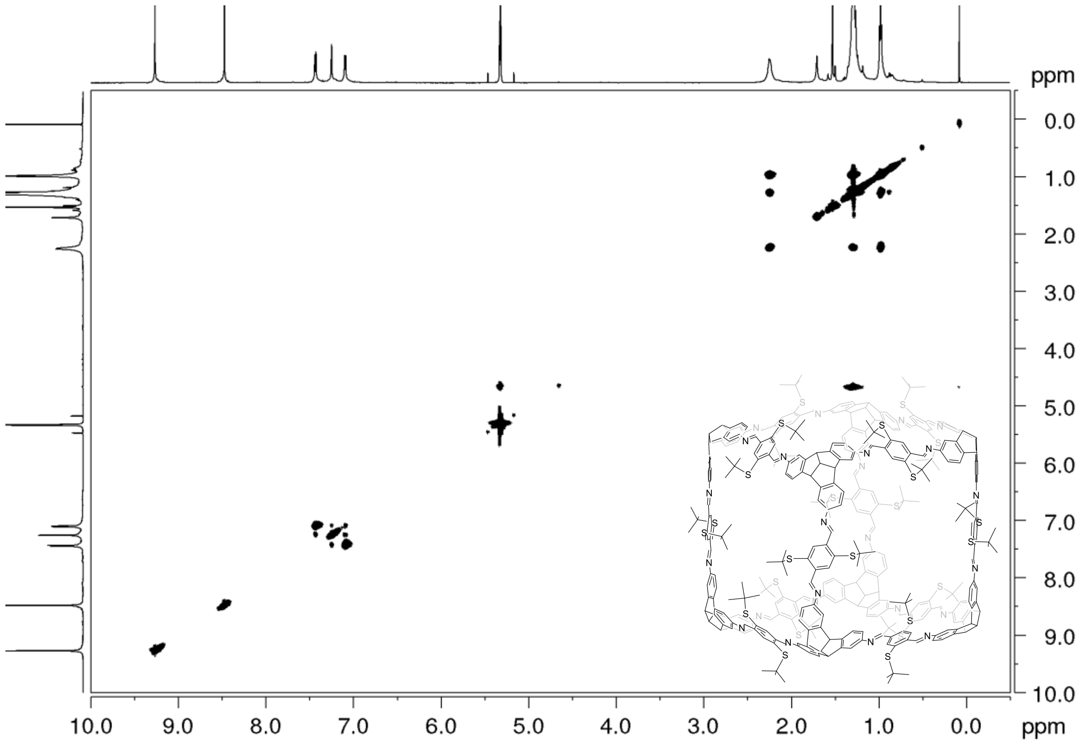

Figure 266. ^1H-^1H TOCSY NMR (600 MHz, CD_2Cl_2) spectrum of **$SC(CH_3)_3$-cube**.

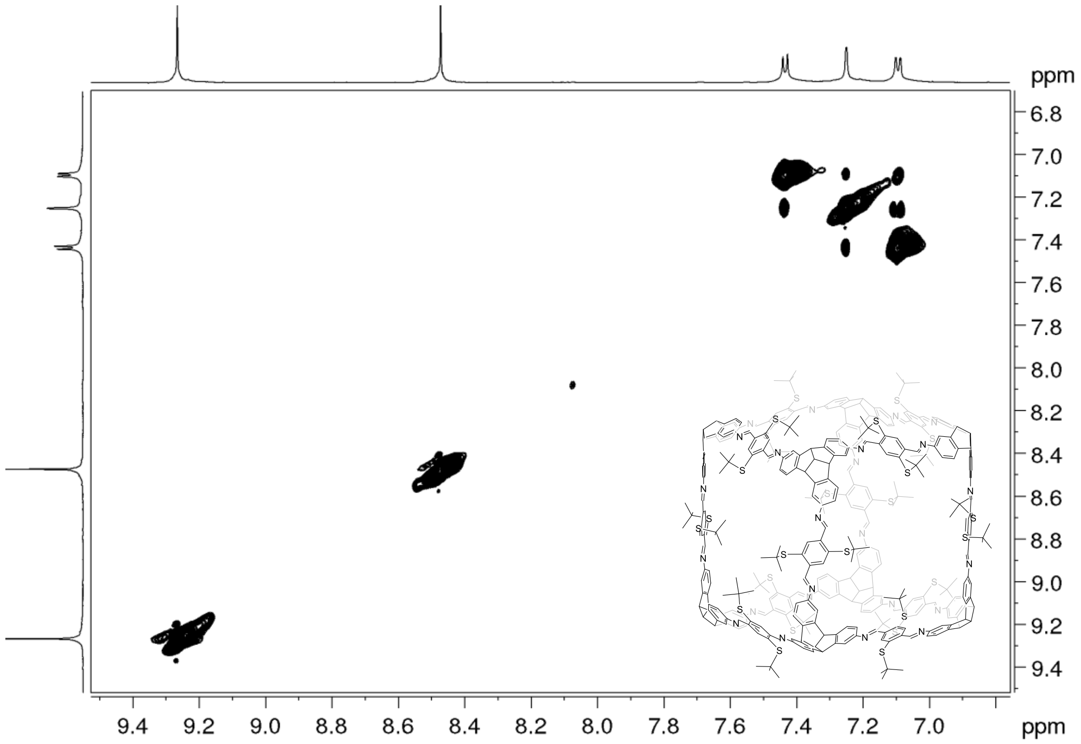

Figure 267. Partial ^1H-^1H TOCSY NMR (600 MHz, CD_2Cl_2) spectrum of **$SC(CH_3)_3$-cube**.

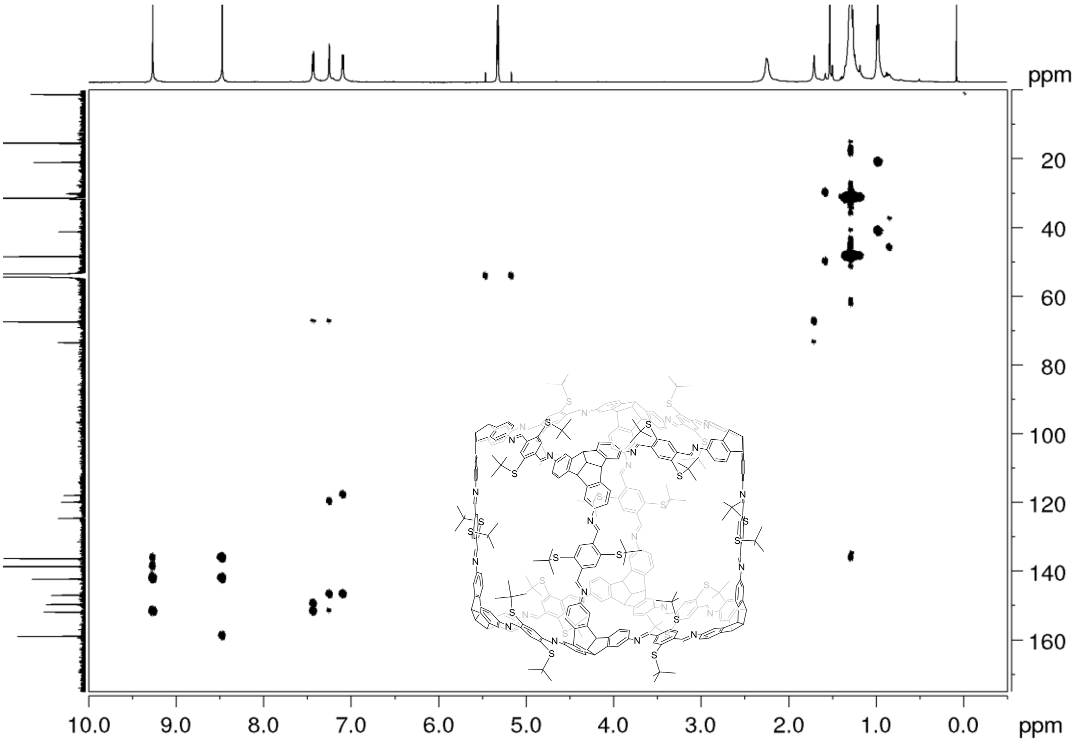

Figure 268. ^{1}H-^{13}C HMBC NMR (600 MHz and 151 MHz, CD_2Cl_2) spectrum of **$SC(CH_3)_3$-cube**.

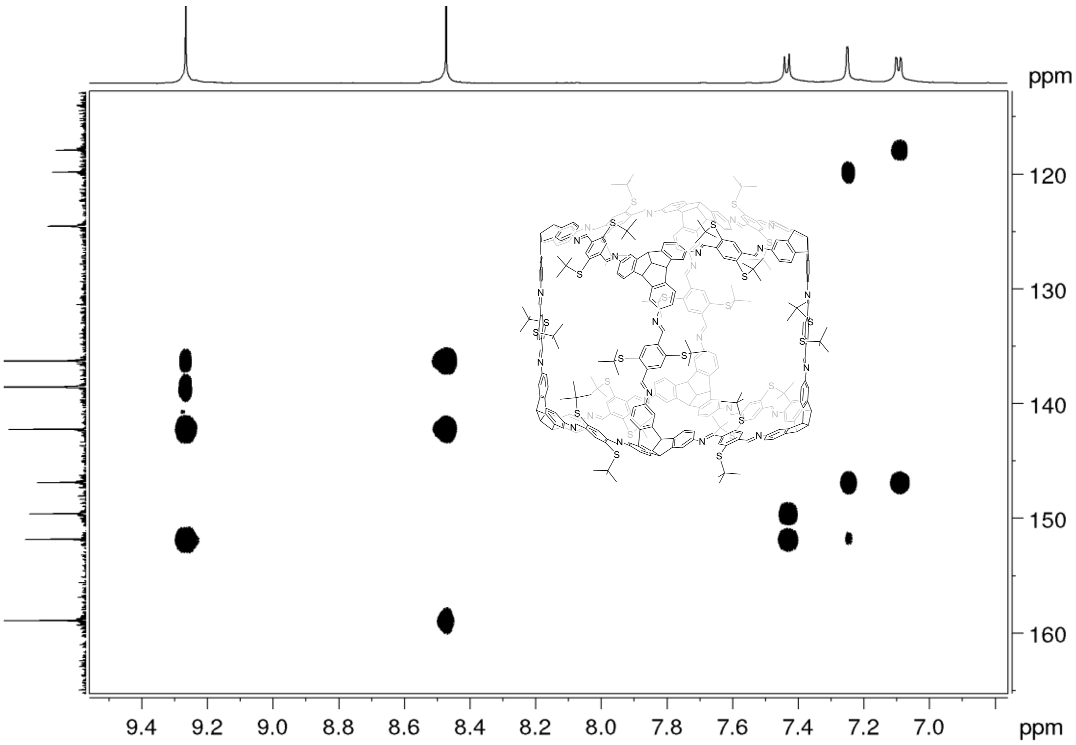

Figure 269. Partial ^{1}H-^{13}C HMBC NMR (600 MHz and 151 MHz, CD_2Cl_2) spectrum of **$SC(CH_3)_3$-cube**.

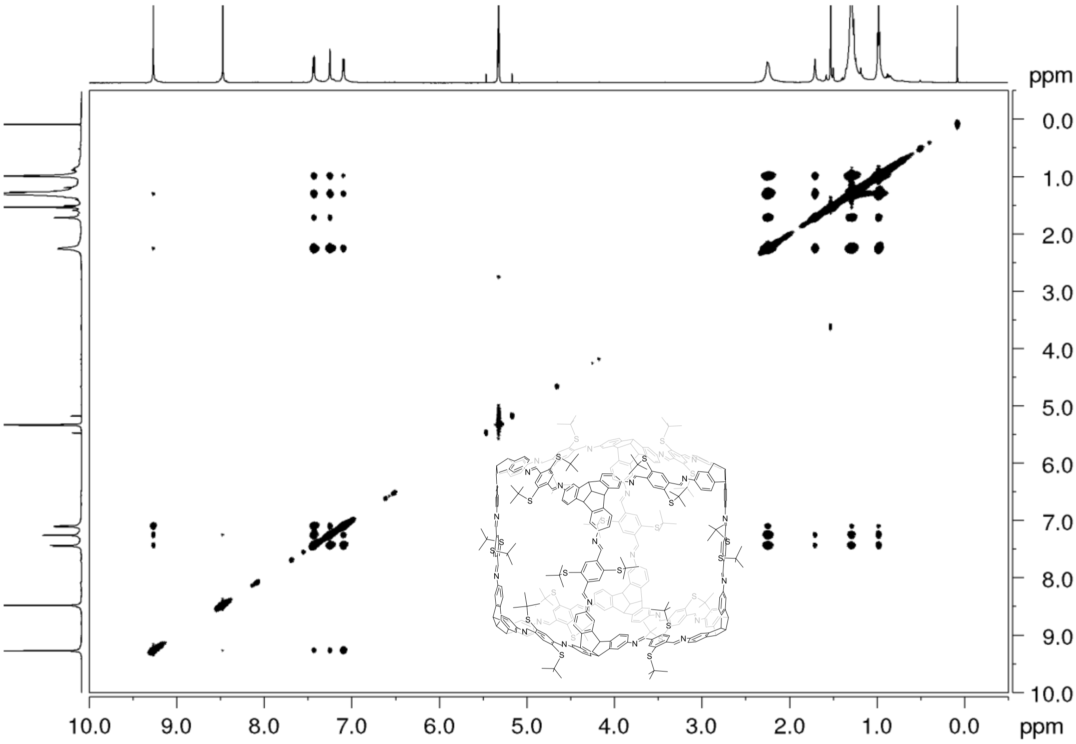

Figure 270. ^{1}H-^{1}H NOESY NMR (600 MHz, CD_2Cl_2) spectrum of **$SC(CH_3)_3$-cube**.

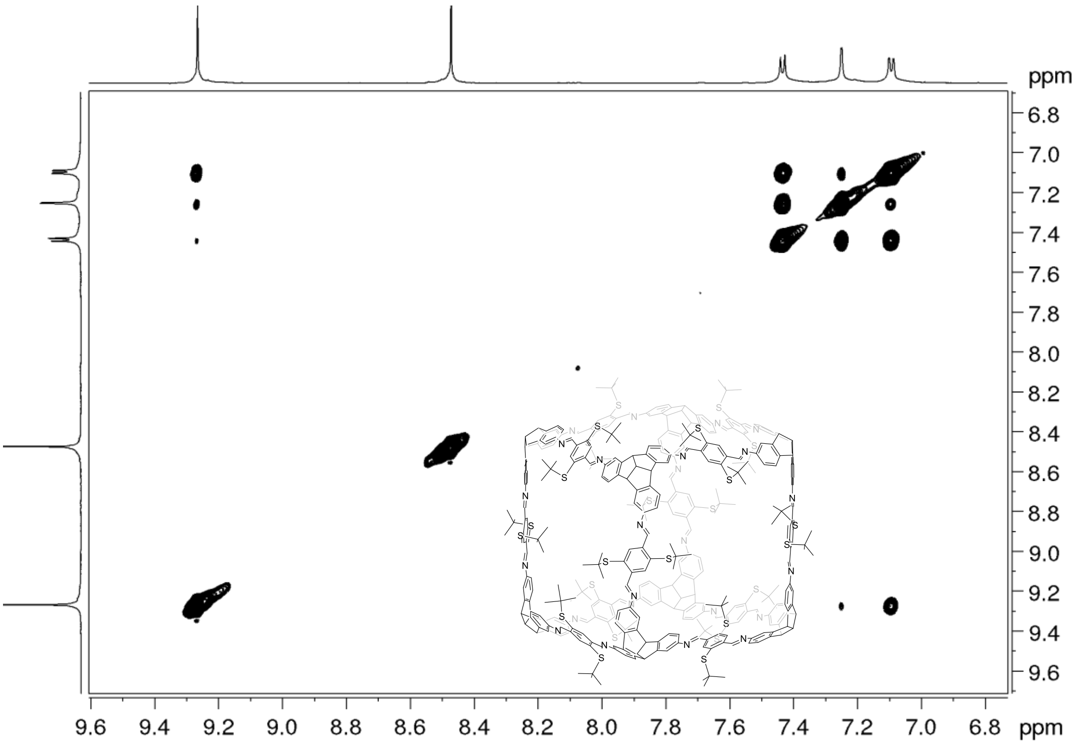

Figure 271. Partial ^{1}H-^{1}H NOESY NMR (600 MHz, CD_2Cl_2) spectrum of **$SC(CH_3)_3$-cube**.

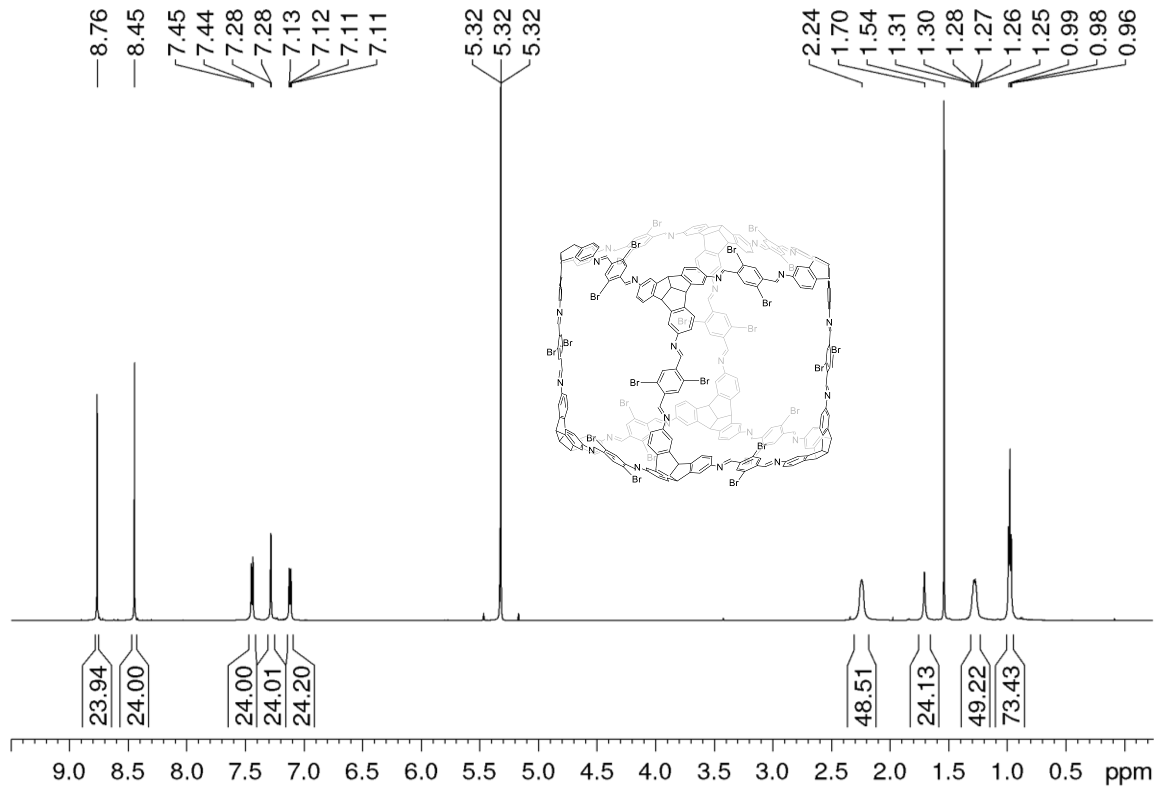

Figure 272. ^{1}H NMR (600 MHz, CD_2Cl_2) spectrum of **Br-cube**.

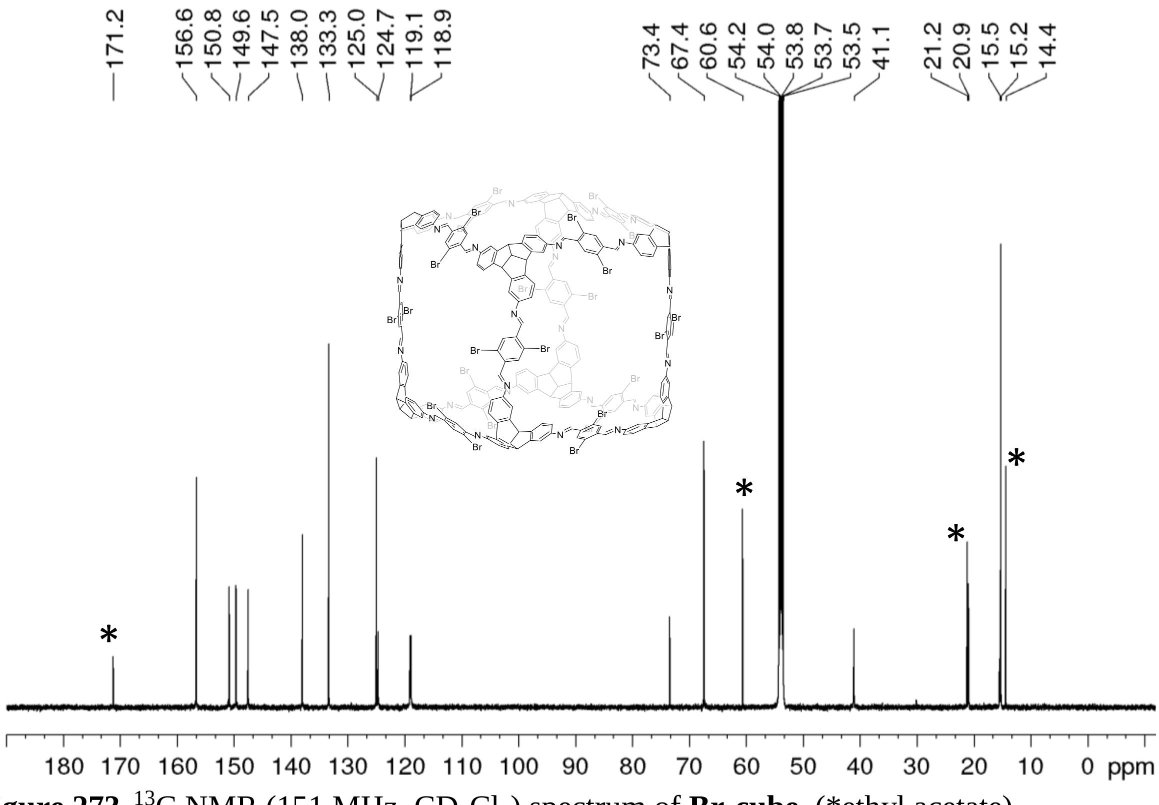

Figure 273. ^{13}C NMR (151 MHz, CD_2Cl_2) spectrum of **Br-cube**. (*ethyl acetate).

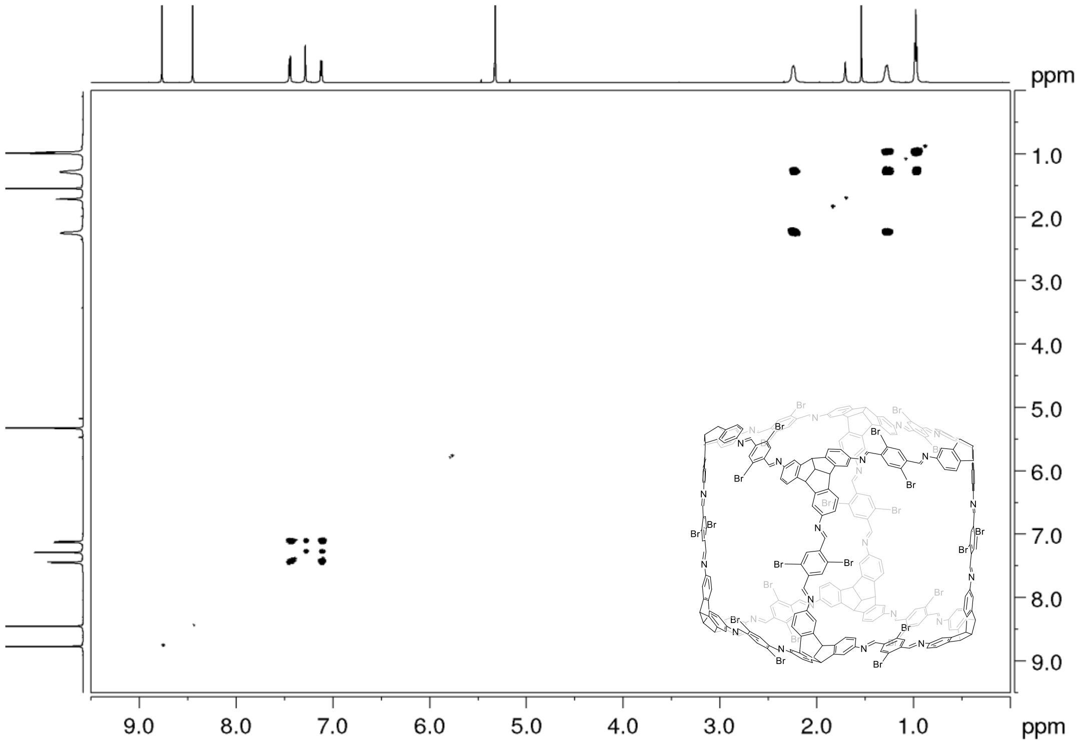

Figure 274. ^{1}H-^{1}H COSY NMR spectrum (600 MHz, CD_2Cl_2) of **Br-cube**.

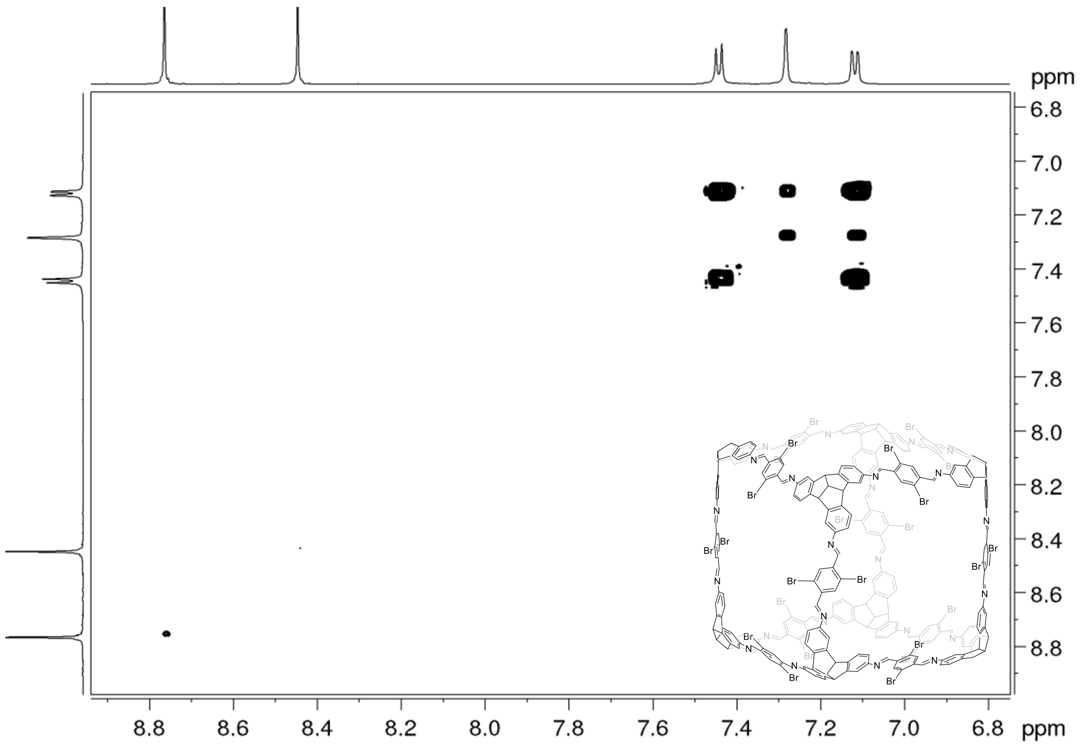

Figure 275. Partial ^{1}H-^{1}H COSY NMR spectrum (600 MHz, CD_2Cl_2) of **Br-cube**.

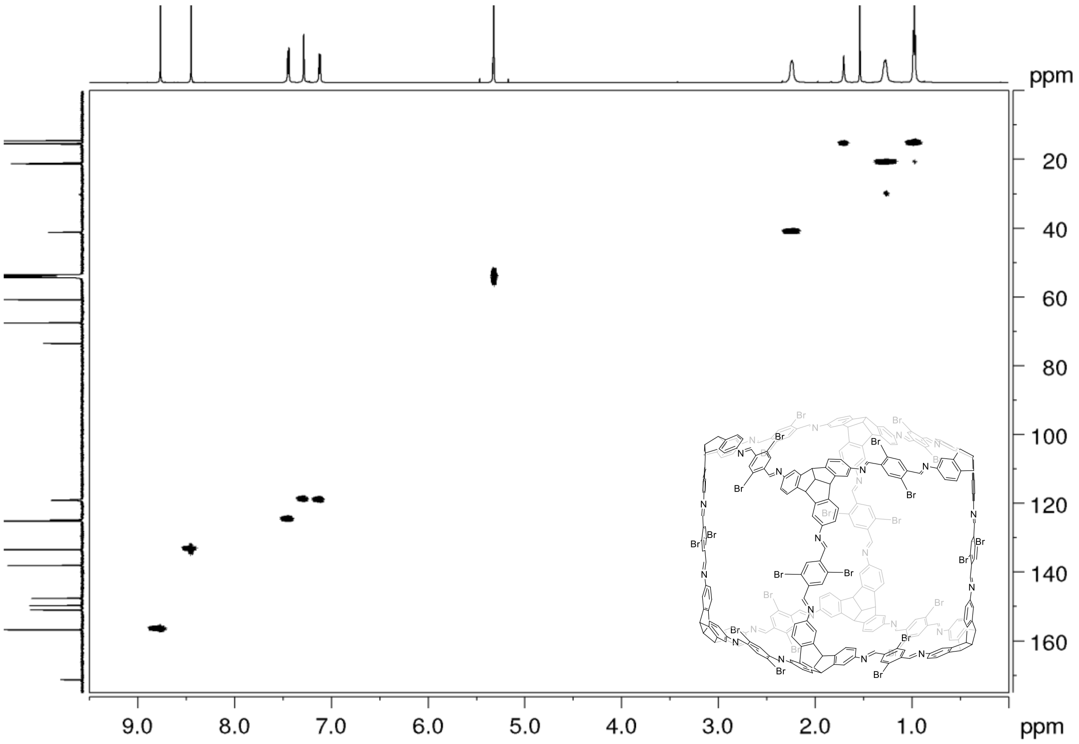

Figure 276. ^{1}H-^{13}C HSQC NMR (600 MHz and 151 MHz, CD_2Cl_2) spectrum of **Br-cube**.

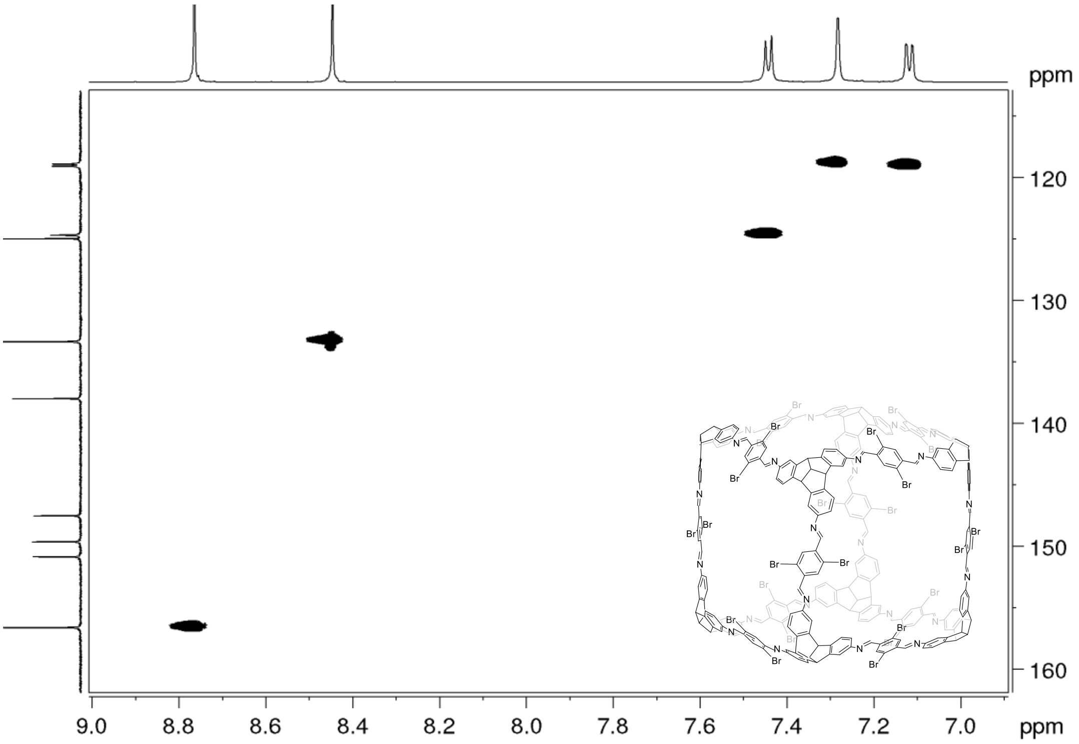

Figure 277. Partial ^{1}H-^{13}C HSQC NMR (600 MHz and 151 MHz, CD_2Cl_2) spectrum of **Br-cube**.

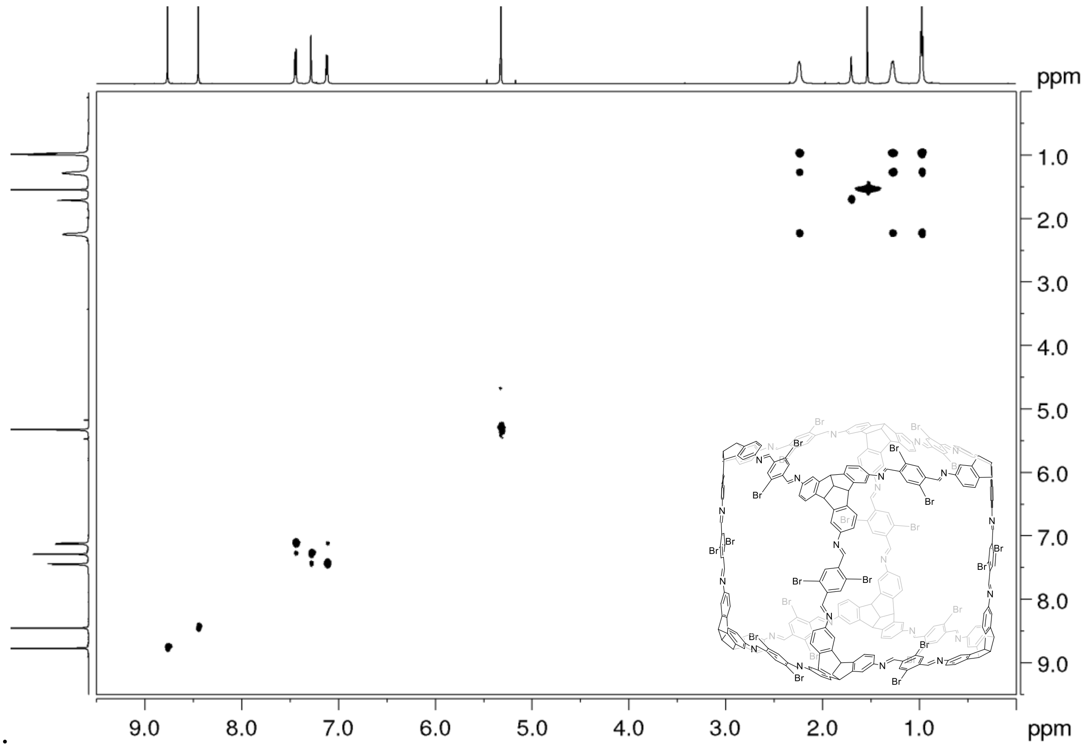

Figure 278. ^{1}H-^{1}H TOCSY NMR (600 MHz, CD_2Cl_2) spectrum of **Br-cube**

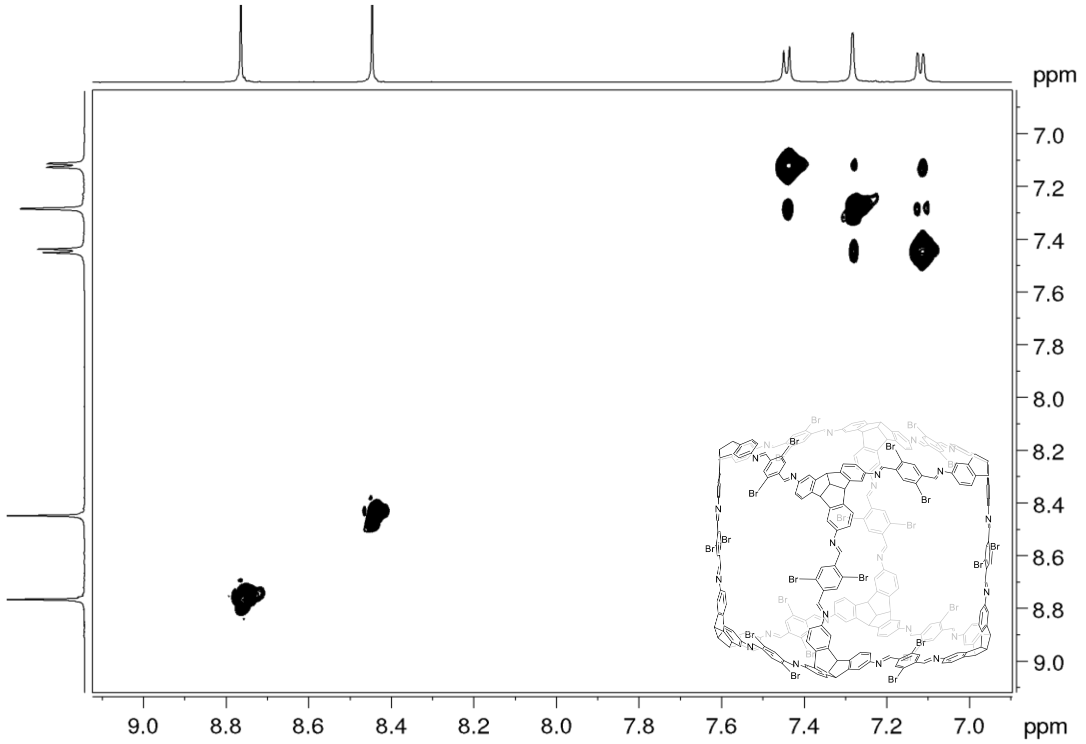

Figure 279. Partial ^{1}H-^{1}H TOCSY NMR (600 MHz, CD_2Cl_2) spectrum of **Br-cube**.

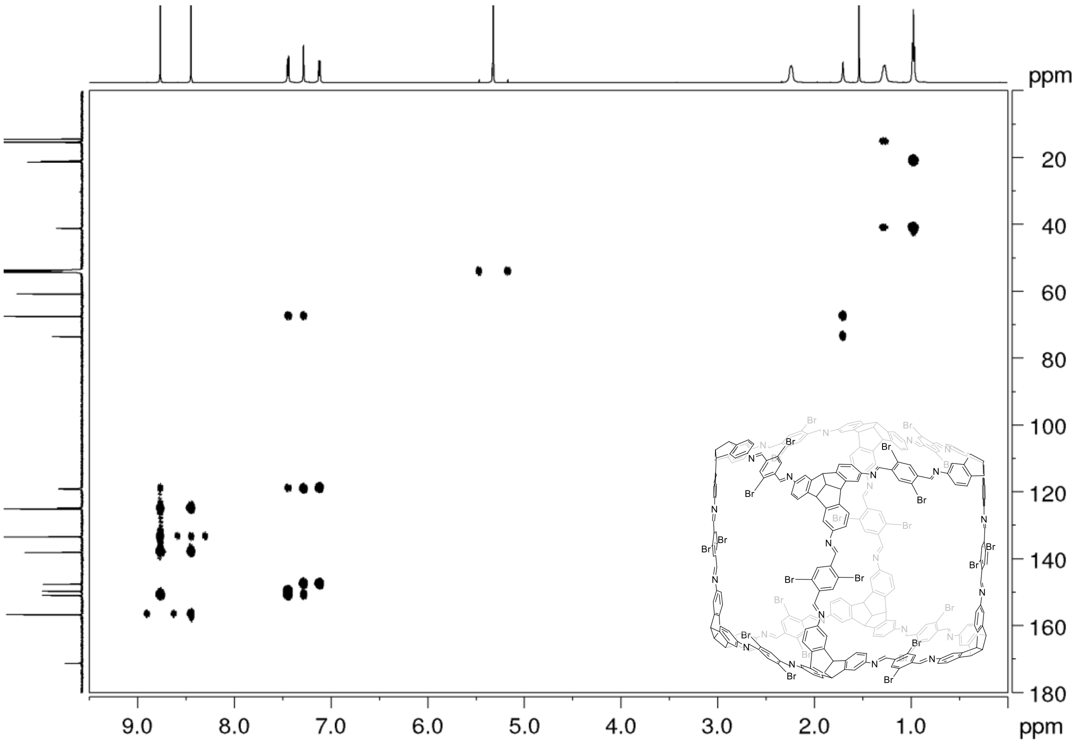

Figure 280. ^{1}H-^{13}C HMBC NMR (600 MHz and 151 MHz, CD_2Cl_2) spectrum of **Br-cube**.

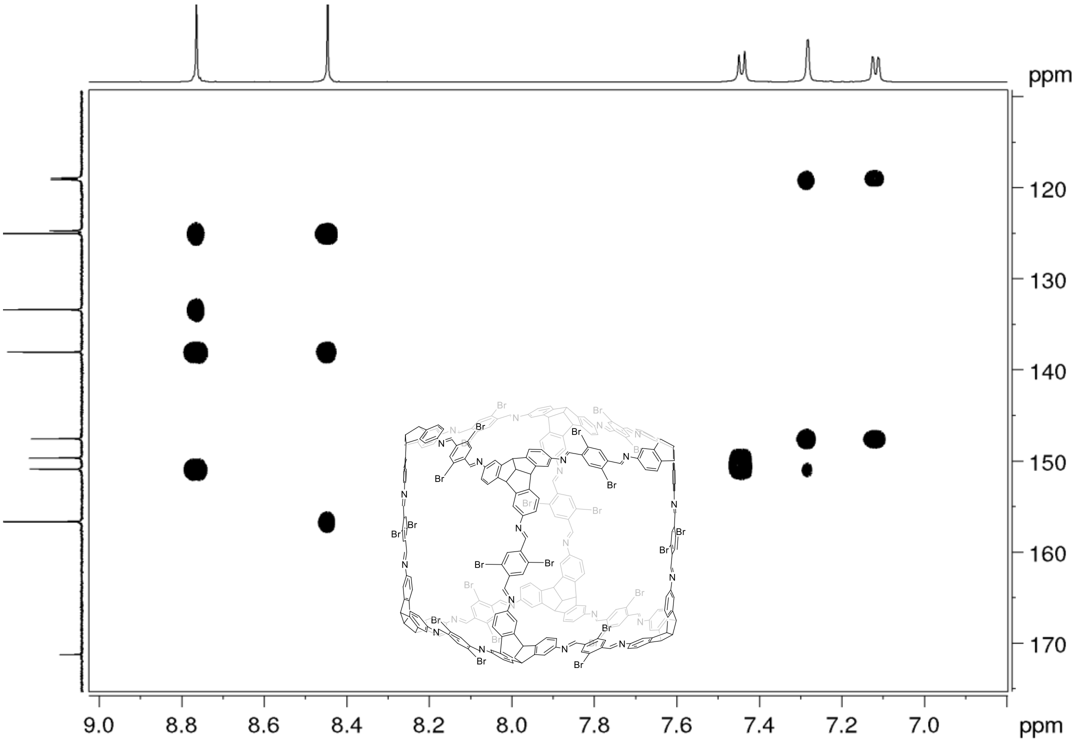

Figure 281. Partial ^{1}H-^{13}C HMBC NMR (600 MHz and 151 MHz, CD_2Cl_2) spectrum of **Br-cube**.

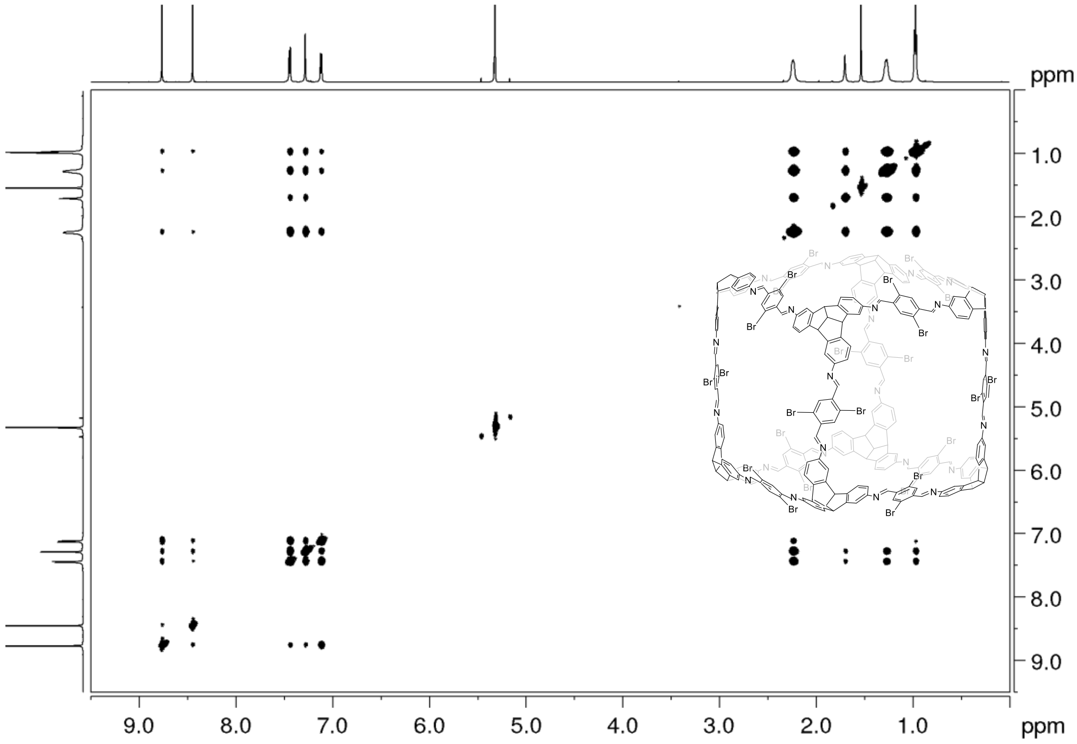

Figure 282. ^1H-^1H NOESY NMR (600 MHz, CD_2Cl_2) spectrum of **Br-cube**.

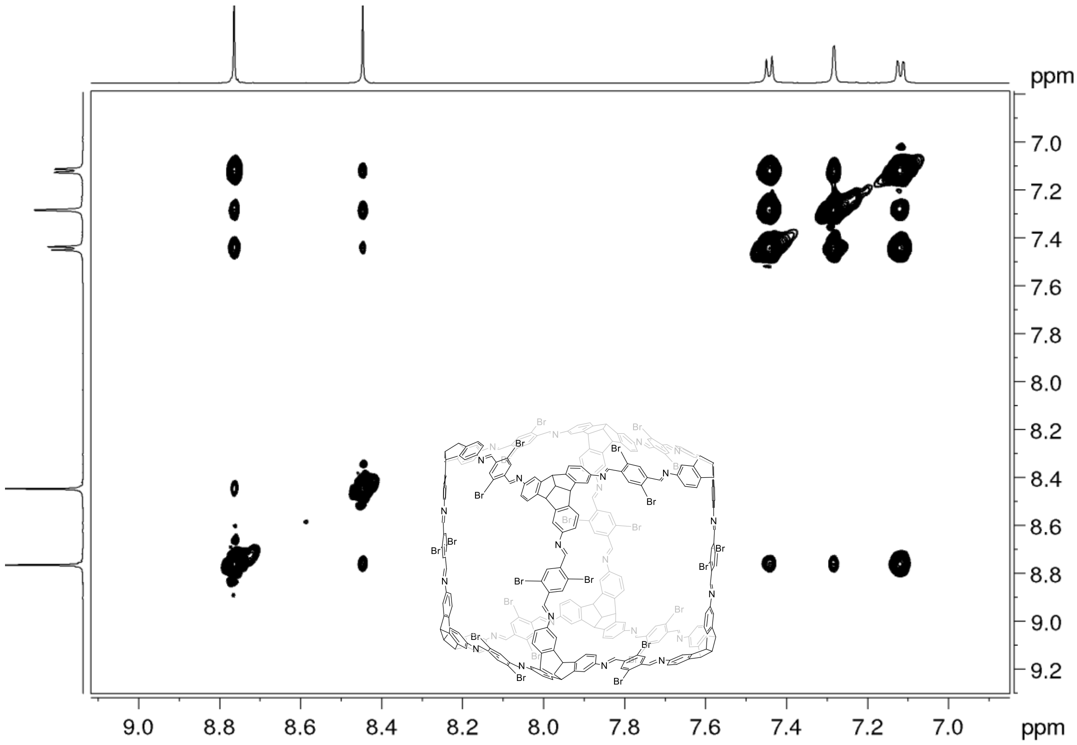

Figure 283. Partial ^1H-^1H NOESY NMR (600 MHz, CD_2Cl_2) spectrum of **Br-cube**.

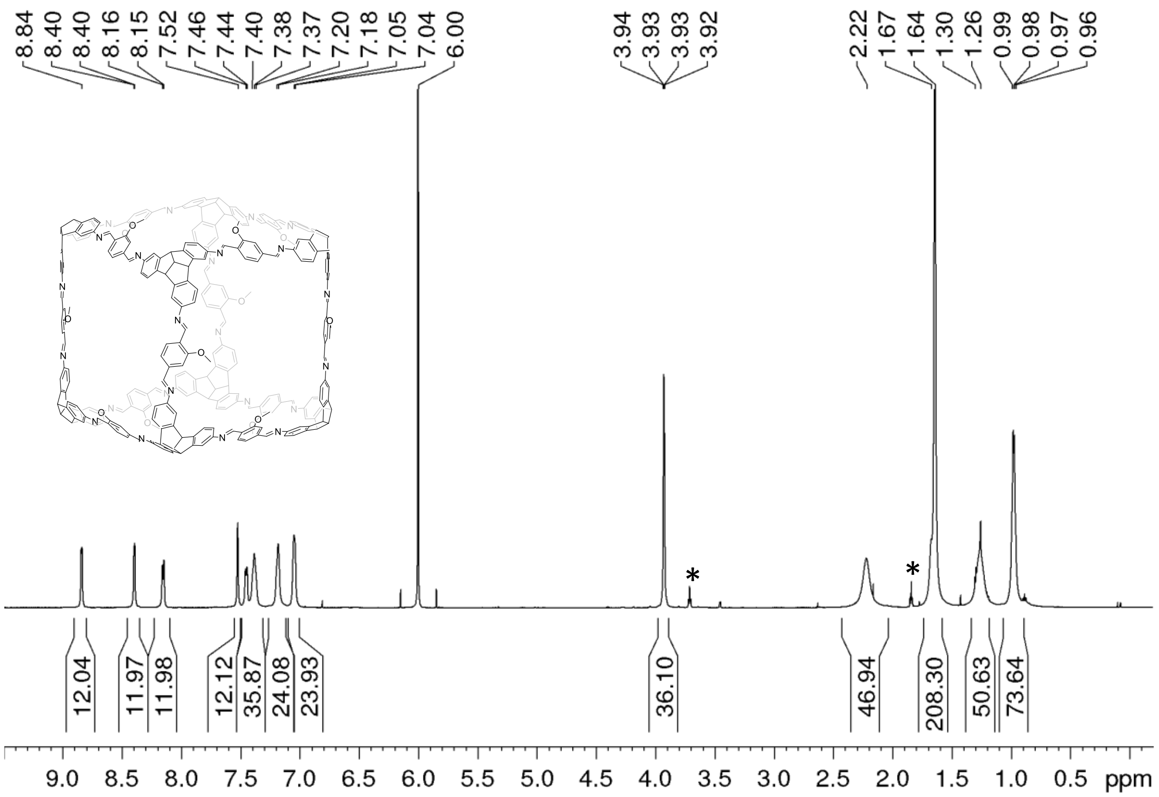

Figure 284. ^{1}H NMR (600 MHz, $C_2D_2Cl_4$) spectrum of **H/OMe-cube**. (*THF)

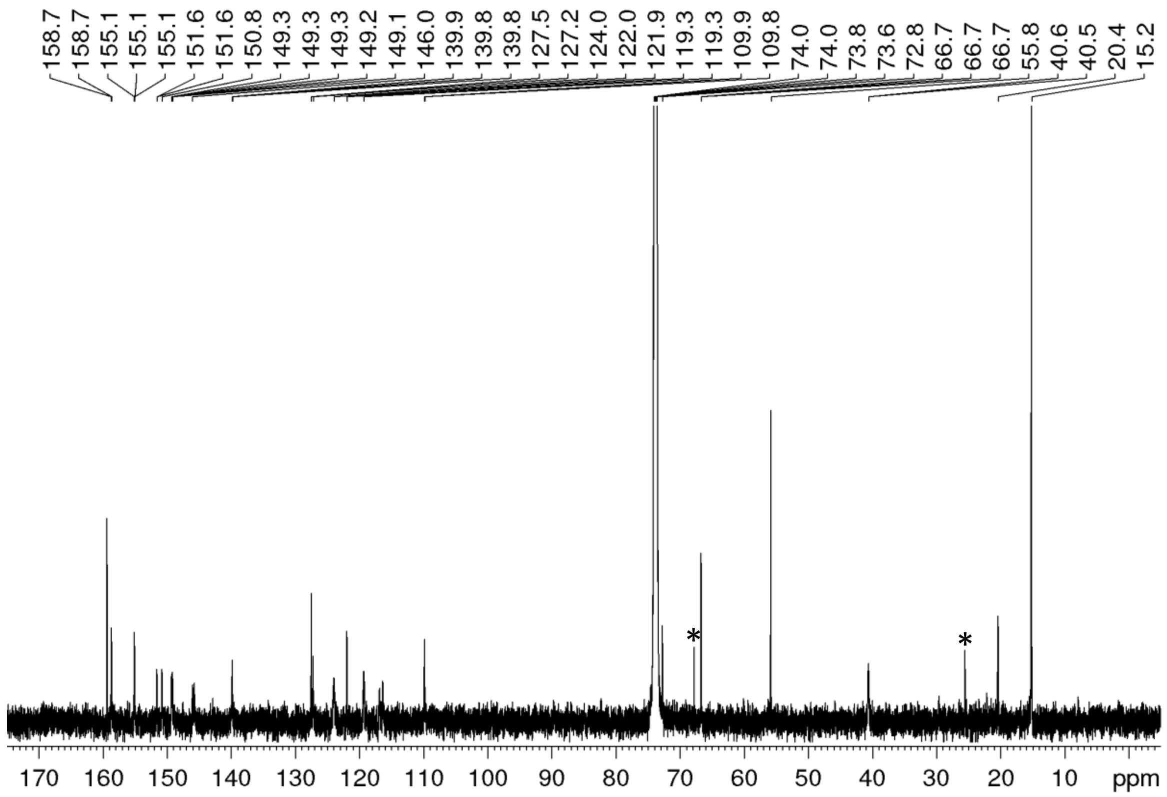

Figure 285. ^{13}C NMR (151 MHz, $C_2D_2Cl_4$) spectrum of **H/OMe-cube**. (*THF)

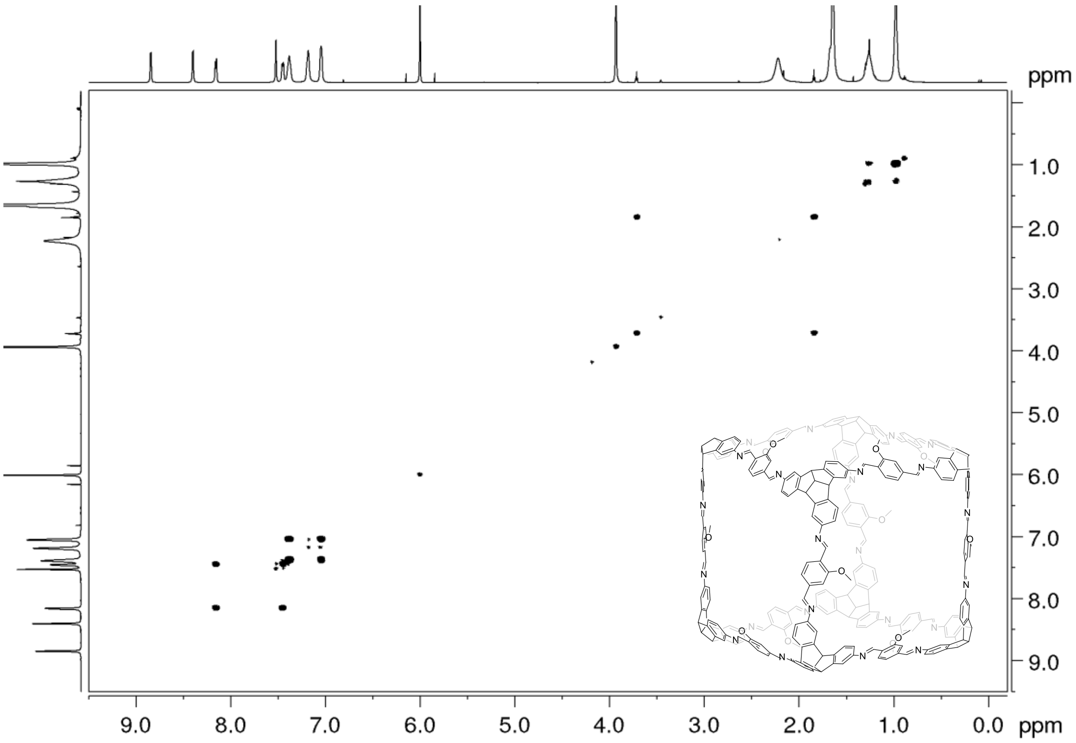

Figure 286. ^{1}H-^{1}H COSY NMR spectrum (600 MHz, $C_2D_2Cl_4$) of **H/OMe-cube**.

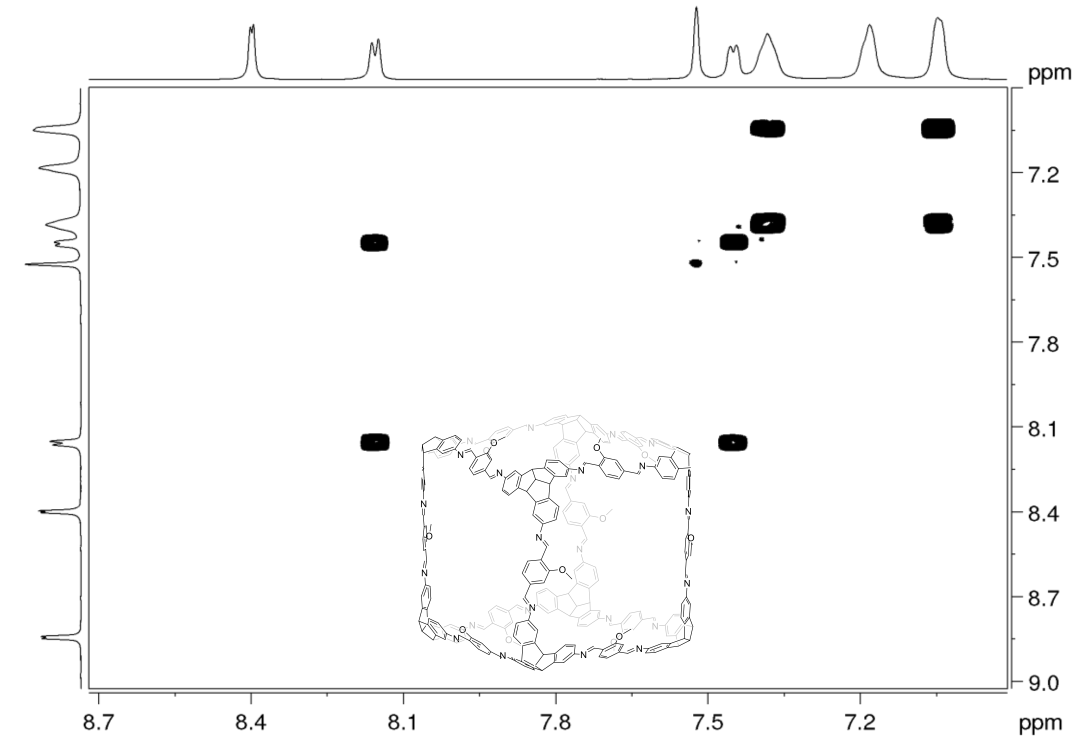

Figure 287. Partial ^{1}H-^{1}H COSY NMR spectrum (600 MHz, $C_2D_2Cl_4$) of **H/OMe-cube**.

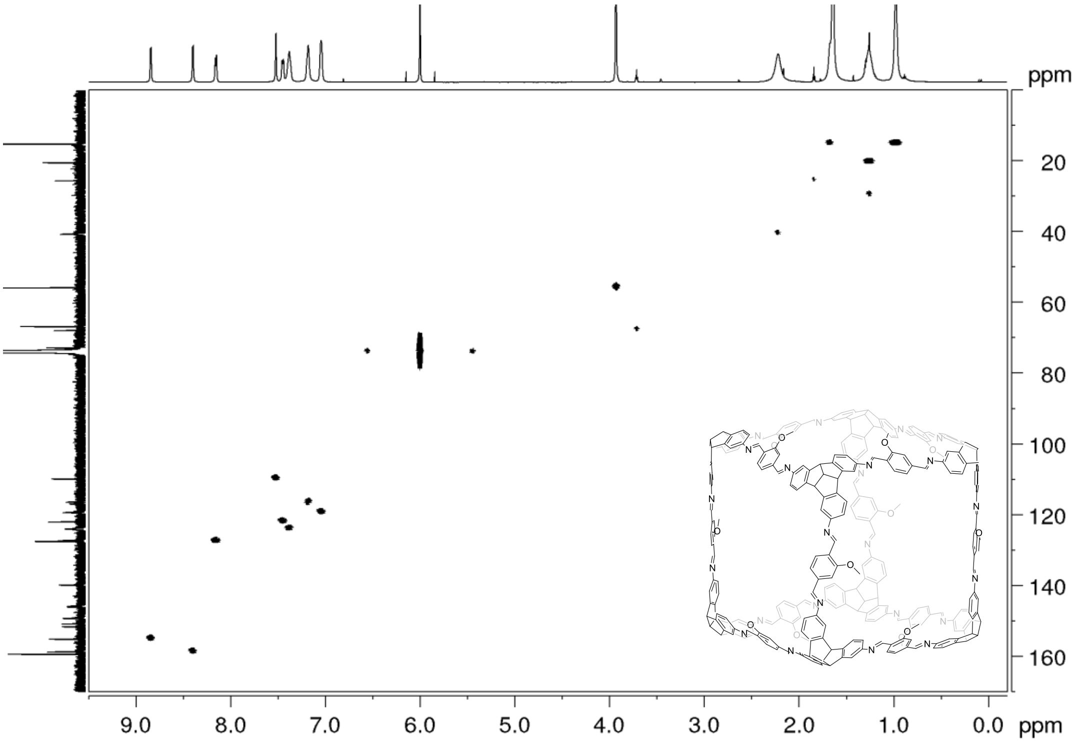

Figure 288. ^{1}H-^{13}C HSQC NMR (600 MHz and 151 MHz, $C_2D_2Cl_4$) spectrum of **H/OMe-cube**.

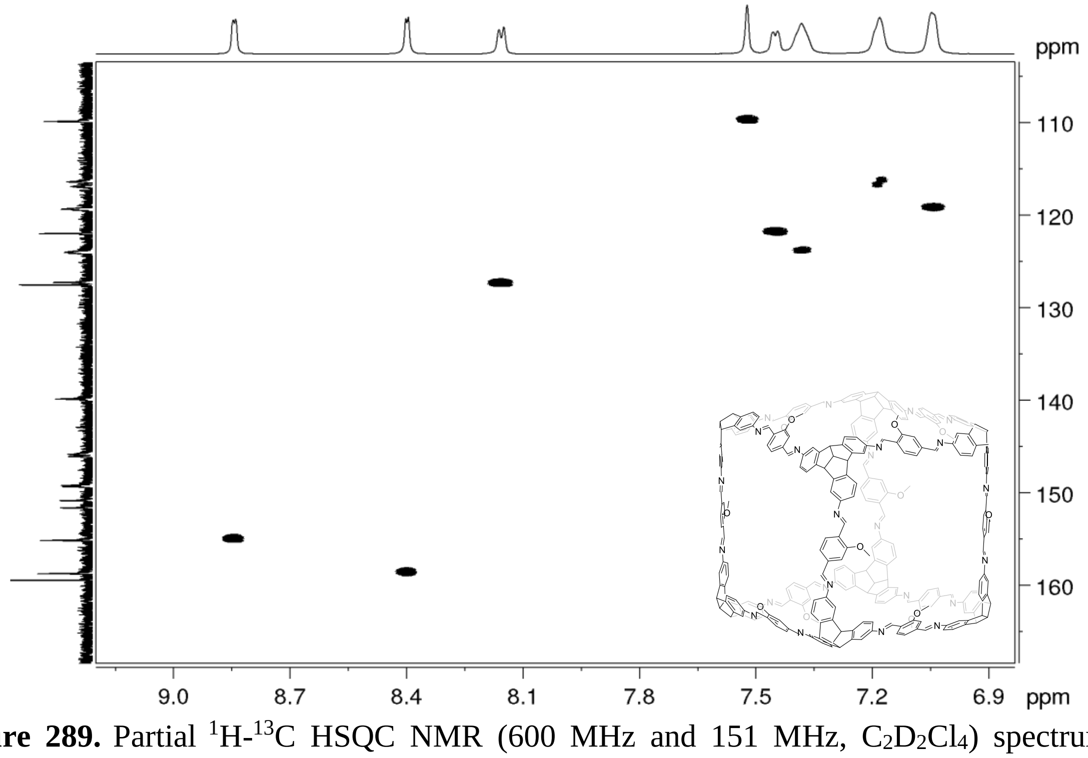

Figure 289. Partial ^{1}H-^{13}C HSQC NMR (600 MHz and 151 MHz, $C_2D_2Cl_4$) spectrum of **H/OMe-cube**.

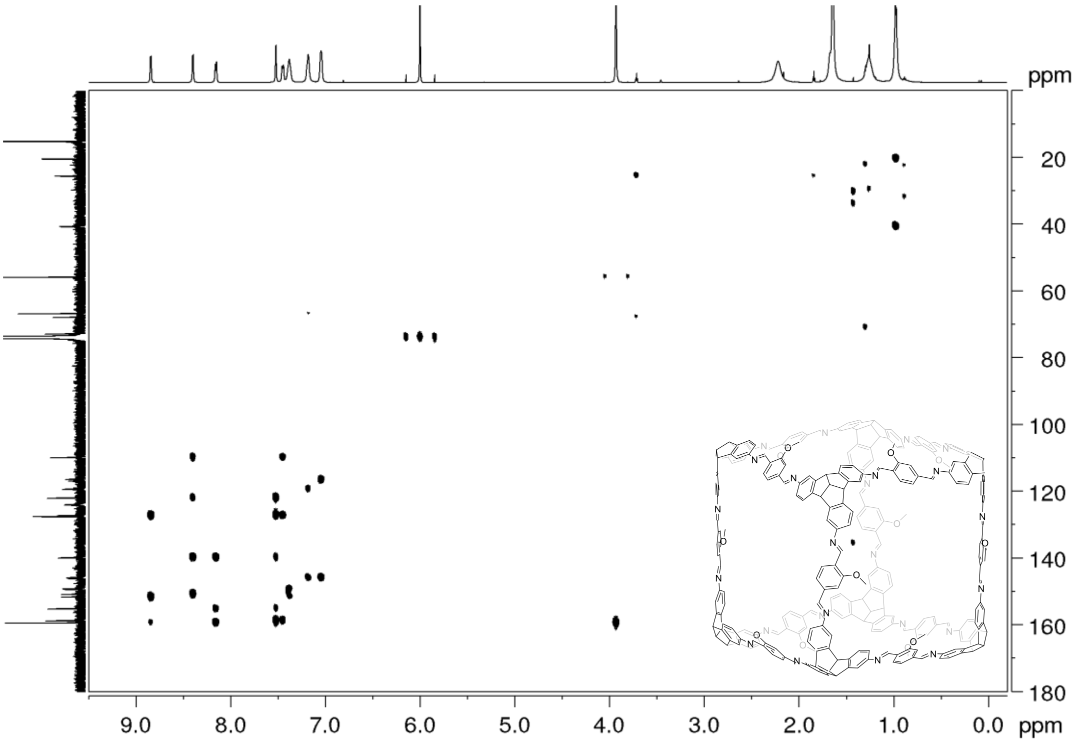

Figure 290. ^{1}H-^{13}C HMBC NMR (600 MHz and 151 MHz, $C_2D_2Cl_4$) spectrum of **H/OMe-cube**.

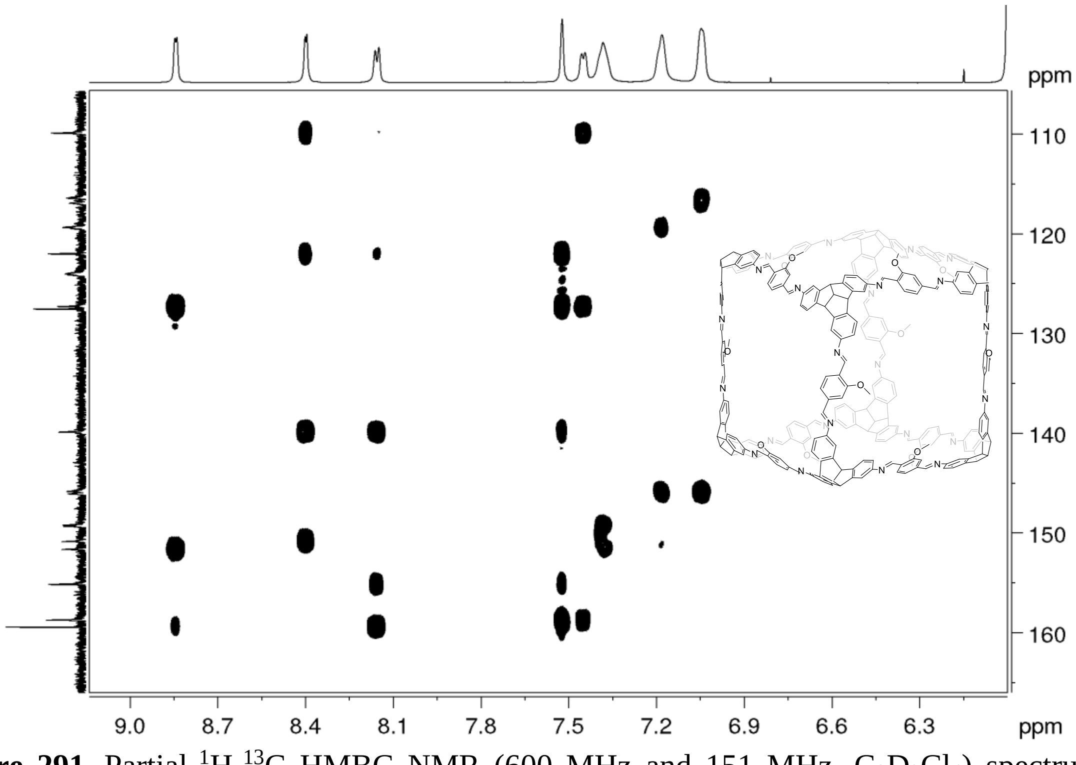

Figure 291. Partial ^{1}H-^{13}C HMBC NMR (600 MHz and 151 MHz, $C_2D_2Cl_4$) spectrum of **H/OMe-cube**.

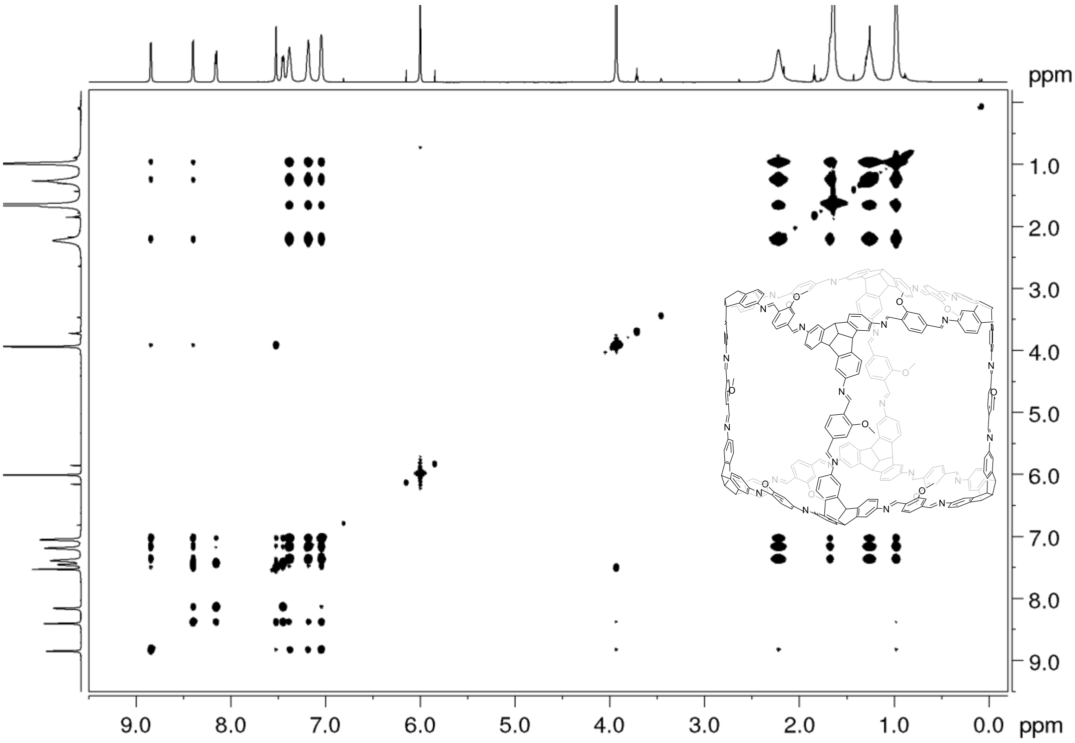

Figure 292. ^1H-^1H NOESY NMR (600 MHz, $C_2D_2Cl_4$) spectrum of **H/OMe-cube**.

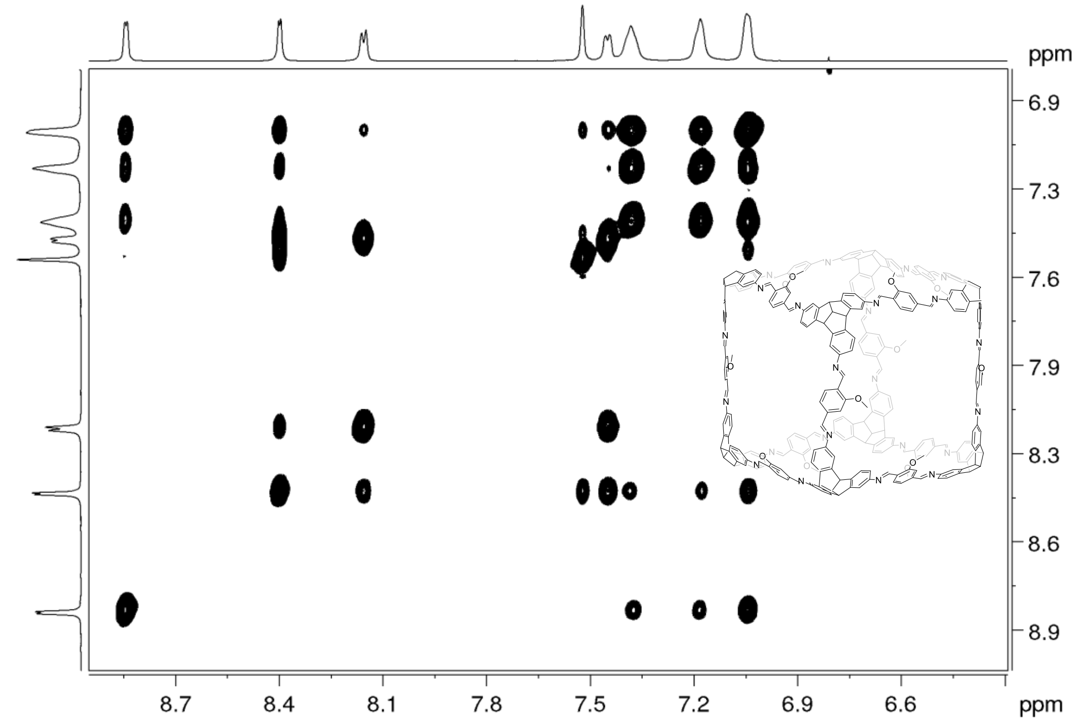

Figure 293. Partial ^1H-^1H NOESY NMR (600 MHz, $C_2D_2Cl_4$) spectrum of **H/OMe-cube**.

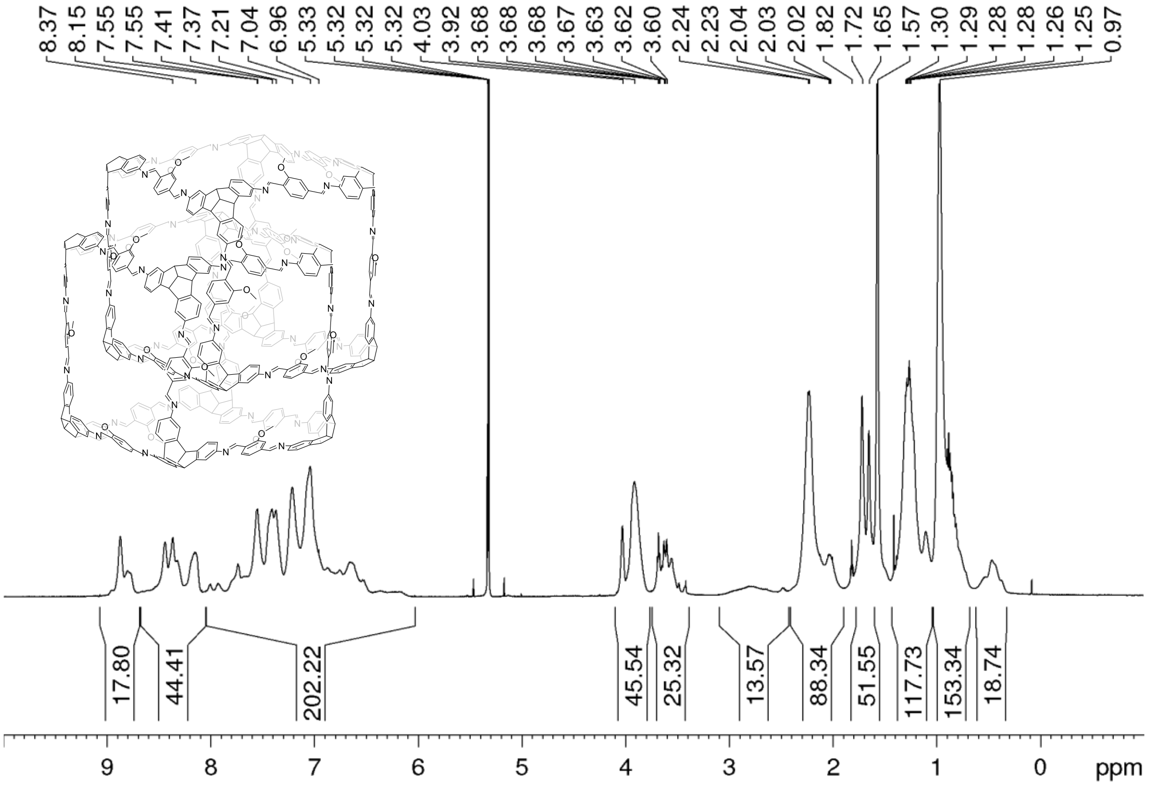

Figure 294. ^{1}H NMR (600 MHz, CD_2Cl_2) spectrum of **(H/OMe-cube)$_2$**.

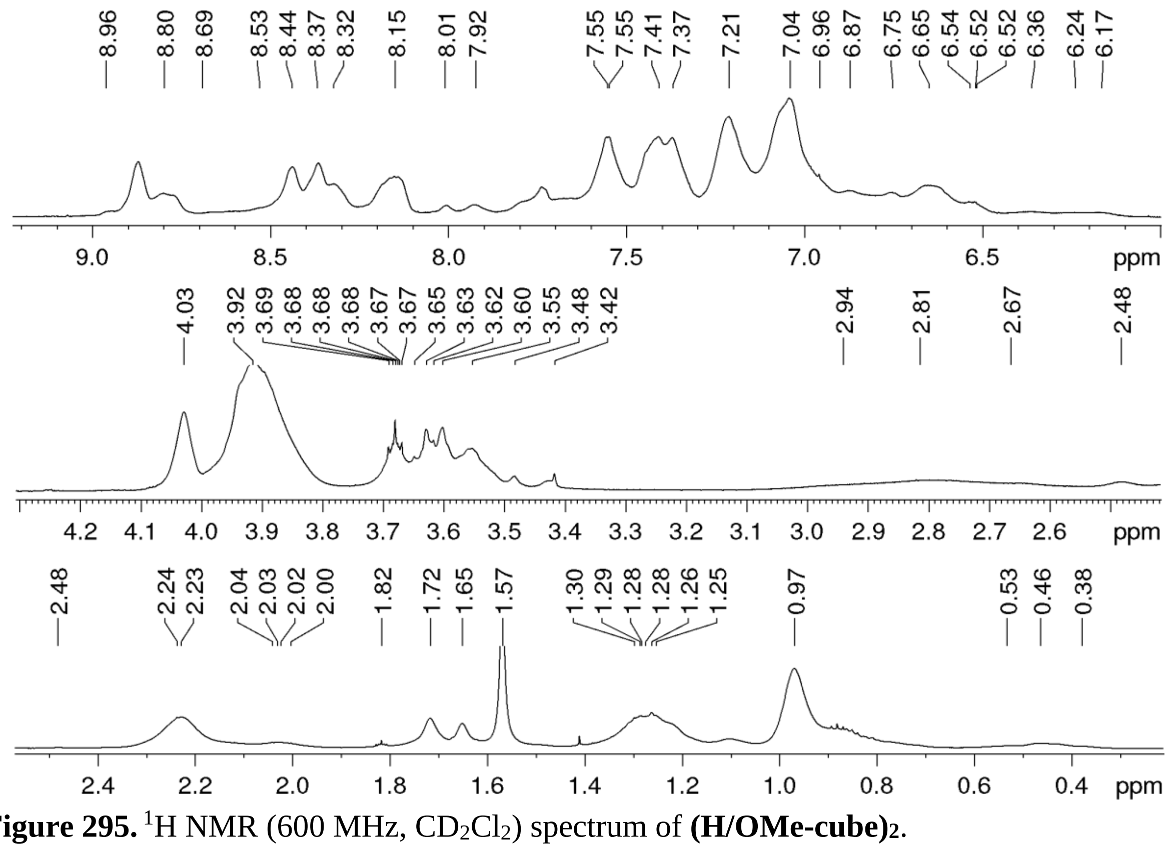

Figure 295. ^{1}H NMR (600 MHz, CD_2Cl_2) spectrum of **(H/OMe-cube)$_2$**.

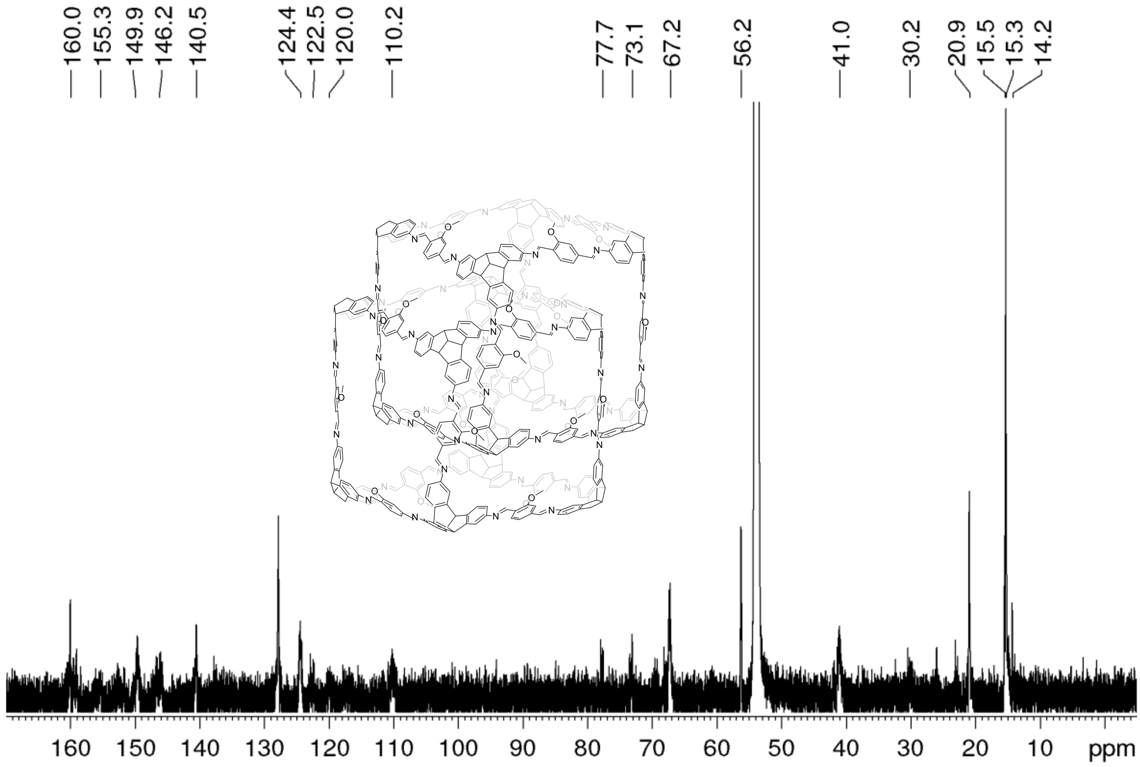

Figure 296. ^{13}C NMR (151 MHz, CD_2Cl_2) spectrum of **(H/OMe-cube)$_2$**.

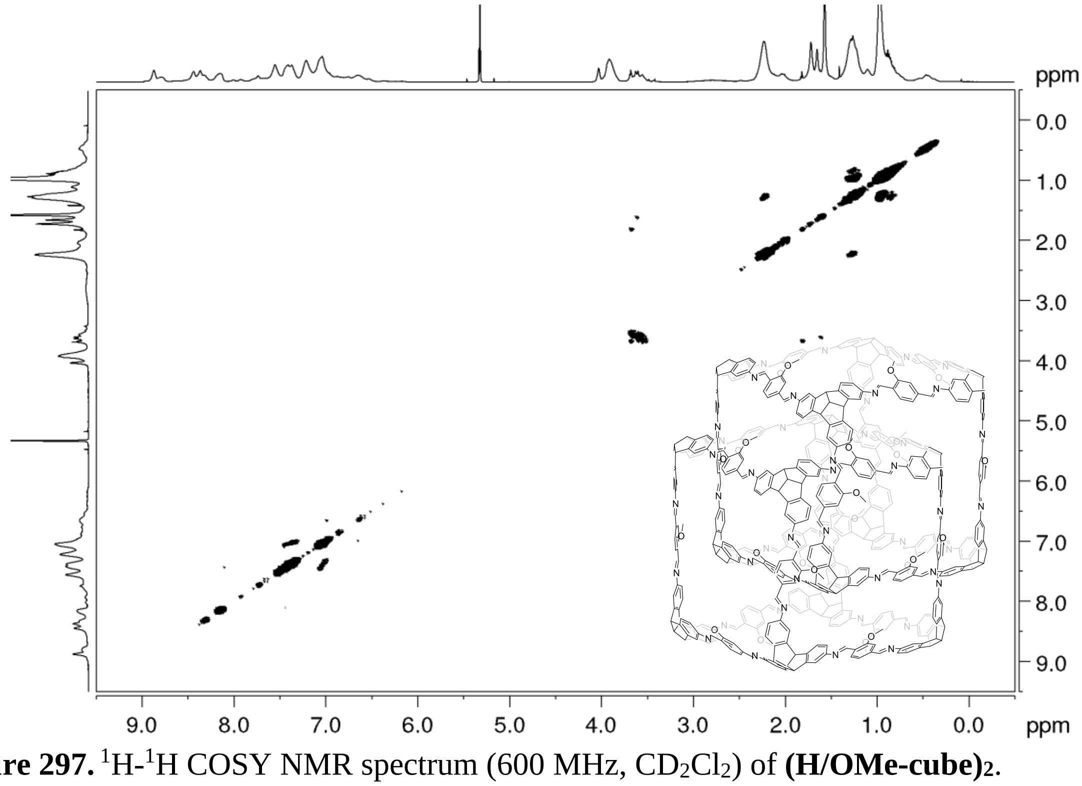

Figure 297. ^{1}H-^{1}H COSY NMR spectrum (600 MHz, CD_2Cl_2) of **(H/OMe-cube)$_2$**.

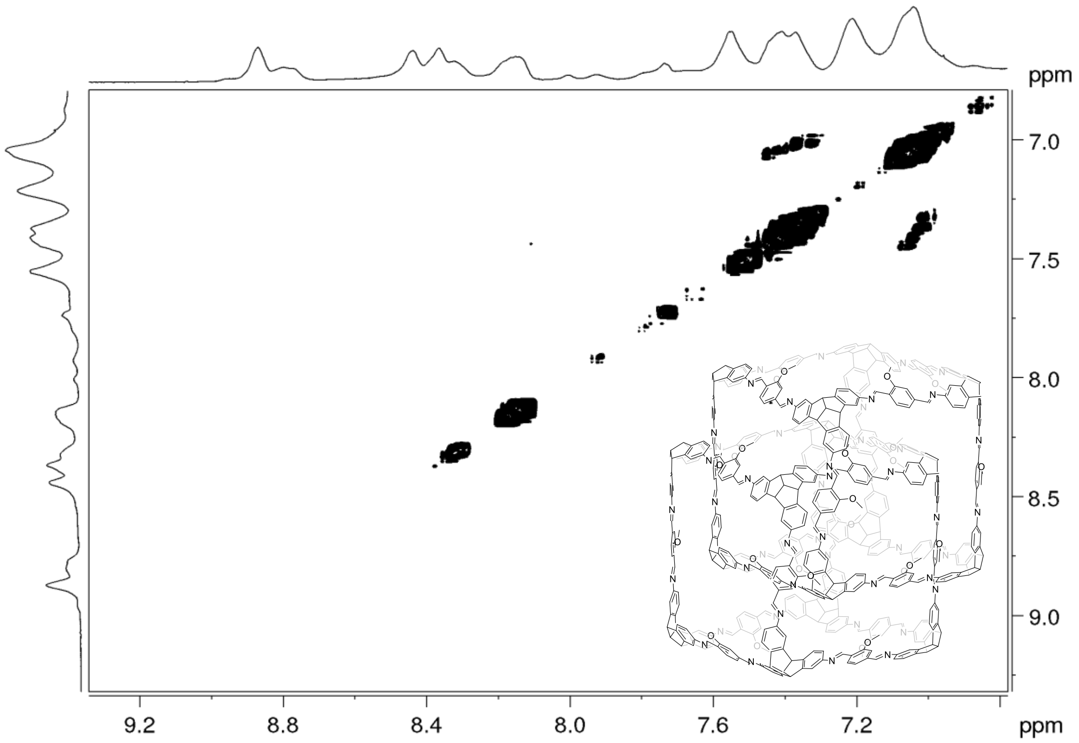

Figure 298. Partial ^{1}H-^{1}H COSY NMR spectrum (600 MHz, CD_2Cl_2) of **(H/OMe-cube)$_2$**.

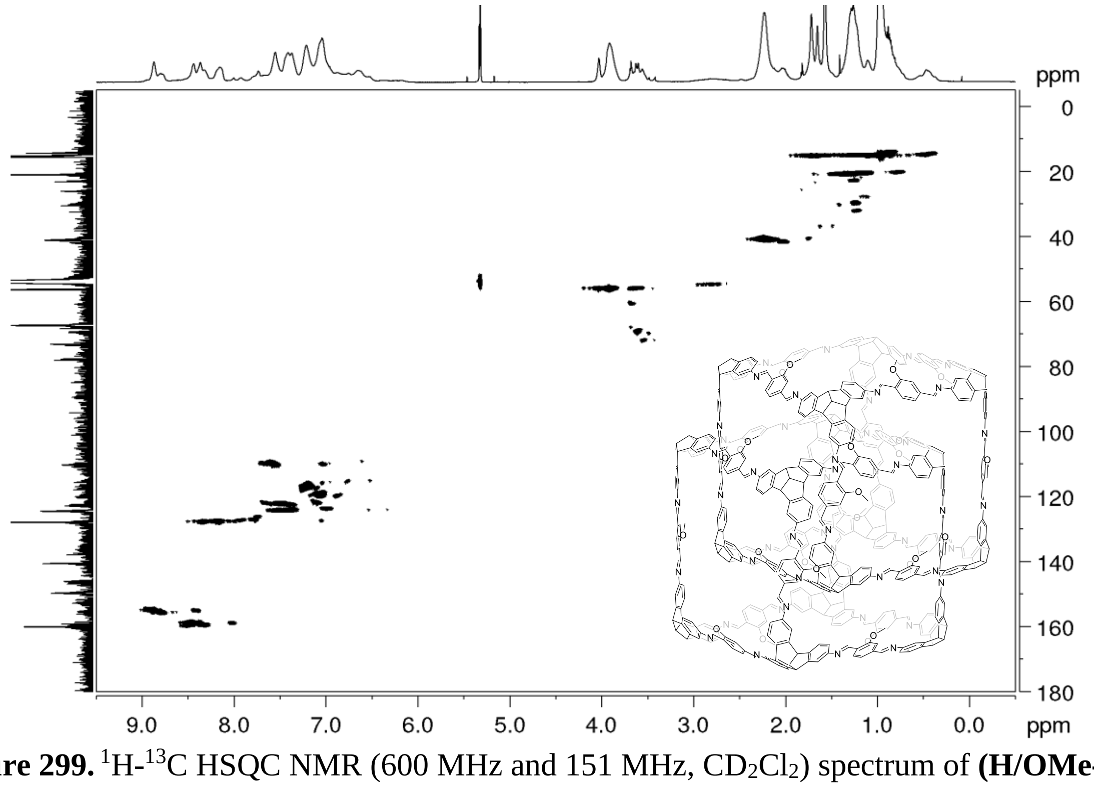

Figure 299. ^{1}H-^{13}C HSQC NMR (600 MHz and 151 MHz, CD_2Cl_2) spectrum of **(H/OMe-cube)$_2$**.

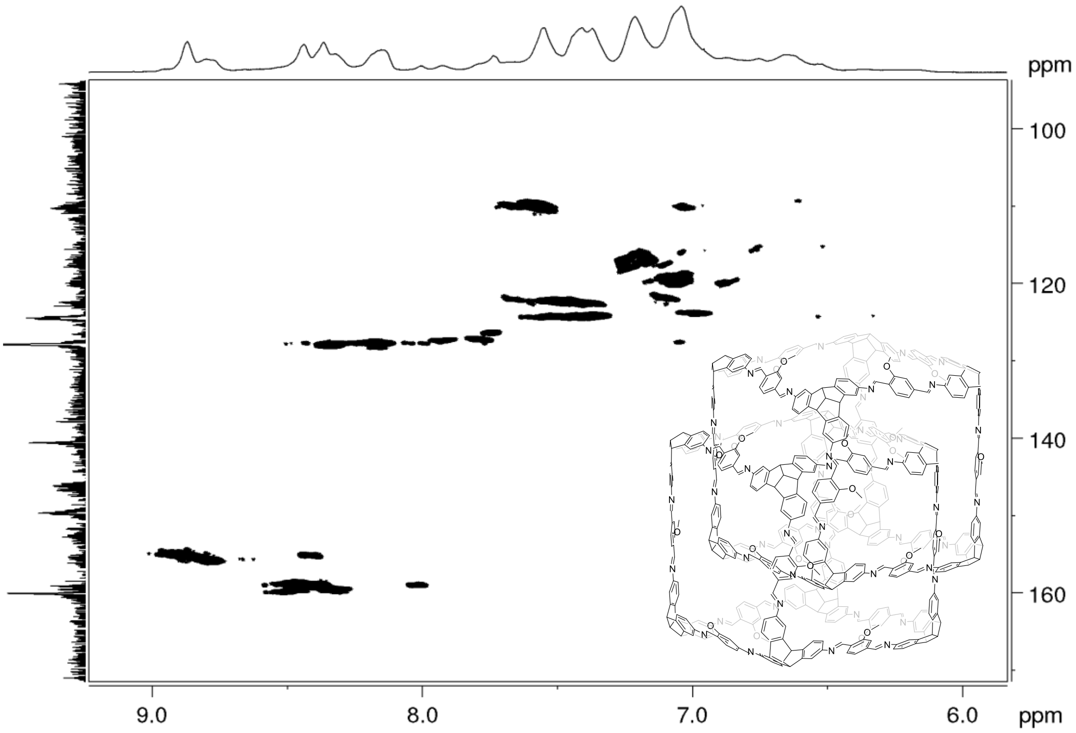

Figure 300. Partial ^{1}H-^{13}C HSQC NMR (600 MHz and 151 MHz, CD_2Cl_2) spectrum of **(H/OMe-cube)$_2$**.

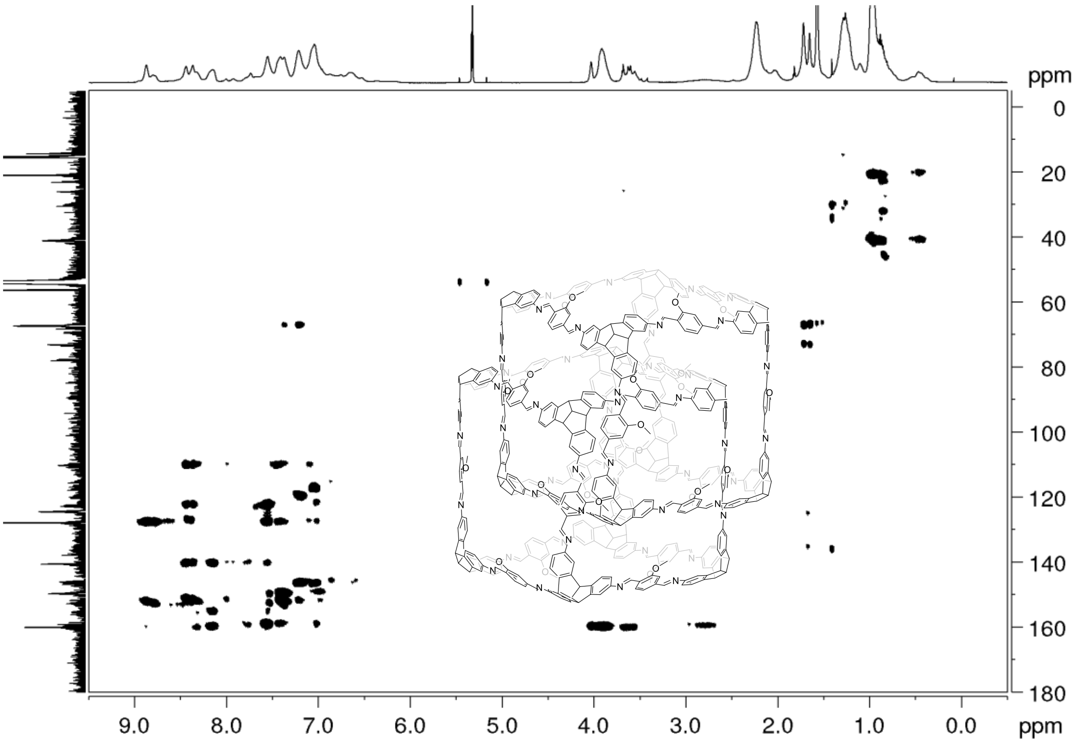

Figure 301. ^{1}H-^{13}C HMBC NMR (600 MHz and 151 MHz, CD_2Cl_2) spectrum of **(H/OMe-cube)$_2$**.

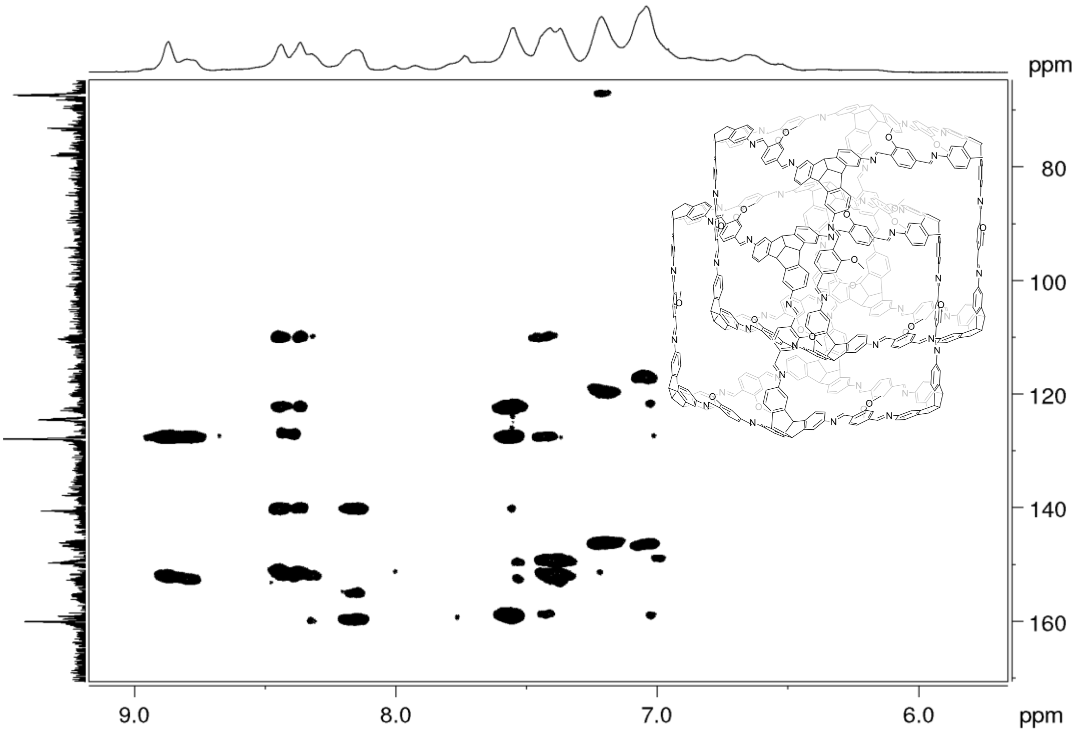

Figure 302. Partial ^{1}H-^{13}C HMBC NMR (600 MHz and 151 MHz, CD_2Cl_2) spectrum of **(H/OMe-cube)$_2$**.

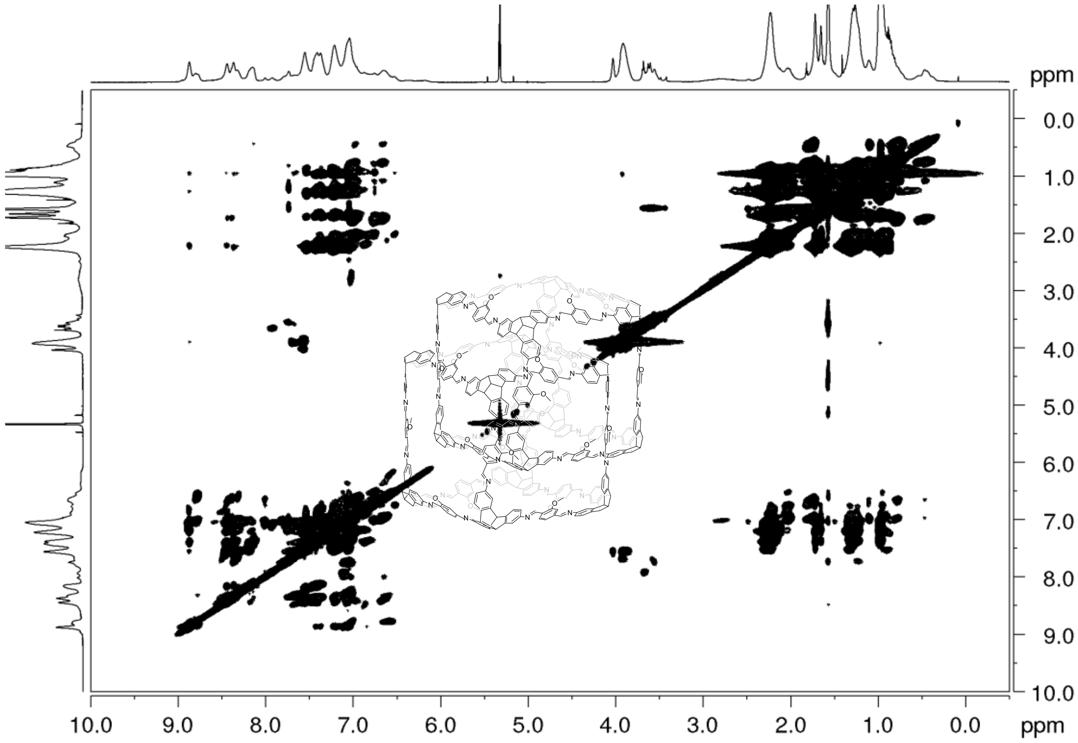

Figure 303. ^{1}H-^{1}H NOESY NMR (600 MHz, CD_2Cl_2) spectrum of **(H/OMe-cube)$_2$**.

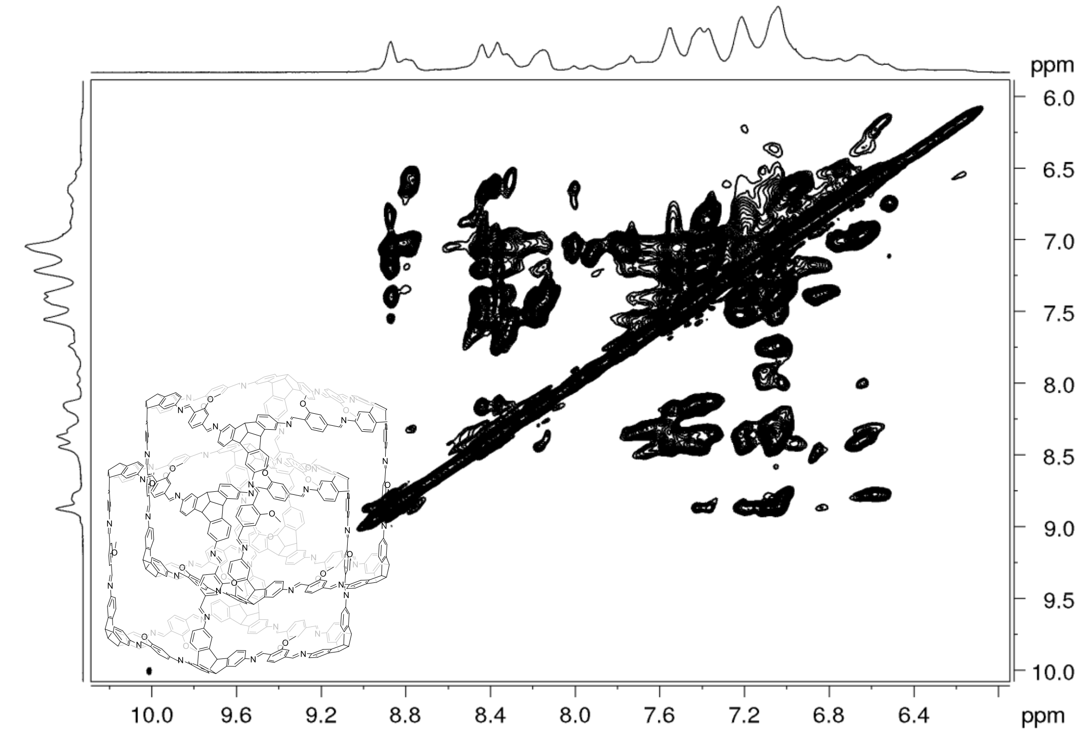

Figure 304. Partial ^{1}H-^{1}H NOESY NMR (600 MHz, CD_2Cl_2) spectrum of **(H/OMe-cube)$_2$**.

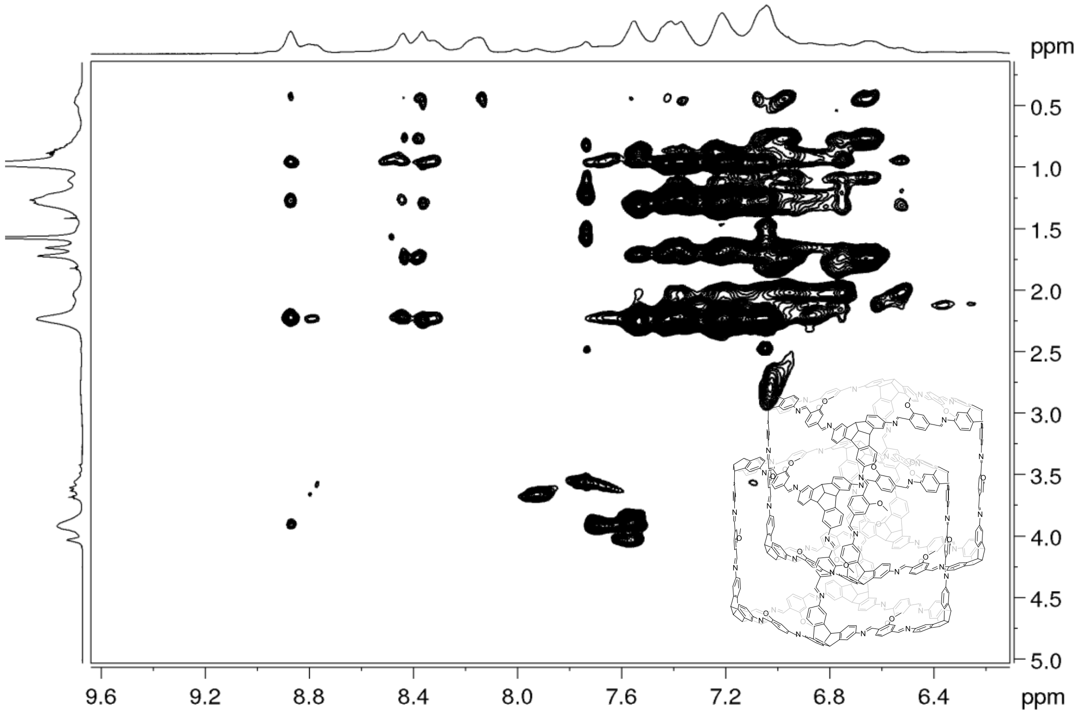

Figure 305. Partial ^{1}H-^{1}H NOESY NMR (600 MHz, CD_2Cl_2) spectrum of **(H/OMe-cube)$_2$**.

3. **DOSY experiments**

DOSY NMR experiments were calibrated using self-diffusion values for the used solvents (D_{solv}).[S8] Tha solvodynamic radii were calculated using the semi-empirical modification of the Stokes-Einstein equation proposed by Chen and Chen. [S9] This equation was solved for r_s using values of r_{solv} and η from the literature. [S10]

$$D = \frac{k_B T}{\left(\frac{6}{1 + 0.695\left(\frac{r_{solv}}{r_s}\right)^{2.234}}\right)\pi\eta r_s}$$

D is the measured diffusion coefficient ($m^2 \cdot s^{-1}$)

k_B is Boltzmann constant ($1.3806485 \cdot 10\ m^2 \cdot kg \cdot s^{-2} \cdot K^{-1}$)

T is the temperature (K)

r_{solv} is the hydrodynamic radius of the solvent (m)

r_s is the hydrodynamic radius of the analyte (m)

η viscosity of the solvent at temperature T ($Kg \cdot m^{-1} \cdot s^{-1}$)

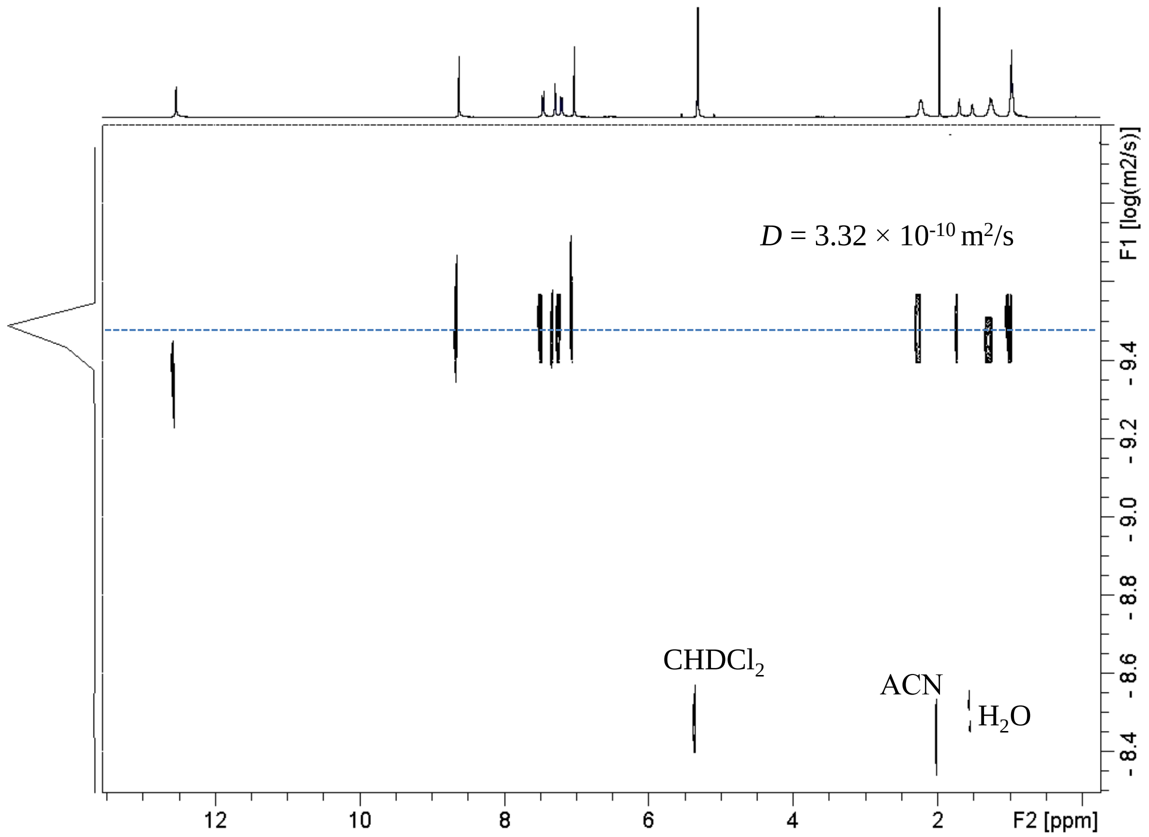

Figure 306. DOSY NMR (400 MHz, 295 K, CD_2Cl_2) spectrum **OH-cube**.

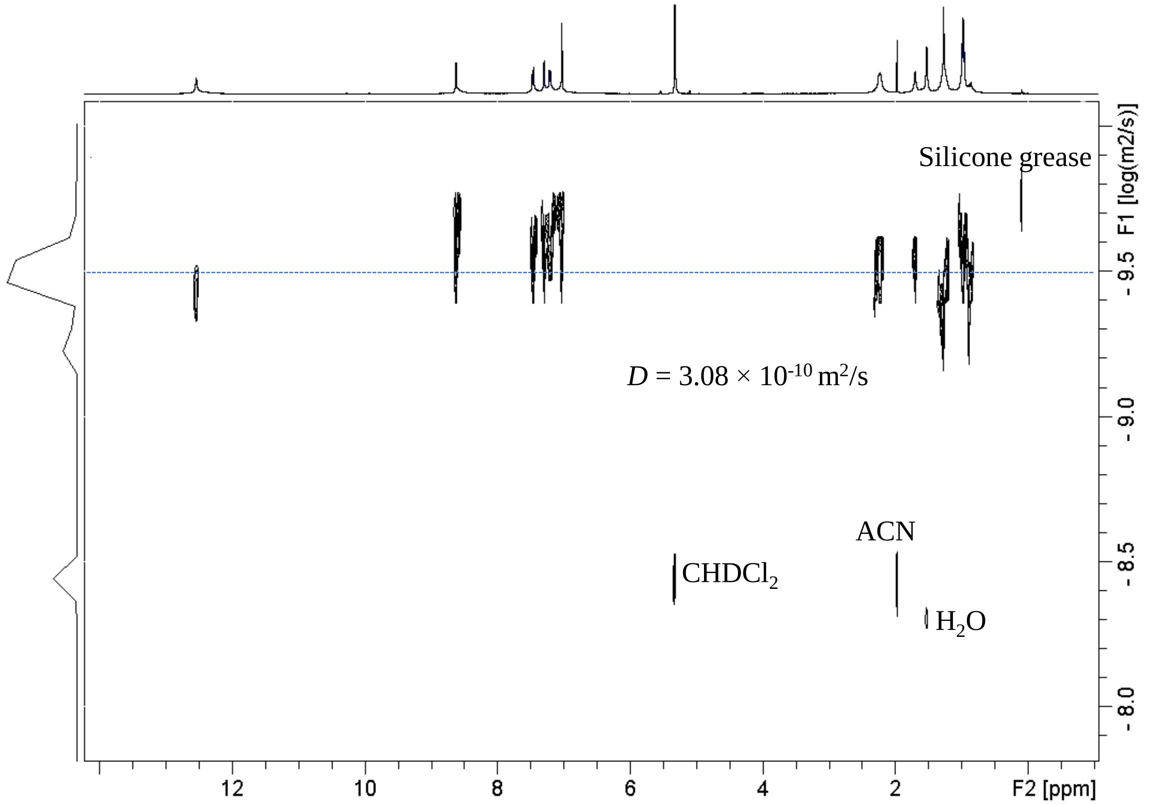

Figure 307. DOSY NMR (400 MHz, 295 K, CD_2Cl_2) spectrum ^{15}N labelled ***OH-cube**.

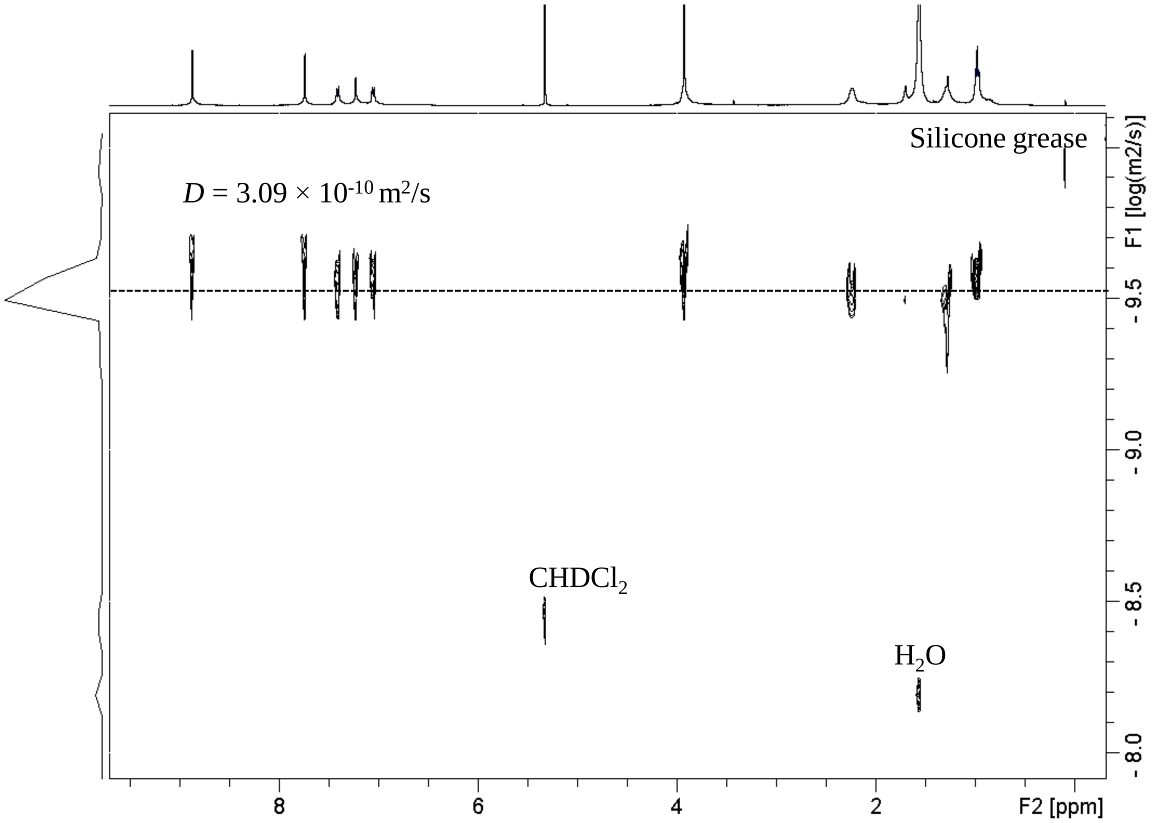

Figure 308. DOSY NMR (400 MHz, 295 K, CD_2Cl_2) spectrum of **OMe-cube**.

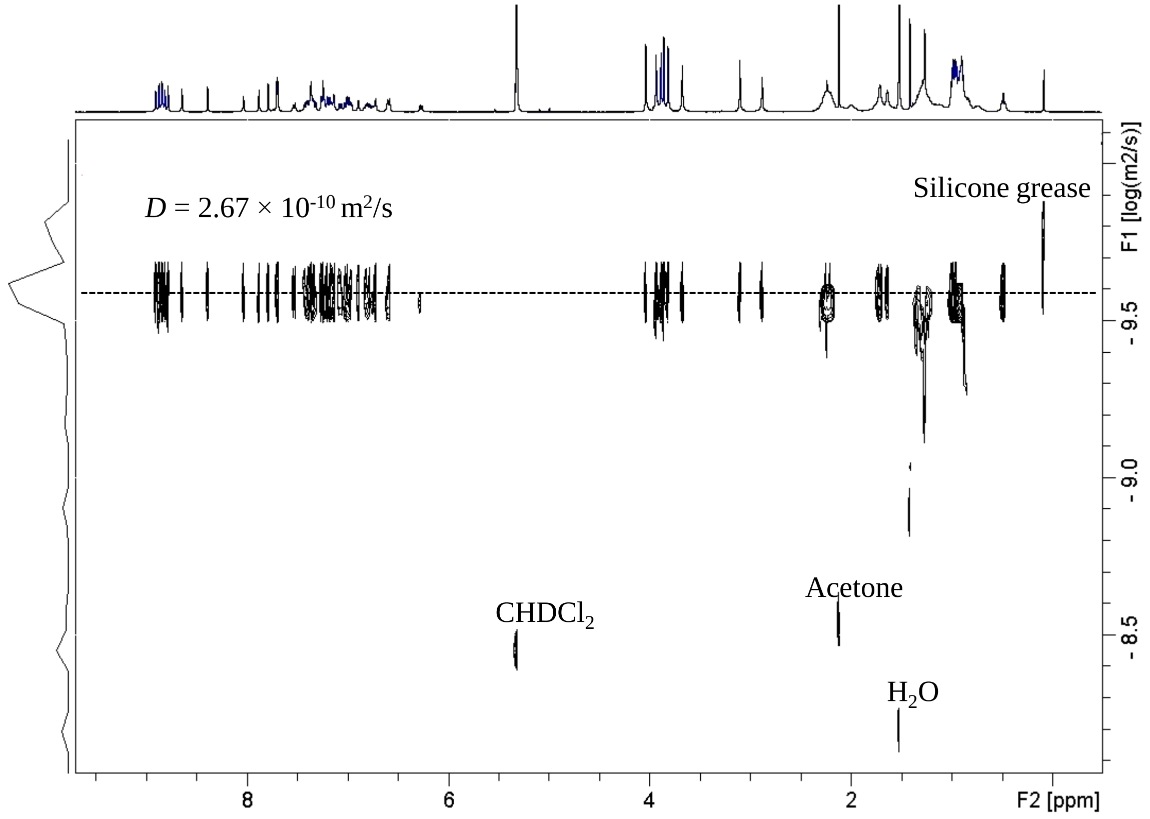

Figure 309. DOSY NMR (400 MHz, 295 K, CD_2Cl_2) spectrum of **(OMe-cube)$_2$**.

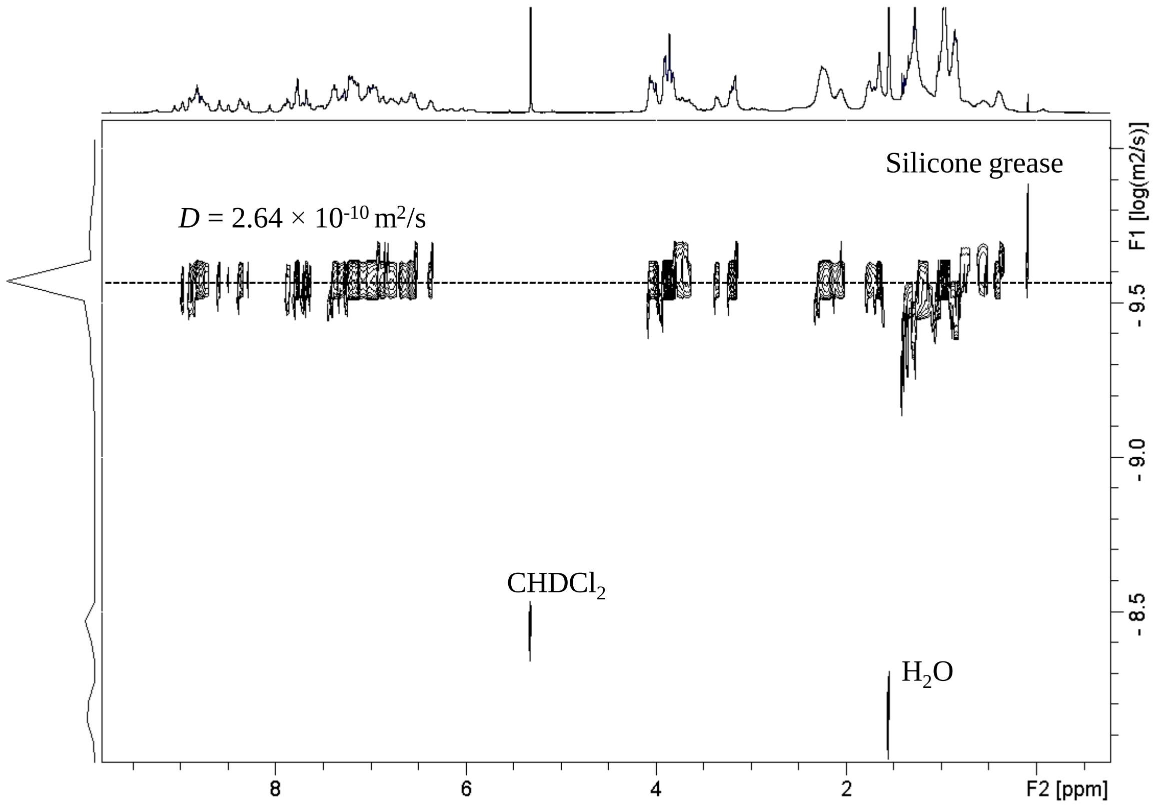

Figure 310. DOSY NMR (400 MHz, 295 K, CD_2Cl_2) spectrum of **(OMe-cube)$_3$**.

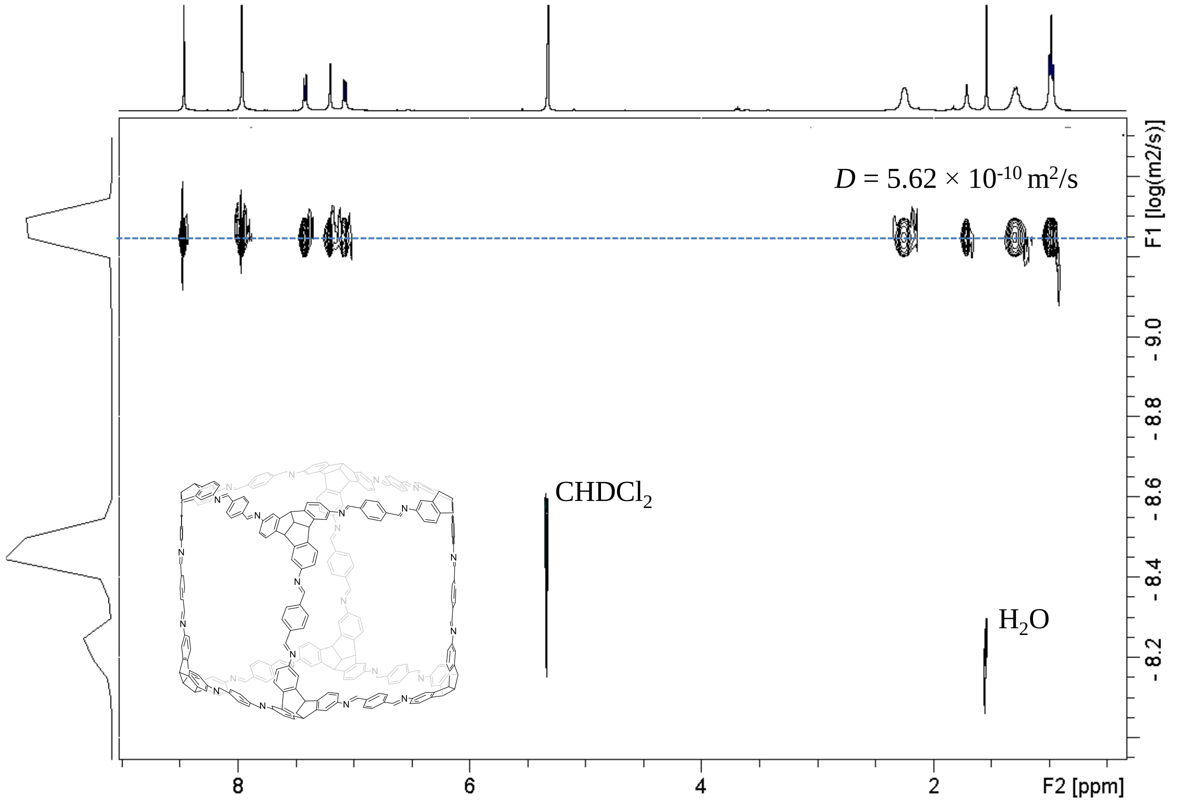

Figure 311. DOSY NMR (400 MHz, 295 K, CD_2Cl_2) spectrum of **H-cube**.

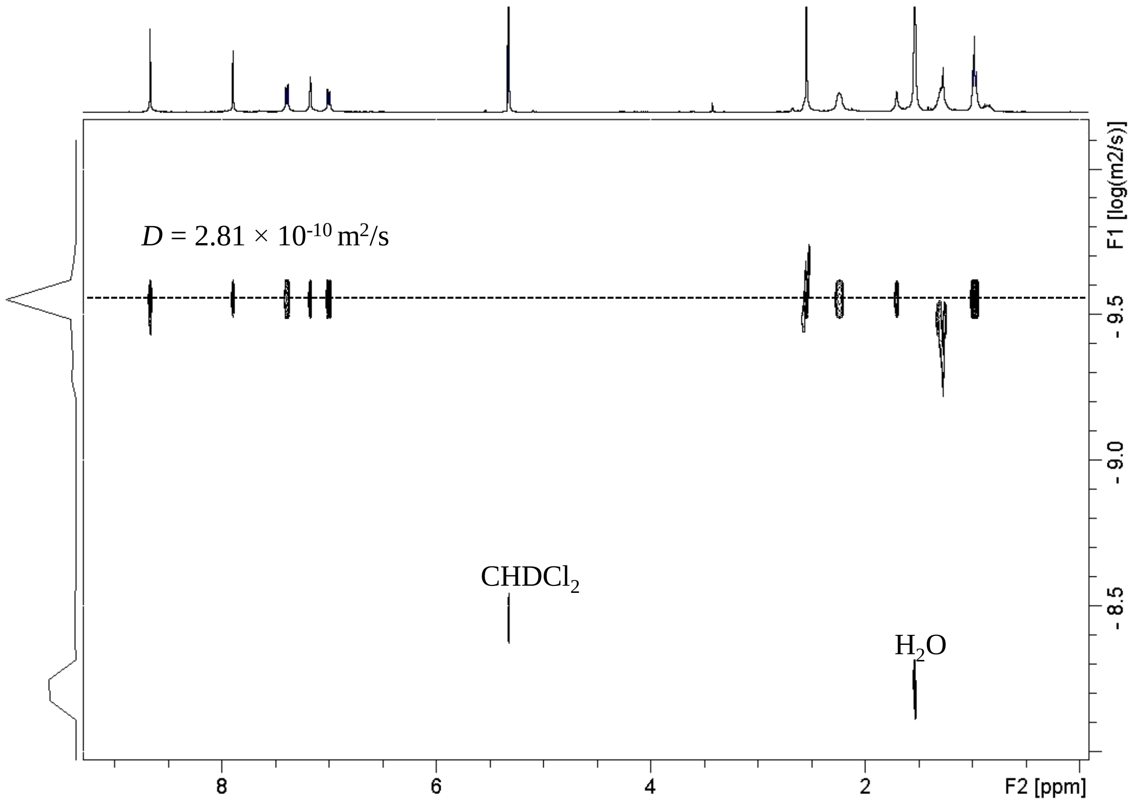

Figure 312. DOSY NMR (400 MHz, 295 K, CD_2Cl_2) spectrum of **Me-cube**.

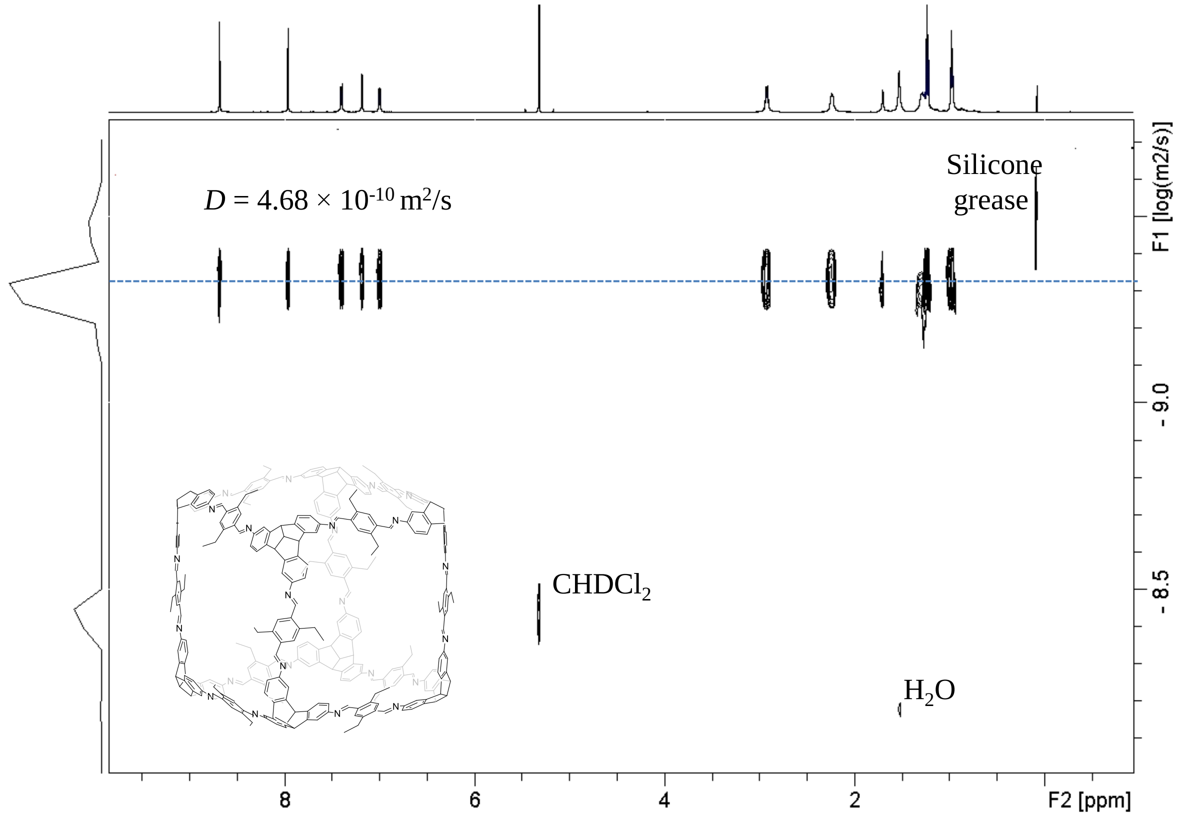

Figure 313. DOSY NMR (400 MHz, 295 K, CD_2Cl_2) spectrum of **Et-cube**.

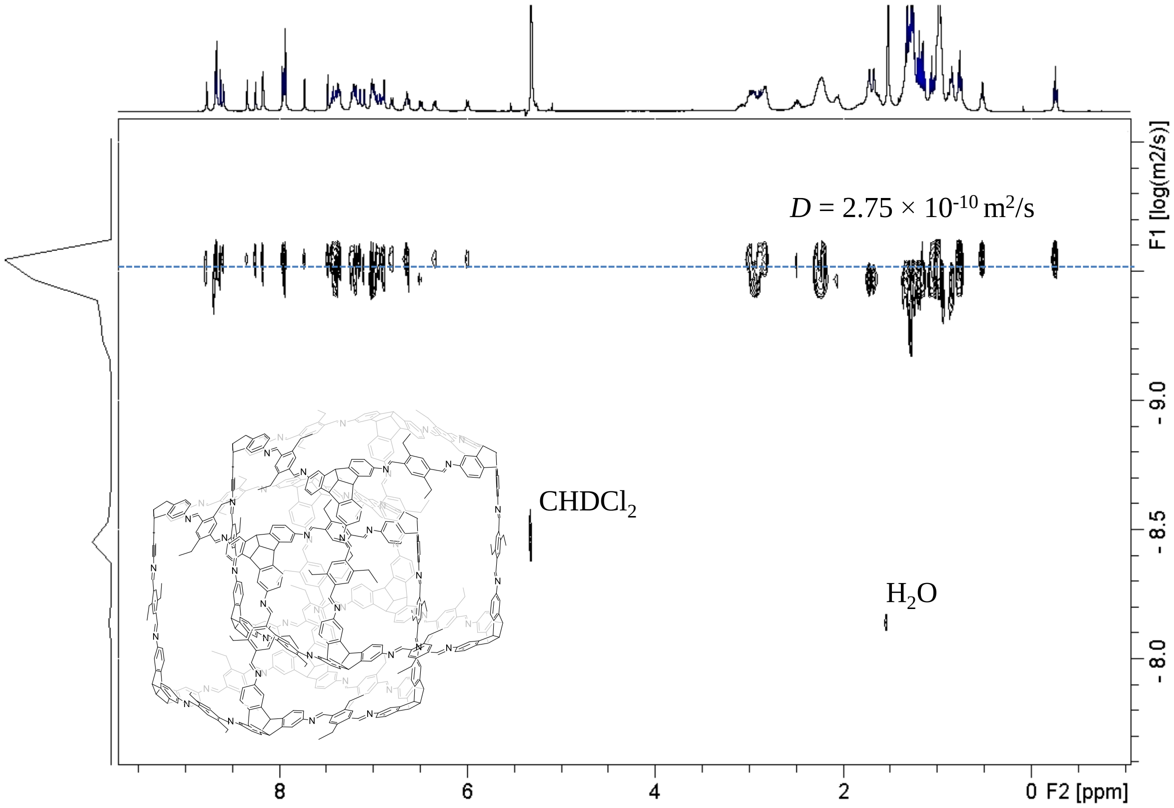

Figure 314. DOSY NMR (400 MHz, 295 K, CD_2Cl_2) spectrum of **(Et-cube)$_2$**.

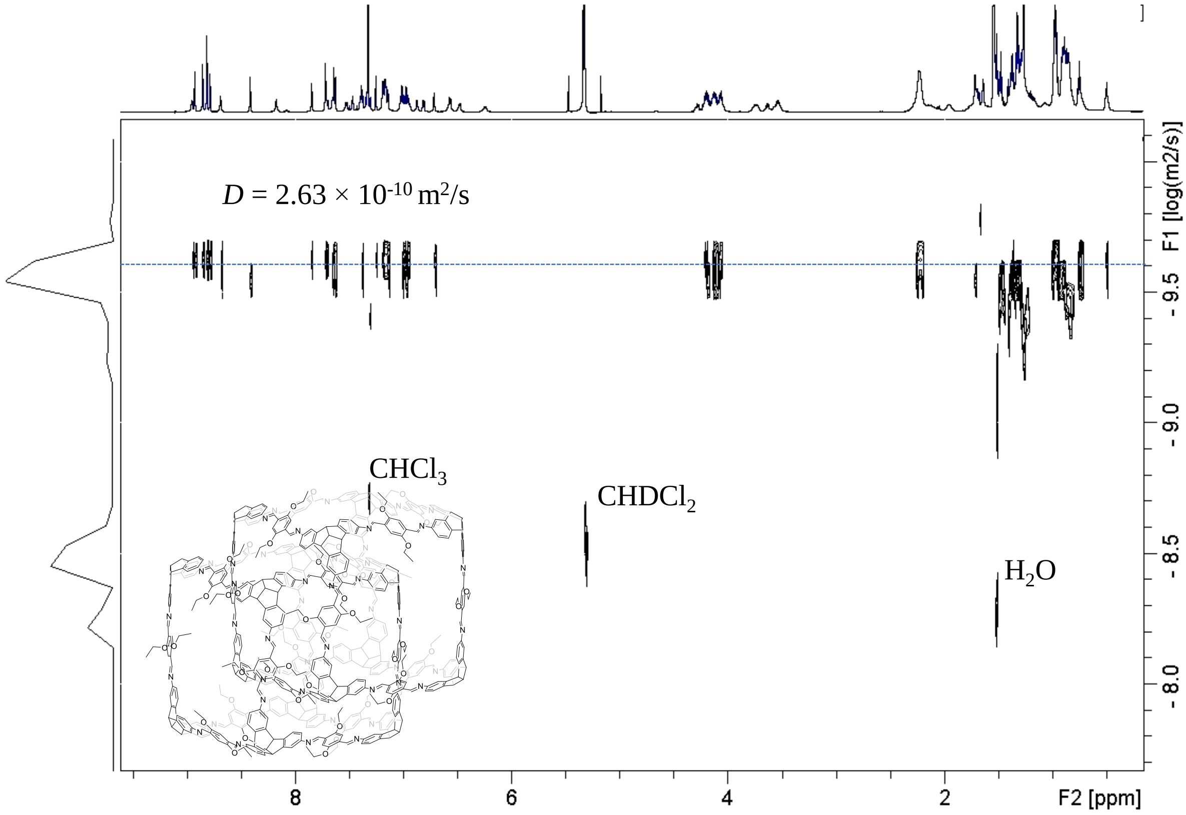

Figure 315. DOSY NMR (400 MHz, 295 K, CD_2Cl_2) spectrum of **(OEt-cube)$_2$**

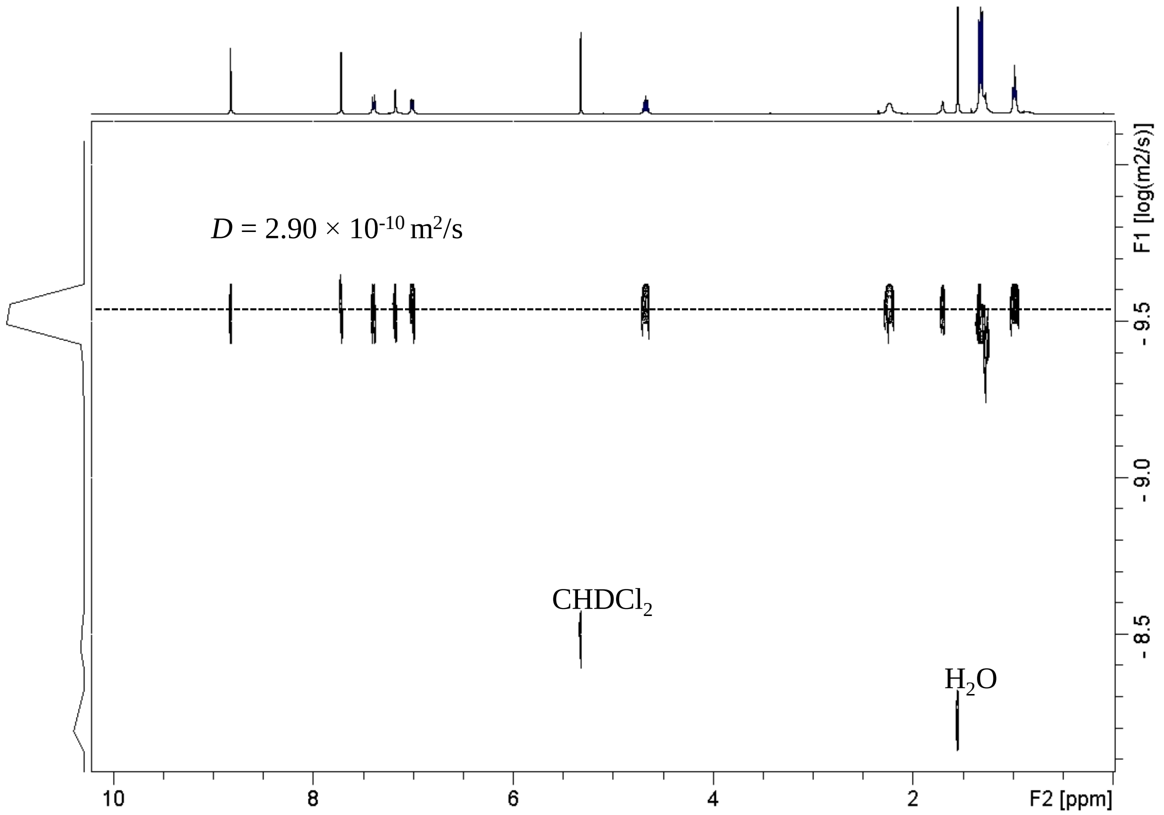

Figure 316. DOSY NMR (400 MHz, 295 K, CD_2Cl_2) spectrum of **O^{i}Pr-cube**.

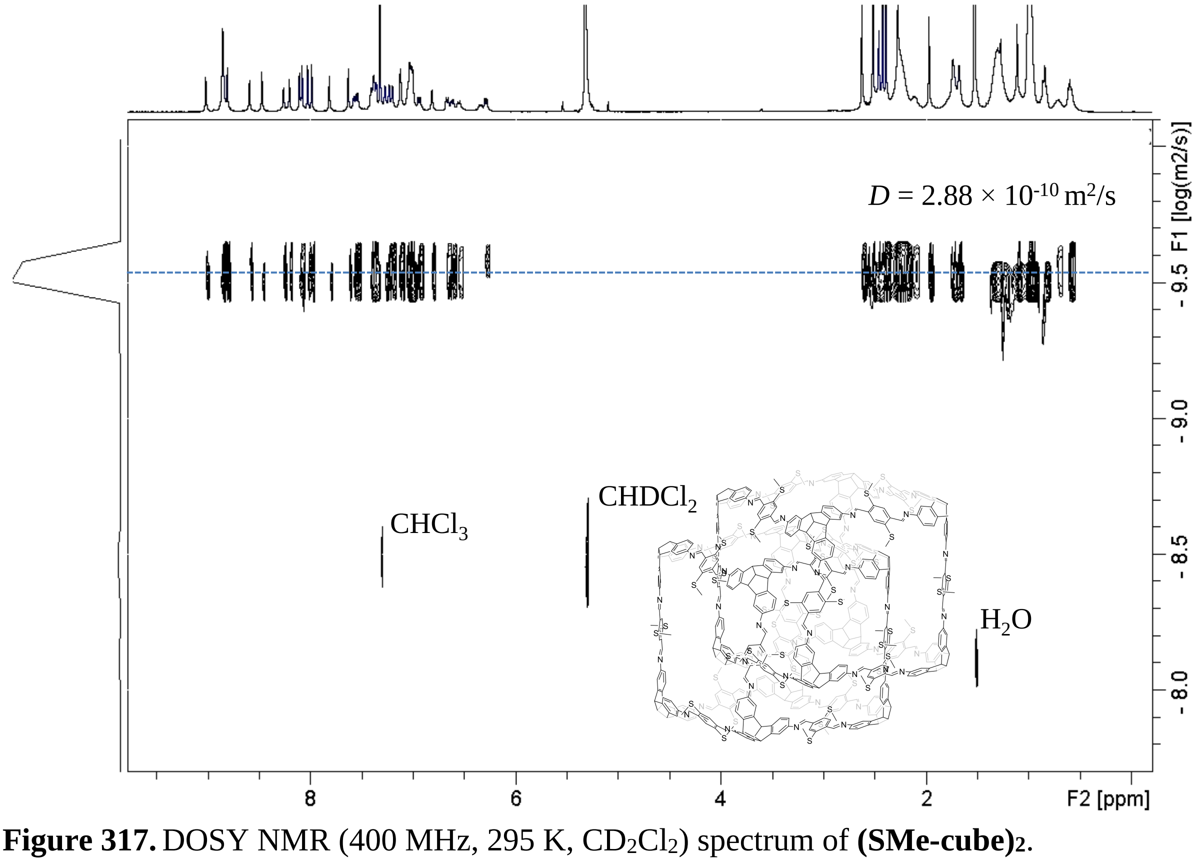

Figure 317. DOSY NMR (400 MHz, 295 K, CD_2Cl_2) spectrum of **(SMe-cube)$_2$**.

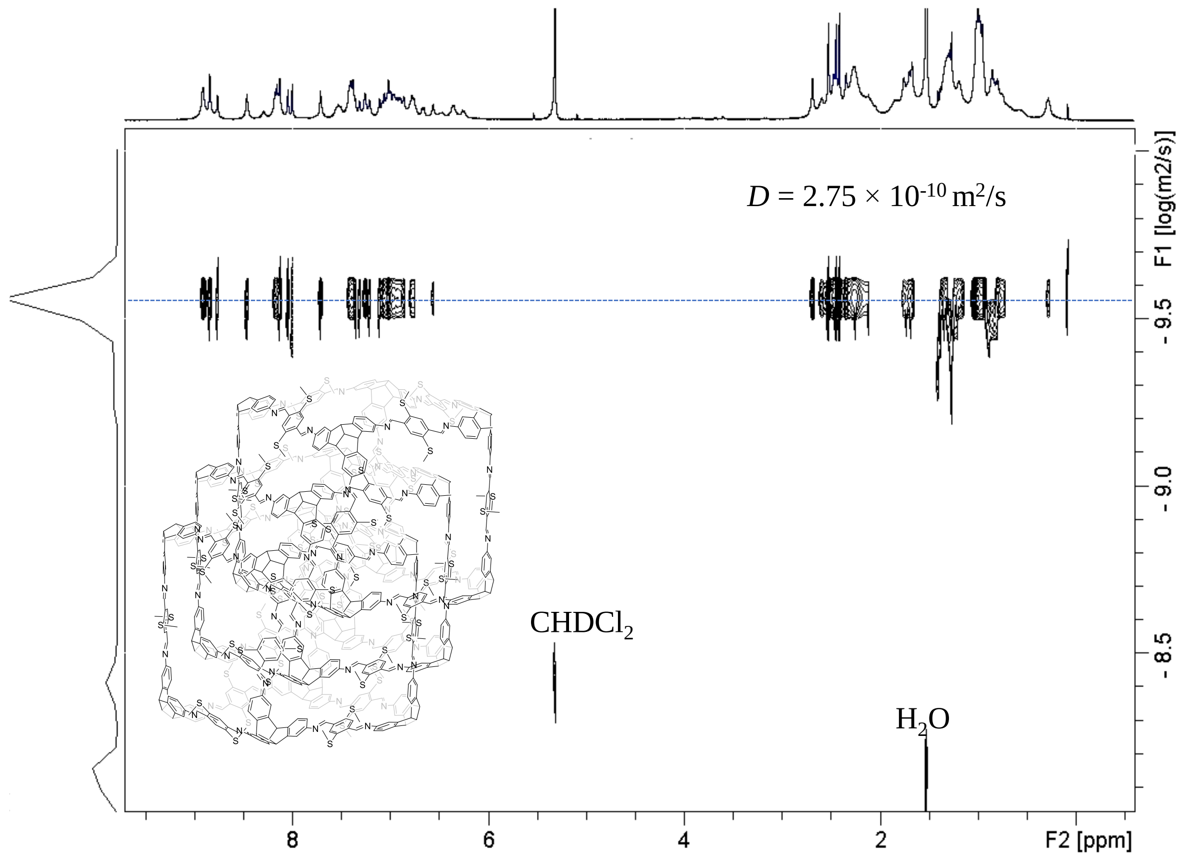

Figure 318. DOSY NMR (400 MHz, 295 K, CD_2Cl_2) spectrum of **(SMe-cube)$_3$**.

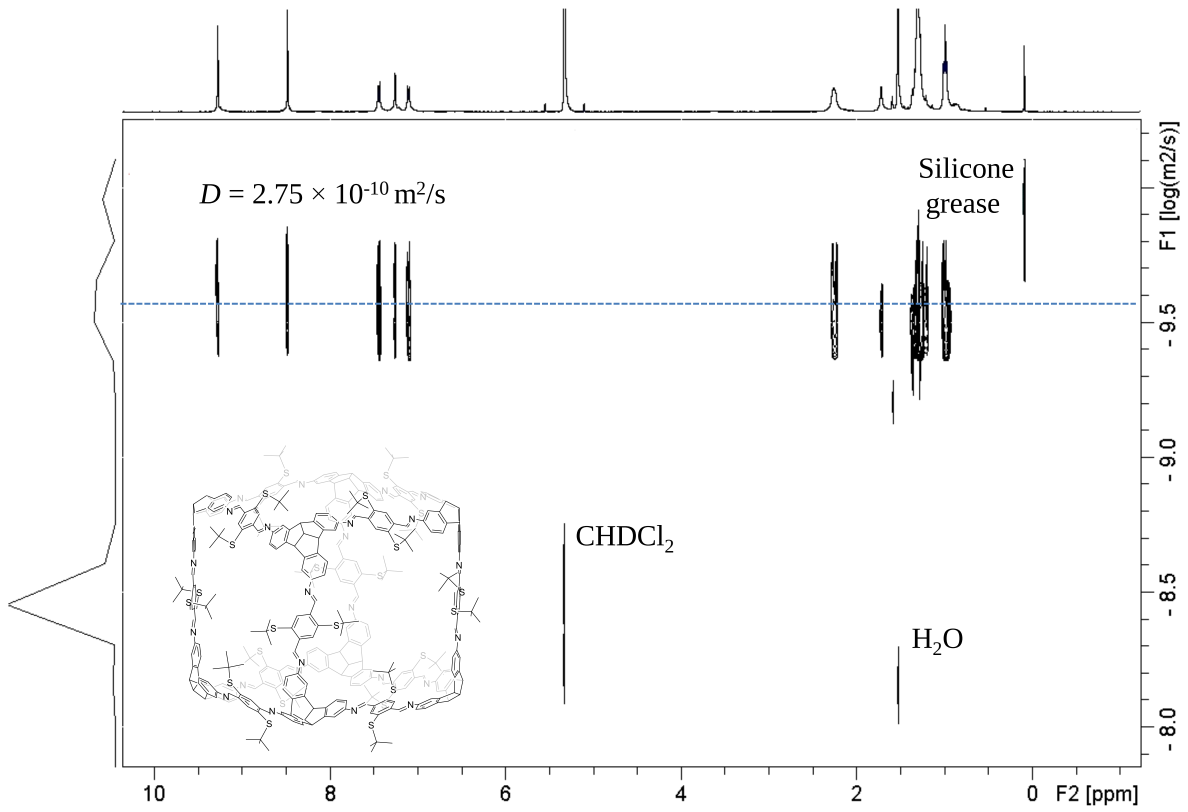

Figure 319. DOSY NMR (400 MHz, 295 K, CD_2Cl_2) spectrum of **SC(CH$_3$)$_3$-cube**.

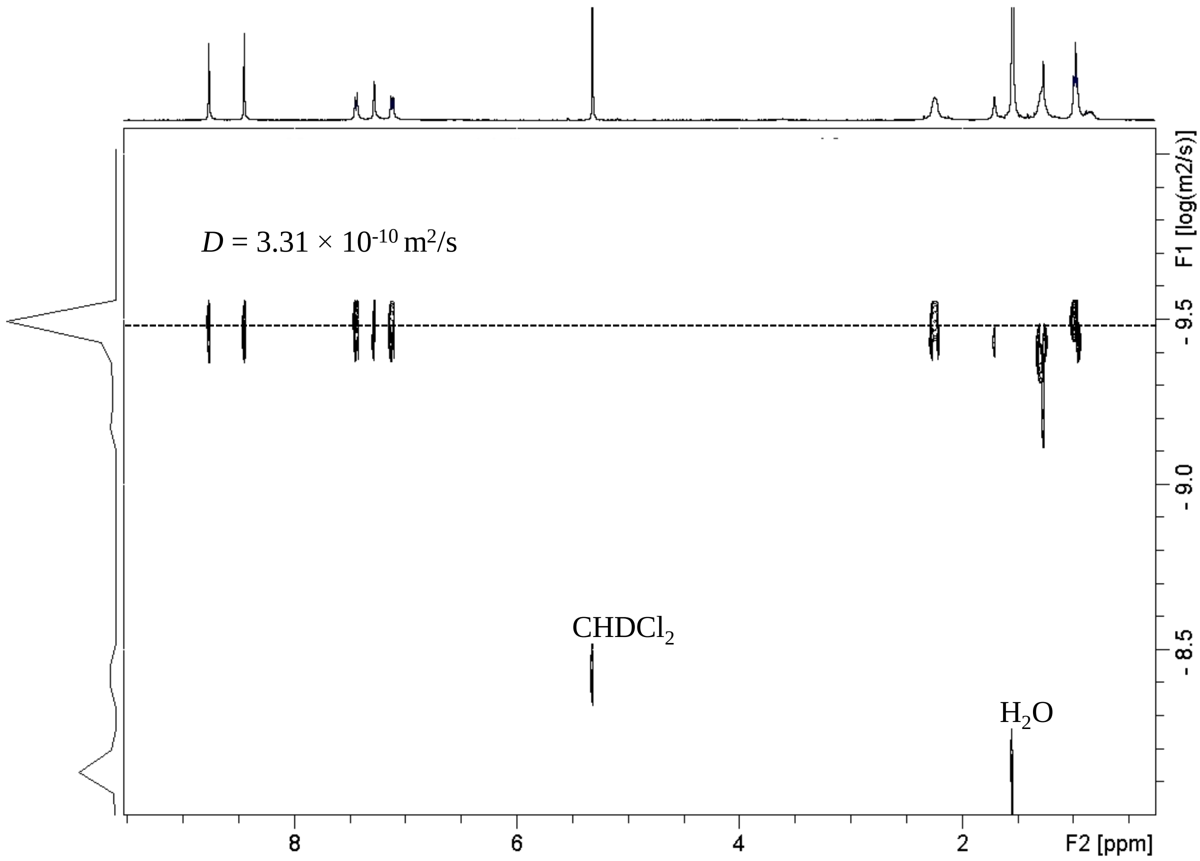

Figure 320. DOSY NMR (400 MHz, 295 K, CD_2Cl_2) spectrum of **Br-cube**.

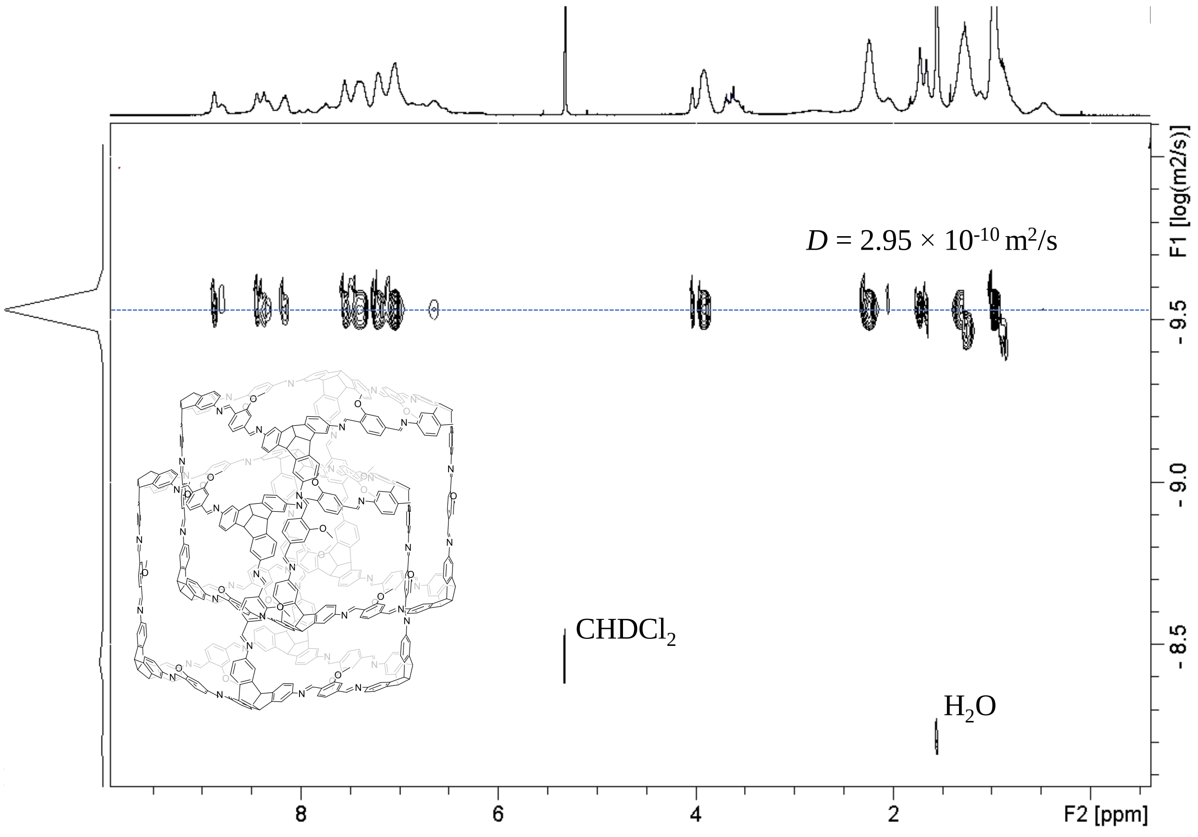

Figure 321. DOSY NMR (400 MHz, 295 K, CD_2Cl_2) spectrum of (**H/OMe-cube)₂**.

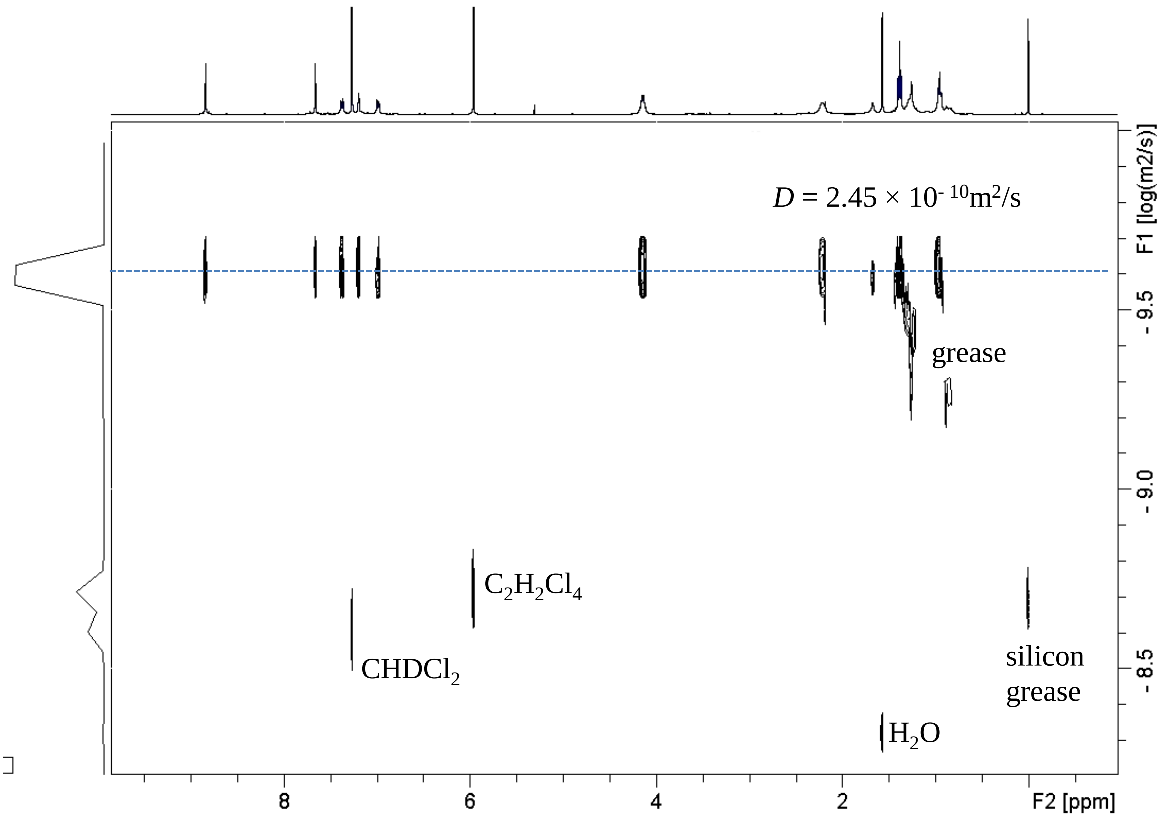

Figure 322. DOSY NMR (400 MHz, 295 K, $CDCl_3$) spectrum of **OEt-cube.**

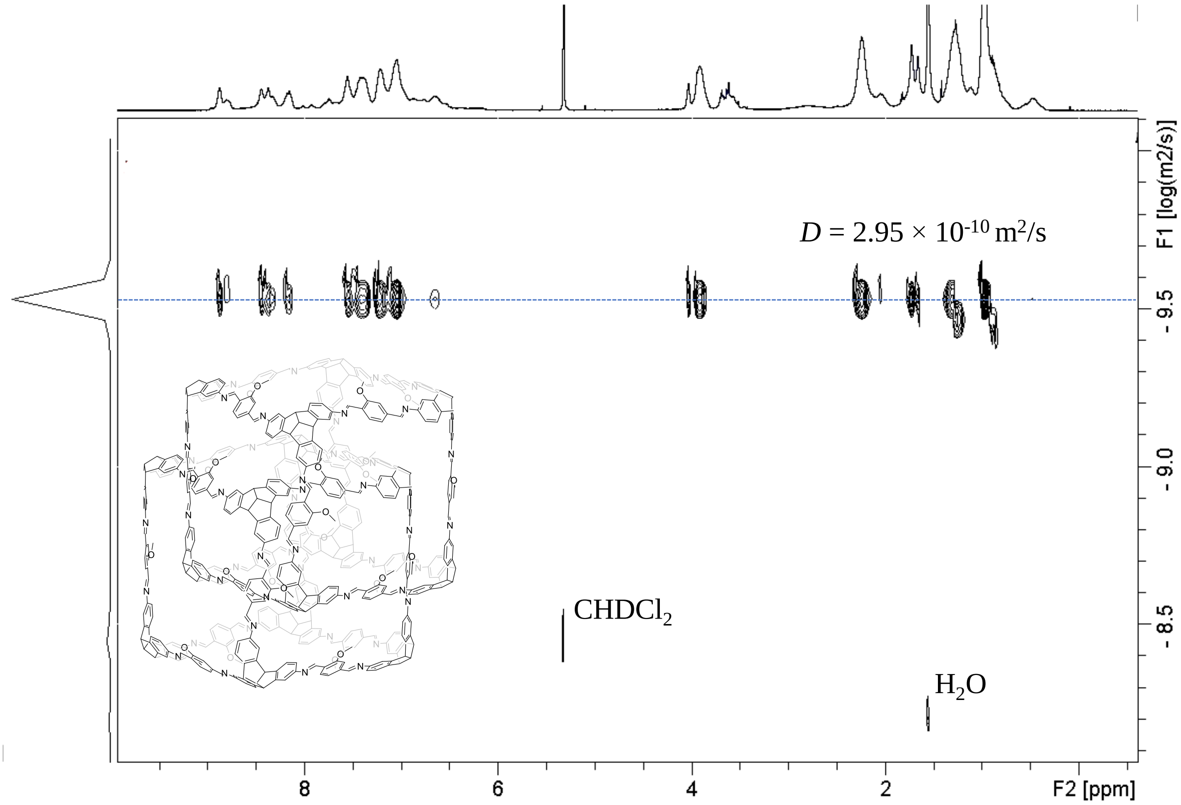

Figure 323. DOSY NMR (400 MHz, 295 K, CD_2Cl_2) spectrum of (**H/OMe-cube)$_2$**

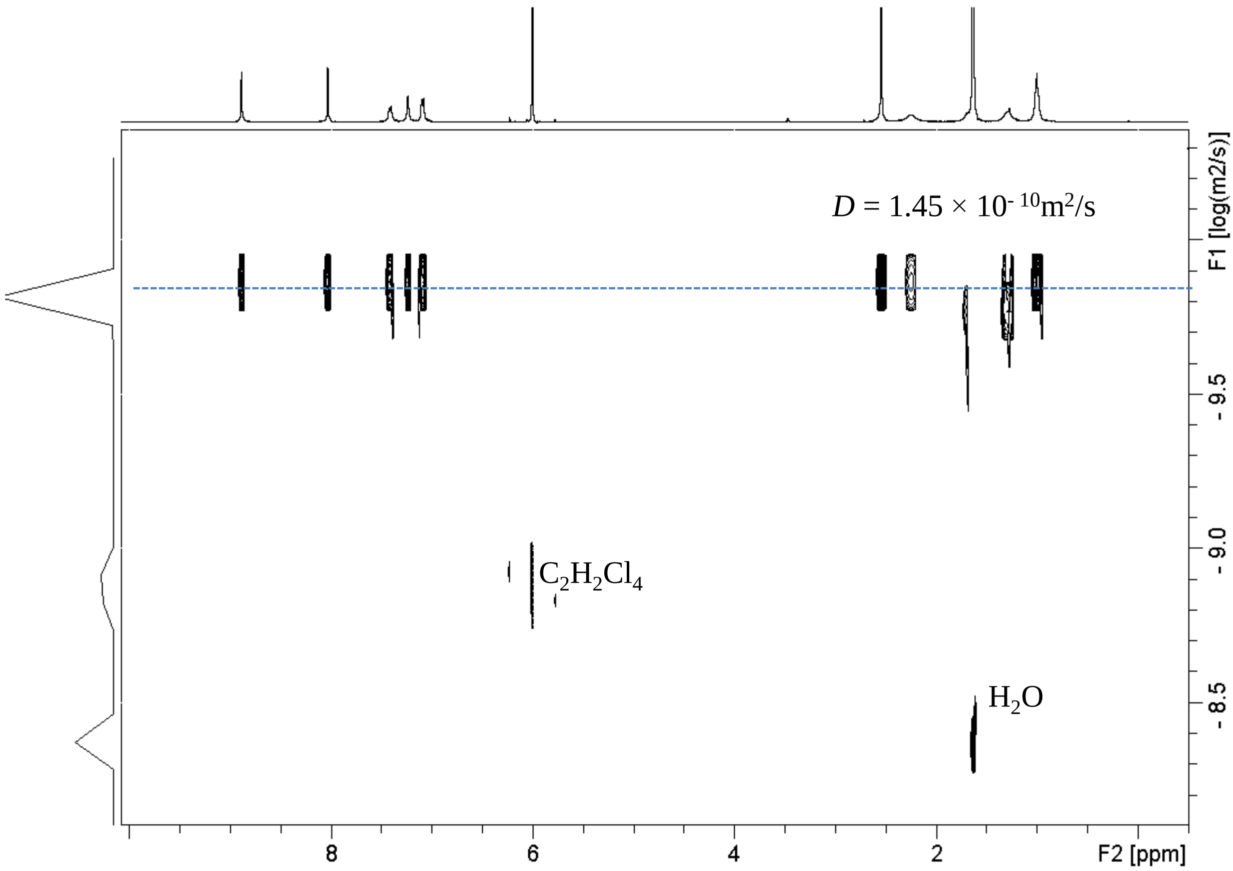

Figure 324. DOSY NMR (400 MHz, 295 K, C2D2Cl4) spectrum of **SMe-cube**.

5. Mass spectra

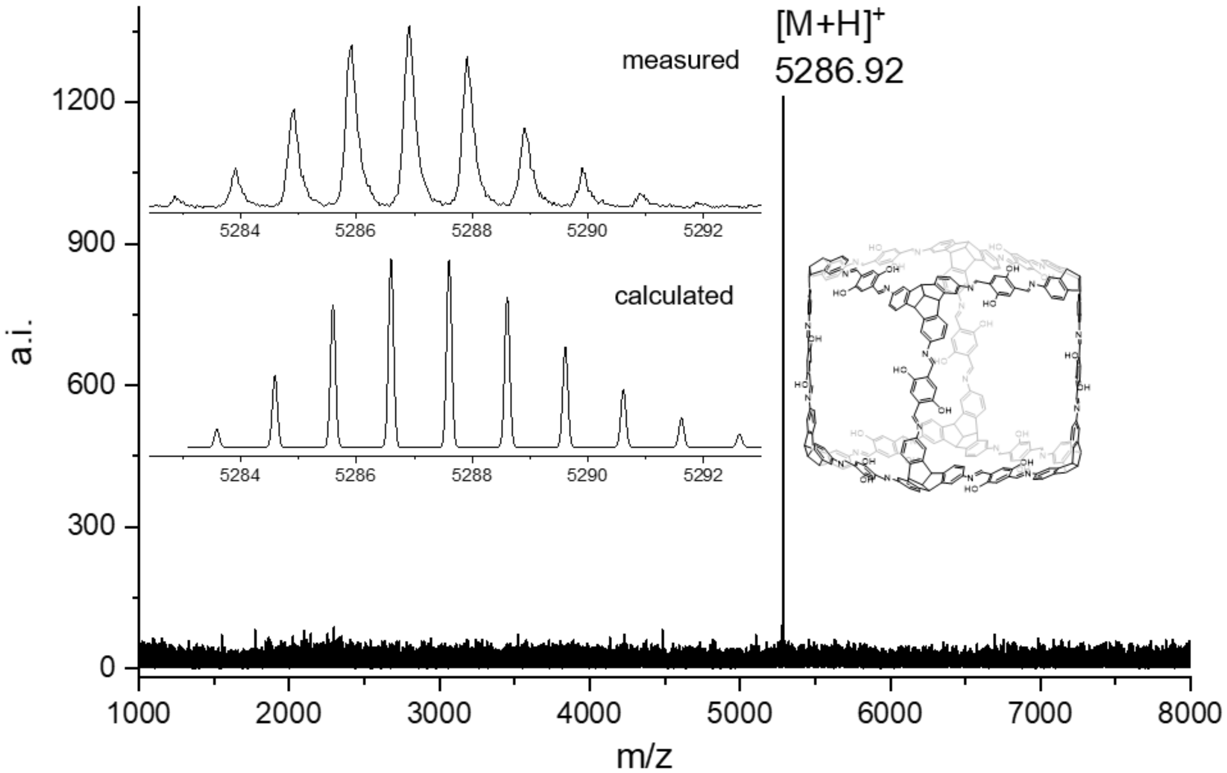

Figure 325. MALDI-TOF mass spectrum of **OH-cub**e.

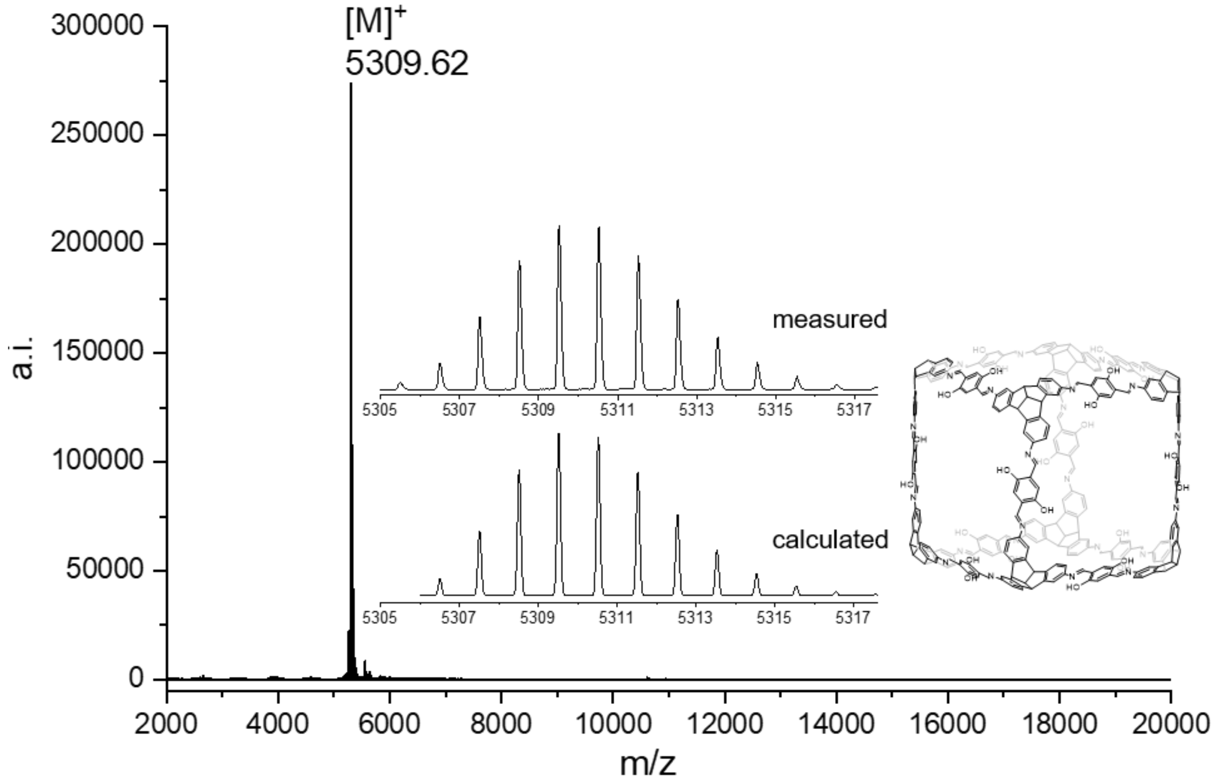

Figure 326. TIMS-TOF mass spectrum of ^{15}N labelled ***OH-cub**e.

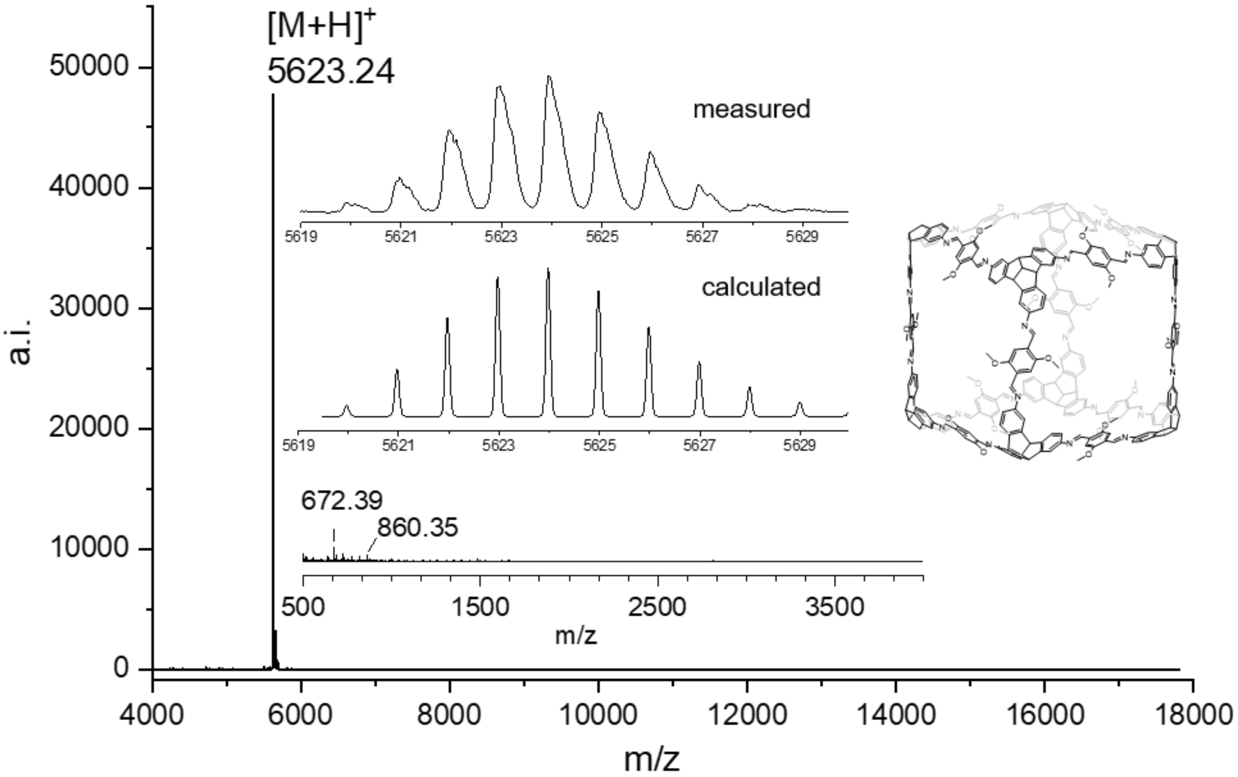

Figure 327. MALDI-TOF mass spectrum of **OMe-cube**.

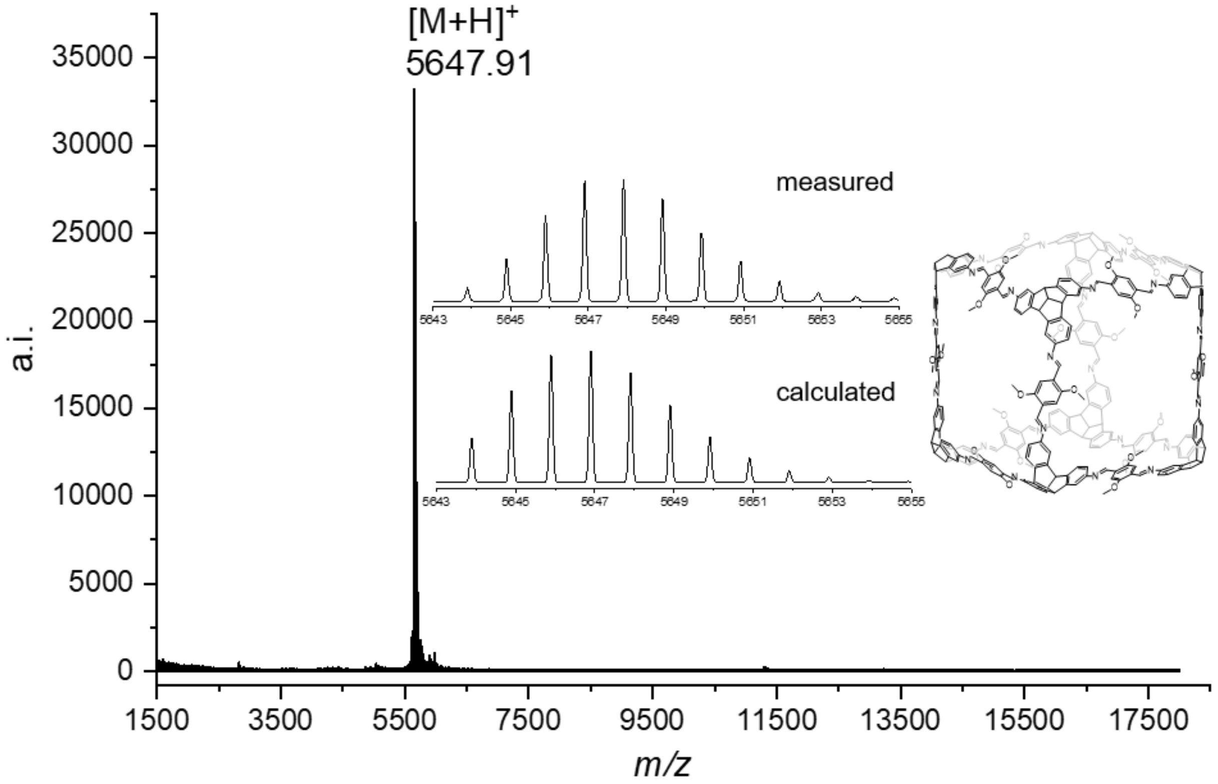

Figure 328. MALDI-TOF mass spectrum of ^{15}**N labelled** ***OMe-cube**.

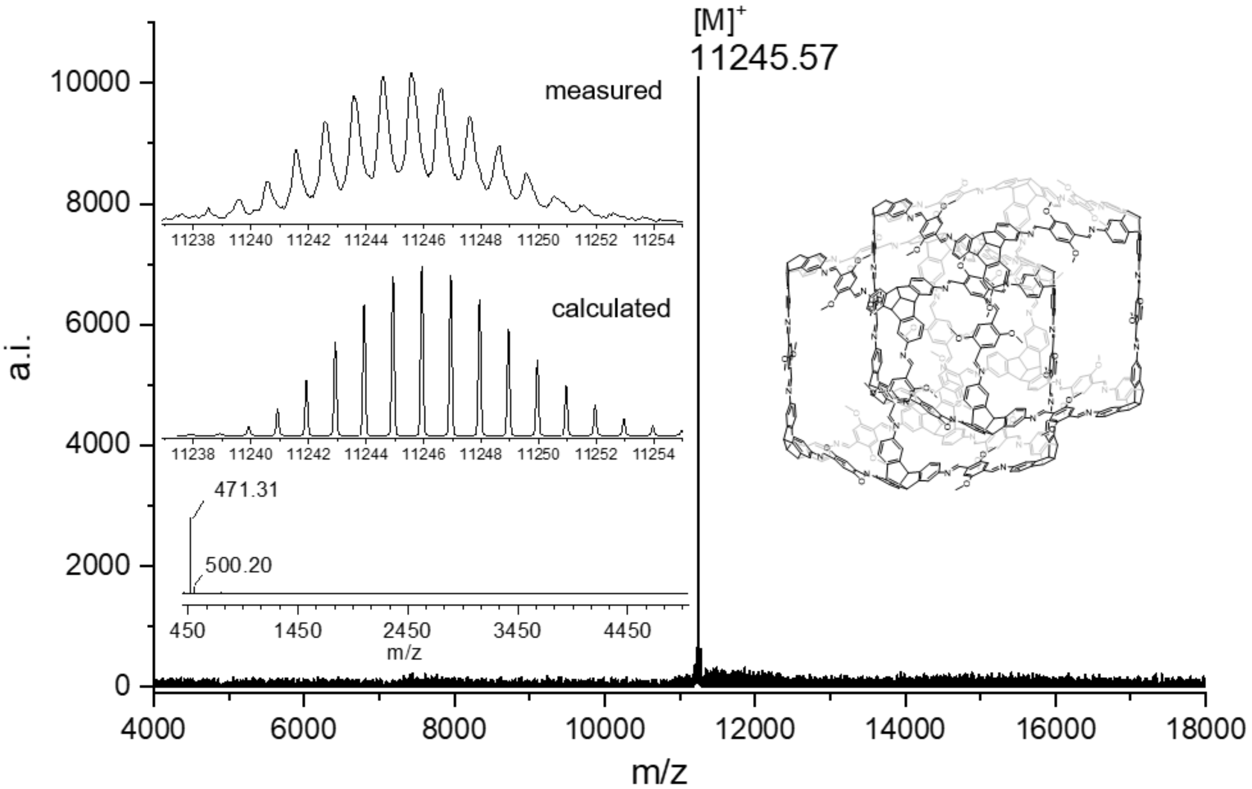

Figure 329. MALDI-TOF mass spectrum of **(OMe-cube)$_2$**.

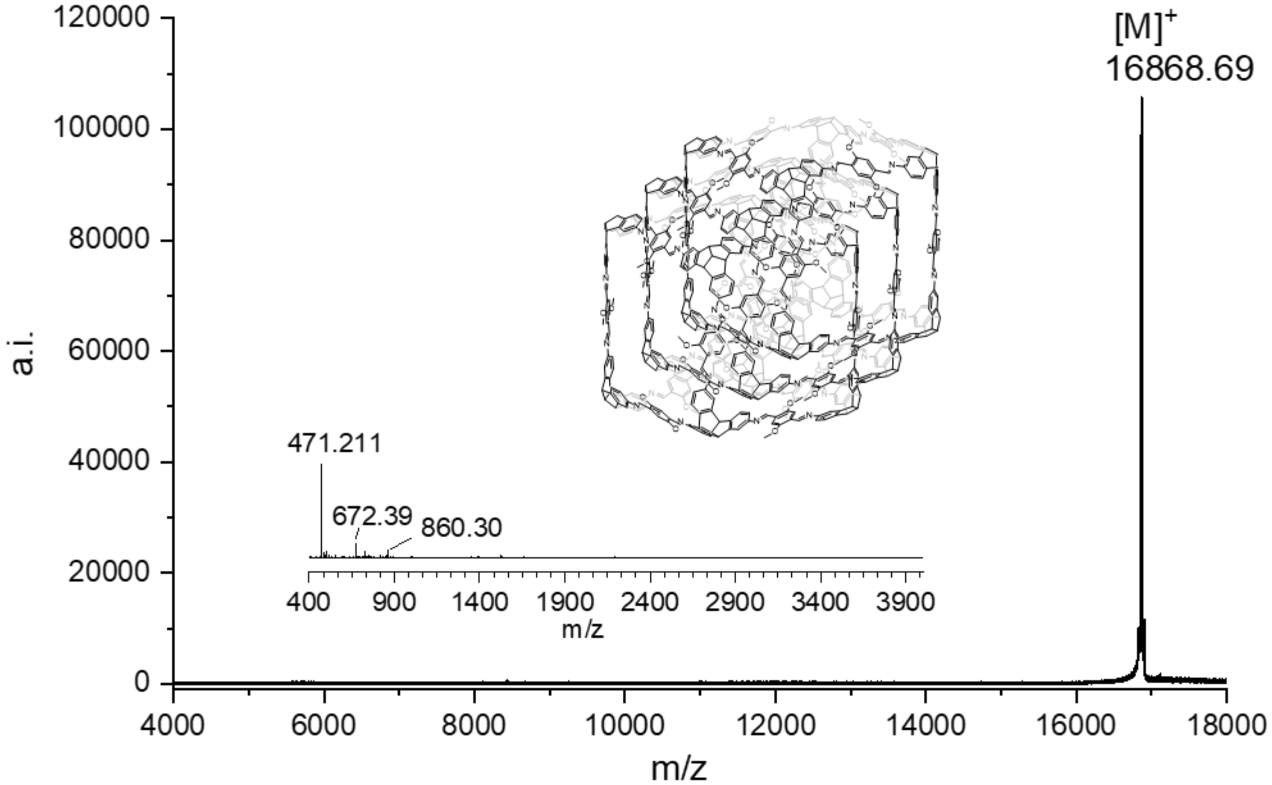

Figure 330. MALDI-TOF (DCTB matrix) mass spectrum of **(OMe-cube)$_3$**. Inset: low *m/z* region.

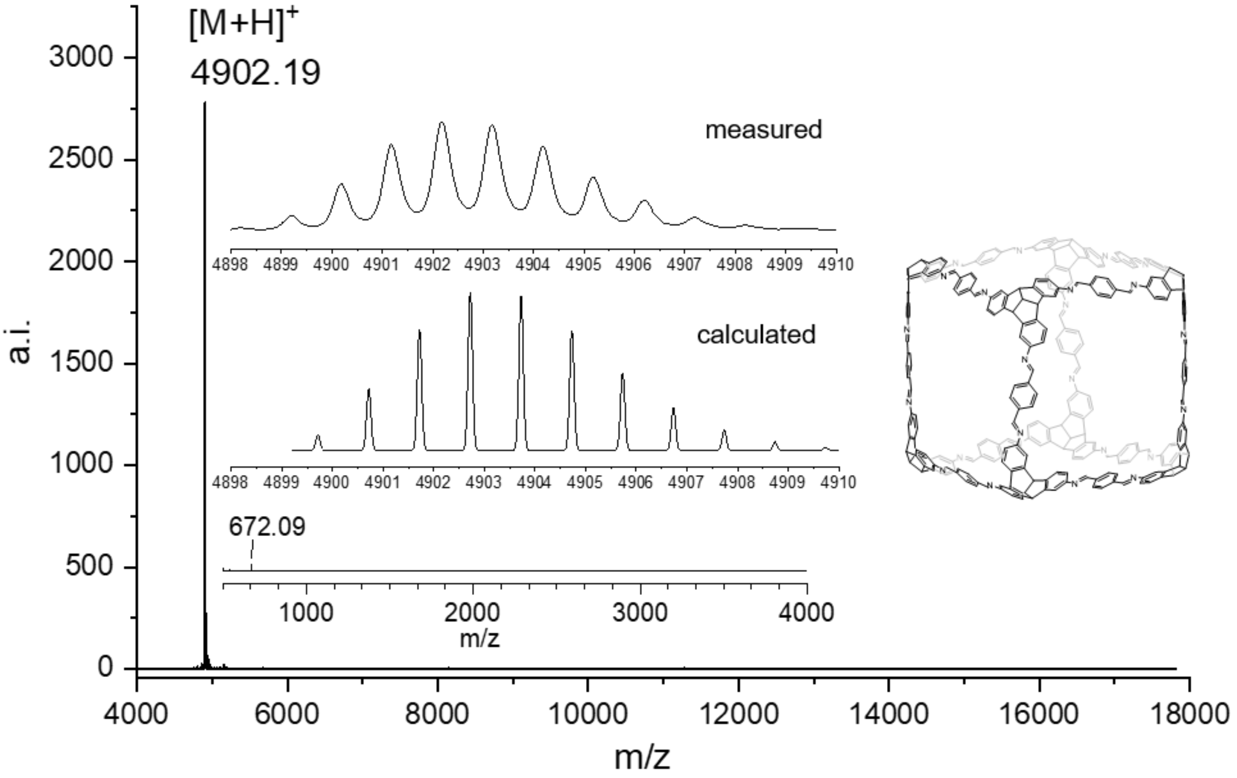

Figure 331. MALDI-TOF mass spectrum of **H-cube**.

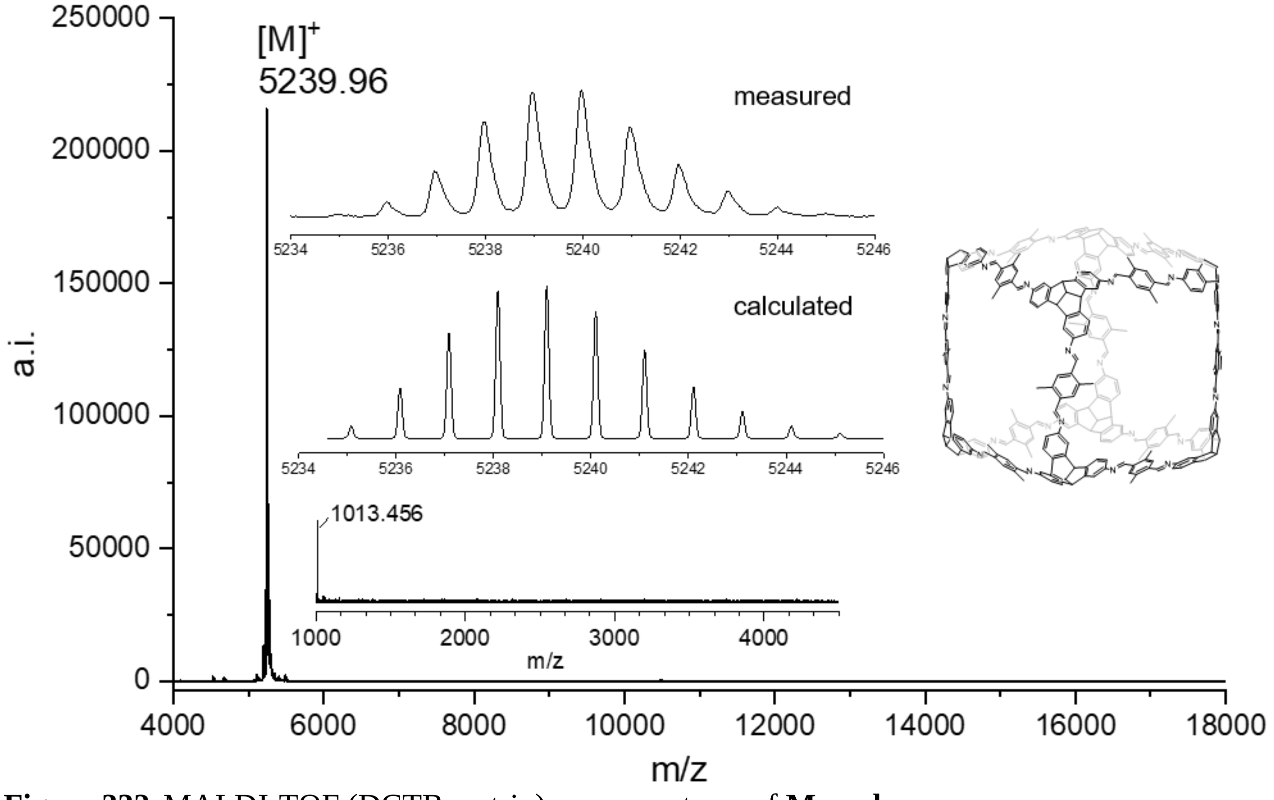

Figure 332. MALDI-TOF (DCTB matrix) mass spectrum of **Me-cube**.

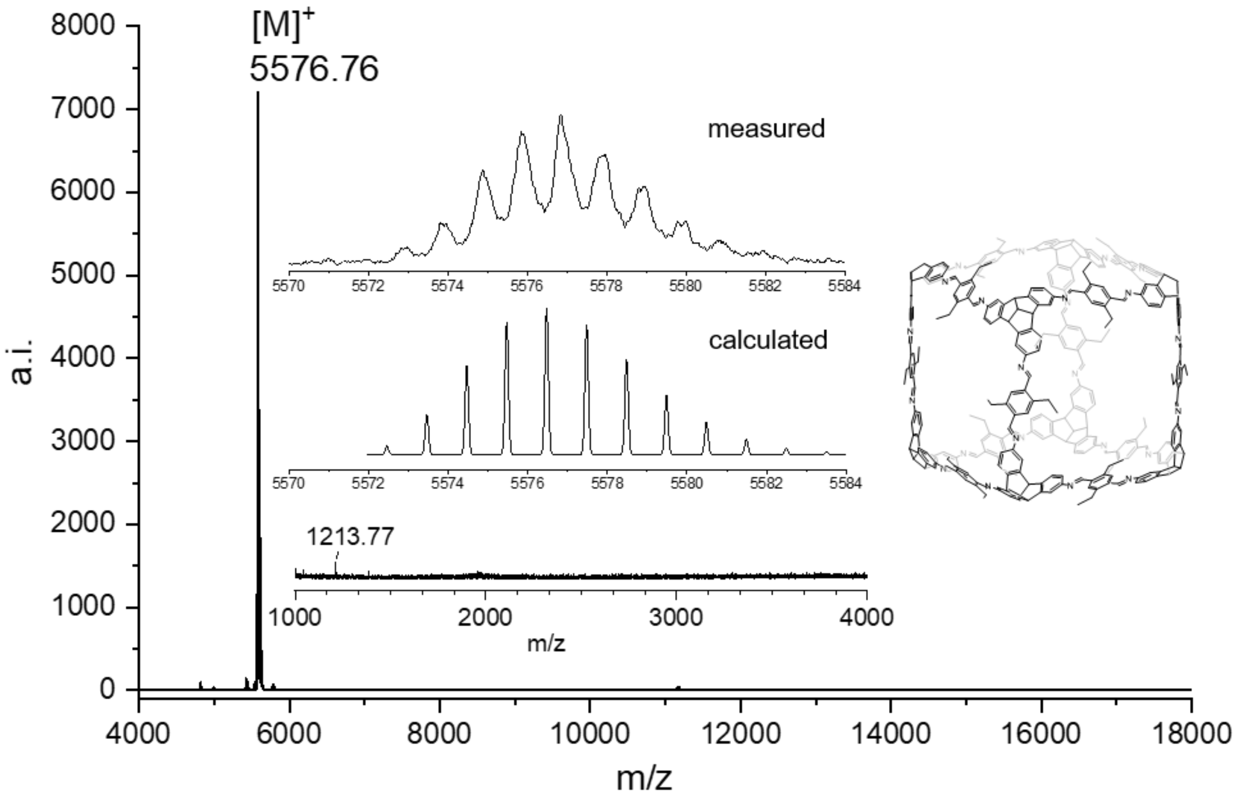

Figure 333. MALDI-TOF mass spectrum of **Et-cube**.

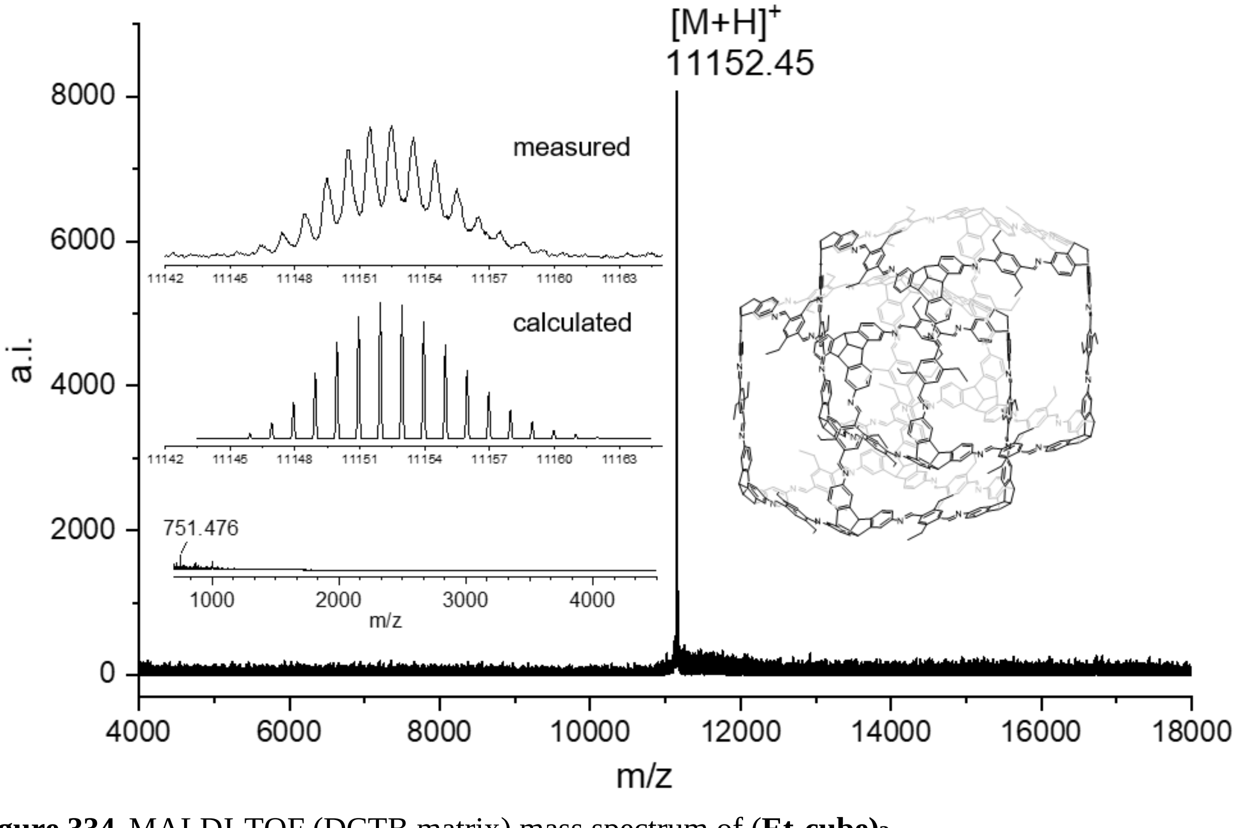

Figure 334. MALDI-TOF (DCTB matrix) mass spectrum of (**Et-cube)2**.

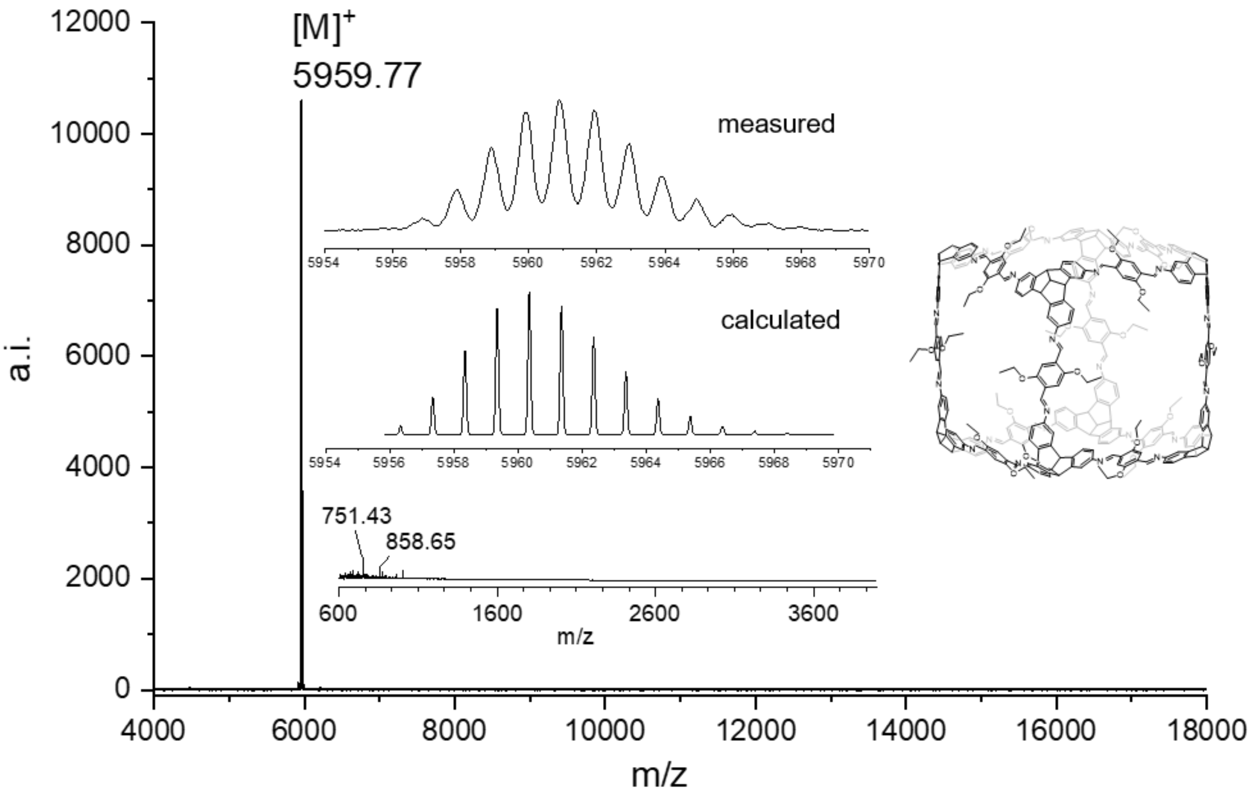

Figure 335. MALDI-TOF (DCTB matrix) mass spectrum of **OEt-cube**.

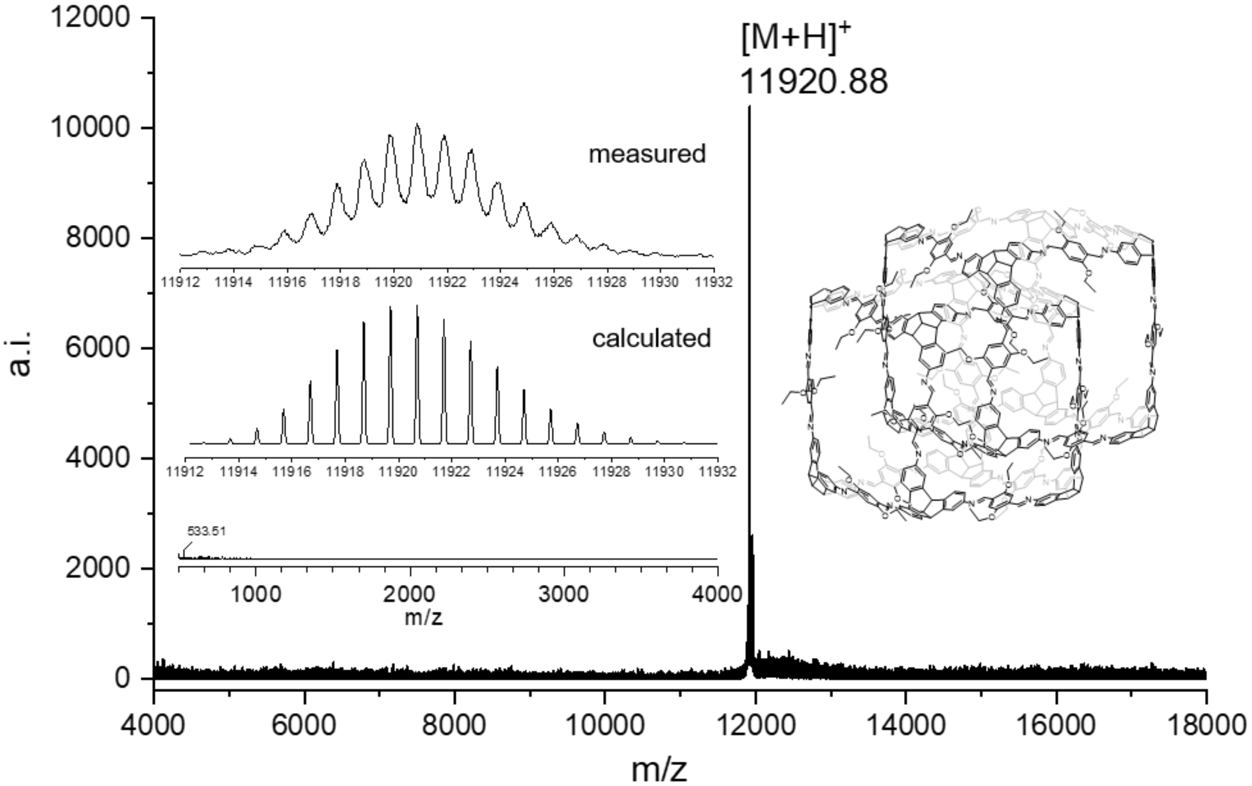

Figure 336. MALDI-TOF (DCTB matrix) mass spectrum of **(OEt-cube)$_2$**

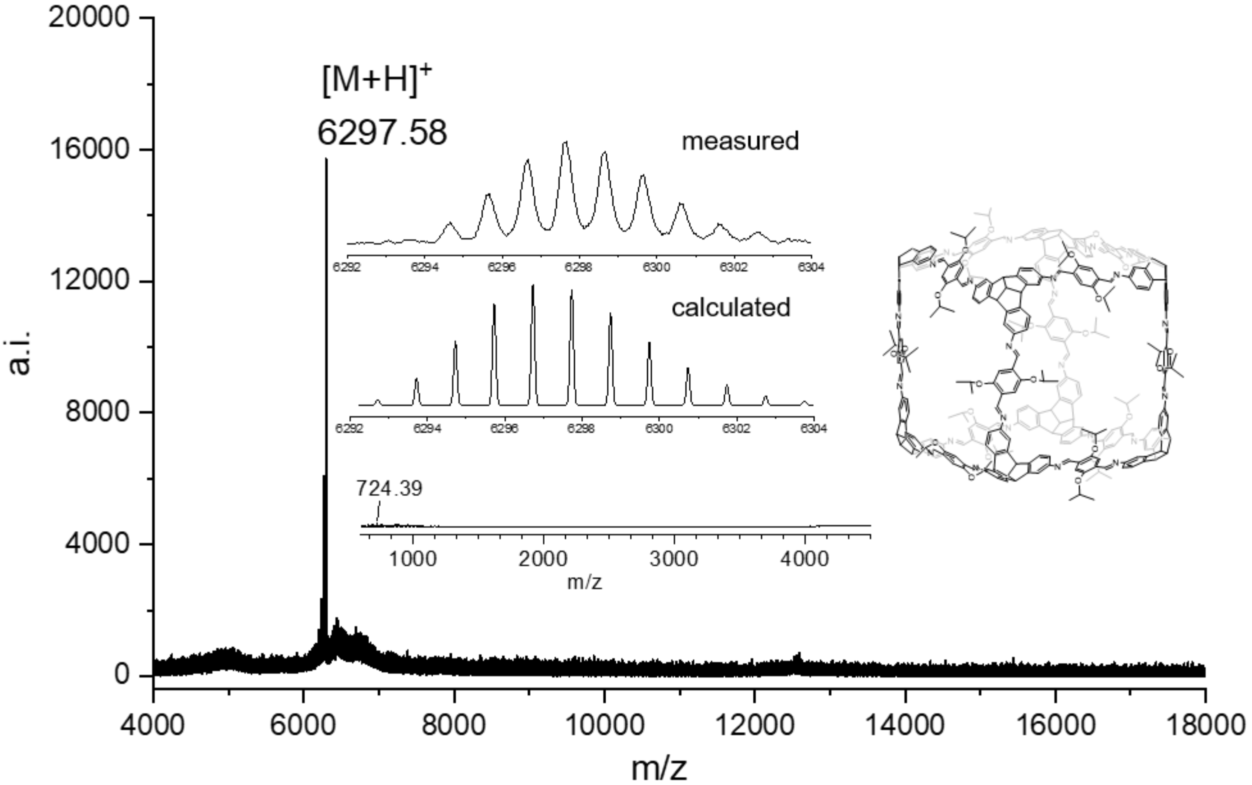

Figure 337. MALDI-TOF (DCTB matrix) mass spectrum of **O^{i}Pr-cube**.

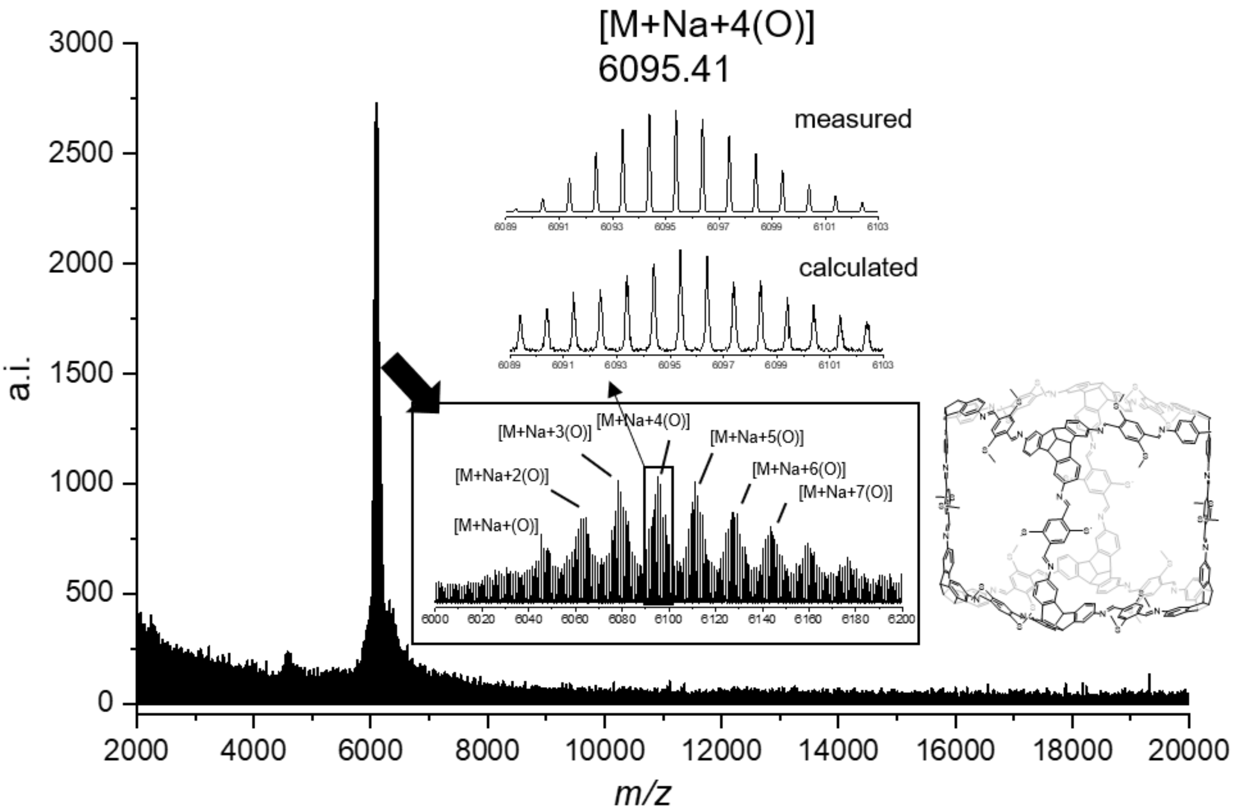

Figure 338. TIMS-TOF (DCTB) mass spectrum of **SMe-cube**.

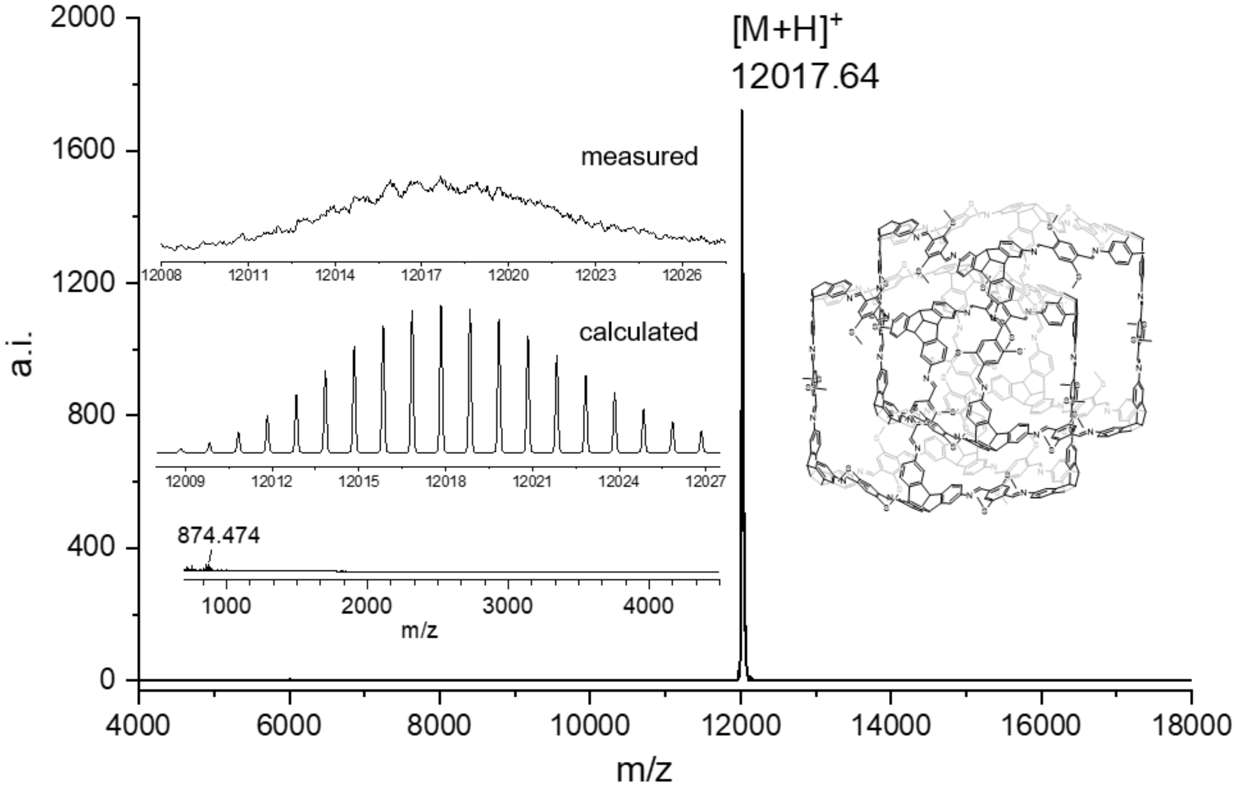

Figure 339. MALDI-TOF (DCTB matrix) mass spectrum of **(SMe-cube)2**.

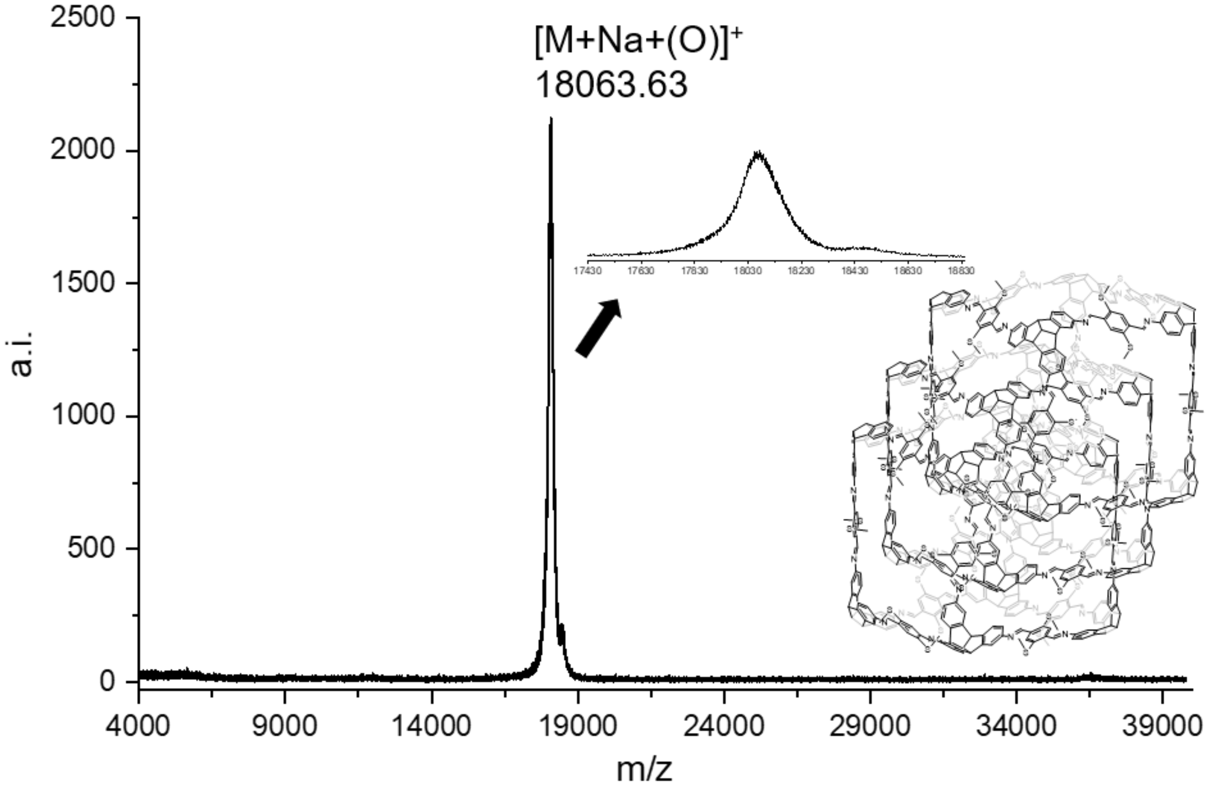

Figure 340. MALDI-TOF (DCTB) mass spectrum of **(SMe-cube)3**.

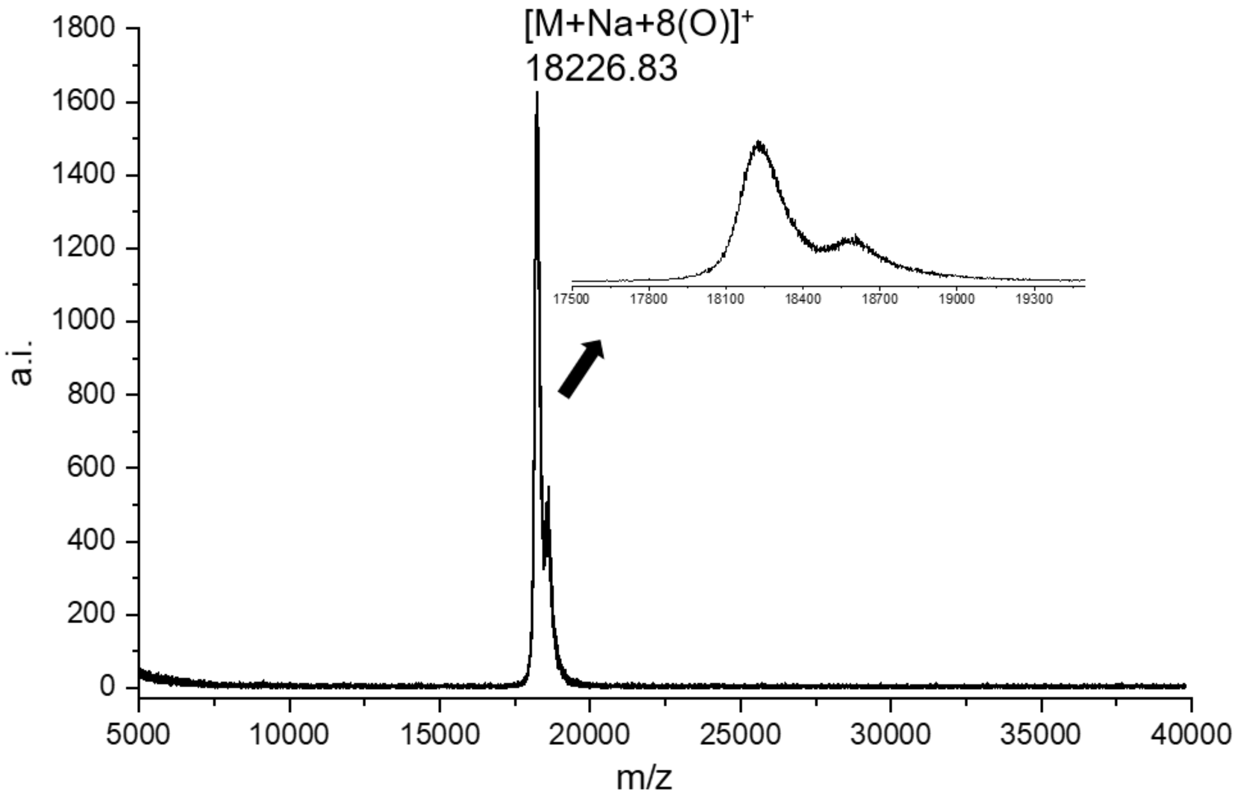

Figure 341. MALDI-TOF (DCTB) mass spectrum of ^{15}**N labelled *(SMe-cube)$_3$**.

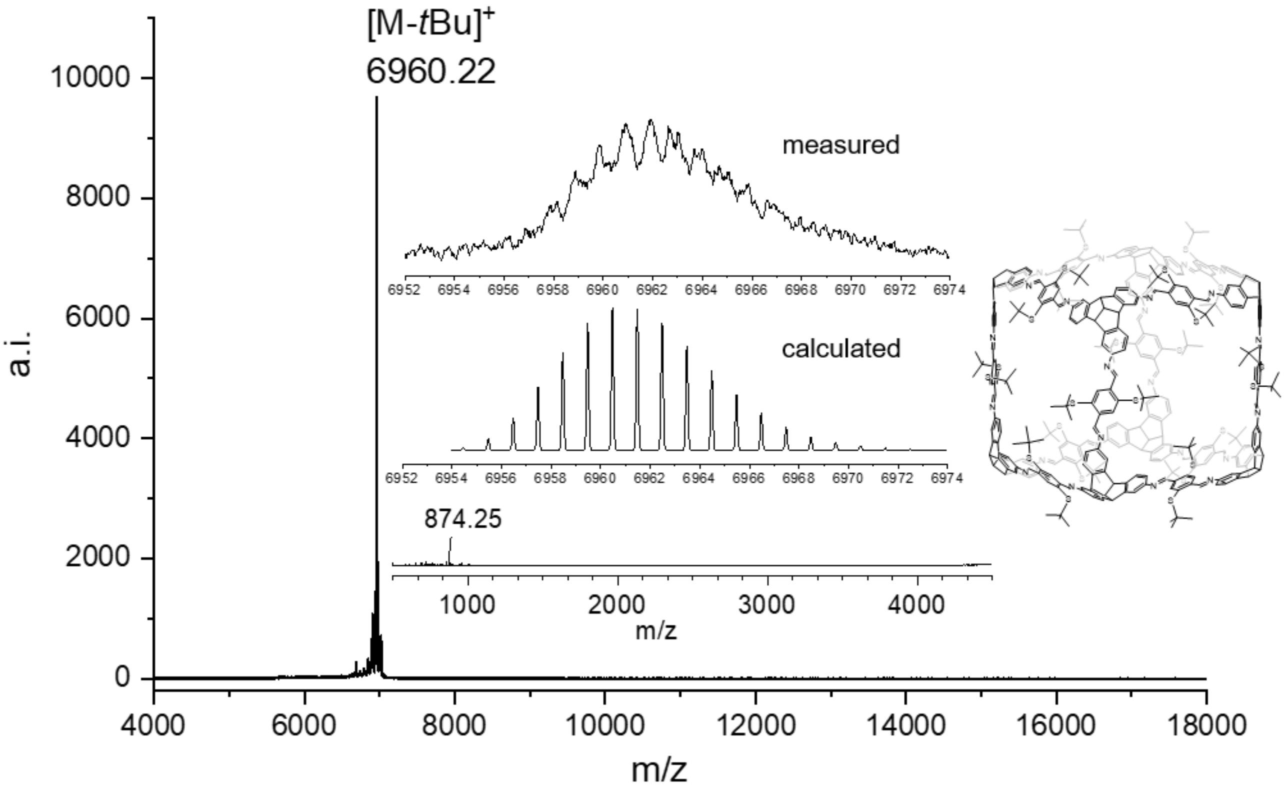

Figure 342. MALDI-TOF (DCTB matrix) mass spectrum of **SC(CH$_3$)$_3$-cube**.

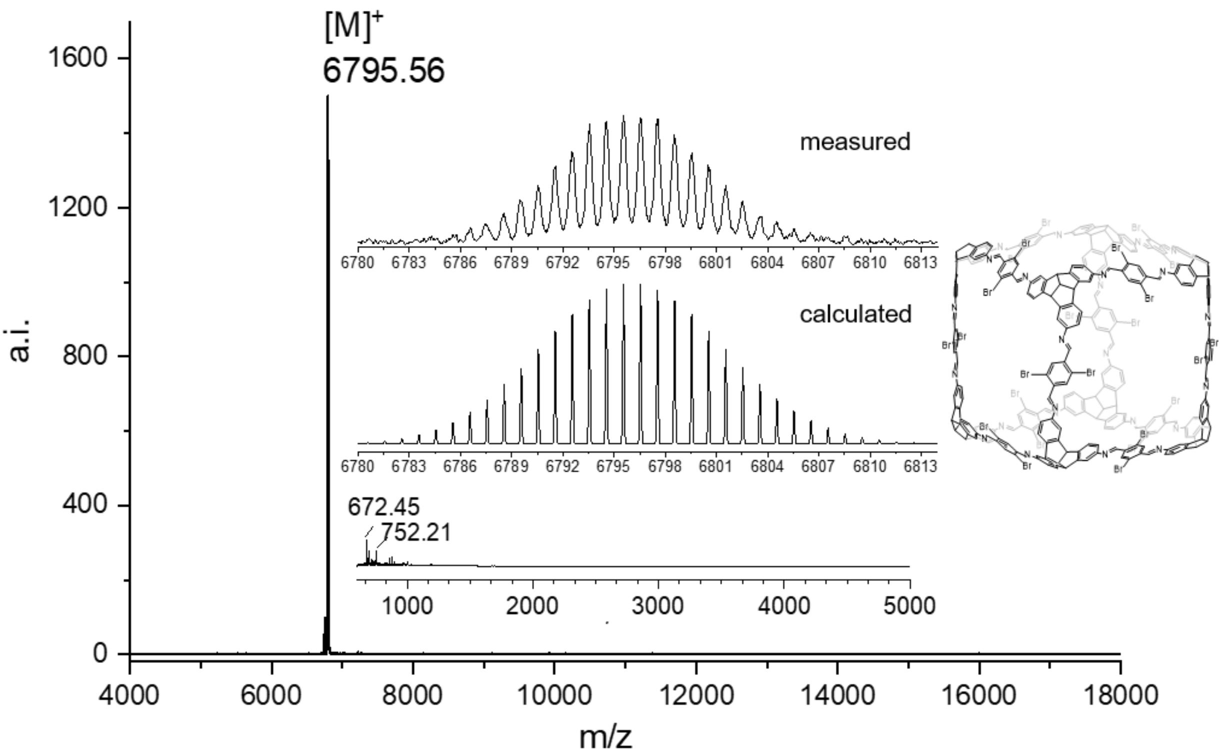

Figure 343. MALDI-TOF mass spectrum of **Br-cube**.

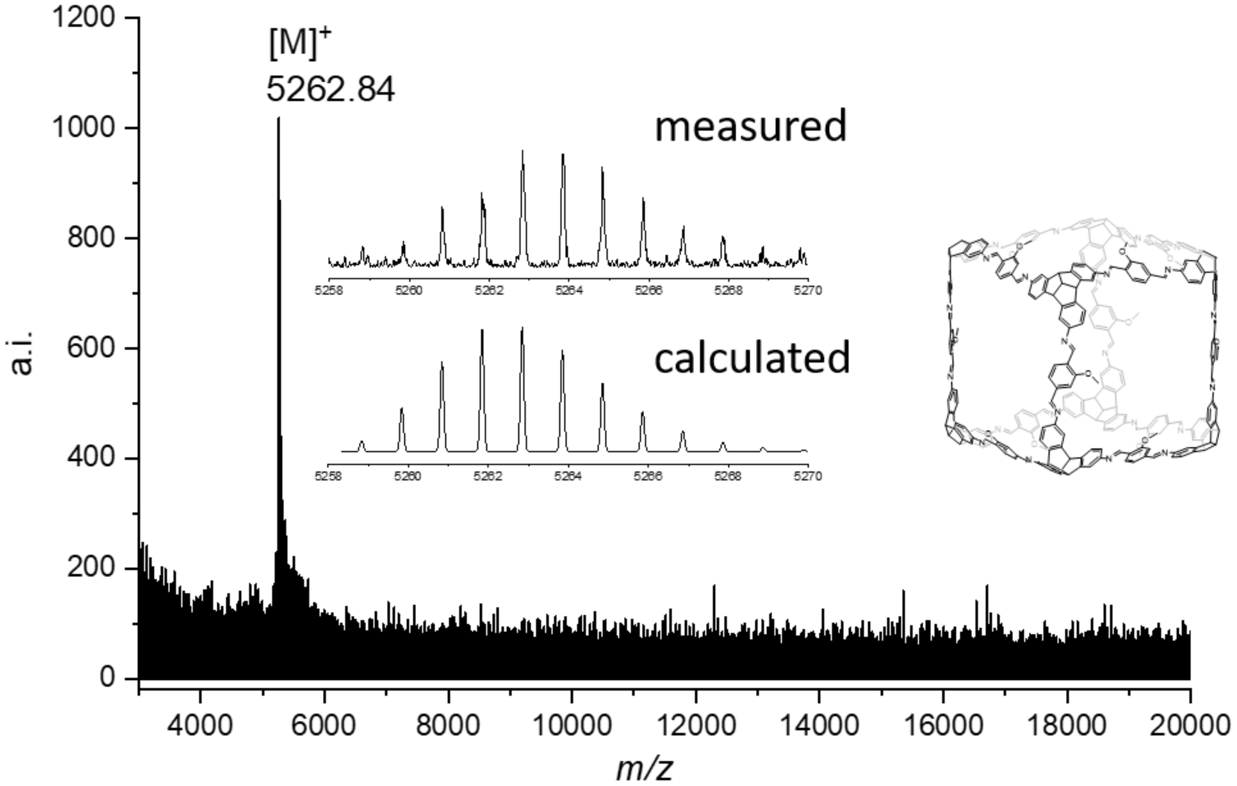

Figure 344. TIMS-TOF (DCTB) mass spectrum of **H/OMe-cube**.

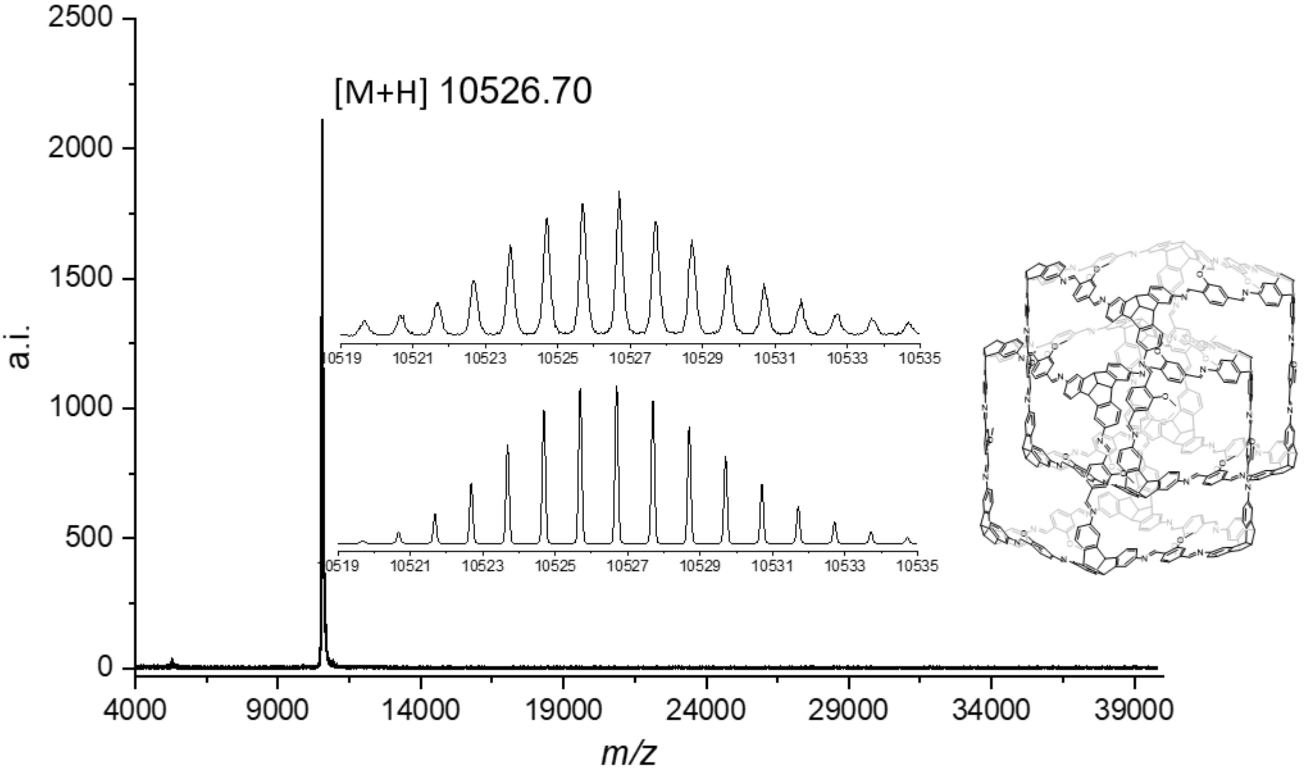

Figure 345. TIMS-TOF (DCTB) mass spectrum of **(H/OMe-cube)$_2$**.

6. IR Spectra

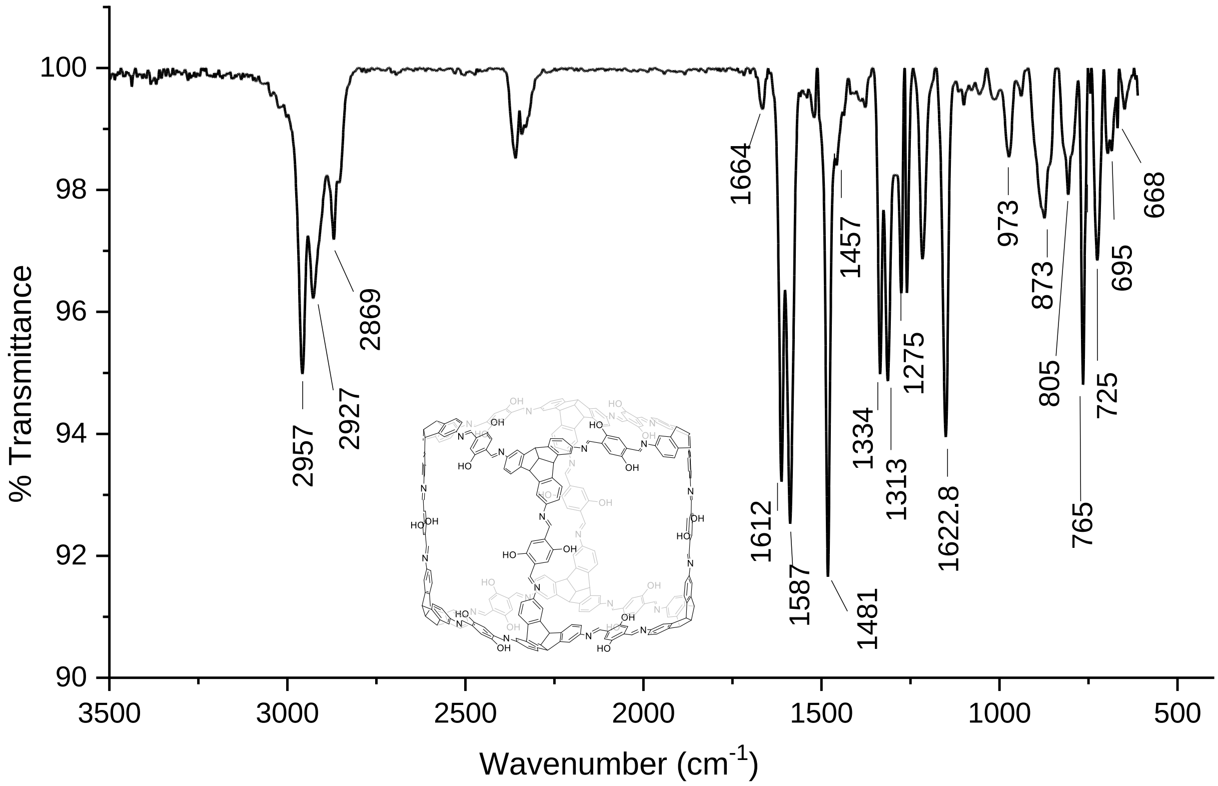

Figure 346. FT-IR (ATR) spectrum of **OH-cube**.

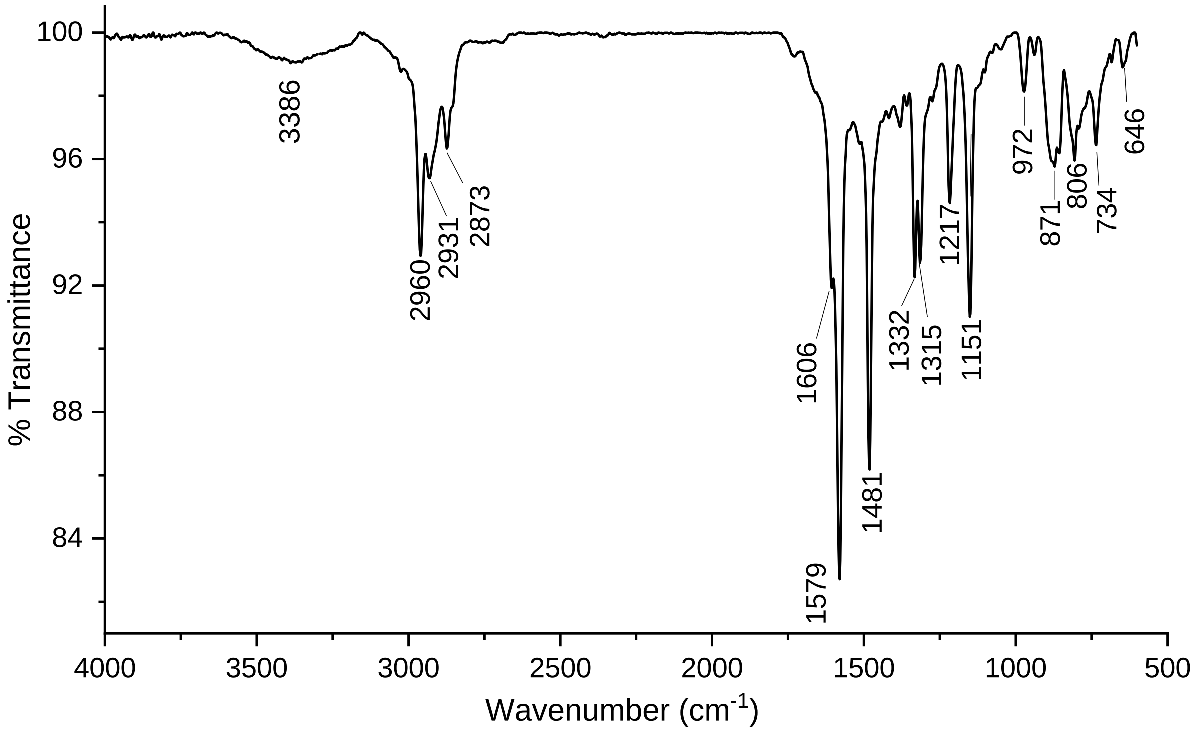

Figure 347. FT-IR (ATR) spectrum of 15N labelled ***OH-cube**.

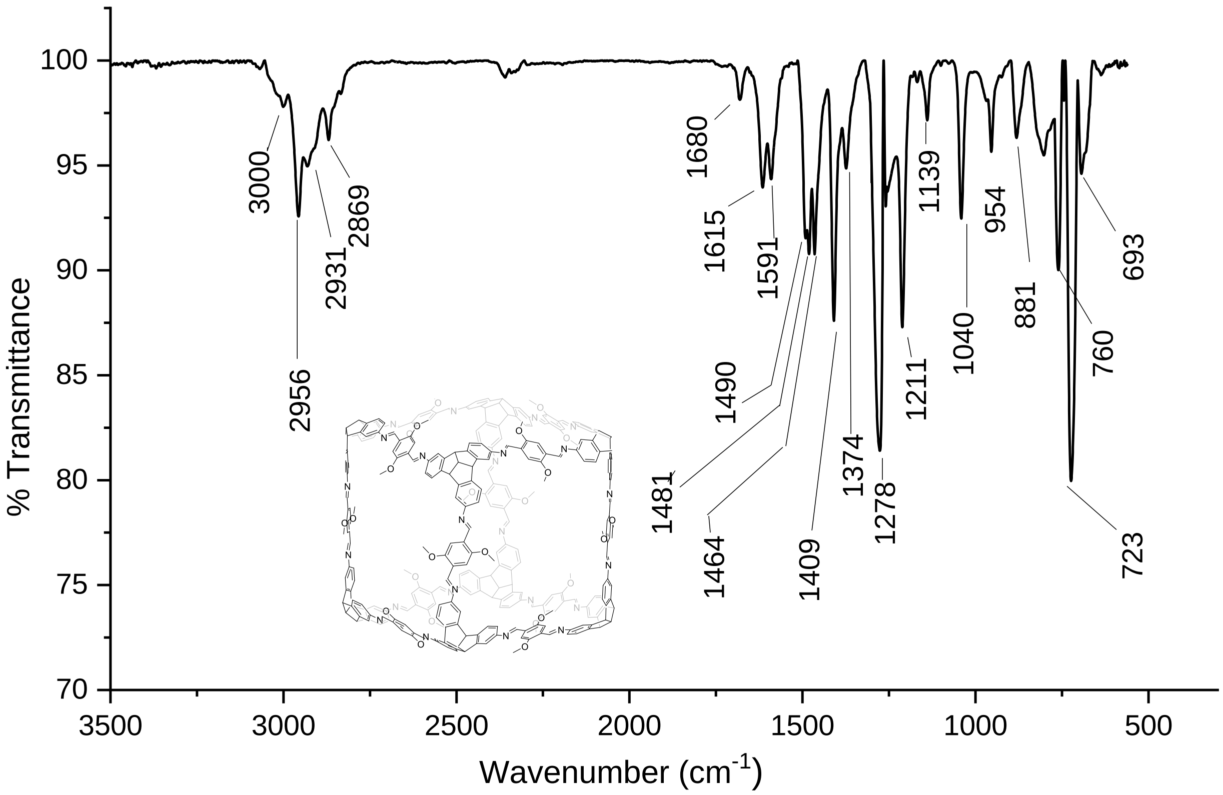

Figure 348. FT-IR (ATR) spectrum of **OMe-cube**.

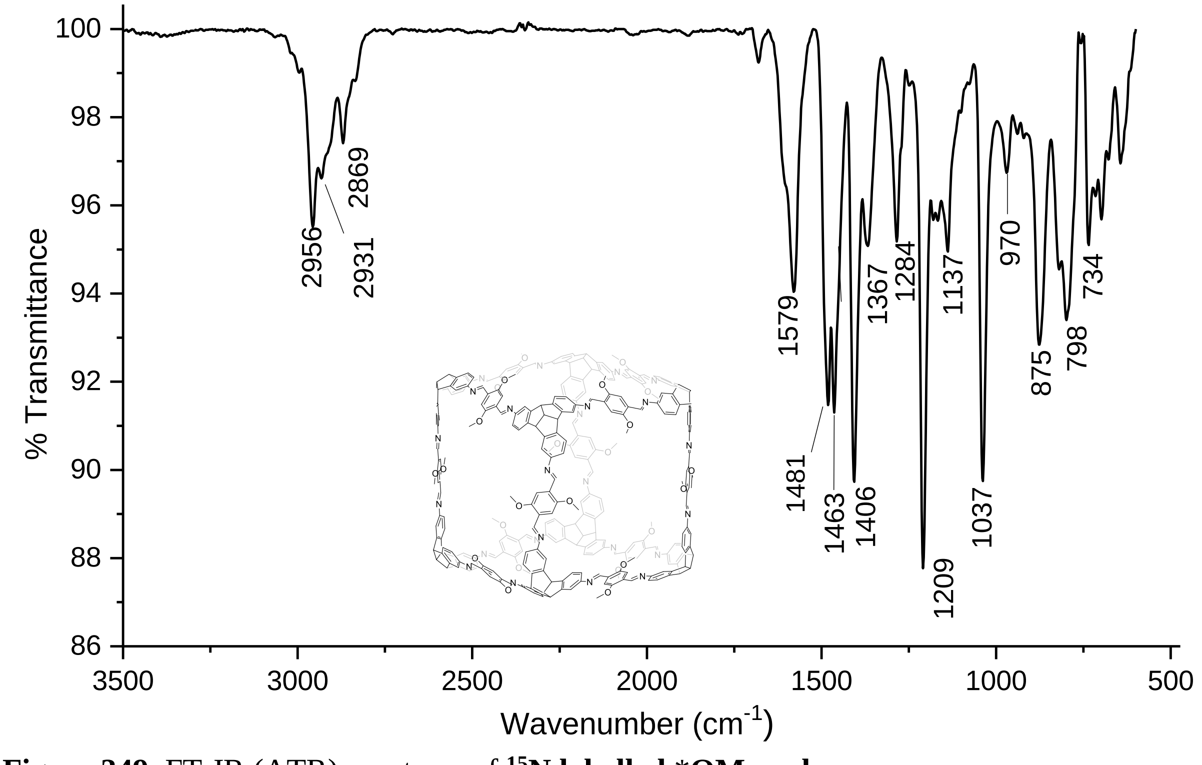

Figure 349. FT-IR (ATR) spectrum of **^{15}N labelled** *****OMe-cube**.

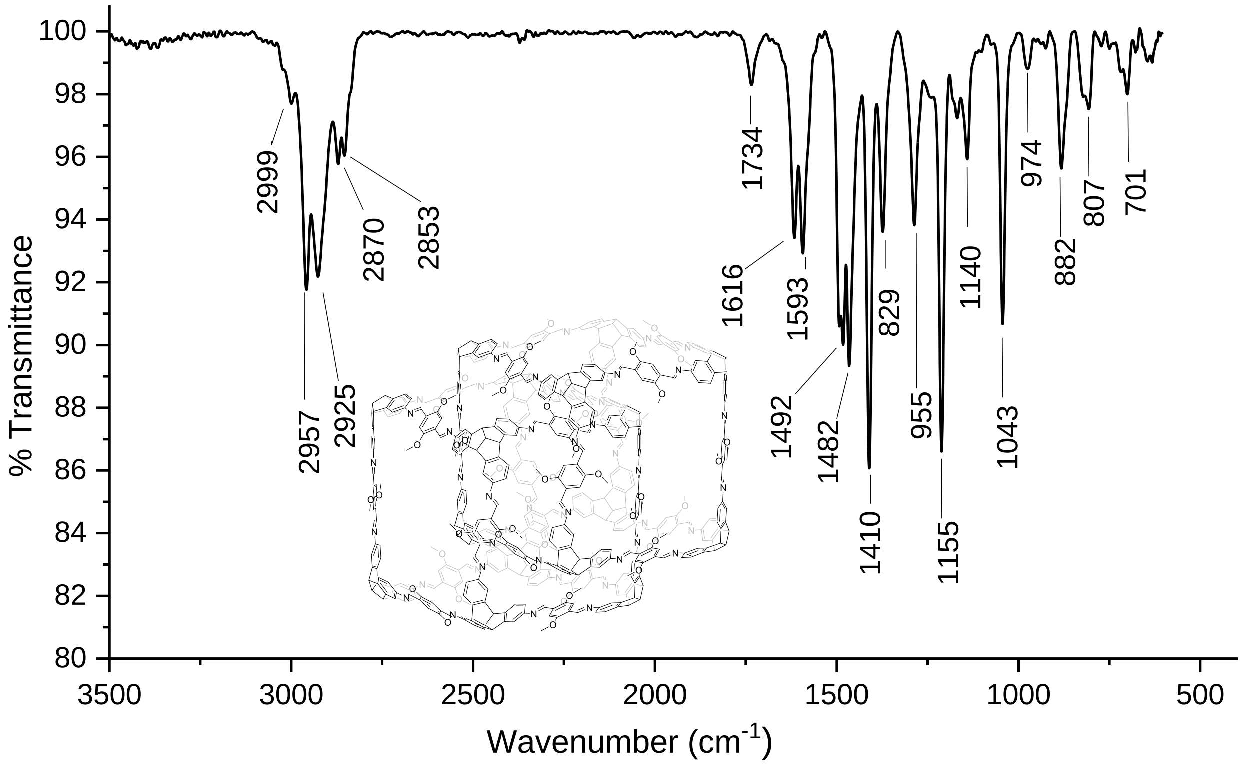

Figure 350. FT-IR (ATR) spectrum of **(OMe-cube)$_2$**.

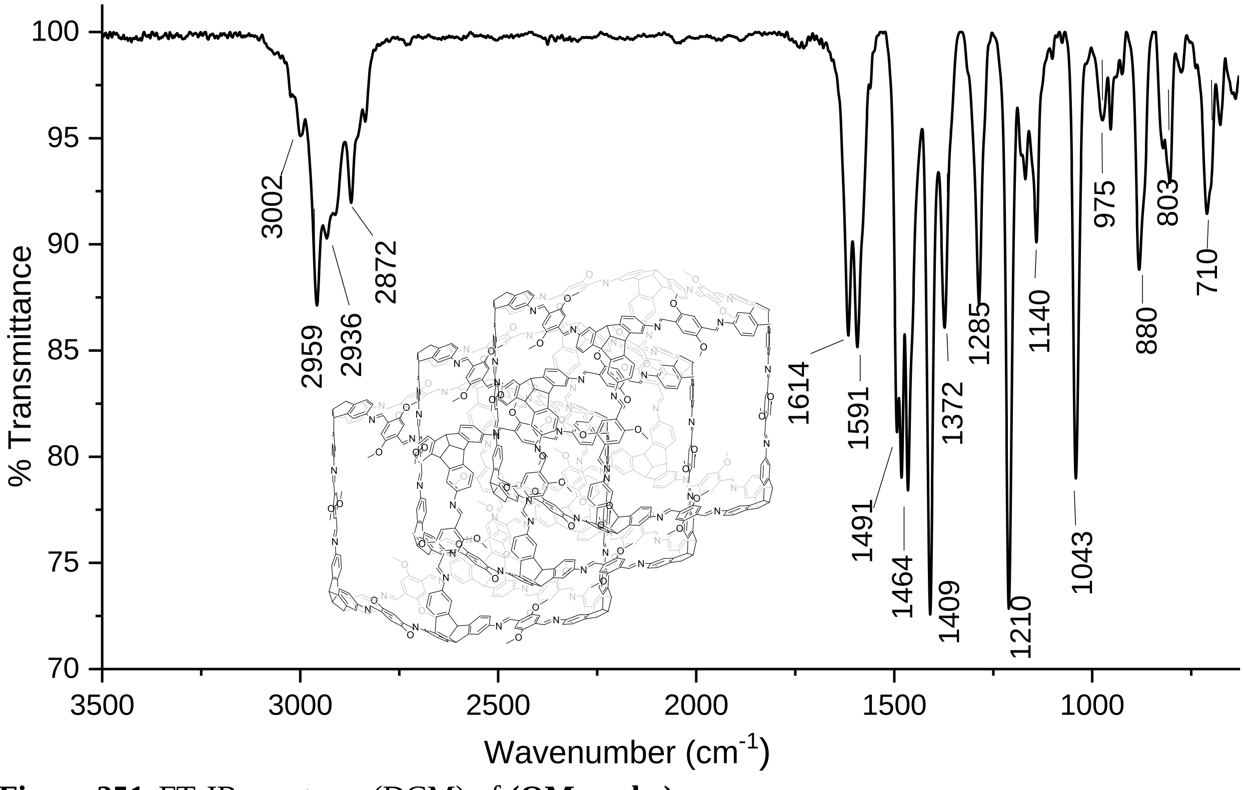

Figure 351. FT-IR spectrum (DCM) of **(OMe-cube)$_3$**.

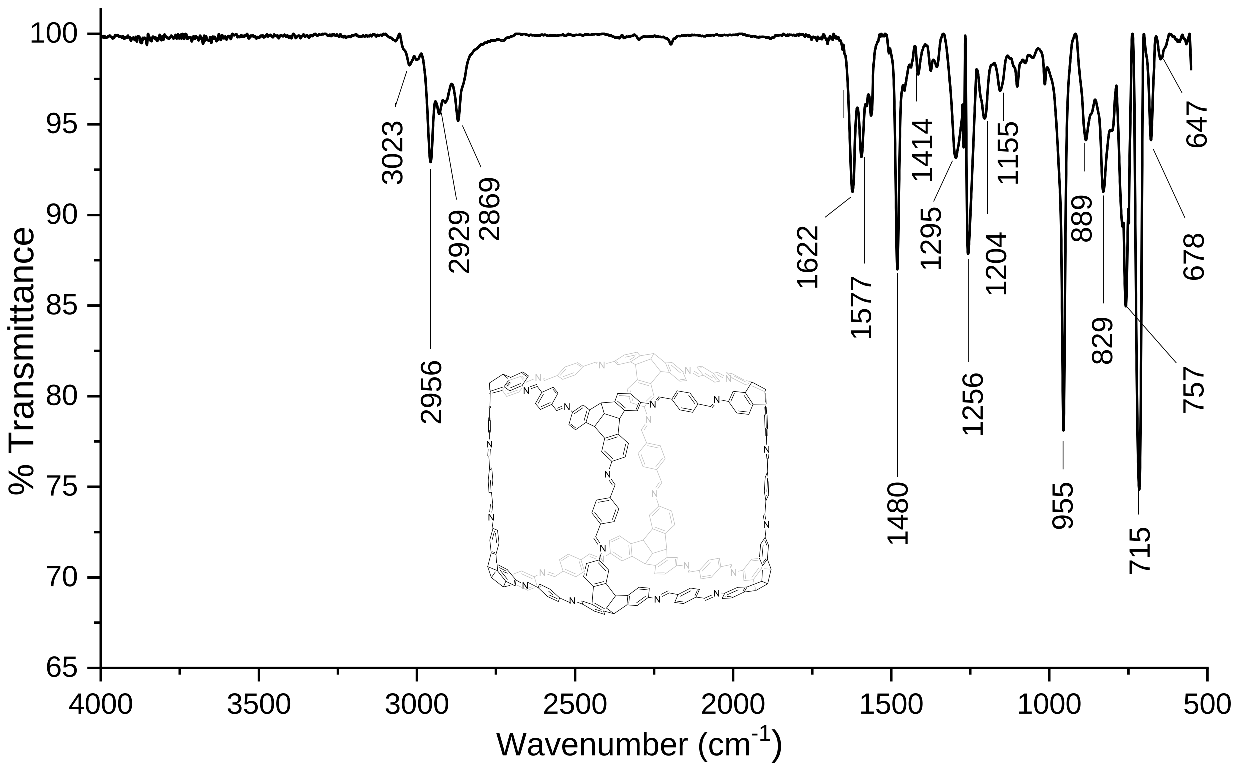

Figure 352. FT-IR (ATR) spectrum of **H-cube**.

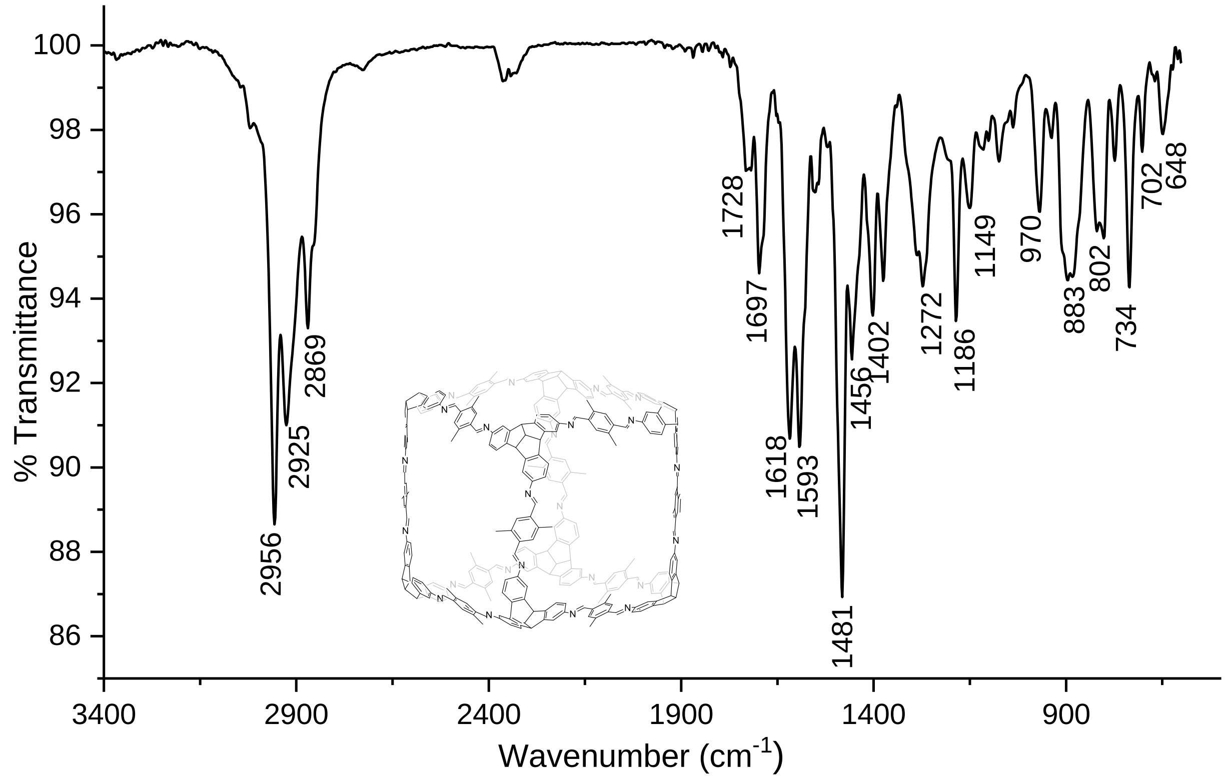

Figure 353. FT-IR (ATR) spectrum of **Me-cube**.

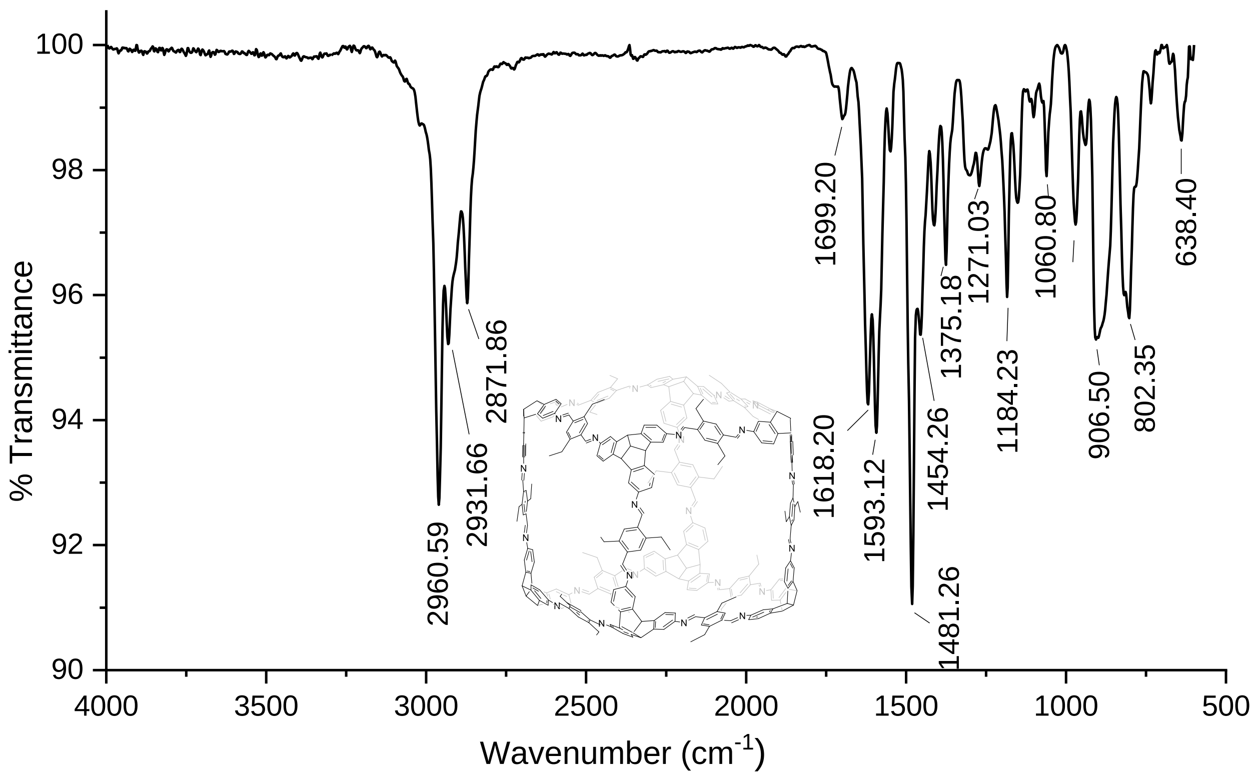

Figure 354. FT-IR (ATR) spectrum of **Et-cube**.

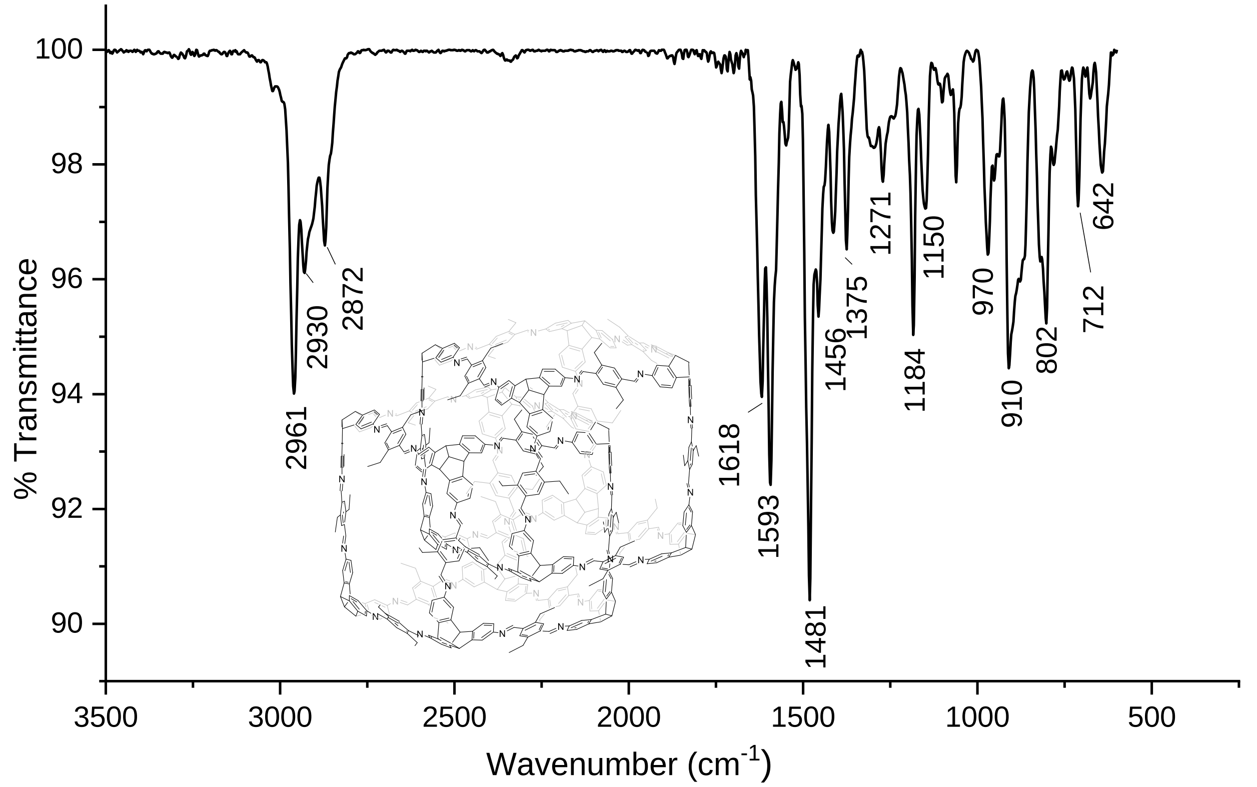

Figure 355. FT-IR spectrum (DCM) of **(Et-cube)$_2$**.

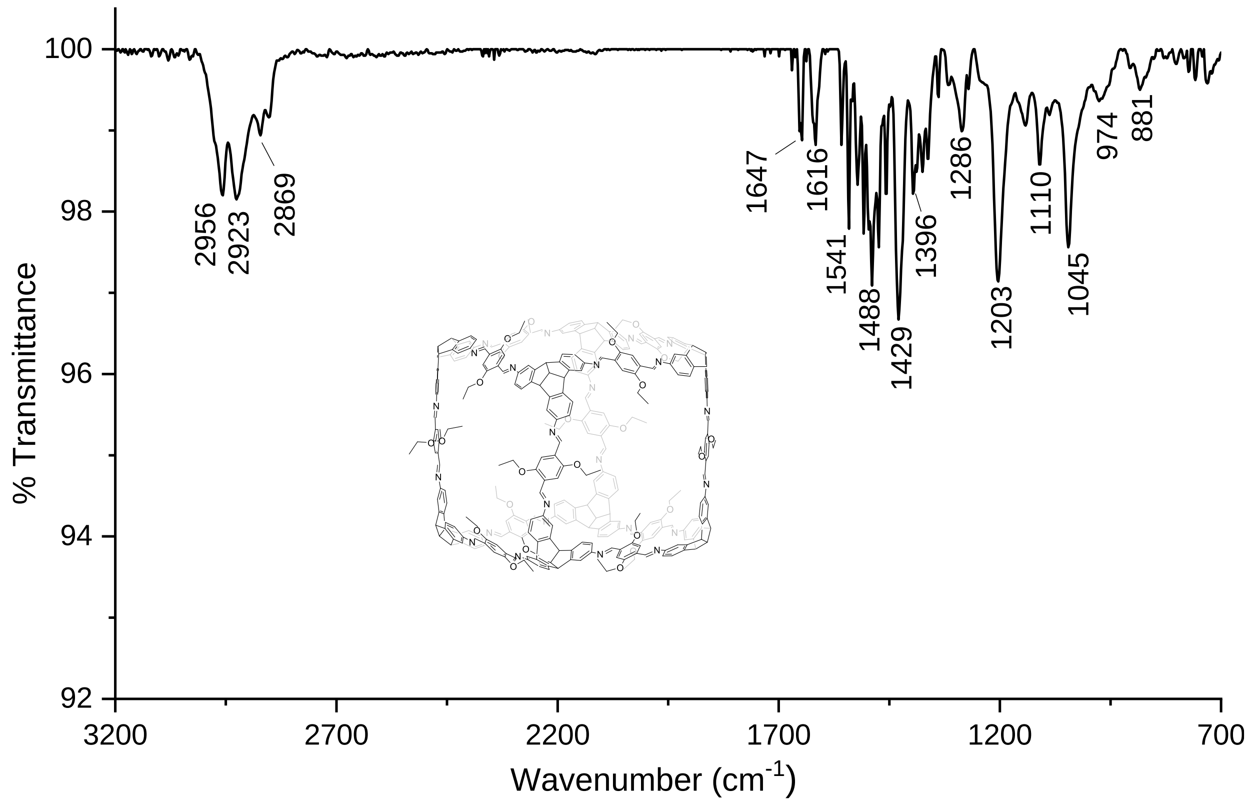

Figure 356. FT-IR (ATR) spectrum of **OEt-cube**.

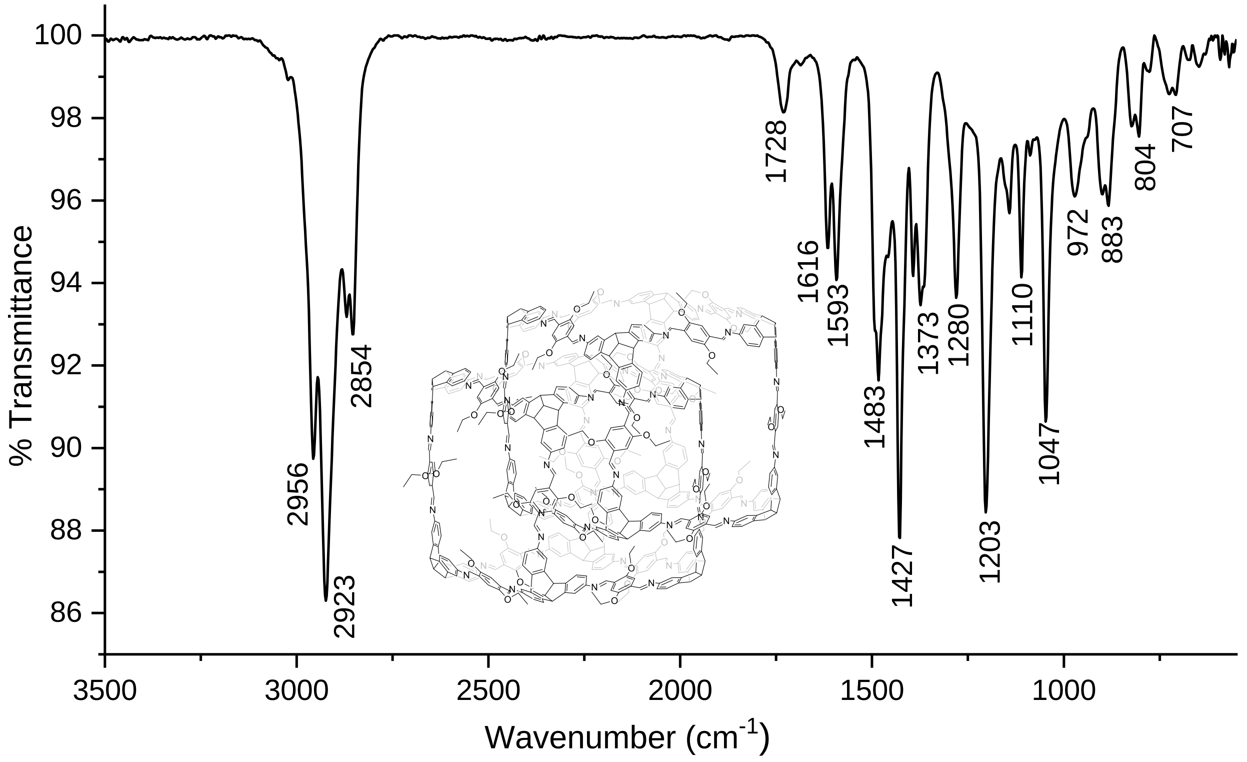

Figure 357. FT-IR (ATR) spectrum of **(OEt-cube)$_2$**.

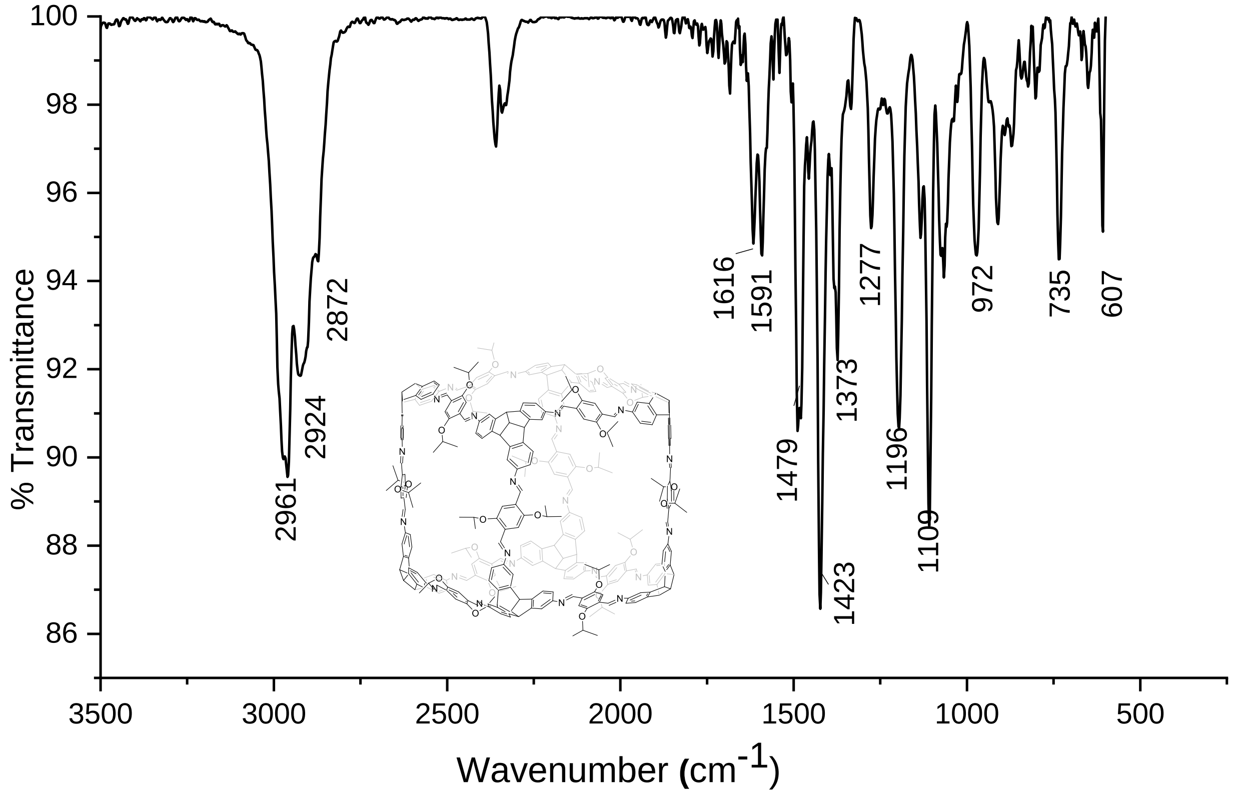

Figure 358. FT-IR (ATR) spectrum of **O^{i}Pr-cube**.

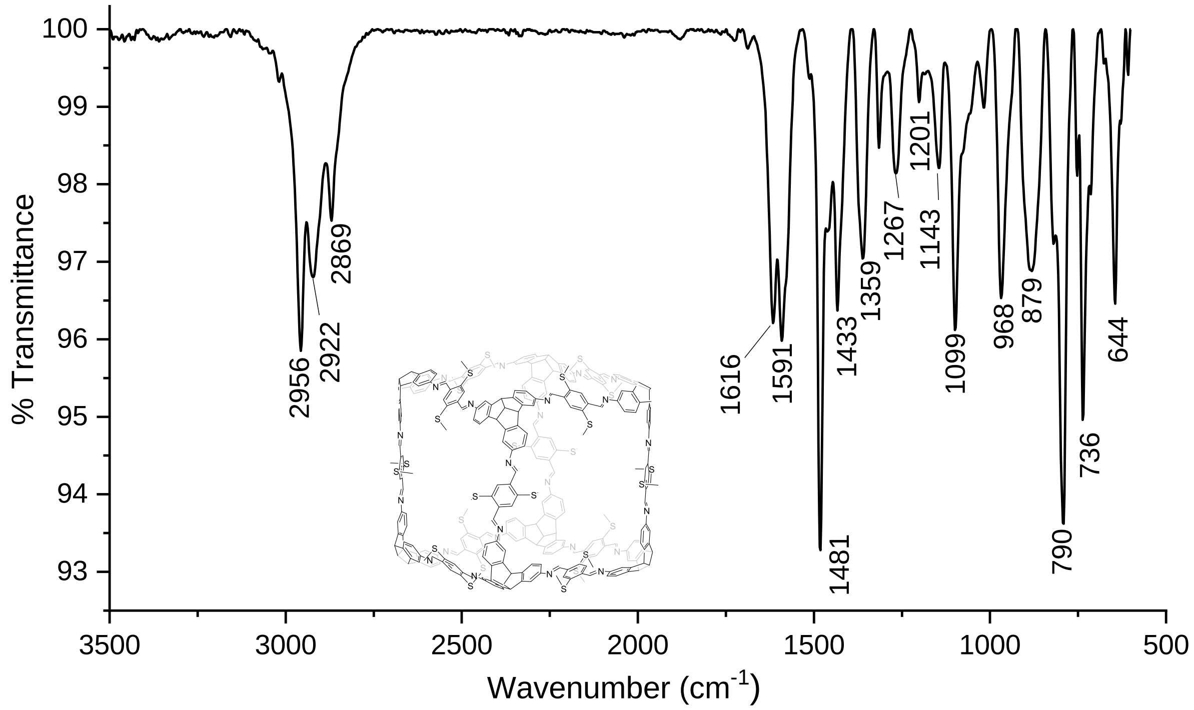

Figure 359. FT-IR (ATR) spectrum of **SMe-cube**.

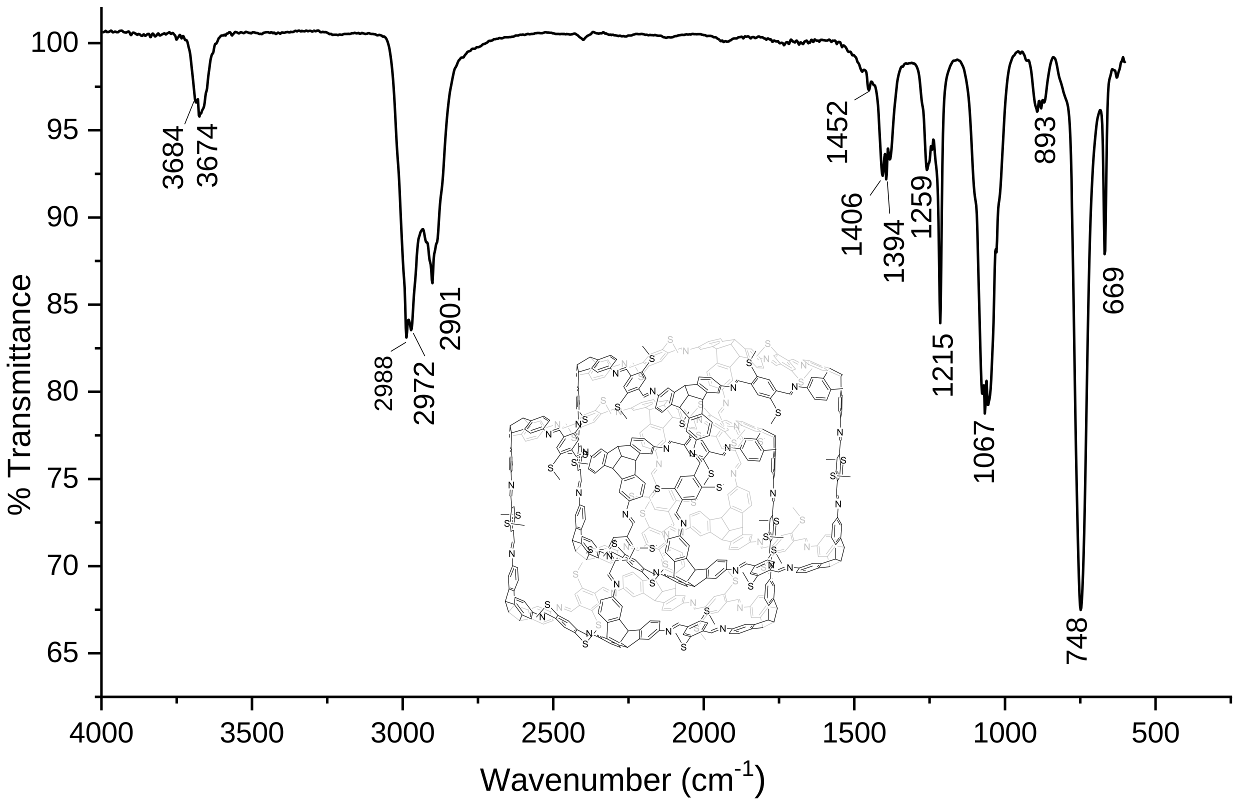

Figure 360. FT-IR spectrum (DCM) of **(SMe-cube)$_2$**.

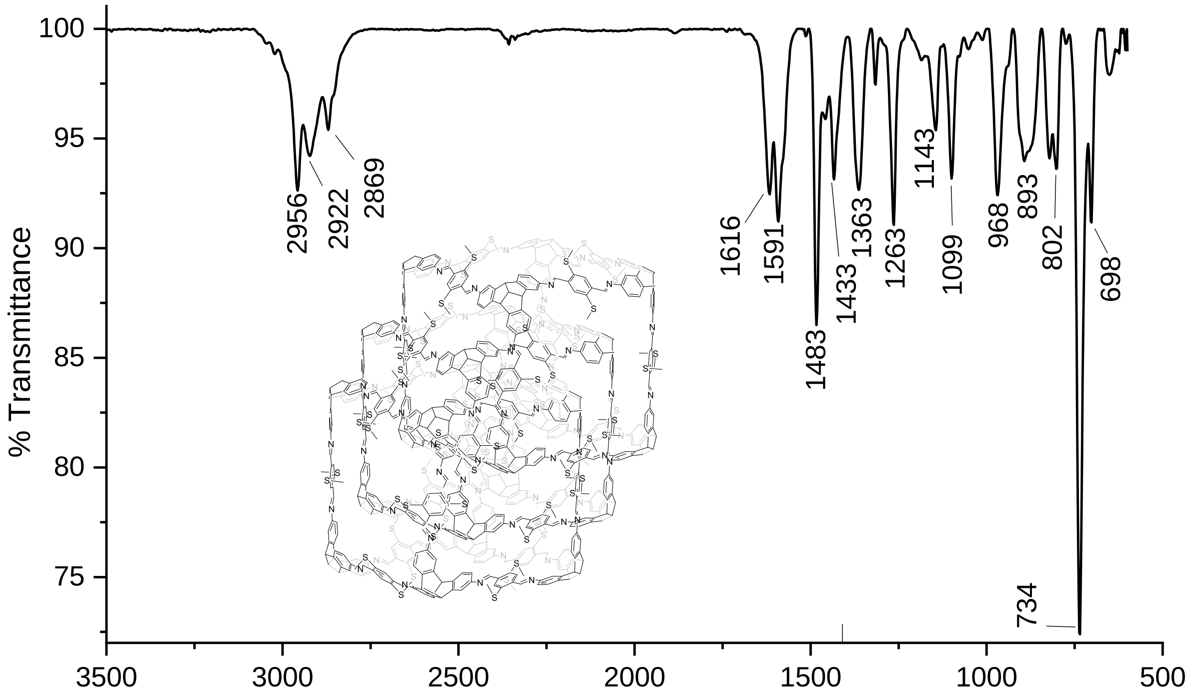

Figure 361. FT-IR (ATR) spectrum of **(SMe-cube)$_3$**.

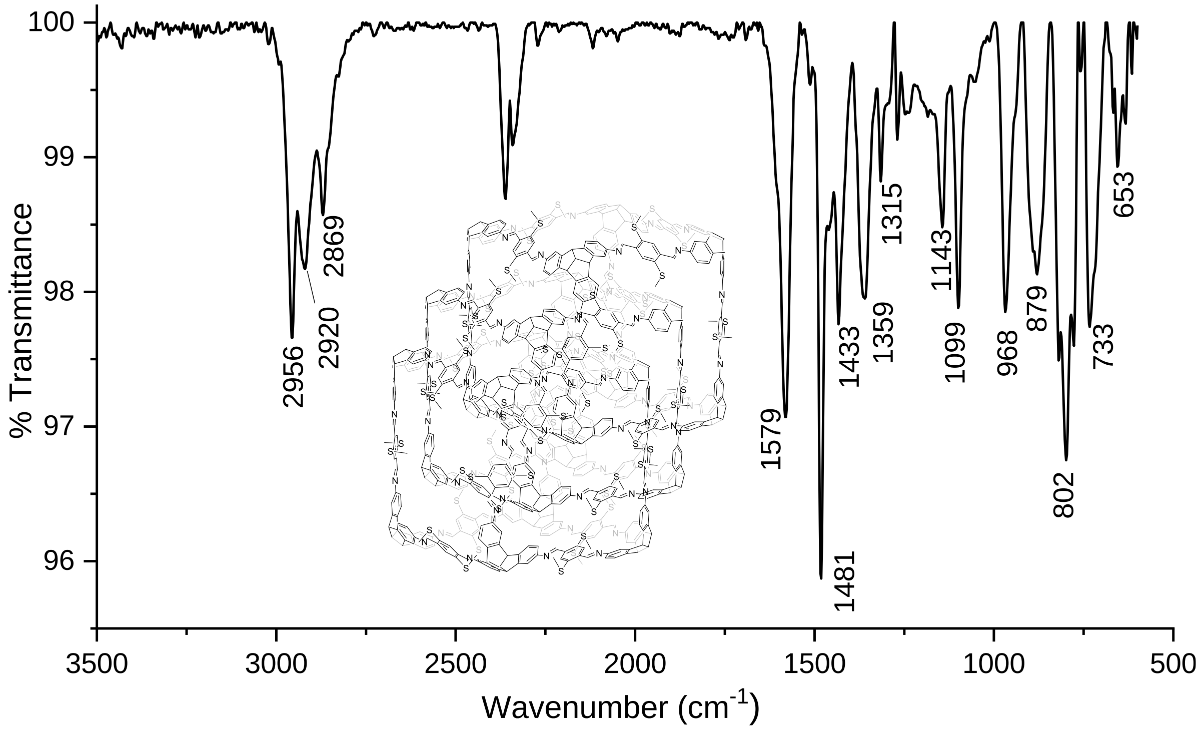

Figure 362. FT-IR (ATR) spectrum of **^{15}N labelled *(SMe-cube)$_3$**.

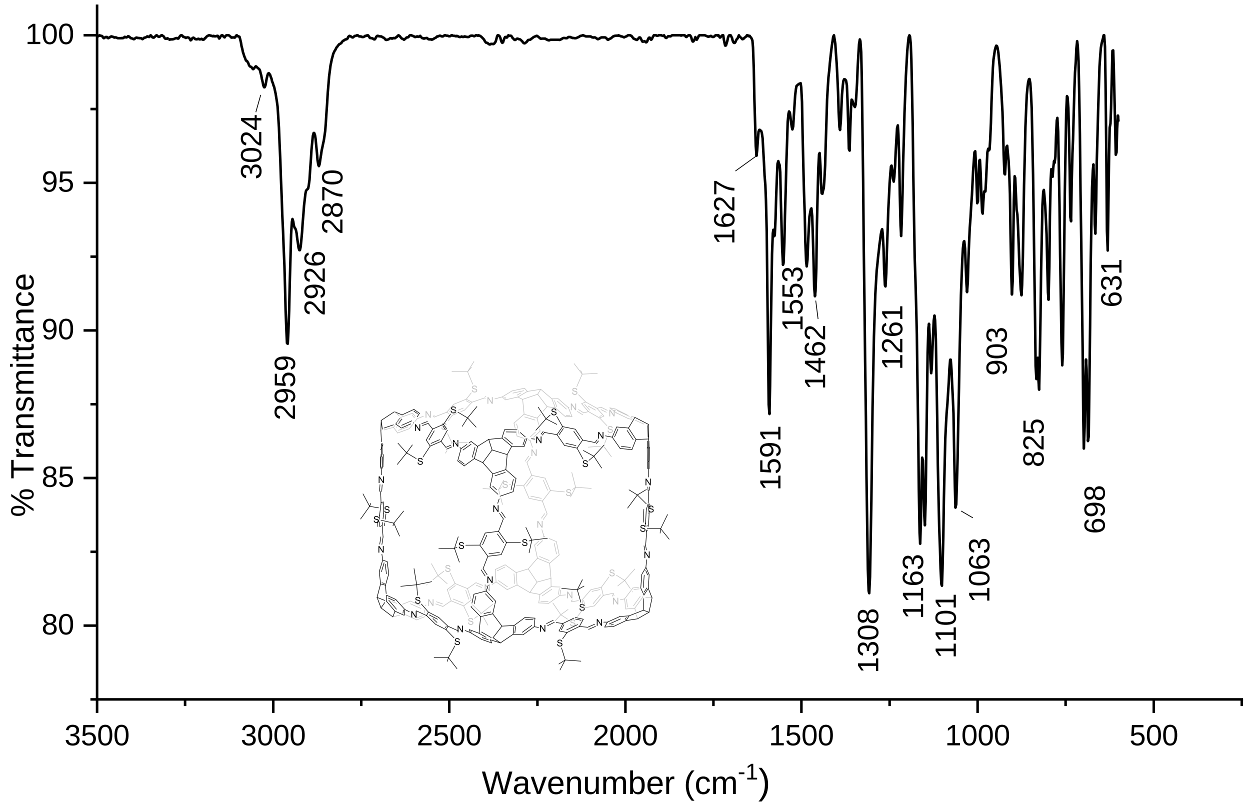

Figure 363. FT-IR spectrum (DCM) of **$SC(CH_3)_3$-cube**.

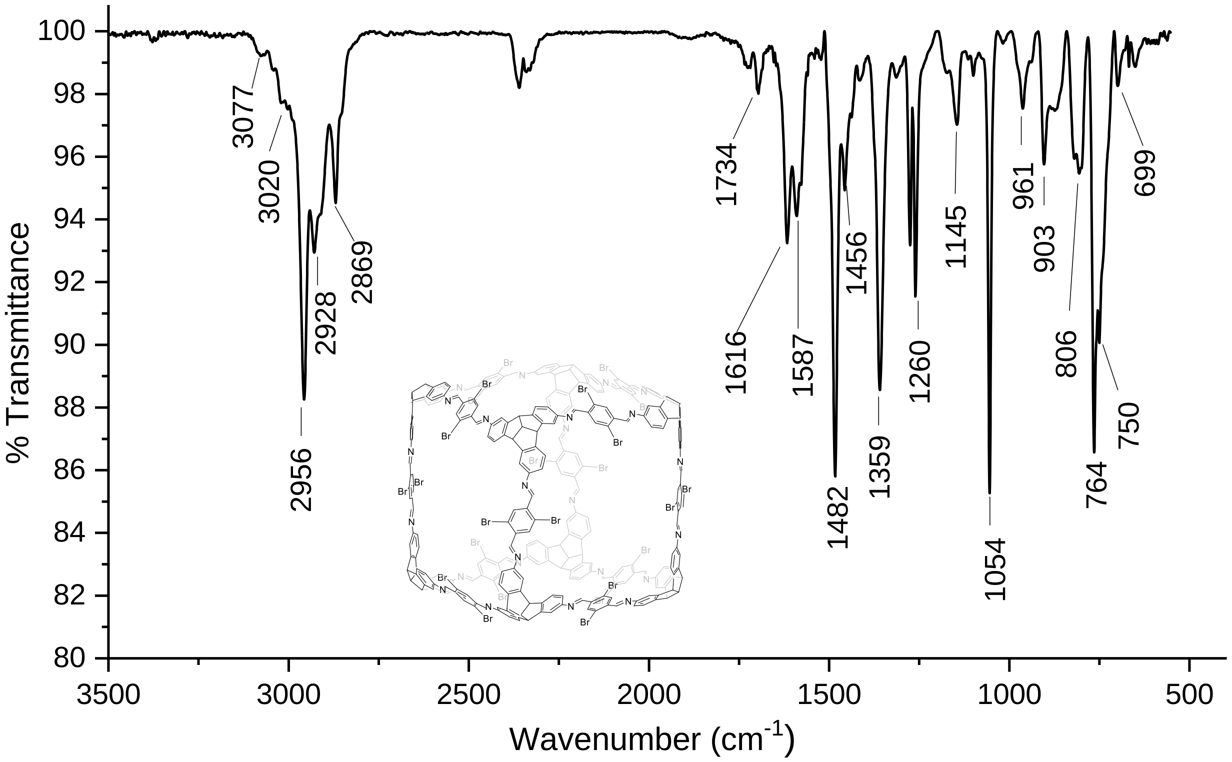

Figure 364. FT-IR (ATR) spectrum of **Br-cube**.

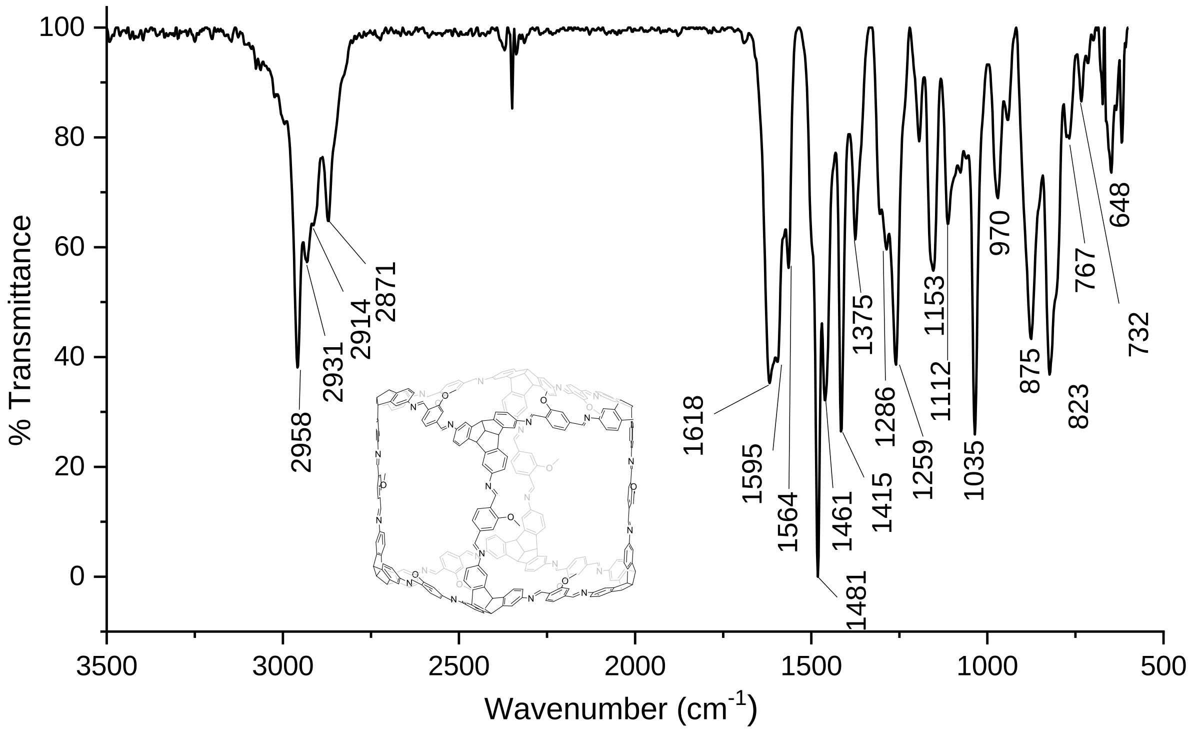

Figure 365. FT-IR (ATR) spectrum of **Mono-OMe-cube**.

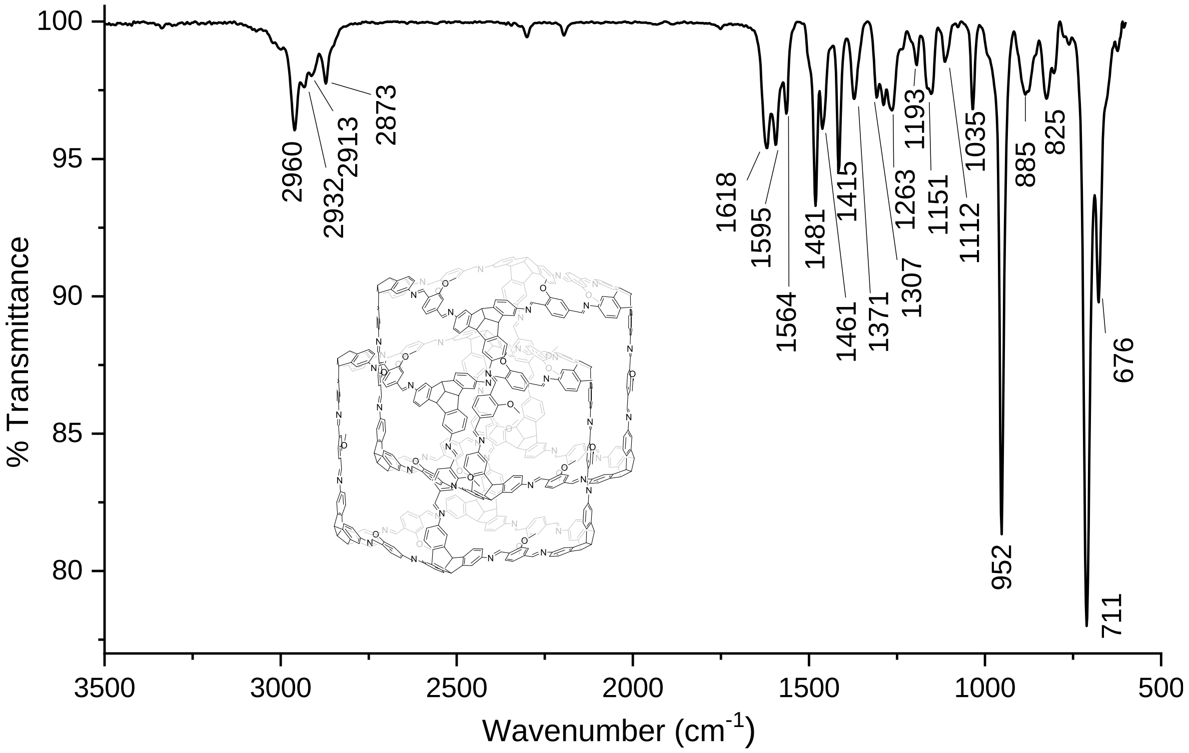

Figure 366. FT-IR (ATR) spectrum of **(mono-OMe-cube)$_2$**.

7. UV-Vis spectra

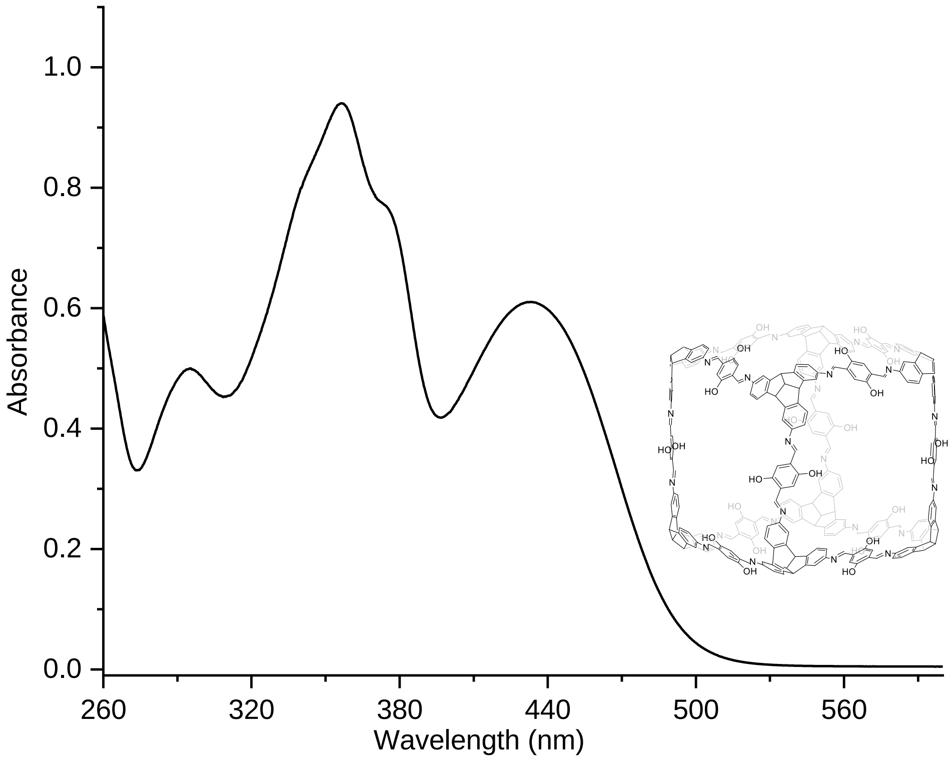

Figure 367. UV-Vis spectrum (DCM, conc. 3.2 µM) of **OH-cube**.

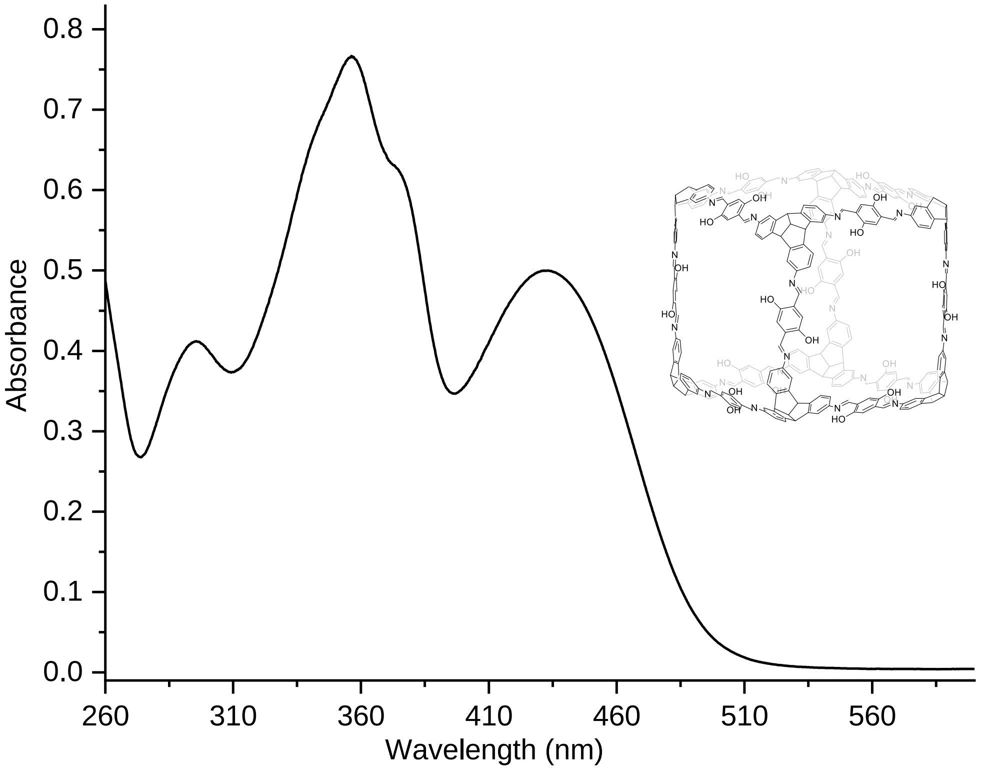

Figure 368. UV-Vis spectrum (DCM, conc. 0.94 µM) of **OH-cube**.

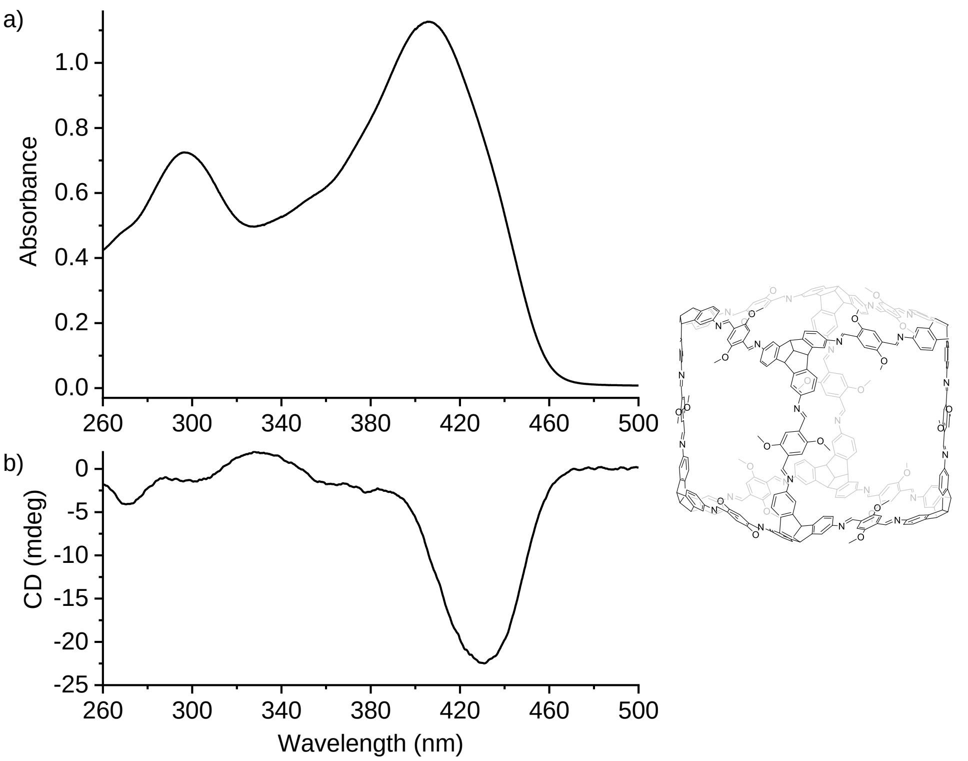

Figure 369. UV-Vis spectrum (DCM, Conc. 3.6 µM) and CD spectrum of **OMe-cube**.

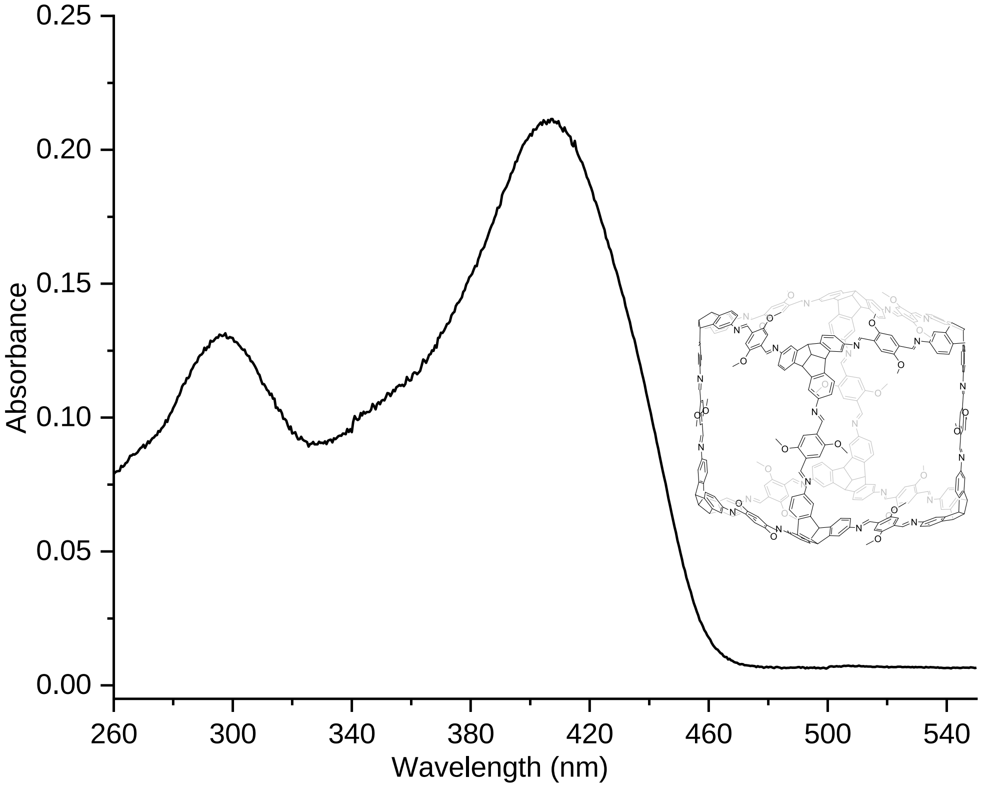

Figure 370. UV-Vis spectrum (DCM, conc. 0.59 µM) of **^{15}N labelled *OMe-cube**.

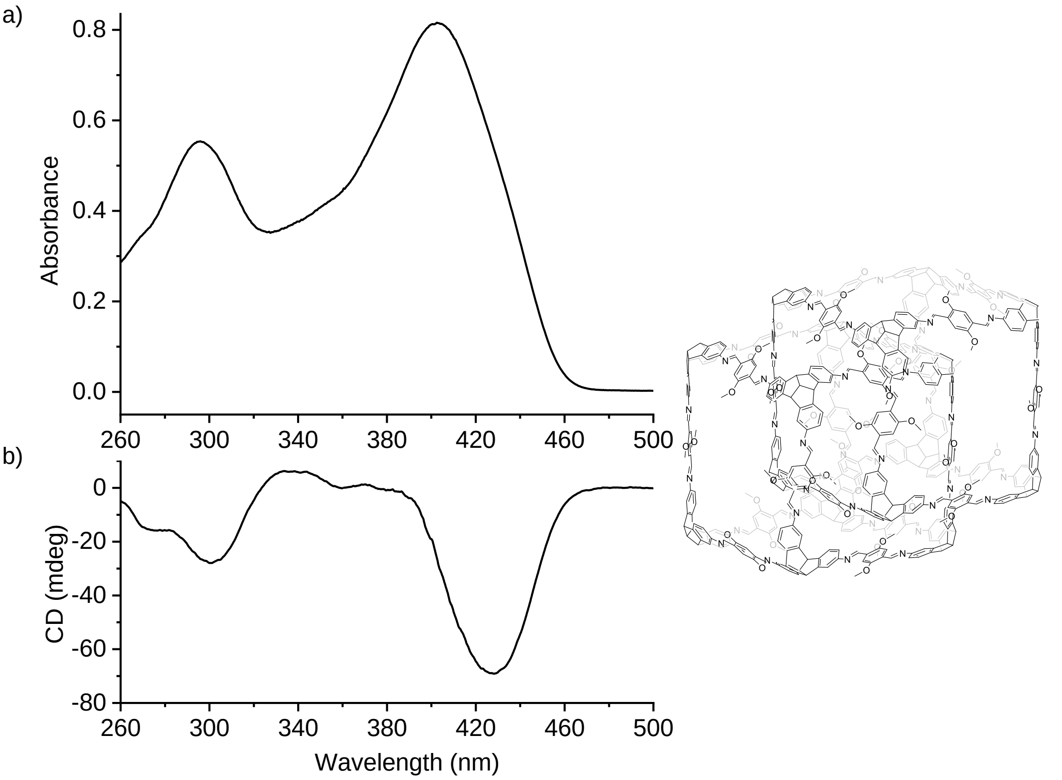

Figure 371. UV-Vis spectrum (DCM, Conc. 1.1 μM) and CD (DCM) spectrum of **(OMe-cube)$_2$**.

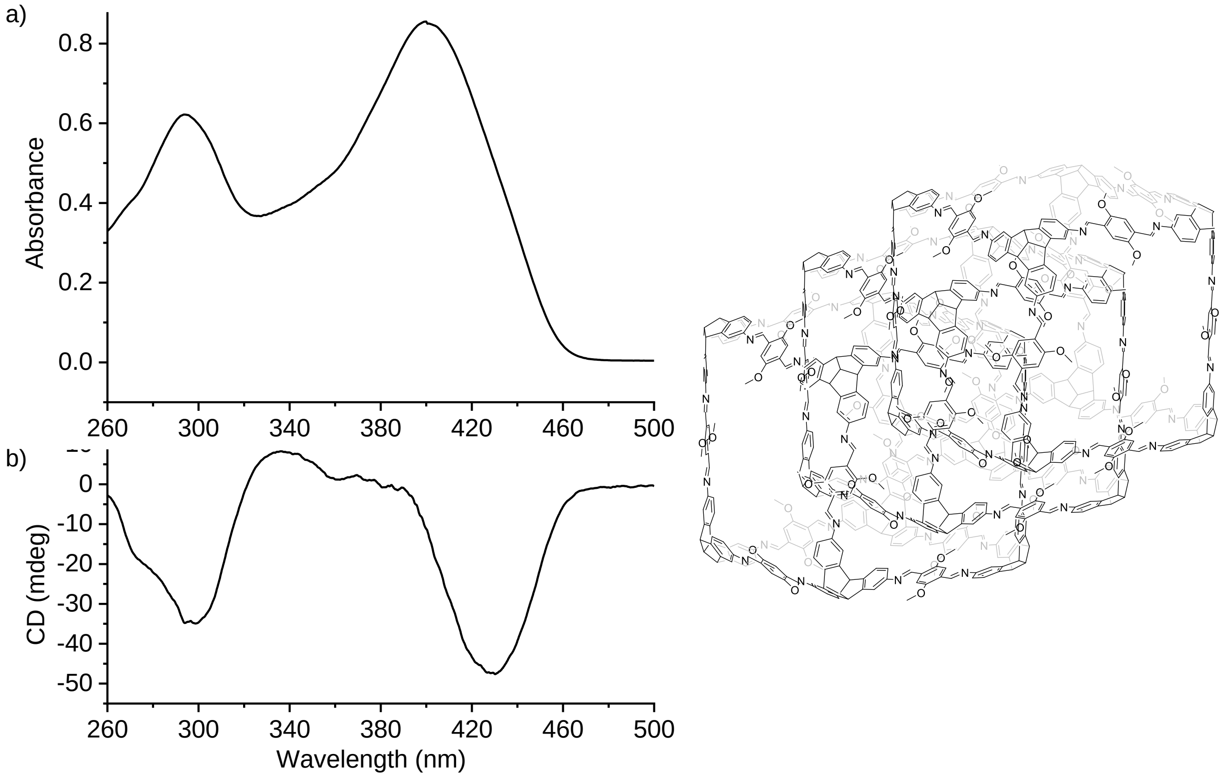

Figure 372. UV-Vis spectrum (DCM, Conc. 1.1 μM) and CD spectrum (DCM) of **(OMe-cube)$_3$**.

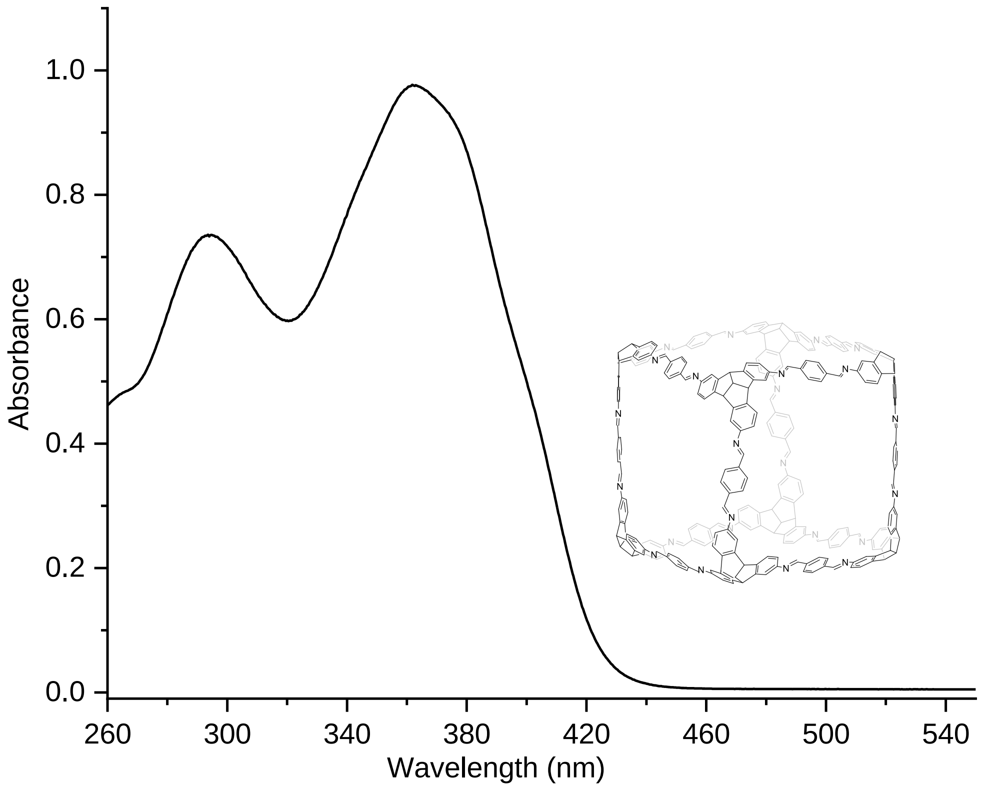

Figure 373. UV-Vis spectrum (DCM, Conc. 4.2 μM) of **H-cube**.

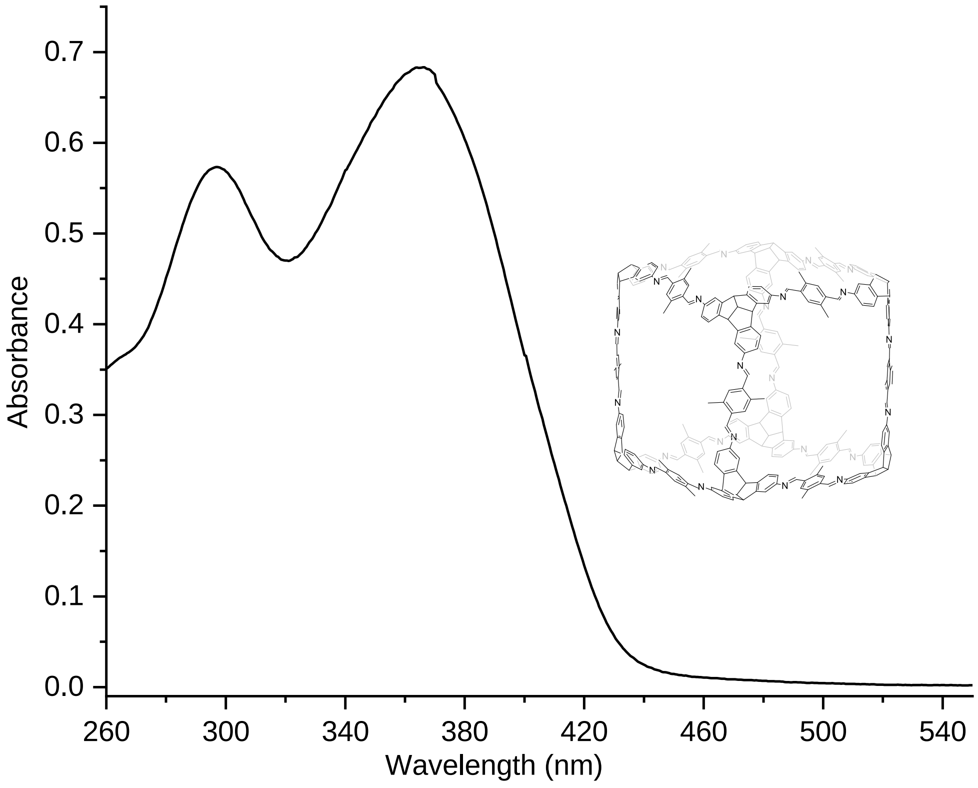

Figure 374. UV-Vis spectrum (DCM, Conc. 3.18 μM) of **Me-cube**.

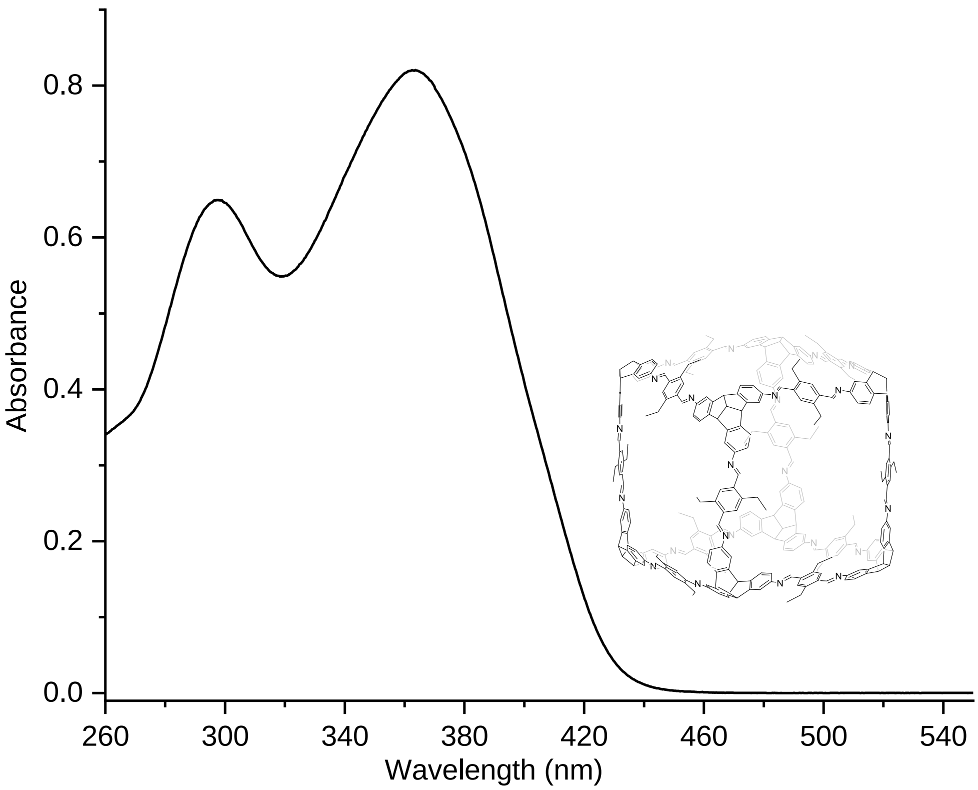

Figure 375. UV-Vis spectrum (DCM, Conc. 5.1 µM) of **Et-cube**.

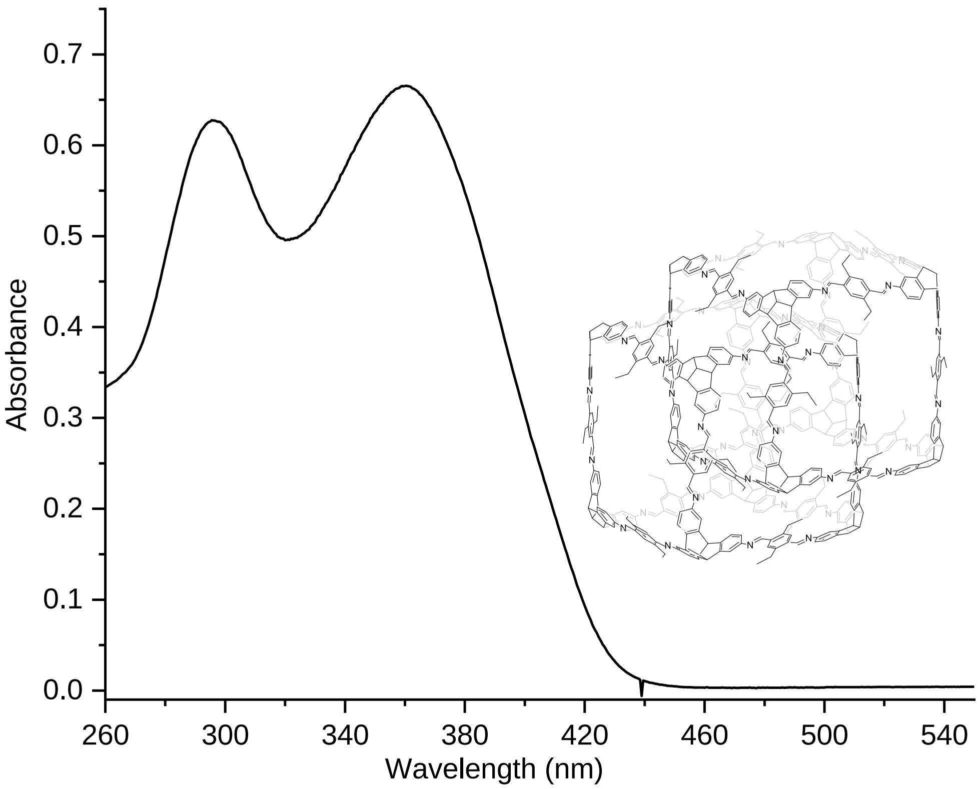

Figure 376. UV-Vis spectrum (DCM, Conc. 2.1 µM) of **(Et-cube)₂**.

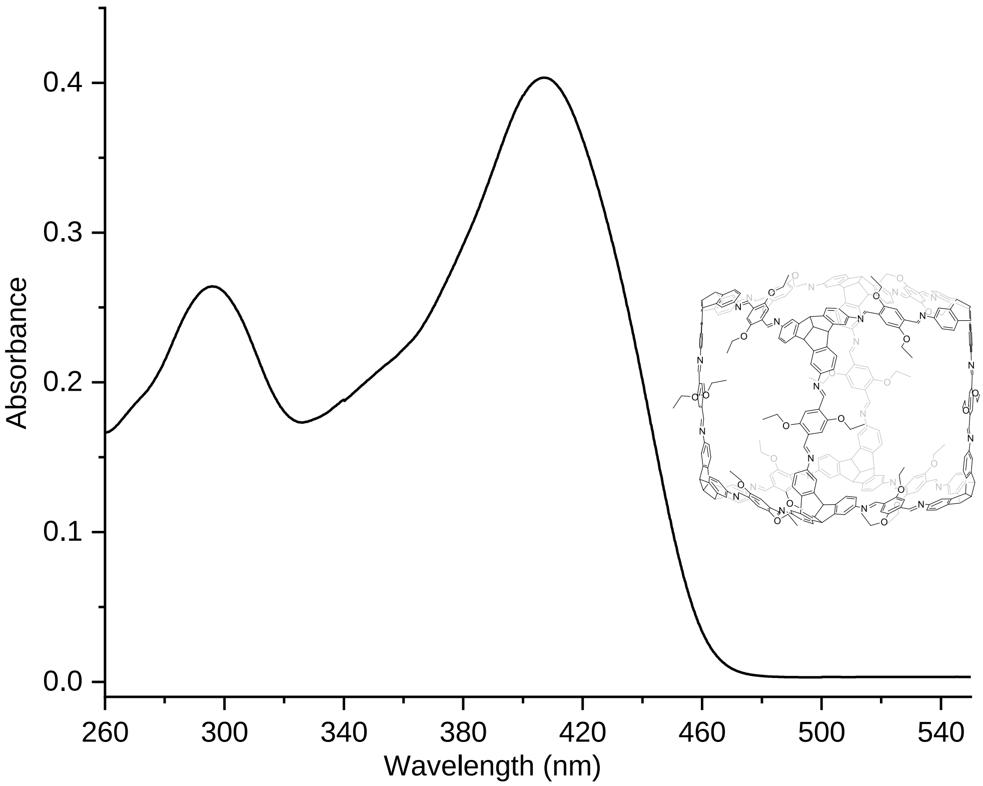

Figure 377. UV-Vis spectrum ($CHCl_3$, Conc. 0.012 µM) of **OEt-cube**.

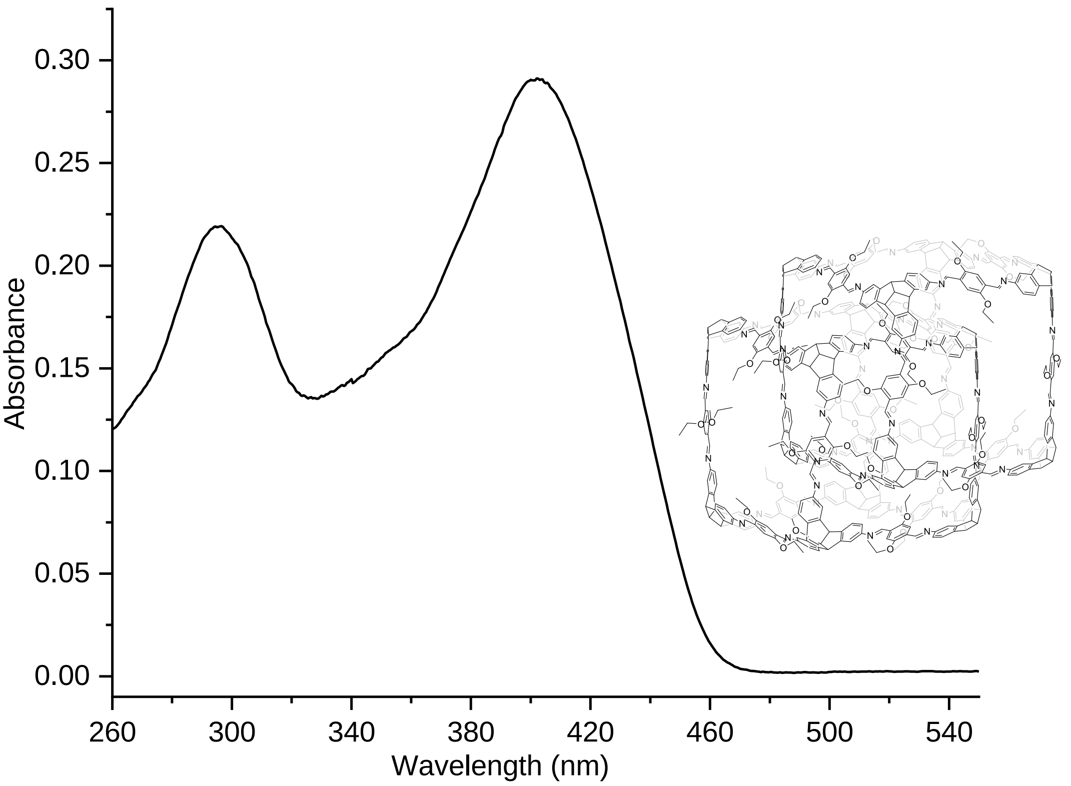

Figure 378. UV-Vis spectrum (DCM, Conc. 0.011 μM) of **(OEt-cube)$_2$**.

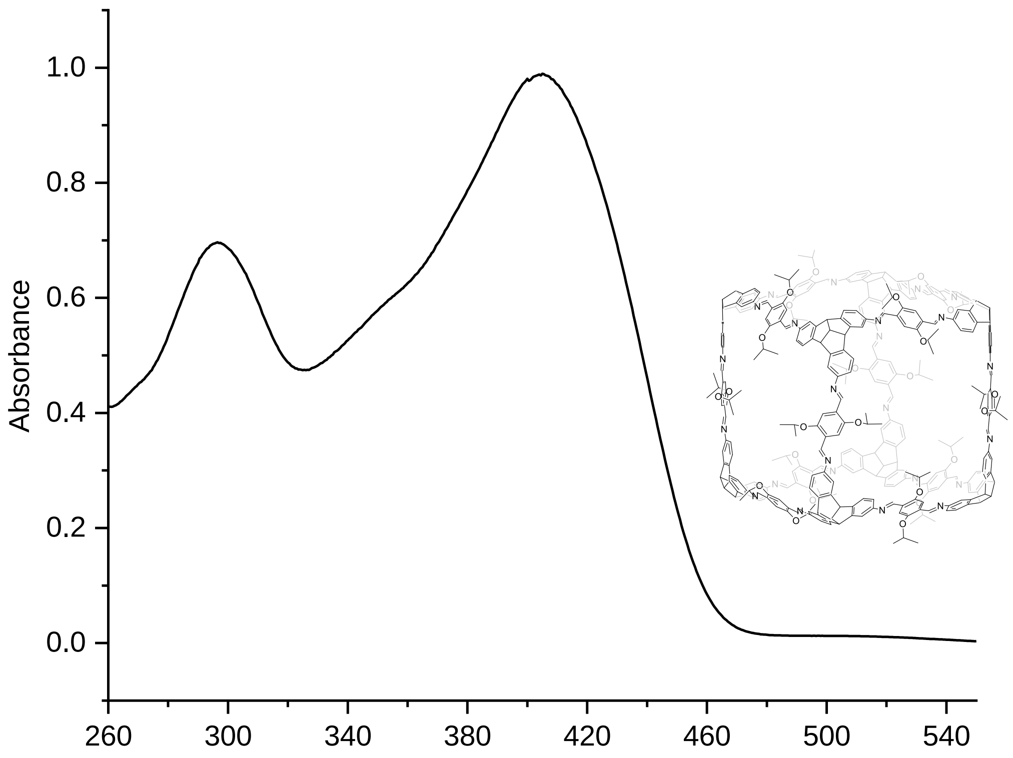

Figure 379. UV-Vis spectrum ($CHCl_3$, Conc. 5.2 μM) of **OiPr-cube**

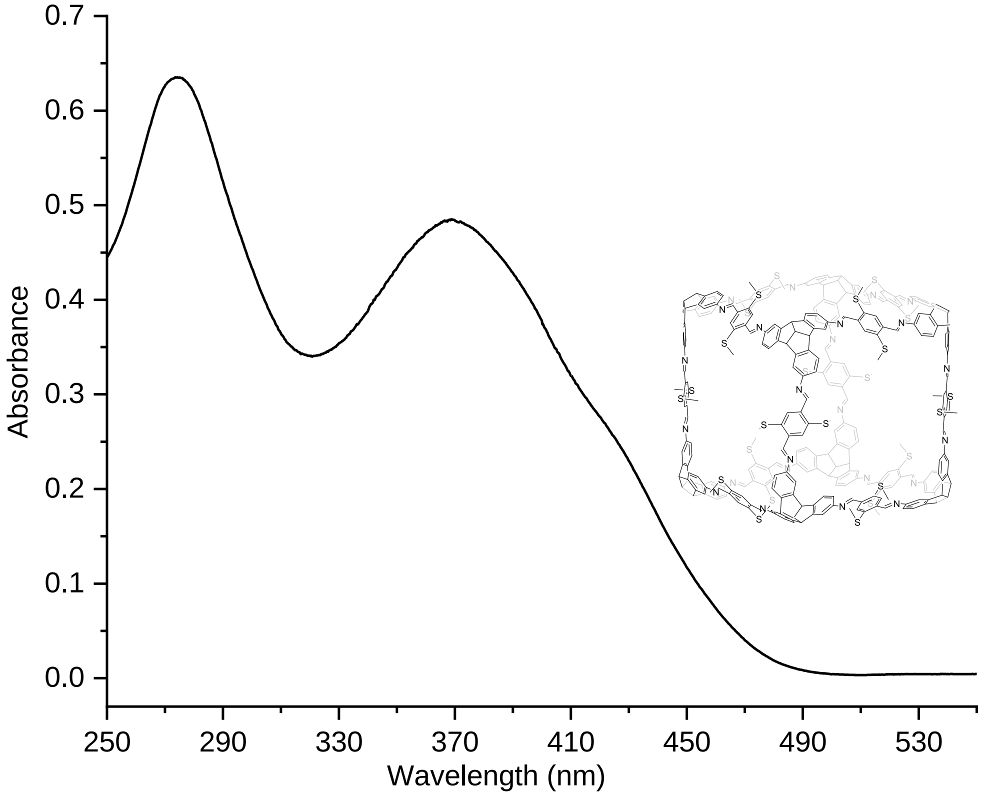

Figure 380. UV-Vis spectrum (DCM, conc.2.77 µM) of **SMe-cube**.

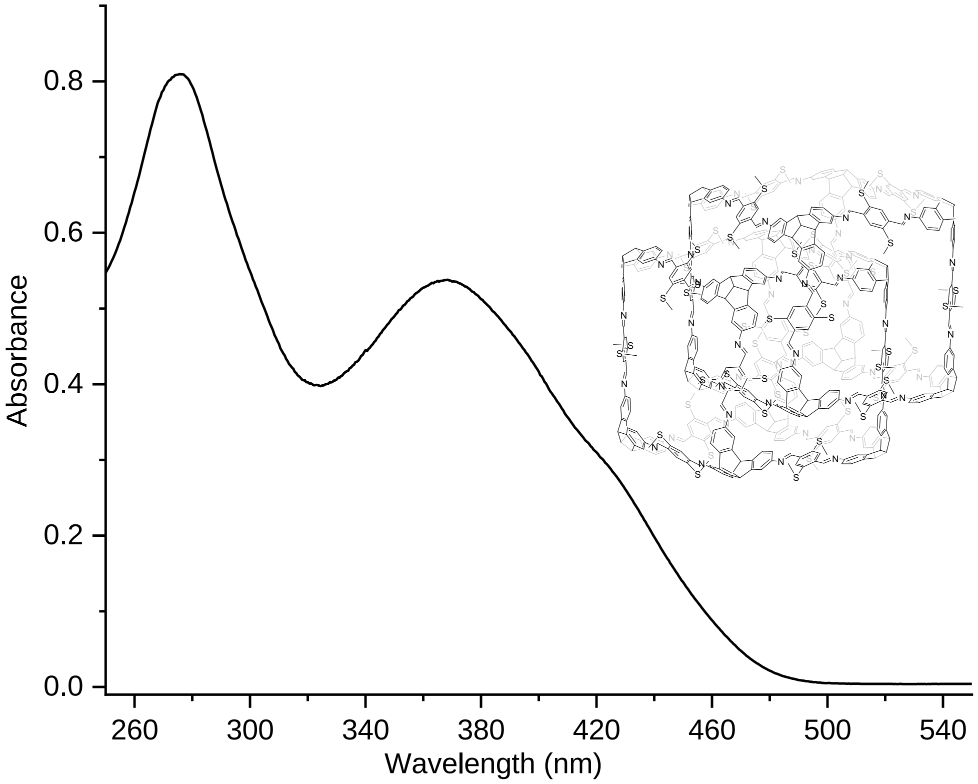

Figure 381. UV-Vis spectrum (DCM, Conc. 2.7 µM) of **(SMe-cube)$_2$**.

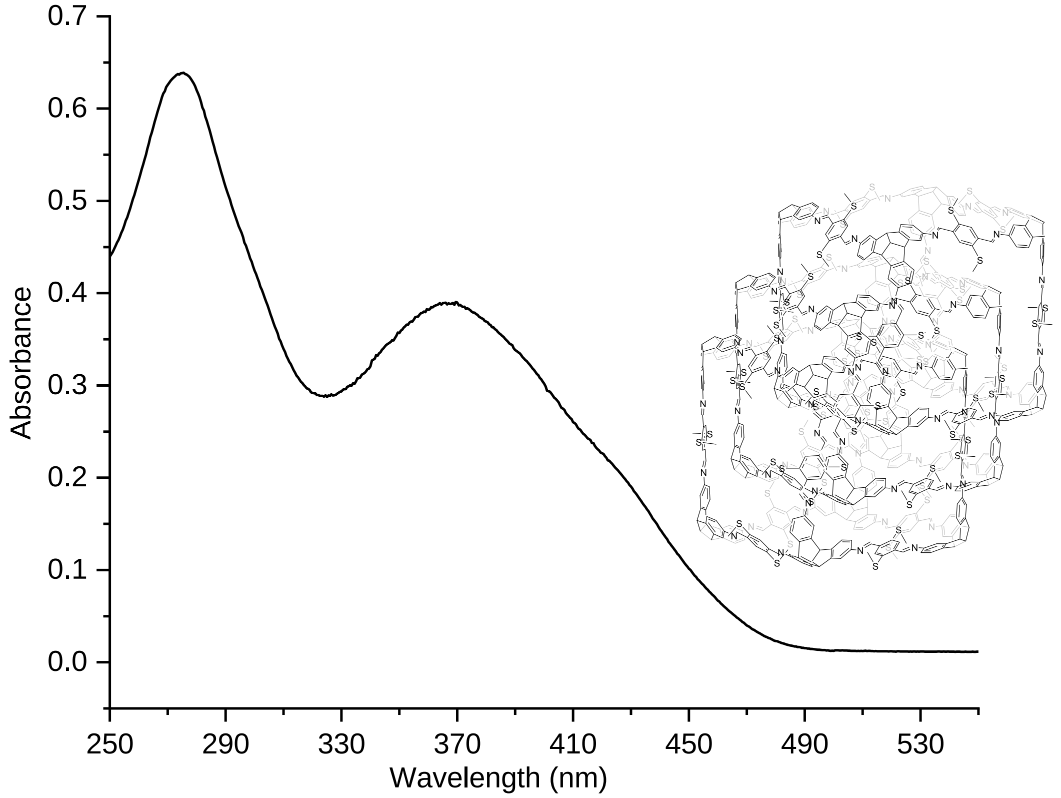

Figure 382. UV-Vis spectrum (DCM, conc. 0.65 µM) of **(SMe-cube)$_3$**.

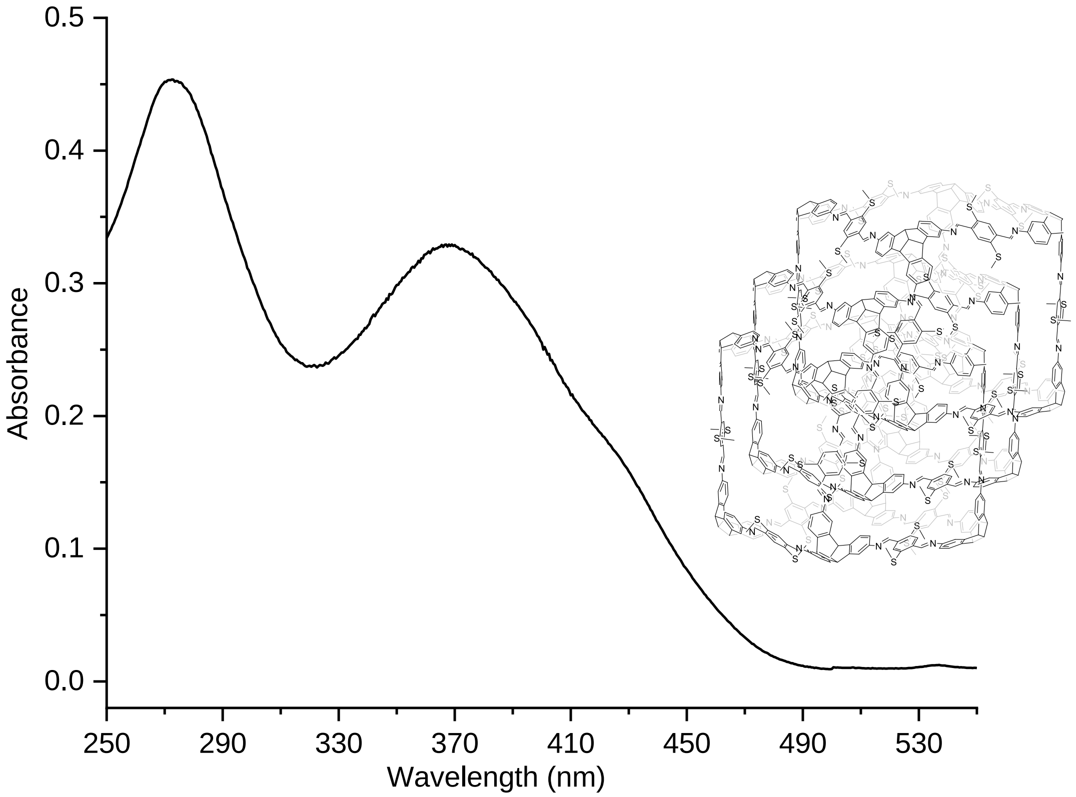

Figure 383. UV-Vis spectrum (DCM, conc. 0.64 µM) of **^{15}N labelled *(SMe-cube)$_3$**.

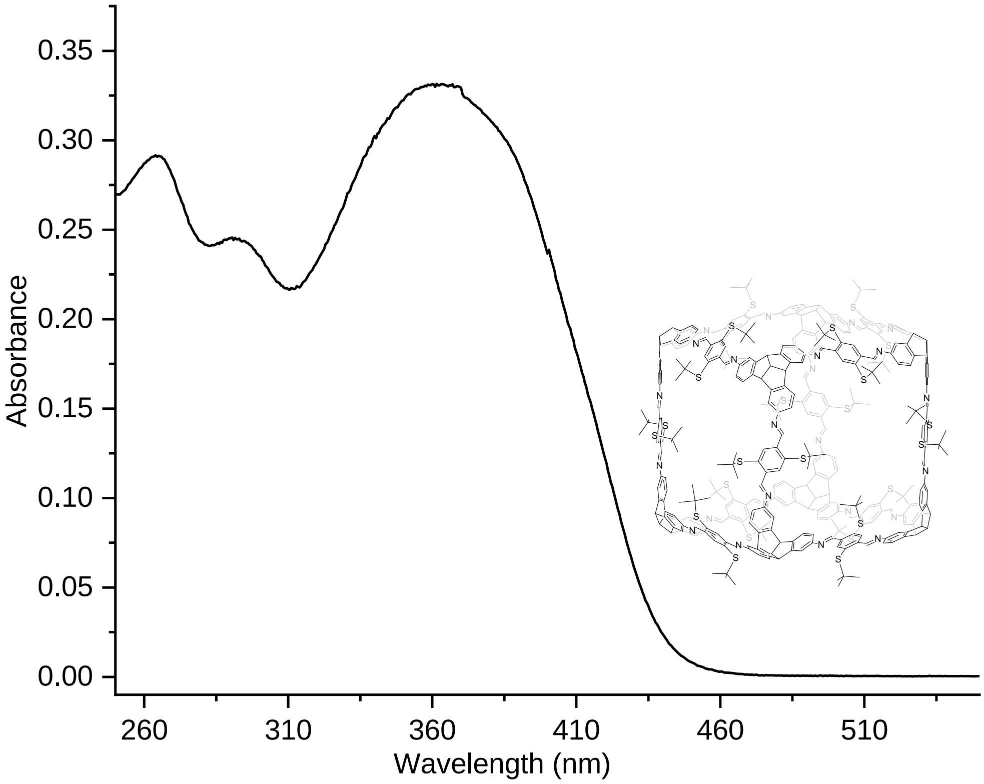

Figure 384. UV-Vis spectrum (DCM, Conc. 1.9 µM) of **$SC(CH_3)_3$-cube**.

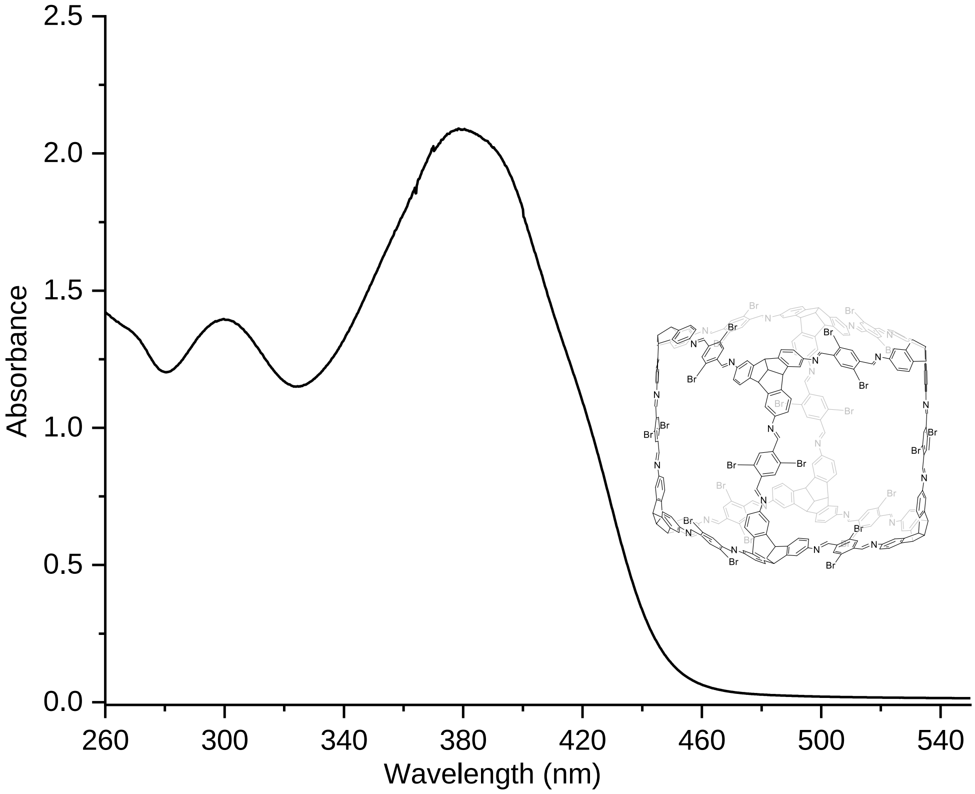

Figure 385. UV-Vis spectrum (DCM, Conc. 9.8 µM) of **Br-cube**.

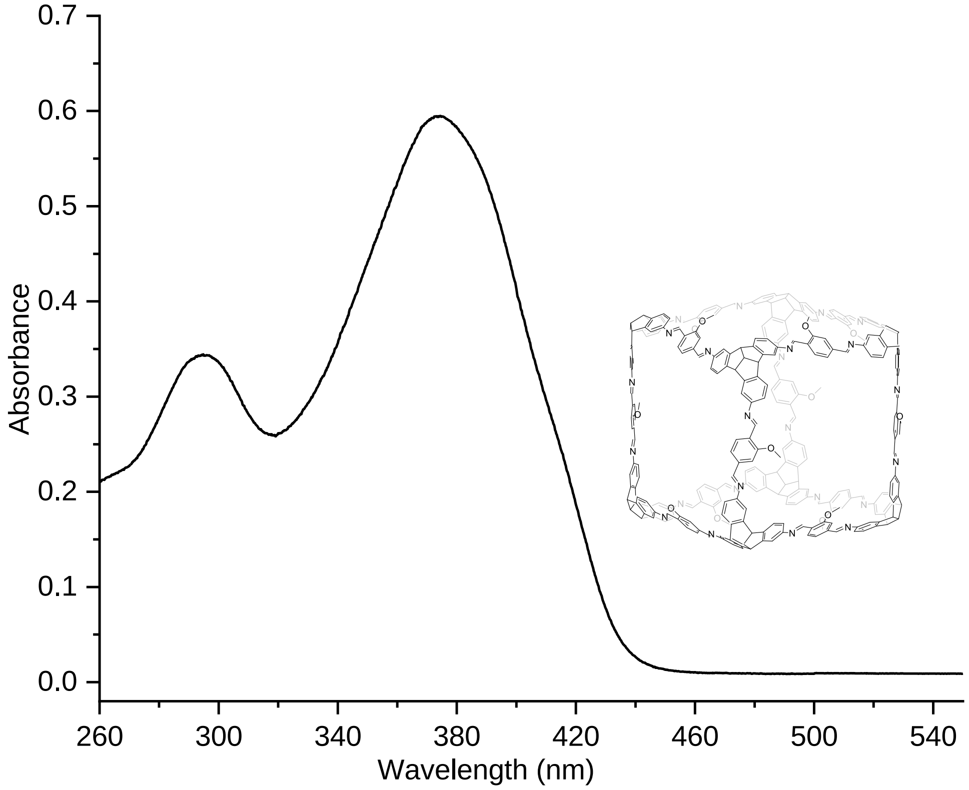

Figure 386. UV-Vis spectrum (DCM, conc. 1.27 µM) of **Mono-OMe-cube**.

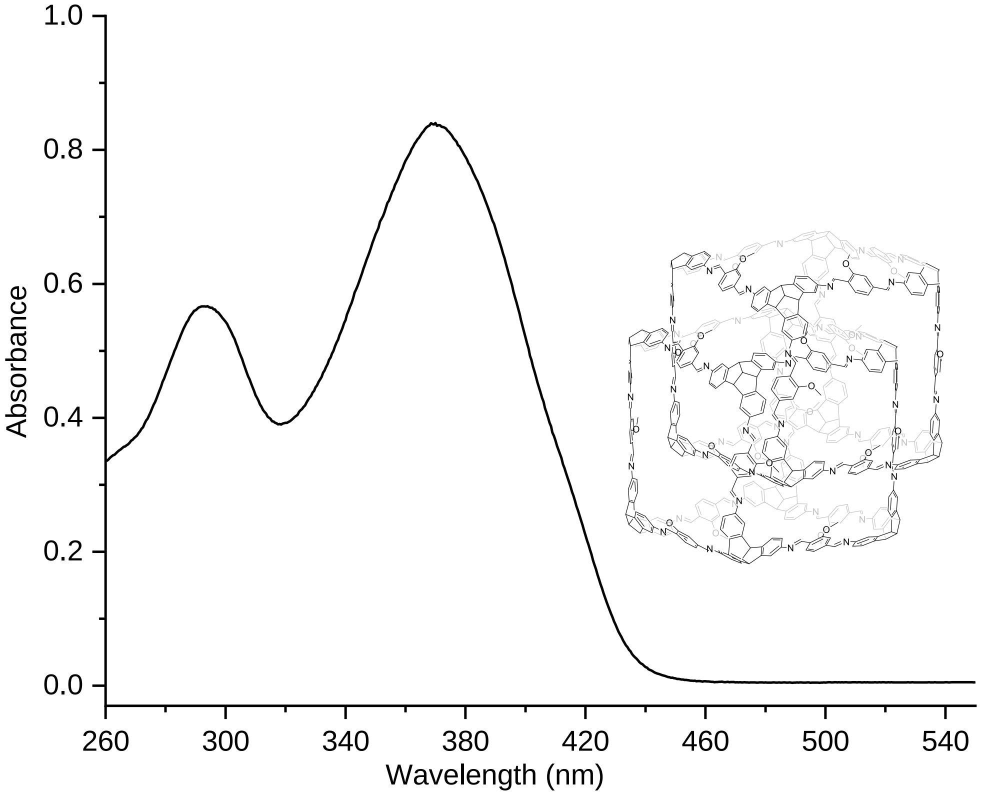

Figure 387. UV-Vis spectrum (DCM, conc. 1.38 µM) of **(Mono-OMe-cube)$_2$**

8. GPC data

OMe-cube:

Sample preparation: To avoid the catenane formation during separation, pure **OMe-cube** in DCM (0.8 mg dissolved in 5 mL of DCM) was injected into the r-GPC to get the trace of the pure **OMe-cube**.

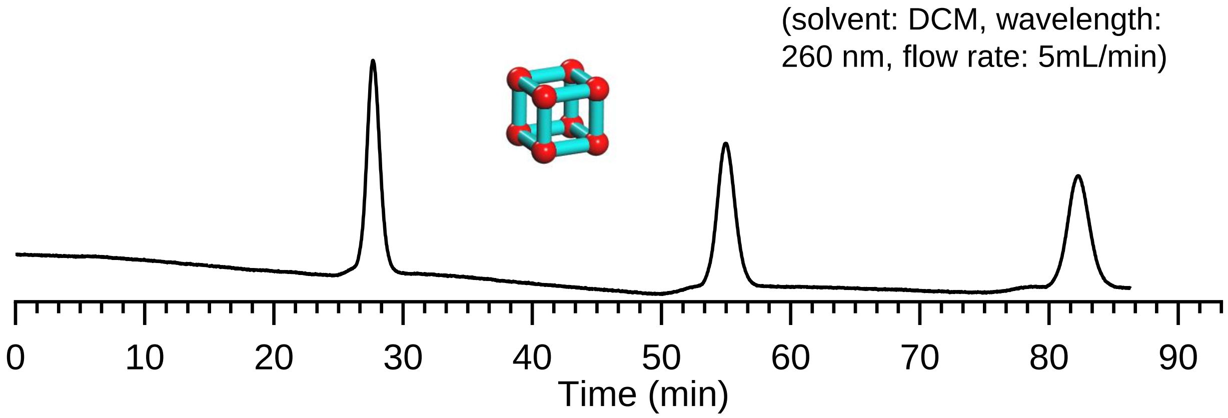

Figure 388. GPC trace of pure **OMe-cube** (solvent: dichloromethane, flow rate: 5mL/min, Temp: 30˚C, wavelength: 260 nm, GPC Columns: 3 x 100 Å (20 x 300 mm))

(OMe-cube)$_2$:

Sample preparation for GPC: The crude reaction mixture (~32 mg) was first passed through a small pre-packed size exclusion column using DCM as a mobile phase and the collected fraction (10 mL) was concentrated to 0.5 mL under reduced pressure and injected into the r-GPC in one portion. Total 14 mg of pure **(OMe-cube)$_2$** was collected.

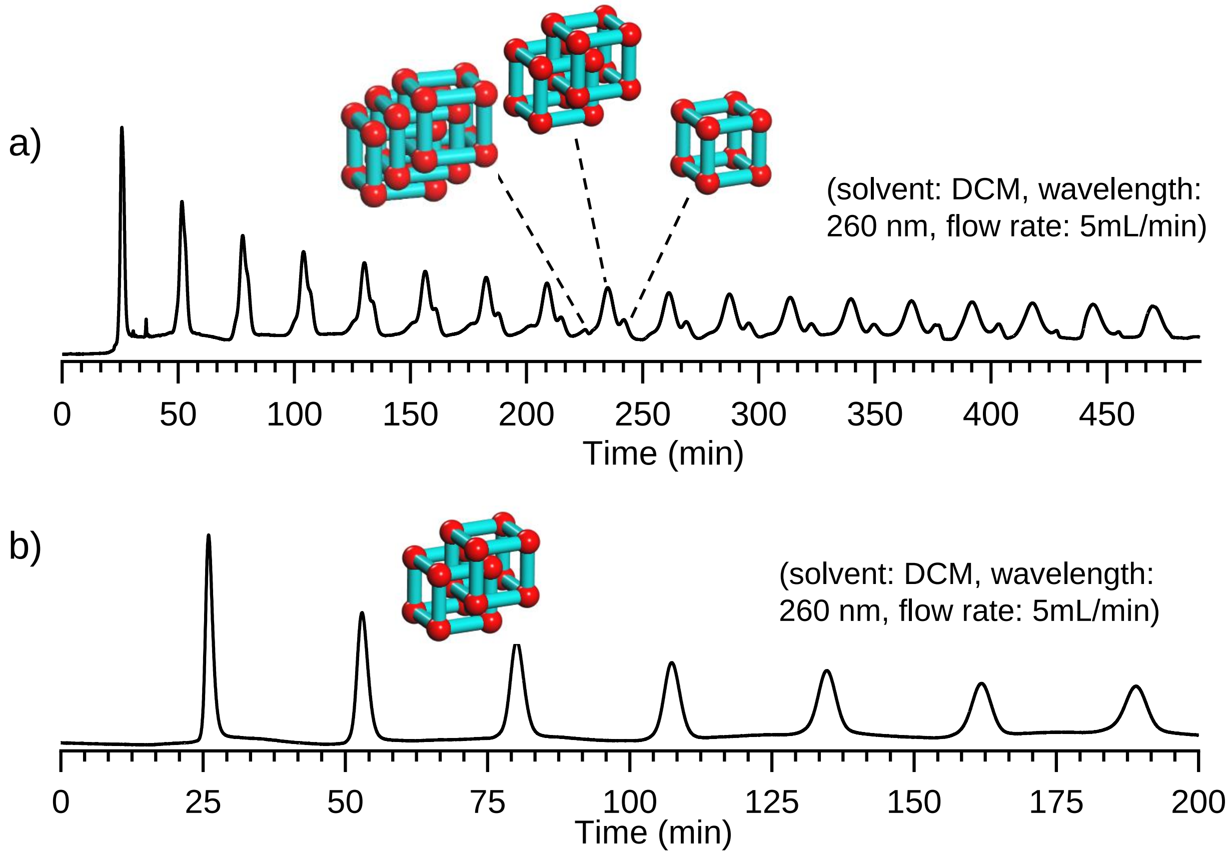

Figure 389. GPC traces of **(OMe-cube)$_2$**. a) GPC trace of crude reaction mixture of **(OMe-cube)$_2$** prepared by improved protocol and b) GPC trace of isolated pure **(OMe-cube)$_2$** (solvent: dichloromethane, flow rate: 5mL/min, temp: 30˚C, wavelength: 260 nm, GPC Columns: 3 x 100 Å (20 x 300 mm)).

***Note:** It was found that **(OMe-cube)$_2$** shows decomposition after multiple cycles on rGPC column when chloroform solvent was used instead of DCM.*

(OMe-cube)$_3$:

Sample preparation for GPC: The crude reaction mixture (~ 16 mg) was first passed through a small pre-packed size exclusion column using DCM as a mobile phase and then the collected fraction (10 mL) was concentrated to 0.5 mL under reduced pressure and injected into the rGPC. Very small amount (< 1mg) of pure **(OMe-cube)$_3$** was collected. **(OMe-cube)$_3$** was confirmed by MALDI-TOF mass technique.

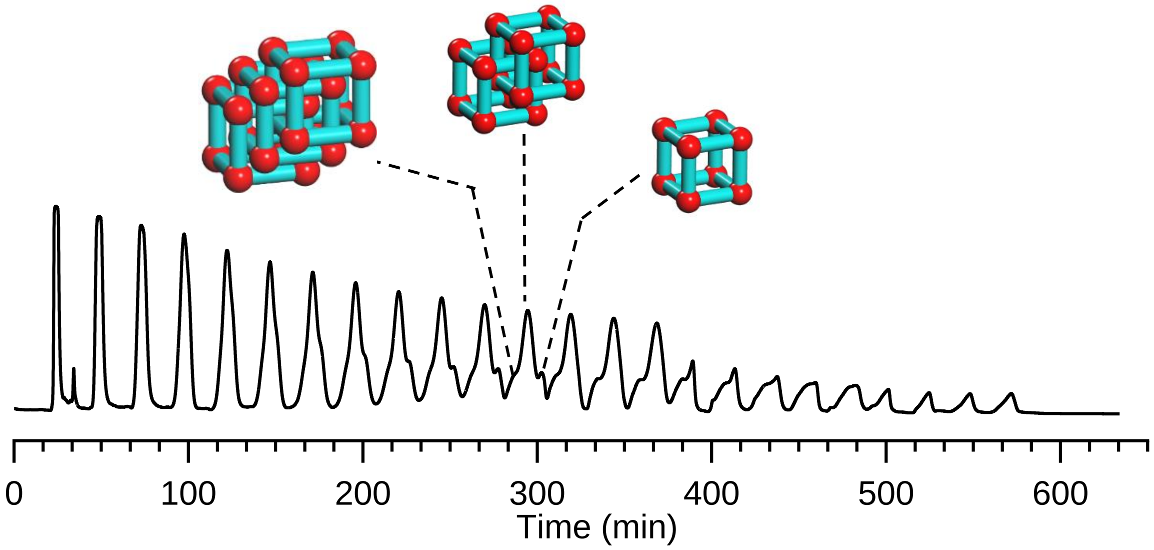

Figure 390. Separation of **(OMe-cube)$_3$** from crude reaction mixture (reaction solvent: $CHCl_3$). (Chloroform: flow rate: 5mL/min, temp: 40˚C, wavelength: 260 nm, GPC columns: 3 x 100 Å (20 x 300 mm)).

(OMe-cube)₃ (synthesized by improved protocol):

Sample preparation for GPC: The crude reaction mixture (~32 mg) was first passed through a small pre-packed size exclusion column using DCM as a mobile phase and then the collected fraction (10 mL) was concentrated to 0.5 mL under reduced pressure and injected into the rGPC in one portion. Total 16 mg of pure **(OMe-cube)$_3$** was collected.

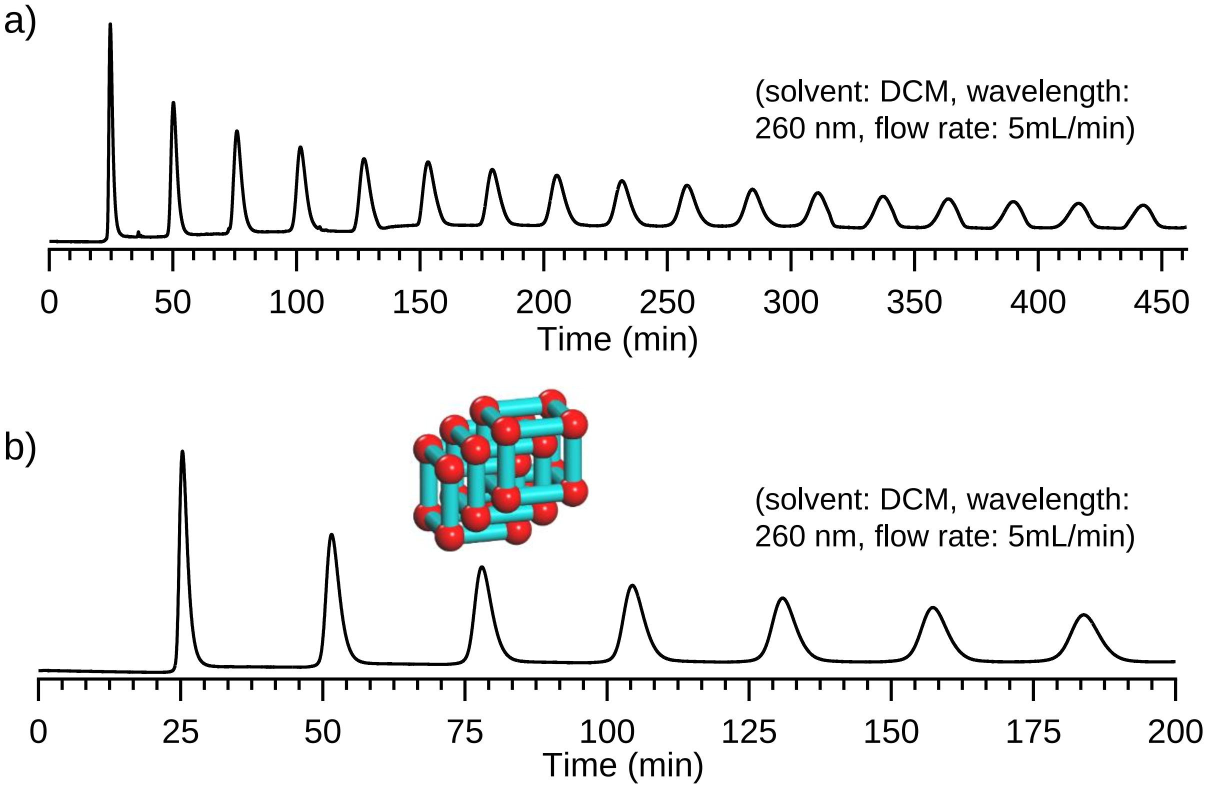

Figure 391. GPC profile of **(OMe-cube)$_3$**. a) GPC trace of crude reaction mixture of **(OMe-cube)$_3$** prepared by improved protocol and b) GPC trace of pure **(OMe-cube)$_3$** (solvent: dichloromethane, flow rate: 5mL/min, temp: 30°C, wavelength: 260 nm, GPC columns: 3 x 100 Å (20 x 300 mm)).

***Note:** It was found that **(OMe-cube)$_3$** shows decomposition after multiple cycles on rGPC column when chloroform solvent was used instead of DCM.*

(Et-cube)2: (synthesized by improved protocol)

Sample preparation for GPC: THF was first removed from the crude reaction mixture under reduced pressure. The crude reaction mixture (~17 mg) was redissolved in chloroform and passed through a small pre-packed size exclusion column using chloroform solvent as a mobile and then the collected fraction (6 mL) was concentrated to 0.5 mL under reduced pressure and injected into rGPC in one portion. Total 5.2 mg of pure **(Et-cube)2** was collected.

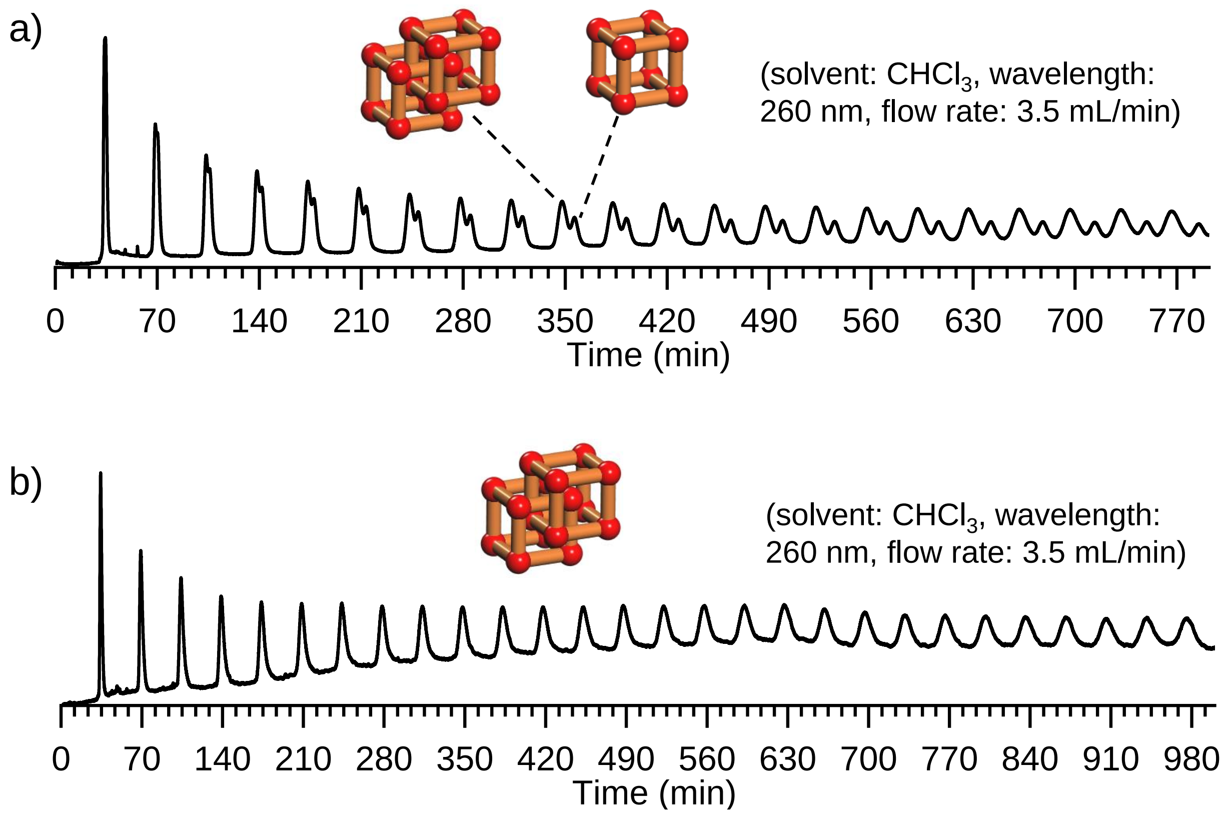

Figure 392. GPC profile of **(Et-cube)2**. a) GPC trace of crude reaction mixture of **(Et-cube)2** prepared by improved protocol and b) GPC trace of pure **(Et-cube)2** (solvent: chloroform, flow rate: 3.5mL/min, Temp: 40˚C, wavelength: 260 nm, GPC columns: 3 x 100 Å (20 x 300 mm)).

(OEt-cube)$_2$: (synthesized by improved protocol)

Sample preparation for GPC: The rude reaction mixture (~17 mg) was first passed through a small pre-packed size exclusion column using chloroform solvent as a mobile phase and then the collected fraction (6 mL) was concentrated to 0.5 mL under reduced pressure and injected into the rGPC in one portion. Total 4 mg of pure **(OEt-cube)$_2$** was collected.

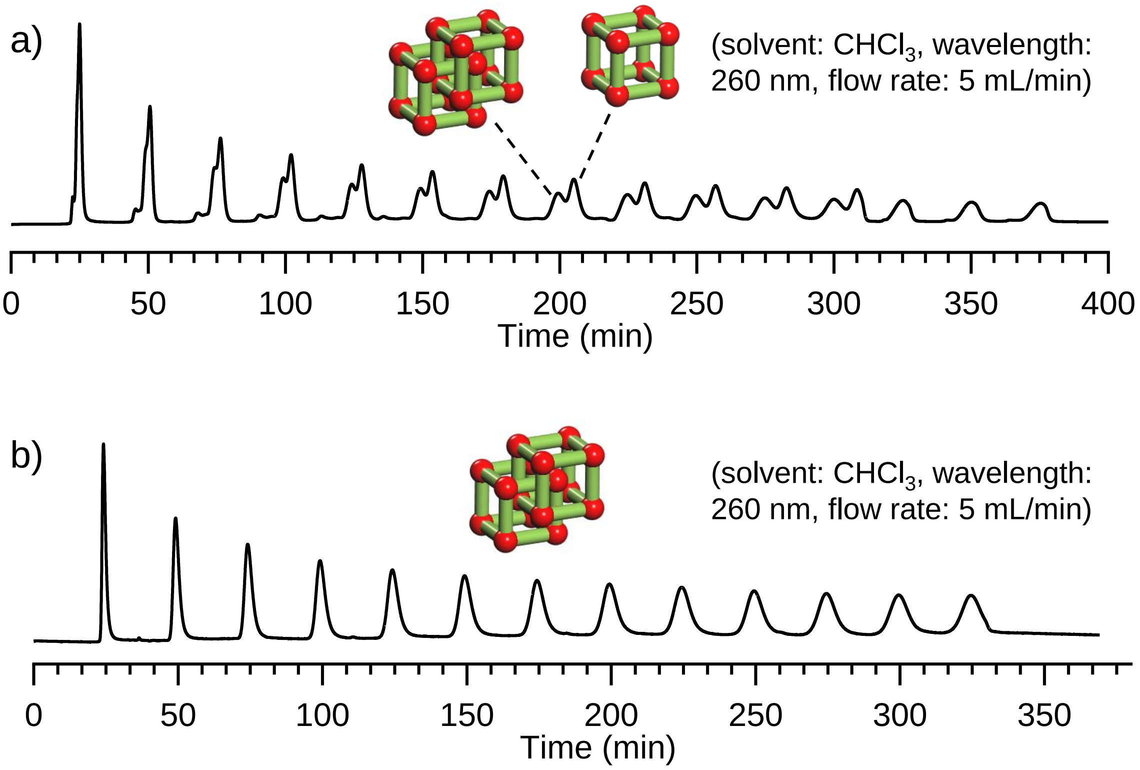

Figure 393. GPC profile of **(OEt-cube)$_2$** a) GPC trace of crude reaction mixture of **(OEt-cube)$_2$** prepared by improved protocol and b) GPC trace of pure **(OEt-cube)$_2$** (solvent: chloroform, flow rate: 5mL/min, Temp: 40˚C, wavelength: 254 nm, GPC columns: 3 x 100 Å (20 x 300 mm)).

(SMe-cube)2: (synthesized by improved protocol)

Sample preparation for GPC: The crude reaction mixture (~17 mg) was first passed through a small pre-packed size exclusion column using chloroform as a mobile phase and then the collected fraction (8 mL) was concentrated to 0.5 mL under reduced pressure and injected into rGPC in one portion. Total 10.7 mg of pure **(SMe-cube)2** was collected.

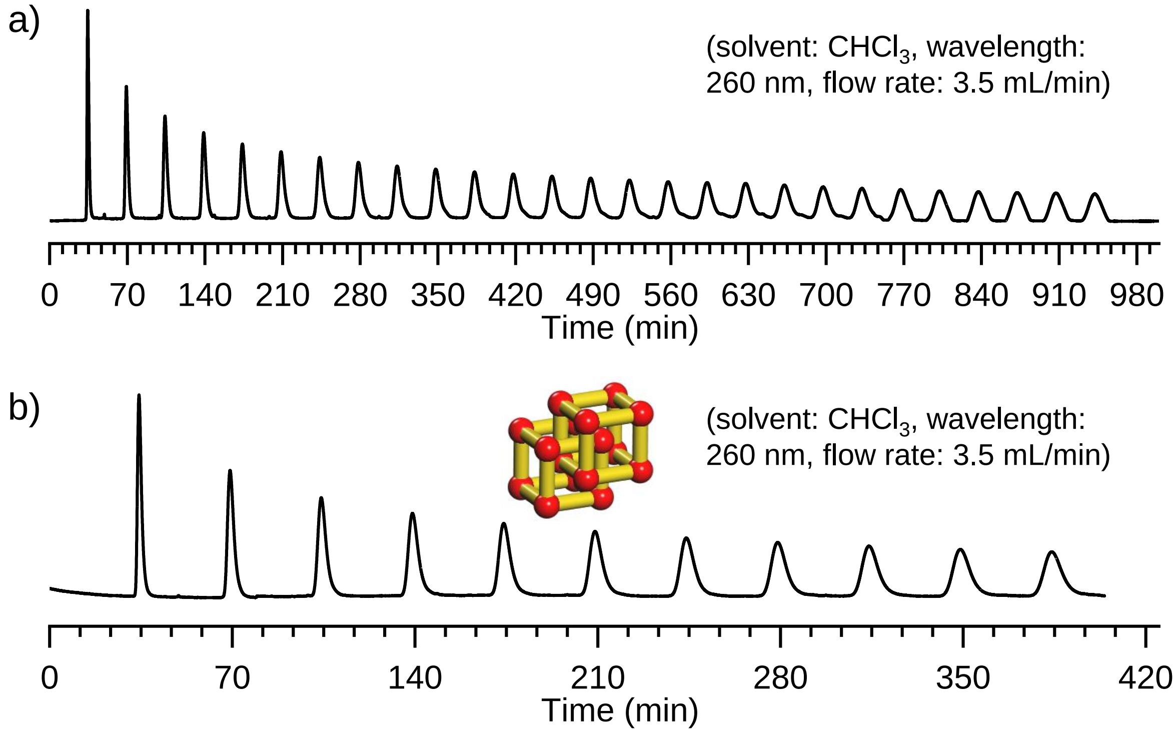

Figure 394. GPC profile of **(SMe-cube)2** a) GPC trace of crude reaction mixture of **(SMe-cube)2** prepared by improved protocol and b) GPC trace of pure **(SMe-cube)2** (solvent: chloroform, flow rate: 3.5mL/min, Temp: 40˚C, wavelength: 260 nm, GPC columns: 3 x 100 Å (20 x 300 mm)).

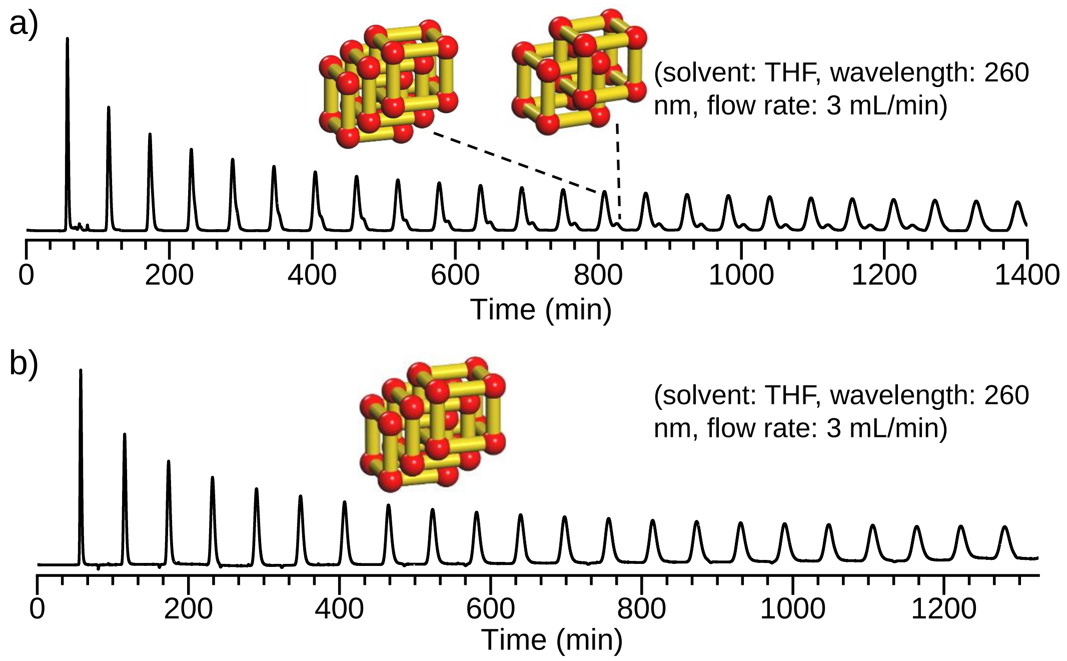

Figure 395. GPC profile of **(SMe-cube)₃**. a) GPC trace of crude reaction mixture of **(SMe-cube)₃** and b) GPC trace of pure **(SMe-cube)₃**. (solvent: THF, flow rate: 3 mL/min, temp: 40˚C, wavelength: 260 nm, GPC columns: (1 x 500 Å (20 x 300 mm) + 3 x 100 Å (20 x 300 mm)).

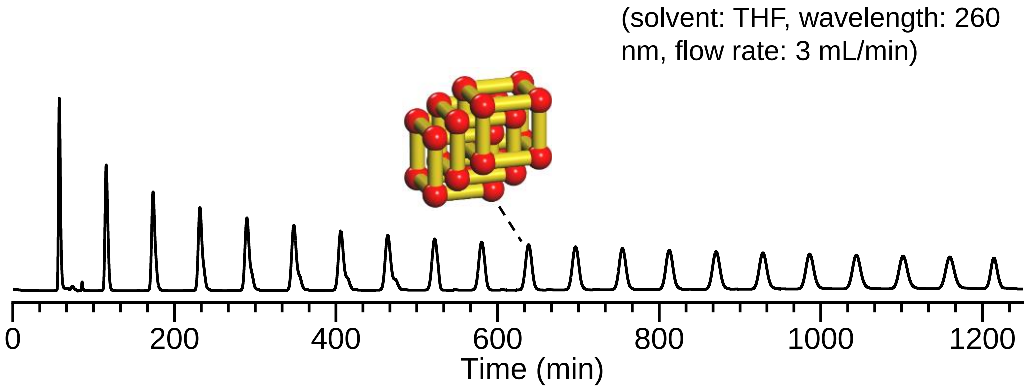

Figure 396. GPC trace of crude reaction mixture of **^{15}N labelled *(SMe-cube)$_3$**. (solvent: THF, flow rate: 3 mL/min, temp: 40˚C, wavelength: 260 nm, GPC columns: (1 x 500 Å (20 x 300 mm) + 3 x 100 Å (20 x 300 mm)).

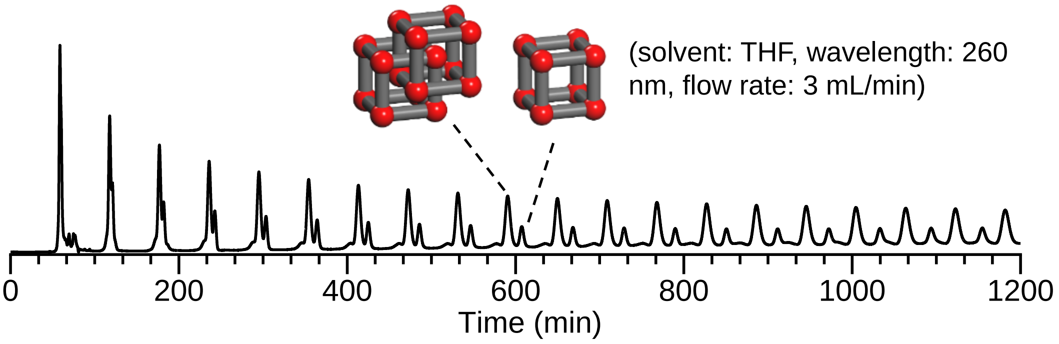

Figure 397. GPC trace of crude reaction mixture of **(mono-OMe-cube)₂**. (solvent: THF, flow rate: 3 mL/min, temp: 40˚C, wavelength: 260 nm, GPC columns: (1 x 500 Å (20 x 300 mm) + 3 x 100 Å (20 x 300 mm)).

9. Imine condensation between TA-TBTQ and dialdehyde linkers at variable concentrations to study the formation of cage /catenanes

General Procedure: To a solution TBTQ **1** (5 mg, 10.7 µmol, 1equiv) and various dialdehyde (16.1 µmol, 1.5 equiv) in $CHCl_3$ of different concentrations (10.7 mM, 21.4 mM and 42.8 mM), a catalytic TFA (0.1 µL, 1.3 µmol, 0.12 equiv) was added and the reaction mixture was stirred at RT. After 3 days, solvent was evaporated and ^1H NMR spectra of the crude reaction mixtures were immediately measured in deuterated dichloromethane (Figure 398-S399) to check the formation of catenane.

2,5-Dihydroxy-terephthaldehyde (2)

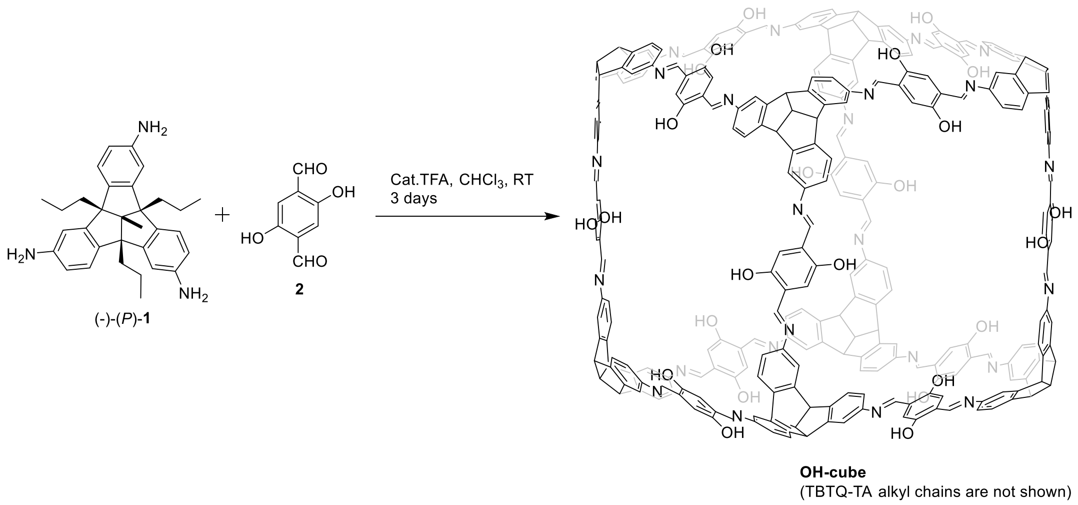

Scheme 1. Imine condensation at different concentrations between TBTQ **1** and 2,5-dihydroxy-terephthaldehyde **2**.

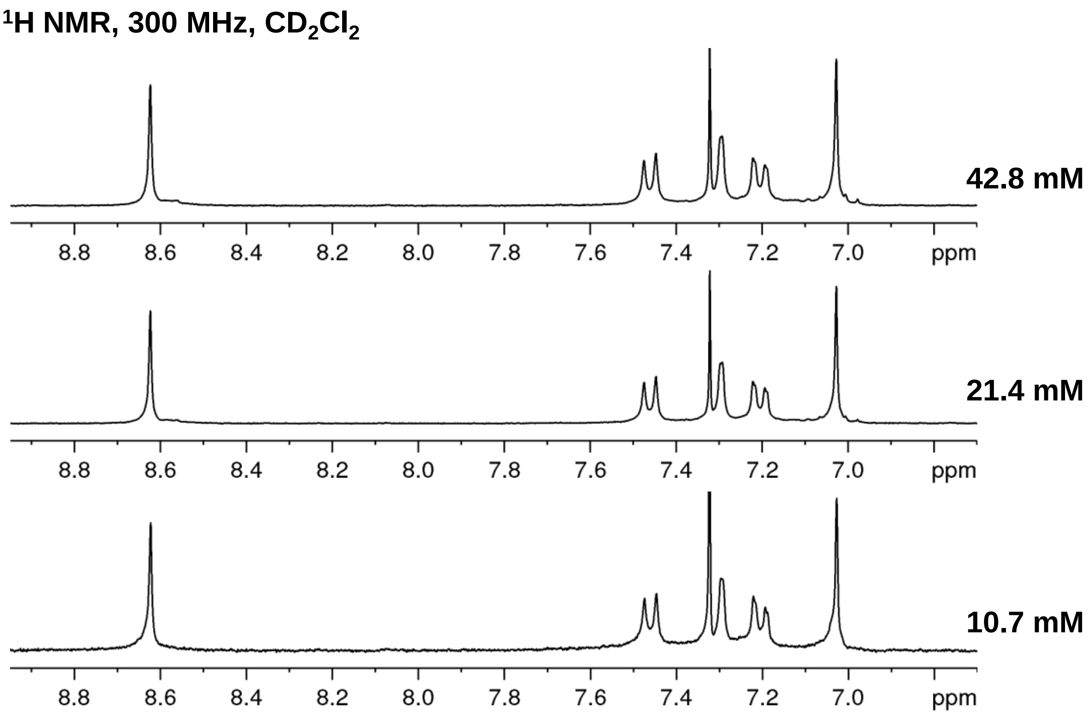

Figure 398. Partial ^{1}H NMR spectra (300 MHz, CD_2Cl_2) of crude reaction mixture of **OH-cube** at 10.7 mM, 21.4 mM and 42.8 mM concentrations.

2,5-Dimethoxy-terephthaldehyde (3)

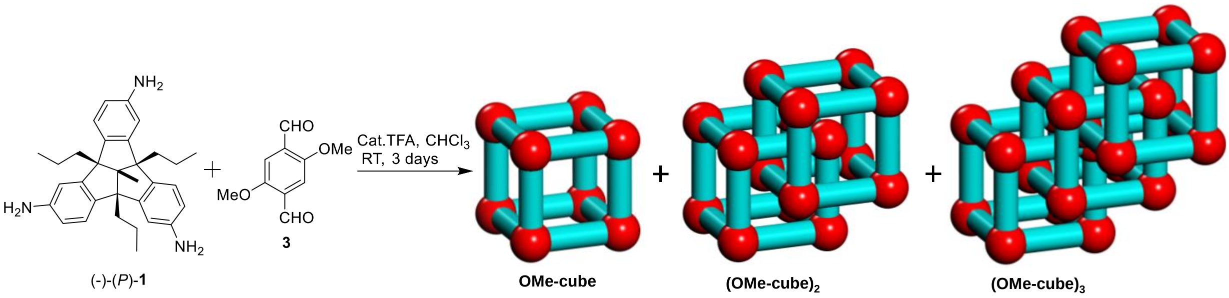

Scheme 2. Imine condensation at different concentrations between TBTQ **1** and 2,5-dimethoxy-terephthaldehyde **3**.

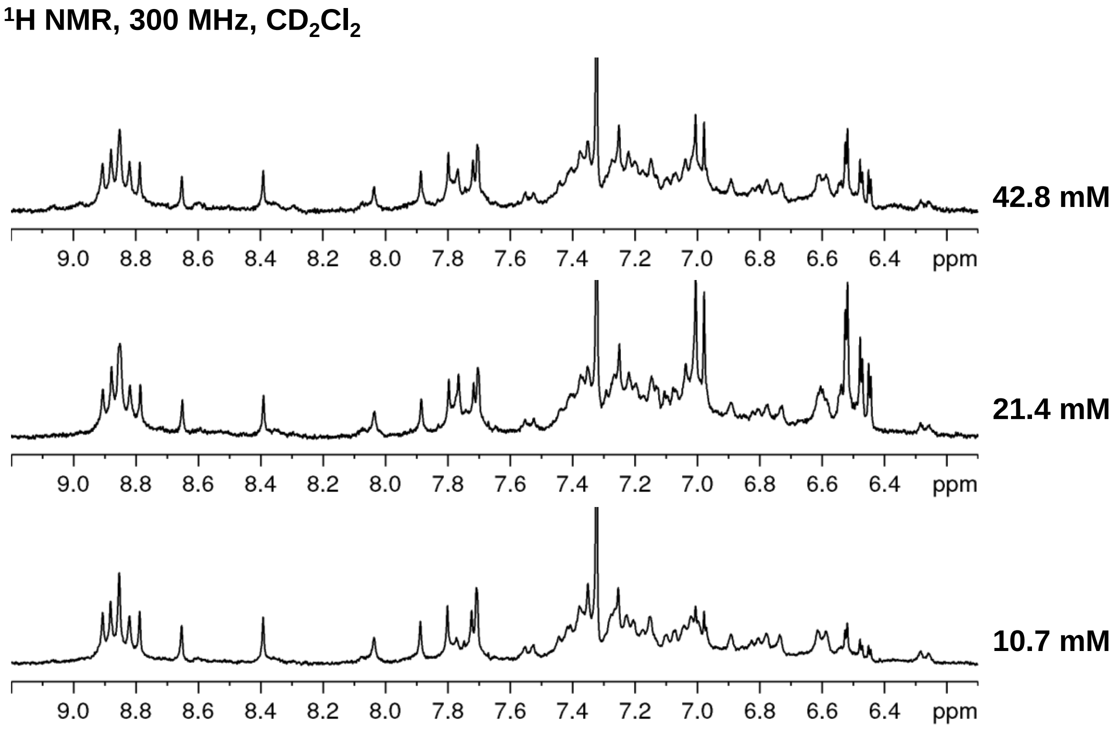

Figure 399. Partial ^{1}H NMR (300 MHz, CD_2Cl_2) spectra of crude reaction mixture of **OMe-cube**, **(OMe-cube)₂** and **(OMe-cube)₃**. at 10.7 mM, 21.4 mM and 42.8 mM concentrations.

Table 1: Synthesis of dimethoxy OMe-cube at different concentrations

Entry	TBTQ 1 (mg)	2,5-dimethoxy-terephthaldehyde (mg)	$CHCl_3$ (mL)	[8+12] cage[a]	[16+24] cage[a]	[24+36] cage[a]
1	5	3.1	0.25	Yes	Yes	Yes
2	5	3.1	0.50	Yes	Yes	Yes
3	5	3.1	1.00	Yes	Yes	Yes
4	5	3.1	2.00	Yes	Yes	No
5	5	3.1	3.00	Yes	Yes	No
6	5	3.1	4.00	Yes	Yes	No
7	5	3.1	6.00	Yes	Yes	No
8	5	3.1	8.00	Yes	Yes	No
9	5	3.1	25.0	Yes	No	No

Reaction time: 24 h, Cat TFA (12 mol%), [a] crude reaction mixture was analyzed by MALDI-TOF mass spectroscopy.

10. Imine condensation between TA-TBTQ and 2,5-dihydroxy dialdehyde linkers at different catalyst concentrations and temperatures to study the formation of cage /catenanes

To a solution TBTQ **1** (5 mg, 10.7 µmol, 1equiv) and 2,5-dihydroxy dialdehyde (2.7 mg, 16.1 µmol, 1.5 equiv) in $CDCl_3$, catalytic TFA was added and the reaction mixture was stirred at RT and at 80 °C After 3 days, ^1H NMR spectra of the crude reaction mixtures were measured (Figure 400) to check the formation of catenane.

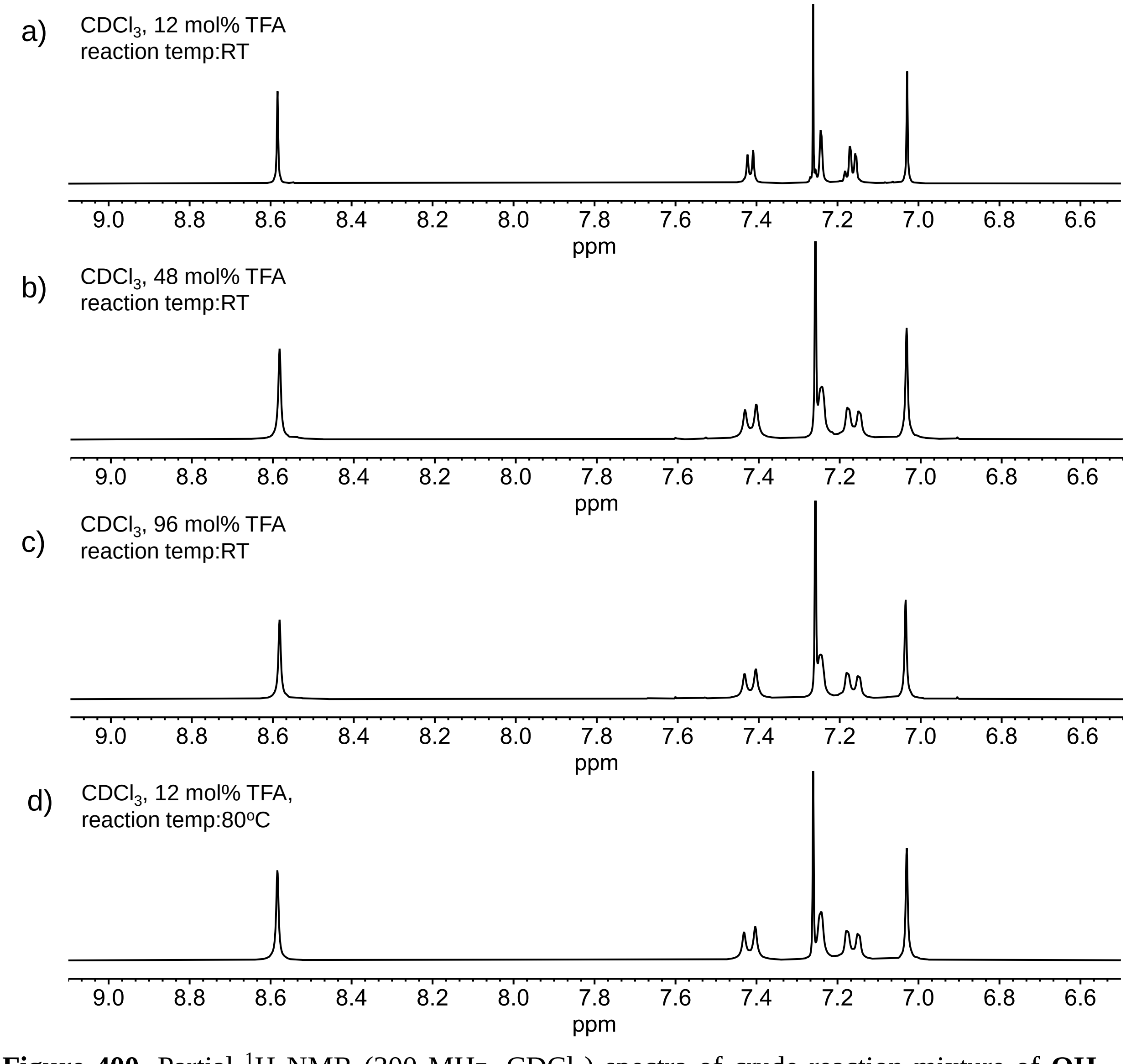

Figure 400. Partial ^1H NMR (300 MHz, $CDCl_3$) spectra of crude reaction mixture of **OH-cube** at diff TFA concentrations (a-c) and at diff. temperatures (a and d).

11. Imine condensation between TA-TBTQ and dialdehyde linkers in different solvents to study the formation of cage /catenanes

General procedure: To a solution of TBTQ **1** (5.6 mg, 12 µmol, 1equiv) and 2,5-substituted terephthaldehyde (16.1 µmol) in $CDCl_3$ or CD_2Cl_2 or THF-d_8 or 1,4 dioxane-d_8 (1 mL), a catalytic amount of TFA (0.1 µL, 1.3 µmol) was added and the reaction mixture was stirred at RT for 3 days. ^{1}H NMR and MALDI-TOF spectra of the crude reaction mixtures were measured after three days (Figure 401-S413) to check the formation of [12+8] cages and its catenanes.

2,5-Dihydroxy-terephthaldehyde (2)

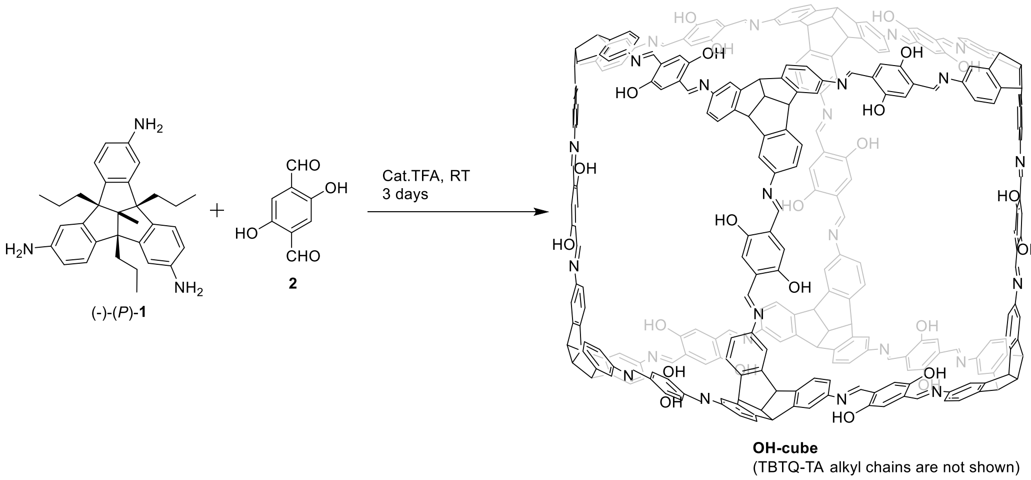

Scheme 3. Imine condensation between TBTQ **1** and 2,5-dihydroxyterephthaldehyde **2** in different solvents.

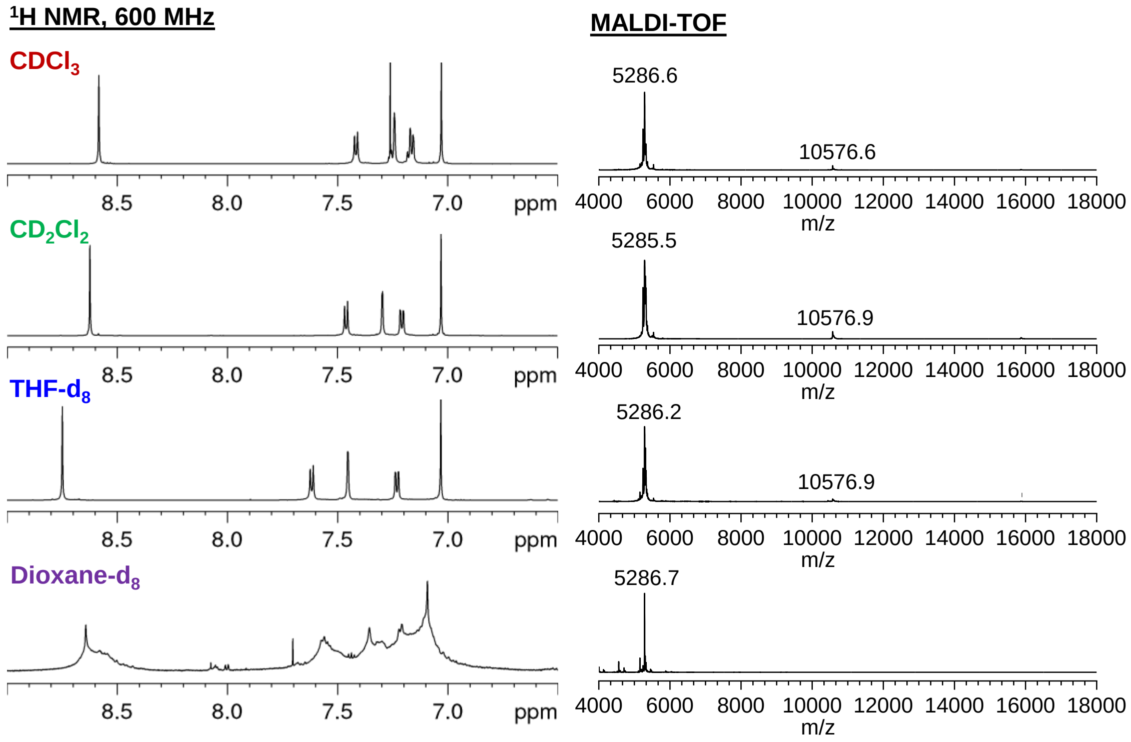

Figure 401. Partial ^{1}H NMR (500 MHz) and MALDI-TOF mass spectra of reaction between TBTQ **1** and 2,5-dihydroxy-terephthaldehyde **2** in different solvents ($CDCl_3$, CD_2Cl_2, THF-d_8 and 1,4-dioxane-d_8) after 3 days of reaction.

Note: *Cage reaction in chloroform solvent was continued for three months and then analyzed by MALDI-TOF MS. The MALDI-TOF spectrum remains unchanged after 3 months for* ***OH-cube****.*

2,5-Dimethoxy-terephthaldehyde (3)

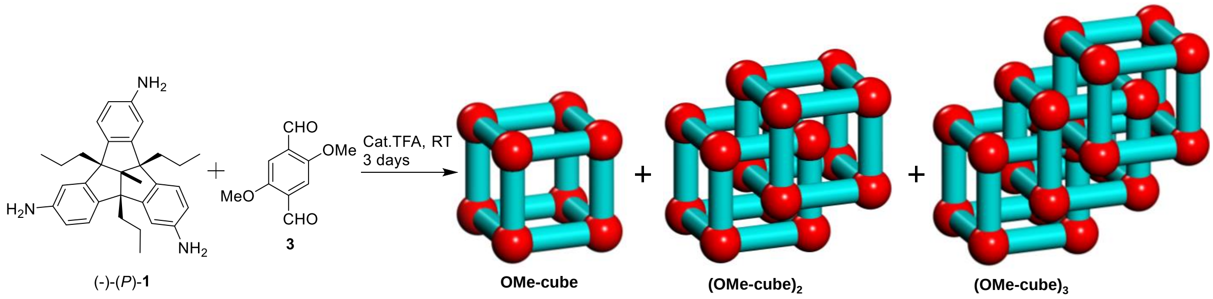

Scheme 4. Imine condensation between TBTQ **1** and 2,5-dimethoxy-terephthaldehyde **3** in different solvents.

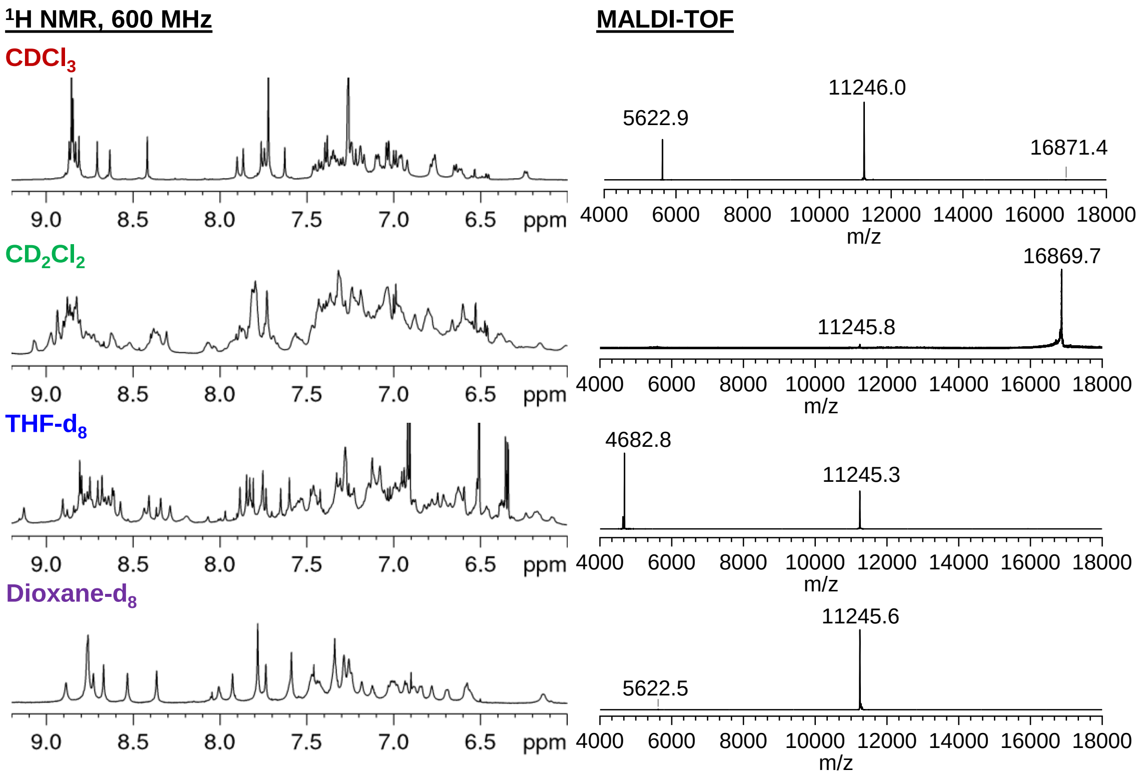

Figure 402. Partial ^{1}H NMR (300 MHz) and MALDI-TOF mass spectra of reaction between TBTQ **1** and 2,5-dimethoxy-terephthaldehyde **3** in different solvents ($CDCl_3$, CD_2Cl_2, THF-d_8 and 1,4-dioxane-d_8) after 3 days of reaction.

2,5-Dimethoxy-terephthaldehyde (3)

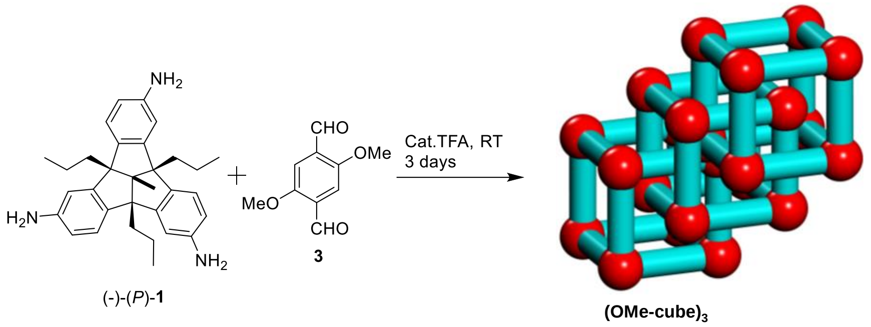

Scheme 5. Imine condensation between TBTQ **1** and 2,5-dimethoxy-terephthaldehyde **3** in mixed solvents (CD_2Cl_2 and CD_3CN).

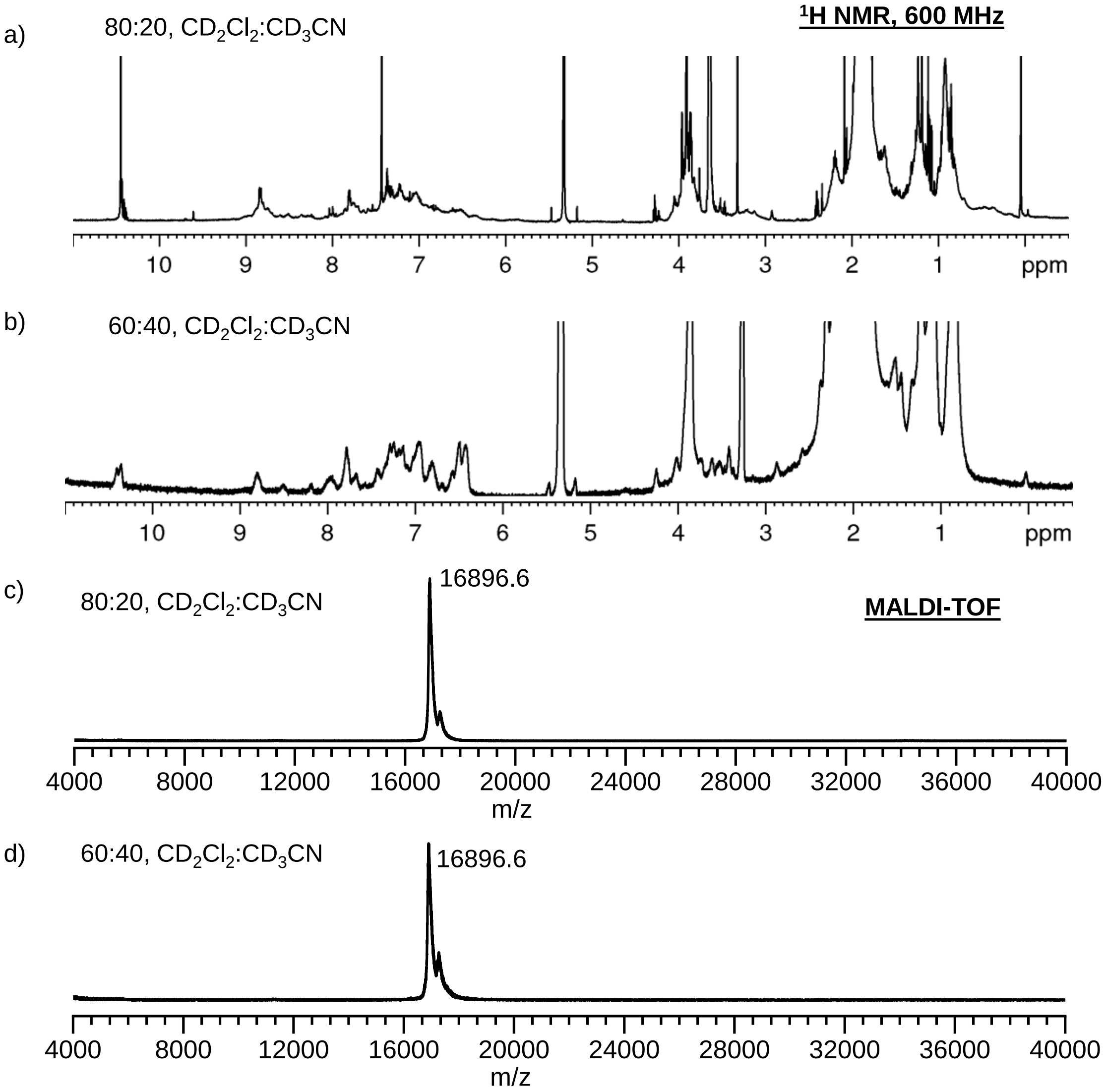

Figure 403. a) and b) ^{1}H NMR (600 MHz) and c) and d) MALDI-TOF mass spectra of reaction between TBTQ **1** and 2,5-dimethoxy-terephthaldehyde **3** in mixed solvents after 3 days of reaction (reaction temp 80 ^oC).

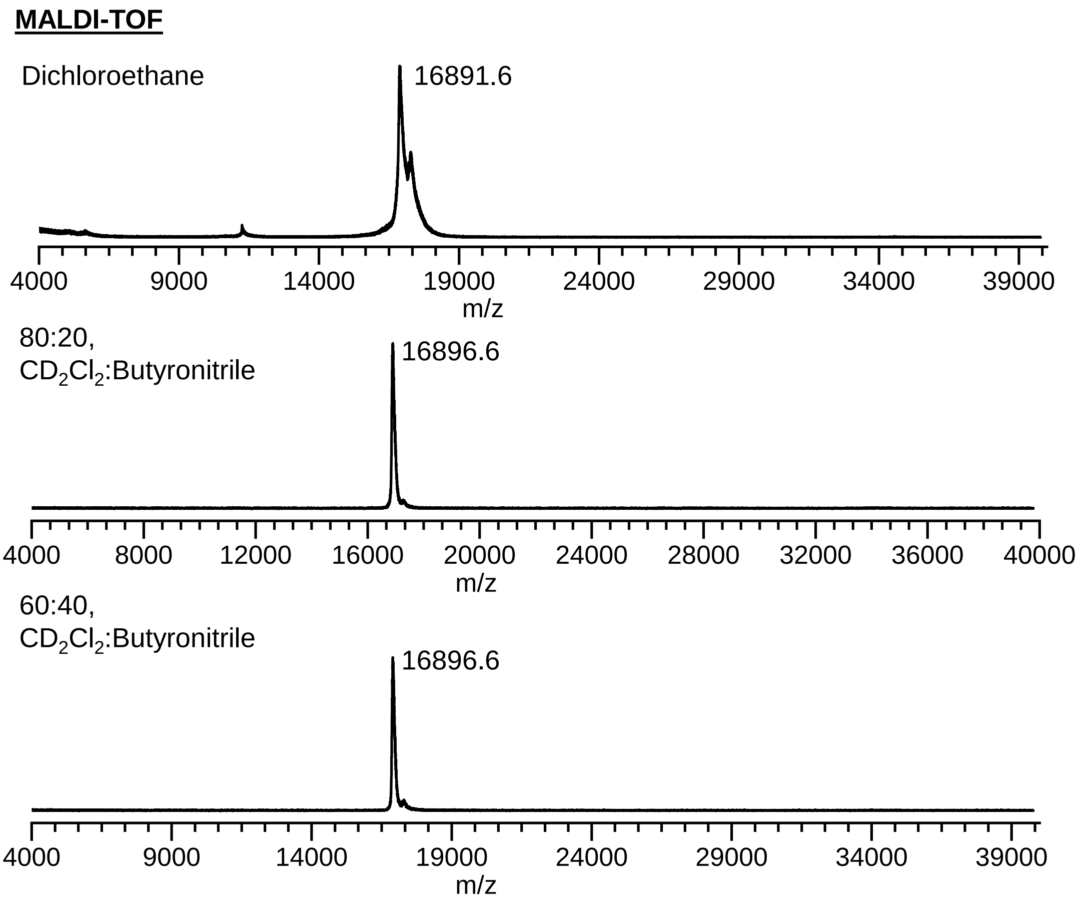

Figure 404. MALDI-TOF (DCTB) mass spectra of reaction between TBTQ **1** and 2,5-dimethoxy-terephthaldehyde **3** in mixed solvents after 3 days of reaction (reaction temp 80 ^{o}C).

Terephthaldehyde (4)

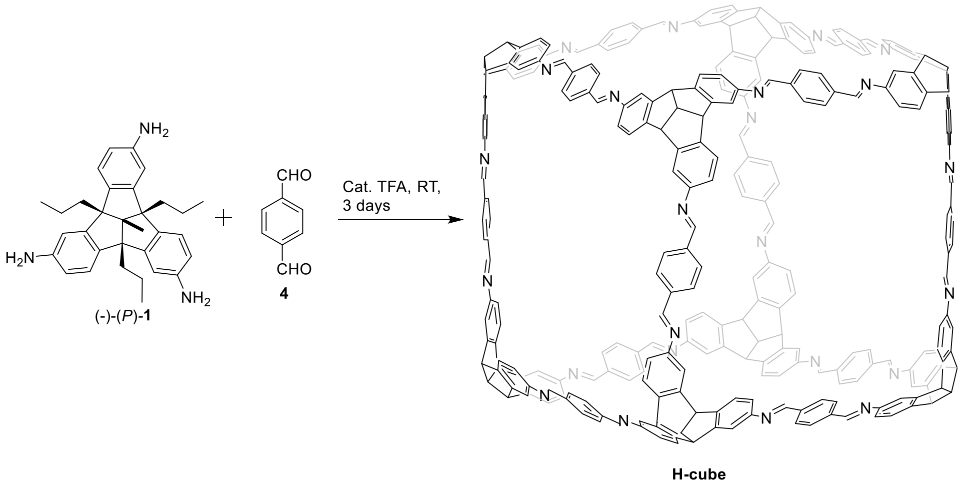

Scheme 6. Imine condensation between TBTQ **1** and terephthaldehyde **4** in different solvents.

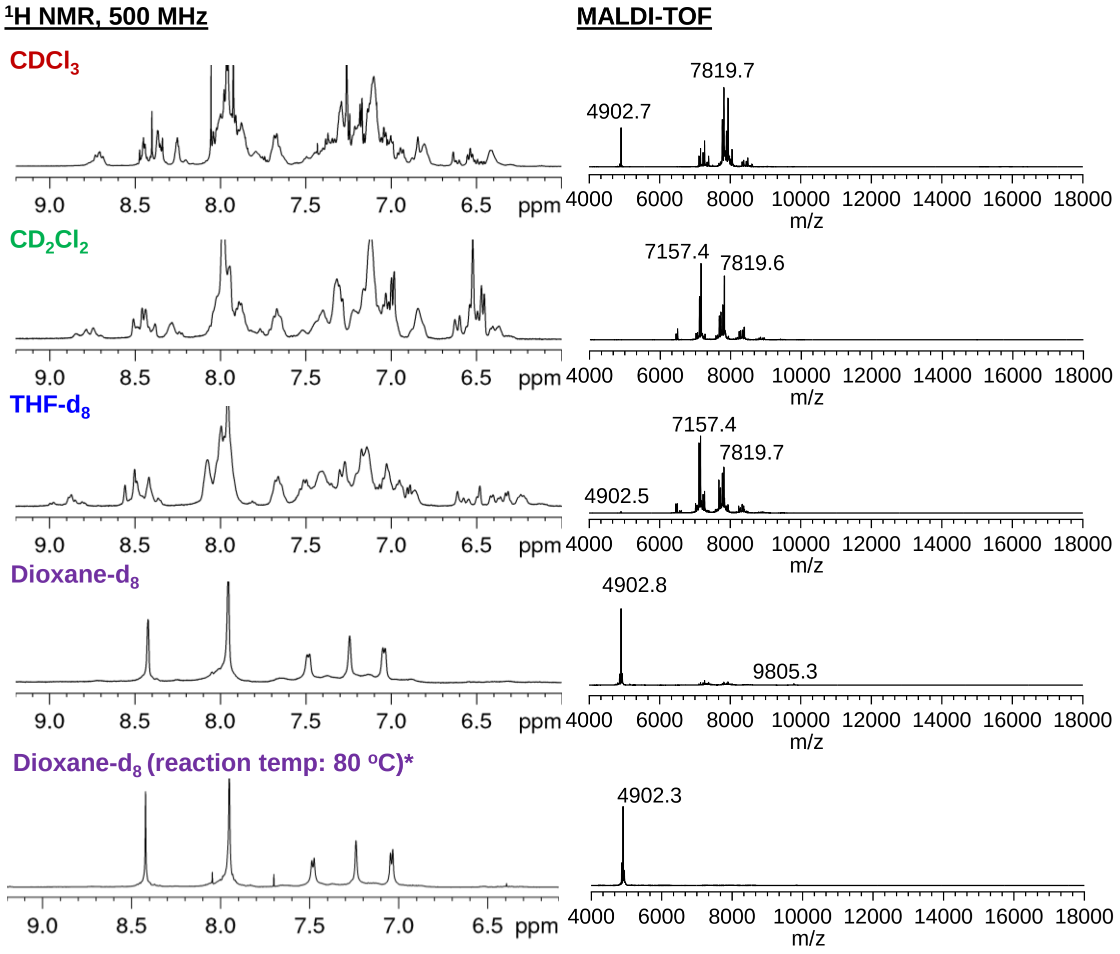

Figure 405. Partial ^{1}H NMR (500 MHz) and MALDI-TOF mass spectra of reaction between –TBTQ **1** and terephthaldehyde **4** in different solvents ($CDCl_3$, CD_2Cl_2, THF-d_8 and 1,4-dioxane-d_8) after 3 days of reaction. *reaction temp 80 °C.

2,5-Dimethyl-terephthaldehyde (5)

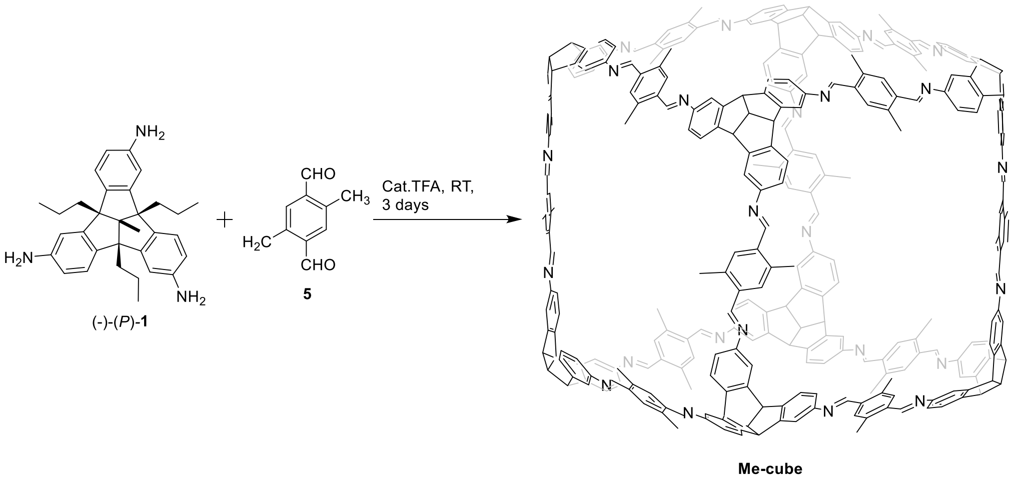

Scheme 7. Imine condensation between TBTQ **1** and 2,5-dimethyl-terephthaldehyde **5** in different solvents.

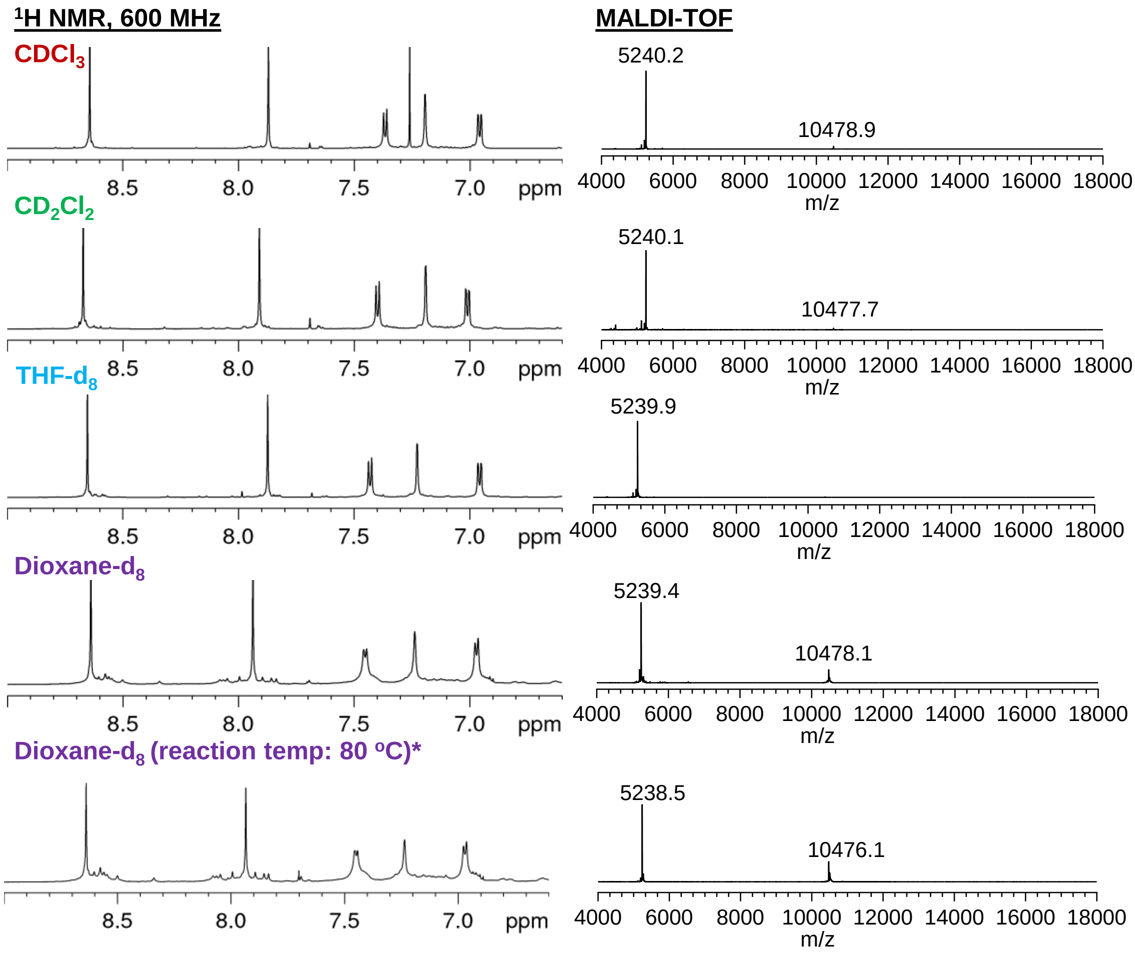

Figure 406. Partial ^{1}H NMR (600 MHz) and MALDI-TOF mass spectra of reaction between TA-TBTQ and 2,5-dimethyl-terephthaldehyde in different solvents ($CDCl_3$, CD_2Cl_2, THF-d_8 and 1,4-dioxane-d_8) after 3 days of reaction.*reaction temp 80 ^{o}C.

2,5-Diethyl-terephthaldehyde (6)

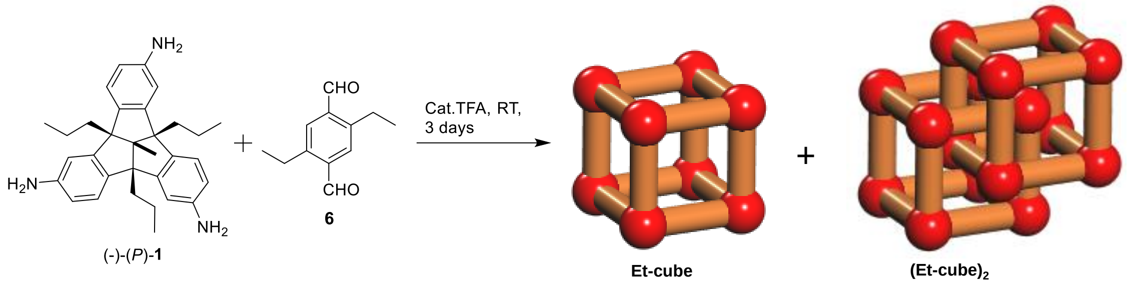

Scheme 8. Imine condensation between TBTQ **1** and 2,5-diethyl-terephthaldehyde **6** in different solvents.

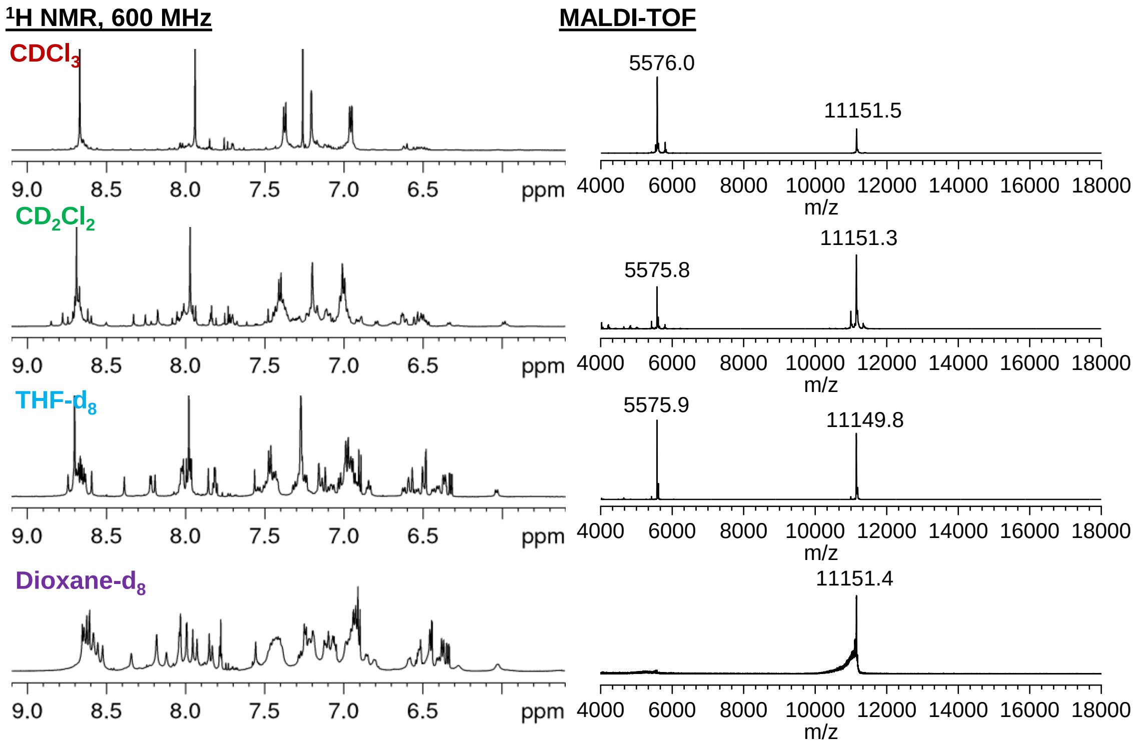

Figure 407. Partial ^{1}H NMR (600 MHz) and MALDI-TOF mass spectra of reaction between TBTQ **1** and 2,5-diethyl-terephthaldehyde **6** in different solvents ($CDCl_3$, CD_2Cl_2, THF-d_8 and 1,4-dioxane-d_8) after 3 days of reaction.

2,5-Diethoxy-terephthaldehyde (7)

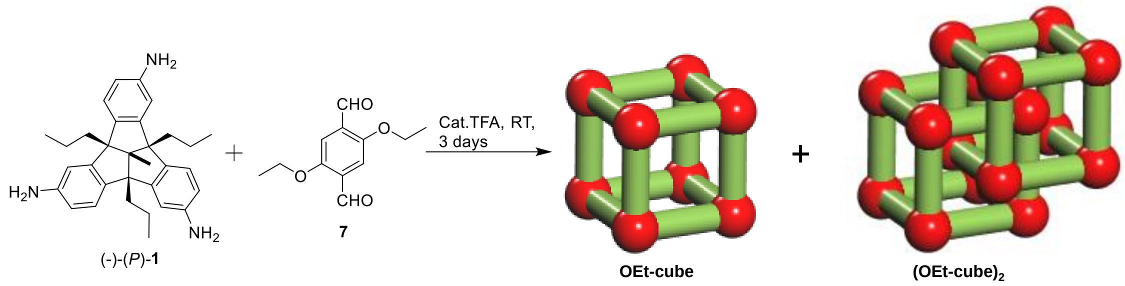

Scheme 9. Imine condensation between TBTQ **1** and 2,5-diethoxy-terephthaldehyde **7** in different solvents.

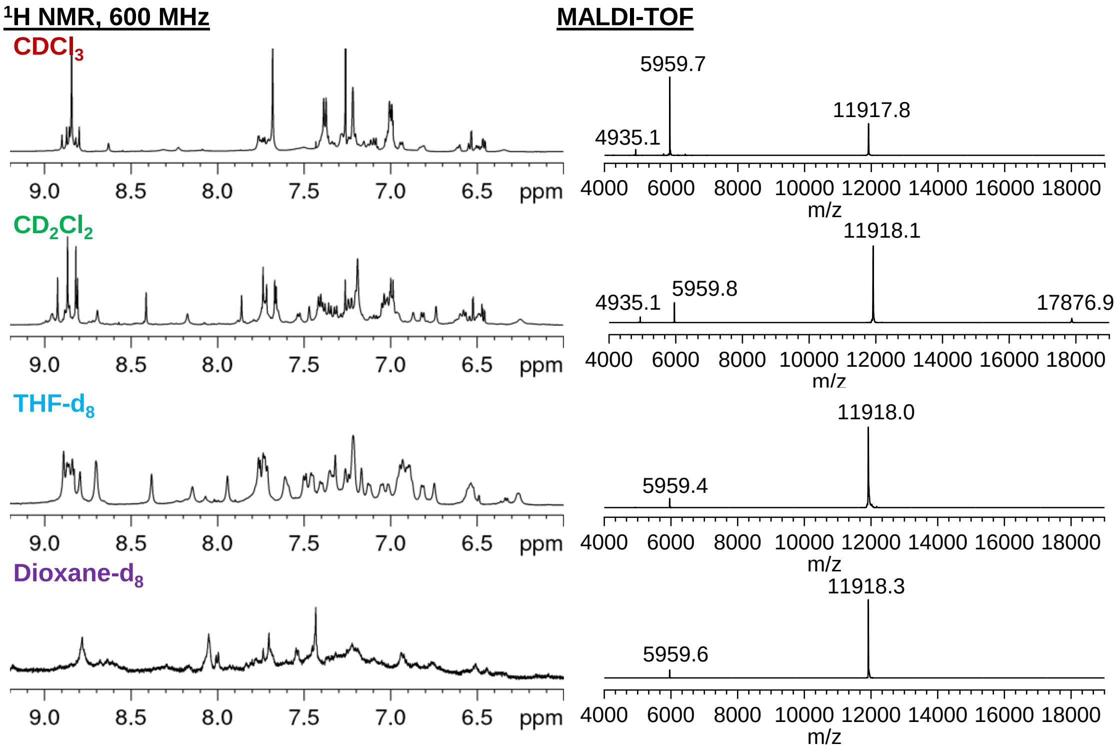

Figure 408. Partial ^{1}H NMR (600 MHz) and MALDI-TOF mass spectra of reaction between TBTQ **1** and 2,5-diethoxy-terephthaldehyde **7** in different solvents ($CDCl_3$, CD_2Cl_2, THF-d_8 and 1,4-dioxane-d_8) after 3 days of reaction.

2,5-Diisopropoxy-terephthaldehyde (8)

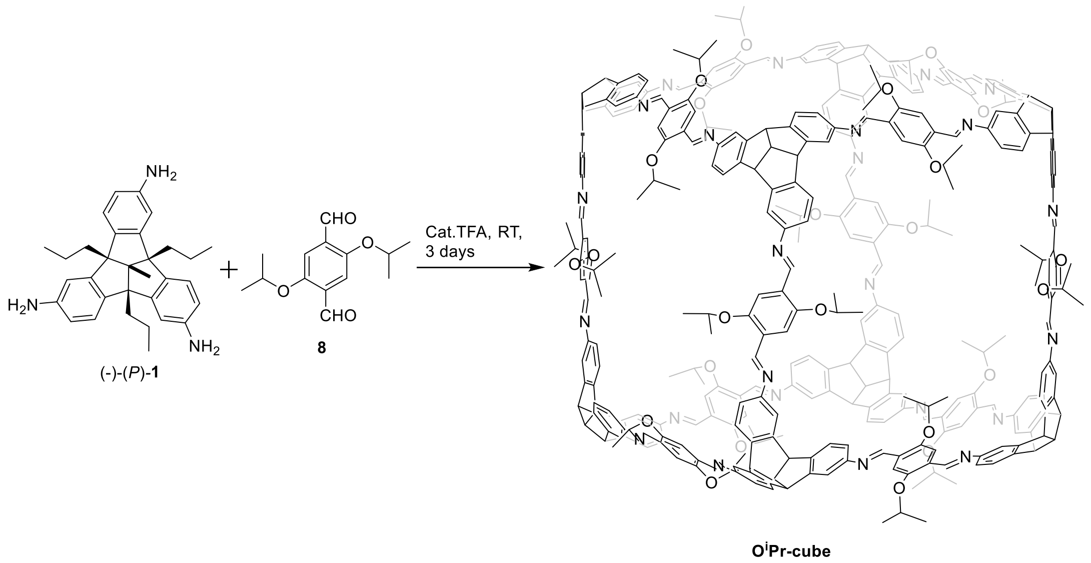

Scheme 10. Imine condensation between TBTQ **1** and 2,5-diisopropoxy-terephthaldehyde **8** in different solvents.

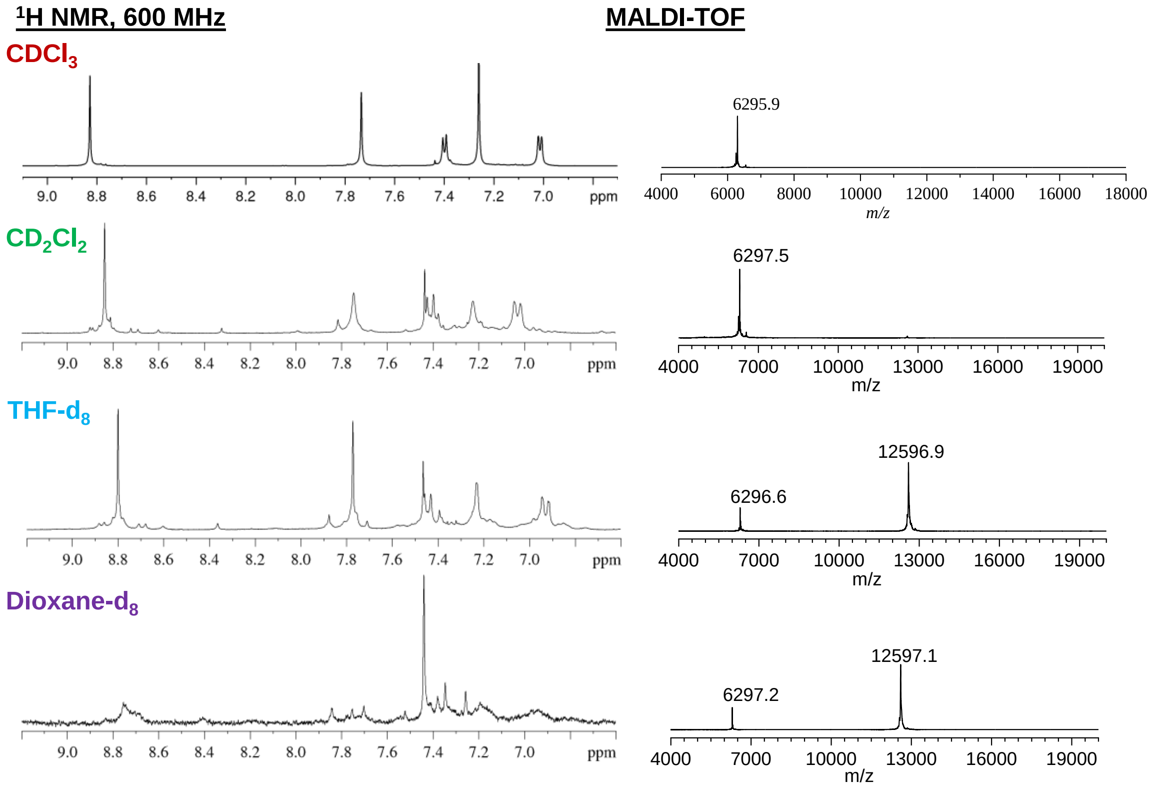

Figure 409. Partial ^{1}H NMR (600 MHz) and MALDI-TOF mass spectra of reaction between TBTQ **1** and 2,5-diisopropoxy-terephthaldehyde **8** in different solvents ($CDCl_3$, CD_2Cl_2, THF-d_8 and 1,4-dioxane-d_8) after 3 days of reaction.

2,5-Bis(methylthio)terephthaldehyde (9)

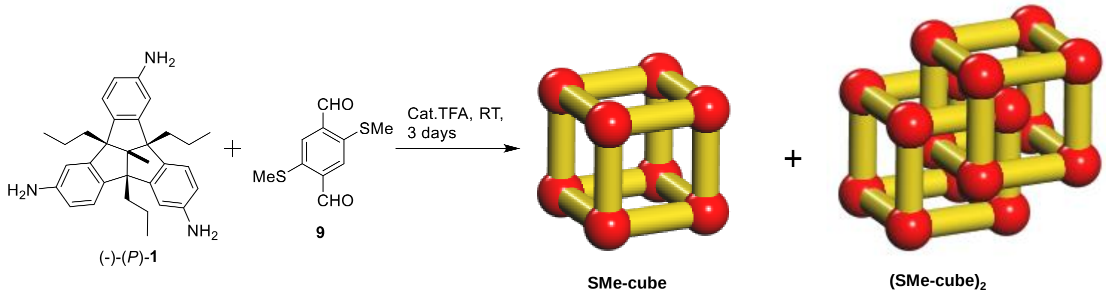

Scheme 11. Imine condensation between TBTQ **1** and 2,5-bis(methylthio)terephthaldehyde **9** in different solvents.

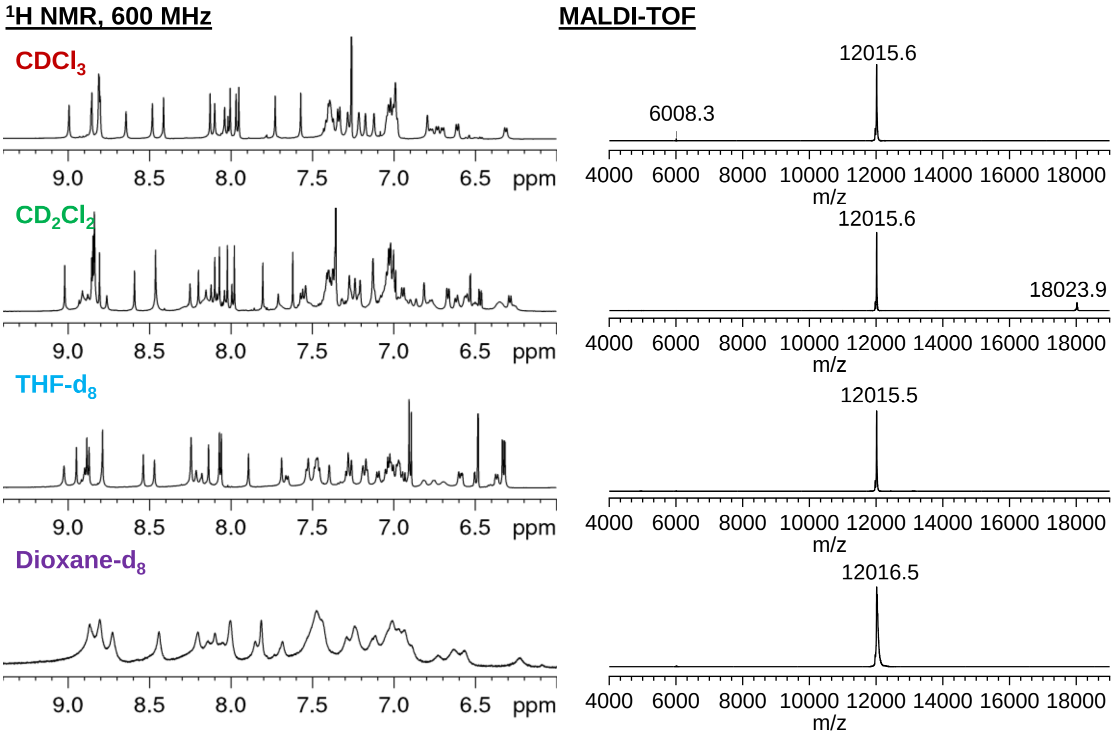

Figure 410. Partial ^{1}H NMR (600 MHz) and MALDI-TOF mass spectra of reaction between TBTQ **1** and 2,5-bis(methylthio)terephthaldehyde **9** in different solvents ($CDCl_3$, CD_2Cl_2, THF-d_8 and 1,4-dioxane-d_8) after 3 days of reaction.

2,5-bis(*tert*-methylthio)terephthaldehyde (10)

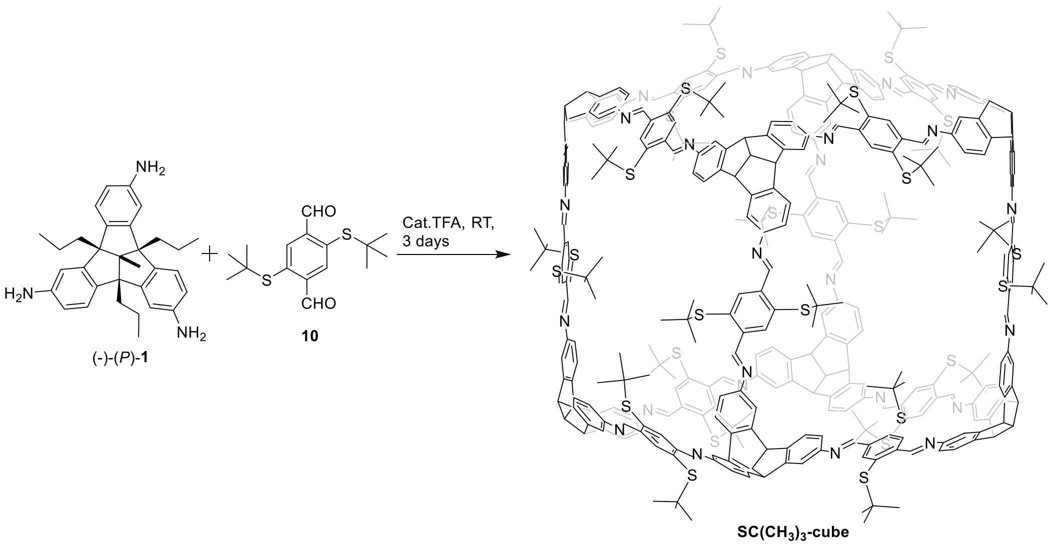

Scheme 12. Imine condensation between TBTQ **1** and 2,5-bis(*tert*-methylthio)terephthaldehyde **10** in different solvents.

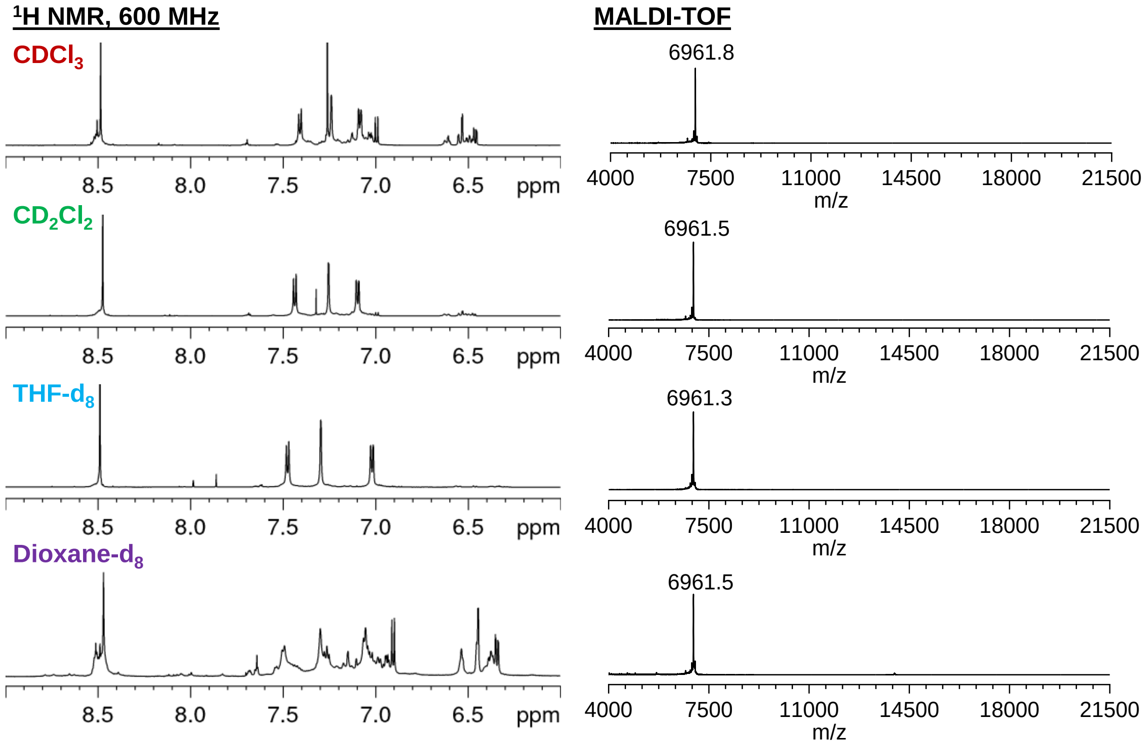

Figure 411. Partial ^{1}H NMR (600 MHz) and MALDI-TOF mass spectra reaction between TBTQ **1** and 2,5-bis(*tert*-methylthio)terephthaldehyde **10** in different solvents ($CDCl_3$, CD_2Cl_2, THF-d_8 and 1,4-dioxane-d_8) after 3 days of reaction.

2,5-Dibromo-terephthaldehyde (11)

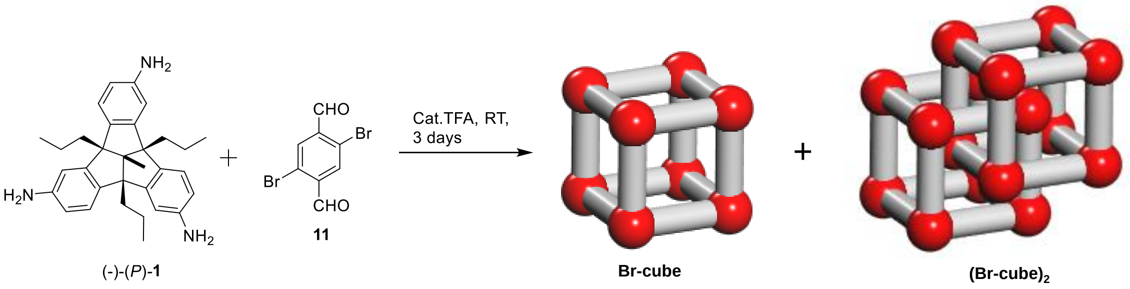

Scheme 13. Imine condensation between TBTQ 1 and 2,5-dibromo-terephthaldehyde **11** in different solvents.

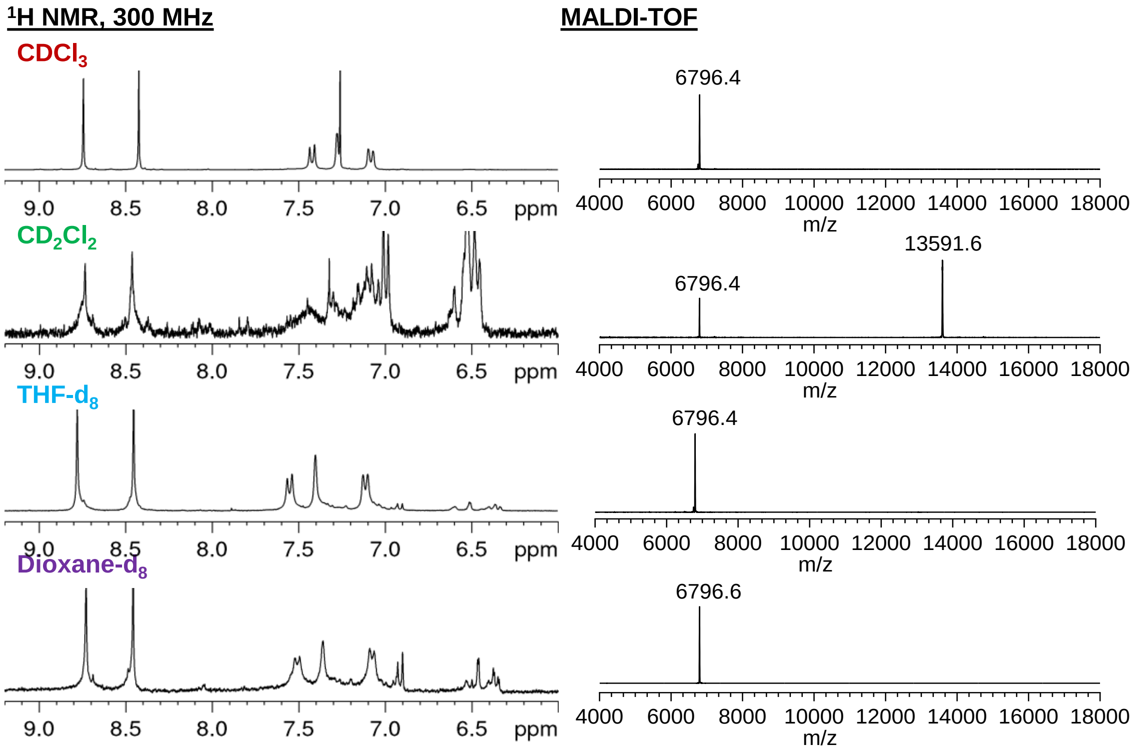

Figure 412. Partial ^{1}H NMR (300 MHz) and MALDI-TOF mass spectra of reaction between TBTQ **1** and 2,5-dibromo-terephthaldehyde **11** in different solvents ($CDCl_3$, CD_2Cl_2, THF-d_8 and 1,4-dioxane-d_8) after 3 days of reaction.

2-methoxyterephthaldehyde (12)

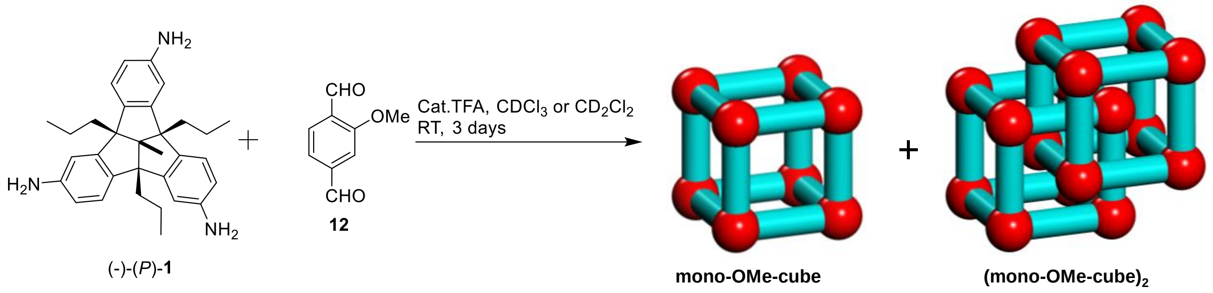

Scheme 14. Imine condensation between TBTQ **1** and 2,5-dimethoxy-terephthaldehyde **12** in different solvents.

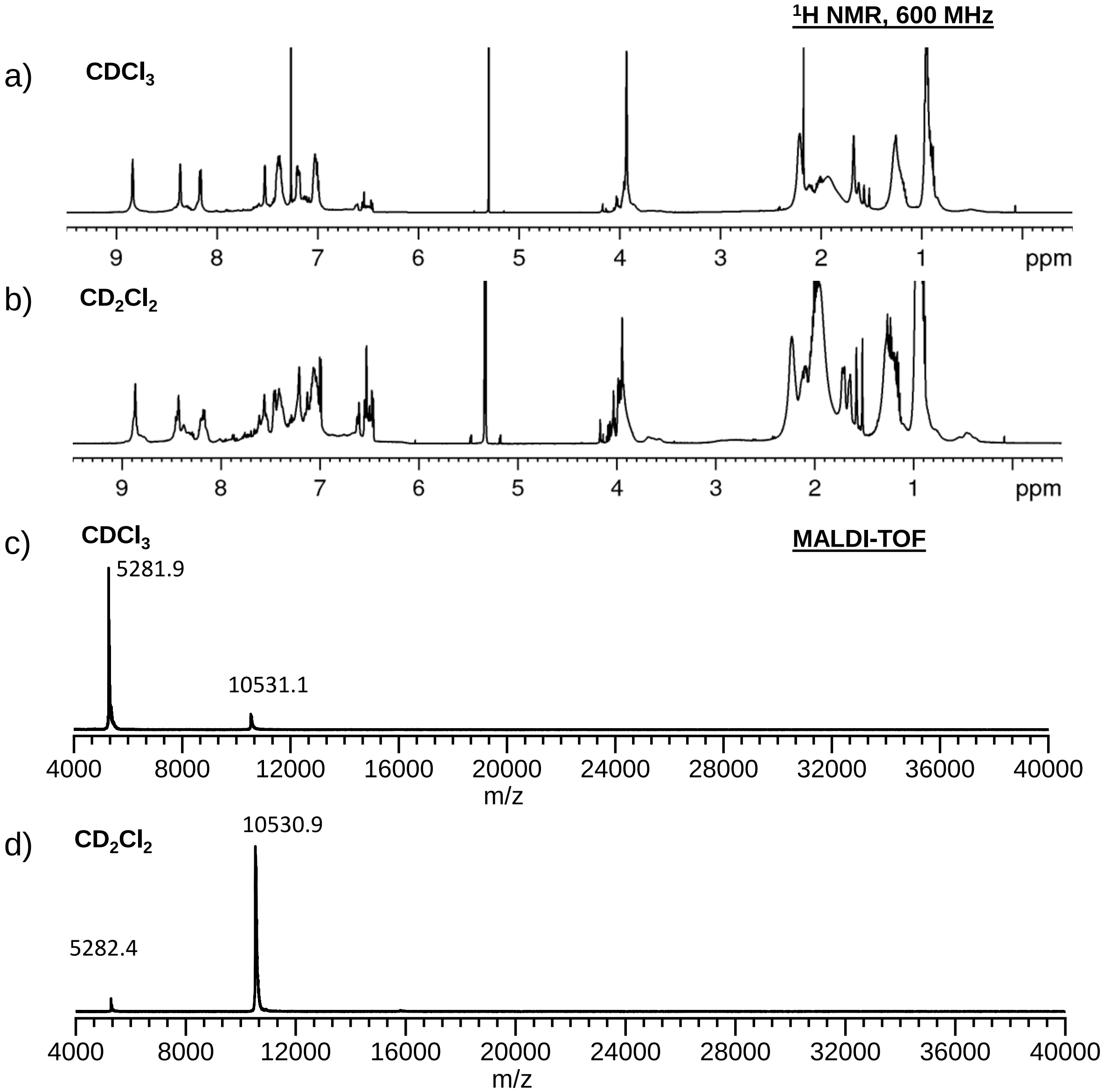

Figure 413. a) and b) ^{1}H NMR (600 MHz) and c) and d) MALDI-TOF (DCTB) mass spectra of reaction between TBTQ **1** and 2-methoxyterephthaldehyde **12** in $CDCl_3$ and CD_2Cl_2 after 3 days of reaction.

12. Thermodynamic data

a) Calculation of ΔG by variable concentration measurements

General procedure: To a solution of TBTQ **1** (5 mg, 10.7 μmol, 1equiv) and 2,5-substituted terephthaldehydes (1.5 equiv) in $CDCl_3$, a catalytic amount of TFA (0.1 μL, 1.3 μmol) was added and the reaction mixture was stirred at RT at different concentrations (0.43 mM, 0.71 mM, 1.07 mM, 2.14 mM and 10.7 mM (concentration is with respect to TA-TBTQ). After three days, ^{1}H NMR spectra (600 MHz, RT) were measured.

Equations used to calculate the K_{eq} and ΔG

$$K_{eq} = [\text{Dicatenane}]/[\text{Cage}]^2$$

where: K_{eq} is the equilibrium constant

[Catenane] is the concentration of $[12+8]_2$ dicatenane (M)

[Cage] is the concentration of [12+8] monomeric cage (M)

$$\Delta G = -RT \ln K_{eq}$$

ΔG is the Gibbs free energy ($kJmol^{-1}$)

R is the universal gas constant ($Jmol^{-1}K^{-1}$)

T is the temperature (K)

- **Calculation of cage to dicatenane ratio from ^{1}H NMR spectrum**

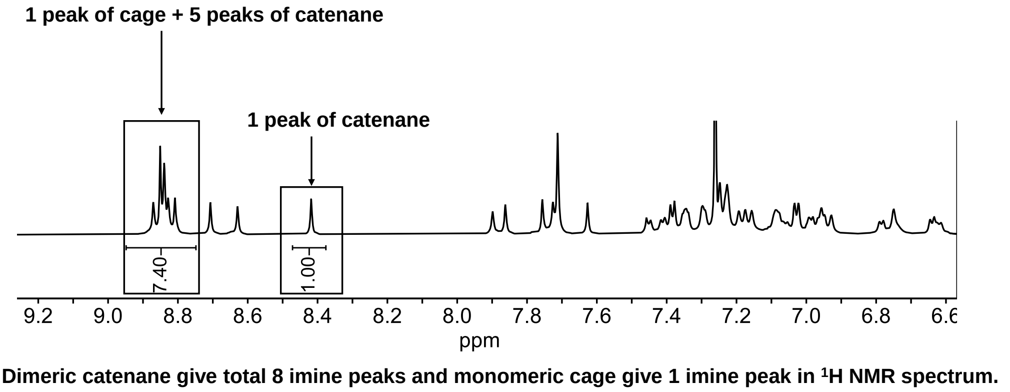

Dimeric catenane give total 8 imine peaks and monomeric cage give 1 imine peak in ^{1}H NMR spectrum.

Integration of cage = (cage+dicatenane peak)-5

Integration of cage = (7.40)-5 = 2.40

cage:dicatenane ratio is 2.40:1

Cage has 24 protons per imine signals and dicatenane has 6 imine protons per imine signals.

Cage:dicatenane ratio is (2.40/24):(1/6) = 0.100:0.167

cage:dicatenane ratio is (0.100/0.100):(0.167/0.100) = 1.00:1.67

a) OMe-cube→ (OMe-cube)

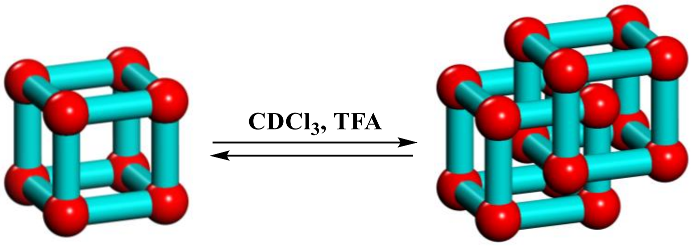

OMe-cube (OMe-cube)$_2$

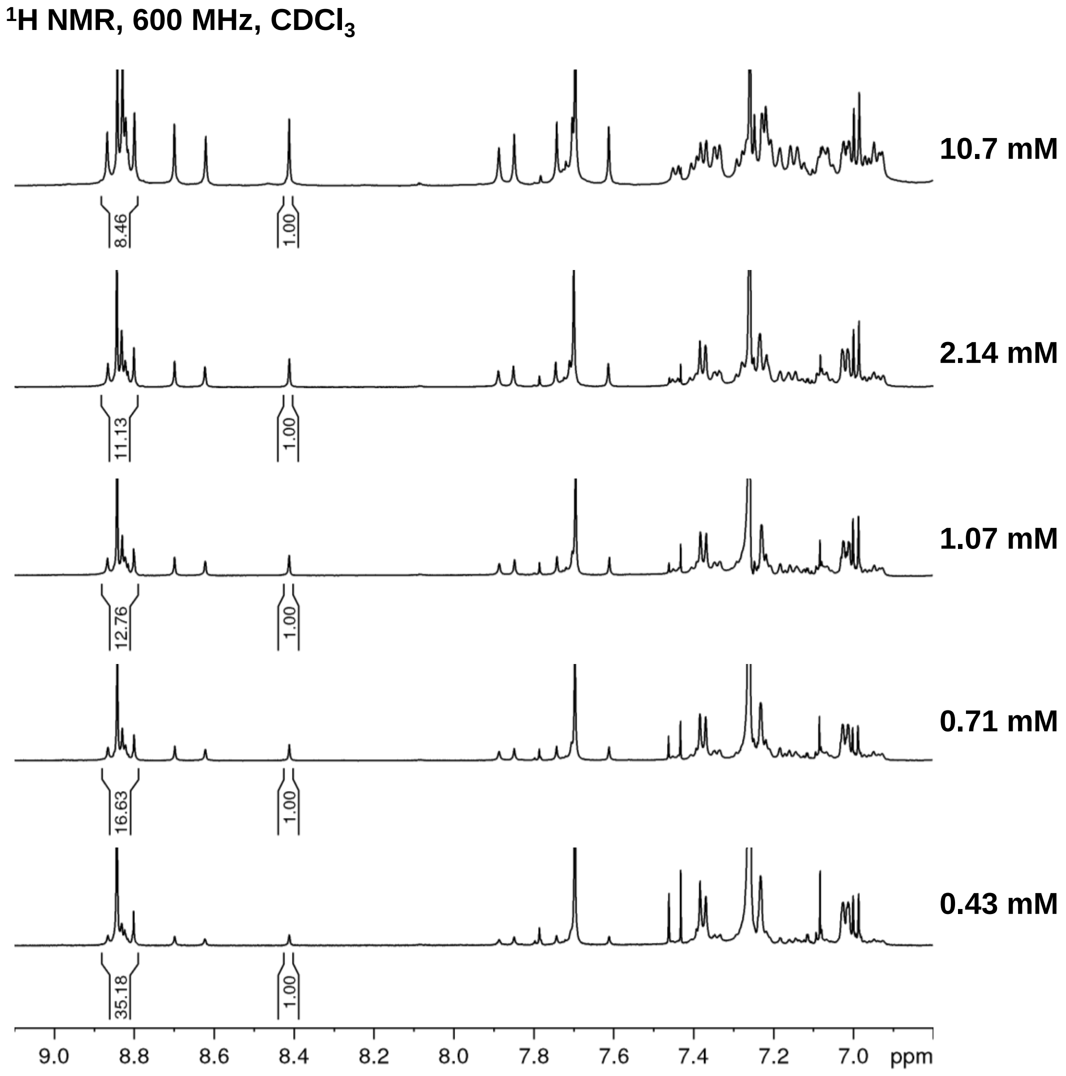

Figure 414. Partial ^{1}H NMR spectra of the reaction **(OMe-cube)→(OMe-cube)$_2$** at various concentrations under thermodynamic equilibrium.

Table 3. Calculation of equilibrium constant and ΔG for the reaction between OMe-cube→ (OMe-cube)$_2$.

Entry	Conc (mM)	T (K)	Cage:Catenane	K_{eq} (M^{-1})	lnK_{eq}	ΔG ($kJmol^{-1}$)
1	10.7	295	1:1.16	2851.75	7.955	**-19.51**
2	2.14	295	1:0.65	5601.06	8.630	**-21.17**
3	1.07	295	1:0.52	7796.71	8.961	**-21.98**
4	0.71	295	1:0.34	6485.31	8.777	**-21.53**
5	0.43	295	1:0.13	3121.88	8.046	**-19.73**

b) OEt-cube → (OEt-cube)$_2$

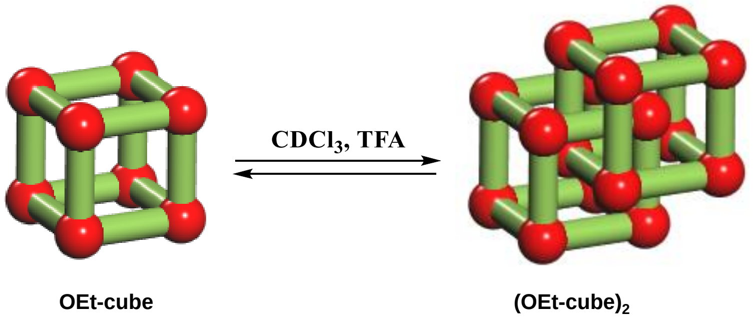

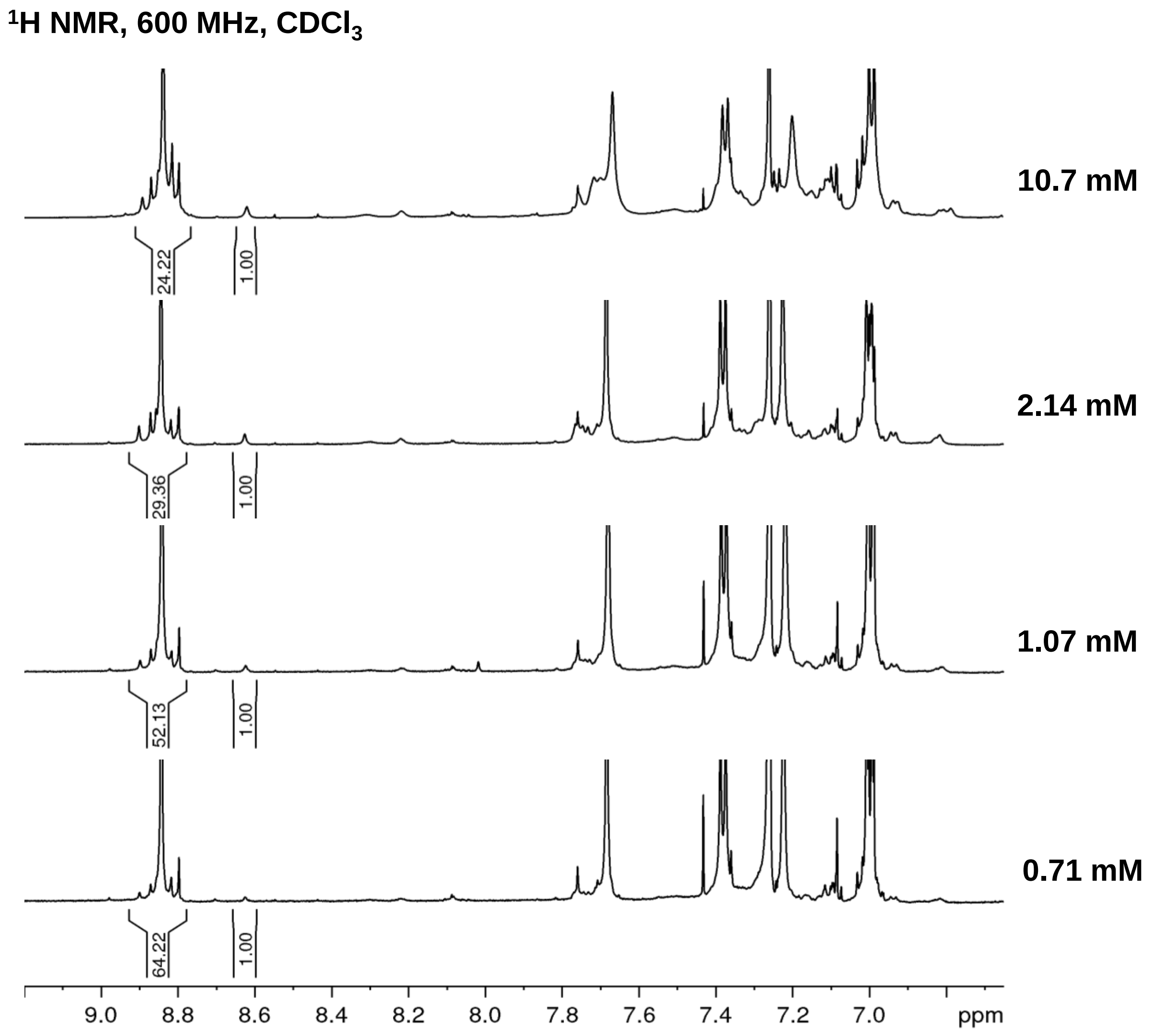

Figure 415. Partial ^{1}H NMR spectra of the reaction **(OEt-cube)→(OEt-cube)$_2$** at various concentrations under thermodynamic equilibrium.

Table 4. Calculation of equilibrium constant and ∆G for the reaction OEt-cube → (OEt-cube)$_2$

Entry	Conc (mM)	T (K)	Cage: Catenane	K_{eq} (M^{-1})	lnK_{eq}	∆G (kJmol^{-1})
1	10.7	295	1:0.22	235.64	5.462	**-13.40**
2	2.14	295	1:0.17	857.26	6.753	**-16.56**
3	1.07	295	1:0.09	758.89	6.631	**-16.27**
4	0.71	295	1:0.07	874.28	6.773	**-16.61**

c) SMe-cube →(SMe-cube)2

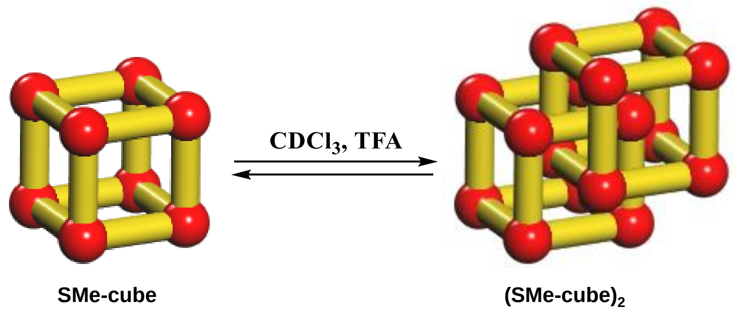

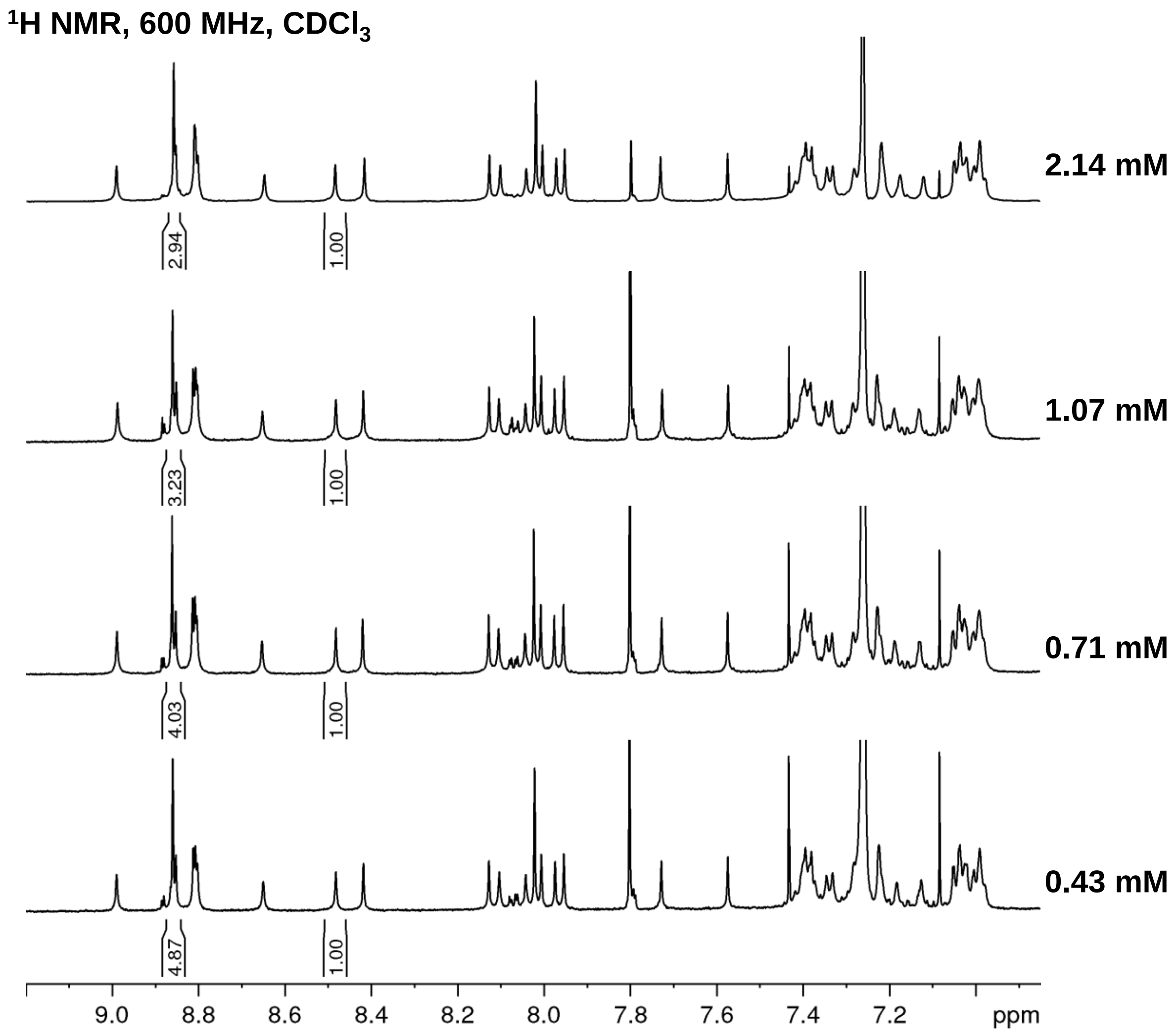

Figure 416. Partial ^{1}H NMR spectra of reaction **(SMe-cube) →(SMe-cube)2** at various concentrations under thermodynamic equilibrium.

Table 5. Calculation of equilibrium constant and ΔG for the reaction SMe-cube →(SMe-cube)$_2$

Entry	Conc (mM)	T (K)	Cage:Catenane	K_{eq} (M^{-1})	lnK_{eq}	ΔG (kJmol^{-1})
2	2.14	295	1:2.06	39376.57	10.581	**-25.95**
3	1.07	295	1:1.79	61341.00	11.024	**-27.04**
4	0.71	295	1:1.32	53736.14	10.892	**-26.71**
5	0.43	295	1:1.03	59081.81	10.986	**-26.95**

b) van't Hoff plots

(OMe-cube) → (OMe-cube)$_2$.

Procedure: A vial 'A' was charged with **OMe-cube** (1.9 mg, 0.34 µmol) and deuterated $CHCl_3$ (0.5 mL). Catalytic TFA (5 µL of a 65.3 mM solution in $CDCl_3$, 0.33 µmol TFA) was added and reaction mixture was stirred at 295 K for 40 hours. In an exactly similar scale, another six reactions were set up in five different vials B, C, D, E, F and G and stirred at 303 K, 308 K, 313 K, 318 K, 323 K and 328 K respectively. After 40 hours, ^{1}H NMR spectra of each reaction mixture were measured at the same stirring temperature on 700 MHz NMR instrument.

Enthalpy and entropy of the reaction **(OMe-cube) → (OMe-cube)$_2$** were calculated according to the van't Hoff equation,:

$$\ln K_{eq} = -\frac{\Delta H}{RT} + \frac{\Delta S}{R} \quad \text{....... Equation (2)}$$

where: K_{eq} is the equilibrium constant

ΔH^{θ} is the difference in enthalpy between **(OMe-cube)** and **(OMe-cube)$_2$** catenane ($J\cdot mol^{-1}$)

ΔS^{θ} is the difference in entropy between **(OMe-cube)** and **(OMe-cube)$_2$** catenane ($J\cdot K^{-1}\cdot mol^{-1}$)

T is the temperature (K)

R is the universal gas constant ($J\cdot K^{-1}\cdot mol^{-1}$)

- **Equation used to find the concentrations (M) of OMe cage and dicatenane in the NMR sample used for thermodynamic and kinetic studies**

mass of cage + mass of dicatenane = 0.0019 g

(no. of moles of cage × mol. wt. of cage) + (no. of moles of dicatenane + mol. wt. of dicatenane) = 0.0019 g

(for example, the ratio of **cage:diatenane** calculated from the ^{1}H NMR spectra is **1.00:1.67**).

(n_{cage} × 5623.23 g/mol) + (n_{cat} × 11246.47 g/mol) = 0.0019 g

(e.g. cage to dicatenane ratio obtained from ^{1}H NMR is 1:1.67)

n_{cage}= A mol, $n_{ca\ t}$= 1.67 × A mol

(A mol × 5623.23 g/mol) + (1.67 × A mol x 11246.47 g/mol) = 0.0019 g

5623.23 g/mol × A mol + 18781.60 g/mol × A mol = 0.0019 g

A mol = 0.0019 g/24404.83 g/mol

A mol = 0.00000007785 mol = n_{cage}

n_{cat} = 1.67 x 0.00000007785 mol = 0.00000013 mol

[cage] = 0.00000007785 mol/ 0.0005 L = 0.0001557 M,

[dicatenane] = 0.00000013 mol/ 0.0005 L = 0.00026 M

Methoxy cage (1st set data)

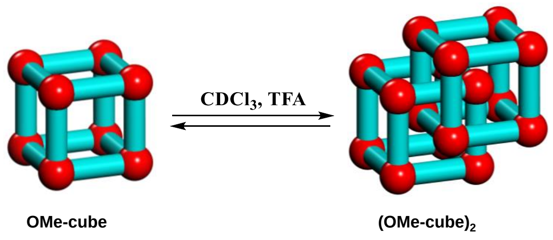

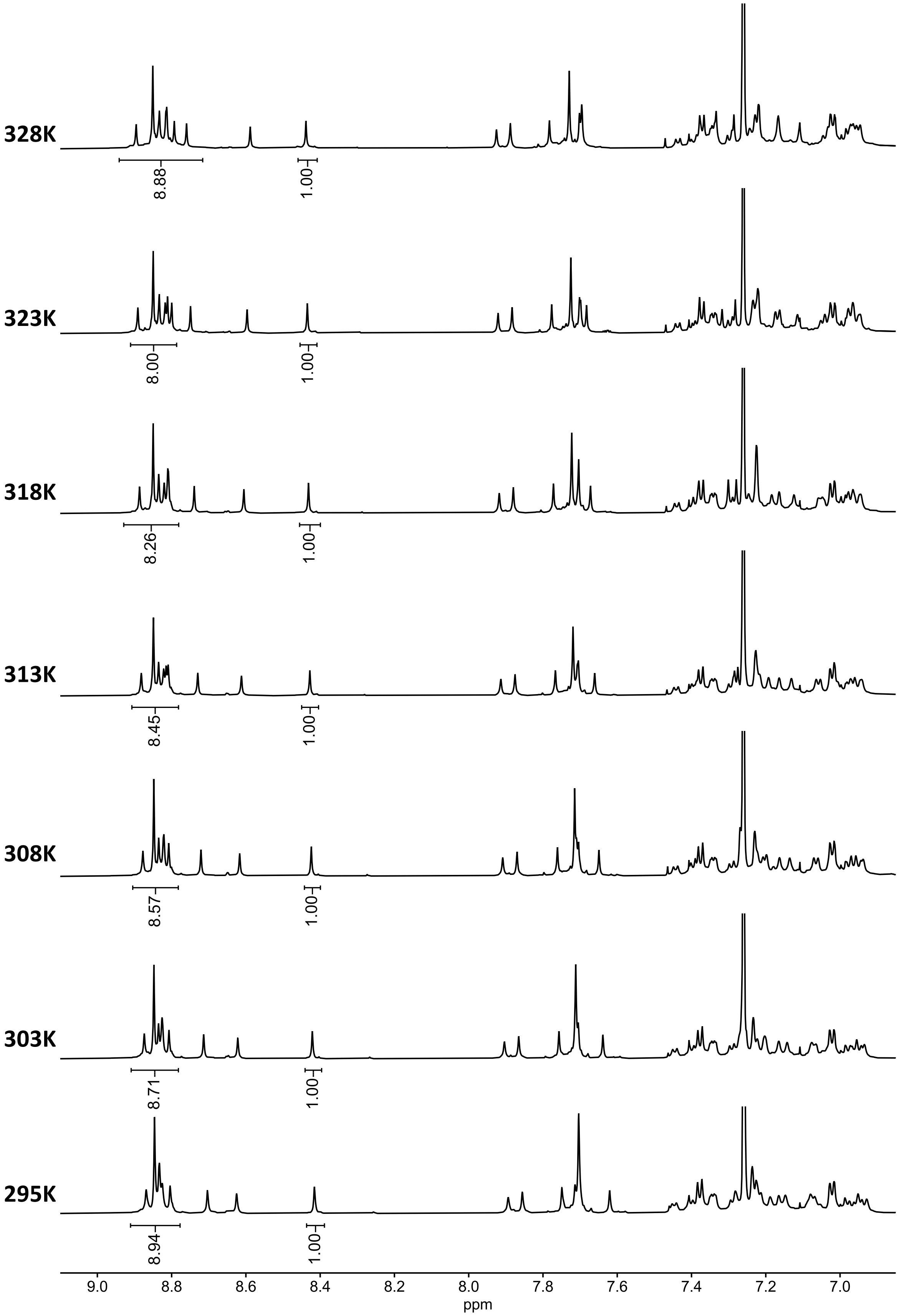

Figure 417. ^{1}H NMR spectra of reaction **(OMe-cube)** →**(OMe-cube)₂** at various temperatures under thermodynamic equilibrium.

Table 6. Calculation of ΔH and ΔS for the reaction **(OMe-cube) → (OMe-cube)$_2$**.

Entry	T (K)	Cage:Catenane	K_{eq} (M^{-1})	lnK_{eq}	1/T (K^{-1})
1	295	1:1.0152	4552.542	8.423	0.00339
2	303	1:1.0782	5035.600	8.524	0.00330
3	308	1:1.1204	5373.275	8.589	0.00325
4	313	1:1.1594	5693.895	8.647	0.00319
5	318	1:1.2270	6271.124	8.744	0.00314
6	323	1:1.3333	7234.230	8.887	0.00310
7	328	1:1.3889	7764.010	8.957	0.00305

The slope value (-1592.68 ± 131) was $-\Delta H^{\theta}/R$ and the intercept value (13.78 ± 0.42) was $\Delta S^{\theta}/R$. Therefore, ΔH^{θ} = 13.2 ± 1.1 kJ/mol, and ΔS = 114.6 ± 3.5 J/Kmol.

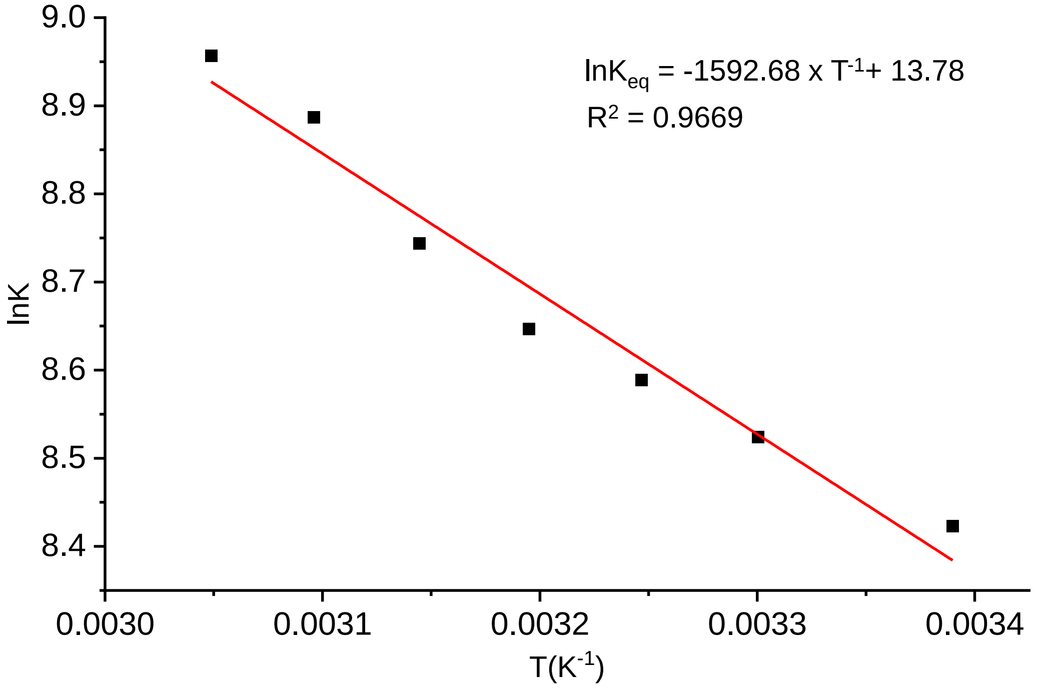

Figure 418. van't Hoff plot of the reaction **(OMe-cube) → (OMe-cube)$_2$**.

Methoxy cage (2nd set data)

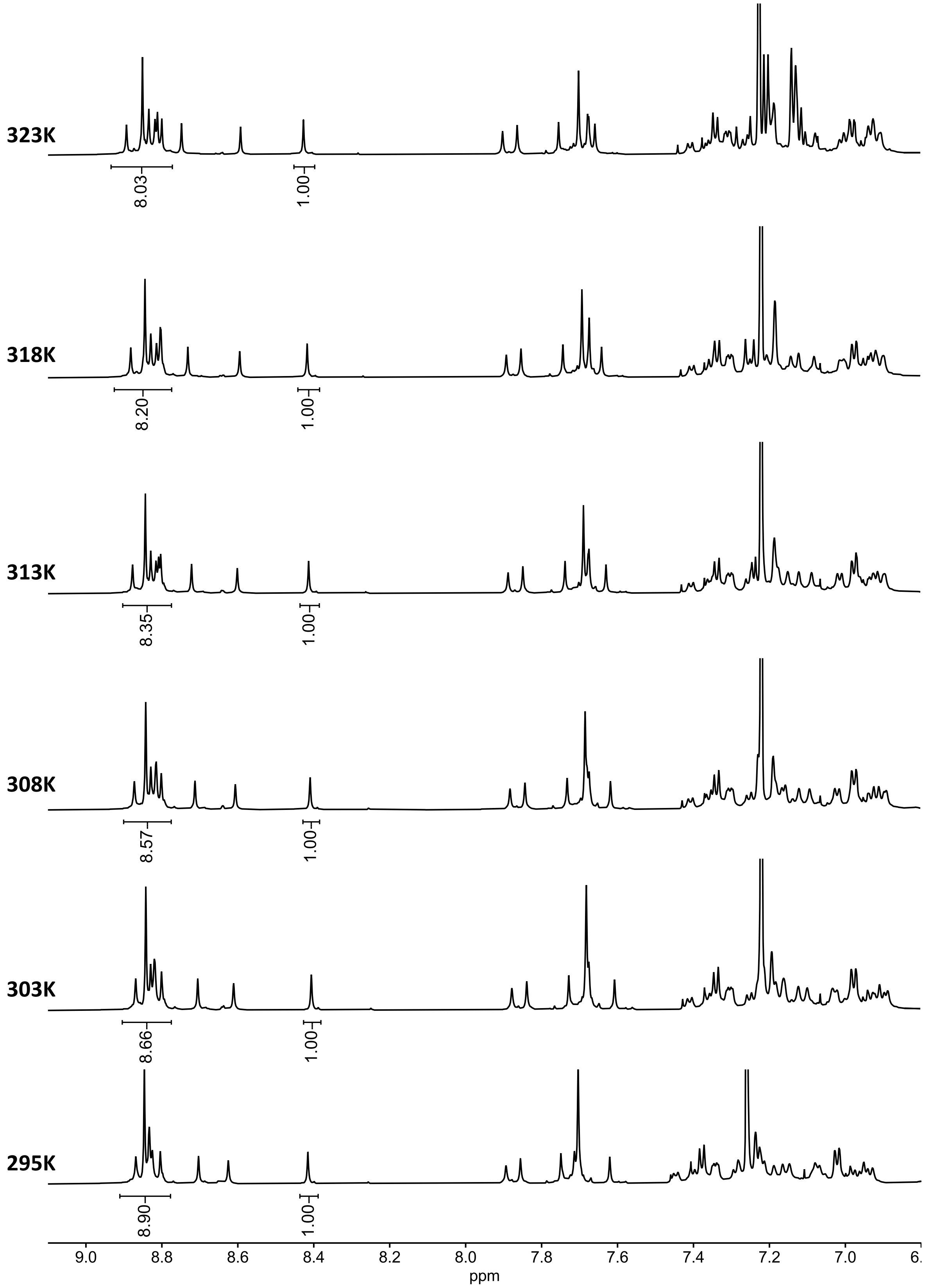

Figure 419. ^{1}H NMR spectra of reaction **(OMe-cube) →(OMe-cube)$_2$** at various temperatures under thermodynamic equilibrium.

Table 7. Calculation of ΔH and ΔS for the reaction **(OMe-cube) → (OMe-cube)2**.

Entry	T (K)	Cage:Catenane	K_{eq} (M^{-1})	lnK_{eq}	1/T (K^{-1})
1	295	1:1.0256	4630.841	8.440	0.00339
2	303	1:1.0929	5152.032	8.547	0.00330
3	308	1:1.1204	5373.275	8.589	0.00325
4	313	1:1.1940	5986.161	8.697	0.00319
5	318	1:1.2500	6473.814	8.776	0.00314
6	323	1:1.3201	7111.029	8.869	0.00310

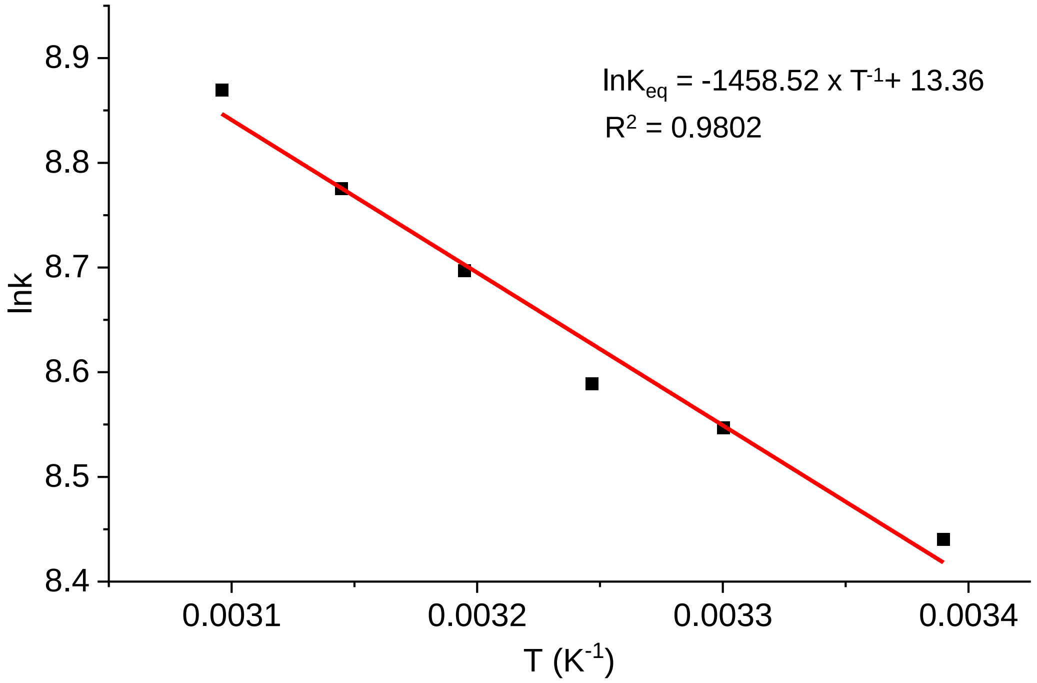

Figure 420. van't Hoff plot of the reaction **(OMe-cube) → (OMe-cube)2**.

The slope value (-1458.52 ± 103.64) was -$\Delta H^{\theta}/R$ and the intercept value (13.36 ± 0.33) was $\Delta S^{\theta}/R$. Therefore, ΔH^{θ} = 12.1 ± 0.9 kJ/mol, and ΔS = 111.1 ± 2.7 J/Kmol

Methoxy cage (3rd set data)

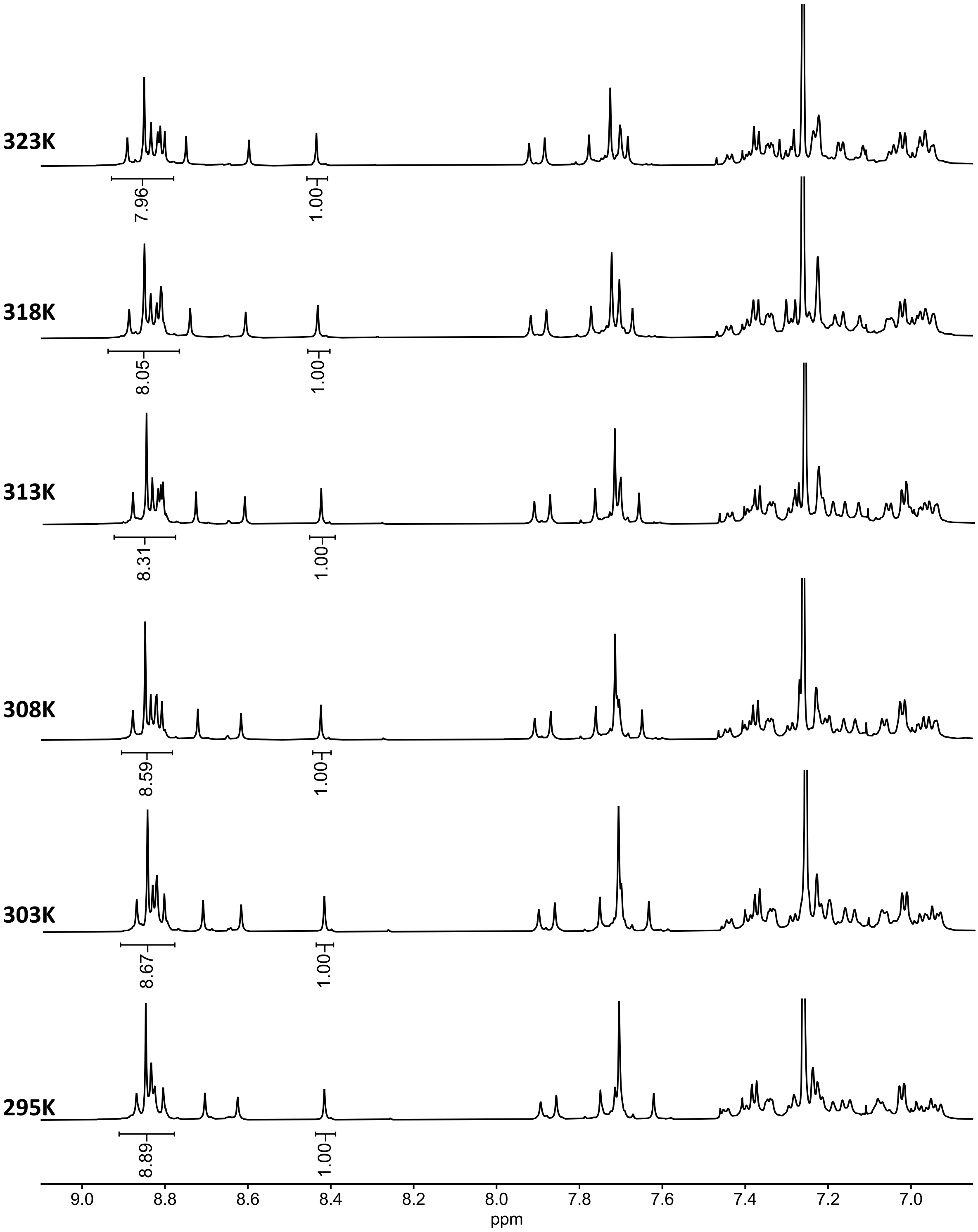

Figure 421. ^{1}H NMR spectra of reaction **(OMe-cube)** →**(OMe-cube)₂** at various temperatures under thermodynamic equilibrium.

Table 8. Calculation of ΔH and ΔS for the reaction **(OMe-cube) → (OMe-cube)₂**.

**Entry**	**T (K)**	**Cage:Catenane**	K_{eq} **(M^{-1})**	**lnK_{eq}**	**1/T (K^{-1})**
1	295	1:1.0283	4650.770	8.445	0.00339
2	303	1:1.0899	5128.388	8.543	0.00330
3	308	1:1.1142	5322.758	8.580	0.00325
4	313	1:1.2085	6110.107	8.718	0.00319
5	318	1:1.3115	7030.800	8.858	0.00314
6	323	1:1.3514	7404.049	8.910	0.00310

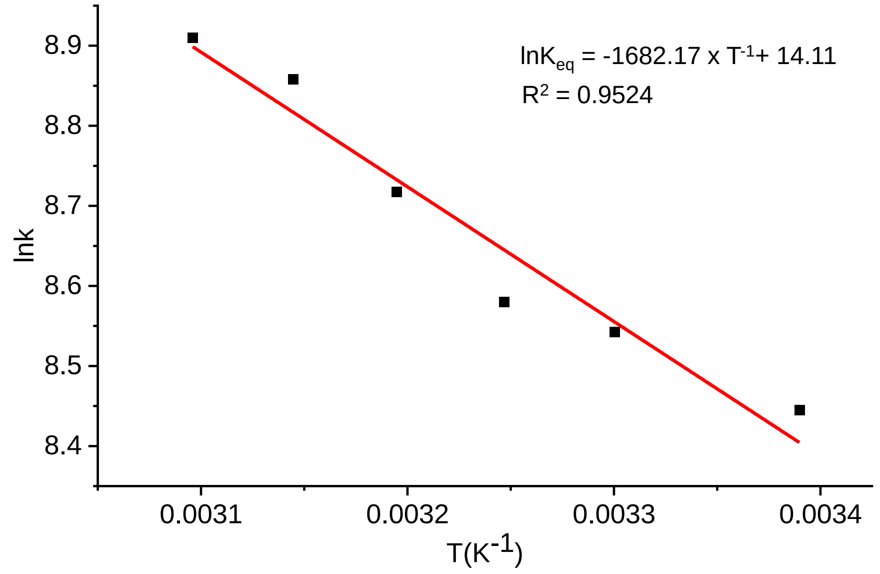

Figure 422. van't Hoff plot of the reaction **(OMe-cube) → (OMe-cube)₂**.

The slope value (-1682.17 ± 187.97) was $-\Delta H^{\theta}/R$ and the intercept value (14.11 ± 0.61) was $\Delta S^{\theta}/R$. Therefore, ΔH^{θ} = 14.0 ± 0.9 kJ/mol, and ΔS = 117.3 ± 5.1 J/Kmol.

Table 9. ΔH and **ΔS** values calculated from Van't Hoff plots from three sets of experiments for **(OMe-cube) → (OMe-cube)₂** reaction.

**Entry**	**Exp No.**	**ΔH**	**ΔH (kJ/mol)**	**Exp No.**	**ΔS**	**ΔS (J/Kmol)**
1	Set 1	13.2		Set 1	114.6	
2	Set 2	12.1	13.1 ± 0.8	Set 2	111.1	114.3 ± 2.5
3	Set 3	14.0		Set 3	117.3	

The average value of three measurements reported as **ΔH** and **ΔS**. The standard deviations calculated using STDEV.P function of Microsoft Excel 2016.

(SMe-cube) → (SMe-cube)$_2$

Procedure: A vial 'A' was charged with **SMe-cube** (2.0 mg, 0.33 μmol) and deuterated $CHCl_3$ (0.5 mL). Catalytic TFA (5 μL of a 0.653 mM solution in $CDCl_3$, 0.33 μmol TFA) was added and reaction mixture was stirred at 295 K for 40 hours. In a exactly similar scale another reactions were set up in five different vials B, C, D, E, F and G and stirred at 303 K, 308 K, 313 K, 318 K, 323 K and 328 K respectively. After 40 hours, ^{1}H NMR spectra of each reaction mixture were measured at the same stirring temperature on 700 MHz NMR instrument.

Thiomethyl cage (1st set data)

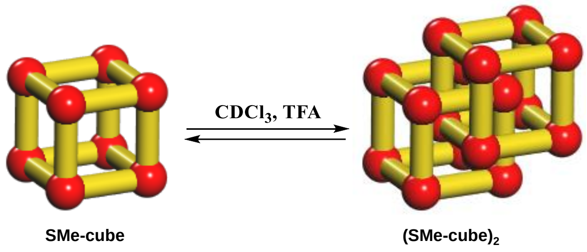

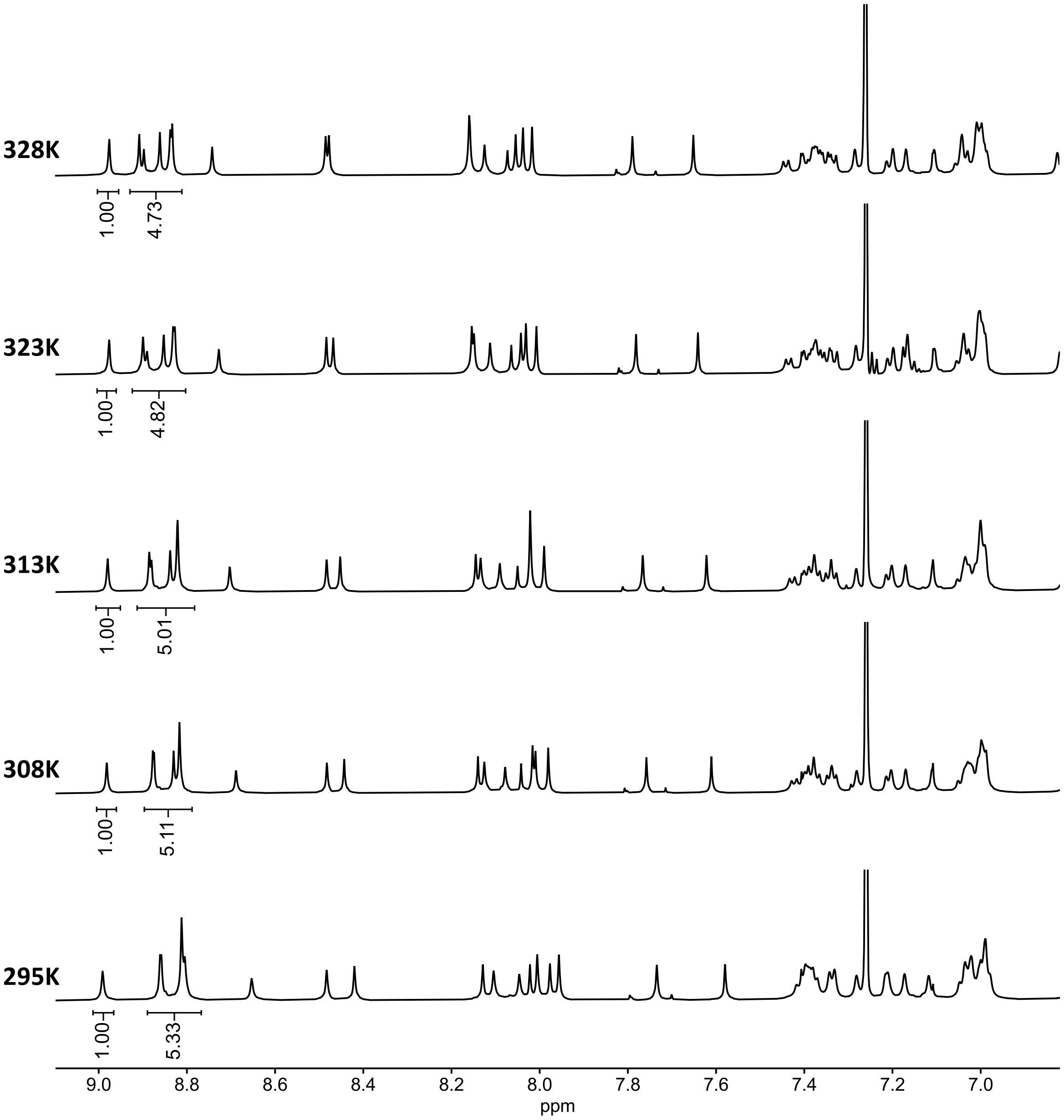

Figure 423. ^{1}H NMR spectra of reaction **(SMe-cube) →(SMe-cube)$_2$** at various temperatures under thermodynamic equilibrium.

Table 10. Calculation of ΔH and ΔS for the reaction **(SMe-cube) → (SMe-cube)$_2$**.

Entry	T (K)	Cage:Catenane	K_{eq} (M^{-1})	lnK_{eq}	1/T (K^{-1})
1	295	1:3.0075	33361.455	10.415	0.00339
3	308	1:3.6036	43763.755	10.687	0.00325
3	313	1:3.9604	52278.631	10.864	0.00319
4	323	1:4.8780	77639.556	11.259	0.00310
5	328	1:5.4795	96964.036	11.482	0.00305

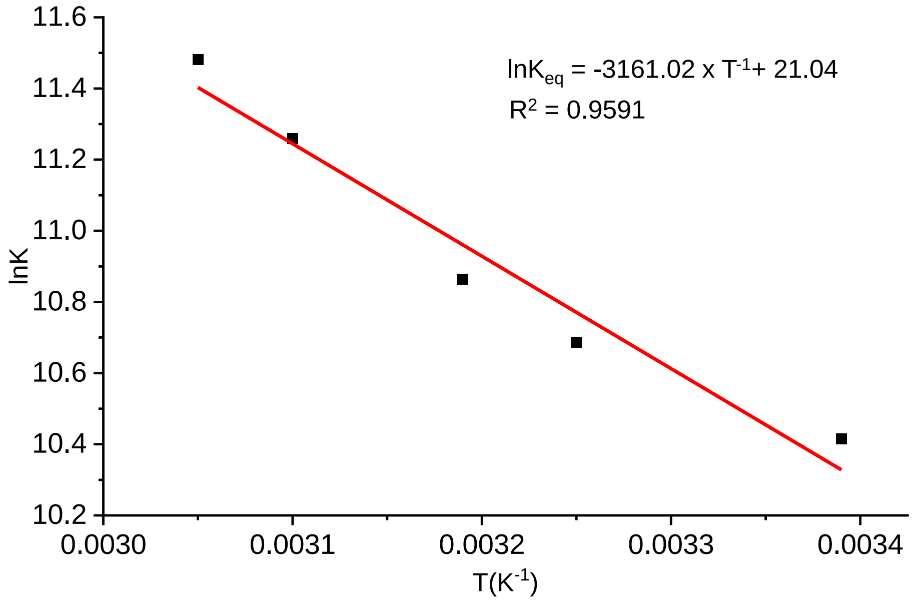

Figure 424. van't Hoff plot of the reaction **(SMe-cube) → (SMe-cube)₂**.

The slope value (-3161.02 ± 376.7) was -$\Delta H^{\theta}/R$ and the intercept value (21 ± 1.21) was $\Delta S^{\theta}/R$. Therefore, ΔH^{θ} = 26.3 ± 3.1 kJ/mol, and ΔS = 174.9 ± 10.1 J/Kmol.

Thiomethyl cage (2nd set data)

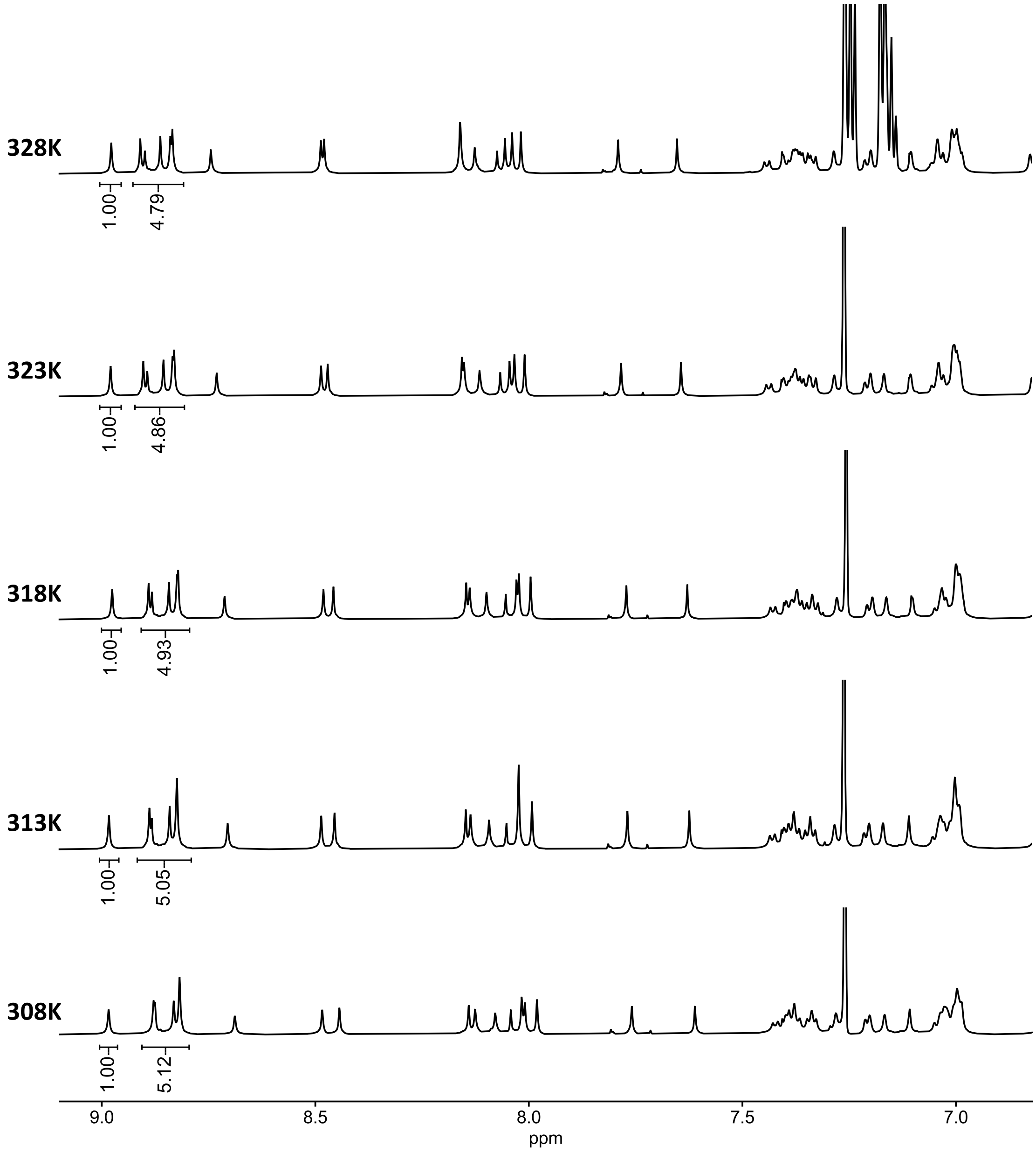

Figure 425. ^{1}H NMR spectra of reaction **(SMe-cube)→(SMe-cube)$_2$** at various temperatures under thermodynamic equilibrium.

Table 11. Calculation of ΔH and ΔS for the reaction **(OMe-cube)** → **(OMe-cube)$_2$**.

Entry	T (K)	Cage:Catenane	K_{eq} (M^{-1})	lnK_{eq}	1/T (K^{-1})
1	308	1:3.5714	43032.934	10.670	0.00325
2	313	1:3.8095	48586.111	10.791	0.00319
3	318	1:4.3011	61112.173	11.020	0.00314
4	323	1:4.6512	70905.346	11.169	0.00310
5	328	1:5.0633	83363.675	11.331	0.00305

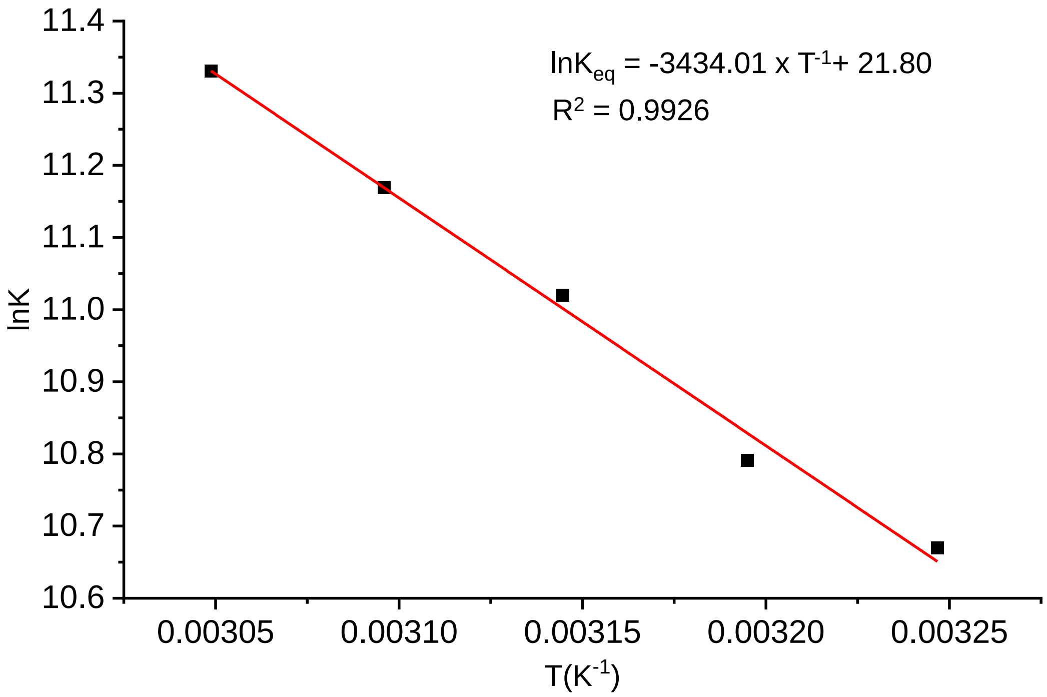

Figure 426. van't Hoff plot of the reaction **(SMe-cube)** → **(SMe-cube)$_2$**.

The slope value (-3435.08 ± 171.0) was -$\Delta H^{\theta}/R$ and the intercept value (21.80 ± 0.54) was $\Delta S^{\theta}/R$. Therefore, ΔH^{θ} = 28.6 ± 1.4 kJ/mol, and ΔS = 181.2 ± 4.5 J/Kmol.

Thiomethyl cage (3rd set data)

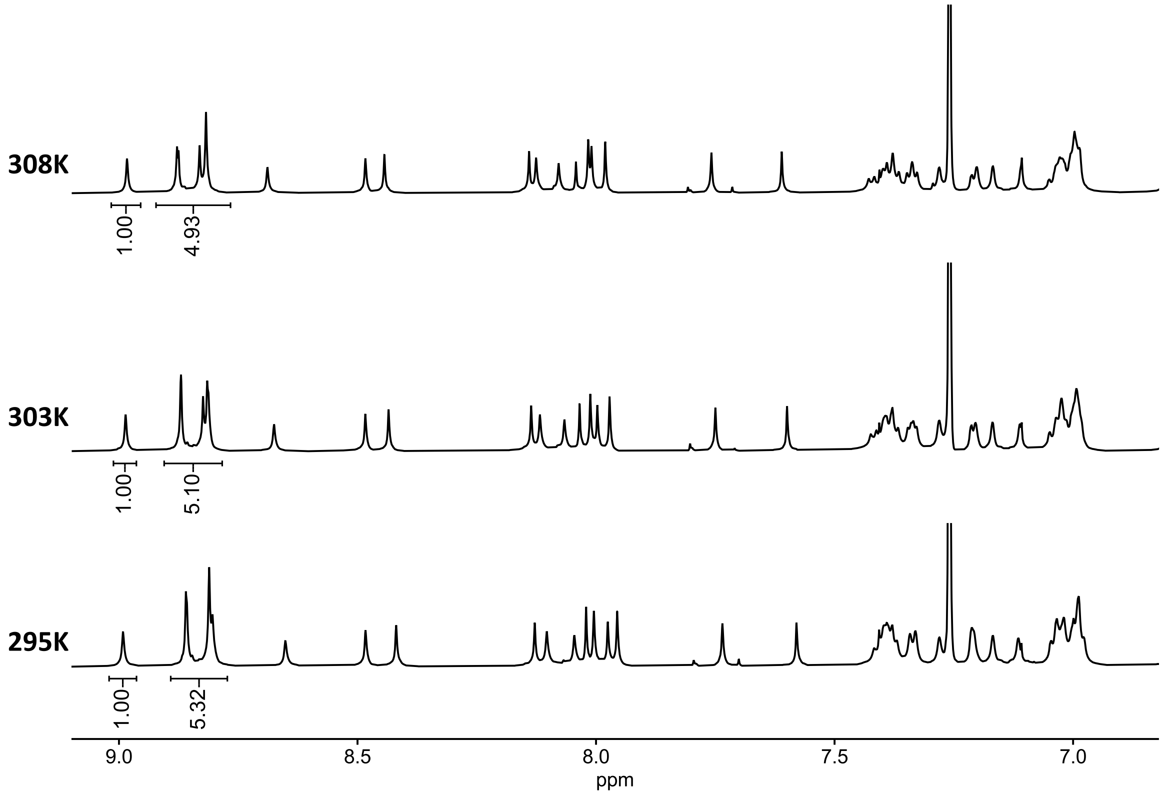

Figure 427. ^{1}H NMR spectra of reaction **(SMe-cube) →(SMe-cube)$_2$** at various temperatures under thermodynamic equilibrium.

Table 12. Calculation of ΔH and ΔS for the reaction **(SMe-cube)** → **(SMe-cube)$_2$**.

Entry	T (K)	Cage:Catenane	K_{eq} (M^{-1})	lnK_{eq}	1/T (K^{-1})
1	295	1:3.0303	33832.545	10.429	0.00339
2	303	1:3.6364	44514.160	10.704	0.00330
3	308	1:4.3011	61112.173	11.021	0.00319

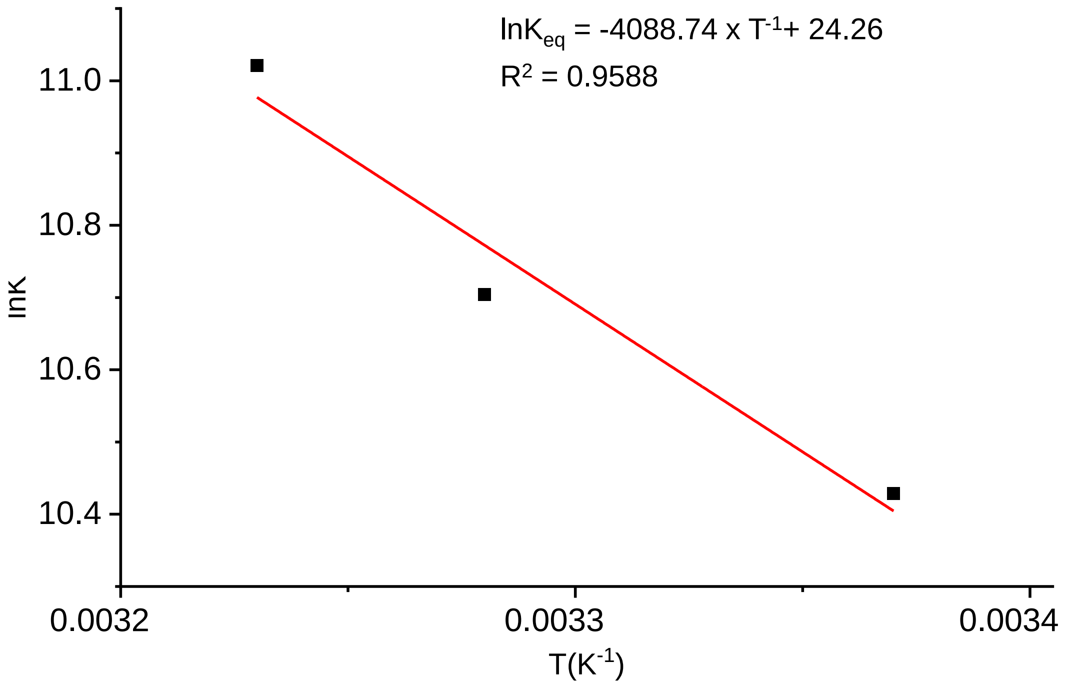

Figure 428. van't Hoff plot of the reaction **(SMe-cube) → (SMe-cube)2**.

The slope value (-4023.2 ± 847.7) was $-\Delta H^{\theta}/R$ and the intercept value (21.36 ± 2.8) was $\Delta S^{\theta}/R$. Therefore, ΔH^{θ} = 33.4 ± 7.0 kJ/mol, and ΔS = 199.9 ± 23.3 J/Kmol.

Table 13. ΔH and **ΔS** values calculated from Van't Hoff plots from three sets of experiments for **(SMe-cube) → (SMe-cube)2** reaction.

Entry	Exp No.	ΔH	ΔH (kJ/mol)	Exp No.	ΔS	ΔS (J/Kmol)
1	Set 1	26.3		Set 1	174.9	
2	Set 2	28.6	29.4 ± 3.0	Set 2	181.2	185.3 ± 10.6
3	Set 3	33.4		Set 3	199.9	

The average value of three measurements reported as **ΔH** and **ΔS**. The standard deviations calculated using STDEV.P function of Microsoft Excel 2016.

13. ^{1}H NMR assignment of (OMe-cube)$_2$ and (SMe-cube)$_2$

a) ^{1}H NMR assignment of (OMe-cube)$_2$

^{1}H NMR, 700 MHz, CD_2Cl_2

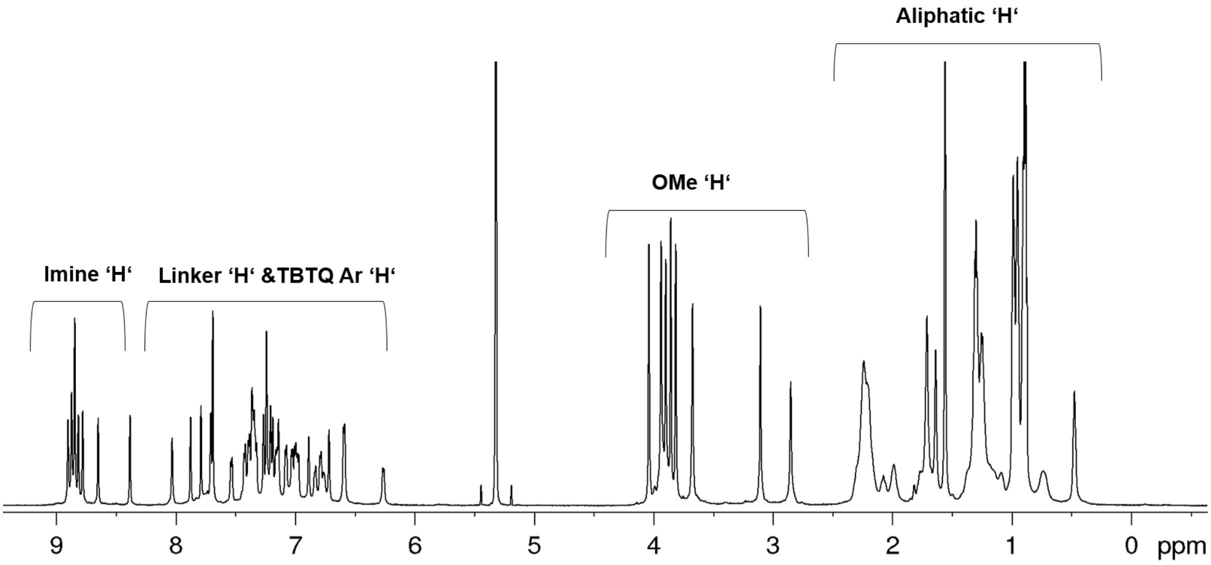

Figure 429. ^{1}H NMR spectrum (700 MHz, CD_2Cl_2) of **(OMe-cube)$_2$**. (The group of protons were assigned by 1D and 2D NMR spectra).

^{13}H NMR, 700 MHz, CD_2Cl_2

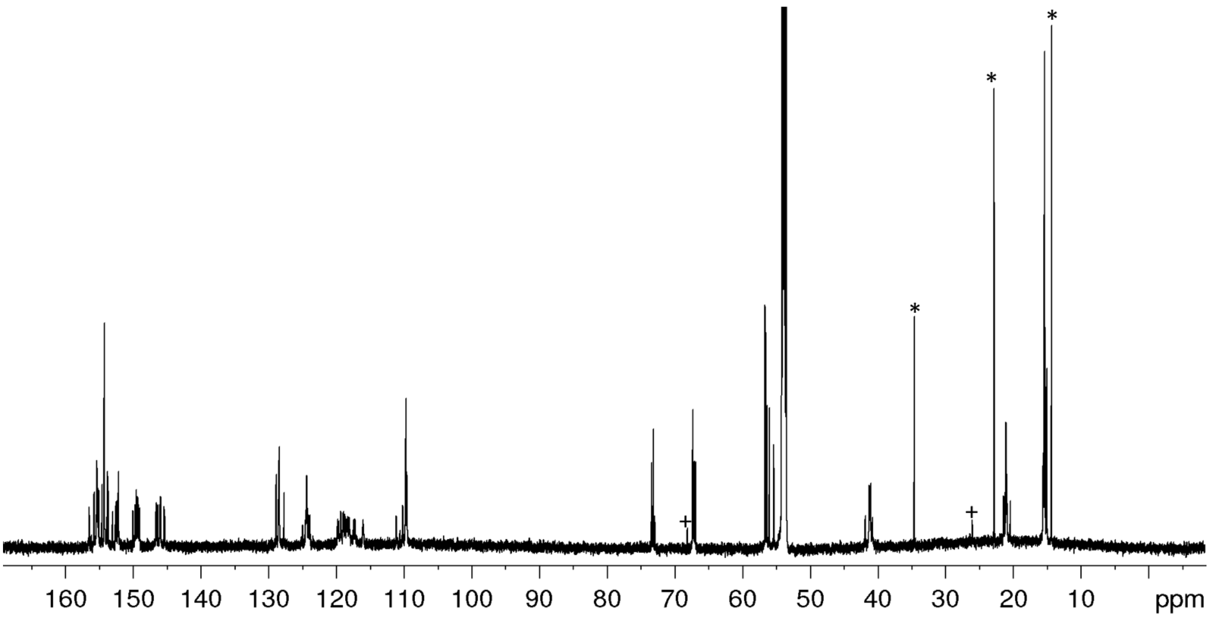

Figure 430. ^{13}C NMR spectrum (700 MHz, CD_2Cl_2) of **(OMe-cube)$_2$**. (*pentane, $^+$THF)

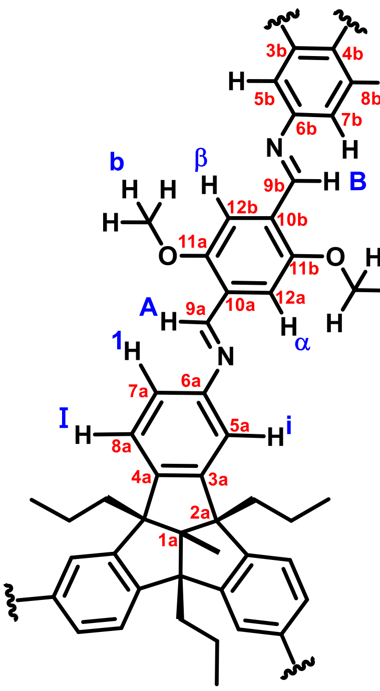

Figure 431. Labelling of protons and carbons to the partial chemical structure of **(OMe-cube)2**.

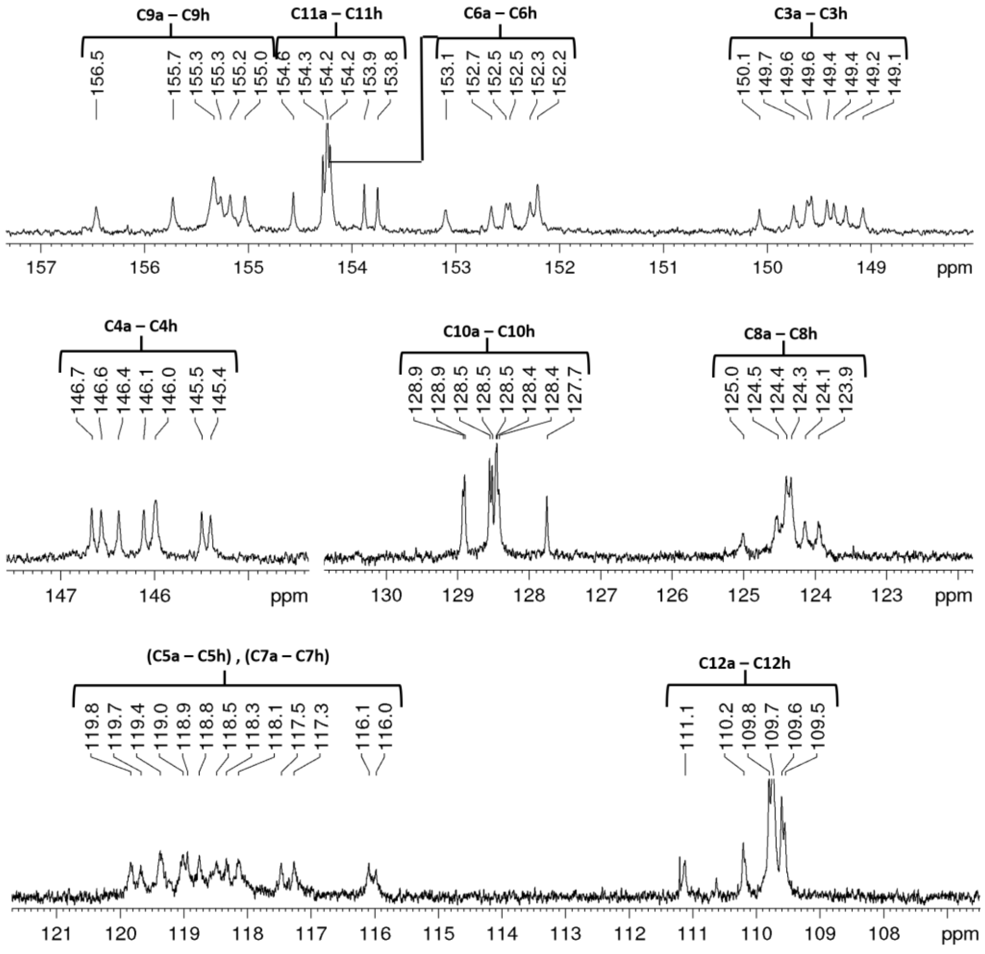

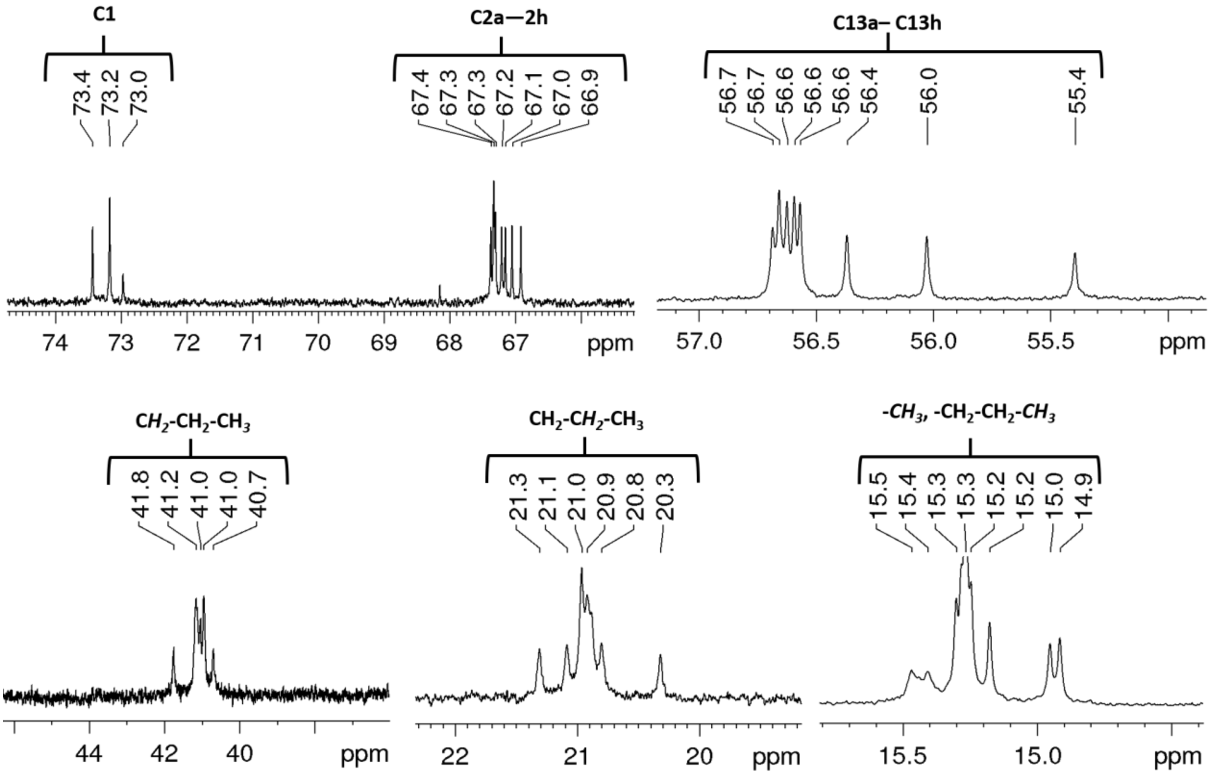

Figure 432. Partial ^{13}C NMR spectrum (700 MHz, CD_2Cl_2) of **(OMe-cube)$_2$**. (All the carbon regions were assigned by HSQC, HMBC, DEPT).

Assignment of eight different signals of imine, linker and methoxy protons to the 3D model of (OMe-cube)$_2$

1. First imine protons were labelled as A, B, C, D, E, F, G and H from lower chemical shift values to higher chemical shift values as shown in Figure 433.

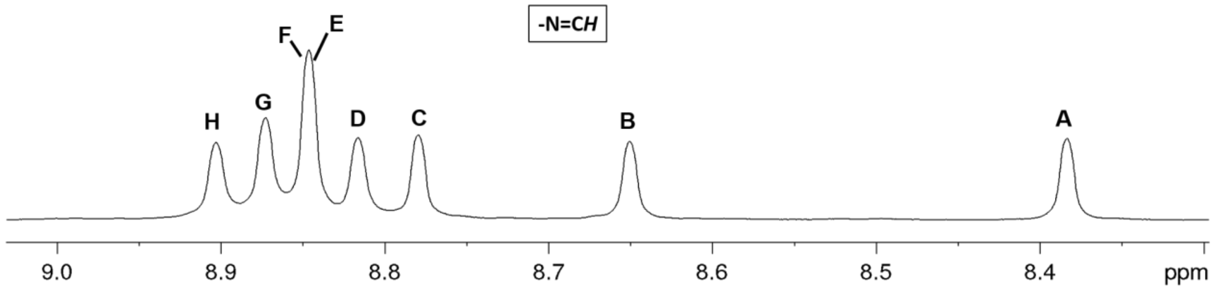

Figure 433. Partial ^1H NMR (700 MHz, CD_2Cl_2) spectrum of **(OMe-cube)$_2$** showing the labelling of imine protons.

2. Four pairs of neighboring imine protons and dialdehyde linker protons as shown in following Figure 434 were identified by multiple HSQC and HMBC interactions (see Figure 435-438, Table 14 and 15).

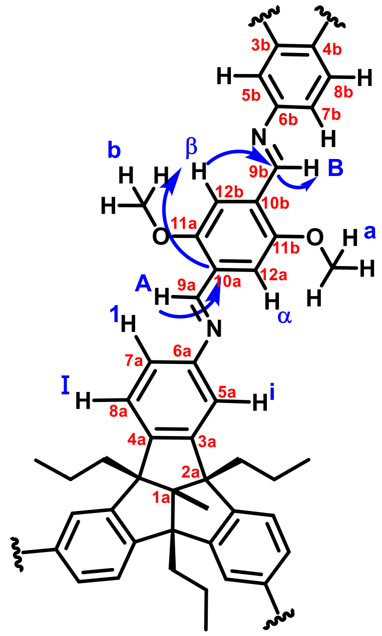

Figure 434. Labelling of protons and carbons of **(OMe-cube)$_2$**. (Arrow indicates that the how neighboring linker and imine protons are identified by HMBC and HSQC measurements).

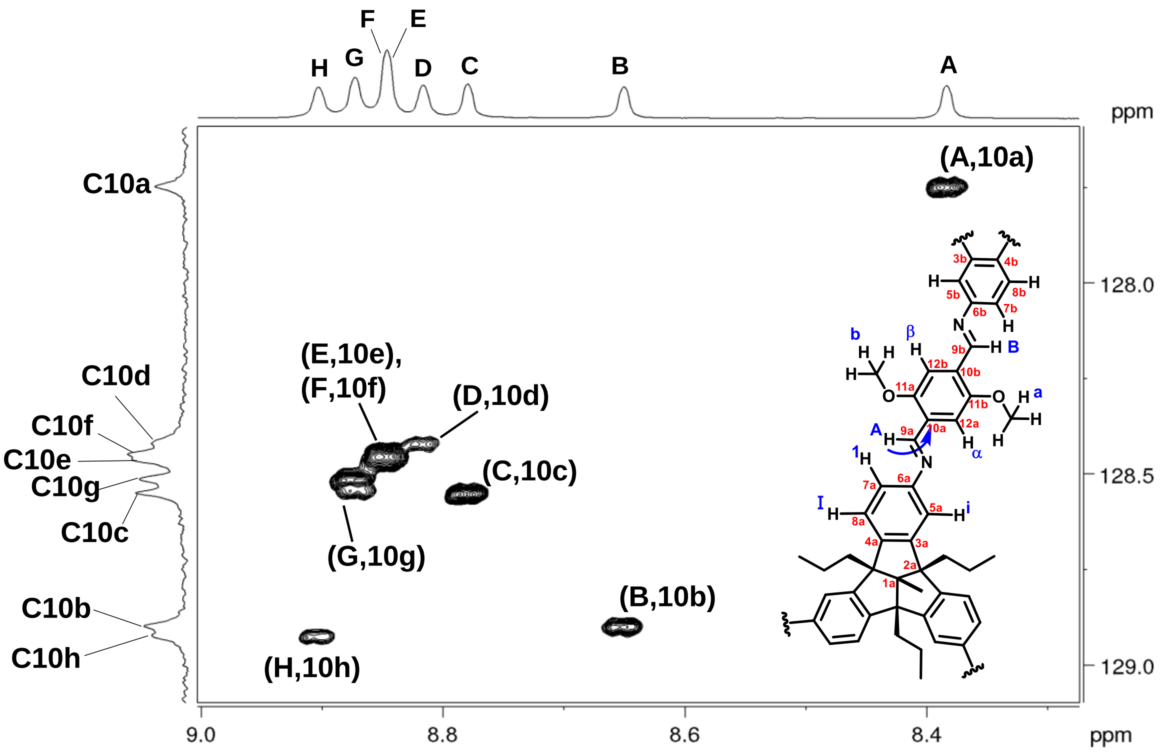

Figure 435. Partial ^{1}H-^{13}C HMBC NMR (700 MHz, CD_2Cl_2) spectrum of **(OMe-cube)$_2$** showing cross peaks between H-(A-H) and C-(10a-10h). (See Table 14 for chemical shift values).

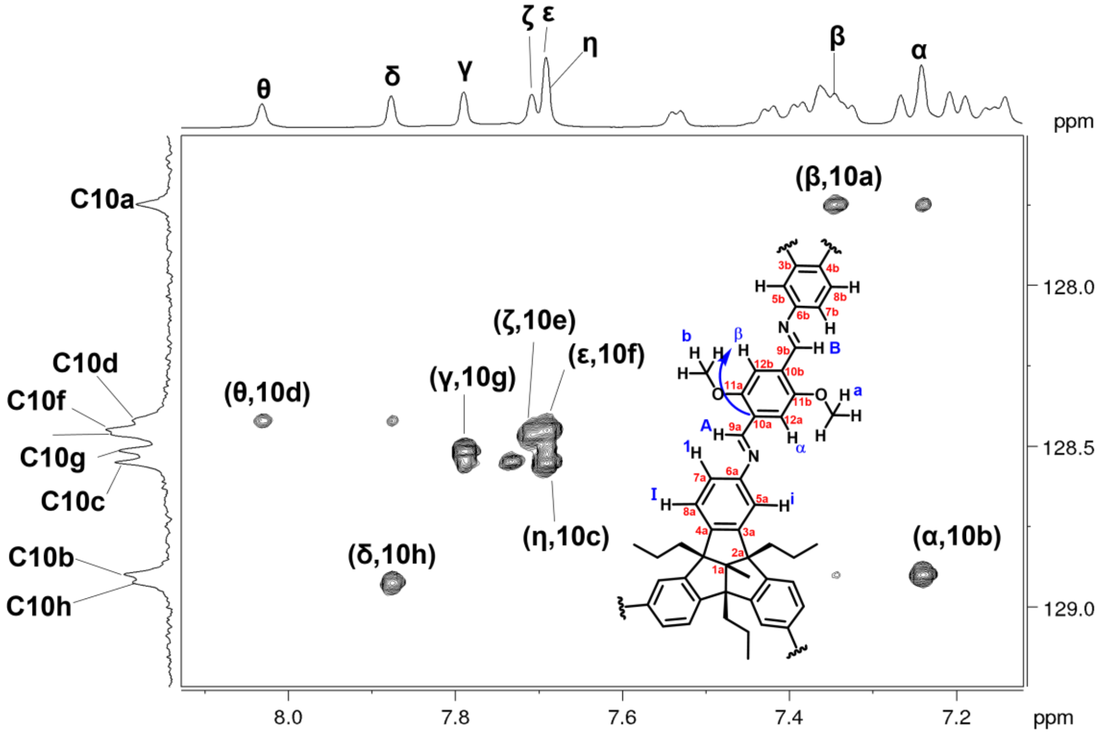

Figure 436. Partial ^{1}H-^{13}C HMBC NMR (700 MHz, CD_2Cl_2) spectrum of **(OMe-cube)$_2$** showing cross peaks between C-(10a-10h) and H-(α - θ). (See Table 14 for chemical shift values). (Here linker aromatic protons were first labelled using HMBC and HSQC correlation through the imine protons, see Figure 438-437).

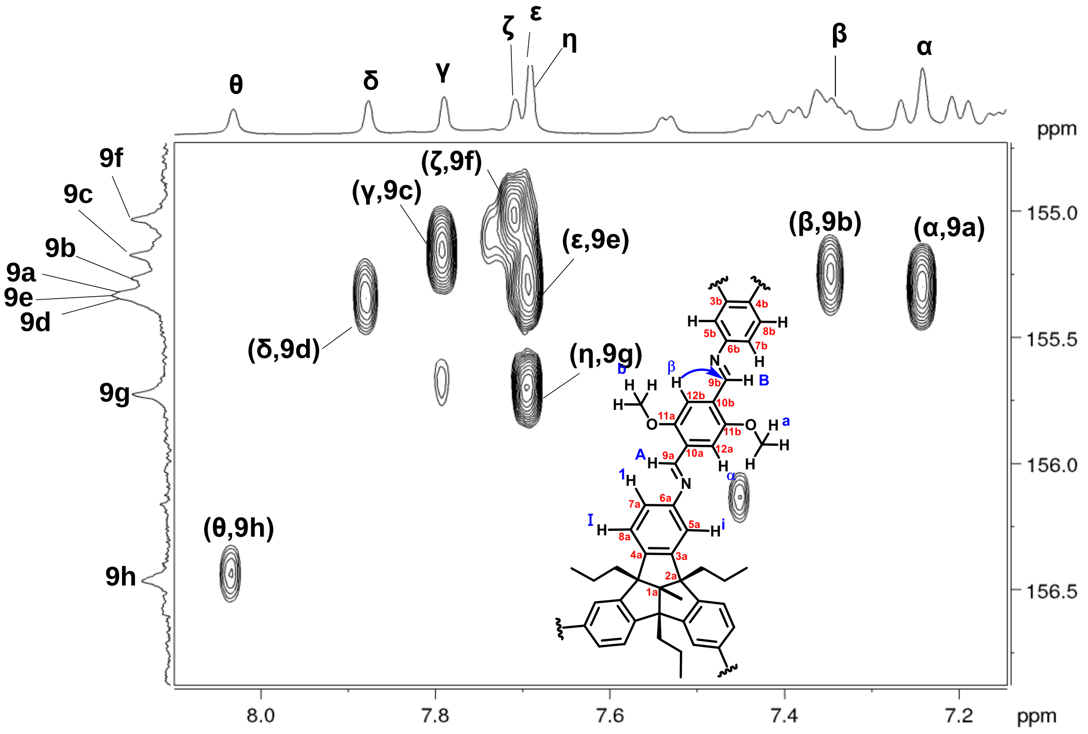

Figure 437. Partial ^{1}H-^{13}C HMBC NMR (700 MHz, CD_2Cl_2) spectrum of **(OMe-cube)$_2$** showing cross peaks between H-(a- θ) and C-(9a-9h). (See Table 15 for chemical shift values).

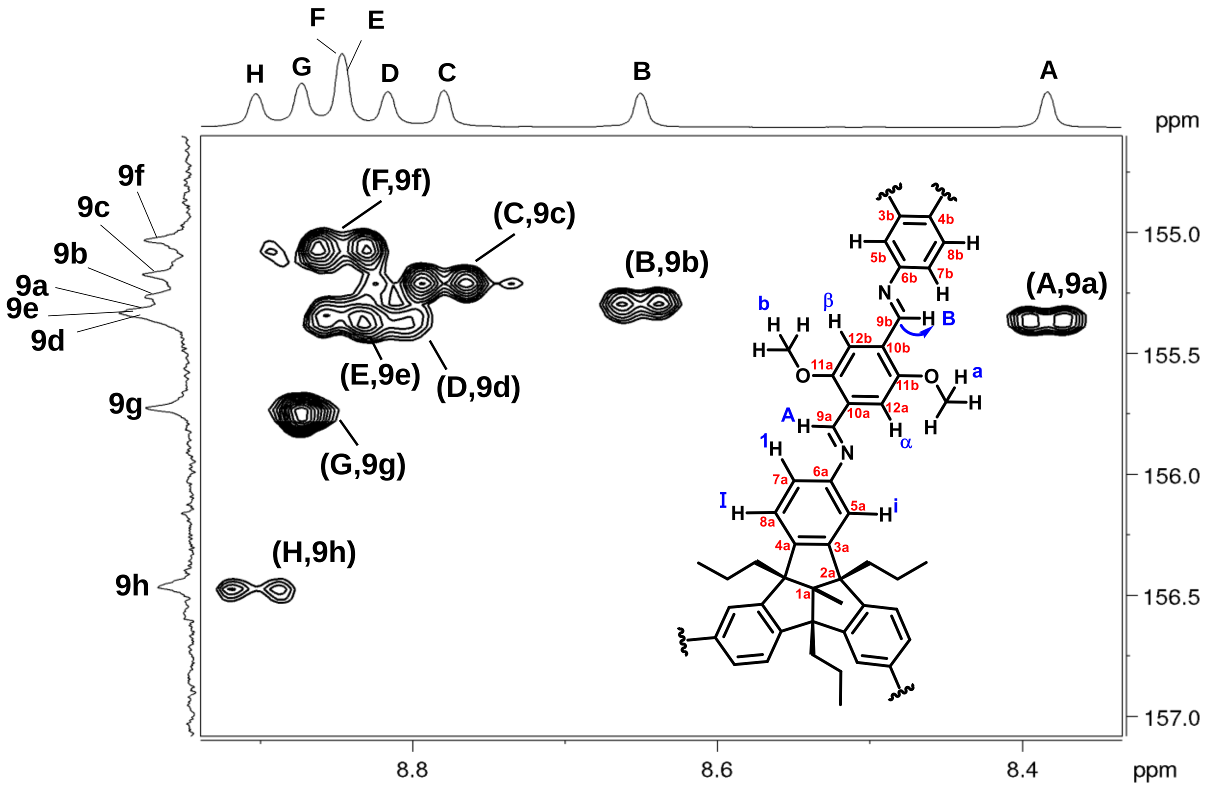

Figure 438. Partial ^{1}H-^{13}C HSQC NMR (700 MHz, CD_2Cl_2) spectrum of **(OMe-cube)$_2$** showing cross peaks between C-(9a-9h) and H-(A-H). (See Table 15 for chemical shift values).

Table 14. ^{1}H-^{13}C HMBC interactions between H-(A-H) and C-(10a-10h), and C-(10a-10h) and H-(α-θ).

^{1}H-Atom	^{1}H-NMR (HMBC)	[13]C Atom	^{13}C Atom (HMBC)	[13]C Atom	^{13}C Atom (HMBC)	^{1}H-Atom	^{1}H-NMR (HMBC)
A	8.39	10a	127.75	10a	127.75	β	7.34
B	8.65	10b	128.90	10b	128.90	α	7.24
C	8.78	10c	128.55	10c	128.55	η	7.69
D	8.82	10d	128.42	10d	128.42	θ	8.03
E	8.85	10e	128.46	10e	128.46	ζ	7.71
F	8.85	10f	128.45	10f	128.45	ε	7.69
G	8.87	10g	128.51	10g	128.51	γ	7.79
H	8.90	10h	128.92	10h	128.92	δ	7.88

Table 15. HMBC interactions between H-(α-θ) and C-(9a-9h) and HSQC interactions between C-(9a-9h) and H-(A-H).

^{1}H-Atom	^{1}H-NMR (HMBC)	^{13}C Atom	^{13}C Atom (HMBC)	^{13}C Atom	^{13}C Atom (HMBC)	^{1}H-Atom	^{1}H-NMR (HSQC)
β	7.34	9b	155.27	9b	155.27	B	8.65
α	7.24	9a	155.53	9a	155.53	A	8.39
η	7.69	9g	155.72	9g	155.72	G	8.87
θ	8.03	9h	156.47	9h	156.47	H	8.90
ζ	7.71	9f	155.03	9f	155.03	F	8.85
ε	7.69	9e	155.33	9e	155.33	E	8.85
γ	7.79	9c	155.17	9c	155.17	C	8.78
δ	7.88	9d	155.33	9d	155.33	D	8.82

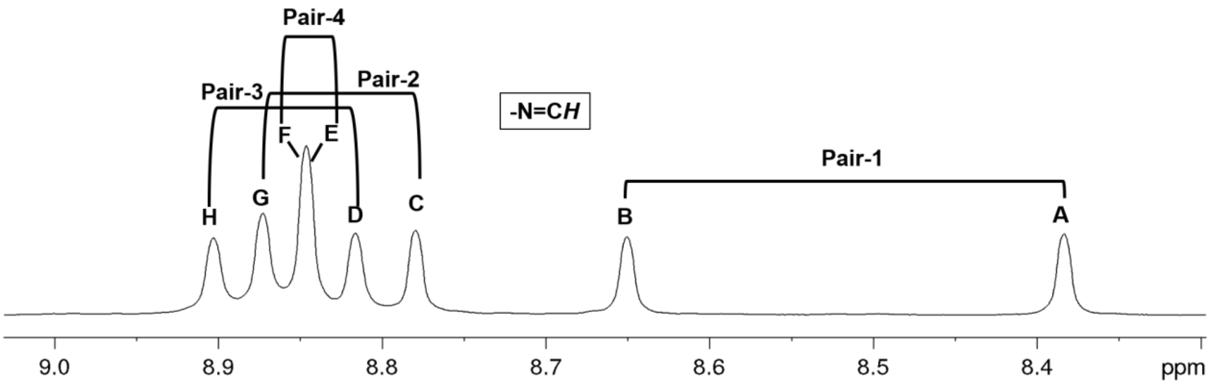

Figure 439. Partial ^{1}H NMR (700 MHz, CD_2Cl_2) spectrum of **(OMe-cube)₂** showing four pairs of neighboring imine protons.

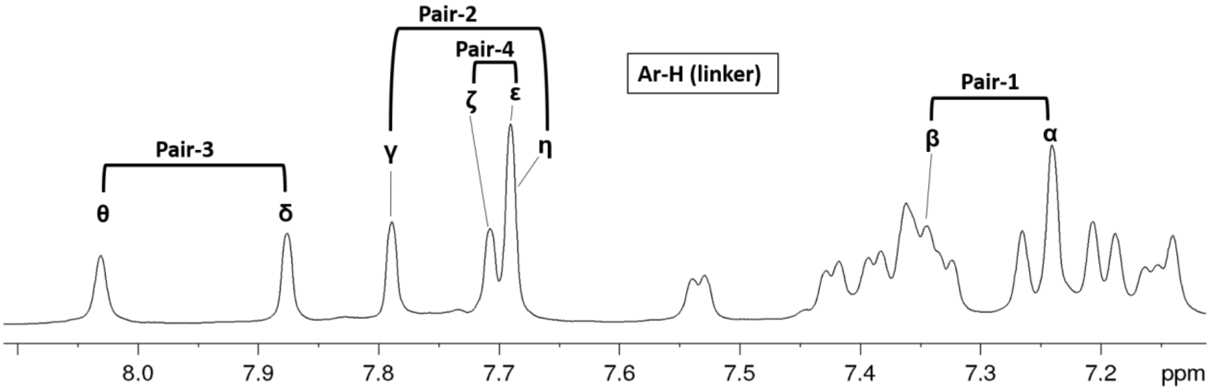

Figure 440. Partial ^{1}H NMR (700 MHz, CD_2Cl_2) spectrum of **(OMe-cube)$_2$** showing four pairs of neighboring linker protons.

3. Four neighboring methoxy protons were assigned by HSQC, HMBC and NOESY interactions as shown in Figure 441 and 442 (Table 16 and 17).

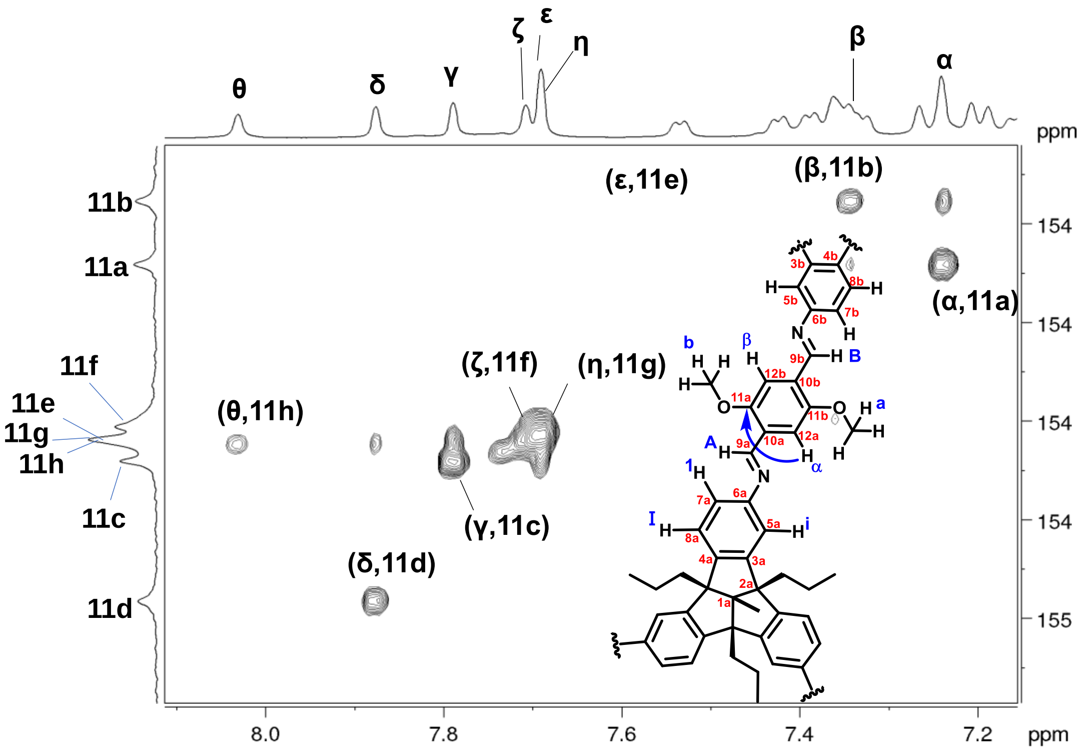

Figure 441. Partial ^{1}H-^{13}C HMBC NMR (700 MHz, CD_2Cl_2) spectrum of **(OMe-cube)₂** showing cross peaks between H-(α- θ) and C-(11a-11h). (See Table 16 for chemical shift values).

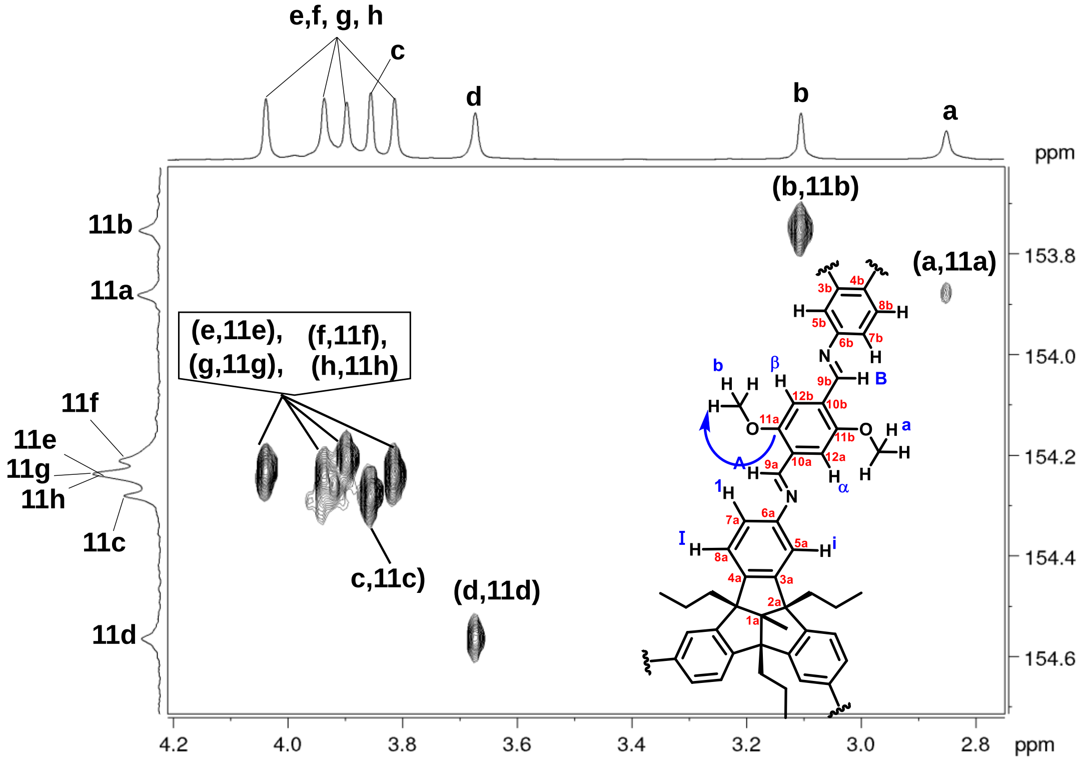

Figure 442. Partial ^{1}H-^{13}C HMBC NMR (700 MHz, CD_2Cl_2) spectrum of **(OMe-cube)$_2$** showing cross peaks between C-(11a-11h) and H-(a-h). (See Table 16 for chemical shift values).

Table 16. ^{1}H-^{1}H HMBC interactions between H-(α-θ) and C-(11a-11h), and C-(11a-11h) and H-(a-h).

^{1}H-Atom	^{1}H-NMR (HMBC)	^{13}C Atom	^{13}C Atom (HMBC)	^{13}C Atom	^{13}C Atom (HMBC)	^{1}H-Atom	^{1}H-NMR (HMBC)
α	7.24	11a	153.88	11a	153.88	a	2.85
β	7.34	11b	153.75	11b	153.75	b	3.11
γ	7.79	11c	154.28	11c	154.28	c	3.86
δ	7.88	11d	154.56	11d	154.56	d	3.67
ε	7.69	11e	154.24	11e	154.24	e/f/g/h	*
ζ	7.71	11f	154.21	11f	154.21	e/f/g/h	*
η	7.69	11g	154.24	11g	154.24	e/f/g/h	*
θ	8.03	11h	154.24	11h	154.24	e/f/g/h	*

*Could not assigned due to overlapped carbon signals. These signals were assigned from ^{1}H-^{1}H NOESY spectrum (See Figure 443).

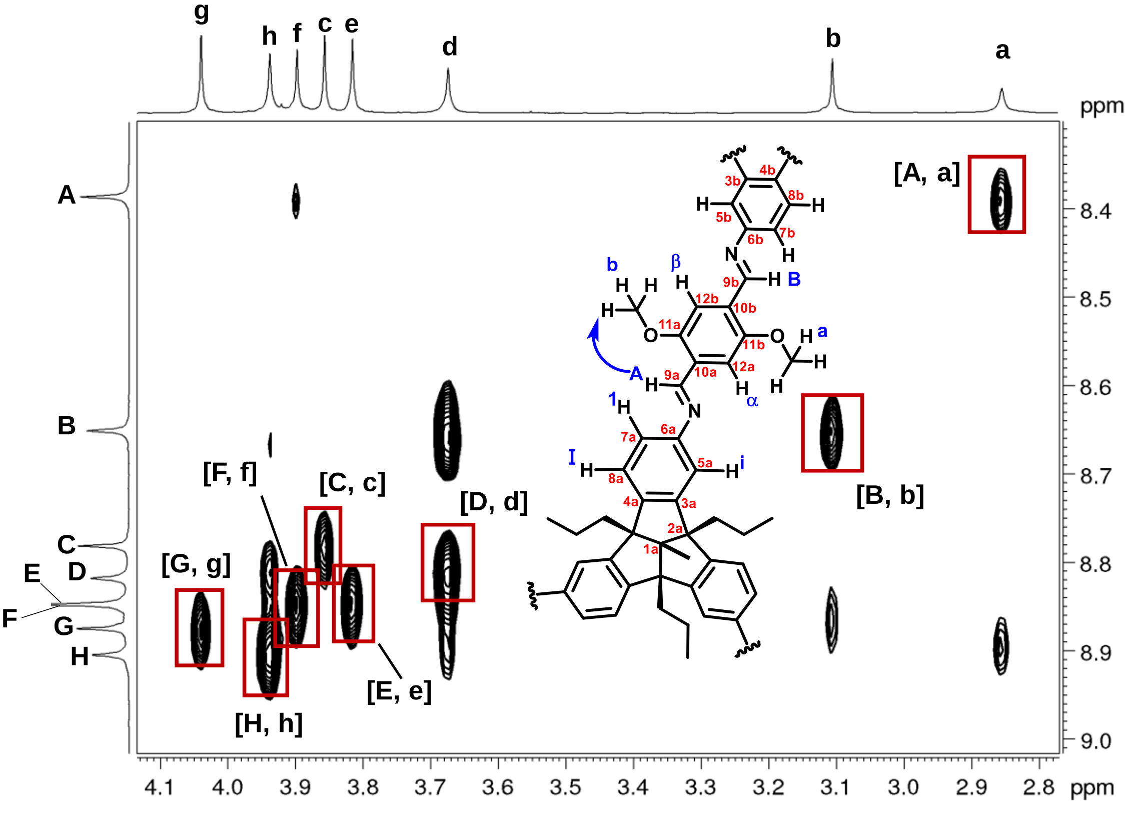

Figure 443. Partial ^{1}H-^{1}H NOESY NMR (700 MHz, CD_2Cl_2) spectrum of **(OMe-cube)$_2$** showing cross peaks between H-(a-h) and H-(A-H). (See Table 17 for chemical shift values).

Table 17. ^1H-^1H NOESY interactions between H-(A-H) and H-(a-h).

^1H-Atom	^1H-NMR (NOESY)	^1H-Atom	^1H-NMR (NOESY)
A	8.39	a	2.85
B	8.65	b	3.11
C	8.78	c	3.86
D	8.82	d	3.67
E	8.85	e	3.82
F	8.85	f	3.90
G	8.87	g	4.04
H	8.90	h	3.94

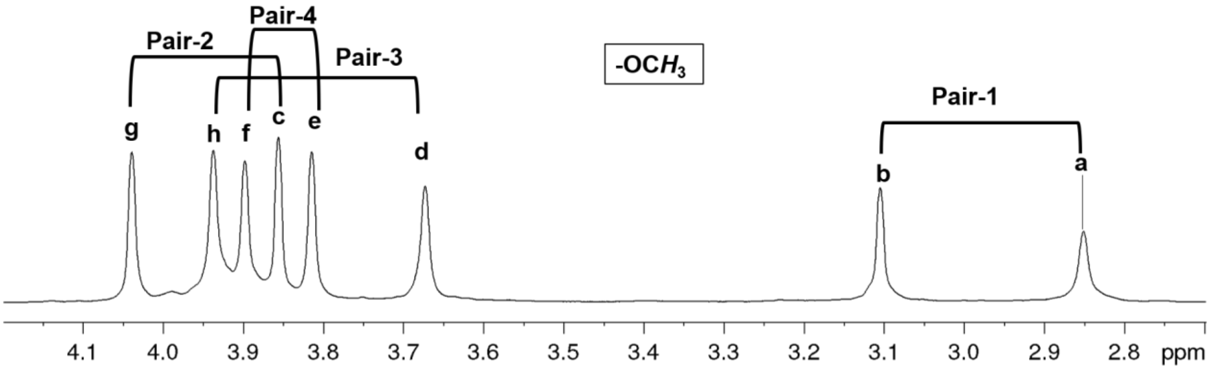

Figure 444. Partial ^1H NMR (700 MHz, CD_2Cl_2) spectrum of **(OMe-cube)₂** showing four pairs of neighboring methoxy protons.

4. The neighboring TBTQ aromatic protons were assigned by HSQC, HMBC and COSY interactions as shown in Figure 445-447.

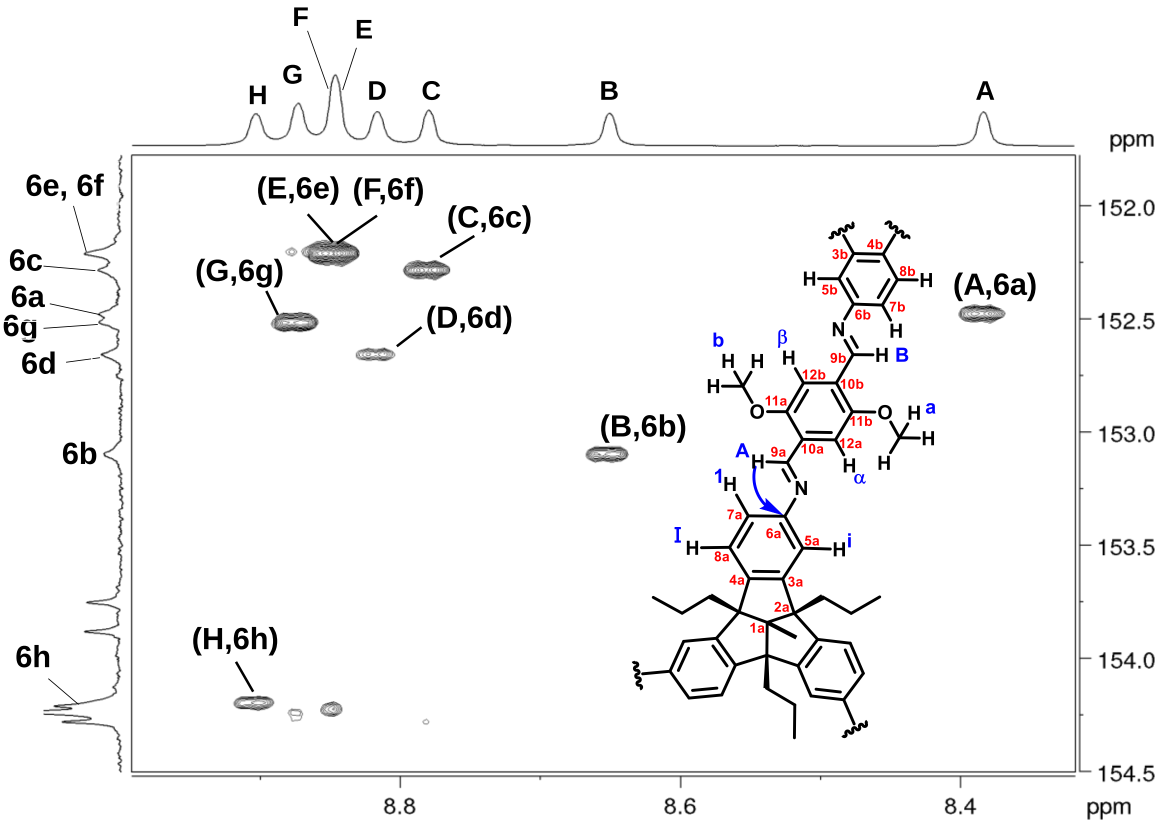

Figure 445. Partial ^{1}H-^{13}C HMBC NMR (700 MHz, CD_2Cl_2) spectrum of **(OMe-cube)$_2$** showing cross peaks between H-(A-H) and C-(6a-6h). (See Table 18 for chemical shift values).

Table 18. ^{1}H-^{1}H HMBC interactions between H-(A-H) and C-(6a-6h).

**^{1}H-Atom**	**^{1}H-NMR (HMBC)**	**^{13}C Atom**	**^{13}C Atom (HMBC)**
A	8.39	6a	152.48
B	8.65	6b	153.09
C	8.78	6c	152.28
D	8.82	6d	152.66
E	8.85	6e	152.21
F	8.85	6f	152.21
G	8.87	6g	152.52
H	8.90	6h	154.21

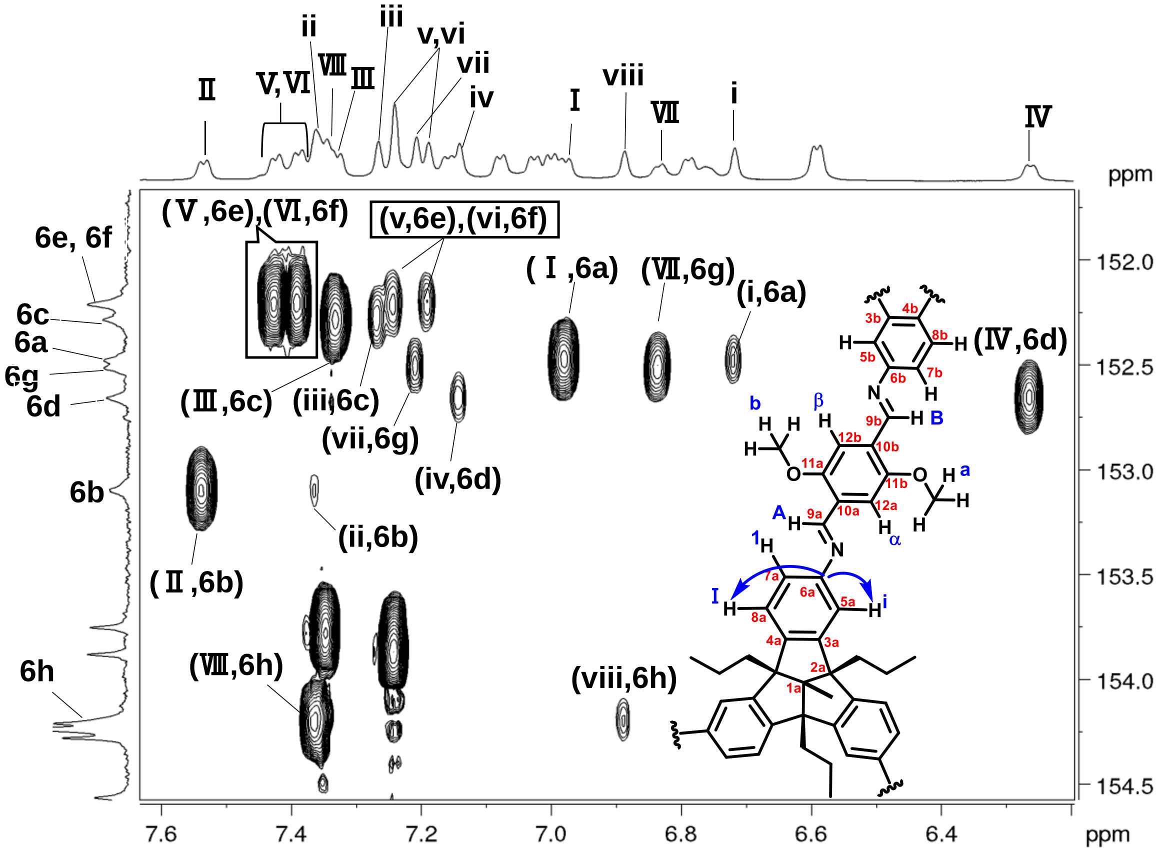

Figure 446. Partial ^{1}H-^{13}C HMBC NMR (700 MHz, CD_2Cl_2) spectrum of **(OMe-cube)$_2$** showing cross peaks for pairs 1) C-(6a-6h) and H-(Ⅰ-Ⅷ) and 2) C-(6a-6h) and H-(i-viii)). (See Table 19 for chemical shift values).

Table 19.^{1}H-^{1}H HMBC interactions between pairs 1) C-(6a-6h) and H-(Ⅰ-Ⅷ) and 2) C-(6a-6h) and H-(i-viii).

^{13}C Atom	^{13}C Atom (HMBC)	^{1}H-Atom	^{1}H-NMR (HMBC)	^{13}C Atom	^{13}C Atom (HMBC)	^{1}H-Atom	^{1}H-NMR (HMBC)
6a	152.48	Ⅰ	6.98	6a	152.48	i	6.72
6b	153.09	Ⅱ	7.54	6b	153.09	ii	7.36
6c	152.28	Ⅲ	7.33	6c	152.28	iii	7.27
6d	152.66	Ⅳ	6.27	6d	152.66	iv	7.14
6e	152.21	Ⅴ	7.39/7.42	6e	152.21	v	7.19/7.24
6f	152.21	Ⅵ	7.39/7.42	6f	152.21	vi	7.19/7.24
6g	152.52	Ⅶ	6.83	6g	152.52	vii	7.21
6h	154.21	Ⅷ	7.35	6h	154.21	viii	6.89

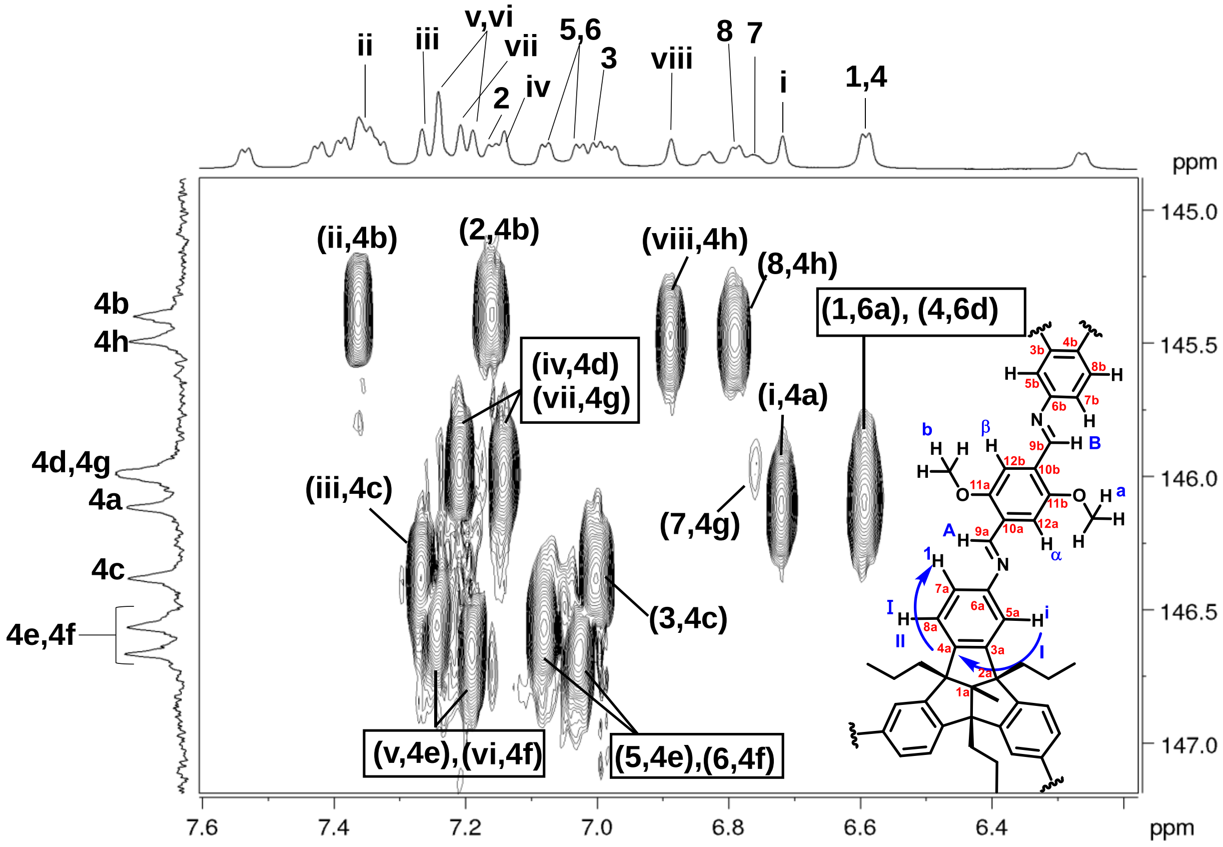

Figure 447. Partial ^{1}H-^{13}C HMBC NMR (700 MHz, CD_2Cl_2) spectrum of **(OMe-cube)₂** showing cross peaks for pairs 1) H-(i-viii) and C-(4a-4h) and 2) C-(4a-4h) and H-(1-8). (To assign the protons 1,4 and 7 clearly ^{1}H-^{1}H COSY spectrum was used). (See Table 20 for chemical shift values).

Table 20. ^{1}H-^{1}H HMBC interactions for pairs 1) H-(i-viii) and C-(4a-4h) 2) C-(4a-4h) and H-(1-8).

^{1}H-Atom	^{1}H-NMR (HMBC)	^{13}C Atom	^{13}C Atom (HMBC)	^{13}C Atom	^{13}C Atom (HMBC)	^{1}H-Atom	^{1}H-NMR (HSQC)
i	6.72	4a	146.11	4a	146.11	1	6.60
ii	7.36	4b	145.49	4b	145.49	2	7.16
iii	7.27	4c	146.38	4c	146.38	3	7.00
iv	7.14	4d	145.99	4d	145.99	4	6.60
v	7.19/7.24	4e	146.56/146.66	4e	146.56/146.66	5	7.06/7.03
vi	7.19/7.24	4f	146.56/146.66	4f	146.56/146.66	6	7.06/7.03
vii	7.21	4g	145.99	4g	145.99	7	6.75
viii	6.89	4h	145.49	4h	145.49	8	6.79

Assignment of Imine protons A and B to the 3D model of (OMe-cube)2

The first imine signal at δ = 8.39 ppm, the most upfield shifted imine peak (labelled as **A)** assigned to the blue imine protons of the dicatenane (Figure 448 and 449) which are found inside the other cube. This imine proton in the catenane with highest shielding effect of the surrounding rings compared to other seven different imine protons of dicatenane. Based on multiple HMBC and HSQC interactions (Figure 435-438, Table 14 and 15), the neighboring imine proton at δ = 8.65 ppm (labelled as **B**) was identified and assigned to the red imine protons of the dicatenane (Figure 448 and 449).

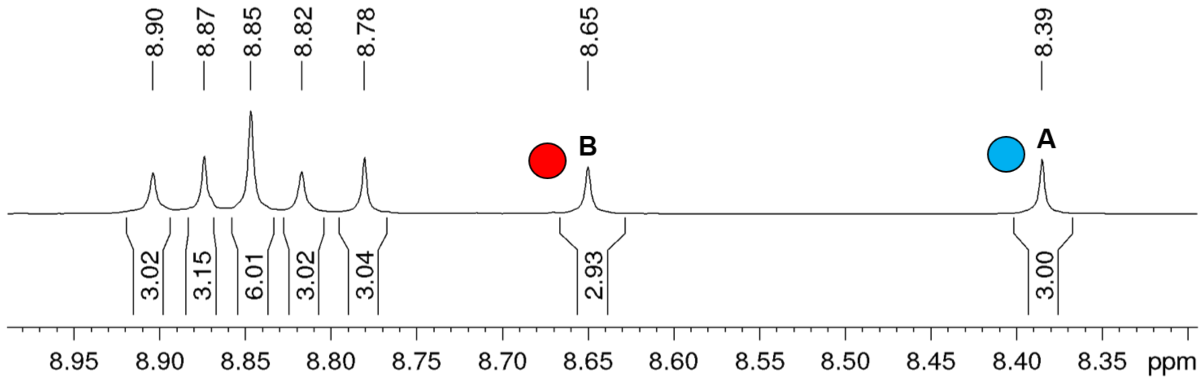

Figure 448. Partial ^{1}H NMR (600 MHz, CD_2Cl_2) spectrum of **(OMe-cube)2** and assignment of imine protons **A** and **B**.

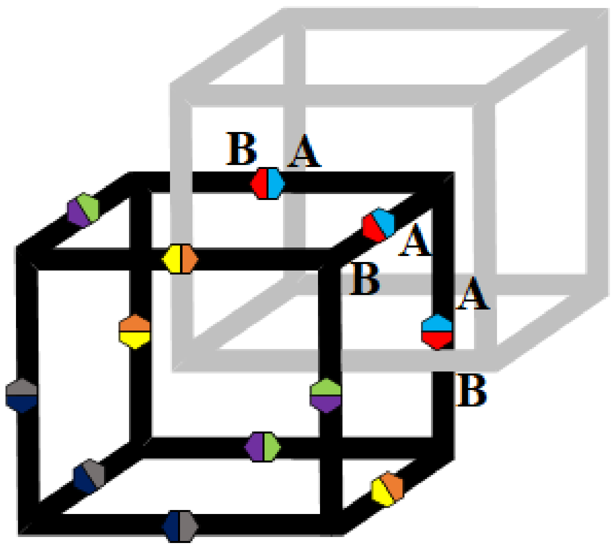

Figure 449. Cartoon representation of one **(OMe-cube)2** showing the assignment of imine protons **A** and **B**.

Assignment of imine protons D and H to the 3D model of (OMe-cube)$_2$

The imine proton **B** (δ = 8.65 ppm) of one cube of the catenane show NOESY cross peaks with the imine proton at δ = 8.90 (labelled as **H)** (Figure 452) and methoxy peak at δ = 3.67 ppm (labelled as **d**) of another cube (Figure 453). Therefore, the imine peak at δ = 8.90 (labelled as **H**) is assigned to the purple imine proton of the dicatenane (Figure 450 and 451). Furthermore, the methoxy peak at δ = 3.67 (labelled as **d**) shows NOESY cross peaks with the methoxy proton at δ = 3.11 (labelled as **b**) (Figure 454) further support the assignment of imine proton **H**. Based on multiple HSQC and HMBC interactions (Figure 435-438, Table 14 and 15), the neighboring imine proton at δ = 8.82 ppm (labelled as **D**) was identified and assigned to the lime green proton of the dicatenane (Figure 450 and 451).

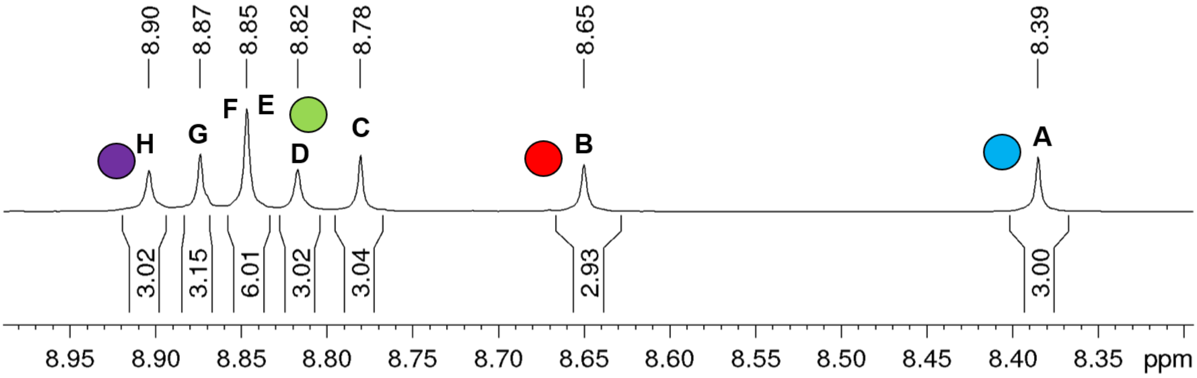

Figure 450. Partial ^{1}H NMR (600 MHz, CD_2Cl_2) spectrum of **(OMe-cube)$_2$** and assignment of imine protons **D** and **H**.

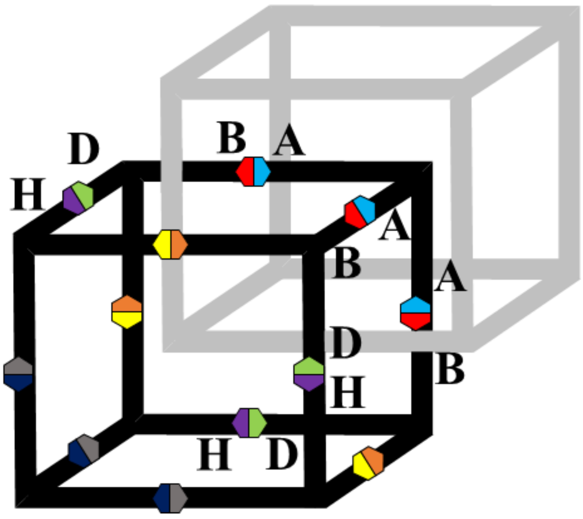

Figure 451. Cartoon representation of **(OMe-cube)$_2$** showing the assignment of imine protons **D** and **H**.

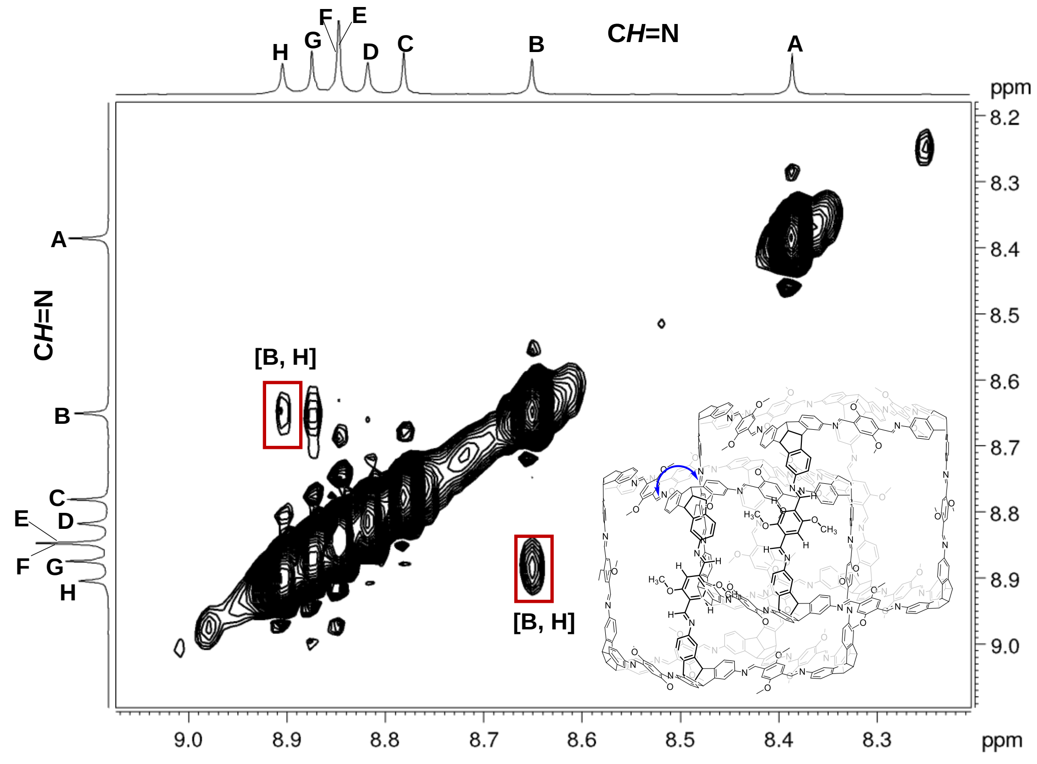

Figure 452. Partial ^{1}H-^{1}H NOESY NMR (600 MHz, CD_2Cl_2) spectrum showing cross peaks between imine protons

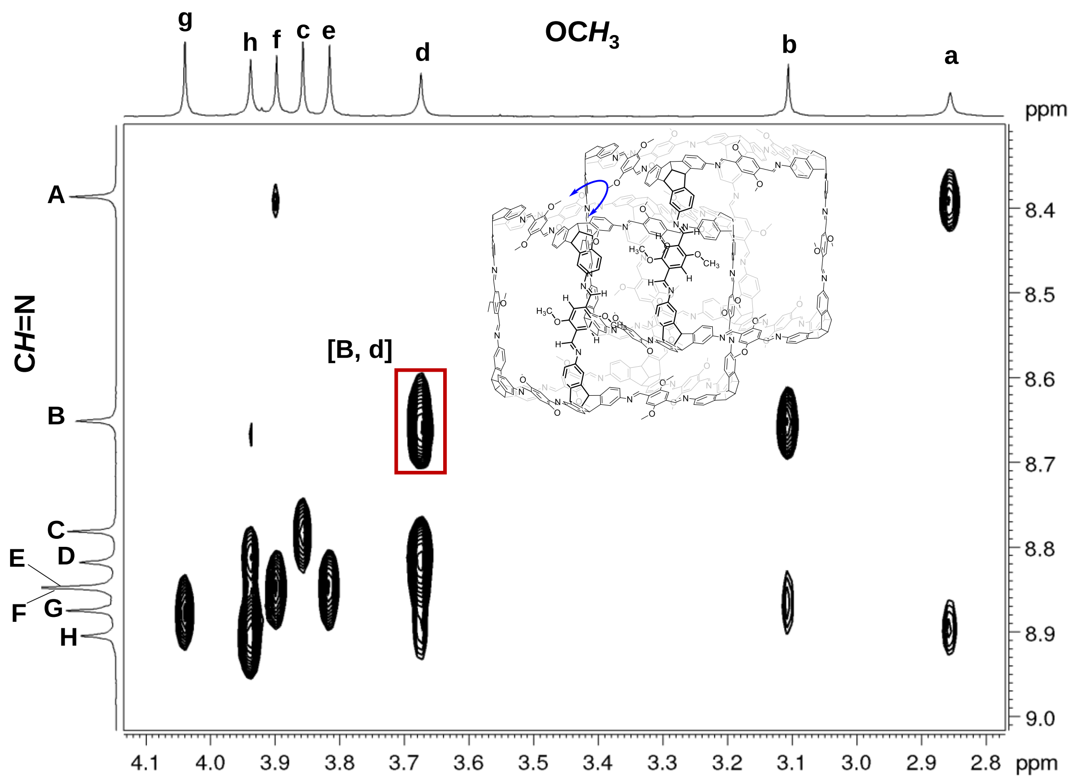

Figure 453. Partial ^{1}H-^{1}H NOESY NMR (600 MHz, CD_2Cl_2) spectrum showing cross peaks between methoxy protons H-(a-h) and imine protons H-(A-H).

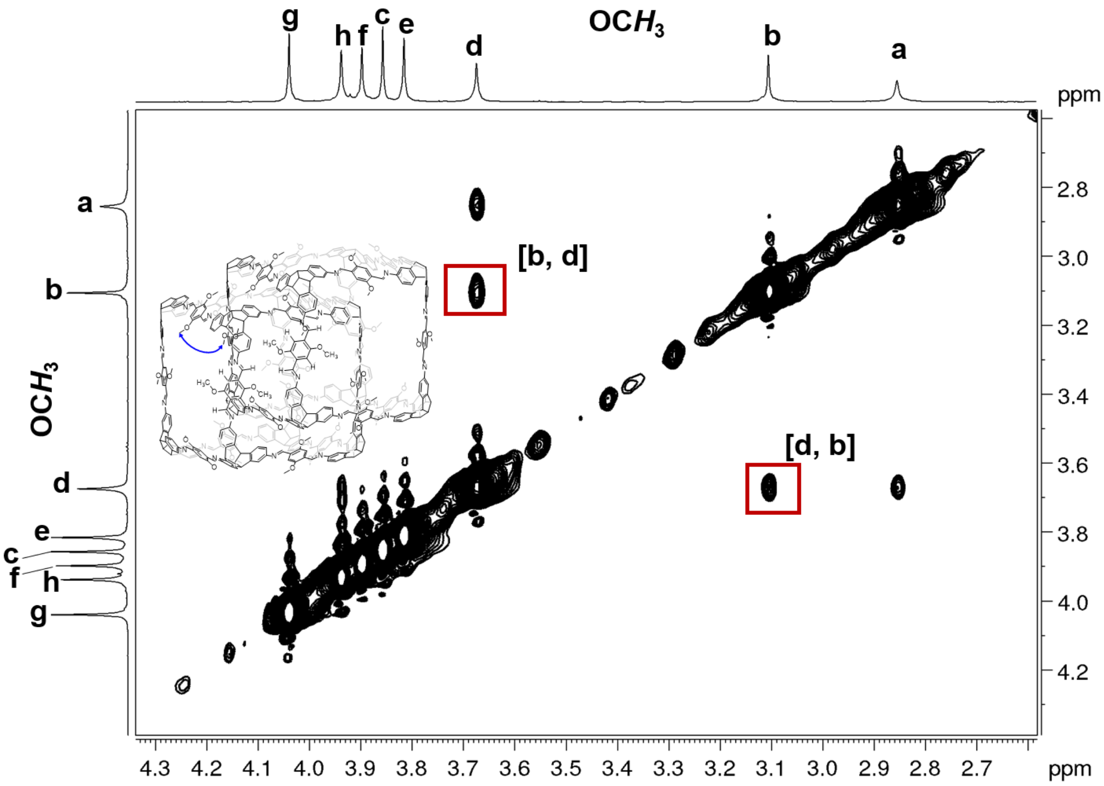

Figure 454. Partial ^{1}H-^{1}H NOESY NMR (600 MHz, CD_2Cl_2) spectrum showing cross peaks between methoxy protons.

Assignment of imine protons C and G to the 3D model of (OMe-cube)₂

The triply interlocked model of dicatenane shows that the chemical environment of the pair of purple imine proton **H** (δ = 8.90 ppm) and lime imine proton **D** (δ = 8.82 ppm) is more similar to the pair of yellow and orange imine protons than the pair of grey and dark blue imine protons (Figure 456). Therefore, downfield shifted imine signal at δ = 8.87 ppm (labelled as **G**) is assigned to the yellow imine proton of the dicatenane and upfield shifted imine peak at δ = 8.78 (labelled as **C**) is assigned to the orange imine proton of the dicatenane (Figure 455 and 456). Multiple interactions from HMBC and HSQC (Figure 435-438, Table 14 and 15) also shows that these both protons are the neighboring imine protons from the same pair.

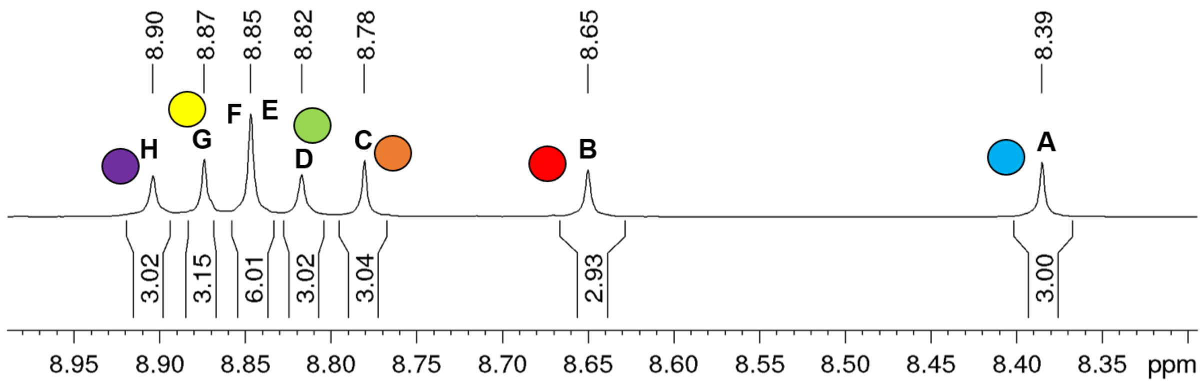

Figure 455. Partial ^{1}H NMR (600 MHz, CD_2Cl_2) spectrum of **(OMe-cube)$_2$** and assignment of proton **G** and **C**.

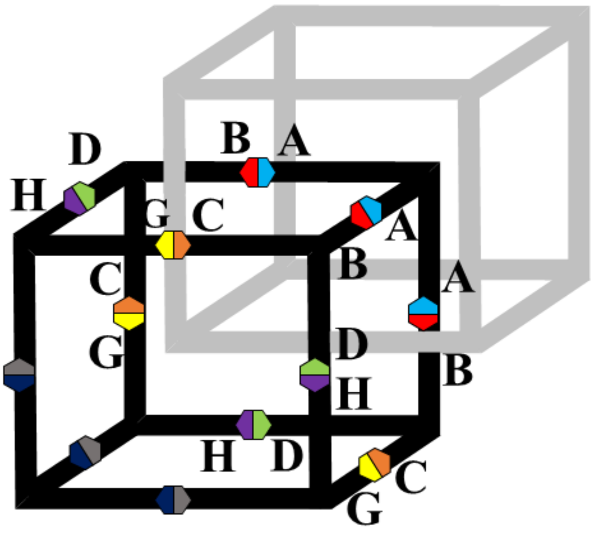

Figure 456. Cartoon representation of **(OMe-cube)$_2$** showing the assignment of imine protons **G** and **C**.

Assignment of imine protons E and F to the 3D model of (OMe-cube)$_2$

For the assignment of protons **E** and **F**, NOE interactions of the highly shielded aliphatic protons of the propyl chains of the TBTQ units that are in direct connection to imine proton **A** were investigated. Figure 459 shows NOESY cross peaks of aliphatic protons of the propyl chain at δ = 0.48 ppm with methoxy protons at δ = 3.82 ppm. Based on this interaction, the methoxy peak at δ = 3.82 (labelled as **e**) assigned to the grey methoxy proton of the dicatenane. The methoxy proton **e** shows a NOESY cross peaks with the imine proton at δ = 8.85 (labelled as **E**) (See Figure 443). Therefore, the imine proton at δ = 8.85 (labelled as **E**) is assigned to the grey imine protons (Figure 457 and 458). Based on multiple HMBC and HSQC interactions, the imine peak at δ = 8.85 ppm (labelled as **F**) is assigned to the dark blue proton of the catenane (see Figure 435-438, Table 14 and 15). This is the last pair of neighbouring imine protons. Figure 460 shows NOESY cross peaks of shielded protons of aliphatic chains with the linker aromatic proton ε at δ = 7.69 further support the assignment of imine protons **E** and **F**.

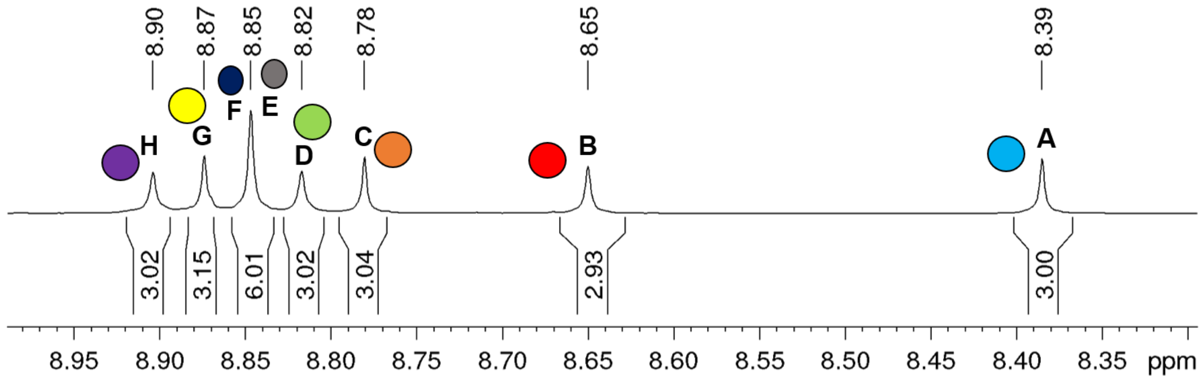

Figure 457. Partial ^{1}H NMR (600 MHz, CD_2Cl_2) spectrum of **(OMe-cube)$_2$** and assignment of protons **E** and **F**.

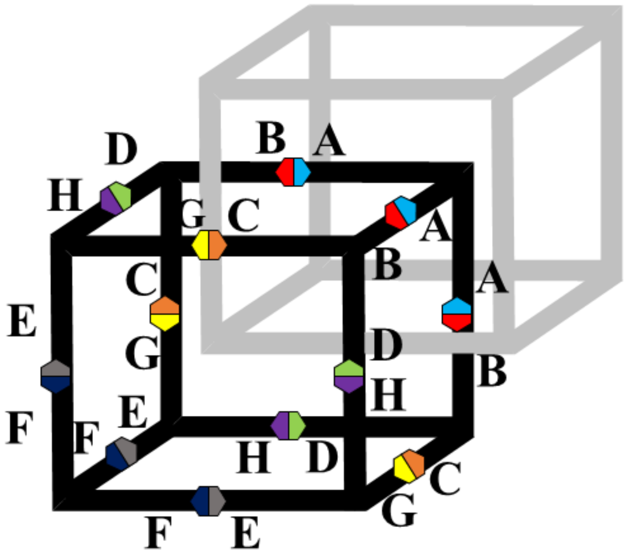

Figure 458. Cartoon representation of **(OMe-cube)$_2$** showing the assignment of linker protons **E** and **F**.

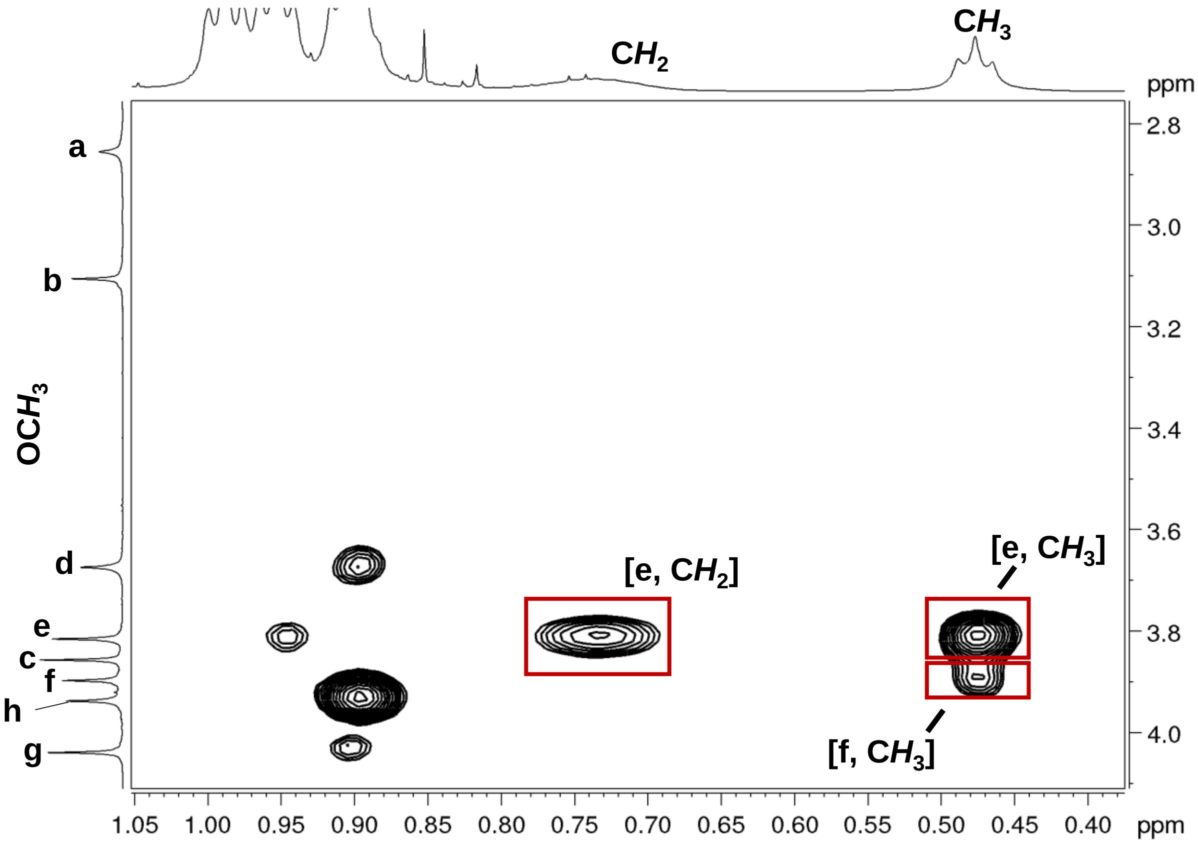

Figure 459. Partial ^{1}H-^{1}H NOESY NMR (600 MHz, CD_2Cl_2) spectrum showing cross peaks between methoxy and aliphatic protons.

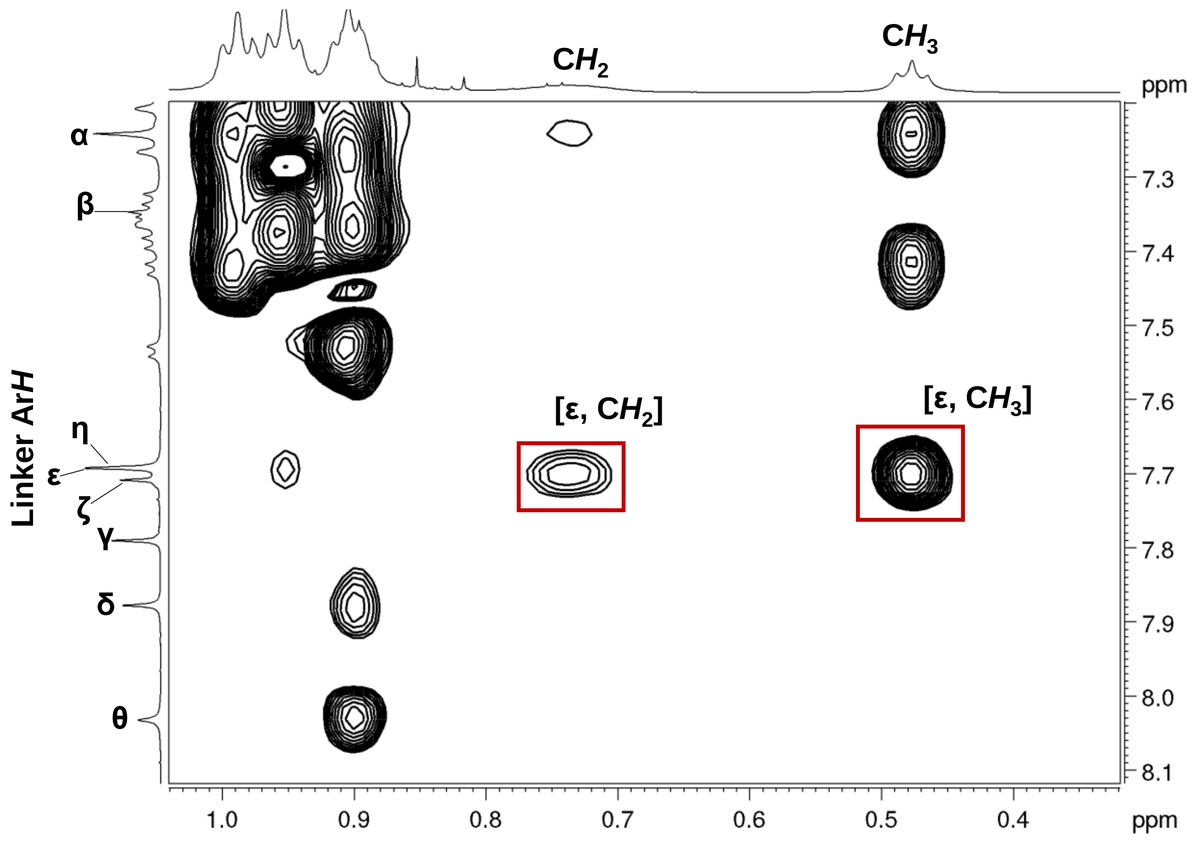

Figure 460. Partial ^{1}H-^{1}H NOESY NMR (600 MHz, CD_2Cl_2) spectrum showing cross peaks between the aliphatic protons and aldehyde linker protons.

Table 21. Assignment of imine, methoxy and linker protons of $(OMe\text{-}cube)_2$

-N=C*H*	δ (ppm)	OCH_3	δ (ppm)	Ar*H* (linker)	δ (ppm)
**A**	8.39	**a**	2.85	**α**	7.24
**B**	8.65	**b**	3.11	**β**	7.34
**C**	8.78	**c**	3.86	**γ**	7.79
**D**	8.82	**d**	3.67	**δ**	7.88
**E**	8.85	**e**	3.82	**ε**	7.69
**F**	8.85	**f**	3.90	**ζ**	7.71
**G**	8.87	**g**	4.04	**η**	7.69
**H**	8.90	**h**	3.94	**θ**	8.03

Table 22. Assignment of TBTQ aromatic protons

TBTQ-ArH_i	δ (ppm)	TBTQ-ArH_1	δ (ppm)	TBTQ-ArH_I	δ (ppm)
i	6.72	**1**	6.60	I	6.98
ii	7.36	**2**	7.16	II	7.54
iii	7.27	**3**	7.00	III	7.33
iv	7.14	**4**	6.60	IV	6.27
v	7.19/7.24	**5**	7.06/7.03	V	7.39/7.42
vi	7.19/7.24	**6**	7.06/7.03	VI	7.39/7.42
vii	7.21	**7**	6.75	VII	6.83
viii	6.89	**8**	6.79	VIII	7.35

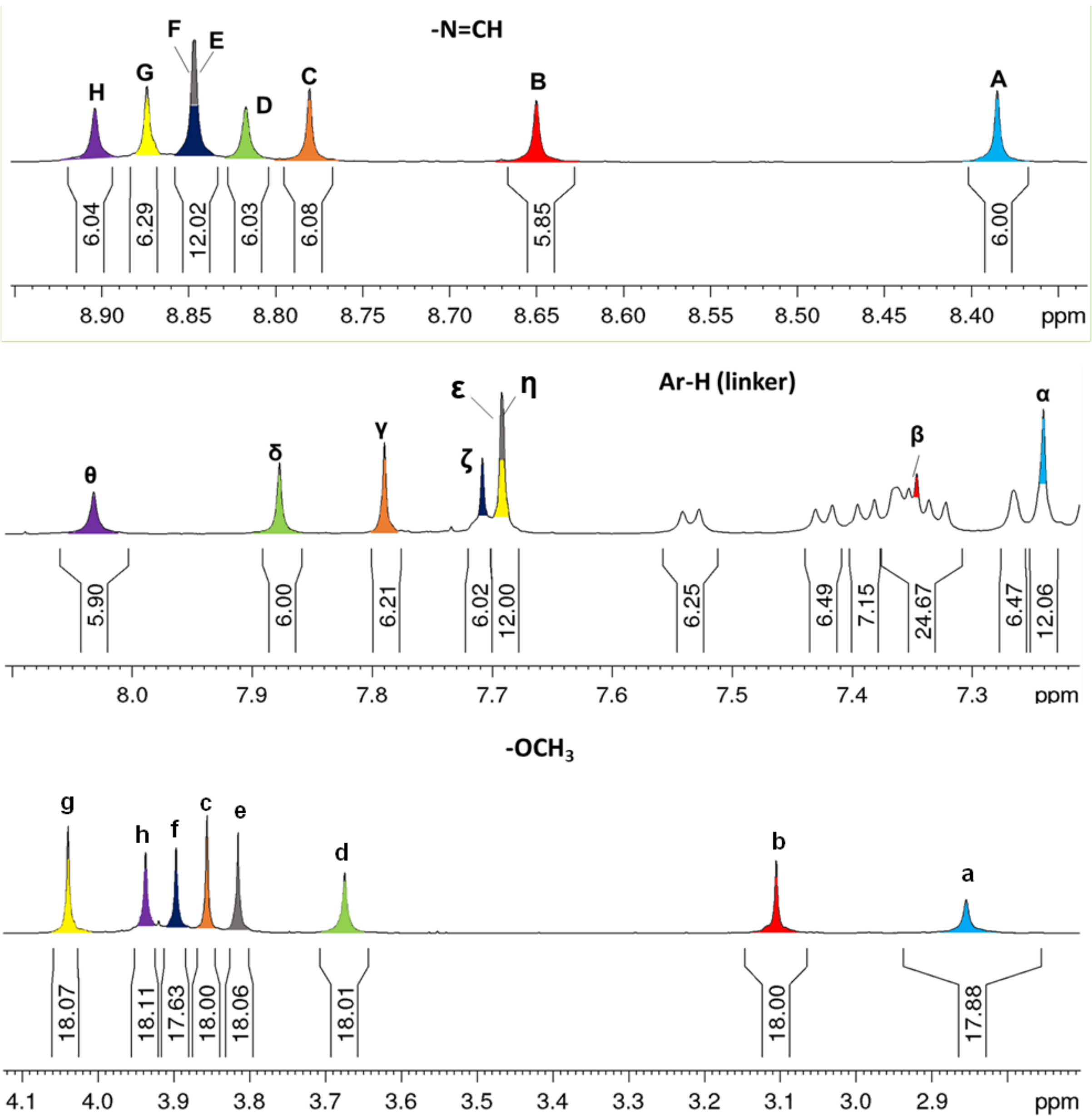

Figure 461. Partial ^{1}H NMR (600 MHz, CD_2Cl_2) spectrum of **(OMe-cube)$_2$** and assignment of imine, methoxy and linker protons.

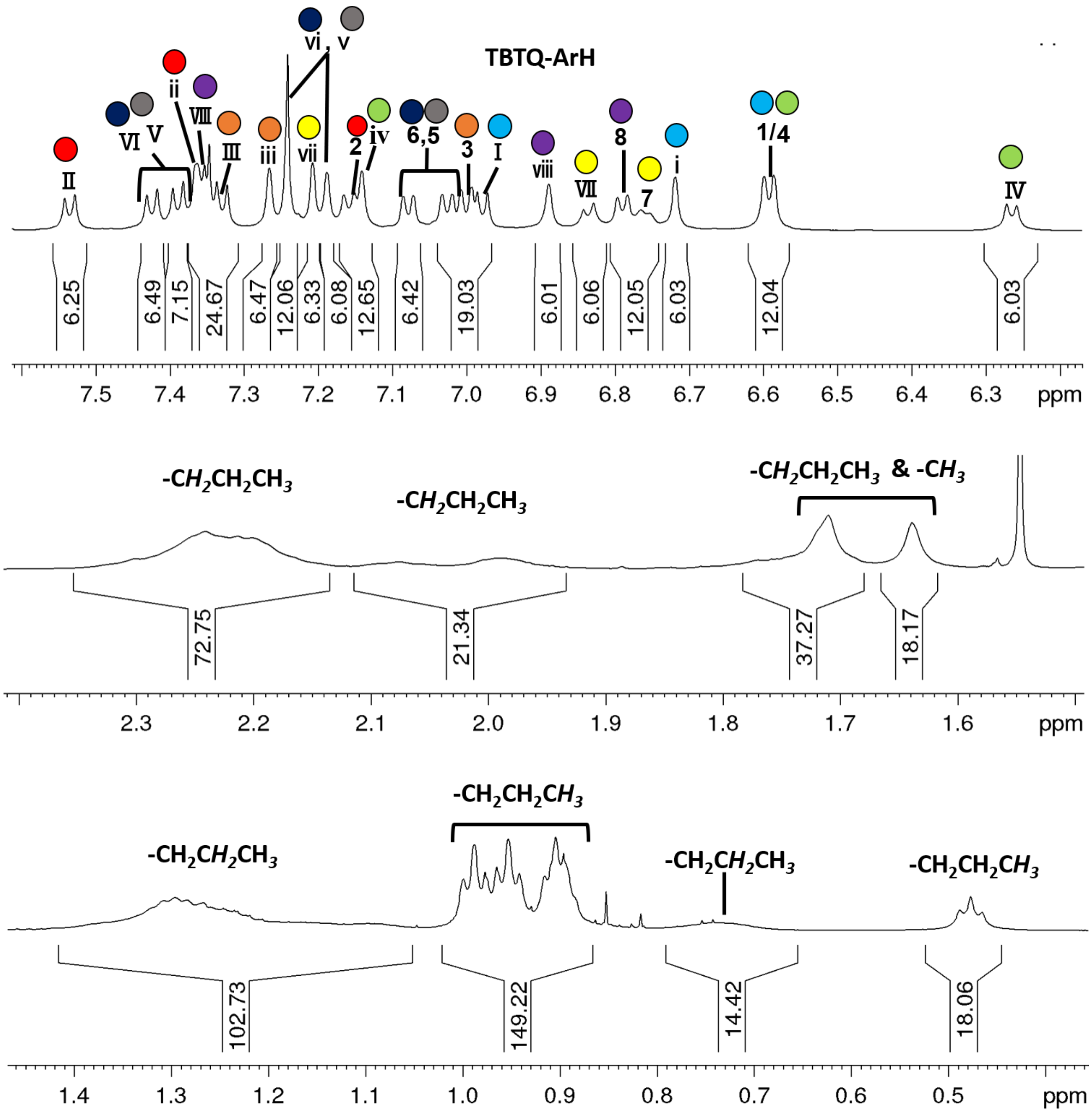

Figure 462. Partial ^{1}H NMR (600 MHz, CD_2Cl_2) spectrum of **(OMe-cube)$_2$** and assignment of TBTQ and aliphatic protons.

b) Assignment of protons and carbons of (SMe-cube)2

^{1}H NMR, 700 MHz, CD_2Cl_2

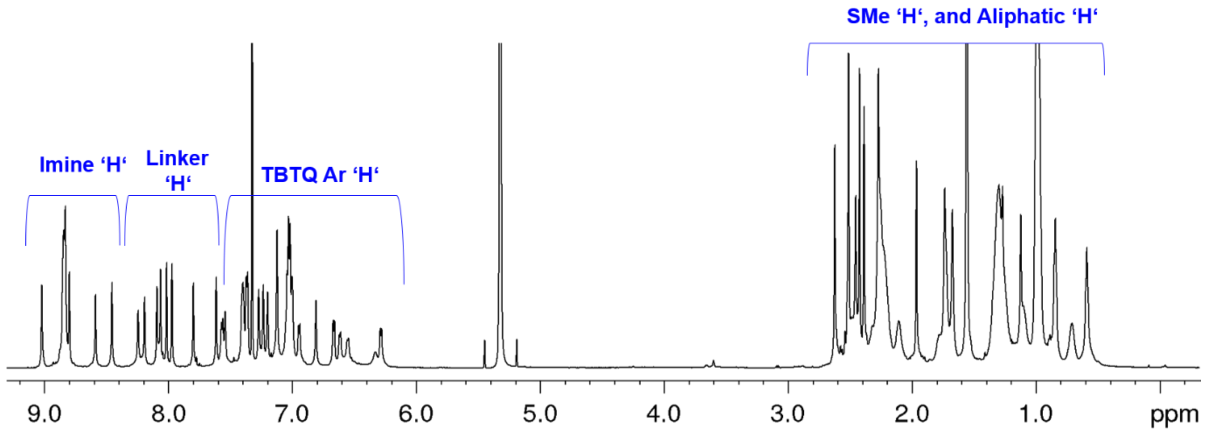

Figure 463. ^{1}H NMR spectrum (700 MHz, CD_2Cl_2) of **(SMe-cube)2**. (The protons were assigned by 1D and 2D NMR spectra).

^{13}C NMR, 700 MHz, CD_2Cl_2

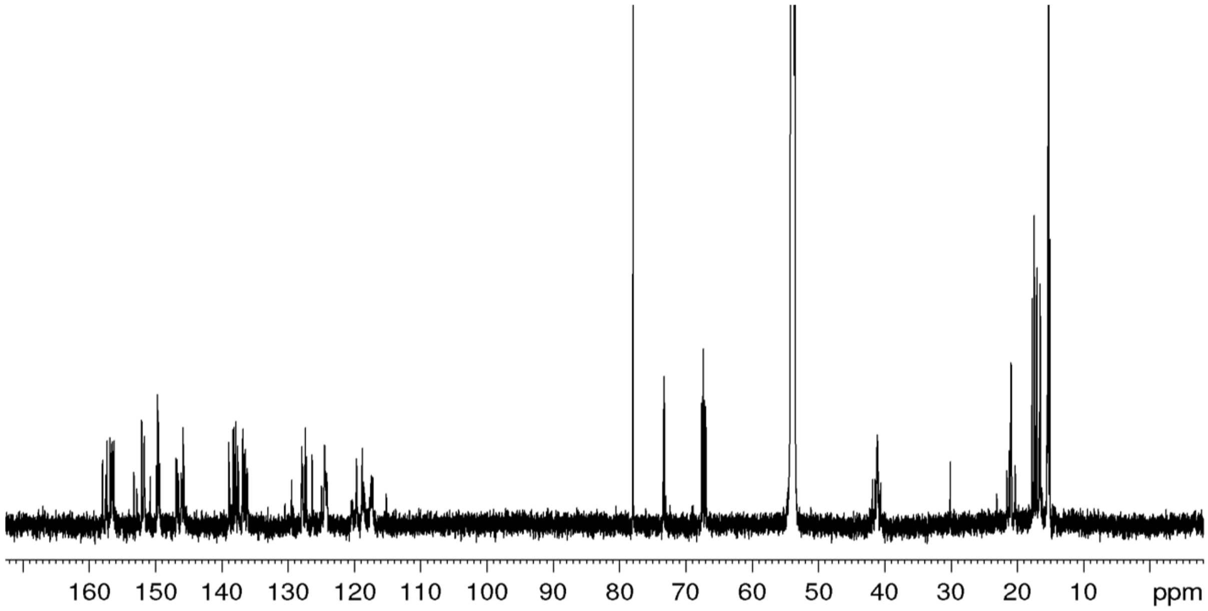

Figure 464. ^{13}C NMR spectrum (700 MHz, CD_2Cl_2) of **(SMe-cube)2**.

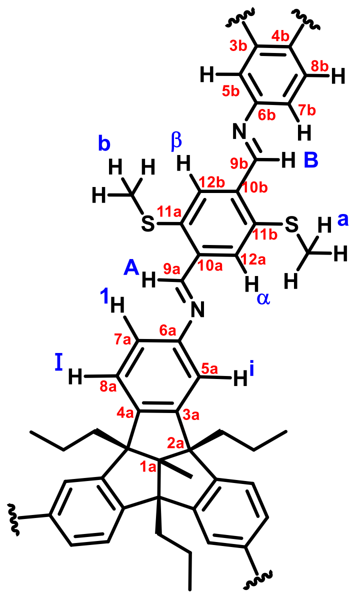

Figure 465. Labelling of protons and carbons to the partial chemical structure of **(SMe-cube)**$_2$.

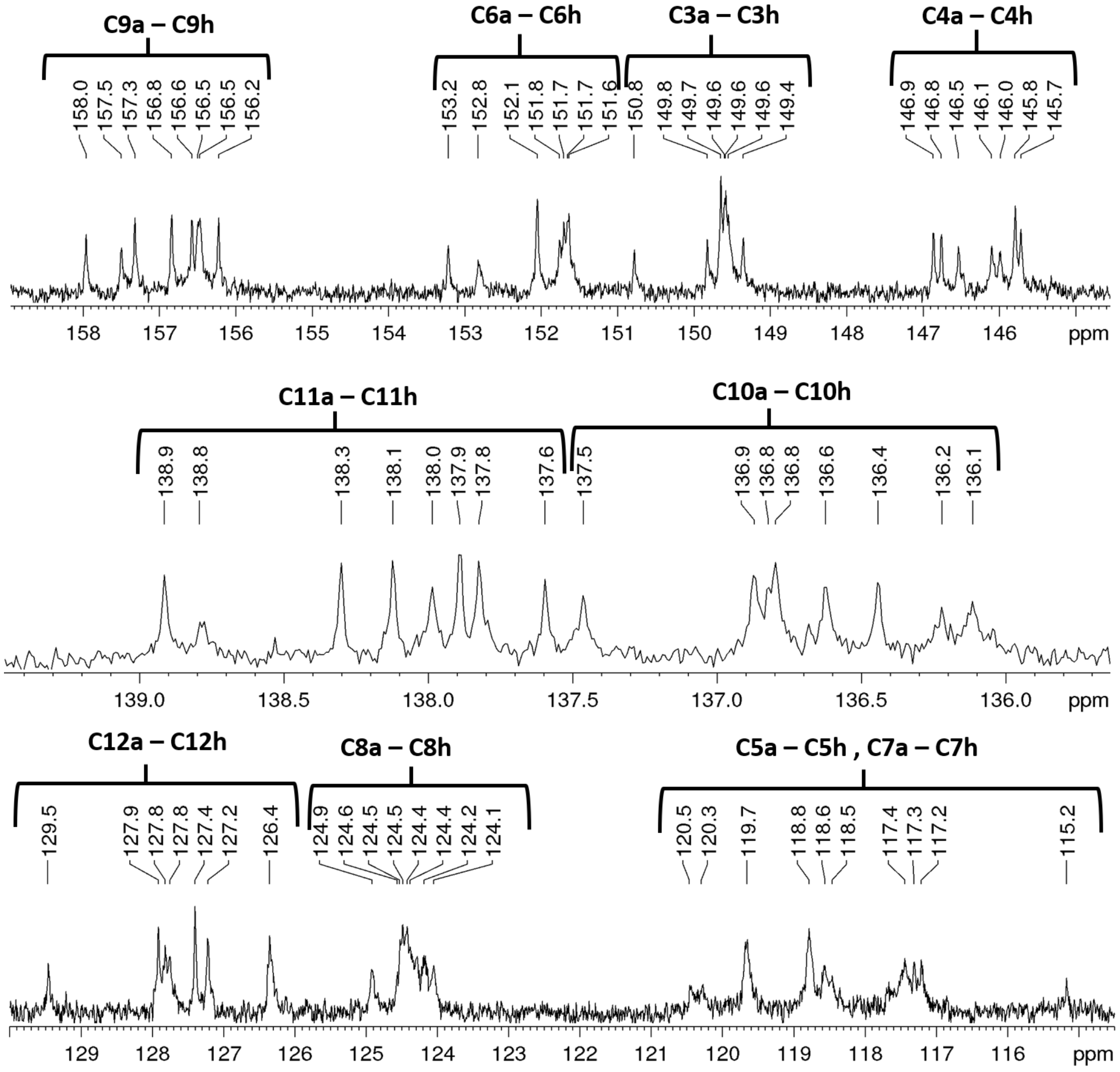

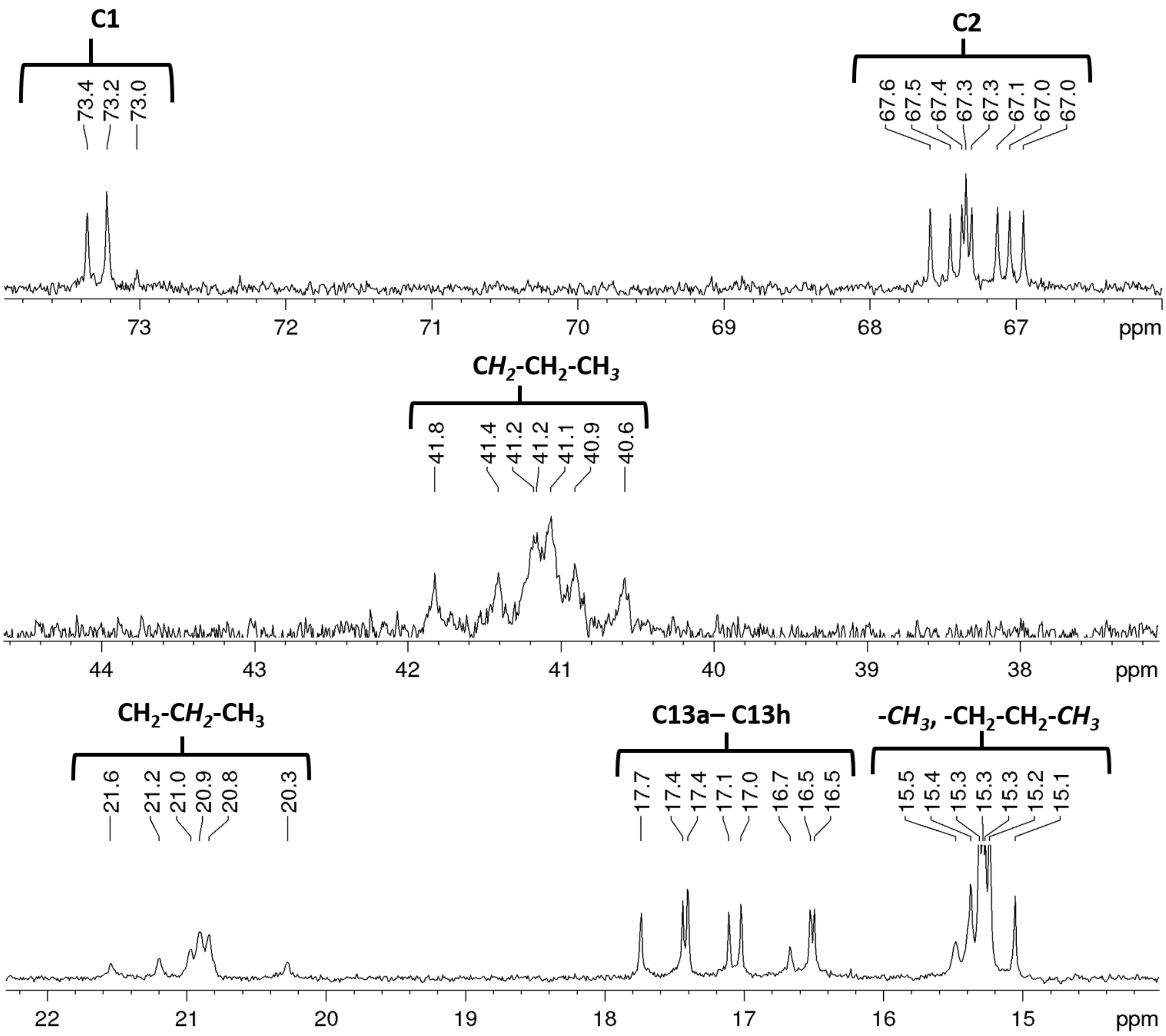

Figure 466. Partial ^{13}C NMR spectrum (700 MHz, CD_2Cl_2) of **(SMe-cube)$_2$**. (All the carbon regions were assigned by HSQC, HMBC, DEPT).

Assignment of eight different signals of dialdehyde protons to the 3D model of (SMe-cube)$_2$

1. First, dialdehyde linker protons were labelled as α, β, γ, δ, ε, ζ, η and θ from lower chemical shift values to higher chemical shift values (Figure 467).

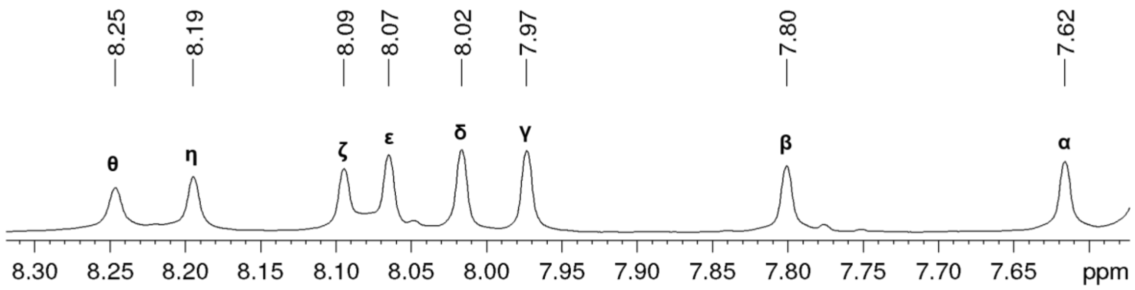

Figure 467. Partial ^{1}H NMR (700 MHz, CD_2Cl_2) spectrum of **(SMe-cube)$_2$** showing the labelling to dialdehyde linker protons.

2. Four pairs of neighboring dialdehyde linker protons and imine protons as shown in Figure 468 were assigned by multiple HSQC and HMBC interactions (see Figure 469-472, Table 23 and 24).

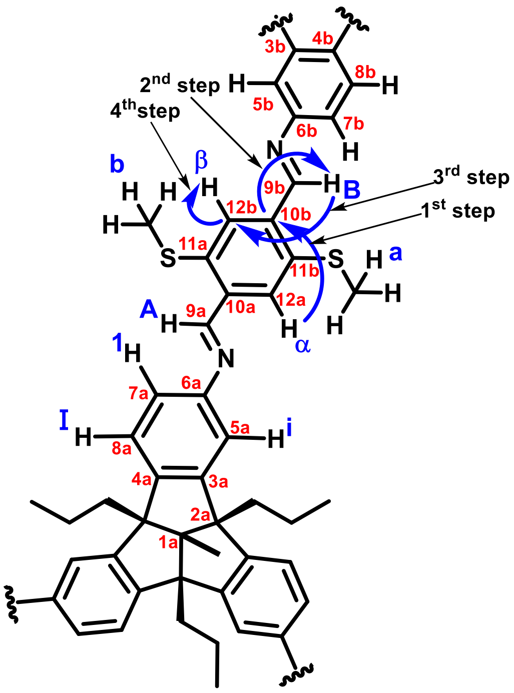

Figure 468. Labelling of protons and carbons of **(SMe-cube)$_2$**. (Arrows indicate how neighboring linker and imine protons are identified by HMBC and HSQC measurements).

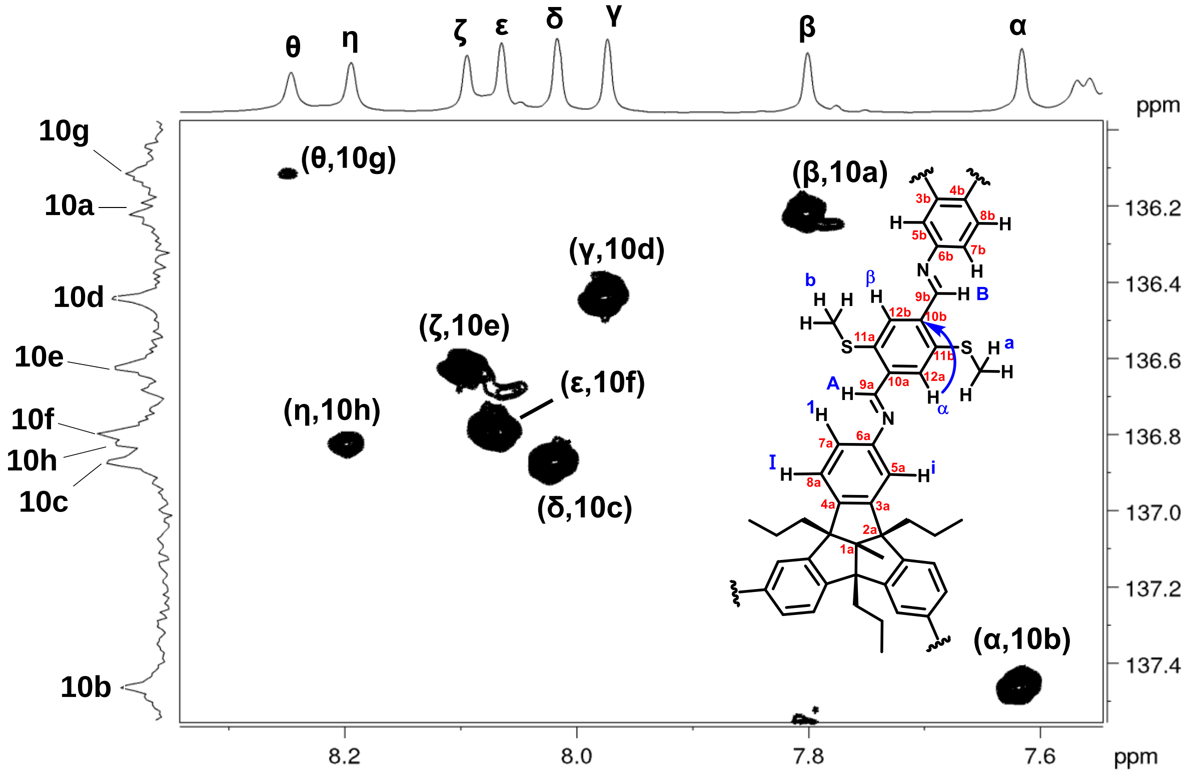

Figure 469. Partial ^{1}H-^{13}C HMBC NMR (700 MHz, CD_2Cl_2) showing cross peaks between H-(a- θ) and C-(10a-10h).

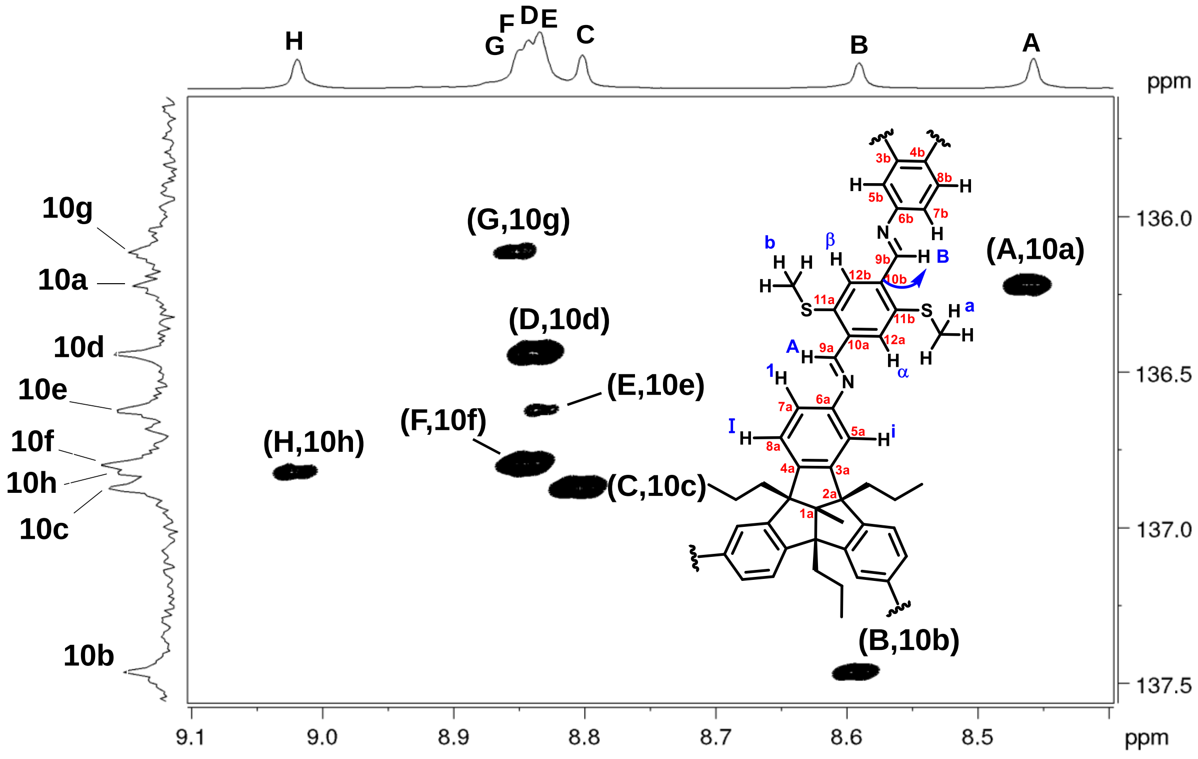

Figure 470. Partial ^{1}H-^{13}C HMBC NMR (700 MHz, CD_2Cl_2) showing cross peaks between C-(10a-10h) and H-(A-H).

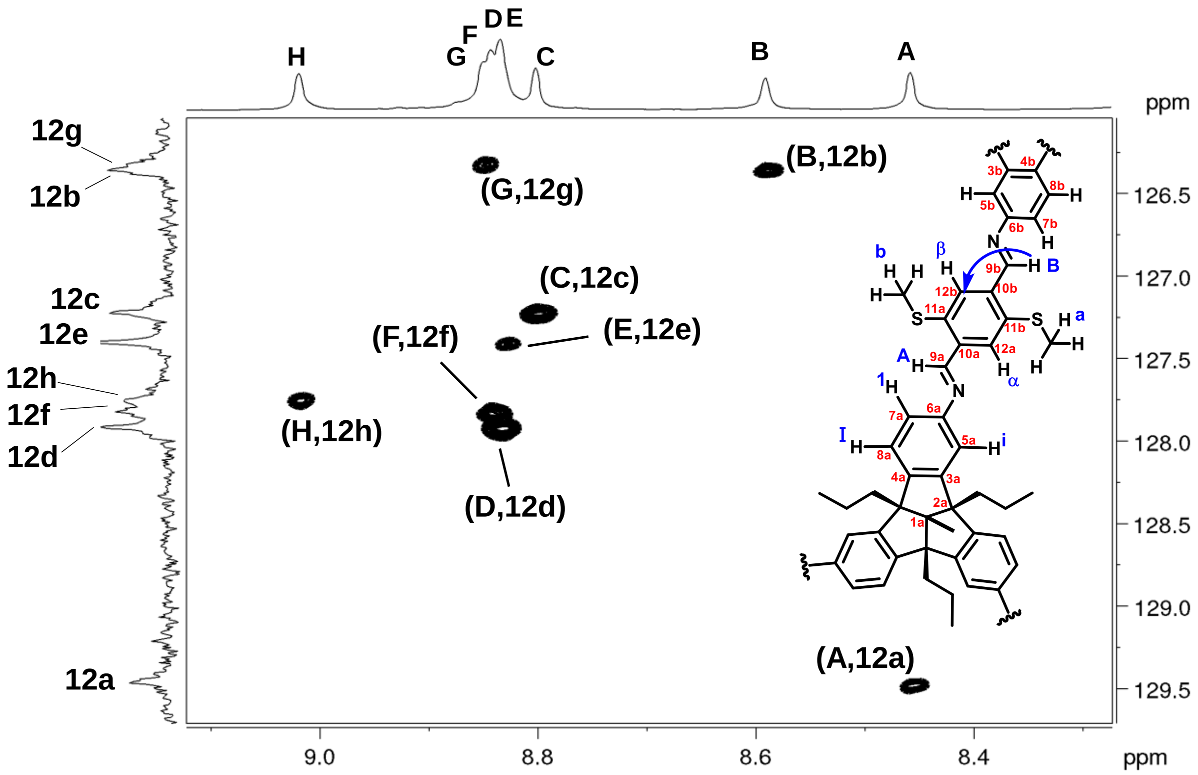

Figure 471. Partial ^{1}H-^{13}C HMBC NMR (700 MHz, CD_2Cl_2) showing cross peaks between H-(A-H) and C-(12a-12h).

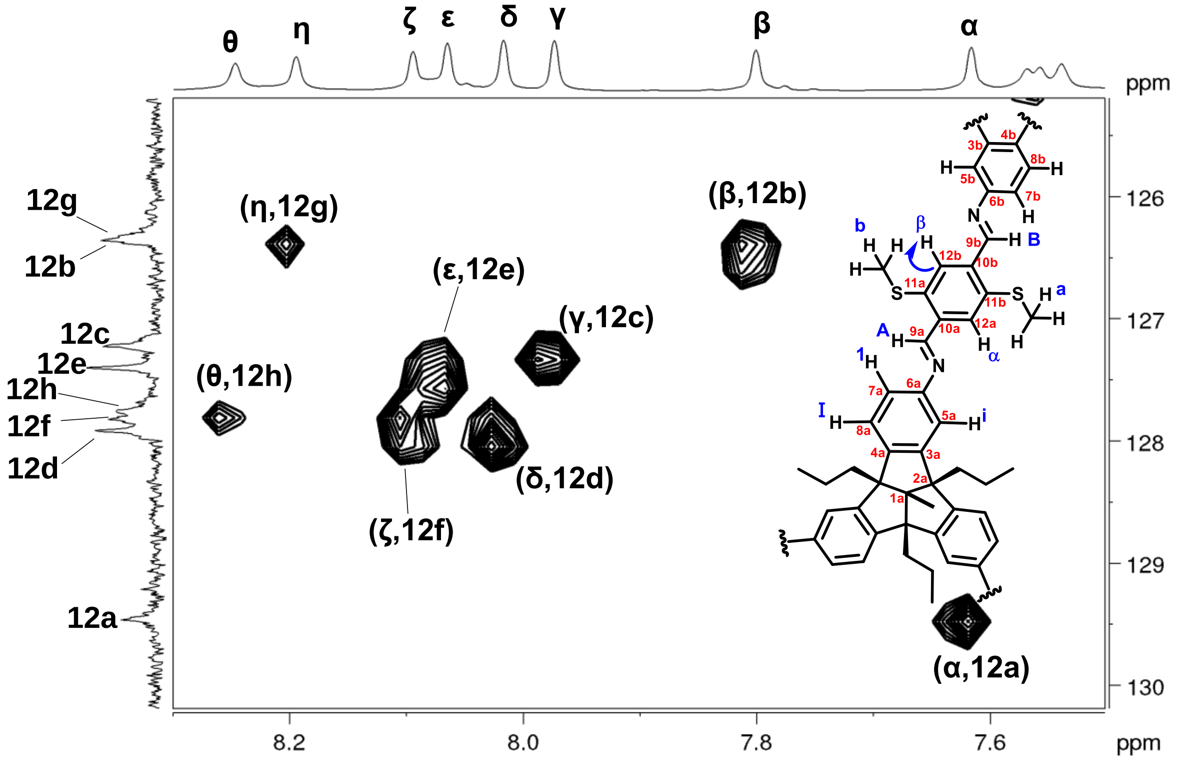

Figure 472. Partial ^{1}H-^{13}C HSQC NMR (700 MHz, CD_2Cl_2) showing cross peaks between C-(12a-12h) and H-(a- θ).

Table 23. HMBC interactions between H-(α-θ) and C-(10a-10h), and C-(10a-10h) and H-(A-H).

^{1}H-Atom	^{1}H-NMR (HMBC)	^{13}C Atom	^{13}C Atom (HMBC)	^{13}C Atom	^{13}C Atom (HMBC)	^{1}H-Atom	^{1}H-NMR (HMBC)
α	7.62	10b	137.46	10b	137.46	B	8.59
β	7.80	10a	136.22	10a	136.22	A	8.46
γ	7.98	10d	136.44	10d	136.44	D	8.84
δ	8.02	10c	136.87	10c	136.87	C	8.80
ε	8.07	10f	136.80	10f	136.80	F	8.845
ζ	8.10	10e	136.63	10e	136.63	E	8.84
η	8.20	10h	136.82	10h	136.82	H	9.02
θ	8.25	10g	136.11	10g	136.11	G	8.85

Table 24. HMBC interactions between H-(A-H) and C-(12a-12h), and HSQC interactions between C-(12a-12h) and H-(α-θ).

^{1}H-Atom	^{1}H-NMR (HMBC)	^{13}C Atom	^{13}C Atom (HMBC)	^{13}C Atom	^{13}C Atom (HMBC)	^{1}H-Atom	^{1}H-NMR (HSQC)
B	8.59	12b	126.37	12b	126.37	β	7.80
A	8.46	12a	129.47	12a	129.47	α	7.62
D	8.84	12d	127.94	12d	127.94	δ	8.02
C	8.80	12c	127.24	12c	127.24	γ	7.98
F	8.845	12f	127.84	12f	127.84	ζ	8.10
E	8.84	12e	127.42	12e	127.42	ε	8.07
H	9.02	12h	127.77	12h	127.77	θ	8.25
G	8.85	12g	126.37	12g	126.37	η	8.20

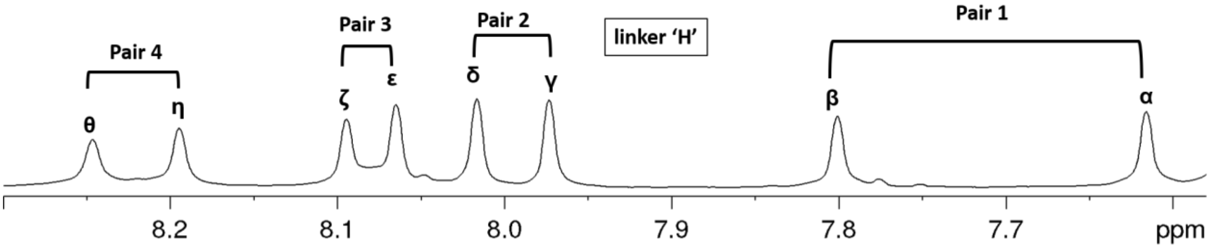

Figure 473. Partial ^{1}H NMR (700 MHz, CD_2Cl_2) spectrum of **(SMe-cube)$_2$** showing four pairs of neighboring linker protons.

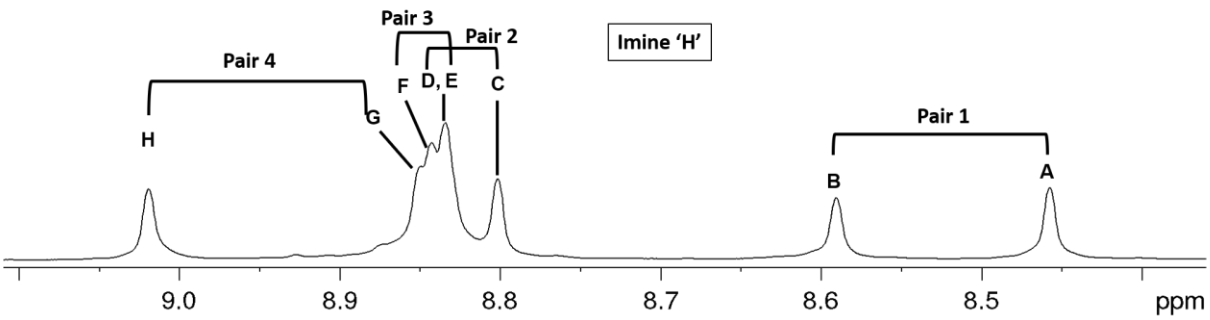

Figure 474. Partial ^{1}H NMR (700 MHz, CD_2Cl_2) spectrum of **(SMe-cube)$_2$** showing four pairs of neighboring imine protons.

3. Four neighboring methoxy protons were assigned by HSQC and HMBC interactions as shown in Figure 475 and 476 (Table 25).

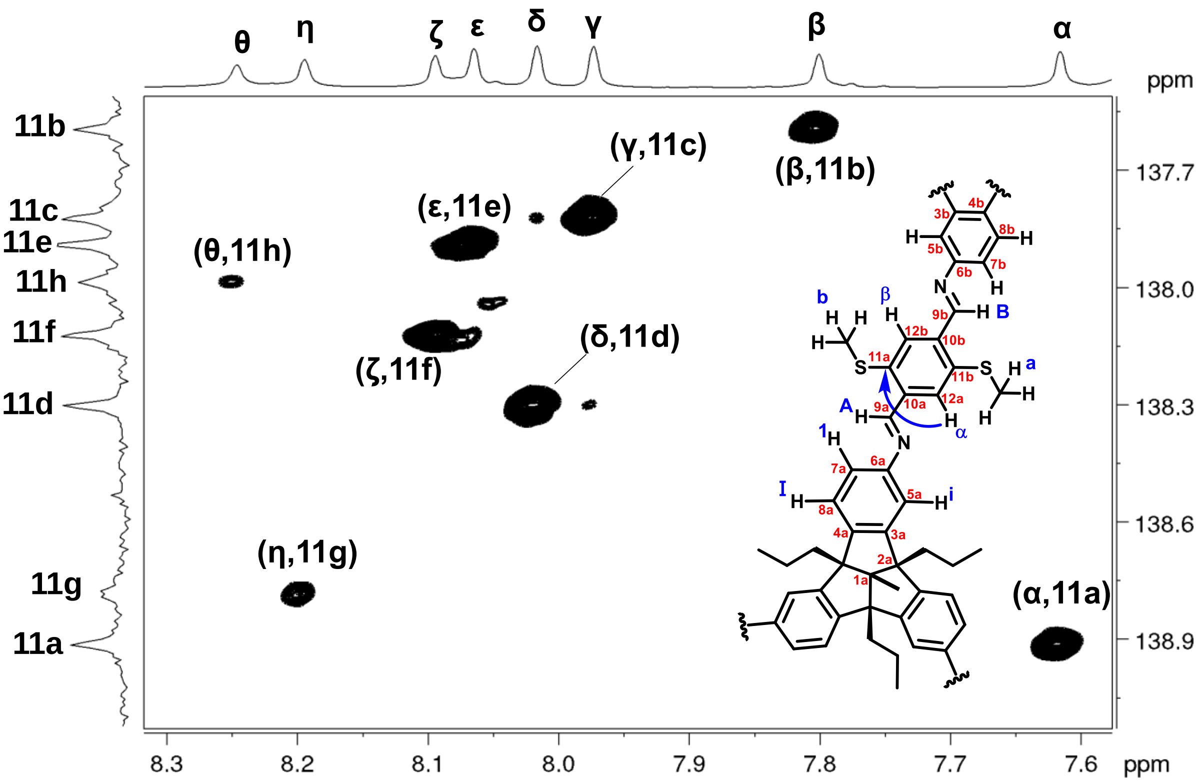

Figure 475. Partial ^{1}H-^{13}C HMBC NMR (700 MHz, CD_2Cl_2) showing cross peaks between H-(a- θ) and C-(11a-11h).

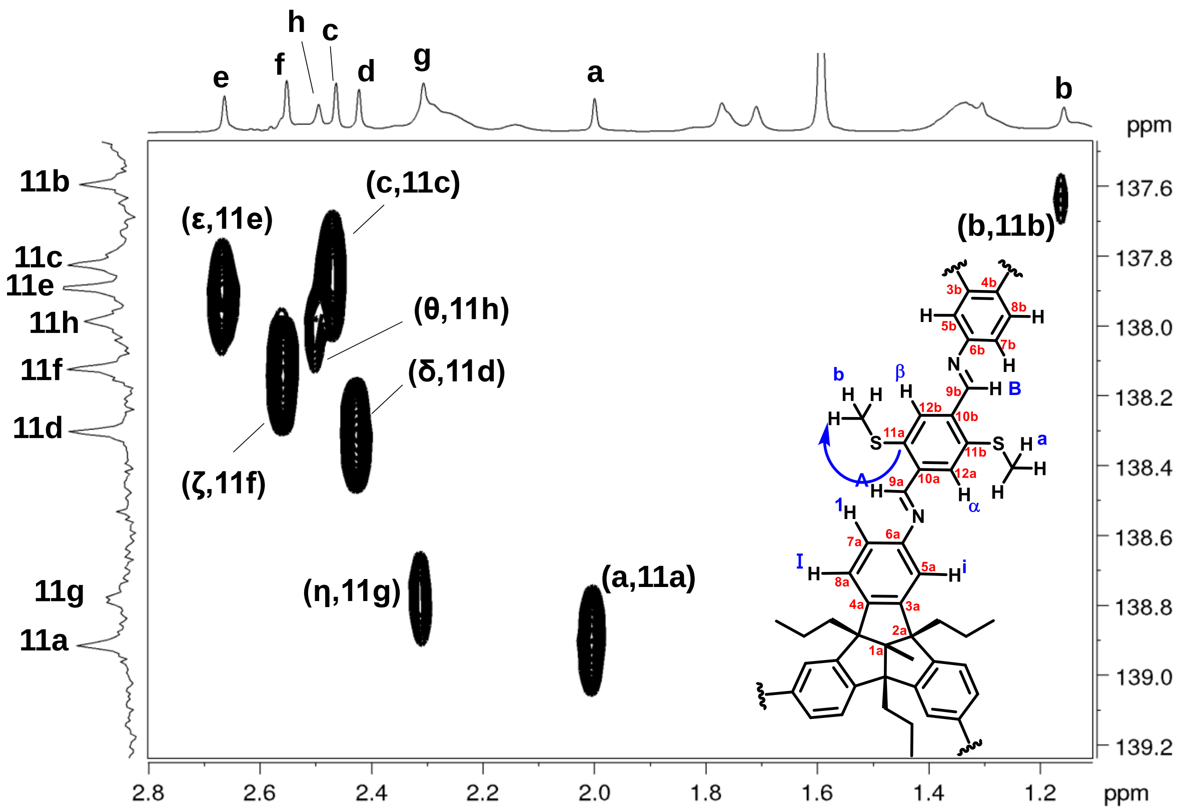

Figure 476. Partial ^{1}H-^{13}C HMBC NMR (700 MHz, CD_2Cl_2) showing cross peaks between C-(11a-11h) and H-(a-h).

Table 25. HMBC interactions between H-(α-θ) and C-(11a-11h), and C-(11a-11h) and H-(a-h).

^{1}H-Atom	^{1}H-NMR (HMBC)	^{13}C Atom	^{13}C Atom (HMBC)	^{13}C Atom	^{13}C Atom (HMBC)	^{1}H-Atom	^{1}H-NMR (HMBC)
α	7.62	11a	138.92	11a	138.92	a	1.96
β	7.80	11b	137.60	11b	137.60	b	1.12
γ	7.98	11c	137.83	11c	137.83	c	2.42
δ	8.02	11d	138.30	11d	138.30	d	2.38
ε	8.07	11e	137.89	11e	137.89	e	2.62
ζ	8.10	11f	138.13	11f	138.13	f	2.51
η	8.20	11g	178.78	11g	178.78	g	2.27
θ	8.25	11h	137.98	11h	137.98	h	2.45

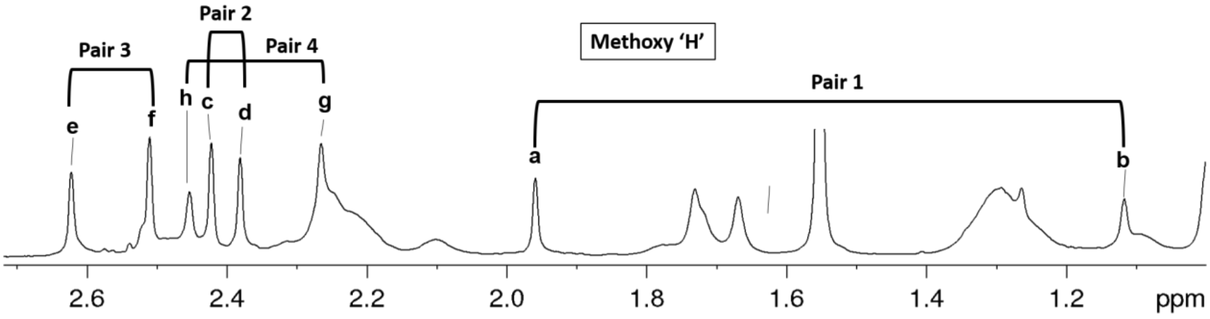

Figure 477. Partial ^{1}H NMR (700 MHz, CD_2Cl_2) spectrum of **(SMe-cube)$_2$** showing four pairs of neighboring methoxy protons.

4. The neighboring TBTQ aromatic protons were assigned by HSQC, HMBC and COSY interactions as shown in Figure 478-481.

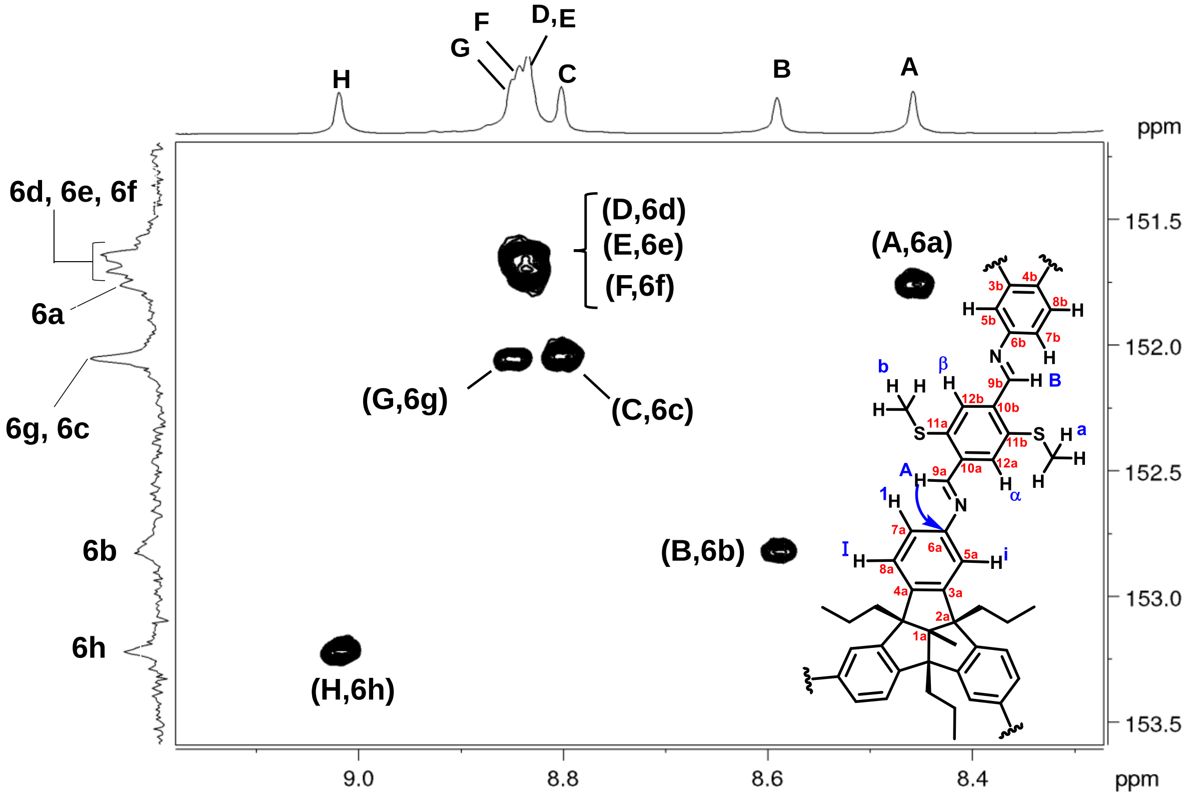

Figure 478. Partial ^{1}H-^{13}C HMBC NMR (700 MHz, CD_2Cl_2) showing cross peaks between H-(A-H) and C-(6a-6h).

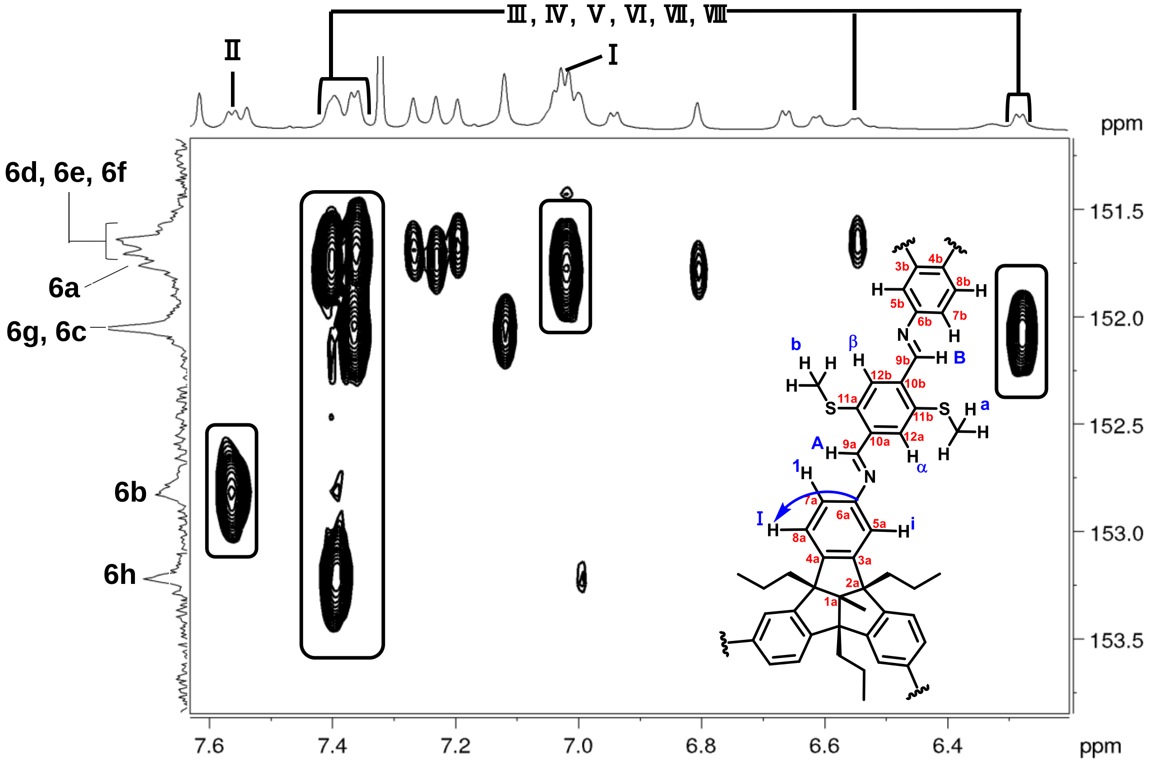

Figure 479. Partial ^{1}H-^{13}C HMBC NMR (700 MHz, CD_2Cl_2) showing cross peaks between C-(6a-6h) and H-(Ⅰ-Ⅷ).

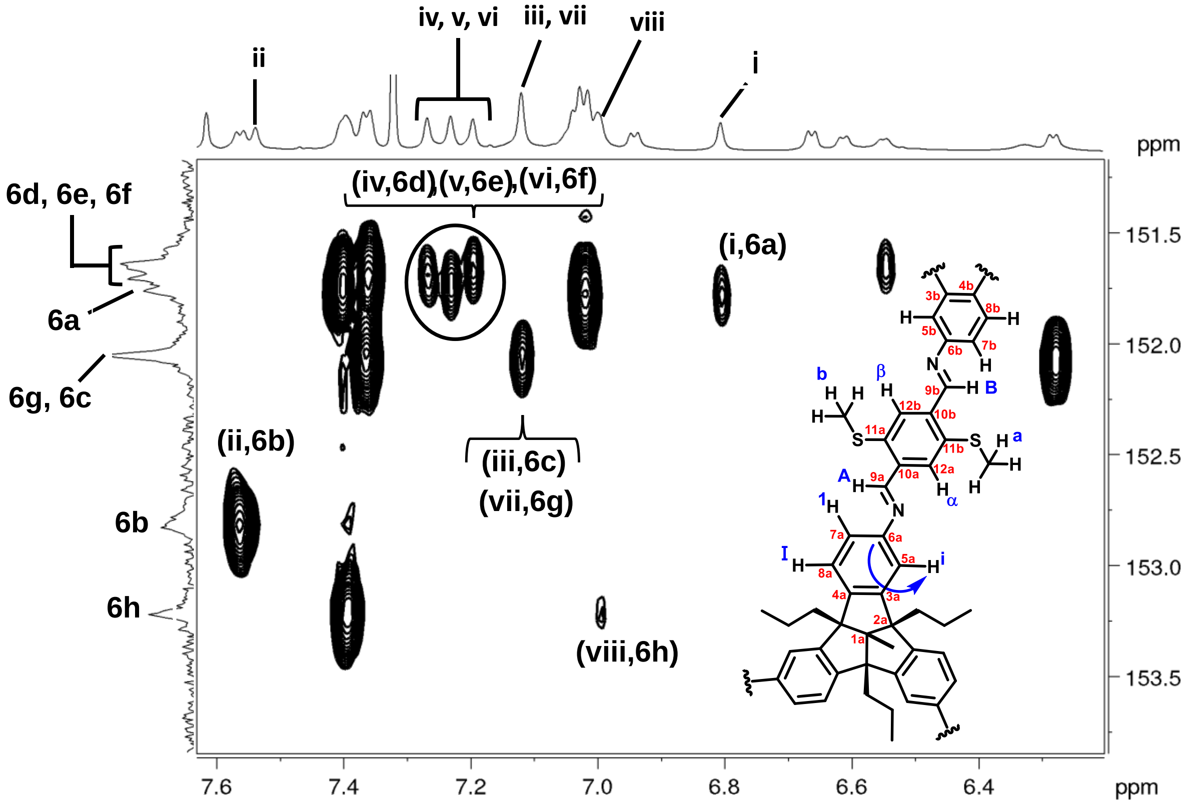

Figure 480. Partial ^{1}H-^{13}C HMBC NMR (700 MHz, CD_2Cl_2) showing cross peaks between C-(6a-6h) and H-(i-viii).

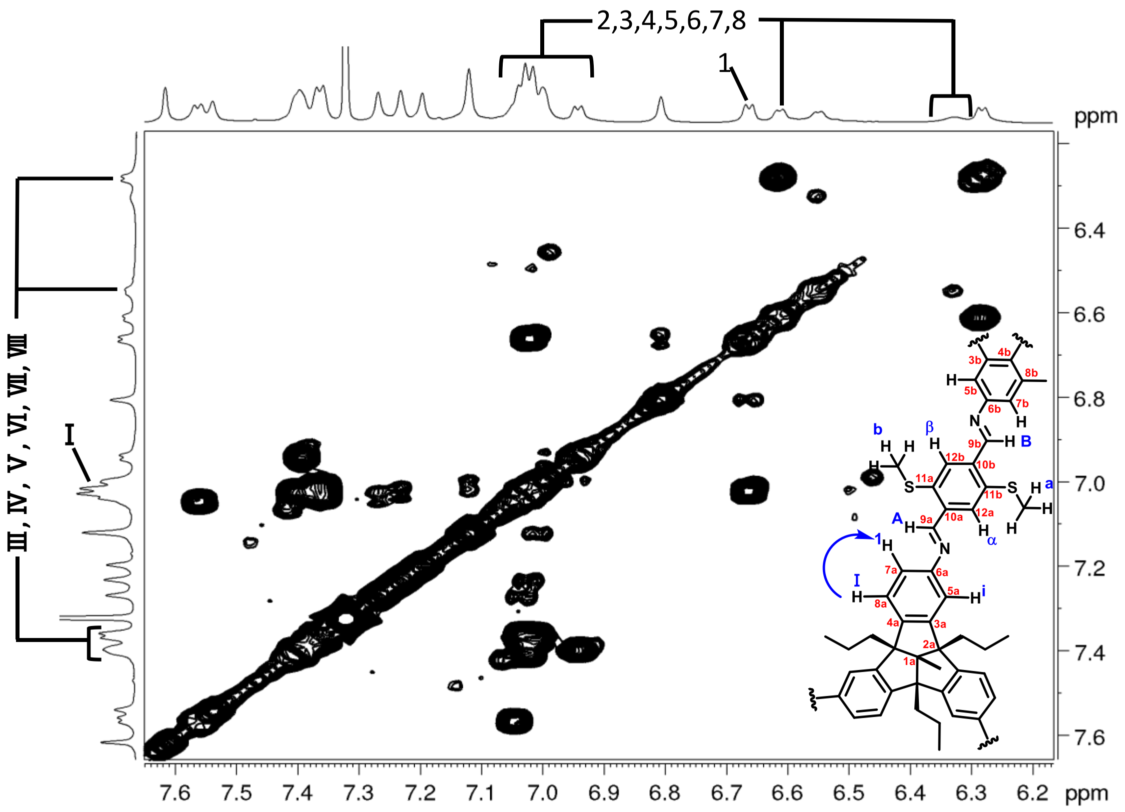

Figure 481. Partial ^1H-^1H COSY NMR (700 MHz, CD_2Cl_2) showing cross peaks between H-(Ⅰ-Ⅷ) and H-(1-8).

Assignment of dialdehyde linker α and β protons to the 3D model of (SMe-cube)$_2$

The first aldehyde linker signal at δ = 7.62 ppm, the most upfield shifted peak (labelled as α**)** is assigned to the blue imine protons of the dicatenane (Figure 482 and 483) which are found inside the other cube. This is the only linker aromatic proton in the catenane which experiences the highest shielding effect of the surrounding rings compared to other seven different imine protons of dicatenane. Based on multiple HMBC and HSQC interactions (Figure 469-472, Table 23), the neighboring dialdehyde linker proton at δ = 7.80 ppm (labelled as β) was identified and assigned to the red aldehyde protons of the dicatenane (Figure 482 and 483).

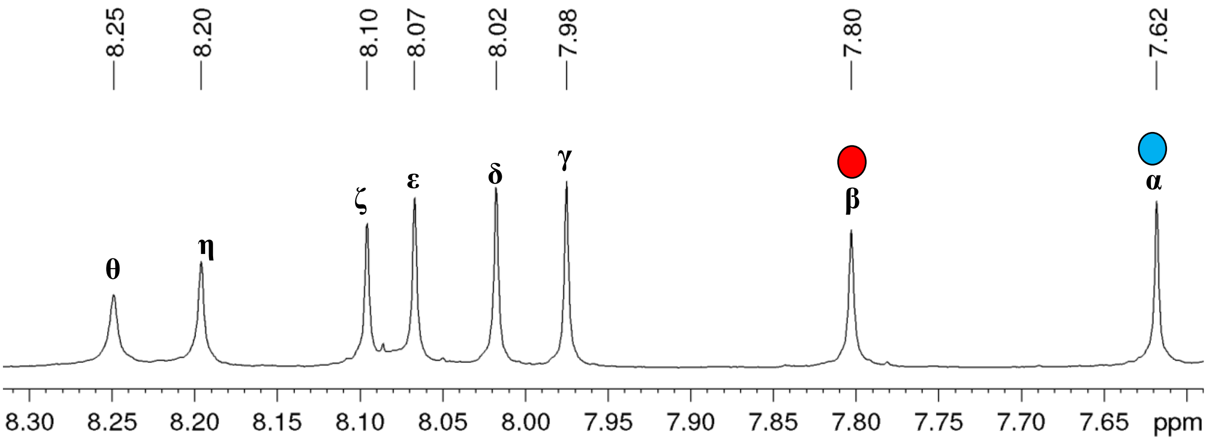

Figure 482. Partial ^{1}H NMR (600 MHz, CD_2Cl_2) spectrum of **(SMe-cube)$_2$** and assignment of linker protons **α** and **β**.

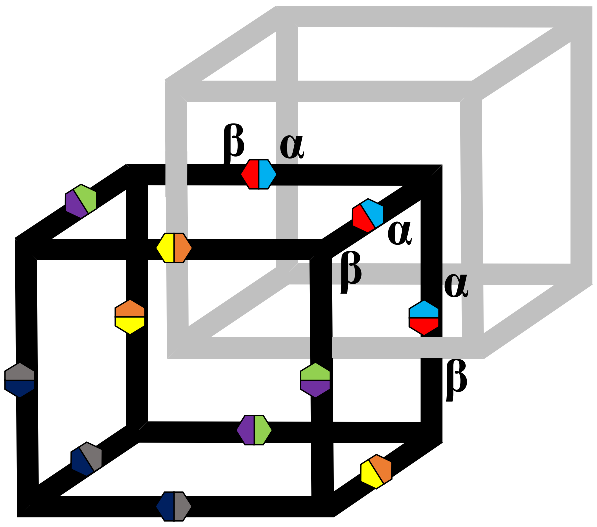

Figure 483. Cartoon representation **[SMe-cube]$_2$** showing the assignment of linker protons **α** and **β**.

Assignment of dialdehyde linker protons η and θ to the 3D model of (SMe-cube)2

The dialdehyde linker proton **β** (δ = 7.80 ppm) of one cube of catenane shows a NOESY cross peaks to the imine proton at δ = 9.02 (labelled as **H)** and SMe proton at δ = 2.27 ppm (labelled as **g**) of another cube (Figure 486 and 487). Therefore, the dialdehyde linker peak at δ = 8.25 (labelled as **θ**) is assigned to the purple linker proton of the dicatenane (Figure 484 and 485). Furthermore, the imine proton at δ = 8.59 (labelled as **B)** shows a NOESY cross peak with dialdehyde linker proton at δ = 8.25 (labelled as **θ) (**Figure 486**)** and SMe proton at δ = 2.27 ppm (labelled as **g**) (Figure 487), which further supports the assignment of dialdehyde linker proton **θ**. Based on multiple HSQC and HMBC interactions (Figure 469-472, Table 23 and 24), the neighboring linker peak at δ = 8.20 ppm (labelled as **η**) was identified and assigned to the lime green proton of the dicatenane (Figure 484 and 485).

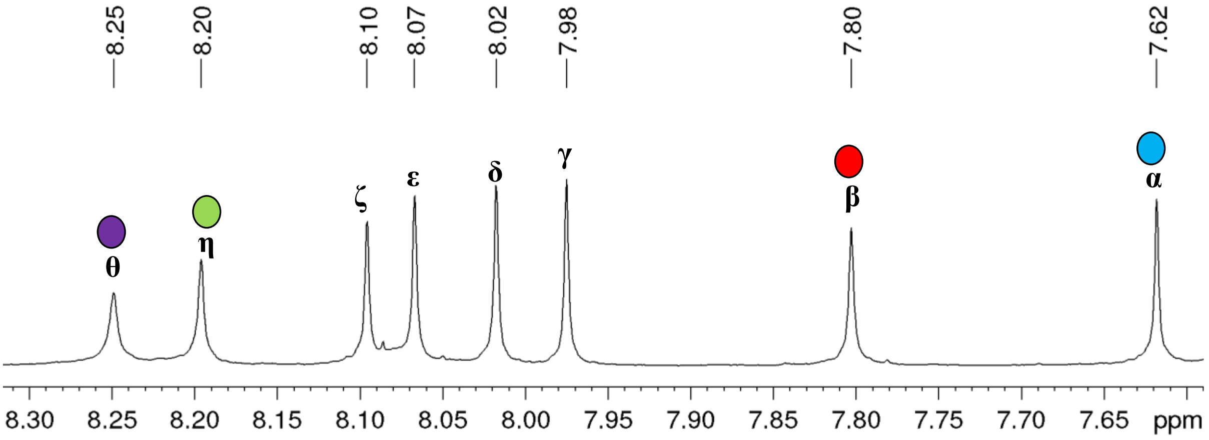

Figure 484. Partial ^{1}H NMR (600 MHz, CD_2Cl_2) spectrum of **(SMe-cube)2** and assignment of Imine protons **η** and **θ**.

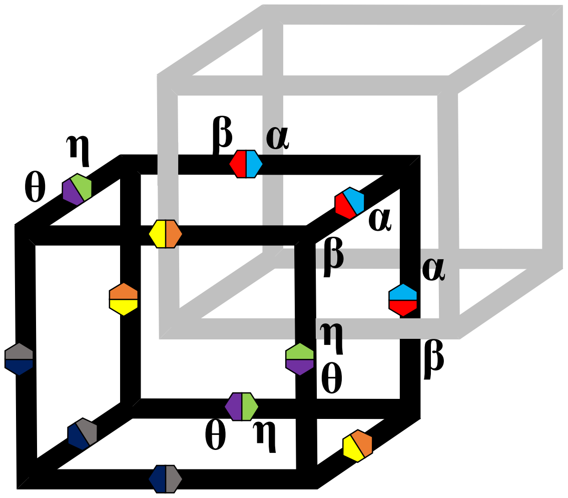

Figure 485. Cartoon representation of **[SMe-cube]2** showing the assignment of linker protons **η** and **θ**.

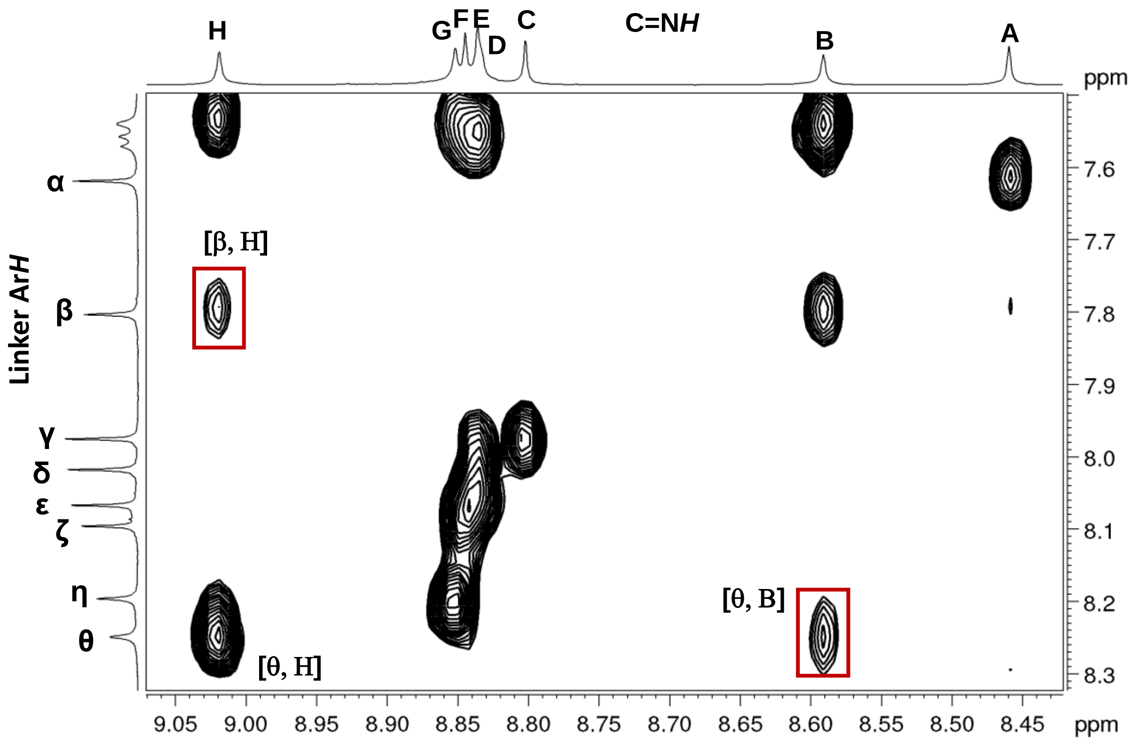

Figure 486. Partial ^{1}H-^{1}H NOESY NMR (600 MHz, CD_2Cl_2) showing cross peaks between dialdehyde linker proton and imine protons.

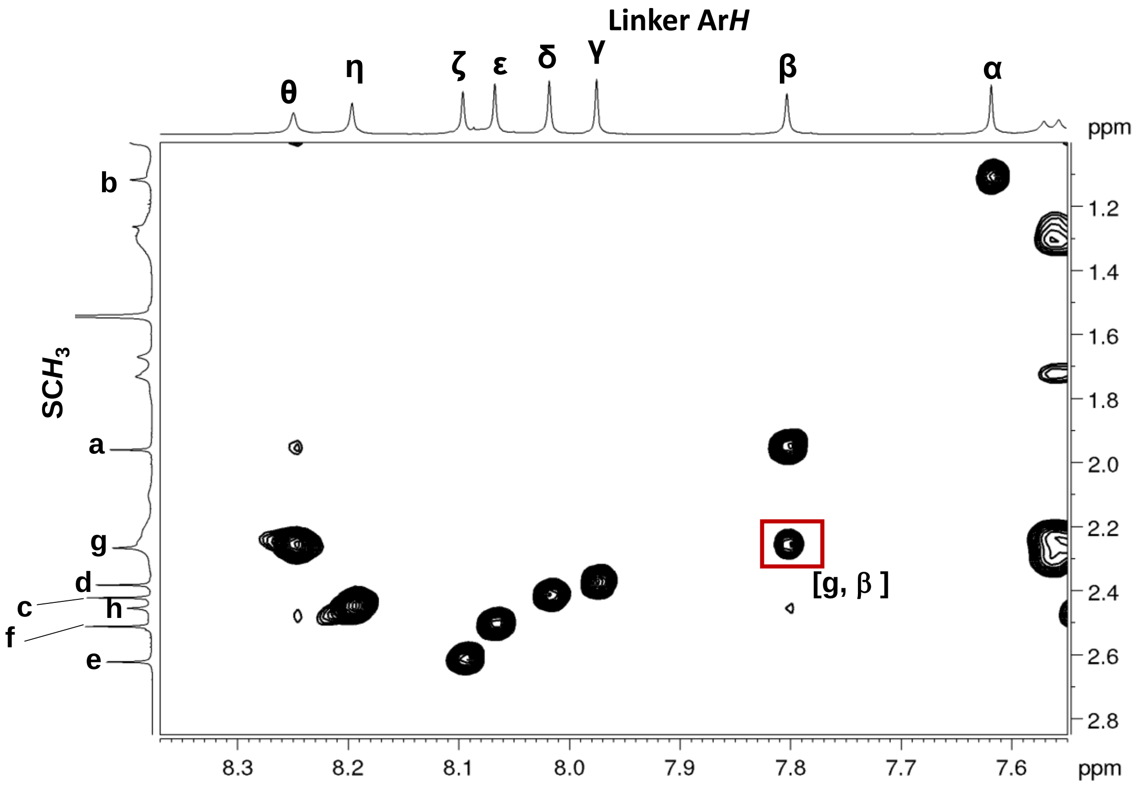

Figure 487. Partial ^{1}H-^{1}H NOESY NMR (600 MHz, CD_2Cl_2) showing cross peaks between dialdehyde linker proton and SMe proton.

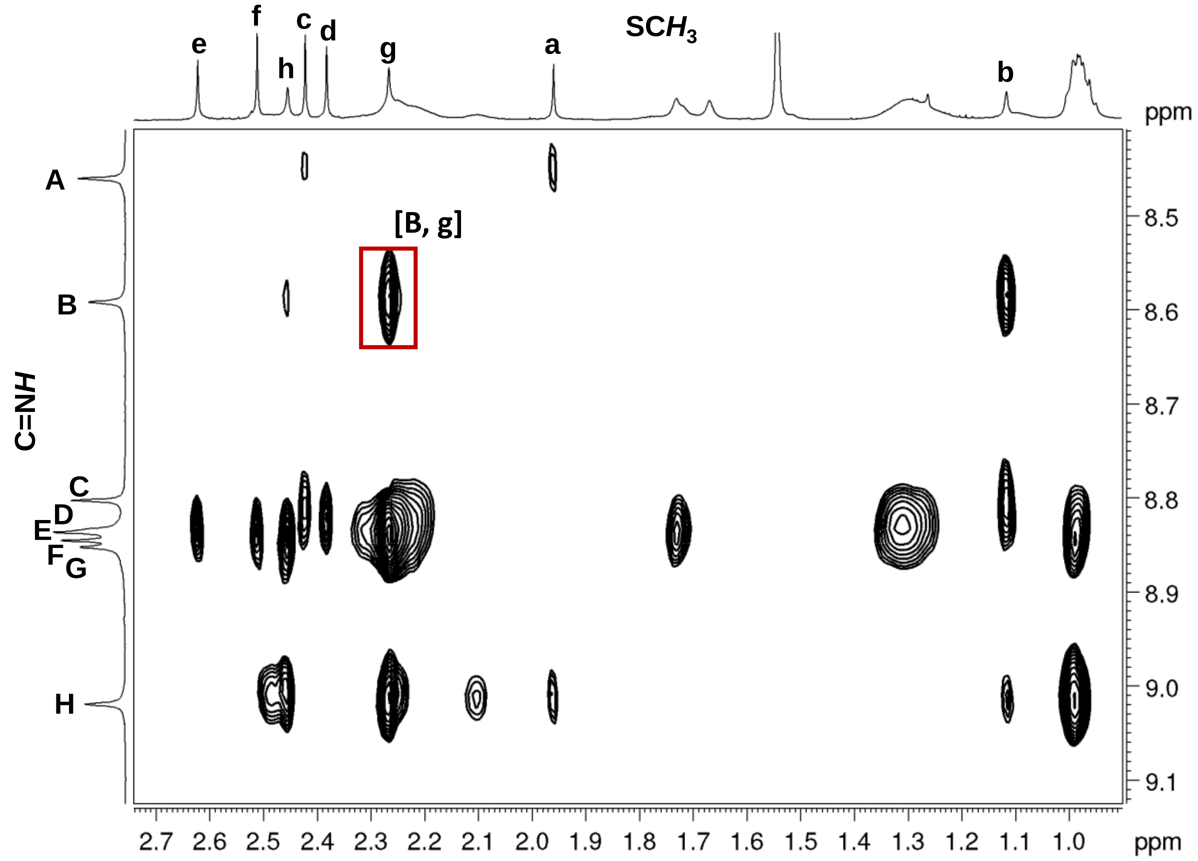

Figure 488. Partial ^{1}H-^{1}H NOESY NMR (600 MHz, CD_2Cl_2) showing cross peaks between imine protons and SMe protons.

Assignment of imine protons γ and δ to the 3D model of (SMe-cube)$_2$

For the assignment of protons **γ** and **δ**, NOE interactions of the highly shielded aliphatic protons of the propyl chain of the TBTQ units that are in direct connection to dialdehyde linker proton **α** were investigated. Figure 491 shows the NOESY cross peaks of aliphatic protons of the propyl chain at δ = 0.58 ppm (CH_3) and 0.70 ppm (CH_2) with dialdehyde linker proton at δ = 8.02 ppm (labelled as δ). Based on this interaction, the dialdehyde linker peak at δ = 8.02 (labelled as δ) assigned to the gray linker proton of the dicatenane (Figure 489 and 490). Based on multiple HMBC and HSQC interactions (Figure 469-472, Table 23 and 24) the dialdehyde linker peak at δ = 7.98 ppm (labelled as γ) is assigned to the dark blue proton of the catenane.

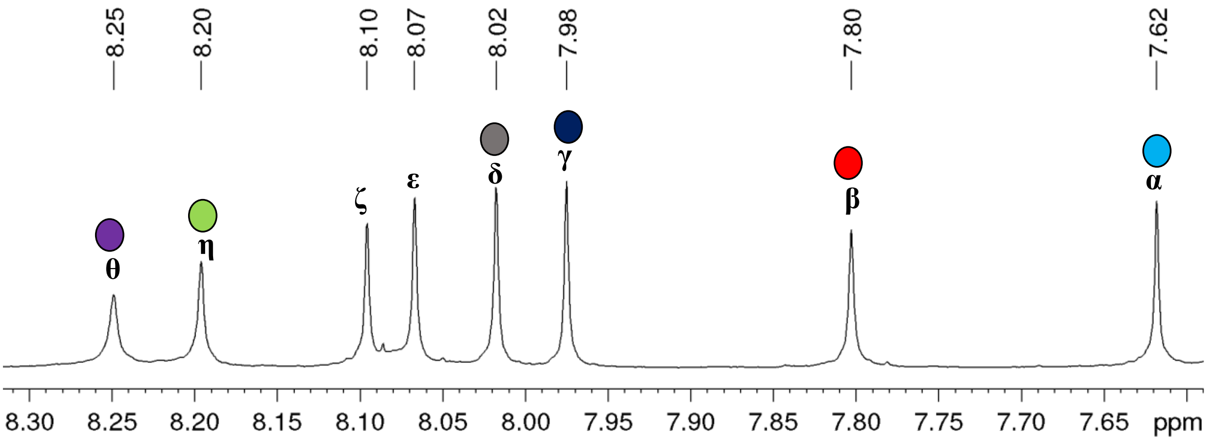

Figure 489. Partial ^{1}H NMR (600 MHz, CD_2Cl_2) spectrum of **(SMe-cube)₂** and assignment of protons γ and **δ**.

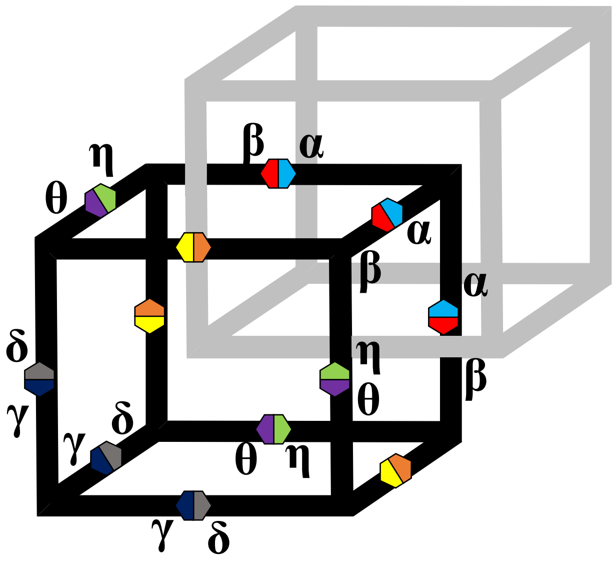

Figure 490. Cartoon representation of **[SMe-cube]₂** showing the assignment of linker protons γ and **δ**.

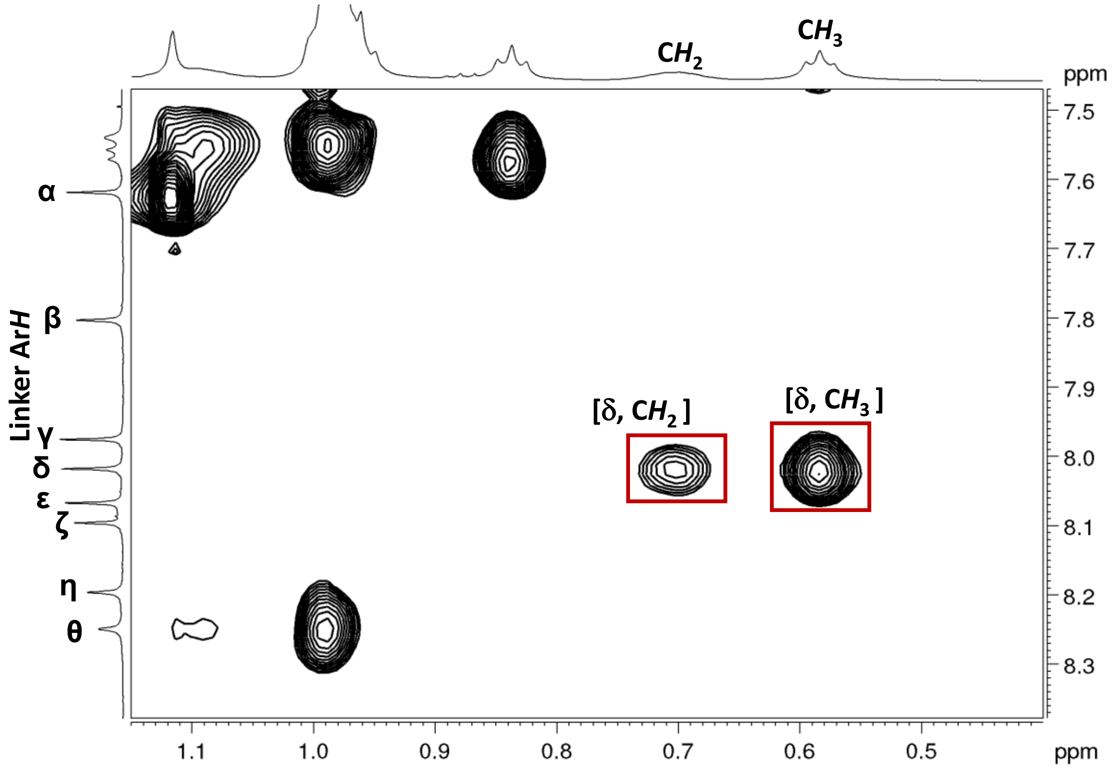

Figure 491. Partial ^{1}H-^{1}H NOESY NMR (700 MHz, CD_2Cl_2) showing cross peaks between dialdehyde linker protons and aliphatic protons.

Assignment of imine protons ε and ζ to the 3D model of (SMe-cube)$_2$

The triply interlocked model of dicatenane shows that the chemical environment of the pair of lime green dialdehyde linker proton **η** (δ = 8.20 ppm) and purple dialdehyde linker proton **θ** (δ = 8.25 ppm) is similar to the pair of yellow and orange dialdehyde protons. Therefore, the downfield shifted imine signal at δ = 8.10 ppm (labelled as ζ) is assigned to the yellow dialdehyde linker proton of the dicatenane and upfield shifted imine peak at δ = 8.07 (labelled as ε) is assigned to the orange imine proton of dicatenane (Figure 492 and 493). Multiple interactions from HMBC and HSQC (Figure 469-472, Table 23 and 24) also shows that these both protons are the neighboring dialdehyde linker protons from the same pair.

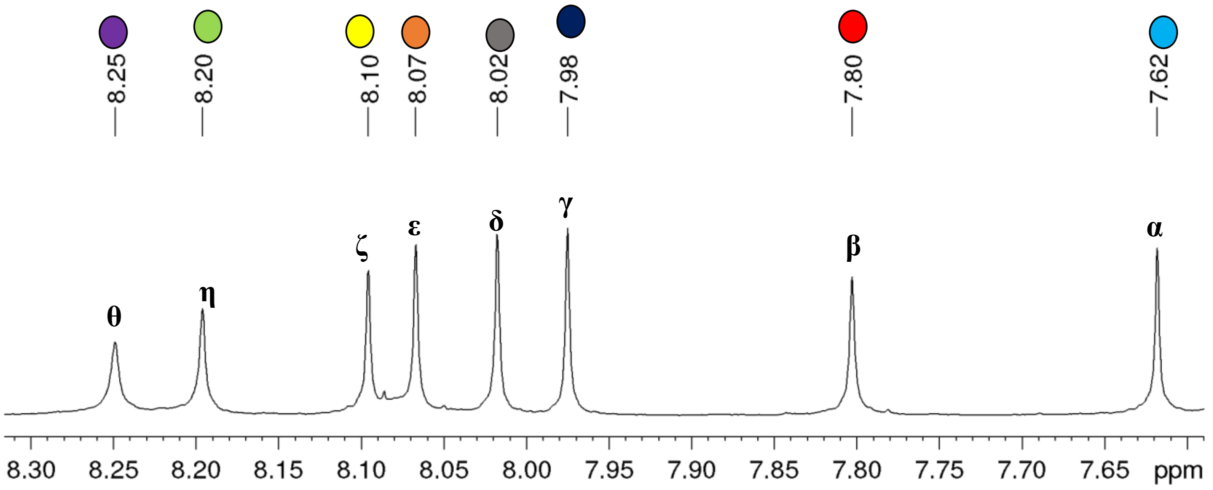

Figure 492. Partial ^{1}H NMR (600 MHz, CD_2Cl_2) spectrum of **(SMe-cube)$_2$** and assignment of proton **ε** and **ζ**.

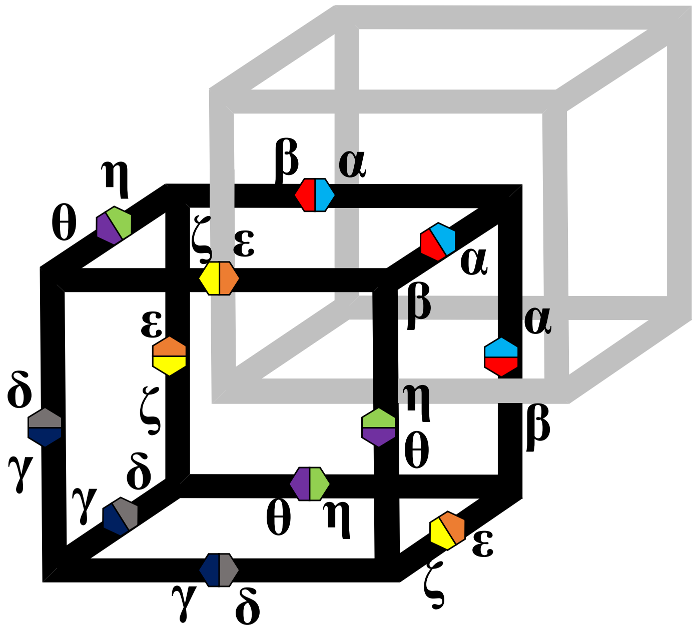

Figure 493. Cartoon representation of **[SMe-cube]$_2$** showing the assignment of linker protons **ε** and **ζ**.

SMe protons and imine protons were assigned similarly like imine protons and the complete assignment is shown in below.

Table 26.

**Ar*H* (linker)**	**δ (ppm)**	**-N=C*H***	**δ (ppm)**	**SC*H*$_3$**	**δ (ppm)**
**α**	7.62	**A**	8.46	**a**	1.96
**β**	7.80	**B**	8.59	**b**	1.12
**γ**	7.98	**C**	8.80	**c**	2.42
**δ**	8.02	**D**	8.84	**d**	2.38
**ε**	8.07	**E**	8.84	**e**	2.62
**ζ**	8.10	**F**	8.845	**f**	2.51
**η**	8.20	**G**	8.85	**g**	2.27
**θ**	8.25	**H**	9.02	**h**	2.45

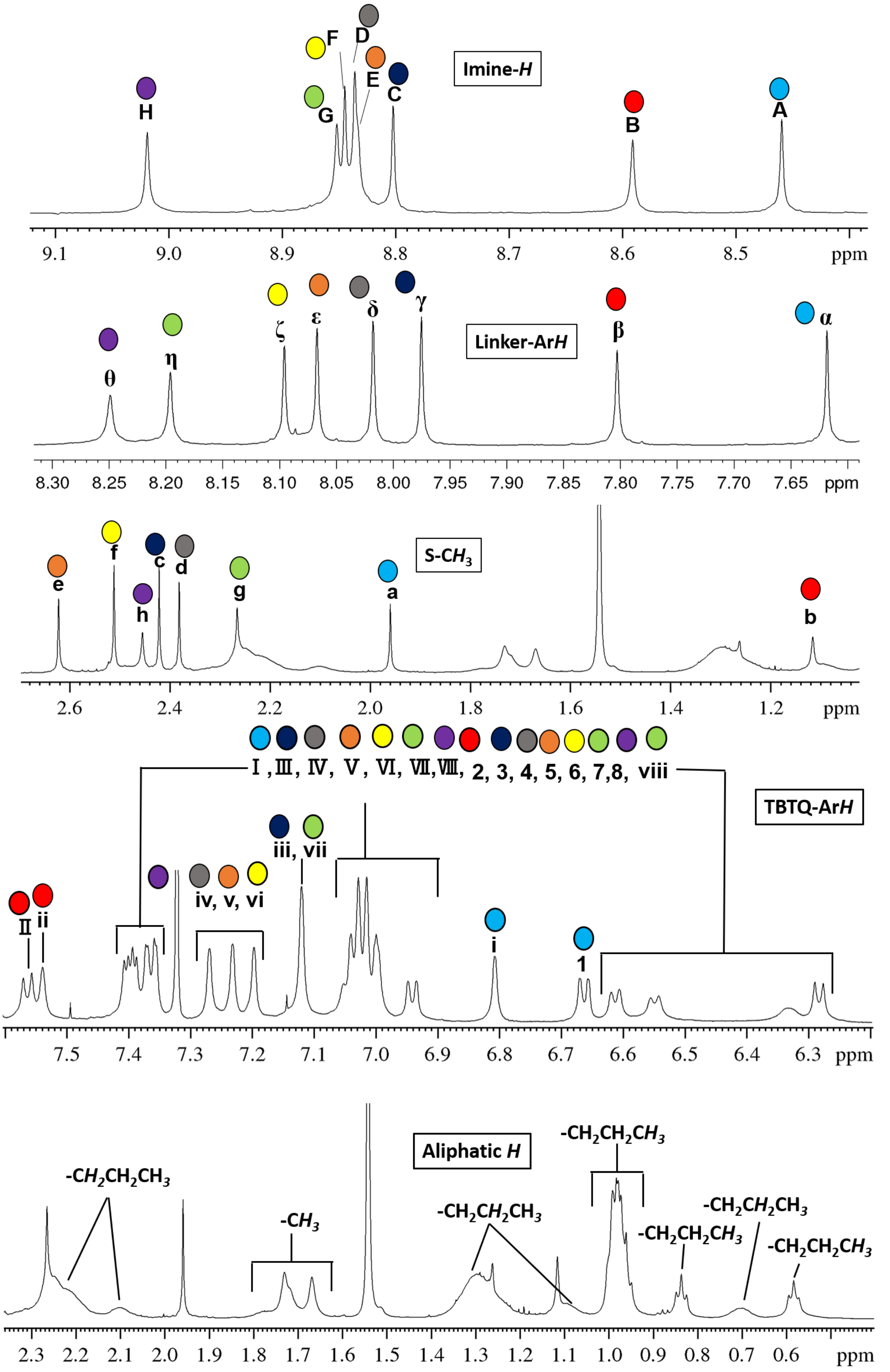

Figure 494. ^{1}H NMR (600 MHz, CD_2Cl_2) and Assignment of protons signals of **(SMe-cube)$_2$**.

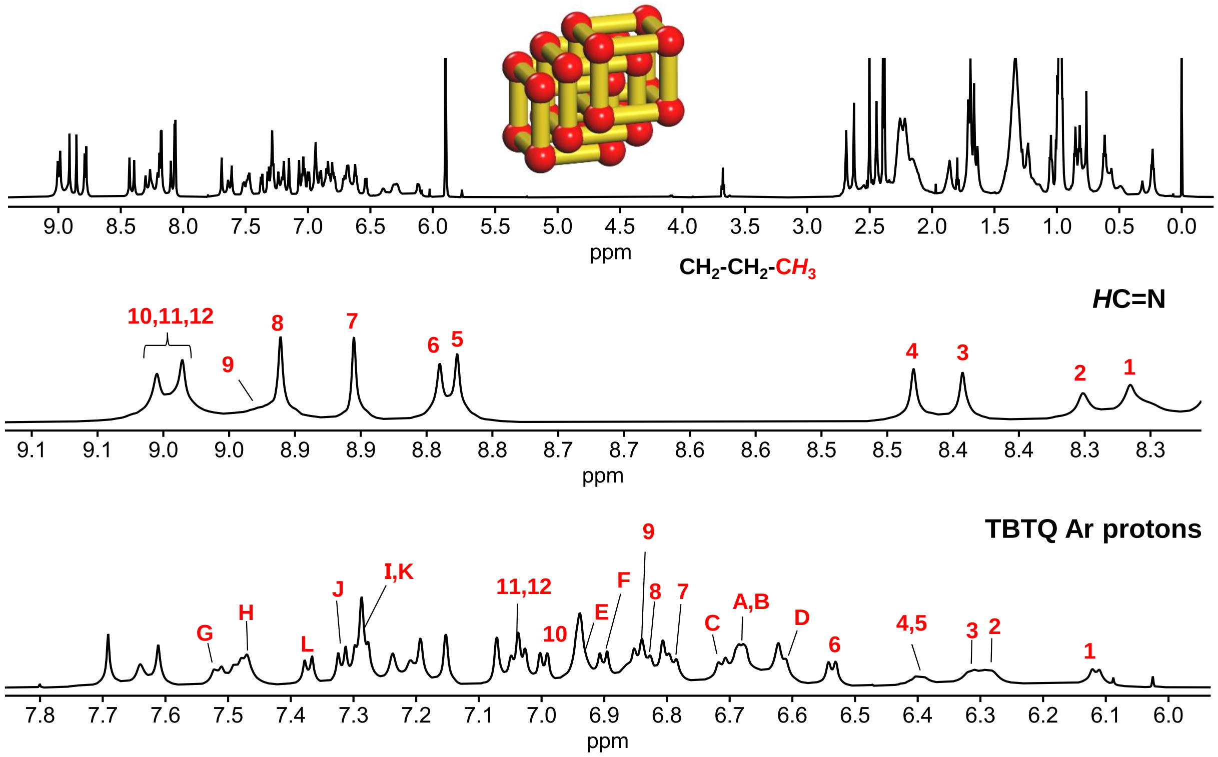

Figure 495. ^{1}H NMR (700 MHz, TCE, 410 K) and number of imine and TBTQ aromatic signals of **(SMe-cube)₃**. (*24 TBTQ aromatic protons (protons ortho to each other) are assigned mainly from ^{1}H-^{1}H COSY (Fig. S232) and other 2D spectra. For 2D spectra please see Figs. S229-S244)*

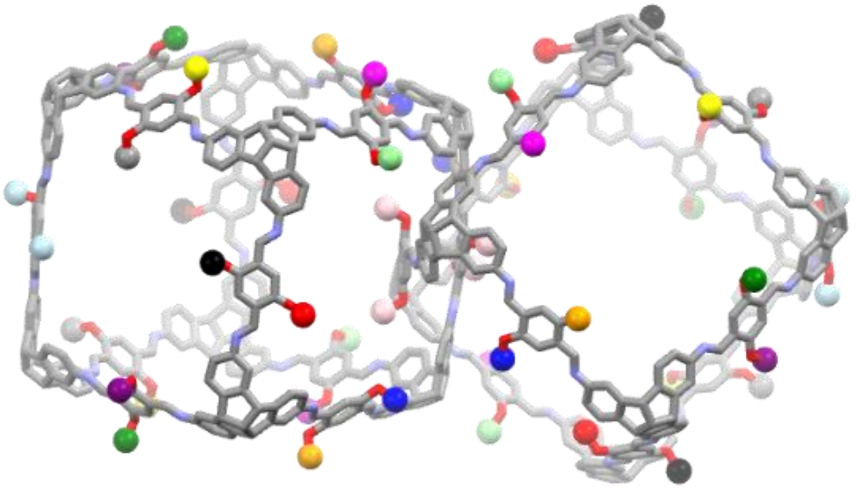

Figure 496. Highlighting of 12 magnetically equivalent methoxy groups for a singly interlocked **(OMe-cube)₂** (not observed).

14. Variable temperature NMR studies with (OMe cube)3 and (SMe cube)3

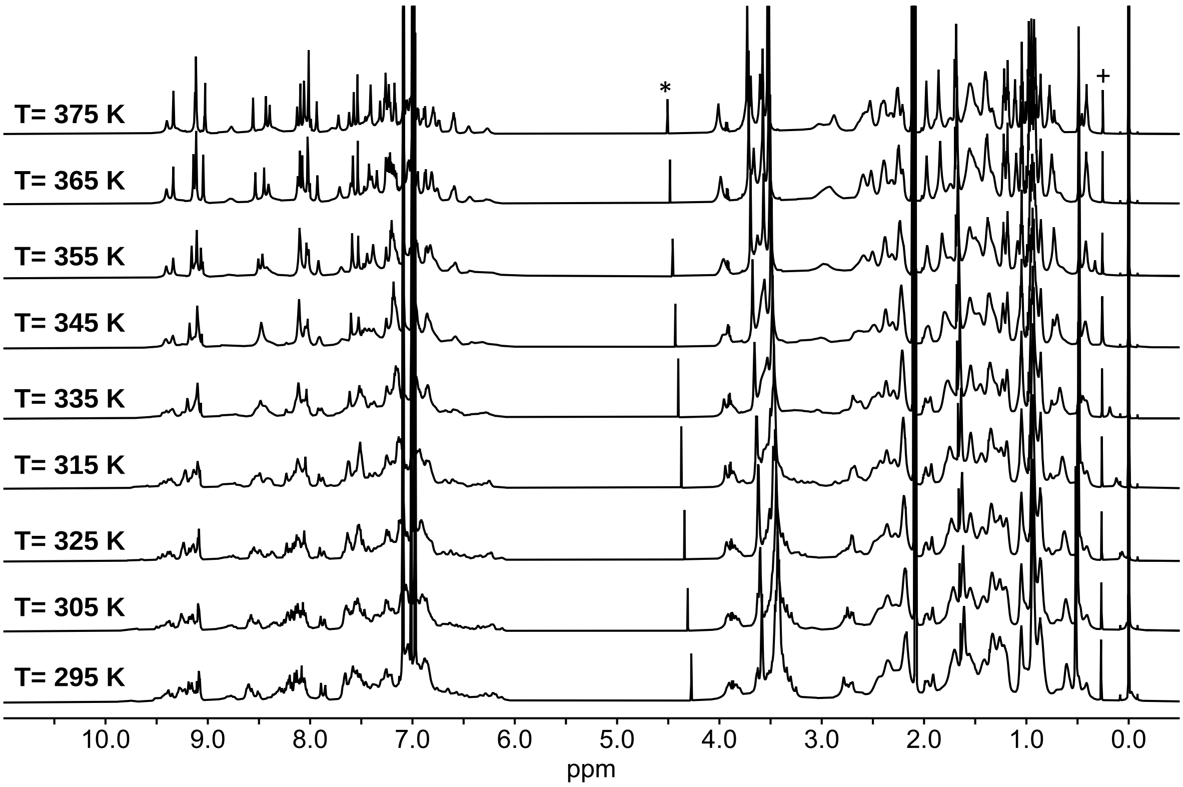

Figure 497 Temperature dependent ^{1}H NMR (700 MHz, Toluene-d8) spectra of **(OMe cube)3**. *DCM, +silicon grease.

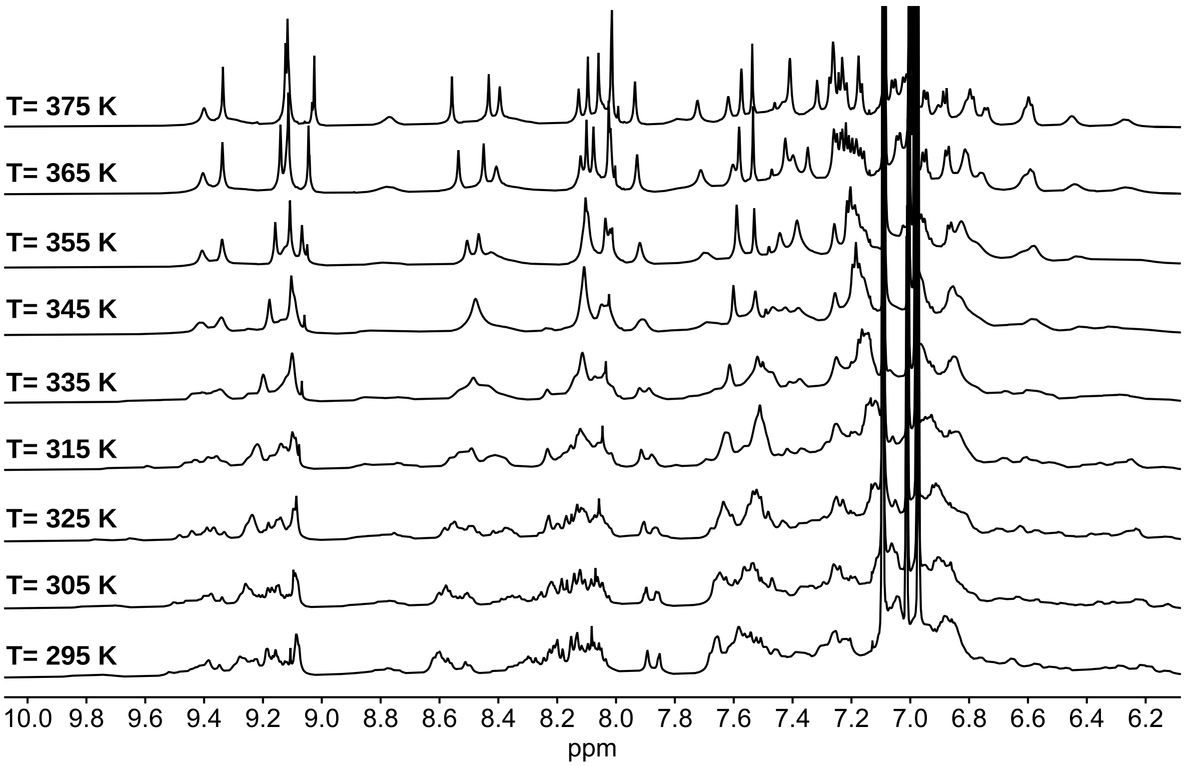

Figure 498. Temperature dependent partial ^{1}H NMR (700 MHz, Toluene-d8) spectra of **(OMe cube)3**.

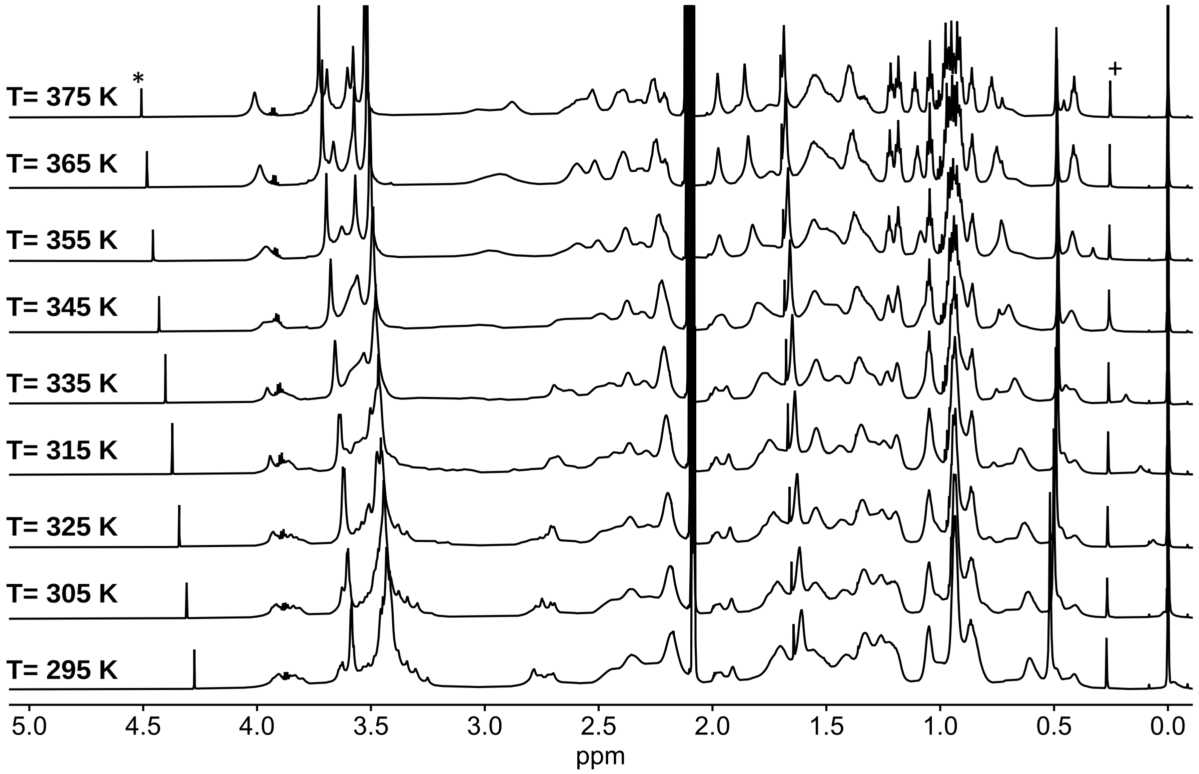

Figure 499. Temperature dependent partial ^1H NMR (700 MHz, Toluene-d_8) spectra of **(OMe cube)$_3$**. *DCM, +silicon grease.

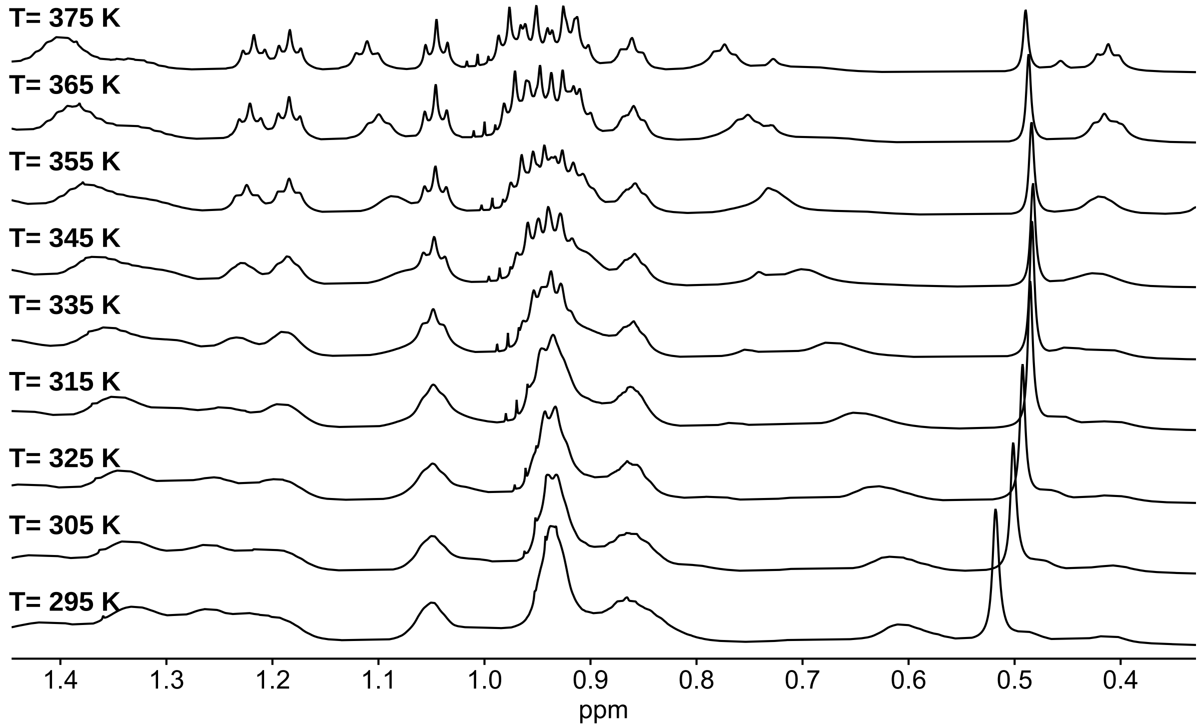

Figure 500. Temperature dependent partial ^1H NMR (700 MHz, Toluene-d_8) spectra of **(OMe cube)$_3$**.

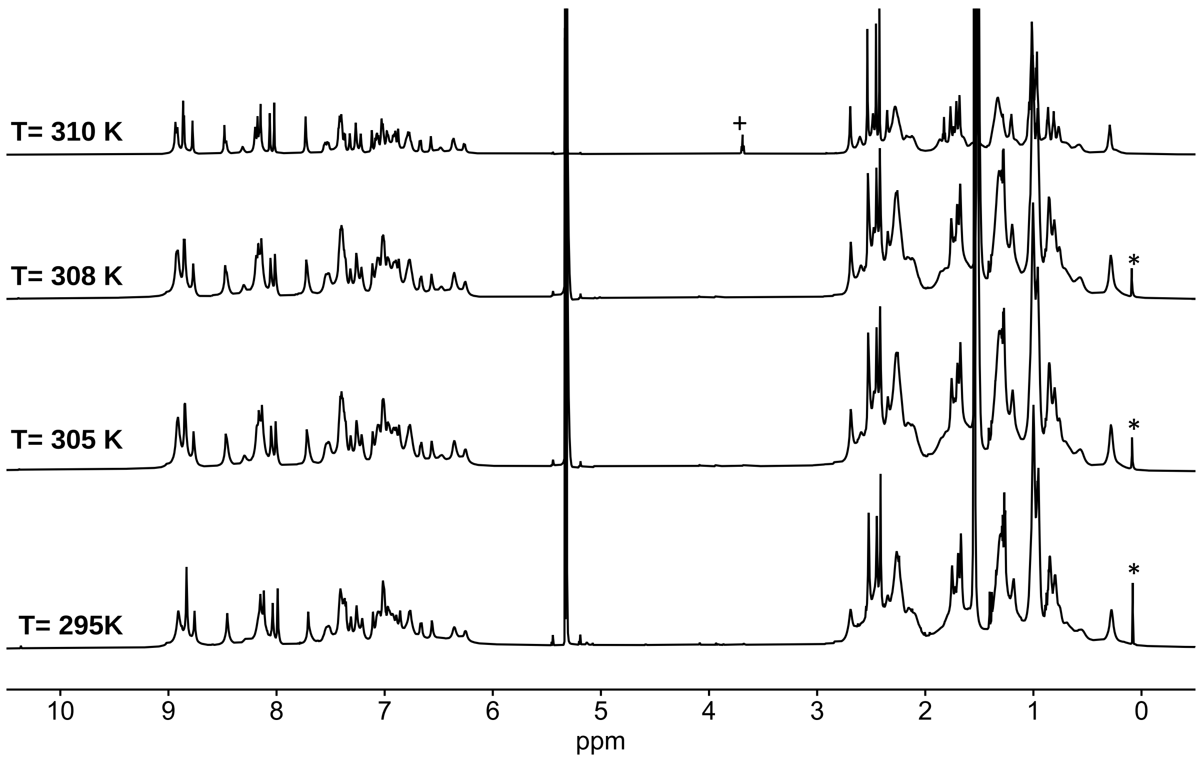

Figure 501. Temperature dependent ^{1}H NMR (700 MHz, CD_2Cl_2) spectra of **(SMe cube)$_3$**. [+]THF, *silicon grease.

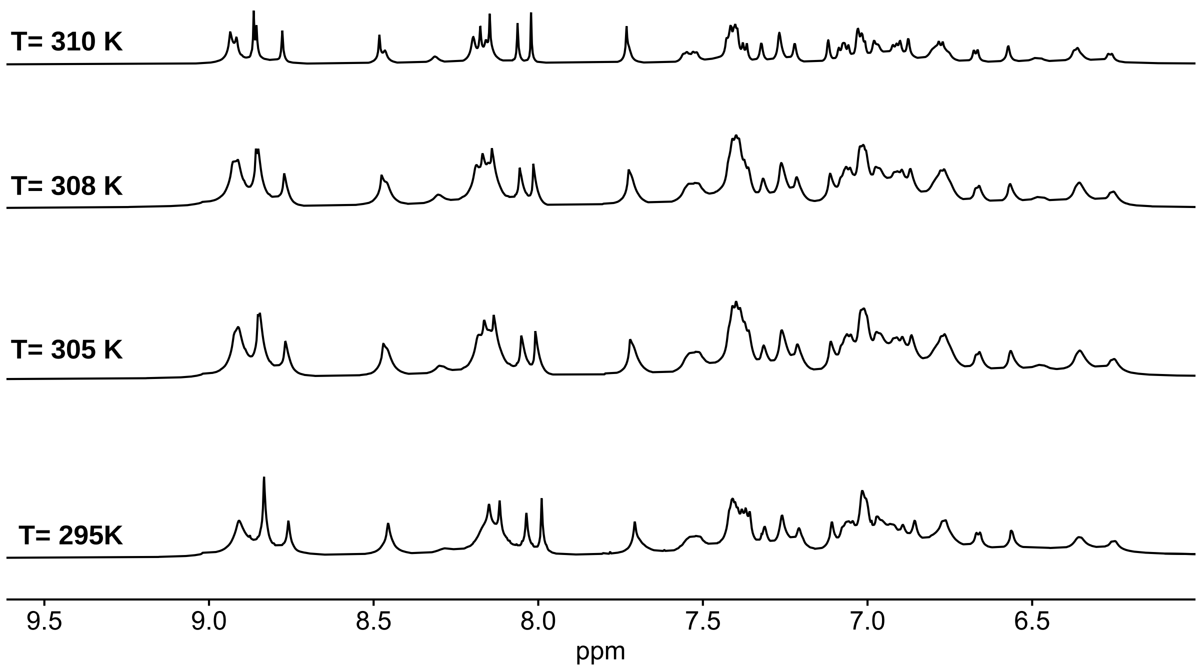

Figure 502. Temperature dependent partial ^{1}H NMR (700 MHz, CD_2Cl_2) spectra of **(SMe cube)$_3$**.

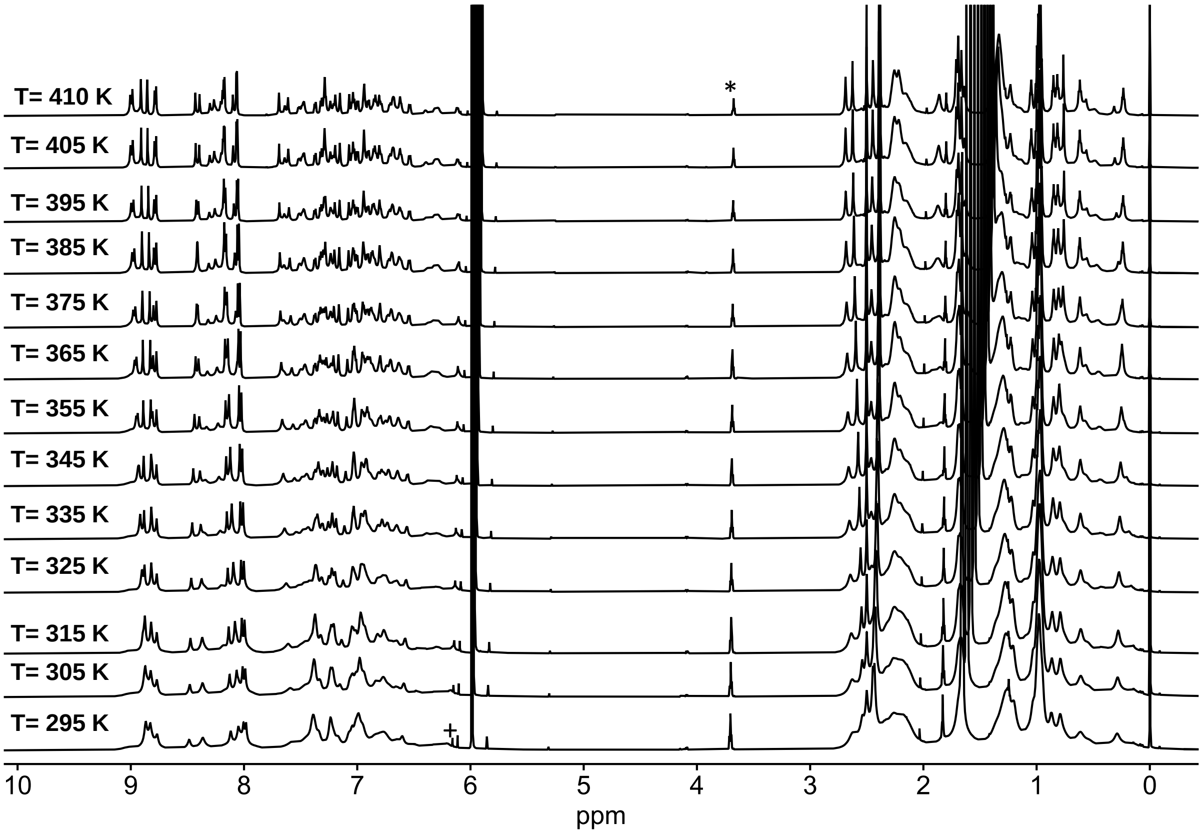

Figure 503. Temperature dependent ^{1}H NMR (700 MHz, $C_2D_2Cl_4$) spectra of **(SMe cube)$_3$**. $^+CHCl_3$ *THF.

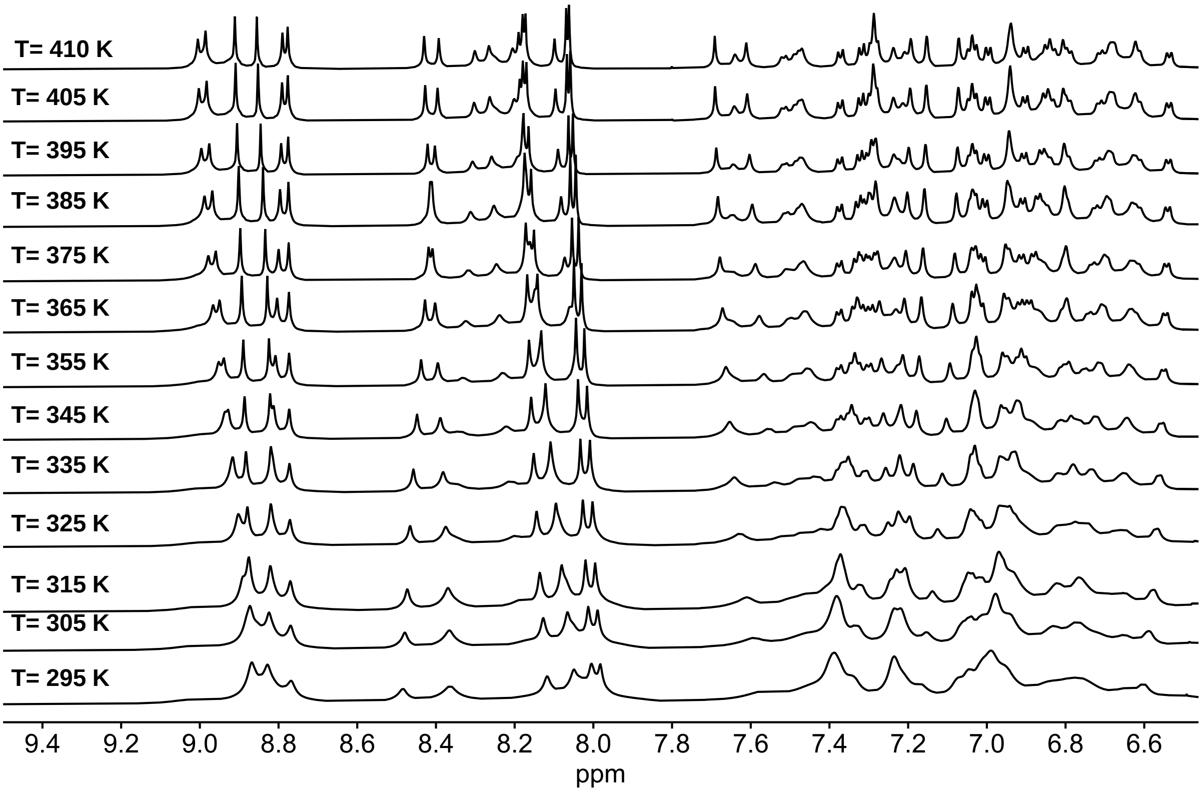

Figure 504. Temperature dependent partial ^{1}H NMR (700 MHz, $C_2D_2Cl_4$) spectra of **(SMe cube)$_3$**.

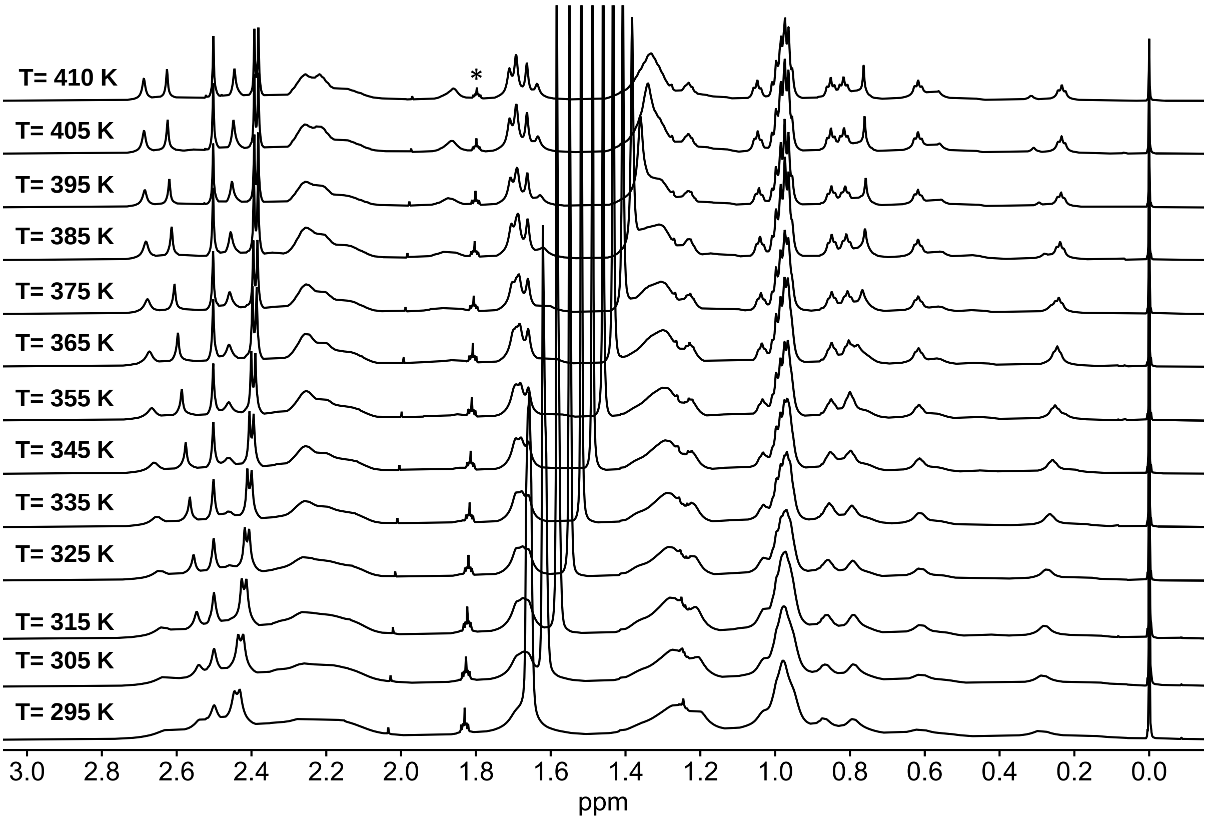

Figure 505. Temperature dependent partial ^{1}H NMR (700 MHz, $C_2D_2Cl_4$) spectra of **(SMe cube)$_3$**. *THF.

15. ^{1}H NMR kinetic studies:

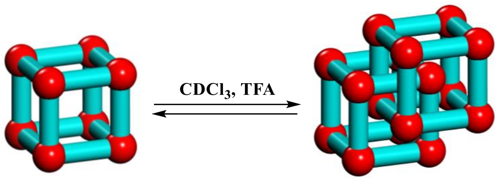

OMe-cube (OMe-cube)$_2$

The **OMe-cube→(OMe-cube)$_2$** reaction was treated as second order reaction. ^{1}H NMR measurements were performed on a 700 MHz NMR instrument. The rate constant $\boldsymbol{k_1}$ was determined from the graphical evaluation of the slope with the following equation (1):

$$\frac{1}{[\text{OMe-cube}]_t} - \frac{1}{[\text{OMe-cube}]_0} = k_1 t \quad \cdots\cdots \quad \text{Equation (1)}$$

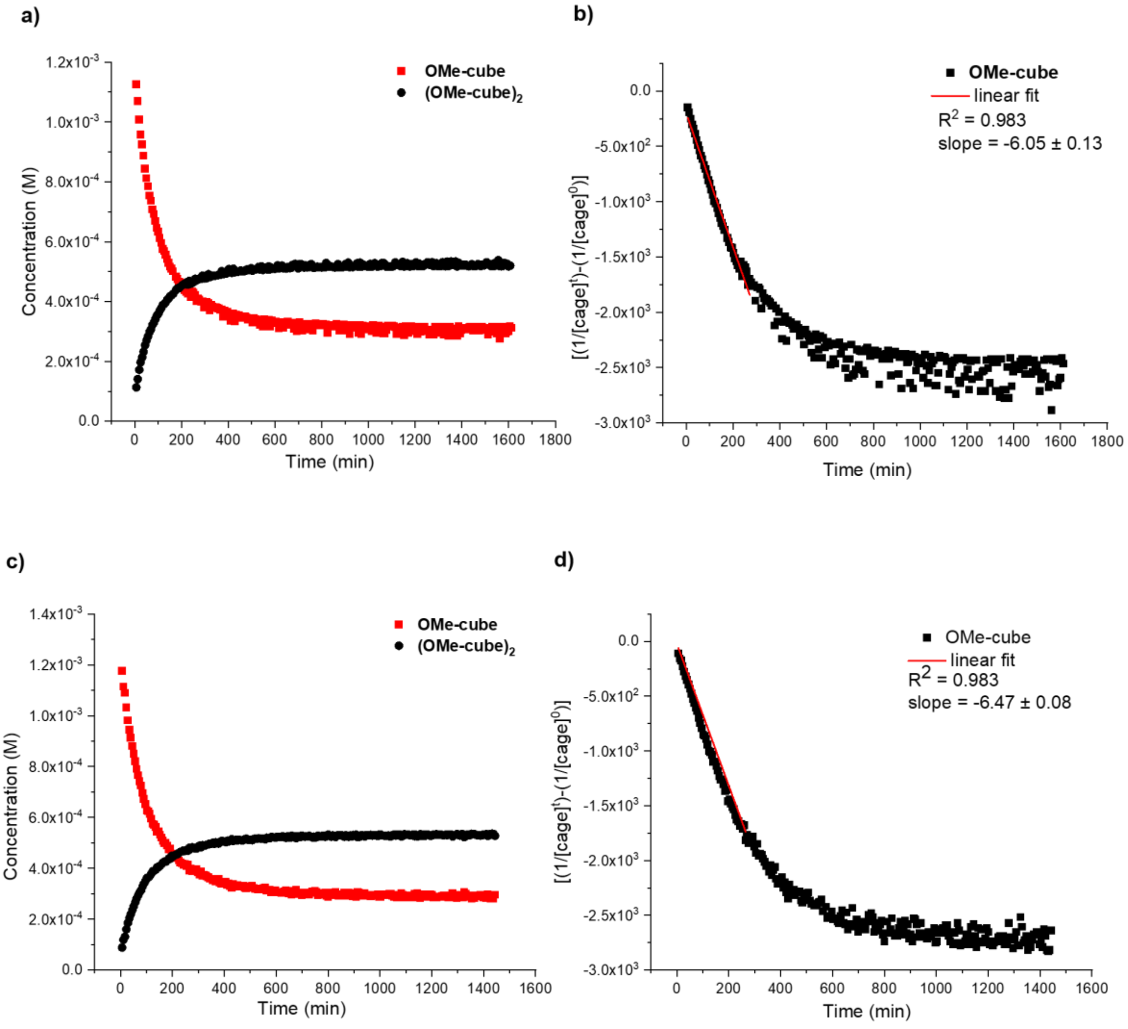

Figure 506. ^{1}H NMR kinetic data of two measurements. Concentration vs. time plot **OMe-cube →(OMe-cube)$_2$** reaction a) and c), and graphical evaluation rate constant $\boldsymbol{k_1}$ b) and d).

Table 27. Experimental details of ^{1}H NMR kinetic measurements

Cage	$CDCl_3$ (μL)	OMe-cube (μmol)	TFA (μmol)	T (K)	k_1 (x 10^{-2} M^{-1} s^{-1})	k_1 (x 10^{-2} M^{-1} s^{-1})
**OMe-cube**	500	0.676	0.653	295	10.1	10.5 ± 0.4
	500	0.676	0.653	295	10.8	

The average value of two measurements reported as k_1. The standard deviations calculated using STDEV.P function of Microsoft Excel 2016.

Calculation of cage to catenane ratio from ^{1}H NMR spectrum

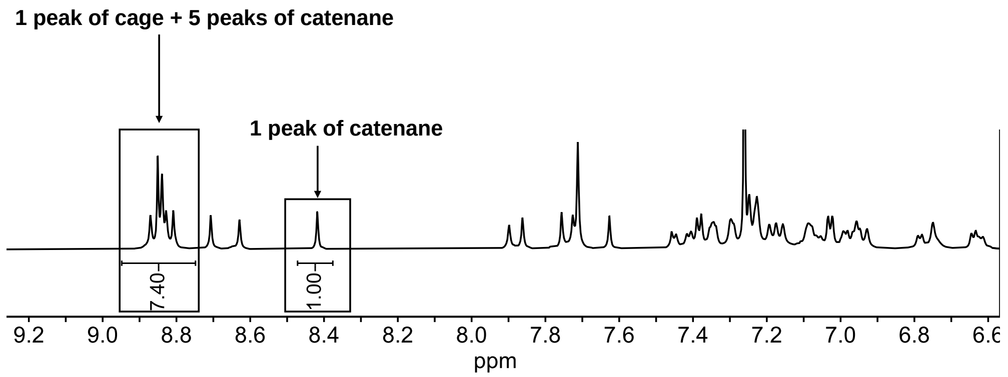

Dimeric catenane give total 8 imine peaks and monomeric cage give 1 imine peak in ^{1}H NMR spectrum.

Integration of cage = (cage+catenane peak)-5

Integration of cage = (7.40)-5 = 2.40

cage:catenane ratio is 2.40:1

Cage has 24 protons per imine signals and catenane has 6 imine protons per imine signals.

Cage:catenane ratio is (2.40/24):(1/6) = 0.100:0.167

cage:catenane ratio is (0.100/0.100):(0.167/0.100) = 1.00:1.67

Equation used to find the concentrations (M) of cage and catenanes in the NMR sample used for kinetic studies

mass of cage + mass of catenane = 0.0038 g

(no. of moles of cage × mol. wt. of cage) + (no. of moles of catenane + mol. wt. of catenane) = 0.0038 g

(for example, the ratio of **Cage:Catenane** calculated from the ^{1}H NMR spectra is **1.00:1.67**) (NMR spectra number 112.)

$(n_{cage} \times 5623.23$ g/mol) + $(n_{cat} \times 11246.47$ g/mol) = 0.0038 g

(e.g. Cage to catenane ratio obtained from ^{1}H NMR is 1:1.67)

n_{cage}=A mol, n_{cat}=1.67 × A mol

(A mol × 5623.23 g/mol) + (1.67 × A mol x 11246.47 g/mol) = 0.0038 g

5623.23 g/mol × A mol + 18781.60 g/mol × A mol = 0.0038 g

A mol = 0.0038 g/24404.83 g/mol

A mol = 0.0000001557 mol = n_{cage}

n_{cat}= 1.67 x 0.0000001557 mol = 0.00000026 mol

[Cage] = 0.0000001557 mol/ 0.0005L = 0.0003114 M,

[Catenane] = 0.00000026 mol/ 0.0005 L = 0.00052 M

16. Catenation test of OMe-cube without acid catalyst trifluoroacetic acid

Procedure: In a 1.5 mL vial **OMe-cube** (4 mg, 0.71 µmol) was completely dissolved in $CDCl_3$ (526 µL) and then reaction mixture was transferred into an NMR tube and analysed by NMR spectroscopy at RT at different time intervals. ($CDCl_3$ was passed through the basic alumina just before using it for the reaction).

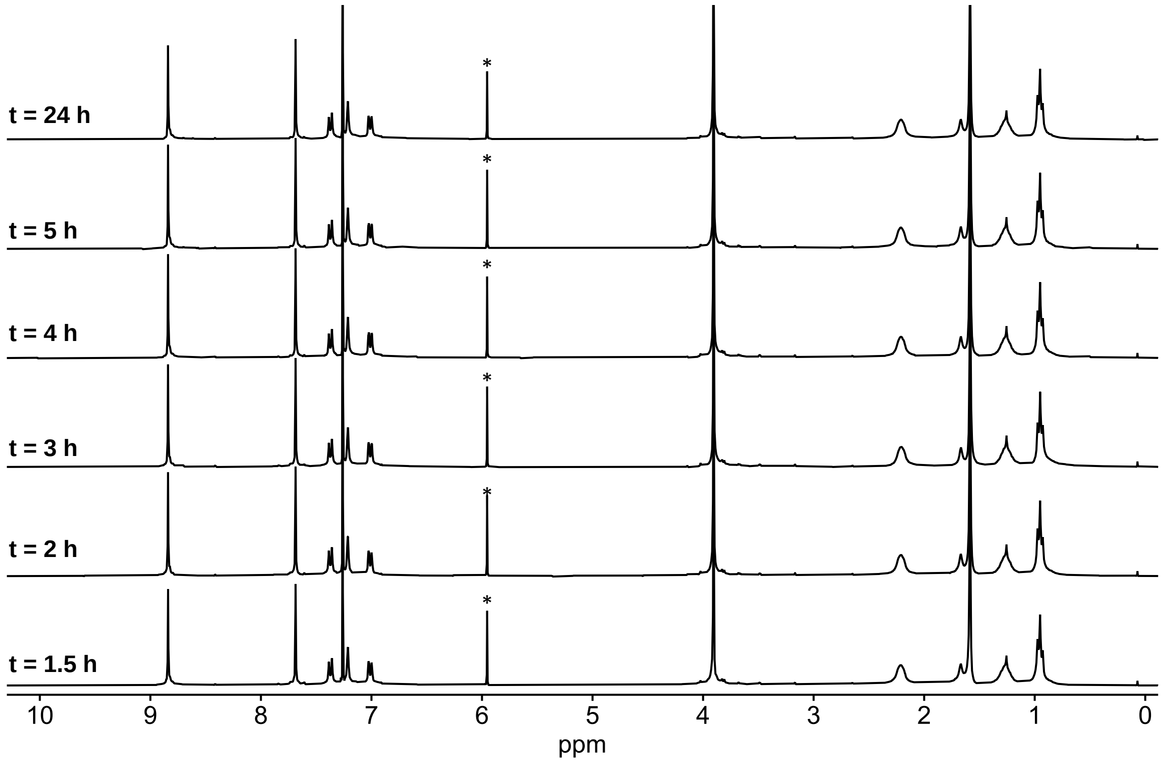

Figure 507. Time dependent ^{1}H NMR (300 MHz, $CDCl_3$) spectra monitoring **(OMe-cube)** →**$(OMe\text{-}cube)_2$** reaction without TFA catalyst. *1,1,2,2-tetrachloroethane.

17. Mechanistic studies of catenane formation by mass spectrometry

1) Mechanistic Studies for OMe-cube and (OMe-cube)$_2$

Procedure: To a mixture of **OMe-cube** (2 mg, 0.36 µmol) and ^{15}N labelled ***OMe-cube** (2 mg, 0.36 µmol) in a 1:1 ratio in $CDCl_3$ (0.53 mL), TFA (5.3 µL of a 0.131M solution in $CDCl_3$, 0.69 µmol) was added and the reaction mixture was stirred for 3 days at RT and then analysed by TIMS-TOF mass spectrometry.

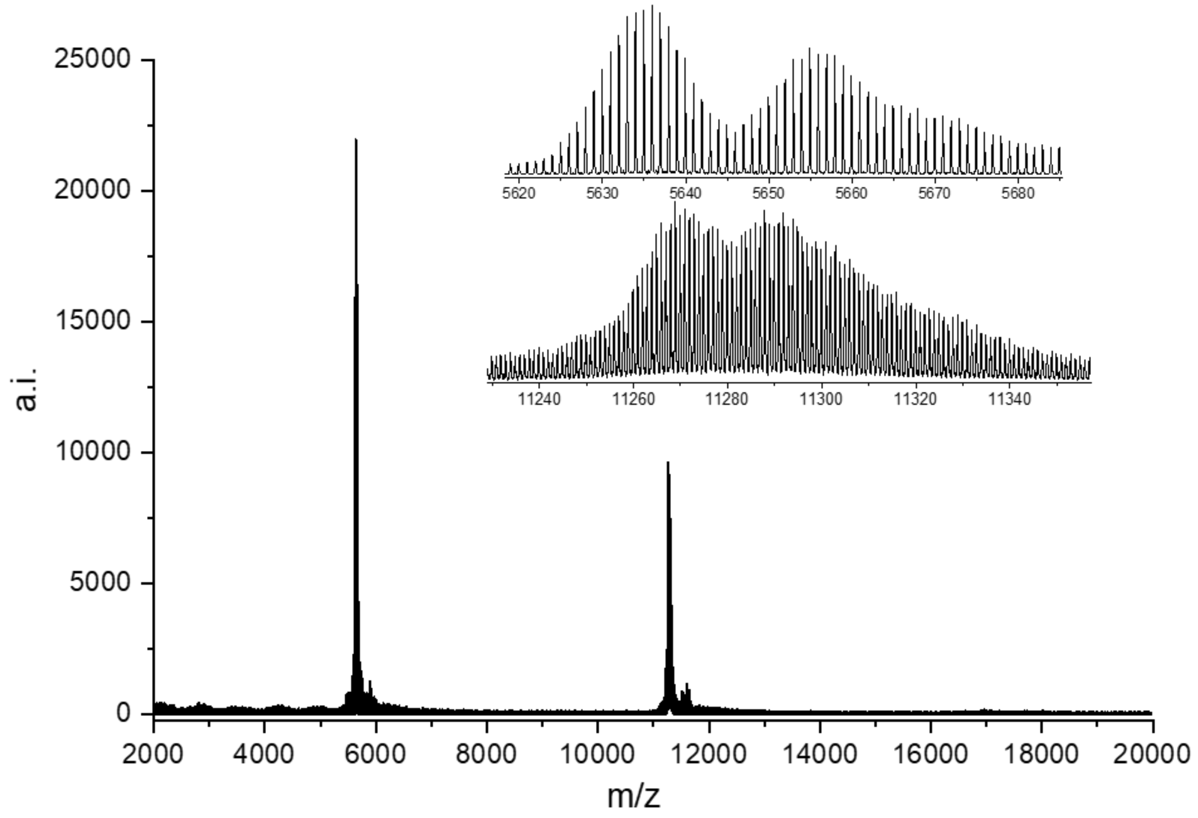

Figure 508. Full TIMS-TOF (DCTB) mass spectra of crude reaction mixture where 1:1 mixture of **OMe-cube** and ^{15}N labelled ***OMe-cube** was used for catenane reaction.

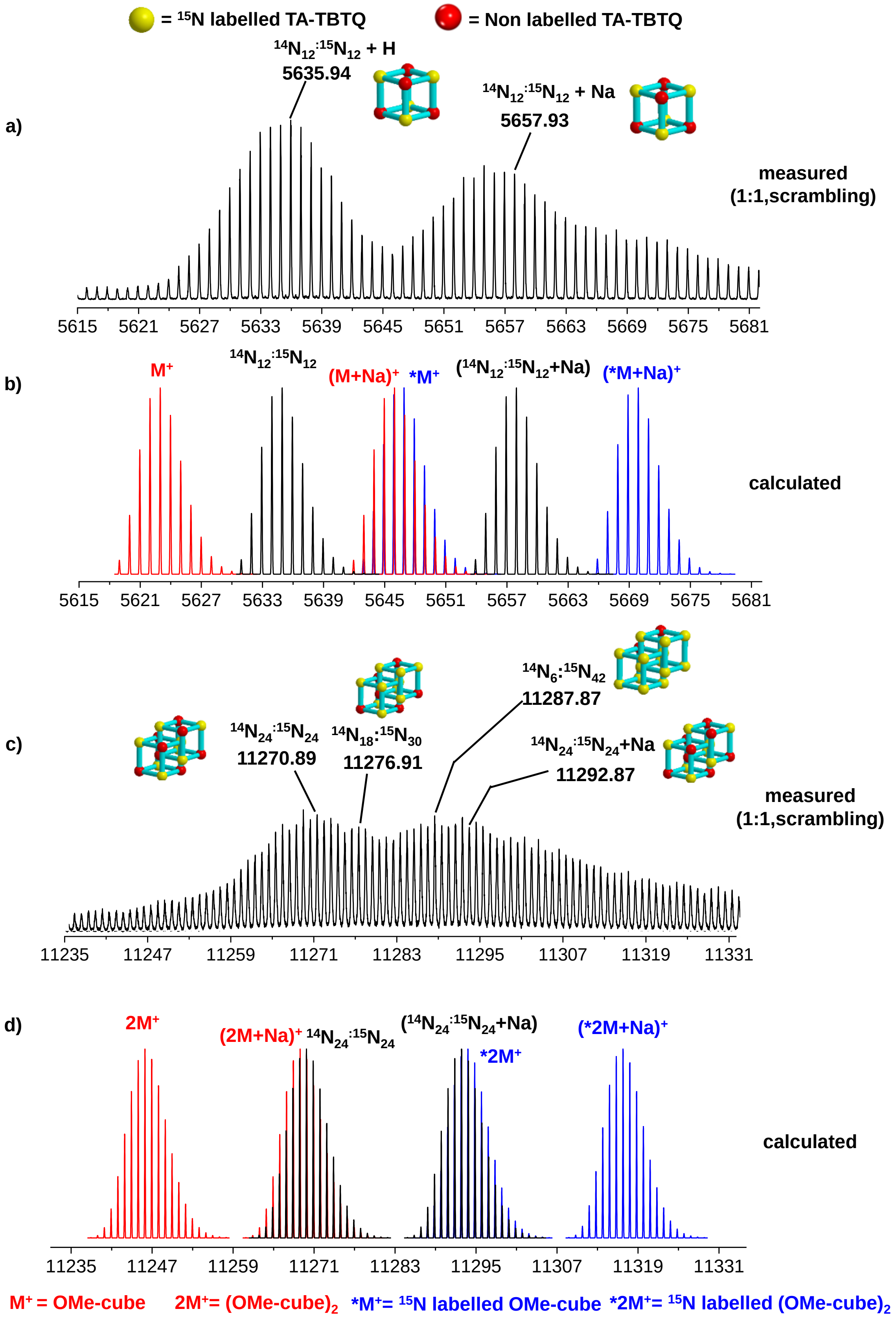

Figure 509. a) and **c)** partial TIMS-TOF (DCTB) mass spectra of crude reaction mixture where 1:1 mixture of **OMe-cube** and ^{15}N labelled ***OMe-cube** was used for catenane reaction. c) and d) calculated mass spectra.

2) Mechanistic Studies for OH-cube

Procedure: To a mixture of **OH-cube** (2 mg, 0.38 µmol) and ^{15}N labelled ***OH-cube** (2 mg, 0.38 µmol) in a 1:1 ratio in CD_2Cl_2 (0.53 mL), TFA (5.6 µL of a 0.131M solution in $CDCl_3$, 0.73 µmol TFA) was added and the reaction mixture was stirred for 3 days and then analysed by TIMS-TOF mass spectrometry.

a)

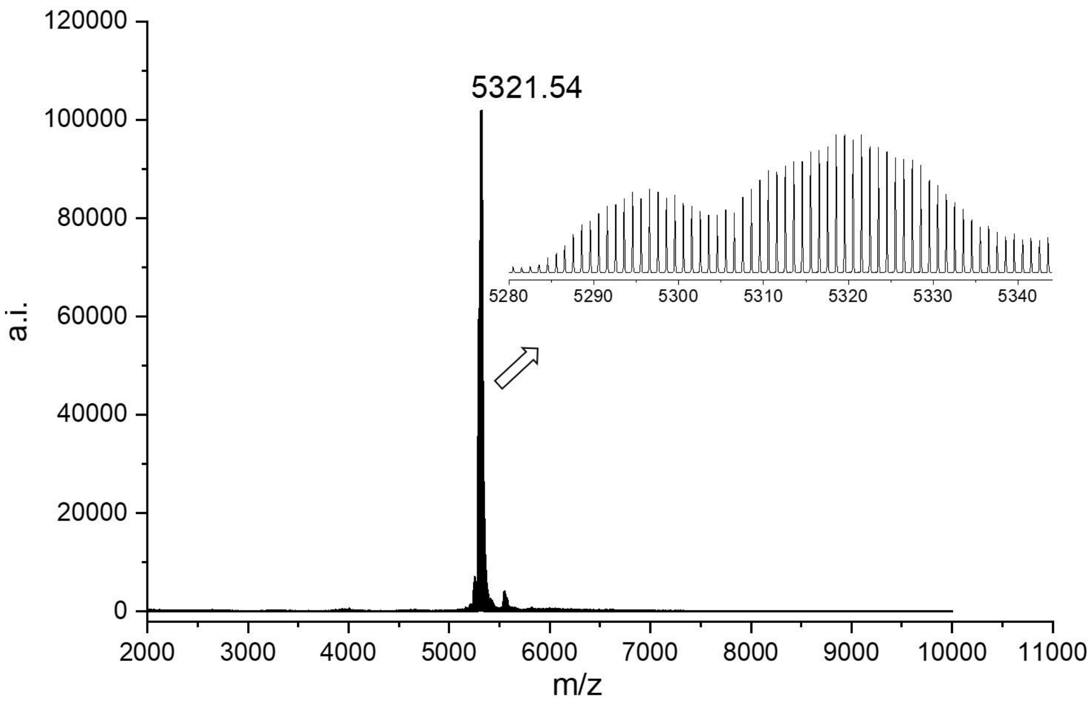

b)

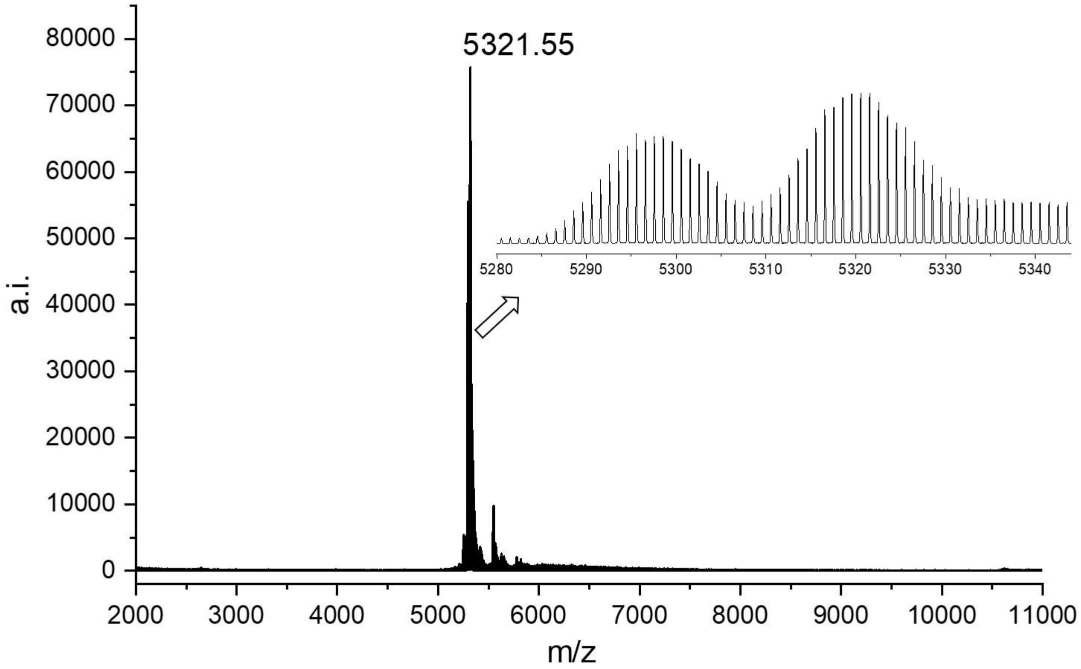

Figure 510. Full TIMS-TOF (DCTB) mass spectra of crude reaction mixture where 1:1 mixture of **OH-cube** and ^{15}N labelled ***OH-cube** was stirred at a) RT and b) at 80 °C for scrambling studies.

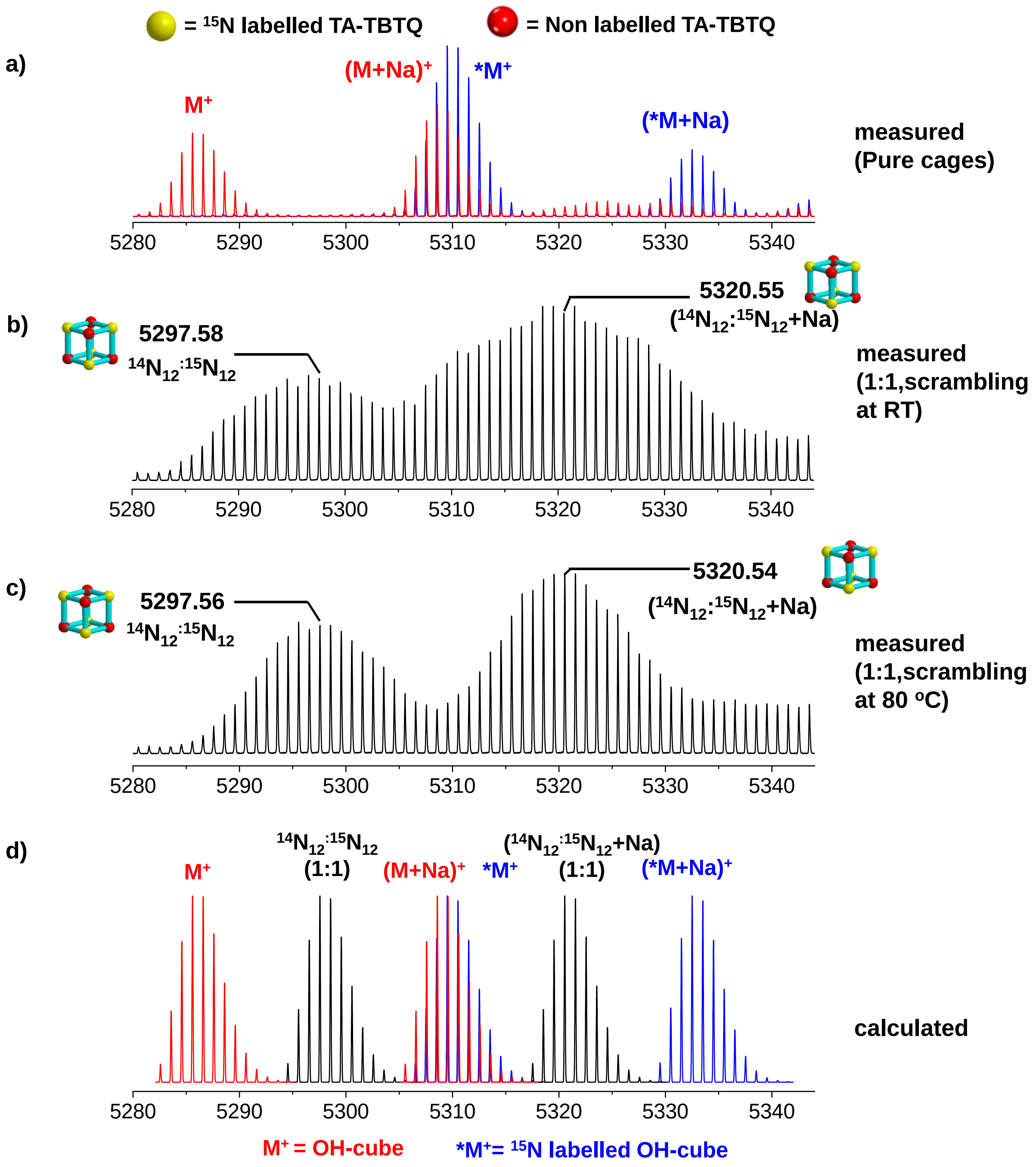

Figure 511. a) TIMS-TOF (DCTB) mass spectra of pure **OH-cube** and ^{15}N labelled ***OH-cube**. b) and c) TIMS-TOF (DCTB) mass spectra of reaction mixture where 1:1 mixture of **OH-cube** and ^{15}N labelled ***OH-cube** was used for catenane reaction at RT and at 80 °C respectively. d) calculated mass spectra of **M$^+$** (**OH-cube**), **$^{14}N_{12}$:$^{15}N_{12}$ (OH-cube: *OH-cube, 1:1), M+Na (OH-cube+Na), *M$^+$ (*OH-cube), $^{14}N_{12}$:$^{15}N_{12}$+Na (OH-cube: *OH-cube +Na, 1:1)** and ***M+Na (*OH-cube+Na)**.

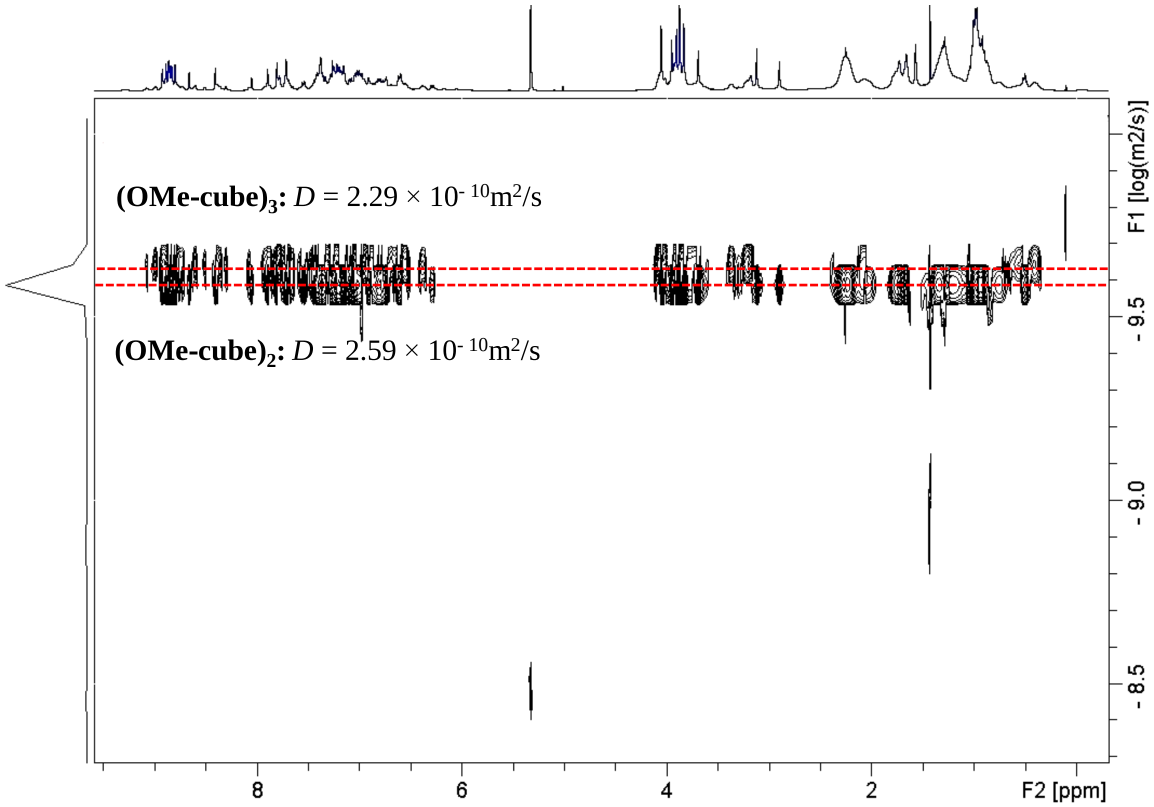

Figure 512: DOSY NMR of 1:1 mixture of pure **(OMe-cube)$_2$** (6.7 mg, 0.593 mmol) and **(OME-cube)$_3$** (10.0 mg, 0.593 µmol) in CD_2Cl_2 (0.5 mL).

18. Models of weak interactions

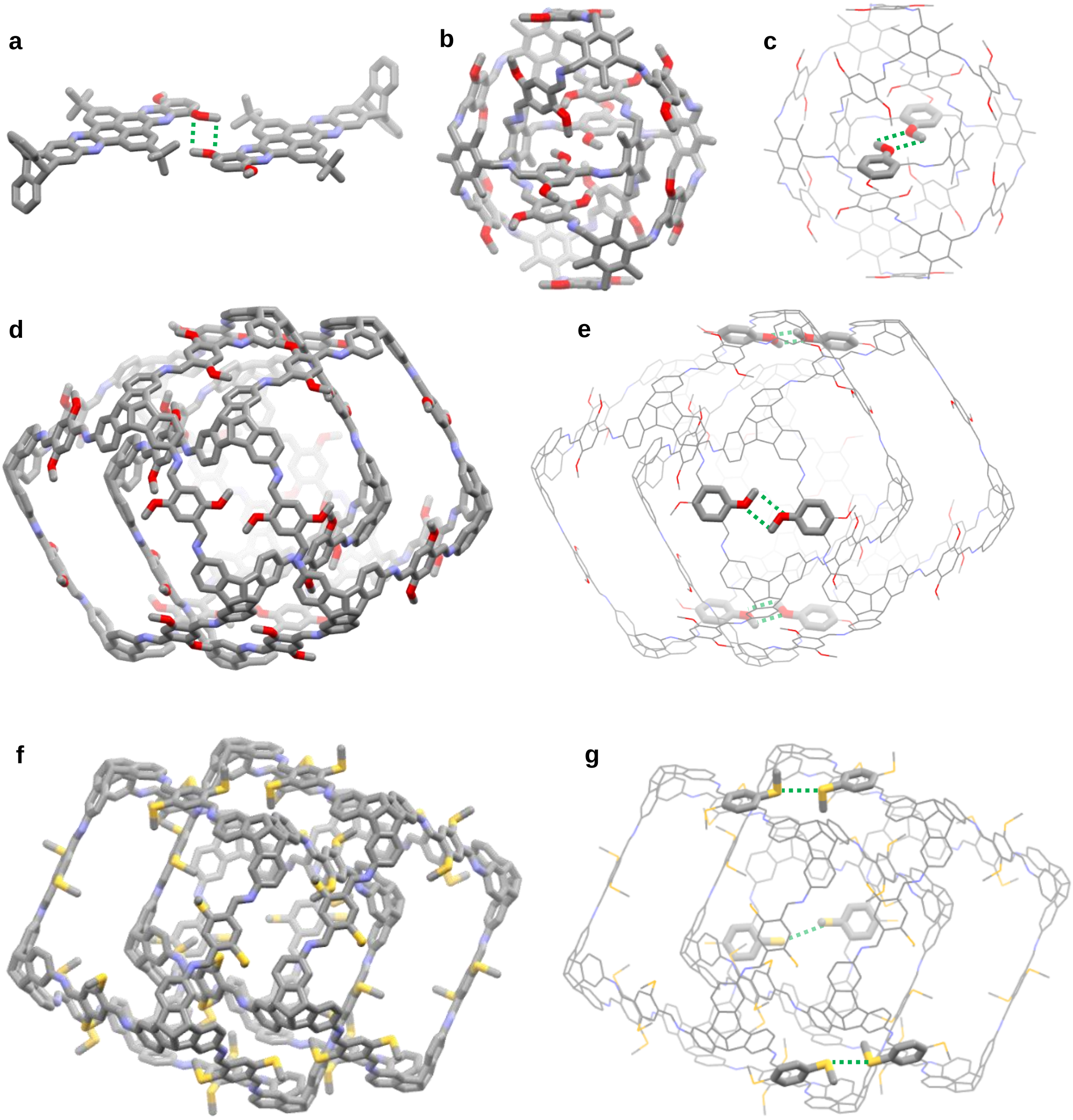

Figure 513. Comparison of weak interactions. **a** X-ray structure of a quinoxalinophenthrophenazine with methoxy-methoxy interactions ($d_{C\cdots O}$ = 3.1 Å).[S11] **b** and **c** Cooper's knot ($d_{C\cdots O}$= 3.5 Å).[S12] **d** and **e** Model of possible conformation of **(OMe-cube)$_2$** with three methoxy-methoxy interactions with $d_{C\cdots O}$ = 3.0 Å. **f** and **g** Model of possible conformation of **(SMe-cube)$_2$** with three sulfur-sulfur interactions with $d_{S\cdots S}$ = 3.8 Å.

19. Computational details

All quantum-chemical calculations were performed by employing the Gaussian16 program package.[13] The theoretical approach is based on the semi-empirical PM6 method.[14] The geometries of the regarded species were fully optimized, and ground states of the monomeric structures were confirmed by using frequency calculations to not exhibit any imaginary frequency. *For detailed computational data, see Supoprting Information File 2 (or xyz zip files).* Here, only the results are discussed.

Heat of formations

All heat of formations were calculated for minimum geometries. As 28 shows, conformer 2 is the more stable conformer for all cage compounds. The regarding geometries are shown in Figures 512, 513 and 522. For all cages the propyl chains of the TA-TBTQ units were shortened to methyl groups.

Table 28. PM6 calculated heat of formations for the different conformers of the cage compounds. For structures, see Figures 512, 513 and 522.

Cage Compound		$\Delta_f H$ [kJ mol^{-1}]
**OH-cube**	Type 1 conformer	742
	Type 2 conformer	728
**OMe-cube**	Type 1 conformer	1462
	Type 2 conformer	1438
**SMe-cube**	Type 1 conformer	5346
	Type 2 conformer	5326

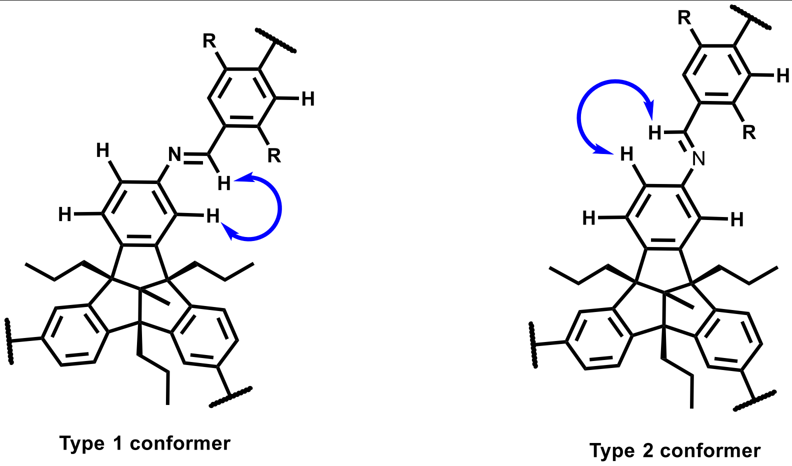

a)

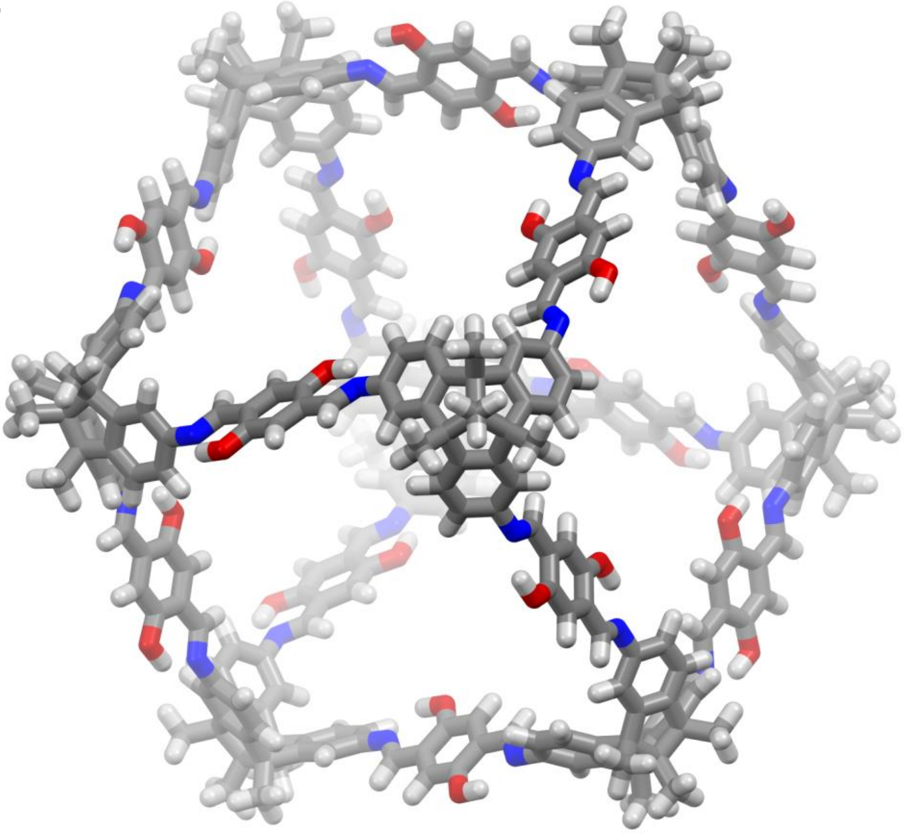

b)

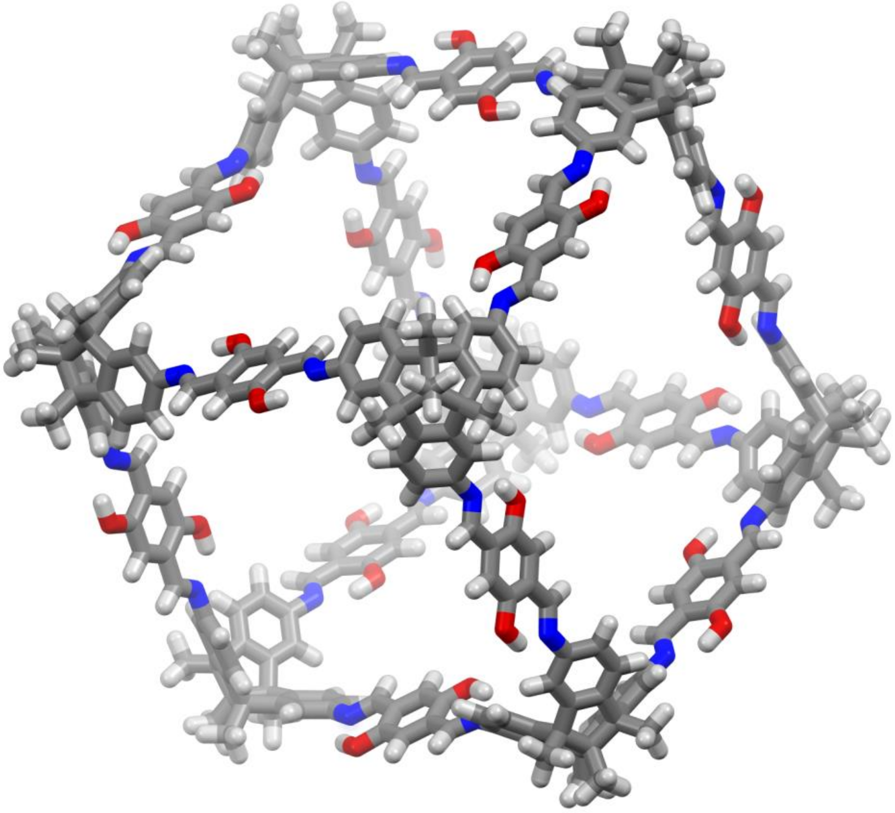

Figure 514. PM6 optimized structures of the **OH-cube**. **a)** Type 1 conformer, **b)** Type 2 conformer. white = hydrogen, grey = carbon, blue = nitrogen, red = oxygen.

a)

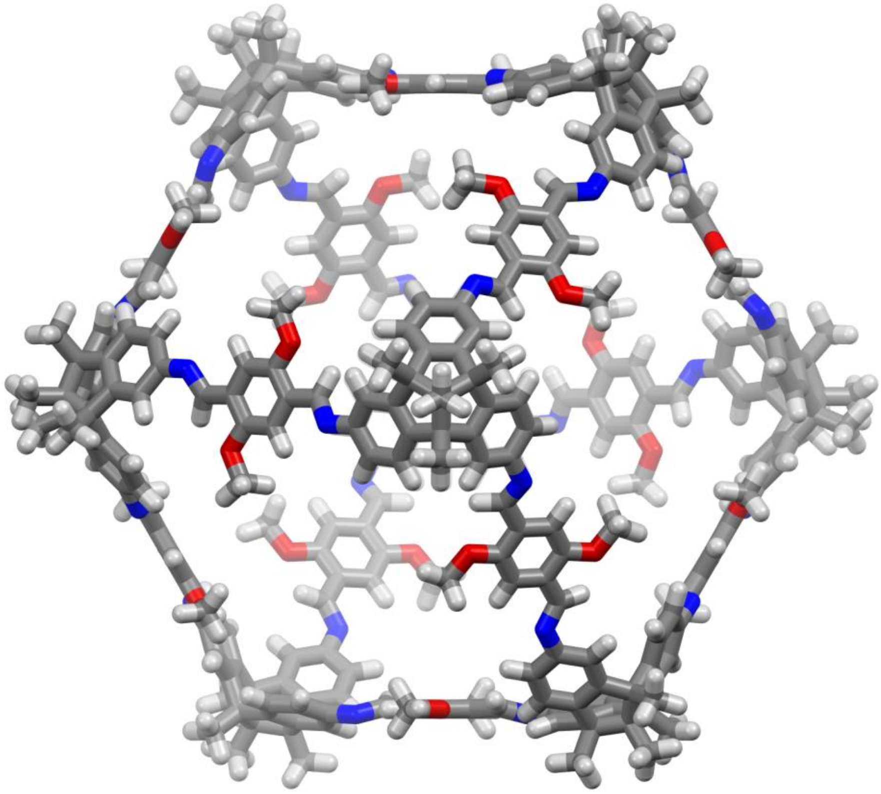

b)

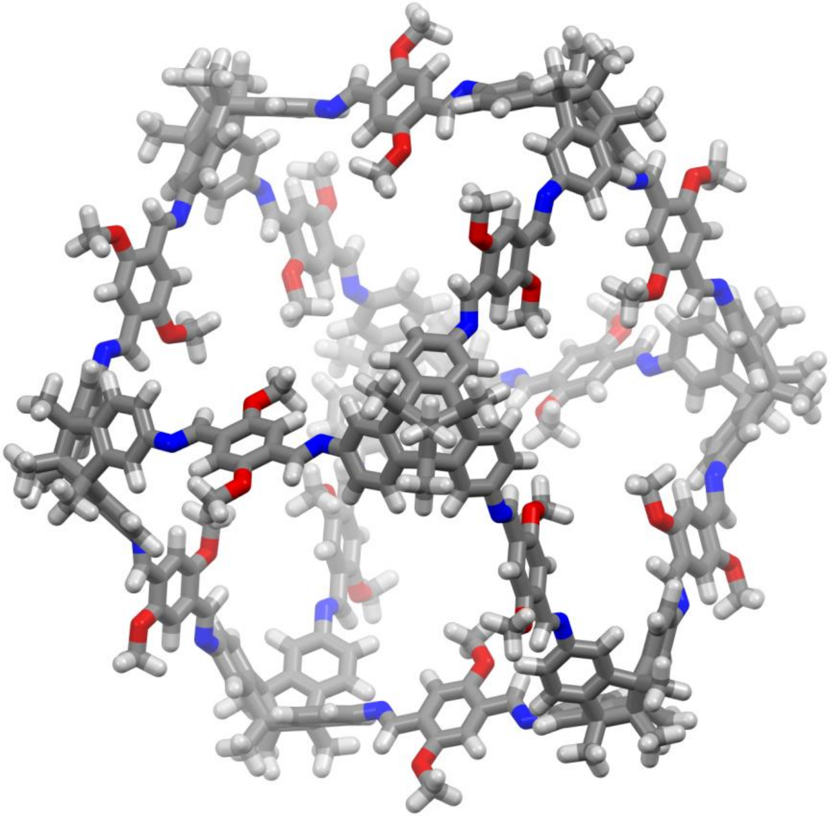

Figure 515. PM6 optimized structures of the **OMe-cube**. **a)** conformer 1, **b)** conformer 2. white = hydrogen, grey = carbon, blue = nitrogen, red = oxygen.

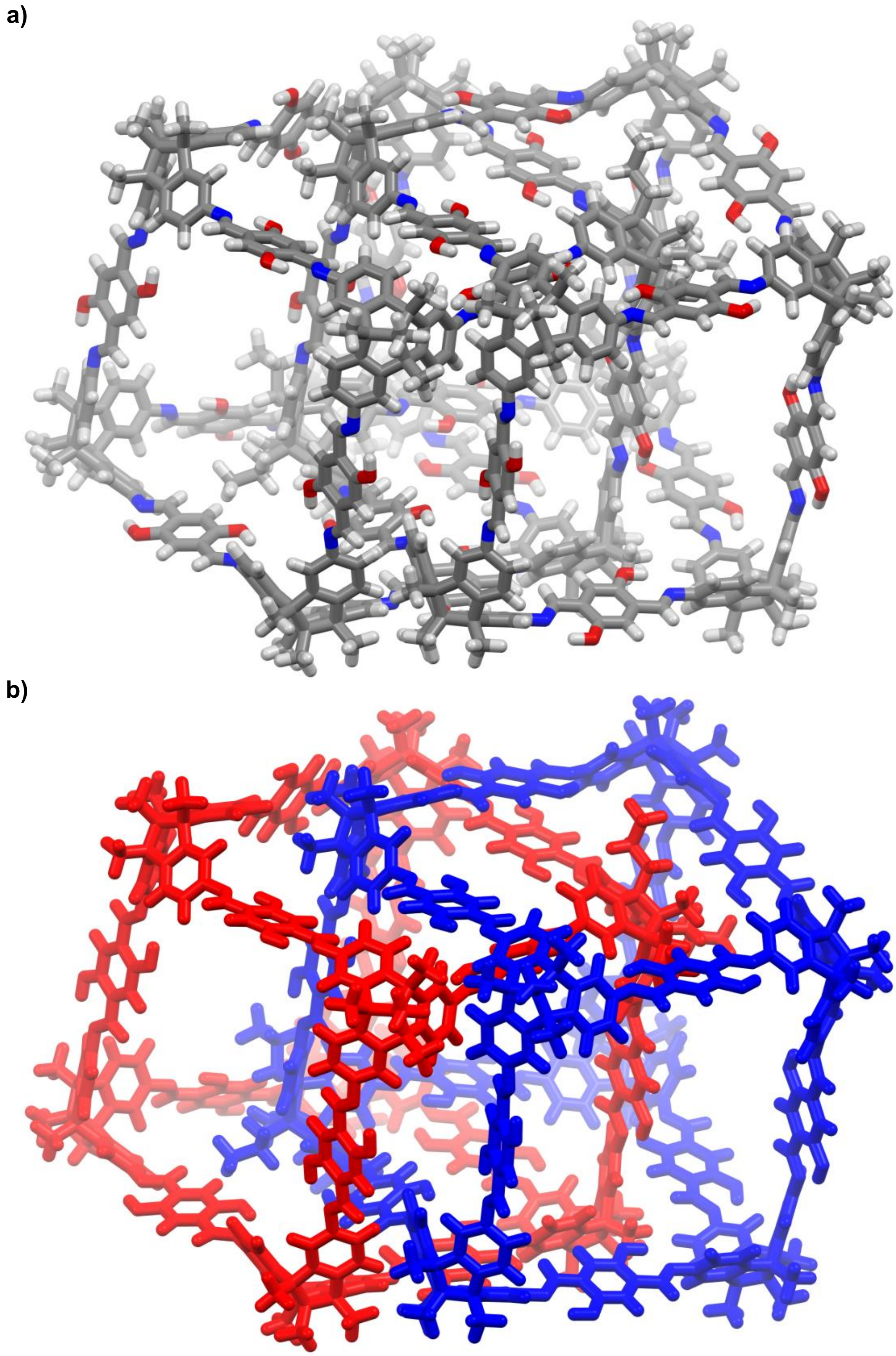

Figure 516. PM6 optimized structure of a hypothetically triply interlocked **(OH-cube)₂** (Type 1 conformer). **a)** structure coloured after the elements (white = hydrogen, grey = carbon, blue = nitrogen, red = oxygen) and **b)** highlighting both single cages in red and blue.

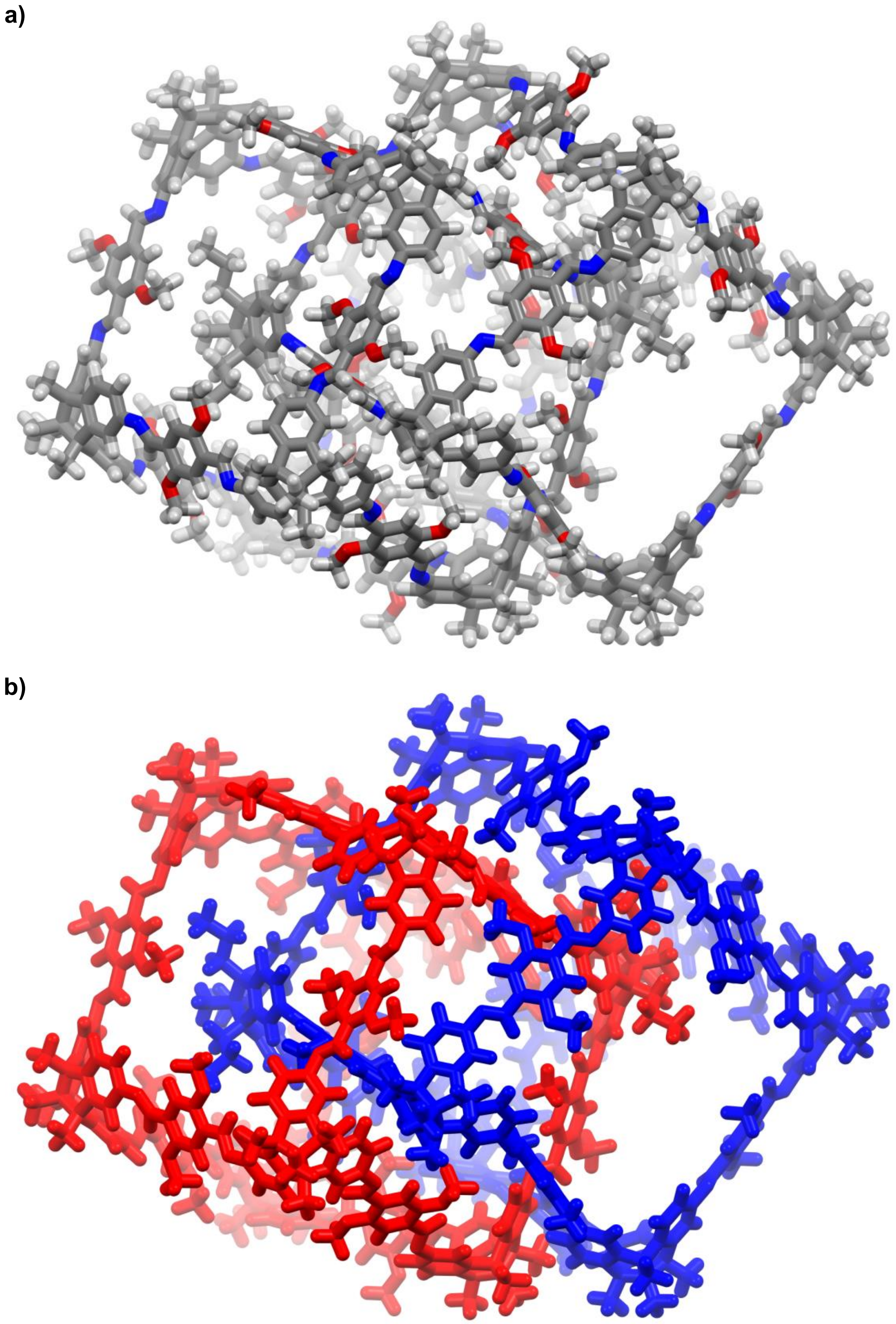

Figure 517. PM6 optimized structure of the triply interlocked (**OMe-cube)**$_2$ (Type 1 conformer). **a)** structure coloured after the elements (white = hydrogen, grey = carbon, blue = nitrogen, red = oxygen) and **b)** highlighting both single cages in red and blue.

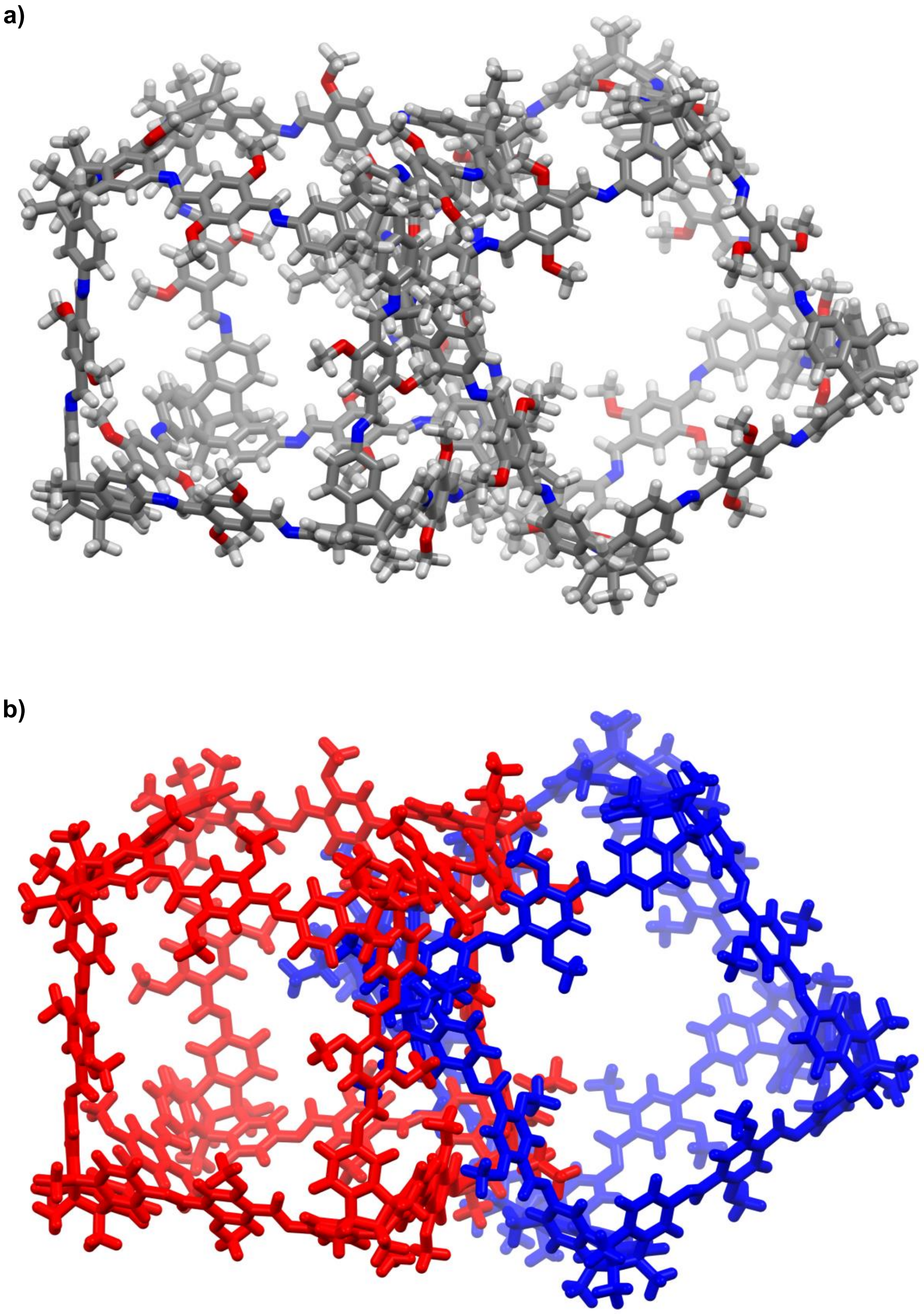

Figure 518. PM6 optimized structure of a hypothetical singly interlocked (**OMe-cube)**$_2$ (Type 1 conformer). **a)** structure coloured after the elements (white = hydrogen, grey = carbon, blue = nitrogen, red = oxygen) and **b)** highlighting both single cages in red and blue.

a)

b)

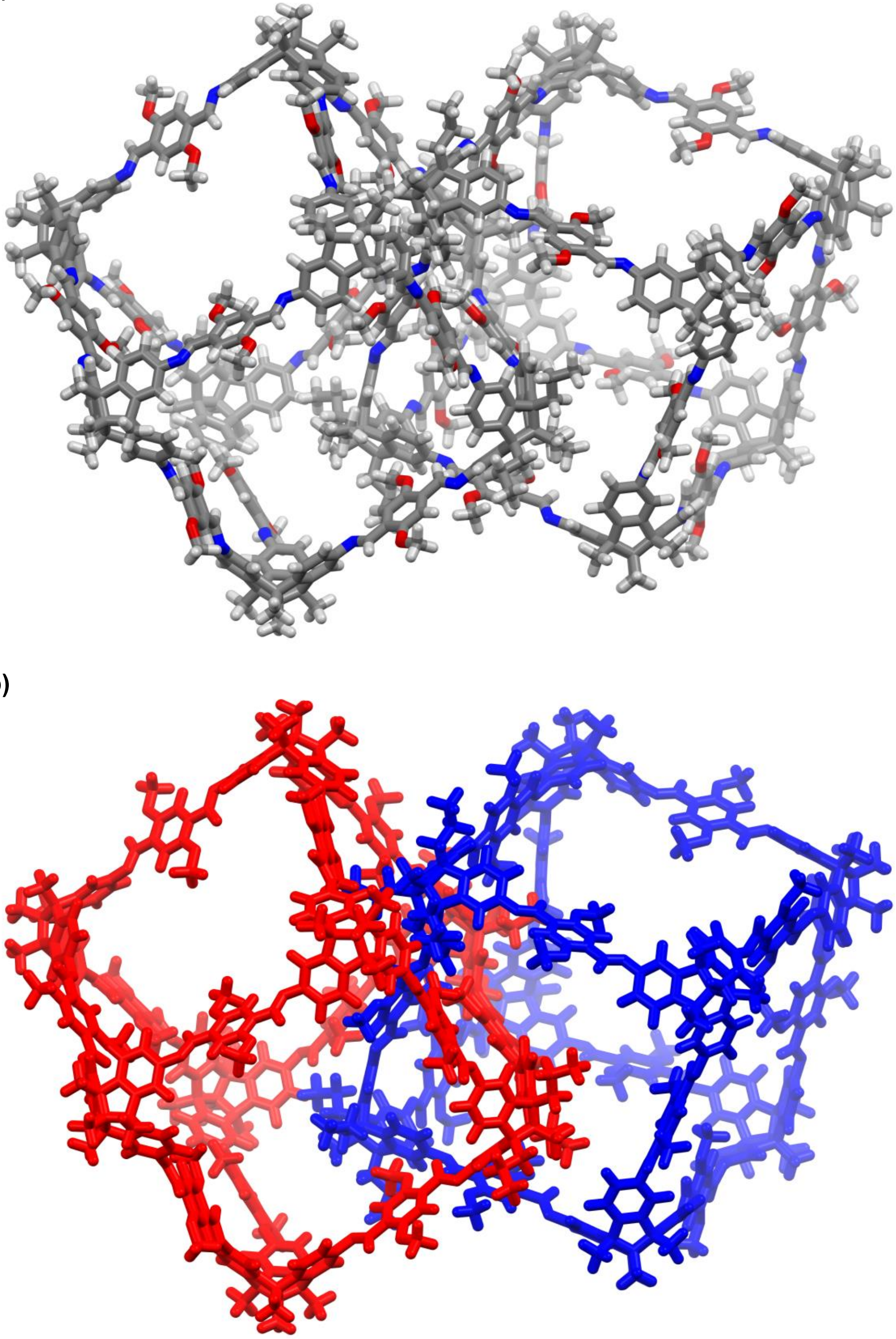

Figure 519. PM6 optimized structure of a hypothetical singly interlocked (**OMe-cube)$_2$** (Type 2 conformer). **a)** structure coloured after the elements (white = hydrogen, grey = carbon, blue = nitrogen, red = oxygen) and **b)** highlighting both single cages in red and blue.

a)

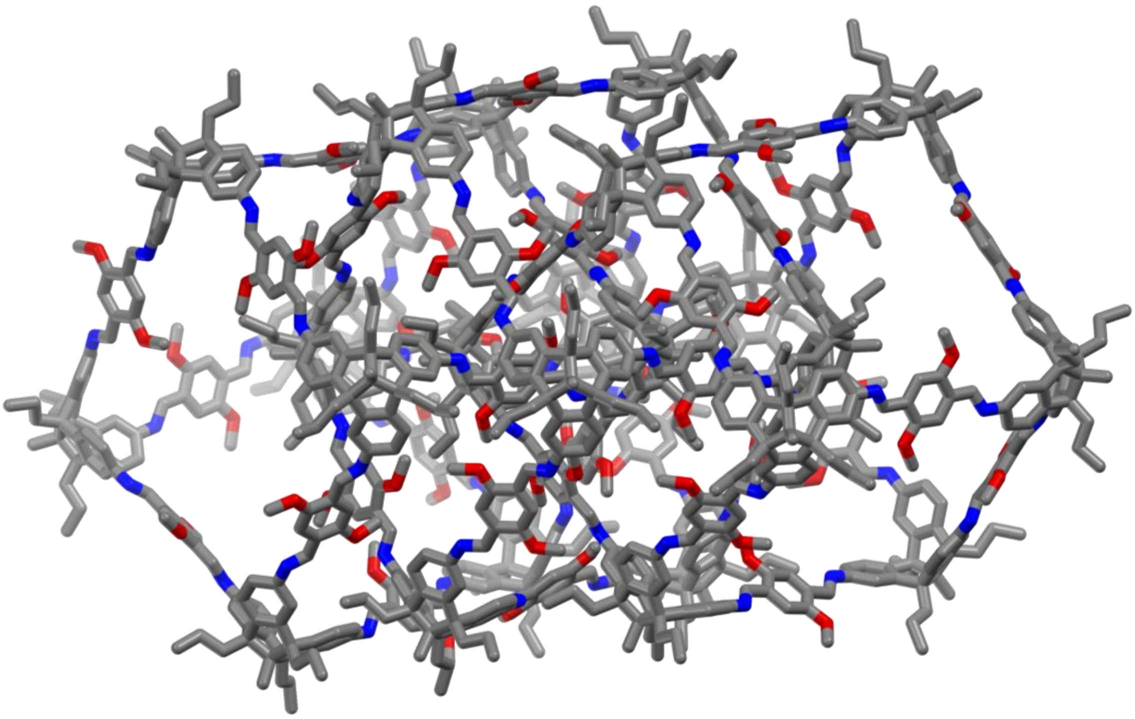

b)

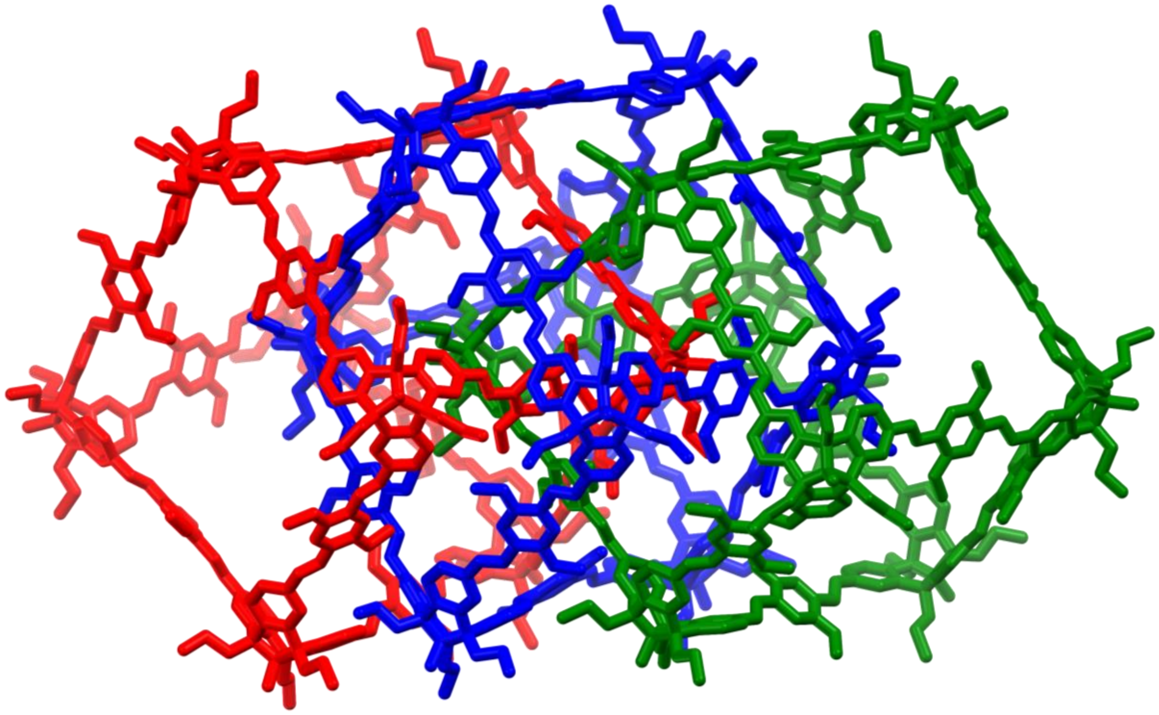

Figure 520. PM6 optimized structure of triply interlocked **[(OMe-cube)@(OMe-cube)]@(OMe-cube)** (Type 1 conformer). **a)** structure coloured after the elements (white = hydrogen, grey = carbon, blue = nitrogen, red = oxygen) and **b)** highlighting three cages in red, blue and green colour. Hydrogen atoms are omitted for clarity.

a)

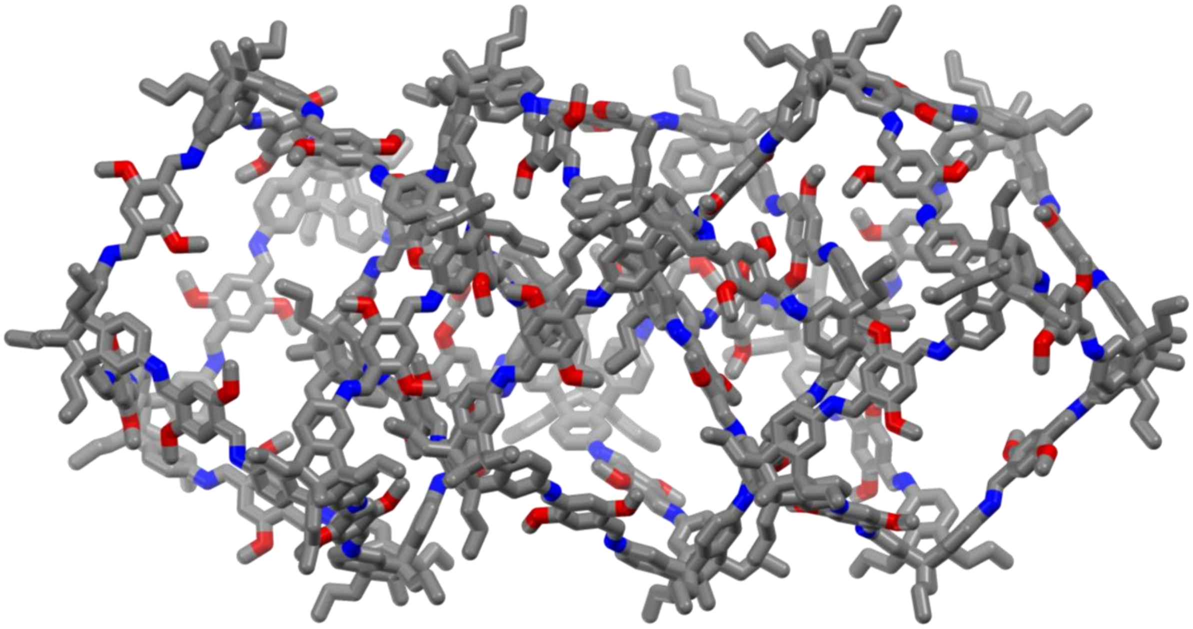

b)

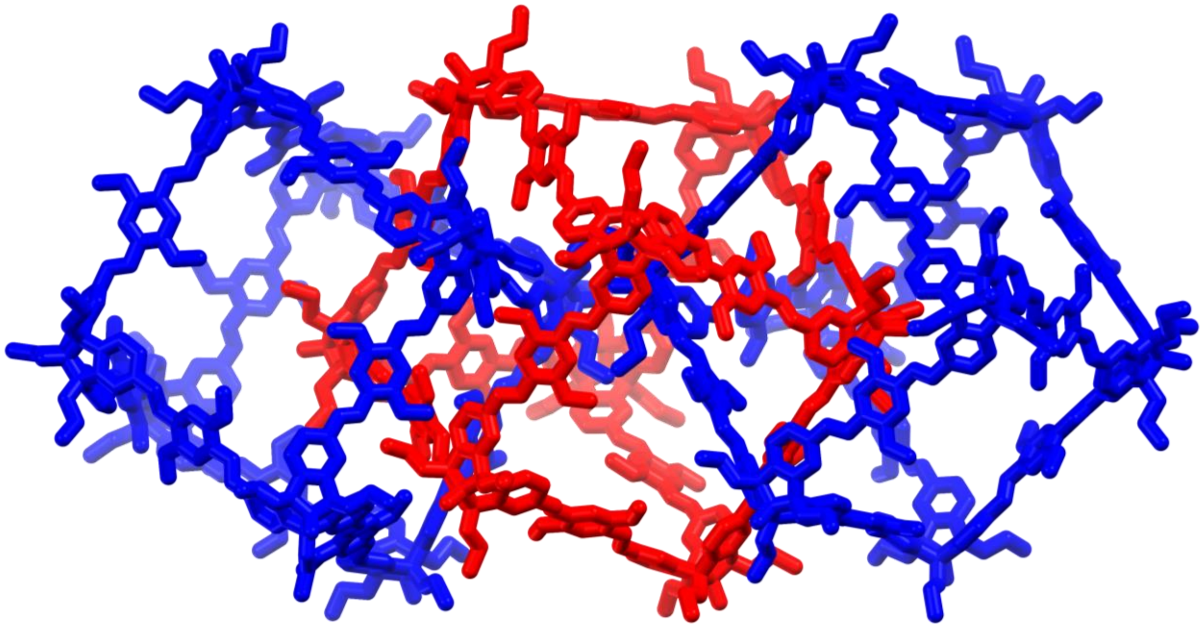

Figure 521. PM6 optimized structure of linear triply interlocked **(OMe-cube)@(OMe-cube)@(OMe-cube)** (Type 1 conformer, 1 and 4 position of middle cage is interlocked). **a)** structure coloured after the elements (white = hydrogen, grey = carbon, blue = nitrogen, red = oxygen) and **b)** highlighting outer two cages in blue color and middle in e colour. Hydrogen atoms are omitted for clarity.

a)

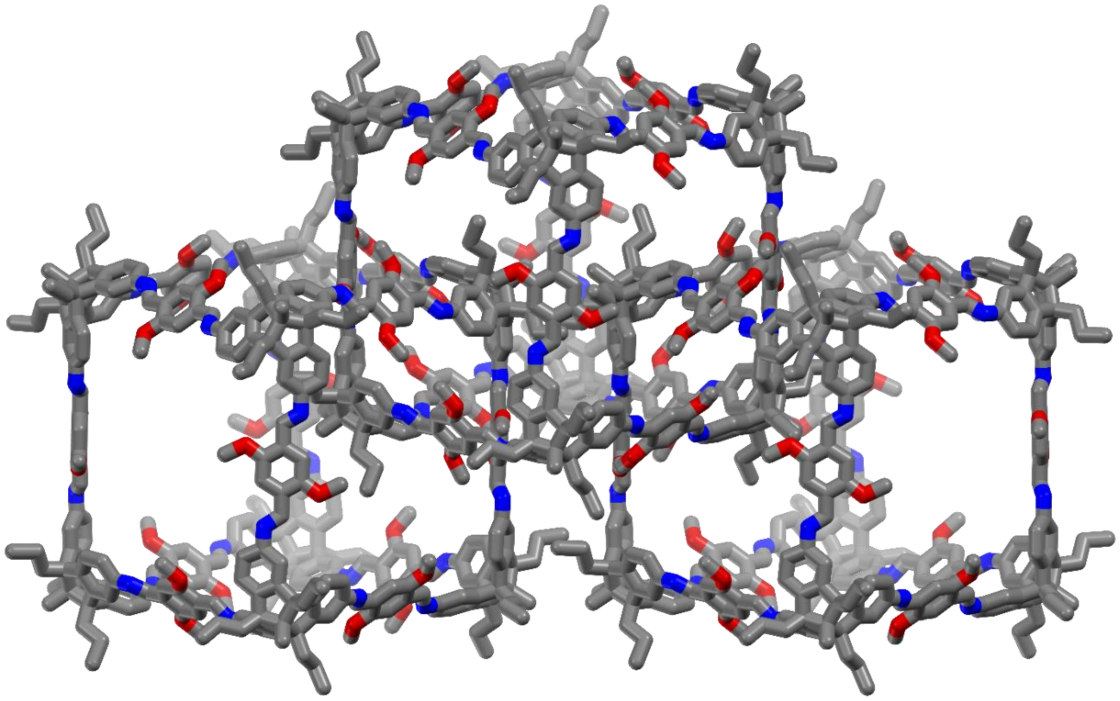

b)

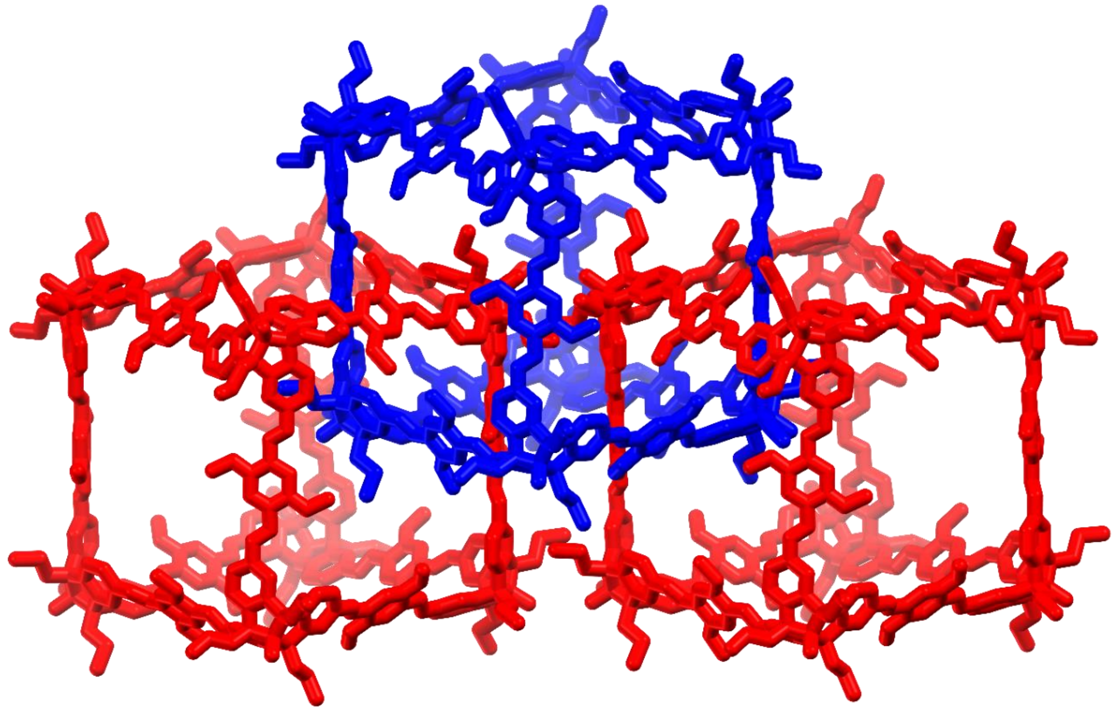

Figure 522. PM6 optimized structure of triply interlocked trimeric catenane with **syn-distal type** connectivity (middle cage is interlocked in 1 and 3 position) (Type 1 conformer). **a)** structure coloured after the elements (grey = carbon, blue = nitrogen, red = oxygen) and **b)** highlighting three cages in red and blue colour. Hydrogen atoms are omitted for clarity. **a)**

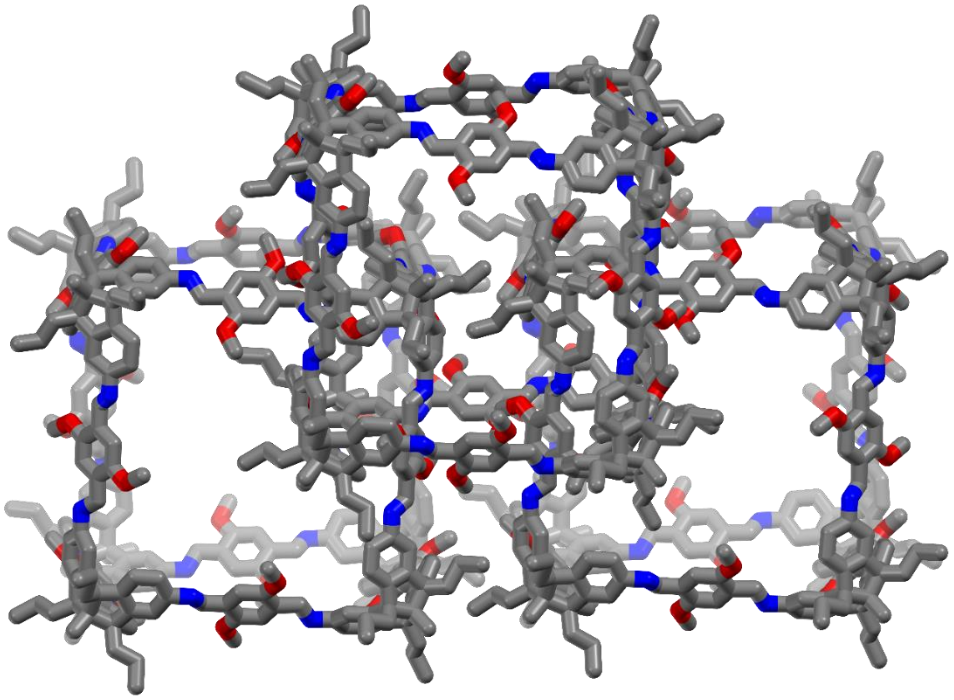

b)

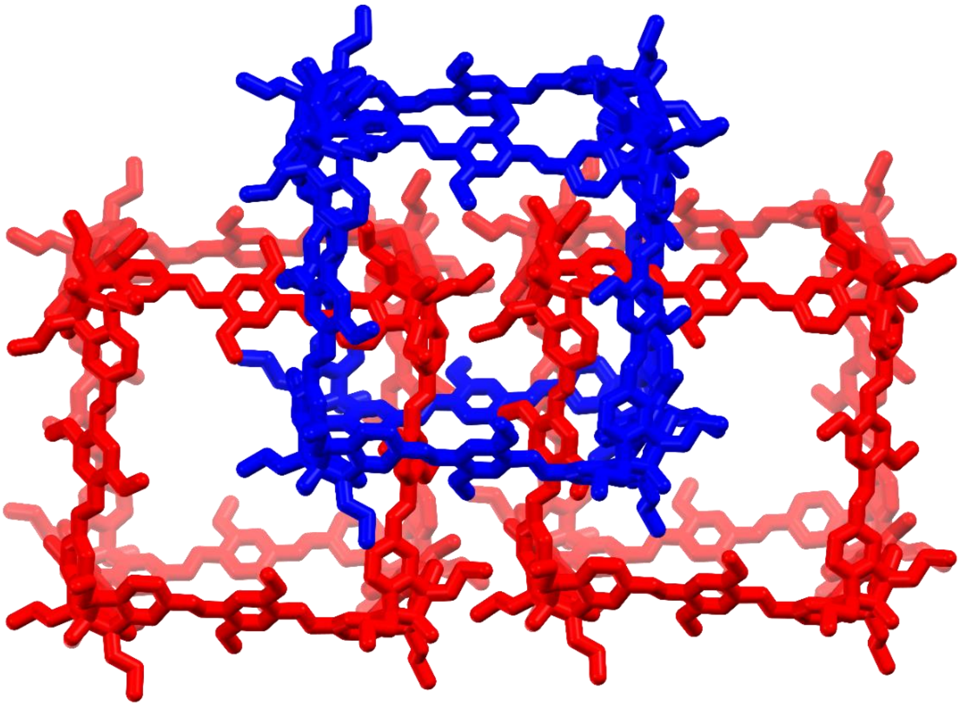

Figure 523. PM6 optimized structure of triply interlocked trimeric catenane with **syn-proximal type** connectivity (middle cage is interlocked in 1 and 2 position) (Type 1 conformer). **a)** structure coloured after the elements (grey = carbon, blue = nitrogen, red = oxygen) and **b)** highlighting three cages in red and blue colour. Hydrogen atoms are omitted for clarity.

a)

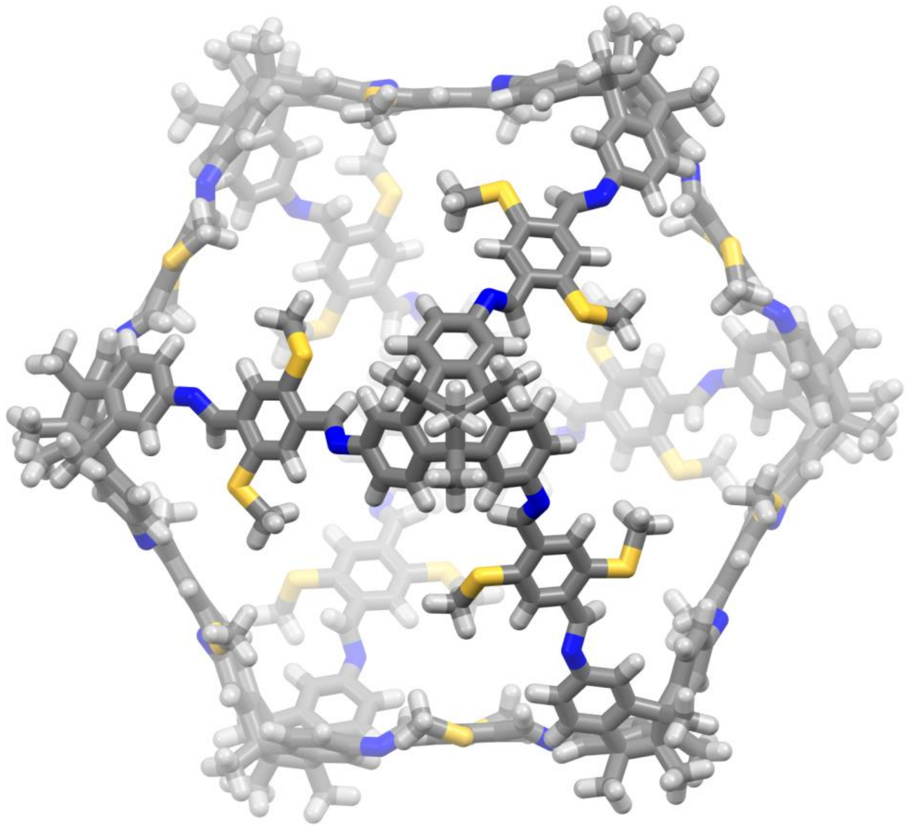

b)

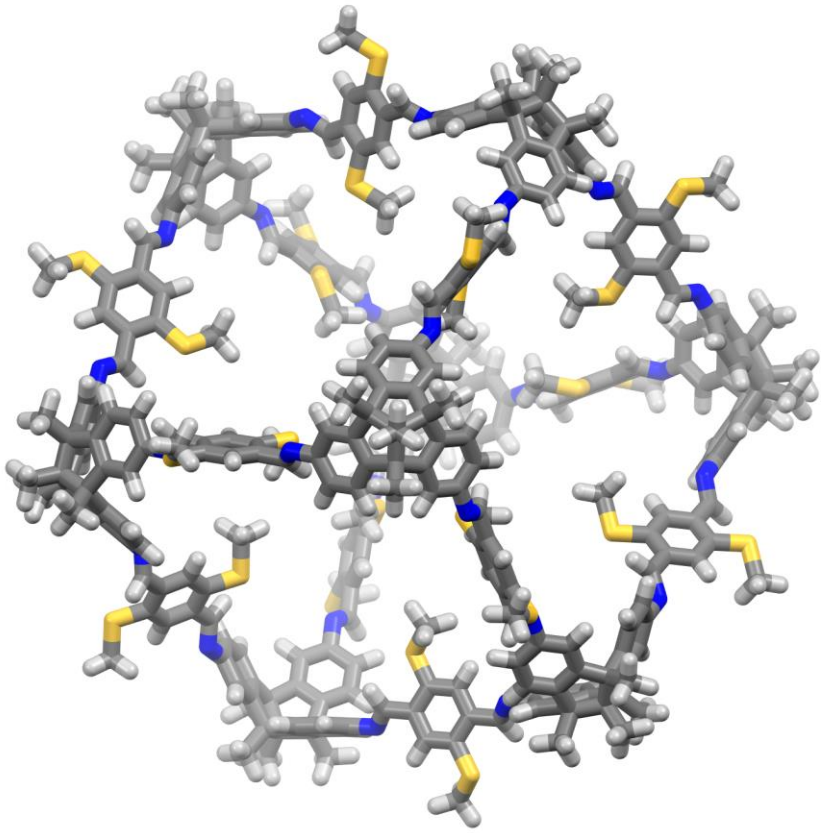

Figure 524. PM6 optimized structures of the **SMe-cube**. **a)** Type 1 conformer, **b)** Type 2 conformer. white = hydrogen, grey = carbon, blue = nitrogen, yellow = sulphur.

Table 29. Cage/catenane reactions reproducibility details

Entry	Cage/catenane	Cage/catenane Synthesis	Yield (%)
1	**OH cage**	1st run	84
		2nd run	85
		3rd run	82
2	**OMe cube**	1st run	80
		2nd run	76
3	**(OMe-cube)$_2$**	1st run	60
		2nd run	46
		3rd run	47
4	**(OMe-cube)$_3$**	1st run	80
		2nd run	79
		3rd run	84
5	**H-cube**	1st run	89
		2nd run	90
6	**Me-cube**	1st run	80
		2nd run	84
7	**Et-cube**	1st run	75
		2nd run	79
8	**(Et-cube)$_2$**	1st run	35
		2nd run	38
9	**OEt-cube**	1st run	84
		2nd run	81
10	**(OEt cube)$_2$**	1st run	25
		2nd run	32
11	**O-ipr-cube**	1st run	95
		2nd run	81
12	**SMe-cube**	1st run	86
		2nd run	80
13	**(Sme-cube)$_2$**	1st run	70
		2nd run	66
14	**(SMe-cube)$_2$**	1st run	58
		2nd run	54
15	**SC(CH$_3$)$_3$-cube**	1st run	75
		2nd run	81
16	**Br-cube**	1st run	93
		2nd run	92
17	**H/OMe-cube**	1st run	35
		2nd run	36
18	**(H/OMe-cube)$_2$**	1st run	42
		2nd run	44

20.References

[1] a) D. Beaudoin, F. Rominger, M. Mastalerz, *Eur. J. Org. Chem.* **2016**, *26*, 4470-4472. b) A. Palmgren, A. Thorarensen, J.-E. Bäckvall, *J. Org. Chem.* **1998**, *63*, 3764-3768; c) N. Kuhnert, G. M. Rossignolo, A. Lopez-Periago, *Org. Biomol. Chem.* **2003**, *1*, 1157-1170; d) T. Kretz, W. Bats Jan, H.-W. Lerner, M. Wagner, *Z. Naturforsch.* **2007**, *62*, 66;

[2] A. Orita, H. Taniguchi, J. Otera, *Chem. Asian J.* **2006**, *1*, 430-437.

[3] Y. Zeng and coworkers *Angew. Chem. Int. Ed.* **2019**, *58*, 4906–4910.

[4] M. S. Kwon, J. Gierschner, S. J. Yoon, S. Y. Park, *Adv. Mater.* **2012**, *24*, 5487-5492.

[5] T. Yamamoto, T. Nishimura, T. Mori, E. Miyazaki, I. Osaka, K. Takimiya, *Org. Lett.* **201**2, *14*, 4914-4917.

[6] Q. Sun, B. Aguila, J. Perman, L. D. Earl, C.W. Abney, Y. Cheng, H. Wei, N. Nguyen, L. Wojtas, S. Ma, *J. Am. Chem. Soc.* **2017**, *139*, 2786-2793. 2017.

[7] K. Otake, M. Matsumoto, S. Tanaka, S. Uchida, R. Goseki, A. Hirao, T. Ishizone, *Macromol. Chem. Phys.* **2017**, *218*, 1600550.

[8] S. Viel, F. Ziarelli, G. Pages, C. Carrara and S. Caldarelli, *Journal of Magnetic Resonance* **2008**, *190*, 113-123.

[9] H. C. Chen and S. H. Chen, *J. Phys. Chem.* **1984**, *88*, 5118-5121.

[10] Y. H. Zhao, M. H. Abraham and A. M. Zissimos, *J. Org. Chem.* **2003**, *68*, 7368-7373.

[11] L. Uebbericke, D. Holub, J. Kranz, F. Rominger, M. Elstner, M. Mastalerz, Chem. Eur. J. 2019, 25,11121-11134.

[12] R. L. Greenaway et al. *Nat. Commun.* **2018**, *9*, 2849-2860-

[13] M. J. Frisch, G. W. Trucks, H. B. Schlegel, G. E. Scuseria, M. A. Robb, J. R. Cheeseman, G. Scalmani, V. Barone, G. A. Petersson, H. Nakatsuji, X. Li, M. Caricato, A. V. Marenich, J. Bloino, B. G. Janesko, R. Gomperts, B. Mennucci, H. P. Hratchian, J. V. Ortiz, A. F. Izmaylov, J. L. Sonnenberg, Williams, F. Ding, F. Lipparini, F. Egidi, J. Goings, B. Peng, A. Petrone, T. Henderson, D. Ranasinghe, V. G. Zakrzewski, J. Gao, N. Rega, G. Zheng, W. Liang, M. Hada, M. Ehara, K. Toyota, R. Fukuda, J. Hasegawa, M. Ishida, T. Nakajima, Y. Honda, O. Kitao, H. Nakai, T. Vreven, K. Throssell, J. A. Montgomery Jr., J. E. Peralta, F. Ogliaro, M. J. Bearpark, J. J. Heyd, E. N. Brothers, K. N. Kudin, V. N. Staroverov, T. A. Keith, R. Kobayashi, J. Normand, K. Raghavachari, A. P. Rendell, J. C. Burant, S. S. Iyengar, J. Tomasi, M. Cossi, J. M. Millam, M. Klene, C. Adamo, R. Cammi, J.

W. Ochterski, R. L. Martin, K. Morokuma, O. Farkas, J. B. Foresman, D. J. Fox: Gaussian 16 Rev. C.01, Wallingford, CT, 2016.
[14] J. J. P. Stewart, J. Mol. Model. 2007, 13, 1173-1213.
