# Supplementary material for: Dimeric and trimeric catenation of giant chiral [8 + 12] imine cubes driven by weak supramolecular interactions
Source: Nat Chem. 2022 Dec 1;15(3):413–23. doi: 10.1038/s41557-022-01094-w (PMC9986109; doi:10.1038/s41557-022-01094-w)
Supplement: Supplementary file 2 — Xyz files of computational data. [file 41557_2022_1094_MOESM2_ESM.zip › xyz coordinates.doc]

**Dimeric and Trimeric Catenation of Giant Chiral [8+12] Imine Cubes Driven by Weak Supramolecular Interactions**

Bahiru P. Benke, ^[a]^ Tobias Kirschbaum,^[a]^ Jürgen Graf, ^[a]^ and Michael Mastalerz*^[a]^

**Table of Contents**

1. **Computational details	3**
2. **Heat of formations	3**
3. **PM6 optimized structures of cages and catenane	4**
   a. **OH-cube (Type 1 conformer)	4**
   b. **OH-cube (Type 2 conformer)	4**
   c. **(OH-cube)_2_ (triply interlocked- Type 1 conformer)	5**
   d. **OMe-cube (Type 1 conformer)	6**
   e. **OMe-cube (Type 2 conformer)	6**
   f. **(OMe-cube)_2_ (Triply interlocked- Type 1 conformer)	7**
   g. **(OMe-cube)_2_ (Singly interlocked- Type 1 conformer)	8**
   h. **(OMe-cube)_2_ (Singly interlocked- Type 2 conformer)	9**
   i. **[(OMe-cube)@(OMe-cube)]_@_ (OMe-cube) (Type 1 conformer)	10**
   j. **(OMe-cube)≡(OMe-cube)≡(OMe-cube) (Type 1 conformer, 1 and 4**

**position of middle cage interlocked)	11**

k. **(OMe-cube)≡(OMe-cube)≡(OMe-cube) (Type 1 conformer, 1 and 2**

**position of middle cage interlocked)	12**

l. **(OMe-cube)≡(OMe-cube)≡(OMe-cube) (Type 1 conformer, 1 and 3**

**position of middle cage interlocked)	13**

m. **SMe-cube (Type 1 conformer)	14**
n. **SMe-cube (Type 2 conformer)	14**

4. **XYZ coordinates of cages and catenanes	15**
   a. **OH-cube (Type 1 conformer)	15**
   b. **OH-cube (Type 2 conformer)	27**
   c. **(OH-cube)_2_ (triply interlocked- Type 1 conformer)	38**
   d. **OMe-cube (Type 1 conformer)	60**
   e. **OMe-cube (Type 2 conformer)	72**
   f. **(OMe-cube)_2_ (Triply interlocked- Type 1 conformer)	84**
   g. **(OMe-cube)_2_ (Singly interlocked- Type 1 conformer)	109**
   h. **(OMe-cube)_2_ (Singly interlocked- Type 2 conformer)	135**
   i. **[(OMe-cube)@(OMe-cube)]_@_ (OMe-cube) (Type 1 conformer)	161**
   j. **(OMe-cube)≡(OMe-cube)≡(OMe-cube) (Type 1 conformer, 1 and 4**

**position of middle cage interlocked)	205**

k. **OMe-cube)≡(OMe-cube)≡(OMe-cube) (Type 1 conformer, 1 and 2**

**position of middle cage interlocked)	249**

l. **OMe-cube)≡(OMe-cube)≡(OMe-cube) (Type 1 conformer, 1 and 3**

**position of middle cage interlocked)	293**

m. **SMe-cube (Type 1 conformer)	337**
n. **SMe-cube (Type 2 conformer)	349**

5. **References	361**

.

**1. Computational details**

All quantum-chemical calculations were performed by employing the Gaussian16 program package. ^[S1]^ The theoretical approach is based on the semi-empirical PM6 method. ^[S2]^ The geometries of the regarded species were fully optimized, and ground states of the monomeric structures were confirmed by using frequency calculations to not exhibit any imaginary frequency.

**2. Heat of formations**

All heat of formations were calculated for minimum geometries. As shows, conformer 2 is the more stable conformer for all cage compounds. The regarding geometries are shown in Figure S2, S4 and S12. For all cages the propyl chains of the TA-TBTQ units were shortened to methyl groups.

**Table 1.** PM6 calculated heat of formations for the different conformers of the cage compounds. For structures, see Figures S2, S4 and S12.

| **Cage Compound** | | **Δ_f_H [kJ mol^-1^]** |
| --- | --- | --- |
| **OH-cube** | Type 1 conformer | 742 |
|  | Type 2 conformer | 728 |
| **OMe-cube** | Type 1 conformer | 1462 |
|  | Type 2 conformer | 1438 |
| **SMe-cube** | Type 1 conformer | 5346 |
|  | Type 2 conformer | 5326 |

**Figure 1.** Partial [8+12] cage structure showing type 1 and type 2 conformers.

**3. PM6 optimized structures of cages and catenanes**

**Figure 2.** PM6 optimized structures of the **OH-cube**. **a)** Type 1 conformer, **b)** Type 2 conformer. white = hydrogen, grey = carbon, blue = nitrogen, red = oxygen.

**Figure 3.** PM6 optimized structure of a hypothetically triply interlocked **(OH-cube)_2_** (Type 1 conformer). **a)** structure coloured after the elements (white = hydrogen, grey = carbon, blue = nitrogen, red = oxygen) and **b)** highlighting both single cages in red and blue.

**Figure 4.** PM6 optimized structures of the **OMe-cube**. **a)** Type 1 conformer **b)** Type 2 conformer white = hydrogen, grey = carbon, blue = nitrogen, red = oxygen.

**Figure 5.** PM6 optimized structure of the triply interlocked (**OMe-cube)_2_** (Type 1 conformer). **a)** structure coloured after the elements (white = hydrogen, grey = carbon, blue = nitrogen, red = oxygen) and **b)** highlighting both single cages in red and blue.

**Figure 6.** PM6 optimized structure of a hypothetical singly interlocked (**OMe-cube)_2_** (Type 1 conformer). **a)** structure coloured after the elements (white = hydrogen, grey = carbon, blue = nitrogen, red = oxygen) and **b)** highlighting both single cages in red and blue.

**Figure 7.** PM6 optimized structure of a hypothetical singly interlocked (**OMe-cube)_2_** (Type 2 conformer). **a)** structure coloured after the elements (white = hydrogen, grey = carbon, blue = nitrogen, red = oxygen) and **b)** highlighting both single cages in red and blue.

**a)**

**b)**

**Figure 8.** PM6 optimized structure of triply interlocked [(**OMe-cube)@**(**OMe-cube)]_@_** (**OMe-cube)** (Type 1 conformer). **a)** structure coloured after the elements (white = hydrogen, grey = carbon, blue = nitrogen, red = oxygen) and **b)** highlighting three cages in red, blue and green colour. Hydrogen atoms are omitted for clarity.

**a)**

**b)**

**Figure 9.** PM6 optimized structure of linear triply interlocked (**OMe-cube)≡**(**OMe-cube)**≡(**OMe-cube)** (Type 1 conformer, 1 and 4 position of middle cage is interlocked). **a)** structure coloured after the elements (white = hydrogen, grey = carbon, blue = nitrogen, red = oxygen) and **b)** highlighting outer two cages in blue color and middle in e colour. Hydrogen atoms are omitted for clarity.

**a)**

**b)**

**Figure 10.** PM6 optimized structure of triply interlocked (**OMe-cube)≡**(**OMe-cube)**≡(**OMe-cube)** (Type 1 conformer, 1 and 2 position of middle cage is interlocked). **a)** structure coloured after the elements (grey = carbon, blue = nitrogen, red = oxygen) and **b)** highlighting three cages in red and blue colour. Hydrogen atoms are omitted for clarity.

**a)**

**b)**

**Figure 11.** PM6 optimized structure of triply interlocked (**OMe-cube)≡**(**OMe-cube)**≡(**OMe-cube)** (Type 1 conformer, 1 and 3 position of middle cage is interlocked). **a)** structure coloured after the elements (grey = carbon, blue = nitrogen, red = oxygen) and **b)** highlighting three cages in red and blue colour. Hydrogen atoms are omitted for clarity.

**Figure 12**. PM6 optimized structures of the **SMe-cube**. **a)** Type 1 conformer, **b)** Type 2 conformer. white = hydrogen, grey = carbon, blue = nitrogen, yellow = sulphur.

4. **XYZ coordinates of cages and catenanes**
a) **OH-cube (Type 1 conformer)**

| **atom** |  | **x** | **y** | **z** |
| --- | --- | --- | --- | --- |
| C |  | -9.8227 | 8.4829 | 8.3377 |
| C |  | -9.2642 | 7.9566 | 9.5200 |
| C |  | -8.6466 | 9.0363 | 10.3918 |
| C |  | -8.9005 | 10.3957 | 9.5916 |
| C |  | -7.1387 | 8.8887 | 10.4343 |
| C |  | -6.4824 | 9.9275 | 9.7439 |
| C |  | -7.4593 | 10.9356 | 9.1638 |
| C |  | -7.4563 | 10.8703 | 7.6498 |
| C |  | -8.6544 | 10.3346 | 7.1361 |
| C |  | -9.6412 | 9.9840 | 8.2366 |
| C |  | -6.4073 | 7.8738 | 11.0452 |
| C |  | -5.0110 | 7.8817 | 10.9529 |
| C |  | -4.3594 | 8.9126 | 10.2520 |
| C |  | -5.0970 | 9.9532 | 9.6459 |
| C |  | -6.4303 | 11.2602 | 6.7934 |
| C |  | -6.5934 | 11.1063 | 5.4128 |
| C |  | -7.7884 | 10.5668 | 4.9020 |
| C |  | -8.8344 | 10.1782 | 5.7671 |
| C |  | -10.4508 | 7.6465 | 7.4193 |
| C |  | -10.5171 | 6.2717 | 7.6741 |
| C |  | -9.9414 | 5.7510 | 8.8450 |
| C |  | -9.3091 | 6.5934 | 9.7838 |
| C |  | -9.2389 | 8.9918 | 11.7996 |
| C |  | -7.1252 | 12.3459 | 9.6490 |
| C |  | -9.6872 | 11.4105 | 10.4014 |
| C |  | -10.9811 | 10.6787 | 7.9950 |
| N |  | -10.0956 | 4.3505 | 9.0986 |
| N |  | -7.8302 | 10.3732 | 3.4880 |
| N |  | -2.9429 | 8.7918 | 10.1147 |
| C |  | -8.9096 | 10.4866 | 2.7746 |
| C |  | -8.8426 | 10.2541 | 1.3206 |
| C |  | -2.1356 | 9.8094 | 10.1100 |
| C |  | -0.6924 | 9.5935 | 9.9055 |
| C |  | -9.0810 | 3.5499 | 9.2101 |
| C |  | -9.2988 | 2.1137 | 9.4632 |
| C |  | -10.5759 | 1.5251 | 9.6486 |
| C |  | -10.7161 | 0.1441 | 9.8698 |
| C |  | -9.5935 | -0.6924 | 9.9055 |
| C |  | -8.3164 | -0.1035 | 9.7248 |
| C |  | -8.1764 | 1.2799 | 9.5091 |
| C |  | -0.1035 | 8.3164 | 9.7248 |
| C |  | 1.2799 | 8.1764 | 9.5091 |
| C |  | 2.1137 | 9.2988 | 9.4632 |
| C |  | 1.5251 | 10.5759 | 9.6486 |
| C |  | 0.1441 | 10.7161 | 9.8698 |
| C |  | -7.6465 | 9.9304 | 0.6303 |
| C |  | -7.6405 | 9.7339 | -0.7627 |
| C |  | -8.8205 | 9.8462 | -1.5061 |
| C |  | -10.0185 | 10.1567 | -0.8139 |
| C |  | -10.0241 | 10.3616 | 0.5770 |
| C |  | -8.7372 | 9.6369 | -2.9629 |
| C |  | -9.8094 | -2.1356 | 10.1100 |
| C |  | 3.5499 | 9.0810 | 9.2101 |
| H |  | -7.1682 | 1.6767 | 9.3721 |
| H |  | -10.9742 | 10.6042 | 1.0555 |
| H |  | -0.2534 | 11.7230 | 10.0064 |
| H |  | -6.6896 | 9.4891 | -1.2404 |
| H |  | 1.6767 | 7.1682 | 9.3721 |
| H |  | -11.7230 | -0.2534 | 10.0064 |
| C |  | -7.7240 | 8.3624 | -10.4323 |
| C |  | -8.9493 | 7.9637 | -9.8605 |
| C |  | -9.7717 | 9.1491 | -9.3850 |
| C |  | -8.8886 | 10.4282 | -9.7563 |
| C |  | -9.8619 | 9.1682 | -7.8723 |
| C |  | -9.1311 | 10.2341 | -7.3091 |
| C |  | -8.4680 | 11.0973 | -8.3682 |
| C |  | -6.9607 | 10.9505 | -8.3092 |
| C |  | -6.4484 | 10.2597 | -9.4261 |
| C |  | -7.5409 | 9.8663 | -10.4053 |
| C |  | -10.5581 | 8.2808 | -7.0569 |
| C |  | -10.5187 | 8.4467 | -5.6671 |
| C |  | -9.7705 | 9.4974 | -5.1114 |
| C |  | -9.0695 | 10.4065 | -5.9323 |
| C |  | -6.1089 | 11.4039 | -7.3059 |
| C |  | -4.7353 | 11.1574 | -7.4075 |
| C |  | -4.2275 | 10.4622 | -8.5198 |
| C |  | -5.0871 | 10.0096 | -9.5446 |
| C |  | -6.8405 | 7.4158 | -10.9427 |
| C |  | -7.1760 | 6.0581 | -10.8813 |
| C |  | -8.3927 | 5.6668 | -10.2986 |
| C |  | -9.2937 | 6.6202 | -9.7793 |
| C |  | -11.1645 | 9.1192 | -10.0131 |
| C |  | -8.8804 | 12.5593 | -8.2015 |
| C |  | -9.6185 | 11.3945 | -10.6718 |
| C |  | -7.2241 | 10.4004 | -11.8021 |
| N |  | -2.8280 | 10.1760 | -8.5031 |
| N |  | -9.8105 | 9.6743 | -3.6910 |
| N |  | -8.7414 | 4.2788 | -10.3372 |
| C |  | -10.8703 | -7.4563 | 7.6498 |
| C |  | -10.3346 | -8.6544 | 7.1361 |
| C |  | -9.9840 | -9.6412 | 8.2366 |
| C |  | -10.3957 | -8.9005 | 9.5916 |
| C |  | -8.4829 | -9.8227 | 8.3377 |
| C |  | -7.9566 | -9.2642 | 9.5200 |
| C |  | -9.0363 | -8.6466 | 10.3918 |
| C |  | -8.8887 | -7.1387 | 10.4343 |
| C |  | -9.9275 | -6.4824 | 9.7439 |
| C |  | -10.9356 | -7.4593 | 9.1638 |
| C |  | -7.6465 | -10.4508 | 7.4193 |
| C |  | -6.2717 | -10.5171 | 7.6741 |
| C |  | -5.7510 | -9.9414 | 8.8450 |
| C |  | -6.5934 | -9.3091 | 9.7838 |
| C |  | -7.8738 | -6.4073 | 11.0452 |
| C |  | -7.8817 | -5.0110 | 10.9529 |
| C |  | -8.9126 | -4.3594 | 10.2520 |
| C |  | -9.9532 | -5.0970 | 9.6459 |
| C |  | -11.2602 | -6.4303 | 6.7934 |
| C |  | -11.1063 | -6.5934 | 5.4128 |
| C |  | -10.5668 | -7.7884 | 4.9020 |
| C |  | -10.1782 | -8.8344 | 5.7671 |
| C |  | -10.6787 | -10.9811 | 7.9950 |
| C |  | -8.9918 | -9.2389 | 11.7996 |
| C |  | -11.4105 | -9.6872 | 10.4014 |
| C |  | -12.3459 | -7.1252 | 9.6490 |
| N |  | -8.7918 | -2.9429 | 10.1147 |
| N |  | -4.3505 | -10.0956 | 9.0986 |
| N |  | -10.3732 | -7.8302 | 3.4880 |
| C |  | 8.4829 | 9.8227 | 8.3377 |
| C |  | 7.9566 | 9.2642 | 9.5200 |
| C |  | 9.0363 | 8.6466 | 10.3918 |
| C |  | 10.3957 | 8.9005 | 9.5916 |
| C |  | 8.8887 | 7.1387 | 10.4343 |
| C |  | 9.9275 | 6.4824 | 9.7439 |
| C |  | 10.9356 | 7.4593 | 9.1638 |
| C |  | 10.8703 | 7.4563 | 7.6498 |
| C |  | 10.3346 | 8.6544 | 7.1361 |
| C |  | 9.9840 | 9.6412 | 8.2366 |
| C |  | 7.8738 | 6.4073 | 11.0452 |
| C |  | 7.8817 | 5.0110 | 10.9529 |
| C |  | 8.9126 | 4.3594 | 10.2520 |
| C |  | 9.9532 | 5.0970 | 9.6459 |
| C |  | 11.2602 | 6.4303 | 6.7934 |
| C |  | 11.1063 | 6.5934 | 5.4128 |
| C |  | 10.5668 | 7.7884 | 4.9020 |
| C |  | 10.1782 | 8.8344 | 5.7671 |
| C |  | 7.6465 | 10.4508 | 7.4193 |
| C |  | 6.2717 | 10.5171 | 7.6741 |
| C |  | 5.7510 | 9.9414 | 8.8450 |
| C |  | 6.5934 | 9.3091 | 9.7838 |
| C |  | 8.9918 | 9.2389 | 11.7996 |
| C |  | 12.3459 | 7.1252 | 9.6490 |
| C |  | 11.4105 | 9.6872 | 10.4014 |
| C |  | 10.6787 | 10.9811 | 7.9950 |
| C |  | 10.4866 | 8.9096 | 2.7746 |
| N |  | 8.7918 | 2.9429 | 10.1147 |
| N |  | 4.3505 | 10.0956 | 9.0986 |
| C |  | 9.8094 | 2.1356 | 10.1100 |
| C |  | -2.0852 | 10.1835 | -9.5685 |
| C |  | -10.4866 | -8.9096 | 2.7746 |
| C |  | -0.6548 | 9.8450 | -9.4619 |
| C |  | -0.0083 | 9.5251 | -8.2410 |
| C |  | 1.3612 | 9.2045 | -8.2043 |
| C |  | 2.1231 | 9.1866 | -9.3775 |
| C |  | 1.4759 | 9.5040 | -10.5989 |
| C |  | 0.1093 | 9.8316 | -10.6352 |
| H |  | -0.3348 | 10.0702 | -11.6030 |
| H |  | 1.8049 | 8.9662 | -7.2355 |
| C |  | 3.5498 | 8.8285 | -9.2686 |
| C |  | -10.2541 | -8.8426 | 1.3206 |
| C |  | -9.9304 | -7.6465 | 0.6303 |
| C |  | -9.7339 | -7.6405 | -0.7627 |
| C |  | -9.8462 | -8.8205 | -1.5061 |
| C |  | -10.1567 | -10.0185 | -0.8139 |
| C |  | -10.3616 | -10.0241 | 0.5770 |
| C |  | -9.6369 | -8.7372 | -2.9629 |
| H |  | -10.6042 | -10.9742 | 1.0555 |
| H |  | -9.4891 | -6.6896 | -1.2404 |
| C |  | 9.5935 | 0.6924 | 9.9055 |
| C |  | 8.3164 | 0.1035 | 9.7248 |
| C |  | 8.1764 | -1.2799 | 9.5091 |
| C |  | 9.2988 | -2.1137 | 9.4632 |
| C |  | 10.5759 | -1.5251 | 9.6486 |
| C |  | 10.7161 | -0.1441 | 9.8698 |
| H |  | 11.7230 | 0.2534 | 10.0064 |
| C |  | 9.0810 | -3.5499 | 9.2101 |
| H |  | 7.1682 | -1.6767 | 9.3721 |
| C |  | 9.8619 | -9.1682 | -7.8723 |
| C |  | 9.1311 | -10.2341 | -7.3091 |
| C |  | 8.4680 | -11.0973 | -8.3682 |
| C |  | 8.8886 | -10.4282 | -9.7563 |
| C |  | 6.9607 | -10.9505 | -8.3092 |
| C |  | 6.4484 | -10.2597 | -9.4261 |
| C |  | 7.5409 | -9.8663 | -10.4053 |
| C |  | 7.7240 | -8.3624 | -10.4323 |
| C |  | 8.9493 | -7.9637 | -9.8605 |
| C |  | 9.7717 | -9.1491 | -9.3850 |
| C |  | 6.1089 | -11.4039 | -7.3059 |
| C |  | 4.7353 | -11.1574 | -7.4075 |
| C |  | 4.2275 | -10.4622 | -8.5198 |
| C |  | 5.0871 | -10.0096 | -9.5446 |
| C |  | 6.8405 | -7.4158 | -10.9427 |
| C |  | 7.1760 | -6.0581 | -10.8813 |
| C |  | 8.3927 | -5.6668 | -10.2986 |
| C |  | 9.2937 | -6.6202 | -9.7793 |
| C |  | 10.5581 | -8.2808 | -7.0569 |
| C |  | 10.5187 | -8.4467 | -5.6671 |
| C |  | 9.7705 | -9.4974 | -5.1114 |
| C |  | 9.0695 | -10.4065 | -5.9323 |
| C |  | 8.8804 | -12.5593 | -8.2015 |
| C |  | 7.2241 | -10.4004 | -11.8021 |
| C |  | 9.6185 | -11.3945 | -10.6718 |
| C |  | 11.1645 | -9.1192 | -10.0131 |
| N |  | 9.8105 | -9.6743 | -3.6910 |
| N |  | 8.7414 | -4.2788 | -10.3372 |
| N |  | 2.8280 | -10.1760 | -8.5031 |
| C |  | 8.8285 | -3.5498 | -9.2686 |
| C |  | 9.1866 | -2.1231 | -9.3775 |
| C |  | 2.0852 | -10.1835 | -9.5685 |
| C |  | 0.6548 | -9.8450 | -9.4619 |
| C |  | 8.7372 | -9.6369 | -2.9629 |
| C |  | 8.8205 | -9.8462 | -1.5061 |
| C |  | 10.0185 | -10.1567 | -0.8139 |
| C |  | 10.0241 | -10.3616 | 0.5770 |
| C |  | 8.8426 | -10.2541 | 1.3206 |
| C |  | 7.6465 | -9.9304 | 0.6303 |
| C |  | 7.6405 | -9.7339 | -0.7627 |
| C |  | 0.0083 | -9.5251 | -8.2410 |
| C |  | -1.3612 | -9.2045 | -8.2043 |
| C |  | -2.1231 | -9.1866 | -9.3775 |
| C |  | -1.4759 | -9.5040 | -10.5989 |
| C |  | -0.1093 | -9.8316 | -10.6352 |
| C |  | 9.5040 | -1.4759 | -10.5989 |
| C |  | 9.8316 | -0.1093 | -10.6352 |
| C |  | 9.8450 | 0.6548 | -9.4619 |
| C |  | 9.5251 | 0.0083 | -8.2410 |
| C |  | 9.2045 | -1.3612 | -8.2043 |
| C |  | 10.1835 | 2.0852 | -9.5685 |
| C |  | 8.9096 | -10.4866 | 2.7746 |
| C |  | -3.5498 | -8.8285 | -9.2686 |
| H |  | 6.6896 | -9.4891 | -1.2404 |
| H |  | 8.9662 | -1.8049 | -7.2355 |
| H |  | 0.3348 | -10.0702 | -11.6030 |
| H |  | 10.0702 | 0.3348 | -11.6030 |
| H |  | -1.8049 | -8.9662 | -7.2355 |
| H |  | 10.9742 | -10.6042 | 1.0555 |
| C |  | 9.1682 | 9.8619 | -7.8723 |
| C |  | 10.2341 | 9.1311 | -7.3091 |
| C |  | 11.0973 | 8.4680 | -8.3682 |
| C |  | 10.4282 | 8.8886 | -9.7563 |
| C |  | 10.9505 | 6.9607 | -8.3092 |
| C |  | 10.2597 | 6.4484 | -9.4261 |
| C |  | 9.8663 | 7.5409 | -10.4053 |
| C |  | 8.3624 | 7.7240 | -10.4323 |
| C |  | 7.9637 | 8.9493 | -9.8605 |
| C |  | 9.1491 | 9.7717 | -9.3850 |
| C |  | 11.4039 | 6.1089 | -7.3059 |
| C |  | 11.1574 | 4.7353 | -7.4075 |
| C |  | 10.4622 | 4.2275 | -8.5198 |
| C |  | 10.0096 | 5.0871 | -9.5446 |
| C |  | 7.4158 | 6.8405 | -10.9427 |
| C |  | 6.0581 | 7.1760 | -10.8813 |
| C |  | 5.6668 | 8.3927 | -10.2986 |
| C |  | 6.6202 | 9.2937 | -9.7793 |
| C |  | 8.2808 | 10.5581 | -7.0569 |
| C |  | 8.4467 | 10.5187 | -5.6671 |
| C |  | 9.4974 | 9.7705 | -5.1114 |
| C |  | 10.4065 | 9.0695 | -5.9323 |
| C |  | 12.5593 | 8.8804 | -8.2015 |
| C |  | 10.4004 | 7.2241 | -11.8021 |
| C |  | 11.3945 | 9.6185 | -10.6718 |
| C |  | 9.1192 | 11.1645 | -10.0131 |
| N |  | 4.2788 | 8.7414 | -10.3372 |
| N |  | 10.1760 | 2.8280 | -8.5031 |
| N |  | 9.6743 | 9.8105 | -3.6910 |
| C |  | 9.8227 | -8.4829 | 8.3377 |
| C |  | 9.2642 | -7.9566 | 9.5200 |
| C |  | 8.6466 | -9.0363 | 10.3918 |
| C |  | 8.9005 | -10.3957 | 9.5916 |
| C |  | 7.1387 | -8.8887 | 10.4343 |
| C |  | 6.4824 | -9.9275 | 9.7439 |
| C |  | 7.4593 | -10.9356 | 9.1638 |
| C |  | 7.4563 | -10.8703 | 7.6498 |
| C |  | 8.6544 | -10.3346 | 7.1361 |
| C |  | 9.6412 | -9.9840 | 8.2366 |
| C |  | 6.4073 | -7.8738 | 11.0452 |
| C |  | 5.0110 | -7.8817 | 10.9529 |
| C |  | 4.3594 | -8.9126 | 10.2520 |
| C |  | 5.0970 | -9.9532 | 9.6459 |
| C |  | 6.4303 | -11.2602 | 6.7934 |
| C |  | 6.5934 | -11.1063 | 5.4128 |
| C |  | 7.7884 | -10.5668 | 4.9020 |
| C |  | 8.8344 | -10.1782 | 5.7671 |
| C |  | 10.4508 | -7.6465 | 7.4193 |
| C |  | 10.5171 | -6.2717 | 7.6741 |
| C |  | 9.9414 | -5.7510 | 8.8450 |
| C |  | 9.3091 | -6.5934 | 9.7838 |
| C |  | 9.2389 | -8.9918 | 11.7996 |
| C |  | 7.1252 | -12.3459 | 9.6490 |
| C |  | 9.6872 | -11.4105 | 10.4014 |
| C |  | 10.9811 | -10.6787 | 7.9950 |
| N |  | 7.8302 | -10.3732 | 3.4880 |
| N |  | 2.9429 | -8.7918 | 10.1147 |
| N |  | 10.0956 | -4.3505 | 9.0986 |
| C |  | -8.3624 | -7.7240 | -10.4323 |
| C |  | -7.9637 | -8.9493 | -9.8605 |
| C |  | -9.1491 | -9.7717 | -9.3850 |
| C |  | -10.4282 | -8.8886 | -9.7563 |
| C |  | -9.1682 | -9.8619 | -7.8723 |
| C |  | -10.2341 | -9.1311 | -7.3091 |
| C |  | -11.0973 | -8.4680 | -8.3682 |
| C |  | -10.9505 | -6.9607 | -8.3092 |
| C |  | -10.2597 | -6.4484 | -9.4261 |
| C |  | -9.8663 | -7.5409 | -10.4053 |
| C |  | -8.2808 | -10.5581 | -7.0569 |
| C |  | -8.4467 | -10.5187 | -5.6671 |
| C |  | -9.4974 | -9.7705 | -5.1114 |
| C |  | -10.4065 | -9.0695 | -5.9323 |
| C |  | -11.4039 | -6.1089 | -7.3059 |
| C |  | -11.1574 | -4.7353 | -7.4075 |
| C |  | -10.4622 | -4.2275 | -8.5198 |
| C |  | -10.0096 | -5.0871 | -9.5446 |
| C |  | -7.4158 | -6.8405 | -10.9427 |
| C |  | -6.0581 | -7.1760 | -10.8813 |
| C |  | -5.6668 | -8.3927 | -10.2986 |
| C |  | -6.6202 | -9.2937 | -9.7793 |
| C |  | -9.1192 | -11.1645 | -10.0131 |
| C |  | -12.5593 | -8.8804 | -8.2015 |
| C |  | -11.3945 | -9.6185 | -10.6718 |
| C |  | -10.4004 | -7.2241 | -11.8021 |
| N |  | -10.1760 | -2.8280 | -8.5031 |
| N |  | -9.6743 | -9.8105 | -3.6910 |
| N |  | -4.2788 | -8.7414 | -10.3372 |
| C |  | 2.1356 | -9.8094 | 10.1100 |
| C |  | 0.6924 | -9.5935 | 9.9055 |
| C |  | 0.1035 | -8.3164 | 9.7248 |
| C |  | -1.2799 | -8.1764 | 9.5091 |
| C |  | -2.1137 | -9.2988 | 9.4632 |
| C |  | -1.5251 | -10.5759 | 9.6486 |
| C |  | -0.1441 | -10.7161 | 9.8698 |
| C |  | -3.5499 | -9.0810 | 9.2101 |
| H |  | 0.2534 | -11.7230 | 10.0064 |
| H |  | -1.6767 | -7.1682 | 9.3721 |
| C |  | 9.6369 | 8.7372 | -2.9629 |
| C |  | 9.8462 | 8.8205 | -1.5061 |
| C |  | 10.1567 | 10.0185 | -0.8139 |
| C |  | 10.3616 | 10.0241 | 0.5770 |
| C |  | 10.2541 | 8.8426 | 1.3206 |
| C |  | 9.9304 | 7.6465 | 0.6303 |
| C |  | 9.7339 | 7.6405 | -0.7627 |
| H |  | 9.4891 | 6.6896 | -1.2404 |
| N |  | 10.3732 | 7.8302 | 3.4880 |
| H |  | 10.6042 | 10.9742 | 1.0555 |
| C |  | -10.1835 | -2.0852 | -9.5685 |
| C |  | -9.8450 | -0.6548 | -9.4619 |
| C |  | -9.5251 | -0.0083 | -8.2410 |
| C |  | -9.2045 | 1.3612 | -8.2043 |
| C |  | -9.1866 | 2.1231 | -9.3775 |
| C |  | -9.5040 | 1.4759 | -10.5989 |
| C |  | -9.8316 | 0.1093 | -10.6352 |
| H |  | -10.0702 | -0.3348 | -11.6030 |
| H |  | -8.9662 | 1.8049 | -7.2355 |
| C |  | -8.8285 | 3.5498 | -9.2686 |
| H |  | -6.9105 | 7.0738 | 11.5851 |
| H |  | -4.4255 | 7.0879 | 11.4166 |
| H |  | -4.5936 | 10.7403 | 9.0916 |
| H |  | -5.5035 | 11.6737 | 7.1869 |
| H |  | -5.7949 | 11.3973 | 4.7299 |
| H |  | -9.7486 | 9.7439 | 5.3729 |
| H |  | -10.8864 | 8.0491 | 6.5068 |
| H |  | -11.0088 | 5.6054 | 6.9663 |
| H |  | -8.8794 | 6.1822 | 10.6929 |
| H |  | -10.3283 | 9.1168 | 11.7833 |
| H |  | -9.0345 | 8.0303 | 12.2864 |
| H |  | -8.8158 | 9.7741 | 12.4404 |
| H |  | -6.1234 | 12.6510 | 9.3243 |
| H |  | -7.8310 | 13.0854 | 9.2521 |
| H |  | -7.1444 | 12.4114 | 10.7432 |
| H |  | -9.8497 | 12.3395 | 9.8405 |
| H |  | -10.6755 | 11.0253 | 10.6835 |
| H |  | -9.1679 | 11.6790 | 11.3301 |
| H |  | -11.7011 | 10.4500 | 8.7900 |
| H |  | -11.4326 | 10.3533 | 7.0504 |
| H |  | -10.8682 | 11.7678 | 7.9415 |
| H |  | -9.9080 | 10.7596 | 3.1579 |
| H |  | -2.4326 | 10.8637 | 10.2497 |
| H |  | -8.0206 | 3.8488 | 9.1154 |
| O |  | -11.7589 | 2.1999 | 9.6342 |
| O |  | -7.1343 | -0.7762 | 9.7397 |
| O |  | -0.7762 | 7.1343 | 9.7397 |
| O |  | 2.1999 | 11.7589 | 9.6342 |
| O |  | -6.4235 | 9.7854 | 1.2069 |
| O |  | -11.2445 | 10.2907 | -1.3923 |
| H |  | -7.7167 | 9.4478 | -3.3449 |
| H |  | -10.8637 | -2.4326 | 10.2497 |
| H |  | 3.8488 | 8.0206 | 9.1154 |
| H |  | 11.2265 | -10.1191 | -2.3951 |
| H |  | 6.4496 | -9.9042 | 2.2213 |
| H |  | -6.4496 | 9.9042 | 2.2213 |
| H |  | -11.2265 | 10.1191 | -2.3951 |
| H |  | -10.1191 | -11.2265 | -2.3951 |
| H |  | 10.1191 | 11.2265 | -2.3951 |
| H |  | -11.1304 | 7.4614 | -7.4883 |
| H |  | -11.0635 | 7.7633 | -5.0169 |
| H |  | -8.5070 | 11.2260 | -5.4934 |
| H |  | -6.5011 | 11.9417 | -6.4446 |
| H |  | -4.0568 | 11.4988 | -6.6259 |
| H |  | -4.6952 | 9.4570 | -10.3938 |
| H |  | -5.8941 | 7.7195 | -11.3862 |
| H |  | -6.4973 | 5.3070 | -11.2831 |
| H |  | -10.2365 | 6.3064 | -9.3400 |
| H |  | -11.1143 | 9.1273 | -11.1084 |
| H |  | -11.7692 | 9.9759 | -9.6931 |
| H |  | -11.7113 | 8.2133 | -9.7241 |
| H |  | -8.5694 | 12.9514 | -7.2255 |
| H |  | -9.9685 | 12.6810 | -8.2634 |
| H |  | -8.4225 | 13.1973 | -8.9664 |
| H |  | -9.0018 | 12.2691 | -10.9139 |
| H |  | -9.8970 | 10.9209 | -11.6219 |
| H |  | -10.5433 | 11.7673 | -10.2131 |
| H |  | -8.0115 | 10.1395 | -12.5196 |
| H |  | -7.1121 | 11.4908 | -11.8023 |
| H |  | -6.2879 | 9.9783 | -12.1861 |
| H |  | -8.0491 | -10.8864 | 6.5068 |
| H |  | -5.6054 | -11.0088 | 6.9663 |
| H |  | -6.1822 | -8.8794 | 10.6929 |
| H |  | -7.0738 | -6.9105 | 11.5851 |
| H |  | -7.0879 | -4.4255 | 11.4166 |
| H |  | -10.7403 | -4.5936 | 9.0916 |
| H |  | -11.6737 | -5.5035 | 7.1869 |
| H |  | -11.3973 | -5.7949 | 4.7299 |
| H |  | -9.7439 | -9.7486 | 5.3729 |
| H |  | -10.4500 | -11.7011 | 8.7900 |
| H |  | -10.3533 | -11.4326 | 7.0504 |
| H |  | -11.7678 | -10.8682 | 7.9415 |
| H |  | -8.0303 | -9.0345 | 12.2864 |
| H |  | -9.7741 | -8.8158 | 12.4404 |
| H |  | -9.1168 | -10.3283 | 11.7833 |
| H |  | -12.3395 | -9.8497 | 9.8405 |
| H |  | -11.0253 | -10.6755 | 10.6835 |
| H |  | -11.6790 | -9.1679 | 11.3301 |
| H |  | -12.4114 | -7.1444 | 10.7432 |
| H |  | -13.0854 | -7.8310 | 9.2521 |
| H |  | -12.6510 | -6.1234 | 9.3243 |
| H |  | 7.0738 | 6.9105 | 11.5851 |
| H |  | 7.0879 | 4.4255 | 11.4166 |
| H |  | 10.7403 | 4.5936 | 9.0916 |
| H |  | 11.6737 | 5.5035 | 7.1869 |
| H |  | 11.3973 | 5.7949 | 4.7299 |
| H |  | 9.7439 | 9.7486 | 5.3729 |
| H |  | 8.0491 | 10.8864 | 6.5068 |
| H |  | 5.6054 | 11.0088 | 6.9663 |
| H |  | 6.1822 | 8.8794 | 10.6929 |
| H |  | 9.1168 | 10.3283 | 11.7833 |
| H |  | 8.0303 | 9.0345 | 12.2864 |
| H |  | 9.7741 | 8.8158 | 12.4404 |
| H |  | 12.6510 | 6.1234 | 9.3243 |
| H |  | 12.4114 | 7.1444 | 10.7432 |
| H |  | 13.0854 | 7.8310 | 9.2521 |
| H |  | 12.3395 | 9.8497 | 9.8405 |
| H |  | 11.0253 | 10.6755 | 10.6835 |
| H |  | 11.6790 | 9.1679 | 11.3301 |
| H |  | 10.4500 | 11.7011 | 8.7900 |
| H |  | 11.7678 | 10.8682 | 7.9415 |
| H |  | 10.3533 | 11.4326 | 7.0504 |
| H |  | 10.8637 | 2.4326 | 10.2497 |
| H |  | -2.4290 | 10.4333 | -10.5877 |
| H |  | -10.7596 | -9.9080 | 3.1579 |
| O |  | -0.6093 | 9.4953 | -7.0209 |
| O |  | 2.0763 | 9.5283 | -11.8210 |
| H |  | 9.9042 | 6.4496 | 2.2213 |
| H |  | -9.9042 | -6.4496 | 2.2213 |
| H |  | 10.7596 | 9.9080 | 3.1579 |
| O |  | -9.7854 | -6.4235 | 1.2069 |
| O |  | -10.2907 | -11.2445 | -1.3923 |
| H |  | -9.4478 | -7.7167 | -3.3449 |
| H |  | 7.2558 | 1.7829 | 9.8551 |
| H |  | 11.6428 | -3.1926 | 9.4402 |
| O |  | 7.1343 | 0.7762 | 9.7397 |
| O |  | 11.7589 | -2.1999 | 9.6342 |
| H |  | -9.6996 | -1.6080 | -7.0695 |
| H |  | 8.0206 | -3.8488 | 9.1154 |
| H |  | -9.2615 | 3.0581 | -11.7825 |
| H |  | 6.5011 | -11.9417 | -6.4446 |
| H |  | 4.0568 | -11.4988 | -6.6259 |
| H |  | 4.6952 | -9.4570 | -10.3938 |
| H |  | 5.8941 | -7.7195 | -11.3862 |
| H |  | 6.4973 | -5.3070 | -11.2831 |
| H |  | 10.2365 | -6.3064 | -9.3400 |
| H |  | 11.1304 | -7.4614 | -7.4883 |
| H |  | 11.0635 | -7.7633 | -5.0169 |
| H |  | 8.5070 | -11.2260 | -5.4934 |
| H |  | 8.4225 | -13.1973 | -8.9664 |
| H |  | 9.9685 | -12.6810 | -8.2634 |
| H |  | 8.5694 | -12.9514 | -7.2255 |
| H |  | 6.2879 | -9.9783 | -12.1861 |
| H |  | 8.0115 | -10.1395 | -12.5196 |
| H |  | 7.1121 | -11.4908 | -11.8023 |
| H |  | 10.5433 | -11.7673 | -10.2131 |
| H |  | 9.8970 | -10.9209 | -11.6219 |
| H |  | 9.0018 | -12.2691 | -10.9139 |
| H |  | 11.1143 | -9.1273 | -11.1084 |
| H |  | 11.7113 | -8.2133 | -9.7241 |
| H |  | 11.7692 | -9.9759 | -9.6931 |
| H |  | 8.6414 | -3.9040 | -8.2382 |
| H |  | 2.4290 | -10.4333 | -10.5877 |
| H |  | 7.7167 | -9.4478 | -3.3449 |
| O |  | 11.2445 | -10.2907 | -1.3923 |
| O |  | 6.4235 | -9.7854 | 1.2069 |
| O |  | 0.6093 | -9.4953 | -7.0209 |
| O |  | -2.0763 | -9.5283 | -11.8210 |
| O |  | 9.5283 | -2.0763 | -11.8210 |
| O |  | 9.4953 | 0.6093 | -7.0209 |
| H |  | 10.4333 | 2.4290 | -10.5877 |
| H |  | 9.9080 | -10.7596 | 3.1579 |
| H |  | -3.9040 | -8.6414 | -8.2382 |
| H |  | 9.2615 | -3.0581 | -11.7825 |
| H |  | 9.6996 | 1.6080 | -7.0695 |
| H |  | -11.6428 | 3.1926 | 9.4402 |
| H |  | -7.2558 | -1.7829 | 9.8551 |
| H |  | 1.6080 | -9.6996 | -7.0695 |
| H |  | -3.0581 | -9.2615 | -11.7825 |
| H |  | 11.9417 | 6.5011 | -6.4446 |
| H |  | 11.4988 | 4.0568 | -6.6259 |
| H |  | 9.4570 | 4.6952 | -10.3938 |
| H |  | 7.7195 | 5.8941 | -11.3862 |
| H |  | 5.3070 | 6.4973 | -11.2831 |
| H |  | 6.3064 | 10.2365 | -9.3400 |
| H |  | 7.4614 | 11.1304 | -7.4883 |
| H |  | 7.7633 | 11.0635 | -5.0169 |
| H |  | 11.2260 | 8.5070 | -5.4934 |
| H |  | 12.6810 | 9.9685 | -8.2634 |
| H |  | 12.9514 | 8.5694 | -7.2255 |
| H |  | 13.1973 | 8.4225 | -8.9664 |
| H |  | 9.9783 | 6.2879 | -12.1861 |
| H |  | 11.4908 | 7.1121 | -11.8023 |
| H |  | 10.1395 | 8.0115 | -12.5196 |
| H |  | 10.9209 | 9.8970 | -11.6219 |
| H |  | 12.2691 | 9.0018 | -10.9139 |
| H |  | 11.7673 | 10.5433 | -10.2131 |
| H |  | 9.1273 | 11.1143 | -11.1084 |
| H |  | 9.9759 | 11.7692 | -9.6931 |
| H |  | 8.2133 | 11.7113 | -9.7241 |
| H |  | 3.9040 | 8.6414 | -8.2382 |
| H |  | 6.9105 | -7.0738 | 11.5851 |
| H |  | 4.4255 | -7.0879 | 11.4166 |
| H |  | 4.5936 | -10.7403 | 9.0916 |
| H |  | 5.5035 | -11.6737 | 7.1869 |
| H |  | 5.7949 | -11.3973 | 4.7299 |
| H |  | 9.7486 | -9.7439 | 5.3729 |
| H |  | 10.8864 | -8.0491 | 6.5068 |
| H |  | 11.0088 | -5.6054 | 6.9663 |
| H |  | 8.8794 | -6.1822 | 10.6929 |
| H |  | 8.8158 | -9.7741 | 12.4404 |
| H |  | 10.3283 | -9.1168 | 11.7833 |
| H |  | 9.0345 | -8.0303 | 12.2864 |
| H |  | 6.1234 | -12.6510 | 9.3243 |
| H |  | 7.8310 | -13.0854 | 9.2521 |
| H |  | 7.1444 | -12.4114 | 10.7432 |
| H |  | 10.6755 | -11.0253 | 10.6835 |
| H |  | 9.1679 | -11.6790 | 11.3301 |
| H |  | 9.8497 | -12.3395 | 9.8405 |
| H |  | 10.8682 | -11.7678 | 7.9415 |
| H |  | 11.4326 | -10.3533 | 7.0504 |
| H |  | 11.7011 | -10.4500 | 8.7900 |
| H |  | -7.4614 | -11.1304 | -7.4883 |
| H |  | -7.7633 | -11.0635 | -5.0169 |
| H |  | -11.2260 | -8.5070 | -5.4934 |
| H |  | -11.9417 | -6.5011 | -6.4446 |
| H |  | -11.4988 | -4.0568 | -6.6259 |
| H |  | -9.4570 | -4.6952 | -10.3938 |
| H |  | -7.7195 | -5.8941 | -11.3862 |
| H |  | -5.3070 | -6.4973 | -11.2831 |
| H |  | -6.3064 | -10.2365 | -9.3400 |
| H |  | -9.1273 | -11.1143 | -11.1084 |
| H |  | -8.2133 | -11.7113 | -9.7241 |
| H |  | -9.9759 | -11.7692 | -9.6931 |
| H |  | -12.9514 | -8.5694 | -7.2255 |
| H |  | -12.6810 | -9.9685 | -8.2634 |
| H |  | -13.1973 | -8.4225 | -8.9664 |
| H |  | -12.2691 | -9.0018 | -10.9139 |
| H |  | -10.9209 | -9.8970 | -11.6219 |
| H |  | -11.7673 | -10.5433 | -10.2131 |
| H |  | -11.4908 | -7.1121 | -11.8023 |
| H |  | -10.1395 | -8.0115 | -12.5196 |
| H |  | -9.9783 | -6.2879 | -12.1861 |
| H |  | 2.4326 | -10.8637 | 10.2497 |
| O |  | 0.7762 | -7.1343 | 9.7397 |
| O |  | -2.1999 | -11.7589 | 9.6342 |
| H |  | -3.8488 | -8.0206 | 9.1154 |
| H |  | -1.7829 | 7.2558 | 9.8551 |
| H |  | 3.1926 | 11.6428 | 9.4402 |
| H |  | 9.4478 | 7.7167 | -3.3449 |
| O |  | 10.2907 | 11.2445 | -1.3923 |
| O |  | 9.7854 | 6.4235 | 1.2069 |
| H |  | 1.7829 | -7.2558 | 9.8551 |
| H |  | -3.1926 | -11.6428 | 9.4402 |
| H |  | -10.4333 | -2.4290 | -10.5877 |
| O |  | -9.4953 | -0.6093 | -7.0209 |
| O |  | -9.5283 | 2.0763 | -11.8210 |
| H |  | -1.6080 | 9.6996 | -7.0695 |
| H |  | 3.0581 | 9.2615 | -11.7825 |
| H |  | -8.6414 | 3.9040 | -8.2382 |
|  |  |  |  |  |

b) **OH-cube (Type 2 conformer)**

| **atom** | **x** |  | **y** | **z** |
| --- | --- | --- | --- | --- |
| C | 10.3895 |  | -8.5183 | -8.0214 |
| C | 9.6554 |  | -9.6580 | -7.6307 |
| C | 9.0766 |  | -10.4006 | -8.8220 |
| C | 9.5682 |  | -9.5703 | -10.0956 |
| C | 7.5654 |  | -10.2866 | -8.8464 |
| C | 7.1083 |  | -9.4923 | -9.9193 |
| C | 8.2518 |  | -8.9643 | -10.7678 |
| C | 8.3889 |  | -7.4637 | -10.6027 |
| C | 9.5643 |  | -7.1096 | -9.9074 |
| C | 10.3902 |  | -8.3252 | -9.5248 |
| C | 6.6626 |  | -10.8597 | -7.9573 |
| C | 5.2896 |  | -10.6480 | -8.1396 |
| C | 4.8404 |  | -9.8517 | -9.2058 |
| C | 5.7527 |  | -9.2630 | -10.1091 |
| C | 7.5057 |  | -6.4834 | -11.0422 |
| C | 7.7931 |  | -5.1357 | -10.7888 |
| C | 8.9573 |  | -4.7902 | -10.0831 |
| C | 9.8595 |  | -5.7807 | -9.6369 |
| C | 11.0101 |  | -7.7167 | -7.0694 |
| C | 10.8896 |  | -8.0435 | -5.7126 |
| C | 10.1472 |  | -9.1722 | -5.3259 |
| C | 9.5278 |  | -9.9977 | -6.2910 |
| C | 9.5086 |  | -11.8667 | -8.7983 |
| C | 8.0453 |  | -9.3329 | -12.2366 |
| C | 10.3819 |  | -10.4127 | -11.0617 |
| C | 11.8171 |  | -8.1917 | -10.0558 |
| N | 10.0304 |  | -9.5935 | -3.9643 |
| N | 9.2250 |  | -3.4410 | -9.6930 |
| N | 3.4595 |  | -9.5205 | -9.3734 |
| C | 9.2676 |  | -2.4550 | -10.5354 |
| C | 9.5052 |  | -1.0852 | -10.0450 |
| C | 2.5185 |  | -10.4141 | -9.3948 |
| C | 1.1131 |  | -9.9944 | -9.5412 |
| C | 9.8874 |  | -8.7542 | -2.9828 |
| C | 9.7849 |  | -9.2513 | -1.5991 |
| C | 9.6983 |  | -8.3096 | -0.5682 |
| C | 9.5872 |  | -8.7201 | 0.7730 |
| C | 9.5405 |  | -10.0941 | 1.1185 |
| C | 9.6340 |  | -11.0370 | 0.0871 |
| C | 9.7624 |  | -10.6265 | -1.2508 |
| C | 0.1354 |  | -10.9916 | -9.6455 |
| C | -1.2229 |  | -10.6543 | -9.7744 |
| C | -1.6460 |  | -9.3004 | -9.7895 |
| C | -0.6687 |  | -8.3040 | -9.6907 |
| C | 0.6926 |  | -8.6410 | -9.5762 |
| C | 9.6481 |  | -0.0614 | -10.9899 |
| C | 9.8521 |  | 1.2680 | -10.5819 |
| C | 9.9009 |  | 1.6161 | -9.2076 |
| C | 9.7690 |  | 0.5917 | -8.2638 |
| C | 9.5845 |  | -0.7421 | -8.6718 |
| C | 10.0707 |  | 2.9937 | -8.7128 |
| C | 9.3902 |  | -10.5880 | 2.4993 |
| C | -3.0545 |  | -8.8801 | -9.8972 |
| O | 9.8531 |  | -11.6570 | -2.1368 |
| O | 9.4919 |  | -1.6206 | -7.6367 |
| O | 1.5175 |  | -7.5611 | -9.5053 |
| O | 9.9842 |  | 2.1470 | -11.6140 |
| O | -2.0499 |  | -11.7319 | -9.8740 |
| O | 9.5305 |  | -7.6864 | 1.6564 |
| C | 8.8265 |  | 10.6743 | -7.5529 |
| C | 9.9492 |  | 9.9328 | -7.1283 |
| C | 10.8296 |  | 9.5112 | -8.2918 |
| C | 10.1086 |  | 10.1121 | -9.5842 |
| C | 10.7735 |  | 8.0084 | -8.4814 |
| C | 10.0878 |  | 7.6451 | -9.6597 |
| C | 9.6129 |  | 8.8576 | -10.4408 |
| C | 8.1003 |  | 8.9517 | -10.4233 |
| C | 7.6430 |  | 10.0454 | -9.6582 |
| C | 8.7883 |  | 10.8426 | -9.0590 |
| C | 11.3035 |  | 7.0335 | -7.6432 |
| C | 11.1359 |  | 5.6818 | -7.9702 |
| C | 10.4364 |  | 5.3224 | -9.1348 |
| C | 9.9132 |  | 6.3107 | -9.9989 |
| C | 7.1965 |  | 8.1082 | -11.0604 |
| C | 5.8226 |  | 8.3480 | -10.9284 |
| C | 5.3706 |  | 9.4337 | -10.1595 |
| C | 6.2858 |  | 10.2999 | -9.5201 |
| C | 7.9047 |  | 11.1520 | -6.6277 |
| C | 8.1000 |  | 10.8911 | -5.2649 |
| C | 9.2121 |  | 10.1408 | -4.8493 |
| C | 10.1530 |  | 9.6550 | -5.7839 |
| C | 12.2675 |  | 9.9804 | -8.0739 |
| C | 10.1362 |  | 8.8057 | -11.8760 |
| C | 11.0115 |  | 11.0392 | -10.3780 |
| C | 8.6702 |  | 12.3186 | -9.4372 |
| N | 3.9873 |  | 9.7736 | -10.0369 |
| N | 10.2631 |  | 3.9661 | -9.5533 |
| N | 9.3875 |  | 9.7459 | -3.4866 |
| C | 10.7735 |  | -8.0084 | 8.4814 |
| C | 10.0878 |  | -7.6451 | 9.6597 |
| C | 9.6129 |  | -8.8576 | 10.4408 |
| C | 10.1086 |  | -10.1121 | 9.5842 |
| C | 8.1003 |  | -8.9517 | 10.4233 |
| C | 7.6430 |  | -10.0454 | 9.6582 |
| C | 8.7883 |  | -10.8426 | 9.0590 |
| C | 8.8265 |  | -10.6743 | 7.5529 |
| C | 9.9492 |  | -9.9328 | 7.1283 |
| C | 10.8296 |  | -9.5112 | 8.2918 |
| C | 7.1965 |  | -8.1082 | 11.0604 |
| C | 5.8226 |  | -8.3480 | 10.9284 |
| C | 5.3706 |  | -9.4337 | 10.1595 |
| C | 6.2858 |  | -10.2999 | 9.5201 |
| C | 7.9047 |  | -11.1520 | 6.6277 |
| C | 8.1000 |  | -10.8911 | 5.2649 |
| C | 9.2121 |  | -10.1408 | 4.8493 |
| C | 10.1530 |  | -9.6550 | 5.7839 |
| C | 11.3035 |  | -7.0335 | 7.6432 |
| C | 11.1359 |  | -5.6818 | 7.9702 |
| C | 10.4364 |  | -5.3224 | 9.1348 |
| C | 9.9132 |  | -6.3107 | 9.9989 |
| C | 10.1362 |  | -8.8057 | 11.8760 |
| C | 8.6702 |  | -12.3186 | 9.4372 |
| C | 11.0115 |  | -11.0392 | 10.3780 |
| C | 12.2675 |  | -9.9804 | 8.0739 |
| N | 9.3875 |  | -9.7459 | 3.4866 |
| N | 3.9873 |  | -9.7736 | 10.0369 |
| N | 10.2631 |  | -3.9661 | 9.5533 |
| C | -8.1003 |  | -8.9517 | -10.4233 |
| C | -7.6430 |  | -10.0454 | -9.6582 |
| C | -8.7883 |  | -10.8426 | -9.0590 |
| C | -10.1086 |  | -10.1121 | -9.5842 |
| C | -8.8265 |  | -10.6743 | -7.5529 |
| C | -9.9492 |  | -9.9328 | -7.1283 |
| C | -10.8296 |  | -9.5112 | -8.2918 |
| C | -10.7735 |  | -8.0084 | -8.4814 |
| C | -10.0878 |  | -7.6451 | -9.6597 |
| C | -9.6129 |  | -8.8576 | -10.4408 |
| C | -7.9047 |  | -11.1520 | -6.6277 |
| C | -8.1000 |  | -10.8911 | -5.2649 |
| C | -9.2121 |  | -10.1408 | -4.8493 |
| C | -10.1530 |  | -9.6550 | -5.7839 |
| C | -11.3035 |  | -7.0335 | -7.6432 |
| C | -11.1359 |  | -5.6818 | -7.9702 |
| C | -10.4364 |  | -5.3224 | -9.1348 |
| C | -9.9132 |  | -6.3107 | -9.9989 |
| C | -7.1965 |  | -8.1082 | -11.0604 |
| C | -5.8226 |  | -8.3480 | -10.9284 |
| C | -5.3706 |  | -9.4337 | -10.1595 |
| C | -6.2858 |  | -10.2999 | -9.5201 |
| C | -8.6702 |  | -12.3186 | -9.4372 |
| C | -12.2675 |  | -9.9804 | -8.0739 |
| C | -11.0115 |  | -11.0392 | -10.3780 |
| C | -10.1362 |  | -8.8057 | -11.8760 |
| C | -10.0707 |  | -2.9937 | -8.7128 |
| N | -9.3875 |  | -9.7459 | -3.4866 |
| N | -3.9873 |  | -9.7736 | -10.0369 |
| C | -9.3902 |  | -10.5880 | -2.4993 |
| C | 3.0545 |  | 8.8801 | -9.8972 |
| C | 10.0707 |  | -2.9937 | 8.7128 |
| C | 1.6460 |  | 9.3004 | -9.7895 |
| C | 0.6687 |  | 8.3040 | -9.6907 |
| C | -0.6926 |  | 8.6410 | -9.5762 |
| C | -1.1131 |  | 9.9944 | -9.5412 |
| C | -0.1354 |  | 10.9916 | -9.6455 |
| C | 1.2229 |  | 10.6543 | -9.7744 |
| O | 2.0499 |  | 11.7319 | -9.8740 |
| O | -1.5175 |  | 7.5611 | -9.5053 |
| C | -2.5185 |  | 10.4141 | -9.3948 |
| C | 9.9009 |  | -1.6161 | 9.2076 |
| C | 9.7690 |  | -0.5917 | 8.2638 |
| C | 9.5845 |  | 0.7421 | 8.6718 |
| C | 9.5052 |  | 1.0852 | 10.0450 |
| C | 9.6481 |  | 0.0614 | 10.9899 |
| C | 9.8521 |  | -1.2680 | 10.5819 |
| C | 9.2676 |  | 2.4550 | 10.5354 |
| O | 9.9842 |  | -2.1470 | 11.6140 |
| O | 9.4919 |  | 1.6206 | 7.6367 |
| C | -9.5405 |  | -10.0941 | -1.1185 |
| C | -9.6340 |  | -11.0370 | -0.0871 |
| C | -9.7624 |  | -10.6265 | 1.2508 |
| C | -9.7849 |  | -9.2513 | 1.5991 |
| C | -9.6983 |  | -8.3096 | 0.5682 |
| C | -9.5872 |  | -8.7201 | -0.7730 |
| O | -9.5305 |  | -7.6864 | -1.6564 |
| C | -9.8874 |  | -8.7542 | 2.9828 |
| O | -9.8531 |  | -11.6570 | 2.1368 |
| C | -10.7735 |  | 8.0084 | 8.4814 |
| C | -10.0878 |  | 7.6451 | 9.6597 |
| C | -9.6129 |  | 8.8576 | 10.4408 |
| C | -10.1086 |  | 10.1121 | 9.5842 |
| C | -8.1003 |  | 8.9517 | 10.4233 |
| C | -7.6430 |  | 10.0454 | 9.6582 |
| C | -8.7883 |  | 10.8426 | 9.0590 |
| C | -8.8265 |  | 10.6743 | 7.5529 |
| C | -9.9492 |  | 9.9328 | 7.1283 |
| C | -10.8296 |  | 9.5112 | 8.2918 |
| C | -7.1965 |  | 8.1082 | 11.0604 |
| C | -5.8226 |  | 8.3480 | 10.9284 |
| C | -5.3706 |  | 9.4337 | 10.1595 |
| C | -6.2858 |  | 10.2999 | 9.5201 |
| C | -7.9047 |  | 11.1520 | 6.6277 |
| C | -8.1000 |  | 10.8911 | 5.2649 |
| C | -9.2121 |  | 10.1408 | 4.8493 |
| C | -10.1530 |  | 9.6550 | 5.7839 |
| C | -11.3035 |  | 7.0335 | 7.6432 |
| C | -11.1359 |  | 5.6818 | 7.9702 |
| C | -10.4364 |  | 5.3224 | 9.1348 |
| C | -9.9132 |  | 6.3107 | 9.9989 |
| C | -10.1362 |  | 8.8057 | 11.8760 |
| C | -8.6702 |  | 12.3186 | 9.4372 |
| C | -11.0115 |  | 11.0392 | 10.3780 |
| C | -12.2675 |  | 9.9804 | 8.0739 |
| N | -10.2631 |  | 3.9661 | 9.5533 |
| N | -9.3875 |  | 9.7459 | 3.4866 |
| N | -3.9873 |  | 9.7736 | 10.0369 |
| C | -9.3902 |  | 10.5880 | 2.4993 |
| C | -9.5405 |  | 10.0941 | 1.1185 |
| C | -3.0545 |  | 8.8801 | 9.8972 |
| C | -1.6460 |  | 9.3004 | 9.7895 |
| C | -10.0707 |  | 2.9937 | 8.7128 |
| C | -9.9009 |  | 1.6161 | 9.2076 |
| C | -9.7690 |  | 0.5917 | 8.2638 |
| C | -9.5845 |  | -0.7421 | 8.6718 |
| C | -9.5052 |  | -1.0852 | 10.0450 |
| C | -9.6481 |  | -0.0614 | 10.9899 |
| C | -9.8521 |  | 1.2680 | 10.5819 |
| C | -0.6687 |  | 8.3040 | 9.6907 |
| C | 0.6926 |  | 8.6410 | 9.5762 |
| C | 1.1131 |  | 9.9944 | 9.5412 |
| C | 0.1354 |  | 10.9916 | 9.6455 |
| C | -1.2229 |  | 10.6543 | 9.7744 |
| C | -9.6340 |  | 11.0370 | 0.0871 |
| C | -9.7624 |  | 10.6265 | -1.2508 |
| C | -9.7849 |  | 9.2513 | -1.5991 |
| C | -9.6983 |  | 8.3096 | -0.5682 |
| C | -9.5872 |  | 8.7201 | 0.7730 |
| C | -9.8874 |  | 8.7542 | -2.9828 |
| C | -9.2676 |  | -2.4550 | 10.5354 |
| C | 2.5185 |  | 10.4141 | 9.3948 |
| O | -9.9842 |  | 2.1470 | 11.6140 |
| O | -9.5305 |  | 7.6864 | 1.6564 |
| O | -2.0499 |  | 11.7319 | 9.8740 |
| O | -9.8531 |  | 11.6570 | -2.1368 |
| O | 1.5175 |  | 7.5611 | 9.5053 |
| O | -9.4919 |  | -1.6206 | 7.6367 |
| C | -8.3889 |  | 7.4637 | -10.6027 |
| C | -9.5643 |  | 7.1096 | -9.9074 |
| C | -10.3902 |  | 8.3252 | -9.5248 |
| C | -9.5682 |  | 9.5703 | -10.0956 |
| C | -10.3895 |  | 8.5183 | -8.0214 |
| C | -9.6554 |  | 9.6580 | -7.6307 |
| C | -9.0766 |  | 10.4006 | -8.8220 |
| C | -7.5654 |  | 10.2866 | -8.8464 |
| C | -7.1083 |  | 9.4923 | -9.9193 |
| C | -8.2518 |  | 8.9643 | -10.7678 |
| C | -11.0101 |  | 7.7167 | -7.0694 |
| C | -10.8896 |  | 8.0435 | -5.7126 |
| C | -10.1472 |  | 9.1722 | -5.3259 |
| C | -9.5278 |  | 9.9977 | -6.2910 |
| C | -6.6626 |  | 10.8597 | -7.9573 |
| C | -5.2896 |  | 10.6480 | -8.1396 |
| C | -4.8404 |  | 9.8517 | -9.2058 |
| C | -5.7527 |  | 9.2630 | -10.1091 |
| C | -7.5057 |  | 6.4834 | -11.0422 |
| C | -7.7931 |  | 5.1357 | -10.7888 |
| C | -8.9573 |  | 4.7902 | -10.0831 |
| C | -9.8595 |  | 5.7807 | -9.6369 |
| C | -11.8171 |  | 8.1917 | -10.0558 |
| C | -9.5086 |  | 11.8667 | -8.7983 |
| C | -10.3819 |  | 10.4127 | -11.0617 |
| C | -8.0453 |  | 9.3329 | -12.2366 |
| N | -3.4595 |  | 9.5205 | -9.3734 |
| N | -10.0304 |  | 9.5935 | -3.9643 |
| N | -9.2250 |  | 3.4410 | -9.6930 |
| C | -10.3895 |  | -8.5183 | 8.0214 |
| C | -9.6554 |  | -9.6580 | 7.6307 |
| C | -9.0766 |  | -10.4006 | 8.8220 |
| C | -9.5682 |  | -9.5703 | 10.0956 |
| C | -7.5654 |  | -10.2866 | 8.8464 |
| C | -7.1083 |  | -9.4923 | 9.9193 |
| C | -8.2518 |  | -8.9643 | 10.7678 |
| C | -8.3889 |  | -7.4637 | 10.6027 |
| C | -9.5643 |  | -7.1096 | 9.9074 |
| C | -10.3902 |  | -8.3252 | 9.5248 |
| C | -6.6626 |  | -10.8597 | 7.9573 |
| C | -5.2896 |  | -10.6480 | 8.1396 |
| C | -4.8404 |  | -9.8517 | 9.2058 |
| C | -5.7527 |  | -9.2630 | 10.1091 |
| C | -7.5057 |  | -6.4834 | 11.0422 |
| C | -7.7931 |  | -5.1357 | 10.7888 |
| C | -8.9573 |  | -4.7902 | 10.0831 |
| C | -9.8595 |  | -5.7807 | 9.6369 |
| C | -11.0101 |  | -7.7167 | 7.0694 |
| C | -10.8896 |  | -8.0435 | 5.7126 |
| C | -10.1472 |  | -9.1722 | 5.3259 |
| C | -9.5278 |  | -9.9977 | 6.2910 |
| C | -9.5086 |  | -11.8667 | 8.7983 |
| C | -8.0453 |  | -9.3329 | 12.2366 |
| C | -10.3819 |  | -10.4127 | 11.0617 |
| C | -11.8171 |  | -8.1917 | 10.0558 |
| N | -9.2250 |  | -3.4410 | 9.6930 |
| N | -3.4595 |  | -9.5205 | 9.3734 |
| N | -10.0304 |  | -9.5935 | 3.9643 |
| C | 7.5654 |  | 10.2866 | 8.8464 |
| C | 7.1083 |  | 9.4923 | 9.9193 |
| C | 8.2518 |  | 8.9643 | 10.7678 |
| C | 9.5682 |  | 9.5703 | 10.0956 |
| C | 8.3889 |  | 7.4637 | 10.6027 |
| C | 9.5643 |  | 7.1096 | 9.9074 |
| C | 10.3902 |  | 8.3252 | 9.5248 |
| C | 10.3895 |  | 8.5183 | 8.0214 |
| C | 9.6554 |  | 9.6580 | 7.6307 |
| C | 9.0766 |  | 10.4006 | 8.8220 |
| C | 7.5057 |  | 6.4834 | 11.0422 |
| C | 7.7931 |  | 5.1357 | 10.7888 |
| C | 8.9573 |  | 4.7902 | 10.0831 |
| C | 9.8595 |  | 5.7807 | 9.6369 |
| C | 11.0101 |  | 7.7167 | 7.0694 |
| C | 10.8896 |  | 8.0435 | 5.7126 |
| C | 10.1472 |  | 9.1722 | 5.3259 |
| C | 9.5278 |  | 9.9977 | 6.2910 |
| C | 6.6626 |  | 10.8597 | 7.9573 |
| C | 5.2896 |  | 10.6480 | 8.1396 |
| C | 4.8404 |  | 9.8517 | 9.2058 |
| C | 5.7527 |  | 9.2630 | 10.1091 |
| C | 8.0453 |  | 9.3329 | 12.2366 |
| C | 11.8171 |  | 8.1917 | 10.0558 |
| C | 10.3819 |  | 10.4127 | 11.0617 |
| C | 9.5086 |  | 11.8667 | 8.7983 |
| N | 10.0304 |  | 9.5935 | 3.9643 |
| N | 9.2250 |  | 3.4410 | 9.6930 |
| N | 3.4595 |  | 9.5205 | 9.3734 |
| C | -2.5185 |  | -10.4141 | 9.3948 |
| C | -1.1131 |  | -9.9944 | 9.5412 |
| C | -0.1354 |  | -10.9916 | 9.6455 |
| C | 1.2229 |  | -10.6543 | 9.7744 |
| C | 1.6460 |  | -9.3004 | 9.7895 |
| C | 0.6687 |  | -8.3040 | 9.6907 |
| C | -0.6926 |  | -8.6410 | 9.5762 |
| C | 3.0545 |  | -8.8801 | 9.8972 |
| O | -1.5175 |  | -7.5611 | 9.5053 |
| O | 2.0499 |  | -11.7319 | 9.8740 |
| C | -9.2676 |  | 2.4550 | -10.5354 |
| C | -9.5052 |  | 1.0852 | -10.0450 |
| C | -9.6481 |  | 0.0614 | -10.9899 |
| C | -9.8521 |  | -1.2680 | -10.5819 |
| C | -9.9009 |  | -1.6161 | -9.2076 |
| C | -9.7690 |  | -0.5917 | -8.2638 |
| C | -9.5845 |  | 0.7421 | -8.6718 |
| O | -9.4919 |  | 1.6206 | -7.6367 |
| N | -10.2631 |  | -3.9661 | -9.5533 |
| O | -9.9842 |  | -2.1470 | -11.6140 |
| C | 9.8874 |  | 8.7542 | 2.9828 |
| C | 9.7849 |  | 9.2513 | 1.5991 |
| C | 9.6983 |  | 8.3096 | 0.5682 |
| C | 9.5872 |  | 8.7201 | -0.7730 |
| C | 9.5405 |  | 10.0941 | -1.1185 |
| C | 9.6340 |  | 11.0370 | -0.0871 |
| C | 9.7624 |  | 10.6265 | 1.2508 |
| O | 9.8531 |  | 11.6570 | 2.1368 |
| O | 9.5305 |  | 7.6864 | -1.6564 |
| C | 9.3902 |  | 10.5880 | -2.4993 |
| H | 7.0110 |  | -11.4657 | -7.1225 |
| H | 4.5791 |  | -11.0817 | -7.4392 |
| H | 5.3892 |  | -8.6415 | -10.9246 |
| H | 6.5940 |  | -6.7519 | -11.5734 |
| H | 7.0990 |  | -4.3645 | -11.1161 |
| H | 10.7545 |  | -5.4969 | -9.0876 |
| H | 11.5813 |  | -6.8388 | -7.3666 |
| H | 11.3835 |  | -7.4251 | -4.9663 |
| H | 8.9622 |  | -10.8728 | -5.9774 |
| H | 10.6006 |  | -11.9659 | -8.8006 |
| H | 9.1427 |  | -12.3737 | -7.8970 |
| H | 9.1147 |  | -12.4171 | -9.6605 |
| H | 7.1233 |  | -8.8887 | -12.6312 |
| H | 8.8702 |  | -8.9719 | -12.8619 |
| H | 7.9639 |  | -10.4175 | -12.3734 |
| H | 10.7100 |  | -9.8297 | -11.9315 |
| H | 11.2833 |  | -10.8186 | -10.5851 |
| H | 9.8047 |  | -11.2650 | -11.4418 |
| H | 12.4269 |  | -9.0647 | -9.7951 |
| H | 12.3172 |  | -7.3116 | -9.6332 |
| H | 11.8334 |  | -8.0783 | -11.1460 |
| H | 9.1419 |  | -2.5432 | -11.6293 |
| H | 2.6724 |  | -11.5053 | -9.3178 |
| H | 9.8216 |  | -7.6557 | -3.0858 |
| H | 9.7147 |  | -7.2378 | -0.7757 |
| H | 9.6118 |  | -12.1079 | 0.2955 |
| H | 0.4022 |  | -12.0496 | -9.6313 |
| H | -0.9346 |  | -7.2452 | -9.6999 |
| H | 9.6064 |  | -0.2692 | -12.0603 |
| H | 9.8053 |  | 0.7987 | -7.1925 |
| H | 10.0068 |  | 3.1013 | -7.6146 |
| H | 9.2926 |  | -11.6842 | 2.5942 |
| H | -3.2184 |  | -7.7888 | -9.8382 |
| H | 9.9706 |  | -11.3359 | -3.0977 |
| H | 9.3578 |  | -2.5827 | -7.9462 |
| H | 2.4997 |  | -7.8198 | -9.4185 |
| H | 10.1574 |  | 3.0999 | -11.2945 |
| H | -3.0275 |  | -11.4644 | -9.9884 |
| H | 9.4505 |  | -7.9981 | 2.6235 |
| H | 11.8399 |  | 7.3092 | -6.7367 |
| H | 11.5599 |  | 4.9177 | -7.3222 |
| H | 9.3828 |  | 6.0185 | -10.9030 |
| H | 7.5447 |  | 7.2653 | -11.6550 |
| H | 5.1133 |  | 7.6976 | -11.4356 |
| H | 5.9214 |  | 11.1402 | -8.9327 |
| H | 7.0339 |  | 11.7201 | -6.9508 |
| H | 7.3751 |  | 11.2492 | -4.5371 |
| H | 11.0067 |  | 9.0714 | -5.4464 |
| H | 12.3243 |  | 11.0695 | -7.9613 |
| H | 12.9164 |  | 9.6904 | -8.9088 |
| H | 12.6956 |  | 9.5402 | -7.1651 |
| H | 9.7662 |  | 7.9169 | -12.4018 |
| H | 11.2314 |  | 8.7623 | -11.9052 |
| H | 9.8142 |  | 9.6796 | -12.4544 |
| H | 10.5023 |  | 11.4401 | -11.2637 |
| H | 11.3411 |  | 11.8968 | -9.7781 |
| H | 11.9142 |  | 10.5233 | -10.7292 |
| H | 9.4998 |  | 12.9071 | -9.0287 |
| H | 8.6575 |  | 12.4569 | -10.5249 |
| H | 7.7423 |  | 12.7552 | -9.0480 |
| H | 7.5447 |  | -7.2653 | 11.6550 |
| H | 5.1133 |  | -7.6976 | 11.4356 |
| H | 5.9214 |  | -11.1402 | 8.9327 |
| H | 7.0339 |  | -11.7201 | 6.9508 |
| H | 7.3751 |  | -11.2492 | 4.5371 |
| H | 11.0067 |  | -9.0714 | 5.4464 |
| H | 11.8399 |  | -7.3092 | 6.7367 |
| H | 11.5599 |  | -4.9177 | 7.3222 |
| H | 9.3828 |  | -6.0185 | 10.9030 |
| H | 9.8142 |  | -9.6796 | 12.4544 |
| H | 9.7662 |  | -7.9169 | 12.4018 |
| H | 11.2314 |  | -8.7623 | 11.9052 |
| H | 7.7423 |  | -12.7552 | 9.0480 |
| H | 9.4998 |  | -12.9071 | 9.0287 |
| H | 8.6575 |  | -12.4569 | 10.5249 |
| H | 11.9142 |  | -10.5233 | 10.7292 |
| H | 10.5023 |  | -11.4401 | 11.2637 |
| H | 11.3411 |  | -11.8968 | 9.7781 |
| H | 12.3243 |  | -11.0695 | 7.9613 |
| H | 12.9164 |  | -9.6904 | 8.9088 |
| H | 12.6956 |  | -9.5402 | 7.1651 |
| H | -7.0339 |  | -11.7201 | -6.9508 |
| H | -7.3751 |  | -11.2492 | -4.5371 |
| H | -11.0067 |  | -9.0714 | -5.4464 |
| H | -11.8399 |  | -7.3092 | -6.7367 |
| H | -11.5599 |  | -4.9177 | -7.3222 |
| H | -9.3828 |  | -6.0185 | -10.9030 |
| H | -7.5447 |  | -7.2653 | -11.6550 |
| H | -5.1133 |  | -7.6976 | -11.4356 |
| H | -5.9214 |  | -11.1402 | -8.9327 |
| H | -8.6575 |  | -12.4569 | -10.5249 |
| H | -7.7423 |  | -12.7552 | -9.0480 |
| H | -9.4998 |  | -12.9071 | -9.0287 |
| H | -12.6956 |  | -9.5402 | -7.1651 |
| H | -12.3243 |  | -11.0695 | -7.9613 |
| H | -12.9164 |  | -9.6904 | -8.9088 |
| H | -11.9142 |  | -10.5233 | -10.7292 |
| H | -10.5023 |  | -11.4401 | -11.2637 |
| H | -11.3411 |  | -11.8968 | -9.7781 |
| H | -9.8142 |  | -9.6796 | -12.4544 |
| H | -11.2314 |  | -8.7623 | -11.9052 |
| H | -9.7662 |  | -7.9169 | -12.4018 |
| H | -9.2926 |  | -11.6842 | -2.5942 |
| H | 3.2184 |  | 7.7888 | -9.8382 |
| H | 10.0068 |  | -3.1013 | 7.6146 |
| H | 0.9346 |  | 7.2452 | -9.6999 |
| H | -0.4022 |  | 12.0496 | -9.6313 |
| H | 3.0275 |  | 11.4644 | -9.9884 |
| H | -2.4997 |  | 7.8198 | -9.4185 |
| H | -10.0068 |  | -3.1013 | -7.6146 |
| H | 9.8053 |  | -0.7987 | 7.1925 |
| H | 9.6064 |  | 0.2692 | 12.0603 |
| H | 9.1419 |  | 2.5432 | 11.6293 |
| H | 10.1574 |  | -3.0999 | 11.2945 |
| H | 9.3578 |  | 2.5827 | 7.9462 |
| H | -9.6118 |  | -12.1079 | -0.2955 |
| H | -9.7147 |  | -7.2378 | 0.7757 |
| H | -9.4505 |  | -7.9981 | -2.6235 |
| H | -9.8216 |  | -7.6557 | 3.0858 |
| H | -9.9706 |  | -11.3359 | 3.0977 |
| H | -7.5447 |  | 7.2653 | 11.6550 |
| H | -5.1133 |  | 7.6976 | 11.4356 |
| H | -5.9214 |  | 11.1402 | 8.9327 |
| H | -7.0339 |  | 11.7201 | 6.9508 |
| H | -7.3751 |  | 11.2492 | 4.5371 |
| H | -11.0067 |  | 9.0714 | 5.4464 |
| H | -11.8399 |  | 7.3092 | 6.7367 |
| H | -11.5599 |  | 4.9177 | 7.3222 |
| H | -9.3828 |  | 6.0185 | 10.9030 |
| H | -9.8142 |  | 9.6796 | 12.4544 |
| H | -11.2314 |  | 8.7623 | 11.9052 |
| H | -9.7662 |  | 7.9169 | 12.4018 |
| H | -7.7423 |  | 12.7552 | 9.0480 |
| H | -9.4998 |  | 12.9071 | 9.0287 |
| H | -8.6575 |  | 12.4569 | 10.5249 |
| H | -11.9142 |  | 10.5233 | 10.7292 |
| H | -11.3411 |  | 11.8968 | 9.7781 |
| H | -10.5023 |  | 11.4401 | 11.2637 |
| H | -12.3243 |  | 11.0695 | 7.9613 |
| H | -12.6956 |  | 9.5402 | 7.1651 |
| H | -12.9164 |  | 9.6904 | 8.9088 |
| H | -9.2926 |  | 11.6842 | 2.5942 |
| H | -3.2184 |  | 7.7888 | 9.8382 |
| H | -10.0068 |  | 3.1013 | 7.6146 |
| H | -9.8053 |  | 0.7987 | 7.1925 |
| H | -9.6064 |  | -0.2692 | 12.0603 |
| H | -0.9346 |  | 7.2452 | 9.6999 |
| H | 0.4022 |  | 12.0496 | 9.6313 |
| H | -9.6118 |  | 12.1079 | 0.2955 |
| H | -9.7147 |  | 7.2378 | -0.7757 |
| H | -9.8216 |  | 7.6557 | -3.0858 |
| H | -9.1419 |  | -2.5432 | 11.6293 |
| H | 2.6724 |  | 11.5053 | 9.3178 |
| H | -10.1574 |  | 3.0999 | 11.2945 |
| H | -9.4505 |  | 7.9981 | 2.6235 |
| H | -3.0275 |  | 11.4644 | 9.9884 |
| H | -9.9706 |  | 11.3359 | -3.0977 |
| H | 2.4997 |  | 7.8198 | 9.4185 |
| H | -9.3578 |  | -2.5827 | 7.9462 |
| H | -11.5813 |  | 6.8388 | -7.3666 |
| H | -11.3835 |  | 7.4251 | -4.9663 |
| H | -8.9622 |  | 10.8728 | -5.9774 |
| H | -7.0110 |  | 11.4657 | -7.1225 |
| H | -4.5791 |  | 11.0817 | -7.4392 |
| H | -5.3892 |  | 8.6415 | -10.9246 |
| H | -6.5940 |  | 6.7519 | -11.5734 |
| H | -7.0990 |  | 4.3645 | -11.1161 |
| H | -10.7545 |  | 5.4969 | -9.0876 |
| H | -11.8334 |  | 8.0783 | -11.1460 |
| H | -12.3172 |  | 7.3116 | -9.6332 |
| H | -12.4269 |  | 9.0647 | -9.7951 |
| H | -9.1427 |  | 12.3737 | -7.8970 |
| H | -10.6006 |  | 11.9659 | -8.8006 |
| H | -9.1147 |  | 12.4171 | -9.6605 |
| H | -9.8047 |  | 11.2650 | -11.4418 |
| H | -11.2833 |  | 10.8186 | -10.5851 |
| H | -10.7100 |  | 9.8297 | -11.9315 |
| H | -7.9639 |  | 10.4175 | -12.3734 |
| H | -8.8702 |  | 8.9719 | -12.8619 |
| H | -7.1233 |  | 8.8887 | -12.6312 |
| H | -2.6724 |  | 11.5053 | -9.3178 |
| H | -7.0110 |  | -11.4657 | 7.1225 |
| H | -4.5791 |  | -11.0817 | 7.4392 |
| H | -5.3892 |  | -8.6415 | 10.9246 |
| H | -6.5940 |  | -6.7519 | 11.5734 |
| H | -7.0990 |  | -4.3645 | 11.1161 |
| H | -10.7545 |  | -5.4969 | 9.0876 |
| H | -11.5813 |  | -6.8388 | 7.3666 |
| H | -11.3835 |  | -7.4251 | 4.9663 |
| H | -8.9622 |  | -10.8728 | 5.9774 |
| H | -9.1147 |  | -12.4171 | 9.6605 |
| H | -10.6006 |  | -11.9659 | 8.8006 |
| H | -9.1427 |  | -12.3737 | 7.8970 |
| H | -7.1233 |  | -8.8887 | 12.6312 |
| H | -8.8702 |  | -8.9719 | 12.8619 |
| H | -7.9639 |  | -10.4175 | 12.3734 |
| H | -11.2833 |  | -10.8186 | 10.5851 |
| H | -9.8047 |  | -11.2650 | 11.4418 |
| H | -10.7100 |  | -9.8297 | 11.9315 |
| H | -11.8334 |  | -8.0783 | 11.1460 |
| H | -12.3172 |  | -7.3116 | 9.6332 |
| H | -12.4269 |  | -9.0647 | 9.7951 |
| H | 6.5940 |  | 6.7519 | 11.5734 |
| H | 7.0990 |  | 4.3645 | 11.1161 |
| H | 10.7545 |  | 5.4969 | 9.0876 |
| H | 11.5813 |  | 6.8388 | 7.3666 |
| H | 11.3835 |  | 7.4251 | 4.9663 |
| H | 8.9622 |  | 10.8728 | 5.9774 |
| H | 7.0110 |  | 11.4657 | 7.1225 |
| H | 4.5791 |  | 11.0817 | 7.4392 |
| H | 5.3892 |  | 8.6415 | 10.9246 |
| H | 7.9639 |  | 10.4175 | 12.3734 |
| H | 7.1233 |  | 8.8887 | 12.6312 |
| H | 8.8702 |  | 8.9719 | 12.8619 |
| H | 12.3172 |  | 7.3116 | 9.6332 |
| H | 11.8334 |  | 8.0783 | 11.1460 |
| H | 12.4269 |  | 9.0647 | 9.7951 |
| H | 11.2833 |  | 10.8186 | 10.5851 |
| H | 9.8047 |  | 11.2650 | 11.4418 |
| H | 10.7100 |  | 9.8297 | 11.9315 |
| H | 10.6006 |  | 11.9659 | 8.8006 |
| H | 9.1147 |  | 12.4171 | 9.6605 |
| H | 9.1427 |  | 12.3737 | 7.8970 |
| H | -2.6724 |  | -11.5053 | 9.3178 |
| H | -0.4022 |  | -12.0496 | 9.6313 |
| H | 0.9346 |  | -7.2452 | 9.6999 |
| H | 3.2184 |  | -7.7888 | 9.8382 |
| H | -2.4997 |  | -7.8198 | 9.4185 |
| H | 3.0275 |  | -11.4644 | 9.9884 |
| H | -9.1419 |  | 2.5432 | -11.6293 |
| H | -9.6064 |  | 0.2692 | -12.0603 |
| H | -9.8053 |  | -0.7987 | -7.1925 |
| H | -9.3578 |  | 2.5827 | -7.9462 |
| H | -10.1574 |  | -3.0999 | -11.2945 |
| H | 9.8216 |  | 7.6557 | 3.0858 |
| H | 9.7147 |  | 7.2378 | 0.7757 |
| H | 9.6118 |  | 12.1079 | -0.2955 |
| H | 9.9706 |  | 11.3359 | 3.0977 |
| H | 9.4505 |  | 7.9981 | -2.6235 |
| H | 9.2926 |  | 11.6842 | -2.5942 |

c) **Triply interlocked (OH-cube)_2_ (Type 1 conformer)**

| **atom** | **x** | **y** | **z** |
| --- | --- | --- | --- |
| C | -11.7484 | 10.2735 | -10.1719 |
| C | -12.5481 | 9.1632 | -10.5102 |
| C | -14.0302 | 9.4546 | -10.3467 |
| C | -14.0924 | 10.9684 | -9.8395 |
| C | -14.6117 | 8.6419 | -9.2075 |
| C | -14.9535 | 9.4456 | -8.0990 |
| C | -14.6722 | 10.9169 | -8.3509 |
| C | -13.5350 | 11.4041 | -7.4752 |
| C | -12.3747 | 11.6923 | -8.2242 |
| C | -12.5771 | 11.4559 | -9.7102 |
| C | -14.8119 | 7.2664 | -9.1719 |
| C | -15.3554 | 6.6805 | -8.0212 |
| C | -15.6796 | 7.4828 | -6.9146 |
| C | -15.4817 | 8.8809 | -6.9464 |
| C | -13.5411 | 11.5663 | -6.0940 |
| C | -12.3791 | 12.0095 | -5.4472 |
| C | -11.2211 | 12.2693 | -6.1965 |
| C | -11.2124 | 12.1231 | -7.6013 |
| C | -10.3618 | 10.2035 | -10.2703 |
| C | -9.7620 | 9.0156 | -10.7046 |
| C | -10.5624 | 7.9061 | -11.0249 |
| C | -11.9696 | 7.9728 | -10.9321 |
| C | -14.7754 | 9.1773 | -11.6518 |
| C | -15.9328 | 11.7490 | -8.1173 |
| C | -14.9154 | 11.8568 | -10.7549 |
| C | -12.2287 | 12.7116 | -10.5079 |
| N | -9.9005 | 6.7441 | -11.5331 |
| N | -9.9719 | 12.5602 | -5.5690 |
| N | -16.1262 | 6.9155 | -5.6806 |
| C | -9.7438 | 13.6074 | -4.8401 |
| C | -8.4083 | 13.7834 | -4.2392 |
| C | -17.1166 | 6.0795 | -5.6087 |
| C | -17.4890 | 5.4919 | -4.3098 |
| C | -10.1413 | 5.5491 | -11.0856 |
| C | -9.4588 | 4.3899 | -11.6886 |
| C | -9.7372 | 3.1188 | -11.1743 |
| C | -9.1374 | 1.9727 | -11.7284 |
| C | -8.2483 | 2.0675 | -12.8293 |
| C | -7.9574 | 3.3414 | -13.3329 |
| C | -8.5450 | 4.4860 | -12.7686 |
| C | -18.6097 | 4.6529 | -4.2649 |
| C | -18.9978 | 4.0377 | -3.0626 |
| C | -18.2622 | 4.2343 | -1.8653 |
| C | -17.1505 | 5.0824 | -1.9073 |
| C | -16.7708 | 5.7113 | -3.1076 |
| C | -8.1371 | 14.9740 | -3.5531 |
| C | -6.8782 | 15.1934 | -2.9674 |
| C | -5.8552 | 14.2141 | -3.0377 |
| C | -6.1269 | 13.0239 | -3.7203 |
| C | -7.3798 | 12.8111 | -4.3230 |
| C | -4.5170 | 14.3655 | -2.4412 |
| C | -7.6240 | 0.9025 | -13.4812 |
| C | -18.5916 | 3.5866 | -0.5830 |
| O | -8.1429 | 5.6528 | -13.3492 |
| O | -7.4757 | 11.6154 | -4.9651 |
| O | -15.6806 | 6.5153 | -2.9803 |
| O | -6.7584 | 16.4009 | -2.3477 |
| O | -20.1098 | 3.2597 | -3.1772 |
| O | -9.4994 | 0.8138 | -11.1154 |
| C | 1.1989 | 14.4785 | 2.4812 |
| C | 1.4095 | 14.7051 | 1.1054 |
| C | 0.9229 | 16.0790 | 0.6745 |
| C | 0.4320 | 16.7770 | 2.0238 |
| C | -0.3383 | 15.9179 | -0.1534 |
| C | -1.4883 | 16.3633 | 0.5301 |
| C | -1.1500 | 16.9635 | 1.8835 |
| C | -1.7402 | 16.1340 | 3.0065 |
| C | -0.7476 | 15.4346 | 3.7259 |
| C | 0.6444 | 15.7008 | 3.1856 |
| C | -0.4417 | 15.3658 | -1.4259 |
| C | -1.7062 | 15.2174 | -2.0060 |
| C | -2.8546 | 15.6199 | -1.3017 |
| C | -2.7518 | 16.2238 | -0.0309 |
| C | -3.0802 | 16.0170 | 3.3593 |
| C | -3.4410 | 15.1866 | 4.4296 |
| C | -2.4497 | 14.4897 | 5.1387 |
| C | -1.0850 | 14.6172 | 4.7947 |
| C | 1.4956 | 13.2431 | 3.0479 |
| C | 2.0546 | 12.2378 | 2.2494 |
| C | 2.3033 | 12.4861 | 0.8894 |
| C | 1.9714 | 13.7241 | 0.2991 |
| C | 2.0024 | 16.8320 | -0.0991 |
| C | -1.6405 | 18.4094 | 1.9555 |
| C | 1.1510 | 18.0845 | 2.3026 |
| C | 1.5876 | 16.1352 | 4.3076 |
| N | -2.7329 | 13.6763 | 6.2796 |
| N | -4.1678 | 15.4726 | -1.8571 |
| N | 2.8020 | 11.4189 | 0.0767 |
| C | -3.1245 | -2.6637 | -14.7659 |
| C | -2.4282 | -3.8308 | -14.3874 |
| C | -3.0591 | -5.0833 | -14.9688 |
| C | -4.3158 | -4.5553 | -15.8044 |
| C | -3.6560 | -5.9643 | -13.8904 |
| C | -5.0649 | -6.0089 | -13.9581 |
| C | -5.6115 | -5.1362 | -15.0735 |
| C | -6.3097 | -3.9182 | -14.4999 |
| C | -5.6147 | -2.7243 | -14.7806 |
| C | -4.3554 | -2.9730 | -15.5934 |
| C | -2.9726 | -6.6975 | -12.9264 |
| C | -3.6957 | -7.4883 | -12.0235 |
| C | -5.0978 | -7.5366 | -12.0997 |
| C | -5.7966 | -6.7905 | -13.0755 |
| C | -7.4904 | -3.8924 | -13.7634 |
| C | -7.9823 | -2.6677 | -13.2980 |
| C | -7.2869 | -1.4766 | -13.5774 |
| C | -6.0892 | -1.5008 | -14.3257 |
| C | -2.6631 | -1.4091 | -14.3819 |
| C | -1.5080 | -1.3139 | -13.5972 |
| C | -0.8336 | -2.4790 | -13.1952 |
| C | -1.2890 | -3.7549 | -13.5976 |
| C | -2.0249 | -5.8631 | -15.7828 |
| C | -6.5610 | -5.9302 | -15.9694 |
| C | -4.2467 | -4.9485 | -17.2692 |
| C | -4.3775 | -2.1484 | -16.8797 |
| N | -7.8458 | -0.2893 | -13.0132 |
| N | -5.8926 | -8.3875 | -11.2709 |
| N | 0.3837 | -2.4429 | -12.4449 |
| C | -20.9138 | 0.8130 | 2.9787 |
| C | -20.9959 | 0.2031 | 1.7088 |
| C | -21.6033 | -1.1862 | 1.7803 |
| C | -21.9759 | -1.3872 | 3.3215 |
| C | -20.5604 | -2.2485 | 1.4934 |
| C | -20.2870 | -3.0450 | 2.6235 |
| C | -21.0792 | -2.6015 | 3.8418 |
| C | -20.1394 | -1.9805 | 4.8566 |
| C | -20.3153 | -0.5848 | 4.9620 |
| C | -21.4447 | -0.0838 | 4.0788 |
| C | -19.8966 | -2.4763 | 0.2913 |
| C | -18.9499 | -3.5046 | 0.2122 |
| C | -18.6902 | -4.3019 | 1.3394 |
| C | -19.3663 | -4.0835 | 2.5583 |
| C | -19.1700 | -2.6307 | 5.6127 |
| C | -18.3404 | -1.8803 | 6.4553 |
| C | -18.4972 | -0.4860 | 6.5319 |
| C | -19.5057 | 0.1739 | 5.7962 |
| C | -20.3960 | 2.0966 | 3.1133 |
| C | -19.9449 | 2.7787 | 1.9756 |
| C | -20.0209 | 2.1652 | 0.7140 |
| C | -20.5577 | 0.8656 | 0.5711 |
| C | -22.7765 | -1.3114 | 0.8086 |
| C | -21.8543 | -3.7698 | 4.4466 |
| C | -23.4603 | -1.6205 | 3.5363 |
| C | -22.4895 | 0.6469 | 4.9218 |
| C | -16.3991 | 0.1915 | 7.4374 |
| N | -17.6430 | -5.2723 | 1.2416 |
| N | -19.6403 | 2.8246 | -0.4957 |
| C | -17.8315 | -6.5353 | 1.4669 |
| C | -3.7075 | 12.8191 | 6.3149 |
| C | 0.6026 | -1.5633 | -11.5119 |
| C | -3.9004 | 11.9880 | 7.5176 |
| C | -4.9516 | 11.0656 | 7.5220 |
| C | -5.1480 | 10.2044 | 8.6181 |
| C | -4.2821 | 10.2347 | 9.7391 |
| C | -3.2463 | 11.1781 | 9.7470 |
| C | -3.0579 | 12.0464 | 8.6587 |
| O | -2.0144 | 12.9057 | 8.8177 |
| O | -6.2163 | 9.3732 | 8.4750 |
| C | -4.3795 | 9.3102 | 10.8815 |
| C | 1.9058 | -1.5330 | -10.8233 |
| C | 2.1388 | -0.5076 | -9.9013 |
| C | 3.3806 | -0.3998 | -9.2461 |
| C | 4.4206 | -1.3338 | -9.4846 |
| C | 4.1843 | -2.3640 | -10.4050 |
| C | 2.9505 | -2.4597 | -11.0663 |
| C | 5.7327 | -1.2848 | -8.8133 |
| O | 2.8698 | -3.4982 | -11.9545 |
| O | 3.4596 | 0.6592 | -8.3979 |
| C | -16.7002 | -7.4755 | 1.3564 |
| C | -16.9668 | -8.8423 | 1.4926 |
| C | -15.9335 | -9.7904 | 1.3820 |
| C | -14.5953 | -9.3923 | 1.1330 |
| C | -14.3292 | -8.0252 | 1.0003 |
| C | -15.3617 | -7.0781 | 1.1111 |
| O | -14.9426 | -5.7878 | 0.9700 |
| C | -13.4713 | -10.3379 | 0.9912 |
| O | -16.3541 | -11.0753 | 1.5345 |
| C | 2.3751 | -9.7180 | 9.2831 |
| C | 3.0060 | -10.2556 | 8.1412 |
| C | 4.5176 | -10.1145 | 8.2114 |
| C | 4.7933 | -9.3786 | 9.6056 |
| C | 5.0499 | -9.1583 | 7.1636 |
| C | 5.5699 | -7.9764 | 7.7328 |
| C | 5.4292 | -7.9586 | 9.2446 |
| C | 4.3762 | -6.9459 | 9.6548 |
| C | 3.2235 | -7.5631 | 10.1826 |
| C | 3.3600 | -9.0753 | 10.2373 |
| C | 5.0906 | -9.3539 | 5.7869 |
| C | 5.6462 | -8.3621 | 4.9682 |
| C | 6.1548 | -7.1821 | 5.5367 |
| C | 6.1252 | -6.9850 | 6.9360 |
| C | 4.4428 | -5.5615 | 9.5360 |
| C | 3.3446 | -4.7840 | 9.9277 |
| C | 2.1872 | -5.4078 | 10.4215 |
| C | 2.1217 | -6.8106 | 10.5660 |
| C | 0.9958 | -9.7994 | 9.4382 |
| C | 0.2306 | -10.4076 | 8.4371 |
| C | 0.8552 | -10.9206 | 7.2859 |
| C | 2.2586 | -10.8539 | 7.1342 |
| C | 5.1755 | -11.4872 | 8.0626 |
| C | 6.7635 | -7.6527 | 9.9233 |
| C | 5.6762 | -10.1996 | 10.5288 |
| C | 3.1596 | -9.5772 | 11.6665 |
| N | 0.1192 | -11.5973 | 6.2606 |
| N | 0.9925 | -4.6655 | 10.6805 |
| N | 6.7888 | -6.1533 | 4.7730 |
| C | 0.9215 | -3.7022 | 11.5453 |
| C | -0.3468 | -2.9688 | 11.7186 |
| C | 6.4053 | -5.8317 | 3.5732 |
| C | 7.1008 | -4.7599 | 2.8394 |
| C | -1.0973 | -11.2529 | 5.9554 |
| C | -1.8654 | -12.0144 | 4.9547 |
| C | -3.2123 | -11.6810 | 4.7773 |
| C | -4.0082 | -12.3761 | 3.8473 |
| C | -3.4666 | -13.4152 | 3.0498 |
| C | -2.1207 | -13.7581 | 3.2382 |
| C | -1.3322 | -13.0696 | 4.1729 |
| C | 6.6978 | -4.4972 | 1.5249 |
| C | 7.3008 | -3.4677 | 0.7801 |
| C | 8.3153 | -2.6516 | 1.3426 |
| C | 8.7304 | -2.9252 | 2.6512 |
| C | 8.1418 | -3.9684 | 3.3877 |
| C | -0.4293 | -2.0297 | 12.7568 |
| C | -1.6047 | -1.2921 | 12.9592 |
| C | -2.7377 | -1.4594 | 12.1204 |
| C | -2.6571 | -2.3961 | 11.0867 |
| C | -1.4835 | -3.1522 | 10.8932 |
| C | -3.9899 | -0.6979 | 12.2803 |
| C | -4.2286 | -14.1222 | 2.0044 |
| C | 8.9392 | -1.5167 | 0.6368 |
| O | -0.0331 | -13.4927 | 4.2266 |
| O | -1.5737 | -4.0411 | 9.8678 |
| O | 8.6601 | -4.1191 | 4.6385 |
| O | -1.5463 | -0.4220 | 14.0112 |
| O | 6.8116 | -3.3511 | -0.4843 |
| O | -5.3001 | -11.9530 | 3.8071 |
| C | -9.1320 | 5.3810 | 13.4407 |
| C | -9.3381 | 4.0045 | 13.6700 |
| C | -8.4671 | 3.4765 | 14.7953 |
| C | -7.6328 | 4.7442 | 15.2945 |
| C | -7.4245 | 2.5102 | 14.2707 |
| C | -6.1170 | 3.0333 | 14.3658 |
| C | -6.1036 | 4.4374 | 14.9440 |
| C | -5.7398 | 5.4602 | 13.8861 |
| C | -6.8231 | 6.3054 | 13.5652 |
| C | -8.0720 | 5.9602 | 14.3567 |
| C | -7.6407 | 1.2353 | 13.7591 |
| C | -6.5443 | 0.4594 | 13.3602 |
| C | -5.2423 | 0.9714 | 13.4820 |
| C | -5.0208 | 2.2783 | 13.9747 |
| C | -4.5100 | 5.6206 | 13.2574 |
| C | -4.3552 | 6.6373 | 12.3071 |
| C | -5.4381 | 7.4724 | 11.9859 |
| C | -6.6901 | 7.3110 | 12.6173 |
| C | -9.8534 | 6.0489 | 12.4570 |
| C | -10.7924 | 5.3422 | 11.6939 |
| C | -10.9821 | 3.9690 | 11.9175 |
| C | -10.2528 | 3.2846 | 12.9143 |
| C | -9.3299 | 2.8072 | 15.8657 |
| C | -5.1366 | 4.5232 | 16.1253 |
| C | -7.8443 | 5.0367 | 16.7692 |
| C | -8.5999 | 7.1900 | 15.0942 |
| N | -5.3460 | 8.4452 | 10.9415 |
| N | -4.0703 | 0.1990 | 13.2182 |
| N | -11.8403 | 3.1875 | 11.0824 |
| C | -10.8020 | -14.6448 | 0.6561 |
| C | -11.7082 | -14.3766 | -0.3912 |
| C | -11.4909 | -15.2892 | -1.5860 |
| C | -10.3100 | -16.2659 | -1.1375 |
| C | -10.9157 | -14.4882 | -2.7383 |
| C | -9.5677 | -14.8179 | -2.9932 |
| C | -9.0709 | -15.9323 | -2.0884 |
| C | -7.9947 | -15.4172 | -1.1551 |
| C | -8.4425 | -15.3302 | 0.1797 |
| C | -9.8778 | -15.8016 | 0.3305 |
| C | -11.5543 | -13.5020 | -3.4822 |
| C | -10.8388 | -12.8167 | -4.4733 |
| C | -9.4858 | -13.1217 | -4.6952 |
| C | -8.8401 | -14.1424 | -3.9625 |
| C | -6.6997 | -15.0393 | -1.4933 |
| C | -5.8415 | -14.5650 | -0.4947 |
| C | -6.2931 | -14.4684 | 0.8318 |
| C | -7.6061 | -14.8566 | 1.1813 |
| C | -10.8256 | -13.8849 | 1.8208 |
| C | -11.7574 | -12.8454 | 1.9467 |
| C | -12.6624 | -12.5931 | 0.9047 |
| C | -12.6488 | -13.3619 | -0.2787 |
| C | -12.7908 | -15.9815 | -1.9890 |
| C | -8.5594 | -17.1050 | -2.9243 |
| C | -10.7089 | -17.7296 | -1.1909 |
| C | -9.9762 | -16.8833 | 1.4065 |
| N | -5.4991 | -13.8864 | 1.8682 |
| N | -8.6705 | -12.3423 | -5.5714 |
| N | -13.6962 | -11.6128 | 1.0502 |
| C | 9.9776 | 1.9499 | -2.9457 |
| C | 10.1436 | 0.6195 | -3.3909 |
| C | 10.6730 | 0.5472 | -4.8155 |
| C | 10.9381 | 2.0774 | -5.2120 |
| C | 9.5137 | 0.1246 | -5.7015 |
| C | 9.0355 | 1.1856 | -6.5038 |
| C | 9.8847 | 2.4362 | -6.3691 |
| C | 9.0768 | 3.5819 | -5.7934 |
| C | 9.4314 | 3.8717 | -4.4559 |
| C | 10.5240 | 2.9587 | -3.9348 |
| C | 8.8531 | -1.1006 | -5.7019 |
| C | 7.7228 | -1.2865 | -6.5076 |
| C | 7.2319 | -0.2185 | -7.2738 |
| C | 7.8838 | 1.0337 | -7.2661 |
| C | 8.0824 | 4.3194 | -6.4272 |
| C | 7.4274 | 5.3491 | -5.7388 |
| C | 7.7939 | 5.6396 | -4.4163 |
| C | 8.8048 | 4.8999 | -3.7663 |
| C | 9.3499 | 2.2057 | -1.7302 |
| C | 8.9438 | 1.1392 | -0.9180 |
| C | 9.1528 | -0.1813 | -1.3464 |
| C | 9.7552 | -0.4488 | -2.5933 |
| C | 11.9372 | -0.3193 | -4.9427 |
| C | 10.5771 | 2.8143 | -7.6967 |
| C | 12.3735 | 2.3414 | -5.6377 |
| C | 11.7150 | 3.7547 | -3.3556 |
| N | 7.2458 | 6.7471 | -3.6957 |
| N | 6.0026 | -0.3108 | -7.9988 |
| N | 8.6456 | -1.2952 | -0.6072 |
| C | -8.9899 | -12.0662 | -6.7982 |
| C | -8.1053 | -11.2015 | -7.6002 |
| C | -8.4340 | -10.9845 | -8.9443 |
| C | -7.6418 | -10.1494 | -9.7505 |
| C | -6.4973 | -9.4928 | -9.2284 |
| C | -6.1642 | -9.7163 | -7.8885 |
| C | -6.9487 | -10.5640 | -7.0847 |
| C | -5.6404 | -8.5847 | -10.0120 |
| O | -6.4862 | -10.6875 | -5.8111 |
| O | -8.0842 | -10.0438 | -11.0343 |
| C | -13.1076 | 3.4281 | 10.9501 |
| C | -13.9211 | 2.5858 | 10.0541 |
| C | -15.3056 | 2.7924 | 10.0330 |
| C | -16.1339 | 2.0233 | 9.1969 |
| C | -15.5942 | 1.0275 | 8.3442 |
| C | -14.2101 | 0.8240 | 8.3637 |
| C | -13.3818 | 1.5807 | 9.2112 |
| O | -12.0667 | 1.2474 | 9.1228 |
| N | -17.6912 | 0.3219 | 7.3961 |
| O | -17.4565 | 2.3354 | 9.2950 |
| C | 5.9748 | 6.9042 | -3.4922 |
| C | 5.5037 | 8.0628 | -2.7073 |
| C | 4.1415 | 8.1510 | -2.4156 |
| C | 3.6392 | 9.2089 | -1.6290 |
| C | 4.4961 | 10.2144 | -1.1228 |
| C | 5.8630 | 10.1338 | -1.4285 |
| C | 6.3626 | 9.0786 | -2.2057 |
| O | 7.7040 | 9.1478 | -2.4269 |
| O | 2.2963 | 9.1458 | -1.4282 |
| C | 4.0440 | 11.3404 | -0.2854 |
| H | -14.5507 | 6.6432 | -10.0250 |
| H | -15.5014 | 5.6033 | -7.9808 |
| H | -15.7336 | 9.4875 | -6.0791 |
| H | -14.4335 | 11.3456 | -5.5106 |
| H | -12.3706 | 12.1221 | -4.3651 |
| H | -10.3073 | 12.3309 | -8.1674 |
| H | -9.7420 | 11.0599 | -10.0094 |
| H | -8.6779 | 8.9484 | -10.7902 |
| H | -12.5827 | 7.1161 | -11.1969 |
| H | -14.3783 | 9.7781 | -12.4787 |
| H | -14.6831 | 8.1251 | -11.9467 |
| H | -15.8461 | 9.3934 | -11.5580 |
| H | -16.2786 | 11.6622 | -7.0799 |
| H | -15.7535 | 12.8135 | -8.3088 |
| H | -16.7585 | 11.4220 | -8.7600 |
| H | -14.9512 | 12.8914 | -10.3909 |
| H | -14.5024 | 11.8855 | -11.7713 |
| H | -15.9519 | 11.5058 | -10.8362 |
| H | -12.3781 | 12.5597 | -11.5834 |
| H | -11.1785 | 12.9939 | -10.3653 |
| H | -12.8372 | 13.5692 | -10.1976 |
| H | -10.4676 | 14.4125 | -4.6254 |
| H | -17.7369 | 5.7510 | -6.4621 |
| H | -10.8355 | 5.3042 | -10.2611 |
| H | -10.4209 | 2.9835 | -10.3341 |
| H | -7.2733 | 3.4758 | -14.1723 |
| H | -19.2059 | 4.4565 | -5.1575 |
| H | -16.5498 | 5.2759 | -1.0163 |
| H | -8.8903 | 15.7581 | -3.4610 |
| H | -5.3784 | 12.2340 | -3.8050 |
| H | -3.8725 | 13.4710 | -2.5362 |
| H | -6.9998 | 1.1428 | -14.3594 |
| H | -17.8831 | 3.7965 | 0.2386 |
| H | -8.6282 | 6.4662 | -12.9705 |
| H | -8.3841 | 11.4757 | -5.4085 |
| H | -15.4069 | 6.9520 | -3.8591 |
| H | -5.8283 | 16.5497 | -1.9612 |
| H | -20.3814 | 2.8338 | -2.2911 |
| H | -9.0460 | -0.0056 | -11.5270 |
| H | 0.4460 | 15.0290 | -1.9703 |
| H | -1.7906 | 14.7955 | -3.0047 |
| H | -3.6463 | 16.5519 | 0.4934 |
| H | -3.8497 | 16.5586 | 2.8118 |
| H | -4.4871 | 15.0979 | 4.7135 |
| H | -0.3275 | 14.0784 | 5.3608 |
| H | 1.2895 | 13.0499 | 4.0998 |
| H | 2.2814 | 11.2619 | 2.6751 |
| H | 2.1212 | 13.8813 | -0.7704 |
| H | 2.9320 | 16.9409 | 0.4825 |
| H | 1.6630 | 17.8351 | -0.3847 |
| H | 2.2580 | 16.3079 | -1.0294 |
| H | -2.7270 | 18.4664 | 1.8157 |
| H | -1.1847 | 19.0288 | 1.1740 |
| H | -1.4150 | 18.8644 | 2.9269 |
| H | 0.7972 | 18.5479 | 3.2326 |
| H | 2.2336 | 17.9393 | 2.4049 |
| H | 0.9953 | 18.8132 | 1.4972 |
| H | 2.5950 | 16.3497 | 3.9299 |
| H | 1.2194 | 17.0297 | 4.8233 |
| H | 1.6914 | 15.3498 | 5.0665 |
| H | -1.8861 | -6.6629 | -12.8665 |
| H | -3.1627 | -8.0747 | -11.2782 |
| H | -6.8827 | -6.8369 | -13.1220 |
| H | -8.0271 | -4.8131 | -13.5398 |
| H | -8.9028 | -2.6337 | -12.7148 |
| H | -5.5375 | -0.5863 | -14.5246 |
| H | -3.1850 | -0.5042 | -14.6865 |
| H | -1.1249 | -0.3252 | -13.3231 |
| H | -0.7513 | -4.6484 | -13.2886 |
| H | -2.4598 | -6.7648 | -16.2295 |
| H | -1.1874 | -6.1870 | -15.1529 |
| H | -1.6023 | -5.2525 | -16.5893 |
| H | -7.4326 | -6.2874 | -15.4070 |
| H | -6.9400 | -5.3193 | -16.7970 |
| H | -6.0703 | -6.8136 | -16.3950 |
| H | -3.3520 | -4.5403 | -17.7561 |
| H | -4.2159 | -6.0384 | -17.3944 |
| H | -5.1159 | -4.5830 | -17.8303 |
| H | -5.2495 | -2.3891 | -17.4988 |
| H | -3.4761 | -2.3181 | -17.4810 |
| H | -4.4205 | -1.0744 | -16.6632 |
| H | -20.0989 | -1.8577 | -0.5820 |
| H | -18.4102 | -3.6826 | -0.7174 |
| H | -19.1488 | -4.6958 | 3.4287 |
| H | -19.0432 | -3.7096 | 5.5479 |
| H | -17.5835 | -2.3828 | 7.0534 |
| H | -19.6269 | 1.2519 | 5.8809 |
| H | -20.3361 | 2.5739 | 4.0900 |
| H | -19.5509 | 3.7877 | 2.0758 |
| H | -20.6173 | 0.4063 | -0.4136 |
| H | -23.5450 | -0.5537 | 1.0028 |
| H | -22.4482 | -1.1761 | -0.2294 |
| H | -23.2483 | -2.2988 | 0.8709 |
| H | -21.1784 | -4.5667 | 4.7787 |
| H | -22.5467 | -4.2134 | 3.7216 |
| H | -22.4352 | -3.4556 | 5.3223 |
| H | -23.7006 | -1.7571 | 4.5983 |
| H | -24.0587 | -0.7743 | 3.1750 |
| H | -23.8121 | -2.5152 | 3.0074 |
| H | -23.3161 | 1.0199 | 4.3058 |
| H | -22.9095 | -0.0041 | 5.6977 |
| H | -22.0518 | 1.5130 | 5.4333 |
| H | -18.7980 | -6.9999 | 1.7321 |
| H | -4.4196 | 12.6240 | 5.4933 |
| H | -0.1178 | -0.7943 | -11.1770 |
| H | -5.6383 | 10.9833 | 6.6772 |
| H | -2.5595 | 11.2581 | 10.5909 |
| H | -1.8778 | 13.5116 | 8.0074 |
| H | -6.3256 | 8.7334 | 9.2594 |
| H | -15.8133 | -0.5163 | 6.8210 |
| H | 1.3730 | 0.2365 | -9.6733 |
| H | 4.9531 | -3.1051 | -10.6323 |
| H | 6.4168 | -2.1137 | -9.0679 |
| H | 1.9469 | -3.5651 | -12.3851 |
| H | 4.3679 | 0.7253 | -7.9323 |
| H | -17.9781 | -9.2071 | 1.6811 |
| H | -13.3129 | -7.6562 | 0.7954 |
| H | -15.7143 | -5.1201 | 0.9785 |
| H | -12.4841 | -9.8688 | 0.8271 |
| H | -15.5987 | -11.7534 | 1.4287 |
| H | 4.7165 | -10.2754 | 5.3368 |
| H | 5.6970 | -8.5274 | 3.8931 |
| H | 6.5258 | -6.0681 | 7.3647 |
| H | 5.3331 | -5.0770 | 9.1365 |
| H | 3.3832 | -3.7022 | 9.8188 |
| H | 1.2198 | -7.2795 | 10.9522 |
| H | 0.5089 | -9.3946 | 10.3237 |
| H | -0.8468 | -10.4882 | 8.5647 |
| H | 2.7318 | -11.2633 | 6.2436 |
| H | 6.2654 | -11.4318 | 8.1645 |
| H | 4.8006 | -12.1993 | 8.8066 |
| H | 4.9723 | -11.9184 | 7.0729 |
| H | 7.1512 | -6.6746 | 9.6094 |
| H | 6.6558 | -7.6171 | 11.0142 |
| H | 7.5342 | -8.3977 | 9.6820 |
| H | 5.2158 | -11.1662 | 10.7718 |
| H | 5.8634 | -9.6834 | 11.4785 |
| H | 6.6517 | -10.4130 | 10.0752 |
| H | 3.8973 | -9.1445 | 12.3524 |
| H | 2.1679 | -9.3044 | 12.0476 |
| H | 3.2391 | -10.6691 | 11.7248 |
| H | 1.7472 | -3.3659 | 12.1966 |
| H | 5.5622 | -6.2930 | 3.0271 |
| H | -1.6475 | -10.4018 | 6.3994 |
| H | -3.6779 | -10.8744 | 5.3470 |
| H | -1.6605 | -14.5634 | 2.6611 |
| H | 5.9087 | -5.0803 | 1.0462 |
| H | 9.5164 | -2.3388 | 3.1302 |
| H | 0.4119 | -1.8565 | 13.4286 |
| H | -3.4931 | -2.5628 | 10.4033 |
| H | -4.7874 | -0.9464 | 11.5553 |
| H | -3.6346 | -14.8171 | 1.3832 |
| H | 9.6369 | -0.9152 | 1.2463 |
| H | 0.4883 | -13.0434 | 4.9789 |
| H | -0.7193 | -4.5829 | 9.7379 |
| H | 8.2253 | -4.8853 | 5.1505 |
| H | -2.4429 | 0.0437 | 14.1765 |
| H | 7.2540 | -2.5983 | -1.0111 |
| H | -5.8547 | -12.4531 | 3.1084 |
| H | -8.6493 | 0.8345 | 13.6666 |
| H | -6.7115 | -0.5448 | 12.9741 |
| H | -4.0069 | 2.6698 | 14.0477 |
| H | -3.6678 | 4.9663 | 13.4882 |
| H | -3.3983 | 6.7561 | 11.8003 |
| H | -7.5232 | 7.9585 | 12.3527 |
| H | -9.6932 | 7.1097 | 12.2712 |
| H | -11.3515 | 5.8545 | 10.9137 |
| H | -10.4066 | 2.2188 | 13.0693 |
| H | -10.0716 | 3.5008 | 16.2783 |
| H | -9.8820 | 1.9536 | 15.4535 |
| H | -8.7212 | 2.4267 | 16.6944 |
| H | -4.0981 | 4.3201 | 15.8091 |
| H | -5.3841 | 3.7914 | 16.9029 |
| H | -5.1454 | 5.5202 | 16.5799 |
| H | -7.2655 | 5.9092 | 17.0972 |
| H | -7.5400 | 4.1901 | 17.3976 |
| H | -8.8981 | 5.2449 | 16.9940 |
| H | -7.8491 | 7.6055 | 15.7763 |
| H | -9.4974 | 6.9548 | 15.6782 |
| H | -8.8725 | 7.9871 | 14.3916 |
| H | -3.5798 | 9.4222 | 11.6360 |
| H | -12.5969 | -13.2510 | -3.2958 |
| H | -11.3263 | -12.0302 | -5.0446 |
| H | -7.7931 | -14.3717 | -4.1507 |
| H | -6.3492 | -15.1027 | -2.5222 |
| H | -4.8274 | -14.2470 | -0.7610 |
| H | -7.9417 | -14.7764 | 2.2128 |
| H | -10.1238 | -14.0837 | 2.6294 |
| H | -11.7829 | -12.2461 | 2.8541 |
| H | -13.3585 | -13.1555 | -1.0759 |
| H | -12.6413 | -16.6531 | -2.8425 |
| H | -13.2079 | -16.5691 | -1.1624 |
| H | -13.5557 | -15.2520 | -2.2820 |
| H | -7.7281 | -16.7977 | -3.5706 |
| H | -8.1904 | -17.9199 | -2.2904 |
| H | -9.3427 | -17.5084 | -3.5764 |
| H | -11.5576 | -17.9431 | -0.5288 |
| H | -11.0037 | -18.0339 | -2.2030 |
| H | -9.8839 | -18.3844 | -0.8826 |
| H | -9.3262 | -17.7369 | 1.1817 |
| H | -9.6705 | -16.4959 | 2.3861 |
| H | -11.0023 | -17.2541 | 1.5125 |
| H | 9.1893 | -1.9187 | -5.0658 |
| H | 7.2077 | -2.2447 | -6.5010 |
| H | 7.4645 | 1.8606 | -7.8366 |
| H | 7.8165 | 4.1042 | -7.4670 |
| H | 6.6522 | 5.9268 | -6.2367 |
| H | 9.0964 | 5.1479 | -2.7410 |
| H | 9.1554 | 3.2272 | -1.4058 |
| H | 8.4446 | 1.3392 | 0.0275 |
| H | 9.9059 | -1.4799 | -2.9230 |
| H | 12.6754 | 0.0400 | -4.1947 |
| C | 11.7640 | -1.8331 | -4.7737 |
| H | 12.4075 | -0.1376 | -5.9365 |
| C | 9.6743 | 2.9415 | -8.9302 |
| H | 11.3571 | 2.0565 | -7.9201 |
| H | 11.1128 | 3.7744 | -7.5436 |
| H | 12.5251 | 3.3828 | -5.9461 |
| H | 13.0865 | 2.1359 | -4.8265 |
| H | 12.6825 | 1.7015 | -6.4821 |
| H | 12.0071 | 4.5258 | -4.0980 |
| H | 12.5845 | 3.0697 | -3.2566 |
| C | 11.4961 | 4.4324 | -1.9971 |
| H | -9.8910 | -12.4294 | -7.3231 |
| H | -9.3077 | -11.4562 | -9.3966 |
| H | -5.2924 | -9.2421 | -7.4330 |
| H | -4.8196 | -8.1133 | -9.4419 |
| H | -7.0554 | -11.3154 | -5.2438 |
| H | -7.4843 | -9.4486 | -11.6059 |
| H | -13.6642 | 4.2283 | 11.4698 |
| H | -15.7748 | 3.5494 | 10.6637 |
| H | -13.7347 | 0.0693 | 7.7192 |
| H | -11.4755 | 1.7831 | 9.7572 |
| H | -18.0403 | 1.7560 | 8.6954 |
| H | 5.1791 | 6.2214 | -3.8409 |
| H | 3.4307 | 7.4048 | -2.7800 |
| H | 6.5699 | 10.8770 | -1.0532 |
| H | 8.0654 | 8.3311 | -2.9211 |
| H | 1.9476 | 9.9018 | -0.8409 |
| H | 4.8352 | 12.0648 | -0.0136 |
| C | -2.2176 | 7.1554 | -10.8617 |
| C | -2.8324 | 5.9089 | -11.1049 |
| C | -4.3349 | 5.9618 | -10.8866 |
| C | -4.6255 | 7.4652 | -10.4259 |
| C | -4.7567 | 5.1105 | -9.7065 |
| C | -5.2048 | 5.8903 | -8.6187 |
| C | -5.1333 | 7.3781 | -8.9132 |
| C | -4.0350 | 8.0331 | -8.0976 |
| C | -2.9697 | 8.4823 | -8.9055 |
| C | -3.2075 | 8.1962 | -10.3780 |
| C | -4.7495 | 3.7224 | -9.6225 |
| C | -5.2054 | 3.1011 | -8.4521 |
| C | -5.6533 | 3.8819 | -7.3738 |
| C | -5.6499 | 5.2925 | -7.4480 |
| C | -3.9918 | 8.2017 | -6.7175 |
| C | -2.8756 | 8.8163 | -6.1336 |
| C | -1.8105 | 9.2429 | -6.9428 |
| C | -1.8524 | 9.0853 | -8.3452 |
| C | -0.8501 | 7.3208 | -11.0523 |
| C | -0.0794 | 6.2299 | -11.4733 |
| C | -0.6896 | 4.9835 | -11.6944 |
| C | -2.0820 | 4.8166 | -11.5180 |
| C | -5.0571 | 5.5243 | -12.1605 |
| C | -6.4794 | 8.0483 | -8.6413 |
| C | -5.6187 | 8.1668 | -11.3350 |
| C | -3.1101 | 9.4794 | -11.2008 |
| N | 0.0252 | 3.8416 | -12.1726 |
| N | -0.5953 | 9.7499 | -6.3845 |
| N | -6.0518 | 3.3022 | -6.1283 |
| C | -0.5461 | 10.7871 | -5.6082 |
| C | 0.7525 | 11.2282 | -5.0646 |
| C | -6.9049 | 2.3262 | -6.0525 |
| C | -7.2604 | 1.7586 | -4.7396 |
| C | 1.2638 | 3.6006 | -11.8605 |
| C | 1.9364 | 2.4136 | -12.4194 |
| C | 3.2935 | 2.2274 | -12.1394 |
| C | 3.9808 | 1.1043 | -12.6386 |
| C | 3.3174 | 0.1231 | -13.4166 |
| C | 1.9618 | 0.3204 | -13.7126 |
| C | 1.2820 | 1.4456 | -13.2233 |
| C | -8.2300 | 0.7488 | -4.7020 |
| C | -8.6060 | 0.1591 | -3.4829 |
| C | -8.0125 | 0.5591 | -2.2577 |
| C | -7.0481 | 1.5722 | -2.2946 |
| C | -6.6782 | 2.1693 | -3.5140 |
| C | 0.7940 | 12.4353 | -4.3534 |
| C | 1.9996 | 12.8965 | -3.8043 |
| C | 3.2027 | 12.1550 | -3.9327 |
| C | 3.1633 | 10.9537 | -4.6462 |
| C | 1.9584 | 10.4991 | -5.2165 |
| C | 4.4818 | 12.5743 | -3.3331 |
| C | 3.9601 | -1.1123 | -13.9002 |
| C | -8.3446 | -0.0389 | -0.9512 |
| O | -0.0397 | 1.4933 | -13.5686 |
| O | 2.0876 | 9.3308 | -5.9013 |
| O | -5.7367 | 3.1431 | -3.3819 |
| O | 1.8997 | 14.0921 | -3.1499 |
| O | -9.5624 | -0.8020 | -3.6106 |
| O | 5.2984 | 1.0723 | -12.3008 |
| C | 9.5384 | 15.0439 | 2.2913 |
| C | 9.7476 | 14.9958 | 0.8971 |
| C | 8.9071 | 16.0211 | 0.1567 |
| C | 8.0708 | 16.7623 | 1.2982 |
| C | 7.8657 | 15.3286 | -0.6990 |
| C | 6.5598 | 15.5045 | -0.1936 |
| C | 6.5412 | 16.3641 | 1.0580 |
| C | 6.1620 | 15.5451 | 2.2748 |
| C | 7.2387 | 15.3910 | 3.1731 |
| C | 8.4992 | 16.0785 | 2.6773 |
| C | 8.0840 | 14.5841 | -1.8530 |
| C | 6.9931 | 14.0031 | -2.5129 |
| C | 5.6954 | 14.1763 | -2.0054 |
| C | 5.4693 | 14.9328 | -0.8321 |
| C | 4.9225 | 14.9793 | 2.5528 |
| C | 4.7584 | 14.2419 | 3.7309 |
| C | 5.8354 | 14.0817 | 4.6192 |
| C | 7.0902 | 14.6689 | 4.3501 |
| C | 10.2298 | 14.1846 | 3.1385 |
| C | 11.1264 | 13.2554 | 2.5938 |
| C | 11.3069 | 13.1917 | 1.2025 |
| C | 10.6233 | 14.0758 | 0.3386 |
| C | 9.7963 | 16.9301 | -0.6905 |
| C | 5.5798 | 17.5402 | 0.8823 |
| C | 8.2865 | 18.2648 | 1.2980 |
| C | 9.0457 | 17.0362 | 3.7343 |
| N | 5.6838 | 13.3835 | 5.8598 |
| N | 4.5218 | 13.6795 | -2.6487 |
| N | 12.0845 | 12.1638 | 0.5872 |
| C | 10.3011 | -2.9867 | -14.7806 |
| C | 11.1476 | -4.0161 | -14.3188 |
| C | 10.8252 | -5.3531 | -14.9629 |
| C | 9.5774 | -5.0535 | -15.9150 |
| C | 10.2986 | -6.3301 | -13.9310 |
| C | 8.9201 | -6.5848 | -14.0943 |
| C | 8.3355 | -5.8292 | -15.2739 |
| C | 7.3934 | -4.7353 | -14.8117 |
| C | 7.8883 | -3.4477 | -15.1051 |
| C | 9.2590 | -3.4969 | -15.7573 |
| C | 11.0159 | -6.9493 | -12.9133 |
| C | 10.3549 | -7.8228 | -12.0397 |
| C | 8.9806 | -8.0667 | -12.1994 |
| C | 8.2504 | -7.4492 | -13.2399 |
| C | 6.1697 | -4.8906 | -14.1683 |
| C | 5.4253 | -3.7553 | -13.8255 |
| C | 5.9151 | -2.4756 | -14.1344 |
| C | 7.1613 | -2.3114 | -14.7785 |
| C | 10.4523 | -1.6842 | -14.3165 |
| C | 11.4340 | -1.4079 | -13.3565 |
| C | 12.2562 | -2.4422 | -12.8794 |
| C | 12.1318 | -3.7588 | -13.3743 |
| C | 12.0599 | -5.9131 | -15.6683 |
| C | 7.6143 | -6.7898 | -16.2199 |
| C | 9.8345 | -5.4560 | -17.3559 |
| C | 9.2799 | -2.6935 | -17.0555 |
| N | 5.2376 | -1.2809 | -13.7359 |
| N | 8.2595 | -8.9961 | -11.3870 |
| N | 13.2923 | -2.2120 | -11.9194 |
| C | -10.4819 | -2.9671 | 2.6173 |
| C | -10.3084 | -3.6632 | 1.3995 |
| C | -10.6648 | -5.1331 | 1.5198 |
| C | -10.9761 | -5.3355 | 3.0822 |
| C | -9.4610 | -6.0140 | 1.2614 |
| C | -8.9820 | -6.6325 | 2.4379 |
| C | -9.7944 | -6.2529 | 3.6627 |
| C | -8.9947 | -5.3188 | 4.5481 |
| C | -9.5727 | -4.0325 | 4.6393 |
| C | -10.8139 | -3.8955 | 3.7708 |
| C | -8.8256 | -6.2359 | 0.0440 |
| C | -7.7005 | -7.0670 | -0.0152 |
| C | -7.2186 | -7.6653 | 1.1596 |
| C | -7.8662 | -7.4572 | 2.3959 |
| C | -7.7915 | -5.5961 | 5.1905 |
| C | -7.1733 | -4.6101 | 5.9685 |
| C | -7.7616 | -3.3405 | 6.0752 |
| C | -8.9604 | -3.0392 | 5.3939 |
| C | -10.2802 | -1.5920 | 2.6729 |
| C | -9.8592 | -0.9007 | 1.5300 |
| C | -9.6287 | -1.6059 | 0.3385 |
| C | -9.8517 | -2.9993 | 0.2690 |
| C | -11.8641 | -5.5108 | 0.6259 |
| C | -10.3273 | -7.4842 | 4.4257 |
| C | -12.3492 | -5.9484 | 3.3138 |
| C | -12.0752 | -3.4733 | 4.5473 |
| C | -5.9613 | -2.0102 | 6.9180 |
| N | -6.0041 | -8.4186 | 1.1825 |
| N | -9.2468 | -0.9706 | -0.8836 |
| C | -5.7801 | -9.4304 | 0.4017 |
| C | 5.0919 | 12.2279 | 5.9152 |
| C | 13.0869 | -1.5193 | -10.8401 |
| C | 4.8836 | 11.5524 | 7.2071 |
| C | 4.1846 | 10.3403 | 7.1980 |
| C | 3.9120 | 9.6583 | 8.3976 |
| C | 4.3480 | 10.1697 | 9.6469 |
| C | 5.0616 | 11.3743 | 9.6543 |
| C | 5.3237 | 12.0601 | 8.4553 |
| O | 6.0187 | 13.2204 | 8.6243 |
| O | 3.2115 | 8.5072 | 8.2187 |
| C | 4.0731 | 9.5100 | 10.9371 |
| C | 14.1856 | -1.3123 | -9.8800 |
| C | 13.9235 | -0.5495 | -8.7361 |
| C | 14.9302 | -0.3202 | -7.7825 |
| C | 16.2297 | -0.8625 | -7.9390 |
| C | 16.4919 | -1.6252 | -9.0830 |
| C | 15.4893 | -1.8456 | -10.0444 |
| C | 17.3244 | -0.6757 | -6.9678 |
| O | 15.8973 | -2.5994 | -11.1010 |
| O | 14.5230 | 0.4500 | -6.7327 |
| C | -4.4896 | -10.1388 | 0.4831 |
| C | -4.3104 | -11.2761 | -0.3151 |
| C | -3.1112 | -12.0023 | -0.2603 |
| C | -2.0440 | -11.6068 | 0.5844 |
| C | -2.2155 | -10.4625 | 1.3694 |
| C | -3.4238 | -9.7407 | 1.3296 |
| O | -3.4457 | -8.6668 | 2.1627 |
| C | -0.7780 | -12.3528 | 0.7071 |
| O | -3.0927 | -13.1092 | -1.0647 |
| C | 11.9846 | -8.3279 | 11.6404 |
| C | 12.8929 | -8.9717 | 10.7735 |
| C | 14.3375 | -8.7416 | 11.1824 |
| C | 14.2467 | -7.8741 | 12.5194 |
| C | 15.0155 | -7.8314 | 10.1769 |
| C | 15.2918 | -6.5563 | 10.7131 |
| C | 14.8796 | -6.4482 | 12.1709 |
| C | 13.7369 | -5.4670 | 12.3380 |
| C | 12.5259 | -6.1131 | 12.6654 |
| C | 12.6909 | -7.6172 | 12.7786 |
| C | 15.3426 | -8.1308 | 8.8587 |
| C | 15.9270 | -7.1431 | 8.0558 |
| C | 16.1789 | -5.8667 | 8.5867 |
| C | 15.8746 | -5.5670 | 9.9329 |
| C | 13.7845 | -4.0838 | 12.2022 |
| C | 12.6174 | -3.3323 | 12.3963 |
| C | 11.4140 | -3.9833 | 12.7093 |
| C | 11.3580 | -5.3874 | 12.8498 |
| C | 10.6159 | -8.3933 | 11.4029 |
| C | 10.1422 | -9.1115 | 10.2964 |
| C | 11.0480 | -9.7692 | 9.4480 |
| C | 12.4407 | -9.7016 | 9.6845 |
| C | 15.0845 | -10.0667 | 11.3166 |
| C | 16.0769 | -6.0433 | 13.0304 |
| C | 14.9310 | -8.5422 | 13.6984 |
| C | 12.1677 | -8.1163 | 14.1256 |
| N | 10.6548 | -10.5964 | 8.3529 |
| N | 10.1756 | -3.2748 | 12.7924 |
| N | 16.8102 | -4.8325 | 7.8254 |
| C | 9.9465 | -2.3312 | 13.6520 |
| C | 8.6333 | -1.6613 | 13.6560 |
| C | 16.4691 | -4.5743 | 6.5986 |
| C | 17.1542 | -3.5059 | 5.8496 |
| C | 9.6006 | -10.3811 | 7.6250 |
| C | 9.2882 | -11.3058 | 6.5202 |
| C | 8.0784 | -11.1334 | 5.8401 |
| C | 7.7345 | -11.9695 | 4.7633 |
| C | 8.6191 | -12.9789 | 4.3071 |
| C | 9.8247 | -13.1607 | 4.9954 |
| C | 10.1501 | -12.3504 | 6.0966 |
| C | 16.7819 | -3.2991 | 4.5173 |
| C | 17.3923 | -2.2909 | 3.7485 |
| C | 18.3876 | -1.4473 | 4.3024 |
| C | 18.7627 | -1.6566 | 5.6357 |
| C | 18.1626 | -2.6727 | 6.3987 |
| C | 8.3661 | -0.7321 | 14.6695 |
| C | 7.1270 | -0.0712 | 14.7276 |
| C | 6.1217 | -0.3104 | 13.7564 |
| C | 6.3903 | -1.2347 | 12.7415 |
| C | 7.6225 | -1.9112 | 12.6942 |
| C | 4.8053 | 0.3505 | 13.7487 |
| C | 8.3526 | -13.8372 | 3.1377 |
| C | 19.0481 | -0.3606 | 3.5573 |
| O | 11.3406 | -12.6638 | 6.6784 |
| O | 7.7189 | -2.7943 | 11.6629 |
| O | 18.6378 | -2.7586 | 7.6724 |
| O | 7.0080 | 0.7896 | 15.7766 |
| O | 16.9319 | -2.2274 | 2.4696 |
| O | 6.5051 | -11.7081 | 4.2344 |
| C | -0.7926 | 5.3593 | 12.8103 |
| C | -1.0452 | 4.0786 | 13.3428 |
| C | -0.5278 | 3.9422 | 14.7650 |
| C | 0.0198 | 5.3951 | 15.1387 |
| C | 0.7043 | 3.0572 | 14.7683 |
| C | 1.8809 | 3.7779 | 15.0575 |
| C | 1.5975 | 5.2423 | 15.3458 |
| C | 2.2172 | 6.1204 | 14.2769 |
| C | 1.2432 | 6.6805 | 13.4229 |
| C | -0.1654 | 6.2899 | 13.8282 |
| C | 0.7588 | 1.6949 | 14.4931 |
| C | 2.0012 | 1.0518 | 14.4689 |
| C | 3.1757 | 1.7823 | 14.7204 |
| C | 3.1225 | 3.1548 | 15.0423 |
| C | 3.5676 | 6.3818 | 14.0733 |
| C | 3.9578 | 7.1995 | 13.0033 |
| C | 2.9854 | 7.7316 | 12.1425 |
| C | 1.6106 | 7.4818 | 12.3522 |
| C | -1.1197 | 5.6544 | 11.4904 |
| C | -1.7506 | 4.6820 | 10.7056 |
| C | -2.0378 | 3.4219 | 11.2560 |
| C | -1.6765 | 3.1027 | 12.5823 |
| C | -1.6037 | 3.3872 | 15.6955 |
| C | 2.1089 | 5.6183 | 16.7356 |
| C | -0.6744 | 5.9876 | 16.3519 |
| C | -1.0361 | 7.5347 | 14.0107 |
| N | 3.3273 | 8.4495 | 10.9563 |
| N | 4.4658 | 1.1593 | 14.7073 |
| N | -2.6042 | 2.4167 | 10.4104 |
| C | 2.7913 | -15.9150 | 0.2725 |
| C | 2.1198 | -15.7072 | -0.9500 |
| C | 2.7696 | -16.4659 | -2.0938 |
| C | 4.0238 | -17.2034 | -1.4327 |
| C | 3.3720 | -15.5142 | -3.1070 |
| C | 4.7825 | -15.5534 | -3.1038 |
| C | 5.3240 | -16.5355 | -2.0795 |
| C | 5.9897 | -15.7980 | -0.9340 |
| C | 5.2697 | -15.9295 | 0.2719 |
| C | 4.0234 | -16.7818 | 0.1069 |
| C | 2.6899 | -14.6684 | -3.9750 |
| C | 3.4165 | -13.8604 | -4.8589 |
| C | 4.8207 | -13.9036 | -4.8534 |
| C | 5.5187 | -14.7534 | -3.9670 |
| C | 7.1571 | -15.0433 | -0.9791 |
| C | 7.6078 | -14.4029 | 0.1833 |
| C | 6.8711 | -14.5174 | 1.3732 |
| C | 5.6951 | -15.2959 | 1.4309 |
| C | 2.3256 | -15.3300 | 1.4456 |
| C | 1.1976 | -14.5024 | 1.3984 |
| C | 0.5499 | -14.2729 | 0.1734 |
| C | 0.9994 | -14.8905 | -1.0151 |
| C | 1.7510 | -17.3919 | -2.7593 |
| C | 6.2995 | -17.5141 | -2.7322 |
| C | 3.9835 | -18.7086 | -1.6271 |
| C | 4.0249 | -17.9418 | 1.1001 |
| N | 7.2060 | -13.7590 | 2.5376 |
| N | 5.6073 | -13.0343 | -5.6727 |
| N | -0.6326 | -13.4755 | 0.0678 |
| C | 20.3409 | 2.8305 | -0.1825 |
| C | 20.4050 | 1.4734 | -0.5634 |
| C | 21.0447 | 1.2917 | -1.9284 |
| C | 21.4323 | 2.7698 | -2.3940 |
| C | 20.0202 | 0.8228 | -2.9424 |
| C | 19.7328 | 1.8119 | -3.9060 |
| C | 20.5316 | 3.0830 | -3.6756 |
| C | 19.6212 | 4.2138 | -3.2403 |
| C | 19.8286 | 4.5771 | -1.8929 |
| C | 20.9209 | 3.7490 | -1.2395 |
| C | 19.3874 | -0.4149 | -2.9902 |
| C | 18.4652 | -0.6771 | -4.0118 |
| C | 18.1801 | 0.3143 | -4.9638 |
| C | 18.8140 | 1.5742 | -4.9197 |
| C | 18.6688 | 4.8716 | -4.0112 |
| C | 17.9047 | 5.8934 | -3.4334 |
| C | 18.1062 | 6.2450 | -2.0878 |
| C | 19.0828 | 5.5895 | -1.3054 |
| C | 19.7893 | 3.1950 | 1.0410 |
| C | 19.3013 | 2.2004 | 1.8991 |
| C | 19.3608 | 0.8520 | 1.5113 |
| C | 19.9166 | 0.4759 | 0.2683 |
| C | 22.2140 | 0.3107 | -1.8460 |
| C | 21.3078 | 3.4644 | -4.9357 |
| C | 22.9168 | 2.9233 | -2.6728 |
| C | 21.9929 | 4.6580 | -0.6389 |
| N | 17.4000 | 7.3186 | -1.4599 |
| N | 17.1585 | 0.1045 | -5.9462 |
| N | 18.7803 | -0.1900 | 2.2988 |
| C | 5.3754 | -12.8715 | -6.9399 |
| C | 6.1917 | -11.9266 | -7.7224 |
| C | 5.9484 | -11.8306 | -9.0979 |
| C | 6.6764 | -10.9299 | -9.8946 |
| C | 7.6676 | -10.0860 | -9.3314 |
| C | 7.9177 | -10.1890 | -7.9582 |
| C | 7.1980 | -11.0991 | -7.1629 |
| C | 8.4425 | -9.0997 | -10.1049 |
| O | 7.5657 | -11.0900 | -5.8527 |
| O | 6.3302 | -10.9579 | -11.2114 |
| C | -3.8599 | 2.0979 | 10.4377 |
| C | -4.3632 | 1.0422 | 9.5393 |
| C | -5.7234 | 0.7086 | 9.6119 |
| C | -6.2639 | -0.2823 | 8.7780 |
| C | -5.4552 | -0.9714 | 7.8349 |
| C | -4.0996 | -0.6425 | 7.7662 |
| C | -3.5545 | 0.3502 | 8.6057 |
| O | -2.2234 | 0.5468 | 8.4189 |
| N | -7.2211 | -2.3232 | 6.9244 |
| O | -7.5911 | -0.5097 | 8.9811 |
| C | 16.1283 | 7.5120 | -1.6428 |
| C | 15.4503 | 8.6458 | -0.9905 |
| C | 14.0949 | 8.8450 | -1.2736 |
| C | 13.3841 | 9.9060 | -0.6832 |
| C | 14.0132 | 10.7920 | 0.2282 |
| C | 15.3710 | 10.5951 | 0.5087 |
| C | 16.0837 | 9.5443 | -0.0947 |
| O | 17.3956 | 9.4883 | 0.2692 |
| O | 12.0843 | 9.9690 | -1.0749 |
| C | 13.3158 | 11.9036 | 0.9007 |
| H | -4.3969 | 3.1159 | -10.4544 |
| H | -5.1937 | 2.0158 | -8.3778 |
| H | -5.9895 | 5.8840 | -6.6000 |
| H | -4.8106 | 7.8561 | -6.0876 |
| H | -2.8275 | 8.9335 | -5.0530 |
| H | -1.0186 | 9.4188 | -8.9585 |
| H | -0.3747 | 8.2835 | -10.8729 |
| H | 0.9882 | 6.3599 | -11.6352 |
| H | -2.5434 | 3.8483 | -11.6990 |
| H | -4.7595 | 6.1303 | -13.0241 |
| H | -4.8361 | 4.4795 | -12.4052 |
| H | -6.1497 | 5.6046 | -12.0638 |
| H | -6.7577 | 7.9641 | -7.5829 |
| H | -6.4528 | 9.1178 | -8.8792 |
| H | -7.2862 | 7.5858 | -9.2222 |
| H | -5.8204 | 9.1923 | -11.0035 |
| H | -5.2514 | 8.2247 | -12.3677 |
| H | -6.5795 | 7.6349 | -11.3666 |
| H | -3.2778 | 9.2869 | -12.2671 |
| H | -2.1185 | 9.9381 | -11.1063 |
| H | -3.8427 | 10.2252 | -10.8706 |
| H | -1.4114 | 11.4036 | -5.3085 |
| H | -7.4209 | 1.8626 | -6.9131 |
| H | 1.8877 | 4.2142 | -11.1862 |
| H | 3.8499 | 2.9375 | -11.5246 |
| H | 1.4083 | -0.3976 | -14.3221 |
| H | -8.7158 | 0.3963 | -5.6136 |
| H | -6.5589 | 1.9232 | -1.3839 |
| H | -0.1030 | 13.0386 | -4.2126 |
| H | 4.0566 | 10.3379 | -4.7730 |
| H | 5.3288 | 11.8825 | -3.4947 |
| H | 3.2753 | -1.8367 | -14.3778 |
| H | -7.7676 | 0.3573 | -0.0968 |
| H | -0.4847 | 2.3669 | -13.2719 |
| H | 1.2076 | 9.0136 | -6.3100 |
| H | -5.4741 | 3.5605 | -4.2736 |
| H | 2.8124 | 14.4296 | -2.8237 |
| H | -9.8279 | -1.2108 | -2.7140 |
| H | 5.7730 | 0.2407 | -12.6563 |
| H | 9.0904 | 14.4450 | -2.2456 |
| H | 7.1609 | 13.4285 | -3.4220 |
| H | 4.4571 | 15.0560 | -0.4486 |
| H | 4.0818 | 15.0931 | 1.8670 |
| H | 3.7872 | 13.8029 | 3.9563 |
| H | 7.9132 | 14.5445 | 5.0503 |
| H | 10.0749 | 14.2198 | 4.2156 |
| H | 11.6555 | 12.5670 | 3.2492 |
| H | 10.7765 | 14.0155 | -0.7369 |
| H | 10.5334 | 17.4601 | -0.0759 |
| H | 9.2068 | 17.6773 | -1.2348 |
| H | 10.3558 | 16.3538 | -1.4376 |
| H | 4.5428 | 17.1914 | 0.7341 |
| H | 5.8420 | 18.1487 | 0.0091 |
| H | 5.5772 | 18.1917 | 1.7636 |
| H | 7.7029 | 18.7582 | 2.0854 |
| H | 9.3396 | 18.5238 | 1.4663 |
| H | 7.9907 | 18.7164 | 0.3426 |
| H | 9.9480 | 17.5508 | 3.3840 |
| H | 8.3059 | 17.7962 | 4.0122 |
| H | 9.3156 | 16.5010 | 4.6531 |
| H | 12.0808 | -6.7615 | -12.7881 |
| H | 10.9154 | -8.3194 | -11.2510 |
| H | 7.1876 | -7.6533 | -13.3555 |
| H | 5.7893 | -5.8810 | -13.9244 |
| H | 4.4745 | -3.8680 | -13.2937 |
| H | 7.5331 | -1.3138 | -15.0008 |
| H | 9.8089 | -0.8847 | -14.6805 |
| H | 11.5588 | -0.3910 | -12.9909 |
| H | 12.7873 | -4.5455 | -13.0070 |
| H | 11.8513 | -6.8815 | -16.1379 |
| H | 12.8850 | -6.0673 | -14.9624 |
| H | 12.4253 | -5.2301 | -16.4443 |
| H | 6.7677 | -7.2765 | -15.7201 |
| H | 7.2129 | -6.2664 | -17.0955 |
| H | 8.2800 | -7.5858 | -16.5734 |
| H | 10.6886 | -4.9153 | -17.7828 |
| H | 10.0523 | -6.5280 | -17.4435 |
| H | 8.9676 | -5.2475 | -17.9958 |
| H | 8.5357 | -3.0635 | -17.7706 |
| H | 10.2638 | -2.7315 | -17.5373 |
| H | 9.0508 | -1.6365 | -16.8721 |
| H | -9.2085 | -5.7692 | -0.8694 |
| H | -7.1929 | -7.2255 | -0.9642 |
| H | -7.4931 | -7.9463 | 3.3003 |
| H | -7.3106 | -6.5678 | 5.0825 |
| H | -6.2452 | -4.8363 | 6.4888 |
| H | -9.3726 | -2.0335 | 5.4575 |
| H | -10.4524 | -1.0479 | 3.6068 |
| H | -9.7194 | 0.1769 | 1.5715 |
| H | -9.6531 | -3.5287 | -0.6616 |
| H | -12.7845 | -5.0255 | 1.0302 |
| C | -11.7696 | -5.1504 | -0.8614 |
| H | -12.0411 | -6.6069 | 0.7183 |
| C | -9.2956 | -8.3288 | 5.1843 |
| H | -10.8586 | -8.1351 | 3.7012 |
| H | -11.0892 | -7.1433 | 5.1583 |
| H | -12.5892 | -6.0283 | 4.3828 |
| H | -13.1504 | -5.3538 | 2.8444 |
| H | -12.4245 | -6.9578 | 2.8897 |
| H | -12.8983 | -3.3405 | 3.8135 |
| H | -12.3803 | -4.3111 | 5.2106 |
| C | -11.9726 | -2.2085 | 5.4083 |
| H | -6.4914 | -9.8382 | -0.3387 |
| H | 4.7043 | 11.6730 | 5.0398 |
| H | 12.1244 | -1.0586 | -10.5508 |
| H | 3.8284 | 9.8962 | 6.2662 |
| H | 5.4231 | 11.8123 | 10.5860 |
| H | 6.1654 | 13.7177 | 7.7496 |
| H | 2.9926 | 8.0345 | 9.0965 |
| H | -5.1955 | -2.4575 | 6.2577 |
| H | 12.9359 | -0.1186 | -8.5590 |
| H | 17.4756 | -2.0645 | -9.2586 |
| H | 18.2481 | -1.2370 | -7.1982 |
| H | 15.1585 | -2.7386 | -11.7925 |
| H | 15.2679 | 0.6223 | -6.0588 |
| H | -5.0992 | -11.6253 | -0.9850 |
| H | -1.4281 | -10.1088 | 2.0378 |
| H | -4.3398 | -8.1715 | 2.1433 |
| H | -0.0221 | -11.8882 | 1.3662 |
| H | -2.1952 | -13.5961 | -1.0319 |
| H | 15.1413 | -9.1172 | 8.4444 |
| H | 16.1912 | -7.3740 | 7.0260 |
| H | 16.0870 | -4.5778 | 10.3333 |
| H | 14.7152 | -3.5806 | 11.9452 |
| H | 12.6435 | -2.2510 | 12.2800 |
| H | 10.4164 | -5.8758 | 13.0896 |
| H | 9.9124 | -7.8875 | 12.0619 |
| H | 9.0676 | -9.1550 | 10.1056 |
| H | 13.1317 | -10.2105 | 9.0151 |
| H | 16.1262 | -9.9125 | 11.6222 |
| H | 14.6082 | -10.7268 | 12.0510 |
| H | 15.1061 | -10.6095 | 10.3635 |
| H | 16.4743 | -5.0682 | 12.7228 |
| H | 15.8050 | -5.9621 | 14.0891 |
| H | 16.8981 | -6.7649 | 12.9438 |
| H | 14.4846 | -9.5182 | 13.9273 |
| H | 14.8601 | -7.9312 | 14.6071 |
| H | 15.9977 | -8.7127 | 13.5049 |
| H | 12.6744 | -7.6236 | 14.9636 |
| H | 11.0951 | -7.9151 | 14.2340 |
| H | 12.3051 | -9.1987 | 14.2342 |
| H | 10.6577 | -1.9747 | 14.4177 |
| H | 15.6709 | -5.0937 | 6.0366 |
| H | 8.8940 | -9.5396 | 7.7422 |
| H | 7.3769 | -10.3430 | 6.1252 |
| H | 10.5360 | -13.9326 | 4.6976 |
| H | 16.0124 | -3.9105 | 4.0411 |
| H | 19.5273 | -1.0407 | 6.1118 |
| H | 9.1068 | -0.5034 | 15.4373 |
| H | 5.6553 | -1.4549 | 11.9653 |
| H | 4.1711 | 0.0989 | 12.8779 |
| H | 9.1815 | -14.5083 | 2.8512 |
| H | 19.7570 | 0.2465 | 4.1480 |
| H | 11.5385 | -12.0843 | 7.4957 |
| H | 8.6149 | -3.2808 | 11.6424 |
| H | 18.2031 | -3.5137 | 8.2013 |
| H | 6.0906 | 1.2317 | 15.8162 |
| H | 17.3812 | -1.4924 | 1.9244 |
| H | 6.2859 | -12.3086 | 3.4383 |
| H | -0.1496 | 1.1223 | 14.2805 |
| H | 2.0478 | -0.0144 | 14.2607 |
| H | 4.0373 | 3.7037 | 15.2529 |
| H | 4.3232 | 5.9544 | 14.7303 |
| H | 5.0134 | 7.3943 | 12.8283 |
| H | 0.8703 | 7.9033 | 11.6754 |
| H | -0.8828 | 6.6270 | 11.0616 |
| H | -2.0091 | 4.8970 | 9.6693 |
| H | -1.8632 | 2.1045 | 12.9779 |
| H | -2.5218 | 3.9970 | 15.6798 |
| H | -1.8845 | 2.3656 | 15.4072 |
| H | -1.2485 | 3.3354 | 16.7319 |
| H | 3.1907 | 5.4547 | 16.8168 |
| H | 1.6351 | 5.0119 | 17.5167 |
| H | 1.9229 | 6.6741 | 16.9628 |
| H | -0.2828 | 6.9838 | 16.5941 |
| H | -0.5385 | 5.3612 | 17.2428 |
| H | -1.7543 | 6.0925 | 16.1907 |
| H | -0.6228 | 8.2087 | 14.7700 |
| H | -2.0581 | 7.2698 | 14.3086 |
| H | -1.1075 | 8.1060 | 13.0779 |
| H | 4.5236 | 9.9958 | 11.8198 |
| H | 1.6020 | -14.6262 | -3.9720 |
| H | 2.8887 | -13.1851 | -5.5290 |
| H | 6.6065 | -14.7670 | -3.9677 |
| H | 7.7162 | -14.9366 | -1.9071 |
| H | 8.5093 | -13.7949 | 0.1508 |
| H | 5.1353 | -15.3753 | 2.3602 |
| H | 2.8310 | -15.5012 | 2.3946 |
| H | 0.8244 | -14.0478 | 2.3221 |
| H | 0.4788 | -14.7133 | -1.9534 |
| H | 2.1985 | -17.9577 | -3.5844 |
| H | 1.3297 | -18.1077 | -2.0436 |
| H | 0.9109 | -16.8224 | -3.1753 |
| H | 7.1692 | -16.9898 | -3.1466 |
| H | 6.6787 | -18.2460 | -2.0095 |
| H | 5.8306 | -18.0623 | -3.5576 |
| H | 3.0869 | -19.1511 | -1.1747 |
| H | 3.9783 | -18.9792 | -2.6905 |
| H | 4.8519 | -19.2004 | -1.1712 |
| H | 4.9029 | -18.5849 | 0.9695 |
| H | 4.0424 | -17.5779 | 2.1348 |
| H | 3.1279 | -18.5633 | 0.9933 |
| H | 19.5986 | -1.1782 | -2.2427 |
| H | 17.9591 | -1.6395 | -4.0523 |
| H | 18.5756 | 2.3349 | -5.6589 |
| H | 18.5090 | 4.5980 | -5.0527 |
| H | 17.1677 | 6.4194 | -4.0363 |
| H | 19.2329 | 5.8778 | -0.2669 |
| H | 19.7304 | 4.2413 | 1.3362 |
| H | 18.8563 | 2.4798 | 2.8517 |
| H | 19.9484 | -0.5726 | -0.0203 |
| H | 22.9736 | 0.6468 | -1.1304 |
| H | 21.8782 | -0.6802 | -1.5166 |
| H | 22.6990 | 0.1793 | -2.8203 |
| H | 20.6305 | 3.6580 | -5.7763 |
| H | 21.9895 | 2.6648 | -5.2481 |
| H | 21.8993 | 4.3751 | -4.7832 |
| H | 23.1657 | 3.9430 | -2.9933 |
| H | 23.5208 | 2.7075 | -1.7823 |
| H | 23.2536 | 2.2432 | -3.4653 |
| H | 22.4368 | 5.3145 | -1.3967 |
| H | 22.7997 | 4.0785 | -0.1754 |
| H | 21.5728 | 5.3063 | 0.1399 |
| H | 4.5932 | -13.3967 | -7.5174 |
| H | 5.1913 | -12.4486 | -9.5836 |
| H | 8.6716 | -9.5682 | -7.4708 |
| H | 9.1423 | -8.4878 | -9.5077 |
| H | 7.0222 | -11.7407 | -5.2864 |
| H | 6.8757 | -10.3041 | -11.7730 |
| H | -4.6236 | 2.5565 | 11.0943 |
| H | -6.3935 | 1.2203 | 10.3072 |
| H | -3.4274 | -1.1395 | 7.0623 |
| H | -1.8374 | 1.2653 | 9.0320 |
| H | -7.9773 | -1.1918 | 8.3284 |
| H | 15.4695 | 6.8755 | -2.2624 |
| H | 13.5544 | 8.1869 | -1.9579 |
| H | 15.9075 | 11.2504 | 1.1967 |
| H | 17.8987 | 8.7380 | -0.2009 |
| H | 11.5751 | 10.7420 | -0.6467 |
| H | 13.9194 | 12.4550 | 1.6429 |
| H | 10.5296 | 4.9831 | -1.9870 |
| C | 12.6325 | 5.4014 | -1.6723 |
| H | 11.4268 | 3.6665 | -1.1991 |
| H | 12.6925 | 6.2103 | -2.4094 |
| H | 13.6020 | 4.8922 | -1.6470 |
| H | 12.4803 | 5.8668 | -0.6904 |
| H | 11.1857 | -2.0585 | -3.8517 |
| C | 13.1194 | -2.5360 | -4.7099 |
| H | 11.1778 | -2.2442 | -5.6192 |
| H | 13.6963 | -2.2217 | -3.8324 |
| H | 13.7277 | -2.3183 | -5.5961 |
| H | 12.9983 | -3.6231 | -4.6503 |
| H | 8.7731 | 3.5476 | -8.6915 |
| H | 9.3016 | 1.9425 | -9.2324 |
| C | 10.4200 | 3.5811 | -10.1002 |
| H | 10.7358 | 4.6047 | -9.8675 |
| H | 9.7834 | 3.6311 | -10.9912 |
| H | 11.3181 | 3.0146 | -10.3689 |
| H | -8.4120 | -8.5374 | 4.5423 |
| C | -9.9002 | -9.6500 | 5.6569 |
| H | -8.9173 | -7.7650 | 6.0603 |
| H | -10.2295 | -10.2695 | 4.8154 |
| H | -10.7656 | -9.4900 | 6.3095 |
| H | -9.1665 | -10.2355 | 6.2238 |
| H | -11.5172 | -1.3769 | 4.8276 |
| H | -11.2991 | -2.3898 | 6.2707 |
| C | -13.3462 | -1.7785 | 5.9220 |
| H | -14.0072 | -1.4820 | 5.0997 |
| H | -13.2546 | -0.9179 | 6.6071 |
| H | -13.8394 | -2.5864 | 6.4739 |
| H | -10.7850 | -5.4484 | -1.2796 |
| H | -11.8405 | -4.0506 | -0.9863 |
| C | -12.8904 | -5.8202 | -1.6554 |
| H | -12.7437 | -6.9019 | -1.7362 |
| H | -12.9486 | -5.4187 | -2.6728 |
| H | -13.8687 | -5.6569 | -1.1792 |

d) **OMe-cube** (Type 1 conformer)

| **atom** | **x** | **y** | **z** |
| --- | --- | --- | --- |
| C | 9.2599 | 10.3289 | -8.2430 |
| C | 10.3289 | 8.2430 | -9.2599 |
| C | 8.2430 | 9.2599 | -10.3289 |
| C | 9.6317 | 9.6317 | -9.6317 |
| C | 9.7777 | 9.3737 | -7.1819 |
| C | 10.3691 | 8.2249 | -7.7444 |
| C | 10.8988 | 7.2313 | -6.9258 |
| C | 10.8280 | 7.3761 | -5.5359 |
| N | 10.2221 | 8.5919 | -3.5467 |
| C | 10.2215 | 8.5153 | -4.9753 |
| C | 9.7015 | 9.5339 | -5.8045 |
| C | 9.3737 | 7.1819 | -9.7777 |
| C | 8.2249 | 7.7444 | -10.3691 |
| C | 7.2313 | 6.9258 | -10.8988 |
| C | 7.3761 | 5.5359 | -10.8280 |
| N | 8.5919 | 3.5467 | -10.2221 |
| C | 8.5153 | 4.9753 | -10.2215 |
| C | 9.5339 | 5.8045 | -9.7015 |
| C | 7.1819 | 9.7777 | -9.3737 |
| C | 7.7444 | 10.3691 | -8.2249 |
| C | 6.9258 | 10.8988 | -7.2313 |
| C | 5.5359 | 10.8280 | -7.3761 |
| N | 3.5467 | 10.2221 | -8.5919 |
| C | 4.9753 | 10.2215 | -8.5153 |
| C | 5.8045 | 9.7015 | -9.5339 |
| C | 10.3289 | 9.2599 | 8.2430 |
| C | 8.2430 | 10.3289 | 9.2599 |
| C | 9.2599 | 8.2430 | 10.3289 |
| C | 9.6317 | 9.6317 | 9.6317 |
| C | 9.3737 | 9.7777 | 7.1819 |
| C | 8.2249 | 10.3691 | 7.7444 |
| C | 7.2313 | 10.8988 | 6.9258 |
| C | 7.3761 | 10.8280 | 5.5359 |
| N | 8.5919 | 10.2221 | 3.5467 |
| C | 8.5153 | 10.2215 | 4.9753 |
| C | 9.5339 | 9.7015 | 5.8045 |
| C | 7.1819 | 9.3737 | 9.7777 |
| C | 7.7444 | 8.2249 | 10.3691 |
| C | 6.9258 | 7.2313 | 10.8988 |
| C | 5.5359 | 7.3761 | 10.8280 |
| N | 3.5467 | 8.5919 | 10.2221 |
| C | 4.9753 | 8.5153 | 10.2215 |
| C | 5.8045 | 9.5339 | 9.7015 |
| C | 9.7777 | 7.1819 | 9.3737 |
| C | 10.3691 | 7.7444 | 8.2249 |
| C | 10.8988 | 6.9258 | 7.2313 |
| C | 10.8280 | 5.5359 | 7.3761 |
| N | 10.2221 | 3.5467 | 8.5919 |
| C | 10.2215 | 4.9753 | 8.5153 |
| C | 9.7015 | 5.8045 | 9.5339 |
| C | -10.3289 | 8.2430 | 9.2599 |
| C | -8.2430 | 9.2599 | 10.3289 |
| C | -9.2599 | 10.3289 | 8.2430 |
| C | -9.6317 | 9.6317 | 9.6317 |
| C | -9.3737 | 7.1819 | 9.7777 |
| C | -8.2249 | 7.7444 | 10.3691 |
| C | -7.2313 | 6.9258 | 10.8988 |
| C | -7.3761 | 5.5359 | 10.8280 |
| N | -8.5919 | 3.5467 | 10.2221 |
| C | -8.5153 | 4.9753 | 10.2215 |
| C | -9.5339 | 5.8045 | 9.7015 |
| C | -7.1819 | 9.7777 | 9.3737 |
| C | -7.7444 | 10.3691 | 8.2249 |
| C | -6.9258 | 10.8988 | 7.2313 |
| C | -5.5359 | 10.8280 | 7.3761 |
| N | -3.5467 | 10.2221 | 8.5919 |
| C | -4.9753 | 10.2215 | 8.5153 |
| C | -5.8045 | 9.7015 | 9.5339 |
| C | -9.7777 | 9.3737 | 7.1819 |
| C | -10.3691 | 8.2249 | 7.7444 |
| C | -10.8988 | 7.2313 | 6.9258 |
| C | -10.8280 | 7.3761 | 5.5359 |
| N | -10.2221 | 8.5919 | 3.5467 |
| C | -10.2215 | 8.5153 | 4.9753 |
| C | -9.7015 | 9.5339 | 5.8045 |
| C | -2.8951 | 9.2482 | 9.1488 |
| C | -1.4184 | 9.2805 | 9.1931 |
| C | -0.6663 | 10.1245 | 8.3683 |
| C | 0.7310 | 10.0779 | 8.4115 |
| C | 1.4184 | 9.1931 | 9.2805 |
| C | 0.6663 | 8.3683 | 10.1245 |
| C | -0.7310 | 8.4115 | 10.0779 |
| C | 2.8951 | 9.1488 | 9.2482 |
| C | -10.3289 | 9.2599 | -8.2430 |
| C | -8.2430 | 10.3289 | -9.2599 |
| C | -9.2599 | 8.2430 | -10.3289 |
| C | -9.6317 | 9.6317 | -9.6317 |
| C | -9.3737 | 9.7777 | -7.1819 |
| C | -8.2249 | 10.3691 | -7.7444 |
| C | -7.2313 | 10.8988 | -6.9258 |
| C | -7.3761 | 10.8280 | -5.5359 |
| N | -8.5919 | 10.2221 | -3.5467 |
| C | -8.5153 | 10.2215 | -4.9753 |
| C | -9.5339 | 9.7015 | -5.8045 |
| C | -7.1819 | 9.3737 | -9.7777 |
| C | -7.7444 | 8.2249 | -10.3691 |
| C | -6.9258 | 7.2313 | -10.8988 |
| C | -5.5359 | 7.3761 | -10.8280 |
| N | -3.5467 | 8.5919 | -10.2221 |
| C | -4.9753 | 8.5153 | -10.2215 |
| C | -5.8045 | 9.5339 | -9.7015 |
| C | -9.7777 | 7.1819 | -9.3737 |
| C | -10.3691 | 7.7444 | -8.2249 |
| C | -10.8988 | 6.9258 | -7.2313 |
| C | -10.8280 | 5.5359 | -7.3761 |
| N | -10.2221 | 3.5467 | -8.5919 |
| C | -10.2215 | 4.9753 | -8.5153 |
| C | -9.7015 | 5.8045 | -9.5339 |
| C | -9.1488 | 9.2482 | -2.8951 |
| C | -9.1931 | 9.2805 | -1.4184 |
| C | -8.3683 | 10.1245 | -0.6663 |
| C | -8.4115 | 10.0779 | 0.7310 |
| C | -9.2805 | 9.1931 | 1.4184 |
| C | -10.1245 | 8.3683 | 0.6663 |
| C | -10.0779 | 8.4115 | -0.7310 |
| C | 2.8951 | 9.2482 | -9.1488 |
| C | 1.4184 | 9.2805 | -9.1931 |
| C | 0.6663 | 10.1245 | -8.3683 |
| C | -0.7310 | 10.0779 | -8.4115 |
| C | -1.4184 | 9.1931 | -9.2805 |
| C | -0.6663 | 8.3683 | -10.1245 |
| C | 0.7310 | 8.4115 | -10.0779 |
| C | 9.1931 | 9.2805 | 1.4184 |
| C | -2.8951 | 9.1488 | -9.2482 |
| C | -9.2482 | 9.1488 | 2.8951 |
| C | 9.1488 | 9.2482 | 2.8951 |
| C | -10.3289 | -9.2599 | 8.2430 |
| C | -8.2430 | -10.3289 | 9.2599 |
| C | -9.2599 | -8.2430 | 10.3289 |
| C | -9.6317 | -9.6317 | 9.6317 |
| C | -9.3737 | -9.7777 | 7.1819 |
| C | -8.2249 | -10.3691 | 7.7444 |
| C | -7.2313 | -10.8988 | 6.9258 |
| C | -7.3761 | -10.8280 | 5.5359 |
| N | -8.5919 | -10.2221 | 3.5467 |
| C | -8.5153 | -10.2215 | 4.9753 |
| C | -9.5339 | -9.7015 | 5.8045 |
| C | -7.1819 | -9.3737 | 9.7777 |
| C | -7.7444 | -8.2249 | 10.3691 |
| C | -6.9258 | -7.2313 | 10.8988 |
| C | -5.5359 | -7.3761 | 10.8280 |
| N | -3.5467 | -8.5919 | 10.2221 |
| C | -4.9753 | -8.5153 | 10.2215 |
| C | -5.8045 | -9.5339 | 9.7015 |
| C | -9.7777 | -7.1819 | 9.3737 |
| C | -10.3691 | -7.7444 | 8.2249 |
| C | -10.8988 | -6.9258 | 7.2313 |
| C | -10.8280 | -5.5359 | 7.3761 |
| N | -10.2221 | -3.5467 | 8.5919 |
| C | -10.2215 | -4.9753 | 8.5153 |
| C | -9.7015 | -5.8045 | 9.5339 |
| C | -9.2482 | -2.8951 | 9.1488 |
| C | -9.2805 | -1.4184 | 9.1931 |
| C | 2.8951 | -9.2482 | 9.1488 |
| C | 1.4184 | -9.2805 | 9.1931 |
| C | 9.1488 | -2.8951 | 9.2482 |
| C | 9.1931 | -1.4184 | 9.2805 |
| C | 0.6663 | -10.1245 | 8.3683 |
| C | -0.7310 | -10.0779 | 8.4115 |
| C | -1.4184 | -9.1931 | 9.2805 |
| C | -0.6663 | -8.3683 | 10.1245 |
| C | 0.7310 | -8.4115 | 10.0779 |
| C | -2.8951 | -9.1488 | 9.2482 |
| C | 10.3289 | -9.2599 | -8.2430 |
| C | 8.2430 | -10.3289 | -9.2599 |
| C | 9.2599 | -8.2430 | -10.3289 |
| C | 9.6317 | -9.6317 | -9.6317 |
| C | 9.3737 | -9.7777 | -7.1819 |
| C | 8.2249 | -10.3691 | -7.7444 |
| C | 7.2313 | -10.8988 | -6.9258 |
| C | 7.3761 | -10.8280 | -5.5359 |
| N | 8.5919 | -10.2221 | -3.5467 |
| C | 8.5153 | -10.2215 | -4.9753 |
| C | 9.5339 | -9.7015 | -5.8045 |
| C | 7.1819 | -9.3737 | -9.7777 |
| C | 7.7444 | -8.2249 | -10.3691 |
| C | 6.9258 | -7.2313 | -10.8988 |
| C | 5.5359 | -7.3761 | -10.8280 |
| N | 3.5467 | -8.5919 | -10.2221 |
| C | 4.9753 | -8.5153 | -10.2215 |
| C | 5.8045 | -9.5339 | -9.7015 |
| C | 9.7777 | -7.1819 | -9.3737 |
| C | 10.3691 | -7.7444 | -8.2249 |
| C | 10.8988 | -6.9258 | -7.2313 |
| C | 10.8280 | -5.5359 | -7.3761 |
| N | 10.2221 | -3.5467 | -8.5919 |
| C | 10.2215 | -4.9753 | -8.5153 |
| C | 9.7015 | -5.8045 | -9.5339 |
| C | 9.1488 | -9.2482 | -2.8951 |
| C | 9.2482 | -2.8951 | -9.1488 |
| C | 9.1931 | -9.2805 | -1.4184 |
| C | 9.2805 | -1.4184 | -9.1931 |
| C | 8.3683 | -10.1245 | -0.6663 |
| C | 8.4115 | -10.0779 | 0.7310 |
| C | 9.2805 | -9.1931 | 1.4184 |
| C | 10.1245 | -8.3683 | 0.6663 |
| C | 10.0779 | -8.4115 | -0.7310 |
| C | 10.1245 | -0.6663 | -8.3683 |
| C | 10.0779 | 0.7310 | -8.4115 |
| C | 9.1931 | 1.4184 | -9.2805 |
| C | 8.3683 | 0.6663 | -10.1245 |
| C | 8.4115 | -0.7310 | -10.0779 |
| C | -10.3289 | -8.2430 | -9.2599 |
| C | -8.2430 | -9.2599 | -10.3289 |
| C | -9.2599 | -10.3289 | -8.2430 |
| C | -9.6317 | -9.6317 | -9.6317 |
| C | -9.3737 | -7.1819 | -9.7777 |
| C | -8.2249 | -7.7444 | -10.3691 |
| C | -7.2313 | -6.9258 | -10.8988 |
| C | -7.3761 | -5.5359 | -10.8280 |
| N | -8.5919 | -3.5467 | -10.2221 |
| C | -8.5153 | -4.9753 | -10.2215 |
| C | -9.5339 | -5.8045 | -9.7015 |
| C | -7.1819 | -9.7777 | -9.3737 |
| C | -7.7444 | -10.3691 | -8.2249 |
| C | -6.9258 | -10.8988 | -7.2313 |
| C | -5.5359 | -10.8280 | -7.3761 |
| N | -3.5467 | -10.2221 | -8.5919 |
| C | -4.9753 | -10.2215 | -8.5153 |
| C | -5.8045 | -9.7015 | -9.5339 |
| C | -9.7777 | -9.3737 | -7.1819 |
| C | -10.3691 | -8.2249 | -7.7444 |
| C | -10.8988 | -7.2313 | -6.9258 |
| C | -10.8280 | -7.3761 | -5.5359 |
| N | -10.2221 | -8.5919 | -3.5467 |
| C | -10.2215 | -8.5153 | -4.9753 |
| C | -9.7015 | -9.5339 | -5.8045 |
| C | -1.4184 | -9.2805 | -9.1931 |
| C | -9.1488 | -2.8951 | -9.2482 |
| C | -9.2482 | -9.1488 | -2.8951 |
| C | -9.1931 | -1.4184 | -9.2805 |
| C | -9.2805 | -9.1931 | -1.4184 |
| C | -8.3683 | -0.6663 | -10.1245 |
| C | -8.4115 | 0.7310 | -10.0779 |
| C | -9.2805 | 1.4184 | -9.1931 |
| C | -10.1245 | 0.6663 | -8.3683 |
| C | -10.0779 | -0.7310 | -8.4115 |
| C | -0.6663 | -10.1245 | -8.3683 |
| C | 0.7310 | -10.0779 | -8.4115 |
| C | 1.4184 | -9.1931 | -9.2805 |
| C | 0.6663 | -8.3683 | -10.1245 |
| C | -0.7310 | -8.4115 | -10.0779 |
| C | -10.1245 | -8.3683 | -0.6663 |
| C | -10.0779 | -8.4115 | 0.7310 |
| C | -9.1931 | -9.2805 | 1.4184 |
| C | -8.3683 | -10.1245 | 0.6663 |
| C | -8.4115 | -10.0779 | -0.7310 |
| C | 2.8951 | -9.1488 | -9.2482 |
| C | 9.2482 | -9.1488 | 2.8951 |
| C | -9.1488 | -9.2482 | 2.8951 |
| C | 8.3683 | -0.6663 | 10.1245 |
| C | 8.4115 | 0.7310 | 10.0779 |
| C | 9.2805 | 1.4184 | 9.1931 |
| C | 10.1245 | 0.6663 | 8.3683 |
| C | 10.0779 | -0.7310 | 8.4115 |
| C | -8.4115 | -0.7310 | 10.0779 |
| C | -8.3683 | 0.6663 | 10.1245 |
| C | -9.1931 | 1.4184 | 9.2805 |
| C | -10.0779 | 0.7310 | 8.4115 |
| C | -10.1245 | -0.6663 | 8.3683 |
| C | -9.2482 | 2.8951 | -9.1488 |
| C | -9.1488 | 2.8951 | 9.2482 |
| C | 9.1488 | 2.8951 | -9.2482 |
| C | 9.2482 | 2.8951 | 9.1488 |
| C | 8.3683 | 10.1245 | 0.6663 |
| C | 8.4115 | 10.0779 | -0.7310 |
| C | 9.2805 | 9.1931 | -1.4184 |
| C | 10.1245 | 8.3683 | -0.6663 |
| C | 10.0779 | 8.4115 | 0.7310 |
| C | 9.2482 | 9.1488 | -2.8951 |
| C | 10.3289 | -8.2430 | 9.2599 |
| C | 8.2430 | -9.2599 | 10.3289 |
| C | 9.2599 | -10.3289 | 8.2430 |
| C | 9.6317 | -9.6317 | 9.6317 |
| C | 9.3737 | -7.1819 | 9.7777 |
| C | 8.2249 | -7.7444 | 10.3691 |
| C | 7.2313 | -6.9258 | 10.8988 |
| C | 7.3761 | -5.5359 | 10.8280 |
| N | 8.5919 | -3.5467 | 10.2221 |
| C | 8.5153 | -4.9753 | 10.2215 |
| C | 9.5339 | -5.8045 | 9.7015 |
| C | 7.1819 | -9.7777 | 9.3737 |
| C | 7.7444 | -10.3691 | 8.2249 |
| C | 6.9258 | -10.8988 | 7.2313 |
| C | 5.5359 | -10.8280 | 7.3761 |
| N | 3.5467 | -10.2221 | 8.5919 |
| C | 4.9753 | -10.2215 | 8.5153 |
| C | 5.8045 | -9.7015 | 9.5339 |
| C | 9.7777 | -9.3737 | 7.1819 |
| C | 10.3691 | -8.2249 | 7.7444 |
| C | 10.8988 | -7.2313 | 6.9258 |
| C | 10.8280 | -7.3761 | 5.5359 |
| N | 10.2221 | -8.5919 | 3.5467 |
| C | 10.2215 | -8.5153 | 4.9753 |
| C | 9.7015 | -9.5339 | 5.8045 |
| C | -2.8951 | -9.2482 | -9.1488 |
| O | 7.6418 | 1.5729 | 10.8595 |
| O | 10.8595 | -1.5729 | 7.6418 |
| C | 6.6932 | 0.9631 | 11.7682 |
| C | 11.7682 | -0.9631 | 6.6932 |
| O | -7.6418 | 1.5729 | -10.8595 |
| C | -6.6932 | 0.9631 | -11.7682 |
| O | -10.8595 | -1.5729 | -7.6418 |
| C | -11.7682 | -0.9631 | -6.6932 |
| O | -10.8595 | 1.5729 | 7.6418 |
| O | -7.6418 | -1.5729 | 10.8595 |
| C | -11.7682 | 0.9631 | 6.6932 |
| C | -6.6932 | -0.9631 | 11.7682 |
| O | 10.8595 | 1.5729 | -7.6418 |
| O | 7.6418 | -1.5729 | -10.8595 |
| C | 6.6932 | -0.9631 | -11.7682 |
| C | 11.7682 | 0.9631 | -6.6932 |
| O | 1.5729 | -10.8595 | -7.6418 |
| C | 0.9631 | -11.7682 | -6.6932 |
| O | -1.5729 | -7.6418 | -10.8595 |
| C | -0.9631 | -6.6932 | -11.7682 |
| O | 1.5729 | 7.6418 | -10.8595 |
| C | 0.9631 | 6.6932 | -11.7682 |
| O | -1.5729 | 10.8595 | -7.6418 |
| C | -0.9631 | 11.7682 | -6.6932 |
| O | 1.5729 | -7.6418 | 10.8595 |
| O | -1.5729 | -10.8595 | 7.6418 |
| C | 0.9631 | -6.6932 | 11.7682 |
| C | -0.9631 | -11.7682 | 6.6932 |
| O | 1.5729 | 10.8595 | 7.6418 |
| C | 0.9631 | 11.7682 | 6.6932 |
| O | -1.5729 | 7.6418 | 10.8595 |
| C | -0.9631 | 6.6932 | 11.7682 |
| O | -7.6418 | 10.8595 | 1.5729 |
| O | -10.8595 | 7.6418 | -1.5729 |
| C | -11.7682 | 6.6932 | -0.9631 |
| C | -6.6932 | 11.7682 | 0.9631 |
| O | 10.8595 | 7.6418 | 1.5729 |
| O | 7.6418 | 10.8595 | -1.5729 |
| C | 11.7682 | 6.6932 | 0.9631 |
| C | 6.6932 | 11.7682 | -0.9631 |
| O | -10.8595 | -7.6418 | 1.5729 |
| C | -11.7682 | -6.6932 | 0.9631 |
| O | -7.6418 | -10.8595 | -1.5729 |
| C | -6.6932 | -11.7682 | -0.9631 |
| O | 7.6418 | -10.8595 | 1.5729 |
| C | 6.6932 | -11.7682 | 0.9631 |
| O | 10.8595 | -7.6418 | -1.5729 |
| C | 11.7682 | -6.6932 | -0.9631 |
| C | 9.8443 | 11.7264 | -8.0418 |
| C | 11.7264 | 8.0418 | -9.8443 |
| C | 8.0418 | 9.8443 | -11.7264 |
| C | 10.5083 | 10.5083 | -10.5083 |
| H | 11.3615 | 6.3457 | -7.3564 |
| H | 11.2373 | 6.6082 | -4.8806 |
| H | 9.2562 | 10.4259 | -5.3722 |
| H | 6.3457 | 7.3564 | -11.3615 |
| H | 6.6082 | 4.8806 | -11.2373 |
| H | 10.4259 | 5.3722 | -9.2562 |
| H | 7.3564 | 11.3615 | -6.3457 |
| H | 4.8806 | 11.2373 | -6.6082 |
| H | 5.3722 | 9.2562 | -10.4259 |
| C | 11.7264 | 9.8443 | 8.0418 |
| C | 8.0418 | 11.7264 | 9.8443 |
| C | 9.8443 | 8.0418 | 11.7264 |
| C | 10.5083 | 10.5083 | 10.5083 |
| H | 6.3457 | 11.3615 | 7.3564 |
| H | 6.6082 | 11.2373 | 4.8806 |
| H | 10.4259 | 9.2562 | 5.3722 |
| H | 7.3564 | 6.3457 | 11.3615 |
| H | 4.8806 | 6.6082 | 11.2373 |
| H | 5.3722 | 10.4259 | 9.2562 |
| H | 11.3615 | 7.3564 | 6.3457 |
| H | 11.2373 | 4.8806 | 6.6082 |
| H | 9.2562 | 5.3722 | 10.4259 |
| C | -11.7264 | 8.0418 | 9.8443 |
| C | -8.0418 | 9.8443 | 11.7264 |
| C | -9.8443 | 11.7264 | 8.0418 |
| C | -10.5083 | 10.5083 | 10.5083 |
| H | -6.3457 | 7.3564 | 11.3615 |
| H | -6.6082 | 4.8806 | 11.2373 |
| H | -10.4259 | 5.3722 | 9.2562 |
| H | -7.3564 | 11.3615 | 6.3457 |
| H | -4.8806 | 11.2373 | 6.6082 |
| H | -5.3722 | 9.2562 | 10.4259 |
| H | -11.3615 | 6.3457 | 7.3564 |
| H | -11.2373 | 6.6082 | 4.8806 |
| H | -9.2562 | 10.4259 | 5.3722 |
| H | -3.3381 | 8.3440 | 9.6095 |
| H | -1.1951 | 10.8105 | 7.7027 |
| H | 1.1951 | 7.7027 | 10.8105 |
| H | 3.3381 | 9.6095 | 8.3440 |
| C | -11.7264 | 9.8443 | -8.0418 |
| C | -8.0418 | 11.7264 | -9.8443 |
| C | -9.8443 | 8.0418 | -11.7264 |
| C | -10.5083 | 10.5083 | -10.5083 |
| H | -6.3457 | 11.3615 | -7.3564 |
| H | -6.6082 | 11.2373 | -4.8806 |
| H | -10.4259 | 9.2562 | -5.3722 |
| H | -7.3564 | 6.3457 | -11.3615 |
| H | -4.8806 | 6.6082 | -11.2373 |
| H | -5.3722 | 10.4259 | -9.2562 |
| H | -11.3615 | 7.3564 | -6.3457 |
| H | -11.2373 | 4.8806 | -6.6082 |
| H | -9.2562 | 5.3722 | -10.4259 |
| H | -9.6095 | 8.3440 | -3.3381 |
| H | -7.7027 | 10.8105 | -1.1951 |
| H | -10.8105 | 7.7027 | 1.1951 |
| H | 3.3381 | 8.3440 | -9.6095 |
| H | 1.1951 | 10.8105 | -7.7027 |
| H | -1.1951 | 7.7027 | -10.8105 |
| H | -3.3381 | 9.6095 | -8.3440 |
| H | -8.3440 | 9.6095 | 3.3381 |
| H | 9.6095 | 8.3440 | 3.3381 |
| C | -11.7264 | -9.8443 | 8.0418 |
| C | -8.0418 | -11.7264 | 9.8443 |
| C | -9.8443 | -8.0418 | 11.7264 |
| C | -10.5083 | -10.5083 | 10.5083 |
| H | -6.3457 | -11.3615 | 7.3564 |
| H | -6.6082 | -11.2373 | 4.8806 |
| H | -10.4259 | -9.2562 | 5.3722 |
| H | -7.3564 | -6.3457 | 11.3615 |
| H | -4.8806 | -6.6082 | 11.2373 |
| H | -5.3722 | -10.4259 | 9.2562 |
| H | -11.3615 | -7.3564 | 6.3457 |
| H | -11.2373 | -4.8806 | 6.6082 |
| H | -9.2562 | -5.3722 | 10.4259 |
| H | -8.3440 | -3.3381 | 9.6095 |
| H | 3.3381 | -8.3440 | 9.6095 |
| H | 9.6095 | -3.3381 | 8.3440 |
| H | 1.1951 | -10.8105 | 7.7027 |
| H | -1.1951 | -7.7027 | 10.8105 |
| H | -3.3381 | -9.6095 | 8.3440 |
| C | 11.7264 | -9.8443 | -8.0418 |
| C | 8.0418 | -11.7264 | -9.8443 |
| C | 9.8443 | -8.0418 | -11.7264 |
| C | 10.5083 | -10.5083 | -10.5083 |
| H | 6.3457 | -11.3615 | -7.3564 |
| H | 6.6082 | -11.2373 | -4.8806 |
| H | 10.4259 | -9.2562 | -5.3722 |
| H | 7.3564 | -6.3457 | -11.3615 |
| H | 4.8806 | -6.6082 | -11.2373 |
| H | 5.3722 | -10.4259 | -9.2562 |
| H | 11.3615 | -7.3564 | -6.3457 |
| H | 11.2373 | -4.8806 | -6.6082 |
| H | 9.2562 | -5.3722 | -10.4259 |
| H | 9.6095 | -8.3440 | -3.3381 |
| H | 8.3440 | -3.3381 | -9.6095 |
| H | 7.7027 | -10.8105 | -1.1951 |
| H | 10.8105 | -7.7027 | 1.1951 |
| H | 10.8105 | -1.1951 | -7.7027 |
| H | 7.7027 | 1.1951 | -10.8105 |
| C | -11.7264 | -8.0418 | -9.8443 |
| C | -8.0418 | -9.8443 | -11.7264 |
| C | -9.8443 | -11.7264 | -8.0418 |
| C | -10.5083 | -10.5083 | -10.5083 |
| H | -6.3457 | -7.3564 | -11.3615 |
| H | -6.6082 | -4.8806 | -11.2373 |
| H | -10.4259 | -5.3722 | -9.2562 |
| H | -7.3564 | -11.3615 | -6.3457 |
| H | -4.8806 | -11.2373 | -6.6082 |
| H | -5.3722 | -9.2562 | -10.4259 |
| H | -11.3615 | -6.3457 | -7.3564 |
| H | -11.2373 | -6.6082 | -4.8806 |
| H | -9.2562 | -10.4259 | -5.3722 |
| H | -9.6095 | -3.3381 | -8.3440 |
| H | -8.3440 | -9.6095 | -3.3381 |
| H | -7.7027 | -1.1951 | -10.8105 |
| H | -10.8105 | 1.1951 | -7.7027 |
| H | -1.1951 | -10.8105 | -7.7027 |
| H | 1.1951 | -7.7027 | -10.8105 |
| H | -10.8105 | -7.7027 | -1.1951 |
| H | -7.7027 | -10.8105 | 1.1951 |
| H | 3.3381 | -9.6095 | -8.3440 |
| H | 8.3440 | -9.6095 | 3.3381 |
| H | -9.6095 | -8.3440 | 3.3381 |
| H | 7.7027 | -1.1951 | 10.8105 |
| H | 10.8105 | 1.1951 | 7.7027 |
| H | -7.7027 | 1.1951 | 10.8105 |
| H | -10.8105 | -1.1951 | 7.7027 |
| H | -8.3440 | 3.3381 | -9.6095 |
| H | -9.6095 | 3.3381 | 8.3440 |
| H | 9.6095 | 3.3381 | -8.3440 |
| H | 8.3440 | 3.3381 | 9.6095 |
| H | 7.7027 | 10.8105 | 1.1951 |
| H | 10.8105 | 7.7027 | -1.1951 |
| H | 8.3440 | 9.6095 | -3.3381 |
| C | 11.7264 | -8.0418 | 9.8443 |
| C | 8.0418 | -9.8443 | 11.7264 |
| C | 9.8443 | -11.7264 | 8.0418 |
| C | 10.5083 | -10.5083 | 10.5083 |
| H | 6.3457 | -7.3564 | 11.3615 |
| H | 6.6082 | -4.8806 | 11.2373 |
| H | 10.4259 | -5.3722 | 9.2562 |
| H | 7.3564 | -11.3615 | 6.3457 |
| H | 4.8806 | -11.2373 | 6.6082 |
| H | 5.3722 | -9.2562 | 10.4259 |
| H | 11.3615 | -6.3457 | 7.3564 |
| H | 11.2373 | -6.6082 | 4.8806 |
| H | 9.2562 | -10.4259 | 5.3722 |
| H | -3.3381 | -8.3440 | -9.6095 |
| H | 6.2366 | 1.8446 | 12.2363 |
| H | 5.9506 | 0.3839 | 11.2117 |
| H | 7.2166 | 0.3514 | 12.5095 |
| H | 12.2363 | -1.8446 | 6.2366 |
| H | 11.2117 | -0.3839 | 5.9506 |
| H | 12.5095 | -0.3514 | 7.2166 |
| H | -6.2366 | 1.8446 | -12.2363 |
| H | -5.9506 | 0.3839 | -11.2117 |
| H | -7.2166 | 0.3514 | -12.5095 |
| H | -12.2363 | -1.8446 | -6.2366 |
| H | -11.2117 | -0.3839 | -5.9506 |
| H | -12.5095 | -0.3514 | -7.2166 |
| H | -12.2363 | 1.8446 | 6.2366 |
| H | -11.2117 | 0.3839 | 5.9506 |
| H | -12.5095 | 0.3514 | 7.2166 |
| H | -6.2366 | -1.8446 | 12.2363 |
| H | -5.9506 | -0.3839 | 11.2117 |
| H | -7.2166 | -0.3514 | 12.5095 |
| H | 6.2366 | -1.8446 | -12.2363 |
| H | 5.9506 | -0.3839 | -11.2117 |
| H | 7.2166 | -0.3514 | -12.5095 |
| H | 12.2363 | 1.8446 | -6.2366 |
| H | 11.2117 | 0.3839 | -5.9506 |
| H | 12.5095 | 0.3514 | -7.2166 |
| H | 1.8446 | -12.2363 | -6.2366 |
| H | 0.3839 | -11.2117 | -5.9506 |
| H | 0.3514 | -12.5095 | -7.2166 |
| H | -1.8446 | -6.2366 | -12.2363 |
| H | -0.3839 | -5.9506 | -11.2117 |
| H | -0.3514 | -7.2166 | -12.5095 |
| H | 1.8446 | 6.2366 | -12.2363 |
| H | 0.3839 | 5.9506 | -11.2117 |
| H | 0.3514 | 7.2166 | -12.5095 |
| H | -1.8446 | 12.2363 | -6.2366 |
| H | -0.3839 | 11.2117 | -5.9506 |
| H | -0.3514 | 12.5095 | -7.2166 |
| H | 1.8446 | -6.2366 | 12.2363 |
| H | 0.3839 | -5.9506 | 11.2117 |
| H | 0.3514 | -7.2166 | 12.5095 |
| H | -1.8446 | -12.2363 | 6.2366 |
| H | -0.3839 | -11.2117 | 5.9506 |
| H | -0.3514 | -12.5095 | 7.2166 |
| H | 1.8446 | 12.2363 | 6.2366 |
| H | 0.3839 | 11.2117 | 5.9506 |
| H | 0.3514 | 12.5095 | 7.2166 |
| H | -1.8446 | 6.2366 | 12.2363 |
| H | -0.3839 | 5.9506 | 11.2117 |
| H | -0.3514 | 7.2166 | 12.5095 |
| H | -12.2363 | 6.2366 | -1.8446 |
| H | -11.2117 | 5.9506 | -0.3839 |
| H | -12.5095 | 7.2166 | -0.3514 |
| H | -6.2366 | 12.2363 | 1.8446 |
| H | -5.9506 | 11.2117 | 0.3839 |
| H | -7.2166 | 12.5095 | 0.3514 |
| H | 12.2363 | 6.2366 | 1.8446 |
| H | 11.2117 | 5.9506 | 0.3839 |
| H | 12.5095 | 7.2166 | 0.3514 |
| H | 6.2366 | 12.2363 | -1.8446 |
| H | 5.9506 | 11.2117 | -0.3839 |
| H | 7.2166 | 12.5095 | -0.3514 |
| H | -12.2363 | -6.2366 | 1.8446 |
| H | -11.2117 | -5.9506 | 0.3839 |
| H | -12.5095 | -7.2166 | 0.3514 |
| H | -6.2366 | -12.2363 | -1.8446 |
| H | -5.9506 | -11.2117 | -0.3839 |
| H | -7.2166 | -12.5095 | -0.3514 |
| H | 6.2366 | -12.2363 | 1.8446 |
| H | 5.9506 | -11.2117 | 0.3839 |
| H | 7.2166 | -12.5095 | 0.3514 |
| H | 12.2363 | -6.2366 | -1.8446 |
| H | 11.2117 | -5.9506 | -0.3839 |
| H | 12.5095 | -7.2166 | -0.3514 |
| H | -9.4905 | -12.4247 | -8.8092 |
| H | -10.9402 | -11.7132 | -8.0707 |
| H | -9.5534 | -12.1413 | -7.0691 |
| H | 10.7526 | -11.4579 | -10.0157 |
| H | 11.4579 | -10.0157 | -10.7526 |
| H | 10.0157 | -10.7526 | -11.4579 |
| H | 9.4905 | -8.8092 | -12.4247 |
| H | 10.9402 | -8.0707 | -11.7132 |
| H | 9.5534 | -7.0691 | -12.1413 |
| H | 8.8092 | -12.4247 | -9.4905 |
| H | 8.0707 | -11.7132 | -10.9402 |
| H | 7.0691 | -12.1413 | -9.5534 |
| H | 11.7132 | -10.9402 | -8.0707 |
| H | 12.1413 | -9.5534 | -7.0691 |
| H | 12.4247 | -9.4905 | -8.8092 |
| H | -8.8092 | -9.4905 | -12.4247 |
| H | -8.0707 | -10.9402 | -11.7132 |
| H | -7.0691 | -9.5534 | -12.1413 |
| H | -11.7132 | -8.0707 | -10.9402 |
| H | -12.1413 | -7.0691 | -9.5534 |
| H | -12.4247 | -8.8092 | -9.4905 |
| H | -10.7526 | -10.0157 | -11.4579 |
| H | -11.4579 | -10.7526 | -10.0157 |
| H | -10.0157 | -11.4579 | -10.7526 |
| H | 11.7132 | -8.0707 | 10.9402 |
| H | 12.1413 | -7.0691 | 9.5534 |
| H | 12.4247 | -8.8092 | 9.4905 |
| H | 10.7526 | -10.0157 | 11.4579 |
| H | 11.4579 | -10.7526 | 10.0157 |
| H | 10.0157 | -11.4579 | 10.7526 |
| H | 9.4905 | -12.4247 | 8.8092 |
| H | 10.9402 | -11.7132 | 8.0707 |
| H | 9.5534 | -12.1413 | 7.0691 |
| H | 8.8092 | -9.4905 | 12.4247 |
| H | 8.0707 | -10.9402 | 11.7132 |
| H | 7.0691 | -9.5534 | 12.1413 |
| H | -8.8092 | -12.4247 | 9.4905 |
| H | -8.0707 | -11.7132 | 10.9402 |
| H | -7.0691 | -12.1413 | 9.5534 |
| H | -10.7526 | -11.4579 | 10.0157 |
| H | -11.4579 | -10.0157 | 10.7526 |
| H | -10.0157 | -10.7526 | 11.4579 |
| H | -11.7132 | -10.9402 | 8.0707 |
| H | -12.1413 | -9.5534 | 7.0691 |
| H | -12.4247 | -9.4905 | 8.8092 |
| H | -9.4905 | -8.8092 | 12.4247 |
| H | -10.9402 | -8.0707 | 11.7132 |
| H | -9.5534 | -7.0691 | 12.1413 |
| H | 11.7132 | 10.9402 | 8.0707 |
| H | 12.1413 | 9.5534 | 7.0691 |
| H | 12.4247 | 9.4905 | 8.8092 |
| H | 10.7526 | 11.4579 | 10.0157 |
| H | 11.4579 | 10.0157 | 10.7526 |
| H | 10.0157 | 10.7526 | 11.4579 |
| H | 9.4905 | 8.8092 | 12.4247 |
| H | 10.9402 | 8.0707 | 11.7132 |
| H | 9.5534 | 7.0691 | 12.1413 |
| H | 8.8092 | 12.4247 | 9.4905 |
| H | 8.0707 | 11.7132 | 10.9402 |
| H | 7.0691 | 12.1413 | 9.5534 |
| H | -9.4905 | 12.4247 | 8.8092 |
| H | -10.9402 | 11.7132 | 8.0707 |
| H | -9.5534 | 12.1413 | 7.0691 |
| H | -11.7132 | 8.0707 | 10.9402 |
| H | -12.1413 | 7.0691 | 9.5534 |
| H | -12.4247 | 8.8092 | 9.4905 |
| H | -8.8092 | 9.4905 | 12.4247 |
| H | -8.0707 | 10.9402 | 11.7132 |
| H | -7.0691 | 9.5534 | 12.1413 |
| H | -10.7526 | 10.0157 | 11.4579 |
| H | -11.4579 | 10.7526 | 10.0157 |
| H | -10.0157 | 11.4579 | 10.7526 |
| H | 12.4247 | 8.8092 | -9.4905 |
| H | 11.7132 | 8.0707 | -10.9402 |
| H | 12.1413 | 7.0691 | -9.5534 |
| H | 11.4579 | 10.7526 | -10.0157 |
| H | 10.0157 | 11.4579 | -10.7526 |
| H | 10.7526 | 10.0157 | -11.4579 |
| H | 10.9402 | 11.7132 | -8.0707 |
| H | 9.5534 | 12.1413 | -7.0691 |
| H | 9.4905 | 12.4247 | -8.8092 |
| H | 8.8092 | 9.4905 | -12.4247 |
| H | 8.0707 | 10.9402 | -11.7132 |
| H | 7.0691 | 9.5534 | -12.1413 |
| H | -8.8092 | 12.4247 | -9.4905 |
| H | -8.0707 | 11.7132 | -10.9402 |
| H | -7.0691 | 12.1413 | -9.5534 |
| H | -10.7526 | 11.4579 | -10.0157 |
| H | -11.4579 | 10.0157 | -10.7526 |
| H | -10.0157 | 10.7526 | -11.4579 |
| H | -11.7132 | 10.9402 | -8.0707 |
| H | -12.1413 | 9.5534 | -7.0691 |
| H | -12.4247 | 9.4905 | -8.8092 |
| H | -9.4905 | 8.8092 | -12.4247 |
| H | -10.9402 | 8.0707 | -11.7132 |
| H | -9.5534 | 7.0691 | -12.1413 |

e) **OMe-cube** (Type 2 conformer)

| **atom** | **x** | **y** | **z** |
| --- | --- | --- | --- |
| C | 9.5098 | 8.2223 | 10.8169 |
| C | 10.8927 | 9.0154 | 8.8204 |
| C | 8.9059 | 10.4026 | 9.6301 |
| C | 10.1398 | 9.5223 | 10.1350 |
| C | 9.9461 | 7.0707 | 9.9280 |
| C | 10.7148 | 7.5103 | 8.8297 |
| C | 11.2084 | 6.5977 | 7.9038 |
| C | 10.9404 | 5.2331 | 8.0731 |
| N | 9.7599 | 3.4370 | 9.2975 |
| C | 10.1651 | 4.8009 | 9.1626 |
| C | 9.6605 | 5.7243 | 10.1052 |
| C | 10.1229 | 9.6401 | 7.6701 |
| C | 9.0285 | 10.4087 | 8.1196 |
| C | 8.2058 | 11.0644 | 7.2101 |
| C | 8.4657 | 10.9457 | 5.8387 |
| N | 9.9104 | 10.0851 | 4.0116 |
| C | 9.5532 | 10.1743 | 5.3941 |
| C | 10.3995 | 9.5179 | 6.3157 |
| C | 7.6750 | 9.6279 | 10.0670 |
| C | 8.0108 | 8.4282 | 10.7291 |
| C | 7.0151 | 7.5870 | 11.2138 |
| C | 5.6711 | 7.9347 | 11.0266 |
| N | 3.9875 | 9.5655 | 10.1958 |
| C | 5.3394 | 9.1251 | 10.3567 |
| C | 6.3488 | 9.9899 | 9.8773 |
| C | 10.3640 | -8.8615 | 9.0455 |
| C | 8.2471 | -9.5341 | 10.3076 |
| C | 8.9221 | -10.7878 | 8.1866 |
| C | 9.5043 | -10.1227 | 9.5171 |
| C | 9.6247 | -7.6594 | 9.6063 |
| C | 8.4576 | -8.0333 | 10.3052 |
| C | 7.6491 | -7.0681 | 10.8956 |
| C | 7.9984 | -5.7163 | 10.7823 |
| N | 9.6044 | -3.9895 | 10.0020 |
| C | 9.1589 | -5.3469 | 10.0807 |
| C | 9.9894 | -6.3255 | 9.4899 |
| C | 7.0461 | -9.9117 | 9.4583 |
| C | 7.4207 | -10.6106 | 8.2914 |
| C | 6.4560 | -11.0453 | 7.3891 |
| C | 5.1040 | -10.7890 | 7.6515 |
| N | 3.3828 | -9.6865 | 9.0415 |
| C | 4.7364 | -10.0825 | 8.8093 |
| C | 5.7127 | -9.6373 | 9.7281 |
| C | 9.4847 | -9.9444 | 7.0559 |
| C | 10.2836 | -8.8834 | 7.5322 |
| C | 10.8863 | -8.0001 | 6.6432 |
| C | 10.6999 | -8.1765 | 5.2659 |
| N | 9.5783 | -9.3741 | 3.4103 |
| C | 9.8971 | -9.2299 | 4.7959 |
| C | 9.2800 | -10.1277 | 5.6957 |
| C | -10.8927 | -9.0154 | 8.8204 |
| C | -8.9059 | -10.4026 | 9.6301 |
| C | -9.5098 | -8.2223 | 10.8169 |
| C | -10.1398 | -9.5223 | 10.1350 |
| C | -10.1229 | -9.6401 | 7.6701 |
| C | -9.0285 | -10.4087 | 8.1196 |
| C | -8.2058 | -11.0644 | 7.2101 |
| C | -8.4657 | -10.9457 | 5.8387 |
| N | -9.9104 | -10.0851 | 4.0116 |
| C | -9.5532 | -10.1743 | 5.3941 |
| C | -10.3995 | -9.5179 | 6.3157 |
| C | -7.6750 | -9.6279 | 10.0670 |
| C | -8.0108 | -8.4282 | 10.7291 |
| C | -7.0151 | -7.5870 | 11.2138 |
| C | -5.6711 | -7.9347 | 11.0266 |
| N | -3.9875 | -9.5655 | 10.1958 |
| C | -5.3394 | -9.1251 | 10.3567 |
| C | -6.3488 | -9.9899 | 9.8773 |
| C | -9.9461 | -7.0707 | 9.9280 |
| C | -10.7148 | -7.5103 | 8.8297 |
| C | -11.2084 | -6.5977 | 7.9038 |
| C | -10.9404 | -5.2331 | 8.0731 |
| N | -9.7599 | -3.4370 | 9.2975 |
| C | -10.1651 | -4.8009 | 9.1626 |
| C | -9.6605 | -5.7243 | 10.1052 |
| C | -3.0564 | -8.7543 | 9.7992 |
| C | -1.6668 | -9.2365 | 9.6561 |
| C | -0.6400 | -8.3090 | 9.3441 |
| C | 0.6883 | -8.7144 | 9.1800 |
| C | 1.0308 | -10.0640 | 9.3223 |
| C | 0.0066 | -10.9899 | 9.6431 |
| C | -1.3222 | -10.5841 | 9.8091 |
| C | 2.4166 | -10.5441 | 9.1317 |
| C | -10.3640 | 8.8615 | 9.0455 |
| C | -8.2471 | 9.5341 | 10.3076 |
| C | -8.9221 | 10.7878 | 8.1866 |
| C | -9.5043 | 10.1227 | 9.5171 |
| C | -9.6247 | 7.6594 | 9.6063 |
| C | -8.4576 | 8.0333 | 10.3052 |
| C | -7.6491 | 7.0681 | 10.8956 |
| C | -7.9984 | 5.7163 | 10.7823 |
| N | -9.6044 | 3.9895 | 10.0020 |
| C | -9.1589 | 5.3469 | 10.0807 |
| C | -9.9894 | 6.3255 | 9.4899 |
| C | -7.0461 | 9.9117 | 9.4583 |
| C | -7.4207 | 10.6106 | 8.2914 |
| C | -6.4560 | 11.0453 | 7.3891 |
| C | -5.1040 | 10.7890 | 7.6515 |
| N | -3.3828 | 9.6865 | 9.0415 |
| C | -4.7364 | 10.0825 | 8.8093 |
| C | -5.7127 | 9.6373 | 9.7281 |
| C | -9.4847 | 9.9444 | 7.0559 |
| C | -10.2836 | 8.8834 | 7.5322 |
| C | -10.8863 | 8.0001 | 6.6432 |
| C | -10.6999 | 8.1765 | 5.2659 |
| N | -9.5783 | 9.3741 | 3.4103 |
| C | -9.8971 | 9.2299 | 4.7959 |
| C | -9.2800 | 10.1277 | 5.6957 |
| C | -8.7977 | 3.0324 | 9.6633 |
| C | -9.2858 | 1.6383 | 9.6096 |
| C | -8.3718 | 0.5942 | 9.3160 |
| C | -8.7836 | -0.7402 | 9.2404 |
| C | -10.1262 | -1.0716 | 9.4556 |
| C | -11.0388 | -0.0293 | 9.7557 |
| C | -10.6266 | 1.3055 | 9.8333 |
| C | 3.0564 | 8.7543 | 9.7992 |
| C | 1.6668 | 9.2365 | 9.6561 |
| C | 0.6400 | 8.3090 | 9.3441 |
| C | -0.6883 | 8.7144 | 9.1800 |
| C | -1.0308 | 10.0640 | 9.3223 |
| C | -0.0066 | 10.9899 | 9.6431 |
| C | 1.3222 | 10.5841 | 9.8091 |
| C | 9.2858 | -1.6383 | 9.6096 |
| C | -2.4166 | 10.5441 | 9.1317 |
| C | -10.6132 | -2.4649 | 9.3637 |
| C | 8.7977 | -3.0324 | 9.6633 |
| C | -10.3640 | -8.8615 | -9.0455 |
| C | -8.2471 | -9.5341 | -10.3076 |
| C | -8.9221 | -10.7878 | -8.1866 |
| C | -9.5043 | -10.1227 | -9.5171 |
| C | -9.6247 | -7.6594 | -9.6063 |
| C | -8.4576 | -8.0333 | -10.3052 |
| C | -7.6491 | -7.0681 | -10.8956 |
| C | -7.9984 | -5.7163 | -10.7823 |
| N | -9.6044 | -3.9895 | -10.0020 |
| C | -9.1589 | -5.3469 | -10.0807 |
| C | -9.9894 | -6.3255 | -9.4899 |
| C | -7.0461 | -9.9117 | -9.4583 |
| C | -7.4207 | -10.6106 | -8.2914 |
| C | -6.4560 | -11.0453 | -7.3891 |
| C | -5.1040 | -10.7890 | -7.6515 |
| N | -3.3828 | -9.6865 | -9.0415 |
| C | -4.7364 | -10.0825 | -8.8093 |
| C | -5.7127 | -9.6373 | -9.7281 |
| C | -9.4847 | -9.9444 | -7.0559 |
| C | -10.2836 | -8.8834 | -7.5322 |
| C | -10.8863 | -8.0001 | -6.6432 |
| C | -10.6999 | -8.1765 | -5.2659 |
| N | -9.5783 | -9.3741 | -3.4103 |
| C | -9.8971 | -9.2299 | -4.7959 |
| C | -9.2800 | -10.1277 | -5.6957 |
| C | -10.4921 | -9.4453 | -2.4947 |
| C | -10.0949 | -9.5489 | -1.0741 |
| C | 3.0564 | -8.7543 | -9.7992 |
| C | 1.6668 | -9.2365 | -9.6561 |
| C | 9.0309 | -9.7939 | -3.1042 |
| C | 9.4294 | -9.7262 | -1.6827 |
| C | 0.6400 | -8.3090 | -9.3441 |
| C | -0.6883 | -8.7144 | -9.1800 |
| C | -1.0308 | -10.0640 | -9.3223 |
| C | -0.0066 | -10.9899 | -9.6431 |
| C | 1.3222 | -10.5841 | -9.8091 |
| C | -2.4166 | -10.5441 | -9.1317 |
| C | 10.3640 | 8.8615 | -9.0455 |
| C | 8.2471 | 9.5341 | -10.3076 |
| C | 8.9221 | 10.7878 | -8.1866 |
| C | 9.5043 | 10.1227 | -9.5171 |
| C | 9.6247 | 7.6594 | -9.6063 |
| C | 8.4576 | 8.0333 | -10.3052 |
| C | 7.6491 | 7.0681 | -10.8956 |
| C | 7.9984 | 5.7163 | -10.7823 |
| N | 9.6044 | 3.9895 | -10.0020 |
| C | 9.1589 | 5.3469 | -10.0807 |
| C | 9.9894 | 6.3255 | -9.4899 |
| C | 7.0461 | 9.9117 | -9.4583 |
| C | 7.4207 | 10.6106 | -8.2914 |
| C | 6.4560 | 11.0453 | -7.3891 |
| C | 5.1040 | 10.7890 | -7.6515 |
| N | 3.3828 | 9.6865 | -9.0415 |
| C | 4.7364 | 10.0825 | -8.8093 |
| C | 5.7127 | 9.6373 | -9.7281 |
| C | 9.4847 | 9.9444 | -7.0559 |
| C | 10.2836 | 8.8834 | -7.5322 |
| C | 10.8863 | 8.0001 | -6.6432 |
| C | 10.6999 | 8.1765 | -5.2659 |
| N | 9.5783 | 9.3741 | -3.4103 |
| C | 9.8971 | 9.2299 | -4.7959 |
| C | 9.2800 | 10.1277 | -5.6957 |
| C | 8.7977 | 3.0324 | -9.6633 |
| C | 10.4921 | 9.4453 | -2.4947 |
| C | 9.2858 | 1.6383 | -9.6096 |
| C | 10.0949 | 9.5489 | -1.0741 |
| C | 8.3718 | 0.5942 | -9.3160 |
| C | 8.7836 | -0.7402 | -9.2404 |
| C | 10.1262 | -1.0716 | -9.4556 |
| C | 11.0388 | -0.0293 | -9.7557 |
| C | 10.6266 | 1.3055 | -9.8333 |
| C | 11.0789 | 9.8219 | -0.0906 |
| C | 10.7531 | 9.9105 | 1.2669 |
| C | 9.4294 | 9.7262 | 1.6827 |
| C | 8.4440 | 9.4601 | 0.6980 |
| C | 8.7700 | 9.3725 | -0.6594 |
| C | -10.8927 | 9.0154 | -8.8204 |
| C | -8.9059 | 10.4026 | -9.6301 |
| C | -9.5098 | 8.2223 | -10.8169 |
| C | -10.1398 | 9.5223 | -10.1350 |
| C | -10.1229 | 9.6401 | -7.6701 |
| C | -9.0285 | 10.4087 | -8.1196 |
| C | -8.2058 | 11.0644 | -7.2101 |
| C | -8.4657 | 10.9457 | -5.8387 |
| N | -9.9104 | 10.0851 | -4.0116 |
| C | -9.5532 | 10.1743 | -5.3941 |
| C | -10.3995 | 9.5179 | -6.3157 |
| C | -7.6750 | 9.6279 | -10.0670 |
| C | -8.0108 | 8.4282 | -10.7291 |
| C | -7.0151 | 7.5870 | -11.2138 |
| C | -5.6711 | 7.9347 | -11.0266 |
| N | -3.9875 | 9.5655 | -10.1958 |
| C | -5.3394 | 9.1251 | -10.3567 |
| C | -6.3488 | 9.9899 | -9.8773 |
| C | -9.9461 | 7.0707 | -9.9280 |
| C | -10.7148 | 7.5103 | -8.8297 |
| C | -11.2084 | 6.5977 | -7.9038 |
| C | -10.9404 | 5.2331 | -8.0731 |
| N | -9.7599 | 3.4370 | -9.2975 |
| C | -10.1651 | 4.8009 | -9.1626 |
| C | -9.6605 | 5.7243 | -10.1052 |
| C | -1.6668 | 9.2365 | -9.6561 |
| C | -9.0309 | 9.7939 | -3.1042 |
| C | -10.6132 | 2.4649 | -9.3637 |
| C | -9.4294 | 9.7262 | -1.6827 |
| C | -10.1262 | 1.0716 | -9.4556 |
| C | -8.4440 | 9.4601 | -0.6980 |
| C | -8.7700 | 9.3725 | 0.6594 |
| C | -10.0949 | 9.5489 | 1.0741 |
| C | -11.0789 | 9.8219 | 0.0906 |
| C | -10.7531 | 9.9105 | -1.2669 |
| C | -0.6400 | 8.3090 | -9.3441 |
| C | 0.6883 | 8.7144 | -9.1800 |
| C | 1.0308 | 10.0640 | -9.3223 |
| C | 0.0066 | 10.9899 | -9.6431 |
| C | -1.3222 | 10.5841 | -9.8091 |
| C | -11.0388 | 0.0293 | -9.7557 |
| C | -10.6266 | -1.3055 | -9.8333 |
| C | -9.2858 | -1.6383 | -9.6096 |
| C | -8.3718 | -0.5942 | -9.3160 |
| C | -8.7836 | 0.7402 | -9.2404 |
| C | 2.4166 | 10.5441 | -9.1317 |
| C | 10.6132 | -2.4649 | -9.3637 |
| C | -8.7977 | -3.0324 | -9.6633 |
| C | 8.4440 | -9.4601 | -0.6980 |
| C | 8.7700 | -9.3725 | 0.6594 |
| C | 10.0949 | -9.5489 | 1.0741 |
| C | 11.0789 | -9.8219 | 0.0906 |
| C | 10.7531 | -9.9105 | -1.2669 |
| C | -8.7700 | -9.3725 | -0.6594 |
| C | -8.4440 | -9.4601 | 0.6980 |
| C | -9.4294 | -9.7262 | 1.6827 |
| C | -10.7531 | -9.9105 | 1.2669 |
| C | -11.0789 | -9.8219 | -0.0906 |
| C | -10.4921 | 9.4453 | 2.4947 |
| C | -9.0309 | -9.7939 | 3.1042 |
| C | 9.0309 | 9.7939 | 3.1042 |
| C | 10.4921 | -9.4453 | 2.4947 |
| C | 8.3718 | -0.5942 | 9.3160 |
| C | 8.7836 | 0.7402 | 9.2404 |
| C | 10.1262 | 1.0716 | 9.4556 |
| C | 11.0388 | 0.0293 | 9.7557 |
| C | 10.6266 | -1.3055 | 9.8333 |
| C | 10.6132 | 2.4649 | 9.3637 |
| C | 10.8927 | -9.0154 | -8.8204 |
| C | 8.9059 | -10.4026 | -9.6301 |
| C | 9.5098 | -8.2223 | -10.8169 |
| C | 10.1398 | -9.5223 | -10.1350 |
| C | 10.1229 | -9.6401 | -7.6701 |
| C | 9.0285 | -10.4087 | -8.1196 |
| C | 8.2058 | -11.0644 | -7.2101 |
| C | 8.4657 | -10.9457 | -5.8387 |
| N | 9.9104 | -10.0851 | -4.0116 |
| C | 9.5532 | -10.1743 | -5.3941 |
| C | 10.3995 | -9.5179 | -6.3157 |
| C | 7.6750 | -9.6279 | -10.0670 |
| C | 8.0108 | -8.4282 | -10.7291 |
| C | 7.0151 | -7.5870 | -11.2138 |
| C | 5.6711 | -7.9347 | -11.0266 |
| N | 3.9875 | -9.5655 | -10.1958 |
| C | 5.3394 | -9.1251 | -10.3567 |
| C | 6.3488 | -9.9899 | -9.8773 |
| C | 9.9461 | -7.0707 | -9.9280 |
| C | 10.7148 | -7.5103 | -8.8297 |
| C | 11.2084 | -6.5977 | -7.9038 |
| C | 10.9404 | -5.2331 | -8.0731 |
| N | 9.7599 | -3.4370 | -9.2975 |
| C | 10.1651 | -4.8009 | -9.1626 |
| C | 9.6605 | -5.7243 | -10.1052 |
| C | -3.0564 | 8.7543 | -9.7992 |
| O | 7.0742 | 1.0310 | -9.1252 |
| C | 6.0774 | 0.0408 | -8.7729 |
| O | 12.3344 | -0.4690 | -9.9539 |
| C | 13.3411 | 0.5253 | -10.2630 |
| O | -7.0742 | 1.0310 | 9.1252 |
| C | -6.0774 | 0.0408 | 8.7729 |
| O | -12.3344 | -0.4690 | 9.9539 |
| C | -13.3411 | 0.5253 | 10.2630 |
| O | 12.3344 | 0.4690 | 9.9539 |
| C | 13.3411 | -0.5253 | 10.2630 |
| O | 7.0742 | -1.0310 | 9.1252 |
| C | 6.0774 | -0.0408 | 8.7729 |
| O | -7.1716 | -9.3071 | 1.2149 |
| C | -6.1029 | -8.9917 | 0.2891 |
| O | -12.3501 | -9.9828 | -0.6102 |
| C | -13.4252 | -10.2605 | 0.3196 |
| O | 7.1716 | 9.3071 | 1.2149 |
| O | 12.3501 | 9.9828 | -0.6102 |
| C | 13.4252 | 10.2605 | 0.3196 |
| O | 7.1716 | -9.3071 | -1.2149 |
| C | 6.1029 | -8.9917 | -0.2891 |
| O | 12.3501 | -9.9828 | 0.6102 |
| C | 13.4252 | -10.2605 | -0.3196 |
| O | -0.4564 | 12.2914 | 9.7648 |
| C | 0.5178 | 13.3105 | 10.0966 |
| O | 1.0870 | 7.0060 | 9.2272 |
| C | 0.1128 | 5.9901 | 8.8854 |
| O | 0.4564 | -12.2914 | 9.7648 |
| C | -0.5178 | -13.3105 | 10.0966 |
| O | -1.0870 | 7.0060 | -9.2272 |
| C | -0.1128 | 5.9901 | -8.8854 |
| O | 0.4564 | 12.2914 | -9.7648 |
| C | -0.5178 | 13.3105 | -10.0966 |
| O | -0.4564 | -12.2914 | -9.7648 |
| C | 0.5178 | -13.3105 | -10.0966 |
| O | 1.0870 | -7.0060 | -9.2272 |
| C | 0.1128 | -5.9901 | -8.8854 |
| O | -1.0870 | -7.0060 | 9.2272 |
| C | -0.1128 | -5.9901 | 8.8854 |
| O | -7.1716 | 9.3071 | -1.2149 |
| C | -6.1029 | 8.9917 | -0.2891 |
| O | -12.3501 | 9.9828 | 0.6102 |
| C | -13.4252 | 10.2605 | -0.3196 |
| O | -12.3344 | 0.4690 | -9.9539 |
| C | -13.3411 | -0.5253 | -10.2630 |
| O | -7.0742 | -1.0310 | -9.1252 |
| C | -6.0774 | -0.0408 | -8.7729 |
| C | 9.9406 | 7.9753 | 12.2628 |
| C | 12.3734 | 9.3869 | 8.7378 |
| C | 8.8613 | 11.8296 | 10.1769 |
| C | 11.0561 | 10.2873 | 11.0739 |
| C | 11.8204 | -8.8593 | 9.5112 |
| C | 8.0771 | -10.0552 | 11.7350 |
| C | 9.2829 | -12.2600 | 7.9865 |
| C | 10.3127 | -11.1000 | 10.3525 |
| C | -12.3734 | -9.3869 | 8.7378 |
| C | -8.8613 | -11.8296 | 10.1769 |
| C | -9.9406 | -7.9753 | 12.2628 |
| C | -11.0561 | -10.2873 | 11.0739 |
| C | -11.8204 | 8.8593 | 9.5112 |
| C | -8.0771 | 10.0552 | 11.7350 |
| C | -9.2829 | 12.2600 | 7.9865 |
| C | -10.3127 | 11.1000 | 10.3525 |
| C | -11.8204 | -8.8593 | -9.5112 |
| C | -8.0771 | -10.0552 | -11.7350 |
| C | -9.2829 | -12.2600 | -7.9865 |
| C | -10.3127 | -11.1000 | -10.3525 |
| C | 11.8204 | 8.8593 | -9.5112 |
| C | 8.0771 | 10.0552 | -11.7350 |
| C | 9.2829 | 12.2600 | -7.9865 |
| C | 10.3127 | 11.1000 | -10.3525 |
| C | -12.3734 | 9.3869 | -8.7378 |
| C | -8.8613 | 11.8296 | -10.1769 |
| C | -9.9406 | 7.9753 | -12.2628 |
| C | -11.0561 | 10.2873 | -11.0739 |
| C | 12.3734 | -9.3869 | -8.7378 |
| C | 8.8613 | -11.8296 | -10.1769 |
| C | 9.9406 | -7.9753 | -12.2628 |
| C | 11.0561 | -10.2873 | -11.0739 |
| C | 6.1029 | 8.9917 | 0.2891 |
| H | 11.7948 | 6.9329 | 7.0504 |
| H | 11.3137 | 4.5142 | 7.3478 |
| H | 9.0543 | 5.3750 | 10.9378 |
| H | 7.3616 | 11.6598 | 7.5523 |
| H | 7.8303 | 11.4610 | 5.1222 |
| H | 11.2418 | 8.9312 | 5.9564 |
| H | 7.2694 | 6.6617 | 11.7272 |
| H | 4.8881 | 7.2851 | 11.4110 |
| H | 6.0766 | 10.9129 | 9.3702 |
| H | 6.7475 | -7.3517 | 11.4354 |
| H | 7.3746 | -4.9570 | 11.2481 |
| H | 10.8881 | -6.0244 | 8.9566 |
| H | 6.7410 | -11.5761 | 6.4826 |
| H | 4.3442 | -11.1171 | 6.9464 |
| H | 5.4129 | -9.0836 | 10.6147 |
| H | 11.4951 | -7.1743 | 7.0063 |
| H | 11.1585 | -7.4831 | 4.5651 |
| H | 8.6524 | -10.9310 | 5.3167 |
| H | -7.3616 | -11.6598 | 7.5523 |
| H | -7.8303 | -11.4610 | 5.1222 |
| H | -11.2418 | -8.9312 | 5.9564 |
| H | -7.2694 | -6.6617 | 11.7272 |
| H | -4.8881 | -7.2851 | 11.4110 |
| H | 4.3442 | 11.1171 | -6.9464 |
| H | -6.0766 | -10.9129 | 9.3702 |
| H | -11.7948 | -6.9329 | 7.0504 |
| H | -11.3137 | -4.5142 | 7.3478 |
| H | -9.0543 | -5.3750 | 10.9378 |
| H | -3.1951 | -7.6878 | 9.5337 |
| H | 1.4788 | -7.9973 | 8.9434 |
| H | -2.1103 | -11.2987 | 10.0594 |
| H | 2.5153 | -11.6455 | 9.0871 |
| H | -6.7475 | 7.3517 | 11.4354 |
| H | -7.3746 | 4.9570 | 11.2481 |
| H | -10.8881 | 6.0244 | 8.9566 |
| H | -6.7410 | 11.5761 | 6.4826 |
| H | -4.3442 | 11.1171 | 6.9464 |
| H | -5.4129 | 9.0836 | 10.6147 |
| H | -11.4951 | 7.1743 | 7.0063 |
| H | -11.1585 | 7.4831 | 4.5651 |
| H | -8.6524 | 10.9310 | 5.3167 |
| H | -7.7314 | 3.1505 | 9.3870 |
| H | -8.0765 | -1.5437 | 9.0183 |
| H | -11.3309 | 2.1077 | 10.0674 |
| H | 3.1951 | 7.6878 | 9.5337 |
| H | -1.4788 | 7.9973 | 8.9434 |
| H | 2.1103 | 11.2987 | 10.0594 |
| H | -2.5153 | 11.6455 | 9.0871 |
| H | -11.7156 | -2.5635 | 9.3676 |
| H | 7.7314 | -3.1505 | 9.3870 |
| H | -6.7475 | -7.3517 | -11.4354 |
| H | -7.3746 | -4.9570 | -11.2481 |
| H | -10.8881 | -6.0244 | -8.9566 |
| H | -6.7410 | -11.5761 | -6.4826 |
| H | -4.3442 | -11.1171 | -6.9464 |
| H | -5.4129 | -9.0836 | -10.6147 |
| H | -11.4951 | -7.1743 | -7.0063 |
| H | -11.1585 | -7.4831 | -4.5651 |
| H | -8.6524 | -10.9310 | -5.3167 |
| H | -11.5859 | -9.4453 | -2.6634 |
| H | 3.1951 | -7.6878 | -9.5337 |
| H | 7.9615 | -9.5692 | -3.2868 |
| H | -1.4788 | -7.9973 | -8.9434 |
| H | 2.1103 | -11.2987 | -10.0594 |
| H | -2.5153 | -11.6455 | -9.0871 |
| H | 6.7475 | 7.3517 | -11.4354 |
| H | 7.3746 | 4.9570 | -11.2481 |
| H | 10.8881 | 6.0244 | -8.9566 |
| H | 6.7410 | 11.5761 | -6.4826 |
| H | 5.4129 | 9.0836 | -10.6147 |
| H | 11.4951 | 7.1743 | -7.0063 |
| H | 11.1585 | 7.4831 | -4.5651 |
| H | 8.6524 | 10.9310 | -5.3167 |
| H | 7.7314 | 3.1505 | -9.3870 |
| H | 11.5859 | 9.4453 | -2.6634 |
| H | 8.0765 | -1.5437 | -9.0183 |
| H | 11.3309 | 2.1077 | -10.0674 |
| H | 11.5127 | 10.1222 | 2.0237 |
| H | 8.0081 | 9.1698 | -1.4169 |
| H | -7.8303 | 11.4610 | -5.1222 |
| H | -7.3616 | 11.6598 | -7.5523 |
| H | -11.2418 | 8.9312 | -5.9564 |
| H | -7.2694 | 6.6617 | -11.7272 |
| H | -4.8881 | 7.2851 | -11.4110 |
| H | -6.0766 | 10.9129 | -9.3702 |
| H | -11.7948 | 6.9329 | -7.0504 |
| H | -11.3137 | 4.5142 | -7.3478 |
| H | -9.0543 | 5.3750 | -10.9378 |
| H | -7.9615 | 9.5692 | -3.2868 |
| H | -11.7156 | 2.5635 | -9.3676 |
| H | -8.0081 | 9.1698 | 1.4169 |
| H | -11.5127 | 10.1222 | -2.0237 |
| H | 1.4788 | 7.9973 | -8.9434 |
| H | -2.1103 | 11.2987 | -10.0594 |
| H | -11.3309 | -2.1077 | -10.0674 |
| H | -8.0765 | 1.5437 | -9.0183 |
| H | 2.5153 | 11.6455 | -9.0871 |
| H | 11.7156 | -2.5635 | -9.3676 |
| H | -7.7314 | -3.1505 | -9.3870 |
| H | 8.0081 | -9.1698 | 1.4169 |
| H | 11.5127 | -10.1222 | -2.0237 |
| H | -8.0081 | -9.1698 | -1.4169 |
| H | -11.5127 | -10.1222 | 2.0237 |
| H | -11.5859 | 9.4453 | 2.6634 |
| H | -7.9615 | -9.5692 | 3.2868 |
| H | 7.9615 | 9.5692 | 3.2868 |
| H | 11.5859 | -9.4453 | 2.6634 |
| H | 8.0765 | 1.5437 | 9.0183 |
| H | 11.3309 | -2.1077 | 10.0674 |
| H | 11.7156 | 2.5635 | 9.3676 |
| H | 7.3616 | -11.6598 | -7.5523 |
| H | 7.8303 | -11.4610 | -5.1222 |
| H | 11.2418 | -8.9312 | -5.9564 |
| H | 7.2694 | -6.6617 | -11.7272 |
| H | 4.8881 | -7.2851 | -11.4110 |
| H | 6.0766 | -10.9129 | -9.3702 |
| H | 11.7948 | -6.9329 | -7.0504 |
| H | 11.3137 | -4.5142 | -7.3478 |
| H | 9.0543 | -5.3750 | -10.9378 |
| H | -3.1951 | 7.6878 | -9.5337 |
| H | 5.1767 | 0.6599 | -8.6733 |
| H | 5.9661 | -0.6902 | -9.5796 |
| H | 6.3336 | -0.4368 | -7.8224 |
| H | 14.2401 | -0.0955 | -10.3682 |
| H | 13.1005 | 1.0309 | -11.2033 |
| H | 13.4451 | 1.2323 | -9.4347 |
| H | -5.1767 | 0.6599 | 8.6733 |
| H | -5.9661 | -0.6902 | 9.5796 |
| H | -6.3336 | -0.4368 | 7.8224 |
| H | -14.2401 | -0.0955 | 10.3682 |
| H | -13.1005 | 1.0309 | 11.2033 |
| H | -13.4451 | 1.2323 | 9.4347 |
| H | 14.2401 | 0.0955 | 10.3682 |
| H | 13.1005 | -1.0309 | 11.2033 |
| H | 13.4451 | -1.2323 | 9.4347 |
| H | 5.1767 | -0.6599 | 8.6733 |
| H | 5.9661 | 0.6902 | 9.5796 |
| H | 6.3336 | 0.4368 | 7.8224 |
| H | -5.2404 | -8.9193 | 0.9642 |
| H | -5.9718 | -9.8045 | -0.4316 |
| H | -6.2967 | -8.0351 | -0.2055 |
| H | -14.2869 | -10.3395 | -0.3558 |
| H | -13.2447 | -11.2069 | 0.8385 |
| H | -13.5480 | -9.4286 | 1.0193 |
| H | 14.2869 | 10.3395 | -0.3558 |
| H | 13.2447 | 11.2069 | 0.8385 |
| H | 13.5480 | 9.4286 | 1.0193 |
| H | 5.2404 | -8.9193 | -0.9642 |
| H | 5.9718 | -9.8045 | 0.4316 |
| H | 6.2967 | -8.0351 | 0.2055 |
| H | 14.2869 | -10.3395 | 0.3558 |
| H | 13.2447 | -11.2069 | -0.8385 |
| H | 13.5480 | -9.4286 | -1.0193 |
| H | -0.1084 | 14.2113 | 10.1334 |
| H | 0.9670 | 13.1043 | 11.0730 |
| H | 1.2723 | 13.3871 | 9.3083 |
| H | 0.7358 | 5.0871 | 8.8574 |
| H | -0.6509 | 5.9176 | 9.6659 |
| H | -0.3254 | 6.1968 | 7.9044 |
| H | 0.1084 | -14.2113 | 10.1334 |
| H | -0.9670 | -13.1043 | 11.0730 |
| H | -1.2723 | -13.3871 | 9.3083 |
| H | -0.7358 | 5.0871 | -8.8574 |
| H | 0.6509 | 5.9176 | -9.6659 |
| H | 0.3254 | 6.1968 | -7.9044 |
| H | 0.1084 | 14.2113 | -10.1334 |
| H | -0.9670 | 13.1043 | -11.0730 |
| H | -1.2723 | 13.3871 | -9.3083 |
| H | -0.1084 | -14.2113 | -10.1334 |
| H | 0.9670 | -13.1043 | -11.0730 |
| H | 1.2723 | -13.3871 | -9.3083 |
| H | 0.7358 | -5.0871 | -8.8574 |
| H | -0.6509 | -5.9176 | -9.6659 |
| H | -0.3254 | -6.1968 | -7.9044 |
| H | -0.7358 | -5.0871 | 8.8574 |
| H | 0.6509 | -5.9176 | 9.6659 |
| H | 0.3254 | -6.1968 | 7.9044 |
| H | -5.2404 | 8.9193 | -0.9642 |
| H | -5.9718 | 9.8045 | 0.4316 |
| H | -6.2967 | 8.0351 | 0.2055 |
| H | -14.2869 | 10.3395 | 0.3558 |
| H | -13.2447 | 11.2069 | -0.8385 |
| H | -13.5480 | 9.4286 | -1.0193 |
| H | -14.2401 | 0.0955 | -10.3682 |
| H | -13.1005 | -1.0309 | -11.2033 |
| H | -13.4451 | -1.2323 | -9.4347 |
| H | -5.1767 | -0.6599 | -8.6733 |
| H | -5.9661 | 0.6902 | -9.5796 |
| H | -6.3336 | 0.4368 | -7.8224 |
| H | 9.4795 | 7.0638 | 12.6623 |
| H | 11.0262 | 7.8496 | 12.3444 |
| H | 9.6416 | 8.8019 | 12.9177 |
| H | 12.8228 | 9.0141 | 7.8094 |
| H | 12.5171 | 10.4736 | 8.7502 |
| H | 12.9430 | 8.9565 | 9.5692 |
| H | 7.9863 | 12.3709 | 9.7972 |
| H | 8.7954 | 11.8400 | 11.2710 |
| H | 9.7486 | 12.4012 | 9.8814 |
| H | 11.8988 | 9.6700 | 11.4090 |
| H | 11.4774 | 11.1779 | 10.5909 |
| H | 10.5238 | 10.6257 | 11.9717 |
| H | 12.3467 | -7.9647 | 9.1569 |
| H | 11.8932 | -8.8626 | 10.6050 |
| H | 12.3662 | -9.7291 | 9.1287 |
| H | 7.1921 | -9.6175 | 12.2125 |
| H | 7.9490 | -11.1434 | 11.7547 |
| H | 8.9404 | -9.8007 | 12.3608 |
| H | 8.8586 | -12.6470 | 7.0521 |
| H | 10.3679 | -12.4055 | 7.9322 |
| H | 8.8946 | -12.8830 | 8.8003 |
| H | 10.7024 | -10.6274 | 11.2629 |
| H | 9.7084 | -11.9603 | 10.6661 |
| H | 11.1731 | -11.4924 | 9.7963 |
| H | -12.8228 | -9.0141 | 7.8094 |
| H | -12.5171 | -10.4736 | 8.7502 |
| H | -12.9430 | -8.9565 | 9.5692 |
| H | -7.9863 | -12.3709 | 9.7972 |
| H | -8.7954 | -11.8400 | 11.2710 |
| H | -9.7486 | -12.4012 | 9.8814 |
| H | -9.4795 | -7.0638 | 12.6623 |
| H | -11.0262 | -7.8496 | 12.3444 |
| H | -9.6416 | -8.8019 | 12.9177 |
| H | -11.4774 | -11.1779 | 10.5909 |
| H | -10.5238 | -10.6257 | 11.9717 |
| H | -11.8988 | -9.6700 | 11.4090 |
| H | -12.3467 | 7.9647 | 9.1569 |
| H | -11.8932 | 8.8626 | 10.6050 |
| H | -12.3662 | 9.7291 | 9.1287 |
| H | -7.1921 | 9.6175 | 12.2125 |
| H | -7.9490 | 11.1434 | 11.7547 |
| H | -8.9404 | 9.8007 | 12.3608 |
| H | -8.8586 | 12.6470 | 7.0521 |
| H | -10.3679 | 12.4055 | 7.9322 |
| H | -8.8946 | 12.8830 | 8.8003 |
| H | -10.7024 | 10.6274 | 11.2629 |
| H | -9.7084 | 11.9603 | 10.6661 |
| H | -11.1731 | 11.4924 | 9.7963 |
| H | -12.3467 | -7.9647 | -9.1569 |
| H | -11.8932 | -8.8626 | -10.6050 |
| H | -12.3662 | -9.7291 | -9.1287 |
| H | -7.1921 | -9.6175 | -12.2125 |
| H | -7.9490 | -11.1434 | -11.7547 |
| H | -8.9404 | -9.8007 | -12.3608 |
| H | -8.8586 | -12.6470 | -7.0521 |
| H | -10.3679 | -12.4055 | -7.9322 |
| H | -8.8946 | -12.8830 | -8.8003 |
| H | -10.7024 | -10.6274 | -11.2629 |
| H | -9.7084 | -11.9603 | -10.6661 |
| H | -11.1731 | -11.4924 | -9.7963 |
| H | 12.3467 | 7.9647 | -9.1569 |
| H | 11.8932 | 8.8626 | -10.6050 |
| H | 12.3662 | 9.7291 | -9.1287 |
| H | 7.1921 | 9.6175 | -12.2125 |
| H | 7.9490 | 11.1434 | -11.7547 |
| H | 8.9404 | 9.8007 | -12.3608 |
| H | 8.8586 | 12.6470 | -7.0521 |
| H | 10.3679 | 12.4055 | -7.9322 |
| H | 8.8946 | 12.8830 | -8.8003 |
| H | 10.7024 | 10.6274 | -11.2629 |
| H | 9.7084 | 11.9603 | -10.6661 |
| H | 11.1731 | 11.4924 | -9.7963 |
| H | -12.8228 | 9.0141 | -7.8094 |
| H | -12.5171 | 10.4736 | -8.7502 |
| H | -12.9430 | 8.9565 | -9.5692 |
| H | -7.9863 | 12.3709 | -9.7972 |
| H | -8.7954 | 11.8400 | -11.2710 |
| H | -9.7486 | 12.4012 | -9.8814 |
| H | -9.4795 | 7.0638 | -12.6623 |
| H | -11.0262 | 7.8496 | -12.3444 |
| H | -9.6416 | 8.8019 | -12.9177 |
| H | -11.4774 | 11.1779 | -10.5909 |
| H | -10.5238 | 10.6257 | -11.9717 |
| H | -11.8988 | 9.6700 | -11.4090 |
| H | 12.8228 | -9.0141 | -7.8094 |
| H | 12.5171 | -10.4736 | -8.7502 |
| H | 12.9430 | -8.9565 | -9.5692 |
| H | 7.9863 | -12.3709 | -9.7972 |
| H | 8.7954 | -11.8400 | -11.2710 |
| H | 9.7486 | -12.4012 | -9.8814 |
| H | 9.4795 | -7.0638 | -12.6623 |
| H | 11.0262 | -7.8496 | -12.3444 |
| H | 9.6416 | -8.8019 | -12.9177 |
| H | 11.4774 | -11.1779 | -10.5909 |
| H | 10.5238 | -10.6257 | -11.9717 |
| H | 11.8988 | -9.6700 | -11.4090 |
| H | 5.2404 | 8.9193 | 0.9642 |
| H | 5.9718 | 9.8045 | -0.4316 |
| H | 6.2967 | 8.0351 | -0.2055 |

f) **Triply interlocked (OMe-cube)_2_** (Type 1 conformer)

| **atom** | **x** | **y** | **z** |
| --- | --- | --- | --- |
| C | 0.2221 | -8.9652 | -11.8400 |
| C | -1.2363 | -10.7900 | -10.8102 |
| C | -2.2498 | -9.1256 | -12.4638 |
| C | -0.9448 | -10.0034 | -12.1688 |
| C | 0.5107 | -9.1647 | -10.3620 |
| C | -0.2918 | -10.1745 | -9.7952 |
| C | -0.1878 | -10.4819 | -8.4416 |
| C | 0.7032 | -9.7589 | -7.6404 |
| N | 2.3685 | -8.0565 | -7.3031 |
| C | 1.4861 | -8.7340 | -8.2029 |
| C | 1.4083 | -8.4469 | -9.5834 |
| C | -2.6677 | -10.4284 | -10.4581 |
| C | -3.2145 | -9.4859 | -11.3520 |
| C | -4.5112 | -9.0134 | -11.1675 |
| C | -5.2723 | -9.4899 | -10.0934 |
| N | -5.5755 | -10.9692 | -8.1919 |
| C | -4.7240 | -10.4344 | -9.2077 |
| C | -3.4065 | -10.9106 | -9.3861 |
| C | -1.7766 | -7.6859 | -12.3613 |
| C | -0.4033 | -7.6021 | -12.0527 |
| C | 0.2252 | -6.3657 | -11.9466 |
| C | -0.5174 | -5.1964 | -12.1431 |
| N | -2.5928 | -4.0808 | -12.7513 |
| C | -1.8891 | -5.2836 | -12.4378 |
| C | -2.5327 | -6.5360 | -12.5486 |
| C | 10.4079 | -2.9947 | -0.3084 |
| C | 10.6697 | -0.4639 | -0.6673 |
| C | 10.3563 | -1.4000 | 1.7088 |
| C | 11.0823 | -1.6741 | 0.3012 |
| C | 9.5930 | -2.4869 | -1.4875 |
| C | 9.7334 | -1.0959 | -1.6783 |
| C | 8.9951 | -0.4449 | -2.6646 |
| C | 8.1361 | -1.1767 | -3.4900 |
| N | 7.1382 | -3.2582 | -4.2030 |
| C | 7.9915 | -2.5603 | -3.2926 |
| C | 8.7148 | -3.2208 | -2.2767 |
| C | 9.8782 | 0.4728 | 0.2281 |
| C | 9.6800 | -0.0580 | 1.5209 |
| C | 8.9339 | 0.6509 | 2.4589 |
| C | 8.3785 | 1.8885 | 2.1195 |
| N | 8.0429 | 3.7146 | 0.5736 |
| C | 8.5756 | 2.4129 | 0.8297 |
| C | 9.3377 | 1.7040 | -0.1222 |
| C | 9.3514 | -2.5299 | 1.8359 |
| C | 9.4477 | -3.4543 | 0.7720 |
| C | 8.6582 | -4.6005 | 0.7646 |
| C | 7.7376 | -4.8218 | 1.7944 |
| N | 6.6677 | -4.1777 | 3.8642 |
| C | 7.5973 | -3.8705 | 2.8185 |
| C | 8.4075 | -2.7134 | 2.8389 |
| C | 0.0336 | 14.9787 | 0.0815 |
| C | 2.3129 | 13.8195 | 0.1611 |
| C | 1.2841 | 14.4741 | -2.0842 |
| C | 1.4882 | 14.9708 | -0.5819 |
| C | 0.1078 | 13.9295 | 1.1763 |
| C | 1.3680 | 13.3012 | 1.2268 |
| C | 1.6316 | 12.3252 | 2.1837 |
| C | 0.6278 | 11.9550 | 3.0854 |
| N | -1.6555 | 12.0715 | 3.8880 |
| C | -0.6343 | 12.5722 | 3.0218 |
| C | -0.8997 | 13.5771 | 2.0651 |
| C | 2.5594 | 12.7693 | -0.9101 |
| C | 1.9980 | 13.1389 | -2.1488 |
| C | 2.1088 | 12.3043 | -3.2572 |
| C | 2.7879 | 11.0891 | -3.1315 |
| N | 3.9997 | 9.4439 | -1.8431 |
| C | 3.3710 | 10.7301 | -1.9038 |
| C | 3.2542 | 11.5730 | -0.7755 |
| C | -0.2119 | 14.2556 | -2.2213 |
| C | -0.9020 | 14.5306 | -1.0242 |
| C | -2.2818 | 14.3601 | -0.9526 |
| C | -2.9752 | 13.8814 | -2.0710 |
| N | -3.0493 | 13.0699 | -4.3496 |
| C | -2.2779 | 13.5750 | -3.2529 |
| C | -0.8849 | 13.7906 | -3.3426 |
| C | 5.1312 | 9.2796 | -1.2332 |
| C | 5.7205 | 7.9276 | -1.1147 |
| C | 5.5622 | 6.9346 | -2.0859 |
| C | 6.1368 | 5.6726 | -1.8889 |
| C | 6.8739 | 5.3638 | -0.7158 |
| C | 7.0474 | 6.3670 | 0.2445 |
| C | 6.4784 | 7.6274 | 0.0440 |
| C | 7.4123 | 3.9998 | -0.5229 |
| C | -9.0010 | 8.4155 | -13.5409 |
| C | -7.8231 | 6.3913 | -14.5555 |
| C | -10.3474 | 6.3351 | -14.1731 |
| C | -9.1485 | 7.2773 | -14.6525 |
| C | -7.6340 | 8.1763 | -12.9221 |
| C | -6.9900 | 7.0524 | -13.4768 |
| C | -5.7419 | 6.6492 | -13.0103 |
| C | -5.1348 | 7.3682 | -11.9769 |
| N | -5.0478 | 9.2025 | -10.4204 |
| C | -5.7749 | 8.4938 | -11.4272 |
| C | -7.0351 | 8.9121 | -11.9068 |
| C | -8.3034 | 5.0301 | -14.0863 |
| C | -9.6998 | 4.9913 | -13.9015 |
| C | -10.3224 | 3.8182 | -13.4841 |
| C | -9.5459 | 2.6847 | -13.2166 |
| N | -7.4094 | 1.5853 | -12.9579 |
| C | -8.1491 | 2.7355 | -13.3740 |
| C | -7.5186 | 3.9147 | -13.8283 |
| C | -10.8242 | 6.9556 | -12.8709 |
| C | -10.0895 | 8.1088 | -12.5306 |
| C | -10.3833 | 8.8146 | -11.3670 |
| C | -11.3991 | 8.3542 | -10.5221 |
| N | -13.1381 | 6.7885 | -9.9344 |
| C | -12.1099 | 7.1845 | -10.8476 |
| C | -11.8364 | 6.4856 | -12.0440 |
| C | -5.6273 | 9.6722 | -9.3600 |
| C | -4.8194 | 10.3575 | -8.3293 |
| C | -3.4275 | 10.2190 | -8.2671 |
| C | -2.7128 | 10.8256 | -7.2301 |
| C | -3.3649 | 11.5884 | -6.2268 |
| C | -4.7507 | 11.7566 | -6.3091 |
| C | -5.4659 | 11.1472 | -7.3464 |
| C | -3.4185 | -3.5183 | -11.9296 |
| C | -4.1140 | -2.2772 | -12.3347 |
| C | -3.7147 | -1.5328 | -13.4505 |
| C | -4.4255 | -0.3849 | -13.8131 |
| C | -5.5713 | 0.0376 | -13.0908 |
| C | -5.9442 | -0.6829 | -11.9511 |
| C | -5.2217 | -1.8220 | -11.5752 |
| C | 5.4247 | -4.8412 | -4.7719 |
| C | -6.3325 | 1.2055 | -13.5750 |
| C | -2.5660 | 12.1385 | -5.1136 |
| C | 6.2850 | -4.1440 | -3.7928 |
| C | -12.0771 | 9.6404 | 11.3905 |
| C | -11.3020 | 7.8061 | 12.9916 |
| C | -9.7488 | 9.7550 | 12.4393 |
| C | -11.2716 | 9.3849 | 12.7469 |
| C | -12.5738 | 8.2665 | 10.9739 |
| C | -12.1356 | 7.2556 | 11.8525 |
| C | -12.4787 | 5.9252 | 11.6277 |
| C | -13.2665 | 5.5983 | 10.5193 |
| N | -14.5434 | 6.1766 | 8.5683 |
| C | -13.7012 | 6.6076 | 9.6398 |
| C | -13.3585 | 7.9585 | 9.8696 |
| C | -9.8538 | 7.3673 | 12.8665 |
| C | -8.9950 | 8.4491 | 12.5890 |
| C | -7.6236 | 8.2481 | 12.4561 |
| C | -7.1054 | 6.9547 | 12.5739 |
| N | -7.3842 | 4.5712 | 12.7997 |
| C | -7.9678 | 5.8723 | 12.8315 |
| C | -9.3554 | 6.0767 | 12.9942 |
| C | -9.7471 | 10.1962 | 10.9855 |
| C | -11.0338 | 10.1446 | 10.4127 |
| C | -11.2303 | 10.5208 | 9.0871 |
| C | -10.1361 | 10.9375 | 8.3198 |
| N | -7.7721 | 11.4672 | 8.0958 |
| C | -8.8513 | 10.9637 | 8.8891 |
| C | -8.6479 | 10.5968 | 10.2369 |
| C | -6.7597 | 10.7257 | 7.7755 |
| C | -5.6615 | 11.2907 | 6.9630 |
| C | -5.0482 | -1.5072 | 13.2220 |
| C | -5.7634 | -0.2211 | 13.3460 |
| C | 2.3525 | -4.4759 | 8.7911 |
| C | 3.2695 | -4.1294 | 7.6874 |
| C | -6.5623 | 0.0922 | 14.4526 |
| C | -7.1775 | 1.3450 | 14.5374 |
| C | -7.0127 | 2.3233 | 13.5238 |
| C | -6.2313 | 2.0020 | 12.4092 |
| C | -5.6167 | 0.7476 | 12.3214 |
| C | -7.6554 | 3.6478 | 13.6671 |
| C | -11.9305 | -14.4981 | 2.6411 |
| C | -14.0862 | -13.1353 | 2.4589 |
| C | -13.0182 | -14.1219 | 0.3611 |
| C | -13.3405 | -14.4322 | 1.8951 |
| C | -11.9327 | -13.2961 | 3.5708 |
| C | -13.1112 | -12.5327 | 3.4512 |
| C | -13.2765 | -11.3690 | 4.1971 |
| C | -12.2563 | -10.9575 | 5.0620 |
| N | -10.0995 | -11.2159 | 6.0910 |
| C | -11.0749 | -11.7144 | 5.1717 |
| C | -10.9148 | -12.9065 | 4.4314 |
| C | -14.2622 | -12.2347 | 1.2490 |
| C | -13.6761 | -12.7848 | 0.0912 |
| C | -13.7386 | -12.1078 | -1.1237 |
| C | -14.3882 | -10.8709 | -1.1872 |
| N | -15.5448 | -9.0209 | -0.1705 |
| C | -14.9769 | -10.3245 | -0.0317 |
| C | -14.9161 | -11.0096 | 1.2019 |
| C | -11.5045 | -14.0108 | 0.3008 |
| C | -10.9038 | -14.2770 | 1.5470 |
| C | -9.5179 | -14.2666 | 1.6805 |
| C | -8.7247 | -13.9445 | 0.5746 |
| N | -8.4513 | -13.1753 | -1.6883 |
| C | -9.3272 | -13.6368 | -0.6601 |
| C | -10.7316 | -13.6873 | -0.8068 |
| C | -8.8296 | -11.4228 | 5.9259 |
| C | -8.6812 | -13.3721 | -2.9492 |
| C | -7.8666 | -10.8512 | 6.8908 |
| C | -7.7639 | -12.7892 | -3.9512 |
| C | -8.2362 | -9.8506 | 7.7970 |
| C | -7.2840 | -9.3074 | 8.6661 |
| C | -5.9350 | -9.7456 | 8.6569 |
| C | -5.5704 | -10.7600 | 7.7642 |
| C | -6.5222 | -11.2989 | 6.8931 |
| C | -6.5387 | -12.2156 | -3.5973 |
| C | -5.7138 | -11.6569 | -4.5821 |
| C | -6.0966 | -11.6464 | -5.9470 |
| C | -7.3226 | -12.2247 | -6.3015 |
| C | -8.1389 | -12.7908 | -5.3194 |
| C | -21.4161 | 3.5005 | -2.1927 |
| C | -21.7578 | 0.9759 | -1.9944 |
| C | -21.8074 | 2.4397 | 0.0983 |
| C | -22.2656 | 2.3829 | -1.4311 |
| C | -20.5359 | 2.7238 | -3.1562 |
| C | -20.7124 | 1.3308 | -3.0333 |
| C | -19.9722 | 0.4545 | -3.8218 |
| C | -19.0466 | 0.9685 | -4.7372 |
| N | -17.9225 | 2.7857 | -5.8419 |
| C | -18.8666 | 2.3591 | -4.8554 |
| C | -19.6267 | 3.2494 | -4.0643 |
| C | -21.1266 | 0.2870 | -0.7977 |
| C | -21.1645 | 1.0922 | 0.3586 |
| C | -20.6459 | 0.6239 | 1.5626 |
| C | -20.0828 | -0.6559 | 1.6175 |
| N | -19.5245 | -2.7803 | 0.6005 |
| C | -20.0352 | -1.4520 | 0.4593 |
| C | -20.5617 | -0.9817 | -0.7638 |
| C | -20.7681 | 3.5461 | 0.1478 |
| C | -20.5643 | 4.1404 | -1.1136 |
| C | -19.6511 | 5.1802 | -1.2647 |
| C | -18.9196 | 5.6192 | -0.1555 |
| N | -18.3362 | 5.5294 | 2.1854 |
| C | -19.1050 | 5.0067 | 1.0971 |
| C | -20.0501 | 3.9693 | 1.2586 |
| C | -18.1464 | -4.6438 | -0.0304 |
| C | -17.2960 | 3.9178 | -5.7443 |
| C | -17.8377 | 4.7458 | 3.0913 |
| C | -16.3243 | 4.3130 | -6.7848 |
| C | -17.0366 | 5.3060 | 4.1987 |
| C | -15.7559 | 3.3858 | -7.6647 |
| C | -14.8032 | 3.8050 | -8.5998 |
| C | -14.3946 | 5.1597 | -8.6877 |
| C | -14.9827 | 6.0916 | -7.8245 |
| C | -15.9302 | 5.6716 | -6.8858 |
| C | -18.6389 | -5.4835 | 0.9763 |
| C | -18.1472 | -6.7864 | 1.1053 |
| C | -17.1486 | -7.2911 | 0.2349 |
| C | -16.6652 | -6.4565 | -0.7783 |
| C | -17.1512 | -5.1506 | -0.9039 |
| C | -16.4929 | 6.5947 | 4.1526 |
| C | -15.6977 | 7.0541 | 5.2079 |
| C | -15.4282 | 6.2459 | 6.3411 |
| C | -15.9928 | 4.9668 | 6.3983 |
| C | -16.7824 | 4.5048 | 5.3404 |
| C | -16.6443 | -8.6693 | 0.4191 |
| C | -4.9601 | -9.0965 | 9.5572 |
| C | -14.5448 | 6.7590 | 7.4096 |
| C | 3.7519 | -2.8313 | 7.4858 |
| C | 4.6184 | -2.5674 | 6.4198 |
| C | 5.0297 | -3.5905 | 5.5266 |
| C | 4.5436 | -4.8861 | 5.7247 |
| C | 3.6774 | -5.1514 | 6.7919 |
| C | -4.7283 | 10.4206 | 6.3476 |
| C | -3.6741 | 10.9119 | 5.5677 |
| C | -3.5236 | 12.2895 | 5.3812 |
| C | -4.4563 | 13.1603 | 6.0024 |
| C | -5.5096 | 12.6710 | 6.7797 |
| C | -13.3462 | 5.5382 | -9.6571 |
| C | -2.4420 | 12.8607 | 4.5499 |
| C | -5.2009 | -11.0380 | -6.9521 |
| C | 5.9496 | -3.2510 | 4.4206 |
| C | 5.4603 | -4.5487 | -6.1383 |
| C | 4.6047 | -5.2204 | -7.0210 |
| C | 3.6886 | -6.1956 | -6.5594 |
| C | 3.6577 | -6.4983 | -5.1923 |
| C | 4.5192 | -5.8351 | -4.3144 |
| C | 2.7789 | -6.8455 | -7.5244 |
| C | 0.1155 | -7.0453 | 13.0365 |
| C | -0.9592 | -5.1418 | 14.3583 |
| C | -2.2386 | -7.3213 | 13.9943 |
| C | -0.7885 | -6.7304 | 14.3143 |
| C | 0.4529 | -5.6813 | 12.4585 |
| C | -0.1727 | -4.6346 | 13.1658 |
| C | -0.0250 | -3.3156 | 12.7476 |
| C | 0.7573 | -3.0334 | 11.6209 |
| N | 2.2441 | -3.7069 | 9.8323 |
| C | 1.3954 | -4.0762 | 10.9268 |
| C | 1.2473 | -5.4162 | 11.3516 |
| C | -2.4475 | -4.9098 | 14.1742 |
| C | -3.1557 | -6.1152 | 13.9922 |
| C | -4.5383 | -6.1032 | 13.8334 |
| C | -5.2230 | -4.8816 | 13.8431 |
| N | -5.2430 | -2.4551 | 14.0841 |
| C | -4.5098 | -3.6819 | 14.0023 |
| C | -3.1078 | -3.6888 | 14.1743 |
| C | -2.1139 | -7.9059 | 12.5976 |
| C | -0.8071 | -7.7827 | 12.0847 |
| C | -0.4924 | -8.2829 | 10.8243 |
| C | -1.4942 | -8.8839 | 10.0540 |
| N | -3.7752 | -9.6021 | 9.7082 |
| C | -2.8069 | -8.9684 | 10.5513 |
| C | -3.1216 | -8.4934 | 11.8436 |
| C | -18.6288 | -3.2577 | -0.2059 |
| O | 5.1573 | -1.3307 | 6.1240 |
| O | 3.1484 | -6.3906 | 7.0893 |
| C | 4.8093 | -0.2185 | 6.9850 |
| C | 3.4913 | -7.4998 | 6.2203 |
| O | -14.1754 | 2.9779 | -9.5128 |
| C | -14.4811 | 1.5631 | -9.4568 |
| O | -16.5690 | 6.4995 | -5.9808 |
| C | -16.2080 | 7.9021 | -5.9852 |
| O | -4.2141 | 14.4989 | 5.7532 |
| O | -4.9703 | 9.0819 | 6.5905 |
| C | -5.1197 | 15.4653 | 6.3391 |
| C | -4.0796 | 8.1146 | 5.9793 |
| O | -4.4861 | -11.0754 | -4.3396 |
| O | -9.3647 | -13.3880 | -5.5568 |
| C | -9.8236 | -13.4510 | -6.9289 |
| C | -3.9964 | -11.0770 | -2.9734 |
| O | -18.5549 | -7.6954 | 2.0643 |
| C | -19.6047 | -7.2847 | 2.9738 |
| O | -16.7183 | -4.2379 | -1.8464 |
| C | -15.7839 | -4.6994 | -2.8526 |
| O | -5.5017 | -2.6082 | -10.4787 |
| C | -6.5712 | -2.1799 | -9.5964 |
| O | -4.1098 | 0.4342 | -14.8815 |
| C | -2.8860 | 0.1503 | -15.6055 |
| O | -4.8270 | 0.3320 | 11.2694 |
| O | -7.9824 | 1.7621 | 15.5823 |
| C | -4.5642 | 1.2865 | 10.2085 |
| C | -8.2048 | 0.8318 | 16.6696 |
| O | 6.0573 | 4.6199 | -2.7784 |
| C | 5.2881 | 4.8109 | -3.9906 |
| O | 6.5423 | 8.6789 | 0.9402 |
| C | 7.4548 | 8.5603 | 2.0593 |
| O | -1.3423 | 10.7563 | -7.0649 |
| O | -6.8339 | 11.2399 | -7.5252 |
| C | -7.5919 | 11.9686 | -6.5291 |
| C | -0.5899 | 9.9430 | -7.9979 |
| O | 4.5765 | -6.0510 | -2.9514 |
| O | 4.5486 | -5.0080 | -8.3847 |
| C | 3.7891 | -7.1393 | -2.4083 |
| C | 5.5611 | -4.1517 | -8.9715 |
| O | -15.1063 | 8.3027 | 5.2682 |
| C | -15.2777 | 9.1809 | 4.1294 |
| O | -17.3880 | 3.2627 | 5.2896 |
| C | -17.1172 | 2.3404 | 6.3731 |
| O | -7.5389 | -8.3232 | 9.6005 |
| C | -8.8649 | -7.7391 | 9.6182 |
| O | -6.2550 | -12.2749 | 5.9449 |
| C | -4.9787 | -12.9559 | 6.0340 |
| C | 1.4951 | -9.1347 | -12.6669 |
| C | -1.0482 | -12.3038 | -10.8812 |
| C | -2.8958 | -9.3682 | -13.8291 |
| C | -0.5970 | -10.9313 | -13.3190 |
| H | -0.7995 | -11.2674 | -8.0019 |
| H | 0.7854 | -9.9785 | -6.5766 |
| H | 2.0338 | -7.6767 | -10.0257 |
| H | -4.9366 | -8.2797 | -11.8496 |
| H | -6.2905 | -9.1350 | -9.9407 |
| H | -2.9901 | -11.6472 | -8.7049 |
| H | 1.2835 | -6.3037 | -11.7073 |
| H | -0.0399 | -4.2215 | -12.0729 |
| H | -3.5920 | -6.5888 | -12.7846 |
| C | 11.4482 | -4.0627 | -0.6967 |
| C | 11.8934 | 0.2166 | -1.3132 |
| C | 11.3551 | -1.3783 | 2.8886 |
| C | 12.5911 | -1.8034 | 0.4546 |
| H | 9.0654 | 0.6338 | -2.7928 |
| H | 7.5696 | -0.6802 | -4.2763 |
| H | 8.5744 | -4.2870 | -2.1180 |
| H | 8.7951 | 0.2484 | 3.4665 |
| H | 7.7958 | 2.4500 | 2.8482 |
| H | 9.5221 | 2.1256 | -1.1131 |
| H | 8.7615 | -5.3321 | -0.0432 |
| H | 7.1214 | -5.7189 | 1.7970 |
| H | 8.2917 | -1.9821 | 3.6357 |
| C | -0.4102 | 16.3252 | 0.6532 |
| C | 3.6379 | 14.2624 | 0.7832 |
| C | 1.8008 | 15.4257 | -3.1615 |
| C | 2.1754 | 16.3225 | -0.5004 |
| H | 2.6138 | 11.8513 | 2.2329 |
| H | 0.8167 | 11.1894 | 3.8383 |
| H | -1.8787 | 14.0447 | 2.0132 |
| H | 1.6711 | 12.5863 | -4.2129 |
| H | 2.8713 | 10.4152 | -3.9889 |
| H | 3.6839 | 11.2835 | 0.1822 |
| H | -2.8217 | 14.5804 | -0.0339 |
| H | -4.0531 | 13.7310 | -2.0272 |
| H | -0.3524 | 13.5911 | -4.2689 |
| H | 5.7326 | 10.0719 | -0.7437 |
| H | 4.9811 | 7.1661 | -2.9853 |
| H | 7.6229 | 6.1388 | 1.1463 |
| H | 7.2028 | 3.3031 | -1.3567 |
| C | -9.1213 | 9.8479 | -14.0578 |
| C | -7.0306 | 6.2635 | -15.8564 |
| C | -11.5040 | 6.2005 | -15.1617 |
| C | -9.3722 | 7.8438 | -16.0431 |
| H | -5.2350 | 5.7744 | -13.4299 |
| H | -4.1665 | 7.0523 | -11.5811 |
| H | -7.5215 | 9.7898 | -11.4905 |
| H | -11.4023 | 3.7789 | -13.3544 |
| H | -10.0171 | 1.7623 | -12.8808 |
| H | -6.4391 | 3.9553 | -13.9460 |
| H | -9.8261 | 9.7123 | -11.1069 |
| H | -11.6394 | 8.8920 | -9.6058 |
| H | -12.4081 | 5.6009 | -12.3103 |
| H | -6.7045 | 9.6001 | -9.1158 |
| H | -2.9259 | 9.6319 | -9.0392 |
| H | -5.2486 | 12.3659 | -5.5514 |
| H | -3.6811 | -3.8787 | -10.9159 |
| H | -2.8538 | -1.8766 | -14.0301 |
| H | -6.7985 | -0.3359 | -11.3599 |
| H | -5.9097 | 1.6757 | -14.4849 |
| H | -1.5665 | 11.6694 | -5.0110 |
| H | 6.1099 | -4.4410 | -2.7403 |
| C | -13.2398 | 10.6257 | 11.5027 |
| C | -11.8759 | 7.3709 | 14.3406 |
| C | -9.1519 | 10.8433 | 13.3304 |
| C | -11.8379 | 10.1674 | 13.9184 |
| H | -12.1382 | 5.1420 | 12.3020 |
| H | -13.5468 | 4.5625 | 10.3291 |
| H | -13.7092 | 8.7396 | 9.2010 |
| H | -6.9578 | 9.0836 | 12.2506 |
| H | -6.0378 | 6.7740 | 12.4526 |
| H | -10.0180 | 5.2376 | 13.1861 |
| H | -12.2230 | 10.4889 | 8.6425 |
| H | -10.2752 | 11.2407 | 7.2837 |
| H | -7.6541 | 10.6333 | 10.6732 |
| H | -6.6239 | 9.6599 | 8.0432 |
| H | -4.3627 | -1.5593 | 12.3519 |
| H | 1.8070 | -5.4282 | 8.6314 |
| H | -6.6850 | -0.6594 | 15.2360 |
| H | -6.1080 | 2.7510 | 11.6205 |
| H | -8.3232 | 3.7436 | 14.5429 |
| C | -11.6600 | -15.7883 | 3.4109 |
| C | -15.4327 | -13.4044 | 3.1317 |
| C | -13.5053 | -15.1803 | -0.6296 |
| C | -14.1586 | -15.6986 | 2.0742 |
| H | -14.1834 | -10.7745 | 4.1058 |
| H | -12.3663 | -10.0451 | 5.6482 |
| H | -10.0139 | -13.5053 | 4.5322 |
| H | -13.2821 | -12.5276 | -2.0180 |
| H | -14.4353 | -10.3217 | -2.1269 |
| H | -15.3531 | -10.5753 | 2.0966 |
| H | -9.0509 | -14.4912 | 2.6369 |
| H | -7.6392 | -13.9102 | 0.6645 |
| H | -11.1978 | -13.4464 | -1.7583 |
| H | -8.3604 | -11.9947 | 5.1040 |
| H | -9.5242 | -13.9428 | -3.3816 |
| H | -9.2760 | -9.5138 | 7.8082 |
| H | -4.5339 | -11.1072 | 7.7645 |
| H | -6.2511 | -12.2042 | -2.5420 |
| H | -7.6177 | -12.2161 | -7.3539 |
| C | -22.2414 | 4.5510 | -2.9338 |
| C | -22.8456 | 0.0935 | -2.6075 |
| C | -22.9278 | 2.7146 | 1.1010 |
| C | -23.7629 | 2.5711 | -1.5989 |
| H | -20.1054 | -0.6215 | -3.7309 |
| H | -18.4597 | 0.2965 | -5.3629 |
| H | -19.5055 | 4.3245 | -4.1678 |
| H | -20.6755 | 1.2423 | 2.4576 |
| H | -19.6807 | -1.0405 | 2.5533 |
| H | -20.5346 | -1.6064 | -1.6520 |
| H | -19.4981 | 5.6478 | -2.2351 |
| H | -18.2001 | 6.4308 | -0.2561 |
| H | -20.2128 | 3.5166 | 2.2329 |
| H | -17.4041 | 4.6589 | -4.9290 |
| H | -17.9408 | 3.6432 | 3.1265 |
| H | -16.0753 | 2.3430 | -7.6023 |
| H | -14.6837 | 7.1393 | -7.9058 |
| H | -19.4068 | -5.0945 | 1.6500 |
| H | -15.8983 | -6.8453 | -1.4535 |
| H | -16.7061 | 7.2202 | 3.2829 |
| H | -15.8032 | 4.3509 | 7.2807 |
| H | -17.2743 | -9.3028 | 1.0704 |
| H | -5.3387 | -8.1735 | 10.0400 |
| H | -13.9280 | 7.6265 | 7.1083 |
| H | 3.4416 | -2.0440 | 8.1738 |
| H | 4.8547 | -5.6769 | 5.0391 |
| H | -2.9588 | 10.2382 | 5.0871 |
| H | -6.2339 | 13.3422 | 7.2472 |
| H | -12.7889 | 4.6770 | -10.0750 |
| H | -2.3955 | 13.9665 | 4.5621 |
| H | -4.2417 | -10.6671 | -6.5418 |
| H | 5.9520 | -2.1757 | 4.1538 |
| H | 6.1632 | -3.7901 | -6.4948 |
| H | 2.9524 | -7.2498 | -4.8363 |
| H | 2.5203 | -6.2107 | -8.3925 |
| C | 1.3807 | -7.8526 | 13.3169 |
| C | -0.4675 | -4.4731 | 15.6426 |
| C | -2.7338 | -8.3877 | 14.9710 |
| C | -0.2022 | -7.2883 | 15.5988 |
| H | -0.5142 | -2.5051 | 13.2846 |
| H | 0.8670 | -2.0062 | 11.2771 |
| H | 1.7512 | -6.2228 | 10.8247 |
| H | -5.0902 | -7.0317 | 13.7011 |
| H | -6.3048 | -4.8567 | 13.7274 |
| H | -2.5668 | -2.7571 | 14.3138 |
| H | 0.5195 | -8.2036 | 10.4319 |
| H | -1.2658 | -9.2789 | 9.0650 |
| H | -4.1301 | -8.5882 | 12.2368 |
| H | -18.1547 | -2.7135 | -1.0459 |
| H | 5.3675 | 0.6044 | 6.5188 |
| H | 3.7326 | -0.0300 | 6.9497 |
| H | 5.1626 | -0.4004 | 8.0047 |
| H | 2.9808 | -8.3345 | 6.7167 |
| H | 3.0912 | -7.3298 | 5.2174 |
| H | 4.5783 | -7.6626 | 6.2049 |
| H | -13.8502 | 1.1676 | -10.2630 |
| H | -14.1880 | 1.1479 | -8.4878 |
| H | -15.5414 | 1.3949 | -9.6694 |
| H | -16.8500 | 8.2990 | -5.1884 |
| H | -15.1499 | 8.0241 | -5.7359 |
| H | -16.4549 | 8.3550 | -6.9505 |
| H | -4.6976 | 16.4118 | 5.9776 |
| H | -6.1347 | 15.3162 | 5.9583 |
| H | -5.0815 | 15.4094 | 7.4310 |
| H | -4.5326 | 7.1609 | 6.3015 |
| H | -4.1048 | 8.2051 | 4.8895 |
| H | -3.0661 | 8.2279 | 6.3735 |
| H | -10.7857 | -13.9668 | -6.8184 |
| H | -9.9616 | -12.4422 | -7.3301 |
| H | -9.1291 | -14.0389 | -7.5366 |
| H | -3.0238 | -10.5781 | -3.0784 |
| H | -4.6636 | -10.4983 | -2.3262 |
| H | -3.8678 | -12.1016 | -2.6138 |
| H | -19.7284 | -8.1840 | 3.5905 |
| H | -19.2722 | -6.4357 | 3.5788 |
| H | -20.5213 | -7.0572 | 2.4212 |
| H | -15.5662 | -3.7713 | -3.3964 |
| H | -14.8690 | -5.1054 | -2.3864 |
| H | -16.2672 | -5.4358 | -3.5005 |
| H | -6.4647 | -2.8826 | -8.7610 |
| H | -6.4160 | -1.1434 | -9.2617 |
| H | -7.5336 | -2.3007 | -10.0997 |
| H | -2.8833 | 0.9476 | -16.3587 |
| H | -2.0207 | 0.2388 | -14.9377 |
| H | -2.9414 | -0.8359 | -16.0754 |
| H | -3.8432 | 0.7413 | 9.5883 |
| H | -5.4846 | 1.4991 | 9.6566 |
| H | -4.1141 | 2.2008 | 10.6111 |
| H | -8.8588 | 1.4147 | 17.3305 |
| H | -8.7127 | -0.0661 | 16.3048 |
| H | -7.2589 | 0.5918 | 17.1646 |
| H | 5.3922 | 3.8298 | -4.4739 |
| H | 4.2413 | 5.0170 | -3.7500 |
| H | 5.7341 | 5.5980 | -4.6058 |
| H | 7.3024 | 9.5238 | 2.5667 |
| H | 7.1639 | 7.7260 | 2.7037 |
| H | 8.4830 | 8.4588 | 1.6999 |
| H | -8.6159 | 11.8651 | -6.9101 |
| H | -7.4872 | 11.4953 | -5.5483 |
| H | -7.2835 | 13.0185 | -6.5107 |
| H | 0.4365 | 10.0726 | -7.6273 |
| H | -0.9019 | 8.8971 | -7.9288 |
| H | -0.6896 | 10.3376 | -9.0135 |
| H | 4.0675 | -7.1045 | -1.3466 |
| H | 2.7222 | -6.9382 | -2.5395 |
| H | 4.0879 | -8.0905 | -2.8602 |
| H | 5.3323 | -4.2377 | -10.0465 |
| H | 5.4339 | -3.1236 | -8.6221 |
| H | 6.5617 | -4.5375 | -8.7547 |
| H | -14.7086 | 10.0653 | 4.4434 |
| H | -14.8424 | 8.7311 | 3.2321 |
| H | -16.3361 | 9.4230 | 3.9925 |
| H | -17.6917 | 1.4584 | 6.0624 |
| H | -16.0477 | 2.1153 | 6.4244 |
| H | -17.4954 | 2.7434 | 7.3177 |
| H | -8.7645 | -6.9884 | 10.4132 |
| H | -9.0867 | -7.2657 | 8.6570 |
| H | -9.6091 | -8.4962 | 9.8831 |
| H | -5.0751 | -13.7174 | 5.2499 |
| H | -4.1637 | -12.2599 | 5.8123 |
| H | -4.8599 | -13.4196 | 7.0183 |
| H | -23.6904 | 1.9277 | 1.0782 |
| H | -23.4197 | 3.6739 | 0.9023 |
| H | -22.5397 | 2.7592 | 2.1258 |
| H | -14.3762 | -15.8948 | 3.1316 |
| H | -13.6330 | -16.5791 | 1.6838 |
| H | -15.1206 | -15.6349 | 1.5503 |
| H | -14.5966 | -15.2795 | -0.6086 |
| H | -13.0686 | -16.1628 | -0.4159 |
| H | -13.2236 | -14.9190 | -1.6567 |
| H | -15.3259 | -14.0848 | 3.9847 |
| H | -16.1530 | -13.8440 | 2.4324 |
| H | -15.8758 | -12.4769 | 3.5136 |
| H | -12.4091 | -15.9548 | 4.1939 |
| H | -10.6809 | -15.7577 | 3.9041 |
| H | -11.6601 | -16.6603 | 2.7468 |
| H | -23.3299 | 0.5855 | -3.4590 |
| H | -23.6195 | -0.1609 | -1.8739 |
| H | -22.4279 | -0.8514 | -2.9757 |
| H | -22.8687 | 4.0971 | -3.7099 |
| H | -21.5952 | 5.2848 | -3.4304 |
| H | -22.8929 | 5.1064 | -2.2491 |
| H | -24.0608 | 2.5269 | -2.6540 |
| H | -24.0955 | 3.5412 | -1.2087 |
| H | -24.3296 | 1.7960 | -1.0680 |
| H | 2.0495 | -7.3272 | 14.0087 |
| H | 1.9481 | -8.0338 | 12.3958 |
| H | 1.1446 | -8.8327 | 13.7476 |
| H | 0.7905 | -6.8694 | 15.8066 |
| H | -0.0902 | -8.3789 | 15.5534 |
| H | -0.8385 | -7.0626 | 16.4638 |
| H | -2.8370 | -7.9869 | 15.9859 |
| H | -2.0531 | -9.2462 | 15.0094 |
| H | -3.7173 | -8.7708 | 14.6728 |
| H | 0.6002 | -4.6576 | 15.8089 |
| H | -1.0175 | -4.8319 | 16.5204 |
| H | -0.6040 | -3.3857 | 15.5997 |
| H | -12.9181 | 7.6913 | 14.4555 |
| H | -11.2978 | 7.7817 | 15.1762 |
| H | -11.8614 | 6.2792 | 14.4424 |
| H | -12.8825 | 9.8968 | 14.1179 |
| H | -11.8128 | 11.2486 | 13.7334 |
| H | -11.2720 | 9.9824 | 14.8400 |
| H | -13.9964 | 10.2774 | 12.2154 |
| H | -13.7432 | 10.7553 | 10.5371 |
| H | -12.8975 | 11.6164 | 11.8234 |
| H | -9.1476 | 10.5424 | 14.3843 |
| H | -9.7092 | 11.7836 | 13.2454 |
| H | -8.1137 | 11.0587 | 13.0503 |
| H | 12.2474 | -3.5783 | -1.2976 |
| C | 10.9347 | -5.2703 | -1.4909 |
| H | 11.9319 | -4.4253 | 0.2340 |
| H | 13.0841 | -2.0040 | -0.5076 |
| H | 12.8630 | -2.6227 | 1.1342 |
| H | 13.0395 | -0.8863 | 0.8596 |
| H | 12.0313 | -0.5093 | 2.7476 |
| H | 11.9936 | -2.2853 | 2.8320 |
| C | 10.7612 | -1.3160 | 4.3017 |
| H | 12.5519 | -0.5693 | -1.7408 |
| H | 12.4772 | 0.7116 | -0.5093 |
| C | 11.6114 | 1.2406 | -2.4200 |
| H | 2.8820 | 15.5855 | -3.0725 |
| H | 1.3040 | 16.4013 | -3.1097 |
| H | 1.6167 | 15.0243 | -4.1656 |
| H | 0.2620 | 16.6667 | 1.4486 |
| H | -1.4157 | 16.2579 | 1.0856 |
| H | -0.4439 | 17.0987 | -0.1227 |
| H | 3.4882 | 15.0516 | 1.5285 |
| H | 4.3421 | 14.6321 | 0.0213 |
| H | 4.1259 | 13.4237 | 1.2985 |
| H | 2.3111 | 16.6441 | 0.5396 |
| H | 1.5946 | 17.1023 | -1.0091 |
| H | 3.1671 | 16.3008 | -0.9689 |
| H | -0.0232 | -12.5688 | -11.1657 |
| H | -1.7330 | -12.7616 | -11.6047 |
| H | -1.2451 | -12.7738 | -9.9100 |
| H | 0.2976 | -11.5288 | -13.1037 |
| H | -0.3998 | -10.3731 | -14.2429 |
| H | -1.4126 | -11.6342 | -13.5310 |
| H | 1.9249 | -10.1359 | -12.5423 |
| H | 2.2645 | -8.4153 | -12.3643 |
| H | 1.3039 | -8.9766 | -13.7348 |
| H | -3.2282 | -10.4069 | -13.9382 |
| H | -2.2047 | -9.1414 | -14.6492 |
| H | -3.7779 | -8.7309 | -13.9670 |
| H | -6.6889 | 7.2418 | -16.2145 |
| H | -7.6294 | 5.8004 | -16.6492 |
| H | -6.1395 | 5.6404 | -15.7174 |
| H | -8.5389 | 8.4845 | -16.3578 |
| H | -10.2842 | 8.4523 | -16.0903 |
| H | -9.4734 | 7.0492 | -16.7928 |
| H | -8.3461 | 10.0761 | -14.7984 |
| H | -9.0105 | 10.5733 | -13.2426 |
| H | -10.0992 | 10.0284 | -14.5191 |
| H | -11.1753 | 5.7582 | -16.1091 |
| H | -11.9628 | 7.1723 | -15.3793 |
| H | -12.2952 | 5.5556 | -14.7603 |
| C | 12.9864 | -6.9707 | -12.3925 |
| C | 12.1427 | -9.1709 | -11.4065 |
| C | 10.7170 | -7.9054 | -13.1058 |
| C | 12.2192 | -8.3227 | -12.7595 |
| C | 13.3803 | -7.1352 | -10.9351 |
| C | 12.8907 | -8.3373 | -10.3860 |
| C | 13.1282 | -8.6509 | -9.0505 |
| C | 13.8683 | -7.7665 | -8.2582 |
| N | 15.1695 | -5.7625 | -7.9486 |
| C | 14.3620 | -6.5697 | -8.8095 |
| C | 14.1193 | -6.2479 | -10.1633 |
| C | 10.6613 | -9.2505 | -11.0841 |
| C | 9.8747 | -8.5823 | -12.0433 |
| C | 8.4863 | -8.5740 | -11.9378 |
| C | 7.8772 | -9.2146 | -10.8546 |
| N | 7.9680 | -10.3755 | -8.7504 |
| C | 8.6651 | -9.8637 | -9.8847 |
| C | 10.0722 | -9.8936 | -10.0023 |
| C | 10.6836 | -6.3980 | -12.9163 |
| C | 11.9376 | -5.8837 | -12.5300 |
| C | 12.1021 | -4.5201 | -12.3040 |
| C | 11.0051 | -3.6631 | -12.4463 |
| N | 8.6901 | -3.2280 | -12.9457 |
| C | 9.7479 | -4.1814 | -12.8074 |
| C | 9.5846 | -5.5610 | -13.0618 |
| C | 22.2974 | 0.5459 | -1.0315 |
| C | 22.0277 | 3.0788 | -1.2251 |
| C | 22.2693 | 1.9922 | 1.0748 |
| C | 22.8081 | 1.9343 | -0.4288 |
| C | 21.3166 | 0.9372 | -2.1233 |
| C | 21.1701 | 2.3346 | -2.2301 |
| C | 20.3046 | 2.8827 | -3.1727 |
| C | 19.5654 | 2.0330 | -4.0035 |
| N | 18.9227 | -0.1571 | -4.7851 |
| C | 19.6961 | 0.6377 | -3.8804 |
| C | 20.5931 | 0.0802 | -2.9422 |
| C | 21.1559 | 3.7494 | -0.1780 |
| C | 21.2950 | 3.1535 | 1.0916 |
| C | 20.5728 | 3.6351 | 2.1798 |
| C | 19.6896 | 4.7060 | 1.9998 |
| N | 18.6267 | 6.3868 | 0.6278 |
| C | 19.5338 | 5.2832 | 0.7270 |
| C | 20.2861 | 4.8138 | -0.3730 |
| C | 21.5326 | 0.6784 | 1.2702 |
| C | 21.5619 | -0.1242 | 0.1123 |
| C | 20.9428 | -1.3710 | 0.1009 |
| C | 20.2690 | -1.8131 | 1.2445 |
| N | 19.5165 | -1.5390 | 3.5151 |
| C | 20.2160 | -0.9985 | 2.3905 |
| C | 20.8690 | 0.2538 | 2.4139 |
| C | 8.3508 | 15.8794 | -1.0679 |
| C | 10.8467 | 15.3523 | -0.8771 |
| C | 9.6710 | 15.1927 | -3.1397 |
| C | 9.7551 | 16.0645 | -1.8047 |
| C | 8.6864 | 15.1309 | 0.2103 |
| C | 10.0626 | 14.8441 | 0.3167 |
| C | 10.5547 | 14.1621 | 1.4257 |
| C | 9.6699 | 13.7444 | 2.4280 |
| N | 7.4217 | 13.6136 | 3.3653 |
| C | 8.2960 | 14.0080 | 2.3015 |
| C | 7.7933 | 14.7176 | 1.1892 |
| C | 11.3707 | 14.1985 | -1.7159 |
| C | 10.7272 | 14.1211 | -2.9671 |
| C | 11.0745 | 13.1337 | -3.8850 |
| C | 12.0677 | 12.2089 | -3.5507 |
| N | 13.7218 | 11.2999 | -2.0814 |
| C | 12.7013 | 12.2758 | -2.2947 |
| C | 12.3587 | 13.2848 | -1.3680 |
| C | 8.2828 | 14.5767 | -3.1143 |
| C | 7.5430 | 14.9847 | -1.9869 |
| C | 6.2396 | 14.5337 | -1.7976 |
| C | 5.6816 | 13.6413 | -2.7173 |
| N | 5.8477 | 12.1924 | -4.6343 |
| C | 6.4339 | 13.2117 | -3.8259 |
| C | 7.7420 | 13.6958 | -4.0430 |
| C | 14.0062 | 10.8160 | -0.9126 |
| C | 15.0417 | 9.7653 | -0.8083 |
| C | 15.4808 | 9.0546 | -1.9318 |
| C | 16.4144 | 8.0247 | -1.7854 |
| C | 16.9411 | 7.6753 | -0.5160 |
| C | 16.5256 | 8.4045 | 0.6033 |
| C | 15.5870 | 9.4326 | 0.4568 |
| C | 17.8798 | 6.5390 | -0.4220 |
| C | -1.1508 | 6.4520 | -12.4718 |
| C | 0.2353 | 4.8753 | -13.9254 |
| C | -2.0721 | 4.1509 | -13.1012 |
| C | -1.2518 | 5.3996 | -13.6697 |
| C | 0.3306 | 6.5233 | -12.1437 |
| C | 1.0936 | 5.6492 | -12.9445 |
| C | 2.4736 | 5.5731 | -12.7850 |
| C | 3.1032 | 6.3808 | -11.8297 |
| N | 3.0030 | 8.2061 | -10.2066 |
| C | 2.3386 | 7.2615 | -11.0456 |
| C | 0.9359 | 7.3320 | -11.1906 |
| C | 0.1927 | 3.4040 | -13.5546 |
| C | -1.0843 | 3.0009 | -13.1138 |
| C | -1.3127 | 1.6821 | -12.7314 |
| C | -0.2560 | 0.7637 | -12.7625 |
| N | 2.0483 | 0.1738 | -13.1661 |
| C | 1.0249 | 1.1755 | -13.1722 |
| C | 1.2490 | 2.5045 | -13.5967 |
| C | -2.4172 | 4.5382 | -11.6737 |
| C | -1.9164 | 5.8102 | -11.3321 |
| C | -2.1449 | 6.3414 | -10.0651 |
| C | -2.8746 | 5.5990 | -9.1309 |
| N | -4.0466 | 3.6173 | -8.4334 |
| C | -3.3742 | 4.3283 | -9.4743 |
| C | -3.1439 | 3.7877 | -10.7583 |
| C | 3.2322 | 8.0247 | -8.9474 |
| C | 3.9305 | 9.0871 | -8.1882 |
| C | 4.5258 | 10.1782 | -8.8367 |
| C | 5.1577 | 11.1773 | -8.0925 |
| C | 5.2246 | 11.1154 | -6.6766 |
| C | 4.6560 | 10.0129 | -6.0324 |
| C | 4.0074 | 9.0187 | -6.7762 |
| C | 7.4693 | -3.5102 | -12.6097 |
| C | 6.4144 | -2.4836 | -12.7452 |
| C | 6.7090 | -1.1512 | -13.0597 |
| C | 5.6798 | -0.2080 | -13.1368 |
| C | 4.3259 | -0.5653 | -12.9106 |
| C | 4.0273 | -1.9001 | -12.6202 |
| C | 5.0598 | -2.8403 | -12.5309 |
| C | 17.6128 | -2.0884 | -5.3716 |
| C | 3.2878 | 0.4855 | -12.9429 |
| C | 5.8667 | 12.2186 | -5.9287 |
| C | 18.4131 | -1.2926 | -4.4183 |
| C | -1.4392 | 7.9825 | 11.1748 |
| C | -0.1429 | 6.4569 | 12.7656 |
| C | 0.9371 | 8.5951 | 11.8833 |
| C | -0.4401 | 7.9882 | 12.4210 |
| C | -1.6665 | 6.5109 | 10.8735 |
| C | -0.9433 | 5.6716 | 11.7452 |
| C | -1.0079 | 4.2882 | 11.6082 |
| C | -1.8035 | 3.7353 | 10.5986 |
| N | -3.3042 | 3.9004 | 8.7331 |
| C | -2.5404 | 4.5718 | 9.7392 |
| C | -2.4670 | 5.9767 | 9.8730 |
| C | 1.3459 | 6.2878 | 12.5207 |
| C | 1.9355 | 7.4634 | 12.0148 |
| C | 3.2946 | 7.4982 | 11.7146 |
| C | 4.0774 | 6.3607 | 11.9388 |
| N | 4.3605 | 4.1117 | 12.7869 |
| C | 3.4879 | 5.1925 | 12.4555 |
| C | 2.1062 | 5.1462 | 12.7426 |
| C | 0.6659 | 8.9347 | 10.4288 |
| C | -0.6570 | 8.6306 | 10.0498 |
| C | -1.1060 | 8.9117 | 8.7622 |
| C | -0.2240 | 9.4760 | 7.8341 |
| N | 1.9881 | 10.2125 | 7.1848 |
| C | 1.1023 | 9.7567 | 8.2089 |
| C | 1.5548 | 9.4960 | 9.5212 |
| C | 2.7879 | 11.2170 | 7.3572 |
| C | 3.7104 | 11.6044 | 6.2683 |
| C | 7.8436 | -1.3155 | 13.9024 |
| C | 6.8800 | -0.2602 | 13.5190 |
| C | 15.7561 | -3.0257 | 8.6790 |
| C | 16.5841 | -2.4340 | 7.6080 |
| C | 5.8283 | -0.5084 | 12.6321 |
| C | 4.9512 | 0.5227 | 12.2718 |
| C | 5.1028 | 1.8332 | 12.7880 |
| C | 6.1533 | 2.0800 | 13.6833 |
| C | 7.0240 | 1.0492 | 14.0445 |
| C | 4.1645 | 2.8986 | 12.3756 |
| C | 2.9880 | -15.3996 | 2.1713 |
| C | 0.6134 | -14.4607 | 2.2051 |
| C | 1.6231 | -15.3012 | 0.0132 |
| C | 1.5228 | -15.6153 | 1.5782 |
| C | 2.8604 | -14.1921 | 3.0843 |
| C | 1.5560 | -13.6543 | 3.0762 |
| C | 1.2580 | -12.5226 | 3.8270 |
| C | 2.2590 | -11.9287 | 4.6086 |
| N | 4.5367 | -12.0167 | 5.5491 |
| C | 3.5480 | -12.4849 | 4.6329 |
| C | 3.8626 | -13.6231 | 3.8561 |
| C | 0.1365 | -13.6587 | 1.0088 |
| C | 0.7061 | -14.1132 | -0.1989 |
| C | 0.3754 | -13.5010 | -1.4044 |
| C | -0.5486 | -12.4486 | -1.4134 |
| N | -2.0571 | -10.9211 | -0.3086 |
| C | -1.1357 | -12.0132 | -0.2118 |
| C | -0.7743 | -12.6114 | 1.0172 |
| C | 3.0831 | -14.9503 | -0.2143 |
| C | 3.8409 | -15.0448 | 0.9702 |
| C | 5.2126 | -14.8082 | 0.9560 |
| C | 5.8363 | -14.4606 | -0.2467 |
| N | 5.7812 | -14.0215 | -2.6253 |
| C | 5.0739 | -14.3387 | -1.4219 |
| C | 3.6841 | -14.5910 | -1.4141 |
| C | 5.2311 | -10.9392 | 5.3804 |
| C | 5.3915 | -13.0444 | -3.3839 |
| C | 6.2578 | -10.5838 | 6.3857 |
| C | 6.1365 | -12.7003 | -4.6106 |
| C | 6.6263 | -11.4795 | 7.4001 |
| C | 7.6262 | -11.1328 | 8.3104 |
| C | 8.2958 | -9.8837 | 8.2353 |
| C | 7.9242 | -8.9878 | 7.2296 |
| C | 6.9114 | -9.3301 | 6.3223 |
| C | 7.2947 | -13.3786 | -5.0111 |
| C | 7.9711 | -12.9803 | -6.1678 |
| C | 7.5206 | -11.8895 | -6.9551 |
| C | 6.3582 | -11.2200 | -6.5608 |
| C | 5.6737 | -11.6233 | -5.4078 |
| C | -11.5284 | -0.6670 | -0.3141 |
| C | -11.3300 | -3.2228 | -0.0972 |
| C | -11.1392 | -1.7364 | 1.9899 |
| C | -11.9357 | -1.9169 | 0.6097 |
| C | -10.7111 | -1.2854 | -1.4331 |
| C | -10.7071 | -2.6967 | -1.3762 |
| C | -10.0934 | -3.4364 | -2.3829 |
| C | -9.4278 | -2.7819 | -3.4245 |
| N | -8.6789 | -0.7459 | -4.5179 |
| C | -9.3560 | -1.3796 | -3.4270 |
| C | -10.0041 | -0.6212 | -2.4277 |
| C | -10.2007 | -3.6573 | 0.8258 |
| C | -10.0862 | -2.8256 | 1.9598 |
| C | -9.0466 | -3.0151 | 2.8679 |
| C | -8.1403 | -4.0628 | 2.6809 |
| N | -7.3750 | -6.0105 | 1.4697 |
| C | -8.2614 | -4.8941 | 1.5544 |
| C | -9.2839 | -4.6801 | 0.6066 |
| C | -10.4720 | -0.3793 | 1.8579 |
| C | -10.6777 | 0.2020 | 0.5879 |
| C | -10.1167 | 1.4403 | 0.2877 |
| C | -9.3289 | 2.1006 | 1.2370 |
| N | -8.3285 | 2.2377 | 3.4454 |
| C | -9.1070 | 1.5073 | 2.4907 |
| C | -9.6952 | 0.2653 | 2.8116 |
| C | -5.7233 | -7.4160 | 0.4660 |
| C | -7.7275 | 0.1083 | -4.3012 |
| C | -7.3670 | 1.6584 | 4.0950 |
| C | -7.0514 | 0.7709 | -5.4332 |
| C | -6.5827 | 2.4116 | 5.0956 |
| C | -7.4208 | 0.5537 | -6.7642 |
| C | -6.7422 | 1.2172 | -7.7925 |
| C | -5.6917 | 2.1228 | -7.5333 |
| C | -5.3073 | 2.3338 | -6.2030 |
| C | -5.9797 | 1.6686 | -5.1726 |
| C | -5.8783 | -8.4372 | 1.4050 |
| C | -4.9983 | -9.5283 | 1.4099 |
| C | -3.9372 | -9.6264 | 0.4768 |
| C | -3.7875 | -8.6045 | -0.4730 |
| C | -4.6708 | -7.5227 | -0.4822 |
| C | -6.8125 | 3.7627 | 5.3694 |
| C | -6.0351 | 4.4231 | 6.3288 |
| C | -5.0231 | 3.7511 | 7.0579 |
| C | -4.7825 | 2.4004 | 6.7724 |
| C | -5.5509 | 1.7435 | 5.8082 |
| C | -3.0191 | -10.7778 | 0.5542 |
| C | 9.3317 | -9.5570 | 9.2390 |
| C | -4.2689 | 4.4878 | 8.0914 |
| C | 16.8619 | -1.0644 | 7.5497 |
| C | 17.6069 | -0.5491 | 6.4831 |
| C | 18.0992 | -1.3845 | 5.4492 |
| C | 17.8446 | -2.7592 | 5.5228 |
| C | 17.0958 | -3.2733 | 6.5856 |
| C | 4.6374 | 12.6589 | 6.4781 |
| C | 5.5309 | 13.0591 | 5.4801 |
| C | 5.5259 | 12.4117 | 4.2381 |
| C | 4.5979 | 11.3632 | 4.0273 |
| C | 3.7038 | 10.9620 | 5.0264 |
| C | -5.0418 | 2.8202 | -8.6662 |
| C | 6.4453 | 12.7901 | 3.1454 |
| C | 8.2867 | -11.4805 | -8.1530 |
| C | 18.8328 | -0.7822 | 4.3173 |
| C | 17.0797 | -1.5376 | -6.5427 |
| C | 16.2938 | -2.3262 | -7.3895 |
| C | 16.0220 | -3.6869 | -7.0975 |
| C | 16.5718 | -4.2429 | -5.9373 |
| C | 17.3526 | -3.4526 | -5.0871 |
| C | 15.1582 | -4.4672 | -8.0089 |
| C | 13.7699 | -6.1559 | 12.6889 |
| C | 12.4064 | -4.4887 | 14.0660 |
| C | 11.4607 | -6.8054 | 13.5664 |
| C | 12.8031 | -6.0349 | 13.9547 |
| C | 13.9603 | -4.7251 | 12.2157 |
| C | 13.1871 | -3.8105 | 12.9591 |
| C | 13.2058 | -2.4541 | 12.6466 |
| C | 13.9947 | -2.0045 | 11.5814 |
| N | 15.5615 | -2.3743 | 9.7838 |
| C | 14.7579 | -2.9200 | 10.8348 |
| C | 14.7542 | -4.2945 | 11.1612 |
| C | 10.9107 | -4.4536 | 13.8031 |
| C | 10.3817 | -5.7430 | 13.5971 |
| C | 9.0166 | -5.9278 | 13.3938 |
| C | 8.1763 | -4.8124 | 13.3435 |
| N | 7.8065 | -2.4440 | 13.2678 |
| C | 8.7121 | -3.5195 | 13.5015 |
| C | 10.0888 | -3.3339 | 13.7569 |
| C | 11.6783 | -7.2540 | 12.1300 |
| C | 12.9632 | -6.9231 | 11.6581 |
| C | 13.3429 | -7.2495 | 10.3586 |
| C | 12.4187 | -7.8620 | 9.5051 |
| N | 10.1626 | -8.5919 | 9.0052 |
| C | 11.1217 | -8.1509 | 9.9665 |
| C | 10.7525 | -7.8748 | 11.3017 |
| C | -6.6107 | -6.2307 | 0.4487 |
| O | 17.9364 | 0.7837 | 6.3201 |
| O | 16.7792 | -4.6081 | 6.7590 |
| C | 17.4371 | 1.7232 | 7.3029 |
| C | 17.2129 | -5.5385 | 5.7374 |
| O | -7.0210 | 0.9176 | -9.1262 |
| C | -8.2981 | 1.4227 | -9.5998 |
| O | -5.6941 | 1.8066 | -3.8326 |
| C | -4.6072 | 2.6895 | -3.4561 |
| O | 4.6384 | 10.8213 | 2.7556 |
| O | 4.5607 | 13.2182 | 7.7395 |
| C | 4.0577 | 9.5042 | 2.5675 |
| C | 5.4551 | 14.3163 | 8.0435 |
| O | 9.1228 | -13.5739 | -6.6552 |
| O | 4.5253 | -11.0306 | -4.9305 |
| C | 3.9092 | -9.9996 | -5.7469 |
| C | 9.6443 | -14.7151 | -5.9327 |
| O | -5.0618 | -10.5768 | 2.3067 |
| C | -6.1533 | -10.5734 | 3.2625 |
| O | -4.6200 | -6.4633 | -1.3667 |
| C | -3.6363 | -6.5188 | -2.4285 |
| O | 4.8827 | -4.1729 | -12.1996 |
| C | 3.5239 | -4.6725 | -12.1287 |
| O | 5.8534 | 1.1318 | -13.4296 |
| C | 7.2074 | 1.6022 | -13.6387 |
| O | 8.0911 | 1.1814 | 14.9157 |
| O | 3.8980 | 0.3760 | 11.3919 |
| C | 8.3089 | 2.4818 | 15.5143 |
| C | 3.5932 | -0.9665 | 10.9279 |
| O | 16.9145 | 7.2569 | -2.8213 |
| C | 16.3866 | 7.4967 | -4.1485 |
| O | 15.0989 | 10.2118 | 1.4895 |
| C | 15.5722 | 9.9241 | 2.8277 |
| O | 5.7672 | 12.2968 | -8.6275 |
| O | 3.4031 | 7.9044 | -6.2263 |
| C | 3.1865 | 7.8977 | -4.7920 |
| C | 5.6968 | 12.4783 | -10.0629 |
| O | 17.9391 | -3.8939 | -3.9151 |
| O | 15.7168 | -1.8898 | -8.5680 |
| C | 17.7158 | -5.2725 | -3.5312 |
| C | 15.8928 | -0.4974 | -8.9249 |
| O | -6.1384 | 5.7663 | 6.6374 |
| C | -7.2691 | 6.5034 | 6.1075 |
| O | -5.4096 | 0.4157 | 5.4553 |
| C | -4.3542 | -0.3451 | 6.0920 |
| O | 8.0673 | -11.9335 | 9.3478 |
| C | 7.4207 | -13.2173 | 9.5253 |
| O | 6.4391 | -8.4941 | 5.3283 |
| C | 7.2030 | -7.2910 | 5.0408 |
| C | 14.2065 | -6.6610 | -13.2582 |
| C | 12.7415 | -10.5763 | -11.4905 |
| C | 10.2458 | -8.2799 | -14.5101 |
| C | 12.8886 | -9.0865 | -13.8883 |
| H | 12.7431 | -9.5730 | -8.6196 |
| H | 14.0661 | -7.9987 | -7.2123 |
| H | 14.5121 | -5.3303 | -10.5924 |
| H | 7.8761 | -8.0673 | -12.6824 |
| H | 6.7931 | -9.2036 | -10.7449 |
| H | 10.6790 | -10.3866 | -9.2481 |
| H | 13.0703 | -4.1190 | -12.0114 |
| H | 11.1158 | -2.5937 | -12.2705 |
| H | 8.6199 | -5.9578 | -13.3657 |
| C | 23.3923 | -0.3628 | -1.5877 |
| C | 22.9179 | 4.1055 | -1.9241 |
| C | 23.3469 | 2.1675 | 2.1439 |
| C | 24.3160 | 2.0888 | -0.5140 |
| H | 20.1948 | 3.9614 | -3.2642 |
| H | 18.8826 | 2.4454 | -4.7452 |
| H | 20.7157 | -0.9970 | -2.8668 |
| H | 20.6850 | 3.1844 | 3.1638 |
| H | 19.1156 | 5.0938 | 2.8403 |
| H | 20.1879 | 5.2807 | -1.3495 |
| H | 20.9738 | -1.9981 | -0.7877 |
| H | 19.7774 | -2.7851 | 1.2531 |
| H | 20.8539 | 0.8691 | 3.3095 |
| C | 7.6026 | 17.1746 | -0.7576 |
| C | 11.9977 | 16.2510 | -0.4253 |
| C | 9.8854 | 15.9713 | -4.4376 |
| C | 10.0851 | 17.5212 | -2.0778 |
| H | 11.6172 | 13.9458 | 1.5173 |
| H | 10.0412 | 13.2132 | 3.3023 |
| H | 6.7316 | 14.9335 | 1.1089 |
| H | 10.5796 | 13.0748 | -4.8523 |
| H | 12.3551 | 11.4296 | -4.2562 |
| H | 12.8626 | 13.3481 | -0.4078 |
| H | 5.6502 | 14.8499 | -0.9310 |
| H | 4.6679 | 13.2595 | -2.5725 |
| H | 8.3207 | 13.3601 | -4.8987 |
| H | 13.5369 | 11.0908 | 0.0496 |
| H | 15.0786 | 9.3249 | -2.9113 |
| H | 16.9494 | 8.1513 | 1.5774 |
| H | 17.8580 | 5.8703 | -1.3058 |
| C | -1.6910 | 7.8457 | -12.7865 |
| C | 0.7485 | 5.0550 | -15.3542 |
| C | -3.3429 | 3.8010 | -13.8758 |
| C | -1.8845 | 5.9953 | -14.9149 |
| H | 3.0667 | 4.8941 | -13.3944 |
| H | 4.1823 | 6.3368 | -11.7035 |
| H | 0.3536 | 8.0144 | -10.5795 |
| H | -2.3000 | 1.3611 | -12.3996 |
| H | -0.4170 | -0.2700 | -12.4542 |
| H | 2.2293 | 2.8164 | -13.9470 |
| H | -1.7653 | 7.3258 | -9.8005 |
| H | -3.0619 | 5.9983 | -8.1341 |
| H | -3.5134 | 2.7984 | -11.0173 |
| H | 2.9533 | 7.1382 | -8.3479 |
| H | 4.4726 | 10.2298 | -9.9274 |
| H | 4.7293 | 9.9494 | -4.9402 |
| H | 7.1125 | -4.4755 | -12.2017 |
| H | 7.7512 | -0.8761 | -13.2403 |
| H | 2.9827 | -2.1810 | -12.4662 |
| H | 3.6828 | 1.5029 | -12.7534 |
| H | 6.3038 | 13.0111 | -6.5639 |
| H | 18.5075 | -1.7561 | -3.4167 |
| C | -2.7701 | 8.6966 | 11.4058 |
| C | -0.5139 | 6.0274 | 14.1846 |
| C | 1.4309 | 9.8357 | 12.6304 |
| C | -0.9916 | 8.7567 | 13.6089 |
| H | -0.4420 | 3.6374 | 12.2721 |
| H | -1.8487 | 2.6541 | 10.4694 |
| H | -3.0162 | 6.6287 | 9.1985 |
| H | 3.7511 | 8.3996 | 11.3108 |
| H | 5.1441 | 6.3752 | 11.7204 |
| H | 1.6572 | 4.2418 | 13.1424 |
| H | -2.1326 | 8.6942 | 8.4734 |
| H | -0.5554 | 9.6963 | 6.8192 |
| H | 2.5820 | 9.7085 | 9.8032 |
| H | 2.8595 | 11.8478 | 8.2633 |
| H | 8.5326 | -1.0370 | 14.7198 |
| H | 15.3455 | -4.0233 | 8.4277 |
| H | 5.7158 | -1.5171 | 12.2215 |
| H | 6.2710 | 3.0911 | 14.0814 |
| H | 3.3467 | 2.5573 | 11.7129 |
| C | 3.5649 | -16.5946 | 2.9281 |
| C | -0.5758 | -14.9547 | 3.0320 |
| C | 1.2189 | -16.4573 | -0.9043 |
| C | 0.9759 | -17.0045 | 1.8566 |
| H | 0.2587 | -12.0923 | 3.8115 |
| H | 2.0308 | -11.0510 | 5.2073 |
| H | 4.8671 | -14.0378 | 3.8766 |
| H | 0.8243 | -13.8356 | -2.3379 |
| H | -0.8176 | -11.9642 | -2.3511 |
| H | -1.1960 | -12.2528 | 1.9523 |
| H | 5.7994 | -14.8875 | 1.8690 |
| H | 6.9096 | -14.2782 | -0.2785 |
| H | 3.1055 | -14.5132 | -2.3301 |
| H | 5.1570 | -10.2342 | 4.5316 |
| H | 4.5181 | -12.3892 | -3.1888 |
| H | 6.1144 | -12.4436 | 7.4568 |
| H | 8.4374 | -8.0231 | 7.1686 |
| H | 7.6481 | -14.2113 | -4.3989 |
| H | 6.0076 | -10.3740 | -7.1606 |
| C | -12.7603 | 0.0927 | -0.8561 |
| C | -12.3919 | -4.3144 | -0.3231 |
| C | -12.0657 | -1.8254 | 3.2203 |
| C | -13.4406 | -2.0035 | 0.8181 |
| H | -10.1309 | -4.5302 | -2.3609 |
| H | -8.9523 | -3.3520 | -4.2204 |
| H | -9.9438 | 0.4640 | -2.4445 |
| H | -8.9206 | -2.3479 | 3.7187 |
| H | -7.3396 | -4.2343 | 3.3981 |
| H | -9.3429 | -5.3100 | -0.2771 |
| H | -10.3006 | 1.9035 | -0.6865 |
| H | -8.8862 | 3.0687 | 1.0092 |
| H | -9.5606 | -0.1740 | 3.8028 |
| H | -7.3413 | 0.4044 | -3.3053 |
| H | -7.0461 | 0.6048 | 3.9698 |
| H | -8.2349 | -0.1392 | -6.9961 |
| H | -4.4840 | 3.0222 | -5.9979 |
| H | -6.6890 | -8.3590 | 2.1360 |
| H | -2.9617 | -8.6703 | -1.1843 |
| H | -7.6034 | 4.2831 | 4.8216 |
| H | -3.9834 | 1.8871 | 7.3106 |
| H | -3.2402 | -11.4680 | 1.3930 |
| H | 9.3072 | -10.1974 | 10.1409 |
| H | -4.6170 | 5.5277 | 8.2484 |
| H | 16.4882 | -0.4213 | 8.3497 |
| H | 18.2438 | -3.4068 | 4.7388 |
| H | 6.2474 | 13.8673 | 5.6445 |
| H | 2.9886 | 10.1518 | 4.8543 |
| H | -5.4944 | 2.5946 | -9.6517 |
| H | 6.2194 | 12.3019 | 2.1756 |
| H | 9.0928 | -12.1785 | -8.4435 |
| H | 18.7180 | 0.3164 | 4.2402 |
| H | 17.2965 | -0.4922 | -6.7732 |
| H | 16.3792 | -5.2965 | -5.7215 |
| H | 14.5451 | -3.8470 | -8.6903 |
| C | 15.1111 | -6.8349 | 12.9558 |
| C | 12.7158 | -3.8378 | 15.4157 |
| C | 11.1113 | -7.9967 | 14.4554 |
| C | 13.4364 | -6.5656 | 15.2285 |
| H | 12.6099 | -1.7445 | 13.2170 |
| H | 14.0180 | -0.9467 | 11.3220 |
| H | 15.3628 | -4.9980 | 10.5996 |
| H | 8.6047 | -6.9253 | 13.2559 |
| H | 7.1089 | -4.9327 | 13.1582 |
| H | 10.4984 | -2.3357 | 13.8812 |
| H | 14.3409 | -7.0118 | 9.9962 |
| H | 12.6883 | -8.0947 | 8.4759 |
| H | 9.7510 | -8.1018 | 11.6562 |
| H | -6.5227 | -5.6025 | -0.4573 |
| H | 17.8356 | 2.6730 | 6.9240 |
| H | 16.3431 | 1.7281 | 7.3043 |
| H | 17.8457 | 1.4917 | 8.2914 |
| H | 16.8222 | -6.4890 | 6.1232 |
| H | 16.7574 | -5.2880 | 4.7750 |
| H | 18.3056 | -5.5570 | 5.6800 |
| H | -8.2555 | 1.1831 | -10.6746 |
| H | -9.1168 | 0.9031 | -9.0989 |
| H | -8.3568 | 2.5041 | -9.4508 |
| H | -4.6122 | 2.5894 | -2.3623 |
| H | -3.6634 | 2.3326 | -3.8795 |
| H | -4.8299 | 3.7168 | -3.7592 |
| H | 4.5103 | 9.1978 | 1.6083 |
| H | 4.3573 | 8.8188 | 3.3639 |
| H | 2.9698 | 9.5927 | 2.4920 |
| H | 5.1723 | 14.5521 | 9.0775 |
| H | 6.4967 | 13.9860 | 7.9858 |
| H | 5.2580 | 15.1630 | 7.3792 |
| H | 3.0087 | -9.7601 | -5.1695 |
| H | 4.5683 | -9.1295 | -5.8278 |
| H | 3.6418 | -10.3964 | -6.7310 |
| H | 10.5226 | -14.9780 | -6.5357 |
| H | 9.9359 | -14.4222 | -4.9195 |
| H | 8.9132 | -15.5292 | -5.9263 |
| H | -5.9441 | -11.4774 | 3.8541 |
| H | -6.1070 | -9.6816 | 3.8949 |
| H | -7.1109 | -10.6556 | 2.7395 |
| H | -3.8441 | -5.5857 | -2.9696 |
| H | -2.6254 | -6.5125 | -2.0111 |
| H | -3.8128 | -7.3895 | -3.0672 |
| H | 3.6930 | -5.7376 | -11.9224 |
| H | 2.9870 | -4.1916 | -11.3045 |
| H | 3.0134 | -4.5314 | -13.0868 |
| H | 7.0432 | 2.6687 | -13.8393 |
| H | 7.8050 | 1.4590 | -12.7336 |
| H | 7.6530 | 1.1037 | -14.5052 |
| H | 9.1884 | 2.2928 | 16.1427 |
| H | 8.5298 | 3.2240 | 14.7411 |
| H | 7.4460 | 2.7713 | 16.1215 |
| H | 2.7250 | -0.7922 | 10.2806 |
| H | 4.4322 | -1.3801 | 10.3612 |
| H | 3.3251 | -1.6137 | 11.7696 |
| H | 16.9374 | 6.7568 | -4.7432 |
| H | 15.3108 | 7.2994 | -4.1726 |
| H | 16.6222 | 8.5153 | -4.4723 |
| H | 15.0316 | 10.6731 | 3.4202 |
| H | 15.2884 | 8.9094 | 3.1221 |
| H | 16.6537 | 10.0800 | 2.8900 |
| H | 2.4955 | 7.0557 | -4.6697 |
| H | 4.1356 | 7.7213 | -4.2634 |
| H | 2.7233 | 8.8368 | -4.4502 |
| H | 6.2266 | 13.4310 | -10.1917 |
| H | 6.2197 | 11.6650 | -10.5747 |
| H | 4.6549 | 12.5611 | -10.3869 |
| H | 18.2826 | -5.3364 | -2.5936 |
| H | 16.6504 | -5.4529 | -3.3606 |
| H | 18.1282 | -5.9489 | -4.2865 |
| H | 15.3353 | -0.4382 | -9.8685 |
| H | 15.4477 | 0.1503 | -8.1639 |
| H | 16.9533 | -0.2762 | -9.0804 |
| H | -7.0638 | 7.5174 | 6.4840 |
| H | -7.2637 | 6.4849 | 5.0142 |
| H | -8.2010 | 6.1055 | 6.5199 |
| H | -4.4596 | -1.3217 | 5.5998 |
| H | -3.3777 | 0.0981 | 5.8752 |
| H | -4.5406 | -0.4262 | 7.1667 |
| H | 7.9496 | -13.6123 | 10.4022 |
| H | 6.3553 | -13.0828 | 9.7348 |
| H | 7.5870 | -13.8505 | 8.6485 |
| H | 6.6639 | -6.8741 | 4.1776 |
| H | 7.1597 | -6.6005 | 5.8892 |
| H | 8.2317 | -7.5438 | 4.7722 |
| H | -12.7059 | -2.7272 | 3.1169 |
| H | -12.7491 | -0.9511 | 3.2010 |
| C | -11.3841 | -1.8904 | 4.5929 |
| H | 0.9112 | -17.2071 | 2.9329 |
| H | 1.6122 | -17.7843 | 1.4192 |
| H | -0.0304 | -17.1335 | 1.4391 |
| H | 0.1714 | -16.7432 | -0.7569 |
| H | 1.8426 | -17.3428 | -0.7339 |
| H | 1.3347 | -16.1828 | -1.9599 |
| H | -0.2459 | -15.5774 | 3.8720 |
| H | -1.2737 | -15.5418 | 2.4245 |
| H | -1.1380 | -14.1145 | 3.4552 |
| H | 2.9406 | -16.8677 | 3.7866 |
| H | 4.5661 | -16.3710 | 3.3165 |
| H | 3.6596 | -17.4730 | 2.2793 |
| H | -13.1443 | -3.9179 | -1.0357 |
| H | -12.9305 | -4.4898 | 0.6331 |
| C | -11.8918 | -5.6719 | -0.8327 |
| H | -13.4775 | -0.6433 | -1.2774 |
| C | -12.4962 | 1.1551 | -1.9308 |
| H | -13.2734 | 0.5723 | 0.0032 |
| H | -13.9797 | -2.1152 | -0.1332 |
| H | -13.8361 | -1.1043 | 1.3084 |
| H | -13.7190 | -2.8624 | 1.4455 |
| H | 15.7048 | -6.2803 | 13.6918 |
| H | 15.7114 | -6.8985 | 12.0401 |
| H | 14.9767 | -7.8580 | 13.3253 |
| H | 14.3523 | -6.0172 | 15.4823 |
| H | 13.7080 | -7.6245 | 15.1364 |
| H | 12.7555 | -6.4786 | 16.0843 |
| H | 10.9292 | -7.6858 | 15.4905 |
| H | 11.9130 | -8.7443 | 14.4590 |
| H | 10.2026 | -8.4995 | 14.1031 |
| H | 13.7889 | -3.8643 | 15.6379 |
| H | 12.1843 | -4.3356 | 16.2347 |
| H | 12.4100 | -2.7848 | 15.4244 |
| H | -1.5822 | 6.1735 | 14.3830 |
| H | 0.0539 | 6.5872 | 14.9368 |
| H | -0.2993 | 4.9638 | 14.3452 |
| H | -1.9345 | 8.3258 | 13.9683 |
| H | -1.1900 | 9.8055 | 13.3553 |
| H | -0.2906 | 8.7522 | 14.4530 |
| H | -3.3309 | 8.2457 | 12.2327 |
| H | -3.4080 | 8.6430 | 10.5150 |
| H | -2.6217 | 9.7590 | 11.6315 |
| H | 1.6184 | 9.6184 | 13.6884 |
| H | 0.7066 | 10.6561 | 12.5725 |
| H | 2.3721 | 10.2038 | 12.2052 |
| H | 23.9412 | 0.1207 | -2.4042 |
| H | 22.9700 | -1.2915 | -1.9901 |
| H | 24.1130 | -0.6457 | -0.8116 |
| H | 24.6691 | 2.0498 | -1.5522 |
| H | 24.8361 | 1.2941 | 0.0356 |
| H | 24.6490 | 3.0460 | -0.0934 |
| H | 23.8951 | 3.1076 | 2.0123 |
| H | 24.0706 | 1.3441 | 2.1258 |
| H | 22.9081 | 2.1875 | 3.1488 |
| H | 23.5654 | 3.6334 | -2.6721 |
| H | 23.5544 | 4.6403 | -1.2095 |
| H | 22.3173 | 4.8597 | -2.4469 |
| H | 10.8837 | 16.4228 | -4.4749 |
| H | 9.1443 | 16.7702 | -4.5551 |
| H | 9.7950 | 15.3154 | -5.3117 |
| H | 8.1887 | 17.8313 | -0.1039 |
| H | 6.6557 | 16.9712 | -0.2435 |
| H | 7.3630 | 17.7288 | -1.6724 |
| H | 11.6364 | 17.1024 | 0.1629 |
| H | 12.5645 | 16.6407 | -1.2790 |
| H | 12.7055 | 15.6999 | 0.2056 |
| H | 10.1438 | 18.1038 | -1.1499 |
| H | 9.3267 | 17.9967 | -2.7121 |
| H | 11.0500 | 17.6275 | -2.5896 |
| H | 13.8080 | -10.5440 | -11.7423 |
| H | 12.2307 | -11.1880 | -12.2428 |
| H | 12.6524 | -11.1011 | -10.5318 |
| H | 13.9175 | -9.3679 | -13.6310 |
| H | 12.9358 | -8.4915 | -14.8089 |
| H | 12.3478 | -10.0109 | -14.1269 |
| H | 14.9727 | -7.4400 | -13.1684 |
| H | 14.6737 | -5.7145 | -12.9602 |
| H | 13.9356 | -6.5679 | -14.3163 |
| H | 10.2604 | -9.3652 | -14.6631 |
| H | 10.8723 | -7.8174 | -15.2817 |
| H | 9.2174 | -7.9422 | -14.6858 |
| H | 0.7735 | 6.1130 | -15.6409 |
| H | 0.1241 | 4.5194 | -16.0781 |
| H | 1.7692 | 4.6682 | -15.4596 |
| H | -1.3022 | 6.8437 | -15.2967 |
| H | -2.8983 | 6.3661 | -14.7157 |
| H | -1.9572 | 5.2596 | -15.7248 |
| H | -1.1172 | 8.3315 | -13.5838 |
| H | -1.6436 | 8.5016 | -11.9082 |
| H | -2.7439 | 7.8171 | -13.0966 |
| H | -3.1310 | 3.6035 | -14.9325 |
| H | -4.0851 | 4.6131 | -13.8144 |
| H | -3.8124 | 2.8922 | -13.4749 |
| H | -11.2231 | -5.5356 | -1.7102 |
| C | -13.0543 | -6.5844 | -1.2132 |
| H | -11.2804 | -6.1680 | -0.0512 |
| H | -13.7216 | -6.1121 | -1.9494 |
| H | -13.6648 | -6.8570 | -0.3404 |
| H | -12.6969 | -7.5273 | -1.6437 |
| H | -12.1914 | 0.6664 | -2.8781 |
| H | -11.6494 | 1.8096 | -1.6313 |
| C | -13.7370 | 2.0111 | -2.1791 |
| H | -14.5956 | 1.4028 | -2.4861 |
| H | -13.5574 | 2.7448 | -2.9731 |
| H | -14.0342 | 2.5658 | -1.2816 |
| H | -10.8696 | -2.8649 | 4.7118 |
| C | -12.3960 | -1.7099 | 5.7236 |
| H | -10.5980 | -1.1083 | 4.6734 |
| H | -13.1833 | -2.4709 | 5.6870 |
| H | -12.8822 | -0.7287 | 5.6771 |
| H | -11.9093 | -1.7872 | 6.7024 |
| H | 10.6383 | -4.9522 | -2.5113 |
| H | 10.0202 | -5.6878 | -1.0161 |
| C | 11.9985 | -6.3623 | -1.5912 |
| H | 12.9104 | -5.9944 | -2.0745 |
| H | 11.6349 | -7.2097 | -2.1844 |
| H | 12.2783 | -6.7487 | -0.6050 |
| H | 9.9610 | -0.5473 | 4.3563 |
| C | 11.8370 | -1.0030 | 5.3404 |
| H | 10.2817 | -2.2836 | 4.5531 |
| H | 12.2922 | -0.0208 | 5.1714 |
| H | 12.6429 | -1.7458 | 5.3268 |
| H | 11.4168 | -1.0001 | 6.3531 |
| H | 11.2218 | 0.7245 | -3.3202 |
| C | 12.8737 | 2.0186 | -2.7883 |
| H | 10.8202 | 1.9523 | -2.0980 |
| H | 13.6770 | 1.3523 | -3.1232 |
| H | 13.2582 | 2.5931 | -1.9374 |
| H | 12.6766 | 2.7283 | -3.5992 |

g) **Singly interlocked (OMe-cube)_2_ (Type 1 conformer)**

| **atom** | **x** | **y** | **z** |
| --- | --- | --- | --- |
| C | -4.9196 | 4.3851 | -4.2761 |
| C | -4.3049 | 4.0423 | -6.7482 |
| C | -5.1612 | 2.0648 | -5.3423 |
| C | -5.3703 | 3.6293 | -5.6149 |
| C | -3.8128 | 5.3159 | -4.7333 |
| C | -3.4417 | 5.0911 | -6.0789 |
| C | -2.3985 | 5.8192 | -6.6409 |
| C | -1.7147 | 6.7709 | -5.8730 |
| N | -1.5064 | 8.0243 | -3.7376 |
| C | -2.0990 | 7.0000 | -4.5420 |
| C | -3.1635 | 6.2731 | -3.9676 |
| C | -3.5545 | 2.7549 | -7.0377 |
| C | -4.1184 | 1.6500 | -6.3616 |
| C | -3.6439 | 0.3625 | -6.5933 |
| C | -2.5656 | 0.1650 | -7.4600 |
| N | -0.9189 | 1.0250 | -9.0229 |
| C | -1.9560 | 1.2745 | -8.0691 |
| C | -2.4448 | 2.5812 | -7.8579 |
| C | -4.4943 | 2.0085 | -3.9747 |
| C | -4.3257 | 3.2903 | -3.4113 |
| C | -3.6178 | 3.4482 | -2.2214 |
| C | -3.1155 | 2.3257 | -1.5576 |
| N | -2.8537 | -0.0558 | -1.3198 |
| C | -3.3013 | 1.0452 | -2.1092 |
| C | -3.9776 | 0.8855 | -3.3373 |
| C | 6.4738 | 15.9749 | 0.7284 |
| C | 6.5162 | 15.6829 | 3.2645 |
| C | 8.6775 | 16.1974 | 2.0005 |
| C | 7.1215 | 16.5535 | 2.0704 |
| C | 5.5392 | 14.8726 | 1.1952 |
| C | 5.5288 | 14.7458 | 2.5991 |
| C | 4.7044 | 13.8116 | 3.2186 |
| C | 3.9088 | 12.9689 | 2.4338 |
| N | 3.3002 | 12.0784 | 0.2433 |
| C | 3.9557 | 13.0708 | 1.0329 |
| C | 4.7577 | 14.0453 | 0.3997 |
| C | 7.6964 | 14.8846 | 3.7930 |
| C | 8.8890 | 15.1760 | 3.1015 |
| C | 10.0759 | 14.5317 | 3.4392 |
| C | 10.0705 | 13.5696 | 4.4556 |
| N | 8.9511 | 12.2769 | 6.1607 |
| C | 8.8718 | 13.2573 | 5.1217 |
| C | 7.6733 | 13.9335 | 4.8046 |
| C | 8.8617 | 15.5421 | 0.6427 |
| C | 7.6424 | 15.4071 | -0.0510 |
| C | 7.6074 | 14.8085 | -1.3074 |
| C | 8.7951 | 14.3347 | -1.8753 |
| N | 11.1733 | 13.9739 | -1.8535 |
| C | 10.0106 | 14.4607 | -1.1776 |
| C | 10.0501 | 15.0819 | 0.0905 |
| C | 10.3005 | 2.2944 | 15.6593 |
| C | 9.8084 | 4.7343 | 15.0856 |
| C | 7.8867 | 3.0689 | 15.3347 |
| C | 9.2944 | 3.4993 | 15.9578 |
| C | 11.3180 | 2.8724 | 14.6911 |
| C | 11.0508 | 4.2215 | 14.3838 |
| C | 11.8784 | 4.9248 | 13.5130 |
| C | 12.9723 | 4.2755 | 12.9307 |
| N | 14.3807 | 2.3522 | 12.5984 |
| C | 13.2259 | 2.9221 | 13.2211 |
| C | 12.4002 | 2.2134 | 14.1219 |
| C | 8.7012 | 4.9795 | 14.0757 |
| C | 7.6451 | 4.0555 | 14.2087 |
| C | 6.5424 | 4.1197 | 13.3608 |
| C | 6.4968 | 5.1048 | 12.3684 |
| N | 7.4141 | 7.0053 | 11.2004 |
| C | 7.5588 | 6.0151 | 12.2253 |
| C | 8.6696 | 5.9634 | 13.0963 |
| C | 8.1195 | 1.6708 | 14.7901 |
| C | 9.4499 | 1.2432 | 14.9747 |
| C | 9.8523 | -0.0182 | 14.5449 |
| C | 8.9255 | -0.8590 | 13.9188 |
| N | 6.7091 | -1.3628 | 13.1120 |
| C | 7.6018 | -0.4254 | 13.7218 |
| C | 7.1877 | 0.8490 | 14.1693 |
| C | 8.4464 | 7.4512 | 10.5534 |
| C | 8.2884 | 8.4716 | 9.4975 |
| C | 7.0716 | 8.7128 | 8.8518 |
| C | 7.0068 | 9.6538 | 7.8168 |
| C | 8.1417 | 10.4016 | 7.4208 |
| C | 9.3574 | 10.1710 | 8.0758 |
| C | 9.4271 | 9.2181 | 9.0958 |
| C | 8.0190 | 11.3908 | 6.3282 |
| C | -0.3009 | -9.2417 | 7.9896 |
| C | -2.0912 | -8.3052 | 6.4027 |
| C | -0.8871 | -10.5018 | 5.8234 |
| C | -1.5218 | -9.6651 | 7.0361 |
| C | -0.3363 | -7.7250 | 7.9861 |
| C | -1.3203 | -7.2113 | 7.1138 |
| C | -1.4808 | -5.8356 | 6.9744 |
| C | -0.6542 | -4.9599 | 7.6864 |
| N | 1.1155 | -4.5137 | 9.2703 |
| C | 0.3320 | -5.4720 | 8.5471 |
| C | 0.4806 | -6.8664 | 8.7102 |
| C | -1.6866 | -8.3779 | 4.9419 |
| C | -1.0847 | -9.6112 | 4.6088 |
| C | -0.6509 | -9.8444 | 3.3060 |
| C | -0.7968 | -8.8526 | 2.3286 |
| N | -1.5073 | -6.6213 | 1.6515 |
| C | -1.3487 | -7.6110 | 2.6772 |
| C | -1.8007 | -7.3698 | 3.9934 |
| C | 0.6024 | -10.5231 | 6.1302 |
| C | 0.9286 | -9.7568 | 7.2695 |
| C | 2.2629 | -9.5281 | 7.5988 |
| C | 3.2794 | -10.1012 | 6.8278 |
| N | 4.0391 | -11.4870 | 4.9988 |
| C | 2.9512 | -10.8937 | 5.7143 |
| C | 1.6019 | -11.1113 | 5.3649 |
| C | 2.3588 | -4.7551 | 9.5553 |
| C | 3.1729 | -3.7697 | 10.2951 |
| C | 2.9121 | -2.3958 | 10.2574 |
| C | 3.7634 | -1.5080 | 10.9270 |
| C | 4.8968 | -1.9680 | 11.6487 |
| C | 5.1379 | -3.3440 | 11.7108 |
| C | 4.2876 | -4.2294 | 11.0400 |
| C | -2.2877 | -1.1090 | -1.8178 |
| C | -1.9090 | -2.2001 | -0.8916 |
| C | -1.5805 | -1.9399 | 0.4414 |
| C | -1.2596 | -2.9993 | 1.2972 |
| C | -1.3096 | -4.3461 | 0.8596 |
| C | -1.6080 | -4.6044 | -0.4839 |
| C | -1.8815 | -3.5414 | -1.3518 |
| C | 1.5540 | 11.1678 | -1.1195 |
| C | -1.0742 | -5.4140 | 1.8505 |
| C | 5.7946 | -0.9771 | 12.2762 |
| C | 2.1434 | 12.2547 | -0.3060 |
| C | 23.7555 | -4.6670 | 6.7879 |
| C | 24.8956 | -3.0072 | 5.2156 |
| C | 23.9736 | -2.2119 | 7.4616 |
| C | 24.7489 | -3.4159 | 6.7530 |
| C | 23.4283 | -4.9398 | 5.3304 |
| C | 24.0602 | -4.0251 | 4.4642 |
| C | 23.8770 | -4.1149 | 3.0872 |
| C | 23.0491 | -5.1165 | 2.5685 |
| N | 21.6057 | -7.0271 | 2.8095 |
| C | 22.4058 | -6.0197 | 3.4349 |
| C | 22.6054 | -5.9411 | 4.8312 |
| C | 24.2632 | -1.6299 | 5.1211 |
| C | 23.7564 | -1.1919 | 6.3610 |
| C | 23.1347 | 0.0489 | 6.4701 |
| C | 23.0057 | 0.8553 | 5.3337 |
| N | 23.3529 | 1.3120 | 2.9924 |
| C | 23.4969 | 0.4093 | 4.0930 |
| C | 24.1450 | -0.8411 | 3.9841 |
| C | 22.6485 | -2.8115 | 7.8982 |
| C | 22.5289 | -4.1651 | 7.5243 |
| C | 21.3772 | -4.8832 | 7.8338 |
| C | 20.3322 | -4.2467 | 8.5129 |
| N | 19.3447 | -2.3352 | 9.5964 |
| C | 20.4470 | -2.8916 | 8.8736 |
| C | 21.6219 | -2.1661 | 8.5750 |
| C | 19.0035 | -1.0906 | 9.4642 |
| C | 17.8519 | -0.5574 | 10.2212 |
| C | 22.3289 | 4.5516 | -2.6234 |
| C | 22.5866 | 3.6057 | -1.5178 |
| C | 16.7402 | 11.2479 | -3.9985 |
| C | 15.6136 | 11.8742 | -3.2770 |
| C | 22.8516 | 2.2502 | -1.7423 |
| C | 23.0374 | 1.3867 | -0.6576 |
| C | 22.9687 | 1.8494 | 0.6810 |
| C | 22.7298 | 3.2100 | 0.9049 |
| C | 22.5399 | 4.0727 | -0.1797 |
| C | 23.1234 | 0.8844 | 1.7892 |
| C | 10.0877 | -1.6135 | -15.5827 |
| C | 10.6973 | -4.0571 | -15.1466 |
| C | 8.2519 | -3.3228 | -15.0968 |
| C | 9.6318 | -3.1170 | -15.8757 |
| C | 11.3535 | -1.7505 | -14.7542 |
| C | 11.6921 | -3.0987 | -14.5217 |
| C | 12.8250 | -3.4256 | -13.7819 |
| C | 13.6221 | -2.4016 | -13.2595 |
| N | 14.1701 | -0.0860 | -12.9260 |
| C | 13.2747 | -1.0552 | -13.4774 |
| C | 12.1351 | -0.7223 | -14.2430 |
| C | 9.9059 | -4.7704 | -14.0644 |
| C | 8.5599 | -4.3546 | -14.0300 |
| C | 7.6811 | -4.8857 | -13.0900 |
| C | 8.1463 | -5.8373 | -12.1756 |
| N | 9.8692 | -7.2570 | -11.2671 |
| C | 9.4920 | -6.2464 | -12.2067 |
| C | 10.3829 | -5.7160 | -13.1660 |
| C | 7.9558 | -1.9701 | -14.4748 |
| C | 8.9639 | -1.0250 | -14.7527 |
| C | 8.8597 | 0.2779 | -14.2734 |
| C | 7.7477 | 0.6421 | -13.5059 |
| N | 5.6869 | 0.1150 | -12.3602 |
| C | 6.7438 | -0.3032 | -13.2274 |
| C | 6.8447 | -1.6247 | -13.7168 |
| C | 13.7636 | 1.0806 | -12.5306 |
| C | 4.4603 | -0.2661 | -12.5411 |
| C | 14.7318 | 2.0313 | -11.9445 |
| C | 3.4150 | 0.1448 | -11.5805 |
| C | 15.9774 | 1.6178 | -11.4586 |
| C | 16.8360 | 2.5461 | -10.8607 |
| C | 16.4807 | 3.9128 | -10.7347 |
| C | 15.2472 | 4.3317 | -11.2459 |
| C | 14.3861 | 3.4022 | -11.8390 |
| C | 3.6397 | 1.1235 | -10.6061 |
| C | 2.6237 | 1.4542 | -9.7032 |
| C | 1.3579 | 0.8211 | -9.7493 |
| C | 1.1279 | -0.1533 | -10.7261 |
| C | 2.1431 | -0.4836 | -11.6317 |
| C | 13.0639 | -16.1013 | -1.1316 |
| C | 12.8840 | -15.7328 | -3.6529 |
| C | 15.1125 | -15.3521 | -2.4628 |
| C | 13.8388 | -16.3157 | -2.5128 |
| C | 11.7397 | -15.4709 | -1.5263 |
| C | 11.6368 | -15.2811 | -2.9189 |
| C | 10.4901 | -14.7186 | -3.4725 |
| C | 9.4456 | -14.3215 | -2.6306 |
| N | 8.4357 | -14.0559 | -0.4652 |
| C | 9.5600 | -14.4865 | -1.2377 |
| C | 10.7107 | -15.0842 | -0.6773 |
| C | 13.6305 | -14.5296 | -4.2024 |
| C | 14.8622 | -14.3243 | -3.5493 |
| C | 15.6857 | -13.2631 | -3.9147 |
| C | 15.2702 | -12.3860 | -4.9231 |
| N | 13.6860 | -11.6422 | -6.5839 |
| C | 14.0281 | -12.5769 | -5.5553 |
| C | 13.2049 | -13.6708 | -5.2071 |
| C | 15.0463 | -14.6981 | -1.0937 |
| C | 13.9159 | -15.1123 | -0.3609 |
| C | 13.6821 | -14.6133 | 0.9174 |
| C | 14.5733 | -13.6843 | 1.4660 |
| N | 16.5625 | -12.3288 | 1.3789 |
| C | 15.6920 | -13.2582 | 0.7266 |
| C | 15.9421 | -13.7795 | -0.5623 |
| C | 12.1357 | -10.2693 | -7.8099 |
| C | 8.5713 | -13.5951 | 0.7399 |
| C | 17.2011 | -11.4153 | 0.7148 |
| C | 7.3838 | -13.1251 | 1.4836 |
| C | 18.0786 | -10.4639 | 1.4275 |
| C | 6.1823 | -12.8078 | 0.8411 |
| C | 5.1076 | -12.3032 | 1.5813 |
| C | 5.1988 | -12.1038 | 2.9820 |
| C | 6.3905 | -12.4511 | 3.6292 |
| C | 7.4659 | -12.9520 | 2.8888 |
| C | 13.1122 | -9.4675 | -8.4107 |
| C | 12.7409 | -8.5119 | -9.3629 |
| C | 11.3876 | -8.3303 | -9.7451 |
| C | 10.4147 | -9.1518 | -9.1643 |
| C | 10.7855 | -10.1052 | -8.2108 |
| C | 17.9862 | -10.2546 | 2.8080 |
| C | 18.8042 | -9.3024 | 3.4252 |
| C | 19.7406 | -8.5363 | 2.6860 |
| C | 19.8518 | -8.7647 | 1.3099 |
| C | 19.0301 | -9.7130 | 0.6918 |
| C | 11.0443 | -7.2718 | -10.7174 |
| C | 17.4035 | 4.8383 | -10.0454 |
| C | 20.5444 | -7.5083 | 3.3796 |
| C | 15.6885 | 12.2053 | -1.9192 |
| C | 14.5716 | 12.7405 | -1.2694 |
| C | 13.3505 | 12.9650 | -1.9542 |
| C | 13.2849 | 12.6616 | -3.3186 |
| C | 14.4007 | 12.1227 | -3.9677 |
| C | 17.6953 | 0.8462 | 10.3478 |
| C | 16.5990 | 1.4043 | 11.0133 |
| C | 15.6214 | 0.5709 | 11.5689 |
| C | 15.7896 | -0.8327 | 11.4616 |
| C | 16.8887 | -1.3911 | 10.8002 |
| C | 4.0539 | -11.5100 | 3.7017 |
| C | 14.4146 | 1.1085 | 12.2312 |
| C | 0.3347 | 1.1724 | -8.7413 |
| C | 12.1830 | 13.4779 | -1.2074 |
| C | 2.1801 | 9.9261 | -1.2666 |
| C | 1.5795 | 8.9290 | -2.0440 |
| C | 0.3462 | 9.1506 | -2.7046 |
| C | -0.2819 | 10.3932 | -2.5585 |
| C | 0.3127 | 11.3866 | -1.7724 |
| C | -0.2273 | 8.0706 | -3.5342 |
| C | 20.6577 | 9.9189 | -7.5793 |
| C | 22.3980 | 8.7997 | -6.0807 |
| C | 21.7235 | 7.7089 | -8.2908 |
| C | 22.0394 | 9.1172 | -7.6049 |
| C | 20.3160 | 10.0504 | -6.1054 |
| C | 21.2812 | 9.4401 | -5.2796 |
| C | 21.1383 | 9.4580 | -3.8950 |
| C | 20.0152 | 10.0709 | -3.3282 |
| N | 17.9336 | 11.2758 | -3.4901 |
| C | 19.0398 | 10.6575 | -4.1549 |
| C | 19.1970 | 10.6647 | -5.5587 |
| C | 22.3200 | 7.2868 | -5.9743 |
| C | 21.9476 | 6.6866 | -7.1938 |
| C | 21.8171 | 5.3039 | -7.2884 |
| C | 22.0463 | 4.5128 | -6.1570 |
| N | 22.6468 | 4.2293 | -3.8392 |
| C | 22.4004 | 5.1148 | -4.9357 |
| C | 22.5534 | 6.5160 | -4.8428 |
| C | 20.2520 | 7.7814 | -8.6595 |
| C | 19.6671 | 9.0034 | -8.2712 |
| C | 18.3216 | 9.2544 | -8.5254 |
| C | 17.5480 | 8.2753 | -9.1585 |
| N | 17.2660 | 6.1197 | -10.1935 |
| C | 18.1280 | 7.0479 | -9.5285 |
| C | 19.4984 | 6.8004 | -9.2907 |
| C | 12.4606 | -11.2516 | -6.7553 |
| O | 14.5284 | 13.0974 | 0.0661 |
| O | 14.4485 | 11.7823 | -5.3069 |
| C | 15.7216 | 12.8785 | 0.8570 |
| C | 13.2402 | 11.9560 | -6.0866 |
| O | 3.8778 | -11.9575 | 1.0529 |
| C | 3.7229 | -12.0225 | -0.3858 |
| O | 8.6886 | -13.3213 | 3.4187 |
| C | 8.8857 | -13.1359 | 4.8415 |
| O | 14.7780 | -1.5568 | 12.0649 |
| O | 18.7156 | 1.5701 | 9.7585 |
| C | 14.8385 | -3.0008 | 11.9706 |
| C | 18.6241 | 3.0148 | 9.8017 |
| O | 2.7272 | 2.4050 | -8.7040 |
| O | 2.0252 | -1.4177 | -12.6386 |
| C | 0.8078 | -2.2076 | -12.6833 |
| C | 4.0287 | 3.0009 | -8.4806 |
| O | 13.6172 | -7.6660 | -10.0171 |
| C | 15.0190 | -7.7467 | -9.6619 |
| O | 9.9122 | -10.9665 | -7.5716 |
| C | 8.5040 | -10.8496 | -7.8878 |
| O | -2.1682 | -3.6653 | -2.6970 |
| C | -2.0644 | -4.9844 | -3.2891 |
| O | -0.8777 | -2.8546 | 2.6192 |
| C | -0.8515 | -1.5116 | 3.1672 |
| O | 22.2950 | 5.4297 | -0.0775 |
| O | 23.3008 | 0.0332 | -0.7603 |
| C | 22.1849 | 5.9931 | 1.2522 |
| C | 23.3473 | -0.5421 | -2.0886 |
| O | 5.8768 | 9.8791 | 7.0463 |
| C | 4.5926 | 9.7516 | 7.7053 |
| O | 10.5692 | 8.9055 | 9.8076 |
| C | 11.7911 | 9.6005 | 9.4570 |
| O | 3.6068 | -0.1421 | 10.9691 |
| O | 4.4306 | -5.6059 | 11.0218 |
| C | 5.5653 | -6.1699 | 11.7229 |
| C | 2.5633 | 0.4502 | 10.1521 |
| O | -0.2021 | 12.6499 | -1.5803 |
| O | 2.0935 | 7.6634 | -2.2465 |
| C | -1.5918 | 12.8735 | -1.9358 |
| C | 3.3360 | 7.3233 | -1.5816 |
| O | 18.7956 | -9.0084 | 4.7764 |
| C | 17.8454 | -9.7150 | 5.6102 |
| O | 19.0490 | -10.0175 | -0.6570 |
| C | 19.9629 | -9.2740 | -1.4993 |
| O | 18.0837 | 2.2506 | -10.3425 |
| C | 18.5186 | 0.8700 | -10.3882 |
| O | 13.1450 | 3.7000 | -12.3708 |
| C | 12.6867 | 5.0707 | -12.2769 |
| C | -6.0858 | 5.1233 | -3.5888 |
| C | -4.9906 | 4.5961 | -8.0199 |
| C | -6.4745 | 1.2682 | -5.4303 |
| C | -6.7953 | 3.9741 | -6.0211 |
| H | -2.1169 | 5.6583 | -7.6859 |
| H | -0.8997 | 7.3388 | -6.3148 |
| H | -3.4749 | 6.4738 | -2.9383 |
| H | -4.1172 | -0.4944 | -6.1016 |
| H | -2.1983 | -0.8403 | -7.6629 |
| H | -1.9542 | 3.4272 | -8.3319 |
| H | -3.4409 | 4.4389 | -1.8058 |
| H | -2.5845 | 2.4348 | -0.6123 |
| H | -4.0870 | -0.1060 | -3.7701 |
| C | 5.7078 | 16.9959 | -0.1138 |
| C | 5.8604 | 16.4844 | 4.3870 |
| C | 9.6241 | 17.3845 | 2.1686 |
| C | 6.8698 | 18.0407 | 2.2441 |
| H | 4.6793 | 13.7242 | 4.3029 |
| H | 3.2723 | 12.2218 | 2.9047 |
| H | 4.7768 | 14.1208 | -0.6839 |
| H | 11.0009 | 14.7621 | 2.9149 |
| H | 10.9891 | 13.0525 | 4.7290 |
| H | 6.7545 | 13.7117 | 5.3405 |
| H | 6.6677 | 14.7017 | -1.8459 |
| H | 8.7856 | 13.8628 | -2.8575 |
| H | 10.9917 | 15.2013 | 0.6192 |
| C | 11.0002 | 1.7147 | 16.8883 |
| C | 10.1129 | 6.0057 | 15.8765 |
| C | 6.7150 | 3.0744 | 16.3163 |
| C | 9.2003 | 3.8276 | 17.4372 |
| H | 11.6792 | 5.9686 | 13.2797 |
| H | 13.6302 | 4.8104 | 12.2469 |
| H | 12.6112 | 1.1760 | 14.3668 |
| H | 5.7218 | 3.4120 | 13.4616 |
| H | 5.6336 | 5.1805 | 11.6957 |
| H | 9.4791 | 6.6827 | 13.0054 |
| H | 10.8773 | -0.3530 | 14.6900 |
| H | 9.2227 | -1.8501 | 13.5791 |
| H | 6.1591 | 1.1739 | 14.0372 |
| H | 9.4967 | 7.1323 | 10.7077 |
| H | 6.1857 | 8.1539 | 9.1658 |
| H | 10.2312 | 10.7550 | 7.7774 |
| H | 7.1006 | 11.2812 | 5.7196 |
| C | -0.4338 | -9.8236 | 9.4139 |
| C | -3.6173 | -8.1610 | 6.5862 |
| C | -1.5081 | -11.9036 | 5.6897 |
| C | -2.5927 | -10.4385 | 7.7925 |
| H | -2.2598 | -5.4380 | 6.3158 |
| H | -0.7693 | -3.8830 | 7.5720 |
| H | 1.2134 | -7.2714 | 9.4136 |
| H | -0.1793 | -10.7888 | 3.0364 |
| H | -0.4597 | -9.0320 | 1.3090 |
| H | -2.2217 | -6.4008 | 4.2526 |
| H | 2.5270 | -8.8938 | 8.4433 |
| H | 4.3251 | -9.9323 | 7.0809 |
| H | 1.3446 | -11.7416 | 4.5110 |
| H | 2.9183 | -5.6744 | 9.2884 |
| H | 2.0477 | -2.0367 | 9.6974 |
| H | 5.9965 | -3.6993 | 12.2856 |
| H | -2.0471 | -1.3016 | -2.8792 |
| H | -1.5913 | -0.9058 | 0.7940 |
| H | -1.6352 | -5.6398 | -0.8268 |
| H | -0.5363 | -5.0696 | 2.7578 |
| H | 5.6090 | 0.0659 | 11.9502 |
| H | 1.5153 | 13.1636 | -0.2338 |
| C | 24.3158 | -5.9213 | 7.4578 |
| C | 26.3286 | -2.9802 | 4.6853 |
| C | 24.6997 | -1.5833 | 8.6503 |
| C | 26.0859 | -3.7145 | 7.4076 |
| H | 24.3674 | -3.4137 | 2.4151 |
| H | 22.8949 | -5.2016 | 1.4934 |
| H | 22.1290 | -6.6534 | 5.4994 |
| H | 22.7468 | 0.3930 | 7.4266 |
| H | 22.5220 | 1.8289 | 5.4016 |
| H | 24.5469 | -1.1747 | 3.0313 |
| H | 21.2826 | -5.9294 | 7.5504 |
| H | 19.4243 | -4.7941 | 8.7634 |
| H | 21.7214 | -1.1263 | 8.8746 |
| H | 19.4938 | -0.3440 | 8.8097 |
| H | 21.8523 | 5.4983 | -2.3021 |
| H | 16.4499 | 10.7724 | -4.9559 |
| H | 22.9098 | 1.8936 | -2.7733 |
| H | 22.6986 | 3.5717 | 1.9352 |
| H | 23.0139 | -0.1738 | 1.4818 |
| C | 10.3515 | -0.7626 | -16.8247 |
| C | 11.4044 | -5.0625 | -16.0543 |
| C | 7.0803 | -3.7810 | -15.9660 |
| C | 9.5127 | -3.4111 | -17.3606 |
| H | 13.0912 | -4.4655 | -13.6047 |
| H | 14.5129 | -2.6388 | -12.6789 |
| H | 11.8786 | 0.3167 | -14.4310 |
| H | 6.6418 | -4.5645 | -13.0577 |
| H | 7.4717 | -6.2633 | -11.4342 |
| H | 11.4172 | -6.0467 | -13.2029 |
| H | 9.6360 | 1.0104 | -14.4848 |
| H | 7.6568 | 1.6542 | -13.1140 |
| H | 6.0782 | -2.3595 | -13.4853 |
| H | 12.7258 | 1.4631 | -12.5711 |
| H | 4.0894 | -0.9005 | -13.3692 |
| H | 16.2545 | 0.5661 | -11.5606 |
| H | 14.9829 | 5.3888 | -11.1704 |
| H | 4.6195 | 1.6054 | -10.5646 |
| H | 0.1548 | -0.6564 | -10.7588 |
| C | 12.8312 | -17.3718 | -0.3146 |
| C | 12.5375 | -16.7116 | -4.7736 |
| C | 16.4589 | -16.0448 | -2.6673 |
| C | 14.2143 | -17.7673 | -2.7513 |
| H | 10.4029 | -14.5811 | -4.5483 |
| H | 8.5418 | -13.8772 | -3.0461 |
| H | 10.7872 | -15.2399 | 0.3954 |
| H | 16.6422 | -13.1070 | -3.4201 |
| H | 15.9007 | -11.5485 | -5.2187 |
| H | 12.2597 | -13.8361 | -5.7172 |
| H | 12.8141 | -14.9355 | 1.4888 |
| H | 14.4051 | -13.2832 | 2.4648 |
| H | 16.8203 | -13.4711 | -1.1231 |
| H | 9.5205 | -13.4935 | 1.3003 |
| H | 17.1559 | -11.2501 | -0.3792 |
| H | 6.1092 | -12.9645 | -0.2376 |
| H | 6.4541 | -12.3184 | 4.7119 |
| H | 14.1555 | -9.6093 | -8.1197 |
| H | 9.3746 | -9.0280 | -9.4746 |
| H | 17.2695 | -10.8490 | 3.3791 |
| H | 20.5889 | -8.1912 | 0.7434 |
| H | 11.8568 | -6.5402 | -10.8924 |
| H | 18.1648 | 4.3300 | -9.4222 |
| H | 20.1439 | -7.2180 | 4.3700 |
| H | 16.6303 | 12.0362 | -1.3923 |
| H | 12.3519 | 12.8566 | -3.8525 |
| H | 16.4821 | 2.4856 | 11.1151 |
| H | 17.0205 | -2.4727 | 10.7232 |
| H | 3.2701 | -11.0966 | 3.0364 |
| H | 13.5999 | 0.3683 | 12.3505 |
| H | 0.7523 | 1.5443 | -7.7862 |
| H | 12.2730 | 13.3774 | -0.1088 |
| H | 3.1377 | 9.7573 | -0.7662 |
| H | -1.2318 | 10.5693 | -3.0674 |
| H | 0.5202 | 7.3451 | -3.9101 |
| C | 20.6917 | 11.2923 | -8.2484 |
| C | 23.7630 | 9.3051 | -5.6161 |
| C | 22.5710 | 7.3816 | -9.5198 |
| C | 23.1426 | 9.8787 | -8.3177 |
| H | 21.8864 | 8.9964 | -3.2538 |
| H | 19.8864 | 10.0920 | -2.2468 |
| H | 18.4585 | 11.1441 | -6.1957 |
| H | 21.5354 | 4.8362 | -8.2295 |
| H | 21.9480 | 3.4295 | -6.2136 |
| H | 22.8523 | 6.9789 | -3.9062 |
| H | 17.8685 | 10.1996 | -8.2336 |
| H | 16.4935 | 8.4549 | -9.3649 |
| H | 19.9533 | 5.8634 | -9.6006 |
| H | 11.5835 | -11.5779 | -6.1626 |
| H | 15.4043 | 13.2420 | 1.8427 |
| H | 15.9665 | 11.8128 | 0.8902 |
| H | 16.5513 | 13.4760 | 0.4665 |
| H | 13.5633 | 11.6084 | -7.0763 |
| H | 12.4375 | 11.3268 | -5.6911 |
| H | 12.9550 | 13.0123 | -6.1148 |
| H | 2.6878 | -11.6794 | -0.5127 |
| H | 4.4258 | -11.3417 | -0.8757 |
| H | 3.8405 | -13.0535 | -0.7335 |
| H | 9.9146 | -13.4951 | 4.9715 |
| H | 8.8022 | -12.0770 | 5.1033 |
| H | 8.1800 | -13.7530 | 5.4063 |
| H | 13.9327 | -3.2966 | 12.5153 |
| H | 14.7895 | -3.3168 | 10.9244 |
| H | 15.7373 | -3.3766 | 12.4693 |
| H | 19.5374 | 3.3108 | 9.2698 |
| H | 17.7309 | 3.3562 | 9.2704 |
| H | 18.6419 | 3.3657 | 10.8382 |
| H | 1.0096 | -2.8794 | -13.5252 |
| H | 0.6819 | -2.7665 | -11.7477 |
| H | -0.0546 | -1.5659 | -12.8846 |
| H | 3.8300 | 3.6528 | -7.6202 |
| H | 4.7648 | 2.2311 | -8.2295 |
| H | 4.3316 | 3.5847 | -9.3548 |
| H | 15.4571 | -6.9804 | -10.3142 |
| H | 15.1600 | -7.4931 | -8.6071 |
| H | 15.4148 | -8.7388 | -9.9002 |
| H | 8.0682 | -11.6293 | -7.2500 |
| H | 8.1321 | -9.8593 | -7.6092 |
| H | 8.3330 | -11.0672 | -8.9467 |
| H | -2.3107 | -4.7765 | -4.3386 |
| H | -1.0411 | -5.3650 | -3.1972 |
| H | -2.7977 | -5.6613 | -2.8413 |
| H | -0.5831 | -1.7035 | 4.2135 |
| H | -0.0783 | -0.9162 | 2.6640 |
| H | -1.8397 | -1.0482 | 3.0955 |
| H | 21.9877 | 7.0496 | 1.0295 |
| H | 21.3462 | 5.5409 | 1.7896 |
| H | 23.1285 | 5.8751 | 1.7940 |
| H | 23.5597 | -1.5957 | -1.8665 |
| H | 22.3791 | -0.4320 | -2.5858 |
| H | 24.1582 | -0.0916 | -2.6694 |
| H | 3.9027 | 10.1040 | 6.9264 |
| H | 4.3905 | 8.6930 | 7.9471 |
| H | 4.5514 | 10.3846 | 8.5943 |
| H | 12.5066 | 9.1607 | 10.1636 |
| H | 12.0718 | 9.3792 | 8.4231 |
| H | 11.6793 | 10.6756 | 9.6288 |
| H | 5.4281 | -7.2434 | 11.5407 |
| H | 6.5011 | -5.8118 | 11.2841 |
| H | 5.5026 | -5.9423 | 12.7917 |
| H | 2.7329 | 1.5226 | 10.3133 |
| H | 2.7033 | 0.1944 | 9.0975 |
| H | 1.5775 | 0.1537 | 10.5196 |
| H | -1.7539 | 13.8956 | -1.5693 |
| H | -1.7184 | 12.8158 | -3.0185 |
| H | -2.2461 | 12.1735 | -1.4016 |
| H | 3.4688 | 6.2751 | -1.8835 |
| H | 4.1504 | 7.9497 | -1.9559 |
| H | 3.2252 | 7.4013 | -0.4953 |
| H | 18.0576 | -9.2882 | 6.5988 |
| H | 16.8219 | -9.4906 | 5.2959 |
| H | 18.0513 | -10.7898 | 5.5939 |
| H | 19.7614 | -9.7117 | -2.4854 |
| H | 19.7148 | -8.2087 | -1.4833 |
| H | 20.9972 | -9.4573 | -1.1920 |
| H | 19.5081 | 0.9335 | -9.9177 |
| H | 17.8440 | 0.2384 | -9.8027 |
| H | 18.5979 | 0.5309 | -11.4257 |
| H | 11.7034 | 5.0093 | -12.7607 |
| H | 12.5917 | 5.3698 | -11.2289 |
| H | 13.3569 | 5.7342 | -12.8320 |
| H | 16.5141 | -16.5357 | -3.6460 |
| H | 16.6453 | -16.8002 | -1.8950 |
| H | 17.2856 | -15.3258 | -2.6200 |
| H | 10.4685 | -3.2667 | -17.8800 |
| H | 8.7778 | -2.7560 | -17.8450 |
| H | 9.1971 | -4.4458 | -17.5443 |
| H | 7.2867 | -4.7471 | -16.4412 |
| H | 6.8542 | -3.0539 | -16.7544 |
| H | 6.1690 | -3.9041 | -15.3686 |
| H | 11.9668 | -4.5599 | -16.8498 |
| H | 10.6930 | -5.7530 | -16.5222 |
| H | 12.1199 | -5.6718 | -15.4890 |
| H | 11.1451 | -1.1929 | -17.4468 |
| H | 10.6718 | 0.2497 | -16.5503 |
| H | 9.4512 | -0.6605 | -17.4416 |
| H | 12.0041 | -17.5894 | -4.3905 |
| H | 13.4359 | -17.0591 | -5.2972 |
| H | 11.8899 | -16.2420 | -5.5238 |
| H | 12.2336 | -18.1043 | -0.8696 |
| H | 12.2899 | -17.1522 | 0.6136 |
| H | 13.7777 | -17.8469 | -0.0315 |
| H | 13.3287 | -18.4143 | -2.7850 |
| H | 14.8676 | -18.1519 | -1.9580 |
| H | 14.7471 | -17.8970 | -3.7018 |
| H | 21.4205 | 11.9563 | -7.7690 |
| H | 19.7153 | 11.7879 | -8.1855 |
| H | 20.9468 | 11.2156 | -9.3118 |
| H | 23.3481 | 10.8417 | -7.8335 |
| H | 22.8790 | 10.0900 | -9.3617 |
| H | 24.0831 | 9.3136 | -8.3282 |
| H | 23.6383 | 7.3413 | -9.2733 |
| H | 22.4315 | 8.1234 | -10.3149 |
| H | 22.2995 | 6.4055 | -9.9396 |
| H | 23.8381 | 10.3955 | -5.7002 |
| H | 24.5776 | 8.8603 | -6.1997 |
| H | 23.9435 | 9.0491 | -4.5651 |
| H | 26.8034 | -3.9656 | 4.7587 |
| H | 26.9478 | -2.2606 | 5.2337 |
| H | 26.3546 | -2.6873 | 3.6288 |
| H | 26.6052 | -4.5423 | 6.9084 |
| H | 25.9670 | -3.9945 | 8.4619 |
| H | 26.7552 | -2.8455 | 7.3761 |
| H | 25.2145 | -6.2845 | 6.9458 |
| H | 23.5847 | -6.7385 | 7.4429 |
| H | 24.5716 | -5.7364 | 8.5076 |
| H | 25.6662 | -1.1600 | 8.3530 |
| H | 24.8774 | -2.3150 | 9.4470 |
| H | 24.1112 | -0.7679 | 9.0878 |
| H | 4.8768 | 17.4391 | 0.4468 |
| H | 5.2804 | 16.5280 | -1.0089 |
| H | 6.3616 | 17.8071 | -0.4546 |
| H | 5.7972 | 18.2662 | 2.2926 |
| H | 7.2875 | 18.6208 | 1.4116 |
| H | 7.3237 | 18.4240 | 3.1665 |
| H | 9.4799 | 17.8795 | 3.1360 |
| H | 9.4802 | 18.1302 | 1.3780 |
| H | 10.6722 | 17.0649 | 2.1226 |
| H | 5.0026 | 17.0604 | 4.0213 |
| H | 6.5681 | 17.1816 | 4.8506 |
| H | 5.4923 | 15.8243 | 5.1817 |
| H | 6.5464 | 4.0726 | 16.7366 |
| H | 6.8805 | 2.3773 | 17.1461 |
| H | 5.7836 | 2.7708 | 15.8228 |
| H | 11.5979 | 2.4732 | 17.4071 |
| H | 11.6828 | 0.9029 | 16.6093 |
| H | 10.2799 | 1.2994 | 17.6027 |
| H | 10.9010 | 5.8376 | 16.6197 |
| H | 9.2239 | 6.3776 | 16.3993 |
| H | 10.4582 | 6.8103 | 15.2162 |
| H | 10.1734 | 4.1222 | 17.8498 |
| H | 8.8473 | 2.9677 | 18.0204 |
| H | 8.5044 | 4.6550 | 17.6250 |
| H | -5.4918 | 5.5487 | -7.7519 |
| H | -5.7979 | 3.8955 | -8.3238 |
| C | -4.0941 | 4.8343 | -9.2417 |
| H | -6.9239 | 5.0514 | -6.1866 |
| H | -7.5234 | 3.6787 | -5.2512 |
| H | -7.0905 | 3.4652 | -6.9496 |
| H | -6.3919 | 5.9713 | -4.2353 |
| C | -5.8222 | 5.6499 | -2.1720 |
| H | -6.9591 | 4.4382 | -3.5381 |
| H | -6.8222 | 1.2925 | -6.4844 |
| H | -7.2510 | 1.7932 | -4.8335 |
| C | -6.4307 | -0.1906 | -4.9581 |
| H | -3.8203 | -8.0284 | 7.6695 |
| H | -4.1016 | -9.1135 | 6.2861 |
| C | -4.2989 | -7.0254 | 5.8116 |
| H | -3.0206 | -9.8436 | 8.6104 |
| H | -2.1904 | -11.3578 | 8.2419 |
| H | -3.4212 | -10.7377 | 7.1363 |
| H | -1.3057 | -9.3431 | 9.9043 |
| C | 0.7882 | -9.6841 | 10.3309 |
| H | -0.6745 | -10.9053 | 9.3354 |
| H | -2.6123 | -11.7981 | 5.6384 |
| H | -1.2887 | -12.4672 | 6.6211 |
| C | -1.0620 | -12.7515 | 4.4919 |
| C | -21.1014 | 8.3819 | 8.1673 |
| C | -22.8128 | 7.1390 | 6.7344 |
| C | -21.9389 | 6.0780 | 8.8880 |
| C | -22.4069 | 7.4637 | 8.2450 |
| C | -20.8542 | 8.5751 | 6.6815 |
| C | -21.8012 | 7.8918 | 5.8925 |
| C | -21.7356 | 7.9492 | 4.5032 |
| C | -20.7120 | 8.6832 | 3.8939 |
| N | -18.7665 | 10.0952 | 3.9661 |
| C | -19.7569 | 9.3518 | 4.6820 |
| C | -19.8339 | 9.3102 | 6.0920 |
| C | -22.6073 | 5.6410 | 6.5938 |
| C | -22.1263 | 5.0570 | 7.7827 |
| C | -21.8715 | 3.6897 | 7.8440 |
| C | -22.0857 | 2.8994 | 6.7093 |
| N | -22.7696 | 2.5971 | 4.4191 |
| C | -22.5498 | 3.4865 | 5.5178 |
| C | -22.8277 | 4.8703 | 5.4597 |
| C | -20.4626 | 6.2713 | 9.1865 |
| C | -20.0015 | 7.5414 | 8.7859 |
| C | -18.6692 | 7.8991 | 8.9724 |
| C | -17.7866 | 6.9825 | 9.5545 |
| N | -17.2796 | 4.8551 | 10.5621 |
| C | -18.2449 | 5.7099 | 9.9425 |
| C | -19.6002 | 5.3512 | 9.7685 |
| C | -10.6268 | 16.1516 | -2.0874 |
| C | -8.3556 | 16.3032 | -0.9279 |
| C | -8.4795 | 15.8518 | -3.4393 |
| C | -9.1384 | 16.7064 | -2.2617 |
| C | -10.6496 | 15.5401 | -0.6973 |
| C | -9.3967 | 15.6270 | -0.0583 |
| C | -9.2217 | 15.1156 | 1.2247 |
| C | -10.2978 | 14.4970 | 1.8702 |
| N | -12.5889 | 13.7636 | 1.9699 |
| C | -11.5429 | 14.3917 | 1.2229 |
| C | -11.7288 | 14.9323 | -0.0688 |
| C | -7.3087 | 15.3017 | -1.3836 |
| C | -7.3618 | 15.0733 | -2.7737 |
| C | -6.4715 | 14.1920 | -3.3799 |
| C | -5.5410 | 13.5072 | -2.5905 |
| N | -4.5537 | 12.9101 | -0.4951 |
| C | -5.5083 | 13.7102 | -1.1979 |
| C | -6.3876 | 14.6333 | -0.5888 |
| C | -9.5759 | 14.8989 | -3.8836 |
| C | -10.7607 | 15.0693 | -3.1407 |
| C | -11.8763 | 14.2778 | -3.4006 |
| C | -11.8060 | 13.2971 | -4.3959 |
| N | -10.6335 | 12.1235 | -6.1515 |
| C | -10.6138 | 13.1134 | -5.1201 |
| C | -9.4880 | 13.9293 | -4.8744 |
| C | 4.6198 | 4.4032 | 5.0139 |
| C | 3.9523 | 6.8289 | 4.5097 |
| C | 3.4517 | 5.8105 | 6.8191 |
| C | 4.5538 | 5.8728 | 5.6479 |
| C | 3.9380 | 4.5502 | 3.6614 |
| C | 3.5668 | 5.8831 | 3.3868 |
| C | 2.8354 | 6.1838 | 2.2392 |
| C | 2.4677 | 5.1627 | 1.3557 |
| N | 2.4340 | 2.8228 | 0.7057 |
| C | 2.8165 | 3.8341 | 1.6454 |
| C | 3.5651 | 3.5243 | 2.8019 |
| C | 2.6788 | 7.3767 | 5.1273 |
| C | 2.3906 | 6.7986 | 6.3832 |
| C | 1.2263 | 7.1525 | 7.0592 |
| C | 0.3228 | 8.0515 | 6.4796 |
| N | -0.3422 | 9.5108 | 4.6393 |
| C | 0.5993 | 8.5957 | 5.2155 |
| C | 1.8016 | 8.2819 | 4.5470 |
| C | 2.9556 | 4.3762 | 6.7876 |
| C | 3.6956 | 3.5774 | 5.8879 |
| C | 3.4668 | 2.2070 | 5.8095 |
| C | 2.4610 | 1.6243 | 6.5868 |
| N | 0.6981 | 1.7706 | 8.2329 |
| C | 1.6717 | 2.4338 | 7.4208 |
| C | 1.9155 | 3.8209 | 7.5230 |
| C | -0.7862 | 9.2922 | 3.4388 |
| C | -1.7319 | 10.2240 | 2.7951 |
| C | -2.1416 | 11.4174 | 3.3997 |
| C | -3.0363 | 12.2632 | 2.7358 |
| C | -3.5380 | 11.9468 | 1.4494 |
| C | -3.1294 | 10.7546 | 0.8422 |
| C | -2.2414 | 9.9029 | 1.5089 |
| C | -4.5065 | 12.8550 | 0.8007 |
| C | -7.2382 | -3.5597 | 15.2129 |
| C | -9.2761 | -2.1119 | 15.7416 |
| C | -9.5707 | -4.6021 | 15.2542 |
| C | -8.6285 | -3.5494 | 16.0005 |
| C | -7.1332 | -2.1747 | 14.5990 |
| C | -8.2571 | -1.3768 | 14.8930 |
| C | -8.3447 | -0.0750 | 14.4079 |
| C | -7.3120 | 0.4309 | 13.6110 |
| N | -5.1991 | 0.2491 | 12.4891 |
| C | -6.1975 | -0.3720 | 13.3026 |
| C | -6.0975 | -1.6849 | 13.8143 |
| C | -10.5309 | -2.3940 | 14.9341 |
| C | -10.6943 | -3.7685 | 14.6697 |
| C | -11.7879 | -4.2206 | 13.9366 |
| C | -12.7178 | -3.2958 | 13.4486 |
| N | -13.5546 | -1.0535 | 13.1767 |
| C | -12.5423 | -1.9217 | 13.6953 |
| C | -11.4462 | -1.4635 | 14.4596 |
| C | -8.7052 | -5.1745 | 14.1454 |
| C | -7.4172 | -4.6028 | 14.1271 |
| C | -6.4820 | -5.0003 | 13.1757 |
| C | -6.8375 | -5.9617 | 12.2230 |
| N | -8.3956 | -7.5012 | 11.2248 |
| C | -8.1305 | -6.5162 | 12.2285 |
| C | -9.0717 | -6.1331 | 13.2101 |
| C | -4.3628 | -0.4381 | 11.7732 |
| C | -3.3722 | 0.2699 | 10.9356 |
| C | -3.5268 | 1.6143 | 10.5855 |
| C | -2.5815 | 2.2335 | 9.7596 |
| C | -1.4561 | 1.5305 | 9.2656 |
| C | -1.2942 | 0.1865 | 9.6251 |
| C | -2.2392 | -0.4352 | 10.4484 |
| C | -17.3213 | 3.5658 | 10.4242 |
| C | -16.2953 | 2.7229 | 11.0726 |
| C | -15.0825 | 3.2474 | 11.5335 |
| C | -14.1224 | 2.3973 | 12.0922 |
| C | -14.3452 | 1.0021 | 12.2126 |
| C | -15.5689 | 0.4818 | 11.7769 |
| C | -16.5268 | 1.3312 | 11.2131 |
| C | -14.5544 | 12.3991 | 2.2003 |
| C | -13.2751 | 0.1432 | 12.7610 |
| C | -0.5021 | 2.2364 | 8.3855 |
| C | -13.5059 | 13.0524 | 1.3898 |
| C | 2.2044 | -8.1065 | -6.1002 |
| C | 1.4569 | -7.2626 | -8.4133 |
| C | 2.9868 | -5.8113 | -6.9411 |
| C | 2.7076 | -7.3198 | -7.4032 |
| C | 0.8012 | -8.5589 | -6.4625 |
| C | 0.3790 | -8.0649 | -7.7154 |
| C | -0.9072 | -8.3384 | -8.1736 |
| C | -1.7870 | -9.0893 | -7.3862 |
| N | -2.2728 | -10.3770 | -5.3803 |
| C | -1.3659 | -9.5668 | -6.1341 |
| C | -0.0567 | -9.3130 | -5.6735 |
| C | 1.1131 | -5.7866 | -8.4934 |
| C | 2.0272 | -4.9856 | -7.7740 |
| C | 1.9377 | -3.5973 | -7.8203 |
| C | 0.9040 | -2.9911 | -8.5398 |
| N | -1.0717 | -3.1024 | -9.9199 |
| C | -0.0543 | -3.7910 | -9.1850 |
| C | 0.0478 | -5.1989 | -9.1653 |
| C | 2.5141 | -5.7707 | -5.4955 |
| C | 2.1000 | -7.0369 | -5.0307 |
| C | 1.5768 | -7.1766 | -3.7471 |
| C | 1.4814 | -6.0630 | -2.9072 |
| N | 1.8615 | -3.7118 | -2.4591 |
| C | 1.8790 | -4.8007 | -3.3796 |
| C | 2.3845 | -4.6465 | -4.6882 |
| C | 1.2110 | -2.6127 | -2.6705 |
| C | 1.3140 | -1.5296 | -1.6683 |
| C | -6.4539 | -0.5529 | -12.6469 |
| C | -5.3886 | -1.3534 | -12.0151 |
| C | -9.8874 | 7.3185 | -10.5277 |
| C | -9.7818 | 8.3352 | -9.4613 |
| C | -5.5065 | -2.7280 | -11.7851 |
| C | -4.4874 | -3.4030 | -11.1057 |
| C | -3.3289 | -2.7290 | -10.6436 |
| C | -3.1984 | -1.3602 | -10.8959 |
| C | -4.2130 | -0.6860 | -11.5852 |
| C | -2.3104 | -3.4831 | -9.8841 |
| C | -24.1074 | -3.8661 | -6.8595 |
| C | -23.5642 | -6.2785 | -6.2168 |
| C | -24.7939 | -4.7640 | -4.5677 |
| C | -24.6931 | -5.1525 | -6.1139 |
| C | -22.7531 | -4.3091 | -7.3848 |
| C | -22.4561 | -5.6407 | -7.0312 |
| C | -21.2544 | -6.2239 | -7.4230 |
| C | -20.3353 | -5.4721 | -8.1632 |
| N | -19.6399 | -3.4590 | -9.2861 |
| C | -20.6253 | -4.1375 | -8.5016 |
| C | -21.8523 | -3.5501 | -8.1207 |
| C | -23.1115 | -6.5024 | -4.7846 |
| C | -23.7992 | -5.6746 | -3.8748 |
| C | -23.5224 | -5.7405 | -2.5121 |
| C | -22.5394 | -6.6240 | -2.0527 |
| N | -20.8646 | -8.3225 | -2.4081 |
| C | -21.8360 | -7.4315 | -2.9650 |
| C | -22.1333 | -7.3849 | -4.3451 |
| C | -24.3193 | -3.3229 | -4.5015 |
| C | -23.9405 | -2.8291 | -5.7660 |
| C | -23.4701 | -1.5260 | -5.9019 |
| C | -23.3623 | -0.7121 | -4.7684 |
| N | -23.6099 | -0.3055 | -2.4035 |
| C | -23.7228 | -1.2130 | -3.5042 |
| C | -24.2209 | -2.5277 | -3.3673 |
| C | -19.4264 | -2.1858 | -9.1585 |
| C | -23.2441 | -0.7132 | -1.2273 |
| C | -18.3851 | -1.5282 | -9.9752 |
| C | -23.1257 | 0.2510 | -0.1141 |
| C | -17.3864 | -2.2527 | -10.6352 |
| C | -16.3895 | -1.5774 | -11.3476 |
| C | -16.3613 | -0.1621 | -11.4255 |
| C | -17.3773 | 0.5612 | -10.7901 |
| C | -18.3718 | -0.1137 | -10.0749 |
| C | -23.0694 | 1.6331 | -0.3260 |
| C | -22.9042 | 2.4991 | 0.7600 |
| C | -22.7950 | 2.0136 | 2.0877 |
| C | -22.8780 | 0.6333 | 2.3023 |
| C | -23.0388 | -0.2330 | 1.2159 |
| C | -10.4800 | -15.8349 | 3.1949 |
| C | -12.7830 | -15.7087 | 2.0927 |
| C | -10.6867 | -15.9556 | 0.6533 |
| C | -11.3666 | -16.4444 | 2.0148 |
| C | -11.3829 | -14.8121 | 3.8629 |
| C | -12.6567 | -14.7529 | 3.2634 |
| C | -13.6202 | -13.8690 | 3.7411 |
| C | -13.3037 | -13.0151 | 4.8041 |
| N | -11.7653 | -12.1517 | 6.4604 |
| C | -12.0194 | -13.0508 | 5.3755 |
| C | -11.0523 | -13.9732 | 4.9186 |
| C | -12.8833 | -14.9294 | 0.7927 |
| C | -11.7194 | -15.0542 | 0.0079 |
| C | -11.6160 | -14.3929 | -1.2127 |
| C | -12.6794 | -13.5990 | -1.6554 |
| N | -14.8877 | -12.6725 | -1.4246 |
| C | -13.8388 | -13.4698 | -0.8684 |
| C | -13.9495 | -14.1484 | 0.3655 |
| C | -9.4665 | -15.1658 | 1.0927 |
| C | -9.3343 | -15.1307 | 2.4953 |
| C | -8.2594 | -14.4740 | 3.0882 |
| C | -7.3196 | -13.8257 | 2.2795 |
| N | -6.5273 | -13.0676 | 0.1299 |
| C | -7.4628 | -13.8453 | 0.8802 |
| C | -8.5417 | -14.5287 | 0.2756 |
| C | -16.7379 | -11.1473 | -1.3110 |
| C | -10.6392 | -11.5117 | 6.5383 |
| C | -6.0705 | -13.4624 | -1.0174 |
| C | -10.3911 | -10.5739 | 7.6519 |
| C | -5.1485 | -12.5945 | -1.7799 |
| C | -11.4188 | -10.1085 | 8.4797 |
| C | -11.1353 | -9.1795 | 9.4859 |
| C | -9.8204 | -8.6940 | 9.6997 |
| C | -8.7881 | -9.1778 | 8.8886 |
| C | -9.0728 | -10.1031 | 7.8789 |
| C | -16.7019 | -10.8059 | -2.6674 |
| C | -17.6959 | -9.9822 | -3.2061 |
| C | -18.7555 | -9.4789 | -2.4099 |
| C | -18.8065 | -9.8427 | -1.0593 |
| C | -17.8100 | -10.6626 | -0.5198 |
| C | -4.5733 | -11.4438 | -1.2323 |
| C | -3.7112 | -10.6548 | -2.0055 |
| C | -3.4102 | -10.9911 | -3.3498 |
| C | -3.9859 | -12.1446 | -3.8979 |
| C | -4.8379 | -12.9340 | -3.1225 |
| C | -19.7472 | -8.5693 | -3.0195 |
| C | -15.2518 | 0.5036 | -12.1393 |
| C | -2.5019 | -10.1264 | -4.1277 |
| C | -8.5957 | 8.5749 | -8.7612 |
| C | -8.5683 | 9.5398 | -7.7464 |
| C | -9.7179 | 10.2999 | -7.4139 |
| C | -10.9019 | 10.0659 | -8.1240 |
| C | -10.9328 | 9.0939 | -9.1273 |
| C | 1.0123 | -0.1950 | -2.0356 |
| C | 1.2179 | 0.8699 | -1.1487 |
| C | 1.6662 | 0.6123 | 0.1498 |
| C | 1.8651 | -0.7331 | 0.5490 |
| C | 1.7246 | -1.7886 | -0.3554 |
| C | -9.5886 | -7.6775 | 10.7465 |
| C | 1.9681 | 1.6859 | 1.1193 |
| C | -22.5704 | 2.9691 | 3.1923 |
| C | -9.6448 | 11.3033 | -6.3313 |
| C | -14.4146 | 12.1992 | 3.5784 |
| C | -15.4105 | 11.5215 | 4.2893 |
| C | -16.5759 | 11.0291 | 3.6488 |
| C | -16.7290 | 11.2546 | 2.2763 |
| C | -15.7304 | 11.9276 | 1.5644 |
| C | -17.5705 | 10.2717 | 4.4368 |
| C | -11.1588 | 4.6391 | -15.0814 |
| C | -9.1033 | 3.1822 | -15.4930 |
| C | -11.4433 | 2.1788 | -15.7147 |
| C | -10.5782 | 3.4882 | -16.0240 |
| C | -10.0340 | 4.9443 | -14.1071 |
| C | -8.9043 | 4.1308 | -14.3257 |
| C | -7.7860 | 4.2409 | -13.5035 |
| C | -7.7940 | 5.1627 | -12.4501 |
| N | -8.8289 | 6.9278 | -11.1683 |
| C | -8.9207 | 5.9755 | -12.2338 |
| C | -10.0501 | 5.8757 | -13.0781 |
| C | -9.1662 | 1.7508 | -14.9942 |
| C | -10.4581 | 1.1988 | -15.1081 |
| C | -10.7063 | -0.1022 | -14.6800 |
| C | -9.6681 | -0.8512 | -14.1113 |
| N | -7.3699 | -1.1058 | -13.3824 |
| C | -8.3871 | -0.2890 | -13.9770 |
| C | -8.1239 | 1.0180 | -14.4463 |
| C | -12.4629 | 2.6276 | -14.6824 |
| C | -12.3149 | 3.9885 | -14.3470 |
| C | -13.1670 | 4.5849 | -13.4217 |
| C | -14.1631 | 3.8158 | -12.8108 |
| N | -15.3543 | 1.7551 | -12.4651 |
| C | -14.2946 | 2.4509 | -13.1275 |
| C | -13.4470 | 1.8505 | -14.0849 |
| C | -15.6825 | -11.9678 | -0.6804 |
| O | -7.4593 | 9.8529 | -6.9848 |
| O | -12.0439 | 8.7735 | -9.8865 |
| C | -6.2461 | 9.0945 | -7.2137 |
| C | -13.2739 | 9.4850 | -9.6065 |
| O | -12.0678 | -8.6463 | 10.3570 |
| C | -13.4478 | -9.0539 | 10.1924 |
| O | -8.1378 | -10.6540 | 7.0222 |
| C | -6.7793 | -10.1625 | 7.0998 |
| O | 2.2085 | -0.8738 | 1.8829 |
| O | 0.5348 | -0.0686 | -3.3259 |
| C | 2.4548 | -2.2179 | 2.3694 |
| C | 0.0300 | 1.2290 | -3.7310 |
| O | -22.8313 | 3.8770 | 0.6691 |
| O | -23.1302 | -1.6095 | 1.3089 |
| C | -22.9995 | -2.2032 | 2.6235 |
| C | -22.8957 | 4.4660 | -0.6524 |
| O | -17.7590 | -9.5800 | -4.5275 |
| C | -16.6857 | -9.9954 | -5.4069 |
| O | -17.7557 | -11.0806 | 0.7973 |
| C | -18.7874 | -10.6037 | 1.6948 |
| O | -17.7642 | 0.9281 | 10.7454 |
| C | -18.0767 | -0.4847 | 10.8075 |
| O | -12.8913 | 2.8023 | 12.5743 |
| C | -12.5543 | 4.2059 | 12.4571 |
| O | -4.1773 | 0.6457 | -11.9428 |
| O | -4.4870 | -4.7552 | -10.8130 |
| C | -3.1716 | 1.4833 | -11.3167 |
| C | -5.6559 | -5.5217 | -11.1917 |
| O | -3.5172 | 13.4612 | 3.2336 |
| C | -3.0755 | 13.8615 | 4.5535 |
| O | -1.7854 | 8.6996 | 1.0167 |
| C | -2.2297 | 8.2898 | -0.3022 |
| O | -2.6293 | 3.5546 | 9.3531 |
| O | -2.1875 | -1.7487 | 10.8593 |
| C | -1.0361 | -2.5418 | 10.4673 |
| C | -3.7834 | 4.3362 | 9.7465 |
| O | -15.7718 | 12.2003 | 0.2093 |
| O | -15.3762 | 11.2617 | 5.6469 |
| C | -16.9169 | 11.7204 | -0.5363 |
| C | -14.2055 | 11.6925 | 6.3827 |
| O | -3.0794 | -9.5107 | -1.5648 |
| C | -3.3325 | -9.0706 | -0.2054 |
| O | -5.4608 | -14.0900 | -3.5593 |
| C | -5.1793 | -14.5338 | -4.9087 |
| O | -15.3540 | -2.1879 | -12.0308 |
| C | -15.2774 | -3.6333 | -11.9807 |
| O | -19.4175 | 0.4960 | -9.4063 |
| C | -19.4663 | 1.9434 | -9.4160 |
| C | -21.2155 | 9.7321 | 8.8741 |
| C | -24.2396 | 7.5265 | 6.3482 |
| C | -22.6948 | 5.6597 | 10.1485 |
| C | -23.5340 | 8.1175 | 9.0244 |
| H | -22.4683 | 7.4275 | 3.8909 |
| H | -20.6456 | 8.7387 | 2.8079 |
| H | -19.1122 | 9.8467 | 6.7021 |
| H | -21.5050 | 3.2341 | 8.7616 |
| H | -21.8911 | 1.8280 | 6.7399 |
| H | -23.2105 | 5.3200 | 4.5476 |
| H | -18.3101 | 8.8800 | 8.6679 |
| H | -16.7409 | 7.2465 | 9.7084 |
| H | -19.9606 | 4.3776 | 10.0894 |
| C | -11.7307 | 17.1974 | -2.2370 |
| C | -7.6968 | 17.4659 | -0.1862 |
| C | -7.9575 | 16.6663 | -4.6211 |
| C | -9.0999 | 18.1998 | -2.5316 |
| H | -8.2588 | 15.1892 | 1.7254 |
| H | -10.1781 | 14.0897 | 2.8737 |
| H | -12.6979 | 14.8770 | -0.5574 |
| H | -6.5012 | 14.0235 | -4.4547 |
| H | -4.8482 | 12.8002 | -3.0467 |
| H | -6.3527 | 14.8086 | 0.4835 |
| H | -12.7958 | 14.4118 | -2.8344 |
| H | -12.6691 | 12.6689 | -4.6118 |
| H | -8.5770 | 13.8013 | -5.4524 |
| C | 6.0545 | 3.8544 | 4.9302 |
| C | 4.9398 | 7.9278 | 4.0742 |
| C | 4.0405 | 6.1617 | 8.2032 |
| C | 5.9019 | 6.3578 | 6.1560 |
| H | 2.5178 | 7.2053 | 2.0346 |
| H | 1.8914 | 5.3882 | 0.4592 |
| H | 3.8267 | 2.4908 | 3.0179 |
| H | 1.0154 | 6.7291 | 8.0459 |
| H | -0.5962 | 8.3204 | 6.9968 |
| H | 2.0438 | 8.7491 | 3.5884 |
| H | 4.0759 | 1.5865 | 5.1436 |
| H | 2.2785 | 0.5526 | 6.5371 |
| H | 1.2968 | 4.4365 | 8.1713 |
| H | -0.5174 | 8.4201 | 2.8067 |
| H | -1.7495 | 11.6627 | 4.3896 |
| H | -3.5167 | 10.5061 | -0.1478 |
| H | -5.1305 | 13.4267 | 1.5122 |
| C | -6.0099 | -3.8670 | 16.0680 |
| C | -9.6124 | -1.3151 | 17.0013 |
| C | -10.1232 | -5.7192 | 16.1385 |
| C | -8.4644 | -3.8578 | 17.4780 |
| H | -9.2075 | 0.5464 | 14.6382 |
| H | -7.3650 | 1.4468 | 13.2202 |
| H | -5.2271 | -2.2993 | 13.5988 |
| H | -11.9219 | -5.2818 | 13.7370 |
| H | -13.5784 | -3.6323 | 12.8717 |
| H | -11.3252 | -0.4051 | 14.6743 |
| H | -5.4835 | -4.5678 | 13.1635 |
| H | -6.1184 | -6.2819 | 11.4695 |
| H | -10.0612 | -6.5817 | 13.2338 |
| H | -4.3028 | -1.5409 | 11.7058 |
| H | -4.3893 | 2.1609 | 10.9744 |
| H | -0.4270 | -0.3585 | 9.2487 |
| H | -18.0680 | 2.9939 | 9.8401 |
| H | -14.9124 | 4.3229 | 11.4467 |
| H | -15.7506 | -0.5894 | 11.8904 |
| H | -12.2746 | 0.6177 | 12.7587 |
| H | -0.9124 | 3.1497 | 7.9117 |
| H | -13.5928 | 12.8575 | 0.3033 |
| C | 3.1386 | -9.2713 | -5.7188 |
| C | 1.7977 | -7.8495 | -9.8023 |
| C | 4.4614 | -5.4004 | -7.1105 |
| C | 3.9260 | -7.9707 | -8.0424 |
| H | -1.2277 | -7.9765 | -9.1547 |
| H | -2.7945 | -9.3058 | -7.7372 |
| H | 0.2843 | -9.7201 | -4.7189 |
| H | 2.6792 | -2.9813 | -7.2998 |
| H | 0.8325 | -1.9044 | -8.5947 |
| H | -0.6974 | -5.8057 | -9.6733 |
| H | 1.2264 | -8.1431 | -3.3894 |
| H | 1.1066 | -6.1683 | -1.8902 |
| H | 2.6619 | -3.6584 | -5.0476 |
| H | 0.5756 | -2.3769 | -3.5442 |
| H | -6.3773 | 0.5308 | -12.4175 |
| H | -10.9233 | 6.9660 | -10.7025 |
| H | -6.3991 | -3.2439 | -12.1434 |
| H | -2.3066 | -0.8305 | -10.5415 |
| H | -2.7155 | -4.3467 | -9.3228 |
| C | -24.9749 | -3.3234 | -7.9946 |
| C | -24.0164 | -7.5946 | -6.8486 |
| C | -26.1854 | -4.9040 | -3.9524 |
| C | -26.0228 | -5.6054 | -6.6901 |
| H | -21.0242 | -7.2535 | -7.1567 |
| H | -19.3904 | -5.9131 | -8.4786 |
| H | -22.0877 | -2.5277 | -8.4035 |
| H | -24.0582 | -5.1097 | -1.8058 |
| H | -22.3093 | -6.6877 | -0.9900 |
| H | -21.6096 | -8.0298 | -5.0456 |
| H | -23.1825 | -1.1388 | -6.8772 |
| H | -22.9953 | 0.3095 | -4.8571 |
| H | -24.5245 | -2.9057 | -2.3947 |
| H | -19.9521 | -1.5007 | -8.4654 |
| H | -22.9864 | -1.7537 | -0.9486 |
| H | -17.4093 | -3.3434 | -10.5788 |
| H | -17.3700 | 1.6507 | -10.8693 |
| H | -23.1586 | 2.0082 | -1.3481 |
| H | -22.8167 | 0.2597 | 3.3270 |
| C | -9.9683 | -16.8497 | 4.2154 |
| C | -13.9862 | -16.6331 | 2.2697 |
| C | -10.2767 | -17.0762 | -0.3035 |
| C | -11.4882 | -17.9556 | 2.0958 |
| H | -14.6094 | -13.8295 | 3.2896 |
| H | -14.0436 | -12.3132 | 5.1857 |
| H | -10.0714 | -14.0211 | 5.3837 |
| H | -10.7177 | -14.4844 | -1.8199 |
| H | -12.6154 | -13.0745 | -2.6083 |
| H | -14.8527 | -14.0651 | 0.9637 |
| H | -8.1510 | -14.4521 | 4.1707 |
| H | -6.4814 | -13.2930 | 2.7268 |
| H | -8.6582 | -14.5317 | -0.8047 |
| H | -9.8006 | -11.5835 | 5.8170 |
| H | -6.3064 | -14.4215 | -1.5177 |
| H | -12.4309 | -10.4870 | 8.3185 |
| H | -7.7715 | -8.8216 | 9.0673 |
| H | -15.8887 | -11.1976 | -3.2831 |
| H | -19.6350 | -9.4736 | -0.4506 |
| H | -4.8172 | -11.1748 | -0.2009 |
| H | -3.7541 | -12.4012 | -4.9346 |
| H | -19.4285 | -8.1448 | -3.9917 |
| H | -14.3814 | -0.1535 | -12.3301 |
| H | -2.0734 | -9.2844 | -3.5491 |
| H | -7.7045 | 8.0053 | -9.0318 |
| H | -11.7856 | 10.6596 | -7.8765 |
| H | 1.0554 | 1.9045 | -1.4546 |
| H | 1.9389 | -2.8208 | -0.0660 |
| H | -10.5011 | -7.1234 | 11.0402 |
| H | 1.7927 | 1.3974 | 2.1740 |
| H | -22.2240 | 3.9667 | 2.8594 |
| H | -8.7141 | 11.2610 | -5.7333 |
| H | -13.5197 | 12.5833 | 4.0734 |
| H | -17.6375 | 10.8966 | 1.7868 |
| H | -17.1838 | 9.8942 | 5.4029 |
| C | -11.6094 | 5.9064 | -15.8054 |
| C | -7.9914 | 3.3540 | -16.5264 |
| C | -12.1437 | 1.5639 | -16.9261 |
| C | -10.6025 | 3.8676 | -17.4938 |
| H | -6.9122 | 3.6134 | -13.6687 |
| H | -6.9263 | 5.2525 | -11.7858 |
| H | -10.9139 | 6.5179 | -12.9261 |
| H | -11.6985 | -0.5381 | -14.7760 |
| H | -9.8483 | -1.8678 | -13.7643 |
| H | -7.1238 | 1.4395 | -14.3798 |
| H | -13.0614 | 5.6376 | -13.1679 |
| H | -14.8384 | 4.2658 | -12.0838 |
| H | -13.5664 | 0.8036 | -14.3506 |
| H | -15.6560 | -11.8960 | 0.4237 |
| H | -5.5659 | 9.5351 | -6.4718 |
| H | -6.4124 | 8.0335 | -7.0069 |
| H | -5.8797 | 9.2618 | -8.2308 |
| H | -13.9605 | 9.0368 | -10.3361 |
| H | -13.6038 | 9.2878 | -8.5823 |
| H | -13.1451 | 10.5554 | -9.7948 |
| H | -13.9433 | -8.4872 | 10.9910 |
| H | -13.8191 | -8.7542 | 9.2080 |
| H | -13.5476 | -10.1316 | 10.3556 |
| H | -6.2800 | -10.7724 | 6.3360 |
| H | -6.7420 | -9.0922 | 6.8312 |
| H | -6.3589 | -10.3481 | 8.0921 |
| H | 2.7364 | -2.0297 | 3.4126 |
| H | 1.5351 | -2.8145 | 2.3089 |
| H | 3.2800 | -2.6791 | 1.8187 |
| H | -0.3621 | 1.0128 | -4.7338 |
| H | -0.7739 | 1.5570 | -3.0624 |
| H | 0.8473 | 1.9540 | -3.7743 |
| H | -23.0989 | -3.2721 | 2.3947 |
| H | -22.0152 | -1.9812 | 3.0460 |
| H | -23.8102 | -1.8638 | 3.2757 |
| H | -22.8114 | 5.5359 | -0.4227 |
| H | -22.0534 | 4.1266 | -1.2624 |
| H | -23.8578 | 4.2398 | -1.1227 |
| H | -16.9856 | -9.5333 | -6.3561 |
| H | -15.7298 | -9.5935 | -5.0582 |
| H | -16.6640 | -11.0864 | -5.4901 |
| H | -18.4947 | -11.0771 | 2.6407 |
| H | -18.7507 | -9.5131 | 1.7725 |
| H | -19.7698 | -10.9568 | 1.3662 |
| H | -19.0858 | -0.5078 | 10.3766 |
| H | -17.3740 | -1.0577 | 10.1954 |
| H | -18.0855 | -0.8264 | 11.8472 |
| H | -11.5510 | 4.2308 | 12.9015 |
| H | -12.5264 | 4.5047 | 11.4051 |
| H | -13.2556 | 4.8146 | 13.0362 |
| H | -3.3425 | 2.4461 | -11.8108 |
| H | -3.3606 | 1.5419 | -10.2388 |
| H | -2.1633 | 1.1067 | -11.5197 |
| H | -5.3890 | -6.5251 | -10.8360 |
| H | -6.5433 | -5.1442 | -10.6753 |
| H | -5.7810 | -5.5106 | -12.2789 |
| H | -3.6035 | 14.8145 | 4.6850 |
| H | -3.3929 | 13.1284 | 5.3009 |
| H | -1.9912 | 14.0095 | 4.5623 |
| H | -1.6840 | 7.3464 | -0.4343 |
| H | -3.3103 | 8.1279 | -0.3043 |
| H | -1.9211 | 9.0194 | -1.0600 |
| H | -1.2785 | -3.5212 | 10.9004 |
| H | -0.9635 | -2.6130 | 9.3784 |
| H | -0.1239 | -2.1368 | 10.9132 |
| H | -3.5656 | 5.3043 | 9.2769 |
| H | -4.6986 | 3.8981 | 9.3376 |
| H | -3.8255 | 4.4279 | 10.8362 |
| H | -16.6818 | 12.0665 | -1.5509 |
| H | -16.9682 | 10.6284 | -0.4948 |
| H | -17.8346 | 12.1868 | -0.1648 |
| H | -14.4478 | 11.3591 | 7.3999 |
| H | -13.3112 | 11.1878 | 6.0055 |
| H | -14.1089 | 12.7818 | 6.3390 |
| H | -2.7079 | -8.1649 | -0.1393 |
| H | -4.3898 | -8.8255 | -0.0754 |
| H | -2.9940 | -9.8243 | 0.5107 |
| H | -5.7809 | -15.4496 | -4.9712 |
| H | -5.5214 | -13.7883 | -5.6330 |
| H | -4.1138 | -14.7543 | -5.0234 |
| H | -14.3848 | -3.8292 | -12.5887 |
| H | -15.1339 | -3.9718 | -10.9504 |
| H | -16.1681 | -4.0771 | -12.4361 |
| H | -20.3685 | 2.1386 | -8.8222 |
| H | -18.5781 | 2.3580 | -8.9303 |
| H | -19.5822 | 2.3128 | -10.4396 |
| H | -11.1422 | -17.6696 | -0.6206 |
| H | -9.5483 | -17.7534 | 0.1571 |
| H | -9.8136 | -16.6710 | -1.2111 |
| H | -25.9347 | -5.8703 | -7.7513 |
| H | -26.7858 | -4.8206 | -6.6135 |
| H | -26.4114 | -6.4879 | -6.1663 |
| H | -26.5454 | -5.9381 | -4.0034 |
| H | -26.9162 | -4.2621 | -4.4580 |
| H | -26.1816 | -4.6153 | -2.8943 |
| H | -24.3567 | -7.4497 | -7.8805 |
| H | -24.8335 | -8.0546 | -6.2805 |
| H | -23.1963 | -8.3221 | -6.8781 |
| H | -25.1165 | -4.0690 | -8.7857 |
| H | -24.5136 | -2.4441 | -8.4600 |
| H | -25.9633 | -3.0161 | -7.6336 |
| H | -13.9116 | -17.2171 | 3.1944 |
| H | -14.0853 | -17.3317 | 1.4308 |
| H | -14.9203 | -16.0610 | 2.3241 |
| H | -10.7940 | -17.3805 | 4.7038 |
| H | -9.3906 | -16.3577 | 5.0074 |
| H | -9.3118 | -17.5934 | 3.7497 |
| H | -11.9594 | -18.2746 | 3.0340 |
| H | -10.5070 | -18.4438 | 2.0435 |
| H | -12.0962 | -18.3568 | 1.2751 |
| H | -10.7827 | 6.3718 | -16.3549 |
| H | -11.9869 | 6.6545 | -15.0980 |
| H | -12.4174 | 5.6977 | -16.5164 |
| H | -10.0040 | 4.7659 | -17.6907 |
| H | -11.6224 | 4.0747 | -17.8417 |
| H | -10.2004 | 3.0651 | -18.1250 |
| H | -11.4234 | 1.2462 | -17.6889 |
| H | -12.8431 | 2.2697 | -17.3891 |
| H | -12.7230 | 0.6775 | -16.6404 |
| H | -7.9532 | 4.3803 | -16.9096 |
| H | -8.1234 | 2.6758 | -17.3776 |
| H | -7.0085 | 3.1357 | -16.0909 |
| H | 2.0082 | -8.9316 | -9.6739 |
| H | 2.7402 | -7.3860 | -10.1639 |
| C | 0.7430 | -7.6791 | -10.9033 |
| H | 3.7244 | -9.0070 | -8.3423 |
| H | 4.7842 | -7.9914 | -7.3555 |
| H | 4.2489 | -7.4287 | -8.9425 |
| H | 3.0927 | -10.0287 | -6.5289 |
| C | 2.8657 | -9.9661 | -4.3786 |
| H | 4.1829 | -8.8935 | -5.6978 |
| H | 4.6850 | -5.3560 | -8.1968 |
| H | 5.1065 | -6.2013 | -6.6905 |
| C | 4.8841 | -4.0747 | -6.4644 |
| H | -11.6379 | 17.9924 | -1.4878 |
| H | -12.7225 | 16.7476 | -2.1071 |
| H | -11.7144 | 17.6601 | -3.2306 |
| H | -9.5536 | 18.7708 | -1.7116 |
| H | -9.6449 | 18.4600 | -3.4479 |
| H | -8.0719 | 18.5640 | -2.6515 |
| H | -7.1688 | 17.3627 | -4.3142 |
| H | -8.7587 | 17.2455 | -5.0957 |
| H | -7.5320 | 16.0152 | -5.3945 |
| H | -8.4380 | 18.2037 | 0.1429 |
| H | -6.9598 | 17.9796 | -0.8145 |
| H | -7.1699 | 17.1167 | 0.7098 |
| H | 4.3612 | 7.2312 | 8.1763 |
| H | 4.9655 | 5.5671 | 8.3629 |
| C | 3.1276 | 5.9531 | 9.4175 |
| H | 6.7128 | 4.6403 | 4.5009 |
| C | 6.2519 | 2.5754 | 4.1069 |
| H | 6.4178 | 3.6771 | 5.9639 |
| H | 5.9258 | 7.4595 | 3.8692 |
| H | 5.1011 | 8.6176 | 4.9324 |
| C | 4.5468 | 8.7507 | 2.8409 |
| H | 6.6622 | 6.3598 | 5.3610 |
| H | 6.2893 | 5.7253 | 6.9662 |
| H | 5.8526 | 7.3882 | 6.5394 |
| H | -24.4071 | 8.6044 | 6.4565 |
| H | -24.9801 | 7.0020 | 6.9634 |
| H | -24.4507 | 7.2703 | 5.3030 |
| H | -23.8437 | 9.0666 | 8.5692 |
| H | -23.2382 | 8.3346 | 10.0586 |
| H | -24.4217 | 7.4741 | 9.0692 |
| H | -22.0201 | 10.3432 | 8.4486 |
| H | -20.2879 | 10.3093 | 8.7779 |
| H | -21.4095 | 9.6099 | 9.9461 |
| H | -23.7657 | 5.5324 | 9.9517 |
| H | -22.5800 | 6.3977 | 10.9510 |
| H | -22.3219 | 4.7036 | 10.5351 |
| H | -8.7174 | -1.1157 | 17.6021 |
| H | -10.3364 | -1.8444 | 17.6318 |
| H | -10.0550 | -0.3438 | 16.7494 |
| H | -7.8165 | -3.1249 | 17.9750 |
| H | -8.0172 | -4.8471 | 17.6378 |
| H | -9.4278 | -3.8484 | 18.0030 |
| H | -5.8794 | -3.1277 | 16.8669 |
| H | -5.0938 | -3.8536 | 15.4649 |
| H | -6.0780 | -4.8595 | 16.5284 |
| H | -10.7407 | -5.3204 | 16.9517 |
| H | -9.3186 | -6.3174 | 16.5819 |
| H | -10.7538 | -6.4060 | 15.5612 |
| H | 4.5562 | 8.1074 | 1.9392 |
| C | 5.4975 | 9.9261 | 2.6291 |
| H | 3.5076 | 9.1325 | 2.9429 |
| H | 6.5285 | 9.5951 | 2.4662 |
| H | 5.5028 | 10.6090 | 3.4859 |
| H | 5.2013 | 10.5190 | 1.7512 |
| H | 3.0086 | 4.8689 | 9.6164 |
| H | 2.1075 | 6.3436 | 9.2123 |
| C | 3.6917 | 6.6355 | 10.6603 |
| H | 4.6928 | 6.2480 | 10.9223 |
| H | 3.0453 | 6.4755 | 11.5300 |
| H | 3.7984 | 7.7173 | 10.5285 |
| H | 6.0716 | 2.7858 | 3.0333 |
| H | 5.5072 | 1.8047 | 4.4037 |
| C | 7.6615 | 2.0144 | 4.2842 |
| H | 8.4269 | 2.7395 | 3.9847 |
| H | 7.8076 | 1.1162 | 3.6733 |
| H | 7.8614 | 1.7381 | 5.3259 |
| H | 0.6919 | -6.6166 | -11.2153 |
| H | -0.2668 | -7.9406 | -10.5208 |
| C | 1.0661 | -8.5475 | -12.1182 |
| H | 2.0530 | -8.3133 | -12.5332 |
| H | 0.3316 | -8.3955 | -12.9173 |
| H | 1.0599 | -9.6144 | -11.8674 |
| H | 1.7851 | -10.2102 | -4.2825 |
| H | 3.1080 | -9.2794 | -3.5430 |
| C | 3.6889 | -11.2461 | -4.2414 |
| H | 3.4572 | -11.9629 | -5.0383 |
| H | 3.4871 | -11.7460 | -3.2884 |
| H | 4.7648 | -11.0421 | -4.2859 |
| H | 4.1646 | -3.2682 | -6.7269 |
| C | 6.2873 | -3.6655 | -6.9074 |
| H | 4.8587 | -4.1674 | -5.3597 |
| H | 6.3403 | -3.4959 | -7.9893 |
| H | 7.0321 | -4.4297 | -6.6581 |
| H | 6.5993 | -2.7359 | -6.4173 |
| C | -7.7190 | -0.9281 | -5.3180 |
| H | -6.2805 | -0.2277 | -3.8600 |
| H | -5.5636 | -0.7195 | -5.4110 |
| H | -8.5988 | -0.4470 | -4.8757 |
| H | -7.6958 | -1.9614 | -4.9526 |
| H | -7.8761 | -0.9676 | -6.4020 |
| H | -5.7135 | 4.7998 | -1.4686 |
| C | -6.9555 | 6.5577 | -1.6986 |
| H | -4.8620 | 6.2099 | -2.1393 |
| H | -7.9211 | 6.0390 | -1.7064 |
| H | -7.0567 | 7.4473 | -2.3310 |
| H | -6.7822 | 6.9042 | -0.6732 |
| C | -4.7942 | 5.6951 | -10.2911 |
| H | -3.8082 | 3.8626 | -9.6914 |
| H | -3.1442 | 5.3257 | -8.9387 |
| H | -5.7309 | 5.2381 | -10.6526 |
| H | -4.1530 | 5.8513 | -11.1660 |
| H | -5.0565 | 6.6849 | -9.9004 |
| C | -5.7077 | -6.7590 | 6.3365 |
| H | -4.3453 | -7.2803 | 4.7335 |
| H | -3.6998 | -6.0910 | 5.8816 |
| H | -6.3254 | -7.6723 | 6.3431 |
| H | -6.2287 | -6.0227 | 5.7127 |
| H | -5.6936 | -6.3647 | 7.3595 |
| H | 1.6067 | -10.3388 | 9.9700 |
| C | 0.4429 | -10.0527 | 11.7726 |
| H | 1.1811 | -8.6445 | 10.3001 |
| H | 0.0649 | -11.0785 | 11.8499 |
| H | -0.3230 | -9.3882 | 12.1893 |
| H | 1.3242 | -9.9798 | 12.4199 |
| C | -1.6049 | -14.1761 | 4.5896 |
| H | 0.0476 | -12.7836 | 4.4308 |
| H | -1.4127 | -12.2893 | 3.5474 |
| H | -1.2354 | -14.6910 | 5.4840 |
| H | -1.3006 | -14.7732 | 3.7219 |
| H | -2.6998 | -14.1925 | 4.6279 |

h) **Singly interlocked (OMe-cube)_2_ (Type 2 conformer)**

| **atom** |  | **x** | **y** | **z** |
| --- | --- | --- | --- | --- |
| C |  | 3.0737 | 7.4946 | -5.0987 |
| C |  | 2.2761 | 6.5499 | -7.3519 |
| C |  | 1.5821 | 8.9169 | -6.6384 |
| C |  | 2.8086 | 7.8811 | -6.6327 |
| C |  | 2.6798 | 6.0280 | -5.0082 |
| C |  | 2.1526 | 5.5440 | -6.2263 |
| C |  | 1.5749 | 4.2788 | -6.2885 |
| C |  | 1.5981 | 3.4462 | -5.1637 |
| N |  | 2.1609 | 3.0366 | -2.8328 |
| C |  | 2.1882 | 3.9032 | -3.9752 |
| C |  | 2.7085 | 5.2121 | -3.8834 |
| C |  | 0.9143 | 6.9411 | -7.8924 |
| C |  | 0.5299 | 8.2406 | -7.4910 |
| C |  | -0.6957 | 8.7622 | -7.8909 |
| C |  | -1.5529 | 7.9965 | -8.6903 |
| N |  | -1.9386 | 5.9155 | -9.9960 |
| C |  | -1.1645 | 6.7083 | -9.0937 |
| C |  | 0.0810 | 6.1755 | -8.6947 |
| C |  | 1.1572 | 8.9977 | -5.1847 |
| C |  | 2.0360 | 8.2940 | -4.3304 |
| C |  | 1.8440 | 8.3245 | -2.9535 |
| C |  | 0.7592 | 9.0249 | -2.4123 |
| N |  | -1.2440 | 10.4548 | -2.7934 |
| C |  | -0.1463 | 9.6725 | -3.2685 |
| C |  | 0.0500 | 9.6560 | -4.6682 |
| C |  | 4.7196 | -6.7528 | 3.8272 |
| C |  | 4.1291 | -6.1365 | 6.2499 |
| C |  | 3.3524 | -8.3850 | 5.2721 |
| C |  | 4.5833 | -7.3537 | 5.3080 |
| C |  | 4.4218 | -5.2720 | 3.9944 |
| C |  | 3.9973 | -4.9596 | 5.3051 |
| C |  | 3.4971 | -3.6938 | 5.5938 |
| C |  | 3.4849 | -2.7068 | 4.6009 |
| N |  | 4.0504 | -2.0232 | 2.2753 |
| C |  | 3.9668 | -3.0026 | 3.3150 |
| C |  | 4.4407 | -4.2964 | 3.0071 |
| C |  | 2.7718 | -6.5602 | 6.7739 |
| C |  | 2.3525 | -7.8058 | 6.2544 |
| C |  | 1.1199 | -8.3337 | 6.6243 |
| C |  | 0.2789 | -7.6159 | 7.4854 |
| N |  | -0.1640 | -5.4772 | 8.6924 |
| C |  | 0.6853 | -6.3623 | 7.9695 |
| C |  | 1.9544 | -5.8404 | 7.6309 |
| C |  | 2.8084 | -8.2674 | 3.8586 |
| C |  | 3.5655 | -7.3772 | 3.0635 |
| C |  | 3.1636 | -7.0881 | 1.7630 |
| C |  | 2.0388 | -7.7228 | 1.2210 |
| N |  | 0.0783 | -9.2051 | 1.5476 |
| C |  | 1.2947 | -8.6143 | 2.0098 |
| C |  | 1.6675 | -8.8725 | 3.3478 |
| C |  | -10.5724 | 0.6567 | 16.4519 |
| C |  | -8.5158 | -0.8576 | 16.4497 |
| C |  | -8.2496 | 1.6579 | 16.0889 |
| C |  | -9.0529 | 0.5660 | 16.9353 |
| C |  | -10.8418 | -0.6743 | 15.7721 |
| C |  | -9.7058 | -1.5108 | 15.7759 |
| C |  | -9.7590 | -2.7761 | 15.2017 |
| C |  | -10.9500 | -3.2103 | 14.6057 |
| N |  | -13.3382 | -2.7980 | 14.0714 |
| C |  | -12.0778 | -2.3715 | 14.5978 |
| C |  | -12.0322 | -1.0913 | 15.1934 |
| C |  | -7.4338 | -0.5351 | 15.4348 |
| C |  | -7.2861 | 0.8544 | 15.2384 |
| C |  | -6.3279 | 1.3427 | 14.3567 |
| C |  | -5.5108 | 0.4420 | 13.6606 |
| N |  | -4.8346 | -1.9283 | 13.2317 |
| C |  | -5.6664 | -0.9424 | 13.8488 |
| C |  | -6.6362 | -1.4401 | 14.7497 |
| C |  | -9.3057 | 2.3345 | 15.2325 |
| C |  | -10.5888 | 1.7866 | 15.4426 |
| C |  | -11.6910 | 2.2862 | 14.7576 |
| C |  | -11.5199 | 3.3502 | 13.8630 |
| N |  | -10.0216 | 4.8910 | 12.6556 |
| C |  | -10.2391 | 3.8883 | 13.6522 |
| C |  | -9.1146 | 3.3790 | 14.3382 |
| C |  | -4.3313 | -1.7683 | 12.0481 |
| C |  | -3.4645 | -2.8197 | 11.4759 |
| C |  | -2.4946 | -2.4697 | 10.5050 |
| C |  | -1.6709 | -3.4364 | 9.9187 |
| C |  | -1.7909 | -4.7820 | 10.2838 |
| C |  | -2.7529 | -5.1282 | 11.2650 |
| C |  | -3.5789 | -4.1635 | 11.8526 |
| C |  | -0.9450 | -5.8268 | 9.6641 |
| C |  | -11.7459 | 15.0242 | 5.8980 |
| C |  | -9.4577 | 15.3567 | 4.8130 |
| C |  | -11.5816 | 15.6230 | 3.4189 |
| C |  | -10.9472 | 15.9311 | 4.8527 |
| C |  | -10.7149 | 14.0399 | 6.4223 |
| C |  | -9.4504 | 14.2301 | 5.8266 |
| C |  | -8.3745 | 13.4226 | 6.1791 |
| C |  | -8.5597 | 12.4061 | 7.1246 |
| N |  | -10.0301 | 11.2458 | 8.7431 |
| C |  | -9.8221 | 12.2170 | 7.7125 |
| C |  | -10.9140 | 13.0444 | 7.3690 |
| C |  | -9.2939 | 14.8060 | 3.4074 |
| C |  | -10.4665 | 14.9570 | 2.6376 |
| C |  | -10.5085 | 14.5076 | 1.3223 |
| C |  | -9.3739 | 13.9031 | 0.7650 |
| N |  | -7.0871 | 13.0031 | 1.0653 |
| C |  | -8.2133 | 13.7416 | 1.5407 |
| C |  | -8.1642 | 14.2009 | 2.8759 |
| C |  | -12.7048 | 14.6398 | 3.6984 |
| C |  | -12.7957 | 14.3129 | 5.0681 |
| C |  | -13.7665 | 13.4251 | 5.5185 |
| C |  | -14.6627 | 12.8622 | 4.6004 |
| N |  | -15.3775 | 12.5458 | 2.2496 |
| C |  | -14.5648 | 13.1854 | 3.2362 |
| C |  | -13.5767 | 14.0832 | 2.7735 |
| C |  | -9.7498 | 9.9945 | 8.5541 |
| C |  | -9.9497 | 9.0184 | 9.6463 |
| C |  | -9.7062 | 7.6431 | 9.4015 |
| C |  | -9.8784 | 6.6790 | 10.4002 |
| C |  | -10.3058 | 7.0634 | 11.6758 |
| C |  | -10.5496 | 8.4379 | 11.9216 |
| C |  | -10.3713 | 9.4027 | 10.9242 |
| C |  | -2.0375 | 10.0341 | -1.8580 |
| C |  | -3.1495 | 10.8940 | -1.4008 |
| C |  | -3.9488 | 10.4770 | -0.3063 |
| C |  | -5.0170 | 11.2534 | 0.1567 |
| C |  | -5.3218 | 12.4696 | -0.4643 |
| C |  | -4.5207 | 12.8878 | -1.5565 |
| C |  | -3.4507 | 12.1143 | -2.0180 |
| C |  | 3.1851 | -0.1975 | 0.9701 |
| C |  | -6.4533 | 13.3126 | -0.0208 |
| C |  | -10.5243 | 6.0795 | 12.7582 |
| C |  | 3.0793 | -1.1901 | 2.0592 |
| C |  | -23.1769 | -7.6878 | 6.8845 |
| C |  | -22.5088 | -9.5569 | 5.2754 |
| C |  | -21.3685 | -9.3638 | 7.5529 |
| C |  | -22.7797 | -9.2337 | 6.8166 |
| C |  | -23.1745 | -7.2257 | 5.4380 |
| C |  | -22.8062 | -8.2594 | 4.5513 |
| C |  | -22.7499 | -8.0271 | 3.1813 |
| C |  | -23.0531 | -6.7514 | 2.6876 |
| N |  | -23.8083 | -4.4244 | 3.1196 |
| C |  | -23.4141 | -5.7225 | 3.5744 |
| C |  | -23.4847 | -5.9582 | 4.9656 |
| C |  | -21.0287 | -9.8900 | 5.2051 |
| C |  | -20.4041 | -9.7954 | 6.4666 |
| C |  | -19.0485 | -10.0723 | 6.6060 |
| C |  | -18.3052 | -10.4504 | 5.4806 |
| N |  | -18.1755 | -10.7780 | 3.0336 |
| C |  | -18.9280 | -10.5295 | 4.2230 |
| C |  | -20.3051 | -10.2503 | 4.0768 |
| C |  | -21.0465 | -7.9555 | 8.0214 |
| C |  | -22.0493 | -7.0324 | 7.6568 |
| C |  | -21.9294 | -5.6897 | 7.9979 |
| C |  | -20.8020 | -5.2588 | 8.7097 |
| N |  | -18.5707 | -5.7664 | 9.6597 |
| C |  | -19.7991 | -6.1791 | 9.0586 |
| C |  | -19.9187 | -7.5452 | 8.7174 |
| C |  | -18.5273 | -5.0645 | 10.7474 |
| C |  | -17.2162 | -4.6372 | 11.2821 |
| C |  | -14.1799 | -12.5416 | -1.8506 |
| C |  | -15.0224 | -12.3968 | -0.6451 |
| C |  | -5.0133 | -12.1246 | -1.3631 |
| C |  | -3.7196 | -11.5558 | -0.9291 |
| C |  | -15.6745 | -11.1615 | -0.3987 |
| C |  | -16.4686 | -10.9658 | 0.7357 |
| C |  | -16.6348 | -12.0027 | 1.6612 |
| C |  | -15.9919 | -13.2408 | 1.4099 |
| C |  | -15.1982 | -13.4370 | 0.2745 |
| C |  | -17.4381 | -11.8333 | 2.8909 |
| C |  | -11.0221 | -0.9021 | -16.5530 |
| C |  | -13.4445 | -0.1753 | -16.1917 |
| C |  | -11.5874 | 1.5761 | -16.3002 |
| C |  | -12.0949 | 0.2102 | -16.9555 |
| C |  | -11.7790 | -1.8582 | -15.6478 |
| C |  | -13.1152 | -1.4534 | -15.4463 |
| C |  | -13.9631 | -2.1994 | -14.6347 |
| C |  | -13.4739 | -3.3510 | -14.0042 |
| N |  | -11.6154 | -4.9529 | -13.6395 |
| C |  | -12.1404 | -3.7466 | -14.2031 |
| C |  | -11.2811 | -3.0030 | -15.0419 |
| C |  | -13.6787 | 0.9696 | -15.2219 |
| C |  | -12.6553 | 1.9386 | -15.2881 |
| C |  | -12.6976 | 3.0661 | -14.4754 |
| C |  | -13.7711 | 3.2370 | -13.5918 |
| N |  | -15.8207 | 2.3375 | -12.5417 |
| C |  | -14.7826 | 2.2641 | -13.5215 |
| C |  | -14.7433 | 1.1159 | -14.3436 |
| C |  | -10.2871 | 1.2113 | -15.6053 |
| C |  | -9.9690 | -0.1544 | -15.7597 |
| C |  | -8.8107 | -0.6782 | -15.1963 |
| C |  | -7.9596 | 0.1635 | -14.4691 |
| N |  | -7.5221 | 2.3859 | -13.4624 |
| C |  | -8.2868 | 1.5208 | -14.3041 |
| C |  | -9.4601 | 2.0576 | -14.8802 |
| C |  | -11.6659 | -5.1762 | -12.3636 |
| C |  | -6.2322 | 2.4736 | -13.5542 |
| C |  | -11.1156 | -6.4317 | -11.8102 |
| C |  | -5.4886 | 3.3348 | -12.6106 |
| C |  | -11.1488 | -6.6445 | -10.4081 |
| C |  | -10.6308 | -7.8089 | -9.8320 |
| C |  | -10.0619 | -8.7977 | -10.6428 |
| C |  | -10.0321 | -8.5871 | -12.0440 |
| C |  | -10.5523 | -7.4233 | -12.6209 |
| C |  | -4.0928 | 3.5158 | -12.7832 |
| C |  | -3.3413 | 4.2967 | -11.8989 |
| C |  | -3.9645 | 4.9202 | -10.8116 |
| C |  | -5.3622 | 4.7479 | -10.6455 |
| C |  | -6.1138 | 3.9678 | -11.5303 |
| C |  | -25.1847 | 7.1628 | -2.8838 |
| C |  | -24.5894 | 7.1876 | -5.3682 |
| C |  | -25.6365 | 5.1033 | -4.3265 |
| C |  | -25.6961 | 6.6996 | -4.3246 |
| C |  | -23.8899 | 7.9083 | -3.1556 |
| C |  | -23.5648 | 7.9237 | -4.5284 |
| C |  | -22.4141 | 8.5648 | -4.9740 |
| C |  | -21.5712 | 9.1880 | -4.0449 |
| N |  | -21.1129 | 9.8559 | -1.6979 |
| C |  | -21.8951 | 9.1662 | -2.6774 |
| C |  | -23.0701 | 8.5270 | -2.2223 |
| C |  | -24.0017 | 5.9069 | -5.9345 |
| C |  | -24.5874 | 4.7572 | -5.3640 |
| C |  | -24.1860 | 3.4876 | -5.7651 |
| C |  | -23.1819 | 3.3587 | -6.7333 |
| N |  | -21.6115 | 4.4368 | -8.3314 |
| C |  | -22.5921 | 4.5060 | -7.2916 |
| C |  | -23.0092 | 5.7975 | -6.8983 |
| C |  | -25.1558 | 4.7337 | -2.9342 |
| C |  | -24.9135 | 5.8723 | -2.1369 |
| C |  | -24.4700 | 5.7341 | -0.8263 |
| C |  | -24.2690 | 4.4515 | -0.2997 |
| N |  | -24.1772 | 2.0050 | -0.6462 |
| C |  | -24.4998 | 3.3207 | -1.1015 |
| C |  | -24.9506 | 3.4558 | -2.4336 |
| C |  | -19.6194 | 3.5601 | -9.3553 |
| C |  | -19.8261 | 9.7081 | -1.6345 |
| C |  | -24.7103 | 1.4882 | 0.4147 |
| C |  | -19.0456 | 10.4503 | -0.6225 |
| C |  | -24.2957 | 0.1386 | 0.8550 |
| C |  | -17.6338 | 10.3167 | -0.6002 |
| C |  | -16.8493 | 10.9882 | 0.3435 |
| C |  | -17.4548 | 11.8157 | 1.2958 |
| C |  | -18.8656 | 11.9542 | 1.2697 |
| C |  | -19.6503 | 11.2836 | 0.3256 |
| C |  | -18.5998 | 2.5749 | -9.3178 |
| C |  | -17.6293 | 2.4865 | -10.3208 |
| C |  | -17.6486 | 3.3843 | -11.3944 |
| C |  | -18.6725 | 4.3636 | -11.4368 |
| C |  | -19.6447 | 4.4509 | -10.4343 |
| C |  | -25.0357 | -0.5270 | 1.8643 |
| C |  | -24.6685 | -1.7957 | 2.3257 |
| C |  | -23.5450 | -2.4368 | 1.7916 |
| C |  | -22.8090 | -1.7735 | 0.7766 |
| C |  | -23.1774 | -0.5061 | 0.3140 |
| C |  | -16.6284 | 3.3469 | -12.4643 |
| C |  | -9.4907 | -10.0405 | -10.0789 |
| C |  | -23.1009 | -3.7689 | 2.2527 |
| C |  | -3.6800 | -10.6967 | 0.1992 |
| C |  | -2.4825 | -10.1336 | 0.6495 |
| C |  | -1.2825 | -10.4151 | -0.0155 |
| C |  | -1.3220 | -11.2699 | -1.1452 |
| C |  | -2.5221 | -11.8327 | -1.5963 |
| C |  | -16.0339 | -4.7861 | 10.5482 |
| C |  | -14.8175 | -4.3527 | 11.0855 |
| C |  | -14.7435 | -3.7579 | 12.3709 |
| C |  | -15.9251 | -3.6148 | 13.1073 |
| C |  | -17.1419 | -4.0473 | 12.5690 |
| C |  | -16.6708 | 12.5312 | 2.3253 |
| C |  | -13.4323 | -3.3089 | 12.8833 |
| C |  | -3.2130 | 5.7381 | -9.8366 |
| C |  | 0.0105 | -9.8492 | 0.4245 |
| C |  | 2.2198 | 0.8417 | 0.8865 |
| C |  | 2.2330 | 1.7664 | -0.1643 |
| C |  | 3.2161 | 1.6759 | -1.1556 |
| C |  | 4.2024 | 0.6658 | -1.0491 |
| C |  | 4.1869 | -0.2631 | -0.0026 |
| C |  | 3.2616 | 2.5940 | -2.3136 |
| C |  | -7.8416 | -14.9131 | -5.9775 |
| C |  | -9.6354 | -15.7270 | -4.3500 |
| C |  | -10.3075 | -14.8082 | -6.6369 |
| C |  | -9.2216 | -15.7132 | -5.8933 |
| C |  | -7.5081 | -14.5679 | -4.5370 |
| C |  | -8.5020 | -15.0138 | -3.6401 |
| C |  | -8.3708 | -14.7897 | -2.2741 |
| C |  | -7.2416 | -14.1135 | -1.7936 |
| N |  | -5.0331 | -13.0670 | -2.2531 |
| C |  | -6.2523 | -13.6727 | -2.6892 |
| C |  | -6.3809 | -13.9020 | -4.0779 |
| C |  | -10.9156 | -14.9132 | -4.2868 |
| C |  | -11.2891 | -14.4131 | -5.5521 |
| C |  | -12.4449 | -13.6537 | -5.6980 |
| C |  | -13.2336 | -13.3804 | -4.5731 |
| N |  | -13.6602 | -13.6932 | -2.1435 |
| C |  | -12.8554 | -13.8738 | -3.3124 |
| C |  | -11.6874 | -14.6549 | -3.1627 |
| C |  | -9.5311 | -13.5979 | -7.1258 |
| C |  | -8.1687 | -13.6606 | -6.7656 |
| C |  | -7.2958 | -12.6406 | -7.1281 |
| C |  | -7.7789 | -11.5490 | -7.8614 |
| N |  | -9.6768 | -10.3178 | -8.8291 |
| C |  | -9.1386 | -11.4878 | -8.2094 |
| C |  | -10.0302 | -12.5206 | -7.8447 |
| C |  | -20.6079 | 3.6185 | -8.2584 |
| O |  | -11.7361 | -5.6108 | -9.7042 |
| C |  | -11.7437 | -5.7008 | -8.2583 |
| O |  | -9.4505 | -9.6256 | -12.7475 |
| C |  | -9.3727 | -9.5000 | -14.1883 |
| O |  | -9.2769 | 7.3743 | 8.1148 |
| C |  | -9.1737 | 5.9845 | 7.7204 |
| O |  | -10.9635 | 8.7029 | 13.2134 |
| C |  | -11.2790 | 10.0763 | 13.5489 |
| O |  | 5.1379 | 0.7007 | -2.0670 |
| C |  | 6.1680 | -0.3177 | -2.0650 |
| O |  | 1.3213 | 0.8409 | 1.9281 |
| C |  | 0.1640 | 1.7098 | 1.8394 |
| O |  | -13.5942 | -4.4555 | 10.4510 |
| C |  | -13.5752 | -5.0076 | 9.1118 |
| O |  | -18.3658 | -3.9525 | 13.2051 |
| C |  | -18.3920 | -3.3566 | 14.5249 |
| O |  | -5.8770 | 5.4192 | -9.5527 |
| O |  | -3.5806 | 2.8561 | -13.8845 |
| C |  | -2.1529 | 2.9414 | -14.1151 |
| O |  | -4.9179 | -10.4904 | 0.7804 |
| C |  | -4.9735 | -9.6517 | 1.9599 |
| O |  | -0.0921 | -11.4626 | -1.7423 |
| C |  | 0.0080 | -12.4926 | -2.7595 |
| O |  | -4.9084 | 14.1017 | -2.0942 |
| C |  | -4.1357 | 14.6215 | -3.2032 |
| O |  | -3.5785 | 9.2570 | 0.2242 |
| C |  | -4.2768 | 8.8035 | 1.4110 |
| O |  | -2.7866 | -6.4807 | 11.5525 |
| C |  | -3.6853 | -6.9215 | 12.5986 |
| O |  | -18.6746 | 1.7416 | -8.2173 |
| C |  | -17.6573 | 0.7199 | -8.0783 |
| O |  | -18.5991 | 5.1924 | -12.5407 |
| C |  | -19.6064 | 6.2246 | -12.6739 |
| O |  | -16.2281 | -14.1939 | 2.3830 |
| C |  | -15.6191 | -15.4964 | 2.2093 |
| O |  | -15.4435 | -10.2092 | -1.3736 |
| C |  | -16.0519 | -8.9065 | -1.1977 |
| O |  | -2.4624 | -1.1250 | 10.1874 |
| C |  | -1.3259 | -0.6600 | 9.4144 |
| O |  | -17.1409 | 9.4811 | -1.5841 |
| C |  | -15.7109 | 9.2517 | -1.6181 |
| O |  | -19.3567 | 12.7966 | 2.2501 |
| C |  | -20.7897 | 12.9988 | 2.3053 |
| O |  | -26.1204 | 0.1973 | 2.3226 |
| C |  | -26.9375 | -0.4003 | 3.3585 |
| O |  | -21.7285 | -2.4992 | 0.3114 |
| C |  | -20.8881 | -1.8824 | -0.6943 |
| C |  | 4.5196 | 7.7981 | -4.6663 |
| C |  | 3.2289 | 6.0563 | -8.4636 |
| C |  | 1.9824 | 10.3012 | -7.1973 |
| C |  | 4.0378 | 8.4519 | -7.3258 |
| C |  | 6.0990 | -7.0335 | 3.2022 |
| C |  | 5.1334 | -5.8584 | 7.3915 |
| C |  | 3.7793 | -9.8226 | 5.6331 |
| C |  | 5.8681 | -8.0041 | 5.8002 |
| C |  | -11.5923 | 0.9045 | 17.5638 |
| C |  | -7.9616 | -1.7532 | 17.5580 |
| C |  | -7.4961 | 2.6959 | 16.9218 |
| C |  | -8.9041 | 0.7656 | 18.4335 |
| C |  | -12.3931 | 15.7862 | 7.0550 |
| C |  | -8.3607 | 16.3754 | 5.1223 |
| C |  | -12.1135 | 16.8449 | 2.6694 |
| C |  | -10.9945 | 17.4073 | 5.2062 |
| C |  | -24.5265 | -7.3934 | 7.5398 |
| C |  | -23.3408 | -10.7025 | 4.6977 |
| C |  | -21.3380 | -10.3397 | 8.7293 |
| C |  | -23.8403 | -10.1357 | 7.4234 |
| C |  | -10.3937 | -1.6531 | -17.7263 |
| C |  | -14.6646 | -0.3737 | -17.0915 |
| C |  | -11.3602 | 2.7228 | -17.2859 |
| C |  | -12.2868 | 0.3202 | -18.4580 |
| C |  | -26.1581 | 8.0474 | -2.1043 |
| C |  | -25.1101 | 8.0804 | -6.4949 |
| C |  | -26.9605 | 4.4053 | -4.6384 |
| C |  | -27.0804 | 7.2319 | -4.6514 |
| C |  | -6.6880 | -15.6778 | -6.6270 |
| C |  | -9.8569 | -17.1168 | -3.7519 |
| C |  | -11.0288 | -15.4868 | -7.8018 |
| C |  | -9.1237 | -17.1095 | -6.4826 |
| C |  | -7.2835 | 5.2407 | -9.2554 |
| H |  | 1.0879 | 3.9348 | -7.2001 |
| H |  | 1.1524 | 2.4543 | -5.2051 |
| H |  | 3.0916 | 5.5710 | -2.9329 |
| H |  | -0.9860 | 9.7743 | -7.5923 |
| H |  | -2.5071 | 8.4087 | -9.0099 |
| H |  | 0.3827 | 5.1807 | -9.0359 |
| H |  | 2.5370 | 7.7983 | -2.2921 |
| H |  | 0.6231 | 9.0648 | -1.3335 |
| H |  | -0.6727 | 10.1516 | -5.3143 |
| H |  | 3.0855 | -3.4706 | 6.5775 |
| H |  | 3.0998 | -1.7157 | 4.8284 |
| H |  | 4.8099 | -4.5177 | 2.0017 |
| H |  | 0.8030 | -9.3084 | 6.2420 |
| H |  | -0.6956 | -8.0182 | 7.7494 |
| H |  | 2.2730 | -4.8766 | 8.0384 |
| H |  | 3.6997 | -6.3545 | 1.1621 |
| H |  | 1.7231 | -7.4914 | 0.2057 |
| H |  | 1.0390 | -9.5175 | 3.9594 |
| H |  | -8.8856 | -3.4252 | 15.2041 |
| H |  | -11.0012 | -4.2007 | 14.1595 |
| H |  | -12.9158 | -0.4573 | 15.1864 |
| H |  | -6.2089 | 2.4132 | 14.2015 |
| H |  | -4.7473 | 0.8239 | 12.9856 |
| H |  | -13.8066 | 4.1108 | -12.9459 |
| H |  | -6.7391 | -2.5138 | 14.8935 |
| H |  | -12.6807 | 1.8593 | 14.9092 |
| H |  | -12.3749 | 3.7441 | 13.3188 |
| H |  | -8.1283 | 3.7976 | 14.1545 |
| H |  | -4.4830 | -0.8898 | 11.3755 |
| H |  | -0.9291 | -3.1618 | 9.1622 |
| H |  | -4.3132 | -4.4301 | 12.6146 |
| H |  | -1.0540 | -6.8351 | 10.1002 |
| H |  | -7.3976 | 13.5654 | 5.7207 |
| H |  | -7.7234 | 11.7714 | 7.4073 |
| H |  | -11.8825 | 12.8898 | 7.8384 |
| H |  | -11.4131 | 14.6143 | 0.7266 |
| H |  | -9.4030 | 13.5377 | -0.2585 |
| H |  | -7.2609 | 14.0622 | 3.4653 |
| H |  | -13.8339 | 13.1621 | 6.5723 |
| H |  | -15.4175 | 12.1588 | 4.9435 |
| H |  | -13.5063 | 14.3137 | 1.7129 |
| H |  | -9.3667 | 9.5517 | 7.6136 |
| H |  | -9.6789 | 5.6211 | 10.2121 |
| H |  | -10.5573 | 10.4627 | 11.1156 |
| H |  | -1.9790 | 9.0543 | -1.3458 |
| H |  | -5.6285 | 10.9313 | 1.0041 |
| H |  | -2.8315 | 12.4406 | -2.8573 |
| H |  | -6.6656 | 14.1769 | -0.6779 |
| H |  | -11.1201 | 6.4651 | 13.6076 |
| H |  | 2.1221 | -1.1459 | 2.6167 |
| H |  | -22.4661 | -8.8213 | 2.4933 |
| H |  | -23.0181 | -6.5653 | 1.6169 |
| H |  | -23.7733 | -5.1538 | 5.6383 |
| H |  | -18.5610 | -9.9926 | 7.5759 |
| H |  | -17.2426 | -10.6580 | 5.5790 |
| H |  | -20.7704 | -10.3082 | 3.0957 |
| H |  | -22.6967 | -4.9726 | 7.7127 |
| H |  | -20.6948 | -4.2081 | 8.9678 |
| H |  | -19.1298 | -8.2435 | 8.9875 |
| H |  | -19.3953 | -4.7418 | 11.3528 |
| H |  | -14.0498 | -11.6050 | -2.4276 |
| H |  | -5.8964 | -11.6748 | -0.8697 |
| H |  | -16.9723 | -10.0137 | 0.9224 |
| H |  | -14.7047 | -14.3928 | 0.0812 |
| H |  | -17.3403 | -12.6632 | 3.6167 |
| H |  | -14.9949 | -1.8915 | -14.4761 |
| H |  | -14.1306 | -3.9401 | -13.3686 |
| H |  | -10.2543 | -3.3274 | -15.1933 |
| H |  | -11.9053 | 3.8114 | -14.5161 |
| H |  | -15.5290 | 0.3677 | -14.2707 |
| H |  | -8.5645 | -1.7323 | -15.3087 |
| H |  | -7.0603 | -0.2425 | -14.0122 |
| H |  | -9.7031 | 3.1081 | -14.7372 |
| H |  | -12.0847 | -4.4891 | -11.6020 |
| H |  | -5.5951 | 1.9539 | -14.2954 |
| H |  | -10.6626 | -7.9729 | -8.7515 |
| H |  | -10.5293 | -7.2625 | -13.7018 |
| H |  | -2.2652 | 4.4345 | -12.0333 |
| H |  | -7.1913 | 3.8352 | -11.3996 |
| H |  | -20.6730 | 9.6968 | -4.3870 |
| H |  | -22.1602 | 8.5804 | -6.0322 |
| H |  | -23.3111 | 8.5254 | -1.1617 |
| H |  | -24.6367 | 2.5978 | -5.3297 |
| H |  | -22.8695 | 2.3686 | -7.0571 |
| H |  | -22.5509 | 6.6758 | -7.3472 |
| H |  | -24.2745 | 6.6095 | -0.2099 |
| H |  | -23.9123 | 4.3350 | 0.7208 |
| H |  | -25.1161 | 2.5712 | -3.0441 |
| H |  | -19.2172 | 9.0465 | -2.2817 |
| H |  | -25.4813 | 1.9593 | 1.0537 |
| H |  | -15.7609 | 10.8857 | 0.3569 |
| H |  | -20.7375 | 11.3927 | 0.3059 |
| H |  | -16.8467 | 1.7237 | -10.2921 |
| H |  | -20.4355 | 5.2043 | -10.4700 |
| H |  | -25.2417 | -2.3089 | 3.1019 |
| H |  | -22.6114 | 0.0012 | -0.4717 |
| H |  | -16.6471 | 4.2212 | -13.1426 |
| H |  | -8.9339 | -10.6585 | -10.8089 |
| H |  | -22.1475 | -4.1097 | 1.8032 |
| H |  | -2.4532 | -9.4752 | 1.5215 |
| H |  | -2.5469 | -12.4919 | -2.4683 |
| H |  | -16.0878 | -5.2476 | 9.5585 |
| H |  | -15.8689 | -3.1618 | 14.1003 |
| H |  | -17.2838 | 13.0232 | 3.1046 |
| H |  | -12.5960 | -3.4444 | 12.1691 |
| H |  | -3.8274 | 6.1388 | -9.0071 |
| H |  | 0.8538 | -10.0450 | -0.2673 |
| H |  | 1.4866 | 2.5594 | -0.2294 |
| H |  | 4.9415 | -1.0500 | 0.0743 |
| H |  | 4.2860 | 2.8042 | -2.6774 |
| H |  | -9.1358 | -15.1294 | -1.5786 |
| H |  | -7.1281 | -13.9450 | -0.7252 |
| H |  | -5.6037 | -13.5598 | -4.7579 |
| H |  | -12.7371 | -13.2660 | -6.6721 |
| H |  | -14.1441 | -12.7957 | -4.6829 |
| H |  | -11.4121 | -15.0366 | -2.1821 |
| H |  | -6.2456 | -12.6808 | -6.8449 |
| H |  | -7.1068 | -10.7417 | -8.1410 |
| H |  | -11.0816 | -12.4543 | -8.1122 |
| H |  | -20.3931 | 2.9290 | -7.4183 |
| H |  | -12.2327 | -4.7578 | -7.9814 |
| H |  | -12.3353 | -6.5623 | -7.9342 |
| H |  | -10.7202 | -5.7398 | -7.8729 |
| H |  | -8.8821 | -10.4416 | -14.4665 |
| H |  | -10.3766 | -9.4376 | -14.6195 |
| H |  | -8.7569 | -8.6384 | -14.4630 |
| H |  | -8.8645 | 6.0746 | 6.6709 |
| H |  | -8.4094 | 5.4759 | 8.3161 |
| H |  | -10.1475 | 5.4919 | 7.8016 |
| H |  | -11.5814 | 9.9833 | 14.6000 |
| H |  | -10.3890 | 10.7054 | 13.4504 |
| H |  | -12.1058 | 10.4398 | 12.9315 |
| H |  | 6.7436 | -0.0522 | -2.9611 |
| H |  | 6.7823 | -0.2371 | -1.1632 |
| H |  | 5.7202 | -1.3111 | -2.1670 |
| H |  | -0.3286 | 1.5199 | 2.8061 |
| H |  | 0.4649 | 2.7552 | 1.7536 |
| H |  | -0.4914 | 1.3877 | 1.0170 |
| H |  | -12.5069 | -4.9522 | 8.8654 |
| H |  | -13.9237 | -6.0447 | 9.1231 |
| H |  | -14.1678 | -4.3855 | 8.4343 |
| H |  | -19.4595 | -3.4158 | 14.7732 |
| H |  | -17.7929 | -3.9500 | 15.2224 |
| H |  | -18.0549 | -2.3168 | 14.4801 |
| H |  | -2.0381 | 2.3314 | -15.0204 |
| H |  | -1.8589 | 3.9790 | -14.3002 |
| H |  | -1.6040 | 2.5053 | -13.2750 |
| H |  | -6.0444 | -9.6756 | 2.1987 |
| H |  | -4.3803 | -10.0912 | 2.7677 |
| H |  | -4.6450 | -8.6359 | 1.7208 |
| H |  | 1.0919 | -12.5147 | -2.9454 |
| H |  | -0.3454 | -13.4524 | -2.3754 |
| H |  | -0.5327 | -12.1807 | -3.6598 |
| H |  | -4.6494 | 15.5699 | -3.4060 |
| H |  | -3.0990 | 14.7934 | -2.8977 |
| H |  | -4.2012 | 13.9465 | -4.0618 |
| H |  | -3.7821 | 7.8430 | 1.6075 |
| H |  | -4.1111 | 9.5013 | 2.2383 |
| H |  | -5.3401 | 8.6616 | 1.1988 |
| H |  | -3.4790 | -7.9990 | 12.6293 |
| H |  | -3.4279 | -6.4445 | 13.5492 |
| H |  | -4.7233 | -6.7276 | 12.3129 |
| H |  | -17.9587 | 0.2339 | -7.1415 |
| H |  | -17.7042 | 0.0196 | -8.9179 |
| H |  | -16.6678 | 1.1773 | -7.9839 |
| H |  | -19.3088 | 6.7039 | -13.6155 |
| H |  | -20.6014 | 5.7769 | -12.7583 |
| H |  | -19.5447 | 6.9270 | -11.8375 |
| H |  | -15.9781 | -16.0289 | 3.0995 |
| H |  | -15.9869 | -15.9697 | 1.2936 |
| H |  | -14.5285 | -15.4105 | 2.2145 |
| H |  | -15.7018 | -8.3765 | -2.0929 |
| H |  | -17.1427 | -8.9935 | -1.1931 |
| H |  | -15.6770 | -8.4306 | -0.2866 |
| H |  | -1.5445 | 0.4170 | 9.3077 |
| H |  | -0.3985 | -0.8072 | 9.9772 |
| H |  | -1.2939 | -1.1503 | 8.4380 |
| H |  | -15.6182 | 8.5620 | -2.4672 |
| H |  | -15.1798 | 10.1885 | -1.8119 |
| H |  | -15.3783 | 8.7787 | -0.6892 |
| H |  | -20.8828 | 13.6932 | 3.1502 |
| H |  | -21.1453 | 13.4584 | 1.3780 |
| H |  | -21.2999 | 12.0538 | 2.5138 |
| H |  | -27.6997 | 0.3742 | 3.5134 |
| H |  | -27.3893 | -1.3296 | 2.9982 |
| H |  | -26.3467 | -0.5587 | 4.2655 |
| H |  | -20.1293 | -2.6589 | -0.8546 |
| H |  | -21.4617 | -1.6973 | -1.6076 |
| H |  | -20.4363 | -0.9656 | -0.3037 |
| C |  | 4.9431 | 7.3403 | -3.2649 |
| H |  | 5.2127 | 7.3344 | -5.3992 |
| H |  | 4.6701 | 8.8966 | -4.7436 |
| C |  | 2.9411 | 4.6693 | -9.0506 |
| H |  | 3.2126 | 6.8003 | -9.2875 |
| H |  | 4.2659 | 6.0502 | -8.0675 |
| C |  | 0.9658 | 11.4359 | -7.0179 |
| H |  | 2.9324 | 10.6190 | -6.7172 |
| H |  | 2.2016 | 10.1849 | -8.2790 |
| H |  | 4.8788 | 7.7455 | -7.3185 |
| H |  | 3.8307 | 8.6957 | -8.3769 |
| H |  | 4.3877 | 9.3775 | -6.8469 |
| C |  | 6.2726 | -6.6740 | 1.7213 |
| H |  | 6.8624 | -6.4852 | 3.7930 |
| H |  | 6.3299 | -8.1135 | 3.3200 |
| C |  | 4.8949 | -4.5900 | 8.2193 |
| H |  | 5.1340 | -6.7345 | 8.0724 |
| H |  | 6.1548 | -5.7963 | 6.9602 |
| C |  | 2.7292 | -10.9235 | 5.4377 |
| H |  | 4.6712 | -10.0923 | 5.0294 |
| H |  | 4.1068 | -9.8266 | 6.6938 |
| H |  | 6.6990 | -7.2860 | 5.8412 |
| H |  | 5.7531 | -8.4191 | 6.8103 |
| H |  | 6.1872 | -8.8285 | 5.1471 |
| H |  | -12.6123 | 0.9432 | 17.1625 |
| H |  | -11.5726 | 0.1048 | 18.3133 |
| H |  | -11.4081 | 1.8567 | 18.0738 |
| H |  | -7.6069 | -2.7089 | 17.1534 |
| H |  | -7.1110 | -1.2851 | 18.0669 |
| H |  | -8.7261 | -1.9836 | 18.3087 |
| H |  | -6.9764 | 3.4166 | 16.2789 |
| H |  | -8.1753 | 3.2677 | 17.5643 |
| H |  | -6.7376 | 2.2258 | 17.5585 |
| H |  | -9.4588 | 0.0068 | 18.9995 |
| H |  | -7.8547 | 0.7019 | 18.7474 |
| H |  | -9.2798 | 1.7464 | 18.7503 |
| H |  | -12.9143 | 15.1020 | 7.7355 |
| H |  | -11.6463 | 16.3243 | 7.6502 |
| H |  | -13.1321 | 16.5111 | 6.6956 |
| H |  | -7.3670 | 15.9145 | 5.0670 |
| H |  | -8.3701 | 17.2071 | 4.4086 |
| H |  | -8.4695 | 16.7888 | 6.1316 |
| H |  | -12.5369 | 16.5589 | 1.6989 |
| H |  | -12.9078 | 17.3490 | 3.2317 |
| H |  | -11.3183 | 17.5730 | 2.4729 |
| H |  | -10.5529 | 17.6013 | 6.1917 |
| H |  | -10.4434 | 18.0153 | 4.4779 |
| H |  | -12.0245 | 17.7842 | 5.2314 |
| H |  | -24.7346 | -6.3166 | 7.5483 |
| H |  | -25.3499 | -7.8753 | 6.9998 |
| H |  | -24.5517 | -7.7366 | 8.5802 |
| H |  | -23.0962 | -10.8750 | 3.6426 |
| H |  | -23.1564 | -11.6430 | 5.2292 |
| H |  | -24.4139 | -10.4850 | 4.7489 |
| H |  | -20.3443 | -10.3685 | 9.1927 |
| H |  | -22.0498 | -10.0509 | 9.5111 |
| H |  | -21.5759 | -11.3609 | 8.4104 |
| H |  | -24.8013 | -10.0355 | 6.9035 |
| H |  | -23.5507 | -11.1926 | 7.3706 |
| H |  | -24.0163 | -9.8992 | 8.4801 |
| H |  | -9.6754 | -2.4039 | -17.3752 |
| H |  | -11.1501 | -2.1835 | -18.3163 |
| H |  | -9.8519 | -0.9740 | -18.3944 |
| H |  | -15.5521 | -0.6332 | -16.5017 |
| H |  | -14.9049 | 0.5366 | -17.6524 |
| H |  | -14.5057 | -1.1865 | -17.8096 |
| H |  | -10.9990 | 3.6208 | -16.7701 |
| H |  | -10.6101 | 2.4620 | -18.0414 |
| H |  | -12.2862 | 2.9974 | -17.8037 |
| H |  | -12.6379 | -0.6247 | -18.8911 |
| H |  | -13.0245 | 1.0903 | -18.7157 |
| H |  | -11.3510 | 0.5828 | -18.9671 |
| H |  | -25.7402 | 8.3320 | -1.1311 |
| H |  | -26.3779 | 8.9763 | -2.6434 |
| H |  | -27.1051 | 7.5307 | -1.9118 |
| H |  | -24.3006 | 8.3690 | -7.1763 |
| H |  | -25.8692 | 7.5677 | -7.0970 |
| H |  | -25.5495 | 9.0054 | -6.1043 |
| H |  | -26.8479 | 3.3147 | -4.6121 |
| H |  | -27.7360 | 4.6670 | -3.9096 |
| H |  | -27.3272 | 4.6679 | -5.6375 |
| H |  | -27.1026 | 8.3289 | -4.6488 |
| H |  | -27.4166 | 6.9025 | -5.6426 |
| H |  | -27.8275 | 6.8899 | -3.9245 |
| H |  | -5.7742 | -15.0716 | -6.6469 |
| H |  | -6.4520 | -16.5952 | -6.0753 |
| H |  | -6.9188 | -15.9505 | -7.6629 |
| H |  | -10.1435 | -17.0509 | -2.6953 |
| H |  | -10.6593 | -17.6552 | -4.2695 |
| H |  | -8.9470 | -17.7258 | -3.8025 |
| H |  | -11.7488 | -14.8058 | -8.2715 |
| H |  | -10.3268 | -15.8027 | -8.5818 |
| H |  | -11.5888 | -16.3682 | -7.4682 |
| H |  | -8.3756 | -17.7175 | -5.9586 |
| H |  | -10.0800 | -17.6435 | -6.4178 |
| H |  | -8.8371 | -17.0830 | -7.5412 |
| H |  | -7.3968 | 5.8501 | -8.3494 |
| H |  | -7.8970 | 5.6316 | -10.0726 |
| H |  | -7.4997 | 4.1878 | -9.0503 |
| C |  | 24.5404 | -5.8796 | -5.9630 |
| C |  | 23.6379 | -5.2623 | -8.2719 |
| C |  | 25.0055 | -3.5649 | -6.9391 |
| C |  | 24.9092 | -5.1145 | -7.3159 |
| C |  | 23.1532 | -6.4477 | -6.2069 |
| C |  | 22.6588 | -6.1097 | -7.4844 |
| C |  | 21.4018 | -6.5429 | -7.8915 |
| C |  | 20.6298 | -7.3262 | -7.0235 |
| N |  | 20.3146 | -8.3380 | -4.7890 |
| C |  | 21.1224 | -7.6523 | -5.7485 |
| C |  | 22.3977 | -7.2123 | -5.3292 |
| C |  | 23.1131 | -3.8469 | -8.4371 |
| C |  | 23.8722 | -2.9107 | -7.7037 |
| C |  | 23.5472 | -1.5590 | -7.7355 |
| C |  | 22.4495 | -1.1338 | -8.4950 |
| N |  | 20.6107 | -1.6821 | -10.0727 |
| C |  | 21.6916 | -2.0699 | -9.2194 |
| C |  | 22.0269 | -3.4424 | -9.2001 |
| C |  | 24.7457 | -3.5147 | -5.4438 |
| C |  | 24.4963 | -4.7947 | -4.9056 |
| C |  | 24.2460 | -4.9525 | -3.5468 |
| C |  | 24.2307 | -3.8250 | -2.7158 |
| N |  | 24.5369 | -1.3730 | -2.4453 |
| C |  | 24.4699 | -2.5497 | -3.2566 |
| C |  | 24.7396 | -2.3876 | -4.6341 |
| C |  | 12.3226 | -16.4948 | -0.1895 |
| C |  | 12.6564 | -16.2342 | 2.3301 |
| C |  | 10.3036 | -16.5128 | 1.3757 |
| C |  | 11.8093 | -17.0232 | 1.2282 |
| C |  | 13.4462 | -15.5293 | 0.1432 |
| C |  | 13.6265 | -15.3840 | 1.5351 |
| C |  | 14.6117 | -14.5388 | 2.0340 |
| C |  | 15.4210 | -13.8241 | 1.1413 |
| N |  | 16.0870 | -13.3299 | -1.2010 |
| C |  | 15.2370 | -13.9693 | -0.2444 |
| C |  | 14.2441 | -14.8352 | -0.7552 |
| C |  | 11.6385 | -15.3707 | 3.0545 |
| C |  | 10.3355 | -15.5469 | 2.5430 |
| C |  | 9.2611 | -14.8544 | 3.0904 |
| C |  | 9.4841 | -13.9716 | 4.1546 |
| N |  | 11.0734 | -12.7806 | 5.6255 |
| C |  | 10.7871 | -13.7807 | 4.6459 |
| C |  | 11.8801 | -14.4895 | 4.0993 |
| C |  | 10.0229 | -15.7609 | 0.0861 |
| C |  | 11.1387 | -15.7525 | -0.7772 |
| C |  | 11.0768 | -15.0982 | -2.0026 |
| C |  | 9.8947 | -14.4440 | -2.3738 |
| N |  | 7.6102 | -13.6829 | -1.7722 |
| C |  | 8.7894 | -14.4440 | -1.5055 |
| C |  | 8.8464 | -15.1135 | -0.2623 |
| C |  | 9.5080 | -2.9985 | 15.9697 |
| C |  | 9.3765 | -5.4863 | 15.4030 |
| C |  | 11.6516 | -4.3454 | 15.6331 |
| C |  | 10.1866 | -4.4130 | 16.2666 |
| C |  | 8.3574 | -3.3133 | 15.0303 |
| C |  | 8.2890 | -4.6867 | 14.7130 |
| C |  | 7.2977 | -5.1646 | 13.8631 |
| C |  | 6.3660 | -4.2697 | 13.3189 |
| N |  | 5.4706 | -1.9473 | 13.1991 |
| C |  | 6.4367 | -2.9035 | 13.6381 |
| C |  | 7.4403 | -2.4148 | 14.5061 |
| C |  | 10.3864 | -6.0133 | 14.3995 |
| C |  | 11.6437 | -5.3865 | 14.5323 |
| C |  | 12.7035 | -5.7480 | 13.7078 |
| C |  | 12.5108 | -6.7381 | 12.7359 |
| N |  | 11.0198 | -8.4280 | 11.6841 |
| C |  | 11.2558 | -7.3570 | 12.6022 |
| C |  | 10.1792 | -6.9983 | 13.4443 |
| C |  | 11.7576 | -2.9459 | 15.0526 |
| C |  | 10.5766 | -2.2000 | 15.2502 |
| C |  | 10.4844 | -0.8897 | 14.7937 |
| C |  | 11.5787 | -0.3124 | 14.1367 |
| N |  | 13.8303 | -0.5553 | 13.1463 |
| C |  | 12.7484 | -1.0634 | 13.9300 |
| C |  | 12.8469 | -2.3946 | 14.3925 |
| C |  | 11.4478 | -8.3791 | 10.4608 |
| C |  | 11.1999 | -9.5147 | 9.5481 |
| C |  | 11.7479 | -9.4861 | 8.2402 |
| C |  | 11.5281 | -10.5260 | 7.3316 |
| C |  | 10.7513 | -11.6292 | 7.7046 |
| C |  | 10.2133 | -11.6636 | 9.0153 |
| C |  | 10.4335 | -10.6233 | 9.9247 |
| C |  | 10.4620 | -12.7349 | 6.7667 |
| C |  | 22.0234 | 7.4746 | 8.6528 |
| C |  | 23.5816 | 5.9596 | 7.3095 |
| C |  | 22.8579 | 8.2158 | 6.3545 |
| C |  | 23.3069 | 7.4837 | 7.7014 |
| C |  | 21.6656 | 6.0065 | 8.8034 |
| C |  | 22.5300 | 5.1715 | 8.0644 |
| C |  | 22.3648 | 3.7909 | 8.0837 |
| C |  | 21.3236 | 3.2342 | 8.8376 |
| N |  | 19.4316 | 3.5505 | 10.4164 |
| C |  | 20.4605 | 4.0691 | 9.5680 |
| C |  | 20.6327 | 5.4714 | 9.5602 |
| C |  | 23.3322 | 5.8950 | 5.8129 |
| C |  | 22.9399 | 7.1438 | 5.2864 |
| C |  | 22.6705 | 7.2842 | 3.9294 |
| C |  | 22.7981 | 6.1739 | 3.0847 |
| N |  | 23.1894 | 3.7475 | 2.8118 |
| C |  | 23.1781 | 4.9294 | 3.6153 |
| C |  | 23.4513 | 4.7814 | 4.9934 |
| C |  | 21.4147 | 8.6207 | 6.5985 |
| C |  | 20.9565 | 8.2121 | 7.8687 |
| C |  | 19.6559 | 8.4927 | 8.2731 |
| C |  | 18.8036 | 9.1941 | 7.4101 |
| N |  | 18.3913 | 10.1985 | 5.1850 |
| C |  | 19.2619 | 9.5928 | 6.1430 |
| C |  | 20.5812 | 9.3061 | 5.7262 |
| C |  | 18.6108 | 2.6361 | 10.0015 |
| C |  | 17.5710 | 2.1088 | 10.9097 |
| C |  | 16.7405 | 1.0461 | 10.4705 |
| C |  | 15.7311 | 0.5208 | 11.2836 |
| C |  | 15.5202 | 1.0450 | 12.5640 |
| C |  | 16.3558 | 2.1009 | 13.0070 |
| C |  | 17.3670 | 2.6251 | 12.1944 |
| C |  | 23.6361 | -1.1210 | -1.5469 |
| C |  | 23.7423 | 0.0945 | -0.7131 |
| C |  | 22.8082 | 0.3005 | 0.3341 |
| C |  | 22.8542 | 1.4403 | 1.1432 |
| C |  | 23.8383 | 2.4121 | 0.9281 |
| C |  | 24.7769 | 2.2030 | -0.1133 |
| C |  | 24.7320 | 1.0618 | -0.9217 |
| C |  | 17.2609 | -11.4446 | -2.1214 |
| C |  | 23.9115 | 3.6450 | 1.7415 |
| C |  | 14.4463 | 0.5433 | 13.4482 |
| C |  | 16.3656 | -12.0659 | -1.1228 |
| C |  | -3.0616 | 5.9899 | 7.2069 |
| C |  | -4.1734 | 4.8501 | 5.1876 |
| C |  | -3.7724 | 3.5240 | 7.3534 |
| C |  | -4.2162 | 4.9576 | 6.7909 |
| C |  | -2.5479 | 6.5239 | 5.8823 |
| C |  | -3.1158 | 5.8518 | 4.7758 |
| C |  | -2.6944 | 6.1532 | 3.4856 |
| C |  | -1.7221 | 7.1399 | 3.2805 |
| N |  | -0.2674 | 8.9170 | 4.2425 |
| C |  | -1.1840 | 7.8289 | 4.3800 |
| C |  | -1.6012 | 7.5200 | 5.6934 |
| C |  | -3.7465 | 3.4200 | 4.9196 |
| C |  | -3.6209 | 2.6683 | 6.1102 |
| C |  | -3.3178 | 1.3121 | 6.0546 |
| C |  | -3.1119 | 0.6940 | 4.8156 |
| N |  | -2.8923 | 0.9059 | 2.3472 |
| C |  | -3.1854 | 1.4548 | 3.6378 |
| C |  | -3.4904 | 2.8330 | 3.6876 |
| C |  | -2.3886 | 3.7668 | 7.9346 |
| C |  | -1.9922 | 5.1194 | 7.8384 |
| C |  | -0.7138 | 5.5042 | 8.2320 |
| C |  | 0.1667 | 4.5575 | 8.7709 |
| N |  | 0.6604 | 2.1817 | 9.2946 |
| C |  | -0.2323 | 3.2157 | 8.8702 |
| C |  | -1.5127 | 2.8132 | 8.4338 |
| C |  | 1.2879 | 2.2388 | 10.4263 |
| C |  | 2.2282 | 1.1590 | 10.7948 |
| C |  | -2.4819 | -2.4861 | -3.2790 |
| C |  | -2.7379 | -1.9196 | -1.9378 |
| C |  | 2.4240 | -10.3272 | -3.9653 |
| C |  | 3.5665 | -11.2280 | -3.7001 |
| C |  | -2.0493 | -0.7443 | -1.5344 |
| C |  | -2.2484 | -0.1802 | -0.2695 |
| C |  | -3.1622 | -0.7599 | 0.6202 |
| C |  | -3.8501 | -1.9306 | 0.2129 |
| C |  | -3.6405 | -2.5051 | -1.0453 |
| C |  | -3.4518 | -0.1972 | 1.9539 |
| C |  | 9.9483 | 3.5592 | -15.6481 |
| C |  | 9.3340 | 5.9136 | -14.8683 |
| C |  | 11.7881 | 5.2232 | -15.0405 |
| C |  | 10.3714 | 5.0949 | -15.7667 |
| C |  | 8.7076 | 3.5685 | -14.7730 |
| C |  | 8.3658 | 4.8700 | -14.3483 |
| C |  | 7.2472 | 5.0799 | -13.5494 |
| C |  | 6.4651 | 3.9846 | -13.1593 |
| N |  | 6.0312 | 1.5305 | -13.2726 |
| C |  | 6.8141 | 2.6881 | -13.5742 |
| C |  | 7.9435 | 2.4734 | -14.3972 |
| C |  | 10.1716 | 6.4873 | -13.7388 |
| C |  | 11.5279 | 6.1133 | -13.8420 |
| C |  | 12.4549 | 6.5501 | -12.9021 |
| C |  | 12.0320 | 7.3759 | -11.8528 |
| N |  | 10.1956 | 8.4775 | -10.6233 |
| C |  | 10.6783 | 7.7385 | -11.7487 |
| C |  | 9.7317 | 7.2927 | -12.6973 |
| C |  | 12.1226 | 3.8108 | -14.5937 |
| C |  | 11.1064 | 2.8927 | -14.9328 |
| C |  | 11.2323 | 1.5478 | -14.6028 |
| C |  | 12.3823 | 1.1085 | -13.9337 |
| N |  | 14.5129 | 1.6499 | -12.7939 |
| C |  | 13.3875 | 2.0282 | -13.5890 |
| C |  | 13.2634 | 3.3958 | -13.9217 |
| C |  | 5.4976 | 1.3443 | -12.1054 |
| C |  | 15.3049 | 0.6815 | -13.1298 |
| C |  | 4.7130 | 0.1177 | -11.8504 |
| C |  | 16.4241 | 0.3121 | -12.2367 |
| C |  | 3.9108 | 0.0273 | -10.6854 |
| C |  | 3.1976 | -1.1367 | -10.3760 |
| C |  | 3.2586 | -2.2424 | -11.2308 |
| C |  | 4.0403 | -2.1413 | -12.4105 |
| C |  | 4.7572 | -0.9812 | -12.7169 |
| C |  | 17.4030 | -0.6098 | -12.6856 |
| C |  | 18.4677 | -1.0039 | -11.8682 |
| C |  | 18.5845 | -0.4871 | -10.5728 |
| C |  | 17.6096 | 0.4415 | -10.1268 |
| C |  | 16.5454 | 0.8357 | -10.9444 |
| C |  | 9.3101 | 17.1573 | 0.6685 |
| C |  | 9.7729 | 17.1160 | -1.8448 |
| C |  | 7.3821 | 16.8686 | -0.9821 |
| C |  | 8.7499 | 17.6564 | -0.7422 |
| C |  | 10.6068 | 16.4401 | 0.3351 |
| C |  | 10.8608 | 16.4194 | -1.0526 |
| C |  | 12.0056 | 15.8072 | -1.5511 |
| C |  | 12.9008 | 15.1966 | -0.6632 |
| N |  | 13.5559 | 14.6703 | 1.6748 |
| C |  | 12.6406 | 15.2124 | 0.7179 |
| C |  | 11.4869 | 15.8476 | 1.2295 |
| C |  | 8.9704 | 16.1163 | -2.6596 |
| C |  | 7.6440 | 15.9978 | -2.1942 |
| C |  | 6.7472 | 15.1406 | -2.8227 |
| C |  | 7.1793 | 14.3751 | -3.9129 |
| N |  | 8.9726 | 13.7739 | -5.5159 |
| C |  | 8.5060 | 14.4833 | -4.3642 |
| C |  | 9.4133 | 15.3704 | -3.7433 |
| C |  | 7.2136 | 16.0030 | 0.2545 |
| C |  | 8.2733 | 16.1718 | 1.1701 |
| C |  | 8.2935 | 15.4584 | 2.3635 |
| C |  | 7.2566 | 14.5590 | 2.6455 |
| N |  | 5.2418 | 13.3470 | 1.8846 |
| C |  | 6.2180 | 14.3748 | 1.7173 |
| C |  | 6.1825 | 15.1112 | 0.5120 |
| C |  | 9.2628 | 11.7978 | -6.8531 |
| C |  | 14.0464 | 13.4775 | 1.5392 |
| C |  | 4.4182 | 13.3067 | 2.8819 |
| C |  | 14.9968 | 12.9505 | 2.5408 |
| C |  | 3.4713 | 12.1749 | 2.9889 |
| C |  | 15.5764 | 11.6715 | 2.3415 |
| C |  | 16.4670 | 11.1186 | 3.2676 |
| C |  | 16.8059 | 11.8304 | 4.4239 |
| C |  | 16.2337 | 13.1127 | 4.6189 |
| C |  | 15.3436 | 13.6663 | 3.6925 |
| C |  | 9.0352 | 10.4048 | -6.9895 |
| C |  | 9.4618 | 9.7003 | -8.1200 |
| C |  | 10.1367 | 10.3695 | -9.1470 |
| C |  | 10.3634 | 11.7622 | -9.0120 |
| C |  | 9.9310 | 12.4677 | -7.8843 |
| C |  | 2.4711 | 12.1888 | 3.9931 |
| C |  | 1.5689 | 11.1299 | 4.1425 |
| C |  | 1.6420 | 10.0226 | 3.2897 |
| C |  | 2.6357 | 10.0139 | 2.2781 |
| C |  | 3.5367 | 11.0728 | 2.1286 |
| C |  | 10.6309 | 9.6661 | -10.3498 |
| C |  | 2.5576 | -3.5106 | -10.9397 |
| C |  | 0.7294 | 8.8674 | 3.4140 |
| C |  | 4.2643 | -11.1332 | -2.4702 |
| C |  | 5.3726 | -11.9440 | -2.1953 |
| C |  | 5.8138 | -12.8746 | -3.1417 |
| C |  | 5.1052 | -12.9788 | -4.3660 |
| C |  | 4.0000 | -12.1693 | -4.6425 |
| C |  | 2.8876 | 0.3871 | 9.8317 |
| C |  | 3.7928 | -0.6020 | 10.2314 |
| C |  | 4.0671 | -0.8475 | 11.6014 |
| C |  | 3.3964 | -0.0834 | 12.5629 |
| C |  | 2.4928 | 0.9076 | 12.1635 |
| C |  | 17.7242 | 11.2785 | 5.4432 |
| C |  | 5.0564 | -1.8816 | 11.9734 |
| C |  | 19.6786 | -0.8803 | -9.6604 |
| C |  | 6.9967 | -13.7315 | -2.9126 |
| C |  | 17.6220 | -10.0808 | -1.9770 |
| C |  | 18.4546 | -9.4415 | -2.9014 |
| C |  | 18.9517 | -10.1489 | -4.0019 |
| C |  | 18.5968 | -11.5141 | -4.1427 |
| C |  | 17.7656 | -12.1541 | -3.2171 |
| C |  | 19.8164 | -9.5112 | -5.0181 |
| C |  | -1.1729 | -8.2734 | -8.4180 |
| C |  | -2.9010 | -7.5422 | -6.6615 |
| C |  | -2.4751 | -6.0836 | -8.7380 |
| C |  | -2.6176 | -7.6009 | -8.2427 |
| C |  | -0.8019 | -8.7212 | -7.0166 |
| C |  | -1.7389 | -8.2949 | -6.0484 |
| C |  | -1.5417 | -8.6009 | -4.7054 |
| C |  | -0.4123 | -9.3286 | -4.3117 |
| N |  | 1.6399 | -10.5772 | -4.9673 |
| C |  | 0.5146 | -9.7512 | -5.2780 |
| C |  | 0.3171 | -9.4519 | -6.6435 |
| C |  | -2.8717 | -6.0611 | -6.3348 |
| C |  | -2.7564 | -5.2616 | -7.4950 |
| C |  | -2.8214 | -3.8756 | -7.4031 |
| C |  | -2.9490 | -3.2664 | -6.1501 |
| N |  | -3.1785 | -3.4997 | -3.6904 |
| C |  | -2.9917 | -4.0606 | -4.9922 |
| C |  | -2.9524 | -5.4702 | -5.0809 |
| C |  | -0.9966 | -5.9326 | -9.0647 |
| C |  | -0.2776 | -7.1299 | -8.8511 |
| C |  | 1.1076 | -7.1497 | -8.9839 |
| C |  | 1.7842 | -5.9949 | -9.3944 |
| N |  | 1.7205 | -3.5817 | -9.9526 |
| C |  | 1.0648 | -4.8112 | -9.6254 |
| C |  | -0.3333 | -4.7694 | -9.4349 |
| C |  | 8.7926 | 12.4956 | -5.6379 |
| O |  | 3.9032 | 1.1840 | -9.9286 |
| C |  | 3.2133 | 1.1527 | -8.6546 |
| O |  | 4.0166 | -3.2837 | -13.1894 |
| C |  | 4.7912 | -3.2785 | -14.4130 |
| O |  | 17.0361 | 0.6026 | 9.1950 |
| C |  | 16.2027 | -0.4430 | 8.6379 |
| O |  | 16.0645 | 2.5363 | 14.2863 |
| C |  | 16.8588 | 3.6219 | 14.8233 |
| O |  | 19.1459 | -12.1155 | -5.2599 |
| C |  | 18.8391 | -13.5121 | -5.4898 |
| O |  | 17.0799 | -9.4795 | -0.8565 |
| C |  | 17.3568 | -8.0729 | -0.6490 |
| O |  | 4.4822 | -1.4340 | 9.3716 |
| C |  | 4.4152 | -1.1470 | 7.9531 |
| O |  | 1.7854 | 1.7284 | 13.0206 |
| C |  | 1.9600 | 1.5228 | 14.4440 |
| O |  | 17.8264 | 0.8972 | -8.8403 |
| O |  | 17.1893 | -1.0585 | -13.9758 |
| C |  | 18.1391 | -2.0048 | -14.5235 |
| O |  | 3.7466 | -10.1887 | -1.6071 |
| C |  | 4.4011 | -10.0191 | -0.3247 |
| O |  | 5.6176 | -13.9368 | -5.2221 |
| C |  | 4.9751 | -14.0995 | -6.5095 |
| O |  | 25.7044 | 3.2208 | -0.2340 |
| C |  | 26.7105 | 3.0937 | -1.2681 |
| O |  | 21.8825 | -0.7186 | 0.4596 |
| C |  | 20.8714 | -0.5843 | 1.4880 |
| O |  | 9.4672 | -12.7973 | 9.2777 |
| C |  | 8.8982 | -12.9355 | 10.6026 |
| O |  | 8.3578 | 9.8439 | -5.9217 |
| C |  | 8.1815 | 8.4071 | -5.9200 |
| O |  | 11.0349 | 12.3193 | -10.0841 |
| C |  | 11.3254 | 13.7376 | -10.0312 |
| O |  | -4.7284 | -2.4186 | 1.1638 |
| C |  | -5.4195 | -3.6562 | 0.8664 |
| O |  | -1.2155 | -0.2392 | -2.5083 |
| C |  | -0.3803 | 0.8968 | -2.1700 |
| O |  | 12.4947 | -8.3530 | 7.9768 |
| C |  | 13.0899 | -8.2315 | 6.6618 |
| O |  | 15.1774 | 11.0591 | 1.1687 |
| C |  | 15.6829 | 9.7285 | 0.8990 |
| O |  | 16.6414 | 13.7281 | 5.7879 |
| C |  | 16.1031 | 15.0408 | 6.0786 |
| O |  | 2.4953 | 13.3235 | 4.7823 |
| C |  | 1.4794 | 13.4506 | 5.8067 |
| O |  | 2.6140 | 8.8788 | 1.4895 |
| C |  | 3.5859 | 8.7847 | 0.4193 |
| C |  | 25.5108 | -6.9905 | -5.5612 |
| C |  | 23.9232 | -5.8896 | -9.6368 |
| C |  | 26.3407 | -2.8989 | -7.2725 |
| C |  | 26.1834 | -5.6394 | -7.9541 |
| C |  | 12.8106 | -17.5809 | -1.1483 |
| C |  | 13.4049 | -17.1194 | 3.3277 |
| C |  | 9.2638 | -17.6106 | 1.5965 |
| C |  | 11.9323 | -18.5303 | 1.3686 |
| C |  | 9.0057 | -2.2486 | 17.2034 |
| C |  | 8.7702 | -6.6380 | 16.2057 |
| C |  | 12.7948 | -4.6021 | 16.6155 |
| C |  | 10.2067 | -4.7523 | 17.7468 |
| C |  | 22.2295 | 8.1179 | 10.0246 |
| C |  | 24.9841 | 5.4481 | 7.6400 |
| C |  | 23.6977 | 9.4335 | 5.9677 |
| C |  | 24.5099 | 8.1444 | 8.3516 |
| C |  | -3.5719 | 7.0990 | 8.1484 |
| C |  | -5.5416 | 5.1753 | 4.5432 |
| C |  | -4.7743 | 2.9485 | 8.3733 |
| C |  | -5.5864 | 5.3852 | 7.2979 |
| C |  | 9.6599 | 2.8622 | -16.9781 |
| C |  | 8.5864 | 7.0305 | -15.5975 |
| C |  | 12.9119 | 5.7914 | -15.9073 |
| C |  | 10.4064 | 5.5809 | -17.2051 |
| C |  | 9.5546 | 18.2614 | 1.6968 |
| C |  | 10.3683 | 18.1888 | -2.7572 |
| C |  | 6.1522 | 17.7507 | -1.1931 |
| C |  | 8.5741 | 19.1637 | -0.8000 |
| C |  | -1.1835 | -9.4372 | -9.4318 |
| C |  | -4.2576 | -8.1820 | -6.2836 |
| C |  | -3.4086 | -5.7590 | -9.9188 |
| C |  | -3.7044 | -8.3655 | -8.9857 |
| C |  | 16.8619 | 1.8233 | -8.2831 |
| H |  | 21.0125 | -6.2772 | -8.8725 |
| H |  | 19.6418 | -7.6607 | -7.3304 |
| H |  | 22.7606 | -7.4646 | -4.3356 |
| H |  | 24.1309 | -0.8330 | -7.1729 |
| H |  | 22.1942 | -0.0773 | -8.5299 |
| H |  | 21.4356 | -4.1535 | -9.7725 |
| H |  | 24.0547 | -5.9382 | -3.1269 |
| H |  | 24.0439 | -3.9429 | -1.6509 |
| H |  | 24.9326 | -1.3956 | -5.0361 |
| H |  | 14.7543 | -14.4226 | 3.1068 |
| H |  | 16.1984 | -13.1676 | 1.5252 |
| H |  | 14.1187 | -14.9426 | -1.8302 |
| H |  | 8.2551 | -14.9856 | 2.6961 |
| H |  | 8.6517 | -13.4165 | 4.5797 |
| H |  | 12.8826 | -14.3276 | 4.4883 |
| H |  | 11.9371 | -15.0825 | -2.6689 |
| H |  | 9.8458 | -13.9154 | -3.3228 |
| H |  | 7.9853 | -15.0979 | 0.4022 |
| H |  | 7.2416 | -6.2225 | 13.6139 |
| H |  | 5.5821 | -4.6399 | 12.6622 |
| H |  | 7.4786 | -1.3544 | 14.7467 |
| H |  | 13.6748 | -5.2667 | 13.8066 |
| H |  | 13.3392 | -7.0329 | 12.0959 |
| H |  | 12.7483 | 7.7161 | -11.1090 |
| H |  | 9.2162 | -7.4918 | 13.3337 |
| H |  | 9.5725 | -0.3131 | 14.9374 |
| H |  | 11.5110 | 0.7080 | 13.7671 |
| H |  | 13.7557 | -2.9654 | 14.2172 |
| H |  | 11.9976 | -7.5365 | 9.9980 |
| H |  | 11.9525 | -10.5030 | 6.3243 |
| H |  | 10.0187 | -10.6514 | 10.9356 |
| H |  | 9.7155 | -13.4635 | 7.1364 |
| H |  | 23.0290 | 3.1428 | 7.5150 |
| H |  | 21.1941 | 2.1547 | 8.8636 |
| H |  | 19.9612 | 6.1038 | 10.1364 |
| H |  | 22.3577 | 8.2432 | 3.5205 |
| H |  | 22.5797 | 6.2730 | 2.0243 |
| H |  | 23.7350 | 3.8095 | 5.3904 |
| H |  | 19.2946 | 8.1705 | 9.2478 |
| H |  | 17.7820 | 9.4078 | 7.7148 |
| H |  | 20.9171 | 9.6112 | 4.7379 |
| H |  | 18.5957 | 2.1901 | 8.9876 |
| H |  | 15.0951 | -0.3011 | 10.9445 |
| H |  | 18.0118 | 3.4378 | 12.5381 |
| H |  | 22.7455 | -1.7429 | -1.3294 |
| H |  | 22.1357 | 1.5951 | 1.9524 |
| H |  | 25.4581 | 0.9005 | -1.7224 |
| H |  | 24.6139 | 4.4067 | 1.3527 |
| H |  | 14.2557 | 1.1752 | 14.3366 |
| H |  | 15.9805 | -11.3627 | -0.3585 |
| H |  | -3.1246 | 5.6244 | 2.6305 |
| H |  | -1.3960 | 7.3776 | 2.2699 |
| H |  | -1.1850 | 8.0711 | 6.5425 |
| H |  | -3.2491 | 0.7251 | 6.9765 |
| H |  | -2.8745 | -0.3669 | 4.7727 |
| H |  | -3.5152 | 3.4076 | 2.7637 |
| H |  | -0.3786 | 6.5334 | 8.1103 |
| H |  | 1.1645 | 4.8595 | 9.0796 |
| H |  | -1.7804 | 1.7538 | 8.4779 |
| H |  | 1.1852 | 3.0302 | 11.1932 |
| H |  | -1.6758 | -1.9770 | -3.8426 |
| H |  | 2.3358 | -9.4803 | -3.2587 |
| H |  | -1.6985 | 0.7134 | 0.0495 |
| H |  | -4.1725 | -3.4083 | -1.3562 |
| H |  | -4.1832 | -0.7884 | 2.5423 |
| H |  | 6.9784 | 6.0820 | -13.2209 |
| H |  | 5.5822 | 4.1456 | -12.5446 |
| H |  | 8.1954 | 1.4650 | -14.7183 |
| H |  | 13.5002 | 6.2547 | -12.9721 |
| H |  | 8.6860 | 7.5728 | -12.5962 |
| H |  | 10.4471 | 0.8375 | -14.8542 |
| H |  | 12.4826 | 0.0607 | -13.6615 |
| H |  | 14.0456 | 4.0967 | -13.6392 |
| H |  | 5.5779 | 2.0209 | -11.2327 |
| H |  | 15.2407 | 0.0770 | -14.0547 |
| H |  | 2.5851 | -1.2071 | -9.4729 |
| H |  | 5.3595 | -0.9054 | -13.6254 |
| H |  | 19.2222 | -1.7139 | -12.2162 |
| H |  | 15.7963 | 1.5533 | -10.5991 |
| H |  | 13.8009 | 14.7212 | -1.0457 |
| H |  | 12.2076 | 15.7918 | -2.6203 |
| H |  | 11.3043 | 15.8596 | 2.3017 |
| H |  | 5.7213 | 15.0514 | -2.4699 |
| H |  | 6.4830 | 13.7054 | -4.4120 |
| H |  | 10.4328 | 15.4519 | -4.1128 |
| H |  | 9.1084 | 15.5849 | 3.0736 |
| H |  | 7.2729 | 13.9853 | 3.5689 |
| H |  | 5.3718 | 14.9574 | -0.1962 |
| H |  | 13.8116 | 12.7694 | 0.7202 |
| H |  | 4.3333 | 14.0547 | 3.6922 |
| H |  | 16.9143 | 10.1334 | 3.1107 |
| H |  | 14.9041 | 14.6554 | 3.8437 |
| H |  | 9.2757 | 8.6288 | -8.2289 |
| H |  | 10.1025 | 13.5427 | -7.7831 |
| H |  | 0.7963 | 11.1432 | 4.9151 |
| H |  | 4.3002 | 11.0652 | 1.3457 |
| H |  | 11.3596 | 10.2469 | -10.9475 |
| H |  | 2.8164 | -4.3380 | -11.6294 |
| H |  | 0.9911 | 8.0105 | 2.7628 |
| H |  | 5.9133 | -11.8655 | -1.2479 |
| H |  | 3.4570 | -12.2494 | -5.5871 |
| H |  | 2.6770 | 0.5712 | 8.7758 |
| H |  | 3.6039 | -0.2722 | 13.6194 |
| H |  | 17.7717 | 11.8662 | 6.3798 |
| H |  | 5.3886 | -2.5291 | 11.1264 |
| H |  | 19.6019 | -0.4430 | -8.6454 |
| H |  | 7.2689 | -14.3731 | -3.7724 |
| H |  | 18.7353 | -8.3913 | -2.7857 |
| H |  | 17.4950 | -13.2074 | -3.3258 |
| H |  | 19.9784 | -10.1225 | -5.9264 |
| H |  | -2.2760 | -8.2864 | -3.9564 |
| H |  | -0.2675 | -9.5826 | -3.2600 |
| H |  | 1.0328 | -9.8066 | -7.3904 |
| H |  | -2.7715 | -3.2607 | -8.3073 |
| H |  | -3.0155 | -2.1834 | -6.0783 |
| H |  | -2.9789 | -6.0643 | -4.1693 |
| H |  | 1.6771 | -8.0494 | -8.7565 |
| H |  | 2.8658 | -6.0110 | -9.5025 |
| H |  | -0.8591 | -3.8241 | -9.5570 |
| H |  | 8.3088 | 11.8346 | -4.8926 |
| H |  | 3.3441 | 2.1944 | -8.3073 |
| H |  | 2.1540 | 0.9200 | -8.7964 |
| H |  | 3.7002 | 0.4443 | -7.9788 |
| H |  | 4.5804 | -4.2790 | -14.8123 |
| H |  | 4.4272 | -2.5003 | -15.0909 |
| H |  | 5.8553 | -3.1598 | -14.1892 |
| H |  | 16.6471 | -0.5730 | 7.6428 |
| H |  | 16.2923 | -1.3575 | 9.2322 |
| H |  | 15.1647 | -0.1048 | 8.5639 |
| H |  | 16.4173 | 3.7468 | 15.8204 |
| H |  | 17.9100 | 3.3258 | 14.8934 |
| H |  | 16.7286 | 4.5244 | 14.2188 |
| H |  | 19.3977 | -13.7121 | -6.4133 |
| H |  | 19.2131 | -14.1250 | -4.6640 |
| H |  | 17.7641 | -13.6457 | -5.6427 |
| H |  | 16.8040 | -7.8734 | 0.2779 |
| H |  | 18.4304 | -7.9132 | -0.5099 |
| H |  | 16.9605 | -7.4798 | -1.4788 |
| H |  | 4.9744 | -2.0047 | 7.5338 |
| H |  | 3.3800 | -1.1486 | 7.6010 |
| H |  | 4.9180 | -0.1998 | 7.7398 |
| H |  | 1.2868 | 2.2830 | 14.8610 |
| H |  | 1.6355 | 0.5160 | 14.7249 |
| H |  | 2.9979 | 1.7132 | 14.7321 |
| H |  | 17.7310 | -2.1713 | -15.5288 |
| H |  | 19.1365 | -1.5574 | -14.5736 |
| H |  | 18.1357 | -2.9294 | -13.9387 |
| H |  | 3.7835 | -9.2353 | 0.1387 |
| H |  | 4.3519 | -10.9459 | 0.2541 |
| H |  | 5.4306 | -9.6774 | -0.4641 |
| H |  | 5.5774 | -14.9001 | -6.9579 |
| H |  | 3.9358 | -14.4158 | -6.3792 |
| H |  | 5.0507 | -13.1766 | -7.0922 |
| H |  | 27.2925 | 4.0133 | -1.1246 |
| H |  | 27.3274 | 2.2070 | -1.0922 |
| H |  | 26.2409 | 3.0746 | -2.2560 |
| H |  | 20.2933 | -1.5072 | 1.3513 |
| H |  | 21.3372 | -0.5542 | 2.4778 |
| H |  | 20.2530 | 0.2988 | 1.3016 |
| H |  | 8.3914 | -13.9061 | 10.5254 |
| H |  | 9.6908 | -12.9652 | 11.3565 |
| H |  | 8.1821 | -12.1311 | 10.7949 |
| H |  | 7.6663 | 8.2471 | -4.9516 |
| H |  | 7.5481 | 8.1007 | -6.7575 |
| H |  | 9.1512 | 7.9009 | -5.9348 |
| H |  | 11.8508 | 13.8914 | -10.9828 |
| H |  | 10.3960 | 14.3149 | -9.9982 |
| H |  | 11.9764 | 13.9622 | -9.1810 |
| H |  | -6.0070 | -3.8069 | 1.7811 |
| H |  | -6.0708 | -3.5276 | -0.0036 |
| H |  | -4.7004 | -4.4682 | 0.7214 |
| H |  | 0.1528 | 1.0783 | -3.1144 |
| H |  | -0.9926 | 1.7619 | -1.9050 |
| H |  | 0.3246 | 0.6215 | -1.3803 |
| H |  | 13.6045 | -7.2653 | 6.7408 |
| H |  | 13.8023 | -9.0442 | 6.4900 |
| H |  | 12.3123 | -8.2017 | 5.8927 |
| H |  | 15.2023 | 9.5003 | -0.0611 |
| H |  | 16.7723 | 9.7486 | 0.7992 |
| H |  | 15.3573 | 9.0319 | 1.6774 |
| H |  | 16.5898 | 15.2727 | 7.0348 |
| H |  | 16.3999 | 15.7541 | 5.3036 |
| H |  | 15.0162 | 14.9902 | 6.1918 |
| H |  | 1.7251 | 14.4278 | 6.2419 |
| H |  | 0.4823 | 13.4608 | 5.3556 |
| H |  | 1.5842 | 12.6520 | 6.5469 |
| H |  | 3.3710 | 7.7877 | 0.0144 |
| H |  | 3.4009 | 9.5653 | -0.3264 |
| H |  | 4.6038 | 8.8338 | 0.8175 |
| H |  | 25.1917 | -7.4769 | -4.6315 |
| H |  | 25.5714 | -7.7704 | -6.3288 |
| H |  | 26.5205 | -6.5993 | -5.3906 |
| H |  | 23.0099 | -5.9536 | -10.2406 |
| H |  | 24.6458 | -5.2964 | -10.2093 |
| H |  | 24.3178 | -6.9071 | -9.5371 |
| H |  | 26.3390 | -1.8421 | -6.9791 |
| H |  | 27.1728 | -3.3776 | -6.7433 |
| H |  | 26.5512 | -2.9358 | -8.3474 |
| H |  | 26.1001 | -6.7034 | -8.2085 |
| H |  | 26.4230 | -5.1014 | -8.8799 |
| H |  | 27.0452 | -5.5322 | -7.2836 |
| H |  | 13.1422 | -17.1476 | -2.0996 |
| H |  | 13.6617 | -18.1326 | -0.7324 |
| H |  | 12.0162 | -18.2996 | -1.3793 |
| H |  | 13.9501 | -16.5134 | 4.0615 |
| H |  | 12.7191 | -17.7645 | 3.8884 |
| H |  | 14.1411 | -17.7574 | 2.8252 |
| H |  | 8.2541 | -17.1891 | 1.6719 |
| H |  | 9.2523 | -18.3272 | 0.7673 |
| H |  | 9.4537 | -18.1635 | 2.5236 |
| H |  | 12.9736 | -18.8614 | 1.2679 |
| H |  | 11.5738 | -18.8761 | 2.3462 |
| H |  | 11.3484 | -19.0574 | 0.6038 |
| H |  | 8.5483 | -1.2914 | 16.9247 |
| H |  | 8.2434 | -2.8239 | 17.7417 |
| H |  | 9.8226 | -2.0267 | 17.8993 |
| H |  | 8.2333 | -7.3357 | 15.5517 |
| H |  | 9.5419 | -7.2157 | 16.7276 |
| H |  | 8.0520 | -6.2749 | 16.9497 |
| H |  | 13.7689 | -4.5251 | 16.1172 |
| H |  | 12.7936 | -3.8739 | 17.4346 |
| H |  | 12.7331 | -5.6064 | 17.0505 |
| H |  | 9.1934 | -4.7969 | 18.1652 |
| H |  | 10.6781 | -5.7257 | 17.9318 |
| H |  | 10.7643 | -4.0057 | 18.3257 |
| H |  | 21.3123 | 8.0710 | 10.6241 |
| H |  | 23.0129 | 7.6062 | 10.5956 |
| H |  | 22.5049 | 9.1748 | 9.9358 |
| H |  | 25.1047 | 4.4015 | 7.3353 |
| H |  | 25.7570 | 6.0262 | 7.1209 |
| H |  | 25.1857 | 5.4960 | 8.7164 |
| H |  | 23.3294 | 9.8886 | 5.0402 |
| H |  | 23.6648 | 10.2084 | 6.7420 |
| H |  | 24.7461 | 9.1616 | 5.8001 |
| H |  | 24.8057 | 7.6276 | 9.2732 |
| H |  | 25.3812 | 8.1441 | 7.6849 |
| H |  | 24.3037 | 9.1889 | 8.6166 |
| C |  | -2.5086 | 7.9991 | 8.7909 |
| H |  | -4.2830 | 7.7352 | 7.5813 |
| H |  | -4.1570 | 6.6291 | 8.9674 |
| C |  | -5.6706 | 4.9419 | 3.0325 |
| H |  | -6.3236 | 4.5679 | 5.0456 |
| H |  | -5.7777 | 6.2374 | 4.7624 |
| C |  | -4.3263 | 1.7034 | 9.1492 |
| H |  | -5.0234 | 3.7372 | 9.1147 |
| H |  | -5.7167 | 2.7148 | 7.8363 |
| H |  | -5.8699 | 6.3769 | 6.9204 |
| H |  | -6.3684 | 4.6789 | 6.9894 |
| H |  | -5.6135 | 5.4424 | 8.3964 |
| H |  | 9.3719 | 1.8157 | -16.8211 |
| H |  | 8.8351 | 3.3449 | -17.5152 |
| H |  | 10.5401 | 2.8617 | -17.6309 |
| H |  | 7.9026 | 7.5561 | -14.9199 |
| H |  | 9.2760 | 7.7791 | -16.0040 |
| H |  | 7.9828 | 6.6371 | -16.4237 |
| H |  | 13.8543 | 5.8425 | -15.3486 |
| H |  | 13.0943 | 5.1682 | -16.7903 |
| H |  | 12.6823 | 6.8076 | -16.2475 |
| H |  | 9.4251 | 5.4877 | -17.6870 |
| H |  | 10.7010 | 6.6358 | -17.2688 |
| H |  | 11.1206 | 5.0071 | -17.8088 |
| H |  | 9.9387 | 17.8471 | 2.6368 |
| H |  | 10.2939 | 18.9879 | 1.3400 |
| H |  | 8.6308 | 18.8010 | 1.9343 |
| H |  | 11.0483 | 17.7464 | -3.4953 |
| H |  | 9.5891 | 18.7200 | -3.3161 |
| H |  | 10.9447 | 18.9266 | -2.1874 |
| H |  | 5.2497 | 17.1432 | -1.3335 |
| H |  | 5.9707 | 18.4023 | -0.3308 |
| H |  | 6.2585 | 18.3820 | -2.0828 |
| H |  | 9.5243 | 19.6877 | -0.6375 |
| H |  | 8.1857 | 19.4884 | -1.7734 |
| H |  | 7.8721 | 19.5190 | -0.0354 |
| C |  | 0.1825 | -9.9909 | -9.8566 |
| H |  | -1.7866 | -10.2637 | -9.0021 |
| H |  | -1.7146 | -9.1039 | -10.3483 |
| C |  | -4.7355 | -7.9858 | -4.8390 |
| H |  | -5.0430 | -7.7760 | -6.9559 |
| H |  | -4.1945 | -9.2695 | -6.4940 |
| C |  | -3.2065 | -4.4038 | -10.6084 |
| H |  | -3.2982 | -6.5511 | -10.6892 |
| H |  | -4.4555 | -5.8208 | -9.5541 |
| H |  | -3.7761 | -9.4051 | -8.6407 |
| H |  | -4.6933 | -7.9075 | -8.8421 |
| H |  | -3.5174 | -8.3942 | -10.0684 |
| H |  | 17.2752 | 1.9959 | -7.2809 |
| H |  | 16.8428 | 2.7492 | -8.8659 |
| H |  | 15.8739 | 1.3566 | -8.2262 |
| H |  | -1.9097 | 7.4158 | 9.5181 |
| C |  | -3.1489 | 9.1904 | 9.5013 |
| H |  | -1.7959 | 8.3679 | 8.0203 |
| H |  | -3.8553 | 8.8692 | 10.2754 |
| H |  | -3.6985 | 9.8316 | 8.8024 |
| H |  | -2.3904 | 9.8128 | 9.9890 |
| H |  | -5.6977 | 3.8537 | 2.8201 |
| H |  | -4.7820 | 5.3368 | 2.4964 |
| C |  | -6.9334 | 5.5967 | 2.4753 |
| H |  | -7.8381 | 5.2054 | 2.9539 |
| H |  | -7.0304 | 5.4108 | 1.3990 |
| H |  | -6.9292 | 6.6827 | 2.6210 |
| H |  | -3.5136 | 1.9707 | 9.8543 |
| H |  | -3.9025 | 0.9438 | 8.4551 |
| C |  | -5.4802 | 1.0784 | 9.9291 |
| H |  | -5.9194 | 1.7790 | 10.6481 |
| H |  | -5.1305 | 0.1967 | 10.4963 |
| H |  | -6.2855 | 0.7391 | 9.2682 |
| H |  | 3.8293 | -4.5219 | 8.5306 |
| H |  | 5.0946 | -3.6861 | 7.6006 |
| C |  | 5.7852 | -4.5443 | 9.4589 |
| H |  | 5.5787 | -5.3760 | 10.1407 |
| H |  | 5.6205 | -3.6037 | 10.0181 |
| H |  | 6.8492 | -4.5863 | 9.2010 |
| H |  | 2.5475 | -11.0857 | 4.3564 |
| H |  | 1.7544 | -10.6124 | 5.8725 |
| C |  | 3.1762 | -12.2365 | 6.0785 |
| H |  | 4.1306 | -12.5839 | 5.6671 |
| H |  | 2.4386 | -13.0288 | 5.9066 |
| H |  | 3.3023 | -12.1363 | 7.1625 |
| H |  | 5.6428 | -7.3346 | 1.0938 |
| C |  | 7.7315 | -6.8039 | 1.2872 |
| H |  | 5.9214 | -5.6352 | 1.5321 |
| H |  | 8.1160 | -7.8173 | 1.4500 |
| H |  | 8.3808 | -6.1145 | 1.8391 |
| H |  | 7.8475 | -6.5779 | 0.2211 |
| H |  | -0.0509 | 11.0976 | -7.3140 |
| C |  | 1.3547 | 12.6622 | -7.8420 |
| H |  | 0.8986 | 11.7182 | -5.9474 |
| H |  | 1.3706 | 12.4415 | -8.9155 |
| H |  | 2.3486 | 13.0349 | -7.5689 |
| H |  | 0.6447 | 13.4822 | -7.6864 |
| H |  | 1.8629 | 4.5737 | -9.3088 |
| H |  | 3.1534 | 3.8791 | -8.2960 |
| C |  | 3.7844 | 4.4043 | -10.2964 |
| H |  | 3.5049 | 5.0676 | -11.1223 |
| H |  | 3.6495 | 3.3681 | -10.6428 |
| H |  | 4.8530 | 4.5464 | -10.1013 |
| H |  | 5.0986 | 6.2428 | -3.2602 |
| H |  | 4.1343 | 7.5408 | -2.5285 |
| C |  | 6.2219 | 8.0434 | -2.8143 |
| H |  | 7.0189 | 7.9703 | -3.5739 |
| H |  | 6.6133 | 7.6078 | -1.8889 |
| H |  | 6.0550 | 9.1122 | -2.6309 |
| H |  | -2.2439 | -4.3987 | -11.1577 |
| H |  | -3.1378 | -3.5906 | -9.8525 |
| C |  | -4.3467 | -4.0993 | -11.5785 |
| H |  | -4.4388 | -4.8700 | -12.3515 |
| H |  | -4.1851 | -3.1420 | -12.0870 |
| H |  | -5.3118 | -4.0350 | -11.0622 |
| H |  | 0.8158 | -10.1851 | -8.9628 |
| H |  | 0.7221 | -9.2379 | -10.4646 |
| C |  | 0.0318 | -11.2805 | -10.6624 |
| H |  | -0.4425 | -12.0727 | -10.0719 |
| H |  | 1.0073 | -11.6558 | -10.9906 |
| H |  | -0.5793 | -11.1282 | -11.5591 |
| H |  | -3.9054 | -8.1716 | -4.1234 |
| C |  | -5.9035 | -8.9144 | -4.5119 |
| H |  | -5.0465 | -6.9327 | -4.6843 |
|  |  |  |  |  |
| H |  | -5.6129 | -9.9701 | -4.5693 |
| H |  | -6.7451 | -8.7697 | -5.1992 |
| H |  | -6.2768 | -8.7355 | -3.4973 |

i) [(**OMe-cube)@**(**OMe-cube)]_@_** (**OMe-cube)** (Type 1 conformer)

| **atom** | **x** | **y** | **z** |
| --- | --- | --- | --- |
| C | 4.4507 | -9.9757 | 10.0417 |
| C | 5.2854 | -11.9466 | 8.6235 |
| C | 6.8557 | -10.8432 | 10.3308 |
| C | 5.3399 | -11.3125 | 10.0939 |
| C | 3.9566 | -9.9089 | 8.6099 |
| C | 4.3447 | -11.0371 | 7.8576 |
| C | 3.9433 | -11.1692 | 6.5304 |
| C | 3.1930 | -10.1588 | 5.9254 |
| N | 2.0337 | -8.0455 | 5.9982 |
| C | 2.8509 | -9.0103 | 6.6610 |
| C | 3.2324 | -8.8835 | 8.0141 |
| C | 6.6841 | -11.7262 | 8.0706 |
| C | 7.5427 | -11.1094 | 9.0044 |
| C | 8.8322 | -10.7375 | 8.6285 |
| C | 9.2830 | -10.9957 | 7.3296 |
| N | 8.9044 | -11.7276 | 5.0602 |
| C | 8.4237 | -11.6074 | 6.4001 |
| C | 7.1109 | -11.9668 | 6.7699 |
| C | 6.7480 | -9.3427 | 10.5426 |
| C | 5.4365 | -8.8630 | 10.3345 |
| C | 5.1764 | -7.4971 | 10.3890 |
| C | 6.2078 | -6.5980 | 10.6785 |
| N | 8.5473 | -6.1204 | 11.0963 |
| C | 7.5067 | -7.0824 | 10.9149 |
| C | 7.7792 | -8.4651 | 10.8517 |
| C | -5.3337 | -1.2112 | 0.3454 |
| C | -5.4360 | 1.2629 | 1.0401 |
| C | -5.3479 | 0.6304 | -1.4501 |
| C | -5.9826 | 0.2073 | -0.0374 |
| C | -4.4631 | -0.9054 | 1.5511 |
| C | -4.5937 | 0.4305 | 1.9880 |
| C | -3.9068 | 0.8693 | 3.1171 |
| C | -3.0254 | 0.0070 | 3.7780 |
| N | -1.9111 | -2.1322 | 3.9971 |
| C | -2.8306 | -1.2940 | 3.2867 |
| C | -3.5690 | -1.7629 | 2.1779 |
| C | -4.4935 | 2.1530 | 0.2451 |
| C | -4.4651 | 1.8202 | -1.1257 |
| C | -3.6082 | 2.4957 | -1.9925 |
| C | -2.7582 | 3.4908 | -1.4992 |
| N | -1.8411 | 4.8020 | 0.3088 |
| C | -2.7543 | 3.7865 | -0.1260 |
| C | -3.6377 | 3.1242 | 0.7522 |
| C | -4.4577 | -0.5431 | -1.8232 |
| C | -4.4628 | -1.5601 | -0.8448 |
| C | -3.6740 | -2.6954 | -1.0119 |
| C | -2.8234 | -2.7996 | -2.1174 |
| N | -1.8413 | -1.9045 | -4.1394 |
| C | -2.7765 | -1.7590 | -3.0599 |
| C | -3.6327 | -0.6435 | -2.9361 |
| C | 7.3919 | 14.2807 | 4.8428 |
| C | 4.9819 | 13.4540 | 4.5157 |
| C | 6.2593 | 12.8781 | 6.6691 |
| C | 5.9991 | 14.0503 | 5.6014 |
| C | 7.1761 | 13.6462 | 3.4802 |
| C | 5.8401 | 13.2390 | 3.2780 |
| C | 5.4804 | 12.5958 | 2.0955 |
| C | 6.4445 | 12.3469 | 1.1109 |
| N | 8.7458 | 12.4407 | 0.3113 |
| C | 7.7811 | 12.7118 | 1.3366 |
| C | 8.1496 | 13.3757 | 2.5278 |
| C | 4.6472 | 12.0686 | 5.0429 |
| C | 5.4743 | 11.7017 | 6.1285 |
| C | 5.4971 | 10.3821 | 6.5708 |
| C | 4.6277 | 9.4424 | 6.0025 |
| N | 2.7606 | 8.8836 | 4.5545 |
| C | 3.7303 | 9.8383 | 4.9966 |
| C | 3.7491 | 11.1599 | 4.5009 |
| C | 7.7571 | 12.6522 | 6.6112 |
| C | 8.3856 | 13.4552 | 5.6344 |
| C | 9.7661 | 13.4016 | 5.4648 |
| C | 10.5306 | 12.5276 | 6.2445 |
| N | 10.7286 | 10.8709 | 8.0069 |
| C | 9.8982 | 11.7064 | 7.1941 |
| C | 8.5029 | 11.7775 | 7.3901 |
| C | 2.5566 | 8.6775 | 3.2888 |
| C | 1.5427 | 7.7049 | 2.8383 |
| C | 0.6264 | 7.1142 | 3.7198 |
| C | -0.2895 | 6.1762 | 3.2398 |
| C | -0.3041 | 5.7867 | 1.8747 |
| C | 0.5890 | 6.3897 | 0.9887 |
| C | 1.4929 | 7.3513 | 1.4655 |
| C | -1.2422 | 4.7219 | 1.4548 |
| C | 17.9440 | 3.4729 | 13.6713 |
| C | 16.7475 | 1.6547 | 15.0355 |
| C | 19.0097 | 1.1449 | 13.9312 |
| C | 18.1890 | 2.2765 | 14.7175 |
| C | 16.4404 | 3.4918 | 13.4714 |
| C | 15.7790 | 2.5675 | 14.3096 |
| C | 14.3882 | 2.5130 | 14.3406 |
| C | 13.6386 | 3.3513 | 13.5094 |
| N | 13.4957 | 5.0094 | 11.7398 |
| C | 14.3004 | 4.2305 | 12.6362 |
| C | 15.7099 | 4.2980 | 12.6061 |
| C | 16.7555 | 0.3148 | 14.3170 |
| C | 18.0007 | 0.0325 | 13.7172 |
| C | 18.1628 | -1.1213 | 12.9530 |
| C | 17.0932 | -2.0083 | 12.7907 |
| N | 14.7838 | -2.6547 | 13.2373 |
| C | 15.8498 | -1.7095 | 13.3707 |
| C | 15.6724 | -0.5370 | 14.1342 |
| C | 19.3241 | 1.7853 | 12.5903 |
| C | 18.6922 | 3.0374 | 12.4296 |
| C | 18.8142 | 3.7315 | 11.2285 |
| C | 19.5729 | 3.1934 | 10.1843 |
| N | 21.0286 | 1.4981 | 9.2685 |
| C | 20.2117 | 1.9520 | 10.3513 |
| C | 20.0920 | 1.2449 | 11.5667 |
| C | 13.6448 | 6.2963 | 11.6771 |
| C | 12.8124 | 7.1064 | 10.7639 |
| C | 11.5571 | 6.6811 | 10.3163 |
| C | 10.7958 | 7.5128 | 9.4853 |
| C | 11.2735 | 8.7856 | 9.0750 |
| C | 12.5314 | 9.2060 | 9.5190 |
| C | 13.2872 | 8.3766 | 10.3531 |
| C | 9.3930 | -6.1966 | 12.0745 |
| C | 10.4788 | -5.1982 | 12.1798 |
| C | 10.4973 | -4.0269 | 11.4158 |
| C | 11.5542 | -3.1189 | 11.5546 |
| C | 12.6227 | -3.3596 | 12.4554 |
| C | 12.6038 | -4.5321 | 13.2201 |
| C | 11.5459 | -5.4353 | 13.0837 |
| C | -0.3505 | -3.9544 | 4.1415 |
| C | 13.7150 | -2.3703 | 12.5624 |
| C | 10.4277 | 9.6238 | 8.2025 |
| C | -1.2222 | -3.0367 | 3.3765 |
| C | 19.8729 | 11.0983 | -6.4013 |
| C | 18.9459 | 10.6133 | -8.7456 |
| C | 17.7634 | 12.3115 | -7.2230 |
| C | 19.2059 | 11.7915 | -7.6852 |
| C | 20.1733 | 9.6839 | -6.8627 |
| C | 19.6441 | 9.4134 | -8.1436 |
| C | 19.8021 | 8.1533 | -8.7145 |
| C | 20.4890 | 7.1544 | -8.0185 |
| N | 21.6661 | 6.3394 | -6.0834 |
| C | 21.0334 | 7.4315 | -6.7501 |
| C | 20.8714 | 8.7052 | -6.1666 |
| C | 17.4375 | 10.4555 | -8.7605 |
| C | 16.7901 | 11.4874 | -8.0462 |
| C | 15.4011 | 11.5812 | -8.0504 |
| C | 14.6416 | 10.6013 | -8.6959 |
| N | 14.4452 | 8.5352 | -9.9214 |
| C | 15.2879 | 9.5135 | -9.3085 |
| C | 16.6968 | 9.4438 | -9.3593 |
| C | 17.6303 | 11.8136 | -5.7902 |
| C | 18.7670 | 11.0942 | -5.3650 |
| C | 18.7610 | 10.4346 | -4.1376 |
| C | 17.6402 | 10.5212 | -3.3053 |
| N | 15.4232 | 11.3547 | -2.8066 |
| C | 16.5133 | 11.2481 | -3.7256 |
| C | 16.5030 | 11.8925 | -4.9811 |
| C | 14.1905 | 11.2372 | -3.1910 |
| C | 13.1029 | 11.3943 | -2.2017 |
| C | 11.1004 | 3.4285 | -12.2756 |
| C | 12.0042 | 4.3839 | -11.6028 |
| C | 2.7371 | -0.9755 | -8.8130 |
| C | 1.6655 | -0.9731 | -7.7964 |
| C | 13.1055 | 3.9642 | -10.8468 |
| C | 13.9396 | 4.9096 | -10.2428 |
| C | 13.7156 | 6.3012 | -10.3911 |
| C | 12.6068 | 6.7202 | -11.1347 |
| C | 11.7599 | 5.7755 | -11.7247 |
| C | 14.6391 | 7.2647 | -9.7543 |
| C | 13.9445 | -15.0998 | -6.4166 |
| C | 16.4542 | -14.5868 | -6.1721 |
| C | 15.1440 | -15.5767 | -4.1949 |
| C | 15.3166 | -15.6498 | -5.7869 |
| C | 14.3361 | -13.7904 | -7.0769 |
| C | 15.7280 | -13.5596 | -7.0205 |
| C | 16.2786 | -12.4413 | -7.6405 |
| C | 15.4475 | -11.5214 | -8.2867 |
| N | 13.2673 | -10.7740 | -9.0102 |
| C | 14.0552 | -11.7172 | -8.2808 |
| C | 13.4919 | -12.8581 | -7.6697 |
| C | 16.8202 | -13.9347 | -4.8471 |
| C | 16.0761 | -14.4597 | -3.7689 |
| C | 16.2009 | -13.9098 | -2.4949 |
| C | 17.1065 | -12.8688 | -2.2718 |
| N | 18.8137 | -11.3460 | -3.0054 |
| C | 17.8598 | -12.3550 | -3.3421 |
| C | 17.7017 | -12.8785 | -4.6437 |
| C | 13.7038 | -15.1410 | -3.9975 |
| C | 13.0502 | -14.8635 | -5.2179 |
| C | 11.7353 | -14.4079 | -5.2182 |
| C | 11.0683 | -14.1941 | -4.0059 |
| N | 11.0702 | -14.3289 | -1.5309 |
| C | 11.7270 | -14.4657 | -2.7970 |
| C | 13.0473 | -14.9628 | -2.7881 |
| C | 12.2048 | -10.2164 | -8.5202 |
| C | 10.8515 | -13.1622 | -1.0173 |
| C | 11.4555 | -9.2420 | -9.3454 |
| C | 10.2165 | -13.0584 | 0.3165 |
| C | 11.9488 | -8.7808 | -10.5739 |
| C | 11.2308 | -7.8304 | -11.3051 |
| C | 9.9992 | -7.3056 | -10.8342 |
| C | 9.5043 | -7.7714 | -9.6132 |
| C | 10.2158 | -8.7344 | -8.8872 |
| C | 9.3508 | -14.0384 | 0.8150 |
| C | 8.8103 | -13.8985 | 2.0973 |
| C | 9.1424 | -12.7996 | 2.9300 |
| C | 9.9814 | -11.8043 | 2.4158 |
| C | 10.5029 | -11.9307 | 1.1244 |
| C | 28.4804 | -2.2732 | 0.9420 |
| C | 27.8120 | -4.5313 | -0.0920 |
| C | 28.2457 | -2.4965 | -1.6071 |
| C | 28.7671 | -3.2568 | -0.2916 |
| C | 27.3525 | -2.9515 | 1.7024 |
| C | 27.0357 | -4.2205 | 1.1731 |
| C | 26.0469 | -5.0004 | 1.7680 |
| C | 25.3293 | -4.5046 | 2.8604 |
| N | 24.8188 | -2.7602 | 4.4528 |
| C | 25.5936 | -3.2087 | 3.3362 |
| C | 26.6198 | -2.4283 | 2.7615 |
| C | 26.8635 | -4.4736 | -1.2780 |
| C | 27.1125 | -3.3633 | -2.1134 |
| C | 26.3342 | -3.1495 | -3.2482 |
| C | 25.2895 | -4.0266 | -3.5555 |
| N | 23.9771 | -6.0070 | -3.1016 |
| C | 25.0194 | -5.1112 | -2.7041 |
| C | 25.8097 | -5.3356 | -1.5552 |
| C | 27.7283 | -1.1664 | -1.0885 |
| C | 27.9530 | -1.0090 | 0.2964 |
| C | 27.6317 | 0.1878 | 0.9310 |
| C | 27.0358 | 1.2230 | 0.2047 |
| N | 26.1587 | 2.1313 | -1.8646 |
| C | 26.7346 | 1.0311 | -1.1547 |
| C | 27.0794 | -0.1706 | -1.8092 |
| C | 22.1493 | -7.5088 | -2.6696 |
| C | 24.3634 | -1.5470 | 4.5106 |
| C | 25.0469 | 2.0063 | -2.5198 |
| C | 23.5579 | -1.1142 | 5.6713 |
| C | 24.4942 | 3.1612 | -3.2583 |
| C | 23.0307 | -2.0232 | 6.5953 |
| C | 22.2413 | -1.5632 | 7.6546 |
| C | 21.9586 | -0.1844 | 7.8263 |
| C | 22.5020 | 0.7266 | 6.9135 |
| C | 23.2876 | 0.2663 | 5.8518 |
| C | 21.7528 | -7.6307 | -4.0059 |
| C | 20.7768 | -8.5684 | -4.3617 |
| C | 20.1749 | -9.4161 | -3.3977 |
| C | 20.5729 | -9.2939 | -2.0615 |
| C | 21.5421 | -8.3516 | -1.7045 |
| C | 25.1450 | 4.4035 | -3.3009 |
| C | 24.5767 | 5.4668 | -4.0099 |
| C | 23.3396 | 5.3257 | -4.6942 |
| C | 22.6904 | 4.0827 | -4.6533 |
| C | 23.2620 | 3.0175 | -3.9494 |
| C | 19.1567 | -10.4003 | -3.8237 |
| C | 9.2757 | -6.3065 | -11.6478 |
| C | 22.7703 | 6.4857 | -5.4120 |
| C | 0.6683 | 0.0126 | -7.7746 |
| C | -0.2949 | 0.0005 | -6.7646 |
| C | -0.2828 | -0.9839 | -5.7415 |
| C | 0.6875 | -1.9845 | -5.7771 |
| C | 1.6439 | -1.9855 | -6.8050 |
| C | 11.7860 | 11.6731 | -2.6510 |
| C | 10.7282 | 11.8500 | -1.7522 |
| C | 10.9569 | 11.7380 | -0.3758 |
| C | 12.2700 | 11.4396 | 0.0699 |
| C | 13.3309 | 11.2781 | -0.8274 |
| C | 21.0943 | 0.2434 | 8.9459 |
| C | 9.9024 | 11.9460 | 0.6347 |
| C | 8.6206 | -12.7473 | 4.3116 |
| C | -1.2630 | -0.8585 | -4.6415 |
| C | 0.5083 | -3.5198 | 5.1519 |
| C | 1.3138 | -4.4512 | 5.8227 |
| C | 1.2894 | -5.8274 | 5.4842 |
| C | 0.4170 | -6.2594 | 4.4761 |
| C | -0.3932 | -5.3336 | 3.8167 |
| C | 2.1798 | -6.7707 | 6.1899 |
| C | 5.2457 | -1.5621 | -13.7286 |
| C | 6.7223 | 0.5351 | -13.7960 |
| C | 7.7317 | -1.7542 | -14.3650 |
| C | 6.4198 | -0.8470 | -14.5517 |
| C | 4.9405 | -0.5955 | -12.5932 |
| C | 5.8554 | 0.4795 | -12.5554 |
| C | 5.8996 | 1.3200 | -11.4467 |
| C | 4.9739 | 1.1528 | -10.4096 |
| N | 2.9875 | 0.1026 | -9.4896 |
| C | 4.0015 | 0.1426 | -10.4970 |
| C | 3.9912 | -0.7491 | -11.5911 |
| C | 8.1943 | 0.4520 | -13.4515 |
| C | 8.7492 | -0.8093 | -13.7591 |
| C | 10.0937 | -1.0602 | -13.4997 |
| C | 10.8956 | -0.0591 | -12.9399 |
| N | 11.2096 | 2.1579 | -12.0352 |
| C | 10.3438 | 1.2014 | -12.6508 |
| C | 8.9786 | 1.4572 | -12.9038 |
| C | 7.3043 | -2.8226 | -13.3757 |
| C | 5.9230 | -2.7627 | -13.0890 |
| C | 5.3542 | -3.6690 | -12.1974 |
| C | 6.1492 | -4.6462 | -11.5923 |
| N | 8.2798 | -5.6595 | -11.1272 |
| C | 7.5273 | -4.6938 | -11.8647 |
| C | 8.1144 | -3.7644 | -12.7514 |
| C | 23.1777 | -6.5425 | -2.2319 |
| O | -1.3193 | 0.9176 | -6.6202 |
| O | 2.6407 | -2.9237 | -6.9524 |
| C | -1.4770 | 1.9168 | -7.6554 |
| C | 2.6652 | -4.0413 | -6.0260 |
| O | 21.6699 | -2.3686 | 8.6224 |
| C | 21.8669 | -3.7989 | 8.5079 |
| O | 23.8724 | 1.0727 | 4.8934 |
| C | 23.6127 | 2.4959 | 4.9632 |
| O | 12.3819 | 11.3518 | 1.4450 |
| O | 11.6848 | 11.7835 | -4.0228 |
| C | 13.6626 | 10.9529 | 1.9918 |
| C | 10.3538 | 11.8294 | -4.5955 |
| O | 7.9021 | -14.7740 | 2.6711 |
| O | 11.3632 | -11.0232 | 0.5325 |
| C | 11.4408 | -9.7028 | 1.1238 |
| C | 7.6706 | -16.0341 | 1.9942 |
| O | 20.3092 | -8.7742 | -5.6464 |
| C | 20.8644 | -7.9532 | -6.7025 |
| O | 22.0083 | -8.1447 | -0.4190 |
| C | 21.4516 | -8.9648 | 0.6369 |
| O | 11.4251 | -6.6237 | 13.7818 |
| C | 12.4576 | -6.9426 | 14.7460 |
| O | 11.6752 | -1.9307 | 10.8645 |
| C | 10.6439 | -1.5900 | 9.9047 |
| O | 10.6380 | 6.0845 | -12.4683 |
| O | 15.0255 | 4.6059 | -9.4395 |
| C | 10.2392 | 7.4781 | -12.5324 |
| C | 15.4084 | 3.2135 | -9.3277 |
| O | -1.2534 | 5.5352 | 3.9945 |
| C | -1.3302 | 5.8557 | 5.4042 |
| O | 2.3952 | 8.0430 | 0.6893 |
| C | 2.4672 | 7.7264 | -0.7254 |
| O | 9.5450 | 7.2095 | 8.9988 |
| O | 14.5448 | 8.6806 | 10.8447 |
| C | 15.0495 | 10.0171 | 10.6061 |
| C | 8.9724 | 5.9211 | 9.3463 |
| O | -1.3023 | -5.6326 | 2.8184 |
| O | 2.1675 | -4.1534 | 6.8647 |
| C | -1.4179 | -7.0156 | 2.4047 |
| C | 2.3900 | -2.7546 | 7.1815 |
| O | 25.1298 | 6.7330 | -4.1127 |
| C | 26.4155 | 6.9624 | -3.4805 |
| O | 22.7115 | 1.7503 | -3.8502 |
| C | 21.4328 | 1.5142 | -4.4943 |
| O | 11.6274 | -7.3032 | -12.5206 |
| C | 12.8469 | -7.8181 | -13.1086 |
| O | 9.7538 | -9.3104 | -7.7181 |
| C | 8.7946 | -8.5494 | -6.9400 |
| C | 3.2906 | -9.9978 | 11.0628 |
| C | 4.8440 | -13.4221 | 8.6274 |
| C | 7.5218 | -11.5692 | 11.5158 |
| C | 4.8569 | -12.2751 | 11.1686 |
| H | 4.2082 | -12.0669 | 5.9648 |
| H | 2.8716 | -10.2546 | 4.8875 |
| H | 2.9594 | -7.9918 | 8.5714 |
| H | 9.4880 | -10.2149 | 9.3232 |
| H | 10.2862 | -10.6989 | 7.0253 |
| H | 6.4325 | -12.3769 | 6.0274 |
| H | 4.1651 | -7.1226 | 10.1878 |
| H | 6.0161 | -5.5270 | 10.7094 |
| H | 8.7905 | -8.8398 | 11.0291 |
| C | -6.3978 | -2.2878 | 0.6446 |
| C | -6.5680 | 2.0360 | 1.7402 |
| C | -6.4227 | 0.9254 | -2.5134 |
| C | -7.5024 | 0.1481 | -0.0781 |
| H | -4.0538 | 1.8891 | 3.4866 |
| H | -2.4777 | 0.3449 | 4.6583 |
| H | -3.4303 | -2.7810 | 1.8193 |
| H | -3.5723 | 2.2447 | -3.0516 |
| H | -2.0831 | 4.0196 | -2.1730 |
| H | -3.6306 | 3.3629 | 1.8135 |
| H | -3.7165 | -3.5057 | -0.2779 |
| H | -2.1857 | -3.6755 | -2.2386 |
| H | -3.6553 | 0.1307 | -3.7066 |
| C | 7.7809 | 15.7696 | 4.7473 |
| C | 3.7679 | 14.3689 | 4.2930 |
| C | 5.7917 | 13.2603 | 8.0926 |
| C | 5.4894 | 15.3208 | 6.2651 |
| H | 4.4587 | 12.2514 | 1.9419 |
| H | 6.1691 | 11.8476 | 0.1839 |
| H | 9.1877 | 13.6541 | 2.6975 |
| H | 6.1966 | 10.0673 | 7.3440 |
| H | 4.6478 | 8.4049 | 6.3318 |
| H | 3.0621 | 11.4644 | 3.7080 |
| H | 10.2538 | 14.0479 | 4.7279 |
| H | 11.6110 | 12.4795 | 6.1210 |
| H | 8.0196 | 11.1756 | 8.1633 |
| H | 3.1125 | 9.1648 | 2.4620 |
| H | 0.6508 | 7.4031 | 4.7723 |
| H | 0.5794 | 6.0915 | -0.0684 |
| H | -1.3577 | 3.9153 | 2.2043 |
| C | 18.4672 | 4.8286 | 14.2016 |
| C | 16.4718 | 1.5345 | 16.5460 |
| C | 20.2690 | 0.6907 | 14.6946 |
| C | 18.9112 | 2.7532 | 15.9695 |
| H | 13.8820 | 1.8178 | 15.0182 |
| H | 12.5507 | 3.3216 | 13.5322 |
| H | 16.2064 | 4.9574 | 11.8983 |
| H | 19.1083 | -1.3335 | 12.4570 |
| H | 17.2156 | -2.9181 | 12.2063 |
| H | 14.6956 | -0.3063 | 14.5511 |
| H | 18.3307 | 4.7045 | 11.1057 |
| H | 19.6756 | 3.7313 | 9.2429 |
| H | 20.6128 | 0.2951 | 11.7134 |
| H | 14.3700 | 6.8933 | 12.2661 |
| H | 11.1857 | 5.7034 | 10.6295 |
| H | 12.8950 | 10.1880 | 9.2070 |
| H | 9.3993 | -6.9623 | 12.8743 |
| H | 9.6804 | -3.8459 | 10.7126 |
| H | 13.4310 | -4.7204 | 13.9090 |
| H | 13.5229 | -1.4215 | 12.0231 |
| H | 9.5618 | 9.0891 | 7.7630 |
| H | -1.2335 | -3.2446 | 2.2894 |
| C | 21.1330 | 11.8439 | -5.9132 |
| C | 19.4883 | 10.9508 | -10.1536 |
| C | 17.6025 | 13.8318 | -7.3856 |
| C | 20.0814 | 12.8931 | -8.2649 |
| H | 19.4002 | 7.9486 | -9.7114 |
| H | 20.6093 | 6.1624 | -8.4523 |
| H | 21.2789 | 8.9227 | -5.1763 |
| H | 14.9048 | 12.4144 | -7.5430 |
| H | 13.5532 | 10.6606 | -8.7062 |
| H | 17.1854 | 8.6082 | -9.8534 |
| H | 19.6129 | 9.8326 | -3.8253 |
| H | 17.6303 | 10.0183 | -2.3395 |
| H | 15.6143 | 12.4273 | -5.3072 |
| H | 13.8395 | 11.0286 | -4.2202 |
| H | 10.3757 | 3.9029 | -12.9651 |
| H | 3.2783 | -1.9381 | -8.9011 |
| H | 13.2876 | 2.8914 | -10.7421 |
| H | 12.4233 | 7.7941 | -11.2406 |
| H | 15.4495 | 6.7998 | -9.1608 |
| C | 13.3219 | -16.0990 | -7.4170 |
| C | 17.6490 | -15.2408 | -6.8917 |
| C | 15.4612 | -16.9194 | -3.5048 |
| C | 15.6409 | -17.0580 | -6.2673 |
| H | 17.3615 | -12.2860 | -7.6299 |
| H | 15.8700 | -10.6516 | -8.7860 |
| H | 12.4129 | -12.9979 | -7.6711 |
| H | 15.5907 | -14.2733 | -1.6692 |
| H | 17.2293 | -12.4494 | -1.2743 |
| H | 18.2629 | -12.4525 | -5.4714 |
| H | 11.2193 | -14.2120 | -6.1636 |
| H | 10.0448 | -13.8172 | -4.0035 |
| H | 13.5375 | -15.2043 | -1.8416 |
| H | 11.7836 | -10.3769 | -7.5089 |
| H | 11.1161 | -12.1846 | -1.4689 |
| H | 12.9004 | -9.1784 | -10.9360 |
| H | 8.5567 | -7.3730 | -9.2395 |
| H | 9.1229 | -14.9027 | 0.1879 |
| H | 10.2244 | -10.9428 | 3.0450 |
| C | 29.7286 | -2.0347 | 1.8151 |
| C | 28.6068 | -5.8493 | -0.0037 |
| C | 29.3588 | -2.3199 | -2.6638 |
| C | 30.2365 | -3.6408 | -0.3950 |
| H | 25.8320 | -6.0010 | 1.3826 |
| H | 24.5571 | -5.1080 | 3.3345 |
| H | 26.8225 | -1.4303 | 3.1423 |
| H | 26.5474 | -2.3021 | -3.9066 |
| H | 24.6826 | -3.8729 | -4.4463 |
| H | 25.5914 | -6.1783 | -0.9035 |
| H | 27.8500 | 0.3200 | 1.9955 |
| H | 26.7942 | 2.1685 | 0.6863 |
| H | 26.8397 | -0.3020 | -2.8612 |
| H | 24.5091 | -0.7599 | 3.7443 |
| H | 24.4282 | 1.0882 | -2.5790 |
| H | 23.2532 | -3.0853 | 6.4679 |
| H | 22.3005 | 1.7910 | 7.0556 |
| H | 22.2287 | -6.9902 | -4.7519 |
| H | 20.1185 | -9.9546 | -1.3191 |
| H | 26.0976 | 4.5146 | -2.7730 |
| H | 21.7389 | 3.9703 | -5.1829 |
| H | 18.7604 | -10.2336 | -4.8425 |
| H | 9.6555 | -6.2027 | -12.6824 |
| H | 23.3679 | 7.4179 | -5.3199 |
| H | 0.6733 | 0.7799 | -8.5514 |
| H | 0.7013 | -2.7447 | -4.9909 |
| H | 9.7223 | 12.0958 | -2.0999 |
| H | 14.3461 | 11.0727 | -0.4797 |
| H | 20.5432 | -0.5851 | 9.4319 |
| H | 10.2087 | 11.6569 | 1.6625 |
| H | 8.0128 | -13.6240 | 4.6033 |
| H | -1.4230 | 0.1921 | -4.3249 |
| H | 0.5400 | -2.4516 | 5.4029 |
| H | 0.3828 | -7.3248 | 4.2329 |
| H | 2.9336 | -6.2876 | 6.8394 |
| C | 4.0375 | -1.9034 | -14.6155 |
| C | 6.3965 | 1.7695 | -14.6665 |
| C | 8.2230 | -2.3649 | -15.6959 |
| C | 6.0793 | -0.6312 | -16.0190 |
| H | 6.6631 | 2.0925 | -11.3666 |
| H | 5.0124 | 1.7914 | -9.5289 |
| H | 3.2440 | -1.5424 | -11.6482 |
| H | 10.5265 | -2.0358 | -13.7407 |
| H | 11.9462 | -0.2503 | -12.7247 |
| H | 8.5395 | 2.4304 | -12.6715 |
| H | 4.2957 | -3.6060 | -11.9467 |
| H | 5.7163 | -5.3558 | -10.8885 |
| H | 9.1905 | -3.7671 | -12.9093 |
| H | 23.1817 | -6.3537 | -1.1403 |
| H | -2.3403 | 2.4879 | -7.2858 |
| H | 0.7112 | 3.1677 | -7.1228 |
| H | -1.6970 | 1.4421 | -8.6155 |
| H | 3.5307 | -4.6085 | -6.3877 |
| H | 2.8277 | -3.6850 | -4.9995 |
| H | 1.7462 | -4.6262 | -6.1101 |
| H | 21.3102 | -4.1696 | 9.3784 |
| H | 21.4317 | -4.1704 | 7.5756 |
| H | 22.9306 | -4.0435 | 8.5882 |
| H | 24.1740 | 2.8657 | 4.0951 |
| H | 22.5427 | 2.6946 | 4.8529 |
| H | 24.0111 | 2.9093 | 5.8948 |
| H | 13.4467 | 10.9345 | 3.0681 |
| H | 13.9390 | 9.9580 | 1.6298 |
| H | 14.4254 | 11.7008 | 1.7550 |
| H | 10.5764 | 11.8076 | -5.6696 |
| H | 9.7761 | 10.9416 | -4.2995 |
| H | 9.8542 | 12.7585 | -4.3103 |
| H | 12.0787 | -9.1730 | 0.3934 |
| H | 10.4458 | -9.2506 | 1.1818 |
| H | 11.9234 | -9.7528 | 2.1054 |
| H | 7.0044 | -16.5481 | 2.6985 |
| H | 7.1724 | -15.8615 | 1.0357 |
| H | 8.6110 | -16.5811 | 1.8722 |
| H | 20.3265 | -8.3301 | -7.5820 |
| H | 20.6365 | -6.8983 | -6.5244 |
| H | 21.9407 | -8.1288 | -6.7950 |
| H | 21.9961 | -8.5950 | 1.5152 |
| H | 20.3774 | -8.7826 | 0.7343 |
| H | 21.6714 | -10.0213 | 0.4543 |
| H | 12.1087 | -7.9086 | 15.1328 |
| H | 13.4261 | -7.0452 | 14.2471 |
| H | 12.4851 | -6.1883 | 15.5380 |
| H | 11.0078 | -0.6234 | 9.5245 |
| H | 10.6071 | -2.3353 | 9.1045 |
| H | 9.6788 | -1.4777 | 10.4054 |
| H | 9.3226 | 7.4188 | -13.1320 |
| H | 10.0309 | 7.8635 | -11.5276 |
| H | 11.0048 | 8.0697 | -13.0426 |
| H | 16.2951 | 3.2784 | -8.6836 |
| H | 14.6112 | 2.6380 | -8.8470 |
| H | 15.6664 | 2.8078 | -10.3105 |
| H | -2.1897 | 5.2503 | 5.7248 |
| H | 0.9678 | 4.9948 | 6.3314 |
| H | -1.5225 | 6.9219 | 5.5502 |
| H | 3.2682 | 8.3950 | -1.0610 |
| H | 2.7485 | 6.6733 | -0.8672 |
| H | 1.5210 | 7.9626 | -1.2173 |
| H | 16.0062 | 9.9928 | 11.1433 |
| H | 15.2045 | 10.1765 | 9.5348 |
| H | 14.3751 | 10.7643 | 11.0356 |
| H | 7.9985 | 5.9670 | 8.8441 |
| H | 9.5831 | 5.1097 | 8.9413 |
| H | 8.8420 | 5.8343 | 10.4308 |
| H | -2.2476 | -6.9638 | 1.6868 |
| H | 0.9259 | -7.4832 | 1.3159 |
| H | -1.6684 | -7.6595 | 3.2525 |
| H | 3.1706 | -2.8194 | 7.9475 |
| H | 2.7517 | -2.2089 | 6.2987 |
| H | 1.4766 | -2.3074 | 7.5819 |
| H | 26.6084 | 8.0131 | -3.7394 |
| H | 26.3334 | 6.8330 | -2.3954 |
| H | 27.1771 | 6.3053 | -3.9161 |
| H | 21.2474 | 0.4598 | -4.2428 |
| H | 20.6626 | 2.1625 | -4.0610 |
| H | 21.5230 | 1.6497 | -5.5782 |
| H | 12.8958 | -7.2541 | -14.0486 |
| H | 13.7031 | -7.5888 | -12.4670 |
| H | 12.7558 | -8.8921 | -13.2978 |
| H | 8.5343 | -9.2754 | -6.1518 |
| H | 9.2759 | -7.6621 | -6.5120 |
| H | 7.9168 | -8.2863 | -7.5362 |
| H | 29.6466 | -3.3280 | -3.0280 |
| H | 30.2582 | -1.8963 | -2.1680 |
| C | 29.0323 | -1.4315 | -3.8709 |
| H | 15.7352 | -17.1037 | -7.3611 |
| H | 14.8603 | -17.7746 | -5.9799 |
| H | 16.5874 | -17.4251 | -5.8454 |
| H | 16.4374 | -17.2908 | -3.8820 |
| H | 14.6979 | -17.6592 | -3.8242 |
| C | 15.5244 | -16.9099 | -1.9721 |
| H | 17.2879 | -15.6329 | -7.8655 |
| H | 17.9810 | -16.1217 | -6.3019 |
| C | 18.8777 | -14.3574 | -7.1417 |
| H | 14.1077 | -16.4280 | -8.1298 |
| C | 12.1291 | -15.5969 | -8.2379 |
| H | 13.0122 | -17.0016 | -6.8503 |
| H | 29.2652 | -5.7915 | 0.8883 |
| H | 29.2781 | -5.9181 | -0.8860 |
| C | 27.7973 | -7.1502 | 0.0669 |
| H | 30.2069 | -3.0142 | 2.0302 |
| C | 29.5035 | -1.3208 | 3.1540 |
| H | 30.4609 | -1.4539 | 1.2171 |
| H | 30.6007 | -4.1176 | 0.5259 |
| H | 30.8730 | -2.7633 | -0.5762 |
| H | 30.4161 | -4.3477 | -1.2166 |
| H | 3.6408 | -0.9546 | -15.0372 |
| C | 2.8764 | -2.6575 | -13.9588 |
| H | 4.3941 | -2.5048 | -15.4785 |
| H | 5.1895 | 0.0024 | -16.1441 |
| H | 5.8735 | -1.5784 | -16.5366 |
| H | 6.9021 | -0.1408 | -16.5557 |
| H | 8.6006 | -1.5402 | -16.3352 |
| H | 7.3540 | -2.8026 | -16.2313 |
| C | 9.3071 | -3.4452 | -15.5918 |
| H | 5.4100 | 1.6142 | -15.1543 |
| H | 7.1432 | 1.8247 | -15.4847 |
| C | 6.3536 | 3.1188 | -13.9403 |
| H | 20.5962 | 10.9714 | -10.1028 |
| H | 19.1677 | 11.9798 | -10.4220 |
| C | 19.0590 | 10.0145 | -11.2906 |
| H | 21.0659 | 12.5108 | -8.5643 |
| H | 20.2537 | 13.7025 | -7.5413 |
| H | 19.6263 | 13.3501 | -9.1546 |
| H | 21.9293 | 11.7195 | -6.6755 |
| C | 21.6836 | 11.4250 | -4.5439 |
| H | 20.9079 | 12.9306 | -5.8702 |
| H | 17.6445 | 14.0682 | -8.4694 |
| H | 18.4779 | 14.3339 | -6.9215 |
| C | 16.3347 | 14.4591 | -6.7925 |
| H | -7.1351 | -1.8691 | 1.3622 |
| C | -5.8919 | -3.6169 | 1.2187 |
| H | -6.9565 | -2.4903 | -0.2922 |
| H | -7.9262 | -0.1573 | 0.8891 |
| H | -7.8652 | -0.5685 | -0.8272 |
| H | -7.9443 | 1.1238 | -0.3266 |
| H | -7.1477 | 1.6536 | -2.0915 |
| H | -6.9912 | -0.0107 | -2.6984 |
| C | -5.9352 | 1.4757 | -3.8596 |
| H | -7.1530 | 1.3153 | 2.3491 |
| H | -7.2641 | 2.4280 | 0.9679 |
| C | -6.1481 | 3.2112 | 2.6320 |
| H | 4.7647 | 13.6783 | 8.0293 |
| H | 6.4409 | 14.0805 | 8.4632 |
| C | 5.7756 | 12.1321 | 9.1320 |
| H | 6.8935 | 16.3510 | 4.4191 |
| C | 8.9427 | 16.1087 | 3.8045 |
| H | 8.0299 | 16.1292 | 5.7671 |
| H | 4.1364 | 15.3949 | 4.0777 |
| H | 3.2018 | 14.4345 | 5.2466 |
| C | 2.7941 | 13.9703 | 3.1801 |
| H | 5.3116 | 16.1236 | 5.5358 |
| H | 6.2082 | 15.7078 | 6.9997 |
| H | 4.5415 | 15.1533 | 6.7954 |
| H | 3.7821 | -13.4601 | 8.9516 |
| H | 5.4267 | -13.9693 | 9.3980 |
| C | 4.9834 | -14.1846 | 7.3035 |
| H | 3.8092 | -12.5676 | 11.0091 |
| H | 4.9185 | -11.8314 | 12.1706 |
| H | 5.4518 | -13.1994 | 11.1869 |
| H | 2.7591 | -10.9694 | 10.9816 |
| C | 2.2423 | -8.8869 | 10.9320 |
| H | 3.7289 | -9.9656 | 12.0820 |
| H | 7.3297 | -12.6589 | 11.4198 |
| H | 7.0165 | -11.2436 | 12.4489 |
| C | 9.0352 | -11.3754 | 11.6753 |
| H | 16.3811 | 2.5584 | 16.9637 |
| H | 17.3578 | 1.0749 | 17.0341 |
| C | 15.2380 | 0.7196 | 16.9544 |
| H | 18.3464 | 3.5382 | 16.4905 |
| H | 19.9018 | 3.1651 | 15.7360 |
| H | 19.0649 | 1.9353 | 16.6883 |
| H | 18.0693 | 4.9881 | 15.2260 |
| C | 18.1319 | 6.0745 | 13.3718 |
| H | 19.5702 | 4.7523 | 14.3014 |
| H | 19.9885 | 0.4811 | 15.7487 |
| H | 20.9825 | 1.5402 | 14.7225 |
| C | 20.9938 | -0.5469 | 14.1504 |
| H | 19.1967 | 8.9526 | -10.9923 |
| C | 19.8514 | 10.2918 | -12.5669 |
| H | 17.9763 | 10.1403 | -11.4948 |
| H | 20.9239 | 10.1169 | -12.4253 |
| H | 19.7282 | 11.3276 | -12.9029 |
| H | 19.5188 | 9.6424 | -13.3849 |
| H | 16.3664 | 14.3988 | -5.6856 |
| H | 15.4345 | 13.8883 | -7.1100 |
| C | 16.1866 | 15.9191 | -7.2167 |
| H | 17.0481 | 16.5207 | -6.9053 |
| H | 15.2949 | 16.3705 | -6.7665 |
| H | 16.0911 | 16.0188 | -8.3038 |
| H | 21.7606 | 10.3178 | -4.4794 |
| H | 20.9795 | 11.7338 | -3.7450 |
| C | 23.0538 | 12.0475 | -4.2816 |
| H | 23.7969 | 11.7182 | -5.0165 |
| H | 23.4276 | 11.7712 | -3.2892 |
| H | 23.0147 | 13.1422 | -4.3219 |
| H | 11.3945 | -15.0811 | -7.5815 |
| C | 11.4384 | -16.7451 | -8.9726 |
| H | 12.4724 | -14.8414 | -8.9742 |
| H | 11.0370 | -17.4889 | -8.2752 |
| H | 12.1276 | -17.2657 | -9.6471 |
| H | 10.6025 | -16.3792 | -9.5796 |
| H | 8.8815 | -4.3651 | -15.1434 |
| H | 10.1199 | -3.1127 | -14.9098 |
| C | 9.8956 | -3.7745 | -16.9626 |
| H | 9.1255 | -4.1196 | -17.6619 |
| H | 10.6477 | -4.5680 | -16.8881 |
| H | 10.3829 | -2.9032 | -17.4149 |
| C | 19.8717 | -15.0419 | -8.0786 |
| H | 19.3787 | -14.1270 | -6.1798 |
| H | 18.5718 | -13.3811 | -7.5767 |
| H | 20.2108 | -16.0033 | -7.6765 |
| H | 20.7614 | -14.4201 | -8.2302 |
| H | 19.4340 | -15.2336 | -9.0648 |
| H | 28.0328 | -1.6864 | -4.2846 |
| C | 30.0868 | -1.5760 | -4.9669 |
| H | 28.9754 | -0.3705 | -3.5533 |
| H | 30.1306 | -2.6001 | -5.3551 |
| H | 31.0878 | -1.3189 | -4.6026 |
| H | 29.8676 | -0.9147 | -5.8130 |
| C | 30.8295 | -0.9338 | 3.8063 |
| H | 28.9330 | -1.9780 | 3.8412 |
| H | 28.8831 | -0.4095 | 3.0079 |
| H | 31.4645 | -1.8093 | 3.9834 |
| H | 30.6640 | -0.4503 | 4.7760 |
| H | 31.3994 | -0.2342 | 3.1845 |
| C | 22.3467 | -0.7427 | 14.8323 |
| H | 21.1440 | -0.4567 | 13.0520 |
| H | 20.3683 | -1.4485 | 14.3073 |
| H | 23.0188 | 0.1038 | 14.6498 |
| H | 22.8486 | -1.6430 | 14.4597 |
| H | 22.2420 | -0.8520 | 15.9175 |
| H | 18.3136 | 5.8854 | 12.2920 |
| C | 18.9585 | 7.2783 | 13.8219 |
| H | 17.0541 | 6.3131 | 13.4681 |
| H | 20.0321 | 7.1077 | 13.6795 |
| H | 18.7999 | 7.5053 | 14.8821 |
| H | 18.6918 | 8.1747 | 13.2513 |
| H | 9.8087 | 15.4398 | 4.0028 |
| C | 9.3778 | 17.5645 | 3.9610 |
| H | 8.6432 | 15.9246 | 2.7529 |
| H | 9.7388 | 17.7743 | 4.9741 |
| H | 8.5546 | 18.2579 | 3.7547 |
| H | 10.1904 | 17.8084 | 3.2668 |
| H | 2.4312 | 12.9313 | 3.3382 |
| H | 3.3119 | 13.9727 | 2.1935 |
| C | 1.6042 | 14.9252 | 3.1163 |
| H | 1.0356 | 14.9265 | 4.0545 |
| H | 0.9181 | 14.6413 | 2.3088 |
| H | 1.9207 | 15.9571 | 2.9255 |
| H | -5.3365 | 2.9015 | 3.3258 |
| C | -7.3305 | 3.7447 | 3.4388 |
| H | -5.7311 | 4.0277 | 2.0085 |
| H | -7.7403 | 2.9828 | 4.1116 |
| H | -8.1461 | 4.0817 | 2.7883 |
| H | -7.0322 | 4.6002 | 4.0551 |
| H | -5.0819 | 0.8754 | -4.2409 |
| H | -5.5562 | 2.5091 | -3.7296 |
| C | -7.0588 | 1.4728 | -4.8951 |
| H | -7.3982 | 0.4545 | -5.1238 |
| H | -6.7275 | 1.9220 | -5.8377 |
| H | -7.9337 | 2.0341 | -4.5472 |
| H | 7.2340 | 3.2279 | -13.2709 |
| H | 5.4525 | 3.1643 | -13.2846 |
| C | 6.3138 | 4.2833 | -14.9271 |
| H | 7.2152 | 4.3210 | -15.5491 |
| H | 6.2357 | 5.2426 | -14.4022 |
| H | 5.4520 | 4.2135 | -15.6016 |
| C | 15.5980 | -18.3293 | -1.4117 |
| H | 14.6344 | -16.3925 | -1.5506 |
| H | 16.4094 | -16.3351 | -1.6340 |
| H | 14.7076 | -18.9134 | -1.6713 |
| H | 15.6721 | -18.3164 | -0.3183 |
| H | 16.4708 | -18.8712 | -1.7926 |
| H | 3.2087 | -3.6664 | -13.6240 |
| C | 1.7023 | -2.8140 | -14.9223 |
| H | 2.5358 | -2.1231 | -13.0450 |
| H | 2.0045 | -3.2929 | -15.8607 |
| H | 1.2585 | -1.8434 | -15.1789 |
| H | 0.9129 | -3.4344 | -14.4820 |
| H | -5.5273 | -3.4641 | 2.2547 |
| H | -5.0228 | -3.9890 | 0.6346 |
| C | -6.9920 | -4.6768 | 1.2208 |
| H | -7.8799 | -4.3422 | 1.7744 |
| H | -6.6423 | -5.5999 | 1.6958 |
| H | -7.3155 | -4.9243 | 0.2039 |
| H | 6.7399 | 11.5784 | 9.1190 |
| H | 4.9896 | 11.3944 | 8.8775 |
| C | 5.5223 | 12.6764 | 10.5366 |
| H | 6.3144 | 13.3647 | 10.8535 |
| H | 5.4825 | 11.8650 | 11.2725 |
| H | 4.5719 | 13.2187 | 10.5971 |
| C | 14.9441 | 0.8667 | 18.4462 |
| H | 15.3969 | -0.3512 | 16.7135 |
| H | 14.3496 | 1.0414 | 16.3681 |
| H | 15.7920 | 0.5416 | 19.0598 |
| H | 14.0794 | 0.2599 | 18.7383 |
| H | 14.7219 | 1.9052 | 18.7163 |
| C | 28.6924 | -8.3421 | 0.4022 |
| H | 27.2912 | -7.3328 | -0.9028 |
| H | 26.9916 | -7.0688 | 0.8277 |
| H | 29.4947 | -8.4661 | -0.3340 |
| H | 28.1164 | -9.2745 | 0.4163 |
| H | 29.1623 | -8.2326 | 1.3862 |
| C | 4.2473 | -15.5221 | 7.3523 |
| H | 6.0548 | -14.3587 | 7.0818 |
| H | 4.5795 | -13.5743 | 6.4660 |
| H | 4.6020 | -16.1518 | 8.1759 |
| H | 4.3864 | -16.0871 | 6.4245 |
| H | 3.1673 | -15.3818 | 7.4889 |
| H | 2.7315 | -7.9035 | 10.7353 |
| C | 1.3821 | -8.7638 | 12.1871 |
| H | 1.5925 | -9.0833 | 10.0551 |
| H | 1.9906 | -8.6051 | 13.0850 |
| H | 0.7714 | -9.6564 | 12.3582 |
| H | 0.7076 | -7.8979 | 12.1082 |
| C | 9.5267 | -11.9188 | 13.0160 |
| H | 9.3002 | -10.2987 | 11.5928 |
| H | 9.5685 | -11.8908 | 10.8517 |
| H | 9.0628 | -11.3960 | 13.8602 |
| H | 10.6118 | -11.8009 | 13.1140 |
| H | 9.3025 | -12.9857 | 13.1270 |
| C | -5.5362 | -11.0962 | 9.5050 |
| C | -4.0398 | -12.3891 | 7.8721 |
| C | -3.0544 | -11.4809 | 10.0655 |
| C | -4.3579 | -12.1776 | 9.4277 |
| C | -5.7161 | -10.6359 | 8.0646 |
| C | -4.8728 | -11.3323 | 7.1733 |
| C | -4.8189 | -10.9699 | 5.8293 |
| C | -5.6379 | -9.9415 | 5.3524 |
| N | -7.3551 | -8.2727 | 5.6734 |
| C | -6.4814 | -9.2530 | 6.2407 |
| C | -6.5103 | -9.5879 | 7.6106 |
| C | -2.5698 | -12.0350 | 7.7487 |
| C | -2.0428 | -11.4890 | 8.9396 |
| C | -0.7162 | -11.0684 | 8.9807 |
| C | 0.0983 | -11.1944 | 7.8490 |
| N | 0.4188 | -11.9919 | 5.5444 |
| C | -0.4303 | -11.7469 | 6.6727 |
| C | -1.7763 | -12.1699 | 6.6182 |
| C | -3.5300 | -10.0918 | 10.4501 |
| C | -4.9238 | -9.9408 | 10.2718 |
| C | -5.5715 | -8.7873 | 10.7028 |
| C | -4.8304 | -7.7466 | 11.2683 |
| N | -2.7572 | -6.7640 | 11.9830 |
| C | -3.4319 | -7.8603 | 11.3629 |
| C | -2.7721 | -9.0377 | 10.9475 |
| C | -15.2314 | -1.0369 | 0.9922 |
| C | -15.1143 | 1.4261 | 1.7242 |
| C | -15.4504 | 0.8345 | -0.7543 |
| C | -15.8707 | 0.4103 | 0.7345 |
| C | -14.2282 | -0.8041 | 2.1105 |
| C | -14.0957 | 0.5624 | 2.4397 |
| C | -13.0942 | 0.9728 | 3.3168 |
| C | -12.2538 | 0.0243 | 3.9114 |
| N | -11.5573 | -2.2682 | 4.2826 |
| C | -12.4148 | -1.3383 | 3.6108 |
| C | -13.4149 | -1.7581 | 2.7082 |
| C | -14.4488 | 2.4214 | 0.7935 |
| C | -14.6625 | 2.1151 | -0.5679 |
| C | -14.1357 | 2.9355 | -1.5617 |
| C | -13.3648 | 4.0484 | -1.2111 |
| N | -12.3304 | 5.4966 | 0.4074 |
| C | -13.1259 | 4.3368 | 0.1446 |
| C | -13.6911 | 3.5271 | 1.1542 |
| C | -14.5167 | -0.2742 | -1.2067 |
| C | -14.4349 | -1.3255 | -0.2691 |
| C | -13.5968 | -2.4119 | -0.5119 |
| C | -12.8312 | -2.4564 | -1.6816 |
| N | -12.0676 | -1.5002 | -3.7631 |
| C | -12.8808 | -1.3866 | -2.5912 |
| C | -13.7341 | -0.2873 | -2.3553 |
| C | -3.8060 | 16.1274 | 3.6066 |
| C | -6.1986 | 15.3469 | 3.0901 |
| C | -5.3074 | 15.2714 | 5.5007 |
| C | -5.3081 | 16.1508 | 4.1630 |
| C | -3.9450 | 15.5761 | 2.2004 |
| C | -5.2423 | 15.0791 | 1.9469 |
| C | -5.5255 | 14.4708 | 0.7267 |
| C | -4.5354 | 14.3830 | -0.2575 |
| N | -2.3368 | 14.9065 | -1.1087 |
| C | -3.2544 | 14.9115 | -0.0140 |
| C | -2.9539 | 15.5060 | 1.2300 |
| C | -6.6347 | 14.0977 | 3.8321 |
| C | -6.2653 | 14.1364 | 5.1954 |
| C | -6.6925 | 13.1446 | 6.0730 |
| C | -7.4509 | 12.0749 | 5.5910 |
| N | -8.5494 | 10.8738 | 3.8354 |
| C | -7.7482 | 11.9913 | 4.2183 |
| C | -7.3349 | 13.0075 | 3.3291 |
| C | -3.9124 | 14.6621 | 5.5464 |
| C | -3.1050 | 15.1029 | 4.4780 |
| C | -1.8452 | 14.5444 | 4.2725 |
| C | -1.3646 | 13.5695 | 5.1513 |
| N | -1.5900 | 12.1687 | 7.1008 |
| C | -2.1690 | 13.1340 | 6.2195 |
| C | -3.4555 | 13.6784 | 6.4171 |
| C | -8.4502 | 10.2911 | 2.6816 |
| C | -9.3145 | 9.1263 | 2.3965 |
| C | -9.8449 | 8.3435 | 3.4306 |
| C | -10.6171 | 7.2201 | 3.1269 |
| C | -10.8959 | 6.8546 | 1.7834 |
| C | -10.3844 | 7.6474 | 0.7523 |
| C | -9.5988 | 8.7667 | 1.0578 |
| C | -11.7016 | 5.6425 | 1.5329 |
| C | 3.7193 | 5.4205 | 14.9046 |
| C | 2.4646 | 3.3296 | 15.7270 |
| C | 5.0031 | 3.2387 | 15.3311 |
| C | 3.8285 | 4.1677 | 15.8979 |
| C | 2.3132 | 5.3193 | 14.3389 |
| C | 1.6601 | 4.1257 | 14.7201 |
| C | 0.4227 | 3.8083 | 14.1663 |
| C | -0.1962 | 4.6912 | 13.2730 |
| N | -0.2566 | 6.8100 | 12.0754 |
| C | 0.4404 | 5.8939 | 12.9283 |
| C | 1.7066 | 6.2114 | 13.4653 |
| C | 2.9207 | 1.9889 | 15.1791 |
| C | 4.3246 | 1.9151 | 15.0467 |
| C | 4.9334 | 0.7353 | 14.6286 |
| C | 4.1461 | -0.3639 | 14.2789 |
| N | 2.0563 | -1.4296 | 13.8688 |
| C | 2.7427 | -0.2750 | 14.3493 |
| C | 2.1275 | 0.9045 | 14.8238 |
| C | 5.3789 | 3.8823 | 14.0065 |
| C | 4.6954 | 5.0972 | 13.7871 |
| C | 4.8821 | 5.7971 | 12.5972 |
| C | 5.7567 | 5.3011 | 11.6232 |
| N | 7.3644 | 3.6253 | 10.8695 |
| C | 6.4124 | 4.0788 | 11.8380 |
| C | 6.2187 | 3.3552 | 13.0326 |
| C | 0.3330 | 7.3212 | 11.0399 |
| C | -0.3667 | 8.2950 | 10.1772 |
| C | -1.7589 | 8.4318 | 10.1655 |
| C | -2.3592 | 9.3643 | 9.3118 |
| C | -1.5900 | 10.1983 | 8.4627 |
| C | -0.1977 | 10.0654 | 8.4797 |
| C | 0.4028 | 9.1206 | 9.3188 |
| C | -1.6428 | -6.2829 | 11.5301 |
| C | -1.0315 | -5.1189 | 12.2068 |
| C | -1.7568 | -4.3063 | 13.0871 |
| C | -1.1555 | -3.1791 | 13.6555 |
| C | 0.1862 | -2.8250 | 13.3615 |
| C | 0.9150 | -3.6496 | 12.4985 |
| C | 0.3184 | -4.7843 | 11.9392 |
| C | -10.1329 | -4.2130 | 4.3243 |
| C | 0.7743 | -1.6034 | 13.9499 |
| C | -2.2647 | 11.1668 | 7.5715 |
| C | -11.0097 | -3.2469 | 3.6309 |
| C | 7.3215 | 13.2290 | -9.7075 |
| C | 6.1348 | 11.8639 | -11.5382 |
| C | 4.9013 | 13.7915 | -10.3674 |
| C | 6.3249 | 13.3495 | -10.9607 |
| C | 7.6243 | 11.7417 | -9.6166 |
| C | 7.0274 | 11.0081 | -10.6641 |
| C | 7.2335 | 9.6349 | -10.7679 |
| C | 8.0024 | 8.9698 | -9.8090 |
| N | 9.3693 | 8.9824 | -7.8071 |
| C | 8.5412 | 9.6928 | -8.7306 |
| C | 8.3514 | 11.0872 | -8.6282 |
| C | 4.6783 | 11.5352 | -11.2510 |
| C | 3.9910 | 12.6192 | -10.6661 |
| C | 2.6350 | 12.5155 | -10.3656 |
| C | 1.9599 | 11.3159 | -10.6021 |
| N | 1.8937 | 9.0351 | -11.3737 |
| C | 2.6610 | 10.2142 | -11.1246 |
| C | 4.0287 | 10.3229 | -11.4562 |
| C | 5.1337 | 13.8834 | -8.8693 |
| C | 6.4813 | 13.6527 | -8.5203 |
| C | 6.8886 | 13.7557 | -7.1924 |
| C | 5.9478 | 14.0227 | -6.1943 |
| N | 3.6951 | 14.3059 | -5.4264 |
| C | 4.5890 | 14.1706 | -6.5318 |
| C | 4.1818 | 14.1089 | -7.8824 |
| C | 2.4638 | 14.6962 | -5.5587 |
| C | 1.5883 | 14.7341 | -4.3691 |
| C | -0.9656 | 3.2607 | -12.3749 |
| C | -0.0866 | 4.4011 | -12.0424 |
| C | -8.0956 | -0.9025 | -8.9544 |
| C | -8.9832 | -0.7746 | -7.7803 |
| C | 1.3063 | 4.2866 | -11.9715 |
| C | 2.0780 | 5.4059 | -11.6379 |
| C | 1.4901 | 6.6785 | -11.4285 |
| C | 0.0986 | 6.7905 | -11.4979 |
| C | -0.6777 | 5.6604 | -11.7747 |
| C | 2.3500 | 7.8451 | -11.1371 |
| C | 4.5536 | -14.0881 | -6.6503 |
| C | 6.9948 | -13.2839 | -6.6454 |
| C | 6.0645 | -14.6087 | -4.6452 |
| C | 6.0414 | -14.5168 | -6.2437 |
| C | 4.7393 | -12.7906 | -7.4196 |
| C | 6.0723 | -12.3279 | -7.3728 |
| C | 6.4097 | -11.1226 | -7.9836 |
| C | 5.4255 | -10.3660 | -8.6245 |
| N | 3.1778 | -9.9602 | -9.3350 |
| C | 4.0935 | -10.8213 | -8.6575 |
| C | 3.7521 | -12.0551 | -8.0658 |
| C | 7.4855 | -12.7466 | -5.3145 |
| C | 7.0642 | -13.5478 | -4.2296 |
| C | 7.4740 | -13.2555 | -2.9326 |
| C | 8.2606 | -12.1258 | -2.6896 |
| N | 9.4909 | -10.1884 | -3.4929 |
| C | 8.6307 | -11.3000 | -3.7616 |
| C | 8.2486 | -11.6090 | -5.0853 |
| C | 4.6937 | -14.1007 | -4.2187 |
| C | 3.8789 | -13.7836 | -5.3252 |
| C | 2.6376 | -13.1806 | -5.1367 |
| C | 2.1888 | -12.9084 | -3.8420 |
| N | 2.4428 | -12.8704 | -1.4699 |
| C | 3.0032 | -13.2173 | -2.7360 |
| C | 4.2634 | -13.8231 | -2.9254 |
| C | 1.8905 | -10.0161 | -9.1748 |
| C | 3.1294 | -12.7916 | -0.3723 |
| C | 1.0452 | -9.0269 | -9.8770 |
| C | 2.4244 | -12.3740 | 0.8594 |
| C | 1.6125 | -7.9810 | -10.6148 |
| C | 0.7967 | -7.0131 | -11.2076 |
| C | -0.6144 | -7.0605 | -11.0879 |
| C | -1.1855 | -8.1270 | -10.3840 |
| C | -0.3682 | -9.0941 | -9.7864 |
| C | 1.2573 | -11.6043 | 0.8021 |
| C | 0.6111 | -11.2285 | 1.9836 |
| C | 1.0778 | -11.6573 | 3.2519 |
| C | 2.2532 | -12.4145 | 3.3100 |
| C | 2.9301 | -12.7446 | 2.1297 |
| C | 14.5250 | 1.8550 | 1.8562 |
| C | 14.6715 | -0.5626 | 0.9916 |
| C | 14.8549 | 1.4023 | -0.6558 |
| C | 15.2877 | 0.9067 | 0.8092 |
| C | 13.5652 | 0.9215 | 2.5725 |
| C | 13.6024 | -0.3902 | 2.0543 |
| C | 12.6883 | -1.3429 | 2.5009 |
| C | 11.7840 | -1.0198 | 3.5177 |
| N | 10.8208 | 0.5698 | 5.0791 |
| C | 11.7998 | 0.2673 | 4.0808 |
| C | 12.6866 | 1.2517 | 3.5980 |
| C | 14.0141 | -0.8589 | -0.3432 |
| C | 14.0451 | 0.2497 | -1.2172 |
| C | 13.3493 | 0.2014 | -2.4230 |
| C | 12.6828 | -0.9708 | -2.7988 |
| N | 11.9611 | -3.2385 | -2.3631 |
| C | 12.7172 | -2.0959 | -1.9582 |
| C | 13.3808 | -2.0376 | -0.7142 |
| C | 13.9666 | 2.6030 | -0.3936 |
| C | 13.7498 | 2.8241 | 0.9839 |
| C | 12.8698 | 3.8227 | 1.3960 |
| C | 12.2561 | 4.6477 | 0.4475 |
| N | 11.8111 | 5.3184 | -1.8218 |
| C | 12.5187 | 4.4623 | -0.9213 |
| C | 13.3688 | 3.4191 | -1.3458 |
| C | 11.5696 | -5.5890 | -2.6322 |
| C | 11.1564 | 1.0543 | 6.2331 |
| C | 12.3098 | 5.6979 | -2.9569 |
| C | 10.1106 | 1.3932 | 7.2218 |
| C | 11.5040 | 6.5618 | -3.8466 |
| C | 8.7491 | 1.2060 | 6.9655 |
| C | 7.7962 | 1.5868 | 7.9202 |
| C | 8.1837 | 2.1802 | 9.1459 |
| C | 9.5477 | 2.3480 | 9.4149 |
| C | 10.4987 | 1.9501 | 8.4704 |
| C | 10.3715 | -5.4219 | -3.3345 |
| C | 9.5871 | -6.5368 | -3.6500 |
| C | 9.9613 | -7.8430 | -3.2503 |
| C | 11.1695 | -8.0144 | -2.5649 |
| C | 11.9624 | -6.9021 | -2.2674 |
| C | 10.5035 | 7.4056 | -3.3552 |
| C | 9.7607 | 8.1952 | -4.2410 |
| C | 9.9700 | 8.1382 | -5.6407 |
| C | 10.9875 | 7.3089 | -6.1295 |
| C | 11.7500 | 6.5408 | -5.2439 |
| C | 9.0529 | -8.9719 | -3.5403 |
| C | -1.4258 | -5.9720 | -11.6701 |
| C | 9.0938 | 8.9148 | -6.5430 |
| C | -9.4482 | 0.4701 | -7.3392 |
| C | -10.2625 | 0.5497 | -6.2055 |
| C | -10.6401 | -0.6108 | -5.4824 |
| C | -10.1916 | -1.8564 | -5.9345 |
| C | -9.3733 | -1.9355 | -7.0672 |
| C | 0.1964 | 14.9583 | -4.5255 |
| C | -0.6733 | 14.9482 | -3.4313 |
| C | -0.1780 | 14.7261 | -2.1391 |
| C | 1.2169 | 14.5334 | -1.9796 |
| C | 2.0848 | 14.5307 | -3.0779 |
| C | 7.1462 | 2.5955 | 10.1163 |
| C | -1.0679 | 14.6824 | -0.9593 |
| C | 0.2816 | -11.3137 | 4.4500 |
| C | -11.4743 | -0.4647 | -4.2723 |
| C | -9.6526 | -4.0045 | 5.6192 |
| C | -8.8035 | -4.9496 | 6.2128 |
| C | -8.4159 | -6.1276 | 5.5321 |
| C | -8.9090 | -6.3428 | 4.2370 |
| C | -9.7505 | -5.4001 | 3.6441 |
| C | -7.4927 | -7.0857 | 6.1773 |
| C | -6.1145 | -2.0542 | -13.9869 |
| C | -4.8730 | 0.1187 | -14.5766 |
| C | -3.6933 | -2.1463 | -14.8543 |
| C | -5.1017 | -1.3968 | -15.0448 |
| C | -6.3514 | -0.9569 | -12.9641 |
| C | -5.6480 | 0.2261 | -13.2758 |
| C | -5.6530 | 1.3002 | -12.3878 |
| C | -6.4109 | 1.2310 | -11.2159 |
| N | -7.9620 | 0.1062 | -9.7580 |
| C | -7.1351 | 0.0625 | -10.9200 |
| C | -7.0909 | -1.0477 | -11.7904 |
| C | -3.3917 | 0.1904 | -14.2521 |
| C | -2.7738 | -1.0777 | -14.2977 |
| C | -1.4656 | -1.2286 | -13.8432 |
| C | -0.7407 | -0.1094 | -13.4192 |
| N | -0.5089 | 2.2703 | -13.0754 |
| C | -1.3387 | 1.1629 | -13.4379 |
| C | -2.6786 | 1.3146 | -13.8554 |
| C | -3.9816 | -3.2224 | -13.8266 |
| C | -5.3103 | -3.1745 | -13.3533 |
| C | -5.7404 | -4.0841 | -12.3906 |
| C | -4.8554 | -5.0432 | -11.8894 |
| N | -2.7051 | -6.1259 | -11.8234 |
| C | -3.5297 | -5.0866 | -12.3580 |
| C | -3.0932 | -4.1744 | -13.3425 |
| C | 12.4258 | -4.4437 | -2.2535 |
| O | -10.7709 | 1.7222 | -5.6800 |
| O | -8.8732 | -3.1075 | -7.6020 |
| C | -10.4321 | 2.9621 | -6.3507 |
| C | -9.2107 | -4.3486 | -6.9341 |
| O | 6.4316 | 1.4550 | 7.7764 |
| C | 5.9361 | 0.7084 | 6.6363 |
| O | 11.8622 | 2.0355 | 8.6400 |
| C | 12.3667 | 2.7077 | 9.8250 |
| O | 1.6194 | 14.3483 | -0.6700 |
| O | -0.1932 | 15.1562 | -5.8389 |
| C | 3.0425 | 14.2114 | -0.4308 |
| C | -1.5663 | 15.5543 | -6.0742 |
| O | -0.4996 | -10.4071 | 2.0347 |
| O | 4.0999 | -13.4806 | 2.0667 |
| C | 4.8725 | -13.6060 | 3.2851 |
| C | -1.1573 | -10.0968 | 0.7790 |
| O | 8.4159 | -6.4953 | -4.3841 |
| C | 7.8099 | -5.1958 | -4.6063 |
| O | 13.1570 | -6.9546 | -1.5720 |
| C | 13.7695 | -8.2564 | -1.4010 |
| O | 0.9700 | -5.6956 | 11.1316 |
| C | 2.2614 | -5.3249 | 10.5888 |
| O | -1.7827 | -2.3048 | 14.5241 |
| C | -3.1299 | -2.6305 | 14.9484 |
| O | -2.0580 | 5.6676 | -11.8471 |
| O | 3.4526 | 5.4066 | -11.4999 |
| C | -2.7314 | 6.7933 | -11.2304 |
| C | 4.1075 | 4.1256 | -11.3372 |
| O | -11.1726 | 6.3648 | 4.0582 |
| C | -10.9771 | 6.6772 | 5.4601 |
| O | -9.0508 | 9.6276 | 0.1227 |
| C | -9.1473 | 9.2509 | -1.2757 |
| O | -3.7225 | 9.5792 | 9.2099 |
| O | 1.7633 | 8.9053 | 9.4119 |
| C | 2.6112 | 9.5837 | 8.4500 |
| C | -4.5907 | 8.6921 | 9.9560 |
| O | -10.2735 | -5.5007 | 2.3691 |
| O | -8.2954 | -4.8446 | 7.4965 |
| C | -10.0666 | -6.7430 | 1.6518 |
| C | -8.3810 | -3.5464 | 8.1414 |
| O | 8.7859 | 9.1022 | -3.8618 |
| C | 8.4188 | 9.1513 | -2.4594 |
| O | 12.7730 | 5.6903 | -5.6157 |
| C | 13.1559 | 5.6816 | -7.0143 |
| O | 1.2666 | -5.9307 | -11.9305 |
| C | 2.6954 | -5.8625 | -12.1557 |
| O | -0.8345 | -10.1678 | -9.0506 |
| C | -2.2687 | -10.3690 | -9.0017 |
| C | -6.8213 | -11.6599 | 10.1371 |
| C | -4.3614 | -13.8186 | 7.3896 |
| C | -2.5251 | -12.2494 | 11.3010 |
| C | -4.7048 | -13.4824 | 10.1299 |
| H | -4.1321 | -11.4680 | 5.1459 |
| H | -5.6105 | -9.6619 | 4.3012 |
| H | -7.1388 | -9.0240 | 8.2939 |
| H | -0.3019 | -10.6505 | 9.9038 |
| H | 1.1380 | -10.8647 | 7.8761 |
| H | -2.1788 | -12.6181 | 5.7061 |
| H | -6.6577 | -8.6994 | 10.6067 |
| H | -5.3250 | -6.8459 | 11.6286 |
| H | -1.6896 | -9.1110 | 11.0223 |
| C | -16.2931 | -2.0993 | 1.3264 |
| C | -16.0822 | 2.1215 | 2.7082 |
| C | -16.6656 | 1.0057 | -1.6885 |
| C | -17.3788 | 0.4358 | 0.9361 |
| H | -12.9442 | 2.0285 | 3.5363 |
| H | -11.4721 | 0.3374 | 4.6024 |
| H | -13.5564 | -2.8191 | 2.4873 |
| H | -14.3293 | 2.7136 | -2.6155 |
| H | -12.9433 | 4.6945 | -1.9810 |
| H | -13.5532 | 3.7743 | 2.2100 |
| H | -13.5119 | -3.2233 | 0.2090 |
| H | -12.1813 | -3.3068 | -1.8834 |
| H | -13.7642 | 0.5404 | -3.0600 |
| C | -3.1309 | 17.5125 | 3.6544 |
| C | -7.4233 | 16.1641 | 2.6109 |
| C | -5.6759 | 16.0892 | 6.7500 |
| C | -5.8219 | 17.5662 | 4.3808 |
| H | -6.5285 | 14.0843 | 0.5261 |
| H | -4.7536 | 13.9188 | -1.2188 |
| H | -1.9657 | 15.9319 | 1.4209 |
| H | -6.4356 | 13.2055 | 7.1353 |
| H | -7.8017 | 11.2949 | 6.2659 |
| H | -7.5636 | 12.9324 | 2.2697 |
| H | -1.2396 | 14.8443 | 3.4188 |
| H | -0.3772 | 13.1315 | 5.0060 |
| H | -4.0780 | 13.3153 | 7.2307 |
| H | -7.7659 | 10.5628 | 1.8573 |
| H | -9.6451 | 8.6332 | 4.4642 |
| H | -10.6180 | 7.3801 | -0.2812 |
| H | -11.7018 | 4.9287 | 2.3796 |
| C | 4.0091 | 6.7582 | 15.6098 |
| C | 1.7217 | 3.1665 | 17.0734 |
| C | 6.1944 | 3.1244 | 16.3020 |
| C | 4.0683 | 4.5825 | 17.3434 |
| H | -0.0805 | 2.8656 | 14.4103 |
| H | -1.1659 | 4.4472 | 12.8382 |
| H | 2.1961 | 7.1542 | 13.2088 |
| H | 6.0246 | 0.6700 | 14.5724 |
| H | 4.6092 | -1.2904 | 13.9379 |
| H | 1.0432 | 0.9672 | 14.9134 |
| H | 4.3392 | 6.7215 | 12.4035 |
| H | 5.9154 | 5.8518 | 10.6991 |
| H | 6.7119 | 2.3966 | 13.1720 |
| H | 1.3672 | 7.0972 | 10.7094 |
| H | -2.3504 | 7.8015 | 10.8344 |
| H | 0.4006 | 10.7147 | 7.8344 |
| H | -1.0785 | -6.6448 | 10.6491 |
| H | -2.7916 | -4.5756 | 13.3129 |
| H | 1.9575 | -3.3983 | 12.2781 |
| H | 0.0305 | -0.9209 | 14.4193 |
| H | -3.3281 | 10.9366 | 7.3726 |
| H | -11.1294 | -3.4490 | 2.5475 |
| C | 8.5931 | 14.0862 | -9.8772 |
| C | 6.4894 | 11.7640 | -13.0359 |
| C | 4.3923 | 15.1138 | -10.9776 |
| C | 6.8383 | 14.3194 | -12.0152 |
| H | 6.7926 | 9.0768 | -11.5993 |
| H | 8.1675 | 7.8964 | -9.8789 |
| H | 8.7666 | 11.6289 | -7.7834 |
| H | 2.0981 | 13.3730 | -9.9497 |
| H | 0.8990 | 11.2229 | -10.3752 |
| H | 4.5571 | 9.4644 | -11.8626 |
| H | 7.9426 | 13.6249 | -6.9317 |
| H | 6.2528 | 14.0963 | -5.1504 |
| H | 3.1293 | 14.2049 | -8.1391 |
| H | 1.9675 | 15.0048 | -6.4977 |
| H | -1.9958 | 3.3645 | -11.9822 |
| H | -7.5946 | -1.8850 | -9.0438 |
| H | 1.7694 | 3.3206 | -12.1894 |
| H | -0.3619 | 7.7690 | -11.3403 |
| H | 3.3448 | 7.5872 | -10.7285 |
| C | 3.8175 | -15.1799 | -7.4557 |
| C | 8.1737 | -13.7127 | -7.5489 |
| C | 6.4001 | -16.0205 | -4.1368 |
| C | 6.4711 | -15.8090 | -6.9214 |
| H | 7.4515 | -10.7798 | -7.9836 |
| H | 5.6814 | -9.4213 | -9.1049 |
| H | 2.7300 | -12.4385 | -8.1189 |
| H | 7.1746 | -13.9033 | -2.1022 |
| H | 8.5672 | -11.8779 | -1.6744 |
| H | 8.5521 | -10.9539 | -5.9056 |
| H | 2.0255 | -12.8925 | -5.9910 |
| H | 1.2169 | -12.4404 | -3.6792 |
| H | 4.8959 | -14.0401 | -2.0683 |
| H | 1.3316 | -10.7253 | -8.5384 |
| H | 4.2032 | -13.0091 | -0.2338 |
| H | 2.7020 | -7.9400 | -10.7058 |
| H | -2.2747 | -8.1799 | -10.3137 |
| H | 0.8600 | -11.3246 | -0.1781 |
| H | 2.6076 | -12.7598 | 4.2834 |
| C | 15.4973 | 2.5563 | 2.8275 |
| C | 15.7401 | -1.6065 | 1.3766 |
| C | 16.0709 | 1.7589 | -1.5389 |
| C | 16.7975 | 0.9289 | 1.0023 |
| H | 12.6583 | -2.3370 | 2.0575 |
| H | 11.0671 | -1.7585 | 3.8726 |
| H | 12.6794 | 2.2597 | 4.0186 |
| H | 13.2988 | 1.0736 | -3.0732 |
| H | 12.1339 | -1.0143 | -3.7388 |
| H | 13.3828 | -2.8992 | -0.0424 |
| H | 12.6372 | 3.9612 | 2.4507 |
| H | 11.5662 | 5.4306 | 0.7624 |
| H | 13.5504 | 3.2468 | -2.4094 |
| H | 12.1903 | 1.2581 | 6.5758 |
| H | 13.3050 | 5.4359 | -3.3633 |
| H | 8.4464 | 0.7699 | 6.0079 |
| H | 9.8471 | 2.7922 | 10.3664 |
| H | 10.0670 | -4.4117 | -3.6233 |
| H | 11.4714 | -9.0252 | -2.2784 |
| H | 10.3134 | 7.4232 | -2.2794 |
| H | 11.1636 | 7.2767 | -7.2084 |
| H | 8.0148 | -8.6619 | -3.7737 |
| H | -0.8426 | -5.0647 | -11.9212 |
| H | 8.2197 | 9.3760 | -6.0426 |
| H | -9.1659 | 1.3639 | -7.9021 |
| H | -10.4993 | -2.7519 | -5.3892 |
| H | -1.7482 | 15.1054 | -3.5569 |
| H | 3.1584 | 14.3612 | -2.9550 |
| H | 6.2260 | 1.9807 | 10.0894 |
| H | -0.5534 | 14.4420 | -0.0102 |
| H | -0.4230 | -10.4720 | 4.2953 |
| H | -11.5180 | 0.5705 | -3.8797 |
| H | -9.9589 | -3.1006 | 6.1538 |
| H | -8.6144 | -7.2505 | 3.7076 |
| H | -6.9704 | -6.6871 | 7.0678 |
| C | -7.4102 | -2.5618 | -14.6511 |
| C | -5.3161 | 1.1451 | -15.6370 |
| C | -3.1626 | -2.7439 | -16.1771 |
| C | -5.6122 | -1.4974 | -16.4749 |
| H | -5.0588 | 2.1907 | -12.5867 |
| H | -6.4413 | 2.0734 | -10.5257 |
| H | -7.6254 | -1.9584 | -11.5321 |
| H | -1.0010 | -2.2124 | -13.8005 |
| H | 0.2877 | -0.2167 | -13.0732 |
| H | -3.1475 | 2.3013 | -13.8800 |
| H | -6.7731 | -4.0526 | -12.0313 |
| H | -5.1820 | -5.7575 | -11.1348 |
| H | -2.0760 | -4.2260 | -13.7384 |
| H | 13.4316 | -4.7282 | -1.8907 |
| H | -10.9594 | 3.7001 | -5.7327 |
| H | -9.3508 | 3.1278 | -6.3234 |
| H | -10.8182 | 2.9601 | -7.3751 |
| H | -8.6929 | -5.0866 | -7.5599 |
| H | -8.8150 | -4.3518 | -5.9143 |
| H | -10.2933 | -4.5085 | -6.9543 |
| H | 4.8514 | 0.7281 | 6.8138 |
| H | 6.1847 | 1.2245 | 5.6921 |
| H | 6.3202 | -0.3146 | 6.6522 |
| H | 13.4528 | 2.6337 | 9.6777 |
| H | 12.0542 | 3.7571 | 9.8361 |
| H | 12.0613 | 2.1781 | 10.7303 |
| H | 3.0764 | 14.0489 | 0.6622 |
| H | 3.4391 | 13.3409 | -0.9628 |
| H | 3.5647 | 15.1319 | -0.7067 |
| H | -1.5738 | 15.6967 | -7.1621 |
| H | -2.2497 | 14.7522 | -5.7771 |
| H | -1.7881 | 16.4906 | -5.5532 |
| H | 5.8062 | -14.0635 | 2.9159 |
| H | 5.0724 | -12.6220 | 3.7186 |
| H | 4.3570 | -14.2681 | 3.9860 |
| H | -2.0174 | -9.5046 | 1.1105 |
| H | -0.4983 | -9.5035 | 0.1336 |
| H | -1.4830 | -11.0124 | 0.2741 |
| H | 6.8714 | -5.4689 | -5.1055 |
| H | 7.6135 | -4.6945 | -3.6531 |
| H | 8.4448 | -4.5923 | -5.2621 |
| H | 14.7474 | -7.9870 | -0.9826 |
| H | 13.1805 | -8.8592 | -0.6910 |
| H | 13.8830 | -8.7598 | -2.3672 |
| H | 2.7415 | -6.3187 | 10.4532 |
| H | 2.0946 | -4.8132 | 9.6351 |
| H | 2.8491 | -4.7155 | 11.2821 |
| H | -3.3512 | -1.8048 | 15.6376 |
| H | -3.8119 | -2.6216 | 14.0924 |
| H | -3.1431 | -3.5928 | 15.4691 |
| H | -3.7964 | 6.5327 | -11.3848 |
| H | -2.4946 | 6.8397 | -10.1613 |
| H | -2.4808 | 7.7219 | -11.7487 |
| H | 5.0585 | 4.4128 | -10.8720 |
| H | 3.5395 | 3.4554 | -10.6839 |
| H | 4.2759 | 3.6743 | -12.3295 |
| H | -11.5226 | 5.8599 | 5.9501 |
| H | -9.9140 | 6.6443 | 5.7137 |
| H | -11.4277 | 7.6460 | 5.7011 |
| H | -8.6085 | 10.0712 | -1.7731 |
| H | -8.6484 | 8.2936 | -1.4470 |
| H | -10.1954 | 9.2315 | -1.5919 |
| H | 3.6019 | 9.1917 | 8.7093 |
| H | 2.3329 | 9.3009 | 7.4266 |
| H | 2.5641 | 10.6663 | 8.5992 |
| H | -5.5845 | 9.0421 | 9.6519 |
| H | -4.4267 | 7.6528 | 9.6530 |
| H | -4.4354 | 8.8290 | 11.0317 |
| H | -10.6261 | -6.5589 | 0.7253 |
| H | -9.0025 | -6.8895 | 1.4446 |
| H | -10.4958 | -7.5828 | 2.2074 |
| H | -7.7861 | -3.7029 | 9.0511 |
| H | -7.9320 | -2.7722 | 7.5124 |
| H | -9.4239 | -3.3196 | 8.3872 |
| H | 7.6121 | 9.8958 | -2.4652 |
| H | 8.0505 | 8.1765 | -2.1256 |
| H | 9.2674 | 9.4967 | -1.8588 |
| H | 14.0184 | 5.0018 | -7.0142 |
| H | 12.3419 | 5.2799 | -7.6256 |
| H | 13.4519 | 6.6843 | -7.3386 |
| H | 2.8069 | -4.8805 | -12.6516 |
| H | 3.2387 | -5.8759 | -11.2051 |
| H | 3.0110 | -6.6768 | -12.8145 |
| H | -2.3507 | -11.2481 | -8.3341 |
| H | -2.7678 | -9.5013 | -8.5614 |
| H | -2.6518 | -10.5964 | -10.0006 |
| H | 16.8074 | 0.9290 | -1.4831 |
| H | 16.5667 | 2.6474 | -1.0958 |
| C | 15.7919 | 2.0346 | -3.0218 |
| H | 6.4440 | -15.7194 | -8.0155 |
| H | 5.8160 | -16.6496 | -6.6511 |
| H | 7.4946 | -16.0952 | -6.6428 |
| H | 7.4509 | -16.2465 | -4.4154 |
| H | 5.7681 | -16.7553 | -4.6786 |
| C | 6.2230 | -16.2664 | -2.6330 |
| H | 7.7466 | -14.0715 | -8.5094 |
| H | 8.6789 | -14.5869 | -7.0886 |
| C | 9.2423 | -12.6497 | -7.8440 |
| H | 4.3231 | -15.3013 | -8.4348 |
| C | 2.3164 | -14.9500 | -7.6877 |
| H | 3.9364 | -16.1497 | -6.9279 |
| H | 16.3469 | -1.2018 | 2.2150 |
| H | 16.4330 | -1.7233 | 0.5177 |
| C | 15.2323 | -2.9915 | 1.7973 |
| H | 15.9861 | 1.7745 | 3.4457 |
| C | 14.9034 | 3.6221 | 3.7571 |
| H | 16.3046 | 3.0388 | 2.2363 |
| H | 17.0891 | 0.5380 | 1.9873 |
| H | 17.2027 | 1.9475 | 0.9267 |
| H | 17.3137 | 0.3177 | 0.2489 |
| H | -7.8263 | -1.7544 | -15.2894 |
| C | -8.5279 | -3.0426 | -13.7160 |
| H | -7.1372 | -3.3910 | -15.3376 |
| H | -6.5667 | -0.9686 | -16.6073 |
| H | -5.7770 | -2.5402 | -16.7766 |
| H | -4.9007 | -1.0634 | -17.1915 |
| H | -3.2207 | -1.9667 | -16.9688 |
| H | -3.8487 | -3.5594 | -16.4863 |
| C | -1.7275 | -3.2842 | -16.1603 |
| H | -6.3154 | 0.8544 | -16.0251 |
| H | -4.6171 | 1.0733 | -16.4963 |
| C | -5.3937 | 2.6087 | -15.1846 |
| H | 7.5755 | 11.9632 | -13.1449 |
| H | 5.9651 | 12.5775 | -13.5817 |
| C | 6.1545 | 10.4421 | -13.7385 |
| H | 7.8345 | 14.0341 | -12.3810 |
| H | 6.9189 | 15.3425 | -11.6222 |
| H | 6.1733 | 14.3605 | -12.8891 |
| H | 9.0260 | 13.8888 | -10.8813 |
| C | 9.7065 | 13.8823 | -8.8419 |
| H | 8.2896 | 15.1537 | -9.8714 |
| H | 4.1832 | 14.9355 | -12.0530 |
| H | 5.2094 | 15.8652 | -10.9364 |
| C | 3.1518 | 15.7410 | -10.3282 |
| H | -16.7642 | -1.8236 | 2.2931 |
| C | -15.8139 | -3.5540 | 1.4099 |
| H | -17.0973 | -2.0509 | 0.5615 |
| H | -17.6582 | 0.1660 | 1.9640 |
| H | -17.8928 | -0.2702 | 0.2680 |
| H | -17.7982 | 1.4309 | 0.7373 |
| H | -17.2447 | 1.8874 | -1.3443 |
| H | -17.3335 | 0.1261 | -1.5694 |
| C | -16.3622 | 1.1651 | -3.1837 |
| H | -16.7280 | 1.3496 | 3.1780 |
| H | -16.7559 | 2.7793 | 2.1205 |
| C | -15.4437 | 2.9466 | 3.8327 |
| H | -6.7428 | 16.3830 | 6.6684 |
| H | -5.0913 | 17.0341 | 6.7434 |
| C | -5.4505 | 15.4079 | 8.1056 |
| H | -3.6210 | 18.1656 | 2.9032 |
| C | -1.6149 | 17.5368 | 3.4199 |
| H | -3.3297 | 17.9717 | 4.6459 |
| H | -7.0502 | 17.0329 | 2.0297 |
| H | -7.9450 | 16.5810 | 3.4982 |
| C | -8.4619 | 15.4129 | 1.7671 |
| H | -5.7951 | 18.1532 | 3.4534 |
| H | -5.2217 | 18.1105 | 5.1241 |
| H | -6.8601 | 17.5716 | 4.7406 |
| H | -5.3645 | -14.1056 | 7.7699 |
| H | -3.6365 | -14.5143 | 7.8608 |
| C | -4.3511 | -14.0464 | 5.8725 |
| H | -5.6004 | -13.9537 | 9.7002 |
| H | -4.9041 | -13.3282 | 11.1994 |
| H | -3.8882 | -14.2122 | 10.0525 |
| H | -7.0456 | -12.6441 | 9.6731 |
| C | -8.0798 | -10.7897 | 10.0239 |
| H | -6.6188 | -11.8599 | 11.2099 |
| H | -2.1458 | -13.2329 | 10.9546 |
| H | -3.3754 | -12.4628 | 11.9825 |
| C | -1.4291 | -11.5573 | 12.1211 |
| H | 1.5850 | 4.1728 | 17.5234 |
| H | 2.3804 | 2.5986 | 17.7636 |
| C | 0.3508 | 2.4815 | 17.0338 |
| H | 3.2490 | 5.2047 | 17.7293 |
| H | 4.9941 | 5.1657 | 17.4530 |
| H | 4.1571 | 3.7125 | 18.0072 |
| H | 3.2048 | 6.9399 | 16.3524 |
| C | 4.1433 | 7.9975 | 14.7169 |
| H | 4.9517 | 6.6545 | 16.1877 |
| H | 5.8611 | 2.5532 | 17.1932 |
| H | 6.4588 | 4.1412 | 16.6624 |
| C | 7.4703 | 2.4787 | 15.7466 |
| H | 6.4972 | 9.5788 | -13.1285 |
| C | 6.7925 | 10.3730 | -15.1249 |
| H | 5.0546 | 10.3352 | -13.8352 |
| H | 7.8859 | 10.4183 | -15.0732 |
| H | 6.4599 | 11.1991 | -15.7641 |
| H | 6.5247 | 9.4406 | -15.6351 |
| H | 3.4082 | 16.1252 | -9.3207 |
| H | 2.3613 | 14.9737 | -10.1790 |
| C | 2.5979 | 16.8828 | -11.1791 |
| H | 3.3451 | 17.6686 | -11.3382 |
| H | 1.7317 | 17.3494 | -10.6965 |
| H | 2.2739 | 16.5329 | -12.1659 |
| H | 10.1704 | 12.8845 | -8.9791 |
| H | 9.2866 | 13.8943 | -7.8129 |
| C | 10.7819 | 14.9605 | -8.9638 |
| H | 11.2368 | 14.9680 | -9.9609 |
| H | 11.5882 | 14.7962 | -8.2397 |
| H | 10.3762 | 15.9618 | -8.7795 |
| H | 1.7773 | -15.0118 | -6.7192 |
| C | 1.7365 | -15.9661 | -8.6688 |
| H | 2.1370 | -13.9235 | -8.0769 |
| H | 1.8913 | -16.9956 | -8.3252 |
| H | 2.1961 | -15.8790 | -9.6607 |
| H | 0.6575 | -15.8219 | -8.7953 |
| H | -1.5621 | -3.9201 | -15.2635 |
| H | -1.0109 | -2.4416 | -16.0846 |
| C | -1.4234 | -4.0957 | -17.4184 |
| H | -2.0706 | -4.9770 | -17.4983 |
| H | -0.3871 | -4.4522 | -17.4156 |
| H | -1.5633 | -3.5016 | -18.3284 |
| C | 9.8728 | -12.8640 | -9.2181 |
| H | 10.0198 | -12.6690 | -7.0544 |
| H | 8.8154 | -11.6203 | -7.8048 |
| H | 10.2915 | -13.8698 | -9.3290 |
| H | 10.6804 | -12.1459 | -9.4048 |
| H | 9.1372 | -12.7262 | -10.0204 |
| H | 15.5235 | 1.0902 | -3.5362 |
| C | 17.0056 | 2.6606 | -3.7072 |
| H | 14.9157 | 2.7099 | -3.1346 |
| H | 17.8908 | 2.0209 | -3.6273 |
| H | 17.2621 | 3.6320 | -3.2667 |
| H | 16.8129 | 2.8297 | -4.7723 |
| C | 15.8931 | 4.0176 | 4.8517 |
| H | 13.9649 | 3.2536 | 4.2253 |
| H | 14.6253 | 4.5204 | 3.1706 |
| H | 16.1495 | 3.1666 | 5.4936 |
| H | 15.4736 | 4.7969 | 5.4985 |
| H | 16.8276 | 4.4067 | 4.4330 |
| C | 8.4886 | 2.2186 | 16.8552 |
| H | 7.9226 | 3.1372 | 14.9776 |
| H | 7.2280 | 1.5218 | 15.2341 |
| H | 8.7695 | 3.1432 | 17.3720 |
| H | 9.4072 | 1.7791 | 16.4500 |
| H | 8.0994 | 1.5251 | 17.6093 |
| H | 5.0537 | 7.9146 | 14.0907 |
| C | 4.2157 | 9.2755 | 15.5503 |
| H | 3.2841 | 8.0641 | 14.0134 |
| H | 5.0546 | 9.2538 | 16.2548 |
| H | 3.3002 | 9.4315 | 16.1325 |
| H | 4.3488 | 10.1552 | 14.9102 |
| H | -1.0949 | 17.0358 | 4.2609 |
| C | -1.0974 | 18.9673 | 3.2809 |
| H | -1.3575 | 16.9598 | 2.5044 |
| H | -1.3162 | 19.5659 | 4.1723 |
| H | -1.5474 | 19.4796 | 2.4226 |
| H | -0.0113 | 18.9812 | 3.1362 |
| H | -9.0232 | 14.7033 | 2.4074 |
| H | -7.9589 | 14.8028 | 0.9862 |
| C | -9.4427 | 16.3777 | 1.1034 |
| H | -9.9599 | 17.0018 | 1.8410 |
| H | -10.2117 | 15.8355 | 0.5405 |
| H | -8.9376 | 17.0504 | 0.4005 |
| H | -14.9619 | 2.2716 | 4.5680 |
| C | -16.4822 | 3.8149 | 4.5416 |
| H | -14.6365 | 3.5958 | 3.4285 |
| H | -17.2863 | 3.2112 | 4.9761 |
| H | -16.9453 | 4.5321 | 3.8523 |
| H | -16.0244 | 4.3937 | 5.3508 |
| H | -15.9602 | 0.2150 | -3.5906 |
| H | -15.5711 | 1.9304 | -3.3390 |
| C | -17.6147 | 1.5606 | -3.9641 |
| H | -18.4151 | 0.8210 | -3.8453 |
| H | -17.4012 | 1.6391 | -5.0360 |
| H | -18.0096 | 2.5280 | -3.6344 |
| H | -4.4754 | 2.8877 | -14.6222 |
| H | -6.2482 | 2.7440 | -14.4804 |
| C | -5.5679 | 3.5506 | -16.3738 |
| H | -4.7148 | 3.4967 | -17.0609 |
| H | -5.6523 | 4.5928 | -16.0445 |
| H | -6.4689 | 3.3162 | -16.9519 |
| C | 6.8286 | -17.6052 | -2.2150 |
| H | 5.1451 | -16.2507 | -2.3740 |
| H | 6.6987 | -15.4468 | -2.0502 |
| H | 6.4014 | -18.4381 | -2.7853 |
| H | 6.6452 | -17.8093 | -1.1548 |
| H | 7.9140 | -17.6243 | -2.3727 |
| H | -8.1075 | -3.6282 | -12.8701 |
| C | -9.5496 | -3.8967 | -14.4640 |
| H | -9.0380 | -2.1697 | -13.2598 |
| H | -9.0945 | -4.7984 | -14.8892 |
| H | -10.0087 | -3.3433 | -15.2928 |
| H | -10.3597 | -4.2172 | -13.7978 |
| H | -14.9045 | -3.6271 | 2.0460 |
| H | -15.5209 | -3.9129 | 0.4031 |
| C | -16.9017 | -4.4645 | 1.9767 |
| H | -17.1769 | -4.1797 | 2.9991 |
| H | -16.5647 | -5.5069 | 2.0107 |
| H | -17.8139 | -4.4342 | 1.3705 |
| H | -4.3641 | 15.2763 | 8.2851 |
| H | -5.8920 | 14.3872 | 8.1033 |
| C | -6.0588 | 16.2231 | 9.2452 |
| H | -5.6337 | 17.2323 | 9.2927 |
| H | -5.8715 | 15.7459 | 10.2140 |
| H | -7.1440 | 16.3289 | 9.1358 |
| C | -0.1642 | 2.1921 | 18.4434 |
| H | 0.4090 | 1.5319 | 16.4625 |
| H | -0.3784 | 3.1263 | 16.5040 |
| H | 0.5009 | 1.5093 | 18.9850 |
| H | -1.1553 | 1.7261 | 18.4128 |
| H | -0.2505 | 3.1077 | 19.0392 |
| C | 16.3796 | -3.9953 | 1.8988 |
| H | 14.4760 | -3.3655 | 1.0733 |
| H | 14.7174 | -2.9220 | 2.7766 |
| H | 16.8792 | -4.1396 | 0.9341 |
| H | 16.0173 | -4.9746 | 2.2309 |
| H | 17.1408 | -3.6680 | 2.6164 |
| C | -4.4574 | -15.5321 | 5.5332 |
| H | -3.4196 | -13.6317 | 5.4269 |
| H | -5.1933 | -13.5006 | 5.4027 |
| H | -3.6078 | -16.0997 | 5.9303 |
| H | -4.4750 | -15.6875 | 4.4485 |
| H | -5.3713 | -15.9771 | 5.9424 |
| H | -7.8577 | -9.7498 | 10.3501 |
| C | -9.2248 | -11.3516 | 10.8645 |
| H | -8.4001 | -10.7264 | 8.9643 |
| H | -8.9652 | -11.3941 | 11.9285 |
| H | -9.4965 | -12.3664 | 10.5517 |
| H | -10.1231 | -10.7300 | 10.7721 |
| C | -0.7672 | -12.5282 | 13.0975 |
| H | -1.8625 | -10.7069 | 12.6861 |
| H | -0.6557 | -11.1233 | 11.4493 |
| H | -1.4963 | -12.9653 | 13.7892 |
| H | -0.0076 | -12.0205 | 13.7028 |
| H | -0.2731 | -13.3549 | 12.5748 |
| H | -22.3861 | 8.4616 | -1.7891 |
| C | -22.8728 | 7.5718 | -1.3693 |
| H | -22.4310 | 6.6552 | -1.7710 |
| H | -23.9556 | 7.6058 | -1.5249 |
| O | -22.5625 | 7.7060 | 0.0389 |
| C | -23.0316 | 6.7264 | 0.8947 |
| C | -23.8066 | 5.6291 | 0.5060 |
| H | -24.1037 | 5.4794 | -0.5346 |
| C | -24.2250 | 4.6963 | 1.4617 |
| C | -25.0150 | 3.5012 | 1.0978 |
| H | -25.0220 | 2.7114 | 1.8731 |
| N | -25.6127 | 3.4553 | -0.0524 |
| C | -26.3901 | 2.3266 | -0.4600 |
| C | -27.1808 | 1.5666 | 0.4284 |
| H | -27.1714 | 1.7904 | 1.4923 |
| C | -27.9579 | 0.5295 | -0.0750 |
| C | -27.9290 | 0.2138 | -1.4503 |
| C | -27.1359 | 0.9586 | -2.3208 |
| H | -27.0828 | 0.7090 | -3.3790 |
| C | -28.7373 | -1.0294 | -1.7779 |
| C | -27.7574 | -2.1268 | -2.1613 |
| C | -27.6027 | -3.0878 | -1.1394 |
| C | -26.6199 | -4.0692 | -1.2472 |
| H | -26.4518 | -4.7808 | -0.4407 |
| C | -25.8244 | -4.1343 | -2.3955 |
| H | -25.0575 | -4.9019 | -2.4915 |
| C | -26.0085 | -3.2014 | -3.4315 |
| N | -25.1805 | -3.3721 | -4.5848 |
| C | -24.7307 | -2.3607 | -5.2610 |
| H | -24.9298 | -1.2923 | -5.0523 |
| C | -23.8438 | -2.5931 | -6.4202 |
| C | -23.2183 | -3.8260 | -6.6390 |
| H | -23.4351 | -4.6501 | -5.9550 |
| C | -22.3394 | -3.9813 | -7.7154 |
| O | -21.6674 | -5.1494 | -8.0251 |
| C | -21.8503 | -6.2830 | -7.1424 |
| H | -21.4955 | -6.0432 | -6.1357 |
| H | -21.2109 | -7.0386 | -7.6168 |
| H | -22.8981 | -6.5990 | -7.1420 |
| C | -22.0595 | -2.9137 | -8.6060 |
| C | -22.7063 | -1.6895 | -8.4039 |
| H | -22.5080 | -0.8735 | -9.1027 |
| C | -21.0828 | -3.1224 | -9.6943 |
| H | -20.4697 | -4.0375 | -9.5763 |
| N | -20.9935 | -2.2656 | -10.6646 |
| C | -20.0526 | -2.4477 | -11.7277 |
| C | -19.7701 | -3.7118 | -12.2888 |
| H | -20.2667 | -4.6118 | -11.9165 |
| C | -18.8706 | -3.7968 | -13.3432 |
| C | -18.2638 | -2.6339 | -13.8655 |
| C | -18.5571 | -1.3879 | -13.3181 |
| H | -18.1000 | -0.4851 | -13.7344 |
| C | -17.3367 | -2.9352 | -15.0231 |
| C | -15.8764 | -2.7350 | -14.6641 |
| C | -15.1294 | -3.9266 | -14.7926 |
| C | -13.7486 | -3.9137 | -14.6182 |
| H | -13.1682 | -4.8296 | -14.7656 |
| C | -13.1056 | -2.7275 | -14.2535 |
| H | -12.0248 | -2.6997 | -14.1215 |
| C | -13.8636 | -1.5642 | -14.0360 |
| N | -13.1441 | -0.3773 | -13.6955 |
| C | -13.4872 | 0.3717 | -12.6956 |
| H | -14.3356 | 0.1976 | -12.0061 |
| C | -12.7037 | 1.5906 | -12.4004 |
| C | -11.4189 | 1.7883 | -12.9135 |
| H | -10.9865 | 1.0201 | -13.5597 |
| C | -10.7137 | 2.9587 | -12.6047 |
| O | -9.4492 | 3.2566 | -13.0768 |
| C | -8.7018 | 2.1768 | -13.6923 |
| H | -8.5650 | 1.3549 | -12.9787 |
| H | -7.7322 | 2.6597 | -13.9153 |
| H | -9.1945 | 1.8489 | -14.6108 |
| C | -11.2770 | 3.9636 | -11.7797 |
| C | -10.5062 | 5.1939 | -11.5132 |
| H | -9.6681 | 5.3566 | -12.2189 |
| N | -10.8377 | 5.9737 | -10.5293 |
| C | -10.1031 | 7.1712 | -10.2559 |
| C | -8.7034 | 7.2614 | -10.4165 |
| H | -8.1332 | 6.4077 | -10.7889 |
| C | -8.0597 | 8.4516 | -10.1077 |
| C | -8.7898 | 9.5496 | -9.6036 |
| C | -10.1689 | 9.4549 | -9.4379 |
| H | -10.7361 | 10.3130 | -9.0638 |
| C | -7.8973 | 10.7272 | -9.2645 |
| C | -7.7072 | 10.7924 | -7.7591 |
| C | -6.3686 | 10.5661 | -7.3718 |
| C | -6.0348 | 10.5203 | -6.0201 |
| H | -5.0147 | 10.3050 | -5.7054 |
| C | -7.0206 | 10.7296 | -5.0476 |
| H | -6.7601 | 10.7116 | -3.9897 |
| C | -8.3534 | 10.9512 | -5.4331 |
| N | -9.2954 | 11.1841 | -4.3782 |
| C | -10.5742 | 11.1106 | -4.6028 |
| H | -11.0389 | 10.8362 | -5.5732 |
| C | -11.5568 | 11.4007 | -3.5430 |
| C | -11.1920 | 11.8328 | -2.2618 |
| H | -10.1326 | 11.9737 | -2.0354 |
| C | -12.1782 | 12.0866 | -1.3023 |
| O | -11.9375 | 12.5285 | -0.0181 |
| C | -10.5718 | 12.4757 | 0.4666 |
| H | -10.1582 | 11.4623 | 0.3572 |
| H | -10.7034 | 12.7281 | 1.5272 |
| H | -9.9659 | 13.2253 | -0.0485 |
| C | -13.5574 | 11.9298 | -1.5982 |
| C | -14.5603 | 12.1714 | -0.5398 |
| H | -14.1437 | 12.1566 | 0.4868 |
| N | -15.8023 | 12.3604 | -0.8629 |
| C | -16.8053 | 12.5723 | 0.1356 |
| C | -16.6103 | 13.4331 | 1.2361 |
| H | -15.6550 | 13.9348 | 1.3698 |
| C | -17.6431 | 13.6030 | 2.1510 |
| C | -18.8644 | 12.9165 | 1.9881 |
| C | -19.0522 | 12.0690 | 0.8979 |
| H | -19.9789 | 11.5100 | 0.7805 |
| C | -19.8154 | 13.1477 | 3.1467 |
| C | -20.0572 | 11.8317 | 3.8639 |
| C | -19.3465 | 11.7437 | 5.0809 |
| C | -19.4131 | 10.5825 | 5.8459 |
| H | -18.8767 | 10.5220 | 6.7976 |
| C | -20.1809 | 9.4991 | 5.4059 |
| H | -20.2382 | 8.5872 | 5.9980 |
| C | -20.8851 | 9.5879 | 4.1924 |
| N | -21.6891 | 8.4609 | 3.8302 |
| C | -21.8103 | 8.0904 | 2.5929 |
| H | -21.3234 | 8.5668 | 1.7195 |
| C | -18.5819 | 13.0109 | 5.3972 |
| C | -18.9703 | 13.5815 | 6.7813 |
| C | -18.4434 | 12.8229 | 8.0062 |
| C | -19.1164 | 13.3063 | 9.2897 |
| H | -20.1977 | 13.1291 | 9.2725 |
| H | -18.7127 | 12.7851 | 10.1652 |
| H | -18.9609 | 14.3793 | 9.4488 |
| H | -18.6107 | 11.7305 | 7.8893 |
| H | -17.3461 | 12.9603 | 8.0917 |
| H | -20.0774 | 13.6276 | 6.8363 |
| H | -18.6082 | 14.6292 | 6.8503 |
| C | -17.0800 | 12.8329 | 5.2761 |
| C | -16.2698 | 11.9496 | 5.9789 |
| H | -16.6932 | 11.2292 | 6.6739 |
| C | -14.8730 | 12.0018 | 5.7782 |
| C | -14.3054 | 12.9363 | 4.8960 |
| H | -13.2232 | 12.9898 | 4.7840 |
| C | -15.1390 | 13.7750 | 4.1503 |
| H | -14.7070 | 14.4869 | 3.4399 |
| N | -13.9736 | 11.1822 | 6.5288 |
| C | -14.1063 | 9.8957 | 6.5858 |
| H | -14.8803 | 9.2885 | 6.0791 |
| C | -13.1562 | 9.1124 | 7.4060 |
| C | -11.8866 | 9.5967 | 7.7323 |
| C | -11.0386 | 8.8390 | 8.5492 |
| O | -9.7603 | 9.2129 | 8.9188 |
| C | -9.2879 | 10.5114 | 8.4793 |
| H | -8.3041 | 10.5648 | 8.9614 |
| H | -9.9477 | 11.3050 | 8.8436 |
| H | -9.1937 | 10.5316 | 7.3859 |
| C | -11.4418 | 7.5859 | 9.0731 |
| C | -12.6999 | 7.0842 | 8.7139 |
| H | -13.0021 | 6.1081 | 9.1008 |
| C | -10.5604 | 6.8743 | 10.0213 |
| H | -9.7607 | 7.5081 | 10.4496 |
| N | -10.7881 | 5.6288 | 10.3048 |
| C | -10.0020 | 4.9235 | 11.2701 |
| C | -8.6266 | 5.1658 | 11.4813 |
| H | -8.1036 | 5.9011 | 10.8754 |
| C | -7.9527 | 4.4329 | 12.4504 |
| C | -8.6125 | 3.3981 | 13.1492 |
| C | -9.9662 | 3.1570 | 12.9326 |
| H | -10.4801 | 2.3710 | 13.4947 |
| C | -7.6682 | 2.6023 | 14.0298 |
| C | -8.1754 | 2.4258 | 15.4731 |
| H | -7.3369 | 2.0588 | 16.1027 |
| H | -8.4461 | 3.4275 | 15.8669 |
| C | -9.3639 | 1.4780 | 15.6769 |
| H | -10.1699 | 1.7037 | 14.9449 |
| H | -9.0508 | 0.4322 | 15.4821 |
| C | -9.9213 | 1.5839 | 17.0952 |
| H | -10.3015 | 2.5893 | 17.3087 |
| H | -10.7497 | 0.8818 | 17.2454 |
| H | -9.1594 | 1.3537 | 17.8486 |
| C | -7.3629 | 1.2890 | 13.3250 |
| C | -6.0189 | 1.2060 | 12.9022 |
| C | -5.5888 | 0.1178 | 12.1463 |
| H | -4.5668 | 0.0680 | 11.7727 |
| C | -6.4808 | -0.9152 | 11.8381 |
| H | -6.1471 | -1.7756 | 11.2595 |
| C | -7.8179 | -0.8359 | 12.2624 |
| N | -8.6563 | -1.9547 | 11.9519 |
| C | -9.9391 | -1.8140 | 11.8081 |
| H | -10.4974 | -0.8577 | 11.8682 |
| C | -10.8010 | -2.9852 | 11.5526 |
| C | -10.3047 | -4.2111 | 11.0948 |
| H | -9.2292 | -4.3205 | 10.9439 |
| C | -11.1824 | -5.2764 | 10.8626 |
| O | -10.8075 | -6.5298 | 10.4233 |
| C | -9.4596 | -6.6917 | 9.9153 |
| H | -8.7320 | -6.5388 | 10.7163 |
| H | -9.4748 | -7.7386 | 9.5855 |
| H | -9.2845 | -6.0178 | 9.0644 |
| C | -12.5758 | -5.1541 | 11.0994 |
| C | -13.0694 | -3.9341 | 11.5729 |
| H | -14.1399 | -3.8447 | 11.7728 |
| C | -13.4657 | -6.3030 | 10.8262 |
| H | -13.0125 | -7.0863 | 10.1880 |
| N | -14.6639 | -6.3119 | 11.3209 |
| C | -15.5701 | -7.3921 | 11.0836 |
| C | -15.1933 | -8.7436 | 11.2295 |
| H | -14.1623 | -8.9965 | 11.4640 |
| C | -16.1524 | -9.7341 | 11.0490 |
| C | -17.4760 | -9.3938 | 10.7004 |
| C | -17.8386 | -8.0574 | 10.5427 |
| H | -18.8472 | -7.7862 | 10.2364 |
| C | -18.3398 | -10.6140 | 10.4441 |
| C | -18.7588 | -10.6237 | 8.9849 |
| C | -18.0292 | -11.5623 | 8.2234 |
| C | -18.2378 | -11.6609 | 6.8503 |
| H | -17.6822 | -12.3968 | 6.2622 |
| C | -19.1699 | -10.8271 | 6.2249 |
| H | -19.3365 | -10.8942 | 5.1504 |
| C | -19.8974 | -9.8934 | 6.9851 |
| N | -20.8493 | -9.1066 | 6.2640 |
| C | -21.1724 | -7.9069 | 6.6376 |
| H | -20.7817 | -7.3663 | 7.5204 |
| C | -22.1370 | -7.1266 | 5.8345 |
| C | -22.4540 | -7.4723 | 4.5162 |
| H | -22.0067 | -8.3739 | 4.0910 |
| C | -23.3273 | -6.6694 | 3.7755 |
| O | -23.7058 | -6.9110 | 2.4675 |
| C | -23.1291 | -8.0623 | 1.8042 |
| H | -23.4220 | -8.9830 | 2.3184 |
| H | -22.0419 | -7.9596 | 1.7389 |
| H | -23.5922 | -8.0011 | 0.8111 |
| C | -23.9122 | -5.5017 | 4.3277 |
| C | -23.6179 | -5.1716 | 5.6556 |
| H | -24.0863 | -4.2850 | 6.0886 |
| C | -24.7810 | -4.6636 | 3.4774 |
| N | -25.5537 | -3.7660 | 4.0084 |
| C | -26.3879 | -2.9409 | 3.1878 |
| C | -27.0401 | -3.4233 | 2.0327 |
| H | -26.9424 | -4.4697 | 1.7328 |
| C | -27.8322 | -2.5565 | 1.2912 |
| C | -27.9978 | -1.2138 | 1.6937 |
| C | -27.3861 | -0.7538 | 2.8569 |
| H | -27.5416 | 0.2780 | 3.1863 |
| C | -26.5786 | -1.6133 | 3.6090 |
| H | -26.0908 | -1.2598 | 4.5162 |
| H | -24.6841 | -4.8778 | 2.3944 |
| C | -22.7412 | -5.9726 | 6.3949 |
| O | -22.3718 | -5.7397 | 7.7070 |
| C | -22.9251 | -4.5726 | 8.3622 |
| H | -22.4746 | -4.6444 | 9.3605 |
| H | -24.0155 | -4.6484 | 8.4171 |
| H | -22.6046 | -3.6607 | 7.8501 |
| C | -17.0769 | -12.3798 | 9.0691 |
| C | -17.3513 | -13.8959 | 8.9309 |
| H | -16.8230 | -14.4300 | 9.7486 |
| H | -18.4357 | -14.0649 | 9.0948 |
| C | -16.9421 | -14.5497 | 7.6048 |
| H | -17.2814 | -13.9323 | 6.7453 |
| H | -15.8371 | -14.5993 | 7.5344 |
| C | -17.5214 | -15.9580 | 7.4791 |
| H | -18.6175 | -15.9462 | 7.4781 |
| H | -17.1986 | -16.4349 | 6.5470 |
| H | -17.2006 | -16.6031 | 8.3048 |
| C | -15.6217 | -12.0530 | 8.7922 |
| C | -14.9599 | -11.5603 | 9.9383 |
| C | -13.5881 | -11.3241 | 9.9114 |
| H | -13.0718 | -10.9871 | 10.8158 |
| C | -12.8744 | -11.5074 | 8.7238 |
| H | -11.8022 | -11.3166 | 8.6869 |
| C | -13.5517 | -11.9117 | 7.5590 |
| N | -12.7904 | -11.9272 | 6.3513 |
| C | -12.9388 | -12.8388 | 5.4408 |
| H | -13.6219 | -13.7085 | 5.4877 |
| C | -12.1302 | -12.7620 | 4.2056 |
| C | -11.0959 | -11.8344 | 4.0508 |
| H | -10.8895 | -11.1353 | 4.8677 |
| C | -10.3477 | -11.8064 | 2.8668 |
| O | -9.3251 | -10.9145 | 2.6135 |
| C | -8.8085 | -10.1495 | 3.7353 |
| H | -8.4308 | -10.8162 | 4.5169 |
| H | -7.9840 | -9.5931 | 3.2727 |
| H | -9.5732 | -9.4705 | 4.1232 |
| C | -10.6126 | -12.7050 | 1.8035 |
| C | -11.6588 | -13.6256 | 1.9527 |
| H | -11.8679 | -14.3135 | 1.1298 |
| C | -9.7690 | -12.6650 | 0.5919 |
| H | -8.9558 | -11.9139 | 0.6306 |
| N | -10.0084 | -13.4751 | -0.3929 |
| C | -9.1634 | -13.5269 | -1.5448 |
| C | -7.7603 | -13.6227 | -1.4254 |
| H | -7.2833 | -13.6020 | -0.4423 |
| C | -6.9955 | -13.7891 | -2.5719 |
| C | -7.6074 | -13.8552 | -3.8425 |
| C | -8.9919 | -13.7584 | -3.9543 |
| H | -9.4683 | -13.8266 | -4.9370 |
| C | -6.5985 | -14.0273 | -4.9611 |
| C | -6.4293 | -12.7277 | -5.7271 |
| C | -5.1234 | -12.2023 | -5.6165 |
| C | -4.8067 | -10.9865 | -6.2189 |
| H | -3.8182 | -10.5467 | -6.0981 |
| C | -5.7745 | -10.3003 | -6.9608 |
| H | -5.5316 | -9.3562 | -7.4458 |
| C | -7.0764 | -10.8191 | -7.0584 |
| N | -8.0083 | -10.1003 | -7.8718 |
| C | -9.2479 | -9.9646 | -7.5142 |
| H | -9.6958 | -10.3402 | -6.5727 |
| C | -10.2038 | -9.2589 | -8.3918 |
| C | -9.7946 | -8.4763 | -9.4785 |
| H | -8.7262 | -8.3998 | -9.6934 |
| C | -10.7483 | -7.8252 | -10.2681 |
| O | -10.4627 | -7.0225 | -11.3565 |
| C | -9.0695 | -6.8243 | -11.7008 |
| H | -8.6058 | -7.7773 | -11.9741 |
| H | -8.5392 | -6.3329 | -10.8804 |
| H | -9.1486 | -6.1608 | -12.5731 |
| C | -12.1367 | -7.9456 | -10.0041 |
| C | -12.5459 | -8.7355 | -8.9250 |
| H | -13.6158 | -8.8423 | -8.7310 |
| C | -13.1084 | -7.2271 | -10.8566 |
| N | -14.3463 | -7.6128 | -10.8723 |
| C | -15.3365 | -6.9559 | -11.6673 |
| C | -15.0754 | -6.4343 | -12.9520 |
| H | -14.0678 | -6.4710 | -13.3584 |
| C | -16.1160 | -5.8596 | -13.6731 |
| C | -17.4131 | -5.7873 | -13.1230 |
| C | -17.6624 | -6.2870 | -11.8463 |
| H | -18.6495 | -6.1972 | -11.3961 |
| C | -16.6298 | -6.8884 | -11.1200 |
| H | -16.8171 | -7.2917 | -10.1257 |
| H | -12.6736 | -6.3839 | -11.4255 |
| C | -11.5915 | -9.3773 | -8.1285 |
| O | -11.8814 | -10.1769 | -7.0387 |
| C | -13.2727 | -10.3231 | -6.6631 |
| H | -13.1970 | -10.9781 | -5.7854 |
| H | -13.8343 | -10.8068 | -7.4682 |
| H | -13.6990 | -9.3524 | -6.3933 |
| C | -4.2385 | -13.0391 | -4.7065 |
| C | -2.9640 | -13.5723 | -5.3828 |
| H | -2.3631 | -14.1037 | -4.6141 |
| H | -3.2526 | -14.3363 | -6.1360 |
| C | -2.0673 | -12.5407 | -6.0751 |
| H | -2.5909 | -12.1067 | -6.9583 |
| H | -1.8545 | -11.6904 | -5.3899 |
| C | -0.7514 | -13.1634 | -6.5358 |
| H | -0.9141 | -14.0103 | -7.2123 |
| H | -0.1406 | -12.4280 | -7.0738 |
| H | -0.1620 | -13.5332 | -5.6871 |
| C | -3.9760 | -12.2096 | -3.4601 |
| C | -4.7848 | -12.6140 | -2.3751 |
| C | -4.8602 | -11.8260 | -1.2304 |
| H | -5.5461 | -12.0807 | -0.4239 |
| C | -4.0516 | -10.6894 | -1.1153 |
| H | -4.1073 | -10.0679 | -0.2244 |
| C | -3.1654 | -10.3529 | -2.1521 |
| N | -2.2380 | -9.2916 | -1.9076 |
| C | -2.0121 | -8.3626 | -2.7854 |
| H | -2.5234 | -8.2583 | -3.7630 |
| C | -1.0184 | -7.3082 | -2.5066 |
| C | -0.1058 | -7.4051 | -1.4445 |
| H | -0.1278 | -8.2966 | -0.8127 |
| C | 0.8075 | -6.3741 | -1.2206 |
| O | 1.7742 | -6.3552 | -0.2328 |
| C | 1.8282 | -7.4746 | 0.6841 |
| H | 1.9692 | -8.4159 | 0.1458 |
| H | 2.7165 | -7.2284 | 1.2815 |
| H | -0.4881 | -7.3330 | 1.9066 |
| C | 0.8227 | -5.2086 | -2.0337 |
| C | -0.0709 | -5.1151 | -3.0984 |
| H | -0.0666 | -4.2180 | -3.7252 |
| C | 1.7597 | -4.1188 | -1.6811 |
| H | 1.8333 | -3.9430 | -0.5896 |
| N | 2.4010 | -3.4797 | -2.6082 |
| C | 3.3162 | -2.4251 | -2.2787 |
| C | 4.1229 | -2.4464 | -1.1194 |
| H | 4.0522 | -3.2775 | -0.4211 |
| C | 4.9925 | -1.3889 | -0.8799 |
| C | 5.0272 | -0.2856 | -1.7599 |
| C | 4.2698 | -0.2943 | -2.9280 |
| H | 4.3422 | 0.5390 | -3.6346 |
| C | 5.8727 | 0.8532 | -1.2253 |
| C | 4.9516 | 1.9651 | -0.7496 |
| C | 5.0294 | 2.1704 | 0.6445 |
| C | 4.2758 | 3.1744 | 1.2478 |
| H | 4.3804 | 3.3699 | 2.3192 |
| C | 3.3849 | 3.9356 | 0.4839 |
| H | 2.7841 | 4.7161 | 0.9521 |
| C | 3.2466 | 3.6725 | -0.8891 |
| N | 2.2929 | 4.4686 | -1.6074 |
| C | 1.6500 | 3.9780 | -2.6207 |
| H | 1.7557 | 2.9515 | -3.0266 |
| C | 0.6697 | 4.7957 | -3.3669 |
| C | -0.1993 | 5.6926 | -2.7458 |
| H | -0.1267 | 5.8445 | -1.6609 |
| C | -1.1691 | 6.3602 | -3.5108 |
| O | -2.0519 | 7.2981 | -3.0277 |
| C | -2.0589 | 7.5632 | -1.6007 |
| H | -1.1023 | 7.9879 | -1.2887 |
| H | -2.3008 | 6.6470 | -1.0439 |
| H | -2.8676 | 8.2988 | -1.5170 |
| C | -1.3167 | 6.1121 | -4.8996 |
| C | -0.4086 | 5.2478 | -5.5275 |
| H | -0.5009 | 5.0810 | -6.6026 |
| C | -2.4334 | 6.7430 | -5.6306 |
| N | -2.6910 | 6.3921 | -6.8537 |
| C | -3.7642 | 6.9740 | -7.5958 |
| C | -3.9929 | 8.3662 | -7.6304 |
| H | -3.3992 | 9.0436 | -7.0101 |
| C | -4.9730 | 8.8675 | -8.4765 |
| C | -5.6956 | 8.0067 | -9.3328 |
| C | -5.5231 | 6.6281 | -9.2363 |
| H | -6.1425 | 5.9480 | -9.8192 |
| C | -4.5520 | 6.1069 | -8.3735 |
| H | -4.4000 | 5.0308 | -8.3033 |
| H | -3.0075 | 7.4796 | -5.0344 |
| C | 0.5765 | 4.6102 | -4.7720 |
| O | 1.5231 | 3.7338 | -5.2691 |
| C | 1.6315 | 3.6067 | -6.7069 |
| H | 2.4732 | 2.9076 | -6.8062 |
| H | 1.8636 | 4.5742 | -7.1608 |
| H | -0.5790 | 2.5523 | -7.7059 |
| C | 5.9157 | 1.1486 | 1.3287 |
| C | 7.0177 | 1.7855 | 2.1970 |
| H | 7.7510 | 0.9985 | 2.4814 |
| H | 7.5761 | 2.5086 | 1.5648 |
| C | 6.5737 | 2.4966 | 3.4800 |
| H | 5.7342 | 3.1933 | 3.2698 |
| H | 6.1881 | 1.7543 | 4.2184 |
| C | 7.7342 | 3.2646 | 4.1102 |
| H | 8.0937 | 4.0650 | 3.4535 |
| H | 7.4385 | 3.7266 | 5.0584 |
| H | 8.5902 | 2.6076 | 4.3182 |
| C | 5.0438 | 0.1718 | 2.0998 |
| C | 5.1123 | -1.1396 | 1.5799 |
| C | 4.4093 | -2.1748 | 2.1898 |
| H | 4.5127 | -3.2002 | 1.8200 |
| C | 3.5651 | -1.9035 | 3.2723 |
| H | 2.9982 | -2.7058 | 3.7445 |
| C | 3.4259 | -0.5824 | 3.7251 |
| N | 2.5045 | -0.3532 | 4.8026 |
| C | 1.7442 | 0.6957 | 4.7869 |
| C | 0.7823 | 0.9541 | 5.8802 |
| C | -0.1324 | -0.0060 | 6.3133 |
| H | -0.1107 | -1.0100 | 5.8694 |
| C | -1.0814 | 0.3370 | 7.2875 |
| O | -2.0080 | -0.5211 | 7.8318 |
| C | -2.0452 | -1.8906 | 7.3526 |
| H | -1.1045 | -2.3988 | 7.5747 |
| H | -2.8702 | -2.3074 | 7.9423 |
| H | -2.2802 | -1.9123 | 6.2788 |
| C | -1.1576 | 1.6531 | 7.8138 |
| C | -0.2124 | 2.6015 | 7.4029 |
| H | -0.2571 | 3.6052 | 7.8305 |
| C | -2.2370 | 1.9772 | 8.7652 |
| H | -2.7844 | 1.0901 | 9.1429 |
| N | -2.4940 | 3.2129 | 9.0726 |
| C | -3.5343 | 3.5312 | 10.0025 |
| C | -3.7633 | 2.7675 | 11.1678 |
| H | -3.1521 | 1.8874 | 11.3864 |
| C | -4.7627 | 3.1592 | 12.0473 |
| C | -5.5200 | 4.3251 | 11.8018 |
| C | -5.2919 | 5.0737 | 10.6505 |
| H | -5.8847 | 5.9706 | 10.4576 |
| C | -4.3002 | 4.6813 | 9.7449 |
| H | -4.1170 | 5.2568 | 8.8391 |
| C | 0.7517 | 2.2493 | 6.4561 |
| O | 1.7407 | 3.0817 | 5.9649 |
| C | 1.8647 | 4.3970 | 6.5557 |
| H | 2.0501 | 4.3215 | 7.6307 |
| H | -0.4118 | 5.5252 | 5.9124 |
| H | 2.7410 | 4.7984 | 6.0283 |
| H | 1.7324 | 1.4841 | 4.0075 |
| C | 4.1894 | 0.4606 | 3.1563 |
| H | 4.0881 | 1.4774 | 3.5293 |
| C | 4.0513 | 2.6954 | -1.5156 |
| H | 3.9555 | 2.5145 | -2.5841 |
| C | 6.9384 | 1.3374 | -2.2273 |
| H | 7.6591 | 1.9952 | -1.6938 |
| H | 7.5199 | 0.4547 | -2.5662 |
| C | 6.4332 | 2.1036 | -3.4565 |
| H | 6.0268 | 3.0872 | -3.1461 |
| C | 7.5547 | 2.3203 | -4.4709 |
| H | 7.2011 | 2.8959 | -5.3334 |
| H | 8.3938 | 2.8746 | -4.0326 |
| H | 7.9512 | 1.3713 | -4.8489 |
| H | 5.5957 | 1.5564 | -3.9407 |
| C | 6.5160 | 0.2711 | 0.1245 |
| C | 8.0359 | 0.2899 | 0.0968 |
| H | 8.4426 | -0.3504 | -0.6992 |
| H | 8.4711 | -0.0637 | 1.0427 |
| H | 8.4327 | 1.3010 | -0.0765 |
| C | 5.9283 | -1.2123 | 0.3054 |
| C | 7.0352 | -2.2841 | 0.3581 |
| C | 6.5813 | -3.7483 | 0.3080 |
| H | 5.7700 | -3.9295 | 1.0461 |
| H | 6.1531 | -3.9777 | -0.6881 |
| C | 7.7458 | -4.6966 | 0.5877 |
| H | 8.1544 | -4.5510 | 1.5942 |
| H | 7.4306 | -5.7429 | 0.5098 |
| H | 8.5685 | -4.5482 | -0.1225 |
| H | 7.6277 | -2.1222 | 1.2829 |
| H | 7.7364 | -2.1171 | -0.4885 |
| C | 3.4153 | -1.3676 | -3.1980 |
| H | 2.8106 | -1.3731 | -4.1056 |
| C | -0.9734 | -6.1635 | -3.3406 |
| O | -1.8809 | -6.1994 | -4.3736 |
| C | -1.9269 | -5.0692 | -5.2846 |
| H | -0.9737 | -4.9625 | -5.8074 |
| H | -2.1998 | -4.1520 | -4.7448 |
| H | -2.7264 | -5.3716 | -5.9714 |
| C | -3.1409 | -11.1062 | -3.3456 |
| H | -2.4635 | -10.8383 | -4.1600 |
| C | -7.4109 | -12.0364 | -6.4262 |
| H | -8.4278 | -12.4177 | -6.4839 |
| C | -6.9329 | -15.2179 | -5.8844 |
| H | -7.1215 | -16.1063 | -5.2462 |
| H | -6.0381 | -15.4568 | -6.4971 |
| C | -8.1202 | -15.0350 | -6.8383 |
| H | -7.8651 | -14.2930 | -7.6217 |
| C | -8.5091 | -16.3560 | -7.4999 |
| H | -9.3373 | -16.2142 | -8.2037 |
| H | -7.6742 | -16.7886 | -8.0625 |
| H | -8.8300 | -17.1006 | -6.7625 |
| H | -8.9965 | -14.6230 | -6.2920 |
| C | -5.1992 | -14.2168 | -4.1986 |
| C | -4.6080 | -15.6023 | -4.4149 |
| H | -3.6598 | -15.7304 | -3.8735 |
| H | -5.2879 | -16.3908 | -4.0660 |
| H | -4.4011 | -15.7996 | -5.4762 |
| C | -5.4894 | -13.9257 | -2.6464 |
| C | -4.9664 | -15.0505 | -1.7230 |
| C | -4.9530 | -14.7501 | -0.2186 |
| H | -4.1866 | -13.9814 | 0.0062 |
| H | -5.9287 | -14.3233 | 0.1007 |
| C | -4.6589 | -16.0090 | 0.5952 |
| H | -3.6933 | -16.4507 | 0.3230 |
| H | -4.6232 | -15.7846 | 1.6675 |
| H | -5.4265 | -16.7770 | 0.4469 |
| H | -3.9286 | -15.3070 | -2.0257 |
| H | -5.5788 | -15.9580 | -1.9018 |
| C | -9.7771 | -13.5958 | -2.8075 |
| H | -10.8608 | -13.5293 | -2.8859 |
| C | -12.4012 | -13.6541 | 3.1356 |
| O | -13.4492 | -14.5206 | 3.3911 |
| C | -13.7678 | -15.5041 | 2.3771 |
| H | -12.9139 | -16.1669 | 2.2068 |
| H | -14.0890 | -15.0092 | 1.4556 |
| H | -14.6000 | -16.0447 | 2.8456 |
| C | -14.9352 | -12.1924 | 7.5920 |
| H | -15.4554 | -12.4779 | 6.6812 |
| C | -19.7004 | -9.8015 | 8.3796 |
| H | -20.2892 | -9.1059 | 8.9831 |
| C | -19.5304 | -10.7301 | 11.4159 |
| H | -19.1424 | -10.9870 | 12.4245 |
| H | -20.1582 | -11.5865 | 11.0941 |
| C | -20.4215 | -9.4897 | 11.5583 |
| H | -20.7019 | -9.0979 | 10.5560 |
| C | -21.6881 | -9.8045 | 12.3524 |
| H | -22.3090 | -8.9098 | 12.4743 |
| H | -22.3024 | -10.5620 | 11.8518 |
| H | -21.4552 | -10.1801 | 13.3551 |
| H | -19.8604 | -8.6792 | 12.0652 |
| C | -17.3225 | -11.8475 | 10.5658 |
| C | -17.8475 | -12.9441 | 11.4811 |
| H | -17.1499 | -13.7906 | 11.5429 |
| H | -18.0037 | -12.5806 | 12.5070 |
| H | -18.8095 | -13.3397 | 11.1299 |
| C | -15.9260 | -11.2394 | 11.0625 |
| C | -15.4942 | -11.7857 | 12.4348 |
| C | -14.3062 | -11.0856 | 13.1069 |
| H | -13.4615 | -10.9839 | 12.3906 |
| H | -14.5915 | -10.0544 | 13.3985 |
| C | -13.8366 | -11.8508 | 14.3427 |
| H | -13.4891 | -12.8586 | 14.0894 |
| H | -13.0057 | -11.3313 | 14.8341 |
| H | -14.6379 | -11.9552 | 15.0829 |
| H | -15.2570 | -12.8635 | 12.3190 |
| H | -16.3590 | -11.7289 | 13.1297 |
| C | -16.8925 | -7.0495 | 10.7522 |
| H | -17.1689 | -6.0021 | 10.6433 |
| C | -12.1941 | -2.8647 | 11.7873 |
| O | -12.5716 | -1.6174 | 12.2525 |
| C | -13.9876 | -1.3698 | 12.4302 |
| H | -14.3944 | -2.0328 | 13.2002 |
| H | -14.5161 | -1.4775 | 11.4783 |
| H | -13.9899 | -0.3256 | 12.7673 |
| C | -5.2128 | 2.4284 | 13.2985 |
| C | -4.0483 | 2.0976 | 14.2522 |
| C | -3.1011 | 0.9762 | 13.8092 |
| H | -3.6260 | 0.0008 | 13.8452 |
| H | -2.7927 | 1.1203 | 12.7500 |
| C | -1.8584 | 0.9174 | 14.6927 |
| H | -2.1106 | 0.9144 | 15.7585 |
| H | -1.2842 | -0.0079 | 14.4951 |
| H | -1.1934 | 1.7769 | 14.5068 |
| H | -4.4671 | 1.8168 | 15.2416 |
| H | -3.4580 | 3.0240 | 14.4134 |
| C | -8.2641 | 0.2816 | 13.0034 |
| H | -9.3071 | 0.3525 | 13.3040 |
| C | -6.2887 | 3.4153 | 13.9591 |
| C | -5.8392 | 3.9338 | 15.3176 |
| H | -5.6966 | 3.1181 | 16.0407 |
| H | -4.8847 | 4.4736 | 15.2509 |
| H | -6.5731 | 4.6257 | 15.7533 |
| C | -6.5169 | 4.6080 | 12.9049 |
| C | -6.2812 | 5.9989 | 13.5395 |
| H | -5.2048 | 6.0760 | 13.8024 |
| H | -6.8422 | 6.0544 | 14.4964 |
| C | -6.6735 | 7.2211 | 12.6998 |
| H | -7.7778 | 7.2907 | 12.6301 |
| C | -6.1264 | 8.5092 | 13.3128 |
| H | -6.4197 | 9.3861 | 12.7258 |
| H | -6.5029 | 8.6622 | 14.3315 |
| H | -5.0313 | 8.4994 | 13.3741 |
| H | -6.3014 | 7.1132 | 11.6597 |
| C | -10.6701 | 3.9242 | 11.9992 |
| H | -11.7306 | 3.7466 | 11.8251 |
| H | -11.5869 | 10.5777 | 7.3525 |
| C | -13.5416 | 7.8356 | 7.8892 |
| O | -14.8033 | 7.4500 | 7.4796 |
| C | -15.2610 | 6.1313 | 7.8664 |
| H | -15.3512 | 6.0651 | 8.9547 |
| H | -14.5955 | 5.3616 | 7.4640 |
| H | -16.2476 | 6.0913 | 7.3857 |
| C | -16.5193 | 13.6994 | 4.3120 |
| C | -20.8335 | 10.7689 | 3.4210 |
| H | -21.4111 | 10.8582 | 2.4972 |
| C | -21.1194 | 13.8537 | 2.7254 |
| H | -21.7952 | 13.8895 | 3.6049 |
| H | -20.8851 | 14.9080 | 2.4669 |
| C | -21.8779 | 13.2377 | 1.5428 |
| C | -23.2549 | 13.8772 | 1.3729 |
| H | -23.1825 | 14.9586 | 1.2116 |
| H | -23.7825 | 13.4525 | 0.5114 |
| H | -23.8874 | 13.7173 | 2.2538 |
| H | -21.2929 | 13.3688 | 0.6106 |
| H | -21.9951 | 12.1413 | 1.6879 |
| C | -18.9582 | 14.0084 | 4.1938 |
| C | -19.7109 | 15.2357 | 4.6866 |
| H | -19.1214 | 15.8082 | 5.4162 |
| H | -19.9582 | 15.9218 | 3.8640 |
| H | -20.6557 | 14.9624 | 5.1744 |
| C | -17.5808 | 14.3725 | 3.4630 |
| C | -17.3822 | 15.8875 | 3.2881 |
| H | -17.2464 | 16.3368 | 4.2938 |
| C | -16.2203 | 16.3254 | 2.3875 |
| H | -16.4271 | 16.0360 | 1.3373 |
| C | -15.9963 | 17.8346 | 2.4637 |
| H | -15.1828 | 18.1450 | 1.7981 |
| H | -16.8916 | 18.3922 | 2.1662 |
| H | -15.7307 | 18.1549 | 3.4774 |
| H | -15.2856 | 15.7970 | 2.6780 |
| H | -18.3158 | 16.3242 | 2.8739 |
| C | -18.0299 | 11.9058 | -0.0422 |
| H | -18.1682 | 11.2488 | -0.8993 |
| C | -13.9241 | 11.5198 | -2.8833 |
| H | -14.9866 | 11.4160 | -3.1157 |
| C | -12.9367 | 11.2549 | -3.8377 |
| O | -13.1872 | 10.8385 | -5.1329 |
| C | -14.5681 | 10.6267 | -5.5151 |
| H | -15.0162 | 9.8397 | -4.9010 |
| H | -15.1286 | 11.5643 | -5.4498 |
| H | -14.4621 | 10.3009 | -6.5577 |
| C | -5.4614 | 10.3035 | -8.5622 |
| C | -4.3254 | 11.3326 | -8.7093 |
| C | -3.4607 | 11.5865 | -7.4673 |
| C | -2.2146 | 12.3977 | -7.8173 |
| H | -2.4688 | 13.3644 | -8.2639 |
| H | -1.6058 | 12.5938 | -6.9254 |
| H | -1.5726 | 11.8656 | -8.5298 |
| H | -4.0509 | 12.1232 | -6.6985 |
| H | -3.1523 | 10.6222 | -7.0053 |
| H | -4.7638 | 12.3041 | -9.0210 |
| H | -3.6686 | 11.0057 | -9.5424 |
| C | -8.6980 | 10.9700 | -6.8036 |
| H | -9.7356 | 11.1071 | -7.1025 |
| C | -8.3922 | 12.0567 | -9.8681 |
| H | -7.5712 | 12.8024 | -9.8094 |
| H | -8.5896 | 11.8927 | -10.9477 |
| C | -9.6386 | 12.6806 | -9.2276 |
| C | -10.1715 | 13.8412 | -10.0657 |
| H | -10.4801 | 13.5132 | -11.0648 |
| H | -11.0441 | 14.3025 | -9.5887 |
| H | -9.4185 | 14.6268 | -10.1954 |
| H | -10.4362 | 11.9153 | -9.1062 |
| H | -9.3973 | 13.0436 | -8.2080 |
| C | -6.4559 | 10.2999 | -9.8196 |
| C | -5.9774 | 11.2021 | -10.9480 |
| H | -4.9957 | 10.8917 | -11.3305 |
| H | -6.6743 | 11.1965 | -11.7965 |
| H | -5.8779 | 12.2475 | -10.6213 |
| C | -6.5891 | 8.7672 | -10.2883 |
| C | -6.1562 | 8.5680 | -11.7611 |
| C | -6.0889 | 7.1268 | -12.2801 |
| H | -7.0061 | 6.5643 | -12.0058 |
| C | -5.9030 | 7.0896 | -13.7954 |
| H | -5.8274 | 6.0574 | -14.1575 |
| H | -6.7442 | 7.5582 | -14.3192 |
| H | -4.9902 | 7.6108 | -14.1069 |
| H | -5.2408 | 6.5867 | -11.7956 |
| H | -5.1565 | 9.0301 | -11.9027 |
| H | -6.8582 | 9.1404 | -12.4033 |
| C | -10.8326 | 8.2672 | -9.7636 |
| H | -11.9111 | 8.1845 | -9.6373 |
| C | -12.5616 | 3.7619 | -11.2581 |
| H | -12.9920 | 4.5328 | -10.6161 |
| C | -13.2609 | 2.5913 | -11.5637 |
| O | -14.5395 | 2.2951 | -11.1271 |
| C | -15.1185 | 3.1583 | -10.1188 |
| H | -14.4881 | 3.1801 | -9.2247 |
| H | -15.2822 | 4.1593 | -10.5280 |
| H | -16.0718 | 2.6532 | -9.9158 |
| C | -16.0140 | -5.1391 | -15.0100 |
| C | -15.5661 | -6.0419 | -16.1729 |
| C | -14.2862 | -6.8591 | -15.9565 |
| C | -13.8275 | -7.5261 | -17.2520 |
| H | -13.5866 | -6.7883 | -18.0257 |
| H | -12.9295 | -8.1322 | -17.0857 |
| H | -14.5976 | -8.1907 | -17.6599 |
| H | -13.4730 | -6.2083 | -15.5671 |
| H | -14.4604 | -7.6358 | -15.1845 |
| H | -15.4359 | -5.4049 | -17.0725 |
| H | -16.3865 | -6.7526 | -16.4091 |
| C | -15.2599 | -1.5622 | -14.2442 |
| H | -15.8335 | -0.6543 | -14.0757 |
| C | -17.7232 | -2.1341 | -16.2882 |
| H | -17.2058 | -2.5792 | -17.1644 |
| H | -18.8103 | -2.2706 | -16.4625 |
| C | -17.4084 | -0.6328 | -16.2726 |
| C | -18.0883 | 0.0884 | -17.4352 |
| H | -19.1801 | 0.0138 | -17.3763 |
| H | -17.8327 | 1.1543 | -17.4394 |
| H | -17.7815 | -0.3242 | -18.4029 |
| H | -17.7337 | -0.1761 | -15.3131 |
| H | -16.3121 | -0.4765 | -16.3335 |
| C | -17.4754 | -4.5227 | -15.2356 |
| C | -18.0591 | -4.8649 | -16.5988 |
| H | -18.1438 | -5.9509 | -16.7471 |
| H | -19.0643 | -4.4423 | -16.7265 |
| H | -17.4369 | -4.4762 | -17.4169 |
| C | -18.3868 | -5.0604 | -14.0308 |
| C | -19.5412 | -5.9600 | -14.5162 |
| H | -19.1357 | -6.7019 | -15.2365 |
| C | -20.3112 | -6.7284 | -13.4347 |
| H | -20.5963 | -6.0457 | -12.6042 |
| C | -21.5675 | -7.3827 | -14.0070 |
| H | -22.1005 | -7.9518 | -13.2370 |
| H | -22.2669 | -6.6381 | -14.4046 |
| H | -21.3286 | -8.0770 | -14.8203 |
| H | -19.6601 | -7.5072 | -12.9897 |
| H | -20.2559 | -5.3298 | -15.0848 |
| C | -19.4512 | -1.2881 | -12.2471 |
| H | -19.6862 | -0.3173 | -11.8135 |
| C | -23.5841 | -1.5337 | -7.3257 |
| O | -24.2713 | -0.3714 | -7.0268 |
| C | -24.0623 | 0.7720 | -7.8908 |
| H | -23.0160 | 1.0892 | -7.8563 |
| H | -24.3854 | 0.5419 | -8.9109 |
| H | -24.7181 | 1.5214 | -7.4296 |
| C | -28.5714 | -2.8732 | 0.0056 |
| C | -29.5504 | -4.0558 | 0.1769 |
| C | -28.9286 | -5.4518 | 0.3109 |
| C | -29.9673 | -6.4813 | 0.7527 |
| H | -30.8056 | -6.5393 | 0.0496 |
| H | -29.5258 | -7.4822 | 0.8190 |
| H | -30.3806 | -6.2419 | 1.7395 |
| H | -28.4913 | -5.7636 | -0.6587 |
| H | -28.0911 | -5.4367 | 1.0419 |
| H | -30.2399 | -4.0763 | -0.6937 |
| H | -30.1784 | -3.8563 | 1.0699 |
| C | -26.9872 | -2.1921 | -3.3162 |
| H | -27.1482 | -1.4763 | -4.1257 |
| C | -29.8432 | -0.8027 | -2.8246 |
| H | -30.3318 | -1.7793 | -3.0251 |
| H | -30.6225 | -0.1471 | -2.3810 |
| C | -29.4170 | -0.1824 | -4.1612 |
| C | -30.5511 | -0.2357 | -5.1835 |
| H | -31.4439 | 0.2901 | -4.8270 |
| H | -30.2516 | 0.2318 | -6.1285 |
| H | -30.8448 | -1.2674 | -5.4095 |
| H | -29.1104 | 0.8715 | -4.0072 |
| H | -28.5280 | -0.7114 | -4.5690 |
| C | -29.3230 | -1.5027 | -0.3621 |
| C | -30.8359 | -1.6687 | -0.3751 |
| H | -31.2189 | -1.9771 | 0.6067 |
| H | -31.3472 | -0.7330 | -0.6424 |
| H | -31.1565 | -2.4300 | -1.0999 |
| C | -28.8413 | -0.4174 | 0.7180 |
| C | -30.0239 | 0.3011 | 1.4000 |
| C | -29.6804 | 1.5053 | 2.2860 |
| H | -29.3286 | 2.3482 | 1.6572 |
| C | -30.8898 | 1.9547 | 3.1042 |
| H | -30.6490 | 2.8356 | 3.7103 |
| H | -31.7364 | 2.2229 | 2.4621 |
| H | -31.2315 | 1.1698 | 3.7885 |
| H | -28.8418 | 1.2565 | 2.9720 |
| H | -30.5744 | -0.4452 | 2.0097 |
| H | -30.7298 | 0.6483 | 0.6160 |
| C | -26.3793 | 2.0289 | -1.8338 |
| H | -25.7692 | 2.6259 | -2.5104 |
| C | -23.8597 | 4.9055 | 2.8155 |
| O | -24.3375 | 3.9310 | 3.6724 |
| C | -24.0110 | 4.0552 | 5.0779 |
| H | -22.9282 | 4.0015 | 5.2235 |
| H | -24.5106 | 3.1738 | 5.5001 |
| H | -24.4325 | 4.9790 | 5.4861 |
| C | -23.0886 | 6.0059 | 3.2052 |
| H | -22.8150 | 6.1714 | 4.2497 |
| C | -22.6551 | 6.9287 | 2.2468 |

j) *(****OMe-cube)****(****OMe-cube)****(****OMe-cube)*** *(Type 1 conformer, 1 and 4 position of middle cage is interlocked).*

| **atom** | **x** | **y** | **z** |
| --- | --- | --- | --- |
| C | -12.9404 | 1.7545 | -15.3933 |
| C | -14.0739 | -0.5307 | -15.7050 |
| C | -15.4668 | 1.6259 | -15.8531 |
| C | -14.0507 | 0.9767 | -16.2492 |
| C | -12.4652 | 0.7272 | -14.3786 |
| C | -13.1053 | -0.5208 | -14.5356 |
| C | -12.8607 | -1.5509 | -13.6280 |
| C | -11.9531 | -1.3586 | -12.5807 |
| N | -10.3719 | 0.0098 | -11.3658 |
| C | -11.3215 | -0.1132 | -12.4265 |
| C | -11.5858 | 0.9412 | -13.3243 |
| C | -15.4952 | -0.7156 | -15.1908 |
| C | -16.2467 | 0.4781 | -15.2507 |
| C | -17.5593 | 0.5042 | -14.7868 |
| C | -18.1354 | -0.6530 | -14.2551 |
| N | -18.0614 | -2.9956 | -13.6873 |
| C | -17.3862 | -1.8405 | -14.1914 |
| C | -16.0602 | -1.8757 | -14.6726 |
| C | -15.1183 | 2.6750 | -14.8149 |
| C | -13.7239 | 2.8172 | -14.6449 |
| C | -13.2186 | 3.7999 | -13.7975 |
| C | -14.0946 | 4.6158 | -13.0732 |
| N | -16.3284 | 5.3004 | -12.4396 |
| C | -15.4818 | 4.4186 | -13.1839 |
| C | -15.9995 | 3.4422 | -14.0631 |
| C | -2.5184 | 1.6275 | -2.6507 |
| C | -3.0498 | 3.6204 | -1.1127 |
| C | -2.6192 | 1.2885 | -0.1069 |
| C | -2.1322 | 2.3073 | -1.2494 |
| C | -3.6810 | 2.4544 | -3.1568 |
| C | -3.9693 | 3.5553 | -2.3229 |
| C | -5.0601 | 4.3750 | -2.6083 |
| C | -5.8519 | 4.1147 | -3.7350 |
| N | -6.4269 | 2.8131 | -5.6807 |
| C | -5.5697 | 3.0031 | -4.5511 |
| C | -4.4776 | 2.1651 | -4.2551 |
| C | -3.8820 | 3.3584 | 0.1280 |
| C | -3.7323 | 2.0384 | 0.6107 |
| C | -4.5584 | 1.5862 | 1.6388 |
| C | -5.4614 | 2.4629 | 2.2528 |
| N | -6.3861 | 4.6820 | 2.5759 |
| C | -5.5500 | 3.7950 | 1.8208 |
| C | -4.7650 | 4.2445 | 0.7333 |
| C | -3.1313 | 0.0891 | -0.8755 |
| C | -3.0227 | 0.2567 | -2.2699 |
| C | -3.4051 | -0.7613 | -3.1377 |
| C | -3.9311 | -1.9511 | -2.6248 |
| N | -4.5498 | -3.3588 | -0.7525 |
| C | -4.0721 | -2.1023 | -1.2351 |
| C | -3.6611 | -1.0795 | -0.3504 |
| C | -14.1677 | 13.6434 | 9.4969 |
| C | -11.7300 | 13.3920 | 8.7175 |
| C | -13.4182 | 14.6523 | 7.2523 |
| C | -12.9603 | 14.4175 | 8.7681 |
| C | -13.5842 | 12.2876 | 9.8459 |
| C | -12.2116 | 12.1960 | 9.5216 |
| C | -11.4835 | 11.0616 | 9.8730 |
| C | -12.1227 | 9.9878 | 10.5006 |
| N | -14.1190 | 8.9681 | 11.4458 |
| C | -13.5032 | 10.0523 | 10.7456 |
| C | -14.2452 | 11.2055 | 10.4144 |
| C | -11.6448 | 12.9843 | 7.2529 |
| C | -12.5493 | 13.7068 | 6.4457 |
| C | -12.6428 | 13.4389 | 5.0826 |
| C | -11.8435 | 12.4453 | 4.5126 |
| N | -10.2103 | 10.6877 | 4.6553 |
| C | -10.9788 | 11.6932 | 5.3256 |
| C | -10.8710 | 11.9641 | 6.7059 |
| C | -14.8462 | 14.1279 | 7.2114 |
| C | -15.2424 | 13.5443 | 8.4341 |
| C | -16.5016 | 12.9592 | 8.5512 |
| C | -17.3640 | 12.9237 | 7.4495 |
| N | -17.8730 | 13.4323 | 5.1295 |
| C | -16.9555 | 13.4845 | 6.2280 |
| C | -15.6992 | 14.1140 | 6.1153 |
| C | -10.0387 | 9.4956 | 5.1344 |
| C | -9.2898 | 8.5087 | 4.3261 |
| C | -9.3192 | 8.5414 | 2.9275 |
| C | -8.6448 | 7.5601 | 2.1917 |
| C | -7.8943 | 6.5386 | 2.8312 |
| C | -7.8685 | 6.5071 | 4.2305 |
| C | -8.5623 | 7.4763 | 4.9660 |
| C | -7.1366 | 5.5735 | 2.0044 |
| C | -24.8828 | 13.2214 | -4.4359 |
| C | -23.6772 | 12.5158 | -6.5938 |
| C | -25.9305 | 11.4477 | -5.9744 |
| C | -25.1213 | 12.8353 | -5.9735 |
| C | -23.3910 | 13.0173 | -4.2275 |
| C | -22.7374 | 12.5929 | -5.4040 |
| C | -21.3886 | 12.2418 | -5.3679 |
| C | -20.6717 | 12.3537 | -4.1721 |
| N | -20.5024 | 12.9177 | -1.8398 |
| C | -21.3224 | 12.7861 | -3.0029 |
| C | -22.6952 | 13.1093 | -3.0282 |
| C | -23.7863 | 11.0794 | -7.0675 |
| C | -25.0103 | 10.4817 | -6.6949 |
| C | -25.2563 | 9.1473 | -7.0082 |
| C | -24.2901 | 8.4012 | -7.6917 |
| N | -22.1715 | 8.1594 | -8.7953 |
| C | -23.0729 | 8.9995 | -8.0677 |
| C | -22.8255 | 10.3555 | -7.7610 |
| C | -26.0813 | 11.1049 | -4.5039 |
| C | -25.6056 | 12.1355 | -3.6645 |
| C | -25.7510 | 12.0399 | -2.2842 |
| C | -26.3263 | 10.8974 | -1.7207 |
| N | -27.3423 | 8.7096 | -1.9221 |
| C | -26.7270 | 9.8379 | -2.5518 |
| C | -26.6044 | 9.9395 | -3.9564 |
| C | -20.9564 | 12.7059 | -0.6435 |
| C | -20.0465 | 12.8499 | 0.5130 |
| C | -18.6556 | 12.8470 | 0.3634 |
| C | -17.8327 | 12.9437 | 1.4901 |
| C | -18.3718 | 13.0508 | 2.7973 |
| C | -19.7630 | 13.0756 | 2.9455 |
| C | -20.5859 | 12.9731 | 1.8186 |
| C | -17.3984 | 4.8651 | -11.8483 |
| C | -18.2757 | 5.8001 | -11.1129 |
| C | -17.9065 | 7.1239 | -10.8431 |
| C | -18.7722 | 7.9585 | -10.1288 |
| C | -20.0344 | 7.5022 | -9.6684 |
| C | -20.4084 | 6.1841 | -9.9484 |
| C | -19.5396 | 5.3470 | -10.6576 |
| C | -7.5792 | 1.5093 | -7.3307 |
| C | -20.9038 | 8.4193 | -8.9006 |
| C | -17.4470 | 13.1095 | 3.9469 |
| C | -6.6683 | 1.6354 | -6.1737 |
| C | -20.5510 | -1.6617 | 15.5517 |
| C | -19.0617 | -3.7588 | 15.5501 |
| C | -18.0022 | -1.4258 | 15.7618 |
| C | -19.2502 | -2.3182 | 16.2293 |
| C | -20.9692 | -2.6722 | 14.4993 |
| C | -20.2217 | -3.8669 | 14.5792 |
| C | -20.5242 | -4.9401 | 13.7462 |
| C | -21.5354 | -4.8159 | 12.7882 |
| N | -23.2463 | -3.5429 | 11.6528 |
| C | -22.2220 | -3.5960 | 12.6521 |
| C | -21.9427 | -2.5160 | 13.5196 |
| C | -17.7953 | -3.6140 | 14.7183 |
| C | -17.2561 | -2.3109 | 14.7857 |
| C | -16.1827 | -1.9524 | 13.9741 |
| C | -15.5985 | -2.9022 | 13.1310 |
| N | -15.4344 | -5.1496 | 12.2538 |
| C | -16.1194 | -4.2056 | 13.0819 |
| C | -17.2350 | -4.5628 | 13.8705 |
| C | -18.6375 | -0.2502 | 15.0433 |
| C | -20.0379 | -0.3855 | 14.9250 |
| C | -20.7856 | 0.5955 | 14.2823 |
| C | -20.1457 | 1.7175 | 13.7428 |
| N | -18.1148 | 2.9378 | 13.2044 |
| C | -18.7521 | 1.8462 | 13.8650 |
| C | -17.9910 | 0.8635 | 14.5269 |
| C | -17.4727 | 3.8622 | 13.8367 |
| C | -16.8338 | 4.9512 | 13.0648 |
| C | -13.3649 | -9.7671 | 8.0687 |
| C | -14.0648 | -8.8428 | 8.9827 |
| C | -7.1455 | -8.4409 | 2.4827 |
| C | -6.7629 | -7.1342 | 1.9022 |
| C | -15.4239 | -8.9894 | 9.2889 |
| C | -16.0552 | -8.0565 | 10.1156 |
| C | -15.3513 | -6.9531 | 10.6637 |
| C | -13.9899 | -6.8211 | 10.3783 |
| C | -13.3554 | -7.7543 | 9.5488 |
| C | -16.0840 | -5.9779 | 11.4974 |
| C | -22.0631 | -14.3492 | -8.5909 |
| C | -24.4292 | -13.8659 | -7.7090 |
| C | -23.5937 | -12.7108 | -9.8513 |
| C | -23.5814 | -14.1025 | -9.0502 |
| C | -22.1077 | -14.2281 | -7.0771 |
| C | -23.4207 | -14.0442 | -6.5930 |
| C | -23.6615 | -13.9750 | -5.2234 |
| C | -22.5936 | -14.0334 | -4.3236 |
| N | -20.2275 | -14.2030 | -3.8444 |
| C | -21.2797 | -14.1346 | -4.8111 |
| C | -21.0318 | -14.2392 | -6.1972 |
| C | -24.8390 | -12.4049 | -7.7817 |
| C | -24.4244 | -11.7890 | -8.9818 |
| C | -24.7375 | -10.4550 | -9.2303 |
| C | -25.4267 | -9.7092 | -8.2696 |
| N | -26.4927 | -9.4860 | -6.1157 |
| C | -25.7809 | -10.3061 | -7.0467 |
| C | -25.4917 | -11.6661 | -6.8025 |
| C | -22.1427 | -12.2583 | -9.8349 |
| C | -21.3000 | -13.1836 | -9.1828 |
| C | -19.9306 | -12.9491 | -9.0964 |
| C | -19.3897 | -11.7747 | -9.6257 |
| N | -19.5881 | -9.6607 | -10.7489 |
| C | -20.2349 | -10.8246 | -10.2258 |
| C | -21.6221 | -11.0698 | -10.3341 |
| C | -19.1519 | -13.4901 | -3.9750 |
| C | -20.2023 | -8.5253 | -10.8786 |
| C | -18.0754 | -13.5801 | -2.9677 |
| C | -19.4762 | -7.3637 | -11.4323 |
| C | -18.2241 | -14.2999 | -1.7760 |
| C | -17.1827 | -14.3291 | -0.8441 |
| C | -15.9597 | -13.6493 | -1.0750 |
| C | -15.8048 | -12.9421 | -2.2716 |
| C | -16.8499 | -12.9069 | -3.2018 |
| C | -18.0796 | -7.2941 | -11.4380 |
| C | -17.4400 | -6.1578 | -11.9444 |
| C | -18.1735 | -5.0628 | -12.4658 |
| C | -19.5714 | -5.1395 | -12.4755 |
| C | -20.2109 | -6.2724 | -11.9622 |
| C | -32.5809 | -0.7890 | 2.9306 |
| C | -32.6037 | -2.8166 | 1.3536 |
| C | -32.0646 | -3.1382 | 3.8466 |
| C | -33.0089 | -2.3300 | 2.8263 |
| C | -31.9020 | -0.4970 | 1.6008 |
| C | -31.8908 | -1.6223 | 0.7484 |
| C | -31.2160 | -1.5731 | -0.4696 |
| C | -30.5869 | -0.3907 | -0.8690 |
| N | -29.9475 | 1.8897 | -0.5535 |
| C | -30.6085 | 0.7370 | -0.0286 |
| C | -31.2585 | 0.6763 | 1.2239 |
| C | -31.6057 | -3.9316 | 1.5926 |
| C | -31.2860 | -4.0874 | 2.9580 |
| C | -30.3459 | -5.0381 | 3.3482 |
| C | -29.7121 | -5.8307 | 2.3848 |
| N | -29.3717 | -6.5402 | 0.1019 |
| C | -30.0301 | -5.6681 | 1.0244 |
| C | -30.9976 | -4.7214 | 0.6272 |
| C | -31.1714 | -2.0728 | 4.4505 |
| C | -31.5159 | -0.7737 | 4.0161 |
| C | -30.8463 | 0.3347 | 4.5311 |
| C | -29.8083 | 0.1591 | 5.4511 |
| N | -28.3721 | -1.2151 | 6.8013 |
| C | -29.4254 | -1.1379 | 5.8364 |
| C | -30.1116 | -2.2633 | 5.3285 |
| C | -28.3834 | -7.0593 | -2.0298 |
| C | -29.7224 | 2.9580 | 0.1473 |
| C | -27.6350 | -2.2741 | 6.9430 |
| C | -29.0355 | 4.1025 | -0.4877 |
| C | -26.5825 | -2.2963 | 7.9804 |
| C | -28.2916 | 3.9606 | -1.6644 |
| C | -27.6532 | 5.0720 | -2.2252 |
| C | -27.7442 | 6.3573 | -1.6339 |
| C | -28.4908 | 6.4996 | -0.4587 |
| C | -29.1215 | 5.3868 | 0.1066 |
| C | -27.7201 | -8.2130 | -1.5976 |
| C | -27.0563 | -9.0238 | -2.5246 |
| C | -27.0401 | -8.7113 | -3.9077 |
| C | -27.7228 | -7.5698 | -4.3421 |
| C | -28.3808 | -6.7560 | -3.4148 |
| C | -26.0501 | -1.1233 | 8.5270 |
| C | -25.0417 | -1.2001 | 9.4938 |
| C | -24.5428 | -2.4468 | 9.9497 |
| C | -25.0841 | -3.6197 | 9.4121 |
| C | -26.0849 | -3.5427 | 8.4379 |
| C | -26.2905 | -9.5806 | -4.8380 |
| C | -14.9021 | -13.6891 | -0.0440 |
| C | -23.4650 | -2.4665 | 10.9609 |
| C | -7.0071 | -5.9104 | 2.5412 |
| C | -6.5964 | -4.7137 | 1.9447 |
| C | -5.9036 | -4.7037 | 0.7103 |
| C | -5.6716 | -5.9196 | 0.0618 |
| C | -6.1089 | -7.1139 | 0.6486 |
| C | -16.0755 | 5.9414 | 13.7415 |
| C | -15.4643 | 6.9938 | 13.0508 |
| C | -15.5937 | 7.0865 | 11.6593 |
| C | -16.3460 | 6.0939 | 10.9828 |
| C | -16.9570 | 5.0421 | 11.6735 |
| C | -27.0544 | 7.4952 | -2.2752 |
| C | -14.9794 | 8.1844 | 10.8803 |
| C | -17.4448 | -3.8781 | -12.9639 |
| C | -5.4514 | -3.4033 | 0.1748 |
| C | -8.4258 | 2.5523 | -7.7073 |
| C | -9.2970 | 2.3919 | -8.7856 |
| C | -9.3644 | 1.1828 | -9.5181 |
| C | -8.5046 | 0.1369 | -9.1526 |
| C | -7.6251 | 0.2993 | -8.0742 |
| C | -10.3450 | 1.0676 | -10.6185 |
| C | -9.2747 | -13.4034 | 3.2886 |
| C | -9.9089 | -13.3605 | 5.7776 |
| C | -11.4380 | -14.5392 | 4.0839 |
| C | -9.9038 | -14.2897 | 4.4727 |
| C | -8.9853 | -12.0640 | 3.9388 |
| C | -9.1991 | -12.0940 | 5.3350 |
| C | -8.8609 | -10.9948 | 6.1194 |
| C | -8.3714 | -9.8317 | 5.5148 |
| N | -7.8666 | -8.5084 | 3.5603 |
| C | -8.2447 | -9.7720 | 4.1146 |
| C | -8.5532 | -10.8974 | 3.3192 |
| C | -11.3764 | -13.0097 | 5.9765 |
| C | -12.2120 | -13.6459 | 5.0344 |
| C | -13.5748 | -13.3588 | 4.9997 |
| C | -14.1182 | -12.4498 | 5.9116 |
| N | -13.9147 | -10.8960 | 7.7444 |
| C | -13.2818 | -11.8036 | 6.8381 |
| C | -11.8995 | -12.0857 | 6.8740 |
| C | -11.5547 | -13.9873 | 2.6710 |
| C | -10.3786 | -13.3243 | 2.2562 |
| C | -10.3268 | -12.7051 | 1.0101 |
| C | -11.4418 | -12.7397 | 0.1677 |
| N | -13.6809 | -13.3987 | -0.3712 |
| C | -12.6114 | -13.4055 | 0.5765 |
| C | -12.6623 | -14.0439 | 1.8341 |
| C | -29.0629 | -6.1412 | -1.0930 |
| O | -6.7766 | -3.4478 | 2.4640 |
| O | -5.9332 | -8.3714 | 0.0980 |
| C | -7.6177 | -3.2917 | 3.6323 |
| C | -5.5441 | -8.4215 | -1.2914 |
| O | -26.8986 | 5.0486 | -3.3836 |
| C | -26.6910 | 3.7665 | -4.0243 |
| O | -29.8766 | 5.4087 | 1.2651 |
| C | -30.0319 | 6.6808 | 1.9395 |
| O | -16.4008 | 6.2782 | 9.6152 |
| O | -16.0139 | 5.7541 | 15.1098 |
| C | -17.1637 | 5.3235 | 8.8367 |
| C | -15.2563 | 6.7142 | 15.8857 |
| O | -16.0704 | -5.9792 | -11.9978 |
| O | -21.5813 | -6.4549 | -11.9136 |
| C | -22.4174 | -5.3906 | -12.4288 |
| C | -15.2339 | -7.0231 | -11.4423 |
| O | -26.3691 | -10.1821 | -2.2129 |
| C | -26.2790 | -10.5541 | -0.8160 |
| O | -29.0797 | -5.6045 | -3.7283 |
| C | -29.1189 | -5.2019 | -5.1189 |
| O | -19.8078 | 4.0330 | -10.9913 |
| C | -21.0509 | 3.4591 | -10.5189 |
| O | -18.5088 | 9.2759 | -9.7985 |
| C | -17.2624 | 9.8471 | -10.2645 |
| O | -12.0216 | -7.7243 | 9.2048 |
| O | -17.3883 | -8.1004 | 10.4864 |
| C | -11.2178 | -6.6299 | 9.7132 |
| C | -18.2045 | -9.1562 | 9.9249 |
| O | -8.6836 | 7.4556 | 0.8152 |
| C | -9.0495 | 8.6391 | 0.0577 |
| O | -8.6112 | 7.5430 | 6.3447 |
| C | -7.9001 | 6.5250 | 7.0918 |
| O | -16.4506 | 12.9536 | 1.4591 |
| O | -21.9683 | 12.9825 | 1.8445 |
| C | -22.6138 | 13.0631 | 3.1385 |
| C | -15.8060 | 12.7494 | 0.1780 |
| O | -6.7693 | -0.6767 | -7.6000 |
| O | -10.1519 | 3.3715 | -9.2432 |
| C | -6.5870 | -1.8756 | -8.3959 |
| C | -10.1547 | 4.6163 | -8.5088 |
| O | -24.4430 | -0.1118 | 10.1013 |
| C | -24.8422 | 1.2081 | 9.6584 |
| O | -26.6866 | -4.6317 | 7.8335 |
| C | -26.2339 | -5.9500 | 8.2260 |
| O | -17.2208 | -14.9984 | 0.3659 |
| C | -18.4343 | -15.7167 | 0.6956 |
| O | -16.8044 | -12.2447 | -4.4137 |
| C | -15.6367 | -11.4340 | -4.6940 |
| C | -11.8123 | 2.3243 | -16.2726 |
| C | -13.6925 | -1.5530 | -16.8026 |
| C | -16.1943 | 2.2388 | -17.0711 |
| C | -13.7926 | 1.0419 | -17.7467 |
| H | -13.3808 | -2.5036 | -13.7118 |
| H | -11.7418 | -2.1639 | -11.8781 |
| H | -11.1100 | 1.9076 | -13.1787 |
| H | -18.1455 | 1.4253 | -14.8523 |
| H | -19.1617 | -0.6420 | -13.8915 |
| H | -15.4891 | -2.8084 | -14.6615 |
| H | -12.1371 | 3.9372 | -13.7010 |
| H | -13.7091 | 5.3939 | -12.4170 |
| H | -17.0743 | 3.2999 | -14.1457 |
| C | -1.3418 | 1.5776 | -3.6377 |
| C | -2.2466 | 4.9273 | -1.0161 |
| C | -1.4653 | 0.8951 | 0.8498 |
| C | -0.6457 | 2.6078 | -1.1380 |
| H | -5.3203 | 5.2059 | -1.9546 |
| H | -6.7000 | 4.7567 | -3.9726 |
| H | -4.2743 | 1.2946 | -4.8741 |
| H | -4.5098 | 0.5519 | 1.9763 |
| H | -6.0920 | 2.1172 | 3.0702 |
| H | -4.8294 | 5.2789 | 0.3848 |
| H | -3.2848 | -0.6345 | -4.2176 |
| H | -4.2314 | -2.7570 | -3.2928 |
| H | -3.7337 | -1.2180 | 0.7292 |
| C | -14.6697 | 14.3917 | 10.7527 |
| C | -10.4207 | 14.0145 | 9.2463 |
| C | -13.2938 | 16.1306 | 6.8272 |
| C | -12.6054 | 15.7166 | 9.4743 |
| H | -10.4100 | 11.0103 | 9.6627 |
| H | -11.5589 | 9.1044 | 10.7920 |
| H | -15.3139 | 11.2354 | 10.6094 |
| H | -13.3487 | 13.9787 | 4.4536 |
| H | -11.9038 | 12.2354 | 3.4458 |
| H | -10.2048 | 11.3711 | 7.3277 |
| H | -16.8204 | 12.5321 | 9.5063 |
| H | -18.3456 | 12.4594 | 7.5310 |
| H | -15.4038 | 14.5966 | 5.1800 |
| H | -10.4105 | 9.1071 | 6.1012 |
| H | -9.8742 | 9.3387 | 2.4286 |
| H | -7.2919 | 5.7202 | 4.7242 |
| H | -7.2662 | 5.7127 | 0.9123 |
| C | -25.3698 | 14.6415 | -4.1023 |
| C | -23.2917 | 13.4862 | -7.7296 |
| C | -27.3015 | 11.5676 | -6.6780 |
| C | -25.8544 | 13.9205 | -6.7486 |
| H | -20.8870 | 11.8640 | -6.2568 |
| H | -19.6135 | 12.0971 | -4.1370 |
| H | -23.1973 | 13.4158 | -2.1139 |
| H | -26.2104 | 8.6866 | -6.7352 |
| H | -24.4750 | 7.3567 | -7.9407 |
| H | -21.8998 | 10.8423 | -8.0793 |
| H | -25.4200 | 12.8601 | -1.6404 |
| H | -26.4548 | 10.8180 | -0.6427 |
| H | -26.9201 | 9.1151 | -4.5915 |
| H | -21.9870 | 12.4082 | -0.3733 |
| H | -18.2381 | 12.7731 | -0.6437 |
| H | -20.1788 | 13.1771 | 3.9506 |
| H | -17.7470 | 3.8135 | -11.8312 |
| H | -16.9394 | 7.4794 | -11.2064 |
| H | -21.3870 | 5.8360 | -9.6102 |
| H | -20.3701 | 9.2856 | -8.4663 |
| H | -16.4003 | 12.8501 | 3.6886 |
| H | -6.2692 | 0.6717 | -5.8048 |
| C | -21.6810 | -1.3919 | 16.5690 |
| C | -19.0138 | -4.8950 | 16.5842 |
| C | -17.1187 | -0.9803 | 16.9469 |
| C | -19.3693 | -2.3860 | 17.7459 |
| H | -19.9727 | -5.8797 | 13.8407 |
| H | -21.7838 | -5.6514 | 12.1358 |
| H | -22.4825 | -1.5779 | 13.4144 |
| H | -15.8020 | -0.9322 | 13.9734 |
| H | -14.7488 | -2.6357 | 12.5046 |
| H | -17.6466 | -5.5669 | 13.8024 |
| H | -21.8730 | 0.4956 | 14.2074 |
| H | -20.7201 | 2.4827 | 13.2247 |
| H | -16.9096 | 0.9744 | 14.6271 |
| H | -17.3446 | 3.9373 | 14.9337 |
| H | -12.3859 | -9.3924 | 7.7109 |
| H | -6.7546 | -9.3084 | 1.9183 |
| H | -15.9644 | -9.8409 | 8.8695 |
| H | -13.4406 | -5.9825 | 10.8135 |
| H | -17.1860 | -6.0372 | 11.3946 |
| C | -21.5165 | -15.7132 | -9.0625 |
| C | -25.6313 | -14.8239 | -7.5940 |
| C | -24.1653 | -12.8591 | -11.2764 |
| C | -24.1263 | -15.2546 | -9.8828 |
| H | -24.6858 | -13.8781 | -4.8511 |
| H | -22.7712 | -13.9862 | -3.2507 |
| H | -20.0105 | -14.3228 | -6.5605 |
| H | -24.4491 | -9.9918 | -10.1782 |
| H | -25.6800 | -8.6667 | -8.4558 |
| H | -25.7739 | -12.1202 | -5.8557 |
| H | -19.2771 | -13.6875 | -8.6222 |
| H | -18.3186 | -11.5849 | -9.5697 |
| H | -22.2694 | -10.3318 | -10.8021 |
| H | -18.9449 | -12.7741 | -4.7952 |
| H | -21.2545 | -8.3110 | -10.6101 |
| H | -19.1635 | -14.8296 | -1.6007 |
| H | -14.8570 | -12.4317 | -2.4587 |
| H | -17.5128 | -8.1427 | -11.0490 |
| H | -20.1370 | -4.3037 | -12.8939 |
| C | -33.7821 | 0.1303 | 3.2313 |
| C | -33.8216 | -3.2767 | 0.5264 |
| C | -32.8750 | -3.8934 | 4.9240 |
| C | -34.4855 | -2.5497 | 3.1200 |
| H | -31.1568 | -2.4509 | -1.1104 |
| H | -30.0686 | -0.3373 | -1.8265 |
| H | -31.2361 | 1.5363 | 1.8889 |
| H | -30.1114 | -5.1747 | 4.4077 |
| H | -28.9730 | -6.5739 | 2.6808 |
| H | -31.2829 | -4.6207 | -0.4231 |
| H | -31.1371 | 1.3438 | 4.2227 |
| H | -29.2862 | 1.0204 | 5.8667 |
| H | -29.8100 | -3.2651 | 5.6255 |
| H | -29.9990 | 3.1267 | 1.2043 |
| H | -27.7055 | -3.2091 | 6.3553 |
| H | -28.2342 | 2.9741 | -2.1303 |
| H | -28.5704 | 7.4918 | -0.0089 |
| H | -27.7397 | -8.4590 | -0.5335 |
| H | -27.7277 | -7.3395 | -5.4100 |
| H | -26.4455 | -0.1631 | 8.1886 |
| H | -24.7128 | -4.5802 | 9.7759 |
| H | -25.5784 | -10.2661 | -4.3383 |
| H | -15.2654 | -13.9762 | 0.9604 |
| H | -22.9085 | -1.5122 | 11.0420 |
| H | -7.5231 | -5.9148 | 3.5073 |
| H | -5.1366 | -5.9139 | -0.8915 |
| H | -14.8761 | 7.7529 | 13.5726 |
| H | -17.5346 | 4.2760 | 11.1488 |
| H | -26.3122 | 7.1991 | -3.0430 |
| H | -15.3171 | 8.2398 | 9.8269 |
| H | -16.3824 | -3.8410 | -12.6547 |
| H | -5.9377 | -2.5305 | 0.6512 |
| H | -8.3823 | 3.4890 | -7.1456 |
| H | -8.5397 | -0.7949 | -9.7249 |
| H | -11.0233 | 1.9387 | -10.7093 |
| C | -7.9902 | -14.0329 | 2.7011 |
| C | -9.2666 | -14.0421 | 6.9960 |
| C | -11.8371 | -16.0258 | 4.1899 |
| C | -9.1420 | -15.5905 | 4.6769 |
| H | -8.9809 | -11.0395 | 7.2071 |
| H | -8.1022 | -8.9693 | 6.1217 |
| H | -8.4779 | -10.8381 | 2.2363 |
| H | -14.2220 | -13.8171 | 4.2540 |
| H | -15.1838 | -12.2251 | 5.8984 |
| H | -11.2586 | -11.5712 | 7.5859 |
| H | -9.4112 | -12.2045 | 0.6836 |
| H | -11.4131 | -12.2542 | -0.8077 |
| H | -13.5559 | -14.5915 | 2.1463 |
| H | -29.2555 | -5.1355 | -1.5144 |
| H | -7.6787 | -2.1989 | 3.7160 |
| H | -8.6122 | -3.7252 | 3.4615 |
| H | -7.1256 | -3.7257 | 4.5068 |
| H | -5.4622 | -9.5016 | -1.4654 |
| H | -6.3419 | -7.9740 | -1.9033 |
| H | -4.5806 | -7.9259 | -1.4503 |
| H | -26.0574 | 4.0452 | -4.8763 |
| H | -26.1651 | 3.0837 | -3.3504 |
| H | -27.6459 | 3.3523 | -4.3622 |
| H | -30.6660 | 6.4045 | 2.7915 |
| H | -29.0604 | 7.0542 | 2.2761 |
| H | -30.5409 | 7.3976 | 1.2875 |
| H | -17.0338 | 5.7154 | 7.8191 |
| H | -16.7303 | 4.3231 | 8.9303 |
| H | -18.2160 | 5.3423 | 9.1347 |
| H | -15.3731 | 6.3181 | 16.9027 |
| H | -14.2058 | 6.7082 | 15.5794 |
| H | -15.7011 | 7.7099 | 15.7979 |
| H | -23.4226 | -5.8017 | -12.2708 |
| H | -22.2702 | -4.4755 | -11.8479 |
| H | -22.2203 | -5.2339 | -13.4939 |
| H | -14.2289 | -6.6136 | -11.6084 |
| H | -15.4361 | -7.1458 | -10.3742 |
| H | -15.3745 | -7.9569 | -11.9953 |
| H | -25.6826 | -11.4741 | -0.8701 |
| H | -25.7558 | -9.7794 | -0.2479 |
| H | -27.2759 | -10.7547 | -0.4117 |
| H | -29.7240 | -4.2877 | -5.0682 |
| H | -28.1096 | -4.9869 | -5.4817 |
| H | -29.6150 | -5.9683 | -5.7226 |
| H | -20.9824 | 2.4345 | -10.9075 |
| H | -21.0816 | 3.4631 | -9.4254 |
| H | -21.9033 | 3.9893 | -10.9543 |
| H | -17.3340 | 10.8749 | -9.8860 |
| H | -16.4130 | 9.3213 | -9.8192 |
| H | -17.2225 | 9.8338 | -11.3582 |
| H | -10.2214 | -6.8776 | 9.3177 |
| H | -11.5709 | -5.6779 | 9.3070 |
| H | -11.2092 | -6.6353 | 10.8062 |
| H | -19.1848 | -8.9385 | 10.3676 |
| H | -18.2380 | -9.0738 | 8.8346 |
| H | -17.8352 | -10.1343 | 10.2485 |
| H | -8.9273 | 8.2923 | -0.9782 |
| H | -10.0948 | 8.9039 | 0.2443 |
| H | -8.3690 | 9.4644 | 0.2808 |
| H | -8.1137 | 6.8210 | 8.1271 |
| H | -8.3101 | 5.5348 | 6.8726 |
| H | -6.8274 | 6.5789 | 6.8811 |
| H | -23.6748 | 13.0434 | 2.8588 |
| H | -22.3518 | 12.1947 | 3.7501 |
| H | -22.3554 | 14.0041 | 3.6340 |
| H | -14.7446 | 12.7765 | 0.4567 |
| H | -16.0785 | 11.7737 | -0.2353 |
| H | -16.0542 | 13.5667 | -0.5059 |
| H | -5.7022 | -2.3261 | -7.9247 |
| H | -7.4576 | -2.5383 | -8.2754 |
| H | -6.3966 | -1.6348 | -9.4439 |
| H | -10.7737 | 5.2514 | -9.1540 |
| H | -10.6193 | 4.4703 | -7.5317 |
| H | -9.1424 | 5.0213 | -8.4213 |
| H | -24.2076 | 1.8518 | 10.2813 |
| H | -24.6136 | 1.3400 | 8.5968 |
| H | -25.9023 | 1.3754 | 9.8720 |
| H | -26.8676 | -6.5963 | 7.6055 |
| H | -25.1765 | -6.0792 | 7.9771 |
| H | -26.4260 | -6.1165 | 9.2905 |
| H | -18.1833 | -16.1379 | 1.6774 |
| H | -19.2788 | -15.0251 | 0.7675 |
| H | -18.6177 | -16.5093 | -0.0365 |
| H | -15.8915 | -11.0118 | -5.6748 |
| H | -15.5298 | -10.6451 | -3.9433 |
| H | -14.7442 | -12.0637 | -4.7555 |
| H | -33.4678 | -4.6826 | 4.4161 |
| H | -33.6074 | -3.1915 | 5.3762 |
| C | -32.0779 | -4.5339 | 6.0673 |
| H | -24.0683 | -16.2100 | -9.3425 |
| H | -23.5674 | -15.3791 | -10.8207 |
| H | -25.1804 | -15.1006 | -10.1525 |
| H | -25.2313 | -13.1552 | -11.1871 |
| H | -23.6463 | -13.7008 | -11.7828 |
| C | -24.0698 | -11.6343 | -12.1945 |
| H | -25.2377 | -15.8547 | -7.4741 |
| H | -26.1911 | -14.8114 | -8.5536 |
| C | -26.6324 | -14.5422 | -6.4670 |
| H | -22.2545 | -16.5026 | -8.8043 |
| C | -20.1575 | -16.1440 | -8.4968 |
| H | -21.4536 | -15.6927 | -10.1703 |
| H | -34.6186 | -2.5069 | 0.6055 |
| H | -34.2276 | -4.1980 | 0.9936 |
| C | -33.5771 | -3.5452 | -0.9636 |
| H | -34.6020 | -0.1180 | 2.5246 |
| C | -33.5474 | 1.6451 | 3.1539 |
| H | -34.1557 | -0.1196 | 4.2465 |
| H | -35.1276 | -1.9845 | 2.4295 |
| H | -34.7497 | -2.2331 | 4.1387 |
| H | -34.7655 | -3.6073 | 3.0279 |
| H | -7.3179 | -14.3190 | 3.5373 |
| C | -7.1830 | -13.1644 | 1.7278 |
| H | -8.2736 | -14.9766 | 2.1899 |
| H | -8.0874 | -15.4103 | 4.9268 |
| H | -9.1607 | -16.2175 | 3.7759 |
| H | -9.5680 | -16.1894 | 5.4947 |
| H | -11.4675 | -16.4221 | 5.1590 |
| H | -11.3000 | -16.5855 | 3.3961 |
| C | -13.3346 | -16.3496 | 4.0963 |
| H | -8.1829 | -14.1673 | 6.7929 |
| H | -9.6858 | -15.0661 | 7.0949 |
| C | -9.4415 | -13.3309 | 8.3431 |
| H | -19.9947 | -4.9298 | 17.1026 |
| H | -18.2584 | -4.6408 | 17.3582 |
| C | -18.6899 | -6.2957 | 16.0527 |
| H | -20.2360 | -2.9841 | 18.0611 |
| H | -19.4881 | -1.3880 | 18.1874 |
| H | -18.4802 | -2.8407 | 18.2053 |
| H | -21.8244 | -2.2981 | 17.1959 |
| C | -23.0432 | -1.0069 | 15.9817 |
| H | -21.3447 | -0.5875 | 17.2540 |
| H | -16.9125 | -1.8637 | 17.5879 |
| H | -17.7038 | -0.2716 | 17.5684 |
| C | -15.7730 | -0.3355 | 16.5918 |
| H | -0.8524 | 2.5751 | -3.6720 |
| C | -1.6969 | 1.1707 | -5.0720 |
| H | -0.5804 | 0.8741 | -3.2394 |
| H | -0.3055 | 3.3076 | -1.9161 |
| H | -0.0368 | 1.6987 | -1.2424 |
| H | -0.3869 | 3.0543 | -0.1680 |
| H | -0.9833 | 1.8238 | 1.2258 |
| H | -0.6953 | 0.3622 | 0.2547 |
| C | -1.8252 | 0.0251 | 2.0624 |
| H | -1.4546 | 4.9418 | -1.8068 |
| H | -1.7093 | 4.9373 | -0.0450 |
| C | -3.0764 | 6.2121 | -1.1523 |
| H | -12.2811 | 16.4909 | 7.1050 |
| H | -14.0150 | 16.7210 | 7.4304 |
| C | -13.5116 | 16.4618 | 5.3439 |
| H | -13.7965 | 14.6430 | 11.3910 |
| C | -15.6925 | 13.6598 | 11.6311 |
| H | -15.1072 | 15.3569 | 10.4228 |
| H | -10.5361 | 14.1793 | 10.3380 |
| H | -10.2969 | 15.0184 | 8.7889 |
| C | -9.1236 | 13.2308 | 8.9988 |
| H | -12.3052 | 15.5421 | 10.5166 |
| H | -13.4518 | 16.4156 | 9.4927 |
| H | -11.7711 | 16.2348 | 8.9810 |
| H | -12.7699 | -1.1973 | -17.3077 |
| H | -14.4948 | -1.5450 | -17.5694 |
| C | -13.4466 | -3.0051 | -16.3657 |
| H | -12.8195 | 0.6052 | -18.0122 |
| H | -13.7938 | 2.0768 | -18.1157 |
| H | -14.5571 | 0.4952 | -18.3143 |
| H | -11.4502 | 1.5223 | -16.9506 |
| C | -10.5938 | 2.9051 | -15.5443 |
| H | -12.2484 | 3.1111 | -16.9229 |
| H | -16.4710 | 1.4116 | -17.7570 |
| H | -15.4796 | 2.8825 | -17.6266 |
| C | -17.4447 | 3.0770 | -16.7777 |
| H | -23.4914 | 14.5265 | -7.3945 |
| H | -23.9675 | 13.2987 | -8.5897 |
| C | -21.8392 | 13.4223 | -8.2177 |
| H | -25.3046 | 14.8727 | -6.7379 |
| H | -26.8503 | 14.1188 | -6.3286 |
| H | -25.9970 | 13.6417 | -7.8008 |
| H | -24.9634 | 15.3458 | -4.8593 |
| C | -25.0065 | 15.1828 | -2.7147 |
| H | -26.4738 | 14.6625 | -4.2165 |
| H | -27.1185 | 11.7596 | -7.7557 |
| H | -27.8262 | 12.4634 | -6.2835 |
| C | -28.2549 | 10.3733 | -6.5430 |
| H | -19.3281 | -6.5339 | 15.1744 |
| C | -18.8911 | -7.3585 | 17.1316 |
| H | -17.6413 | -6.3308 | 15.6929 |
| H | -19.9319 | -7.3996 | 17.4718 |
| H | -18.2644 | -7.1678 | 18.0101 |
| H | -18.6300 | -8.3544 | 16.7551 |
| H | -15.9092 | 0.4387 | 15.8057 |
| H | -15.0922 | -1.0980 | 16.1625 |
| C | -15.1181 | 0.2947 | 17.8198 |
| H | -15.7343 | 1.0965 | 18.2418 |
| H | -14.1440 | 0.7284 | 17.5667 |
| H | -14.9508 | -0.4433 | 18.6124 |
| H | -23.4952 | -1.8819 | 15.4717 |
| H | -22.9221 | -0.2220 | 15.2039 |
| C | -23.9944 | -0.5066 | 17.0672 |
| H | -24.1515 | -1.2608 | 17.8465 |
| H | -24.9769 | -0.2636 | 16.6464 |
| H | -23.6140 | 0.3975 | 17.5555 |
| H | -19.4261 | -15.3095 | -8.5625 |
| C | -19.6083 | -17.3604 | -9.2403 |
| H | -20.2569 | -16.3859 | -7.4192 |
| H | -19.4339 | -17.1436 | -10.3002 |
| H | -20.2955 | -18.2122 | -9.1869 |
| H | -18.6532 | -17.6846 | -8.8110 |
| H | -13.7899 | -15.8333 | 3.2228 |
| H | -13.8596 | -15.9664 | 4.9943 |
| C | -13.5691 | -17.8541 | 3.9743 |
| H | -13.1160 | -18.2626 | 3.0636 |
| H | -14.6400 | -18.0840 | 3.9375 |
| H | -13.1468 | -18.4003 | 4.8253 |
| C | -27.6186 | -15.6971 | -6.3001 |
| H | -27.1919 | -13.6096 | -6.6837 |
| H | -26.0990 | -14.3701 | -5.5069 |
| H | -28.1766 | -15.8898 | -7.2233 |
| H | -28.3521 | -15.4771 | -5.5159 |
| H | -27.1108 | -16.6276 | -6.0220 |
| H | -31.2007 | -5.0878 | 5.6694 |
| C | -32.9502 | -5.4848 | 6.8855 |
| H | -31.6722 | -3.7450 | 6.7326 |
| H | -33.3177 | -6.3209 | 6.2799 |
| H | -33.8236 | -4.9732 | 7.3052 |
| H | -32.3876 | -5.9102 | 7.7245 |
| C | -34.7371 | 2.4187 | 3.7193 |
| H | -33.3799 | 1.9476 | 2.1005 |
| H | -32.6267 | 1.9259 | 3.7108 |
| H | -35.6649 | 2.1806 | 3.1869 |
| H | -34.5803 | 3.5000 | 3.6330 |
| H | -34.8998 | 2.1948 | 4.7797 |
| C | -29.4348 | 10.4952 | -7.5060 |
| H | -28.6349 | 10.3095 | -5.5030 |
| H | -27.7174 | 9.4201 | -6.7367 |
| H | -30.0004 | 11.4187 | -7.3389 |
| H | -30.1322 | 9.6588 | -7.3802 |
| H | -29.1072 | 10.4938 | -8.5518 |
| H | -25.2477 | 14.4320 | -1.9310 |
| C | -25.7465 | 16.4856 | -2.4160 |
| H | -23.9134 | 15.3584 | -2.6522 |
| H | -26.8329 | 16.3424 | -2.4059 |
| H | -25.5234 | 17.2580 | -3.1606 |
| H | -25.4594 | 16.8850 | -1.4364 |
| H | -16.4915 | 13.2068 | 11.0062 |
| C | -16.3220 | 14.6059 | 12.6524 |
| H | -15.2005 | 12.8202 | 12.1627 |
| H | -16.8762 | 15.4168 | 12.1659 |
| H | -15.5660 | 15.0655 | 13.2988 |
| H | -17.0259 | 14.0721 | 13.3010 |
| H | -8.8931 | 13.2149 | 7.9145 |
| H | -9.2449 | 12.1694 | 9.3084 |
| C | -7.9500 | 13.8489 | 9.7555 |
| H | -7.7880 | 14.8941 | 9.4682 |
| H | -7.0185 | 13.3092 | 9.5456 |
| H | -8.1059 | 13.8253 | 10.8395 |
| H | -3.4206 | 6.3244 | -2.2002 |
| C | -2.2861 | 7.4534 | -0.7421 |
| H | -3.9939 | 6.1431 | -0.5277 |
| H | -1.3722 | 7.5707 | -1.3354 |
| H | -1.9942 | 7.4172 | 0.3136 |
| H | -2.8837 | 8.3619 | -0.8836 |
| H | -2.3797 | -0.8799 | 1.7354 |
| H | -2.5008 | 0.5765 | 2.7461 |
| C | -0.5759 | -0.3947 | 2.8328 |
| H | 0.1508 | -0.9014 | 2.1882 |
| H | -0.8271 | -1.0838 | 3.6465 |
| H | -0.0691 | 0.4756 | 3.2827 |
| H | -10.5060 | -13.3651 | 8.6524 |
| H | -9.1794 | -12.2542 | 8.2499 |
| C | -8.5774 | -13.9731 | 9.4266 |
| H | -8.8216 | -15.0324 | 9.5647 |
| H | -8.7263 | -13.4763 | 10.3927 |
| H | -7.5101 | -13.9095 | 9.1871 |
| C | -24.9037 | -11.8250 | -13.4604 |
| H | -23.0123 | -11.4551 | -12.4758 |
| H | -24.4105 | -10.7204 | -11.6620 |
| H | -24.5900 | -12.7121 | -14.0222 |
| H | -24.8041 | -10.9632 | -14.1302 |
| H | -25.9691 | -11.9404 | -13.2309 |
| H | -7.8553 | -12.6905 | 0.9804 |
| C | -6.1185 | -13.9874 | 1.0041 |
| H | -6.6952 | -12.3354 | 2.2782 |
| H | -6.5656 | -14.7799 | 0.3927 |
| H | -5.4290 | -14.4668 | 1.7082 |
| H | -5.5192 | -13.3582 | 0.3368 |
| H | -2.2922 | 1.9710 | -5.5551 |
| H | -2.3380 | 0.2627 | -5.0674 |
| C | -0.4433 | 0.9024 | -5.8992 |
| H | 0.2098 | 1.7811 | -5.9421 |
| H | -0.7018 | 0.6362 | -6.9304 |
| H | 0.1459 | 0.0758 | -5.4859 |
| H | -14.4407 | 15.9792 | 4.9684 |
| H | -12.6789 | 16.0508 | 4.7390 |
| C | -13.6017 | 17.9704 | 5.1223 |
| H | -14.4512 | 18.4086 | 5.6587 |
| H | -13.7319 | 18.2047 | 4.0595 |
| H | -12.6963 | 18.4852 | 5.4624 |
| C | -21.6470 | 14.2384 | -9.4948 |
| H | -21.5406 | 12.3676 | -8.4045 |
| H | -21.1597 | 13.8057 | -7.4304 |
| H | -22.2584 | 13.8520 | -10.3185 |
| H | -20.6023 | 14.2137 | -9.8245 |
| H | -21.9191 | 15.2897 | -9.3485 |
| C | -34.7798 | -4.2328 | -1.6075 |
| H | -32.6733 | -4.1788 | -1.0996 |
| H | -33.3735 | -2.5916 | -1.4906 |
| H | -34.9778 | -5.2112 | -1.1545 |
| H | -34.6122 | -4.3977 | -2.6781 |
| H | -35.6914 | -3.6332 | -1.5077 |
| C | -13.3535 | -3.9373 | -17.5718 |
| H | -14.2614 | -3.3542 | -15.6938 |
| H | -12.5083 | -3.0686 | -15.7787 |
| H | -14.2855 | -3.9474 | -18.1487 |
| H | -13.1539 | -4.9682 | -17.2574 |
| H | -12.5478 | -3.6407 | -18.2523 |
| H | -10.9178 | 3.5909 | -14.7313 |
| C | -9.6781 | 3.6572 | -16.5084 |
| H | -10.0230 | 2.0899 | -15.0552 |
| H | -10.1871 | 4.5115 | -16.9688 |
| H | -9.3235 | 3.0099 | -17.3184 |
| H | -8.7936 | 4.0440 | -15.9892 |
| C | -18.2208 | 3.3801 | -18.0583 |
| H | -17.1545 | 4.0298 | -16.2902 |
| H | -18.1085 | 2.5490 | -16.0597 |
| H | -17.6033 | 3.9133 | -18.7897 |
| H | -19.0940 | 4.0087 | -17.8488 |
| H | -18.5842 | 2.4646 | -18.5388 |
| C | 8.3381 | 6.9845 | -11.9645 |
| C | 7.7010 | 4.7965 | -13.1519 |
| C | 6.2603 | 6.8987 | -13.4735 |
| C | 7.7776 | 6.3883 | -13.3427 |
| C | 8.6246 | 5.7549 | -11.1209 |
| C | 8.1621 | 4.5673 | -11.7283 |
| C | 8.1625 | 3.3733 | -11.0101 |
| C | 8.7004 | 3.3366 | -9.7178 |
| N | 9.7469 | 4.3998 | -7.8175 |
| C | 9.2341 | 4.5049 | -9.1487 |
| C | 9.1819 | 5.7287 | -9.8522 |
| C | 6.2326 | 4.4748 | -13.3346 |
| C | 5.4516 | 5.6278 | -13.5577 |
| C | 4.0856 | 5.5204 | -13.7872 |
| C | 3.4762 | 4.2625 | -13.7627 |
| N | 3.5967 | 1.8487 | -13.5290 |
| C | 4.2520 | 3.1184 | -13.5019 |
| C | 5.6422 | 3.2204 | -13.3013 |
| C | 5.9862 | 7.5919 | -12.1524 |
| C | 7.1370 | 7.6822 | -11.3424 |
| C | 7.0525 | 8.2612 | -10.0759 |
| C | 5.8280 | 8.7607 | -9.6155 |
| N | 3.4620 | 9.1934 | -9.8979 |
| C | 4.6764 | 8.6361 | -10.4141 |
| C | 4.7538 | 8.0417 | -11.6909 |
| C | 16.9941 | 3.2081 | 1.4763 |
| C | 16.5606 | 4.9867 | 3.2814 |
| C | 16.6249 | 2.4972 | 3.9210 |
| C | 17.3285 | 3.6088 | 2.9910 |
| C | 16.0726 | 4.3170 | 0.9944 |
| C | 15.8967 | 5.3308 | 1.9628 |
| C | 15.1193 | 6.4491 | 1.6736 |
| C | 14.4595 | 6.5399 | 0.4442 |
| N | 13.9169 | 5.6212 | -1.7417 |
| C | 14.5714 | 5.4876 | -0.4804 |
| C | 15.3870 | 4.3711 | -0.2117 |
| C | 15.4988 | 4.6058 | 4.3068 |
| C | 15.6103 | 3.2612 | 4.7145 |
| C | 14.7798 | 2.7351 | 5.6918 |
| C | 13.7595 | 3.5243 | 6.2276 |
| N | 12.4954 | 5.5973 | 6.2991 |
| C | 13.5762 | 4.8342 | 5.7485 |
| C | 14.4613 | 5.3920 | 4.7954 |
| C | 15.9181 | 1.5827 | 2.9333 |
| C | 16.1951 | 1.9295 | 1.5945 |
| C | 15.6764 | 1.1773 | 0.5444 |
| C | 14.8177 | 0.1099 | 0.8163 |
| N | 13.5717 | -1.2866 | 2.3009 |
| C | 14.4678 | -0.1835 | 2.1481 |
| C | 15.0333 | 0.5467 | 3.2180 |
| C | 0.8536 | 11.3874 | 11.0059 |
| C | 3.4169 | 11.4972 | 10.8263 |
| C | 1.9526 | 12.7577 | 9.1306 |
| C | 2.0792 | 12.3716 | 10.6849 |
| C | 1.5071 | 10.0309 | 11.2015 |
| C | 2.9165 | 10.1018 | 11.1565 |
| C | 3.6777 | 8.9437 | 11.2995 |
| C | 3.0455 | 7.7138 | 11.5055 |
| N | 1.0797 | 6.3397 | 11.6804 |
| C | 1.6404 | 7.6431 | 11.5406 |
| C | 0.8652 | 8.8081 | 11.3673 |
| C | 3.9811 | 11.4512 | 9.4133 |
| C | 3.1147 | 12.0487 | 8.4739 |
| C | 3.3683 | 11.9306 | 7.1109 |
| C | 4.5209 | 11.2731 | 6.6729 |
| N | 6.5856 | 10.1053 | 7.0727 |
| C | 5.4124 | 10.7246 | 7.6088 |
| C | 5.1441 | 10.8178 | 8.9923 |
| C | 0.6286 | 12.1550 | 8.7092 |
| C | 0.0236 | 11.4039 | 9.7398 |
| C | -1.1997 | 10.7769 | 9.5220 |
| C | -1.8292 | 10.8879 | 8.2780 |
| N | -1.9483 | 11.7819 | 6.0296 |
| C | -1.2212 | 11.6317 | 7.2510 |
| C | 0.0163 | 12.2759 | 7.4701 |
| C | 7.0995 | 9.0657 | 7.6492 |
| C | 8.2878 | 8.3962 | 7.0817 |
| C | 8.6142 | 8.4613 | 5.7201 |
| C | 9.7043 | 7.7277 | 5.2328 |
| C | 10.5087 | 6.9370 | 6.0941 |
| C | 10.1984 | 6.8997 | 7.4559 |
| C | 9.0981 | 7.6159 | 7.9358 |
| C | 11.6280 | 6.1652 | 5.5199 |
| C | -7.7927 | 14.2662 | -3.8264 |
| C | -6.3368 | 14.2738 | -5.9452 |
| C | -8.4284 | 12.7818 | -5.8195 |
| C | -7.8535 | 14.2288 | -5.4316 |
| C | -6.3110 | 14.1616 | -3.5138 |
| C | -5.5111 | 14.2980 | -4.6688 |
| C | -4.1225 | 14.3093 | -4.5580 |
| C | -3.5208 | 14.1008 | -3.3144 |
| N | -3.6229 | 13.5939 | -0.9608 |
| C | -4.3193 | 13.8558 | -2.1837 |
| C | -5.7284 | 13.9128 | -2.2771 |
| C | -6.1159 | 12.9024 | -6.5670 |
| C | -7.2381 | 12.0562 | -6.4168 |
| C | -7.1496 | 10.7111 | -6.7700 |
| C | -5.9620 | 10.2075 | -7.3172 |
| N | -3.6780 | 10.4979 | -8.0886 |
| C | -4.8544 | 11.0568 | -7.4868 |
| C | -4.9326 | 12.4154 | -7.1091 |
| C | -8.8421 | 12.1790 | -4.4875 |
| C | -8.5251 | 13.0199 | -3.3996 |
| C | -8.8592 | 12.6546 | -2.0996 |
| C | -9.4804 | 11.4222 | -1.8646 |
| N | -10.2781 | 9.2711 | -2.6210 |
| C | -9.7657 | 10.5653 | -2.9462 |
| C | -9.4588 | 10.9541 | -4.2683 |
| C | -4.1147 | 12.7882 | -0.0721 |
| C | -3.3755 | 12.5226 | 1.1776 |
| C | -1.9860 | 12.6571 | 1.2712 |
| C | -1.3440 | 12.3708 | 2.4796 |
| C | -2.0706 | 11.9750 | 3.6310 |
| C | -3.4585 | 11.8363 | 3.5352 |
| C | -4.0995 | 12.0971 | 2.3184 |
| C | 2.3342 | 8.5517 | -9.9527 |
| C | 1.1088 | 9.1657 | -9.3933 |
| C | 1.1458 | 10.3409 | -8.6317 |
| C | -0.0341 | 10.8577 | -8.0867 |
| C | -1.2880 | 10.2237 | -8.2887 |
| C | -1.3299 | 9.0631 | -9.0678 |
| C | -0.1467 | 8.5419 | -9.6090 |
| C | 12.3975 | 4.9122 | -3.4559 |
| C | -2.4926 | 10.7987 | -7.6499 |
| C | -1.3327 | 11.7406 | 4.8888 |
| C | 13.0231 | 4.7682 | -2.1251 |
| C | -6.3138 | -4.1834 | 14.3031 |
| C | -4.7093 | -6.1741 | 14.0131 |
| C | -3.8814 | -4.0003 | 15.1156 |
| C | -5.1322 | -4.9986 | 15.0233 |
| C | -6.5551 | -4.9441 | 13.0133 |
| C | -5.6813 | -6.0417 | 12.8607 |
| C | -5.7570 | -6.8458 | 11.7280 |
| C | -6.6800 | -6.5459 | 10.7207 |
| N | -8.4439 | -5.1844 | 9.7866 |
| C | -7.5235 | -5.4290 | 10.8578 |
| C | -7.4776 | -4.6342 | 12.0226 |
| C | -3.3019 | -5.7997 | 13.5754 |
| C | -2.8264 | -4.6445 | 14.2369 |
| C | -1.5357 | -4.1820 | 13.9988 |
| C | -0.7171 | -4.8383 | 13.0769 |
| N | -0.2922 | -6.5870 | 11.4752 |
| C | -1.2083 | -5.9505 | 12.3729 |
| C | -2.5127 | -6.4377 | 12.6219 |
| C | -4.3682 | -2.7262 | 14.4454 |
| C | -5.7028 | -2.8303 | 13.9984 |
| C | -6.2921 | -1.7747 | 13.3050 |
| C | -5.5744 | -0.5937 | 13.0871 |
| N | -3.5848 | 0.7634 | 13.3868 |
| C | -4.2453 | -0.4956 | 13.5327 |
| C | -3.6276 | -1.5764 | 14.1951 |
| C | -2.4867 | 0.9097 | 12.7159 |
| C | -1.8681 | 2.2504 | 12.6238 |
| C | 3.1786 | -9.6665 | 6.8452 |
| C | 2.1727 | -9.0517 | 7.7384 |
| C | 9.6719 | -6.3087 | 3.5667 |
| C | 10.3809 | -5.0122 | 3.5245 |
| C | 0.7963 | -9.2400 | 7.5632 |
| C | -0.1104 | -8.6007 | 8.4179 |
| C | 0.3290 | -7.7648 | 9.4768 |
| C | 1.7054 | -7.5910 | 9.6610 |
| C | 2.6108 | -8.2217 | 8.8014 |
| C | -0.6680 | -7.0995 | 10.3435 |
| C | -1.8769 | -9.7295 | -11.0850 |
| C | -4.3008 | -9.2915 | -10.3582 |
| C | -3.1374 | -7.6806 | -11.9903 |
| C | -3.3068 | -9.2360 | -11.6131 |
| C | -2.0712 | -9.8869 | -9.5841 |
| C | -3.4065 | -9.6573 | -9.1888 |
| C | -3.7552 | -9.6846 | -7.8397 |
| C | -2.7795 | -9.9539 | -6.8767 |
| N | -0.5223 | -10.4161 | -6.2181 |
| C | -1.4448 | -10.1563 | -7.2712 |
| C | -1.0863 | -10.1239 | -8.6328 |
| C | -4.7488 | -7.8538 | -10.1787 |
| C | -4.0636 | -6.9627 | -11.0321 |
| C | -4.2925 | -5.5933 | -10.9330 |
| C | -5.2016 | -5.1005 | -9.9884 |
| N | -6.8397 | -5.4345 | -8.2253 |
| C | -5.8863 | -5.9896 | -9.1438 |
| C | -5.6684 | -7.3822 | -9.2543 |
| C | -1.6727 | -7.3922 | -11.7174 |
| C | -0.9652 | -8.5484 | -11.3246 |
| C | 0.4118 | -8.5074 | -11.1282 |
| C | 1.0992 | -7.2994 | -11.2799 |
| N | 1.0939 | -4.8997 | -11.6383 |
| C | 0.3830 | -6.1368 | -11.6121 |
| C | -1.0114 | -6.1777 | -11.8271 |
| C | 0.7117 | -10.0280 | -6.2850 |
| C | 0.9923 | -4.0746 | -12.6297 |
| C | 1.6115 | -10.2931 | -5.1450 |
| C | 1.7295 | -2.7977 | -12.5906 |
| C | 1.1121 | -10.5982 | -3.8746 |
| C | 1.9939 | -10.7868 | -2.8088 |
| C | 3.3973 | -10.6788 | -2.9777 |
| C | 3.8987 | -10.3992 | -4.2551 |
| C | 3.0135 | -10.2054 | -5.3224 |
| C | 2.6045 | -2.4702 | -11.5482 |
| C | 3.2665 | -1.2356 | -11.5506 |
| C | 3.0606 | -0.2957 | -12.5981 |
| C | 2.1921 | -0.6300 | -13.6421 |
| C | 1.5395 | -1.8626 | -13.6379 |
| C | -16.1772 | -0.6668 | 0.2479 |
| C | -15.9800 | -2.4494 | -1.5874 |
| C | -15.8776 | -3.1429 | 0.8851 |
| C | -16.6178 | -2.1636 | -0.1448 |
| C | -15.4904 | -0.1432 | -1.0034 |
| C | -15.4295 | -1.1152 | -2.0232 |
| C | -14.8737 | -0.8123 | -3.2606 |
| C | -14.3447 | 0.4601 | -3.4905 |
| N | -13.8656 | 2.7179 | -2.7927 |
| C | -14.3705 | 1.4210 | -2.4642 |
| C | -14.9561 | 1.1244 | -1.2129 |
| C | -14.8353 | -3.4048 | -1.3005 |
| C | -14.8182 | -3.8415 | 0.0422 |
| C | -13.8205 | -4.7158 | 0.4720 |
| C | -12.8256 | -5.1402 | -0.4197 |
| N | -11.7785 | -5.1013 | -2.6217 |
| C | -12.8154 | -4.6530 | -1.7370 |
| C | -13.8331 | -3.7847 | -2.1851 |
| C | -15.1380 | -2.2011 | 1.8221 |
| C | -15.2092 | -0.8552 | 1.3986 |
| C | -14.4281 | 0.1127 | 2.0256 |
| C | -13.6210 | -0.2399 | 3.1150 |
| N | -12.7969 | -1.8457 | 4.7241 |
| C | -13.5964 | -1.5712 | 3.5682 |
| C | -14.3637 | -2.5593 | 2.9186 |
| C | -10.0794 | -4.7215 | -4.2896 |
| C | -13.0430 | 3.3490 | -2.0153 |
| C | -12.1310 | -2.9535 | 4.8367 |
| C | -12.5581 | 4.6953 | -2.3965 |
| C | -11.2855 | -3.1924 | 6.0277 |
| C | -12.7924 | 5.2501 | -3.6611 |
| C | -12.3025 | 6.5273 | -3.9670 |
| C | -11.5912 | 7.2969 | -3.0146 |
| C | -11.3581 | 6.7411 | -1.7514 |
| C | -11.8215 | 5.4552 | -1.4538 |
| C | -9.4552 | -5.9651 | -4.1560 |
| C | -8.4713 | -6.3488 | -5.0729 |
| C | -8.0871 | -5.5163 | -6.1520 |
| C | -8.7111 | -4.2715 | -6.2879 |
| C | -9.6876 | -3.8765 | -5.3644 |
| C | -11.1236 | -2.2422 | 7.0426 |
| C | -10.2838 | -2.5194 | 8.1307 |
| C | -9.5898 | -3.7518 | 8.2328 |
| C | -9.7694 | -4.7079 | 7.2306 |
| C | -10.6014 | -4.4289 | 6.1464 |
| C | -7.0538 | -6.0116 | -7.0798 |
| C | 4.2561 | -10.8437 | -1.7895 |
| C | -8.6632 | -3.9794 | 9.3608 |
| C | 10.6162 | -4.2399 | 4.6659 |
| C | 11.3271 | -3.0396 | 4.5570 |
| C | 11.8717 | -2.6115 | 3.3174 |
| C | 11.6076 | -3.3709 | 2.1682 |
| C | 10.8553 | -4.5472 | 2.2716 |
| C | -0.7239 | 2.4445 | 11.8087 |
| C | -0.1200 | 3.7024 | 11.6796 |
| C | -0.6426 | 4.8037 | 12.3674 |
| C | -1.7813 | 4.6050 | 13.1889 |
| C | -2.3850 | 3.3519 | 13.3162 |
| C | -11.1249 | 8.6485 | -3.3856 |
| C | -0.0654 | 6.1617 | 12.2585 |
| C | 3.7529 | 1.0039 | -12.5563 |
| C | 12.7241 | -1.3996 | 3.2737 |
| C | 12.6866 | 5.9903 | -4.2968 |
| C | 12.1028 | 6.0631 | -5.5648 |
| C | 11.2424 | 5.0487 | -6.0454 |
| C | 10.9324 | 3.9806 | -5.1956 |
| C | 11.4896 | 3.9234 | -3.9123 |
| C | 10.7371 | 5.1447 | -7.4267 |
| C | 8.8096 | -11.7012 | 2.7175 |
| C | 7.5148 | -12.3212 | 4.8527 |
| C | 6.6657 | -13.1090 | 2.5622 |
| C | 7.9945 | -12.8870 | 3.4319 |
| C | 8.7856 | -10.5704 | 3.7330 |
| C | 8.1591 | -10.9479 | 4.9428 |
| C | 8.1121 | -10.0622 | 6.0177 |
| C | 8.6428 | -8.7753 | 5.8871 |
| N | 9.7949 | -7.0723 | 4.6073 |
| C | 9.2070 | -8.3786 | 4.6643 |
| C | 9.2848 | -9.2802 | 3.5795 |
| C | 6.0183 | -12.1163 | 4.6881 |
| C | 5.5514 | -12.5451 | 3.4280 |
| C | 4.2167 | -12.3472 | 3.0747 |
| C | 3.3390 | -11.7334 | 3.9758 |
| N | 2.8587 | -10.6778 | 6.0980 |
| C | 3.8135 | -11.2871 | 5.2220 |
| C | 5.1634 | -11.4824 | 5.5818 |
| C | 6.8605 | -12.1911 | 1.3585 |
| C | 8.0058 | -11.3783 | 1.4910 |
| C | 8.3032 | -10.4059 | 0.5445 |
| C | 7.4679 | -10.2370 | -0.5639 |
| N | 5.5480 | -10.9015 | -1.8917 |
| C | 6.3362 | -11.0589 | -0.7098 |
| C | 6.0338 | -12.0495 | 0.2519 |
| C | -11.1235 | -4.2514 | -3.3529 |
| O | 11.5426 | -2.1565 | 5.5976 |
| O | 10.5145 | -5.3914 | 1.2275 |
| C | 11.3087 | -2.6292 | 6.9504 |
| C | 10.8535 | -5.0232 | -0.1329 |
| O | -12.4565 | 7.1593 | -5.1906 |
| C | -13.0634 | 6.3941 | -6.2570 |
| O | -11.6325 | 4.8052 | -0.2549 |
| C | -10.7793 | 5.4435 | 0.7240 |
| O | -2.2132 | 5.7572 | 13.8206 |
| O | -0.2871 | 1.2930 | 11.1834 |
| C | -3.3568 | 5.6498 | 14.7026 |
| C | 0.8342 | 1.4047 | 10.2725 |
| O | 4.1523 | -0.8123 | -10.5789 |
| O | 0.6575 | -2.2933 | -14.6132 |
| C | 0.4144 | -1.4101 | -15.7349 |
| C | 4.4697 | -1.7257 | -9.4921 |
| O | -7.7886 | -7.5522 | -5.0480 |
| C | -8.0639 | -8.4576 | -3.9588 |
| O | -10.3759 | -2.6816 | -5.4023 |
| C | -9.9214 | -1.6680 | -6.3352 |
| O | -0.0715 | 7.4066 | -10.3919 |
| C | -1.2852 | 6.6381 | -10.5814 |
| O | -0.1152 | 12.0065 | -7.3203 |
| C | 1.1229 | 12.6867 | -7.0003 |
| O | 3.9867 | -8.1175 | 8.8881 |
| O | -1.4859 | -8.7137 | 8.3384 |
| C | 4.5300 | -7.2504 | 9.9134 |
| C | -2.0344 | -9.4751 | 7.2348 |
| O | 10.1177 | 7.7058 | 3.9189 |
| C | 9.2128 | 8.2405 | 2.9209 |
| O | 8.6913 | 7.6558 | 9.2563 |
| C | 9.3693 | 6.7803 | 10.1807 |
| O | 0.0244 | 12.4304 | 2.6881 |
| O | -5.4543 | 11.9894 | 2.0923 |
| C | -6.2906 | 11.5068 | 3.1725 |
| C | 0.8518 | 12.9053 | 1.5986 |
| O | 11.2451 | 2.9293 | -2.9888 |
| O | 12.3049 | 7.0977 | -6.4611 |
| C | 10.2262 | 1.9472 | -3.3054 |
| C | 13.0552 | 8.2315 | -5.9691 |
| O | -10.0375 | -1.6586 | 9.1831 |
| C | -10.7151 | -0.3774 | 9.1747 |
| O | -10.8125 | -5.2834 | 5.0896 |
| C | -10.3557 | -6.6495 | 5.2441 |
| O | 1.6146 | -11.0956 | -1.5148 |
| C | 0.1970 | -11.1327 | -1.2215 |
| O | 3.3812 | -9.9010 | -6.6169 |
| C | 4.7976 | -9.8472 | -6.9240 |
| C | 9.5620 | 7.8915 | -12.1652 |
| C | 8.5822 | 4.0401 | -14.1732 |
| C | 6.0387 | 7.8272 | -14.6796 |
| C | 8.6247 | 6.7723 | -14.5475 |
| H | 7.7316 | 2.4684 | -11.4357 |
| H | 8.7097 | 2.4054 | -9.1534 |
| H | 9.5583 | 6.6505 | -9.4016 |
| H | 3.4923 | 6.4165 | -13.9918 |
| H | 2.4069 | 4.1632 | -13.9407 |
| H | 6.2466 | 2.3245 | -13.1463 |
| H | 7.9239 | 8.3123 | -9.4257 |
| H | 5.7571 | 9.2283 | -8.6345 |
| H | 3.8563 | 7.9318 | -12.2947 |
| C | 18.2575 | 3.0509 | 0.6078 |
| C | 17.5043 | 6.0985 | 3.7861 |
| C | 17.5983 | 1.7480 | 4.8274 |
| C | 18.8271 | 3.7254 | 3.2414 |
| H | 15.0229 | 7.2562 | 2.4067 |
| H | 13.8509 | 7.4109 | 0.2075 |
| H | 15.4624 | 3.5689 | -0.9408 |
| H | 14.9235 | 1.7079 | 6.0360 |
| H | 13.0962 | 3.1267 | 6.9944 |
| H | 14.3144 | 6.4123 | 4.4487 |
| H | 15.9413 | 1.4218 | -0.4891 |
| H | 14.4056 | -0.4893 | 0.0046 |
| H | 14.7729 | 0.3029 | 4.2448 |
| C | 0.0485 | 11.8367 | 12.2442 |
| C | 4.3937 | 12.0782 | 11.8625 |
| C | 1.9994 | 14.2820 | 8.8880 |
| C | 2.0741 | 13.6024 | 11.5809 |
| H | 4.7634 | 8.9781 | 11.2313 |
| H | 3.6334 | 6.8036 | 11.6214 |
| H | -0.2192 | 8.7394 | 11.3281 |
| H | 2.6694 | 12.3320 | 6.3788 |
| H | 4.7268 | 11.1736 | 5.6075 |
| H | 5.8409 | 10.4040 | 9.7251 |
| H | -1.6754 | 10.2085 | 10.3265 |
| H | -2.7875 | 10.4025 | 8.0986 |
| H | 0.4740 | 12.8862 | 6.6875 |
| H | 6.7181 | 8.5761 | 8.5656 |
| H | 8.0090 | 9.0850 | 5.0579 |
| H | 10.8246 | 6.2984 | 8.1196 |
| H | 11.6288 | 6.1262 | 4.4107 |
| C | -8.4108 | 15.5380 | -3.2196 |
| C | -6.0720 | 15.4561 | -6.8908 |
| C | -9.6145 | 12.8634 | -6.8031 |
| C | -8.7092 | 15.3523 | -5.9996 |
| H | -3.5022 | 14.4749 | -5.4426 |
| H | -2.4360 | 14.1038 | -3.2174 |
| H | -6.3433 | 13.7552 | -1.3936 |
| H | -7.9886 | 10.0369 | -6.6074 |
| H | -5.8870 | 9.1587 | -7.6013 |
| H | -4.0695 | 13.0658 | -7.2384 |
| H | -8.6384 | 13.3308 | -1.2673 |
| H | -9.7407 | 11.1229 | -0.8502 |
| H | -9.6957 | 10.3012 | -5.1110 |
| H | -5.0769 | 12.2439 | -0.1429 |
| H | -1.4324 | 12.9947 | 0.3933 |
| H | -4.0216 | 11.5386 | 4.4228 |
| H | 2.1802 | 7.5420 | -10.3809 |
| H | 2.1093 | 10.8338 | -8.4789 |
| H | -2.2965 | 8.5853 | -9.2429 |
| H | -2.2660 | 11.4728 | -6.7995 |
| H | -0.2527 | 11.5454 | 4.7425 |
| H | 12.6544 | 3.9040 | -1.5403 |
| C | -7.5848 | -4.0955 | 15.1704 |
| C | -4.7743 | -7.5690 | 14.6690 |
| C | -3.4116 | -3.7845 | 16.5683 |
| C | -5.5474 | -5.5393 | 16.3850 |
| H | -5.1020 | -7.7163 | 11.6287 |
| H | -6.7418 | -7.1657 | 9.8276 |
| H | -8.1698 | -3.8001 | 12.1564 |
| H | -1.1581 | -3.3083 | 14.5386 |
| H | 0.2973 | -4.4878 | 12.8925 |
| H | -2.8855 | -7.2989 | 12.0728 |
| H | -7.3067 | -1.8581 | 12.9193 |
| H | -6.0372 | 0.2449 | 12.5704 |
| H | -2.5874 | -1.4984 | 14.5002 |
| H | -1.9449 | 0.1147 | 12.1680 |
| H | 4.1724 | -9.1796 | 6.8906 |
| H | 9.0970 | -6.5384 | 2.6481 |
| H | 0.4570 | -9.8940 | 6.7568 |
| H | 2.0457 | -6.9616 | 10.4867 |
| H | -1.6955 | -7.0927 | 9.9297 |
| C | -1.3963 | -11.0156 | -11.7785 |
| C | -5.4702 | -10.2690 | -10.5701 |
| C | -3.5152 | -7.3878 | -13.4577 |
| C | -3.8117 | -10.0580 | -12.7897 |
| H | -4.7750 | -9.4755 | -7.5223 |
| H | -3.0399 | -9.9857 | -5.8197 |
| H | -0.0489 | -10.2658 | -8.9256 |
| H | -3.7724 | -4.9008 | -11.5999 |
| H | -5.3793 | -4.0305 | -9.9082 |
| H | -6.2279 | -8.0880 | -8.6339 |
| H | 0.9548 | -9.4198 | -10.8624 |
| H | 2.1754 | -7.2512 | -11.1257 |
| H | -1.5506 | -5.2618 | -12.0539 |
| H | 1.1880 | -9.4861 | -7.1229 |
| H | 0.3849 | -4.2264 | -13.5437 |
| H | 0.0310 | -10.6882 | -3.7443 |
| H | 4.9819 | -10.3334 | -4.3924 |
| H | 2.7529 | -3.1916 | -10.7393 |
| H | 2.0410 | 0.0944 | -14.4459 |
| C | -17.3792 | 0.2169 | 0.6481 |
| C | -16.9956 | -3.0269 | -2.5902 |
| C | -16.8566 | -4.0895 | 1.6026 |
| C | -18.1298 | -2.3345 | -0.1393 |
| H | -14.8617 | -1.5651 | -4.0547 |
| H | -13.9109 | 0.7109 | -4.4571 |
| H | -15.0178 | 1.8871 | -0.4339 |
| H | -13.7856 | -5.0586 | 1.5045 |
| H | -12.0478 | -5.8271 | -0.0898 |
| H | -13.8158 | -3.4082 | -3.2054 |
| H | -14.4258 | 1.1418 | 1.6702 |
| H | -13.0114 | 0.5122 | 3.6139 |
| H | -14.3592 | -3.5931 | 3.2743 |
| H | -12.6327 | 2.9750 | -1.0581 |
| H | -12.1119 | -3.7778 | 4.0973 |
| H | -13.3683 | 4.6698 | -4.3878 |
| H | -10.8049 | 7.3254 | -1.0126 |
| H | -9.7589 | -6.6185 | -3.3352 |
| H | -8.4261 | -3.6267 | -7.1284 |
| H | -11.6593 | -1.2919 | 6.9612 |
| H | -9.2509 | -5.6643 | 7.3151 |
| H | -6.5149 | -6.9058 | -6.7018 |
| H | 3.6892 | -10.8955 | -0.8381 |
| H | -8.2028 | -3.0514 | 9.7528 |
| H | 10.2506 | -4.5964 | 5.6298 |
| H | 12.0229 | -3.0417 | 1.2128 |
| H | 0.7625 | 3.8491 | 11.0507 |
| H | -3.2654 | 3.2044 | 13.9469 |
| H | -11.5658 | 9.0382 | -4.3234 |
| H | -0.6881 | 6.9499 | 12.7208 |
| H | 4.3664 | 1.1665 | -11.6467 |
| H | 12.5724 | -0.6958 | 4.1121 |
| H | 13.3819 | 6.7569 | -3.9429 |
| H | 10.2542 | 3.2041 | -5.5527 |
| H | 11.2757 | 5.8872 | -8.0487 |
| C | 10.2396 | -12.1145 | 2.3233 |
| C | 7.8801 | -13.2608 | 6.0161 |
| C | 6.4520 | -14.5901 | 2.1816 |
| C | 8.8262 | -14.1514 | 3.5555 |
| H | 7.6631 | -10.3740 | 6.9668 |
| H | 8.6096 | -8.0779 | 6.7224 |
| H | 9.7358 | -8.9616 | 2.6429 |
| H | 3.8486 | -12.6462 | 2.0954 |
| H | 2.2929 | -11.5844 | 3.7119 |
| H | 5.5257 | -11.1269 | 6.5434 |
| H | 9.1931 | -9.7823 | 0.6644 |
| H | 7.6833 | -9.4724 | -1.3086 |
| H | 5.1660 | -12.7038 | 0.1215 |
| H | -11.2745 | -3.1529 | -3.3665 |
| H | 11.6263 | -1.7630 | 7.5520 |
| H | 10.2463 | -2.8305 | 7.1089 |
| H | 11.9320 | -3.5011 | 7.1638 |
| H | 10.5376 | -5.9188 | -0.6846 |
| H | 10.2705 | -4.1503 | -0.4391 |
| H | 11.9306 | -4.8614 | -0.2412 |
| H | -13.0110 | 7.0934 | -7.1013 |
| H | -12.4681 | 5.4993 | -6.4373 |
| H | -14.1042 | 6.1518 | -6.0148 |
| H | -10.6942 | 4.6599 | 1.4878 |
| H | -9.7976 | 5.6769 | 0.2930 |
| H | -11.2726 | 6.3340 | 1.1222 |
| H | -3.4575 | 6.6818 | 15.0619 |
| H | -4.2428 | 5.3393 | 14.1408 |
| H | -3.1382 | 4.9661 | 15.5286 |
| H | 0.9329 | 0.3707 | 9.9167 |
| H | 0.5914 | 2.0824 | 9.4483 |
| H | 1.7313 | 1.7230 | 10.8117 |
| H | -0.2910 | -2.0004 | -16.3335 |
| H | -0.0447 | -0.4779 | -15.3918 |
| H | 1.3422 | -1.2284 | -16.2857 |
| H | 5.2236 | -1.1567 | -8.9262 |
| H | 3.5809 | -1.9172 | -8.8864 |
| H | 4.9125 | -2.6477 | -9.8838 |
| H | -7.5424 | -9.3671 | -4.2842 |
| H | -7.6207 | -8.0527 | -3.0376 |
| H | -9.1367 | -8.6433 | -3.8528 |
| H | -10.5429 | -0.8088 | -6.0496 |
| H | -8.8589 | -1.4467 | -6.1852 |
| H | -10.1360 | -1.9805 | -7.3616 |
| H | -0.9238 | 5.8023 | -11.1948 |
| H | -1.6665 | 6.2822 | -9.6195 |
| H | -2.0288 | 7.2311 | -11.1222 |
| H | 0.7644 | 13.5233 | -6.3869 |
| H | 1.7816 | 12.0301 | -6.4243 |
| H | 1.6046 | 13.0477 | -7.9142 |
| H | 5.6091 | -7.3525 | 9.7415 |
| H | 4.2022 | -6.2189 | 9.7537 |
| H | 4.2538 | -7.6175 | 10.9067 |
| H | -3.1133 | -9.3706 | 7.4082 |
| H | -1.7452 | -9.0262 | 6.2800 |
| H | -1.7242 | -10.5221 | 7.3053 |
| H | 9.7385 | 7.9919 | 1.9883 |
| H | 8.2435 | 7.7365 | 2.9693 |
| H | 9.1281 | 9.3293 | 3.0418 |
| H | 8.9590 | 7.1084 | 11.1446 |
| H | 9.0903 | 5.7386 | 9.9622 |
| H | 10.4533 | 6.9295 | 10.1500 |
| H | -7.2872 | 11.5206 | 2.7045 |
| H | -6.0069 | 10.4890 | 3.4525 |
| H | -6.2529 | 12.1952 | 4.0207 |
| H | 1.8525 | 12.8746 | 2.0494 |
| H | 0.7849 | 12.2231 | 0.7460 |
| H | 0.5736 | 13.9280 | 1.3262 |
| H | 10.1541 | 1.3920 | -2.3614 |
| H | 9.2712 | 2.4336 | -3.5473 |
| H | 10.5713 | 1.3021 | -4.1177 |
| H | 13.0020 | 8.9278 | -6.8143 |
| H | 12.5558 | 8.6296 | -5.0848 |
| H | 14.0921 | 7.9449 | -5.7624 |
| H | -10.3475 | 0.0734 | 10.1056 |
| H | -10.4090 | 0.2101 | 8.3041 |
| H | -11.7998 | -0.5209 | 9.2100 |
| H | -10.8224 | -7.1442 | 4.3791 |
| H | -9.2601 | -6.6874 | 5.1574 |
| H | -10.7147 | -7.0847 | 6.1789 |
| H | 0.1987 | -11.3683 | -0.1493 |
| H | -0.2562 | -10.1546 | -1.4078 |
| H | -0.2886 | -11.9256 | -1.7982 |
| H | 4.7850 | -9.5519 | -7.9804 |
| H | 5.2978 | -9.0845 | -6.3169 |
| H | 5.2519 | -10.8338 | -6.7920 |
| H | -17.4985 | -4.5792 | 0.8399 |
| H | -17.5322 | -3.4731 | 2.2322 |
| C | -16.2443 | -5.1936 | 2.4744 |
| H | -3.9118 | -11.1223 | -12.5311 |
| H | -3.1301 | -9.9977 | -13.6491 |
| H | -4.7974 | -9.7169 | -13.1322 |
| H | -4.5962 | -7.6072 | -13.5842 |
| H | -2.9717 | -8.0976 | -14.1166 |
| C | -3.2361 | -5.9682 | -13.9645 |
| H | -5.0660 | -11.2358 | -10.9397 |
| H | -6.1141 | -9.8691 | -11.3806 |
| C | -6.3420 | -10.5620 | -9.3439 |
| H | -2.2277 | -11.7532 | -11.7864 |
| C | -0.1688 | -11.6945 | -11.1620 |
| H | -1.1809 | -10.7779 | -12.8404 |
| H | -17.7349 | -2.2368 | -2.8333 |
| H | -17.5640 | -3.8459 | -2.0989 |
| C | -16.4036 | -3.5738 | -3.8934 |
| H | -18.0110 | 0.3690 | -0.2518 |
| C | -17.0510 | 1.5846 | 1.2605 |
| H | -18.0022 | -0.3409 | 1.3787 |
| H | -18.6150 | -1.6674 | -0.8638 |
| H | -18.5623 | -2.1128 | 0.8460 |
| H | -18.4261 | -3.3617 | -0.3968 |
| H | 10.7520 | -12.5559 | 3.2067 |
| C | 11.1197 | -10.9922 | 1.7705 |
| H | 10.1703 | -12.9263 | 1.5708 |
| H | 9.7454 | -13.9770 | 4.1338 |
| H | 9.1287 | -14.5403 | 2.5758 |
| H | 8.2755 | -14.9552 | 4.0643 |
| H | 6.5814 | -15.2085 | 3.0952 |
| H | 7.2632 | -14.8858 | 1.4846 |
| C | 5.1025 | -14.9716 | 1.5561 |
| H | 8.9858 | -13.2626 | 6.1238 |
| H | 7.5923 | -14.2967 | 5.7391 |
| C | 7.2492 | -12.9520 | 7.3807 |
| H | -5.8355 | -7.7846 | 14.9126 |
| H | -4.2304 | -7.5350 | 15.6373 |
| C | -4.2063 | -8.7379 | 13.8558 |
| H | -6.4134 | -6.2104 | 16.3109 |
| H | -5.8238 | -4.7310 | 17.0773 |
| H | -4.7379 | -6.1103 | 16.8609 |
| H | -7.9750 | -5.1246 | 15.3163 |
| C | -8.7192 | -3.2102 | 14.6410 |
| H | -7.3036 | -3.7205 | 16.1775 |
| H | -3.0336 | -4.7551 | 16.9527 |
| H | -4.2927 | -3.5211 | 17.1911 |
| C | -2.3401 | -2.7120 | 16.8014 |
| H | 18.9086 | 3.9392 | 0.7568 |
| C | 18.0336 | 2.8845 | -0.8999 |
| H | 18.8285 | 2.1772 | 0.9855 |
| H | 19.2956 | 4.4577 | 2.5681 |
| H | 19.3437 | 2.7673 | 3.0841 |
| H | 19.0461 | 4.0464 | 4.2687 |
| H | 18.0170 | 2.4643 | 5.5637 |
| H | 18.4626 | 1.3772 | 4.2318 |
| C | 16.9686 | 0.5715 | 5.5688 |
| H | 18.1904 | 6.3669 | 2.9564 |
| H | 18.1403 | 5.6882 | 4.5989 |
| C | 16.8402 | 7.3769 | 4.3139 |
| H | 2.8484 | 14.7110 | 9.4624 |
| H | 1.0766 | 14.7296 | 9.3111 |
| C | 2.1452 | 14.7313 | 7.4294 |
| H | 0.7575 | 12.0629 | 13.0687 |
| C | -0.9941 | 10.8474 | 12.7794 |
| H | -0.4589 | 12.7920 | 11.9964 |
| H | 3.8409 | 12.2604 | 12.8084 |
| H | 4.7287 | 13.0726 | 11.4991 |
| C | 5.6299 | 11.2346 | 12.1973 |
| H | 2.1722 | 13.3358 | 12.6427 |
| H | 1.1436 | 14.1758 | 11.4753 |
| H | 2.9020 | 14.2849 | 11.3422 |
| H | 9.5879 | 4.5101 | -14.1921 |
| H | 8.1450 | 4.1933 | -15.1822 |
| C | 8.7718 | 2.5356 | -13.9432 |
| H | 9.6529 | 6.3955 | -14.4612 |
| H | 8.6902 | 7.8634 | -14.6706 |
| H | 8.2083 | 6.3679 | -15.4796 |
| H | 10.3995 | 7.2668 | -12.5388 |
| C | 10.0351 | 8.6754 | -10.9361 |
| H | 9.3359 | 8.6237 | -12.9705 |
| H | 6.1532 | 7.2305 | -15.6077 |
| H | 6.8424 | 8.5945 | -14.7011 |
| C | 4.6881 | 8.5492 | -14.7138 |
| H | -6.2720 | 16.3951 | -6.3324 |
| H | -6.8111 | 15.4190 | -7.7186 |
| C | -4.6730 | 15.5482 | -7.5126 |
| H | -8.3208 | 16.3430 | -5.7225 |
| H | -9.7425 | 15.2981 | -5.6331 |
| H | -8.7505 | 15.3196 | -7.0973 |
| H | -8.0412 | 16.4260 | -3.7776 |
| C | -8.1202 | 15.7500 | -1.7312 |
| H | -9.5080 | 15.5091 | -3.3759 |
| H | -9.3420 | 13.5512 | -7.6317 |
| H | -10.4713 | 13.3318 | -6.2769 |
| C | -10.0740 | 11.5386 | -7.4244 |
| H | -4.5733 | -8.6982 | 12.8077 |
| C | -4.5861 | -10.0813 | 14.4767 |
| H | -3.1015 | -8.6583 | 13.8019 |
| H | -5.6718 | -10.2300 | 14.4900 |
| H | -4.2296 | -10.1656 | 15.5095 |
| H | -4.1484 | -10.9127 | 13.9121 |
| H | -2.7706 | -1.7047 | 16.6287 |
| H | -1.5098 | -2.8254 | 16.0710 |
| C | -1.7809 | -2.7871 | 18.2212 |
| H | -2.5682 | -2.6656 | 18.9736 |
| H | -1.0397 | -1.9984 | 18.3938 |
| H | -1.2878 | -3.7470 | 18.4126 |
| H | -8.9064 | -3.4188 | 13.5656 |
| H | -8.4263 | -2.1433 | 14.7062 |
| C | -10.0086 | -3.4303 | 15.4302 |
| H | -10.3680 | -4.4623 | 15.3412 |
| H | -10.8108 | -2.7781 | 15.0649 |
| H | -9.8745 | -3.2189 | 16.4966 |
| H | 0.6358 | -10.9482 | -10.9852 |
| C | 0.3590 | -12.8112 | -12.0610 |
| H | -0.4286 | -12.1137 | -10.1687 |
| H | 0.6821 | -12.4301 | -13.0362 |
| H | -0.4025 | -13.5780 | -12.2436 |
| H | 1.2200 | -13.3111 | -11.6023 |
| H | 4.8448 | -14.2748 | 0.7278 |
| H | 4.2962 | -14.8724 | 2.3101 |
| C | 5.1255 | -16.4030 | 1.0233 |
| H | 5.8717 | -16.5296 | 0.2307 |
| H | 4.1524 | -16.6816 | 0.6023 |
| H | 5.3583 | -17.1260 | 1.8132 |
| C | -7.5974 | -11.3427 | -9.7296 |
| H | -6.6357 | -9.6132 | -8.8436 |
| H | -5.7618 | -11.1429 | -8.5991 |
| H | -8.2302 | -10.7761 | -10.4225 |
| H | -8.2050 | -11.5725 | -8.8469 |
| H | -7.3506 | -12.2945 | -10.2132 |
| H | -15.7197 | -5.9320 | 1.8353 |
| C | -17.3142 | -5.9065 | 3.2990 |
| H | -15.4760 | -4.7687 | 3.1573 |
| H | -18.0837 | -6.3569 | 2.6624 |
| H | -17.8170 | -5.2207 | 3.9905 |
| H | -16.8748 | -6.7112 | 3.9003 |
| C | -18.2983 | 2.4621 | 1.3556 |
| H | -16.2785 | 2.1068 | 0.6556 |
| H | -16.6177 | 1.4504 | 2.2718 |
| H | -18.7217 | 2.6723 | 0.3665 |
| H | -18.0665 | 3.4263 | 1.8221 |
| H | -19.0830 | 1.9878 | 1.9554 |
| C | -11.4071 | 11.7004 | -8.1532 |
| H | -10.1716 | 10.7556 | -6.6421 |
| H | -9.3070 | 11.1727 | -8.1368 |
| H | -12.2087 | 12.0027 | -7.4698 |
| H | -11.7140 | 10.7604 | -8.6249 |
| H | -11.3459 | 12.4577 | -8.9432 |
| H | -8.3294 | 14.8179 | -1.1630 |
| C | -8.9509 | 16.8971 | -1.1584 |
| H | -7.0422 | 15.9657 | -1.5836 |
| H | -10.0258 | 16.6991 | -1.2331 |
| H | -8.7527 | 17.8405 | -1.6796 |
| H | -8.7200 | 17.0568 | -0.0984 |
| H | -1.6106 | 10.4462 | 11.9458 |
| C | -1.9048 | 11.5070 | 13.8137 |
| H | -0.4858 | 9.9765 | 13.2396 |
| H | -2.4753 | 12.3367 | 13.3808 |
| H | -1.3341 | 11.9071 | 14.6594 |
| H | -2.6272 | 10.7887 | 14.2175 |
| H | 6.1314 | 10.8940 | 11.2649 |
| H | 5.3252 | 10.3181 | 12.7408 |
| C | 6.6243 | 12.0224 | 13.0485 |
| H | 6.9965 | 12.9082 | 12.5206 |
| H | 7.4944 | 11.4084 | 13.3073 |
| H | 6.1739 | 12.3643 | 13.9870 |
| H | 16.0617 | 7.7315 | 3.6032 |
| C | 17.8685 | 8.4866 | 4.5354 |
| H | 16.3159 | 7.1640 | 5.2676 |
| H | 18.3617 | 8.7715 | 3.5981 |
| H | 18.6504 | 8.1777 | 5.2381 |
| H | 17.3909 | 9.3847 | 4.9427 |
| H | 16.7203 | -0.2388 | 4.8543 |
| H | 16.0069 | 0.8815 | 6.0314 |
| C | 17.9061 | 0.0367 | 6.6458 |
| H | 18.8643 | -0.2925 | 6.2271 |
| H | 17.4638 | -0.8255 | 7.1602 |
| H | 18.1206 | 0.8087 | 7.4170 |
| H | 6.1577 | -13.1435 | 7.3435 |
| H | 7.3688 | -11.8751 | 7.6308 |
| C | 7.8771 | -13.8004 | 8.4848 |
| H | 7.7647 | -14.8722 | 8.2863 |
| H | 7.4068 | -13.5969 | 9.4536 |
| H | 8.9493 | -13.5981 | 8.5926 |
| C | -3.9241 | -5.7132 | -15.3050 |
| H | -2.1443 | -5.8159 | -14.0779 |
| H | -3.5804 | -5.2149 | -13.2244 |
| H | -3.6048 | -6.4312 | -16.0685 |
| H | -3.6932 | -4.7101 | -15.6812 |
| H | -5.0148 | -5.7860 | -15.2205 |
| H | 10.5130 | -10.2846 | 1.1644 |
| C | 12.2619 | -11.5408 | 0.9143 |
| H | 11.5362 | -10.3980 | 2.6087 |
| H | 11.8922 | -12.0376 | 0.0059 |
| H | 12.8763 | -12.2655 | 1.4631 |
| H | 12.9314 | -10.7354 | 0.5911 |
| H | 17.6551 | 3.8333 | -1.3318 |
| H | 17.2507 | 2.1197 | -1.0942 |
| C | 19.3248 | 2.4830 | -1.6112 |
| H | 20.1191 | 3.2217 | -1.4562 |
| H | 19.1667 | 2.3961 | -2.6924 |
| H | 19.7002 | 1.5165 | -1.2563 |
| H | 1.4260 | 14.1833 | 6.7821 |
| H | 3.1576 | 14.4781 | 7.0561 |
| C | 1.9127 | 16.2346 | 7.2862 |
| H | 0.8968 | 16.5177 | 7.5853 |
| H | 2.0474 | 16.5562 | 6.2471 |
| H | 2.6106 | 16.8138 | 7.9009 |
| C | -4.4977 | 16.8508 | -8.2909 |
| H | -4.5018 | 14.6874 | -8.1911 |
| H | -3.8927 | 15.4807 | -6.7243 |
| H | -5.2366 | 16.9453 | -9.0947 |
| H | -3.5052 | 16.9016 | -8.7535 |
| H | -4.6018 | 17.7283 | -7.6428 |
| C | -17.4987 | -3.9005 | -4.9073 |
| H | -15.8096 | -4.4866 | -3.6847 |
| H | -15.6974 | -2.8366 | -4.3335 |
| H | -18.2060 | -4.6404 | -4.5159 |
| H | -17.0713 | -4.3132 | -5.8284 |
| H | -18.0743 | -3.0102 | -5.1848 |
| C | 9.4463 | 1.8752 | -15.1444 |
| H | 7.7908 | 2.0458 | -13.7567 |
| H | 9.3835 | 2.3668 | -13.0356 |
| H | 8.8329 | 1.9631 | -16.0496 |
| H | 9.6094 | 0.8051 | -14.9708 |
| H | 10.4192 | 2.3275 | -15.3673 |
| H | 9.2886 | 9.4532 | -10.6768 |
| C | 11.3905 | 9.3355 | -11.1853 |
| H | 10.1063 | 8.0036 | -10.0530 |
| H | 11.3559 | 10.0157 | -12.0441 |
| H | 12.1712 | 8.5926 | -11.3843 |
| H | 11.7070 | 9.9223 | -10.3158 |
| C | 4.4623 | 9.2444 | -16.0554 |
| H | 4.6394 | 9.2984 | -13.8976 |
| H | 3.8629 | 7.8286 | -14.5250 |
| H | 5.2495 | 9.9757 | -16.2706 |
| H | 3.5072 | 9.7825 | -16.0628 |
| H | 4.4413 | 8.5293 | -16.8854 |
| C | 23.1431 | 3.1011 | -13.8072 |
| C | 21.3670 | 1.8114 | -15.1434 |
| C | 20.9346 | 4.2478 | -14.4564 |
| C | 22.0648 | 3.2444 | -14.9917 |
| C | 23.0179 | 1.6559 | -13.3617 |
| C | 22.1277 | 0.9245 | -14.1772 |
| C | 21.9612 | -0.4452 | -13.9890 |
| C | 22.6429 | -1.0962 | -12.9574 |
| N | 24.0553 | -1.0470 | -10.9844 |
| C | 23.4819 | -0.3581 | -12.1056 |
| C | 23.6722 | 1.0322 | -12.3036 |
| C | 19.9690 | 2.0169 | -14.5786 |
| C | 19.7406 | 3.3514 | -14.1820 |
| C | 18.5501 | 3.6983 | -13.5447 |
| C | 17.5665 | 2.7273 | -13.3280 |
| N | 16.7344 | 0.4666 | -13.4992 |
| C | 17.7986 | 1.3971 | -13.7202 |
| C | 19.0116 | 1.0363 | -14.3432 |
| C | 21.4909 | 4.7589 | -13.1378 |
| C | 22.6711 | 4.0831 | -12.7566 |
| C | 23.2783 | 4.3731 | -11.5382 |
| C | 22.7292 | 5.3473 | -10.6989 |
| N | 21.1238 | 7.0599 | -10.1903 |
| C | 21.5664 | 6.0374 | -11.0877 |
| C | 20.9448 | 5.7417 | -12.3210 |
| C | 30.3219 | -8.0512 | -3.6330 |
| C | 31.6255 | -6.6837 | -1.8912 |
| C | 30.5100 | -8.8955 | -1.2120 |
| C | 31.3301 | -8.1793 | -2.3877 |
| C | 30.2234 | -6.5590 | -3.8778 |
| C | 30.9773 | -5.8095 | -2.9486 |
| C | 31.0372 | -4.4217 | -3.0535 |
| C | 30.3360 | -3.7665 | -4.0717 |
| N | 28.9487 | -3.8225 | -6.0654 |
| C | 29.5694 | -4.5156 | -4.9795 |
| C | 29.5113 | -5.9218 | -4.8856 |
| C | 30.8530 | -6.5659 | -0.5890 |
| C | 30.2659 | -7.7883 | -0.1995 |
| C | 29.4776 | -7.8494 | 0.9480 |
| C | 29.2842 | -6.7018 | 1.7246 |
| N | 29.6441 | -4.3545 | 2.1804 |
| C | 29.8519 | -5.4806 | 1.3215 |
| C | 30.6371 | -5.4093 | 0.1507 |
| C | 29.1694 | -9.2307 | -1.8485 |
| C | 29.0265 | -8.6468 | -3.1268 |
| C | 27.7931 | -8.6773 | -3.7714 |
| C | 26.7209 | -9.3644 | -3.1914 |
| N | 25.7752 | -10.7768 | -1.4811 |
| C | 26.8869 | -10.0117 | -1.9550 |
| C | 28.1203 | -9.9360 | -1.2720 |
| C | 26.9512 | 6.5568 | 10.1670 |
| C | 28.8409 | 5.0984 | 9.2148 |
| C | 28.1509 | 7.2625 | 8.0080 |
| C | 28.3912 | 6.6173 | 9.4569 |
| C | 26.6433 | 5.0751 | 10.2573 |
| C | 27.7352 | 4.2772 | 9.8507 |
| C | 27.6755 | 2.8920 | 9.9737 |
| C | 26.5122 | 2.2829 | 10.4549 |
| N | 24.2404 | 2.4442 | 11.3145 |
| C | 25.3989 | 3.0762 | 10.7743 |
| C | 25.4579 | 4.4833 | 10.6720 |
| C | 28.7363 | 4.9183 | 7.7050 |
| C | 28.3224 | 6.1004 | 7.0516 |
| C | 28.0605 | 6.0921 | 5.6844 |
| C | 28.2466 | 4.9203 | 4.9456 |
| N | 28.8834 | 2.6000 | 4.7597 |
| C | 28.6628 | 3.7447 | 5.5916 |
| C | 28.8994 | 3.7383 | 6.9855 |
| C | 26.6992 | 7.6981 | 8.0324 |
| C | 26.0315 | 7.2782 | 9.2028 |
| C | 24.6766 | 7.5538 | 9.3701 |
| C | 23.9786 | 8.2511 | 8.3793 |
| N | 23.8682 | 9.4330 | 6.2885 |
| C | 24.6473 | 8.6751 | 7.2167 |
| C | 26.0218 | 8.4028 | 7.0460 |
| C | 28.5498 | 1.4042 | 5.1406 |
| C | 28.8003 | 0.2528 | 4.2495 |
| C | 29.1025 | 0.4146 | 2.8932 |
| C | 29.3035 | -0.7082 | 2.0845 |
| C | 29.2170 | -2.0241 | 2.6049 |
| C | 28.9302 | -2.1869 | 3.9652 |
| C | 28.7214 | -1.0633 | 4.7730 |
| C | 29.4171 | -3.1721 | 1.6959 |
| C | 17.8727 | 16.5951 | -1.0073 |
| C | 18.5303 | 16.2792 | -3.4740 |
| C | 16.0533 | 16.5066 | -2.8264 |
| C | 17.5232 | 17.0644 | -2.5045 |
| C | 19.0179 | 15.6098 | -1.1841 |
| C | 19.4214 | 15.4969 | -2.5323 |
| C | 20.4958 | 14.6833 | -2.8818 |
| C | 21.1540 | 13.9396 | -1.8998 |
| N | 21.4592 | 13.2181 | 0.3711 |
| C | 20.7102 | 13.9970 | -0.5671 |
| C | 19.6354 | 14.8396 | -0.2036 |
| C | 17.6381 | 15.3169 | -4.2394 |
| C | 16.2714 | 15.4928 | -3.9335 |
| C | 15.3063 | 14.7242 | -4.5799 |
| C | 15.6932 | 13.7371 | -5.4928 |
| N | 17.4035 | 12.4892 | -6.6794 |
| C | 17.0586 | 13.5096 | -5.7341 |
| C | 18.0398 | 14.3116 | -5.1110 |
| C | 15.6458 | 15.7840 | -1.5539 |
| C | 16.6260 | 15.8752 | -0.5432 |
| C | 16.4062 | 15.2968 | 0.7033 |
| C | 15.2271 | 14.5849 | 0.9458 |
| N | 13.0789 | 13.7182 | 0.2119 |
| C | 14.2814 | 14.4402 | -0.0822 |
| C | 14.4883 | 15.0473 | -1.3407 |
| C | 20.9099 | 12.6293 | 1.3885 |
| C | 21.7565 | 11.8429 | 2.3105 |
| C | 23.0465 | 11.4286 | 1.9590 |
| C | 23.8008 | 10.6607 | 2.8515 |
| C | 23.2957 | 10.2906 | 4.1233 |
| C | 22.0114 | 10.7167 | 4.4814 |
| C | 21.2530 | 11.4784 | 3.5848 |
| C | 19.9020 | 7.4970 | -10.1659 |
| C | 19.5374 | 8.5678 | -9.2130 |
| C | 20.3089 | 8.8449 | -8.0805 |
| C | 19.9128 | 9.8535 | -7.1948 |
| C | 18.7390 | 10.6180 | -7.4137 |
| C | 17.9731 | 10.3492 | -8.5552 |
| C | 18.3664 | 9.3372 | -9.4372 |
| C | 27.0904 | -3.1215 | -7.4102 |
| C | 18.3688 | 11.6573 | -6.4310 |
| C | 24.1311 | 9.4627 | 5.0195 |
| C | 27.6677 | -3.8390 | -6.2526 |
| C | 12.1090 | -1.6990 | 14.1323 |
| C | 12.1251 | -4.2563 | 13.8984 |
| C | 14.2262 | -3.0277 | 14.7290 |
| C | 12.6235 | -3.0488 | 14.8276 |
| C | 11.4688 | -2.1687 | 12.8339 |
| C | 11.4822 | -3.5736 | 12.7060 |
| C | 11.0061 | -4.1719 | 11.5404 |
| C | 10.4830 | -3.3805 | 10.5094 |
| N | 9.9939 | -1.2267 | 9.5180 |
| C | 10.4701 | -1.9799 | 10.6389 |
| C | 10.9855 | -1.3689 | 11.8038 |
| C | 13.4134 | -4.9371 | 13.4597 |
| C | 14.5627 | -4.2415 | 13.8919 |
| C | 15.8328 | -4.6963 | 13.5429 |
| C | 15.9724 | -5.8438 | 12.7582 |
| N | 15.0190 | -7.7324 | 11.5791 |
| C | 14.8255 | -6.5321 | 12.3274 |
| C | 13.5390 | -6.0841 | 12.6873 |
| C | 14.5387 | -1.7338 | 14.0067 |
| C | 13.3860 | -0.9498 | 13.7850 |
| C | 13.5010 | 0.3201 | 13.2196 |
| C | 14.7548 | 0.7959 | 12.8208 |
| N | 17.1287 | 0.4600 | 12.4382 |
| C | 15.8912 | -0.0172 | 12.9817 |
| C | 15.7850 | -1.2934 | 13.5788 |
| C | 18.2426 | 0.4305 | 13.1080 |
| C | 19.4813 | 0.9317 | 12.4762 |
| C | 14.9351 | -12.5747 | 7.1203 |
| C | 14.7399 | -11.4108 | 8.0062 |
| C | 20.9473 | -13.9531 | 1.6186 |
| C | 22.1432 | -13.2103 | 1.1707 |
| C | 13.7293 | -10.4767 | 7.7773 |
| C | 13.6214 | -9.3555 | 8.6060 |
| C | 14.5043 | -9.1467 | 9.6918 |
| C | 15.5044 | -10.0964 | 9.9390 |
| C | 15.6232 | -11.2083 | 9.0999 |
| C | 14.3347 | -7.9238 | 10.4976 |
| C | 8.3723 | -9.0494 | -10.4247 |
| C | 6.5570 | -7.4776 | -9.5267 |
| C | 7.7877 | -6.8973 | -11.7058 |
| C | 7.1528 | -8.1100 | -10.8778 |
| C | 8.2606 | -9.0869 | -8.9066 |
| C | 7.2941 | -8.1807 | -8.4283 |
| C | 7.0835 | -8.0087 | -7.0677 |
| C | 7.8199 | -8.7659 | -6.1543 |
| N | 9.3934 | -10.5068 | -5.6283 |
| C | 8.7666 | -9.6957 | -6.6239 |
| C | 8.9930 | -9.8572 | -8.0112 |
| C | 6.9255 | -6.0006 | -9.6152 |
| C | 7.4719 | -5.6669 | -10.8749 |
| C | 7.7711 | -4.3444 | -11.1874 |
| C | 7.6076 | -3.3471 | -10.2247 |
| N | 7.0152 | -2.6234 | -7.9996 |
| C | 7.1665 | -3.6914 | -8.9392 |
| C | 6.8051 | -5.0238 | -8.6290 |
| C | 9.2925 | -7.1155 | -11.5795 |
| C | 9.6089 | -8.2752 | -10.8362 |
| C | 10.9316 | -8.5540 | -10.5020 |
| C | 11.9531 | -7.6932 | -10.9150 |
| N | 12.7354 | -5.6850 | -12.0178 |
| C | 11.6399 | -6.5325 | -11.6399 |
| C | 10.2986 | -6.2447 | -11.9864 |
| C | 10.6514 | -10.8165 | -5.6564 |
| C | 12.6196 | -4.3930 | -11.9298 |
| C | 11.2122 | -11.6500 | -4.5742 |
| C | 13.7390 | -3.5023 | -12.2950 |
| C | 10.4979 | -11.9288 | -3.4038 |
| C | 11.0875 | -12.6848 | -2.3836 |
| C | 12.4119 | -13.1923 | -2.5114 |
| C | 13.1147 | -12.9302 | -3.6929 |
| C | 12.5234 | -12.1700 | -4.7060 |
| C | 15.0492 | -3.9660 | -12.4535 |
| C | 16.0790 | -3.0621 | -12.7378 |
| C | 15.8326 | -1.6721 | -12.8735 |
| C | 14.5171 | -1.2125 | -12.7434 |
| C | 13.4877 | -2.1156 | -12.4584 |
| C | 3.4669 | 6.7998 | 1.5154 |
| C | 3.0767 | 5.2199 | -0.4864 |
| C | 2.8824 | 4.3268 | 1.9165 |
| C | 2.5467 | 5.5876 | 0.9839 |
| C | 4.4396 | 7.0492 | 0.3872 |
| C | 4.1887 | 6.2328 | -0.7390 |
| C | 4.9681 | 6.3762 | -1.8867 |
| C | 6.0230 | 7.2952 | -1.9030 |
| N | 7.4438 | 8.9132 | -0.8699 |
| C | 6.3057 | 8.0611 | -0.7567 |
| C | 5.5083 | 7.9319 | 0.3984 |
| C | 3.6634 | 3.8296 | -0.3347 |
| C | 3.4956 | 3.3168 | 0.9698 |
| C | 3.9311 | 2.0330 | 1.2859 |
| C | 4.5690 | 1.2591 | 0.3121 |
| N | 5.3678 | 0.9152 | -1.9290 |
| C | 4.7841 | 1.7946 | -0.9716 |
| C | 4.3357 | 3.0905 | -1.2968 |
| C | 3.9448 | 4.8404 | 2.8736 |
| C | 4.1972 | 6.2179 | 2.7044 |
| C | 5.0890 | 6.8725 | 3.5457 |
| C | 5.7859 | 6.1510 | 4.5215 |
| N | 6.2936 | 4.0566 | 5.6695 |
| C | 5.6028 | 4.7639 | 4.6298 |
| C | 4.6713 | 4.0977 | 3.8008 |
| C | 6.6010 | 0.2627 | -3.8417 |
| C | 7.7981 | 9.7491 | 0.0570 |
| C | 7.0991 | 3.0724 | 5.4133 |
| C | 9.0233 | 10.5546 | -0.1341 |
| C | 7.7234 | 2.3101 | 6.5236 |
| C | 9.6313 | 10.6815 | -1.3875 |
| C | 10.7857 | 11.4550 | -1.5241 |
| C | 11.3831 | 12.1082 | -0.4180 |
| C | 10.7753 | 11.9888 | 0.8347 |
| C | 9.6111 | 11.2219 | 0.9710 |
| C | 6.5729 | -1.0942 | -3.5015 |
| C | 6.8986 | -2.0591 | -4.4580 |
| C | 7.2113 | -1.6944 | -5.7917 |
| C | 7.2830 | -0.3387 | -6.1230 |
| C | 7.0149 | 0.6298 | -5.1480 |
| C | 7.6687 | 2.7347 | 7.8600 |
| C | 8.2268 | 1.9362 | 8.8709 |
| C | 8.8409 | 0.6937 | 8.5841 |
| C | 8.9203 | 0.2847 | 7.2479 |
| C | 8.3786 | 1.0854 | 6.2341 |
| C | 7.4320 | -2.7688 | -6.7821 |
| C | 13.0280 | -13.9572 | -1.4048 |
| C | 9.3478 | -0.1173 | 9.7118 |
| C | 23.1499 | -12.8110 | 2.0563 |
| C | 24.2371 | -12.0661 | 1.5869 |
| C | 24.3560 | -11.7040 | 0.2210 |
| C | 23.3599 | -12.1224 | -0.6686 |
| C | 22.2700 | -12.8622 | -0.1983 |
| C | 20.7045 | 0.9027 | 13.1967 |
| C | 21.8902 | 1.4022 | 12.6445 |
| C | 21.8850 | 1.9451 | 11.3514 |
| C | 20.6698 | 1.9489 | 10.6291 |
| C | 19.4860 | 1.4523 | 11.1799 |
| C | 12.6280 | 12.8650 | -0.6530 |
| C | 23.0914 | 2.5333 | 10.7285 |
| C | 16.9644 | -0.7512 | -13.1136 |
| C | 25.5095 | -10.8904 | -0.2162 |
| C | 27.8951 | -2.5387 | -8.3953 |
| C | 27.3081 | -1.8770 | -9.4776 |
| C | 25.9009 | -1.7795 | -9.6148 |
| C | 25.0940 | -2.3596 | -8.6287 |
| C | 25.6815 | -3.0196 | -7.5424 |
| C | 25.3402 | -1.0754 | -10.7882 |
| C | 17.0019 | -17.7279 | 2.2134 |
| C | 16.2312 | -17.2273 | 4.6137 |
| C | 14.5085 | -17.3389 | 2.7053 |
| C | 15.8398 | -18.0271 | 3.2813 |
| C | 17.9377 | -16.7758 | 2.9369 |
| C | 17.5603 | -16.5722 | 4.2814 |
| C | 18.3434 | -15.7743 | 5.1133 |
| C | 19.4669 | -15.1204 | 4.5994 |
| N | 20.9423 | -14.5433 | 2.7742 |
| C | 19.7883 | -15.2534 | 3.2382 |
| C | 19.0278 | -16.0994 | 2.4015 |
| C | 15.1700 | -16.1455 | 4.7222 |
| C | 14.2018 | -16.2352 | 3.6993 |
| C | 13.1471 | -15.3276 | 3.6537 |
| C | 13.0725 | -14.2967 | 4.5926 |
| N | 13.9245 | -13.0653 | 6.4700 |
| C | 14.0713 | -14.1683 | 5.5716 |
| C | 15.1212 | -15.1086 | 5.6450 |
| C | 14.9411 | -16.7330 | 1.3819 |
| C | 16.2956 | -16.9999 | 1.0888 |
| C | 16.8571 | -16.5572 | -0.1051 |
| C | 16.0893 | -15.8053 | -0.9985 |
| N | 14.0662 | -14.6983 | -1.6633 |
| C | 14.7571 | -15.4802 | -0.6795 |
| C | 14.1757 | -15.9550 | 0.5187 |
| C | 6.1568 | 1.2958 | -2.8815 |
| O | 25.2839 | -11.6161 | 2.3703 |
| O | 21.2317 | -13.3275 | -0.9841 |
| C | 25.2316 | -11.9028 | 3.7891 |
| C | 21.2373 | -12.9658 | -2.3865 |
| O | 11.4403 | 11.6897 | -2.7186 |
| C | 10.9836 | 10.9557 | -3.8759 |
| O | 8.9349 | 11.0126 | 2.1564 |
| C | 9.4285 | 11.6816 | 3.3428 |
| O | 20.7822 | 2.4784 | 9.3626 |
| O | 20.5922 | 0.3520 | 14.4608 |
| C | 19.5848 | 2.5112 | 8.5538 |
| C | 21.7866 | 0.3008 | 15.2783 |
| O | 17.4066 | -3.4051 | -12.9124 |
| O | 12.1578 | -1.7722 | -12.3066 |
| C | 11.8182 | -0.3659 | -12.3729 |
| C | 17.7675 | -4.7976 | -12.7445 |
| O | 6.9335 | -3.4248 | -4.2342 |
| C | 6.6857 | -3.9021 | -2.8851 |
| O | 7.1402 | 1.9922 | -5.3356 |
| C | 7.3768 | 2.4756 | -6.6852 |
| O | 17.6865 | 8.9811 | -10.5886 |
| C | 16.4899 | 9.7267 | -10.9179 |
| O | 20.6019 | 10.2139 | -6.0527 |
| C | 21.7679 | 9.4324 | -5.6951 |
| O | 16.5744 | -12.2062 | 9.2314 |
| O | 12.6751 | -8.3648 | 8.4763 |
| C | 17.5567 | -12.0566 | 10.2846 |
| C | 11.8245 | -8.3829 | 7.3030 |
| O | 29.6061 | -0.6678 | 0.7360 |
| C | 29.6506 | 0.6311 | 0.0971 |
| O | 28.4322 | -1.1042 | 6.1247 |
| C | 28.2644 | -2.4065 | 6.7359 |
| O | 25.0789 | 10.1912 | 2.6081 |
| O | 19.9764 | 11.9496 | 3.8290 |
| C | 19.3609 | 11.5998 | 5.0929 |
| C | 25.6862 | 10.5294 | 1.3378 |
| O | 24.9834 | -3.6338 | -6.5206 |
| O | 28.0072 | -1.2642 | -10.5008 |
| C | 23.5389 | -3.5234 | -6.5305 |
| C | 29.4533 | -1.3326 | -10.4608 |
| O | 8.2335 | 2.2333 | 10.2277 |
| C | 7.6985 | 3.4998 | 10.6802 |
| O | 8.4071 | 0.7806 | 4.8897 |
| C | 9.0438 | -0.4586 | 4.4869 |
| O | 10.4727 | -12.9944 | -1.1841 |
| C | 9.0861 | -12.5895 | -1.0183 |
| O | 13.1262 | -11.8520 | -5.9099 |
| C | 14.4748 | -12.3306 | -6.1326 |
| C | 24.5789 | 3.4293 | -14.2795 |
| C | 21.3794 | 1.2990 | -16.5958 |
| C | 20.6242 | 5.3764 | -15.4610 |
| C | 22.7011 | 3.7295 | -16.2860 |
| H | 21.2972 | -1.0120 | -14.6496 |
| H | 22.5155 | -2.1652 | -12.7998 |
| H | 24.2981 | 1.5978 | -11.6180 |
| H | 18.3826 | 4.7147 | -13.1929 |
| H | 16.6280 | 2.9912 | -12.8426 |
| H | 19.1924 | 0.0001 | -14.6175 |
| H | 24.1937 | 3.8526 | -11.2440 |
| H | 23.2002 | 5.5815 | -9.7446 |
| H | 20.0556 | 6.2897 | -12.6444 |
| C | 30.8387 | -8.7946 | -4.8856 |
| C | 33.1323 | -6.4064 | -1.7142 |
| C | 31.2494 | -10.1158 | -0.6359 |
| C | 32.6024 | -8.9255 | -2.7610 |
| H | 31.6416 | -3.8434 | -2.3470 |
| H | 30.3846 | -2.6834 | -4.1658 |
| H | 28.9403 | -6.5017 | -5.6130 |
| H | 28.9903 | -8.7778 | 1.2407 |
| H | 28.6825 | -6.7446 | 2.6312 |
| H | 31.0557 | -4.4573 | -0.1670 |
| H | 27.6493 | -8.1658 | -4.7216 |
| H | 25.7564 | -9.4046 | -3.6957 |
| H | 28.2572 | -10.4349 | -0.3088 |
| C | 26.9541 | 7.2206 | 11.5635 |
| C | 30.2388 | 4.8000 | 9.7874 |
| C | 29.1137 | 8.4290 | 7.7118 |
| C | 29.4000 | 7.4073 | 10.2791 |
| H | 28.5398 | 2.2792 | 9.6993 |
| H | 26.4607 | 1.2021 | 10.5682 |
| H | 24.5865 | 5.0828 | 10.9222 |
| H | 27.6937 | 6.9860 | 5.1832 |
| H | 28.0588 | 4.9094 | 3.8732 |
| H | 29.1933 | 2.8163 | 7.4817 |
| H | 24.1618 | 7.2425 | 10.2841 |
| H | 22.9196 | 8.4748 | 8.5041 |
| H | 26.5579 | 8.7620 | 6.1641 |
| H | 28.0643 | 1.1353 | 6.0995 |
| H | 29.1800 | 1.4282 | 2.4933 |
| H | 28.8803 | -3.1999 | 4.3711 |
| H | 29.3457 | -2.9109 | 0.6219 |
| C | 18.2417 | 17.7771 | -0.0883 |
| C | 19.3189 | 17.2277 | -4.3993 |
| C | 15.0723 | 17.6275 | -3.2247 |
| C | 17.6191 | 18.5742 | -2.6740 |
| H | 20.8294 | 14.6325 | -3.9229 |
| H | 22.0021 | 13.3081 | -2.1599 |
| H | 19.3024 | 14.8799 | 0.8305 |
| H | 14.2447 | 14.8935 | -4.3759 |
| H | 14.9453 | 13.1363 | -6.0059 |
| H | 19.0938 | 14.1335 | -5.3115 |
| H | 17.1526 | 15.4048 | 1.4957 |
| H | 15.0430 | 14.1342 | 1.9195 |
| H | 13.7460 | 14.9311 | -2.1268 |
| H | 19.8360 | 12.6429 | 1.6571 |
| H | 23.4418 | 11.7248 | 0.9848 |
| H | 21.6293 | 10.4499 | 5.4694 |
| H | 19.0636 | 7.1526 | -10.7996 |
| H | 21.2209 | 8.2666 | -7.9151 |
| H | 17.0772 | 10.9479 | -8.7339 |
| H | 18.9792 | 11.6360 | -5.5053 |
| H | 24.9475 | 8.9228 | 4.5037 |
| H | 26.9221 | -4.3500 | -5.6127 |
| C | 11.1451 | -0.9036 | 15.0272 |
| C | 11.1602 | -5.2045 | 14.6385 |
| C | 14.8990 | -3.0823 | 16.1194 |
| C | 12.1449 | -3.1912 | 16.2644 |
| H | 11.0511 | -5.2524 | 11.4115 |
| H | 10.0925 | -3.8453 | 9.6059 |
| H | 11.0335 | -0.2842 | 11.8727 |
| H | 16.7221 | -4.1621 | 13.8905 |
| H | 16.9605 | -6.2064 | 12.4781 |
| H | 12.6534 | -6.6442 | 12.3781 |
| H | 12.6121 | 0.9430 | 13.0833 |
| H | 14.8543 | 1.7815 | 12.3691 |
| H | 16.6639 | -1.9277 | 13.6675 |
| H | 18.3711 | 0.0729 | 14.1485 |
| H | 15.9810 | -12.9368 | 7.0859 |
| H | 20.1090 | -13.9326 | 0.8938 |
| H | 13.0343 | -10.6416 | 6.9510 |
| H | 16.1767 | -9.9407 | 10.7860 |
| H | 13.5933 | -7.2159 | 10.0772 |
| C | 8.3081 | -10.4513 | -11.0655 |
| C | 5.0388 | -7.6915 | -9.3826 |
| C | 7.2661 | -6.8320 | -13.1513 |
| C | 6.1001 | -8.8771 | -11.6628 |
| H | 6.3325 | -7.2953 | -6.7178 |
| H | 7.6632 | -8.6481 | -5.0826 |
| H | 9.7152 | -10.5922 | -8.3773 |
| H | 8.1384 | -4.0858 | -12.1862 |
| H | 7.8407 | -2.3099 | -10.4594 |
| H | 6.4465 | -5.2746 | -7.6328 |
| H | 11.1808 | -9.4245 | -9.8980 |
| H | 12.9895 | -7.9057 | -10.6581 |
| H | 10.0643 | -5.3417 | -12.5462 |
| H | 11.3856 | -10.5111 | -6.4266 |
| H | 11.7226 | -3.8486 | -11.5678 |
| H | 9.4783 | -11.5444 | -3.3094 |
| H | 14.1219 | -13.3386 | -3.8005 |
| H | 15.2395 | -5.0364 | -12.3502 |
| H | 14.3214 | -0.1444 | -12.8680 |
| C | 2.6666 | 8.0564 | 1.8722 |
| C | 1.9637 | 5.2751 | -1.5563 |
| C | 1.6454 | 3.7723 | 2.6478 |
| C | 1.0600 | 5.9302 | 0.9895 |
| H | 4.7558 | 5.7727 | -2.7755 |
| H | 6.6409 | 7.4120 | -2.7938 |
| H | 5.7431 | 8.5024 | 1.2932 |
| H | 3.7658 | 1.6298 | 2.2901 |
| H | 4.9018 | 0.2472 | 0.5395 |
| H | 4.5154 | 3.4951 | -2.2893 |
| H | 5.2356 | 7.9524 | 3.4539 |
| H | 6.4747 | 6.6576 | 5.1950 |
| H | 4.5340 | 3.0233 | 3.8947 |
| H | 7.2837 | 9.9253 | 1.0185 |
| H | 7.3741 | 2.7061 | 4.4055 |
| H | 9.1883 | 10.1618 | -2.2414 |
| H | 11.2261 | 12.5007 | 1.6879 |
| H | 6.2670 | -1.3749 | -2.4907 |
| H | 7.5306 | -0.0584 | -7.1475 |
| H | 7.1709 | 3.6818 | 8.0860 |
| H | 9.4085 | -0.6647 | 7.0167 |
| H | 7.9403 | -3.6603 | -6.3677 |
| H | 12.5333 | -13.8233 | -0.4234 |
| H | 9.1049 | 0.3114 | 10.7030 |
| H | 23.0632 | -13.0959 | 3.1073 |
| H | 23.4598 | -11.8607 | -1.7247 |
| H | 22.8310 | 1.3856 | 13.2019 |
| H | 18.5479 | 1.4631 | 10.6179 |
| H | 13.1127 | 12.6199 | -1.6210 |
| H | 22.8996 | 3.0292 | 9.7587 |
| H | 17.9585 | -1.1982 | -12.9187 |
| H | 26.0709 | -10.4216 | 0.6149 |
| H | 28.9809 | -2.6113 | -8.2920 |
| H | 24.0079 | -2.2857 | -8.7313 |
| H | 26.1047 | -0.6145 | -11.4433 |
| C | 17.7162 | -19.0081 | 1.7357 |
| C | 16.2957 | -18.1490 | 5.8513 |
| C | 13.3495 | -18.3452 | 2.5520 |
| C | 15.6557 | -19.5159 | 3.5349 |
| H | 18.0798 | -15.6585 | 6.1693 |
| H | 20.0848 | -14.4951 | 5.2419 |
| H | 19.2938 | -16.2090 | 1.3528 |
| H | 12.3764 | -15.4212 | 2.8834 |
| H | 12.2521 | -13.5774 | 4.5621 |
| H | 15.8892 | -15.0067 | 6.4085 |
| H | 17.8957 | -16.8031 | -0.3464 |
| H | 16.5172 | -15.4592 | -1.9385 |
| H | 13.1438 | -15.7088 | 0.7583 |
| H | 6.5186 | 2.3191 | -3.0869 |
| H | 26.1585 | -11.4346 | 4.1443 |
| H | 24.3541 | -11.4310 | 4.2410 |
| H | 25.2470 | -12.9838 | 3.9591 |
| H | 20.3145 | -13.4424 | -2.7415 |
| H | 21.1879 | -11.8789 | -2.5005 |
| H | 22.1154 | -13.3891 | -2.8839 |
| H | 11.5458 | 11.4383 | -4.6862 |
| H | 11.2683 | 9.9078 | -3.7651 |
| H | 9.9065 | 11.0764 | -4.0265 |
| H | 8.6936 | 11.3643 | 4.0985 |
| H | 10.4293 | 11.3174 | 3.5918 |
| H | 9.4076 | 12.7663 | 3.2063 |
| H | 19.9515 | 2.9866 | 7.6350 |
| H | 19.2347 | 1.4697 | 8.3537 |
| H | 18.8118 | 3.1207 | 9.0310 |
| H | 21.4069 | -0.1653 | 16.1964 |
| H | 22.5463 | -0.3271 | 14.8029 |
| H | 22.1577 | 1.3113 | 15.4744 |
| H | 10.7348 | -0.3916 | -12.1983 |
| H | 12.3300 | 0.1895 | -11.5812 |
| H | 12.0471 | 0.0341 | -13.3661 |
| H | 18.8496 | -4.7691 | -12.9266 |
| H | 17.5490 | -5.1279 | -11.7248 |
| H | 17.2589 | -5.4146 | -13.4917 |
| H | 6.7351 | -4.9902 | -3.0254 |
| H | 7.4750 | -3.5488 | -2.2121 |
| H | 5.6916 | -3.5999 | -2.5398 |
| H | 7.4029 | 3.5637 | -6.5335 |
| H | 8.3450 | 2.1256 | -7.0579 |
| H | 6.5534 | 2.1895 | -7.3438 |
| H | 16.1846 | 9.2520 | -11.8591 |
| H | 15.7307 | 9.5894 | -10.1427 |
| H | 16.7298 | 10.7836 | -11.0715 |
| H | 22.0780 | 9.9152 | -4.7590 |
| H | 21.4901 | 8.3880 | -5.5246 |
| H | 22.5403 | 9.5302 | -6.4639 |
| H | 18.1704 | -12.9555 | 10.1465 |
| H | 18.1470 | -11.1485 | 10.1298 |
| H | 17.0672 | -12.0618 | 11.2634 |
| H | 11.2441 | -7.4565 | 7.4334 |
| H | 12.4231 | -8.3330 | 6.3901 |
| H | 11.1701 | -9.2589 | 7.3197 |
| H | 29.8831 | 0.3594 | -0.9407 |
| H | 28.6763 | 1.1239 | 0.1654 |
| H | 30.4482 | 1.2409 | 0.5324 |
| H | 28.0289 | -2.1356 | 7.7732 |
| H | 27.4314 | -2.9419 | 6.2709 |
| H | 29.1978 | -2.9750 | 6.6799 |
| H | 18.3824 | 12.0895 | 5.0055 |
| H | 19.2513 | 10.5144 | 5.1769 |
| H | 19.9367 | 12.0182 | 5.9242 |
| H | 26.6691 | 10.0492 | 1.4269 |
| H | 25.1102 | 10.0983 | 0.5139 |
| H | 25.7871 | 11.6149 | 1.2412 |
| H | 23.2707 | -4.0609 | -5.6114 |
| H | 23.2354 | -2.4737 | -6.4742 |
| H | 23.1263 | -4.0209 | -7.4131 |
| H | 29.7260 | -0.7881 | -11.3740 |
| H | 29.8337 | -0.8236 | -9.5700 |
| H | 29.7872 | -2.3735 | -10.5094 |
| H | 7.7920 | 3.4028 | 11.7692 |
| H | 8.3196 | 4.3285 | 10.3087 |
| H | 6.6494 | 3.6069 | 10.3855 |
| H | 9.0110 | -0.3774 | 3.3929 |
| H | 10.0802 | -0.5088 | 4.8454 |
| H | 8.4552 | -1.3100 | 4.8387 |
| H | 8.8649 | -12.9446 | -0.0029 |
| H | 8.9906 | -11.4997 | -1.0730 |
| H | 8.4535 | -13.0887 | -1.7585 |
| H | 14.6850 | -11.9426 | -7.1376 |
| H | 15.1578 | -11.9010 | -5.3939 |
| H | 14.4966 | -13.4248 | -6.1213 |
| H | 0.9657 | 3.3248 | 1.8936 |
| H | 1.0864 | 4.6168 | 3.1067 |
| C | 1.9256 | 2.7426 | 3.7493 |
| H | 5.6871 | -9.7105 | -11.0807 |
| H | 6.5095 | -9.3015 | -12.5908 |
| H | 5.2568 | -8.2323 | -11.9509 |
| H | 6.1841 | -6.5837 | -13.1150 |
| H | 7.3402 | -7.8440 | -13.6018 |
| C | 7.9654 | -5.8486 | -14.0990 |
| H | 4.8506 | -8.7757 | -9.2562 |
| H | 4.5348 | -7.3976 | -10.3315 |
| C | 4.3694 | -6.9419 | -8.2306 |
| H | 7.4355 | -10.9879 | -10.6415 |
| C | 9.5541 | -11.3323 | -10.9111 |
| H | 8.1071 | -10.3360 | -12.1518 |
| H | 1.6470 | 6.3345 | -1.6685 |
| H | 1.0754 | 4.7247 | -1.1790 |
| C | 2.3169 | 4.7017 | -2.9413 |
| H | 1.9630 | 8.2934 | 1.0442 |
| C | 3.5130 | 9.3004 | 2.1504 |
| H | 2.0348 | 7.8347 | 2.7578 |
| H | 0.8436 | 6.8328 | 0.3983 |
| H | 0.6879 | 6.1173 | 2.0082 |
| H | 0.4503 | 5.1166 | 0.5725 |
| H | 17.9849 | -19.6211 | 2.6227 |
| C | 18.9884 | -18.8096 | 0.9030 |
| H | 16.9913 | -19.6085 | 1.1479 |
| H | 16.5855 | -19.9876 | 3.8827 |
| H | 15.3441 | -20.0480 | 2.6254 |
| H | 14.8916 | -19.7078 | 4.3005 |
| H | 13.0772 | -18.7088 | 3.5650 |
| H | 13.7182 | -19.2288 | 1.9893 |
| C | 12.0815 | -17.8440 | 1.8487 |
| H | 17.1363 | -18.8588 | 5.7017 |
| H | 15.3694 | -18.7605 | 5.8854 |
| C | 16.4564 | -17.4794 | 7.2236 |
| H | 10.3860 | -4.5933 | 15.1465 |
| H | 11.7314 | -5.7184 | 15.4404 |
| C | 10.4375 | -6.2624 | 13.7930 |
| H | 11.0481 | -3.1940 | 16.3320 |
| H | 12.5051 | -2.3680 | 16.8966 |
| H | 12.4978 | -4.1268 | 16.7182 |
| H | 10.3796 | -1.5978 | 15.4336 |
| C | 10.4127 | 0.2697 | 14.3652 |
| H | 11.7174 | -0.5249 | 15.9002 |
| H | 14.6754 | -4.0720 | 16.5688 |
| H | 14.4235 | -2.3237 | 16.7762 |
| C | 16.4148 | -2.8506 | 16.1607 |
| H | 31.7527 | -8.2763 | -5.2425 |
| C | 29.8556 | -8.9291 | -6.0554 |
| H | 31.1530 | -9.8175 | -4.5881 |
| H | 33.1563 | -8.4090 | -3.5559 |
| H | 32.3879 | -9.9412 | -3.1211 |
| H | 33.2844 | -9.0250 | -1.9046 |
| H | 32.3008 | -9.8264 | -0.4250 |
| H | 31.2916 | -10.8969 | -1.4235 |
| C | 30.6698 | -10.7339 | 0.6429 |
| H | 33.6050 | -6.4147 | -2.7183 |
| H | 33.5845 | -7.2476 | -1.1476 |
| C | 33.5118 | -5.1035 | -0.9987 |
| H | 30.1469 | 8.1152 | 7.9729 |
| H | 28.8609 | 9.2696 | 8.3906 |
| C | 29.1295 | 8.9446 | 6.2680 |
| H | 27.8239 | 6.8368 | 12.1373 |
| C | 25.7017 | 7.0179 | 12.4266 |
| H | 27.1232 | 8.3090 | 11.4260 |
| H | 30.1823 | 4.8890 | 10.8922 |
| H | 30.9402 | 5.5901 | 9.4443 |
| C | 30.8571 | 3.4418 | 9.4344 |
| H | 29.5438 | 6.9701 | 11.2769 |
| H | 29.0791 | 8.4472 | 10.4245 |
| H | 30.3870 | 7.4380 | 9.7956 |
| H | 22.4323 | 1.0915 | -16.8780 |
| H | 21.0307 | 2.1147 | -17.2645 |
| C | 20.5328 | 0.0556 | -16.8953 |
| H | 23.4870 | 3.0452 | -16.6342 |
| H | 23.1610 | 4.7192 | -16.1664 |
| H | 21.9638 | 3.8127 | -17.0973 |
| H | 24.7870 | 2.8615 | -15.2110 |
| C | 25.7140 | 3.1369 | -13.2903 |
| H | 24.6116 | 4.5046 | -14.5532 |
| H | 20.4941 | 4.9279 | -16.4687 |
| H | 21.5134 | 6.0374 | -15.5240 |
| C | 19.3876 | 6.2371 | -15.1713 |
| H | 20.0027 | 17.8322 | -3.7681 |
| H | 18.6085 | 17.9429 | -4.8663 |
| C | 20.1301 | 16.5757 | -5.5258 |
| H | 18.6164 | 18.9509 | -2.4064 |
| H | 16.8930 | 19.1022 | -2.0405 |
| H | 17.4259 | 18.8813 | -3.7113 |
| H | 19.0059 | 18.4035 | -0.5969 |
| C | 18.7796 | 17.4202 | 1.3023 |
| H | 17.3411 | 18.4148 | 0.0287 |
| H | 15.4227 | 18.0635 | -4.1835 |
| H | 15.1332 | 18.4397 | -2.4694 |
| C | 13.5962 | 17.2370 | -3.3697 |
| H | 9.7032 | -5.7731 | 13.1231 |
| C | 9.7191 | -7.2807 | 14.6766 |
| H | 11.1591 | -6.7931 | 13.1332 |
| H | 8.9856 | -6.8020 | 15.3346 |
| H | 10.4217 | -7.8318 | 15.3125 |
| H | 9.1828 | -8.0193 | 14.0697 |
| H | 16.6395 | -1.7874 | 15.9429 |
| H | 16.9219 | -3.4447 | 15.3697 |
| C | 16.9948 | -3.2222 | 17.5244 |
| H | 16.5282 | -2.6490 | 18.3333 |
| H | 18.0719 | -3.0239 | 17.5612 |
| H | 16.8506 | -4.2853 | 17.7493 |
| H | 9.6858 | -0.1122 | 13.6208 |
| H | 11.1309 | 0.9062 | 13.8036 |
| C | 9.6797 | 1.1217 | 15.4004 |
| H | 8.9512 | 0.5316 | 15.9682 |
| H | 9.1328 | 1.9411 | 14.9205 |
| H | 10.3729 | 1.5690 | 16.1219 |
| H | 10.3916 | -10.9067 | -11.4995 |
| C | 9.2826 | -12.7614 | -11.3750 |
| H | 9.8908 | -11.3457 | -9.8510 |
| H | 8.9626 | -12.7938 | -12.4225 |
| H | 8.4996 | -13.2408 | -10.7766 |
| H | 10.1831 | -13.3805 | -11.2895 |
| H | 12.2849 | -17.6911 | 0.7695 |
| H | 11.7757 | -16.8546 | 2.2515 |
| C | 10.9304 | -18.8354 | 2.0105 |
| H | 11.1881 | -19.8231 | 1.6117 |
| H | 10.0374 | -18.4917 | 1.4761 |
| H | 10.6533 | -18.9677 | 3.0624 |
| C | 2.9945 | -7.5328 | -7.9313 |
| H | 4.2703 | -5.8661 | -8.4825 |
| H | 5.0007 | -6.9858 | -7.3171 |
| H | 2.3473 | -7.5329 | -8.8185 |
| H | 2.4739 | -6.9523 | -7.1600 |
| H | 3.0716 | -8.5679 | -7.5668 |
| H | 2.6779 | 1.9998 | 3.4052 |
| C | 0.6511 | 2.0167 | 4.1718 |
| H | 2.3721 | 3.2498 | 4.6290 |
| H | 0.2745 | 1.3569 | 3.3706 |
| H | -0.1543 | 2.7163 | 4.4224 |
| H | 0.8262 | 1.3906 | 5.0533 |
| C | 2.6887 | 10.4018 | 2.8124 |
| H | 3.9400 | 9.6798 | 1.2019 |
| H | 4.3744 | 9.0386 | 2.8022 |
| H | 1.8119 | 10.6777 | 2.2100 |
| H | 3.2848 | 11.3098 | 2.9527 |
| H | 2.3239 | 10.0919 | 3.8000 |
| C | 12.7878 | 18.3586 | -4.0199 |
| H | 13.1677 | 17.0057 | -2.3733 |
| H | 13.4955 | 16.3103 | -3.9751 |
| H | 12.8497 | 19.2887 | -3.4438 |
| H | 11.7277 | 18.0889 | -4.0902 |
| H | 13.1387 | 18.5760 | -5.0350 |
| H | 18.1444 | 16.6410 | 1.7764 |
| C | 18.8365 | 18.6498 | 2.2073 |
| H | 19.7947 | 16.9828 | 1.2134 |
| H | 17.8412 | 19.0764 | 2.3756 |
| H | 19.4651 | 19.4390 | 1.7797 |
| H | 19.2543 | 18.3963 | 3.1885 |
| H | 24.7821 | 7.2007 | 11.8300 |
| C | 25.7109 | 7.9443 | 13.6415 |
| H | 25.6454 | 5.9642 | 12.7675 |
| H | 25.6998 | 8.9998 | 13.3467 |
| H | 26.5977 | 7.7858 | 14.2652 |
| H | 24.8321 | 7.7720 | 14.2735 |
| H | 31.1054 | 3.4092 | 8.3542 |
| H | 30.1241 | 2.6244 | 9.6114 |
| C | 32.1198 | 3.1779 | 10.2525 |
| H | 32.8754 | 3.9554 | 10.0931 |
| H | 32.5746 | 2.2199 | 9.9750 |
| H | 31.9073 | 3.1407 | 11.3268 |
| H | 32.9461 | -4.2469 | -1.4262 |
| C | 35.0103 | -4.8276 | -1.1068 |
| H | 33.2245 | -5.1626 | 0.0707 |
| H | 35.3260 | -4.7012 | -2.1485 |
| H | 35.6039 | -5.6436 | -0.6795 |
| H | 35.2808 | -3.9117 | -0.5687 |
| H | 29.5752 | -10.8982 | 0.5321 |
| H | 30.7966 | -10.0324 | 1.4915 |
| C | 31.3501 | -12.0609 | 0.9746 |
| H | 31.1972 | -12.8037 | 0.1832 |
| H | 30.9512 | -12.4865 | 1.9025 |
| H | 32.4309 | -11.9403 | 1.1083 |
| H | 15.5297 | -16.9307 | 7.4871 |
| H | 17.2706 | -16.7224 | 7.1961 |
| C | 16.7596 | -18.5099 | 8.3097 |
| H | 15.9720 | -19.2685 | 8.3793 |
| H | 16.8397 | -18.0324 | 9.2930 |
| H | 17.7043 | -19.0324 | 8.1212 |
| C | 7.2027 | -5.7120 | -15.4151 |
| H | 8.9987 | -6.1933 | -14.3052 |
| H | 8.0587 | -4.8482 | -13.6221 |
| H | 7.1004 | -6.6754 | -15.9268 |
| H | 7.7202 | -5.0314 | -16.1009 |
| H | 6.1932 | -5.3133 | -15.2593 |
| H | 18.8160 | -18.0568 | 0.1034 |
| C | 19.4500 | -20.1230 | 0.2739 |
| H | 19.7982 | -18.4033 | 1.5425 |
| H | 18.7017 | -20.5276 | -0.4169 |
| H | 19.6460 | -20.8889 | 1.0328 |
| H | 20.3772 | -19.9821 | -0.2935 |
| H | 29.3626 | -7.9551 | -6.2634 |
| H | 29.0463 | -9.6378 | -5.7880 |
| C | 30.5616 | -9.4202 | -7.3184 |
| H | 31.3274 | -8.7122 | -7.6554 |
| H | 29.8499 | -9.5479 | -8.1421 |
| H | 31.0532 | -10.3862 | -7.1584 |
| H | 28.0914 | 9.1086 | 5.9049 |
| H | 29.5778 | 8.1818 | 5.6003 |
| C | 29.9194 | 10.2472 | 6.1528 |
| H | 29.4711 | 11.0474 | 6.7531 |
| H | 29.9519 | 10.5975 | 5.1149 |
| H | 30.9550 | 10.1245 | 6.4892 |
| C | 21.0564 | 17.5874 | -6.1984 |
| H | 19.4447 | 16.1442 | -6.2832 |
| H | 20.7324 | 15.7284 | -5.1317 |
| H | 20.4983 | 18.4336 | -6.6146 |
| H | 21.6104 | 17.1248 | -7.0234 |
| H | 21.7933 | 17.9927 | -5.4958 |
| C | 1.2713 | 5.0552 | -3.9961 |
| H | 2.4102 | 3.5995 | -2.8747 |
| H | 3.3086 | 5.0784 | -3.2762 |
| H | 0.2602 | 4.7313 | -3.6981 |
| H | 1.4972 | 4.5729 | -4.9536 |
| H | 1.2259 | 6.1343 | -4.1798 |
| C | 20.8181 | -0.4861 | -18.2947 |
| H | 19.4554 | 0.3030 | -16.8091 |
| H | 20.7319 | -0.7391 | -16.1431 |
| H | 20.6173 | 0.2641 | -19.0679 |
| H | 20.1892 | -1.3566 | -18.5147 |
| H | 21.8621 | -0.8002 | -18.4040 |
| H | 25.4499 | 3.5028 | -12.2748 |
| C | 27.0216 | 3.7868 | -13.7398 |
| H | 25.8610 | 2.0424 | -13.1979 |
| H | 26.9373 | 4.8790 | -13.7817 |
| H | 27.3245 | 3.4424 | -14.7348 |
| H | 27.8375 | 3.5490 | -13.0479 |
| C | 19.3335 | 7.4558 | -16.0907 |
| H | 19.3897 | 6.5741 | -14.1114 |
| H | 18.4682 | 5.6328 | -15.3055 |
| H | 20.1987 | 8.1127 | -15.9440 |
| H | 18.4346 | 8.0530 | -15.9007 |
| H | 19.3164 | 7.1659 | -17.1473 |

k) (**OMe-cube)≡**(**OMe-cube)**≡(**OMe-cube)** (Type 1 conformer, 1 and 2 position of middle cage is interlocked)

| **atom** | **x** | **y** | **z** |
| --- | --- | --- | --- |
| C | -8.5169 | -3.0411 | -0.7310 |
| C | -9.5223 | -2.2041 | 1.4850 |
| C | -10.5031 | -1.4032 | -0.7536 |
| C | -9.9008 | -2.6858 | 0.0021 |
| C | -7.4554 | -2.7550 | 0.3187 |
| C | -8.0075 | -2.2588 | 1.5211 |
| C | -7.1732 | -1.8438 | 2.5564 |
| C | -5.7869 | -1.9638 | 2.4233 |
| N | -3.8076 | -2.5801 | 1.2142 |
| C | -5.2362 | -2.4757 | 1.2363 |
| C | -6.0784 | -2.8689 | 0.1734 |
| C | -9.9848 | -0.7600 | 1.5354 |
| C | -10.5053 | -0.3155 | 0.3014 |
| C | -10.9232 | 1.0049 | 0.1551 |
| C | -10.8215 | 1.8928 | 1.2309 |
| N | -10.2399 | 2.4283 | 3.4978 |
| C | -10.2988 | 1.4497 | 2.4588 |
| C | -9.8940 | 0.1067 | 2.6166 |
| C | -9.4810 | -1.0932 | -1.8313 |
| C | -8.4090 | -2.0115 | -1.8404 |
| C | -7.3824 | -1.8730 | -2.7721 |
| C | -7.4087 | -0.8238 | -3.6962 |
| N | -8.4825 | 1.1239 | -4.6720 |
| C | -8.4628 | 0.1047 | -3.6648 |
| C | -9.5040 | -0.0267 | -2.7212 |
| C | 7.8691 | -4.2980 | 1.1180 |
| C | 9.0189 | -3.7398 | -1.1185 |
| C | 10.1097 | -3.0374 | 1.1038 |
| C | 9.2973 | -4.2217 | 0.3879 |
| C | 6.8786 | -3.9211 | 0.0321 |
| C | 7.5114 | -3.5791 | -1.1835 |
| C | 6.7522 | -3.1210 | -2.2575 |
| C | 5.3596 | -3.0400 | -2.1468 |
| N | 3.3008 | -3.3462 | -0.9215 |
| C | 4.7317 | -3.4018 | -0.9434 |
| C | 5.4968 | -3.8474 | 0.1551 |
| C | 9.6837 | -2.3782 | -1.2020 |
| C | 10.2354 | -1.9711 | 0.0321 |
| C | 10.7786 | -0.6950 | 0.1649 |
| C | 10.8279 | 0.1609 | -0.9402 |
| N | 10.4581 | 0.6607 | -3.2654 |
| C | 10.3134 | -0.2602 | -2.1793 |
| C | 9.7331 | -1.5401 | -2.3087 |
| C | 9.1780 | -2.5543 | 2.2001 |
| C | 7.9282 | -3.2096 | 2.1731 |
| C | 6.9142 | -2.8040 | 3.0385 |
| C | 7.1506 | -1.7842 | 3.9673 |
| N | 8.6247 | -0.1686 | 5.0279 |
| C | 8.4071 | -1.1571 | 4.0131 |
| C | 9.4289 | -1.5442 | 3.1204 |
| C | 10.2723 | 10.6571 | -13.0395 |
| C | 11.2262 | 8.3938 | -12.2759 |
| C | 9.0898 | 8.4691 | -13.7072 |
| C | 10.5341 | 9.1456 | -13.5125 |
| C | 10.7708 | 10.6913 | -11.6054 |
| C | 11.2773 | 9.4437 | -11.1818 |
| C | 11.6967 | 9.2712 | -9.8643 |
| C | 11.6586 | 10.3473 | -8.9721 |
| N | 11.2241 | 12.6593 | -8.4465 |
| C | 11.1672 | 11.5931 | -9.3988 |
| C | 10.7084 | 11.7632 | -10.7227 |
| C | 10.2263 | 7.3197 | -11.8854 |
| C | 9.0316 | 7.4009 | -12.6334 |
| C | 7.9600 | 6.5673 | -12.3222 |
| C | 8.0848 | 5.6245 | -11.2968 |
| N | 9.3041 | 4.5230 | -9.5496 |
| C | 9.2834 | 5.5290 | -10.5660 |
| C | 10.3661 | 6.3808 | -10.8704 |
| C | 8.1118 | 9.5912 | -13.4108 |
| C | 8.7618 | 10.7925 | -13.0547 |
| C | 8.0134 | 11.9223 | -12.7330 |
| C | 6.6169 | 11.8673 | -12.7600 |
| N | 4.5417 | 10.7202 | -13.1593 |
| C | 5.9693 | 10.6677 | -13.1066 |
| C | 6.7237 | 9.5228 | -13.4416 |
| C | 10.0828 | 4.5917 | -8.5128 |
| C | 10.0098 | 3.5458 | -7.4719 |
| C | 9.3047 | 2.3480 | -7.6589 |
| C | 9.2123 | 1.4196 | -6.6184 |
| C | 9.8305 | 1.6528 | -5.3604 |
| C | 10.5643 | 2.8285 | -5.1845 |
| C | 10.6470 | 3.7621 | -6.2255 |
| C | 9.6454 | 0.6670 | -4.2735 |
| C | -7.2395 | 11.3222 | -13.9234 |
| C | -8.4628 | 9.2148 | -13.0967 |
| C | -9.3585 | 11.5454 | -12.4765 |
| C | -8.6962 | 10.6971 | -13.6682 |
| C | -6.2824 | 10.2057 | -13.5422 |
| C | -6.9548 | 9.0673 | -13.0477 |
| C | -6.2269 | 7.9880 | -12.5514 |
| C | -4.8292 | 8.0147 | -12.5793 |
| N | -2.7329 | 9.0644 | -13.1445 |
| C | -4.1609 | 9.1401 | -13.0922 |
| C | -4.8932 | 10.2452 | -13.5758 |
| C | -9.0322 | 9.2637 | -11.6905 |
| C | -9.5240 | 10.5428 | -11.3511 |
| C | -10.0449 | 10.7743 | -10.0805 |
| C | -10.0736 | 9.7433 | -9.1354 |
| N | -9.6546 | 7.4579 | -8.4616 |
| C | -9.5713 | 8.4745 | -9.4695 |
| C | -9.0610 | 8.2298 | -10.7634 |
| C | -8.2911 | 12.5592 | -12.1060 |
| C | -7.1318 | 12.4394 | -12.9024 |
| C | -6.0340 | 13.2631 | -12.6638 |
| C | -6.0820 | 14.2157 | -11.6412 |
| N | -7.2206 | 15.3468 | -9.8393 |
| C | -7.2319 | 14.3244 | -10.8406 |
| C | -8.3458 | 13.4901 | -11.0759 |
| C | -1.9839 | 10.0887 | -12.8809 |
| C | -0.5117 | 9.9587 | -12.9306 |
| C | 0.1299 | 8.7160 | -12.8981 |
| C | 1.5281 | 8.6512 | -12.8917 |
| C | 2.3265 | 9.8223 | -12.9203 |
| C | 1.6839 | 11.0649 | -12.9799 |
| C | 0.2870 | 11.1294 | -12.9830 |
| C | -8.7347 | 2.3549 | -4.3752 |
| C | -8.7742 | 3.3854 | -5.4349 |
| C | -8.1558 | 3.2122 | -6.6745 |
| C | -8.1880 | 4.2501 | -7.6148 |
| C | -8.8161 | 5.4852 | -7.3443 |
| C | -9.4590 | 5.6553 | -6.1113 |
| C | -9.4468 | 4.6128 | -5.1797 |
| C | 1.1750 | -2.9074 | 0.1040 |
| C | -8.7314 | 6.5538 | -8.3632 |
| C | 3.7984 | 9.6996 | -12.8592 |
| C | 2.6533 | -2.9519 | 0.1284 |
| C | 10.0826 | 23.2408 | -0.8403 |
| C | 11.0498 | 22.4671 | 1.4164 |
| C | 12.0664 | 21.6047 | -0.7856 |
| C | 11.4536 | 22.9065 | -0.0745 |
| C | 9.0100 | 23.0406 | 0.2143 |
| C | 9.5400 | 22.6084 | 1.4489 |
| C | 8.6863 | 22.3263 | 2.5126 |
| C | 7.3037 | 22.4683 | 2.3575 |
| N | 5.3560 | 23.0419 | 1.0557 |
| C | 6.7772 | 22.8914 | 1.1245 |
| C | 7.6390 | 23.1939 | 0.0487 |
| C | 11.4279 | 20.9983 | 1.4864 |
| C | 11.9715 | 20.5226 | 0.2735 |
| C | 12.2984 | 19.1752 | 0.1363 |
| C | 12.1214 | 18.2993 | 1.2121 |
| N | 11.4872 | 17.8313 | 3.4903 |
| C | 11.5873 | 18.7761 | 2.4215 |
| C | 11.2329 | 20.1354 | 2.5581 |
| C | 11.0861 | 21.2936 | -1.9031 |
| C | 9.9750 | 22.1639 | -1.9017 |
| C | 8.9195 | 21.9485 | -2.7851 |
| C | 8.9775 | 20.8904 | -3.6975 |
| N | 10.0966 | 19.0066 | -4.7041 |
| C | 10.0950 | 20.0375 | -3.7115 |
| C | 11.1615 | 20.2446 | -2.8116 |
| C | 10.5606 | 17.8227 | -4.4532 |
| C | 10.5209 | 16.7784 | -5.4986 |
| C | 10.0772 | 13.9379 | 8.5792 |
| C | 10.2082 | 14.9773 | 7.5364 |
| C | 9.7342 | 5.3507 | 8.4607 |
| C | 9.7065 | 4.1752 | 7.5678 |
| C | 9.4703 | 16.1660 | 7.5749 |
| C | 9.5937 | 17.0976 | 6.5392 |
| C | 10.4565 | 16.8725 | 5.4366 |
| C | 11.2123 | 15.6953 | 5.4104 |
| C | 11.0879 | 14.7629 | 6.4456 |
| C | 10.5151 | 17.8703 | 4.3481 |
| C | -7.6650 | 10.1762 | 13.7239 |
| C | -8.4962 | 12.4983 | 12.9815 |
| C | -9.7569 | 10.3828 | 12.2404 |
| C | -8.9884 | 11.0537 | 13.4790 |
| C | -6.5260 | 11.1183 | 13.3791 |
| C | -6.9826 | 12.3948 | 12.9850 |
| C | -6.0692 | 13.3754 | 12.6047 |
| C | -4.6981 | 13.1014 | 12.6261 |
| N | -2.8271 | 11.6524 | 13.0612 |
| C | -4.2443 | 11.8275 | 13.0105 |
| C | -5.1669 | 10.8266 | 13.3846 |
| C | -8.9853 | 12.5851 | 11.5457 |
| C | -9.6404 | 11.4068 | 11.1284 |
| C | -10.0548 | 11.2682 | 9.8055 |
| C | -9.8630 | 12.3165 | 8.9001 |
| N | -9.1281 | 14.5454 | 8.3537 |
| C | -9.2313 | 13.4991 | 9.3225 |
| C | -8.7859 | 13.6332 | 10.6548 |
| C | -8.9209 | 9.1631 | 11.8997 |
| C | -7.7612 | 9.0639 | 12.6980 |
| C | -6.8479 | 8.0369 | 12.4725 |
| C | -7.0803 | 7.1045 | 11.4560 |
| N | -8.4100 | 6.1922 | 9.6678 |
| C | -8.2354 | 7.2060 | 10.6604 |
| C | -9.1709 | 8.2363 | 10.8951 |
| C | -2.2602 | 10.5107 | 12.8088 |
| C | -9.0140 | 6.4342 | 8.5407 |
| C | -0.7888 | 10.3937 | 12.8795 |
| C | -9.1588 | 5.3578 | 7.5414 |
| C | 0.0468 | 11.5162 | 12.9100 |
| C | 1.4355 | 11.3523 | 12.9293 |
| C | 2.0327 | 10.0664 | 12.9212 |
| C | 1.1978 | 8.9437 | 12.9177 |
| C | -0.1921 | 9.1075 | 12.8957 |
| C | -8.8526 | 4.0211 | 7.8380 |
| C | -8.9423 | 3.0451 | 6.8425 |
| C | -9.3787 | 3.3698 | 5.5304 |
| C | -9.7226 | 4.6919 | 5.2438 |
| C | -9.6039 | 5.6749 | 6.2354 |
| C | -7.3916 | 23.9433 | -1.7510 |
| C | -8.5330 | 23.3003 | 0.4642 |
| C | -6.3194 | 24.6133 | 0.4909 |
| C | -7.7247 | 24.4756 | -0.2736 |
| C | -8.0205 | 22.5619 | -1.7992 |
| C | -8.6272 | 22.2022 | -0.5766 |
| C | -9.1863 | 20.9359 | -0.4204 |
| C | -9.1637 | 20.0257 | -1.4819 |
| N | -8.6173 | 19.4162 | -3.7486 |
| C | -8.5611 | 20.3861 | -2.6997 |
| C | -7.9892 | 21.6661 | -2.8612 |
| C | -7.6124 | 22.8654 | 1.5901 |
| C | -6.3990 | 23.5860 | 1.6034 |
| C | -5.4204 | 23.2870 | 2.5482 |
| C | -5.6365 | 22.2684 | 3.4815 |
| N | -6.9974 | 20.5425 | 4.4687 |
| C | -6.8398 | 21.5415 | 3.4581 |
| C | -7.8398 | 21.8496 | 2.5109 |
| C | -5.2914 | 24.1764 | -0.5376 |
| C | -5.8809 | 23.8091 | -1.7667 |
| C | -5.0861 | 23.3339 | -2.8074 |
| C | -3.7012 | 23.2348 | -2.6416 |
| N | -1.6893 | 23.5139 | -1.3422 |
| C | -3.1161 | 23.5944 | -1.4152 |
| C | -3.9183 | 24.0663 | -0.3539 |
| C | -7.7699 | 18.4277 | 5.3121 |
| C | -7.6439 | 19.2705 | -4.5958 |
| C | -1.0772 | 23.1946 | -0.2459 |
| C | -7.7414 | 18.2457 | -5.6555 |
| C | 0.3995 | 23.1312 | -0.2194 |
| C | -8.6762 | 17.2056 | -5.6019 |
| C | -8.6954 | 16.2323 | -6.6060 |
| C | -7.7845 | 16.2674 | -7.6920 |
| C | -6.8659 | 17.3210 | -7.7587 |
| C | -6.8463 | 18.2941 | -6.7539 |
| C | -6.9587 | 18.4434 | 6.4524 |
| C | -7.0866 | 17.4354 | 7.4137 |
| C | -8.0287 | 16.3858 | 7.2686 |
| C | -8.8562 | 16.3832 | 6.1403 |
| C | -8.7257 | 17.3892 | 5.1765 |
| C | 1.1647 | 23.0694 | -1.3895 |
| C | 2.5576 | 22.9692 | -1.3079 |
| C | 3.2283 | 22.9311 | -0.0592 |
| C | 2.4648 | 23.0162 | 1.1105 |
| C | 1.0718 | 23.1129 | 1.0290 |
| C | -8.0901 | 15.3227 | 8.2932 |
| C | 3.5060 | 9.9575 | 12.8837 |
| C | 4.6977 | 22.7773 | -0.0308 |
| C | 10.3745 | 4.1841 | 6.3339 |
| C | 10.1950 | 3.1175 | 5.4520 |
| C | 9.3438 | 2.0174 | 5.7637 |
| C | 8.7415 | 1.9846 | 7.0206 |
| C | 8.9324 | 3.0546 | 7.9155 |
| C | 11.2874 | 15.5974 | -5.3309 |
| C | 11.2480 | 14.5584 | -6.2672 |
| C | 10.4341 | 14.6706 | -7.3997 |
| C | 9.6825 | 15.8599 | -7.5772 |
| C | 9.7256 | 16.9006 | -6.6439 |
| C | -7.8176 | 15.1871 | -8.6999 |
| C | 10.3211 | 13.5865 | -8.3986 |
| C | -9.4200 | 2.3115 | 4.5017 |
| C | 9.1199 | 0.9889 | 4.7266 |
| C | 0.4431 | -3.0915 | -1.0740 |
| C | -0.9527 | -3.0081 | -1.0513 |
| C | -1.6651 | -2.7441 | 0.1430 |
| C | -0.9339 | -2.5683 | 1.3252 |
| C | 0.4614 | -2.6507 | 1.3037 |
| C | -3.1407 | -2.6459 | 0.1082 |
| C | 9.7686 | 8.1085 | 13.1996 |
| C | 11.0193 | 10.2551 | 12.5241 |
| C | 8.8709 | 10.4028 | 13.9334 |
| C | 10.2182 | 9.5566 | 13.7283 |
| C | 10.3709 | 8.0196 | 11.8107 |
| C | 11.0467 | 9.2009 | 11.4341 |
| C | 11.6090 | 9.3069 | 10.1648 |
| C | 11.5237 | 8.2361 | 9.2666 |
| N | 10.8522 | 5.9597 | 8.7360 |
| C | 10.8573 | 7.0621 | 9.6477 |
| C | 10.2768 | 6.9503 | 10.9298 |
| C | 10.1228 | 11.4044 | 12.0993 |
| C | 8.9347 | 11.4713 | 12.8580 |
| C | 7.9541 | 12.4081 | 12.5380 |
| C | 8.1624 | 13.3057 | 11.4862 |
| N | 9.5095 | 14.2243 | 9.7077 |
| C | 9.3513 | 13.2446 | 10.7388 |
| C | 10.3404 | 12.2873 | 11.0485 |
| C | 7.7605 | 9.4271 | 13.5872 |
| C | 8.2568 | 8.1917 | 13.1192 |
| C | 7.3763 | 7.2302 | 12.6282 |
| C | 5.9985 | 7.4710 | 12.6448 |
| N | 4.0828 | 8.8351 | 13.1863 |
| C | 5.5050 | 8.6895 | 13.1430 |
| C | 6.3935 | 9.6749 | 13.6220 |
| C | -7.6407 | 19.4370 | 4.2410 |
| O | 10.7973 | 3.0037 | 4.2167 |
| O | 8.2102 | 2.9626 | 9.1019 |
| C | 11.6387 | 4.0936 | 3.7665 |
| C | 5.5973 | 1.1411 | 9.5421 |
| O | -9.5700 | 15.1618 | -6.6529 |
| C | -10.5444 | 15.0456 | -5.5877 |
| O | -5.9839 | 19.3743 | -6.7158 |
| C | -5.0224 | 19.5032 | -7.7912 |
| O | 8.9330 | 15.8696 | -8.7396 |
| O | 12.0525 | 15.5958 | -4.1797 |
| C | 8.0984 | 17.0268 | -8.9879 |
| C | 12.8175 | 14.4019 | -3.8834 |
| O | -8.6353 | 1.7105 | 7.0030 |
| O | -9.9046 | 7.0119 | 6.0481 |
| C | -10.2360 | 7.4434 | 4.7047 |
| C | -8.0667 | 1.2889 | 8.2685 |
| O | -6.3385 | 17.3485 | 8.5737 |
| C | -5.3262 | 18.3601 | 8.7957 |
| O | -9.4844 | 17.4840 | 4.0247 |
| C | -10.4611 | 16.4434 | 3.7765 |
| O | -10.0596 | 4.6460 | -3.9456 |
| C | -10.8679 | 5.8026 | -3.6183 |
| O | -7.5399 | 4.1670 | -8.8434 |
| C | -7.8148 | 2.9574 | -9.5956 |
| O | 11.7804 | 13.5681 | 6.5208 |
| O | 8.9151 | 18.3006 | 6.4719 |
| C | 12.7092 | 13.2634 | 5.4519 |
| C | 7.9977 | 18.6141 | 7.5478 |
| O | 8.5621 | 0.2064 | -6.6942 |
| C | 7.6737 | -0.0208 | -7.8190 |
| O | 11.3301 | 4.9634 | -6.1507 |
| C | 11.8965 | 5.3334 | -4.8696 |
| O | 2.2642 | 7.4816 | -12.8624 |
| O | -0.4516 | 12.2977 | -13.0375 |
| C | 0.2753 | 13.5501 | -13.0428 |
| C | 1.5370 | 6.2333 | -12.7537 |
| O | 1.2748 | -2.4696 | 2.4076 |
| O | -1.7499 | -3.1140 | -2.1836 |
| C | 0.6472 | -2.4806 | 3.7127 |
| C | -1.2216 | -3.8953 | -3.2842 |
| O | 3.4104 | 22.8978 | -2.3942 |
| C | 2.8168 | 22.9127 | -3.7152 |
| O | 0.2199 | 23.1996 | 2.1149 |
| C | 0.8123 | 23.1668 | 3.4360 |
| O | 2.3540 | 12.3859 | 12.9584 |
| C | 1.8405 | 13.7395 | 12.9245 |
| O | -1.1096 | 8.0736 | 12.8913 |
| C | -0.5958 | 6.7197 | 12.8454 |
| C | -8.4900 | -4.4947 | -1.2436 |
| C | -10.1800 | -3.0785 | 2.5732 |
| C | -11.9103 | -1.6734 | -1.3292 |
| C | -10.8751 | -3.8555 | 0.0102 |
| H | -7.5863 | -1.4146 | 3.4687 |
| H | -5.1234 | -1.6531 | 3.2391 |
| H | -5.6531 | -3.2601 | -0.7497 |
| H | -11.3352 | 1.3496 | -0.7956 |
| H | -11.1393 | 2.9300 | 1.1238 |
| H | -9.5265 | -0.2552 | 3.5798 |
| H | -6.5579 | -2.5869 | -2.7927 |
| H | -6.6173 | -0.7162 | -4.4374 |
| H | -10.3165 | 0.6972 | -2.7040 |
| C | 7.5916 | -5.6961 | 1.7107 |
| C | 9.5620 | -4.7389 | -2.1616 |
| C | 11.4747 | -3.5103 | 1.6458 |
| C | 10.0427 | -5.5483 | 0.4393 |
| H | 7.2312 | -2.8192 | -3.1890 |
| H | 4.7622 | -2.6921 | -2.9883 |
| H | 5.0118 | -4.1503 | 1.0853 |
| H | 11.1589 | -0.3501 | 1.1255 |
| H | 11.2609 | 1.1566 | -0.8479 |
| H | 9.3420 | -1.8761 | -3.2708 |
| H | 5.9297 | -3.2661 | 2.9960 |
| H | 6.3649 | -1.4699 | 4.6531 |
| H | 10.4110 | -1.0682 | 3.1643 |
| C | 10.9788 | 11.6825 | -13.9501 |
| C | 12.6113 | 7.8221 | -12.6390 |
| C | 8.9049 | 7.8920 | -15.1279 |
| C | 11.3746 | 9.0732 | -14.7800 |
| H | 12.0444 | 8.3016 | -9.5128 |
| H | 11.9999 | 10.2255 | -7.9450 |
| H | 10.3159 | 12.7259 | -11.0438 |
| H | 7.0180 | 6.6469 | -12.8635 |
| H | 7.2551 | 4.9594 | -11.0606 |
| H | 11.3094 | 6.3016 | -10.3252 |
| H | 8.5141 | 12.8567 | -12.4692 |
| H | 6.0246 | 12.7477 | -12.5131 |
| H | 6.2247 | 8.6011 | -13.7454 |
| H | 10.8077 | 5.3986 | -8.2932 |
| H | 8.8310 | 2.1605 | -8.6256 |
| H | 11.0599 | 2.9972 | -4.2249 |
| H | 8.7767 | -0.0038 | -4.4255 |
| C | -7.0606 | 11.8088 | -15.3757 |
| C | -9.1404 | 8.1408 | -13.9745 |
| C | -10.6898 | 12.2018 | -12.8983 |
| C | -9.5568 | 10.7008 | -14.9244 |
| H | -6.7357 | 7.1216 | -12.1309 |
| H | -4.2540 | 7.1713 | -12.1992 |
| H | -4.3738 | 11.1125 | -13.9835 |
| H | -10.4361 | 11.7586 | -9.8167 |
| H | -10.4774 | 9.9202 | -8.1403 |
| H | -8.7052 | 7.2353 | -11.0375 |
| H | -5.1321 | 13.1709 | -13.2693 |
| H | -5.2305 | 14.8687 | -11.4549 |
| H | -9.2383 | 13.5827 | -10.4597 |
| H | -2.3348 | 11.0996 | -12.5960 |
| H | -0.4834 | 7.8120 | -12.8791 |
| H | 2.2978 | 11.9673 | -13.0248 |
| H | -8.9322 | 2.7543 | -3.3587 |
| H | -7.6413 | 2.2711 | -6.8994 |
| H | -9.9655 | 6.5982 | -5.9083 |
| H | -7.8257 | 6.4767 | -8.9993 |
| H | 4.1543 | 8.7029 | -12.5359 |
| H | 3.0999 | -2.6174 | 1.0850 |
| C | 10.0758 | 24.6678 | -1.4282 |
| C | 11.7566 | 23.3231 | 2.4881 |
| C | 13.5036 | 21.8379 | -1.2932 |
| C | 12.4196 | 24.0830 | -0.0864 |
| H | 9.0912 | 21.9984 | 3.4717 |
| H | 6.6305 | 22.2485 | 3.1850 |
| H | 7.2373 | 23.5611 | -0.8976 |
| H | 12.6836 | 18.7897 | -0.8066 |
| H | 12.3883 | 17.2476 | 1.1164 |
| H | 10.8203 | 20.5012 | 3.4969 |
| H | 8.0388 | 22.5886 | -2.7652 |
| H | 8.1578 | 20.7180 | -4.3934 |
| H | 12.0420 | 19.5994 | -2.8364 |
| H | 10.9934 | 17.4810 | -3.4925 |
| H | 10.4870 | 12.9519 | 8.2846 |
| H | 8.7338 | 5.6308 | 8.8405 |
| H | 8.8085 | 16.3418 | 8.4262 |
| H | 11.8933 | 15.5312 | 4.5717 |
| H | 9.6780 | 18.5947 | 4.3561 |
| C | -7.5813 | 9.6439 | 15.1708 |
| C | -9.0325 | 13.6358 | 13.8740 |
| C | -11.2189 | 10.0284 | 12.5834 |
| C | -9.8615 | 11.1346 | 14.7238 |
| H | -6.4148 | 14.3612 | 12.2921 |
| H | -3.9779 | 13.8676 | 12.3421 |
| H | -4.8122 | 9.8423 | 13.6841 |
| H | -10.5187 | 10.3454 | 9.4614 |
| H | -10.1948 | 12.2209 | 7.8671 |
| H | -8.3031 | 14.5538 | 10.9826 |
| H | -5.9516 | 7.9512 | 13.0898 |
| H | -6.3699 | 6.2993 | 11.2752 |
| H | -10.0895 | 8.2992 | 10.3081 |
| H | -2.7746 | 9.5737 | 12.5245 |
| H | -9.4295 | 7.4086 | 8.2219 |
| H | -0.4113 | 12.5079 | 12.9195 |
| H | 1.6557 | 7.9519 | 12.9333 |
| H | -8.5444 | 3.7681 | 8.8551 |
| H | -10.0826 | 4.9338 | 4.2409 |
| C | -7.9282 | 24.8885 | -2.8455 |
| C | -9.9130 | 23.7718 | 0.9689 |
| C | -6.0787 | 26.0453 | 1.0129 |
| C | -8.5017 | 25.7849 | -0.2919 |
| H | -9.6417 | 20.6442 | 0.5258 |
| H | -9.6052 | 19.0365 | -1.3694 |
| H | -7.5412 | 21.9529 | -3.8128 |
| H | -4.4849 | 23.8491 | 2.5686 |
| H | -4.8768 | 22.0302 | 4.2244 |
| H | -8.7858 | 21.3078 | 2.5123 |
| H | -5.5351 | 23.0367 | -3.7552 |
| H | -3.0727 | 22.8745 | -3.4548 |
| H | -3.4611 | 24.3480 | 0.5929 |
| H | -6.6985 | 19.8455 | -4.6104 |
| H | -1.5545 | 22.9445 | 0.7219 |
| H | -9.3796 | 17.1792 | -4.7662 |
| H | -6.1783 | 17.3613 | -8.6066 |
| H | -6.2358 | 19.2543 | 6.5669 |
| H | -9.5969 | 15.5868 | 6.0374 |
| H | 0.6495 | 23.1045 | -2.3521 |
| H | 2.9821 | 23.0080 | 2.0726 |
| H | -7.1979 | 15.2742 | 8.9460 |
| H | 4.0173 | 10.8888 | 12.5718 |
| H | 5.1298 | 22.4215 | -0.9861 |
| H | 11.0170 | 5.0270 | 6.0893 |
| H | 8.0928 | 1.1444 | 7.3151 |
| H | 11.8447 | 13.6522 | -6.1401 |
| H | 9.1506 | 17.8190 | -6.7838 |
| H | -8.3723 | 14.2858 | -8.3730 |
| H | 9.4257 | 13.6648 | -9.0457 |
| H | -8.7139 | 1.4816 | 4.6854 |
| H | 9.4049 | 1.3216 | 3.7066 |
| H | 0.9842 | -3.2965 | -2.0020 |
| H | -1.4786 | -2.3686 | 2.2501 |
| H | -3.5643 | -2.6332 | -0.9154 |
| C | 10.2524 | 6.9724 | 14.1269 |
| C | 12.4272 | 10.7184 | 12.9519 |
| C | 8.7504 | 10.9755 | 15.3594 |
| C | 11.0492 | 9.4699 | 15.0008 |
| H | 12.1208 | 10.2204 | 9.8602 |
| H | 11.9688 | 8.3112 | 8.2769 |
| H | 9.7786 | 6.0280 | 11.2324 |
| H | 7.0160 | 12.4429 | 13.0898 |
| H | 7.4052 | 14.0475 | 11.2361 |
| H | 11.2677 | 12.2497 | 10.4775 |
| H | 7.7477 | 6.2923 | 12.2198 |
| H | 5.3037 | 6.7206 | 12.2695 |
| H | 6.0138 | 10.6135 | 14.0322 |
| H | -8.1182 | 19.1441 | 3.2867 |
| H | 11.9666 | 3.7285 | 2.7834 |
| H | 11.0526 | 5.0114 | 3.6624 |
| H | 12.4892 | 4.2194 | 4.4419 |
| H | 4.5359 | 1.3834 | 9.6197 |
| H | 6.2156 | 1.9125 | 10.0213 |
| H | 5.8160 | 0.1473 | 9.9451 |
| H | -11.0938 | 14.1441 | -5.8867 |
| H | -10.0418 | 14.9010 | -4.6268 |
| H | -11.2023 | 15.9203 | -5.5761 |
| H | -4.4943 | 20.4223 | -7.5070 |
| H | -4.3431 | 18.6457 | -7.7997 |
| H | -5.5377 | 19.6263 | -8.7489 |
| H | 7.6294 | 16.7617 | -9.9440 |
| H | 7.3491 | 17.1333 | -8.1980 |
| H | 8.7156 | 17.9250 | -9.0884 |
| H | 13.2954 | 14.6746 | -2.9336 |
| H | 12.1509 | 13.5438 | -3.7552 |
| H | 13.5650 | 14.2266 | -4.6630 |
| H | -10.4154 | 8.5157 | 4.8524 |
| H | -9.3828 | 7.2841 | 4.0236 |
| H | -11.1349 | 6.9331 | 4.3497 |
| H | -7.7708 | 0.2525 | 8.0496 |
| H | -7.1820 | 1.8800 | 8.5234 |
| H | -8.8295 | 1.3299 | 9.0489 |
| H | -4.9060 | 18.0455 | 9.7596 |
| H | -4.5708 | 18.3229 | 8.0054 |
| H | -5.7879 | 19.3494 | 8.8727 |
| H | -10.8934 | 16.7681 | 2.8212 |
| H | -9.9666 | 15.4728 | 3.6747 |
| H | -11.2173 | 16.4356 | 4.5675 |
| H | -11.2703 | 5.5236 | -2.6353 |
| H | -10.2431 | 6.6988 | -3.5375 |
| H | -11.6710 | 5.9275 | -4.3492 |
| H | -7.2577 | 3.1322 | -10.5212 |
| H | -8.8876 | 2.8725 | -9.7863 |
| H | -7.4288 | 2.0772 | -9.0586 |
| H | 13.1103 | 12.2949 | 5.7757 |
| H | 12.1773 | 13.1721 | 4.5003 |
| H | 13.4991 | 14.0197 | 5.4065 |
| H | 7.6205 | 19.5971 | 7.2379 |
| H | 7.1903 | 17.8769 | 7.5865 |
| H | 8.5355 | 18.6812 | 8.4986 |
| H | 7.3034 | -1.0324 | -7.6077 |
| H | 6.8509 | 0.7022 | -7.8076 |
| H | 8.2260 | 0.0035 | -8.7610 |
| H | 12.3241 | 6.3184 | -5.0924 |
| H | 11.1097 | 5.4139 | -4.1112 |
| H | 12.6699 | 4.6171 | -4.5768 |
| H | -0.5427 | 14.2810 | -13.0786 |
| H | 0.8582 | 13.6566 | -12.1232 |
| H | 0.9049 | 13.6201 | -13.9352 |
| H | 2.3546 | 5.5012 | -12.7344 |
| H | 0.9630 | 6.2059 | -11.8224 |
| H | 0.8998 | 6.0880 | -13.6313 |
| H | 1.5196 | -2.4268 | 4.3794 |
| H | 0.0052 | -1.6030 | 3.8325 |
| H | 0.0960 | -3.4127 | 3.8671 |
| H | -2.0863 | -3.9523 | -3.9582 |
| H | -0.3867 | -3.3687 | -3.7550 |
| H | -0.9262 | -4.8908 | -2.9401 |
| H | 3.7043 | 22.8481 | -4.3577 |
| H | 2.1679 | 22.0425 | -3.8511 |
| H | 2.2800 | 23.8521 | -3.8800 |
| H | -0.0735 | 23.2513 | 4.0785 |
| H | 1.3276 | 22.2157 | 3.5987 |
| H | 1.4809 | 24.0223 | 3.5737 |
| H | 2.7664 | 14.3281 | 12.9505 |
| H | 1.2881 | 13.9145 | 11.9967 |
| H | 1.2251 | 13.9345 | 13.8083 |
| H | -1.5220 | 6.1308 | 12.8406 |
| H | -0.0237 | 6.5599 | 11.9265 |
| H | -0.0008 | 6.5090 | 13.7393 |
| H | -6.9021 | 26.2994 | 1.7134 |
| H | -6.1631 | 26.7479 | 0.1568 |
| C | -4.7456 | 26.3201 | 1.7194 |
| H | -9.3311 | 11.6035 | 15.5641 |
| H | -10.1847 | 10.1406 | 15.0609 |
| H | -10.7710 | 11.7257 | 14.5465 |
| H | -11.7053 | 10.9259 | 13.0222 |
| H | -11.2074 | 9.2551 | 13.3797 |
| C | -12.1048 | 9.5333 | 11.4336 |
| H | -8.7097 | 13.4400 | 14.9185 |
| H | -10.1419 | 13.5844 | 13.8710 |
| C | -8.6202 | 15.0675 | 13.5101 |
| H | -7.6488 | 10.5086 | 15.8646 |
| C | -6.3374 | 8.8311 | 15.5504 |
| H | -8.4775 | 9.0157 | 15.3592 |
| H | -10.4741 | 24.1912 | 0.1068 |
| H | -9.7531 | 24.6093 | 1.6804 |
| C | -10.8059 | 22.7280 | 1.6507 |
| H | -9.0283 | 24.9738 | -2.7194 |
| C | -7.6415 | 24.5086 | -4.3034 |
| H | -7.5094 | 25.9015 | -2.6663 |
| H | -9.4632 | 25.6814 | -0.8135 |
| H | -7.9436 | 26.5834 | -0.8001 |
| H | -8.7229 | 26.1423 | 0.7229 |
| H | 11.3624 | 6.9760 | 14.1267 |
| C | 9.7702 | 5.5527 | 13.8040 |
| H | 9.9359 | 7.2096 | 15.1653 |
| H | 11.9814 | 8.9112 | 14.8415 |
| H | 10.5038 | 8.9650 | 15.8102 |
| H | 11.3287 | 10.4651 | 15.3741 |
| H | 9.6943 | 11.5064 | 15.6074 |
| H | 8.6726 | 10.1234 | 16.0667 |
| C | 7.5885 | 11.9403 | 15.6267 |
| H | 12.9775 | 9.8393 | 13.3493 |
| H | 12.3149 | 11.4300 | 13.7969 |
| C | 13.3025 | 11.3803 | 11.8805 |
| H | 11.4751 | 24.3846 | 2.3226 |
| H | 12.8524 | 23.2562 | 2.3208 |
| C | 11.4804 | 22.9778 | 3.9566 |
| H | 11.9986 | 24.9625 | 0.4192 |
| H | 12.6741 | 24.3914 | -1.1098 |
| H | 13.3634 | 23.8402 | 0.4212 |
| H | 10.1516 | 25.3870 | -0.5859 |
| C | 8.8741 | 25.0598 | -2.2966 |
| H | 10.9935 | 24.8001 | -2.0403 |
| H | 14.1133 | 22.2351 | -0.4538 |
| H | 13.4706 | 22.6359 | -2.0644 |
| C | 14.2431 | 20.6277 | -1.8771 |
| H | 7.5411 | -6.4213 | 0.8720 |
| C | 6.3316 | -5.8527 | 2.5711 |
| H | 8.4628 | -5.9938 | 2.3331 |
| H | 9.4957 | -6.3457 | -0.0818 |
| H | 10.2003 | -5.8867 | 1.4729 |
| H | 11.0339 | -5.4774 | -0.0303 |
| H | 12.0144 | -4.0386 | 0.8309 |
| H | 11.2860 | -4.2631 | 2.4397 |
| C | 12.4137 | -2.4350 | 2.2062 |
| H | 9.1454 | -5.7433 | -1.9347 |
| H | 10.6614 | -4.8149 | -2.0252 |
| C | 9.2801 | -4.4351 | -3.6383 |
| H | 9.7574 | 7.2134 | -15.3443 |
| H | 8.9768 | 8.7312 | -15.8513 |
| C | 7.6097 | 7.1202 | -15.4085 |
| H | 12.0558 | 11.4169 | -14.0063 |
| C | 10.8796 | 13.1605 | -13.5529 |
| H | 10.5713 | 11.5692 | -14.9769 |
| H | 13.2092 | 8.6238 | -13.1230 |
| H | 12.4662 | 7.0320 | -13.4051 |
| C | 13.4486 | 7.2459 | -11.4905 |
| H | 12.3685 | 9.5204 | -14.6366 |
| H | 10.8998 | 9.6052 | -15.6154 |
| H | 11.5315 | 8.0359 | -15.1063 |
| H | -9.9701 | -4.1451 | 2.3453 |
| H | -11.2804 | -2.9511 | 2.4966 |
| C | -9.7591 | -2.8196 | 4.0253 |
| H | -10.4633 | -4.7278 | 0.5365 |
| H | -11.1292 | -4.1822 | -1.0077 |
| H | -11.8188 | -3.5953 | 0.5089 |
| H | -8.6821 | -5.1720 | -0.3850 |
| C | -7.2124 | -4.9683 | -1.9472 |
| H | -9.3405 | -4.6270 | -1.9463 |
| H | -12.5849 | -1.9277 | -0.4846 |
| H | -11.8565 | -2.5755 | -1.9752 |
| C | -12.5623 | -0.5496 | -2.1440 |
| H | -8.7925 | 8.2752 | -15.0210 |
| H | -10.2333 | 8.3365 | -13.9767 |
| C | -8.9129 | 6.6740 | -13.5889 |
| H | -9.1011 | 10.1119 | -15.7325 |
| H | -9.7083 | 11.7173 | -15.3131 |
| H | -10.5524 | 10.2762 | -14.7376 |
| H | -7.2156 | 10.9429 | -16.0535 |
| C | -5.7216 | 12.4639 | -15.7360 |
| H | -7.8701 | 12.5354 | -15.6005 |
| H | -11.3904 | 11.3961 | -13.2035 |
| H | -10.5031 | 12.8186 | -13.8029 |
| C | -11.3964 | 13.0838 | -11.8617 |
| H | 10.3901 | 23.0010 | 4.1614 |
| C | 12.1932 | 23.9504 | 4.8952 |
| H | 11.8155 | 21.9436 | 4.1774 |
| H | 11.8565 | 24.9821 | 4.7433 |
| H | 13.2791 | 23.9330 | 4.7481 |
| H | 12.0027 | 23.6952 | 5.9440 |
| H | 13.6256 | 20.1363 | -2.6595 |
| H | 14.4114 | 19.8673 | -1.0885 |
| C | 15.5879 | 21.0388 | -2.4749 |
| H | 15.4674 | 21.7707 | -3.2817 |
| H | 16.1117 | 20.1732 | -2.8964 |
| H | 16.2467 | 21.4847 | -1.7213 |
| H | 7.9233 | 24.8450 | -1.7637 |
| H | 8.8587 | 24.4507 | -3.2223 |
| C | 8.9245 | 26.5409 | -2.6695 |
| H | 8.9131 | 27.1838 | -1.7819 |
| H | 8.0624 | 26.8211 | -3.2854 |
| H | 9.8276 | 26.7840 | -3.2407 |
| H | -6.2016 | 7.9800 | 14.8518 |
| C | -6.4408 | 8.3024 | 16.9805 |
| H | -5.4269 | 9.4576 | 15.4576 |
| H | -7.3041 | 7.6396 | 17.1076 |
| H | -6.5407 | 9.1167 | 17.7073 |
| H | -5.5464 | 7.7307 | 17.2536 |
| H | 6.6289 | 11.4967 | 15.2826 |
| H | 7.7284 | 12.8734 | 15.0455 |
| C | 7.4866 | 12.2819 | 17.1124 |
| H | 7.3237 | 11.3876 | 17.7248 |
| H | 6.6497 | 12.9634 | 17.3030 |
| H | 8.3964 | 12.7707 | 17.4789 |
| C | -9.2539 | 16.0821 | 14.4608 |
| H | -8.9209 | 15.3028 | 12.4676 |
| H | -7.5166 | 15.1708 | 13.5446 |
| H | -10.3486 | 16.0386 | 14.4292 |
| H | -8.9614 | 17.1048 | 14.1961 |
| H | -8.9471 | 15.9124 | 15.4989 |
| H | -4.5998 | 25.6118 | 2.5612 |
| C | -4.6867 | 27.7534 | 2.2460 |
| H | -3.8998 | 26.1516 | 1.0219 |
| H | -5.4807 | 27.9517 | 2.9747 |
| H | -4.7908 | 28.4861 | 1.4375 |
| H | -3.7298 | 27.9505 | 2.7429 |
| C | -8.2708 | 25.5125 | -5.2684 |
| H | -8.0325 | 23.4927 | -4.5218 |
| H | -6.5481 | 24.4649 | -4.4814 |
| H | -9.3590 | 25.5628 | -5.1483 |
| H | -8.0708 | 25.2354 | -6.3098 |
| H | -7.8752 | 26.5231 | -5.1170 |
| C | -12.7171 | 13.6248 | -12.4077 |
| H | -10.7452 | 13.9336 | -11.5720 |
| H | -11.5889 | 12.5091 | -10.9321 |
| H | -12.5634 | 14.2397 | -13.3019 |
| H | -13.2221 | 14.2524 | -11.6644 |
| H | -13.4071 | 12.8176 | -12.6777 |
| H | -5.5378 | 13.3463 | -15.0903 |
| C | -5.6961 | 12.8972 | -17.2010 |
| H | -4.8848 | 11.7596 | -15.5443 |
| H | -6.4720 | 13.6404 | -17.4172 |
| H | -5.8534 | 12.0500 | -17.8781 |
| H | -4.7310 | 13.3470 | -17.4611 |
| H | 9.8172 | 13.4576 | -13.4270 |
| C | 11.5387 | 14.0577 | -14.5996 |
| H | 11.3671 | 13.3267 | -12.5707 |
| H | 11.0635 | 13.9544 | -15.5815 |
| H | 12.6022 | 13.8228 | -14.7218 |
| H | 11.4699 | 15.1132 | -14.3120 |
| H | 12.8400 | 6.5435 | -10.8809 |
| H | 13.7649 | 8.0601 | -10.8083 |
| C | 14.6855 | 6.5205 | -12.0183 |
| H | 14.4174 | 5.6912 | -12.6830 |
| H | 15.2773 | 6.1020 | -11.1965 |
| H | 15.3401 | 7.1949 | -12.5821 |
| H | 8.1882 | -4.4248 | -3.8277 |
| C | 9.9376 | -5.4703 | -4.5498 |
| H | 9.6515 | -3.4218 | -3.9026 |
| H | 9.5497 | -6.4782 | -4.3641 |
| H | 11.0241 | -5.5036 | -4.4103 |
| H | 9.7523 | -5.2383 | -5.6052 |
| H | 11.8780 | -1.7973 | 2.9428 |
| H | 12.7482 | -1.7609 | 1.3928 |
| C | 13.6359 | -3.0647 | 2.8731 |
| H | 13.3525 | -3.7229 | 3.7025 |
| H | 14.3012 | -2.2953 | 3.2811 |
| H | 14.2214 | -3.6611 | 2.1644 |
| H | 12.7800 | 12.2555 | 11.4410 |
| H | 13.4852 | 10.6757 | 11.0439 |
| C | 14.6415 | 11.8299 | 12.4631 |
| H | 14.5079 | 12.5635 | 13.2664 |
| H | 15.2666 | 12.2992 | 11.6948 |
| H | 15.2079 | 10.9879 | 12.8764 |
| C | -13.4611 | 9.0547 | 11.9499 |
| H | -11.6037 | 8.7063 | 10.8863 |
| H | -12.2612 | 10.3470 | 10.6974 |
| H | -13.3557 | 8.2385 | 12.6738 |
| H | -14.0865 | 8.6849 | 11.1296 |
| H | -14.0136 | 9.8625 | 12.4434 |
| H | 8.6683 | 5.4923 | 13.9073 |
| C | 10.4175 | 4.5266 | 14.7331 |
| H | 10.0033 | 5.2967 | 12.7491 |
| H | 10.1596 | 4.7132 | 15.7823 |
| H | 11.5107 | 4.5405 | 14.6572 |
| H | 10.0854 | 3.5103 | 14.4933 |
| H | 5.4455 | -5.4437 | 2.0408 |
| H | 6.4366 | -5.2656 | 3.5056 |
| C | 6.0835 | -7.3187 | 2.9238 |
| H | 5.9437 | -7.9353 | 2.0288 |
| H | 5.1863 | -7.4298 | 3.5429 |
| H | 6.9209 | -7.7456 | 3.4881 |
| H | 6.7251 | 7.7283 | -15.1228 |
| H | 7.5700 | 6.2015 | -14.7901 |
| C | 7.5073 | 6.7374 | -16.8844 |
| H | 7.5148 | 7.6200 | -17.5345 |
| H | 6.5773 | 6.1933 | -17.0855 |
| H | 8.3373 | 6.0928 | -17.1945 |
| C | -9.7203 | 5.7383 | -14.4881 |
| H | -9.1955 | 6.5043 | -12.5285 |
| H | -7.8374 | 6.4197 | -13.6700 |
| H | -10.7957 | 5.9389 | -14.4215 |
| H | -9.5654 | 4.6908 | -14.2070 |
| H | -9.4315 | 5.8405 | -15.5404 |
| C | -12.1116 | 23.3553 | 2.1377 |
| H | -10.2753 | 22.2674 | 2.5102 |
| H | -11.0367 | 21.9018 | 0.9487 |
| H | -11.9312 | 24.1594 | 2.8602 |
| H | -12.7456 | 22.6100 | 2.6316 |
| H | -12.6906 | 23.7800 | 1.3099 |
| C | -10.6298 | -3.6088 | 5.0017 |
| H | -9.8282 | -1.7359 | 4.2629 |
| H | -8.6983 | -3.1050 | 4.1711 |
| H | -11.6894 | -3.3446 | 4.9078 |
| H | -10.3357 | -3.4098 | 6.0389 |
| H | -10.5436 | -4.6892 | 4.8400 |
| H | -6.9771 | -4.3042 | -2.8051 |
| C | -7.3596 | -6.4053 | -2.4452 |
| H | -6.3473 | -4.9041 | -1.2568 |
| H | -8.1794 | -6.5016 | -3.1670 |
| H | -7.5639 | -7.1012 | -1.6235 |
| H | -6.4462 | -6.7469 | -2.9444 |
| C | -13.9750 | -0.9348 | -2.5809 |
| H | -11.9525 | -0.3231 | -3.0423 |
| H | -12.6016 | 0.3874 | -1.5505 |
| H | -13.9724 | -1.8284 | -3.2155 |
| H | -14.4413 | -0.1276 | -3.1575 |
| H | -14.6245 | -1.1413 | -1.7230 |
| C | 22.8425 | -8.5871 | 9.6342 |
| C | 23.4899 | -7.4167 | 9.2023 |
| H | 23.8463 | -7.3443 | 8.1758 |
| C | 23.6646 | -6.3491 | 10.0887 |
| H | 24.1392 | -5.4351 | 9.7347 |
| C | 23.2212 | -6.4577 | 11.4050 |
| C | 23.2968 | -5.4015 | 12.4915 |
| C | 24.7409 | -4.9929 | 12.8456 |
| H | 25.2540 | -5.8689 | 13.2966 |
| C | 25.6196 | -4.4737 | 11.7010 |
| C | 26.9572 | -3.9534 | 12.2253 |
| H | 27.5256 | -4.7393 | 12.7352 |
| H | 27.5810 | -3.5769 | 11.4066 |
| H | 26.8221 | -3.1303 | 12.9365 |
| H | 25.8046 | -5.2825 | 10.9663 |
| H | 25.0982 | -3.6620 | 11.1492 |
| H | 24.6936 | -4.2138 | 13.6352 |
| C | 22.5291 | -6.0614 | 13.7368 |
| C | 23.3897 | -6.1064 | 14.9918 |
| H | 24.3144 | -6.6783 | 14.8321 |
| H | 23.6850 | -5.1000 | 15.3190 |
| H | 22.8612 | -6.5761 | 15.8324 |
| C | 22.0643 | -7.5245 | 13.2649 |
| C | 22.6156 | -8.6356 | 14.1821 |
| H | 23.7222 | -8.5473 | 14.2111 |
| C | 22.2653 | -10.0835 | 13.8176 |
| C | 22.8134 | -11.0617 | 14.8557 |
| H | 23.9039 | -10.9936 | 14.9405 |
| H | 22.5714 | -12.0962 | 14.5863 |
| H | 22.3931 | -10.8758 | 15.8505 |
| H | 22.6776 | -10.3379 | 12.8197 |
| H | 21.1655 | -10.2084 | 13.7398 |
| H | 22.2553 | -8.4409 | 15.2145 |
| C | 20.5491 | -7.4525 | 13.2695 |
| C | 20.0702 | -6.1815 | 13.6526 |
| C | 18.7068 | -5.9168 | 13.6757 |
| H | 18.3406 | -4.9424 | 14.0044 |
| C | 17.8033 | -6.9294 | 13.2896 |
| C | 18.2763 | -8.2001 | 12.9193 |
| H | 17.5685 | -8.9778 | 12.6361 |
| N | 16.3843 | -6.7501 | 13.2992 |
| C | 15.8467 | -5.6186 | 12.9586 |
| H | 16.3884 | -4.7085 | 12.6362 |
| C | 14.3773 | -5.4709 | 12.9668 |
| C | 13.8089 | -4.1728 | 12.9236 |
| O | 14.7482 | -3.1585 | 12.9296 |
| C | 14.2653 | -1.7965 | 12.8293 |
| H | 15.2035 | -1.2270 | 12.8432 |
| H | 13.7304 | -1.6498 | 11.8861 |
| H | 13.6429 | -1.5499 | 13.6949 |
| C | 12.4235 | -3.9810 | 12.8839 |
| H | 11.9846 | -2.9812 | 12.8562 |
| C | 11.5686 | -5.0879 | 12.8822 |
| C | 12.1363 | -6.3855 | 12.9466 |
| O | 11.1949 | -7.3986 | 12.9638 |
| C | 11.6792 | -8.7633 | 12.9778 |
| H | 12.2545 | -8.9526 | 13.8893 |
| H | 12.2631 | -8.9698 | 12.0761 |
| H | 10.7403 | -9.3318 | 12.9791 |
| C | 10.1011 | -4.9529 | 12.7920 |
| H | 9.5875 | -5.8758 | 12.4570 |
| N | 9.5278 | -3.8268 | 13.0864 |
| C | 8.1049 | -3.6927 | 13.0111 |
| C | 7.5973 | -2.4911 | 12.4900 |
| H | 8.2832 | -1.7292 | 12.1221 |
| C | 6.2142 | -2.2855 | 12.4376 |
| H | 5.8283 | -1.3605 | 12.0137 |
| C | 5.3457 | -3.2654 | 12.9128 |
| C | 5.8590 | -4.4830 | 13.4092 |
| C | 4.7669 | -5.4851 | 13.7355 |
| C | 4.8578 | -6.0439 | 15.1693 |
| C | 6.0612 | -6.9365 | 15.4970 |
| H | 7.0097 | -6.4127 | 15.2495 |
| C | 6.0679 | -7.3321 | 16.9729 |
| H | 6.9411 | -7.9506 | 17.2101 |
| H | 6.1027 | -6.4551 | 17.6293 |
| H | 5.1758 | -7.9093 | 17.2408 |
| H | 6.0397 | -7.8528 | 14.8741 |
| H | 4.8555 | -5.1866 | 15.8747 |
| H | 3.9352 | -6.6271 | 15.3764 |
| C | 4.7627 | -6.5666 | 12.6706 |
| C | 5.7701 | -7.4878 | 12.3915 |
| H | 6.6872 | -7.5023 | 12.9787 |
| C | 5.6144 | -8.3996 | 11.3418 |
| H | 6.3929 | -9.1297 | 11.1255 |
| C | 4.4502 | -8.3686 | 10.5557 |
| N | 4.3241 | -9.3671 | 9.5395 |
| C | 3.7550 | -9.1057 | 8.4022 |
| C | 3.6093 | -10.1700 | 7.3877 |
| C | 4.3039 | -11.3812 | 7.4688 |
| H | 4.9603 | -11.5504 | 8.3256 |
| C | 4.1431 | -12.3465 | 6.4673 |
| O | 4.7779 | -13.5740 | 6.4443 |
| C | 5.6759 | -13.8851 | 7.5374 |
| H | 6.5110 | -13.1788 | 7.5563 |
| H | 6.0156 | -14.8923 | 7.2646 |
| H | 5.1302 | -13.8980 | 8.4862 |
| C | 3.2857 | -12.1318 | 5.3595 |
| C | 3.2001 | -13.1648 | 4.3147 |
| H | 4.0408 | -13.8751 | 4.3023 |
| N | 2.1754 | -13.1731 | 3.4812 |
| C | 2.0924 | -14.1447 | 2.4429 |
| C | 2.4794 | -15.4922 | 2.6102 |
| H | 2.8666 | -15.8415 | 3.5680 |
| C | 2.3472 | -16.3734 | 1.5431 |
| C | 2.7812 | -17.8273 | 1.5028 |
| C | 4.2946 | -17.9080 | 1.5479 |
| C | 5.1304 | -17.5205 | 2.5930 |
| C | 6.5174 | -17.6409 | 2.4608 |
| H | 7.1764 | -17.3515 | 3.2780 |
| C | 7.0654 | -18.1310 | 1.2630 |
| N | 8.4876 | -18.2781 | 1.2240 |
| C | 9.1663 | -18.0631 | 0.1399 |
| C | 10.6341 | -18.2350 | 0.1451 |
| C | 11.3740 | -18.2944 | 1.3317 |
| C | 12.7661 | -18.4209 | 1.2800 |
| O | 13.5969 | -18.4904 | 2.3835 |
| C | 12.9820 | -18.4010 | 3.6917 |
| H | 13.8540 | -18.4789 | 4.3536 |
| H | 12.4828 | -17.4355 | 3.8139 |
| H | 12.2942 | -19.2384 | 3.8451 |
| C | 13.4594 | -18.4932 | 0.0453 |
| C | 14.9339 | -18.5797 | 0.0496 |
| N | 15.5640 | -18.9654 | -1.0183 |
| C | 16.9914 | -19.0523 | -1.0486 |
| C | 17.7601 | -19.4817 | 0.0542 |
| C | 19.1418 | -19.5558 | -0.0708 |
| C | 20.1349 | -19.9216 | 1.0162 |
| C | 21.5651 | -19.8118 | 0.2947 |
| C | 22.3329 | -21.1258 | 0.3384 |
| H | 23.3187 | -21.0377 | -0.1394 |
| H | 22.5035 | -21.4641 | 1.3698 |
| H | 21.7944 | -21.9308 | -0.1795 |
| C | 22.3518 | -18.6190 | 1.0252 |
| C | 23.7208 | -19.0709 | 1.5725 |
| H | 24.2822 | -19.5661 | 0.7518 |
| C | 24.6257 | -17.9863 | 2.1699 |
| C | 25.8661 | -18.5978 | 2.8198 |
| H | 26.4738 | -19.1516 | 2.0953 |
| H | 26.5058 | -17.8208 | 3.2535 |
| H | 25.6014 | -19.2897 | 3.6276 |
| H | 24.9401 | -17.2762 | 1.3795 |
| H | 24.0697 | -17.3889 | 2.9245 |
| H | 23.5438 | -19.8480 | 2.3454 |
| C | 22.4548 | -17.5375 | -0.0340 |
| C | 22.9775 | -16.2548 | 0.1161 |
| H | 23.3869 | -15.9296 | 1.0713 |
| C | 22.9694 | -15.3653 | -0.9633 |
| H | 23.3892 | -14.3661 | -0.8571 |
| C | 22.4078 | -15.7584 | -2.1902 |
| N | 22.4874 | -14.8132 | -3.2600 |
| C | 21.5414 | -14.6978 | -4.1425 |
| C | 21.6671 | -13.7061 | -5.2306 |
| C | 22.5981 | -12.6624 | -5.1860 |
| H | 23.2765 | -12.6056 | -4.3316 |
| C | 22.6466 | -11.7244 | -6.2227 |
| O | 23.5264 | -10.6592 | -6.2851 |
| C | 24.4316 | -10.4722 | -5.1700 |
| H | 25.1015 | -11.3327 | -5.0813 |
| H | 24.9819 | -9.5741 | -5.4788 |
| H | 23.8730 | -10.2943 | -4.2461 |
| C | 21.7670 | -11.7984 | -7.3322 |
| C | 21.8220 | -10.7490 | -8.3714 |
| H | 22.3717 | -9.8390 | -8.0613 |
| N | 21.2420 | -10.9397 | -9.5150 |
| C | 21.2572 | -9.9393 | -10.5379 |
| C | 22.3860 | -9.1411 | -10.8191 |
| H | 23.3042 | -9.2551 | -10.2396 |
| C | 22.3216 | -8.2187 | -11.8564 |
| C | 23.3785 | -7.1957 | -12.2331 |
| C | 24.7425 | -7.8335 | -12.5659 |
| H | 25.3972 | -7.0514 | -13.0062 |
| C | 25.5075 | -8.4899 | -11.4100 |
| C | 26.7552 | -9.2139 | -11.9142 |
| H | 27.4504 | -8.5261 | -12.4087 |
| H | 27.2963 | -9.6876 | -11.0872 |
| H | 26.5045 | -10.0024 | -12.6330 |
| H | 25.8053 | -7.7239 | -10.6665 |
| H | 24.8543 | -9.2108 | -10.8726 |
| H | 24.5821 | -8.5943 | -13.3585 |
| C | 23.4591 | -6.1405 | -11.1459 |
| C | 23.8638 | -6.3183 | -9.8247 |
| H | 24.1804 | -7.2966 | -9.4665 |
| C | 23.8552 | -5.2356 | -8.9388 |
| H | 24.1835 | -5.3645 | -7.9084 |
| C | 23.4123 | -3.9760 | -9.3771 |
| N | 23.4914 | -2.9112 | -8.4298 |
| C | 22.6022 | -1.9571 | -8.3938 |
| C | 22.7281 | -0.8771 | -7.3955 |
| C | 23.5595 | -0.9862 | -6.2754 |
| H | 24.1645 | -1.8887 | -6.1606 |
| C | 23.6033 | 0.0489 | -5.3353 |
| O | 24.3837 | 0.0486 | -4.1937 |
| C | 25.1876 | -1.1270 | -3.9312 |
| H | 25.9232 | -1.2701 | -4.7289 |
| H | 25.6776 | -0.8545 | -2.9877 |
| H | 24.5479 | -2.0052 | -3.8031 |
| C | 22.8228 | 1.2228 | -5.4858 |
| C | 22.8657 | 2.2604 | -4.4345 |
| H | 23.2984 | 1.9123 | -3.4767 |
| N | 22.4035 | 3.4480 | -4.6781 |
| C | 22.4051 | 4.4714 | -3.6788 |
| C | 23.4758 | 4.6733 | -2.7826 |
| H | 24.3566 | 4.0286 | -2.8160 |
| C | 23.4048 | 5.7165 | -1.8671 |
| C | 24.3908 | 6.0207 | -0.7526 |
| C | 25.8283 | 6.2434 | -1.2640 |
| H | 26.4409 | 6.6455 | -0.4290 |
| C | 26.5615 | 5.0243 | -1.8369 |
| C | 27.9004 | 5.4247 | -2.4546 |
| H | 28.5675 | 5.8809 | -1.7145 |
| H | 28.4184 | 4.5517 | -2.8679 |
| H | 27.7730 | 6.1444 | -3.2715 |
| H | 26.7374 | 4.2763 | -1.0383 |
| H | 25.9359 | 4.5213 | -2.6056 |
| H | 25.7976 | 7.0344 | -2.0424 |
| C | 24.2898 | 4.9385 | 0.3060 |
| C | 24.5979 | 3.5872 | 0.1640 |
| H | 24.9701 | 3.1989 | -0.7827 |
| C | 24.4168 | 2.7118 | 1.2396 |
| H | 24.6695 | 1.6570 | 1.1406 |
| C | 23.8957 | 3.1925 | 2.4532 |
| N | 23.7937 | 2.2478 | 3.5210 |
| C | 22.8189 | 2.2852 | 4.3787 |
| C | 22.7603 | 1.2860 | 5.4654 |
| C | 23.5163 | 0.1085 | 5.4373 |
| H | 24.1981 | -0.0543 | 4.5989 |
| C | 23.3904 | -0.8246 | 6.4714 |
| O | 24.0806 | -2.0205 | 6.5474 |
| C | 24.9771 | -2.3472 | 5.4582 |
| H | 25.7787 | -1.6050 | 5.3909 |
| H | 25.3678 | -3.3219 | 5.7774 |
| H | 24.4210 | -2.4338 | 4.5201 |
| C | 22.5132 | -0.6097 | 7.5646 |
| C | 22.3827 | -1.6519 | 8.6049 |
| H | 22.7711 | -2.6430 | 8.2982 |
| N | 21.8387 | -1.3631 | 9.7444 |
| C | 21.6766 | -2.3499 | 10.7686 |
| C | 22.6549 | -3.3227 | 11.0632 |
| H | 23.5865 | -3.3623 | 10.4952 |
| C | 20.4925 | -2.2819 | 11.5232 |
| C | 20.2799 | -3.1850 | 12.5695 |
| C | 21.2481 | -4.1401 | 12.8720 |
| H | 19.3494 | -3.1382 | 13.1332 |
| H | 19.7429 | -1.5286 | 11.2846 |
| C | 21.7764 | 0.5791 | 7.6054 |
| H | 21.1167 | 0.7558 | 8.4581 |
| C | 21.8984 | 1.5106 | 6.5687 |
| O | 21.2219 | 2.7147 | 6.5041 |
| C | 20.2814 | 3.0143 | 7.5641 |
| H | 20.8004 | 3.0795 | 8.5254 |
| H | 19.9025 | 3.9964 | 7.2534 |
| H | 19.4785 | 2.2714 | 7.5827 |
| H | 21.9822 | 3.0091 | 4.3856 |
| C | 23.5579 | 4.5557 | 2.5937 |
| H | 23.1502 | 4.9227 | 3.5336 |
| C | 23.7595 | 5.4186 | 1.5227 |
| C | 23.3978 | 6.8919 | 1.4562 |
| C | 24.1228 | 7.7420 | 2.5196 |
| H | 25.2156 | 7.6791 | 2.3315 |
| C | 23.8756 | 7.3848 | 3.9906 |
| C | 24.5794 | 8.3703 | 4.9224 |
| H | 25.6635 | 8.3761 | 4.7612 |
| H | 24.4087 | 8.1094 | 5.9732 |
| H | 24.2191 | 9.3950 | 4.7780 |
| H | 24.2382 | 6.3580 | 4.2015 |
| H | 22.7878 | 7.3800 | 4.2115 |
| H | 23.8364 | 8.8039 | 2.3659 |
| C | 21.8894 | 7.0446 | 1.5043 |
| C | 21.3485 | 7.4642 | 0.2701 |
| C | 22.4120 | 7.6623 | -0.7936 |
| C | 22.4017 | 9.0901 | -1.3801 |
| H | 23.3124 | 9.2206 | -2.0031 |
| H | 22.4894 | 9.8084 | -0.5382 |
| C | 21.1911 | 9.4863 | -2.2343 |
| H | 21.1632 | 8.8777 | -3.1601 |
| C | 21.2424 | 10.9674 | -2.6075 |
| H | 22.1410 | 11.2078 | -3.1869 |
| H | 21.2415 | 11.6102 | -1.7198 |
| H | 20.3759 | 11.2503 | -3.2158 |
| H | 20.2460 | 9.2743 | -1.6909 |
| C | 19.9755 | 7.6087 | 0.1130 |
| H | 19.5653 | 7.9627 | -0.8344 |
| C | 19.1236 | 7.3121 | 1.1985 |
| C | 19.6615 | 6.9130 | 2.4343 |
| H | 18.9959 | 6.7026 | 3.2703 |
| N | 17.7012 | 7.4374 | 1.1369 |
| C | 17.0444 | 7.1586 | 0.0474 |
| H | 17.4819 | 6.8182 | -0.9091 |
| C | 15.5727 | 7.2752 | 0.0276 |
| C | 14.9011 | 7.3658 | -1.2181 |
| O | 15.7583 | 7.3882 | -2.3024 |
| C | 15.1715 | 7.4398 | -3.6248 |
| H | 16.0653 | 7.4577 | -4.2629 |
| H | 14.5757 | 6.5419 | -3.8109 |
| H | 14.5848 | 8.3554 | -3.7473 |
| C | 13.5061 | 7.4313 | -1.2953 |
| H | 12.9899 | 7.5100 | -2.2543 |
| C | 12.7388 | 7.4083 | -0.1241 |
| C | 13.4120 | 7.3295 | 1.1204 |
| O | 12.5647 | 7.2937 | 2.2159 |
| C | 13.1590 | 7.4762 | 3.5240 |
| H | 13.7172 | 8.4167 | 3.5661 |
| H | 13.7918 | 6.6197 | 3.7742 |
| H | 12.2719 | 7.5182 | 4.1693 |
| C | 11.2640 | 7.4647 | -0.1440 |
| H | 10.7888 | 7.2839 | 0.8363 |
| N | 10.6399 | 7.7137 | -1.2635 |
| C | 9.2167 | 7.8152 | -1.3224 |
| C | 8.6234 | 7.5105 | -2.5587 |
| H | 9.2415 | 7.1744 | -3.3899 |
| C | 7.2378 | 7.6322 | -2.7157 |
| H | 6.7842 | 7.3702 | -3.6716 |
| C | 6.4531 | 8.0832 | -1.6573 |
| C | 7.0513 | 8.3993 | -0.4173 |
| C | 6.0383 | 8.8413 | 0.6230 |
| C | 6.3019 | 10.2724 | 1.1378 |
| C | 7.6575 | 10.5405 | 1.8030 |
| H | 8.4791 | 10.3887 | 1.0747 |
| C | 7.7276 | 11.9661 | 2.3489 |
| H | 8.6953 | 12.1602 | 2.8245 |
| H | 7.5999 | 12.7106 | 1.5545 |
| H | 6.9521 | 12.1524 | 3.1008 |
| H | 7.8323 | 9.8183 | 2.6278 |
| H | 6.1942 | 10.9770 | 0.2861 |
| H | 5.5012 | 10.5293 | 1.8635 |
| C | 5.9486 | 7.8215 | 1.7424 |
| C | 6.9087 | 7.5177 | 2.7048 |
| H | 7.8439 | 8.0795 | 2.7439 |
| C | 6.6751 | 6.4944 | 3.6313 |
| H | 7.4260 | 6.2450 | 4.3801 |
| C | 5.4670 | 5.7770 | 3.5940 |
| N | 5.2827 | 4.7422 | 4.5675 |
| C | 4.6421 | 3.6566 | 4.2541 |
| C | 4.4520 | 2.5793 | 5.2444 |
| C | 5.2963 | 2.4058 | 6.3403 |
| H | 6.1150 | 3.1083 | 6.5346 |
| C | 5.1050 | 1.3060 | 7.1925 |
| O | 6.0724 | 1.1157 | 8.1684 |
| C | 4.0666 | 0.3784 | 6.9916 |
| C | 3.9917 | -0.8032 | 7.8834 |
| H | 4.9785 | -1.2889 | 8.0330 |
| N | 2.8748 | -1.1661 | 8.4179 |
| C | 2.8316 | -2.2642 | 9.3412 |
| C | 3.3786 | -2.1185 | 10.6342 |
| H | 3.8627 | -1.1857 | 10.9258 |
| C | 3.2707 | -3.1700 | 11.5350 |
| C | 2.6183 | -4.3676 | 11.1666 |
| C | 2.6555 | -5.4102 | 12.2676 |
| C | 1.2569 | -5.9185 | 12.6701 |
| H | 1.3662 | -6.5786 | 13.5555 |
| C | 0.4457 | -6.6717 | 11.6091 |
| H | 1.0294 | -7.5211 | 11.1984 |
| H | 0.2251 | -6.0040 | 10.7510 |
| C | -0.8641 | -7.1970 | 12.1921 |
| H | -0.6951 | -7.9772 | 12.9406 |
| H | -1.5015 | -7.6278 | 11.4077 |
| H | -1.4479 | -6.3896 | 12.6644 |
| H | 0.6559 | -5.0335 | 12.9943 |
| C | 2.0677 | -4.4964 | 9.8947 |
| H | 1.5544 | -5.4146 | 9.6051 |
| C | 2.1678 | -3.4443 | 8.9757 |
| H | 1.7367 | -3.5417 | 7.9827 |
| C | 3.1807 | 0.5741 | 5.9243 |
| H | 2.3517 | -0.1157 | 5.7913 |
| C | 3.3825 | 1.6562 | 5.0616 |
| O | 2.6033 | 1.9517 | 3.9653 |
| C | 1.4823 | 1.0833 | 3.6689 |
| H | 1.8410 | 0.0849 | 3.4000 |
| H | 1.0363 | 1.5895 | 2.8009 |
| H | 0.7790 | 1.0694 | 4.5070 |
| H | 4.2008 | 3.4274 | 3.2635 |
| C | 4.4731 | 6.1110 | 2.6494 |
| H | 3.5061 | 5.6001 | 2.6512 |
| C | 4.7288 | 7.1124 | 1.7230 |
| C | 3.8285 | 7.5306 | 0.5779 |
| C | 3.7818 | 6.4330 | -0.4659 |
| C | 4.3208 | 6.8446 | -1.7029 |
| C | 4.3031 | 5.9867 | -2.7961 |
| H | 4.6789 | 6.3211 | -3.7648 |
| C | 3.7693 | 4.6886 | -2.6442 |
| N | 3.6064 | 3.7690 | -3.7221 |
| C | 4.4129 | 3.7578 | -4.7469 |
| H | 5.2884 | 4.4148 | -4.8972 |
| C | 4.1905 | 2.7901 | -5.8380 |
| C | 4.8201 | 3.0195 | -7.0895 |
| O | 5.4975 | 4.2185 | -7.1516 |
| C | 6.3949 | 4.4225 | -8.2722 |
| H | 7.1476 | 3.6265 | -8.3111 |
| H | 6.8625 | 5.3817 | -8.0159 |
| H | 5.8282 | 4.4959 | -9.2028 |
| C | 4.7196 | 2.0995 | -8.1375 |
| H | 5.2145 | 2.2720 | -9.0950 |
| C | 3.9713 | 0.9282 | -7.9640 |
| C | 3.8701 | -0.1042 | -9.0156 |
| N | 4.8245 | -0.2171 | -9.8868 |
| C | 4.8184 | -1.2176 | -10.9095 |
| C | 5.9983 | -1.3004 | -11.6718 |
| H | 6.8212 | -0.6163 | -11.4647 |
| C | 6.1127 | -2.2574 | -12.6848 |
| H | 7.0282 | -2.3114 | -13.2772 |
| C | 5.0589 | -3.1316 | -12.9384 |
| C | 5.0105 | -4.2450 | -13.9660 |
| C | 5.9777 | -5.3547 | -13.5974 |
| C | 7.3651 | -5.2785 | -13.5607 |
| C | 8.1087 | -6.4163 | -13.1792 |
| N | 9.5369 | -6.4632 | -13.1916 |
| C | 10.2651 | -5.4252 | -12.9080 |
| C | 11.7382 | -5.5311 | -12.9481 |
| C | 12.3977 | -6.7657 | -12.9529 |
| C | 13.7955 | -6.8100 | -12.9481 |
| O | 14.5506 | -7.9689 | -12.9491 |
| C | 13.8415 | -9.2312 | -12.9232 |
| H | 14.6700 | -9.9510 | -12.9248 |
| H | 13.2479 | -9.3164 | -12.0083 |
| H | 13.2253 | -9.3392 | -13.8212 |
| C | 14.5771 | -5.6266 | -12.9427 |
| C | 16.0498 | -5.7348 | -12.8870 |
| N | 16.7889 | -4.7162 | -13.2051 |
| C | 18.2170 | -4.7840 | -13.1489 |
| C | 18.9501 | -5.9344 | -13.5103 |
| C | 20.3379 | -5.9002 | -13.4508 |
| C | 21.2889 | -7.0502 | -13.7254 |
| C | 21.1274 | -7.6261 | -15.1482 |
| H | 21.9722 | -8.3205 | -15.3445 |
| H | 21.2311 | -6.7899 | -15.8710 |
| C | 19.8255 | -8.3762 | -15.4553 |
| H | 19.7655 | -9.3001 | -14.8464 |
| C | 19.7385 | -8.7428 | -16.9364 |
| H | 20.5651 | -9.3943 | -17.2416 |
| H | 19.7658 | -7.8538 | -17.5769 |
| H | 18.8053 | -9.2737 | -17.1562 |
| H | 18.9459 | -7.7581 | -15.1752 |
| C | 21.0115 | -4.7249 | -13.0546 |
| C | 22.5177 | -4.9011 | -13.0203 |
| C | 23.2610 | -3.8958 | -13.9243 |
| H | 24.3383 | -4.1651 | -13.9317 |
| C | 23.1467 | -2.4096 | -13.5635 |
| C | 23.8806 | -1.5375 | -14.5813 |
| H | 24.9465 | -1.7865 | -14.6344 |
| H | 23.8069 | -0.4768 | -14.3154 |
| H | 23.4647 | -1.6520 | -15.5887 |
| H | 23.5667 | -2.2262 | -12.5532 |
| H | 22.0813 | -2.1033 | -13.5167 |
| H | 22.8940 | -4.0292 | -14.9641 |
| C | 20.2836 | -3.5877 | -12.7125 |
| H | 20.7982 | -2.6739 | -12.4105 |
| H | 18.4363 | -6.8355 | -13.8506 |
| C | 18.8860 | -3.6102 | -12.7604 |
| H | 18.3108 | -2.7246 | -12.4937 |
| H | 16.4136 | -6.7252 | -12.5514 |
| C | 13.9183 | -4.3927 | -12.9634 |
| H | 14.5188 | -3.4803 | -12.9819 |
| H | 11.7970 | -7.6780 | -12.9631 |
| C | 12.5194 | -4.3481 | -12.9639 |
| O | 11.7655 | -3.1898 | -12.9848 |
| C | 12.4734 | -1.9267 | -12.9407 |
| H | 13.1066 | -1.8154 | -13.8262 |
| H | 13.0481 | -1.8441 | -12.0134 |
| H | 11.6445 | -1.2071 | -12.9554 |
| H | 9.8928 | -4.4240 | -12.6198 |
| C | 7.4510 | -7.6090 | -12.8305 |
| H | 8.0346 | -8.4825 | -12.5428 |
| C | 6.0540 | -7.6652 | -12.8494 |
| H | 5.5485 | -8.5899 | -12.5675 |
| H | 7.8774 | -4.3575 | -13.8324 |
| C | 5.3165 | -6.5477 | -13.2332 |
| C | 3.8061 | -6.4097 | -13.2602 |
| C | 3.2829 | -6.4182 | -11.8332 |
| C | 3.3141 | -7.4782 | -10.9340 |
| H | 3.6741 | -8.4581 | -11.2445 |
| C | 2.8646 | -7.2731 | -9.6125 |
| N | 2.8032 | -8.3143 | -8.6312 |
| C | 3.7142 | -9.2272 | -8.5413 |
| H | 4.6151 | -9.3240 | -9.1791 |
| C | 3.6062 | -10.2695 | -7.4970 |
| C | 4.3228 | -11.4836 | -7.6449 |
| O | 5.0433 | -11.5573 | -8.8225 |
| C | 5.8568 | -12.7364 | -9.0373 |
| H | 6.6187 | -12.8213 | -8.2569 |
| H | 6.3118 | -12.5175 | -10.0117 |
| H | 5.2250 | -13.6286 | -9.0901 |
| C | 4.2749 | -12.4867 | -6.6709 |
| H | 4.8179 | -13.4271 | -6.7924 |
| C | 3.5161 | -12.2986 | -5.5101 |
| C | 3.4606 | -13.3077 | -4.4308 |
| N | 3.9011 | -14.5080 | -4.6490 |
| C | 3.8679 | -15.5232 | -3.6423 |
| C | 4.9280 | -16.4466 | -3.6526 |
| H | 5.7341 | -16.3352 | -4.3762 |
| C | 4.9439 | -17.4971 | -2.7303 |
| H | 5.7718 | -18.2084 | -2.7364 |
| C | 3.9033 | -17.6375 | -1.8151 |
| C | 3.7865 | -18.6698 | -0.7099 |
| C | 3.8104 | -20.1212 | -1.2308 |
| C | 5.0969 | -20.6002 | -1.9147 |
| H | 5.9483 | -20.5554 | -1.2048 |
| C | 4.9468 | -22.0295 | -2.4335 |
| H | 5.8710 | -22.3724 | -2.9128 |
| H | 4.7199 | -22.7327 | -1.6240 |
| H | 4.1441 | -22.1108 | -3.1750 |
| H | 5.3606 | -19.9279 | -2.7574 |
| H | 3.6024 | -20.8017 | -0.3782 |
| H | 2.9702 | -20.2442 | -1.9466 |
| C | 2.8286 | -16.7224 | -1.8192 |
| C | 1.8050 | -17.0255 | -0.7393 |
| C | 0.3963 | -17.2909 | -1.3108 |
| H | 0.4511 | -18.1796 | -1.9745 |
| C | -0.2682 | -16.1549 | -2.0980 |
| H | 0.3558 | -15.8714 | -2.9697 |
| H | -0.3535 | -15.2458 | -1.4677 |
| C | -1.6565 | -16.5624 | -2.5856 |
| H | -1.6164 | -17.4149 | -3.2710 |
| H | -2.1435 | -15.7347 | -3.1192 |
| H | -2.3206 | -16.8327 | -1.7544 |
| H | -0.2742 | -17.5643 | -0.4682 |
| C | 2.8095 | -15.6630 | -2.7189 |
| H | 1.9813 | -14.9559 | -2.7241 |
| H | 3.0336 | -12.9294 | -3.4820 |
| C | 2.7931 | -11.0874 | -5.3671 |
| O | 2.0537 | -11.0178 | -4.1988 |
| C | 1.4567 | -9.7427 | -3.8628 |
| H | 2.2274 | -8.9704 | -3.7736 |
| H | 1.0043 | -9.9597 | -2.8866 |
| H | 0.6964 | -9.4751 | -4.6019 |
| C | 2.8315 | -10.0888 | -6.3455 |
| H | 2.2626 | -9.1631 | -6.2389 |
| C | 2.3951 | -6.0110 | -9.2087 |
| C | 2.3760 | -4.9513 | -10.1198 |
| H | 2.0408 | -3.9698 | -9.7868 |
| H | 2.0568 | -5.8621 | -8.1843 |
| C | 2.7990 | -5.1548 | -11.4319 |
| C | 2.8703 | -4.1258 | -12.5428 |
| C | 1.4904 | -3.5466 | -12.9179 |
| H | 1.6448 | -2.7621 | -13.6873 |
| C | 0.6540 | -2.9612 | -11.7731 |
| H | 1.2857 | -2.3337 | -11.1077 |
| H | 0.2539 | -3.7798 | -11.1399 |
| C | -0.5061 | -2.1220 | -12.3009 |
| H | -0.1583 | -1.2584 | -12.8761 |
| H | -1.1213 | -1.7448 | -11.4731 |
| H | -1.1777 | -2.7124 | -12.9487 |
| H | 0.8884 | -4.3460 | -13.3992 |
| C | 3.1135 | -7.4463 | -14.1675 |
| H | 3.5033 | -7.3185 | -15.1998 |
| C | 3.2591 | -8.9237 | -13.7831 |
| H | 4.3306 | -9.2106 | -13.7572 |
| H | 2.8674 | -9.0967 | -12.7593 |
| C | 2.5157 | -9.8250 | -14.7678 |
| H | 2.9000 | -9.7159 | -15.7883 |
| H | 2.6200 | -10.8803 | -14.4905 |
| H | 1.4434 | -9.5999 | -14.7940 |
| H | 2.0300 | -7.2054 | -14.2071 |
| C | 5.2192 | -3.7272 | -15.4059 |
| C | 6.5877 | -3.1229 | -15.7441 |
| H | 7.3827 | -3.8888 | -15.6376 |
| C | 6.6085 | -2.5707 | -17.1688 |
| H | 7.5901 | -2.1476 | -17.4120 |
| H | 6.3990 | -3.3519 | -17.9084 |
| H | 5.8671 | -1.7760 | -17.3080 |
| H | 6.8416 | -2.3117 | -15.0306 |
| H | 5.0336 | -4.5663 | -16.1093 |
| H | 4.4411 | -2.9623 | -15.6127 |
| C | 3.5597 | -4.9053 | -13.7632 |
| C | 2.7228 | -4.8448 | -15.0340 |
| H | 3.1995 | -5.3882 | -15.8615 |
| H | 2.5712 | -3.8111 | -15.3726 |
| H | 1.7270 | -5.2857 | -14.8884 |
| C | 3.8754 | -3.0498 | -12.1733 |
| C | 3.7438 | -2.0933 | -11.1749 |
| H | 2.8113 | -2.0233 | -10.6075 |
| H | 2.9611 | -0.7316 | -8.9425 |
| C | 3.3009 | 0.7262 | -6.7334 |
| O | 2.5821 | -0.4543 | -6.6835 |
| C | 1.7001 | -0.6505 | -5.5512 |
| H | 2.2861 | -0.7562 | -4.6330 |
| H | 1.2054 | -1.5925 | -5.8191 |
| H | 0.9757 | 0.1675 | -5.4829 |
| C | 3.4121 | 1.6408 | -5.6789 |
| H | 2.9078 | 1.4761 | -4.7236 |
| C | 3.2959 | 4.2563 | -1.3929 |
| H | 2.9164 | 3.2412 | -1.2804 |
| C | 3.3122 | 5.1313 | -0.3025 |
| H | 2.9626 | 4.7818 | 0.6679 |
| C | 2.4231 | 7.9549 | 1.0557 |
| H | 2.5466 | 8.6816 | 1.8851 |
| C | 1.4889 | 6.8289 | 1.5162 |
| H | 2.0241 | 6.1359 | 2.2020 |
| H | 1.1710 | 6.2215 | 0.6435 |
| C | 0.2530 | 7.3792 | 2.2227 |
| H | 0.5158 | 7.9402 | 3.1260 |
| H | -0.4177 | 6.5629 | 2.5269 |
| H | -0.3328 | 8.0431 | 1.5708 |
| H | 1.9144 | 8.4992 | 0.2321 |
| C | 4.6257 | 8.7242 | -0.1352 |
| C | 3.8467 | 10.0317 | -0.1058 |
| H | 4.3890 | 10.8429 | -0.6109 |
| H | 3.6510 | 10.3649 | 0.9223 |
| H | 2.8717 | 9.9358 | -0.6039 |
| C | 4.9426 | 8.2291 | -1.6292 |
| C | 4.3965 | 9.1992 | -2.6952 |
| H | 4.7975 | 10.2133 | -2.4848 |
| C | 4.6952 | 8.8688 | -4.1629 |
| H | 5.7889 | 8.8777 | -4.3439 |
| H | 4.3491 | 7.8412 | -4.4067 |
| C | 4.0202 | 9.8661 | -5.1029 |
| H | 4.3759 | 10.8885 | -4.9328 |
| H | 4.2266 | 9.6191 | -6.1511 |
| H | 2.9314 | 9.8696 | -4.9790 |
| H | 3.2948 | 9.2633 | -2.5704 |
| C | 8.4220 | 8.2551 | -0.2405 |
| H | 8.8818 | 8.4836 | 0.7187 |
| C | 14.8079 | 7.2681 | 1.1984 |
| H | 15.3244 | 7.2140 | 2.1596 |
| C | 21.0461 | 6.7788 | 2.5803 |
| H | 21.4609 | 6.4690 | 3.5419 |
| C | 23.7893 | 7.3262 | -0.0394 |
| C | 24.7587 | 8.5000 | -0.0642 |
| H | 25.7065 | 8.2567 | 0.4364 |
| H | 25.0052 | 8.8030 | -1.0912 |
| H | 24.3446 | 9.3825 | 0.4416 |
| C | 22.2941 | 6.5871 | -1.8555 |
| C | 21.2336 | 6.3759 | -2.7340 |
| H | 20.3539 | 7.0169 | -2.7081 |
| C | 21.2872 | 5.3234 | -3.6534 |
| H | 20.4649 | 5.1554 | -4.3475 |
| C | 22.0109 | 1.3433 | -6.6193 |
| H | 21.4253 | 2.2565 | -6.7475 |
| C | 21.9649 | 0.3068 | -7.5579 |
| O | 21.2015 | 0.3170 | -8.7107 |
| C | 20.3413 | 1.4602 | -8.9360 |
| H | 20.9391 | 2.3714 | -9.0360 |
| H | 19.8648 | 1.1946 | -9.8884 |
| H | 19.6005 | 1.5435 | -8.1353 |
| H | 21.7192 | -1.8664 | -9.0515 |
| C | 22.9772 | -3.7971 | -10.7081 |
| H | 22.6342 | -2.8202 | -11.0449 |
| C | 23.0038 | -4.8778 | -11.5822 |
| C | 22.7467 | -6.4203 | -13.4881 |
| C | 23.6230 | -6.5160 | -14.7295 |
| H | 24.6257 | -6.1021 | -14.5537 |
| H | 23.7560 | -7.5571 | -15.0549 |
| H | 23.1914 | -5.9666 | -15.5769 |
| C | 21.1557 | -8.1037 | -12.6436 |
| C | 20.0391 | -8.8846 | -12.3532 |
| H | 19.1199 | -8.7760 | -12.9266 |
| C | 20.0870 | -9.8104 | -11.3060 |
| H | 19.2206 | -10.4294 | -11.0775 |
| C | 20.8524 | -12.8562 | -7.3892 |
| H | 20.1895 | -12.9269 | -8.2546 |
| C | 20.8045 | -13.7943 | -6.3525 |
| O | 19.9439 | -14.8756 | -6.3034 |
| C | 19.0001 | -15.0319 | -7.3909 |
| H | 19.5309 | -15.1817 | -8.3362 |
| H | 18.4648 | -15.9419 | -7.0910 |
| H | 18.3235 | -14.1734 | -7.4344 |
| H | 20.6005 | -15.2797 | -4.1700 |
| C | 21.8553 | -17.0486 | -2.3400 |
| H | 21.4194 | -17.3488 | -3.2911 |
| C | 21.8838 | -17.9284 | -1.2643 |
| C | 21.4007 | -18.1568 | 2.1152 |
| C | 21.6154 | -17.1341 | 3.0312 |
| H | 22.5862 | -16.6369 | 3.0843 |
| C | 20.5703 | -16.7614 | 3.9026 |
| N | 20.7093 | -15.7459 | 4.9012 |
| C | 21.3659 | -14.6527 | 4.6697 |
| H | 21.8744 | -14.3841 | 3.7230 |
| C | 21.4701 | -13.6181 | 5.7203 |
| C | 22.4365 | -12.5891 | 5.5882 |
| O | 23.2293 | -12.7186 | 4.4627 |
| C | 24.2254 | -11.6953 | 4.2222 |
| H | 24.9565 | -11.6784 | 5.0364 |
| H | 24.6832 | -12.0470 | 3.2887 |
| H | 23.7463 | -10.7217 | 4.0833 |
| C | 22.5451 | -11.5602 | 6.5296 |
| H | 23.2935 | -10.7706 | 6.4293 |
| C | 21.6851 | -11.5293 | 7.6330 |
| C | 21.7262 | -10.4418 | 8.6331 |
| H | 20.8275 | -10.3873 | 9.2768 |
| C | 20.7325 | -12.5698 | 7.7760 |
| O | 19.9520 | -12.4505 | 8.9113 |
| C | 18.9241 | -13.4478 | 9.1265 |
| H | 19.3741 | -14.4388 | 9.2411 |
| H | 18.4779 | -13.1061 | 10.0693 |
| H | 18.1930 | -13.4229 | 8.3132 |
| C | 20.6259 | -13.6007 | 6.8363 |
| H | 19.8950 | -14.4045 | 6.9504 |
| C | 19.3326 | -17.4259 | 3.8521 |
| C | 19.1333 | -18.4599 | 2.9318 |
| H | 18.1636 | -18.9532 | 2.8857 |
| H | 18.5321 | -17.1276 | 4.5276 |
| C | 21.2829 | -19.3217 | -1.2084 |
| C | 21.8812 | -20.2820 | -2.2570 |
| H | 22.9665 | -20.3934 | -2.0496 |
| C | 21.7193 | -19.8983 | -3.7331 |
| C | 22.2826 | -20.9835 | -4.6496 |
| H | 23.3493 | -21.1554 | -4.4657 |
| H | 22.1752 | -20.7025 | -5.7035 |
| H | 21.7661 | -21.9401 | -4.5118 |
| H | 22.2375 | -18.9394 | -3.9388 |
| H | 20.6492 | -19.7307 | -3.9752 |
| H | 21.4260 | -21.2838 | -2.1065 |
| C | 20.1661 | -18.8396 | 2.0774 |
| C | 19.8822 | -21.3285 | 1.5985 |
| H | 20.7442 | -21.6017 | 2.2438 |
| H | 19.8755 | -22.0527 | 0.7570 |
| C | 18.6026 | -21.5255 | 2.4205 |
| H | 18.6488 | -20.9199 | 3.3474 |
| C | 18.4060 | -22.9955 | 2.7888 |
| H | 19.2385 | -23.3769 | 3.3907 |
| H | 18.3254 | -23.6298 | 1.8985 |
| H | 17.4890 | -23.1358 | 3.3723 |
| H | 17.7174 | -21.1651 | 1.8542 |
| C | 19.7713 | -19.2252 | -1.2899 |
| C | 19.0062 | -18.8222 | -2.3817 |
| H | 19.4868 | -18.5764 | -3.3306 |
| H | 17.2762 | -19.7695 | 0.9893 |
| C | 17.6149 | -18.7361 | -2.2681 |
| H | 17.0108 | -18.4197 | -3.1171 |
| H | 15.3975 | -18.2848 | 1.0106 |
| C | 12.7179 | -18.4563 | -1.1406 |
| H | 13.2501 | -18.5339 | -2.0914 |
| H | 10.8391 | -18.2443 | 2.2828 |
| C | 11.3260 | -18.3273 | -1.0889 |
| O | 10.4946 | -18.2781 | -2.1928 |
| C | 11.1125 | -18.3420 | -3.5011 |
| H | 11.6373 | -19.2940 | -3.6277 |
| H | 11.7776 | -17.4863 | -3.6500 |
| H | 10.2381 | -18.2833 | -4.1620 |
| H | 8.7540 | -17.7438 | -0.8370 |
| C | 6.2233 | -18.5068 | 0.1944 |
| H | 6.6518 | -18.8893 | -0.7306 |
| H | 4.7146 | -17.1175 | 3.5157 |
| C | 4.8462 | -18.3931 | 0.3427 |
| C | 2.4027 | -18.3092 | 0.0191 |
| C | 1.4272 | -19.4785 | 0.0228 |
| H | 1.8378 | -20.3511 | 0.5493 |
| H | 1.1796 | -19.8044 | -0.9970 |
| H | 0.4809 | -19.2177 | 0.5157 |
| C | 2.1001 | -18.6886 | 2.5855 |
| H | 2.3403 | -19.7547 | 2.3890 |
| C | 2.4521 | -18.3853 | 4.0464 |
| H | 3.5331 | -18.5547 | 4.2238 |
| H | 2.2623 | -17.3155 | 4.2758 |
| C | 1.6432 | -19.2617 | 5.0019 |
| H | 1.8421 | -20.3272 | 4.8406 |
| H | 1.8911 | -19.0376 | 6.0458 |
| H | 0.5647 | -19.1056 | 4.8834 |
| H | 0.9931 | -18.5844 | 2.4645 |
| C | 1.8179 | -15.9329 | 0.3113 |
| C | 1.4148 | -14.6088 | 0.1582 |
| H | 0.9881 | -14.2687 | -0.7873 |
| C | 1.5478 | -13.7095 | 1.2229 |
| H | 1.2357 | -12.6730 | 1.1096 |
| C | 2.5782 | -10.9267 | 5.2865 |
| H | 1.9062 | -10.7657 | 4.4417 |
| C | 2.7410 | -9.9620 | 6.2859 |
| O | 2.1041 | -8.7344 | 6.3110 |
| C | 1.1312 | -8.4611 | 5.2749 |
| H | 1.6151 | -8.4401 | 4.2940 |
| H | 0.7772 | -7.4626 | 5.5622 |
| H | 0.3170 | -9.1906 | 5.3156 |
| H | 3.3462 | -8.1254 | 8.0892 |
| C | 3.4356 | -7.4247 | 10.8226 |
| H | 2.5278 | -7.4077 | 10.2197 |
| C | 3.5983 | -6.5323 | 11.8745 |
| C | 3.4049 | -4.6754 | 13.4821 |
| C | 2.5402 | -4.5929 | 14.7327 |
| H | 3.0615 | -4.0770 | 15.5514 |
| H | 2.2639 | -5.5901 | 15.1034 |
| H | 1.6051 | -4.0472 | 14.5507 |
| C | 3.8312 | -3.2232 | 12.9441 |
| C | 3.2868 | -2.0879 | 13.8380 |
| H | 3.5740 | -2.3018 | 14.8899 |
| C | 3.7439 | -0.6608 | 13.5117 |
| H | 4.8333 | -0.5614 | 13.6892 |
| H | 3.5777 | -0.4367 | 12.4369 |
| C | 2.9997 | 0.3637 | 14.3676 |
| H | 3.1808 | 0.2057 | 15.4370 |
| H | 3.3206 | 1.3843 | 14.1302 |
| H | 1.9165 | 0.3177 | 14.2072 |
| H | 2.1776 | -2.1202 | 13.7997 |
| C | 7.2293 | -4.6948 | 13.4807 |
| H | 7.6232 | -5.6197 | 13.9073 |
| C | 13.5205 | -6.5771 | 12.9908 |
| H | 13.9582 | -7.5763 | 13.0465 |
| C | 19.6515 | -8.4547 | 12.9089 |
| H | 20.0152 | -9.4424 | 12.6202 |
| C | 21.1898 | -5.2026 | 13.9519 |
| C | 21.0952 | -4.6119 | 15.3747 |
| H | 22.0349 | -4.0602 | 15.5901 |
| H | 21.0534 | -5.4553 | 16.0958 |
| C | 19.9218 | -3.6672 | 15.6630 |
| H | 20.0190 | -2.7416 | 15.0613 |
| C | 19.8645 | -3.2975 | 17.1446 |
| H | 20.7758 | -2.7823 | 17.4686 |
| H | 19.7428 | -4.1818 | 17.7803 |
| H | 19.0200 | -2.6301 | 17.3505 |
| H | 18.9613 | -4.1372 | 15.3613 |
| C | 22.4266 | -4.2201 | 12.0995 |
| C | 22.5625 | -7.6300 | 11.8347 |
| N | 22.7550 | -9.6529 | 8.6849 |
| C | 22.3700 | -8.6928 | 10.9598 |
| H | 21.8690 | -9.6008 | 11.2917 |
| C | -4.0583 | -7.6584 | 9.6079 |
| C | -3.3890 | -6.5013 | 9.1751 |
| H | -2.9942 | -6.4536 | 8.1618 |
| C | -3.2442 | -5.4131 | 10.0437 |
| H | -2.7346 | -4.5140 | 9.6977 |
| C | -3.7494 | -5.4862 | 11.3395 |
| C | -3.7306 | -4.3988 | 12.3959 |
| C | -2.3044 | -3.9244 | 12.7411 |
| H | -1.7192 | -4.8199 | 13.0657 |
| C | -1.5084 | -3.2182 | 11.6370 |
| C | -0.1935 | -2.6621 | 12.1772 |
| H | 0.4386 | -3.4644 | 12.5930 |
| H | 0.3902 | -2.1826 | 11.3792 |
| H | -0.3540 | -1.9148 | 12.9601 |
| H | -1.2973 | -3.9243 | 10.8080 |
| H | -2.0977 | -2.3884 | 11.1954 |
| H | -2.3643 | -3.2426 | 13.6146 |
| C | -4.4719 | -5.0589 | 13.6566 |
| C | -3.5934 | -5.0778 | 14.8999 |
| H | -2.6760 | -5.6593 | 14.7380 |
| H | -3.2868 | -4.0662 | 15.1999 |
| H | -4.1133 | -5.5231 | 15.7594 |
| C | -4.9127 | -6.5362 | 13.2061 |
| C | -4.3441 | -7.6268 | 14.1390 |
| H | -3.2387 | -7.5220 | 14.1669 |
| C | -4.6728 | -9.0852 | 13.7961 |
| C | -4.0844 | -10.0417 | 14.8324 |
| H | -2.9937 | -9.9524 | 14.8924 |
| H | -4.3108 | -11.0839 | 14.5789 |
| H | -4.4874 | -9.8539 | 15.8338 |
| H | -4.2747 | -9.3443 | 12.7935 |
| H | -5.7713 | -9.2309 | 13.7414 |
| H | -4.7075 | -7.4239 | 15.1686 |
| C | -6.4277 | -6.4889 | 13.2123 |
| C | -6.9275 | -5.2235 | 13.5877 |
| C | -8.2951 | -4.9825 | 13.6067 |
| H | -8.6795 | -4.0110 | 13.9195 |
| C | -9.1812 | -6.0159 | 13.2349 |
| C | -8.6867 | -7.2809 | 12.8740 |
| H | -9.3813 | -8.0740 | 12.6010 |
| N | -10.6034 | -5.8620 | 13.2535 |
| C | -11.1612 | -4.7409 | 12.9146 |
| H | -10.6356 | -3.8239 | 12.5832 |
| C | -12.6319 | -4.6108 | 12.9410 |
| C | -13.2143 | -3.3187 | 12.9145 |
| O | -12.2855 | -2.2944 | 12.9145 |
| C | -12.7847 | -0.9359 | 12.8549 |
| H | -11.8531 | -0.3557 | 12.8664 |
| H | -13.3377 | -0.7740 | 11.9249 |
| H | -13.3940 | -0.7160 | 13.7370 |
| C | -14.6018 | -3.1419 | 12.8941 |
| H | -15.0509 | -2.1460 | 12.8788 |
| C | -15.4467 | -4.2571 | 12.8975 |
| C | -14.8640 | -5.5487 | 12.9461 |
| O | -15.7930 | -6.5729 | 12.9634 |
| C | -15.2916 | -7.9313 | 12.9891 |
| H | -14.7113 | -8.1060 | 13.9005 |
| H | -14.7089 | -8.1376 | 12.0865 |
| H | -16.2228 | -8.5119 | 13.0005 |
| C | -16.9169 | -4.1287 | 12.8306 |
| H | -17.4387 | -5.0578 | 12.5321 |
| N | -17.4759 | -2.9894 | 13.1050 |
| C | -18.8944 | -2.8193 | 13.0587 |
| C | -19.3601 | -1.5548 | 12.6604 |
| H | -18.6488 | -0.7822 | 12.3722 |
| C | -20.7343 | -1.2986 | 12.6266 |
| H | -21.0875 | -0.3215 | 12.2963 |
| C | -21.6371 | -2.2868 | 13.0119 |
| C | -21.1672 | -3.5515 | 13.4274 |
| C | -22.2959 | -4.5093 | 13.7633 |
| C | -22.2324 | -5.0282 | 15.2153 |
| C | -20.9718 | -5.7963 | 15.6310 |
| H | -20.0812 | -5.1393 | 15.5592 |
| C | -21.0943 | -6.3238 | 17.0599 |
| H | -20.1902 | -6.8676 | 17.3569 |
| H | -21.2360 | -5.5100 | 17.7803 |
| H | -21.9404 | -7.0115 | 17.1688 |
| H | -20.7884 | -6.6443 | 14.9386 |
| H | -22.3468 | -4.1622 | 15.9013 |
| H | -23.1120 | -5.6844 | 15.3852 |
| C | -22.3428 | -5.6281 | 12.7410 |
| C | -21.4066 | -6.6419 | 12.5534 |
| H | -20.5395 | -6.7185 | 13.2126 |
| C | -21.5794 | -7.5720 | 11.5233 |
| H | -20.8515 | -8.3673 | 11.3708 |
| C | -22.6963 | -7.4778 | 10.6749 |
| N | -22.8210 | -8.4877 | 9.6715 |
| C | -23.3258 | -8.2244 | 8.5011 |
| C | -23.4347 | -9.2943 | 7.4890 |
| C | -22.7063 | -10.4854 | 7.5797 |
| H | -22.0663 | -10.6412 | 8.4508 |
| C | -22.8127 | -11.4470 | 6.5680 |
| O | -22.1399 | -12.6548 | 6.5521 |
| C | -21.2318 | -12.9319 | 7.6457 |
| H | -20.4245 | -12.1940 | 7.6672 |
| H | -20.8522 | -13.9248 | 7.3725 |
| H | -21.7784 | -12.9673 | 8.5932 |
| C | -23.6506 | -11.2506 | 5.4418 |
| C | -23.6832 | -12.2801 | 4.3900 |
| H | -22.8539 | -13.0012 | 4.4214 |
| N | -24.6613 | -12.2684 | 3.5001 |
| C | -24.7283 | -13.2355 | 2.4601 |
| C | -24.3897 | -14.5950 | 2.6361 |
| H | -24.0267 | -14.9511 | 3.6001 |
| C | -24.5427 | -15.4764 | 1.5713 |
| C | -24.1579 | -16.9450 | 1.5388 |
| C | -22.6471 | -17.0688 | 1.5944 |
| C | -21.8105 | -16.7326 | 2.6562 |
| C | -20.4245 | -16.8655 | 2.5249 |
| H | -19.7651 | -16.6156 | 3.3549 |
| C | -19.8770 | -17.3163 | 1.3115 |
| N | -18.4556 | -17.4720 | 1.2714 |
| C | -17.7757 | -17.2599 | 0.1875 |
| C | -16.3089 | -17.4410 | 0.1912 |
| C | -15.5679 | -17.5008 | 1.3768 |
| C | -14.1759 | -17.6302 | 1.3231 |
| O | -13.3445 | -17.6964 | 2.4260 |
| C | -13.9574 | -17.6038 | 3.7349 |
| H | -13.0840 | -17.6838 | 4.3948 |
| H | -14.4543 | -16.6367 | 3.8554 |
| H | -14.6466 | -18.4394 | 3.8919 |
| C | -13.4835 | -17.7078 | 0.0880 |
| C | -12.0102 | -17.7933 | 0.0957 |
| N | -11.3740 | -18.1991 | -0.9685 |
| C | -9.9477 | -18.2802 | -0.9913 |
| C | -9.1770 | -18.6579 | 0.1295 |
| C | -7.7940 | -18.7226 | 0.0115 |
| C | -6.8009 | -19.0607 | 1.1073 |
| C | -5.3676 | -18.9266 | 0.3927 |
| C | -4.5708 | -20.2219 | 0.4612 |
| H | -3.5837 | -20.1181 | -0.0109 |
| H | -4.4006 | -20.5394 | 1.4991 |
| H | -5.0873 | -21.0470 | -0.0472 |
| C | -4.6127 | -17.7085 | 1.1113 |
| C | -3.2246 | -18.1023 | 1.6578 |
| H | -2.6669 | -18.6432 | 0.8653 |
| C | -2.3349 | -16.9553 | 2.1562 |
| C | -1.2387 | -17.4481 | 3.0963 |
| H | -0.5183 | -18.1100 | 2.5878 |
| H | -0.6603 | -16.6044 | 3.4964 |
| H | -1.6503 | -17.9948 | 3.9518 |
| H | -1.8754 | -16.4335 | 1.2920 |
| H | -2.9462 | -16.1914 | 2.6873 |
| H | -3.3768 | -18.8271 | 2.4843 |
| C | -4.5531 | -16.6265 | 0.0496 |
| C | -4.0836 | -15.3231 | 0.1999 |
| H | -3.6968 | -14.9760 | 1.1573 |
| C | -4.1183 | -14.4398 | -0.8828 |
| H | -3.7332 | -13.4261 | -0.7800 |
| C | -4.6603 | -14.8587 | -2.1106 |
| N | -4.5780 | -13.9223 | -3.1867 |
| C | -5.4928 | -13.8276 | -4.1007 |
| C | -5.3335 | -12.8347 | -5.1841 |
| C | -4.4036 | -11.7913 | -5.1033 |
| H | -3.7569 | -11.7357 | -4.2244 |
| C | -4.3175 | -10.8513 | -6.1345 |
| O | -3.4505 | -9.7730 | -6.1508 |
| C | -2.4460 | -9.7095 | -5.1101 |
| H | -1.8171 | -10.6059 | -5.1330 |
| H | -1.8771 | -8.8196 | -5.4070 |
| H | -2.9212 | -9.5701 | -4.1347 |
| C | -5.1559 | -10.9208 | -7.2758 |
| C | -5.0540 | -9.8730 | -8.3139 |
| H | -4.4889 | -8.9758 | -7.9938 |
| N | -5.6119 | -10.0548 | -9.4681 |
| C | -5.5506 | -9.0697 | -10.5039 |
| C | -4.4291 | -8.2417 | -10.7263 |
| H | -3.5689 | -8.2984 | -10.0623 |
| C | -4.4364 | -7.3655 | -11.8053 |
| C | -3.3468 | -6.3805 | -12.1908 |
| C | -2.0173 | -7.0771 | -12.5534 |
| H | -1.2855 | -6.2945 | -12.8458 |
| C | -1.3711 | -7.9559 | -11.4748 |
| C | -0.0138 | -8.4869 | -11.9315 |
| H | 0.6913 | -7.6744 | -12.1502 |
| H | 0.4475 | -9.1009 | -11.1473 |
| H | -0.0983 | -9.1088 | -12.8285 |
| H | -1.2382 | -7.3814 | -10.5329 |
| H | -2.0329 | -8.8096 | -11.2270 |
| H | -2.1854 | -7.7079 | -13.4512 |
| C | -3.1736 | -5.3254 | -11.1152 |
| C | -2.6291 | -5.4865 | -9.8438 |
| H | -2.2357 | -6.4587 | -9.5330 |
| C | -2.5658 | -4.4008 | -8.9630 |
| H | -2.1380 | -4.5224 | -7.9702 |
| C | -3.0597 | -3.1499 | -9.3643 |
| N | -2.9262 | -2.0470 | -8.4536 |
| C | -3.9426 | -1.3085 | -8.1714 |
| C | -3.8137 | -0.1444 | -7.2510 |
| C | -3.3525 | -0.3288 | -5.9443 |
| H | -2.9833 | -1.3032 | -5.6380 |
| C | -3.3804 | 0.7569 | -5.0600 |
| O | -2.9371 | 0.7220 | -3.7618 |
| C | -2.3391 | -0.5027 | -3.2669 |
| H | -1.4543 | -0.7598 | -3.8539 |
| H | -2.0623 | -0.2173 | -2.2426 |
| H | -3.0721 | -1.3149 | -3.2562 |
| C | -3.8960 | 2.0232 | -5.4498 |
| C | -4.0060 | 3.0870 | -4.4387 |
| H | -3.7737 | 2.7569 | -3.4087 |
| N | -4.3714 | 4.2941 | -4.7766 |
| C | -4.4720 | 5.3212 | -3.7876 |
| C | -3.4489 | 5.5467 | -2.8424 |
| H | -2.5573 | 4.9150 | -2.8311 |
| C | -3.5707 | 6.6012 | -1.9462 |
| C | -2.6063 | 6.9573 | -0.8297 |
| C | -1.1880 | 7.2568 | -1.3585 |
| H | -0.5775 | 7.6718 | -0.5275 |
| C | -0.4054 | 6.0862 | -1.9668 |
| C | 0.8234 | 6.5792 | -2.7265 |
| H | 1.5359 | 7.0878 | -2.0658 |
| H | 1.3616 | 5.7379 | -3.1851 |
| H | 0.5578 | 7.2708 | -3.5325 |
| H | -0.0874 | 5.3854 | -1.1693 |
| H | -1.0475 | 5.4990 | -2.6583 |
| H | -1.2717 | 8.0585 | -2.1221 |
| C | -2.6290 | 5.8847 | 0.2416 |
| C | -2.2147 | 4.5600 | 0.1269 |
| H | -1.8066 | 4.1829 | -0.8098 |
| C | -2.3393 | 3.6919 | 1.2184 |
| H | -2.0254 | 2.6523 | 1.1325 |
| C | -2.8877 | 4.1556 | 2.4259 |
| N | -2.9838 | 3.1974 | 3.4818 |
| C | -3.8774 | 3.3267 | 4.4249 |
| C | -3.9842 | 2.3039 | 5.4824 |
| C | -3.6426 | 0.9680 | 5.2610 |
| H | -3.2401 | 0.6852 | 4.2863 |
| C | -3.8196 | 0.0271 | 6.2829 |
| O | -3.5236 | -1.3197 | 6.1729 |
| C | -3.0522 | -1.8006 | 4.8912 |
| H | -2.1327 | -1.2871 | 4.6014 |
| H | -2.8726 | -2.8620 | 5.1022 |
| H | -3.8406 | -1.6877 | 4.1254 |
| C | -4.3305 | 0.3977 | 7.5508 |
| C | -4.4841 | -0.6338 | 8.5945 |
| H | -3.8440 | -1.5239 | 8.4352 |
| N | -5.3169 | -0.4560 | 9.5772 |
| C | -5.4460 | -1.4237 | 10.6251 |
| C | -4.4725 | -2.4087 | 10.8997 |
| H | -3.5801 | -2.4897 | 10.2767 |
| C | -6.5925 | -1.3278 | 11.4322 |
| C | -6.7673 | -2.2071 | 12.5078 |
| C | -5.7981 | -3.1668 | 12.7904 |
| H | -7.6670 | -2.1326 | 13.1181 |
| H | -7.3433 | -0.5696 | 11.2152 |
| C | -4.6509 | 1.7424 | 7.7791 |
| H | -5.0239 | 2.0329 | 8.7629 |
| C | -4.4905 | 2.6799 | 6.7542 |
| O | -4.7848 | 4.0240 | 6.8482 |
| C | -5.3875 | 4.5040 | 8.0758 |
| H | -4.6650 | 4.4464 | 8.8934 |
| H | -5.6168 | 5.5475 | 7.8192 |
| H | -6.3099 | 3.9590 | 8.2995 |
| H | -4.6051 | 4.1515 | 4.5285 |
| C | -3.2978 | 5.5009 | 2.5491 |
| H | -3.6910 | 5.8793 | 3.4959 |
| C | -3.1894 | 6.3464 | 1.4531 |
| C | -3.6962 | 7.7720 | 1.3551 |
| C | -3.1247 | 8.7034 | 2.4444 |
| H | -2.0160 | 8.6443 | 2.4026 |
| C | -3.5721 | 8.4476 | 3.8887 |
| C | -2.7870 | 9.3150 | 4.8714 |
| H | -1.7094 | 9.1232 | 4.8134 |
| H | -3.0971 | 9.1176 | 5.9042 |
| H | -2.9427 | 10.3833 | 4.6830 |
| H | -3.4385 | 7.3763 | 4.1529 |
| H | -4.6536 | 8.6638 | 3.9972 |
| H | -3.3921 | 9.7505 | 2.1867 |
| C | -5.2132 | 7.7821 | 1.3376 |
| C | -5.7318 | 8.2603 | 0.1143 |
| C | -4.6458 | 8.5243 | -0.9138 |
| C | -4.6883 | 9.9556 | -1.4850 |
| H | -3.7662 | 10.1200 | -2.0833 |
| H | -4.6438 | 10.6682 | -0.6351 |
| C | -5.8879 | 10.3229 | -2.3668 |
| H | -5.8825 | 9.7101 | -3.2908 |
| C | -5.8581 | 11.8031 | -2.7451 |
| H | -4.9513 | 12.0577 | -3.3063 |
| H | -5.8891 | 12.4503 | -1.8614 |
| H | -6.7143 | 12.0669 | -3.3757 |
| H | -6.8396 | 10.0916 | -1.8433 |
| C | -7.1032 | 8.3835 | -0.0670 |
| H | -7.4960 | 8.7933 | -0.9998 |
| C | -7.9747 | 7.9920 | 0.9724 |
| C | -7.4597 | 7.4802 | 2.1754 |
| H | -8.1452 | 7.1700 | 2.9714 |
| N | -9.3977 | 8.1198 | 0.9034 |
| C | -10.0277 | 8.0999 | -0.2321 |
| H | -9.5682 | 7.9775 | -1.2312 |
| C | -11.4974 | 8.2481 | -0.2586 |
| C | -12.1531 | 8.4785 | -1.4927 |
| O | -11.3034 | 8.5272 | -2.5881 |
| C | -11.8102 | 9.1631 | -3.7884 |
| H | -10.9172 | 9.1851 | -4.4271 |
| H | -12.5998 | 8.5522 | -4.2358 |
| H | -12.1594 | 10.1757 | -3.5661 |
| C | -13.5443 | 8.5964 | -1.5747 |
| H | -14.0464 | 8.7661 | -2.5308 |
| C | -14.3224 | 8.4910 | -0.4172 |
| C | -13.6648 | 8.2907 | 0.8239 |
| O | -14.5262 | 8.2518 | 1.9024 |
| C | -13.9611 | 7.9880 | 3.2097 |
| H | -13.2576 | 8.7781 | 3.4878 |
| H | -13.4993 | 6.9962 | 3.2307 |
| H | -14.8559 | 8.0174 | 3.8461 |
| C | -15.7975 | 8.5497 | -0.4489 |
| H | -16.2814 | 8.1923 | 0.4814 |
| N | -16.4071 | 8.9774 | -1.5110 |
| C | -17.8360 | 9.0303 | -1.5686 |
| C | -18.4349 | 8.6935 | -2.7940 |
| H | -17.8137 | 8.3828 | -3.6328 |
| C | -19.8262 | 8.7497 | -2.9244 |
| H | -20.2903 | 8.4844 | -3.8765 |
| C | -20.6124 | 9.1484 | -1.8461 |
| C | -20.0062 | 9.5050 | -0.6222 |
| C | -21.0199 | 9.8771 | 0.4433 |
| C | -20.8004 | 11.2999 | 1.0010 |
| C | -19.5041 | 11.5569 | 1.7791 |
| H | -18.6216 | 11.3059 | 1.1543 |
| C | -19.4110 | 13.0152 | 2.2264 |
| H | -18.4778 | 13.2009 | 2.7703 |
| H | -19.4347 | 13.7037 | 1.3741 |
| H | -20.2371 | 13.2880 | 2.8928 |
| H | -19.4528 | 10.8981 | 2.6691 |
| H | -20.8436 | 12.0146 | 0.1523 |
| H | -21.6539 | 11.5476 | 1.6674 |
| C | -21.0515 | 8.8200 | 1.5293 |
| C | -20.0316 | 8.4871 | 2.4179 |
| H | -19.0770 | 9.0118 | 2.3872 |
| C | -20.2268 | 7.4703 | 3.3581 |
| H | -19.4361 | 7.2090 | 4.0599 |
| C | -21.4491 | 6.7771 | 3.3959 |
| N | -21.5950 | 5.7880 | 4.4199 |
| C | -22.2306 | 4.6789 | 4.2046 |
| C | -22.3602 | 3.6783 | 5.2848 |
| C | -21.5410 | 3.6923 | 6.4190 |
| H | -20.8067 | 4.4943 | 6.5230 |
| C | -21.6774 | 2.6944 | 7.3905 |
| O | -20.9247 | 2.6083 | 8.5469 |
| C | -19.8770 | 3.5894 | 8.7406 |
| H | -19.1332 | 3.5158 | 7.9417 |
| H | -19.4541 | 3.2748 | 9.7033 |
| H | -20.3057 | 4.5940 | 8.8090 |
| C | -22.6363 | 1.6580 | 7.2620 |
| C | -22.7088 | 0.6038 | 8.2954 |
| H | -21.8229 | 0.5595 | 8.9575 |
| N | -23.7468 | -0.1728 | 8.3503 |
| C | -23.8583 | -1.2164 | 9.3216 |
| C | -23.4264 | -1.0786 | 10.6578 |
| H | -22.9567 | -0.1534 | 10.9914 |
| C | -23.6222 | -2.1303 | 11.5455 |
| C | -24.2559 | -3.3178 | 11.1210 |
| C | -24.3511 | -4.3526 | 12.2246 |
| C | -25.8052 | -4.7573 | 12.5459 |
| H | -25.7860 | -5.4837 | 13.3857 |
| C | -26.6374 | -5.3612 | 11.4082 |
| H | -26.1143 | -6.2402 | 10.9742 |
| H | -26.7489 | -4.6299 | 10.5833 |
| C | -28.0216 | -5.7886 | 11.8966 |
| H | -27.9546 | -6.5359 | 12.6954 |
| H | -28.6033 | -6.2328 | 11.0808 |
| H | -28.6055 | -4.9466 | 12.2854 |
| H | -26.3311 | -3.8531 | 12.9180 |
| C | -24.6731 | -3.4521 | 9.7987 |
| H | -25.1421 | -4.3735 | 9.4557 |
| C | -24.4847 | -2.4008 | 8.8962 |
| H | -24.8145 | -2.4960 | 7.8627 |
| C | -23.4710 | 1.6575 | 6.1385 |
| H | -24.2239 | 0.8710 | 6.0483 |
| C | -23.3313 | 2.6519 | 5.1652 |
| O | -24.0968 | 2.7488 | 4.0173 |
| C | -25.0876 | 1.7188 | 3.7835 |
| H | -24.6061 | 0.7411 | 3.6892 |
| H | -25.5201 | 2.0400 | 2.8272 |
| H | -25.8401 | 1.7285 | 4.5781 |
| H | -22.7070 | 4.3737 | 3.2522 |
| C | -22.4818 | 7.1043 | 2.4916 |
| H | -23.4402 | 6.5842 | 2.5353 |
| C | -22.2723 | 8.1124 | 1.5584 |
| C | -23.2160 | 8.5378 | 0.4471 |
| C | -23.2886 | 7.4349 | -0.5919 |
| C | -22.6964 | 7.8060 | -1.8184 |
| C | -22.6255 | 6.8996 | -2.8699 |
| H | -22.1690 | 7.1822 | -3.8167 |
| C | -23.1613 | 5.6049 | -2.7008 |
| N | -23.1946 | 4.6298 | -3.7467 |
| C | -22.2168 | 4.4947 | -4.5880 |
| H | -21.2775 | 5.0807 | -4.5984 |
| C | -22.2970 | 3.4696 | -5.6499 |
| C | -21.3953 | 3.5274 | -6.7428 |
| O | -20.5425 | 4.6145 | -6.6970 |
| C | -19.5523 | 4.7368 | -7.7471 |
| H | -18.8750 | 3.8777 | -7.7344 |
| H | -19.0300 | 5.6563 | -7.4528 |
| H | -20.0410 | 4.8581 | -8.7186 |
| C | -21.4028 | 2.5577 | -7.7513 |
| H | -20.7099 | 2.6032 | -8.5945 |
| C | -22.3155 | 1.4986 | -7.6945 |
| C | -22.3377 | 0.4236 | -8.7097 |
| N | -21.7607 | 0.6019 | -9.8534 |
| C | -21.7590 | -0.4172 | -10.8600 |
| C | -20.6094 | -0.5058 | -11.6643 |
| H | -19.7676 | 0.1597 | -11.4781 |
| C | -20.5509 | -1.4512 | -12.6927 |
| H | -19.6564 | -1.5174 | -13.3148 |
| C | -21.6326 | -2.2973 | -12.9249 |
| C | -21.7302 | -3.4052 | -13.9556 |
| C | -20.7758 | -4.5291 | -13.5935 |
| C | -19.3867 | -4.4782 | -13.5851 |
| C | -18.6575 | -5.6131 | -13.1690 |
| N | -17.2299 | -5.6807 | -13.1916 |
| C | -16.4906 | -4.6483 | -12.9149 |
| C | -15.0184 | -4.7712 | -12.9482 |
| C | -14.3727 | -6.0128 | -12.9511 |
| C | -12.9752 | -6.0735 | -12.9311 |
| O | -12.2330 | -7.2404 | -12.9298 |
| C | -12.9555 | -8.4952 | -12.9054 |
| H | -12.1347 | -9.2238 | -12.9012 |
| H | -13.5548 | -8.5720 | -11.9934 |
| H | -13.5680 | -8.5980 | -13.8065 |
| C | -12.1806 | -4.8998 | -12.9098 |
| C | -10.7098 | -5.0220 | -12.8273 |
| N | -9.9611 | -3.9957 | -13.0823 |
| C | -8.5353 | -4.0620 | -12.9864 |
| C | -7.7801 | -5.1463 | -13.4802 |
| C | -6.3936 | -5.0992 | -13.3946 |
| C | -5.4144 | -6.2158 | -13.7202 |
| C | -5.5437 | -6.7431 | -15.1615 |
| H | -4.6900 | -7.4238 | -15.3664 |
| H | -5.4346 | -5.8823 | -15.8543 |
| C | -6.8351 | -7.4902 | -15.5168 |
| H | -6.9050 | -8.4292 | -14.9321 |
| C | -6.8913 | -7.8179 | -17.0080 |
| H | -6.0595 | -8.4625 | -17.3136 |
| H | -6.8502 | -6.9126 | -17.6245 |
| H | -7.8204 | -8.3421 | -17.2601 |
| H | -7.7227 | -6.8816 | -15.2388 |
| C | -5.7482 | -3.9616 | -12.8657 |
| C | -4.2387 | -4.0792 | -12.9174 |
| C | -3.5951 | -3.0147 | -13.8319 |
| H | -2.4853 | -3.0883 | -13.7162 |
| C | -4.0161 | -1.5588 | -13.5966 |
| C | -3.1784 | -0.5946 | -14.4352 |
| H | -2.1088 | -0.6797 | -14.2105 |
| H | -3.4684 | 0.4456 | -14.2502 |
| H | -3.3029 | -0.7803 | -15.5084 |
| H | -3.9172 | -1.3006 | -12.5209 |
| H | -5.0861 | -1.4302 | -13.8577 |
| H | -3.8207 | -3.2656 | -14.8900 |
| C | -6.4988 | -2.9102 | -12.3436 |
| H | -6.0112 | -2.0617 | -11.8687 |
| H | -8.2759 | -6.0061 | -13.9355 |
| C | -7.8946 | -2.9512 | -12.4095 |
| H | -8.4909 | -2.1315 | -12.0116 |
| H | -10.3578 | -6.0275 | -12.5239 |
| C | -12.8253 | -3.6579 | -12.9354 |
| H | -12.2141 | -2.7523 | -12.9412 |
| H | -14.9832 | -6.9186 | -12.9703 |
| C | -14.2230 | -3.5971 | -12.9532 |
| O | -14.9632 | -2.4297 | -12.9797 |
| C | -14.2390 | -1.1762 | -12.9286 |
| H | -13.5955 | -1.0742 | -13.8077 |
| H | -13.6740 | -1.1013 | -11.9945 |
| H | -15.0565 | -0.4445 | -12.9550 |
| H | -16.8564 | -3.6425 | -12.6343 |
| C | -19.3295 | -6.7786 | -12.7621 |
| H | -18.7568 | -7.6490 | -12.4449 |
| C | -20.7276 | -6.8110 | -12.7598 |
| H | -21.2421 | -7.7118 | -12.4260 |
| H | -18.8626 | -3.5789 | -13.9038 |
| C | -21.4515 | -5.6981 | -13.1815 |
| C | -22.9597 | -5.5404 | -13.2101 |
| C | -23.4945 | -5.5383 | -11.7887 |
| C | -23.5053 | -6.6049 | -10.8980 |
| H | -23.1737 | -7.5938 | -11.2159 |
| C | -23.9698 | -6.3983 | -9.5817 |
| N | -24.0622 | -7.4460 | -8.6103 |
| C | -23.1704 | -8.3798 | -8.5285 |
| H | -22.2674 | -8.4818 | -9.1629 |
| C | -23.3022 | -9.4363 | -7.5021 |
| C | -22.5299 | -10.6198 | -7.6176 |
| O | -21.7438 | -10.6563 | -8.7547 |
| C | -20.8947 | -11.8138 | -8.9458 |
| H | -20.1659 | -11.8898 | -8.1335 |
| H | -20.4026 | -11.5732 | -9.8968 |
| H | -21.5020 | -12.7198 | -9.0358 |
| C | -22.5875 | -11.6300 | -6.6519 |
| H | -21.9969 | -12.5442 | -6.7450 |
| C | -23.4196 | -11.4828 | -5.5364 |
| C | -23.4839 | -12.4959 | -4.4625 |
| N | -23.0331 | -13.6936 | -4.6757 |
| C | -23.0719 | -14.7012 | -3.6609 |
| C | -22.0120 | -15.6248 | -3.6550 |
| H | -21.2055 | -15.5256 | -4.3800 |
| C | -21.9967 | -16.6601 | -2.7152 |
| H | -21.1727 | -17.3764 | -2.7131 |
| C | -23.0329 | -16.7794 | -1.7922 |
| C | -23.1565 | -17.7966 | -0.6746 |
| C | -23.1474 | -19.2524 | -1.1861 |
| C | -21.8573 | -19.7577 | -1.8435 |
| H | -21.0225 | -19.7393 | -1.1135 |
| C | -22.0302 | -21.1799 | -2.3749 |
| H | -21.1048 | -21.5414 | -2.8377 |
| H | -22.2895 | -21.8823 | -1.5745 |
| H | -22.8199 | -21.2382 | -3.1323 |
| H | -21.5577 | -19.0869 | -2.6755 |
| H | -23.3814 | -19.9242 | -0.3332 |
| H | -23.9773 | -19.3657 | -1.9153 |
| C | -24.1041 | -15.8607 | -1.8092 |
| C | -25.1124 | -16.1244 | -0.7057 |
| C | -26.5401 | -16.3537 | -1.2451 |
| H | -26.5071 | -17.2051 | -1.9574 |
| C | -27.2263 | -15.1721 | -1.9413 |
| H | -26.5983 | -14.7970 | -2.7764 |
| H | -27.3433 | -14.3257 | -1.2353 |
| C | -28.5992 | -15.5732 | -2.4795 |
| H | -28.5276 | -16.3903 | -3.2064 |
| H | -29.0852 | -14.7304 | -2.9842 |
| H | -29.2682 | -15.9038 | -1.6771 |
| H | -27.1851 | -16.6733 | -0.3992 |
| C | -24.1319 | -14.8271 | -2.7375 |
| H | -24.9713 | -14.1315 | -2.7632 |
| H | -23.9264 | -12.1222 | -3.5189 |
| C | -24.2064 | -10.3080 | -5.4307 |
| O | -25.0041 | -10.2781 | -4.3015 |
| C | -25.8253 | -9.1038 | -4.0912 |
| H | -25.1976 | -8.2149 | -3.9778 |
| H | -26.3310 | -9.3527 | -3.1494 |
| H | -26.5463 | -8.9933 | -4.9071 |
| C | -24.1519 | -9.2996 | -6.3985 |
| H | -24.7630 | -8.3976 | -6.3189 |
| C | -24.4246 | -5.1302 | -9.1799 |
| C | -24.4077 | -4.0655 | -10.0860 |
| H | -24.7485 | -3.0819 | -9.7631 |
| H | -24.7833 | -4.9829 | -8.1622 |
| C | -23.9556 | -4.2657 | -11.3883 |
| C | -23.8401 | -3.2319 | -12.4913 |
| C | -25.1956 | -2.5966 | -12.8665 |
| H | -25.0273 | -1.8988 | -13.7140 |
| C | -25.9463 | -1.8406 | -11.7634 |
| H | -25.3065 | -1.0380 | -11.3403 |
| H | -26.1848 | -2.5231 | -10.9232 |
| C | -27.2402 | -1.2284 | -12.2987 |
| H | -27.0475 | -0.5203 | -13.1127 |
| H | -27.7716 | -0.6820 | -11.5112 |
| H | -27.9227 | -1.9949 | -12.6828 |
| H | -25.8629 | -3.4000 | -13.2446 |
| C | -23.6609 | -6.5743 | -14.1153 |
| H | -23.3024 | -6.4251 | -15.1558 |
| C | -23.4801 | -8.0553 | -13.7610 |
| H | -22.4073 | -8.3321 | -13.8022 |
| H | -23.8105 | -8.2464 | -12.7183 |
| C | -24.2691 | -8.9505 | -14.7156 |
| H | -23.9382 | -8.8279 | -15.7530 |
| H | -24.1448 | -10.0079 | -14.4555 |
| H | -25.3426 | -8.7316 | -14.6843 |
| H | -24.7483 | -6.3489 | -14.1200 |
| C | -21.5433 | -2.8978 | -15.4008 |
| C | -20.1749 | -2.3104 | -15.7674 |
| H | -19.3842 | -3.0801 | -15.6548 |
| C | -20.1674 | -1.7860 | -17.2027 |
| H | -19.1860 | -1.3751 | -17.4658 |
| H | -20.3922 | -2.5793 | -17.9248 |
| H | -20.9053 | -0.9889 | -17.3476 |
| H | -19.9080 | -1.4871 | -15.0730 |
| H | -21.7517 | -3.7388 | -16.0957 |
| H | -22.3167 | -2.1254 | -15.5974 |
| C | -23.1885 | -4.0353 | -13.7194 |
| C | -24.0533 | -3.9733 | -14.9711 |
| H | -23.6027 | -4.5277 | -15.8059 |
| H | -24.2025 | -2.9404 | -15.3141 |
| H | -25.0501 | -4.4026 | -14.7999 |
| C | -22.7901 | -2.1998 | -12.1229 |
| C | -22.8611 | -1.2660 | -11.0967 |
| H | -23.7639 | -1.1787 | -10.4914 |
| H | -22.8694 | -0.4912 | -8.3789 |
| C | -23.2297 | 1.4512 | -6.6116 |
| O | -24.0944 | 0.3731 | -6.6671 |
| C | -25.0464 | 0.2210 | -5.5864 |
| H | -24.5242 | 0.0772 | -4.6359 |
| H | -25.5790 | -0.6904 | -5.8867 |
| H | -25.7245 | 1.0794 | -5.5536 |
| C | -23.2237 | 2.4216 | -5.6048 |
| H | -23.9319 | 2.3868 | -4.7737 |
| C | -23.7535 | 5.2351 | -1.4808 |
| H | -24.1644 | 4.2336 | -1.3615 |
| C | -23.8023 | 6.1507 | -0.4250 |
| H | -24.2383 | 5.8463 | 0.5257 |
| C | -24.5995 | 8.9799 | 0.9650 |
| H | -24.4475 | 9.7974 | 1.7010 |
| C | -25.4809 | 7.9049 | 1.6128 |
| H | -24.9307 | 7.3955 | 2.4327 |
| H | -25.7335 | 7.1200 | 0.8724 |
| C | -26.7705 | 8.5099 | 2.1658 |
| H | -26.5679 | 9.2774 | 2.9216 |
| H | -27.3932 | 7.7428 | 2.6400 |
| H | -27.3702 | 8.9755 | 1.3756 |
| H | -25.1663 | 9.4197 | 0.1169 |
| C | -22.4380 | 9.7285 | -0.2961 |
| C | -23.2272 | 11.0304 | -0.2901 |
| H | -22.6881 | 11.8359 | -0.8068 |
| H | -23.4287 | 11.3808 | 0.7315 |
| H | -24.1996 | 10.9206 | -0.7902 |
| C | -22.1261 | 9.2130 | -1.7847 |
| C | -22.7324 | 10.1369 | -2.8610 |
| H | -22.2989 | 11.1508 | -2.7290 |
| C | -22.5468 | 9.7226 | -4.3260 |
| H | -21.4696 | 9.5868 | -4.5566 |
| H | -23.0293 | 8.7414 | -4.5115 |
| C | -23.1413 | 10.7641 | -5.2729 |
| H | -22.6671 | 11.7441 | -5.1490 |
| H | -23.0078 | 10.4656 | -6.3191 |
| H | -24.2168 | 10.8948 | -5.1069 |
| H | -23.8219 | 10.2306 | -2.6673 |
| C | -18.6259 | 9.4501 | -0.4771 |
| H | -18.1571 | 9.7423 | 0.4628 |
| C | -12.2749 | 8.1602 | 0.9027 |
| H | -11.7724 | 7.9949 | 1.8574 |
| C | -6.0761 | 7.3662 | 2.3490 |
| H | -5.6871 | 6.9417 | 3.2736 |
| C | -3.2783 | 8.2330 | -0.1243 |
| C | -2.3426 | 9.4340 | -0.1054 |
| H | -1.4015 | 9.2027 | 0.4177 |
| H | -2.0768 | 9.7598 | -1.1205 |
| H | -2.7930 | 10.2977 | 0.4019 |
| C | -4.6979 | 7.4487 | -1.9834 |
| C | -5.7136 | 7.2115 | -2.9077 |
| H | -6.6035 | 7.8378 | -2.9276 |
| C | -5.6022 | 6.1551 | -3.8196 |
| H | -6.3885 | 5.9721 | -4.5519 |
| C | -4.3037 | 2.1863 | -6.7783 |
| H | -4.6847 | 3.1662 | -7.0956 |
| C | -4.2431 | 1.1229 | -7.6911 |
| O | -5.8833 | 0.6162 | -9.3152 |
| C | -4.6383 | 1.3388 | -9.1236 |
| H | -3.8809 | 0.9365 | -9.8221 |
| H | -6.0438 | 0.4754 | -10.2662 |
| H | -4.8280 | 2.4045 | -9.3565 |
| H | -4.9817 | -1.4296 | -8.5490 |
| C | -3.6027 | -2.9743 | -10.6544 |
| H | -3.9564 | -1.9948 | -10.9771 |
| C | -3.6578 | -4.0614 | -11.5169 |
| C | -3.9722 | -5.5749 | -13.4331 |
| C | -3.0602 | -5.6066 | -14.6517 |
| H | -2.0700 | -5.1881 | -14.4285 |
| H | -2.9036 | -6.6317 | -15.0161 |
| H | -3.4765 | -5.0273 | -15.4878 |
| C | -5.5606 | -7.2887 | -12.6564 |
| C | -6.6735 | -8.0932 | -12.4202 |
| H | -7.5579 | -8.0225 | -13.0522 |
| C | -6.6674 | -8.9978 | -11.3544 |
| H | -7.5252 | -9.6444 | -11.1739 |
| C | -6.0697 | -11.9765 | -7.3671 |
| H | -6.7015 | -12.0441 | -8.2557 |
| C | -6.1539 | -12.9195 | -6.3369 |
| O | -7.0148 | -14.0011 | -6.3238 |
| C | -7.8732 | -14.1903 | -7.4749 |
| H | -7.2722 | -14.3445 | -8.3766 |
| H | -8.4121 | -15.1061 | -7.2000 |
| H | -8.5605 | -13.3457 | -7.5806 |
| H | -6.4239 | -14.4221 | -4.1626 |
| C | -5.1792 | -16.1632 | -2.2544 |
| H | -5.6228 | -16.4708 | -3.1983 |
| C | -5.1108 | -17.0416 | -1.1785 |
| C | -5.5706 | -17.2739 | 2.2066 |
| C | -5.3700 | -16.2556 | 3.1293 |
| H | -4.4041 | -15.7447 | 3.1808 |
| C | -6.4185 | -15.9037 | 4.0054 |
| N | -6.2912 | -14.8819 | 5.0000 |
| C | -5.6024 | -13.8077 | 4.7664 |
| H | -5.0693 | -13.5690 | 3.8246 |
| C | -5.4825 | -12.7585 | 5.7990 |
| C | -4.4730 | -11.7718 | 5.6628 |
| O | -3.6744 | -11.9533 | 4.5492 |
| C | -2.5075 | -11.1066 | 4.4142 |
| H | -1.8546 | -11.2126 | 5.2864 |
| H | -2.0310 | -11.5280 | 3.5185 |
| H | -2.8132 | -10.0697 | 4.2508 |
| C | -4.3460 | -10.7218 | 6.5781 |
| H | -3.5715 | -9.9595 | 6.4684 |
| C | -5.2257 | -10.6327 | 7.6626 |
| C | -5.1831 | -9.5234 | 8.6298 |
| H | -6.0835 | -9.4275 | 9.2574 |
| C | -6.2131 | -11.6385 | 7.8173 |
| O | -7.0040 | -11.4681 | 8.9387 |
| C | -8.0179 | -12.4677 | 9.2034 |
| H | -7.5560 | -13.4492 | 9.3489 |
| H | -8.4564 | -12.0968 | 10.1386 |
| H | -8.7586 | -12.4800 | 8.3985 |
| C | -6.3451 | -12.6855 | 6.8976 |
| H | -7.1081 | -13.4581 | 7.0147 |
| C | -7.6435 | -16.5907 | 3.9562 |
| C | -7.8266 | -17.6253 | 3.0323 |
| H | -8.7851 | -18.1406 | 2.9907 |
| H | -8.4468 | -16.3093 | 4.6356 |
| C | -5.6517 | -18.4599 | -1.1171 |
| C | -5.0026 | -19.4027 | -2.1504 |
| H | -3.9278 | -19.5181 | -1.8958 |
| C | -5.0922 | -18.9917 | -3.6253 |
| C | -4.5710 | -20.0994 | -4.5396 |
| H | -3.5227 | -20.3392 | -4.3283 |
| H | -4.6304 | -19.8005 | -5.5925 |
| H | -5.1489 | -21.0239 | -4.4291 |
| H | -4.5041 | -18.0677 | -3.7989 |
| H | -6.1418 | -18.7501 | -3.8977 |
| H | -5.4641 | -20.4061 | -2.0357 |
| C | -6.7918 | -17.9804 | 2.1699 |
| C | -7.0325 | -20.4720 | 1.6899 |
| H | -6.1812 | -20.7201 | 2.3590 |
| H | -6.9988 | -21.1998 | 0.8521 |
| C | -8.3287 | -20.7004 | 2.4774 |
| H | -8.3314 | -20.0806 | 3.3960 |
| C | -8.4861 | -22.1714 | 2.8609 |
| H | -7.6562 | -22.5176 | 3.4871 |
| H | -8.5250 | -22.8190 | 1.9774 |
| H | -9.4117 | -22.3345 | 3.4247 |
| H | -9.2087 | -20.3791 | 1.8812 |
| C | -7.1647 | -18.4206 | -1.2149 |
| C | -7.9310 | -18.0837 | -2.3278 |
| H | -7.4465 | -17.8791 | -3.2859 |
| H | -9.6612 | -18.9133 | 1.0732 |
| C | -9.3242 | -18.0135 | -2.2228 |
| H | -9.9302 | -17.7465 | -3.0873 |
| H | -11.5468 | -17.4822 | 1.0499 |
| C | -14.2266 | -17.6734 | -1.0968 |
| H | -13.6965 | -17.7566 | -2.0482 |
| H | -16.1005 | -17.4476 | 2.3290 |
| C | -15.6183 | -17.5394 | -1.0432 |
| O | -16.4512 | -17.4905 | -2.1460 |
| C | -15.8350 | -17.5543 | -3.4551 |
| H | -15.3120 | -18.5072 | -3.5824 |
| H | -15.1688 | -16.6997 | -3.6046 |
| H | -16.7097 | -17.4930 | -4.1153 |
| H | -18.1855 | -16.9362 | -0.7889 |
| C | -20.7202 | -17.6495 | 0.2294 |
| H | -20.2925 | -18.0013 | -0.7075 |
| H | -22.2271 | -16.3646 | 3.5940 |
| C | -22.0966 | -17.5316 | 0.3796 |
| C | -24.5373 | -17.4181 | 0.0522 |
| C | -25.5271 | -18.5752 | 0.0477 |
| H | -25.1284 | -19.4559 | 0.5701 |
| H | -25.7793 | -18.8929 | -0.9731 |
| H | -26.4701 | -18.3068 | 0.5434 |
| C | -24.8762 | -17.7818 | 2.6171 |
| H | -24.6302 | -18.8517 | 2.4480 |
| C | -24.5690 | -17.4511 | 4.0827 |
| H | -23.4886 | -17.5889 | 4.2900 |
| H | -24.7920 | -16.3839 | 4.2929 |
| C | -25.3815 | -18.3343 | 5.0286 |
| H | -25.1546 | -19.3969 | 4.8863 |
| H | -25.1663 | -18.0912 | 6.0754 |
| H | -26.4596 | -18.2047 | 4.8794 |
| H | -25.9711 | -17.6805 | 2.4612 |
| C | -25.0422 | -15.0220 | 0.3324 |
| C | -25.3675 | -13.6785 | 0.1601 |
| H | -25.7342 | -13.3151 | -0.7991 |
| C | -25.2203 | -12.7816 | 1.2243 |
| H | -25.4800 | -11.7315 | 1.0993 |
| C | -24.3965 | -10.0696 | 5.3631 |
| H | -25.0585 | -9.9263 | 4.5058 |
| C | -24.2869 | -9.1062 | 6.3719 |
| O | -24.9718 | -7.9051 | 6.3962 |
| C | -25.8463 | -7.6094 | 5.2803 |
| H | -25.2720 | -7.5537 | 4.3508 |
| H | -26.2395 | -6.6241 | 5.5611 |
| H | -26.6495 | -8.3504 | 5.2206 |
| H | -23.6944 | -7.2402 | 8.1569 |
| C | -23.6562 | -6.4618 | 10.8709 |
| H | -24.5419 | -6.4082 | 10.2366 |
| C | -23.4703 | -5.5432 | 11.8968 |
| C | -23.6283 | -3.6594 | 13.4802 |
| C | -24.5347 | -3.5871 | 14.7016 |
| H | -24.0326 | -3.1099 | 15.5548 |
| H | -24.8546 | -4.5845 | 15.0321 |
| H | -25.4463 | -3.0079 | 14.4998 |
| C | -23.1515 | -2.2078 | 12.9874 |
| C | -23.7160 | -1.0768 | 13.8707 |
| H | -23.4128 | -1.2731 | 14.9209 |
| C | -23.3062 | 0.3589 | 13.5201 |
| H | -22.2039 | 0.4682 | 13.5727 |
| H | -23.5925 | 0.5975 | 12.4744 |
| C | -23.9586 | 1.3651 | 14.4672 |
| H | -23.6626 | 1.1933 | 15.5083 |
| H | -23.6701 | 2.3908 | 14.2103 |
| H | -25.0525 | 1.3138 | 14.4231 |
| H | -24.8246 | -1.1369 | 13.8451 |
| C | -19.8052 | -3.8265 | 13.4443 |
| H | -19.4397 | -4.8031 | 13.7558 |
| C | -13.4770 | -5.7260 | 12.9694 |
| H | -13.0275 | -6.7207 | 13.0108 |
| C | -7.3072 | -7.5099 | 12.8602 |
| H | -6.9265 | -8.4920 | 12.5752 |
| C | -5.8245 | -4.2228 | 13.8790 |
| C | -5.9111 | -3.6293 | 15.3001 |
| H | -5.0061 | -3.0097 | 15.4745 |
| H | -5.8662 | -4.4683 | 16.0269 |
| C | -7.1398 | -2.7741 | 15.6317 |
| H | -7.1668 | -1.8746 | 14.9848 |
| C | -7.1290 | -2.3444 | 17.0983 |
| H | -6.2375 | -1.7559 | 17.3425 |
| H | -7.1493 | -3.2071 | 17.7742 |
| H | -8.0035 | -1.7270 | 17.3334 |
| H | -8.0730 | -3.3375 | 15.4204 |
| C | -4.6480 | -3.2649 | 11.9778 |
| C | -4.4045 | -6.6559 | 11.7808 |
| N | -4.1267 | -8.7373 | 8.6809 |
| C | -4.5665 | -7.7378 | 10.9233 |
| H | -5.0709 | -8.6418 | 11.2609 |
| C | 8.9536 | 3.1966 | 10.3244 |
| H | 8.4334 | 2.5586 | 11.0488 |
| H | 8.8524 | 4.2521 | 10.5966 |
| H | 10.0024 | 2.9053 | 10.2309 |

l) (**OMe-cube)**(**OMe-cube)**(**OMe-cube)** (Type 1 conformer, 1 and 3 position of middle cage is interlocked).

| **atom** | **x** | **y** | **z** |
| --- | --- | --- | --- |
| C | 13.8121 | 15.4425 | 0.5658 |
| C | 13.1402 | 16.6136 | -1.6240 |
| C | 12.3954 | 17.5874 | 0.6400 |
| C | 13.6225 | 16.8845 | -0.1166 |
| C | 13.5693 | 14.4616 | -0.5661 |
| C | 13.2024 | 15.1056 | -1.7689 |
| C | 12.9266 | 14.3508 | -2.9056 |
| C | 13.0014 | 12.9542 | -2.8575 |
| N | 13.4417 | 10.8821 | -1.6788 |
| C | 13.3539 | 12.3144 | -1.6583 |
| C | 13.6562 | 13.0772 | -0.5076 |
| C | 11.6974 | 17.0833 | -1.6409 |
| C | 11.2902 | 17.6339 | -0.4026 |
| C | 9.9866 | 18.0760 | -0.2283 |
| C | 9.0682 | 17.9887 | -1.2804 |
| N | 8.4927 | 17.4177 | -3.5645 |
| C | 9.4679 | 17.4187 | -2.5074 |
| C | 10.7873 | 16.9612 | -2.6843 |
| C | 11.9923 | 16.5848 | 1.7075 |
| C | 12.7091 | 15.3730 | 1.6035 |
| C | 12.3546 | 14.2849 | 2.3972 |
| C | 11.3333 | 14.4141 | 3.3448 |
| N | 9.6678 | 15.7177 | 4.5161 |
| C | 10.6528 | 15.6364 | 3.4789 |
| C | 10.9829 | 16.7309 | 2.6523 |
| C | 13.6702 | -0.9219 | -0.7876 |
| C | 13.0015 | -1.8696 | 1.5066 |
| C | 12.1531 | -2.9980 | -0.6562 |
| C | 13.4334 | -2.2893 | 0.0170 |
| C | 13.4137 | 0.1641 | 0.2406 |
| C | 12.9874 | -0.3531 | 1.4816 |
| C | 12.5953 | 0.5157 | 2.4974 |
| C | 12.6730 | 1.8994 | 2.3038 |
| N | 13.2407 | 3.8364 | 0.9740 |
| C | 13.1351 | 2.4125 | 1.0791 |
| C | 13.5098 | 1.5344 | 0.0396 |
| C | 11.5889 | -2.4079 | 1.6410 |
| C | 11.1051 | -2.9739 | 0.4402 |
| C | 9.7868 | -3.4170 | 0.3661 |
| C | 8.9582 | -3.3555 | 1.4944 |
| N | 8.5749 | -2.8670 | 3.8327 |
| C | 9.4516 | -2.8302 | 2.6996 |
| C | 10.7768 | -2.3445 | 2.7680 |
| C | 11.7664 | -2.0682 | -1.7916 |
| C | 12.5694 | -0.9058 | -1.8308 |
| C | 12.2796 | 0.1058 | -2.7427 |
| C | 11.2333 | -0.0563 | -3.6557 |
| N | 9.4289 | -1.2935 | -4.6333 |
| C | 10.4617 | -1.2342 | -3.6432 |
| C | 10.7318 | -2.2466 | -2.6993 |
| C | -1.4642 | -1.8973 | 13.2054 |
| C | 0.8098 | -2.8842 | 12.5225 |
| C | 0.7161 | -0.8055 | 14.0388 |
| C | 0.0115 | -2.2195 | 13.7485 |
| C | -1.4709 | -2.4578 | 11.7988 |
| C | -0.1962 | -2.8984 | 11.3881 |
| C | 0.0155 | -3.2851 | 10.0715 |
| C | -1.0603 | -3.3105 | 9.1669 |
| N | -3.4056 | -3.0305 | 8.6364 |
| C | -2.3402 | -2.9289 | 9.5915 |
| C | -2.5529 | -2.4916 | 10.9152 |
| C | 1.9482 | -1.9187 | 12.2541 |
| C | 1.8886 | -0.7669 | 13.0736 |
| C | 2.8295 | 0.2444 | 12.9166 |
| C | 3.8497 | 0.1205 | 11.9670 |
| N | 5.0190 | -1.1184 | 10.2510 |
| C | 3.9229 | -1.0376 | 11.1731 |
| C | 2.9659 | -2.0653 | 11.3230 |
| C | -0.3258 | 0.2194 | 13.6277 |
| C | -1.5060 | -0.3801 | 13.1340 |
| C | -2.5267 | 0.4106 | 12.6084 |
| C | -2.3967 | 1.8030 | 12.6030 |
| N | -1.1932 | 3.8289 | 13.1353 |
| C | -1.2285 | 2.3999 | 13.1075 |
| C | -0.1814 | 1.6004 | 13.6162 |
| C | 4.8522 | -1.7251 | 9.1177 |
| C | 5.9314 | -1.8375 | 8.1175 |
| C | 7.0571 | -1.0080 | 8.0790 |
| C | 7.9492 | -1.1104 | 7.0051 |
| C | 7.7257 | -2.0052 | 5.9234 |
| C | 6.6396 | -2.8825 | 6.0025 |
| C | 5.7796 | -2.8155 | 7.1037 |
| C | 8.6310 | -1.9551 | 4.7555 |
| C | -0.7009 | 15.6190 | 13.8369 |
| C | 1.5459 | 16.6328 | 13.0889 |
| C | -0.6713 | 17.6978 | 12.3222 |
| C | 0.0565 | 17.0088 | 13.5746 |
| C | 0.3325 | 14.5580 | 13.5013 |
| C | 1.5640 | 15.1156 | 13.0914 |
| C | 2.6055 | 14.2838 | 12.6889 |
| C | 2.4450 | 12.8964 | 12.7121 |
| N | 1.1471 | 10.9142 | 13.1576 |
| C | 1.2160 | 12.3413 | 13.1194 |
| C | 0.1559 | 13.1782 | 13.5205 |
| C | 1.6062 | 17.1417 | 11.6604 |
| C | 0.3851 | 17.7030 | 11.2338 |
| C | 0.2409 | 18.1477 | 9.9204 |
| C | 1.3159 | 18.0544 | 9.0299 |
| N | 3.5917 | 17.4805 | 8.4949 |
| C | 2.5315 | 17.4942 | 9.4543 |
| C | 2.6814 | 17.0382 | 10.7839 |
| C | -1.7845 | 16.7293 | 11.9638 |
| C | -1.8027 | 15.5932 | 12.7971 |
| C | -2.7404 | 14.5841 | 12.5838 |
| C | -3.6668 | 14.7012 | 11.5375 |
| N | -4.6384 | 15.8814 | 9.6963 |
| C | -3.6382 | 15.8276 | 10.7012 |
| C | -2.6970 | 16.8578 | 10.9189 |
| C | 0.0540 | 10.2736 | 12.8785 |
| C | 0.0378 | 8.7963 | 12.9266 |
| C | 1.2154 | 8.0408 | 12.9461 |
| C | 1.1484 | 6.6427 | 12.9452 |
| C | -0.0943 | 5.9604 | 12.9247 |
| C | -1.2724 | 6.7155 | 12.9290 |
| C | -1.2048 | 8.1132 | 12.9301 |
| C | 8.5534 | 16.3503 | 4.3438 |
| C | 7.5538 | 16.4100 | 5.4321 |
| C | 7.6222 | 15.5831 | 6.5601 |
| C | 6.6199 | 15.6438 | 7.5321 |
| C | 5.5194 | 16.5314 | 7.4144 |
| C | 5.4669 | 17.3790 | 6.3008 |
| C | 6.4687 | 17.3184 | 5.3290 |
| C | 13.1585 | 5.9109 | -0.2534 |
| C | 4.4591 | 16.5124 | 8.4421 |
| C | -0.1005 | 4.4841 | 12.8789 |
| C | 13.0578 | 4.4392 | -0.1611 |
| C | -13.7351 | -0.9086 | 0.7830 |
| C | -13.0667 | -1.8691 | -1.5088 |
| C | -12.2422 | -3.0010 | 0.6518 |
| C | -13.5102 | -2.2790 | -0.0209 |
| C | -13.4639 | 0.1739 | -0.2442 |
| C | -13.0395 | -0.3509 | -1.4854 |
| C | -12.6360 | 0.5110 | -2.4999 |
| C | -12.7050 | 1.8979 | -2.3131 |
| N | -13.2702 | 3.8431 | -0.9934 |
| C | -13.1658 | 2.4184 | -1.0908 |
| C | -13.5472 | 1.5473 | -0.0497 |
| C | -11.6587 | -2.4190 | -1.6397 |
| C | -11.1856 | -2.9872 | -0.4360 |
| C | -9.8711 | -3.4419 | -0.3583 |
| C | -9.0405 | -3.3878 | -1.4845 |
| N | -8.6497 | -2.9069 | -3.8250 |
| C | -9.5276 | -2.8616 | -2.6926 |
| C | -10.8482 | -2.3668 | -2.7654 |
| C | -11.8453 | -2.0750 | 1.7865 |
| C | -12.6368 | -0.9066 | 1.8304 |
| C | -12.3425 | 0.1003 | 2.7463 |
| C | -11.2959 | -0.0714 | 3.6612 |
| N | -9.5293 | -1.3410 | 4.6507 |
| C | -10.5298 | -1.2517 | 3.6446 |
| C | -10.8077 | -2.2580 | 2.6940 |
| C | -8.5008 | -2.1646 | 4.5626 |
| C | -7.5008 | -2.1756 | 5.6493 |
| C | -4.9180 | -1.7614 | -9.1041 |
| C | -5.9972 | -1.8771 | -8.1021 |
| C | 4.2917 | -2.1116 | -8.6255 |
| C | 5.3886 | -2.2021 | -7.6409 |
| C | -7.1260 | -1.0505 | -8.0688 |
| C | -8.0192 | -1.1552 | -6.9965 |
| C | -7.7959 | -2.0496 | -5.9140 |
| C | -6.7071 | -2.9250 | -5.9891 |
| C | -5.8449 | -2.8545 | -7.0877 |
| C | -8.7027 | -1.9981 | -4.7477 |
| C | 0.6978 | 15.5592 | -13.8854 |
| C | -1.5436 | 16.5860 | -13.1457 |
| C | 0.6717 | 17.6469 | -12.3802 |
| C | -0.0581 | 16.9529 | -13.6310 |
| C | -0.3411 | 14.5049 | -13.5466 |
| C | -1.5689 | 15.0699 | -13.1402 |
| C | -2.6179 | 14.2443 | -12.7382 |
| C | -2.4591 | 12.8551 | -12.7512 |
| N | -1.1691 | 10.8688 | -13.1787 |
| C | -1.2329 | 12.2943 | -13.1476 |
| C | -0.1644 | 13.1267 | -13.5479 |
| C | -1.6060 | 17.0955 | -11.7160 |
| C | -0.3813 | 17.6539 | -11.2893 |
| C | -0.2372 | 18.1012 | -9.9786 |
| C | -1.3115 | 18.0129 | -9.0880 |
| N | -3.5917 | 17.4448 | -8.5519 |
| C | -2.5319 | 17.4585 | -9.5141 |
| C | -2.6813 | 17.0011 | -10.8404 |
| C | 1.7865 | 16.6825 | -12.0181 |
| C | 1.8020 | 15.5397 | -12.8466 |
| C | 2.7394 | 14.5332 | -12.6300 |
| C | 3.6644 | 14.6500 | -11.5883 |
| N | 4.6404 | 15.8446 | -9.7309 |
| C | 3.6377 | 15.7823 | -10.7556 |
| C | 2.6981 | 16.8108 | -10.9775 |
| C | -0.0758 | 10.2212 | -12.8850 |
| C | 4.4104 | 16.3948 | -8.5915 |
| C | -0.0691 | 8.7462 | -12.9322 |
| C | 5.4719 | 16.4291 | -7.5619 |
| C | -1.2502 | 7.9946 | -12.9506 |
| C | -1.1893 | 6.5990 | -12.9501 |
| C | 0.0503 | 5.9091 | -12.9341 |
| C | 1.2327 | 6.6596 | -12.9376 |
| C | 1.1700 | 8.0553 | -12.9363 |
| C | 6.5957 | 15.5966 | -7.6319 |
| C | 7.5515 | 15.6312 | -6.6112 |
| C | 7.4173 | 16.4931 | -5.4939 |
| C | 6.3058 | 17.3438 | -5.4369 |
| C | 5.3493 | 17.3083 | -6.4559 |
| C | -13.1390 | 16.6288 | 1.5669 |
| C | -12.3913 | 17.5937 | -0.7014 |
| C | -13.8125 | 15.4506 | -0.6167 |
| C | -13.6195 | 16.8958 | 0.0587 |
| C | -11.6944 | 17.0897 | 1.5807 |
| C | -11.2908 | 17.6410 | 0.3457 |
| C | -9.9809 | 18.0809 | 0.1649 |
| C | -9.0668 | 18.0001 | 1.2230 |
| N | -8.4900 | 17.4407 | 3.4955 |
| C | -9.4657 | 17.4472 | 2.4491 |
| C | -10.7901 | 16.9853 | 2.6304 |
| C | -11.9903 | 16.5842 | -1.7641 |
| C | -12.7096 | 15.3746 | -1.6539 |
| C | -12.3584 | 14.2820 | -2.4442 |
| C | -11.3357 | 14.4052 | -3.3909 |
| N | -9.6693 | 15.7019 | -4.5712 |
| C | -10.6526 | 15.6248 | -3.5326 |
| C | -10.9807 | 16.7230 | -2.7087 |
| C | -13.5717 | 14.4767 | 0.5205 |
| C | -13.2010 | 15.1228 | 1.7211 |
| C | -12.9247 | 14.3739 | 2.8575 |
| C | -13.0035 | 12.9748 | 2.8199 |
| N | -13.4531 | 10.8968 | 1.6434 |
| C | -13.3549 | 12.3303 | 1.6239 |
| C | -13.6624 | 13.0905 | 0.4752 |
| C | -7.5511 | 16.3889 | -5.4881 |
| C | -8.4333 | 16.4837 | 4.3673 |
| C | -13.0781 | 10.2175 | 0.6053 |
| C | -7.4130 | 16.5220 | 5.4363 |
| C | -13.1800 | 8.7460 | 0.5467 |
| C | -6.3020 | 17.3709 | 5.3761 |
| C | -5.3448 | 17.3394 | 6.3963 |
| C | -5.4687 | 16.4631 | 7.5032 |
| C | -6.5918 | 15.6322 | 7.5754 |
| C | -7.5484 | 15.6624 | 6.5542 |
| C | -7.6198 | 15.5550 | -6.6086 |
| C | -6.6154 | 15.6130 | -7.5857 |
| C | -5.5191 | 16.5021 | -7.4707 |
| C | -5.4675 | 17.3590 | -6.3663 |
| C | -6.4679 | 17.2968 | -5.3861 |
| C | -13.3047 | 7.9201 | 1.6680 |
| C | -13.3139 | 6.5292 | 1.5033 |
| C | -13.1825 | 5.9241 | 0.2254 |
| C | -13.1117 | 6.7507 | -0.8997 |
| C | -13.1194 | 8.1389 | -0.7339 |
| C | -4.4539 | 16.4807 | -8.4972 |
| C | 0.0522 | 4.4328 | -12.8959 |
| C | -13.0835 | 4.4530 | 0.1387 |
| C | 5.5673 | -3.2989 | -6.7879 |
| C | 6.5882 | -3.2782 | -5.8328 |
| C | 7.4704 | -2.1748 | -5.6969 |
| C | 7.3127 | -1.0937 | -6.5694 |
| C | 6.2922 | -1.1153 | -7.5282 |
| C | -6.5283 | -3.2118 | 5.6829 |
| C | -5.5219 | -3.2490 | 6.6520 |
| C | -5.4401 | -2.2351 | 7.6145 |
| C | -6.4352 | -1.2238 | 7.5995 |
| C | -7.4458 | -1.1903 | 6.6359 |
| C | -4.4023 | 16.4392 | 8.5142 |
| C | -4.3556 | -2.1488 | 8.6092 |
| C | 8.4382 | 16.4666 | -4.4191 |
| C | 8.5123 | -2.2033 | -4.6446 |
| C | 13.0807 | 6.7405 | 0.8704 |
| C | 13.0898 | 8.1276 | 0.7020 |
| C | 13.1631 | 8.7351 | -0.5781 |
| C | 13.2881 | 7.9063 | -1.6970 |
| C | 13.2977 | 6.5142 | -1.5296 |
| C | 13.0618 | 10.2055 | -0.6385 |
| C | 1.3785 | -1.9518 | -13.2536 |
| C | -0.8886 | -2.9260 | -12.5304 |
| C | -0.8083 | -0.8483 | -14.0489 |
| C | -0.1070 | -2.2668 | -13.7677 |
| C | 1.4070 | -2.4954 | -11.8362 |
| C | 0.1321 | -2.9335 | -11.4075 |
| C | -0.0574 | -3.3049 | -10.0801 |
| C | 1.0250 | -3.3179 | -9.1929 |
| N | 3.3810 | -3.0401 | -8.6985 |
| C | 2.3051 | -2.9360 | -9.6373 |
| C | 2.4913 | -2.5158 | -10.9712 |
| C | -2.0221 | -1.9567 | -12.2513 |
| C | -1.9665 | -0.8073 | -13.0711 |
| C | -2.9062 | 0.2082 | -12.9109 |
| C | -3.9238 | 0.0806 | -11.9597 |
| N | -5.0856 | -1.1591 | -10.2336 |
| C | -3.9899 | -1.0715 | -11.1592 |
| C | -3.0330 | -2.0995 | -11.3109 |
| C | 0.2439 | 0.1689 | -13.6497 |
| C | 1.4363 | -0.4360 | -13.1961 |
| C | 2.4750 | 0.3515 | -12.7060 |
| C | 2.3527 | 1.7454 | -12.6968 |
| N | 1.1350 | 3.7777 | -13.1768 |
| C | 1.1703 | 2.3467 | -13.1607 |
| C | 0.1057 | 1.5517 | -13.6362 |
| C | -8.5510 | 16.3309 | -4.3981 |
| O | 6.8205 | -4.2726 | -4.9012 |
| O | 6.0449 | -0.1050 | -8.4395 |
| C | 6.1547 | -5.5429 | -5.0924 |
| C | 6.9957 | 0.9832 | -8.5082 |
| O | -4.2103 | 18.1275 | 6.4401 |
| C | -3.9932 | 19.0536 | 5.3488 |
| O | -8.6935 | 14.8862 | 6.5193 |
| C | -8.9216 | 13.9793 | 7.6264 |
| O | -6.2841 | -0.3021 | 8.6193 |
| O | -6.6253 | -4.1041 | 4.6295 |
| C | -7.2106 | 0.8074 | 8.6654 |
| C | -5.9854 | -5.3956 | 4.7786 |
| O | 8.6946 | 14.8521 | -6.5711 |
| O | 4.2158 | 18.0993 | -6.5052 |
| C | 3.9991 | 19.0282 | -5.4159 |
| C | 8.9249 | 13.9440 | -7.6756 |
| O | -6.5823 | 14.8401 | -8.7303 |
| C | -7.6404 | 13.8672 | -8.9180 |
| O | -6.5125 | 18.0864 | -4.2538 |
| C | -5.4269 | 19.0199 | -4.0429 |
| O | 6.5158 | 18.1057 | 4.1921 |
| C | 5.4282 | 19.0377 | 3.9779 |
| O | 6.5867 | 14.8785 | 8.6836 |
| C | 7.6452 | 13.9078 | 8.8753 |
| O | -4.8030 | -3.7291 | -7.3461 |
| O | -9.1697 | -0.4038 | -6.8483 |
| C | -4.2156 | -4.4225 | -6.2235 |
| C | -9.6220 | 0.3704 | -7.9836 |
| O | 9.0953 | -0.3525 | 6.8572 |
| C | 9.5862 | 0.3612 | 8.0156 |
| O | 4.7392 | -3.6919 | 7.3638 |
| C | 4.1634 | -4.4019 | 6.2457 |
| O | 2.2435 | 5.7975 | 12.9626 |
| O | -2.2991 | 8.9577 | 12.9387 |
| C | -3.6146 | 8.3531 | 12.9140 |
| C | 3.5584 | 6.4069 | 12.9724 |
| O | 13.4595 | 5.5933 | -2.5458 |
| O | 13.0362 | 9.0521 | 1.7299 |
| C | 13.3544 | 6.0692 | -3.9084 |
| C | 12.9380 | 8.5452 | 3.0793 |
| O | -13.4692 | 5.6104 | 2.5207 |
| C | -13.3481 | 6.0884 | 3.8825 |
| O | -13.0730 | 9.0651 | -1.7609 |
| C | -12.9558 | 8.5611 | -3.1093 |
| O | -2.2881 | 5.7585 | -12.9664 |
| C | -3.6005 | 6.3721 | -12.9718 |
| O | 2.2672 | 8.8973 | -12.9395 |
| C | 3.5802 | 8.2868 | -12.9061 |
| C | 15.2142 | 15.2772 | 1.1926 |
| C | 14.0168 | 17.3495 | -2.6577 |
| C | 12.7697 | 18.9717 | 1.2042 |
| C | 14.8950 | 17.7180 | -0.0616 |
| H | 12.6605 | 14.8467 | -3.8426 |
| H | 12.7883 | 12.3609 | -3.7443 |
| H | 13.9779 | 12.5871 | 0.4146 |
| H | 9.6563 | 18.4760 | 0.7304 |
| H | 8.0484 | 18.3449 | -1.1588 |
| H | 11.0950 | 16.5280 | -3.6366 |
| H | 12.8603 | 13.3278 | 2.2862 |
| H | 11.0611 | 13.5701 | 3.9767 |
| H | 10.4643 | 17.6850 | 2.7622 |
| C | 15.0830 | -0.8402 | -1.4023 |
| C | 13.9572 | -2.4418 | 2.5758 |
| C | 12.4843 | -4.4244 | -1.1415 |
| C | 14.6672 | -3.1830 | 0.0094 |
| H | 12.2219 | 0.1286 | 3.4458 |
| H | 12.3732 | 2.5834 | 3.0985 |
| H | 13.8895 | 1.9249 | -0.9074 |
| H | 9.3825 | -3.8095 | -0.5656 |
| H | 7.9291 | -3.7131 | 1.4417 |
| H | 11.1692 | -1.9422 | 3.7046 |
| H | 12.8524 | 1.0311 | -2.7469 |
| H | 11.0035 | 0.7297 | -4.3754 |
| H | 10.1328 | -3.1592 | -2.6737 |
| C | -2.5610 | -2.4939 | 14.1102 |
| C | 1.3223 | -4.3039 | 12.8614 |
| C | 1.1446 | -0.6564 | 15.5121 |
| C | -0.0007 | -3.1173 | 14.9789 |
| H | 1.0063 | -3.5665 | 9.7191 |
| H | -0.9069 | -3.6239 | 8.1351 |
| H | -3.5433 | -2.1974 | 11.2667 |
| H | 2.7787 | 1.1448 | 13.5349 |
| H | 4.5954 | 0.9043 | 11.8543 |
| H | 3.0353 | -2.9798 | 10.7248 |
| H | -3.4201 | -0.0439 | 12.1821 |
| H | -3.1955 | 2.4291 | 12.2078 |
| H | 0.7218 | 2.0645 | 14.0075 |
| H | 3.9063 | -2.2023 | 8.7805 |
| H | 7.2098 | -0.2918 | 8.8868 |
| H | 6.4865 | -3.6100 | 5.2022 |
| H | 9.3117 | -1.0802 | 4.7606 |
| C | -1.2355 | 15.5105 | 15.2805 |
| C | 2.6257 | 17.2538 | 13.9956 |
| C | -1.1925 | 19.1103 | 12.6610 |
| C | 0.0615 | 17.9037 | 14.8058 |
| H | 3.5539 | 14.7059 | 12.3538 |
| H | 3.2593 | 12.2397 | 12.4106 |
| H | -0.7924 | 12.7467 | 13.8386 |
| H | -0.7026 | 18.5710 | 9.5785 |
| H | 1.2141 | 18.4060 | 8.0041 |
| H | 3.6312 | 16.6238 | 11.1214 |
| H | -2.7670 | 13.7080 | 13.2334 |
| H | -4.4051 | 13.9203 | 11.3680 |
| H | -2.6914 | 17.7450 | 10.2877 |
| H | -0.9126 | 10.7237 | 12.5836 |
| H | 2.1727 | 8.5674 | 12.9639 |
| H | -2.2303 | 6.1898 | 12.9345 |
| H | 8.2361 | 16.8699 | 3.4164 |
| H | 8.4667 | 14.8961 | 6.6495 |
| H | 4.6321 | 18.0793 | 6.2196 |
| H | 4.4851 | 15.6249 | 9.1016 |
| H | 0.8756 | 4.0368 | 12.6084 |
| H | 12.8046 | 3.9534 | -1.1245 |
| C | -15.1497 | -0.8130 | 1.3912 |
| C | -14.0287 | -2.4278 | -2.5784 |
| C | -12.5812 | -4.4258 | 1.1390 |
| C | -14.7496 | -3.1630 | -0.0135 |
| H | -12.2589 | 0.1205 | -3.4447 |
| H | -12.3985 | 2.5771 | -3.1071 |
| H | -13.9227 | 1.9444 | 0.8978 |
| H | -9.4746 | -3.8362 | 0.5760 |
| H | -8.0140 | -3.7512 | -1.4258 |
| H | -11.2387 | -1.9657 | -3.7049 |
| H | -12.9137 | 1.0278 | 2.7566 |
| H | -11.0690 | 0.7103 | 4.3859 |
| H | -10.2261 | -3.1821 | 2.6786 |
| H | -8.2929 | -2.8571 | 3.7361 |
| H | -3.9689 | -2.2361 | -8.7633 |
| H | 4.3173 | -1.1917 | -9.2416 |
| H | -7.2777 | -0.3363 | -8.8782 |
| H | -6.5557 | -3.6522 | -5.1883 |
| H | -9.3824 | -1.1220 | -4.7560 |
| C | 1.2305 | 15.4394 | -15.3282 |
| C | -2.6217 | 17.2083 | -14.0562 |
| C | 1.1948 | 19.0575 | -12.7236 |
| C | -0.0550 | 17.8433 | -14.8661 |
| H | -3.5649 | 14.6730 | -12.4096 |
| H | -3.2778 | 12.2039 | -12.4474 |
| H | 0.7817 | 12.6887 | -13.8601 |
| H | 0.7101 | 18.5181 | -9.6362 |
| H | -1.2078 | 18.3652 | -8.0629 |
| H | -3.6332 | 16.5906 | -11.1779 |
| H | 2.7609 | 13.6525 | -13.2745 |
| H | 4.4021 | 13.8675 | -11.4139 |
| H | 2.6978 | 17.7040 | -10.3519 |
| H | 0.8866 | 10.6720 | -12.5836 |
| H | 3.4590 | 16.8643 | -8.2630 |
| H | -2.2052 | 8.5261 | -12.9680 |
| H | 2.1861 | 6.1279 | -12.9447 |
| H | 6.7033 | 14.9325 | -8.4919 |
| H | 6.2129 | 18.0236 | -4.5875 |
| C | -14.0123 | 17.3710 | 2.5989 |
| C | -12.7652 | 18.9747 | -1.2735 |
| C | -15.2151 | 15.2857 | -1.2428 |
| C | -14.8926 | 17.7301 | 0.0007 |
| H | -9.6509 | 18.4804 | -0.7945 |
| H | -8.0443 | 18.3555 | 1.0964 |
| H | -11.0915 | 16.5466 | 3.5774 |
| H | -12.8670 | 13.3262 | -2.3278 |
| H | -11.0619 | 13.5582 | -4.0189 |
| H | -10.4596 | 17.6752 | -2.8238 |
| H | -12.6569 | 14.8709 | 3.7926 |
| H | -12.7904 | 12.3855 | 3.7063 |
| H | -13.9843 | 12.5990 | -0.4461 |
| H | -9.0873 | 15.5895 | 4.4028 |
| H | -12.6541 | 10.6590 | -0.3227 |
| H | -6.2093 | 18.0485 | 4.5255 |
| H | -6.6994 | 14.9708 | 8.4394 |
| H | -8.4656 | 14.8700 | -6.6974 |
| H | -4.6304 | 18.0557 | -6.2811 |
| H | -13.3990 | 8.3798 | 2.6521 |
| H | -13.0447 | 6.2892 | -1.8873 |
| H | -4.4814 | 15.5888 | -9.1538 |
| H | -0.9220 | 3.9884 | -12.6096 |
| H | -12.8311 | 3.9702 | 1.1027 |
| H | 4.8910 | -4.1515 | -6.8861 |
| H | 7.9989 | -0.2467 | -6.4824 |
| H | -4.7783 | -4.0492 | 6.6713 |
| H | -8.2007 | -0.3975 | 6.6245 |
| H | -3.4551 | 16.9008 | 8.2040 |
| H | -4.4370 | -1.2707 | 9.2836 |
| H | 9.0933 | 15.5651 | -4.4591 |
| H | 8.4164 | -3.0506 | -3.9362 |
| H | 13.0064 | 6.2774 | 1.8561 |
| H | 13.3910 | 8.3643 | -2.6805 |
| H | 12.6398 | 10.6503 | 0.2881 |
| C | 2.4543 | -2.5692 | -14.1673 |
| C | -1.4064 | -4.3457 | -12.8523 |
| C | -1.2570 | -0.6992 | -15.5183 |
| C | -0.1230 | -3.1660 | -14.9974 |
| H | -1.0475 | -3.5815 | -9.7165 |
| H | 0.8841 | -3.6208 | -8.1574 |
| H | 3.4823 | -2.2217 | -11.3283 |
| H | -2.8599 | 1.1050 | -13.5316 |
| H | -4.6661 | 0.8681 | -11.8369 |
| H | -3.0976 | -3.0130 | -10.7131 |
| H | 3.3835 | -0.1053 | -12.3175 |
| H | 3.1654 | 2.3692 | -12.3282 |
| H | -0.8082 | 2.0185 | -14.0006 |
| H | -8.2345 | 16.8550 | -3.4755 |
| H | 6.7123 | -6.1974 | -4.4086 |
| H | 5.1080 | -5.4634 | -4.7820 |
| H | 6.2518 | -5.8855 | -6.1258 |
| H | 6.5836 | 1.5810 | -9.3302 |
| H | 6.9897 | 1.5487 | -7.5698 |
| H | 7.9916 | 0.6027 | -8.7547 |
| H | -3.0530 | 19.5351 | 5.6482 |
| H | -3.8791 | 18.5139 | 4.4063 |
| H | -4.8087 | 19.7854 | 5.3049 |
| H | -9.8800 | 13.5241 | 7.3462 |
| H | -8.1262 | 13.2288 | 7.6742 |
| H | -9.0127 | 14.5369 | 8.5643 |
| H | -6.8442 | 1.3673 | 9.5367 |
| H | -7.1305 | 1.4061 | 7.7520 |
| H | -8.2274 | 0.4376 | 8.8288 |
| H | -6.2449 | -5.8812 | 3.8266 |
| H | -4.9019 | -5.2880 | 4.8764 |
| H | -6.4179 | -5.9300 | 5.6296 |
| H | 3.0581 | 19.5050 | -5.7174 |
| H | 3.8825 | 18.4903 | -4.4707 |
| H | 4.8136 | 19.7576 | -5.3744 |
| H | 9.8831 | 13.4904 | -7.3918 |
| H | 8.1304 | 13.1937 | -7.7194 |
| H | 9.0178 | 14.4975 | -8.6151 |
| H | -7.3551 | 13.4135 | -9.8760 |
| H | -7.6231 | 13.1279 | -8.1116 |
| H | -8.6075 | 14.3704 | -8.9926 |
| H | -5.7198 | 19.4918 | -3.0958 |
| H | -4.4793 | 18.4831 | -3.9363 |
| H | -5.3905 | 19.7535 | -4.8522 |
| H | 5.7217 | 19.5063 | 3.0300 |
| H | 4.4809 | 18.5009 | 3.8750 |
| H | 5.3943 | 19.7740 | 4.7868 |
| H | 7.3603 | 13.4574 | 9.8345 |
| H | 7.6297 | 13.1643 | 8.0726 |
| H | 8.6127 | 14.4110 | 8.9488 |
| H | -3.4639 | -5.0529 | -6.7180 |
| H | -3.7448 | -3.6957 | -5.5541 |
| H | -4.9633 | -5.0393 | -5.7171 |
| H | -10.6081 | 0.7144 | -7.6417 |
| H | -8.9467 | 1.2253 | -8.1490 |
| H | -9.7113 | -0.2582 | -8.8734 |
| H | 10.5687 | 0.7068 | 7.6658 |
| H | 8.9283 | 1.2177 | 8.2335 |
| H | 9.6856 | -0.3088 | 8.8738 |
| H | 3.4131 | -5.0344 | 6.7411 |
| H | 3.6886 | -3.6852 | 5.5684 |
| H | 4.9184 | -5.0170 | 5.7471 |
| H | -4.2644 | 9.2375 | 12.9214 |
| H | -3.7510 | 7.7721 | 11.9970 |
| H | -3.7695 | 7.7436 | 13.8098 |
| H | 4.2090 | 5.5237 | 12.9938 |
| H | 3.7139 | 6.9925 | 12.0621 |
| H | 3.6905 | 7.0126 | 13.8752 |
| H | 13.4377 | 5.1348 | -4.4791 |
| H | 12.3790 | 6.5394 | -4.0718 |
| H | 14.1858 | 6.7421 | -4.1412 |
| H | 12.9340 | 9.4755 | 3.6633 |
| H | 12.0012 | 7.9908 | 3.2059 |
| H | 13.8146 | 7.9337 | 3.3160 |
| H | -13.4209 | 5.1536 | 4.4546 |
| H | -12.3707 | 6.5606 | 4.0296 |
| H | -14.1773 | 6.7588 | 4.1241 |
| H | -12.9470 | 9.4951 | -3.6870 |
| H | -12.0152 | 8.0119 | -3.2253 |
| H | -13.8271 | 7.9468 | -3.3565 |
| H | -4.2568 | 5.4922 | -12.9896 |
| H | -3.7508 | 6.9572 | -12.0592 |
| H | -3.7316 | 6.9788 | -13.8724 |
| H | 4.2355 | 9.1676 | -12.9120 |
| H | 3.7090 | 7.7098 | -11.9850 |
| H | 3.7388 | 7.6741 | -13.7989 |
| H | -15.4155 | 16.1633 | -1.8941 |
| H | -15.9638 | 15.3226 | -0.4242 |
| C | -15.4641 | 14.0226 | -2.0761 |
| H | -0.5589 | 17.3629 | -15.7162 |
| H | 0.9655 | 18.0862 | -15.1924 |
| H | -0.5697 | 18.7964 | -14.6832 |
| H | 0.3435 | 19.6634 | -13.1004 |
| H | 1.9125 | 18.9633 | -13.5665 |
| C | 1.8767 | 19.8494 | -11.6017 |
| H | -2.4276 | 16.8822 | -15.0995 |
| H | -2.4931 | 18.3111 | -14.0401 |
| C | -4.0863 | 16.8941 | -13.7265 |
| H | 0.3819 | 15.5902 | -16.0288 |
| C | 1.9330 | 14.1314 | -15.7128 |
| H | 1.9376 | 16.2775 | -15.5046 |
| H | -13.2027 | 19.5870 | -0.4566 |
| H | -13.5698 | 18.8308 | -2.0247 |
| C | -11.6392 | 19.7916 | -1.9195 |
| H | -14.0547 | 18.4447 | 2.3156 |
| C | -13.5833 | 17.2897 | 4.0689 |
| H | -15.0479 | 16.9811 | 2.5147 |
| H | -14.7502 | 18.7215 | 0.4535 |
| H | -15.7217 | 17.2474 | 0.5350 |
| H | -15.2258 | 17.8938 | -1.0338 |
| H | 2.3541 | -3.6740 | -14.1204 |
| C | 3.9142 | -2.2003 | -13.8773 |
| H | 2.2347 | -2.2787 | -15.2174 |
| H | 0.3303 | -4.1463 | -14.7913 |
| H | 0.4337 | -2.7250 | -15.8367 |
| H | -1.1459 | -3.3535 | -15.3542 |
| H | -1.9548 | -1.5299 | -15.7553 |
| H | -0.3673 | -0.8376 | -16.1686 |
| C | -1.9387 | 0.6164 | -15.9152 |
| H | -0.5732 | -4.9348 | -13.2911 |
| H | -2.1799 | -4.2580 | -13.6435 |
| C | -1.9838 | -5.1571 | -11.6857 |
| H | -15.0649 | -2.1352 | -2.3078 |
| H | -13.9897 | -3.5355 | -2.5284 |
| C | -13.7888 | -2.0001 | -4.0319 |
| H | -15.6031 | -2.6723 | -0.5013 |
| H | -15.0668 | -3.4169 | 1.0072 |
| H | -14.5775 | -4.1110 | -0.5421 |
| H | -15.8810 | -0.8143 | 0.5552 |
| C | -15.4477 | 0.3900 | 2.2947 |
| H | -15.3439 | -1.7342 | 1.9813 |
| H | -13.0362 | -4.9891 | 0.2965 |
| H | -13.3664 | -4.3412 | 1.9205 |
| C | -11.4313 | -5.2732 | 1.6982 |
| H | 15.8181 | -0.8265 | -0.5701 |
| C | 15.3753 | 0.3458 | -2.3302 |
| H | 15.2747 | -1.7715 | -1.9765 |
| H | 15.5247 | -2.6979 | 0.4960 |
| H | 14.9786 | -3.4381 | -1.0125 |
| H | 14.4875 | -4.1285 | 0.5389 |
| H | 12.9432 | -4.9856 | -0.2986 |
| H | 13.2636 | -4.3463 | -1.9284 |
| C | 11.3257 | -5.2684 | -1.6840 |
| H | 14.9937 | -2.1430 | 2.3085 |
| H | 13.9214 | -3.5491 | 2.5176 |
| C | 13.7125 | -2.0269 | 4.0325 |
| H | 1.8282 | -1.4950 | 15.7606 |
| H | 0.2423 | -0.7832 | 16.1469 |
| C | 1.8349 | 0.6522 | 15.9157 |
| H | -2.4891 | -3.6001 | 14.0554 |
| C | -4.0076 | -2.0833 | 13.8076 |
| H | -2.3446 | -2.2150 | 15.1635 |
| H | 0.4825 | -4.8919 | 13.2905 |
| H | 2.0838 | -4.2142 | 13.6644 |
| C | 1.9191 | -5.1223 | 11.7093 |
| H | -0.4545 | -4.0949 | 14.7638 |
| H | -0.5736 | -2.6735 | 15.8062 |
| H | 1.0132 | -3.3078 | 15.3561 |
| H | 15.0525 | 16.9592 | -2.5726 |
| H | 14.0611 | 18.4245 | -2.3797 |
| C | 13.5880 | 17.2642 | -4.1281 |
| H | 15.7237 | 17.2309 | -0.5936 |
| H | 15.2287 | 17.8845 | 0.9722 |
| H | 14.7570 | 18.7078 | -0.5182 |
| H | 15.9636 | 15.3084 | 0.3743 |
| C | 15.4608 | 14.0177 | 2.0320 |
| H | 15.4167 | 16.1577 | 1.8396 |
| H | 13.2082 | 19.5783 | 0.3833 |
| H | 13.5739 | 18.8311 | 1.9570 |
| C | 11.6452 | 19.7934 | 1.8467 |
| H | 2.4296 | 16.9321 | 15.0404 |
| H | 2.4994 | 18.3566 | 13.9745 |
| C | 4.0878 | 16.9316 | 13.6675 |
| H | 0.5651 | 17.4258 | 15.6573 |
| H | -0.9580 | 18.1511 | 15.1297 |
| H | 0.5797 | 18.8532 | 14.6158 |
| H | -0.3862 | 15.6568 | 15.9805 |
| C | -1.9477 | 14.2089 | 15.6711 |
| H | -1.9383 | 16.3531 | 15.4517 |
| H | -0.3378 | 19.7168 | 13.0289 |
| H | -1.9054 | 19.0202 | 13.5080 |
| C | -1.8799 | 19.8948 | 11.5376 |
| H | -13.9487 | -0.9092 | -4.1412 |
| C | -14.7249 | -2.7461 | -4.9819 |
| H | -12.7340 | -2.1938 | -4.3253 |
| H | -15.7770 | -2.5227 | -4.7710 |
| H | -14.5982 | -3.8324 | -4.9076 |
| H | -14.5345 | -2.4666 | -6.0241 |
| H | -10.8641 | -4.7047 | 2.4665 |
| H | -10.7096 | -5.5159 | 0.8931 |
| C | -11.9579 | -6.5699 | 2.3128 |
| H | -12.6618 | -6.3761 | 3.1305 |
| H | -11.1405 | -7.1740 | 2.7221 |
| H | -12.4787 | -7.1863 | 1.5709 |
| H | -15.1384 | 1.3363 | 1.8002 |
| H | -14.8578 | 0.3190 | 3.2298 |
| C | -16.9350 | 0.4620 | 2.6376 |
| H | -17.5552 | 0.5490 | 1.7378 |
| H | -17.1528 | 1.3321 | 3.2677 |
| H | -17.2682 | -0.4282 | 3.1825 |
| H | 2.7727 | 13.9235 | -15.0177 |
| C | 2.4637 | 14.1936 | -17.1445 |
| H | 1.2327 | 13.2768 | -15.6186 |
| H | 3.1953 | 14.9998 | -17.2707 |
| H | 1.6582 | 14.3625 | -17.8681 |
| H | 2.9581 | 13.2558 | -17.4221 |
| H | -1.2744 | 1.4778 | -15.6978 |
| H | -2.8565 | 0.7726 | -15.3118 |
| C | -2.3040 | 0.6190 | -17.3990 |
| H | -1.4181 | 0.5038 | -18.0339 |
| H | -2.7882 | 1.5601 | -17.6837 |
| H | -2.9960 | -0.1934 | -17.6480 |
| C | -5.0306 | 17.6150 | -14.6877 |
| H | -4.3221 | 17.1936 | -12.6835 |
| H | -4.2688 | 15.8021 | -13.7843 |
| H | -4.9041 | 18.7026 | -14.6409 |
| H | -6.0776 | 17.3972 | -14.4478 |
| H | -4.8626 | 17.3083 | -15.7261 |
| H | -14.8161 | 14.0250 | -2.9751 |
| C | -16.9265 | 13.9361 | -2.5120 |
| H | -15.1941 | 13.1145 | -1.4957 |
| H | -17.2071 | 14.7818 | -3.1505 |
| H | -17.6074 | 13.9325 | -1.6534 |
| H | -17.1150 | 13.0194 | -3.0818 |
| C | -14.5981 | 17.9868 | 4.9739 |
| H | -12.5886 | 17.7595 | 4.2063 |
| H | -13.4737 | 16.2303 | 4.3834 |
| H | -14.6937 | 19.0510 | 4.7296 |
| H | -14.2979 | 17.9199 | 6.0259 |
| H | -15.5943 | 17.5387 | 4.8888 |
| C | -2.3877 | 21.2440 | 12.0449 |
| H | -2.7301 | 19.3140 | 11.1229 |
| H | -1.1768 | 20.0598 | 10.6970 |
| H | -3.1122 | 21.1248 | 12.8587 |
| H | -2.8863 | 21.8020 | 11.2444 |
| H | -1.5702 | 21.8693 | 12.4211 |
| H | -2.7911 | 14.0040 | 14.9792 |
| C | -2.4742 | 14.2785 | 17.1043 |
| H | -1.2540 | 13.3483 | 15.5772 |
| H | -3.1995 | 15.0900 | 17.2310 |
| H | -1.6654 | 14.4436 | 17.8252 |
| H | -2.9753 | 13.3451 | 17.3861 |
| H | -4.1389 | -0.9969 | 13.9827 |
| C | -4.9932 | -2.8574 | 14.6815 |
| H | -4.2439 | -2.2598 | 12.7352 |
| H | -4.8432 | -2.6409 | 15.7455 |
| H | -4.8912 | -3.9405 | 14.5497 |
| H | -6.0282 | -2.5948 | 14.4362 |
| H | 2.6801 | -4.5250 | 11.1621 |
| H | 1.1291 | -5.3735 | 10.9739 |
| C | 2.5566 | -6.4131 | 12.2214 |
| H | 3.3558 | -6.2126 | 12.9440 |
| H | 2.9981 | -6.9877 | 11.3994 |
| H | 1.8215 | -7.0590 | 12.7148 |
| H | 13.8185 | -0.9292 | 4.1440 |
| C | 14.6946 | -2.7254 | 4.9719 |
| H | 12.6718 | -2.2719 | 4.3352 |
| H | 15.7322 | -2.4561 | 4.7431 |
| H | 14.6159 | -3.8165 | 4.9048 |
| H | 14.5075 | -2.4492 | 6.0155 |
| H | 10.7601 | -4.7037 | -2.4549 |
| H | 10.6057 | -5.4956 | -0.8725 |
| C | 11.8409 | -6.5763 | -2.2834 |
| H | 12.5432 | -6.3980 | -3.1058 |
| H | 11.0178 | -7.1792 | -2.6818 |
| H | 12.3601 | -7.1867 | -1.5352 |
| H | -2.7352 | -4.5569 | -11.1283 |
| H | -1.1818 | -5.4056 | -10.9624 |
| C | -2.6308 | -6.4496 | -12.1820 |
| H | -3.4389 | -6.2515 | -12.8956 |
| H | -3.0625 | -7.0182 | -11.3506 |
| H | -1.9030 | -7.1003 | -12.6801 |
| C | 2.3907 | 21.1943 | -12.1146 |
| H | 2.7221 | 19.2694 | -11.1766 |
| H | 1.1675 | 20.0226 | -10.7677 |
| H | 3.1207 | 21.0677 | -12.9223 |
| H | 2.8841 | 21.7576 | -11.3144 |
| H | 1.5770 | 21.8188 | -12.5005 |
| H | 4.0683 | -1.1121 | -14.0201 |
| C | 4.8667 | -2.9683 | -14.7930 |
| H | 4.1631 | -2.4166 | -12.8158 |
| H | 4.6994 | -2.7214 | -15.8480 |
| H | 4.7482 | -4.0526 | -14.6863 |
| H | 5.9114 | -2.7295 | -14.5648 |
| H | 15.0573 | 1.2998 | -1.8557 |
| H | 14.7883 | 0.2498 | -3.2651 |
| C | 16.8623 | 0.4225 | -2.6732 |
| H | 17.4817 | 0.5289 | -1.7743 |
| H | 17.0740 | 1.2839 | -3.3177 |
| H | 17.2025 | -0.4739 | -3.2037 |
| H | 1.1831 | 1.5203 | 15.6872 |
| H | 2.7631 | 0.7973 | 15.3254 |
| C | 2.1785 | 0.6535 | 17.4046 |
| H | 1.2829 | 0.5476 | 18.0272 |
| H | 2.6673 | 1.5906 | 17.6949 |
| H | 2.8600 | -0.1644 | 17.6646 |
| C | 5.0348 | 17.6570 | 14.6222 |
| H | 4.3231 | 17.2219 | 12.6219 |
| H | 4.2655 | 15.8397 | 13.7342 |
| H | 4.9121 | 18.7444 | 14.5667 |
| H | 6.0809 | 17.4346 | 14.3839 |
| H | 4.8668 | 17.3597 | 15.6633 |
| C | -12.1736 | 21.0964 | -2.5084 |
| H | -11.1423 | 19.1998 | -2.7187 |
| H | -10.8554 | 20.0234 | -1.1708 |
| H | -12.9459 | 20.9143 | -3.2646 |
| H | -11.3727 | 21.6675 | -2.9917 |
| H | -12.6128 | 21.7387 | -1.7365 |
| C | 14.6035 | 17.9568 | -5.0360 |
| H | 12.5939 | 17.7350 | -4.2670 |
| H | 13.4764 | 16.2037 | -4.4385 |
| H | 14.6998 | 19.0218 | -4.7953 |
| H | 14.3039 | 17.8869 | -6.0879 |
| H | 15.5993 | 17.5079 | -4.9484 |
| H | 14.8114 | 14.0249 | 2.9296 |
| C | 16.9225 | 13.9317 | 2.4703 |
| H | 15.1904 | 13.1077 | 1.4547 |
| H | 17.2034 | 14.7792 | 3.1060 |
| H | 17.6041 | 13.9240 | 1.6122 |
| H | 17.1092 | 13.0170 | 3.0438 |
| C | 12.1816 | 21.1009 | 2.4278 |
| H | 11.1483 | 19.2073 | 2.6499 |
| H | 10.8609 | 20.0215 | 1.0975 |
| H | 12.9539 | 20.9230 | 3.1849 |
| H | 11.3816 | 21.6761 | 2.9086 |
| H | 12.6216 | 21.7383 | 1.6523 |
| C | 30.7850 | 4.5698 | 0.7956 |
| C | 30.0713 | 5.6085 | -1.4489 |
| C | 29.3043 | 6.6747 | 0.7646 |
| C | 30.5510 | 5.9677 | 0.0402 |
| C | 30.4601 | 3.5217 | -0.2536 |
| C | 30.0644 | 4.0926 | -1.4825 |
| C | 29.6787 | 3.2733 | -2.5409 |
| C | 29.6992 | 1.8826 | -2.3926 |
| N | 30.1340 | -0.1149 | -1.1130 |
| C | 30.0907 | 1.3155 | -1.1676 |
| C | 30.4729 | 2.1417 | -0.0892 |
| C | 28.6448 | 6.1246 | -1.5145 |
| C | 28.2193 | 6.6924 | -0.2941 |
| C | 26.9112 | 7.1500 | -0.1558 |
| C | 26.0225 | 7.0626 | -1.2318 |
| N | 25.4926 | 6.4850 | -3.5109 |
| C | 26.4478 | 6.4981 | -2.4469 |
| C | 27.7709 | 6.0299 | -2.5907 |
| C | 28.9221 | 5.7115 | 1.8742 |
| C | 29.7299 | 4.5540 | 1.8848 |
| C | 29.4823 | 3.5405 | 2.8076 |
| C | 28.4367 | 3.6706 | 3.7267 |
| N | 26.6068 | 4.8900 | 4.7099 |
| C | 27.6272 | 4.8202 | 3.7102 |
| C | 27.8780 | 5.8521 | 2.7807 |
| C | 30.8205 | -11.8806 | -0.7195 |
| C | 30.0907 | -12.9292 | 1.5148 |
| C | 29.3466 | -13.9873 | -0.7101 |
| C | 30.5848 | -13.2823 | 0.0278 |
| C | 30.5127 | -10.8388 | 0.3402 |
| C | 30.0974 | -11.4135 | 1.5596 |
| C | 29.7151 | -10.5920 | 2.6183 |
| C | 29.7449 | -9.2012 | 2.4748 |
| N | 30.1964 | -7.2031 | 1.2032 |
| C | 30.1572 | -8.6328 | 1.2570 |
| C | 30.5548 | -9.4581 | 0.1847 |
| C | 28.6601 | -13.4352 | 1.5619 |
| C | 28.2468 | -13.9973 | 0.3349 |
| C | 26.9331 | -14.4344 | 0.1771 |
| C | 26.0334 | -14.3475 | 1.2434 |
| N | 25.4861 | -13.7929 | 3.5238 |
| C | 26.4493 | -13.7968 | 2.4678 |
| C | 27.7737 | -13.3351 | 2.6273 |
| C | 28.9738 | -13.0216 | -1.8216 |
| C | 29.7569 | -11.8478 | -1.7996 |
| C | 29.4763 | -10.8074 | -2.6835 |
| C | 28.4404 | -10.9407 | -3.6132 |
| N | 26.6607 | -12.1927 | -4.6524 |
| C | 27.6726 | -12.1180 | -3.6443 |
| C | 27.9458 | -13.1701 | -2.7445 |
| C | 15.4216 | -12.9238 | 13.1114 |
| C | 17.6380 | -13.9801 | 12.3405 |
| C | 17.6642 | -11.8899 | 13.8405 |
| C | 16.9105 | -13.2860 | 13.5919 |
| C | 15.3621 | -13.4160 | 11.6759 |
| C | 16.5863 | -13.9731 | 11.2471 |
| C | 16.7343 | -14.3962 | 9.9278 |
| C | 15.6599 | -14.3005 | 9.0373 |
| N | 13.3756 | -13.7435 | 8.5101 |
| C | 14.4377 | -13.7546 | 9.4681 |
| C | 14.2892 | -13.3071 | 10.7986 |
| C | 18.7543 | -13.0168 | 11.9794 |
| C | 18.7456 | -11.8537 | 12.7776 |
| C | 19.6391 | -10.8195 | 12.5173 |
| C | 20.5748 | -10.9494 | 11.4817 |
| N | 21.6088 | -12.1881 | 9.6900 |
| C | 20.5955 | -12.1161 | 10.6964 |
| C | 19.6816 | -13.1592 | 10.9500 |
| C | 16.6103 | -10.8409 | 13.5385 |
| C | 15.3844 | -11.4076 | 13.1292 |
| C | 14.3258 | -10.5826 | 12.7563 |
| C | 14.4761 | -9.1926 | 12.7891 |
| N | 15.7652 | -7.2038 | 13.2293 |
| C | 15.7009 | -8.6312 | 13.1898 |
| C | 16.7717 | -9.4618 | 13.5811 |
| C | 21.3933 | -12.7613 | 8.5431 |
| C | 22.4658 | -12.7966 | 7.5303 |
| C | 23.5918 | -11.9668 | 7.6019 |
| C | 24.5500 | -12.0053 | 6.5839 |
| C | 24.4168 | -12.8699 | 5.4688 |
| C | 23.3033 | -13.7178 | 5.4121 |
| C | 22.3446 | -13.6787 | 6.4283 |
| C | 25.4332 | -12.8333 | 4.3974 |
| C | 16.2395 | 4.5347 | 14.0007 |
| C | 18.4308 | 5.5936 | 13.1656 |
| C | 16.1684 | 6.5714 | 12.4311 |
| C | 16.9521 | 5.9376 | 13.6823 |
| C | 17.2663 | 3.4953 | 13.5913 |
| C | 18.4803 | 4.0757 | 13.1650 |
| C | 19.5256 | 3.2654 | 12.7271 |
| C | 19.3804 | 1.8745 | 12.7297 |
| N | 18.1104 | -0.1292 | 13.1624 |
| C | 18.1656 | 1.2993 | 13.1393 |
| C | 17.0953 | 2.1166 | 13.5643 |
| C | 18.4382 | 6.0619 | 11.7194 |
| C | 17.1687 | 6.5039 | 11.2949 |
| C | 16.9541 | 6.8127 | 9.9498 |
| C | 18.0149 | 6.7472 | 9.0424 |
| N | 20.3458 | 6.3869 | 8.5120 |
| C | 19.2915 | 6.3563 | 9.4774 |
| C | 19.5051 | 6.0059 | 10.8316 |
| C | 15.0049 | 5.6239 | 12.2075 |
| C | 15.0473 | 4.5024 | 13.0651 |
| C | 14.0617 | 3.5227 | 12.9810 |
| C | 13.0288 | 3.6446 | 12.0446 |
| N | 11.8865 | 4.8312 | 10.2728 |
| C | 12.9882 | 4.7585 | 11.1902 |
| C | 13.9781 | 5.7628 | 11.2834 |
| C | 17.0214 | -0.7756 | 12.8826 |
| C | 17.0081 | -2.2519 | 12.9366 |
| C | 18.1850 | -3.0091 | 12.9538 |
| C | 18.1161 | -4.4061 | 12.9678 |
| C | 16.8719 | -5.0870 | 12.9652 |
| C | 15.6951 | -4.3296 | 12.9709 |
| C | 15.7645 | -2.9330 | 12.9564 |
| C | 25.4619 | 5.4575 | 4.4839 |
| C | 24.4341 | 5.4889 | 5.5458 |
| C | 24.5055 | 4.6665 | 6.6754 |
| C | 23.4773 | 4.6906 | 7.6239 |
| C | 22.3469 | 5.5328 | 7.4757 |
| C | 22.2883 | 6.3758 | 6.3598 |
| C | 23.3174 | 6.3526 | 5.4118 |
| C | 29.9445 | -5.0790 | 0.1070 |
| C | 21.2668 | 5.4741 | 8.4836 |
| C | 16.8548 | -6.5638 | 12.9304 |
| C | 29.9066 | -6.5574 | 0.1181 |
| C | 3.1057 | -11.8069 | 0.6080 |
| C | 3.7909 | -12.9904 | -1.5716 |
| C | 4.5382 | -13.9434 | 0.6976 |
| C | 3.3070 | -13.2530 | -0.0637 |
| C | 3.3684 | -10.8312 | -0.5244 |
| C | 3.7314 | -11.4830 | -1.7253 |
| C | 4.0120 | -10.7373 | -2.8651 |
| C | 3.9541 | -9.3403 | -2.8233 |
| N | 3.5487 | -7.2588 | -1.6543 |
| C | 3.6133 | -8.6909 | -1.6237 |
| C | 3.3004 | -9.4459 | -0.4717 |
| C | 5.2372 | -13.4509 | -1.5839 |
| C | 5.6442 | -13.9842 | -0.3421 |
| C | 6.9602 | -14.4039 | -0.1575 |
| C | 7.8732 | -14.3238 | -1.2138 |
| N | 8.4448 | -13.7811 | -3.4924 |
| C | 7.4687 | -13.7858 | -2.4477 |
| C | 6.1428 | -13.3378 | -2.6324 |
| C | 4.9253 | -12.9357 | 1.7667 |
| C | 4.1920 | -11.7331 | 1.6634 |
| C | 4.5225 | -10.6490 | 2.4731 |
| C | 5.5455 | -10.7686 | 3.4214 |
| N | 7.2363 | -12.0486 | 4.5819 |
| C | 6.2490 | -11.9792 | 3.5477 |
| C | 5.9329 | -13.0731 | 2.7141 |
| C | 8.3645 | -12.6665 | 4.4013 |
| C | 9.3713 | -12.7158 | 5.4827 |
| C | 12.5278 | -12.7648 | -8.5294 |
| C | 11.4642 | -12.7978 | -7.5044 |
| C | 21.3416 | -12.8357 | -8.4398 |
| C | 22.4177 | -12.8751 | -7.4277 |
| C | 10.3367 | -11.9712 | -7.5765 |
| C | 9.3833 | -12.0014 | -6.5532 |
| C | 9.5222 | -12.8603 | -5.4341 |
| C | 10.6372 | -13.7045 | -5.3743 |
| C | 11.5930 | -13.6717 | -6.3956 |
| C | 8.5035 | -12.8214 | -4.3653 |
| C | 17.7530 | 4.5616 | -13.8752 |
| C | 15.5305 | 5.6266 | -13.1375 |
| C | 17.7618 | 6.6770 | -12.4062 |
| C | 17.0165 | 5.9682 | -13.6402 |
| C | 16.7016 | 3.5249 | -13.5167 |
| C | 15.5003 | 4.1126 | -13.0624 |
| C | 14.4709 | 3.3077 | -12.5785 |
| C | 14.6070 | 1.9157 | -12.5901 |
| N | 15.8328 | -0.0982 | -13.1150 |
| C | 15.7926 | 1.3315 | -13.0689 |
| C | 16.8503 | 2.1444 | -13.5304 |
| C | 15.4641 | 6.2190 | -11.7421 |
| C | 16.6991 | 6.7658 | -11.3292 |
| C | 16.8345 | 7.2878 | -10.0456 |
| C | 15.7426 | 7.2849 | -9.1709 |
| N | 13.4313 | 6.8199 | -8.6471 |
| C | 14.5108 | 6.7517 | -9.5875 |
| C | 14.3692 | 6.2211 | -10.8876 |
| C | 18.8421 | 5.6884 | -12.0086 |
| C | 18.8464 | 4.5360 | -12.8236 |
| C | 19.7571 | 3.5109 | -12.5783 |
| C | 20.6717 | 3.6244 | -11.5269 |
| N | 21.6551 | 4.8295 | -9.6838 |
| C | 20.6574 | 4.7666 | -10.7078 |
| C | 19.7362 | 5.8079 | -10.9514 |
| C | 16.9206 | -0.7567 | -12.8608 |
| C | 21.4241 | 5.3928 | -8.5404 |
| C | 16.9242 | -2.2338 | -12.9240 |
| C | 22.4854 | 5.4370 | -7.5128 |
| C | 15.7439 | -2.9851 | -12.9345 |
| C | 15.8061 | -4.3830 | -12.9486 |
| C | 17.0457 | -5.0721 | -12.9559 |
| C | 18.2256 | -4.3202 | -12.9714 |
| C | 18.1633 | -2.9226 | -12.9551 |
| C | 23.6179 | 4.6166 | -7.5749 |
| C | 24.5712 | 4.6657 | -6.5525 |
| C | 24.4247 | 5.5339 | -5.4412 |
| C | 23.3046 | 6.3711 | -5.3914 |
| C | 22.3492 | 6.3211 | -6.4128 |
| C | 3.8722 | 5.5048 | 1.5330 |
| C | 4.7050 | 6.6440 | -0.6183 |
| C | 3.1683 | 4.5800 | -0.7618 |
| C | 3.4267 | 5.9383 | 0.0541 |
| C | 5.2790 | 6.0559 | 1.6740 |
| C | 5.7572 | 6.6290 | 0.4737 |
| C | 7.0686 | 7.0920 | 0.4046 |
| C | 7.8947 | 7.0338 | 1.5329 |
| N | 8.2853 | 6.5388 | 3.8679 |
| C | 7.4049 | 6.4989 | 2.7374 |
| C | 6.0839 | 6.0041 | 2.8034 |
| C | 5.0815 | 5.7171 | -1.7587 |
| C | 4.2329 | 4.5905 | -1.8435 |
| C | 4.4548 | 3.6259 | -2.8230 |
| C | 5.5055 | 3.7814 | -3.7341 |
| N | 7.3619 | 4.9938 | -4.6595 |
| C | 6.3468 | 4.9058 | -3.6546 |
| C | 6.1326 | 5.8772 | -2.6522 |
| C | 3.4791 | 3.4802 | 0.2385 |
| C | 3.9123 | 3.9892 | 1.4816 |
| C | 4.3497 | 3.1177 | 2.4768 |
| C | 4.3054 | 1.7353 | 2.2648 |
| N | 3.7545 | -0.1966 | 0.9234 |
| C | 3.8413 | 1.2275 | 1.0389 |
| C | 3.4339 | 2.1093 | 0.0149 |
| C | 9.3943 | 5.8331 | -5.6299 |
| C | 8.2351 | 5.6290 | 4.7904 |
| C | 3.9831 | -0.8036 | -0.2003 |
| C | 9.1476 | 5.6812 | 5.9518 |
| C | 3.8846 | -2.2767 | -0.2833 |
| C | 10.2257 | 6.5710 | 6.0280 |
| C | 11.0937 | 6.5042 | 7.1207 |
| C | 10.9611 | 5.5213 | 8.1337 |
| C | 9.8418 | 4.6829 | 8.1001 |
| C | 8.9436 | 4.7799 | 7.0309 |
| C | 9.4524 | 4.8645 | -6.6380 |
| C | 10.4647 | 4.9195 | -7.6019 |
| C | 11.4564 | 5.9325 | -7.5872 |
| C | 11.3757 | 6.9243 | -6.6023 |
| C | 10.3637 | 6.8703 | -5.6387 |
| C | 3.8649 | -3.0939 | 0.8503 |
| C | 3.8455 | -4.4830 | 0.6988 |
| C | 3.8574 | -5.1029 | -0.5772 |
| C | 3.8315 | -4.2875 | -1.7111 |
| C | 3.8401 | -2.8941 | -1.5618 |
| C | 12.5495 | 5.8747 | -8.5793 |
| C | 17.0546 | -6.5497 | -12.9183 |
| C | 3.9306 | -6.5768 | -0.6175 |
| C | 22.3647 | -13.7191 | -6.3139 |
| C | 23.3881 | -13.6815 | -5.3597 |
| C | 24.4914 | -12.8009 | -5.4903 |
| C | 24.5576 | -11.9759 | -6.6176 |
| C | 23.5348 | -12.0115 | -7.5699 |
| C | 10.4612 | -13.6175 | 5.3732 |
| C | 11.4731 | -13.6690 | 6.3377 |
| C | 11.4269 | -12.8165 | 7.4466 |
| C | 10.3222 | -11.9358 | 7.5704 |
| C | 9.3098 | -11.8837 | 6.6058 |
| C | 12.0460 | 5.4186 | 9.1279 |
| C | 12.4981 | -12.7874 | 8.4653 |
| C | 25.4369 | 5.5092 | -4.3652 |
| C | 25.5183 | -12.7661 | -4.4276 |
| C | 29.9529 | -4.3254 | 1.2851 |
| C | 29.9383 | -2.9289 | 1.2213 |
| C | 29.9157 | -2.2414 | -0.0193 |
| C | 29.9338 | -2.9956 | -1.1990 |
| C | 29.9447 | -4.3914 | -1.1345 |
| C | 29.8554 | -0.7659 | -0.0303 |
| C | 18.4648 | -12.9224 | -13.0984 |
| C | 16.2416 | -13.9886 | -12.3603 |
| C | 16.2294 | -11.8809 | -13.8348 |
| C | 16.9803 | -13.2795 | -13.5961 |
| C | 18.5103 | -13.4319 | -11.6691 |
| C | 17.2848 | -13.9998 | -11.2587 |
| C | 17.1268 | -14.4434 | -9.9475 |
| C | 18.1925 | -14.3566 | -9.0459 |
| N | 20.4723 | -13.7967 | -8.4952 |
| C | 19.4151 | -13.8003 | -9.4587 |
| C | 19.5745 | -13.3325 | -10.7807 |
| C | 15.1303 | -13.0234 | -11.9873 |
| C | 15.1494 | -11.8514 | -12.7713 |
| C | 14.2639 | -10.8108 | -12.4922 |
| C | 13.3358 | -10.9425 | -11.4547 |
| N | 12.3019 | -12.1923 | -9.6725 |
| C | 13.3079 | -12.1177 | -10.6853 |
| C | 14.2086 | -13.1709 | -10.9576 |
| C | 17.2840 | -10.8346 | -13.5243 |
| C | 18.5041 | -11.4062 | -13.1037 |
| C | 19.5594 | -10.5845 | -12.7149 |
| C | 19.4130 | -9.1940 | -12.7476 |
| N | 18.1406 | -7.1976 | -13.2064 |
| C | 18.1962 | -8.6269 | -13.1658 |
| C | 17.1268 | -9.4546 | -13.5680 |
| C | 8.3805 | 5.7903 | -4.5531 |
| O | 23.4400 | -14.4671 | -4.2234 |
| O | 23.4935 | -11.2408 | -8.7174 |
| C | 22.3402 | -15.3786 | -3.9882 |
| C | 24.5793 | -10.3078 | -8.9375 |
| O | 12.1294 | 7.3899 | 7.3731 |
| C | 12.6990 | 8.0915 | 6.2455 |
| O | 7.8040 | 4.0118 | 6.8846 |
| C | 7.3433 | 3.2681 | 8.0367 |
| O | 10.3600 | -11.1639 | 8.7156 |
| O | 10.4092 | -14.4118 | 4.2417 |
| C | 9.3040 | -10.1921 | 8.9095 |
| C | 11.5027 | -15.3359 | 4.0204 |
| O | 25.7236 | 3.9025 | -6.5071 |
| O | 21.2080 | 7.1000 | -6.4676 |
| C | 20.9614 | 8.0074 | -5.3657 |
| C | 25.9495 | 2.9648 | -7.5875 |
| O | 10.6173 | 4.0231 | -8.6444 |
| C | 9.6731 | 2.9307 | -8.7353 |
| O | 10.2366 | 7.7591 | -4.5867 |
| C | 10.9999 | 8.9897 | -4.6410 |
| O | 23.3639 | 7.1390 | 4.2757 |
| C | 22.2471 | 8.0271 | 4.0265 |
| O | 23.4467 | 3.9221 | 8.7734 |
| C | 24.5404 | 2.9988 | 8.9931 |
| O | 12.7307 | -14.4569 | -6.4405 |
| O | 8.2354 | -11.2299 | -6.5193 |
| C | 12.9555 | -15.3742 | -5.3428 |
| C | 8.0001 | -10.3333 | -7.6322 |
| O | 25.6970 | -11.2331 | 6.5487 |
| C | 25.9105 | -10.2989 | 7.6345 |
| O | 21.2083 | -14.4660 | 6.4755 |
| C | 20.9844 | -15.3856 | 5.3792 |
| O | 19.2094 | -5.2542 | 12.9844 |
| O | 14.6708 | -2.0870 | 12.9591 |
| C | 13.3550 | -2.6925 | 12.9771 |
| C | 20.5256 | -4.6488 | 12.9774 |
| O | 29.9603 | -5.2361 | -2.2297 |
| O | 29.9441 | -2.0855 | 2.3174 |
| C | 29.9439 | -4.6274 | -3.5431 |
| C | 29.9418 | -2.6957 | 3.6314 |
| O | 3.8110 | -5.3988 | 1.7359 |
| C | 3.8437 | -4.8792 | 3.0832 |
| O | 3.7979 | -1.9878 | -2.6009 |
| C | 3.9251 | -2.5058 | -3.9480 |
| O | 14.7081 | -5.2233 | -12.9588 |
| C | 13.3952 | -4.6133 | -12.9146 |
| O | 19.2613 | -2.0820 | -12.9672 |
| C | 20.5733 | -2.6940 | -12.9616 |
| C | 32.2205 | 4.4415 | 1.3474 |
| C | 30.9869 | 6.2323 | -2.5216 |
| C | 29.6602 | 8.0819 | 1.2878 |
| C | 31.7968 | 6.8439 | 0.0500 |
| H | 29.3597 | 3.7066 | -3.4888 |
| H | 29.4086 | 1.2343 | -3.2189 |
| H | 30.7862 | 1.6997 | 0.8555 |
| H | 26.5703 | 7.5760 | 0.7877 |
| H | 25.0015 | 7.4253 | -1.1321 |
| H | 28.1069 | 5.6126 | -3.5401 |
| H | 30.1029 | 2.6435 | 2.8218 |
| H | 28.2424 | 2.8831 | 4.4523 |
| H | 27.2687 | 6.7561 | 2.7841 |
| C | 32.2524 | -11.7542 | -1.2806 |
| C | 30.9910 | -13.5669 | 2.5929 |
| C | 29.7055 | -15.3929 | -1.2309 |
| C | 31.8347 | -14.1530 | 0.0257 |
| H | 29.3892 | -11.0294 | 3.5622 |
| H | 29.4477 | -8.5560 | 3.2996 |
| H | 30.9086 | -9.0182 | -0.7501 |
| H | 26.5925 | -14.8386 | -0.7739 |
| H | 25.0085 | -14.6987 | 1.1312 |
| H | 28.0975 | -12.9150 | 3.5792 |
| H | 30.0513 | -9.8834 | -2.6529 |
| H | 28.2198 | -10.1350 | -4.3114 |
| H | 27.3679 | -14.0952 | -2.7823 |
| C | 14.3483 | -13.5635 | 14.0164 |
| C | 18.1542 | -15.3937 | 12.6792 |
| C | 18.2238 | -11.7764 | 15.2747 |
| C | 16.9184 | -14.1712 | 14.8310 |
| H | 17.6840 | -14.7962 | 9.5763 |
| H | 15.7655 | -14.6403 | 8.0073 |
| H | 13.3397 | -12.8927 | 11.1354 |
| H | 19.6192 | -9.9035 | 13.1052 |
| H | 21.2817 | -10.1477 | 11.2759 |
| H | 19.7109 | -14.0772 | 10.3586 |
| H | 13.3755 | -11.0131 | 12.4383 |
| H | 13.6527 | -8.5414 | 12.4999 |
| H | 17.7108 | -9.0282 | 13.9256 |
| H | 20.4486 | -13.2348 | 8.2232 |
| H | 23.6972 | -11.3031 | 8.4626 |
| H | 23.2108 | -14.4001 | 4.5641 |
| H | 26.0876 | -11.9415 | 4.4283 |
| C | 15.8287 | 4.4130 | 15.4837 |
| C | 19.5198 | 6.2509 | 14.0335 |
| C | 15.6982 | 8.0165 | 12.7085 |
| C | 16.9521 | 6.8677 | 14.8874 |
| H | 20.4589 | 3.7029 | 12.3754 |
| H | 20.1998 | 1.2340 | 12.4065 |
| H | 16.1523 | 1.6662 | 13.8631 |
| H | 15.9637 | 7.0971 | 9.5990 |
| H | 17.8585 | 6.9989 | 7.9931 |
| H | 20.4957 | 5.7076 | 11.1742 |
| H | 14.0842 | 2.6624 | 13.6562 |
| H | 12.2556 | 2.8808 | 11.9740 |
| H | 13.9284 | 6.6558 | 10.6536 |
| H | 16.0523 | -0.3289 | 12.5889 |
| H | 19.1428 | -2.4836 | 12.9597 |
| H | 14.7370 | -4.8545 | 12.9907 |
| H | 25.1470 | 5.9410 | 3.5393 |
| H | 25.3752 | 4.0164 | 6.7921 |
| H | 21.4290 | 7.0419 | 6.2531 |
| H | 21.3334 | 4.5987 | 9.1606 |
| H | 17.8172 | -7.0189 | 12.6280 |
| H | 29.6074 | -7.0053 | -0.8500 |
| C | 1.6900 | -11.6366 | 1.2031 |
| C | 2.9154 | -13.7312 | -2.6035 |
| C | 4.1738 | -15.3288 | 1.2665 |
| C | 2.0381 | -14.0934 | -0.0046 |
| H | 4.2685 | -11.2413 | -3.8020 |
| H | 4.1720 | -8.7521 | -3.7120 |
| H | 2.9842 | -8.9458 | 0.4478 |
| H | 7.2937 | -14.7843 | 0.8069 |
| H | 8.8988 | -14.6646 | -1.0824 |
| H | 5.8404 | -12.9073 | -3.5846 |
| H | 3.9986 | -9.6998 | 2.3712 |
| H | 5.8039 | -9.9263 | 4.0619 |
| H | 6.4626 | -14.0228 | 2.8202 |
| H | 8.6798 | -13.1796 | 3.4723 |
| H | 13.4747 | -13.2436 | -8.2140 |
| H | 21.3681 | -11.9402 | -9.0904 |
| H | 10.2286 | -11.3118 | -8.4413 |
| H | 10.7343 | -14.3805 | -4.5216 |
| H | 7.8489 | -11.9293 | -4.3990 |
| C | 18.2879 | 4.4171 | -15.3139 |
| C | 14.4509 | 6.2005 | -14.0790 |
| C | 18.3349 | 8.0580 | -12.7913 |
| C | 17.0171 | 6.8403 | -14.8881 |
| H | 13.5595 | 3.7512 | -12.1800 |
| H | 13.8014 | 1.2799 | -12.2258 |
| H | 17.7672 | 1.6897 | -13.9043 |
| H | 17.7878 | 7.7026 | -9.7144 |
| H | 15.8414 | 7.6968 | -8.1667 |
| H | 13.4068 | 5.8333 | -11.2275 |
| H | 19.7654 | 2.6184 | -13.2044 |
| H | 21.3920 | 2.8306 | -11.3339 |
| H | 19.7370 | 6.7001 | -10.3267 |
| H | 17.8975 | -0.3179 | -12.5788 |
| H | 20.4726 | 5.8631 | -8.2232 |
| H | 14.7881 | -2.4553 | -12.9321 |
| H | 19.1809 | -4.8494 | -12.9985 |
| H | 23.7336 | 3.9509 | -8.4329 |
| H | 23.2023 | 7.0568 | -4.5472 |
| C | 2.9101 | 6.0415 | 2.6128 |
| C | 4.3832 | 8.0728 | -1.1110 |
| C | 1.7316 | 4.5024 | -1.3184 |
| C | 2.2060 | 6.8497 | 0.0623 |
| H | 7.4609 | 7.5038 | -0.5256 |
| H | 8.9190 | 7.4031 | 1.4819 |
| H | 5.6879 | 5.5998 | 3.7394 |
| H | 3.8103 | 2.7482 | -2.8906 |
| H | 5.6801 | 3.0318 | -4.5057 |
| H | 6.7768 | 6.7555 | -2.5886 |
| H | 4.7392 | 3.4998 | 3.4189 |
| H | 4.6351 | 1.0480 | 3.0431 |
| H | 3.0743 | 1.7163 | -0.9362 |
| H | 7.5568 | 4.7510 | 4.7999 |
| H | 4.2715 | -0.3206 | -1.1554 |
| H | 10.3636 | 7.3033 | 5.2308 |
| H | 9.7044 | 3.9599 | 8.9042 |
| H | 8.6964 | 4.0761 | -6.6461 |
| H | 12.1195 | 7.7247 | -6.6041 |
| H | 3.8688 | -2.6212 | 1.8352 |
| H | 3.8001 | -4.7563 | -2.6953 |
| H | 12.5344 | 4.9583 | -9.2049 |
| H | 16.0863 | -6.9972 | -12.6198 |
| H | 4.3346 | -7.0151 | 0.3197 |
| H | 21.5153 | -14.3991 | -6.2146 |
| H | 25.4209 | -11.3160 | -6.7319 |
| H | 12.3110 | -14.3650 | 6.2500 |
| H | 8.4615 | -11.2027 | 6.7055 |
| H | 12.9910 | 5.8860 | 8.7749 |
| H | 12.4651 | -11.9007 | 9.1272 |
| H | 26.0841 | 4.6120 | -4.3756 |
| H | 25.2027 | -13.2462 | -3.4809 |
| H | 29.9742 | -4.8543 | 2.2416 |
| H | 29.9377 | -2.4679 | -2.1543 |
| H | 29.5524 | -0.3225 | 0.9405 |
| C | 19.5470 | -13.5549 | -13.9975 |
| C | 15.7208 | -15.3952 | -12.7218 |
| C | 15.6709 | -11.7568 | -15.2676 |
| C | 16.9847 | -14.1509 | -14.8446 |
| H | 16.1755 | -14.8534 | -9.6111 |
| H | 18.0800 | -14.7125 | -8.0228 |
| H | 20.5262 | -12.9107 | -11.1017 |
| H | 14.2937 | -9.8881 | -13.0699 |
| H | 12.6365 | -10.1361 | -11.2351 |
| H | 14.1728 | -14.0936 | -10.3774 |
| H | 20.5042 | -11.0179 | -12.3852 |
| H | 20.2346 | -8.5459 | -12.4463 |
| H | 16.1928 | -9.0176 | -13.9254 |
| H | 8.5961 | 6.4722 | -3.7080 |
| H | 22.6434 | -15.8529 | -3.0466 |
| H | 21.4031 | -14.8278 | -3.8696 |
| H | 22.2795 | -16.1168 | -4.7952 |
| H | 24.2884 | -9.8500 | -9.8911 |
| H | 24.6103 | -9.5640 | -8.1357 |
| H | 25.5283 | -10.8446 | -9.0320 |
| H | 13.4593 | 8.7203 | 6.7300 |
| H | 13.1578 | 7.3690 | 5.5639 |
| H | 11.9416 | 8.7106 | 5.7547 |
| H | 6.3657 | 2.9034 | 7.6919 |
| H | 8.0267 | 2.4266 | 8.2352 |
| H | 7.2381 | 3.9240 | 8.9062 |
| H | 9.5945 | -9.7344 | 9.8635 |
| H | 9.3128 | -9.4538 | 8.1023 |
| H | 8.3363 | -10.6958 | 8.9933 |
| H | 11.2025 | -15.8114 | 3.0774 |
| H | 12.4448 | -14.7918 | 3.9063 |
| H | 11.5482 | -16.0694 | 4.8322 |
| H | 20.0150 | 8.4718 | -5.6705 |
| H | 20.8435 | 7.4502 | -4.4313 |
| H | 21.7613 | 8.7513 | -5.3017 |
| H | 26.9062 | 2.5140 | -7.2940 |
| H | 25.1529 | 2.2158 | -7.6163 |
| H | 26.0416 | 3.4968 | -8.5396 |
| H | 10.0326 | 2.4032 | -9.6294 |
| H | 9.7452 | 2.2944 | -7.8475 |
| H | 8.6625 | 3.3209 | -8.8863 |
| H | 10.6512 | 9.5047 | -3.7345 |
| H | 12.0719 | 8.7826 | -4.5756 |
| H | 10.7492 | 9.5559 | -5.5403 |
| H | 22.5478 | 8.5028 | 3.0844 |
| H | 21.3244 | 7.4534 | 3.8996 |
| H | 22.1625 | 8.7649 | 4.8300 |
| H | 24.2539 | 2.5385 | 9.9472 |
| H | 24.5761 | 2.2549 | 8.1927 |
| H | 25.4848 | 3.5436 | 9.0871 |
| H | 13.8984 | -15.8495 | -5.6422 |
| H | 13.0717 | -14.8250 | -4.4037 |
| H | 12.1461 | -16.1085 | -5.2898 |
| H | 7.0407 | -9.8808 | -7.3527 |
| H | 8.7920 | -9.5807 | -7.6892 |
| H | 7.9093 | -10.8999 | -8.5643 |
| H | 26.8658 | -9.8408 | 7.3482 |
| H | 25.1091 | -9.5548 | 7.6598 |
| H | 26.0003 | -10.8350 | 8.5840 |
| H | 20.0404 | -15.8603 | 5.6768 |
| H | 20.8698 | -14.8369 | 4.4396 |
| H | 21.7947 | -16.1196 | 5.3267 |
| H | 12.7048 | -1.8086 | 12.9832 |
| H | 13.1985 | -3.2908 | 12.0746 |
| H | 13.2206 | -3.2861 | 13.8871 |
| H | 21.1735 | -5.5341 | 12.9998 |
| H | 20.6751 | -4.0706 | 12.0619 |
| H | 20.6665 | -4.0372 | 13.8745 |
| H | 29.9619 | -5.5086 | -4.1973 |
| H | 29.0267 | -4.0496 | -3.6869 |
| H | 30.8391 | -4.0138 | -3.6901 |
| H | 29.9496 | -1.8141 | 4.2857 |
| H | 29.0318 | -3.2852 | 3.7748 |
| H | 30.8455 | -3.2967 | 3.7727 |
| H | 3.7677 | -5.8016 | 3.6736 |
| H | 4.7946 | -4.3649 | 3.2597 |
| H | 2.9818 | -4.2263 | 3.2580 |
| H | 3.9796 | -1.5797 | -4.5367 |
| H | 4.8479 | -3.0884 | -4.0451 |
| H | 3.0366 | -3.0857 | -4.2115 |
| H | 12.7405 | -5.4945 | -12.9270 |
| H | 13.2702 | -4.0441 | -11.9882 |
| H | 13.2360 | -3.9919 | -13.8015 |
| H | 21.2298 | -1.8145 | -12.9691 |
| H | 20.7160 | -3.2811 | -12.0489 |
| H | 20.7162 | -3.2985 | -13.8626 |
| H | 1.5658 | 5.3809 | -1.9766 |
| H | 1.0258 | 4.6046 | -0.4657 |
| C | 1.3431 | 3.2432 | -2.1034 |
| H | 16.4932 | 6.3563 | -15.7241 |
| H | 18.0369 | 7.0608 | -15.2326 |
| H | 16.5202 | 7.8046 | -14.7116 |
| H | 17.4984 | 8.6910 | -13.1566 |
| H | 19.0279 | 7.9196 | -13.6486 |
| C | 19.0795 | 8.8402 | -11.7028 |
| H | 14.6917 | 5.8939 | -15.1193 |
| H | 14.5248 | 7.3080 | -14.0526 |
| C | 12.9962 | 5.8030 | -13.8003 |
| H | 17.4343 | 4.5339 | -16.0153 |
| C | 19.0188 | 3.1157 | -15.6658 |
| H | 18.9776 | 5.2646 | -15.5140 |
| H | 3.9866 | 8.6568 | -0.2536 |
| H | 3.5598 | 8.0012 | -1.8525 |
| C | 5.5264 | 8.8800 | -1.7391 |
| H | 2.9713 | 7.1504 | 2.6072 |
| C | 3.1253 | 5.5481 | 4.0488 |
| H | 1.8698 | 5.7822 | 2.3201 |
| H | 2.4007 | 7.7820 | 0.6098 |
| H | 1.3399 | 6.3694 | 0.5390 |
| H | 1.9012 | 7.1297 | -0.9555 |
| H | 19.3900 | -14.6546 | -14.0087 |
| C | 21.0119 | -13.2879 | -13.6307 |
| H | 19.3819 | -13.2022 | -15.0378 |
| H | 17.4835 | -15.1139 | -14.6681 |
| H | 17.5066 | -13.6646 | -15.6798 |
| H | 15.9652 | -14.3746 | -15.1891 |
| H | 15.0441 | -12.6487 | -15.4801 |
| H | 16.5268 | -11.7960 | -15.9743 |
| C | 14.8334 | -10.5129 | -15.5892 |
| H | 16.5646 | -15.9868 | -13.1370 |
| H | 14.9773 | -15.2872 | -13.5394 |
| C | 15.0855 | -16.2169 | -11.5926 |
| H | 1.8850 | -13.3233 | -2.5344 |
| H | 2.8532 | -14.8005 | -2.3102 |
| C | 3.3622 | -13.6746 | -4.0696 |
| H | 1.2091 | -13.6151 | -0.5431 |
| H | 1.7037 | -14.2549 | 1.0293 |
| H | 2.1863 | -15.0857 | -0.4538 |
| H | 0.9556 | -11.6870 | 0.3692 |
| C | 1.4169 | -10.3615 | 2.0110 |
| H | 1.4793 | -12.5038 | 1.8642 |
| H | 3.7185 | -15.9359 | 0.4554 |
| H | 3.3855 | -15.1887 | 2.0358 |
| C | 5.3124 | -16.1516 | 1.8829 |
| H | 32.9608 | -11.7949 | -0.4265 |
| C | 32.5723 | -10.5077 | -2.1152 |
| H | 32.4642 | -12.6456 | -1.9092 |
| H | 32.6694 | -13.6691 | 0.5487 |
| H | 32.1748 | -14.3742 | -0.9951 |
| H | 31.6588 | -15.1162 | 0.5208 |
| H | 30.1246 | -15.9851 | -0.3890 |
| H | 30.5182 | -15.2888 | -1.9790 |
| C | 28.5736 | -16.2123 | -1.8597 |
| H | 32.0311 | -13.2124 | 2.4271 |
| H | 31.0025 | -14.6648 | 2.4321 |
| C | 30.6271 | -13.3039 | 4.0594 |
| H | 18.8583 | -12.6661 | 15.4749 |
| H | 17.3680 | -11.8317 | 15.9799 |
| C | 19.0481 | -10.5271 | 15.6108 |
| H | 14.5041 | -14.6631 | 14.0159 |
| C | 12.8801 | -13.2942 | 13.6634 |
| H | 14.5218 | -13.2178 | 15.0567 |
| H | 17.3119 | -15.9852 | 13.0970 |
| H | 18.9063 | -15.2986 | 13.4906 |
| C | 18.7754 | -16.2074 | 11.5372 |
| H | 16.4179 | -15.1320 | 14.6488 |
| H | 16.4037 | -13.6958 | 15.6769 |
| H | 17.9412 | -14.3979 | 15.1628 |
| H | 32.0274 | 5.8930 | -2.3326 |
| H | 30.9836 | 7.3345 | -2.3809 |
| C | 30.6537 | 5.9396 | -3.9898 |
| H | 32.6438 | 6.3545 | -0.4512 |
| H | 32.1191 | 7.0810 | 1.0726 |
| H | 31.6267 | 7.8000 | -0.4641 |
| H | 32.9304 | 4.5708 | 0.5029 |
| C | 32.5829 | 3.1412 | 2.0751 |
| H | 32.3998 | 5.2881 | 2.0435 |
| H | 30.0479 | 8.6813 | 0.4366 |
| H | 30.4971 | 7.9799 | 2.0107 |
| C | 28.5417 | 8.8863 | 1.9616 |
| H | 19.3390 | 5.9729 | 15.0941 |
| H | 19.3953 | 7.3524 | 13.9707 |
| C | 20.9774 | 5.9070 | 13.7047 |
| H | 17.4959 | 6.4339 | 15.7384 |
| H | 15.9320 | 7.0845 | 15.2308 |
| H | 17.4276 | 7.8318 | 14.6587 |
| H | 16.7095 | 4.6537 | 16.1157 |
| C | 15.2817 | 3.0561 | 15.9464 |
| H | 15.0645 | 5.1899 | 15.6932 |
| H | 16.5557 | 8.5946 | 13.1143 |
| H | 14.9329 | 7.9824 | 13.5113 |
| C | 15.1292 | 8.8017 | 11.5204 |
| H | 3.5501 | -12.6244 | -4.3800 |
| C | 2.3100 | -14.2965 | -4.9867 |
| H | 4.3232 | -14.2130 | -4.1965 |
| H | 1.3468 | -13.7791 | -4.9114 |
| H | 2.1371 | -15.3515 | -4.7452 |
| H | 2.6243 | -14.2498 | -6.0355 |
| H | 5.8567 | -15.5506 | 2.6437 |
| H | 6.0551 | -16.4171 | 1.1046 |
| C | 4.7815 | -17.4303 | 2.5292 |
| H | 4.0515 | -17.2144 | 3.3178 |
| H | 5.5937 | -18.0065 | 2.9870 |
| H | 4.2923 | -18.0811 | 1.7959 |
| H | 1.7373 | -9.4625 | 1.4421 |
| H | 2.0130 | -10.3689 | 2.9451 |
| C | -0.0655 | -10.2399 | 2.3585 |
| H | -0.6987 | -10.2028 | 1.4617 |
| H | -0.2672 | -9.3203 | 2.9212 |
| H | -0.4147 | -11.0785 | 2.9703 |
| H | 19.8814 | 2.9614 | -14.9857 |
| C | 19.5110 | 3.1358 | -17.1125 |
| H | 18.3479 | 2.2444 | -15.5163 |
| H | 20.2206 | 3.9532 | -17.2863 |
| H | 18.6831 | 3.2604 | -17.8217 |
| H | 20.0215 | 2.1989 | -17.3664 |
| H | 15.3908 | -9.5900 | -15.3230 |
| H | 13.9088 | -10.5055 | -14.9788 |
| C | 14.4593 | -10.4733 | -17.0704 |
| H | 15.3479 | -10.4537 | -17.7118 |
| H | 13.8689 | -9.5801 | -17.3042 |
| H | 13.8629 | -11.3455 | -17.3609 |
| C | 12.0360 | 6.5721 | -14.7067 |
| H | 12.7372 | 5.9956 | -12.7371 |
| H | 12.8602 | 4.7154 | -13.9636 |
| H | 12.1296 | 7.6557 | -14.5715 |
| H | 10.9949 | 6.3044 | -14.4950 |
| H | 12.2202 | 6.3563 | -15.7654 |
| H | 1.9953 | 3.1260 | -2.9926 |
| C | -0.1176 | 3.3000 | -2.5464 |
| H | 1.5005 | 2.3356 | -1.4834 |
| H | -0.3135 | 4.1516 | -3.2070 |
| H | -0.8043 | 3.3760 | -1.6928 |
| H | -0.3988 | 2.3917 | -3.0934 |
| C | 2.2415 | 6.3169 | 5.0300 |
| H | 4.1911 | 5.6629 | 4.3440 |
| H | 2.8966 | 4.4660 | 4.1175 |
| H | 2.4340 | 7.3953 | 4.9919 |
| H | 2.4202 | 5.9905 | 6.0607 |
| H | 1.1763 | 6.1660 | 4.8199 |
| C | 14.5230 | 10.1270 | 11.9798 |
| H | 14.3551 | 8.2031 | 10.9931 |
| H | 15.9291 | 9.0028 | 10.7801 |
| H | 13.7213 | 9.9753 | 12.7119 |
| H | 14.0927 | 10.6768 | 11.1353 |
| H | 15.2743 | 10.7753 | 12.4451 |
| H | 14.4793 | 2.7035 | 15.2627 |
| C | 14.7325 | 3.1430 | 17.3698 |
| H | 16.0812 | 2.2887 | 15.9090 |
| H | 13.9212 | 3.8761 | 17.4469 |
| H | 15.5107 | 3.4365 | 18.0832 |
| H | 14.3328 | 2.1765 | 17.6994 |
| H | 12.6811 | -12.2033 | 13.6402 |
| C | 11.9446 | -13.9583 | 14.6729 |
| H | 12.6519 | -13.6727 | 12.6451 |
| H | 12.1043 | -13.5730 | 15.6867 |
| H | 12.0898 | -15.0440 | 14.7070 |
| H | 10.8950 | -13.7777 | 14.4154 |
| H | 19.5676 | -15.6193 | 11.0264 |
| H | 18.0099 | -16.4331 | 10.7687 |
| C | 19.3704 | -17.5155 | 12.0577 |
| H | 20.1532 | -17.3362 | 12.8037 |
| H | 19.8227 | -18.0939 | 11.2446 |
| H | 18.6084 | -18.1483 | 12.5264 |
| H | 30.5981 | -12.2136 | 4.2622 |
| C | 31.6319 | -13.9658 | 5.0018 |
| H | 29.6102 | -13.6893 | 4.2803 |
| H | 32.6459 | -13.5769 | 4.8510 |
| H | 31.6688 | -15.0517 | 4.8557 |
| H | 31.3631 | -13.7854 | 6.0493 |
| H | 28.0650 | -15.6412 | -2.6659 |
| H | 27.7949 | -16.4254 | -1.1031 |
| C | 29.1009 | -17.5268 | -2.4320 |
| H | 29.8510 | -17.3613 | -3.2137 |
| H | 28.2945 | -18.1093 | -2.8907 |
| H | 29.5606 | -18.1569 | -1.6631 |
| H | 14.2922 | -15.6268 | -11.0879 |
| H | 15.8459 | -16.4364 | -10.8181 |
| C | 14.5006 | -17.5296 | -12.1196 |
| H | 13.7318 | -17.3555 | -12.8792 |
| H | 14.0438 | -18.1100 | -11.3146 |
| H | 15.2764 | -18.1554 | -12.5764 |
| C | 19.5750 | 10.1843 | -12.2361 |
| H | 19.9427 | 8.2531 | -11.3268 |
| H | 18.4184 | 9.0135 | -10.8299 |
| H | 20.2604 | 10.0562 | -13.0820 |
| H | 20.1155 | 10.7403 | -11.4596 |
| H | 18.7463 | 10.8154 | -12.5764 |
| H | 21.2127 | -12.1975 | -13.6107 |
| C | 21.9580 | -13.9592 | -14.6257 |
| H | 21.2269 | -13.6623 | -12.6084 |
| H | 21.8126 | -13.5789 | -15.6432 |
| H | 21.8102 | -15.0449 | -14.6551 |
| H | 23.0050 | -13.7793 | -14.3556 |
| H | 32.3034 | -9.5863 | -1.5562 |
| H | 31.9634 | -10.5012 | -3.0410 |
| C | 34.0540 | -10.4632 | -2.4870 |
| H | 34.6948 | -10.4427 | -1.5979 |
| H | 34.2855 | -9.5688 | -3.0761 |
| H | 34.3478 | -11.3336 | -3.0844 |
| H | 18.4786 | -9.6082 | 15.3575 |
| H | 19.9704 | -10.5009 | 14.9973 |
| C | 19.4255 | -10.4943 | 17.0915 |
| H | 18.5382 | -10.4858 | 17.7352 |
| H | 20.0127 | -9.6010 | 17.3312 |
| H | 20.0294 | -11.3653 | 17.3700 |
| C | 21.9400 | 6.6847 | 14.6019 |
| H | 21.2039 | 6.1314 | 12.6403 |
| H | 21.1472 | 4.8210 | 13.8369 |
| H | 21.8092 | 7.7711 | 14.4910 |
| H | 22.9816 | 6.4531 | 14.3465 |
| H | 21.7975 | 6.4365 | 15.6621 |
| C | 5.0245 | 10.2291 | -2.2532 |
| H | 5.9858 | 8.3173 | -2.5779 |
| H | 6.3331 | 9.0460 | -0.9971 |
| H | 4.2454 | 10.1076 | -3.0150 |
| H | 5.8369 | 10.8063 | -2.7063 |
| H | 4.6018 | 10.8382 | -1.4456 |
| C | 31.6262 | 6.6556 | -4.9265 |
| H | 29.6157 | 6.2577 | -4.2221 |
| H | 30.6937 | 4.8487 | -4.1830 |
| H | 31.5939 | 7.7429 | -4.7913 |
| H | 31.3842 | 6.4513 | -5.9763 |
| H | 32.6603 | 6.3336 | -4.7598 |
| H | 31.8945 | 2.9709 | 2.9280 |
| C | 34.0231 | 3.1822 | 2.5851 |
| H | 32.4588 | 2.2716 | 1.3974 |
| H | 34.1781 | 4.0007 | 3.2969 |
| H | 34.7389 | 3.3176 | 1.7660 |
| H | 34.2860 | 2.2494 | 3.0962 |
| C | 29.0611 | 10.2343 | 2.4598 |
| H | 28.1162 | 8.3183 | 2.8142 |
| H | 27.7068 | 9.0548 | 1.2523 |
| H | 29.8714 | 10.1131 | 3.1871 |
| H | 28.2652 | 10.8072 | 2.9481 |
| H | 29.4498 | 10.8457 | 1.6383 |
| C | -25.5285 | -12.7459 | 4.4913 |
| H | -25.2113 | -13.2262 | 3.5453 |
| C | -24.5027 | -12.7794 | 5.5524 |
| C | -23.3964 | -13.6580 | 5.4280 |
| O | -23.4507 | -14.4472 | 4.2928 |
| C | -22.3490 | -15.3589 | 4.0599 |
| H | -22.2823 | -16.0916 | 4.8679 |
| H | -22.6489 | -15.8372 | 3.1181 |
| H | -21.4127 | -14.8054 | 3.9359 |
| C | -22.3741 | -13.6899 | 6.3808 |
| H | -21.5222 | -14.3666 | 6.2869 |
| C | -22.4299 | -12.8435 | 7.4945 |
| C | -21.3534 | -12.8044 | 8.5052 |
| N | -20.4797 | -13.7605 | 8.5578 |
| C | -19.4220 | -13.7595 | 9.5218 |
| C | -18.1975 | -14.3107 | 9.1077 |
| H | -18.0841 | -14.6677 | 8.0854 |
| C | -17.1308 | -14.3937 | 10.0096 |
| H | -16.1772 | -14.7996 | 9.6724 |
| C | -17.2917 | -13.9514 | 11.3204 |
| C | -16.2486 | -13.9390 | 12.4219 |
| C | -15.7278 | -15.3449 | 12.7839 |
| H | -16.5706 | -15.9349 | 13.2026 |
| C | -15.0943 | -16.1694 | 11.6563 |
| C | -14.5142 | -17.4775 | 12.1927 |
| H | -15.2863 | -18.1041 | 12.6536 |
| H | -14.0543 | -18.0632 | 11.3887 |
| H | -13.7407 | -17.2988 | 12.9484 |
| H | -15.8498 | -16.3968 | 10.8781 |
| H | -14.2925 | -15.5878 | 11.1535 |
| H | -14.9825 | -15.2356 | 13.5998 |
| C | -16.9883 | -13.2296 | 13.6571 |
| C | -16.9901 | -14.1004 | 14.9073 |
| H | -17.4888 | -15.0637 | 14.7309 |
| H | -15.9720 | -14.3240 | 15.2631 |
| H | -17.5138 | -13.6144 | 15.7411 |
| C | -18.4737 | -12.8743 | 13.1582 |
| C | -19.5550 | -13.5067 | 14.0595 |
| H | -19.3993 | -14.6048 | 14.0699 |
| C | -21.0202 | -13.2373 | 13.6932 |
| C | -21.9656 | -13.9095 | 14.6884 |
| H | -21.8188 | -14.9957 | 14.7166 |
| H | -23.0129 | -13.7307 | 14.4193 |
| H | -21.8186 | -13.5302 | 15.7066 |
| H | -21.2348 | -13.6097 | 12.6701 |
| H | -21.2204 | -12.1473 | 13.6733 |
| H | -19.3908 | -13.1530 | 15.0982 |
| C | -18.5132 | -11.3579 | 13.1613 |
| C | -17.2941 | -10.7844 | 13.5801 |
| C | -17.1356 | -9.4039 | 13.6183 |
| H | -16.2028 | -8.9649 | 13.9762 |
| C | -18.2063 | -8.5778 | 13.2142 |
| C | -19.4221 | -9.1464 | 12.7979 |
| H | -20.2444 | -8.4991 | 12.4957 |
| N | -18.1498 | -7.1495 | 13.2520 |
| C | -17.0610 | -6.5041 | 12.9605 |
| H | -16.0950 | -6.9542 | 12.6621 |
| C | -17.0494 | -5.0276 | 12.9932 |
| C | -15.8092 | -4.3408 | 12.9837 |
| O | -14.7120 | -5.1809 | 12.9961 |
| C | -13.3983 | -4.5752 | 12.9448 |
| H | -12.7468 | -5.4587 | 12.9623 |
| H | -13.2756 | -4.0138 | 12.0145 |
| H | -13.2349 | -3.9485 | 13.8267 |
| C | -15.7440 | -2.9435 | 12.9595 |
| H | -14.7868 | -2.4153 | 12.9543 |
| C | -16.9219 | -2.1891 | 12.9453 |
| C | -18.1630 | -2.8753 | 12.9816 |
| O | -19.2591 | -2.0311 | 12.9900 |
| C | -20.5730 | -2.6399 | 12.9834 |
| H | -20.7196 | -3.2401 | 13.8861 |
| H | -20.7143 | -3.2326 | 12.0751 |
| H | -21.2263 | -1.7580 | 12.9853 |
| C | -16.9198 | -0.7146 | 12.8720 |
| H | -17.8929 | -0.2735 | 12.5907 |
| N | -15.8225 | -0.0517 | 13.1211 |
| C | -15.7838 | 1.3742 | 13.0664 |
| C | -14.5976 | 1.9539 | 12.5847 |
| H | -13.7917 | 1.3170 | 12.2254 |
| C | -14.4592 | 3.3475 | 12.5651 |
| H | -13.5467 | 3.7854 | 12.1646 |
| C | -15.4872 | 4.1550 | 13.0435 |
| C | -16.6887 | 3.5732 | 13.5021 |
| C | -17.7385 | 4.6120 | 13.8562 |
| C | -18.2713 | 4.4773 | 15.2964 |
| C | -19.0048 | 3.1794 | 15.6556 |
| H | -18.3347 | 2.3067 | 15.5115 |
| C | -19.4968 | 3.2095 | 17.1018 |
| H | -20.0081 | 2.2755 | 17.3628 |
| H | -18.6690 | 3.3362 | 17.8090 |
| H | -20.2046 | 4.0286 | 17.2723 |
| H | -19.8678 | 3.0222 | 14.9766 |
| H | -17.4172 | 4.5959 | 15.9955 |
| H | -18.9601 | 5.3265 | 15.4915 |
| C | -18.8323 | 4.5839 | 12.8052 |
| C | -19.7440 | 3.5582 | 12.5651 |
| H | -19.7523 | 2.6680 | 13.1947 |
| C | -20.6606 | 3.6688 | 11.5142 |
| H | -21.3816 | 2.8746 | 11.3246 |
| C | -20.6468 | 4.8079 | 10.6911 |
| N | -21.6463 | 4.8673 | 9.6714 |
| C | -21.4134 | 5.4284 | 8.5190 |
| C | -22.4770 | 5.4661 | 7.4958 |
| C | -23.6098 | 4.6476 | 7.5628 |
| H | -23.7251 | 3.9851 | 8.4249 |
| C | -24.5636 | 4.6920 | 6.5412 |
| O | -25.7166 | 3.9300 | 6.5015 |
| C | -25.9406 | 2.9964 | 7.5842 |
| H | -25.1433 | 2.2464 | 7.6123 |
| H | -26.8966 | 2.5442 | 7.2939 |
| H | -26.0305 | 3.5314 | 8.5347 |
| C | -24.4180 | 5.5581 | 5.4266 |
| C | -25.4304 | 5.5237 | 4.3571 |
| H | -26.0783 | 4.6346 | 4.3677 |
| N | -25.4882 | 6.5084 | 3.4877 |
| C | -26.4431 | 6.5102 | 2.4314 |
| C | -27.7667 | 6.0417 | 2.5779 |
| H | -28.1010 | 5.6251 | 3.5276 |
| C | -28.6419 | 6.1317 | 1.5008 |
| C | -30.0685 | 5.6163 | 1.4390 |
| C | -30.0621 | 4.1003 | 1.4784 |
| C | -29.6761 | 3.2855 | 2.5408 |
| C | -29.6973 | 1.8943 | 2.3985 |
| H | -29.4062 | 1.2506 | 3.2275 |
| C | -30.0907 | 1.3218 | 1.1766 |
| N | -30.1354 | -0.1069 | 1.1287 |
| C | -29.8558 | -0.7632 | 0.0451 |
| C | -29.9179 | -2.2396 | 0.0422 |
| C | -29.9357 | -2.9889 | 1.2235 |
| C | -29.9478 | -4.3867 | 1.1639 |
| O | -29.9631 | -5.2247 | 2.2636 |
| C | -29.9430 | -4.6111 | 3.5745 |
| H | -29.9624 | -5.4917 | 4.2282 |
| H | -29.0244 | -4.0344 | 3.7157 |
| H | -30.8343 | -3.9935 | 3.7202 |
| C | -29.9431 | -5.0774 | -0.0738 |
| C | -29.9071 | -6.5547 | -0.0810 |
| N | -30.1925 | -7.2064 | -1.1653 |
| C | -30.1520 | -8.6354 | -1.2148 |
| C | -30.5529 | -9.4554 | -0.1387 |
| C | -30.5110 | -10.8376 | -0.2885 |
| C | -30.8227 | -11.8757 | 0.7719 |
| C | -30.5828 | -13.2795 | 0.0292 |
| C | -31.8314 | -14.1505 | 0.0311 |
| H | -31.6553 | -15.1164 | -0.4620 |
| H | -32.1770 | -14.3683 | 1.0514 |
| H | -32.6662 | -13.6683 | -0.4966 |
| C | -29.3467 | -13.9834 | 0.7725 |
| C | -29.7062 | -15.3873 | 1.3000 |
| H | -30.1256 | -15.9807 | 0.4605 |
| C | -28.5762 | -16.2089 | 1.9336 |
| C | -29.1099 | -17.5151 | 2.5204 |
| H | -29.5718 | -18.1456 | 1.7522 |
| H | -28.3051 | -18.0992 | 2.9812 |
| H | -29.8644 | -17.3337 | 3.2944 |
| H | -27.7996 | -16.4384 | 1.1770 |
| H | -28.0721 | -15.6236 | 2.7320 |
| H | -30.5200 | -15.2773 | 2.0463 |
| C | -28.2444 | -13.9981 | -0.2690 |
| C | -26.9330 | -14.4373 | -0.1055 |
| H | -26.5964 | -14.8374 | 0.8500 |
| C | -26.0306 | -14.3576 | -1.1707 |
| H | -25.0066 | -14.7098 | -1.0535 |
| C | -26.4436 | -13.8125 | -2.3987 |
| N | -25.4761 | -13.8166 | -3.4530 |
| C | -25.4147 | -12.8629 | -4.3268 |
| C | -24.3907 | -12.9057 | -5.3922 |
| C | -23.2794 | -13.7561 | -5.3272 |
| H | -23.1927 | -14.4332 | -4.4755 |
| C | -22.3142 | -13.7235 | -6.3397 |
| O | -21.1784 | -14.5114 | -6.3737 |
| C | -20.9585 | -15.4200 | -5.2665 |
| H | -21.7669 | -16.1556 | -5.2077 |
| H | -20.0142 | -15.8961 | -5.5580 |
| H | -20.8473 | -14.8622 | -4.3321 |
| C | -22.4281 | -12.8484 | -7.4486 |
| C | -21.3530 | -12.8179 | -8.4622 |
| H | -20.4120 | -13.3027 | -8.1355 |
| N | -21.5623 | -12.2410 | -9.6029 |
| C | -20.5534 | -12.1689 | -10.6149 |
| C | -19.6373 | -13.2106 | -10.8741 |
| H | -19.6505 | -14.1243 | -10.2779 |
| C | -18.7282 | -13.0682 | -11.9184 |
| C | -17.6225 | -14.0336 | -12.3029 |
| C | -18.1533 | -15.4447 | -12.6293 |
| H | -17.3243 | -16.0401 | -13.0669 |
| C | -18.7522 | -16.2554 | -11.4731 |
| C | -19.3576 | -17.5642 | -11.9796 |
| H | -18.6045 | -18.1996 | -12.4589 |
| H | -19.7965 | -18.1408 | -11.1576 |
| H | -20.1526 | -17.3860 | -12.7131 |
| H | -17.9720 | -16.4797 | -10.7191 |
| H | -19.5340 | -15.6656 | -10.9484 |
| H | -18.9241 | -15.3448 | -13.4222 |
| C | -16.5391 | -14.0384 | -11.2400 |
| C | -16.6516 | -14.4643 | -9.9178 |
| H | -17.5982 | -14.8440 | -9.5363 |
| C | -15.5447 | -14.3999 | -9.0643 |
| H | -15.6247 | -14.7438 | -8.0339 |
| C | -14.3249 | -13.8850 | -9.5365 |
| N | -13.2144 | -13.9213 | -8.6339 |
| C | -12.4035 | -12.9197 | -8.4962 |
| C | -11.2838 | -13.0056 | -7.5335 |
| C | -11.1157 | -14.1025 | -6.6796 |
| H | -11.8237 | -14.9312 | -6.7630 |
| C | -10.0743 | -14.1175 | -5.7479 |
| O | -9.8090 | -15.1642 | -4.8827 |
| C | -10.6097 | -16.3636 | -5.0233 |
| H | -10.6184 | -16.7151 | -6.0602 |
| H | -10.0724 | -17.0684 | -4.3773 |
| H | -11.6213 | -16.1732 | -4.6541 |
| C | -9.1838 | -13.0212 | -5.6087 |
| C | -8.1717 | -13.0314 | -4.5289 |
| H | -8.3633 | -13.7960 | -3.7504 |
| N | -7.1977 | -12.1805 | -4.5483 |
| C | -6.2168 | -12.1073 | -3.5066 |
| C | -5.8779 | -13.1788 | -2.6528 |
| H | -6.3737 | -14.1475 | -2.7501 |
| C | -4.8941 | -12.9948 | -1.6898 |
| C | -4.4978 | -13.9693 | -0.5935 |
| C | -4.1159 | -15.3658 | -1.1207 |
| H | -3.6291 | -15.9329 | -0.2987 |
| C | -5.2511 | -16.2349 | -1.6762 |
| C | -4.7062 | -17.5099 | -2.3186 |
| H | -4.1756 | -18.1348 | -1.5913 |
| H | -5.5163 | -18.1158 | -2.7402 |
| H | -4.0078 | -17.2881 | -3.1336 |
| H | -5.9535 | -16.5069 | -0.8630 |
| H | -5.8428 | -15.6659 | -2.4259 |
| H | -3.3483 | -15.2373 | -1.9120 |
| C | -5.6079 | -13.9929 | 0.4422 |
| C | -6.9225 | -14.4178 | 0.2616 |
| H | -7.2542 | -14.8119 | -0.6977 |
| C | -7.8383 | -14.3240 | 1.3143 |
| H | -8.8625 | -14.6726 | 1.1859 |
| C | -7.4405 | -13.7633 | 2.5402 |
| N | -8.4178 | -13.7514 | 3.5823 |
| C | -8.4840 | -12.7825 | 4.4475 |
| C | -9.5057 | -12.8210 | 5.5136 |
| C | -10.6167 | -13.6693 | 5.4557 |
| H | -10.7120 | -14.3500 | 4.6045 |
| C | -11.5747 | -13.6344 | 6.4750 |
| O | -12.7087 | -14.4247 | 6.5206 |
| C | -12.9319 | -15.3449 | 5.4239 |
| H | -12.1200 | -16.0770 | 5.3718 |
| H | -13.8746 | -15.8211 | 5.7227 |
| H | -13.0482 | -14.7960 | 4.4848 |
| C | -11.4525 | -12.7558 | 7.5811 |
| C | -12.5193 | -12.7216 | 8.6033 |
| H | -13.4644 | -13.2023 | 8.2829 |
| N | -12.2995 | -12.1485 | 9.7434 |
| C | -13.3099 | -12.0729 | 10.7534 |
| C | -14.2109 | -13.1236 | 11.0246 |
| H | -14.1745 | -14.0484 | 10.4461 |
| C | -13.3403 | -10.8958 | 11.5226 |
| C | -14.2707 | -10.7612 | 12.5566 |
| C | -15.1571 | -11.8007 | 12.8338 |
| H | -14.2987 | -9.8369 | 13.1317 |
| H | -12.6391 | -10.0915 | 11.3032 |
| C | -10.3293 | -11.9235 | 7.6498 |
| H | -10.2253 | -11.2596 | 8.5109 |
| C | -9.3723 | -11.9573 | 6.6307 |
| O | -8.2294 | -11.1792 | 6.5941 |
| C | -8.0054 | -10.2653 | 7.6953 |
| H | -7.9160 | -10.8175 | 8.6364 |
| H | -7.0476 | -9.8095 | 7.4146 |
| H | -8.8021 | -9.5168 | 7.7360 |
| H | -7.8356 | -11.8872 | 4.4746 |
| C | -6.1175 | -13.3025 | 2.7181 |
| H | -5.8228 | -12.8479 | 3.6607 |
| C | -5.2084 | -13.4342 | 1.6753 |
| C | -3.7640 | -12.9670 | 1.6584 |
| C | -2.8872 | -13.6919 | 2.7004 |
| H | -2.8002 | -14.7598 | 2.4085 |
| C | -3.3551 | -13.6454 | 4.1605 |
| C | -2.2839 | -14.2014 | 5.0970 |
| H | -2.0589 | -15.2508 | 4.8762 |
| H | -2.6090 | -14.1503 | 6.1424 |
| H | -1.3458 | -13.6400 | 5.0200 |
| H | -4.2877 | -14.2325 | 4.2790 |
| H | -3.6030 | -12.6040 | 4.4581 |
| H | -1.8638 | -13.2654 | 2.6435 |
| C | -3.7088 | -11.4581 | 1.7928 |
| C | -3.3673 | -10.8185 | 0.5807 |
| C | -3.1057 | -11.8059 | -0.5412 |
| C | -1.6976 | -11.6229 | -1.1526 |
| H | -1.4730 | -12.5038 | -1.7908 |
| H | -0.9558 | -11.6347 | -0.3237 |
| C | -1.4581 | -10.3662 | -1.9991 |
| H | -2.0470 | -10.4235 | -2.9362 |
| C | 0.0228 | -10.2099 | -2.3392 |
| H | 0.4040 | -11.0553 | -2.9232 |
| H | 0.6476 | -10.1270 | -1.4393 |
| H | 0.2006 | -9.3018 | -2.9275 |
| H | -1.8133 | -9.4602 | -1.4622 |
| C | -3.3025 | -9.4324 | 0.5125 |
| H | -3.0031 | -8.9426 | -0.4181 |
| C | -3.5916 | -8.6666 | 1.6650 |
| C | -3.9122 | -9.3055 | 2.8744 |
| H | -4.1108 | -8.7121 | 3.7638 |
| N | -3.5138 | -7.2347 | 1.6866 |
| C | -3.9062 | -6.5537 | 0.6498 |
| H | -4.3356 | -6.9963 | -0.2738 |
| C | -3.8131 | -5.0822 | 0.6000 |
| C | -3.8917 | -4.4643 | -0.6734 |
| O | -3.9824 | -5.3806 | -1.7062 |
| C | -3.9797 | -4.8658 | -3.0552 |
| H | -3.9775 | -5.7939 | -3.6415 |
| H | -4.8893 | -4.2810 | -3.2322 |
| H | -3.0717 | -4.2801 | -3.2355 |
| C | -3.8862 | -3.0751 | -0.8291 |
| H | -3.9624 | -2.6060 | -1.8124 |
| C | -3.7887 | -2.2555 | 0.3004 |
| C | -3.6554 | -2.8717 | 1.5718 |
| O | -3.4941 | -1.9655 | 2.5996 |
| C | -3.5451 | -2.4747 | 3.9544 |
| H | -2.6872 | -3.1251 | 4.1462 |
| H | -4.5000 | -2.9830 | 4.1288 |
| H | -3.4778 | -1.5514 | 4.5440 |
| C | -3.8568 | -0.7812 | 0.2115 |
| H | -4.0550 | -0.2900 | 1.1837 |
| N | -3.7097 | -0.1852 | -0.9295 |
| C | -3.7882 | 1.2405 | -1.0475 |
| C | -4.2595 | 1.7424 | -2.2742 |
| H | -4.5893 | 1.0478 | -3.0473 |
| C | -4.3147 | 3.1226 | -2.4934 |
| H | -4.7084 | 3.4967 | -3.4364 |
| C | -3.8823 | 4.0036 | -1.5039 |
| C | -3.4400 | 3.5008 | -0.2606 |
| C | -3.1435 | 4.6059 | 0.7381 |
| C | -1.7110 | 4.5377 | 1.3062 |
| C | -1.3340 | 3.3007 | 2.1316 |
| H | -1.5206 | 2.3722 | 1.5498 |
| C | 0.1355 | 3.3431 | 2.5463 |
| H | 0.4116 | 2.4463 | 3.1152 |
| H | 0.8069 | 3.3819 | 1.6785 |
| H | 0.3591 | 4.2096 | 3.1779 |
| H | -1.9703 | 3.2338 | 3.0364 |
| H | -1.0002 | 4.6118 | 0.4544 |
| H | -1.5422 | 5.4340 | 1.9389 |
| C | -4.2167 | 4.6110 | 1.8116 |
| C | -4.4522 | 3.6386 | 2.7805 |
| H | -3.8204 | 2.7514 | 2.8392 |
| C | -5.5044 | 3.7949 | 3.6895 |
| H | -5.6874 | 3.0388 | 4.4531 |
| C | -6.3352 | 4.9278 | 3.6176 |
| N | -7.3500 | 5.0207 | 4.6234 |
| C | -8.3683 | 5.8129 | 4.5162 |
| C | -9.3791 | 5.8610 | 5.5964 |
| C | -9.4314 | 4.8982 | 6.6093 |
| H | -8.6714 | 4.1138 | 6.6235 |
| C | -10.4412 | 4.9565 | 7.5760 |
| O | -10.5841 | 4.0711 | 8.6287 |
| C | -9.6475 | 2.9734 | 8.7144 |
| H | -9.7327 | 2.3331 | 7.8313 |
| H | -10.0009 | 2.4509 | 9.6145 |
| H | -8.6316 | 3.3560 | 8.8528 |
| C | -11.4390 | 5.9635 | 7.5552 |
| C | -12.5302 | 5.9038 | 8.5490 |
| H | -12.5137 | 4.9892 | 9.1749 |
| N | -13.4159 | 6.8493 | 8.6162 |
| C | -14.4949 | 6.7803 | 9.5553 |
| C | -14.3533 | 6.2551 | 10.8578 |
| H | -13.3896 | 5.8698 | 11.1993 |
| C | -15.4478 | 6.2553 | 11.7126 |
| C | -16.6826 | 6.8004 | 11.2973 |
| C | -17.7450 | 6.7203 | 12.3758 |
| C | -18.3134 | 8.1048 | 12.7539 |
| H | -19.0082 | 7.9730 | 13.6111 |
| C | -19.0518 | 8.8844 | 11.6597 |
| H | -19.9123 | 8.2971 | 11.2781 |
| H | -18.3856 | 9.0573 | 10.7905 |
| C | -19.5506 | 10.2271 | 12.1905 |
| H | -20.2407 | 10.1010 | 13.0321 |
| H | -20.0882 | 10.7794 | 11.4107 |
| H | -18.7255 | 10.8630 | 12.5321 |
| H | -17.4752 | 8.7363 | 13.1188 |
| C | -16.8185 | 7.3131 | 10.0097 |
| H | -17.7724 | 7.7247 | 9.6760 |
| C | -15.7268 | 7.3114 | 9.1352 |
| H | -15.8260 | 7.7151 | 8.1281 |
| C | -11.3642 | 6.9487 | 6.5649 |
| H | -12.1116 | 7.7459 | 6.5629 |
| C | -10.3527 | 6.8944 | 5.6006 |
| O | -10.2301 | 7.7772 | 4.5432 |
| C | -10.9937 | 9.0078 | 4.5965 |
| H | -12.0667 | 8.8000 | 4.5469 |
| H | -10.6567 | 9.5164 | 3.6820 |
| H | -10.7311 | 9.5817 | 5.4892 |
| H | -8.5899 | 6.4898 | 3.6683 |
| C | -6.1114 | 5.9038 | 2.6224 |
| H | -6.7485 | 6.7874 | 2.5634 |
| C | -5.0598 | 5.7415 | 1.7295 |
| C | -4.6826 | 6.6661 | 0.5874 |
| C | -5.7359 | 6.6457 | -0.5038 |
| C | -5.2603 | 6.0662 | -1.7011 |
| C | -6.0696 | 6.0048 | -2.8274 |
| H | -5.6763 | 5.5943 | -3.7616 |
| C | -7.3897 | 6.5022 | -2.7611 |
| N | -8.2718 | 6.5369 | -3.8898 |
| C | -8.2176 | 5.6257 | -4.8140 |
| H | -7.5323 | 4.7552 | -4.8248 |
| C | -9.1348 | 5.6726 | -5.9725 |
| C | -8.9231 | 4.7714 | -7.0542 |
| O | -7.7736 | 4.0182 | -6.9120 |
| C | -7.3215 | 3.2494 | -8.0504 |
| H | -7.9978 | 2.3982 | -8.2208 |
| H | -6.3361 | 2.9016 | -7.7105 |
| H | -7.2268 | 3.8834 | -8.9380 |
| C | -9.8272 | 4.6643 | -8.1162 |
| H | -9.6806 | 3.9476 | -8.9253 |
| C | -10.9492 | 5.4964 | -8.1494 |
| C | -12.0395 | 5.3809 | -9.1373 |
| N | -11.8793 | 4.7950 | -10.2865 |
| C | -12.9826 | 4.7120 | -11.1990 |
| C | -13.0185 | 3.5940 | -12.0489 |
| H | -12.2433 | 2.8338 | -11.9745 |
| C | -14.0558 | 3.4626 | -12.9805 |
| H | -14.0747 | 2.5999 | -13.6538 |
| C | -15.0424 | 4.4399 | -13.0706 |
| C | -16.2356 | 4.4642 | -14.0056 |
| C | -17.2591 | 3.4253 | -13.5866 |
| C | -17.0851 | 2.0481 | -13.5470 |
| C | -18.1533 | 1.2318 | -13.1171 |
| N | -18.0949 | -0.1977 | -13.1297 |
| C | -17.0089 | -0.8380 | -12.8389 |
| C | -16.9929 | -2.3157 | -12.8834 |
| C | -18.1695 | -3.0737 | -12.8992 |
| C | -18.0996 | -4.4706 | -12.9082 |
| O | -19.1919 | -5.3200 | -12.9223 |
| C | -20.5079 | -4.7148 | -12.9206 |
| H | -21.1586 | -5.5985 | -12.9370 |
| H | -20.6605 | -4.1326 | -12.0067 |
| H | -20.6491 | -4.1070 | -13.8199 |
| C | -16.8556 | -5.1515 | -12.9046 |
| C | -16.8388 | -6.6282 | -12.8706 |
| N | -15.7524 | -7.2683 | -13.1833 |
| C | -15.6918 | -8.6961 | -13.1544 |
| C | -16.7700 | -9.5206 | -13.5391 |
| C | -16.6102 | -10.9006 | -13.5125 |
| C | -17.6728 | -11.9450 | -13.8022 |
| C | -18.2552 | -11.8191 | -15.2269 |
| H | -18.9070 | -12.6987 | -15.4167 |
| H | -17.4144 | -11.8865 | -15.9495 |
| C | -19.0689 | -10.5590 | -15.5488 |
| H | -19.9801 | -10.5201 | -14.9201 |
| C | -19.4694 | -10.5280 | -17.0236 |
| H | -20.0856 | -11.3921 | -17.2956 |
| H | -18.5927 | -10.5298 | -17.6807 |
| H | -20.0476 | -9.6271 | -17.2531 |
| H | -18.4824 | -9.6481 | -15.3061 |
| C | -15.3801 | -11.4737 | -13.1255 |
| C | -15.4190 | -12.9901 | -13.1306 |
| C | -14.3738 | -13.6179 | -14.0752 |
| H | -14.5105 | -14.7196 | -14.0584 |
| C | -12.8974 | -13.3136 | -13.7940 |
| C | -11.9912 | -14.0155 | -14.8048 |
| H | -12.1240 | -15.1030 | -14.7818 |
| H | -10.9352 | -13.8110 | -14.5956 |
| H | -12.1918 | -13.6808 | -15.8290 |
| H | -12.6263 | -13.6365 | -12.7671 |
| H | -12.7140 | -12.2211 | -13.8347 |
| H | -14.5993 | -13.2879 | -15.1108 |
| C | -14.3186 | -10.6546 | -12.7480 |
| H | -13.3701 | -11.0887 | -12.4351 |
| H | -17.7124 | -9.0812 | -13.8677 |
| C | -14.4658 | -9.2646 | -12.7667 |
| H | -13.6392 | -8.6174 | -12.4756 |
| H | -17.7983 | -7.0820 | -12.5578 |
| C | -15.6795 | -4.3936 | -12.9106 |
| H | -14.7209 | -4.9181 | -12.9283 |
| H | -19.1278 | -2.5490 | -12.9081 |
| C | -15.7498 | -2.9967 | -12.8991 |
| O | -14.6565 | -2.1500 | -12.9013 |
| C | -13.3400 | -2.7540 | -12.9144 |
| H | -13.2038 | -3.3520 | -13.8206 |
| H | -13.1850 | -3.3465 | -12.0080 |
| H | -12.6892 | -1.8702 | -12.9237 |
| H | -16.0417 | -0.3876 | -12.5404 |
| C | -19.3716 | 1.8079 | -12.7143 |
| H | -20.1893 | 1.1665 | -12.3875 |
| C | -19.5190 | 3.1977 | -12.7241 |
| H | -20.4542 | 3.6368 | -12.3770 |
| H | -16.1405 | 1.5968 | -13.8430 |
| C | -18.4754 | 4.0072 | -13.1655 |
| C | -18.4285 | 5.5252 | -13.1788 |
| C | -18.4386 | 6.0028 | -11.7359 |
| C | -19.5034 | 5.9506 | -10.8463 |
| H | -20.4957 | 5.6516 | -11.1896 |
| C | -19.2928 | 6.3079 | -9.4965 |
| N | -20.3492 | 6.3453 | -8.5303 |
| C | -21.2675 | 5.4321 | -8.4962 |
| H | -21.3342 | 4.5523 | -9.1673 |
| C | -22.3482 | 5.4967 | -7.4889 |
| C | -23.4801 | 4.6536 | -7.6332 |
| O | -23.4502 | 3.8815 | -8.7805 |
| C | -24.5439 | 2.9561 | -8.9935 |
| H | -24.5811 | 2.2160 | -8.1885 |
| H | -24.2572 | 2.4910 | -9.9454 |
| H | -25.4891 | 3.5002 | -9.0893 |
| C | -24.5070 | 4.6344 | -6.6840 |
| H | -25.3782 | 3.9847 | -6.7978 |
| C | -24.4356 | 5.4623 | -5.5580 |
| C | -25.4643 | 5.4387 | -4.4963 |
| N | -26.6077 | 4.8684 | -4.7177 |
| C | -27.6284 | 4.8037 | -3.7174 |
| C | -28.4374 | 3.6537 | -3.7274 |
| H | -28.2416 | 2.8624 | -4.4499 |
| C | -29.4830 | 3.5293 | -2.8078 |
| H | -30.1047 | 2.6325 | -2.8165 |
| C | -29.7303 | 4.5480 | -1.8900 |
| C | -30.7847 | 4.5685 | -0.8004 |
| C | -32.2204 | 4.4395 | -1.3510 |
| C | -32.5834 | 3.1361 | -2.0741 |
| H | -32.4578 | 2.2689 | -1.3928 |
| C | -34.0240 | 3.1729 | -2.5837 |
| H | -34.2871 | 2.2391 | -3.0917 |
| H | -34.7387 | 3.3128 | -1.7647 |
| H | -34.1794 | 3.9896 | -3.2979 |
| H | -31.8948 | 2.9614 | -2.9259 |
| H | -32.9297 | 4.5706 | -0.5062 |
| H | -32.3999 | 5.2833 | -2.0503 |
| C | -28.9217 | 5.7049 | -1.8856 |
| C | -29.3033 | 6.6729 | -0.7796 |
| C | -29.6585 | 8.0775 | -1.3099 |
| H | -30.4966 | 7.9725 | -2.0308 |
| C | -28.5405 | 8.8782 | -1.9886 |
| H | -28.1149 | 8.3061 | -2.8393 |
| H | -27.7045 | 9.0488 | -1.2807 |
| C | -29.0610 | 10.2244 | -2.4915 |
| H | -29.8718 | 10.0993 | -3.2180 |
| H | -28.2658 | 10.7942 | -2.9842 |
| H | -29.4473 | 10.8420 | -1.6726 |
| H | -30.0447 | 8.6820 | -0.4617 |
| C | -27.8783 | 5.8408 | -2.7932 |
| H | -27.2680 | 6.7442 | -2.8008 |
| H | -25.1465 | 5.9233 | -3.5529 |
| C | -23.3176 | 6.3262 | -5.4285 |
| O | -23.3632 | 7.1170 | -4.2953 |
| C | -22.2461 | 8.0063 | -4.0519 |
| H | -21.3232 | 7.4323 | -3.9236 |
| H | -22.5465 | 8.4867 | -3.1118 |
| H | -22.1621 | 8.7414 | -4.8594 |
| C | -22.2900 | 6.3454 | -6.3776 |
| H | -21.4296 | 7.0117 | -6.2747 |
| C | -18.0163 | 6.7034 | -9.0610 |
| C | -16.9549 | 6.7637 | -9.9707 |
| H | -15.9656 | 7.0485 | -9.6176 |
| H | -17.8599 | 6.9597 | -8.0134 |
| C | -17.1676 | 6.4484 | -11.3116 |
| C | -16.1680 | 6.5094 | -12.4493 |
| C | -15.7007 | 7.9543 | -12.7330 |
| H | -14.9346 | 7.9169 | -13.5352 |
| C | -15.1331 | 8.7476 | -11.5493 |
| H | -14.3582 | 8.1532 | -11.0181 |
| H | -15.9332 | 8.9514 | -10.8102 |
| C | -14.5286 | 10.0709 | -12.0166 |
| H | -13.7261 | 9.9156 | -12.7470 |
| H | -14.1000 | 10.6276 | -11.1761 |
| H | -15.2805 | 10.7148 | -12.4867 |
| H | -16.5587 | 8.5289 | -13.1424 |
| C | -19.5189 | 6.1730 | -14.0520 |
| H | -19.3351 | 5.8887 | -15.1102 |
| C | -20.9773 | 5.8325 | -13.7207 |
| H | -21.1544 | 4.7449 | -13.8408 |
| H | -21.1979 | 6.0690 | -12.6569 |
| C | -21.9393 | 6.6025 | -14.6244 |
| H | -21.7951 | 6.3475 | -15.6803 |
| H | -22.9812 | 6.3750 | -14.3733 |
| H | -21.8098 | 7.6863 | -14.5263 |
| H | -19.3976 | 7.2753 | -13.9948 |
| C | -15.8260 | 4.3297 | -15.4874 |
| C | -15.2806 | 2.9699 | -15.9408 |
| H | -16.0829 | 2.2058 | -15.9001 |
| C | -14.7287 | 3.0499 | -17.3635 |
| H | -14.3305 | 2.0815 | -17.6867 |
| H | -15.5040 | 3.3402 | -18.0816 |
| H | -13.9153 | 3.7808 | -17.4437 |
| H | -14.4809 | 2.6200 | -15.2530 |
| H | -16.7066 | 4.5686 | -16.1201 |
| H | -15.0625 | 5.1067 | -15.7031 |
| C | -16.9507 | 5.8687 | -13.6956 |
| C | -16.9557 | 6.7921 | -14.9018 |
| H | -17.4788 | 6.3374 | -15.7439 |
| H | -15.9466 | 7.0024 | -15.2348 |
| H | -17.4233 | 7.7599 | -14.6796 |
| C | -15.0029 | 5.5662 | -12.2194 |
| C | -13.9759 | 5.7116 | -11.2957 |
| H | -13.9277 | 6.6072 | -10.6703 |
| H | -12.9890 | 5.8399 | -8.7833 |
| C | -11.0943 | 6.4753 | -7.1360 |
| O | -12.1395 | 7.3486 | -7.3847 |
| C | -12.6999 | 8.0582 | -6.2593 |
| H | -13.1517 | 7.3411 | -5.5674 |
| H | -13.4657 | 8.6797 | -6.7431 |
| H | -11.9408 | 8.6838 | -5.7803 |
| C | -10.2211 | 6.5473 | -6.0440 |
| H | -10.3654 | 7.2791 | -5.2465 |
| C | -7.8758 | 7.0467 | -1.5599 |
| H | -8.8993 | 7.4174 | -1.5091 |
| C | -7.0466 | 7.1107 | -0.4332 |
| H | -7.4376 | 7.5292 | 0.4940 |
| C | -4.3615 | 8.0963 | 1.0760 |
| H | -3.5387 | 8.0283 | 1.8187 |
| C | -5.5052 | 8.9035 | 1.7030 |
| H | -5.9666 | 8.3406 | 2.5411 |
| H | -6.3104 | 9.0704 | 0.9598 |
| C | -5.0027 | 10.2525 | 2.2164 |
| H | -4.2250 | 10.1330 | 2.9795 |
| H | -5.8166 | 10.8308 | 2.6685 |
| H | -4.5802 | 10.8613 | 1.4088 |
| H | -3.9663 | 8.6779 | 0.2166 |
| C | -3.4055 | 5.9591 | -0.0841 |
| C | -2.1862 | 6.8712 | -0.0984 |
| H | -1.3223 | 6.3903 | -0.5779 |
| H | -1.8758 | 7.1548 | 0.9161 |
| H | -2.3817 | 7.8025 | -0.6476 |
| C | -3.8523 | 5.5193 | -1.5617 |
| C | -2.8918 | 6.0570 | -2.6448 |
| H | -1.8510 | 5.7959 | -2.3558 |
| C | -3.1130 | 5.5655 | -4.0804 |
| H | -2.8899 | 4.4820 | -4.1509 |
| H | -4.1792 | 5.6853 | -4.3725 |
| C | -2.2281 | 6.3331 | -5.0614 |
| H | -1.1635 | 6.1787 | -4.8516 |
| H | -2.4087 | 6.0105 | -6.0930 |
| H | -2.4181 | 7.4121 | -5.0202 |
| H | -2.9542 | 7.1657 | -2.6376 |
| C | -3.3772 | 2.1322 | -0.0330 |
| H | -2.9998 | 1.7482 | 0.9167 |
| C | -3.6746 | -4.2646 | 1.7256 |
| H | -3.5773 | -4.7317 | 2.7058 |
| C | -3.9718 | -10.7019 | 2.9314 |
| H | -4.2131 | -11.1990 | 3.8758 |
| C | -3.2785 | -13.2449 | 0.1544 |
| C | -1.9939 | -14.0616 | 0.1053 |
| H | -2.1227 | -15.0506 | 0.5685 |
| H | -1.6601 | -14.2313 | -0.9277 |
| H | -1.1734 | -13.5598 | 0.6354 |
| C | -4.1960 | -11.7703 | -1.5940 |
| C | -4.5534 | -10.7047 | -2.4169 |
| H | -4.0655 | -9.7363 | -2.3161 |
| C | -5.5583 | -10.8702 | -3.3762 |
| H | -5.8389 | -10.0418 | -4.0271 |
| C | -9.3319 | -11.9379 | -6.4828 |
| H | -8.6398 | -11.0969 | -6.3911 |
| C | -10.3534 | -11.9394 | -7.4392 |
| O | -10.5771 | -10.9303 | -8.3564 |
| C | -9.6290 | -9.8357 | -8.3925 |
| H | -8.6233 | -10.2118 | -8.6051 |
| H | -10.0121 | -9.2380 | -9.2284 |
| H | -9.6745 | -9.2643 | -7.4607 |
| H | -12.4614 | -11.9521 | -9.0350 |
| C | -14.2118 | -13.4272 | -10.8664 |
| H | -13.2630 | -13.0349 | -11.2320 |
| C | -15.3188 | -13.5020 | -11.7050 |
| C | -16.9220 | -13.3433 | -13.5729 |
| C | -16.9669 | -14.2300 | -14.8101 |
| H | -16.4684 | -15.1943 | -14.6393 |
| H | -17.9986 | -14.4504 | -15.1167 |
| H | -16.4696 | -13.7584 | -15.6688 |
| C | -18.7349 | -11.9083 | -12.7204 |
| C | -19.6299 | -10.8760 | -12.4501 |
| H | -19.6231 | -9.9629 | -13.0441 |
| C | -20.5481 | -11.0054 | -11.4018 |
| H | -21.2580 | -10.2067 | -11.1901 |
| C | -23.5529 | -12.0187 | -7.5284 |
| H | -23.6505 | -11.3571 | -8.3929 |
| C | -24.5173 | -12.0506 | -6.5157 |
| O | -25.6634 | -11.2761 | -6.4916 |
| C | -25.8688 | -10.3462 | -7.5827 |
| H | -25.9543 | -10.8856 | -8.5308 |
| H | -26.8248 | -9.8846 | -7.3041 |
| H | -25.0655 | -9.6039 | -7.6067 |
| H | -26.0636 | -11.9665 | -4.3675 |
| C | -27.7657 | -13.3483 | -2.5632 |
| H | -28.0867 | -12.9340 | -3.5182 |
| C | -28.6549 | -13.4405 | -1.4996 |
| C | -28.9775 | -13.0135 | 1.8810 |
| C | -27.9510 | -13.1578 | 2.8071 |
| H | -27.3728 | -14.0824 | 2.8491 |
| C | -27.6808 | -12.1017 | 3.7025 |
| C | -28.4491 | -10.9252 | 3.6663 |
| C | -29.4838 | -10.7963 | 2.7339 |
| H | -30.0593 | -9.8719 | 2.7004 |
| H | -28.2304 | -10.1165 | 4.3623 |
| C | -30.0851 | -12.9315 | -1.4571 |
| C | -30.9841 | -13.5710 | -2.5357 |
| H | -30.9929 | -14.6694 | -2.3738 |
| C | -30.6180 | -13.3095 | -4.0015 |
| C | -31.6183 | -13.9793 | -4.9434 |
| H | -31.6509 | -15.0645 | -4.7925 |
| H | -31.3483 | -13.8064 | -5.9910 |
| H | -32.6376 | -13.5945 | -4.7973 |
| H | -29.5965 | -13.6871 | -4.2176 |
| H | -30.5955 | -12.2202 | -4.2069 |
| H | -32.0244 | -13.2173 | -2.3728 |
| C | -29.7614 | -11.8401 | 1.8540 |
| C | -32.2561 | -11.7481 | 1.3278 |
| H | -32.4685 | -12.6368 | 1.9591 |
| H | -32.9613 | -11.7917 | 0.4714 |
| C | -32.5780 | -10.4987 | 2.1570 |
| H | -31.9681 | -10.4880 | 3.0823 |
| C | -34.0596 | -10.4548 | 2.5284 |
| H | -34.3524 | -11.3232 | 3.1291 |
| H | -34.7011 | -10.4385 | 1.6398 |
| H | -34.2929 | -9.5581 | 3.1136 |
| H | -32.3100 | -9.5790 | 1.5945 |
| C | -30.0898 | -11.4148 | -1.5050 |
| C | -29.7002 | -10.6007 | -2.5651 |
| H | -29.3700 | -11.0398 | -3.5075 |
| H | -30.9117 | -9.0140 | 0.7912 |
| C | -29.7327 | -9.2082 | -2.4273 |
| H | -29.4311 | -8.5657 | -3.2533 |
| H | -29.6106 | -6.9993 | 0.8889 |
| C | -29.9551 | -4.3282 | -1.2552 |
| H | -29.9745 | -4.8596 | -2.2105 |
| H | -29.9403 | -2.4577 | 2.1783 |
| C | -29.9398 | -2.9311 | -1.1961 |
| O | -29.9457 | -2.0915 | -2.2955 |
| C | -29.9418 | -2.7057 | -3.6070 |
| H | -30.8452 | -3.3072 | -3.7475 |
| H | -29.0316 | -3.2965 | -3.7492 |
| H | -29.9489 | -1.8259 | -4.2631 |
| H | -29.5537 | -0.3251 | -0.9256 |
| C | -30.4742 | 2.1437 | 0.0950 |
| H | -30.7891 | 1.6979 | -0.8474 |
| H | -29.3531 | 3.7235 | 3.4864 |
| C | -30.4599 | 3.5244 | 0.2528 |
| C | -30.5495 | 5.9696 | -0.0515 |
| C | -31.7949 | 6.8466 | -0.0640 |
| H | -32.6415 | 6.3597 | 0.4399 |
| H | -32.1191 | 7.0794 | -1.0873 |
| H | -31.6252 | 7.8043 | 0.4466 |
| C | -30.9831 | 6.2459 | 2.5098 |
| H | -32.0229 | 5.9042 | 2.3212 |
| C | -30.6493 | 5.9586 | 3.9789 |
| H | -30.6902 | 4.8684 | 4.1768 |
| H | -29.6115 | 6.2777 | 4.2100 |
| C | -31.6207 | 6.6788 | 4.9135 |
| H | -32.6549 | 6.3557 | 4.7501 |
| H | -31.3776 | 6.4792 | 5.9633 |
| H | -31.5888 | 7.7652 | 4.7725 |
| H | -30.9801 | 7.3467 | 2.3636 |
| C | -28.2176 | 6.6951 | 0.2786 |
| C | -26.9098 | 7.1525 | 0.1371 |
| H | -26.5701 | 7.5743 | -0.8082 |
| C | -26.0195 | 7.0698 | 1.2137 |
| H | -24.9972 | 7.4316 | 1.1119 |
| C | -23.2985 | 6.3936 | 5.3724 |
| H | -23.1967 | 7.0744 | 4.5239 |
| C | -22.3418 | 6.3474 | 6.3919 |
| O | -21.2011 | 7.1258 | 6.4427 |
| C | -20.9536 | 8.0284 | 5.3370 |
| H | -20.8374 | 7.4688 | 4.4043 |
| H | -20.0063 | 8.4931 | 5.6391 |
| H | -21.7537 | 8.7744 | 5.2712 |
| H | -20.4624 | 5.8956 | 8.2030 |
| C | -19.7248 | 5.8495 | 10.9296 |
| H | -19.7264 | 6.7396 | 10.3021 |
| C | -18.8275 | 5.7321 | 11.9856 |
| C | -16.9999 | 6.0162 | 13.6125 |
| C | -16.9969 | 6.8952 | 14.8561 |
| H | -16.4721 | 6.4153 | 15.6942 |
| H | -18.0158 | 7.1192 | 15.2003 |
| H | -16.4996 | 7.8576 | 14.6737 |
| C | -15.5153 | 5.6696 | 13.1111 |
| C | -14.4345 | 6.2450 | 14.0498 |
| H | -14.6727 | 5.9383 | 15.0907 |
| C | -12.9789 | 5.8517 | 13.7701 |
| H | -12.8390 | 4.7639 | 13.9287 |
| H | -12.7213 | 6.0499 | 12.7074 |
| C | -12.0223 | 6.6211 | 14.6806 |
| H | -12.2045 | 6.3970 | 15.7380 |
| H | -10.9801 | 6.3626 | 14.4633 |
| H | -12.1214 | 7.7055 | 14.5561 |
| H | -14.5089 | 7.3522 | 14.0198 |
| C | -16.8403 | 2.1912 | 13.5230 |
| H | -17.7596 | 1.7423 | 13.8990 |
| C | -18.2282 | -4.2725 | 13.0077 |
| H | -19.1841 | -4.8001 | 13.0409 |
| C | -19.5688 | -10.5372 | 12.7684 |
| H | -20.5130 | -10.9718 | 12.4392 |
| C | -16.2395 | -11.8296 | 13.8941 |
| C | -15.6862 | -11.7042 | 15.3281 |
| H | -15.0579 | -12.5944 | 15.5432 |
| H | -16.5434 | -11.7434 | 16.0320 |
| C | -14.8535 | -10.4597 | 15.6527 |
| H | -13.9284 | -10.4494 | 15.0423 |
| C | -14.4841 | -10.4183 | 17.1336 |
| H | -13.8842 | -11.2808 | 17.4227 |
| H | -15.3740 | -10.3937 | 17.7696 |
| H | -13.8953 | -9.5236 | 17.3675 |
| H | -15.4125 | -9.5367 | 15.3835 |
| C | -15.1354 | -12.9744 | 12.0505 |
| C | -18.5188 | -13.3861 | 11.7306 |
| C | -19.5833 | -13.2909 | 10.8430 |
| H | -20.5363 | -12.8726 | 11.1645 |
| H | -21.3819 | -11.9095 | 9.1560 |
| C | -23.5475 | -11.9802 | 7.6261 |
| O | -23.5140 | -11.2090 | 8.7710 |
| C | -24.5819 | -10.2572 | 8.9870 |
| H | -25.5302 | -10.7912 | 9.0862 |
| H | -24.2870 | -9.7995 | 9.9415 |
| H | -24.6107 | -9.5144 | 8.1850 |
| C | -24.5702 | -11.9490 | 6.6770 |
| H | -25.4305 | -11.2847 | 6.7866 |
| N | -26.6705 | -12.1716 | 4.7127 |

m) **(SMe-cube) (Type 1 conformer**)

| **atom** | **x** | **y** | **z** |
| --- | --- | --- | --- |
| C | -1.4537 | -0.2506 | 16.0569 |
| C | 0.9439 | -1.1337 | 16.0569 |
| C | 0.5098 | 1.3843 | 16.0569 |
| C | 0.0000 | 0.0000 | 16.6708 |
| C | -1.2968 | -1.4872 | 15.1890 |
| C | 0.0236 | -1.9805 | 15.2008 |
| C | 0.3584 | -3.1208 | 14.4761 |
| C | -0.6258 | -3.7692 | 13.7211 |
| N | -2.9088 | -3.9964 | 12.9436 |
| C | -1.9356 | -3.2595 | 13.6916 |
| C | -2.2834 | -2.1119 | 14.4370 |
| C | 1.9364 | -0.3795 | 15.1890 |
| C | 1.7034 | 1.0107 | 15.2008 |
| C | 2.5235 | 1.8707 | 14.4761 |
| C | 3.5771 | 1.3426 | 13.7211 |
| N | 4.9154 | -0.5209 | 12.9436 |
| C | 3.7906 | -0.0465 | 13.6916 |
| C | 2.9707 | -0.9215 | 14.4370 |
| C | -0.6396 | 1.8667 | 15.1890 |
| C | -1.7270 | 0.9699 | 15.2008 |
| C | -2.8818 | 1.2500 | 14.4761 |
| C | -2.9513 | 2.4266 | 13.7211 |
| N | -2.0066 | 4.5173 | 12.9436 |
| C | -1.8550 | 3.3060 | 13.6916 |
| C | -0.6873 | 3.0335 | 14.4370 |
| C | -10.4742 | -10.3946 | 6.5404 |
| C | -12.2771 | -8.9859 | 5.4029 |
| C | -11.1612 | -10.9013 | 4.1322 |
| C | -11.7350 | -10.4804 | 5.5628 |
| C | -10.3767 | -8.9256 | 6.9141 |
| C | -11.3800 | -8.1522 | 6.2960 |
| C | -11.4622 | -6.7828 | 6.5315 |
| C | -10.5289 | -6.1736 | 7.3783 |
| N | -8.6207 | -6.2732 | 8.8680 |
| C | -9.5141 | -6.9438 | 7.9722 |
| C | -9.4363 | -8.3358 | 7.7489 |
| C | -12.0408 | -8.6454 | 3.9417 |
| C | -11.4372 | -9.7081 | 3.2393 |
| C | -11.1575 | -9.5889 | 1.8810 |
| C | -11.4680 | -8.3964 | 1.2172 |
| N | -12.3869 | -6.1556 | 1.1812 |
| C | -12.0463 | -7.3296 | 1.9268 |
| C | -12.3459 | -7.4513 | 3.3011 |
| C | -9.6677 | -11.0703 | 4.3513 |
| C | -9.2929 | -10.8048 | 5.6839 |
| C | -7.9645 | -10.9251 | 6.0817 |
| C | -6.9965 | -11.2986 | 5.1422 |
| N | -6.3446 | -11.9654 | 2.9058 |
| C | -7.3703 | -11.5367 | 3.8082 |
| C | -8.7188 | -11.4314 | 3.4035 |
| C | -13.7176 | 5.3312 | -6.5374 |
| C | -14.8381 | 3.3308 | -5.4104 |
| C | -14.5688 | 5.5300 | -4.1370 |
| C | -14.9208 | 4.9184 | -5.5704 |
| C | -13.0527 | 4.0167 | -6.9073 |
| C | -13.6849 | 2.9124 | -6.3006 |
| C | -13.2312 | 1.6187 | -6.5405 |
| C | -12.1272 | 1.4215 | -7.3783 |
| N | -10.3845 | 2.2592 | -8.8390 |
| C | -11.4806 | 2.5274 | -7.9562 |
| C | -11.9474 | 3.8404 | -7.7299 |
| C | -14.4891 | 3.1097 | -3.9486 |
| C | -14.3557 | 4.3235 | -3.2447 |
| C | -14.0546 | 4.3230 | -1.8857 |
| C | -13.8685 | 3.1042 | -1.2233 |
| N | -13.8221 | 0.6822 | -1.1920 |
| C | -13.9752 | 1.8965 | -1.9349 |
| C | -14.2973 | 1.8914 | -3.3095 |
| C | -13.2614 | 6.2744 | -4.3466 |
| C | -12.7986 | 6.1715 | -5.6740 |
| C | -11.6187 | 6.8001 | -6.0619 |
| C | -10.8856 | 7.5288 | -5.1184 |
| N | -10.5759 | 8.4165 | -2.8861 |
|  |  |  |  |
| C | -11.3388 | 7.6103 | -3.7901 |
| C | -12.5415 | 6.9852 | -3.3949 |
| C | -13.0197 | -0.2528 | -1.5899 |
| C | -12.8792 | -1.4853 | -0.7772 |
| C | -12.9451 | -1.4227 | 0.6241 |
| C | -12.7066 | -2.5649 | 1.3866 |
| C | -12.3993 | -3.7916 | 0.7708 |
| C | -12.4077 | -3.8702 | -0.6312 |
| C | -12.6448 | -2.7277 | -1.3937 |
| C | -12.0377 | -4.9765 | 1.5860 |
| C | -3.8602 | 15.1165 | 4.1322 |
| C | -3.7649 | 14.2682 | 6.5404 |
| C | -1.6435 | 15.1252 | 5.4029 |
| C | -3.2088 | 15.4031 | 5.5628 |
| C | -4.7533 | 13.9077 | 4.3513 |
| C | -4.7108 | 13.4502 | 5.6839 |
| C | -5.4791 | 12.3599 | 6.0817 |
| C | -6.2866 | 11.7085 | 5.1422 |
| N | -7.1901 | 11.4773 | 2.9058 |
| C | -6.3059 | 12.1512 | 3.8082 |
| C | -5.5405 | 13.2665 | 3.4035 |
| C | -2.5415 | 13.4493 | 6.9141 |
| C | -1.3700 | 13.9315 | 6.2960 |
| C | -0.1430 | 13.3179 | 6.5315 |
| C | -0.0820 | 12.2051 | 7.3783 |
| N | -1.1224 | 10.6023 | 8.8680 |
| C | -1.2565 | 11.7114 | 7.9722 |
| C | -2.5009 | 12.3400 | 7.7489 |
| C | -1.4667 | 14.7503 | 3.9417 |
| C | -2.6888 | 14.7589 | 3.2393 |
| C | -2.7255 | 14.4571 | 1.8810 |
| C | -1.5375 | 14.1297 | 1.2172 |
| N | 0.8625 | 13.8052 | 1.1812 |
| C | -0.3244 | 14.0972 | 1.9268 |
| C | -0.2800 | 14.4176 | 3.3011 |
| C | -6.7762 | 11.0400 | 1.7596 |
| C | -7.7256 | 10.3345 | 0.8650 |
| C | -8.7290 | 9.5118 | 1.4018 |
| C | -9.5522 | 8.7710 | 0.5554 |
| C | -9.3950 | 8.8352 | -0.8408 |
| C | -8.4408 | 9.7135 | -1.3788 |
| C | -7.6162 | 10.4529 | -0.5323 |
| C | -1.1121 | 4.8966 | 12.0878 |
| C | -1.3216 | 6.1516 | 11.3260 |
| C | -2.6129 | 6.5323 | 10.9267 |
| C | -2.7929 | 7.6571 | 10.1234 |
| C | -1.6928 | 8.4259 | 9.7023 |
| C | -0.4101 | 8.0875 | 10.1627 |
| C | -0.2300 | 6.9619 | 10.9650 |
| C | -6.4506 | -5.6789 | 9.7023 |
| C | -1.8776 | 9.5562 | 8.7603 |
| C | -10.1994 | 7.9652 | -1.7327 |
| C | -7.3371 | -6.4041 | 8.7603 |
| C | 0.3891 | 1.4229 | -16.0822 |
| C | 1.0377 | -1.0484 | -16.0822 |
| C | -1.4268 | -0.3744 | -16.0822 |
| C | 0.0000 | 0.0000 | -16.6961 |
| C | 1.6032 | 1.1470 | -15.2117 |
| C | 1.9708 | -0.2137 | -15.2285 |
| C | 3.0758 | -0.6563 | -14.5073 |
| C | 3.8125 | 0.2593 | -13.7469 |
| N | 4.2415 | 2.5174 | -12.9623 |
| C | 3.4241 | 1.6093 | -13.7096 |
| C | 2.3147 | 2.0672 | -14.4525 |
| C | 0.1918 | -1.9620 | -15.2117 |
| C | -1.1704 | -1.5999 | -15.2285 |
| C | -2.1063 | -2.3355 | -14.5073 |
| C | -1.6817 | -3.4314 | -13.7469 |
| N | 0.0594 | -4.9320 | -12.9623 |
| C | -0.3183 | -3.7700 | -13.7096 |
| C | 0.6329 | -3.0382 | -14.4525 |
| C | -1.7950 | 0.8149 | -15.2117 |
| C | -0.8003 | 1.8136 | -15.2285 |
| C | -0.9695 | 2.9919 | -14.5073 |
| C | -2.1308 | 3.1721 | -13.7469 |
| N | -4.3009 | 2.4145 | -12.9623 |
| C | -3.1058 | 2.1607 | -13.7096 |
| C | -2.9476 | 0.9710 | -14.4525 |
| C | -4.6607 | 1.6425 | -11.9885 |
| C | -5.8942 | 1.9586 | -11.2298 |
| C | 2.1461 | -9.4403 | -8.6987 |
| C | 1.8657 | -8.3171 | -9.6284 |
| C | -1.7983 | -12.8155 | -1.7327 |
| C | -2.9540 | -12.5539 | -0.8408 |
| C | 2.8695 | -7.3945 | -9.9593 |
| C | 2.5593 | -6.2921 | -10.7558 |
| C | 1.2508 | -6.0838 | -11.2298 |
| C | 0.2643 | -7.0447 | -10.9494 |
| C | 0.5772 | -8.1461 | -10.1554 |
| C | 0.9079 | -4.8575 | -11.9885 |
| C | 13.9206 | -6.1393 | 5.4029 |
| C | 15.0214 | -4.2153 | 4.1322 |
| C | 14.2391 | -3.8736 | 6.5404 |
| C | 14.9438 | -4.9226 | 5.5628 |
| C | 13.5075 | -6.1050 | 3.9417 |
| C | 14.1260 | -5.0509 | 3.2393 |
| C | 13.8830 | -4.8682 | 1.8810 |
| C | 13.0055 | -5.7333 | 1.2172 |
| N | 11.5244 | -7.6496 | 1.1812 |
| C | 12.3707 | -6.7676 | 1.9268 |
| C | 12.6260 | -6.9663 | 3.3011 |
| C | 14.4211 | -2.8373 | 4.3513 |
| C | 14.0036 | -2.6455 | 5.6839 |
| C | 13.4436 | -1.4349 | 6.0817 |
| C | 13.2831 | -0.4099 | 5.1422 |
| N | 13.5347 | 0.4881 | 2.9058 |
| C | 13.6762 | -0.6145 | 3.8082 |
| C | 14.2593 | -1.8350 | 3.4035 |
| C | 12.9181 | -4.5236 | 6.9141 |
| C | 12.7500 | -5.7793 | 6.2960 |
| C | 11.6052 | -6.5351 | 6.5315 |
| C | 10.6109 | -6.0316 | 7.3783 |
| N | 9.7431 | -4.3291 | 8.8680 |
| C | 10.7706 | -4.7675 | 7.9722 |
| C | 11.9372 | -4.0042 | 7.7489 |
| C | 10.3286 | -7.9367 | 1.5860 |
| C | 9.2147 | -3.1521 | 8.7603 |
| C | 9.4833 | -8.8423 | 0.7708 |
| C | 8.1434 | -2.7470 | 9.7023 |
| C | 9.5556 | -8.8103 | -0.6312 |
| C | 8.6846 | -9.5869 | -1.3937 |
| C | 7.7259 | -10.4111 | -0.7772 |
| C | 7.7046 | -10.4995 | 0.6241 |
| C | 8.5746 | -9.7218 | 1.3866 |
| C | 7.2090 | -3.6886 | 10.1627 |
| C | 6.1442 | -3.2818 | 10.9650 |
| C | 5.9882 | -1.9312 | 11.3260 |
| C | 6.9636 | -1.0033 | 10.9267 |
| C | 8.0277 | -1.4098 | 10.1234 |
| C | 10.3036 | 11.1848 | -5.4104 |
| C | 12.0735 | 9.8520 | -4.1370 |
| C | 11.4758 | 9.2142 | -6.5374 |
| C | 11.7198 | 10.4626 | -5.5704 |
| C | 9.9376 | 10.9931 | -3.9486 |
| C | 10.9221 | 10.2707 | -3.2447 |
| C | 10.7711 | 10.0101 | -1.8857 |
| C | 9.6225 | 10.4584 | -1.2233 |
| N | 7.5018 | 11.6292 | -1.1920 |
| C | 8.6300 | 11.1546 | -1.9349 |
| C | 8.7867 | 11.4361 | -3.3095 |
| C | 12.0645 | 8.3475 | -4.3466 |
| C | 11.7440 | 7.9981 | -5.6740 |
| C | 11.6984 | 6.6621 | -6.0619 |
| C | 11.9630 | 5.6628 | -5.1184 |
| N | 12.5768 | 4.9507 | -2.8861 |
| C | 12.2601 | 6.0146 | -3.7901 |
| C | 12.3201 | 7.3686 | -3.3949 |
| C | 10.0049 | 9.2957 | -6.9073 |
| C | 9.3647 | 10.3953 | -6.3006 |
| C | 8.0174 | 10.6492 | -6.5405 |
| C | 7.2947 | 9.7917 | -7.3783 |
| N | 7.1487 | 7.8637 | -8.8390 |
| C | 7.9291 | 8.6788 | -7.9562 |
| C | 9.2996 | 8.4266 | -7.7299 |
| C | 12.3490 | 3.7187 | -0.8408 |
| C | 6.2909 | 11.4018 | -1.5899 |
| C | 7.1025 | 6.5788 | -8.6987 |
| C | 5.1533 | 11.8964 | -0.7772 |
| C | 6.2700 | 5.7743 | -9.6284 |
| C | 5.2405 | 11.9221 | 0.6241 |
| C | 4.1320 | 12.2867 | 1.3866 |
| C | 2.9160 | 12.6339 | 0.7708 |
| C | 2.8521 | 12.6805 | -0.6312 |
| C | 3.9602 | 12.3146 | -1.3937 |
| C | 12.6326 | 2.4532 | -1.3788 |
| C | 12.8606 | 1.3693 | -0.5323 |
| C | 12.8128 | 1.5233 | 0.8650 |
| C | 12.6020 | 2.8037 | 1.4018 |
| C | 12.3720 | 3.8870 | 0.5554 |
| C | 4.9691 | 6.1823 | -9.9593 |
| C | 4.1694 | 5.3625 | -10.7558 |
| C | 4.6433 | 4.1252 | -11.2298 |
| C | 5.9687 | 3.7512 | -10.9494 |
| C | 6.7661 | 4.5729 | -10.1554 |
| C | 12.9490 | 0.3484 | 1.7596 |
| C | 6.7288 | -11.1490 | -1.5899 |
| C | 3.7528 | 3.2150 | -11.9885 |
| C | -4.1918 | -12.1667 | -1.3788 |
| C | -5.2444 | -11.8222 | -0.5323 |
| C | -5.0872 | -11.8578 | 0.8650 |
| C | -3.8729 | -12.3155 | 1.4018 |
| C | -2.8198 | -12.6580 | 0.5554 |
| C | -6.7288 | 0.9296 | -10.7558 |
| C | -7.8385 | 1.2122 | -9.9593 |
| C | -8.1357 | 2.5428 | -9.6284 |
| C | -7.3433 | 3.5732 | -10.1554 |
| C | -6.2330 | 3.2935 | -10.9494 |
| C | 1.7090 | 12.9132 | 1.5860 |
| C | -9.2486 | 2.8616 | -8.6987 |
| C | 4.7966 | -1.4852 | 12.0878 |
| C | -6.1728 | -11.3884 | 1.7596 |
| C | -6.7989 | -4.3989 | 10.1627 |
| C | -5.9142 | -3.6802 | 10.9650 |
| C | -4.6666 | -4.2204 | 11.3260 |
| C | -4.3506 | -5.5290 | 10.9267 |
| C | -5.2348 | -6.2472 | 10.1234 |
| C | -3.6845 | -3.4114 | 12.0878 |
| C | 2.4953 | -15.3820 | -4.1370 |
| C | 2.2419 | -14.5454 | -6.5374 |
| C | 4.5345 | -14.5156 | -5.4104 |
| C | 3.2010 | -15.3809 | -5.5704 |
| C | 1.1969 | -14.6219 | -4.3466 |
| C | 1.0546 | -14.1696 | -5.6740 |
| C | -0.0797 | -13.4622 | -6.0619 |
| C | -1.0773 | -13.1916 | -5.1184 |
| N | -2.0010 | -13.3672 | -2.8861 |
| C | -0.9213 | -13.6248 | -3.7901 |
| C | 0.2214 | -14.3538 | -3.3949 |
| C | 3.0478 | -13.3123 | -6.9073 |
| C | 4.3202 | -13.3077 | -6.3006 |
| C | 5.2138 | -12.2679 | -6.5405 |
| C | 4.8326 | -11.2132 | -7.3783 |
| N | 3.2358 | -10.1228 | -8.8390 |
| C | 3.5515 | -11.2062 | -7.9562 |
| C | 2.6478 | -12.2670 | -7.7299 |
| C | 4.5515 | -14.1027 | -3.9486 |
| C | 3.4336 | -14.5942 | -3.2447 |
| C | 3.2835 | -14.3331 | -1.8857 |
| C | 4.2459 | -13.5625 | -1.2233 |
| N | 6.3203 | -12.3114 | -1.1920 |
| C | 5.3451 | -13.0511 | -1.9349 |
| C | 5.5106 | -13.3275 | -3.3095 |
| C | 11.9977 | 4.8504 | -1.7327 |
| S | -6.8391 | -11.4028 | -1.1337 |
| S | -1.3021 | -13.3083 | 1.1504 |
| C | -6.5734 | -10.7826 | -2.8071 |
| C | -1.1525 | -12.7048 | 2.8444 |
| S | 4.1902 | 12.4150 | 3.1360 |
| C | 5.5222 | 11.3135 | 3.6542 |
| S | 3.9771 | 12.4213 | -3.1455 |
| C | 2.2493 | 12.2987 | -3.6510 |
| S | -7.7204 | 5.2534 | -9.7869 |
| S | -6.3212 | -0.6946 | -11.2799 |
| C | -9.1354 | 5.5727 | -10.8690 |
| C | -7.2147 | -1.8089 | -10.1798 |
| S | 4.9840 | -4.4133 | 11.6390 |
| S | 9.3226 | -0.3234 | 9.6507 |
| C | 8.6079 | 1.3322 | 9.7145 |
| C | 4.9989 | -5.8353 | 10.5283 |
| S | 13.2947 | -0.2214 | -1.1337 |
| C | 12.6247 | -0.3015 | -2.8071 |
| S | 12.1764 | 5.5265 | 1.1504 |
| C | 11.5789 | 5.3543 | 2.8444 |
| S | 1.3300 | 6.5230 | 11.6390 |
| C | 2.5541 | 7.2469 | 10.5283 |
| S | -4.3812 | 8.2353 | 9.6507 |
| C | -5.4577 | 6.7885 | 9.7145 |
| S | -0.6894 | -9.3128 | -9.7869 |
| S | 3.7622 | -5.1270 | -11.2799 |
| C | -0.2584 | -10.6978 | -10.8690 |
| C | 5.1739 | -5.3436 | -10.1798 |
| S | -12.8468 | -2.5787 | 3.1360 |
| C | -12.5589 | -0.8744 | 3.6542 |
| S | -12.7457 | -2.7664 | -3.1455 |
| C | -11.7756 | -4.2014 | -3.6510 |
| S | -10.8743 | 7.7818 | 1.1504 |
| S | -6.4556 | 11.6243 | -1.1337 |
| C | -6.0513 | 11.0841 | -2.8071 |
| C | -10.4264 | 7.3505 | 2.8444 |
| S | -4.9414 | -7.9119 | 9.6507 |
| S | -6.3140 | -2.1097 | 11.6390 |
| C | -3.1502 | -8.1207 | 9.7145 |
| C | -7.5530 | -1.4115 | 10.5283 |
| S | 2.5591 | 5.8217 | -11.2799 |
| C | 2.0408 | 7.1526 | -10.1798 |
| S | 8.4098 | 4.0594 | -9.7869 |
| C | 9.3938 | 5.1251 | -10.8690 |
| S | 8.7686 | -9.6549 | -3.1455 |
| C | 9.5264 | -8.0973 | -3.6510 |
| S | 8.6566 | -9.8363 | 3.1360 |
| C | 7.0367 | -10.4391 | 3.6542 |
| C | -2.5652 | -0.4560 | 17.0853 |
| C | 1.6776 | -1.9935 | 17.0853 |
| C | 0.8877 | 2.4496 | 17.0853 |
| C | 0.0000 | 0.0000 | 18.1888 |
| H | 1.3747 | -3.5100 | 14.4909 |
| H | -0.3818 | -4.6673 | 13.1563 |
| H | -3.3036 | -1.7372 | 14.4285 |
| H | 2.3524 | 2.9456 | 14.4909 |
| H | 4.2329 | 2.0030 | 13.1563 |
| H | 3.1563 | -1.9924 | 14.4285 |
| H | -3.7272 | 0.5645 | 14.4909 |
| H | -3.8511 | 2.6643 | 13.1563 |
| H | 0.1473 | 3.7297 | 14.4285 |
| C | -10.5664 | -11.2614 | 7.7954 |
| C | -13.7475 | -8.7869 | 5.7680 |
| C | -11.7627 | -12.1758 | 3.5416 |
| C | -12.8007 | -11.4364 | 6.0679 |
| H | -12.2419 | -6.1844 | 6.0639 |
| H | -10.5854 | -5.1052 | 7.5795 |
| H | -8.6636 | -8.9292 | 8.2307 |
| H | -10.7006 | -10.4113 | 1.3335 |
| H | -11.2637 | -8.2911 | 0.1531 |
| H | -12.8153 | -6.6310 | 3.8380 |
| H | -7.6733 | -10.7309 | 7.1124 |
| H | -5.9549 | -11.4056 | 5.4398 |
| H | -9.0039 | -11.6404 | 2.3757 |
| C | -14.1308 | 6.0934 | -7.7962 |
| C | -16.1144 | 2.5745 | -5.7758 |
| C | -15.6287 | 6.4619 | -3.5515 |
| C | -16.2704 | 5.3828 | -6.0874 |
| H | -13.7244 | 0.7629 | -6.0830 |
| H | -11.7662 | 0.4150 | -7.5833 |
| H | -11.4541 | 4.6893 | -8.1949 |
| H | -13.9614 | 5.2584 | -1.3368 |
| H | -13.6409 | 3.0868 | -0.1589 |
| H | -14.4022 | 0.9529 | -3.8476 |
| H | -11.2621 | 6.7289 | -7.0875 |
| H | -9.9653 | 8.0333 | -5.4083 |
| H | -12.8969 | 7.0712 | -2.3713 |
| H | -12.3805 | -0.2150 | -2.4930 |
| H | -13.1956 | -0.4676 | 1.0942 |
| H | -12.2357 | -4.8422 | -1.1019 |
| H | -11.4499 | -4.7530 | 2.4971 |
| C | -4.6632 | 16.2747 | 3.5416 |
| C | -4.4694 | 14.7814 | 7.7954 |
| C | -0.7359 | 16.2991 | 5.7680 |
| C | -3.5039 | 16.8039 | 6.0679 |
| H | -5.4566 | 12.0107 | 7.1124 |
| H | -6.9001 | 10.8599 | 5.4398 |
| H | -5.5789 | 13.6178 | 2.3757 |
| H | 0.7651 | 13.6940 | 6.0639 |
| H | 0.8715 | 11.7198 | 7.5795 |
| H | -3.4012 | 11.9675 | 8.2307 |
| H | -3.6661 | 14.4727 | 1.3335 |
| H | -1.5484 | 13.9002 | 0.1531 |
| H | 0.6650 | 14.4139 | 3.8380 |
| H | -5.7410 | 11.1155 | 1.3744 |
| H | -8.8520 | 9.4770 | 2.4879 |
| H | -8.3704 | 9.8082 | -2.4661 |
| H | -0.1852 | 4.3468 | 11.8346 |
| H | -3.4635 | 5.9356 | 11.2680 |
| H | 0.4311 | 8.7295 | 9.8868 |
| H | -2.6585 | 9.3987 | 7.9914 |
| H | -10.3974 | 6.9499 | -1.3384 |
| H | -6.8103 | -7.0017 | 7.9914 |
| C | 0.7051 | 2.5084 | -17.1104 |
| C | 1.8198 | -1.8649 | -17.1104 |
| C | -2.5249 | -0.6435 | -17.1104 |
| C | 0.0000 | 0.0000 | -18.2143 |
| H | 3.3692 | -1.7043 | -14.5274 |
| H | 4.6845 | -0.0699 | -13.1845 |
| H | 2.0351 | 3.1172 | -14.4339 |
| H | -3.1606 | -2.0657 | -14.5274 |
| H | -2.4028 | -4.0219 | -13.1845 |
| H | 1.6820 | -3.3210 | -14.4339 |
| H | -0.2086 | 3.7699 | -14.5274 |
| H | -2.2817 | 4.0918 | -13.1845 |
| H | -3.7171 | 0.2038 | -14.4339 |
| H | -4.1079 | 0.7529 | -11.6299 |
| H | 1.3738 | -9.5913 | -7.9204 |
| H | -0.8201 | -12.4794 | -1.3384 |
| H | 3.8841 | -7.5690 | -9.5958 |
| H | -0.7429 | -6.9171 | -11.3622 |
| H | 1.4019 | -3.9340 | -11.6299 |
| C | 14.4834 | -7.5123 | 5.7680 |
| C | 16.4259 | -4.0989 | 3.5416 |
| C | 15.0358 | -3.5201 | 7.7954 |
| C | 16.3045 | -5.3675 | 6.0679 |
| H | 14.3668 | -4.0614 | 1.3335 |
| H | 12.8121 | -5.6091 | 0.1531 |
| H | 12.1503 | -7.7828 | 3.8380 |
| H | 13.1299 | -1.2798 | 7.1124 |
| H | 12.8550 | 0.5457 | 5.4398 |
| H | 14.5828 | -1.9775 | 2.3757 |
| H | 11.4768 | -7.5096 | 6.0639 |
| H | 9.7139 | -6.6146 | 7.5795 |
| H | 12.0647 | -3.0382 | 8.2307 |
| H | 9.8412 | -7.5394 | 2.4971 |
| H | 9.4688 | -2.3971 | 7.9914 |
| H | 10.3113 | -8.1753 | -1.1019 |
| H | 7.0027 | -11.1939 | 1.0942 |
| H | 7.3444 | -4.7381 | 9.8868 |
| H | 6.8721 | 0.0317 | 11.2680 |
| C | 10.2868 | 12.6683 | -5.7758 |
| C | 13.4106 | 10.3039 | -3.5515 |
| C | 12.3424 | 9.1909 | -7.7962 |
| C | 12.7969 | 11.3992 | -6.0874 |
| H | 11.5346 | 9.4618 | -1.3368 |
| H | 9.4937 | 10.2699 | -0.1589 |
| H | 8.0264 | 11.9962 | -3.8476 |
| H | 11.4584 | 6.3888 | -7.0875 |
| H | 11.9397 | 4.6136 | -5.4083 |
| H | 12.5723 | 7.6334 | -2.3713 |
| H | 7.5228 | 11.5042 | -6.0830 |
| H | 6.2425 | 9.9823 | -7.5833 |
| H | 9.7881 | 7.5748 | -8.1949 |
| H | 6.0041 | 10.8293 | -2.4930 |
| H | 7.6194 | 5.9853 | -7.9204 |
| H | 6.1929 | 11.6615 | 1.0942 |
| H | 1.9243 | 13.0175 | -1.1019 |
| H | 12.6794 | 2.3449 | -2.4661 |
| H | 12.6333 | 2.9276 | 2.4879 |
| H | 4.6129 | 7.1482 | -9.5958 |
| H | 6.3619 | 2.8152 | -11.3622 |
| H | 12.4968 | -0.5858 | 1.3744 |
| H | 6.3764 | -10.6143 | -2.4930 |
| H | 2.7060 | 3.1811 | -11.6299 |
| H | -4.3090 | -12.1531 | -2.4661 |
| H | -3.7813 | -12.4046 | 2.4879 |
| H | -8.4970 | 0.4208 | -9.5958 |
| H | -5.6190 | 4.1019 | -11.3622 |
| H | 1.6088 | 12.2924 | 2.4971 |
| H | -8.9932 | 3.6059 | -7.9204 |
| H | 3.8570 | -2.0130 | 11.8346 |
| H | -6.7557 | -10.5296 | 1.3744 |
| H | -7.7755 | -3.9914 | 9.8868 |
| H | -3.4086 | -5.9672 | 11.2680 |
| H | -3.6718 | -2.3338 | 11.8346 |
| C | 2.2182 | -16.7658 | -3.5515 |
| C | 1.7883 | -15.2843 | -7.7962 |
| C | 5.8276 | -15.2428 | -5.7758 |
| C | 3.4735 | -16.7820 | -6.0874 |
| H | -0.1964 | -13.1177 | -7.0875 |
| H | -1.9744 | -12.6469 | -5.4083 |
| H | 0.3246 | -14.7046 | -2.3713 |
| H | 6.2015 | -12.2671 | -6.0830 |
| H | 5.5237 | -10.3973 | -7.5833 |
| H | 1.6659 | -12.2642 | -8.1949 |
| H | 2.4269 | -14.7201 | -1.3368 |
| H | 4.1472 | -13.3567 | -0.1589 |
| H | 6.3759 | -12.9492 | -3.8476 |
| H | 11.2175 | 5.5294 | -1.3384 |
| H | -7.5286 | -10.3645 | -3.1558 |
| H | -5.8204 | -9.9874 | -2.8434 |
| H | -6.2813 | -11.5811 | -3.5004 |
| H | -0.1433 | -12.9639 | 3.1950 |
| H | -1.8767 | -13.1822 | 3.5160 |
| H | -1.2682 | -11.6173 | 2.9115 |
| H | 5.4850 | 11.2505 | 4.7511 |
| H | 5.4077 | 10.3014 | 3.2503 |
| H | 6.5089 | 11.7005 | 3.3712 |
| H | 2.2356 | 12.2064 | -4.7464 |
| H | 1.6773 | 13.1948 | -3.3804 |
| H | 1.7521 | 11.4183 | -3.2285 |
| H | -9.4808 | 6.5964 | -10.6783 |
| H | -9.9631 | 4.8794 | -10.6795 |
| H | -8.8571 | 5.4947 | -11.9272 |
| H | -6.8833 | -2.8314 | -10.4150 |
| H | -6.9966 | -1.6178 | -9.1229 |
| H | -8.2996 | -1.7623 | -10.3355 |
| H | 9.3438 | 2.0220 | 9.2772 |
| H | 7.6814 | 1.4094 | 9.1346 |
| H | 8.4117 | 1.6557 | 10.7442 |
| H | 4.1745 | -6.4957 | 10.8331 |
| H | 4.8385 | -5.5499 | 9.4827 |
| H | 5.9334 | -6.4051 | 10.6027 |
| H | 12.7403 | -1.3377 | -3.1558 |
| H | 11.5595 | -0.0469 | -2.8434 |
| H | 13.1702 | 0.3508 | -3.5004 |
| H | 11.2987 | 6.3578 | 3.1950 |
| H | 12.3544 | 4.9659 | 3.5160 |
| H | 10.6949 | 4.7103 | 2.9115 |
| H | 3.5382 | 6.8631 | 10.8331 |
| H | 2.3871 | 6.9652 | 9.4827 |
| H | 2.5802 | 8.3410 | 10.6027 |
| H | -6.4230 | 7.0810 | 9.2772 |
| H | -5.6397 | 6.4569 | 10.7442 |
| H | -5.0613 | 5.9476 | 9.1346 |
| H | -0.9722 | -11.5088 | -10.6783 |
| H | -0.3300 | -10.4178 | -11.9272 |
| H | 0.7559 | -11.0680 | -10.6795 |
| H | 5.8937 | -4.5454 | -10.4150 |
| H | 5.6760 | -6.3065 | -10.3355 |
| H | 4.8993 | -5.2503 | -9.1229 |
| H | -12.4857 | -0.8751 | 4.7511 |
| H | -11.6251 | -0.4675 | 3.2503 |
| H | -13.3874 | -0.2134 | 3.3712 |
| H | -11.6888 | -4.1671 | -4.7464 |
| H | -12.2657 | -5.1449 | -3.3804 |
| H | -10.7646 | -4.1918 | -3.2285 |
| H | -5.2116 | 11.7023 | -3.1558 |
| H | -5.7391 | 10.0343 | -2.8434 |
| H | -6.8888 | 11.2303 | -3.5004 |
| H | -11.1554 | 6.6061 | 3.1950 |
| H | -10.4778 | 8.2163 | 3.5160 |
| H | -9.4267 | 6.9069 | 2.9115 |
| H | -2.9208 | -9.1030 | 9.2772 |
| H | -2.7720 | -8.1126 | 10.7442 |
| H | -2.6201 | -7.3570 | 9.1346 |
| H | -7.7127 | -0.3674 | 10.8331 |
| H | -7.2256 | -1.4153 | 9.4827 |
| H | -8.5137 | -1.9360 | 10.6027 |
| H | 0.9896 | 7.3768 | -10.4150 |
| H | 2.6236 | 8.0688 | -10.3355 |
| H | 2.0973 | 6.8681 | -9.1229 |
| H | 10.4530 | 4.9124 | -10.6783 |
| H | 9.1871 | 4.9231 | -11.9272 |
| H | 9.2072 | 6.1887 | -10.6795 |
| H | 9.4533 | -8.0393 | -4.7464 |
| H | 10.5884 | -8.0500 | -3.3804 |
| H | 9.0125 | -7.2266 | -3.2285 |
| H | 7.0007 | -10.3754 | 4.7511 |
| H | 6.2175 | -9.8339 | 3.2503 |
| H | 6.8785 | -11.4871 | 3.3712 |
| H | 13.7331 | -8.2995 | 5.6258 |
| H | 14.7982 | -7.5523 | 6.8173 |
| H | 15.3452 | -7.7744 | 5.1428 |
| H | 16.7764 | -6.0840 | 5.3835 |
| H | 16.2334 | -5.8546 | 7.0487 |
| H | 16.9936 | -4.5203 | 6.1759 |
| H | 16.4064 | -3.5952 | 2.5674 |
| H | 16.8802 | -5.0844 | 3.3862 |
| H | 17.0897 | -3.5155 | 4.1906 |
| H | 14.2502 | 10.0242 | -4.1987 |
| H | 13.5922 | 9.8415 | -2.5735 |
| H | 13.4394 | 11.3900 | -3.4047 |
| H | 12.5369 | 11.8132 | -7.0698 |
| H | 13.7614 | 10.8871 | -6.1956 |
| H | 12.9551 | 12.2478 | -5.4098 |
| H | 10.5571 | 12.8278 | -6.8261 |
| H | 10.9812 | 13.2450 | -5.1532 |
| H | 9.2900 | 13.1020 | -5.6293 |
| H | 12.1181 | 8.3149 | -8.4161 |
| H | 13.4095 | 9.1459 | -7.5482 |
| H | 12.1740 | 10.0778 | -8.4184 |
| H | -0.1572 | 2.7153 | -17.7549 |
| H | 1.5496 | 2.2251 | -17.7496 |
| H | 0.9761 | 3.4516 | -16.6205 |
| H | -0.2744 | -0.9823 | -18.6192 |
| H | 0.9879 | 0.2535 | -18.6192 |
| H | -0.7134 | 0.7288 | -18.6192 |
| H | -2.2729 | -1.4937 | -17.7549 |
| H | -2.7019 | 0.2295 | -17.7496 |
| H | -3.4772 | -0.8805 | -16.6205 |
| H | -14.8147 | 5.5041 | -8.4184 |
| H | -13.2599 | 6.3371 | -8.4161 |
| H | -14.6254 | 7.0400 | -7.5482 |
| H | -16.4990 | 4.9507 | -7.0698 |
| H | -16.3092 | 6.4742 | -6.1956 |
| H | -17.0845 | 5.0955 | -5.4098 |
| H | -16.3877 | 2.7288 | -6.8261 |
| H | -16.9611 | 2.8875 | -5.1532 |
| H | -15.9917 | 1.4944 | -5.6293 |
| H | -14.4054 | -9.4021 | 5.1428 |
| H | -14.0541 | -7.7434 | 5.6258 |
| H | -13.9395 | -9.0395 | 6.8173 |
| H | -11.3168 | -12.4108 | 2.5674 |
| H | -12.8434 | -12.0765 | 3.3862 |
| H | -11.5893 | -13.0424 | 4.1906 |
| H | -13.6570 | -11.4868 | 5.3835 |
| H | -13.1869 | -11.1312 | 7.0487 |
| H | -12.4115 | -12.4567 | 6.1759 |
| H | -10.6484 | -12.3254 | 7.5446 |
| H | -11.4309 | -10.9883 | 8.4120 |
| H | -9.6751 | -11.1457 | 8.4241 |
| H | -3.5309 | -0.6326 | 16.5961 |
| H | -2.6856 | 0.4231 | 17.7290 |
| H | -2.3672 | -1.3237 | 17.7258 |
| H | 2.3133 | -2.7416 | 16.5961 |
| H | 0.9764 | -2.5374 | 17.7290 |
| H | 2.3299 | -1.3882 | 17.7258 |
| H | 1.5561 | -17.3531 | -4.1987 |
| H | 1.7269 | -16.6919 | -2.5735 |
| H | 3.1443 | -17.3338 | -3.4047 |
| H | 5.8306 | -15.5566 | -6.8261 |
| H | 5.9799 | -16.1325 | -5.1532 |
| H | 6.7017 | -14.5964 | -5.6293 |
| H | 1.1418 | -14.6520 | -8.4161 |
| H | 1.2159 | -16.1860 | -7.5482 |
| H | 2.6406 | -15.5819 | -8.4184 |
| H | 2.5478 | -17.3613 | -6.1956 |
| H | 4.1294 | -17.3434 | -5.4098 |
| H | 3.9620 | -16.7639 | -7.0698 |
| H | 15.2316 | -4.4054 | 8.4120 |
| H | 14.4900 | -2.8060 | 8.4241 |
| H | 15.9983 | -3.0590 | 7.5446 |
| H | -5.3500 | 15.3845 | 7.5446 |
| H | -3.8007 | 15.3936 | 8.4120 |
| H | -4.8149 | 13.9517 | 8.4241 |
| H | -4.5821 | 16.9770 | 6.1759 |
| H | -3.1193 | 17.5707 | 5.3835 |
| H | -3.0465 | 16.9858 | 7.0487 |
| H | -0.9397 | 17.1765 | 5.1428 |
| H | 0.3211 | 16.0429 | 5.6258 |
| H | -0.8586 | 16.5917 | 6.8173 |
| H | -0.3487 | -0.9584 | 18.5941 |
| H | -0.6556 | 0.7811 | 18.5941 |
| H | 1.0043 | 0.1772 | 18.5941 |
| H | 1.2177 | 3.3742 | 16.5961 |
| H | 1.7093 | 2.1142 | 17.7290 |
| H | 0.0373 | 2.7119 | 17.7258 |
| H | -5.0897 | 16.0060 | 2.5674 |
| H | -4.0369 | 17.1609 | 3.3862 |
| H | -5.5004 | 16.5578 | 4.1906 |
| H | -16.5837 | 5.9439 | -3.4047 |
| H | -15.8063 | 7.3289 | -4.1987 |
| H | -15.3190 | 6.8505 | -2.5735 |
| H | 1.1522 | -2.4546 | -17.7496 |
| H | 2.5011 | -2.5711 | -16.6205 |
| H | 2.4301 | -1.2215 | -17.7549 |

**SMe-cube (Type 2 conformer)**

| **atom** | **x** | **y** | **z** |
| --- | --- | --- | --- |
| C | -9.4452 | 8.2453 | -10.7479 |
| C | -10.8206 | 9.0396 | -8.7468 |
| C | -8.8126 | 10.4057 | -9.5399 |
| C | -10.0579 | 9.5479 | -10.0553 |
| C | -9.9003 | 7.0919 | -9.8709 |
| C | -10.6665 | 7.5322 | -8.7711 |
| C | -11.1777 | 6.6185 | -7.8559 |
| C | -10.9286 | 5.2517 | -8.0367 |
| N | -9.7638 | 3.4504 | -9.2606 |
| C | -10.1536 | 4.8197 | -9.1262 |
| C | -9.6328 | 5.7431 | -10.0593 |
| C | -10.0421 | 9.6396 | -7.5892 |
| C | -8.9375 | 10.3989 | -8.0293 |
| C | -8.1070 | 11.0341 | -7.1125 |
| C | -8.3681 | 10.9019 | -5.7425 |
| N | -9.8211 | 10.0240 | -3.9258 |
| C | -9.4637 | 10.1368 | -5.3073 |
| C | -10.3193 | 9.5037 | -6.2362 |
| C | -7.5922 | 9.6170 | -9.9807 |
| C | -7.9435 | 8.4284 | -10.6547 |
| C | -6.9590 | 7.5768 | -11.1442 |
| C | -5.6105 | 7.9026 | -10.9496 |
| N | -3.9068 | 9.5024 | -10.0927 |
| C | -5.2637 | 9.0814 | -10.2674 |
| C | -6.2612 | 9.9572 | -9.7835 |
| C | -10.3797 | -8.7791 | -9.0737 |
| C | -8.2618 | -9.4690 | -10.3251 |
| C | -8.9686 | -10.7297 | -8.2185 |
| C | -9.5324 | -10.0475 | -9.5484 |
| C | -9.6189 | -7.5823 | -9.6164 |
| C | -8.4522 | -7.9654 | -10.3110 |
| C | -7.6259 | -7.0061 | -10.8864 |
| C | -7.9561 | -5.6505 | -10.7605 |
| N | -9.5422 | -3.9096 | -9.9610 |
| C | -9.1149 | -5.2720 | -10.0612 |
| C | -9.9643 | -6.2443 | -9.4876 |
| C | -7.0722 | -9.8687 | -9.4699 |
| C | -7.4643 | -10.5711 | -8.3110 |
| C | -6.5123 | -11.0252 | -7.4048 |
| C | -5.1551 | -10.7843 | -7.6550 |
| N | -3.4088 | -9.6911 | -9.0144 |
| C | -4.7709 | -10.0735 | -8.8045 |
| C | -5.7332 | -9.6094 | -9.7279 |
| C | -9.5290 | -9.8875 | -7.0857 |
| C | -10.3130 | -8.8146 | -7.5599 |
| C | -10.9141 | -7.9319 | -6.6693 |
| C | -10.7392 | -8.1196 | -5.2919 |
| N | -9.6358 | -9.3282 | -3.4371 |
| C | -9.9482 | -9.1829 | -4.8250 |
| C | -9.3349 | -10.0818 | -5.7254 |
| C | 10.8211 | -9.0437 | -8.7422 |
| C | 8.8131 | -10.4101 | -9.5347 |
| C | 9.4458 | -8.2502 | -10.7436 |
| C | 10.0584 | -9.5526 | -10.0504 |
| C | 10.0425 | -9.6431 | -7.5843 |
| C | 8.9379 | -10.4026 | -8.0241 |
| C | 8.1073 | -11.0374 | -7.1070 |
| C | 8.3684 | -10.9045 | -5.7371 |
| N | 9.8214 | -10.0258 | -3.9207 |
| C | 9.4640 | -10.1393 | -5.3022 |
| C | 10.3197 | -9.5066 | -6.2313 |
| C | 7.5927 | -9.6216 | -9.9759 |
| C | 7.9441 | -8.4333 | -10.6505 |
| C | 6.9596 | -7.5820 | -11.1404 |
| C | 5.6111 | -7.9076 | -10.9457 |
| N | 3.9074 | -9.5070 | -10.0882 |
| C | 5.2643 | -9.0861 | -10.2630 |
| C | 6.2618 | -9.9617 | -9.7786 |
| C | 9.9008 | -7.0964 | -9.8671 |
| C | 10.6670 | -7.5362 | -8.7671 |
| C | 11.1781 | -6.6221 | -7.8523 |
| C | 10.9290 | -5.2554 | -8.0338 |
| N | 9.7643 | -3.4547 | -9.2585 |
| C | 10.1541 | -4.8239 | -9.1235 |
| C | 9.6334 | -5.7477 | -10.0562 |
| C | 2.9830 | -8.6788 | -9.7165 |
| C | 1.5921 | -9.1723 | -9.5730 |
| C | 0.7531 | -8.6651 | -8.5655 |
| C | -0.5676 | -9.0968 | -8.4476 |
| C | -1.0712 | -10.0599 | -9.3350 |
| C | -0.2340 | -10.5691 | -10.3415 |
| C | 1.0849 | -10.1345 | -10.4623 |
| C | -2.4627 | -10.5548 | -9.1823 |
| C | 10.3802 | 8.7750 | -9.0771 |
| C | 8.2623 | 9.4642 | -10.3289 |
| C | 8.9691 | 10.7259 | -8.2229 |
| C | 9.5329 | 10.0431 | -9.5525 |
| C | 9.6194 | 7.5779 | -9.6192 |
| C | 8.4527 | 7.9607 | -10.3141 |
| C | 7.6265 | 7.0011 | -10.8891 |
| C | 7.9567 | 5.6455 | -10.7625 |
| N | 9.5428 | 3.9050 | -9.9621 |
| C | 9.1154 | 5.2674 | -10.0629 |
| C | 9.9648 | 6.2400 | -9.4898 |
| C | 7.0727 | 9.8643 | -9.4740 |
| C | 7.4648 | 10.5673 | -8.3153 |
| C | 6.5127 | 11.0218 | -7.4094 |
| C | 5.1555 | 10.7809 | -7.6596 |
| N | 3.4093 | 9.6870 | -9.0185 |
| C | 4.7714 | 10.0695 | -8.8087 |
| C | 5.7337 | 9.6049 | -9.7319 |
| C | 9.5294 | 9.8843 | -7.0897 |
| C | 10.3134 | 8.8111 | -7.5634 |
| C | 10.9145 | 7.9289 | -6.6723 |
| C | 10.7395 | 8.1172 | -5.2950 |
| N | 9.6360 | 9.3267 | -3.4408 |
| C | 9.9485 | 9.1807 | -4.8286 |
| C | 9.3352 | 10.0792 | -5.7295 |
| C | 8.7207 | 2.9584 | -9.6369 |
| C | 9.2217 | 1.5641 | -9.5718 |
| C | 8.7472 | 0.6789 | -8.5880 |
| C | 9.1844 | -0.6444 | -8.5440 |
| C | 10.1204 | -1.1039 | -9.4832 |
| C | 10.5973 | -0.2202 | -10.4652 |
| C | 10.1570 | 1.1016 | -10.5122 |
| C | -2.9824 | 8.6744 | -9.7205 |
| C | -1.5916 | 9.1679 | -9.5772 |
| C | -0.7526 | 8.6613 | -8.5693 |
| C | 0.5680 | 9.0930 | -8.4516 |
| C | 1.0717 | 10.0556 | -9.3395 |
| C | 0.2346 | 10.5643 | -10.3464 |
| C | -1.0843 | 10.1296 | -10.4670 |
| C | -9.2212 | -1.5685 | -9.5717 |
| C | 2.4632 | 10.5505 | -9.1870 |
| C | 10.6213 | -2.4997 | -9.4105 |
| C | -8.7202 | -2.9628 | -9.6362 |
| C | 10.3806 | -8.7739 | 9.0776 |
| C | 8.2627 | -9.4634 | 10.3293 |
| C | 8.9697 | -10.7250 | 8.2234 |
| C | 9.5334 | -10.0421 | 9.5530 |
| C | 9.6196 | -7.5769 | 9.6198 |
| C | 8.4529 | -7.9598 | 10.3145 |
| C | 7.6266 | -7.0003 | 10.8895 |
| C | 7.9567 | -5.6447 | 10.7629 |
| N | 9.5426 | -3.9040 | 9.9627 |
| C | 9.1154 | -5.2665 | 10.0635 |
| C | 9.9649 | -6.2390 | 9.4904 |
| C | 7.0731 | -9.8636 | 9.4743 |
| C | 7.4654 | -10.5665 | 8.3158 |
| C | 6.5134 | -11.0212 | 7.4098 |
| C | 5.1562 | -10.7804 | 7.6599 |
| N | 3.4098 | -9.6867 | 9.0187 |
| C | 4.7719 | -10.0690 | 8.8090 |
| C | 5.7341 | -9.6044 | 9.7322 |
| C | 9.5300 | -9.8833 | 7.0902 |
| C | 10.3139 | -8.8101 | 7.5639 |
| C | 10.9149 | -7.9278 | 6.6729 |
| C | 10.7400 | -8.1162 | 5.2956 |
| N | 9.6368 | -9.3257 | 3.4413 |
| C | 9.9492 | -9.1797 | 4.8292 |
| C | 9.3359 | -10.0783 | 5.7300 |
| C | 10.5484 | -9.4570 | 2.5344 |
| C | 10.1253 | -9.5449 | 1.1142 |
| C | -2.9821 | -8.6747 | 9.7204 |
| C | -1.5912 | -9.1681 | 9.5772 |
| C | -8.9390 | -9.7447 | 3.0184 |
| C | -9.3675 | -9.6628 | 1.6011 |
| C | -0.7522 | -8.6613 | 8.5694 |
| C | 0.5685 | -9.0929 | 8.4516 |
| C | 1.0722 | -10.0555 | 9.3396 |
| C | 0.2351 | -10.5643 | 10.3464 |
| C | -1.0838 | -10.1297 | 10.4669 |
| C | 2.4637 | -10.5503 | 9.1871 |
| C | -10.3811 | 8.7781 | 9.0731 |
| C | -8.2633 | 9.4681 | 10.3246 |
| C | -8.9702 | 10.7288 | 8.2180 |
| C | -9.5339 | 10.0465 | 9.5479 |
| C | -9.6202 | 7.5813 | 9.6158 |
| C | -8.4535 | 7.9645 | 10.3105 |
| C | -7.6272 | 7.0053 | 10.8859 |
| C | -7.9573 | 5.6496 | 10.7600 |
| N | -9.5432 | 3.9086 | 9.9604 |
| C | -9.1159 | 5.2711 | 10.0606 |
| C | -9.9654 | 6.2433 | 9.4870 |
| C | -7.0737 | 9.8679 | 9.4695 |
| C | -7.4659 | 10.5703 | 8.3106 |
| C | -6.5138 | 11.0246 | 7.4044 |
| C | -5.1566 | 10.7839 | 7.6548 |
| N | -3.4103 | 9.6908 | 9.0142 |
| C | -4.7724 | 10.0730 | 8.8042 |
| C | -5.7347 | 9.6088 | 9.7276 |
| C | -9.5304 | 9.8865 | 7.0853 |
| C | -10.3143 | 8.8135 | 7.5594 |
| C | -10.9153 | 7.9309 | 6.6687 |
| C | -10.7403 | 8.1186 | 5.2913 |
| N | -9.6369 | 9.3273 | 3.4366 |
| C | -9.9494 | 9.1819 | 4.8245 |
| C | -9.3362 | 10.0809 | 5.7249 |
| C | -8.7210 | 2.9619 | 9.6357 |
| C | -10.5486 | 9.4581 | 2.5296 |
| C | -9.2219 | 1.5676 | 9.5712 |
| C | -10.1253 | 9.5453 | 1.1093 |
| C | -8.7473 | 0.6819 | 8.5877 |
| C | -9.1843 | -0.6414 | 8.5444 |
| C | -10.1202 | -1.1006 | 9.4838 |
| C | -10.5972 | -0.2165 | 10.4654 |
| C | -10.1571 | 1.1054 | 10.5117 |
| C | -10.6890 | 10.5019 | 0.2488 |
| C | -10.3194 | 10.5623 | -1.0940 |
| C | -9.3684 | 9.6619 | -1.6016 |
| C | -8.8076 | 8.7029 | -0.7401 |
| C | -9.1746 | 8.6453 | 0.6038 |
| C | 10.8197 | 9.0448 | 8.7428 |
| C | 8.8115 | 10.4110 | 9.5351 |
| C | 9.4443 | 8.2511 | 10.7441 |
| C | 10.0569 | 9.5535 | 10.0510 |
| C | 10.0411 | 9.6441 | 7.5848 |
| C | 8.9365 | 10.4036 | 8.0246 |
| C | 8.1059 | 11.0383 | 7.1075 |
| C | 8.3670 | 10.9054 | 5.7376 |
| N | 9.8201 | 10.0268 | 3.9212 |
| C | 9.4627 | 10.1403 | 5.3027 |
| C | 10.3184 | 9.5077 | 6.2319 |
| C | 7.5912 | 9.6223 | 9.9763 |
| C | 7.9426 | 8.4341 | 10.6509 |
| C | 6.9582 | 7.5826 | 11.1407 |
| C | 5.6097 | 7.9081 | 10.9460 |
| N | 3.9059 | 9.5074 | 10.0883 |
| C | 5.2628 | 9.0866 | 10.2632 |
| C | 6.2602 | 9.9623 | 9.7790 |
| C | 9.8996 | 7.0974 | 9.8676 |
| C | 10.6657 | 7.5373 | 8.7677 |
| C | 11.1770 | 6.6232 | 7.8529 |
| C | 10.9280 | 5.2566 | 8.0343 |
| N | 9.7634 | 3.4557 | 9.2590 |
| C | 10.1531 | 4.8249 | 9.1240 |
| C | 9.6322 | 5.7487 | 10.0567 |
| C | 1.5907 | 9.1725 | 9.5730 |
| C | 8.9389 | 9.7462 | 3.0145 |
| C | 10.6205 | 2.5007 | 9.4111 |
| C | 9.3675 | 9.6636 | 1.5973 |
| C | 10.1197 | 1.1049 | 9.4837 |
| C | 8.8068 | 8.7042 | 0.7362 |
| C | 9.1738 | 8.6459 | -0.6077 |
| C | 10.1244 | 9.5459 | -1.1136 |
| C | 10.6879 | 10.5030 | -0.2536 |
| C | 10.3182 | 10.5640 | 1.0892 |
| C | 0.7517 | 8.6653 | 8.5654 |
| C | -0.5690 | 9.0968 | 8.4475 |
| C | -1.0727 | 10.0598 | 9.3350 |
| C | -0.2357 | 10.5690 | 10.3416 |
| C | 1.0833 | 10.1345 | 10.4624 |
| C | 10.5966 | 0.2213 | 10.4658 |
| C | 10.1565 | -1.1006 | 10.5127 |
| C | 9.2213 | -1.5632 | 9.5723 |
| C | 8.7468 | -0.6780 | 8.5884 |
| C | 9.1839 | 0.6453 | 8.5445 |
| C | -2.4643 | 10.5545 | 9.1822 |
| C | -10.6210 | -2.4964 | 9.4118 |
| C | 8.7205 | -2.9575 | 9.6374 |
| C | -8.8067 | -8.7037 | 0.7396 |
| C | -9.1737 | -8.6461 | -0.6043 |
| C | -10.1243 | -9.5463 | -1.1099 |
| C | -10.6878 | -10.5030 | -0.2494 |
| C | -10.3183 | -10.5633 | 1.0934 |
| C | 9.1746 | -8.6450 | 0.6082 |
| C | 8.8077 | -8.7032 | -0.7357 |
| C | 9.3685 | -9.6626 | -1.5967 |
| C | 10.3194 | -10.5629 | -1.0886 |
| C | 10.6889 | -10.5019 | 0.2542 |
| C | 10.5476 | 9.4581 | -2.5338 |
| C | 8.9401 | -9.7452 | -3.0140 |
| C | -8.9399 | 9.7439 | -3.0189 |
| C | -10.5474 | -9.4591 | -2.5301 |
| C | -8.7468 | -0.6829 | -8.5882 |
| C | -9.1840 | 0.6404 | -8.5449 |
| C | -10.1198 | 1.0996 | -9.4843 |
| C | -10.5967 | 0.2154 | -10.4660 |
| C | -10.1564 | -1.1064 | -10.5124 |
| C | -10.6208 | 2.4953 | -9.4123 |
| C | -10.8202 | -9.0408 | 8.7463 |
| C | -8.8120 | -10.4066 | 9.5394 |
| C | -9.4449 | -8.2463 | 10.7474 |
| C | -10.0575 | -9.5490 | 10.0547 |
| C | -10.0415 | -9.6406 | 7.5887 |
| C | -8.9369 | -10.3998 | 8.0289 |
| C | -8.1062 | -11.0349 | 7.1120 |
| C | -8.3673 | -10.9027 | 5.7420 |
| N | -9.8203 | -10.0249 | 3.9252 |
| C | -9.4629 | -10.1378 | 5.3068 |
| C | -10.3187 | -9.5048 | 6.2356 |
| C | -7.5917 | -9.6178 | 9.9803 |
| C | -7.9432 | -8.4293 | 10.6543 |
| C | -6.9589 | -7.5776 | 11.1439 |
| C | -5.6103 | -7.9032 | 10.9493 |
| N | -3.9064 | -9.5028 | 10.0925 |
| C | -5.2634 | -9.0819 | 10.2671 |
| C | -6.2607 | -9.9578 | 9.7832 |
| C | -9.9001 | -7.0929 | 9.8704 |
| C | -10.6662 | -7.5333 | 8.7705 |
| C | -11.1774 | -6.6196 | 7.8554 |
| C | -10.9284 | -5.2528 | 8.0362 |
| N | -9.7639 | -3.4514 | 9.2602 |
| C | -10.1536 | -4.8208 | 9.1258 |
| C | -9.6328 | -5.7441 | 10.0589 |
| C | 2.9816 | 8.6791 | 9.7165 |
| S | -7.6531 | 1.3347 | 7.3789 |
| C | -6.5827 | -0.0491 | 6.9327 |
| S | -11.6989 | -0.8832 | 11.6588 |
| C | -12.7812 | 0.4925 | 12.1022 |
| S | 7.6530 | 1.3321 | -7.3794 |
| C | 6.5827 | -0.0516 | -6.9326 |
| S | 11.6990 | -0.8874 | -11.6583 |
| C | 12.7811 | 0.4883 | -12.1024 |
| S | -11.6983 | 0.8821 | -11.6595 |
| C | -12.7803 | -0.4937 | -12.1031 |
| S | -7.6527 | -1.3356 | -7.3793 |
| C | -6.5825 | 0.0483 | -6.9329 |
| S | 7.6971 | -7.5400 | -1.4416 |
| C | 6.5222 | -7.1698 | -0.1216 |
| S | 11.8056 | -11.6504 | 0.9733 |
| C | 12.9774 | -12.0363 | -0.3453 |
| S | -7.6968 | 7.5395 | -1.4454 |
| S | -11.8058 | 11.6507 | 0.9673 |
| C | -12.9776 | 12.0359 | -0.3515 |
| S | -7.6962 | -7.5401 | 1.4450 |
| C | -6.5213 | -7.1704 | 0.1249 |
| S | -11.8044 | -11.6519 | -0.9680 |
| C | -12.9762 | -12.0372 | 0.3508 |
| S | 0.9588 | 11.6995 | -11.4729 |
| C | -0.3959 | 12.7878 | -11.9631 |
| S | -1.4561 | 7.5327 | -7.4214 |
| C | -0.1046 | 6.4122 | -6.9986 |
| S | -0.9582 | -11.7050 | -11.4674 |
| C | 0.3966 | -12.7935 | -11.9570 |
| S | 1.4553 | 7.5362 | 7.4180 |
| C | 0.1039 | 6.4154 | 6.9957 |
| S | -0.9600 | 11.7047 | 11.4676 |
| C | 0.3946 | 12.7933 | 11.9573 |
| S | 0.9594 | -11.6995 | 11.4729 |
| C | -0.3952 | -12.7879 | 11.9630 |
| S | -1.4558 | -7.5328 | 7.4215 |
| C | -0.1043 | -6.4122 | 6.9987 |
| S | 1.4564 | -7.5358 | -7.4182 |
| C | 0.1048 | -6.4152 | -6.9961 |
| S | 7.6962 | 7.5409 | 1.4421 |
| C | 6.5214 | 7.1705 | 0.1220 |
| S | 11.8044 | 11.6516 | -0.9727 |
| C | 12.9762 | 12.0376 | 0.3460 |
| S | 11.6982 | 0.8886 | 11.6590 |
| C | 12.7804 | -0.4869 | 12.1032 |
| S | 7.6528 | -1.3314 | 7.3798 |
| C | 6.5824 | 0.0522 | 6.9327 |
| C | -9.8759 | 8.0177 | -12.1969 |
| C | -12.2951 | 9.4343 | -8.6616 |
| C | -8.7461 | 11.8369 | -10.0730 |
| C | -10.9613 | 10.3344 | -10.9887 |
| C | -11.8312 | -8.7529 | -9.5529 |
| C | -8.0880 | -9.9803 | -11.7554 |
| C | -9.3499 | -12.1984 | -8.0316 |
| C | -10.3465 | -11.0084 | -10.3970 |
| C | 12.2955 | -9.4383 | -8.6567 |
| C | 8.7466 | -11.8416 | -10.0672 |
| C | 9.8766 | -8.0233 | -12.1927 |
| C | 10.9619 | -10.3395 | -10.9835 |
| C | 11.8317 | 8.7486 | -9.5563 |
| C | 8.0886 | 9.9749 | -11.7594 |
| C | 9.3503 | 12.1948 | -8.0366 |
| C | 10.3471 | 11.0036 | -10.4015 |
| C | 11.8321 | -8.7474 | 9.5569 |
| C | 8.0890 | -9.9740 | 11.7599 |
| C | 9.3511 | -12.1938 | 8.0371 |
| C | 10.3476 | -11.0026 | 10.4020 |
| C | -11.8326 | 8.7517 | 9.5523 |
| C | -8.0896 | 9.9794 | 11.7549 |
| C | -9.3516 | 12.1975 | 8.0311 |
| C | -10.3482 | 11.0073 | 10.3965 |
| C | 12.2941 | 9.4396 | 8.6573 |
| C | 8.7448 | 11.8424 | 10.0677 |
| C | 9.8751 | 8.0243 | 12.1932 |
| C | 10.9602 | 10.3405 | 10.9841 |
| C | -12.2946 | -9.4356 | 8.6609 |
| C | -8.7454 | -11.8378 | 10.0725 |
| C | -9.8758 | -8.0188 | 12.1964 |
| C | -10.9609 | -10.3356 | 10.9881 |
| C | -6.5219 | 7.1700 | -0.1252 |
| H | -11.7634 | 6.9540 | -7.0020 |
| H | -11.3165 | 4.5318 | -7.3203 |
| H | -9.0279 | 5.3925 | -10.8925 |
| H | -7.2551 | 11.6230 | -7.4476 |
| H | -7.7248 | 11.4002 | -5.0212 |
| H | -11.1687 | 8.9227 | -5.8826 |
| H | -7.2253 | 6.6595 | -11.6663 |
| H | -4.8371 | 7.2430 | -11.3360 |
| H | -5.9758 | 10.8709 | -9.2660 |
| H | -6.7246 | -7.2970 | -11.4230 |
| H | -7.3165 | -4.8966 | -11.2133 |
| H | -10.8621 | -5.9346 | -8.9565 |
| H | -6.8107 | -11.5594 | -6.5044 |
| H | -4.4051 | -11.1284 | -6.9471 |
| H | -5.4195 | -9.0529 | -10.6081 |
| H | -11.5129 | -7.0978 | -7.0307 |
| H | -11.1974 | -7.4274 | -4.5898 |
| H | -8.7177 | -10.8939 | -5.3473 |
| H | 7.2555 | -11.6264 | -7.4419 |
| H | 7.7251 | -11.4025 | -5.0156 |
| H | 11.1690 | -8.9254 | -5.8780 |
| H | 7.2260 | -6.6649 | -11.6629 |
| H | 4.8377 | -7.2482 | -11.3325 |
| H | -4.4067 | 11.1280 | 6.9469 |
| H | 5.9763 | -10.8752 | -9.2608 |
| H | 11.7638 | -6.9573 | -6.9982 |
| H | 11.3169 | -4.5352 | -7.3176 |
| H | 9.0285 | -5.3975 | -10.8896 |
| H | 3.1260 | -7.6058 | -9.4869 |
| H | -1.2261 | -8.6979 | -7.6718 |
| H | 1.7421 | -10.5294 | -11.2421 |
| H | -2.5717 | -11.6541 | -9.2195 |
| H | 6.7252 | 7.2917 | -11.4258 |
| H | 7.3171 | 4.8914 | -11.2149 |
| H | 10.8625 | 5.9306 | -8.9585 |
| H | 6.8110 | 11.5565 | -6.5093 |
| H | 4.4055 | 11.1252 | -6.9519 |
| H | 5.4200 | 9.0480 | -10.6118 |
| H | 11.5133 | 7.0947 | -7.0333 |
| H | 11.1977 | 7.4254 | -4.5925 |
| H | 8.7181 | 10.8915 | -5.3517 |
| H | 7.6482 | 3.0828 | -9.3947 |
| H | 8.8112 | -1.3393 | -7.7874 |
| H | 10.5271 | 1.7949 | -11.2725 |
| H | -3.1255 | 7.6015 | -9.4904 |
| H | 1.2264 | 8.6945 | -7.6755 |
| H | -1.7414 | 10.5241 | -11.2470 |
| H | 2.5722 | 11.6499 | -9.2248 |
| H | 11.7189 | -2.6044 | -9.4886 |
| H | -7.6477 | -3.0872 | -9.3939 |
| H | 6.7253 | -7.2910 | 11.4262 |
| H | 7.3170 | -4.8907 | 11.2154 |
| H | 10.8626 | -5.9295 | 8.9591 |
| H | 6.8119 | -11.5558 | 6.5097 |
| H | 4.4063 | -11.1248 | 6.9522 |
| H | 5.4204 | -9.0475 | 10.6121 |
| H | 11.5136 | -7.0935 | 7.0339 |
| H | 11.1982 | -7.4243 | 4.5932 |
| H | 8.7189 | -10.8906 | 5.3522 |
| H | 11.6402 | -9.5072 | 2.6994 |
| H | -3.1253 | -7.6018 | 9.4903 |
| H | -7.8632 | -9.5532 | 3.1940 |
| H | 1.2269 | -8.6944 | 7.6756 |
| H | -1.7409 | -10.5243 | 11.2469 |
| H | 2.5729 | -11.6497 | 9.2249 |
| H | -6.7259 | 7.2962 | 11.4226 |
| H | -7.3176 | 4.8958 | 11.2128 |
| H | -10.8631 | 5.9335 | 8.9558 |
| H | -6.8122 | 11.5588 | 6.5041 |
| H | -5.4210 | 9.0523 | 10.6078 |
| H | -11.5140 | 7.0967 | 7.0301 |
| H | -11.1985 | 7.4264 | 4.5892 |
| H | -8.7192 | 10.8931 | 5.3468 |
| H | -7.6485 | 3.0864 | 9.3934 |
| H | -11.6404 | 9.5083 | 2.6944 |
| H | -8.8111 | -1.3366 | 7.7881 |
| H | -10.5273 | 1.7990 | 11.2717 |
| H | -10.7571 | 11.3028 | -1.7689 |
| H | -8.7340 | 7.9078 | 1.2797 |
| H | 7.7237 | 11.4033 | 5.0160 |
| H | 7.2539 | 11.6272 | 7.4423 |
| H | 11.1678 | 8.9265 | 5.8786 |
| H | 7.2246 | 6.6656 | 11.6632 |
| H | 4.8363 | 7.2487 | 11.3327 |
| H | 5.9747 | 10.8758 | 9.2611 |
| H | 11.7627 | 6.9585 | 6.9988 |
| H | 11.3160 | 4.5364 | 7.3182 |
| H | 9.0273 | 5.3984 | 10.8900 |
| H | 7.8631 | 9.5547 | 3.1901 |
| H | 11.7181 | 2.6056 | 9.4893 |
| H | 8.7333 | 7.9080 | -1.2832 |
| H | 10.7559 | 11.3048 | 1.7638 |
| H | -1.2274 | 8.6979 | 7.6716 |
| H | 1.7403 | 10.5294 | 11.2422 |
| H | 10.5266 | -1.7938 | 11.2731 |
| H | 8.8108 | 1.3401 | 7.7878 |
| H | -2.5734 | 11.6539 | 9.2195 |
| H | -11.7187 | -2.6012 | 9.4899 |
| H | 7.6480 | -3.0821 | 9.3951 |
| H | -8.7331 | -7.9086 | -1.2802 |
| H | -10.7560 | -11.3039 | 1.7683 |
| H | 8.7341 | -7.9072 | 1.2838 |
| H | 10.7571 | -11.3037 | -1.7632 |
| H | 11.6394 | 9.5084 | -2.6987 |
| H | 7.8643 | -9.5539 | -3.1896 |
| H | -7.8641 | 9.5525 | -3.1944 |
| H | -11.6392 | -9.5095 | -2.6951 |
| H | -8.8109 | 1.3356 | -7.7885 |
| H | -10.5264 | -1.8000 | -11.2724 |
| H | -11.7184 | 2.6000 | -9.4905 |
| H | -7.2543 | -11.6238 | 7.4472 |
| H | -7.7239 | -11.4009 | 5.0207 |
| H | -11.1681 | -8.9238 | 5.8820 |
| H | -7.2253 | -6.6603 | 11.6660 |
| H | -4.8370 | -7.2435 | 11.3358 |
| H | -5.9752 | -10.8716 | 9.2657 |
| H | -11.7631 | -6.9552 | 7.0014 |
| H | -11.3164 | -4.5330 | 7.3197 |
| H | -9.0279 | -5.3935 | 10.8921 |
| H | 3.1248 | 7.6061 | 9.4869 |
| H | -5.7223 | 0.3626 | 6.3869 |
| H | -6.2031 | -0.5838 | 7.8118 |
| H | -7.0904 | -0.7670 | 6.2780 |
| H | -13.6383 | 0.0755 | 12.6489 |
| H | -12.2787 | 1.2159 | 12.7548 |
| H | -13.1639 | 1.0218 | 11.2213 |
| H | 5.7223 | 0.3603 | -6.3869 |
| H | 6.2032 | -0.5868 | -7.8115 |
| H | 7.0905 | -0.7692 | -6.2776 |
| H | 13.6383 | 0.0711 | -12.6489 |
| H | 12.2785 | 1.2113 | -12.7553 |
| H | 13.1638 | 1.0180 | -11.2218 |
| H | -13.6375 | -0.0768 | -12.6499 |
| H | -12.2777 | -1.2170 | -12.7557 |
| H | -13.1631 | -1.0231 | -11.2223 |
| H | -5.7221 | -0.3634 | -6.3870 |
| H | -6.2028 | 0.5831 | -7.8120 |
| H | -7.0903 | 0.7661 | -6.2782 |
| H | 5.6598 | -6.6656 | -0.5798 |
| H | 6.1588 | -8.0750 | 0.3799 |
| H | 6.9494 | -6.4989 | 0.6327 |
| H | 13.8352 | -12.5468 | 0.1142 |
| H | 12.5434 | -12.7060 | -1.0968 |
| H | 13.3474 | -11.1358 | -0.8501 |
| H | -13.8354 | 12.5465 | 0.1077 |
| H | -12.5436 | 12.7053 | -1.1032 |
| H | -13.3475 | 11.1352 | -0.8559 |
| H | -5.6589 | -6.6659 | 0.5828 |
| H | -6.1579 | -8.0758 | -0.3762 |
| H | -6.9486 | -6.4999 | -0.6298 |
| H | -13.8340 | -12.5480 | -0.1085 |
| H | -12.5422 | -12.7066 | 1.1025 |
| H | -13.3463 | -11.1366 | 0.8551 |
| H | 0.0461 | 13.6584 | -12.4675 |
| H | -1.0820 | 12.2987 | -12.6644 |
| H | -0.9721 | 13.1490 | -11.1029 |
| H | -0.5453 | 5.5352 | -6.5045 |
| H | 0.4416 | 6.0648 | -7.8840 |
| H | 0.6092 | 6.8726 | -6.3057 |
| H | -0.0453 | -13.6644 | -12.4609 |
| H | 1.0828 | -12.3048 | -12.6585 |
| H | 0.9728 | -13.1543 | -11.0965 |
| H | 0.5446 | 5.5382 | 6.5021 |
| H | -0.4422 | 6.0684 | 7.8813 |
| H | -0.6100 | 6.8754 | 6.3026 |
| H | -0.0474 | 13.6641 | 12.4613 |
| H | 1.0808 | 12.3046 | 12.6588 |
| H | 0.9708 | 13.1542 | 11.0969 |
| H | 0.0468 | -13.6585 | 12.4674 |
| H | -1.0814 | -12.2989 | 12.6643 |
| H | -0.9714 | -13.1492 | 11.1028 |
| H | -0.5451 | -5.5352 | 6.5047 |
| H | 0.4418 | -6.0647 | 7.8842 |
| H | 0.6095 | -6.8724 | 6.3059 |
| H | 0.5455 | -5.5378 | -6.5025 |
| H | -0.4412 | -6.0683 | -7.8818 |
| H | -0.6090 | -6.8751 | -6.3030 |
| H | 5.6590 | 6.6662 | 0.5802 |
| H | 6.1580 | 8.0757 | -0.3795 |
| H | 6.9487 | 6.4997 | -0.6323 |
| H | 13.8339 | 12.5482 | -0.1135 |
| H | 12.5421 | 12.7073 | 1.0974 |
| H | 13.3463 | 11.1372 | 0.8508 |
| H | 13.6375 | -0.0696 | 12.6498 |
| H | 12.2778 | -1.2099 | 12.7561 |
| H | 13.1632 | -1.0166 | 11.2226 |
| H | 5.7221 | -0.3598 | 6.3870 |
| H | 6.2028 | 0.5873 | 7.8116 |
| H | 7.0902 | 0.7698 | 6.2778 |
| H | -9.4274 | 7.1032 | -12.6038 |
| H | -10.9632 | 7.9082 | -12.2821 |
| H | -9.5640 | 8.8458 | -12.8438 |
| H | -12.7510 | 9.0611 | -7.7364 |
| H | -12.4216 | 10.5232 | -8.6649 |
| H | -12.8714 | 9.0201 | -9.4965 |
| H | -7.8636 | 12.3618 | -9.6871 |
| H | -8.6783 | 11.8568 | -11.1669 |
| H | -9.6251 | 12.4190 | -9.7736 |
| H | -11.8122 | 9.7322 | -11.3309 |
| H | -11.3709 | 11.2265 | -10.4986 |
| H | -10.4227 | 10.6733 | -11.8826 |
| H | -12.3483 | -7.8533 | -9.1974 |
| H | -11.8942 | -8.7479 | -10.6473 |
| H | -12.3932 | -9.6174 | -9.1815 |
| H | -7.1939 | -9.5502 | -12.2230 |
| H | -7.9732 | -11.0699 | -11.7832 |
| H | -8.9433 | -9.7103 | -12.3854 |
| H | -8.9381 | -12.5975 | -7.0965 |
| H | -10.4370 | -12.3304 | -7.9865 |
| H | -8.9633 | -12.8210 | -8.8465 |
| H | -10.7234 | -10.5247 | -11.3069 |
| H | -9.7508 | -11.8742 | -10.7122 |
| H | -11.2161 | -11.3940 | -9.8501 |
| H | 12.7515 | -9.0646 | -7.7316 |
| H | 12.4220 | -10.5272 | -8.6594 |
| H | 12.8719 | -9.0244 | -9.4918 |
| H | 7.8641 | -12.3663 | -9.6811 |
| H | 8.6789 | -11.8619 | -11.1610 |
| H | 9.6256 | -12.4235 | -9.7674 |
| H | 9.4281 | -7.1090 | -12.6001 |
| H | 10.9639 | -7.9138 | -12.2779 |
| H | 9.5647 | -8.8517 | -12.8393 |
| H | 11.3714 | -11.2314 | -10.4929 |
| H | 10.4233 | -10.6787 | -11.8772 |
| H | 11.8128 | -9.7374 | -11.3258 |
| H | 12.3488 | 7.8491 | -9.2003 |
| H | 11.8947 | 8.7430 | -10.6506 |
| H | 12.3937 | 9.6132 | -9.1852 |
| H | 7.1945 | 9.5446 | -12.2269 |
| H | 7.9738 | 11.0645 | -11.7878 |
| H | 8.9439 | 9.7045 | -12.3893 |
| H | 8.9385 | 12.5943 | -7.1018 |
| H | 10.4374 | 12.3268 | -7.9916 |
| H | 8.9638 | 12.8169 | -8.8519 |
| H | 10.7240 | 10.5194 | -11.3111 |
| H | 9.7514 | 11.8692 | -10.7171 |
| H | 11.2166 | 11.3894 | -9.8548 |
| H | 12.3491 | -7.8478 | 9.2010 |
| H | 11.8950 | -8.7418 | 10.6513 |
| H | 12.3941 | -9.6119 | 9.1859 |
| H | 7.1948 | -9.5438 | 12.2273 |
| H | 7.9743 | -11.0637 | 11.7882 |
| H | 8.9442 | -9.7036 | 12.3898 |
| H | 8.9394 | -12.5934 | 7.1023 |
| H | 10.4383 | -12.3258 | 7.9922 |
| H | 8.9646 | -12.8160 | 8.8524 |
| H | 10.7244 | -10.5184 | 11.3117 |
| H | 9.7520 | -11.8683 | 10.7176 |
| H | 11.2172 | -11.3883 | 9.8554 |
| H | -12.3496 | 7.8520 | 9.1968 |
| H | -11.8956 | 8.7466 | 10.6467 |
| H | -12.3947 | 9.6161 | 9.1809 |
| H | -7.1955 | 9.5494 | 12.2226 |
| H | -7.9750 | 11.0691 | 11.7828 |
| H | -8.9450 | 9.7093 | 12.3849 |
| H | -8.9398 | 12.5967 | 7.0961 |
| H | -10.4387 | 12.3294 | 7.9860 |
| H | -8.9651 | 12.8201 | 8.8461 |
| H | -10.7251 | 10.5235 | 11.3063 |
| H | -9.7526 | 11.8732 | 10.7117 |
| H | -11.2177 | 11.3928 | 9.8496 |
| H | 12.7501 | 9.0659 | 7.7323 |
| H | 12.4205 | 10.5284 | 8.6601 |
| H | 12.8705 | 9.0257 | 9.4925 |
| H | 7.8623 | 12.3670 | 9.6815 |
| H | 8.6771 | 11.8627 | 11.1616 |
| H | 9.6239 | 12.4244 | 9.7680 |
| H | 9.4266 | 7.1099 | 12.6006 |
| H | 10.9624 | 7.9149 | 12.2785 |
| H | 9.5631 | 8.8526 | 12.8398 |
| H | 11.3697 | 11.2325 | 10.4935 |
| H | 10.4216 | 10.6797 | 11.8778 |
| H | 11.8112 | 9.7386 | 11.3265 |
| H | -12.7505 | -9.0624 | 7.7357 |
| H | -12.4210 | -10.5245 | 8.6642 |
| H | -12.8710 | -9.0214 | 9.4959 |
| H | -7.8628 | -12.3626 | 9.6867 |
| H | -8.6777 | -11.8577 | 11.1664 |
| H | -9.6244 | -12.4200 | 9.7731 |
| H | -9.4274 | -7.1042 | 12.6034 |
| H | -10.9631 | -7.9093 | 12.2815 |
| H | -9.5638 | -8.8468 | 12.8433 |
| H | -11.3703 | -11.2278 | 10.4979 |
| H | -10.4223 | -10.6744 | 11.8820 |
| H | -11.8119 | -9.7335 | 11.3302 |
| H | -5.6595 | 6.6656 | -0.5830 |
| H | -6.1587 | 8.0755 | 0.3759 |
| H | -6.9491 | 6.4995 | 0.6295 |

**References**

[1]	M. J. Frisch, G. W. Trucks, H. B. Schlegel, G. E. Scuseria, M. A. Robb, J. R. Cheeseman, G. Scalmani, V. Barone, G. A. Petersson, H. Nakatsuji, X. Li, M. Caricato, A. V. Marenich, J. Bloino, B. G. Janesko, R. Gomperts, B. Mennucci, H. P. Hratchian, J. V. Ortiz, A. F. Izmaylov, J. L. Sonnenberg, Williams, F. Ding, F. Lipparini, F. Egidi, J. Goings, B. Peng, A. Petrone, T. Henderson, D. Ranasinghe, V. G. Zakrzewski, J. Gao, N. Rega, G. Zheng, W. Liang, M. Hada, M. Ehara, K. Toyota, R. Fukuda, J. Hasegawa, M. Ishida, T. Nakajima, Y. Honda, O. Kitao, H. Nakai, T. Vreven, K. Throssell, J. A. Montgomery Jr., J. E. Peralta, F. Ogliaro, M. J. Bearpark, J. J. Heyd, E. N. Brothers, K. N. Kudin, V. N. Staroverov, T. A. Keith, R. Kobayashi, J. Normand, K. Raghavachari, A. P. Rendell, J. C. Burant, S. S. Iyengar, J. Tomasi, M. Cossi, J. M. Millam, M. Klene, C. Adamo, R. Cammi, J. W. Ochterski, R. L. Martin, K. Morokuma, O. Farkas, J. B. Foresman, D. J. Fox: Gaussian 16 Rev. C.01, Wallingford, CT, 2016.

[2]	J. J. P. Stewart, J. Mol. Model. 2007, 13, 1173-1213.
